# Distinct epigenetic signatures elucidate enhancer-gene relationships that delineate CIMP and non-CIMP colorectal cancers

**DOI:** 10.18632/oncotarget.8473

**Published:** 2016-03-30

**Authors:** Allen Chong, Jing Xian Teo, Kenneth H.K. Ban

**Affiliations:** ^1^ Department of Pathology, National University of Singapore, 119074 Singapore; ^2^ Cancer Science Institute, National University of Singapore, 117599 Singapore; ^3^ Department of Biochemistry, National University of Singapore, 117596 Singapore; ^4^ Institute of Molecular and Cell Biology, 138673 Singapore; ^5^ Present address: Shanxi Guoxin Caregeno Medical Laboratories, Taiyuan, Shanxi Province, China

**Keywords:** DNA methylation, enhancers, epigenetics, bioinformatics, colon cancer

## Abstract

Epigenetic changes, like DNA methylation, affect gene expression and in colorectal cancer (CRC), a distinct phenotype called the CpG island methylator phenotype (“CIMP”) has significantly higher levels of DNA methylation at so-called “Type C loci” within the genome. We postulate that enhancer-gene pairs are coordinately controlled through DNA methylation in order to regulate the expression of key genes/biomarkers for a particular phenotype.

Firstly, we found 24 experimentally-validated enhancers (VISTA enhancer browser) that contained statistically significant (FDR-adjusted q-value of <0.01) differentially methylated regions (DMRs) (1000bp) in a study of CIMP versus non-CIMP CRCs. Of these, the methylation of 2 enhancers, 1702 and 1944, were found to be very well correlated with the methylation of the genes Wnt3A and IGDCC3, respectively, in two separate and independent datasets.

We show for the first time that there are indeed distinct and dynamic changes in the methylation pattern of specific enhancer-gene pairs in CRCs. Such a coordinated epigenetic event could be indicative of an interaction between (1) enhancer 1702 and Wnt3A and (2) enhancer 1944 and IGDCC3. Moreover, our study shows that the methylation patterns of these 2 enhancer-gene pairs can potentially be used as biomarkers to delineate CIMP from non-CIMP CRCs.

## INTRODUCTION

Toyota *et al.* [1] first coined the term “CIMP” and defined the CpG island methylator phenotype as a subset of colorectal cancers (CRCs) which showed DNA methylation in 7 cancer-specific differentially methylated loci (termed Type C for cancer-specific – the other 19 differentially methylated loci that they studied were found to be age-specific and termed Type A). In contrast to Type A methylation, Type C methylation is relatively infrequent in primary colorectal cancer, and is never observed in normal colon mucosa. Some studies have since tried to re-classify the CIMP phenotype into CIMP-high, CIMP-low and CIMP-negative based on different panels of markers [2, 3].

Holliday, Pugh and Riggs were first to suggest that DNA methylation of cytosines in the context of CpG dinucleotides may represent an epigenetic mark associated with gene silencing [4, 5]. CpG density is not evenly distributed within the genome, but rather, shows a bimodal distribution. Regions with an elevated CpG content, so-called CpG islands (CGIs), overlap with the transcriptional start sites (TSSs) of approximately 60–70% of all human genes [6, 7], whereas, regions of low CpG density are frequently located outside the TSS [8, 9]. DNA methylation at gene promoters is important for transcriptional regulation, with dense promoter hypermethylation around the TSS often being associated with gene repression [10, 11]. In contrast, the hypomethylation of CpG sites has been associated with the overexpression of oncogenes within cancer cells [12].

However, in addition to gene promoters, distal regulatory regions such as enhancers, silencers and boundary elements are also often required to establish correct gene expression patterns in mammalian cells [13]. Transcription factors bind enhancers, which play key roles in the control of cell-type-specific gene expression [14–20]. A typical mammalian cell contains thousands of active enhancers, and it has been estimated that there may be ~1 million enhancers active in all human cells [21–23].

Enhancers play an important role in gene regulation and this is evident from mutations within enhancers that have the potential to generate a plethora of transcriptional alterations through both loss- and gain-of-function effects, leading to a gradient of phenotypic severities. A clear example of this is the dysregulation of *SHH* expression and limb malformations. *SHH* expression in a region of limb buds known as the zone of polarizing activity (ZPA) is necessary for limb patterning [24]. This expression pattern is governed by a long-range enhancer element about 1 Mb from *SHH*, known as the ZPA regulatory sequence (ZRS). Point mutations within ZRS have been linked to a congenital disease leading to extra digits known as preaxial polydactyly [25], whereas deletion of the entire ZRS in mice led to a truncation of limbs [26]. If point mutations within enhancers can cause a phenotypic change, then it is not surprising that an epigenetic change, such as DNA methylation, can also affect the activity of an enhancer and therefore cause a phenotypic change [27, 28].

Although it was originally thought that enhancers regulate only a single nearby promoter, many observations over the past 25 years point to a more complex interplay. Enhancers can control multiple neighboring genes [29–32], sometimes over hundreds of kb and often skipping one or more genes [33, 34]. With the advent of next-generation sequencing, there have been much interest in exploring the enhancer-promoter interactome across several cell types [35–38]. As it was previously shown that correlation between enhancer and promoter histone modification patterns can be used to infer their interactions [39], we therefore extended this approach and searched for well-correlated DNA methylation events between enhancers and genes that were unique to the CIMP/non-CIMP CRC phenotypes.

## RESULTS AND DISCUSSION

### Systematic mapping of differentially methylated regions (DMRs) and differentially methylated enhancers (DMEs) across 11 colorectal cancer cell lines

To explore the genome-wide DNA methylation state at single-base resolution across 11 CRC cells, we applied the method known as MethylCap-BS-seq, previously described by Brinkman *et al.* [40]. This involves the enrichment of methylated DNA by capturing with a monoclonal antibody against a methyl-DNA binding protein domain (MBD) followed by bisulfite conversion and deep sequencing to directly assess DNA methylation levels in captured chromatin fragments (see Methods). The 11 CRC cell lines include 6 CIMPs (Colo205, DLD1, HCT116, HT29, LIM2405, RKO) and 5 non-CIMPs (Caco-2, Colo320, LIM1215, SW403, SW480) [2, 41]. The sequence reads were mapped using Bismark [42] and DNA methylation profiles for the 11 cell lines were analyzed with the R package, methylKit [43].

In this study, we created 2 groups, namely, CIMP and non-CIMP, from the 11 cell lines and allowed methylKit to determine regions that are differentially methylated between the CIMP and non-CIMP groups through a logistic regression test [43]. Each differentially methylated region (DMR) identified by methylKit had a window size of 1000 bp. To ensure a very high level of stringency in the detection of the DMRs between the CIMP and non-CIMP, the limiting criteria for identifying these DMRs were that these regions would have a percent methylation difference of >50% with a q-value (i.e. FDR-adjusted) of <0.01. In total, 16,092 regions were discovered by methylKit to be differentially methylated between the CIMP and non-CIMP groups (Supplementary File 2).

We compared these 16,092 DMRs against 1729 experimentally validated human gene enhancers from VISTA Enhancer Browser [44] and found that 24 DMRs were found completely within 24 different gene enhancers.

Of the 24 enhancers identified by methylKit to contain a DMR, we wanted to prioritize and narrow down the field of 24 enhancers and only study those enhancers which we considered most important. How then do we decide which of the 24 enhancers were most important? The size of the enhancers were generally larger than the 1000 bp window that defined the DMRs. Therefore, we chose to calculate the exact methylation levels for the entire enhancer region for all 24 enhancers in all 11 CRC cell lines (Supplementary Table 1). Using the methylation values for each enhancer region in the 11 CRC cells, we performed a two-tailed t-test to ensure that there is a significant difference in methylation states of the 24 enhancers in the CIMP and non-CIMP cell lines (Supplementary Table 1). Out of the 24, only 3 enhancers were found to have a statistically significant difference (p<0.05) in methylation state between the CIMP and non-CIMP groups. These 3 human enhancer elements are **1702** [chr1:18,958,671-18,960,284 (GRCh37/hg19)], **285** (chr10:102,414,915-102,415,578) and **1944** (chr15:65,377,669-65,381,418). Thus, in this way, we chose 3 differentially methylated enhancers (DMEs) for further study. These 3 DMEs are hypermethylated in the CIMP cell lines and hypomethylated in the non-CIMP group (Supplementary Table 1).

### DMRs show significant correlation between enhancer and other gene regulatory regions

We paired each enhancer with all intrachromosomal DMRs and performed a Pearson Correlation test. For the enhancer element 1702, there were 2 DMRs (chr1:228194001-228195000 and chr1:240118001-240119000) with methylation states that correlated strongly with the enhancer's methylation state (Pearson Correlation Coefficient, -0.85 ≥ *r* ≥ 0.85, FDR adjusted *p*-value ≤ 0.05). Of these 2 DMRs, the region, chr1:228194001-228195000 (which we will refer to as “DC1A” from here on), was found to overlap the promoter of the *Wnt3A* gene (Table 1). We define here the promoter as the region 1500 bp upstream of the gene's transcription start site (TSS). The correlation of methylation states between element 1702 and DC1A is a positive one (Correlation coeffiecient, *r* = 0.853, FDR-adjusted *p*-value = 0.02) and therefore, DC1A, like 1702, is hypermethylated in the CIMP cell lines and hypomethylated in the non-CIMP group (Supplementary Table 2). The Wnt/beta-catenin signaling pathway has previously been associated with tumor progression in CRC [45–47] and therefore, it is not surprising to find this association of enhancer 1702 with the gene of a signaling pathway that initiates the carcinogenic process in CRC.

**Table 1:** Summary of information of related enhancer-DMR pairs

Enhancer (VISTA)	Chromo-some	Enhancer's Chromosomal Coordinates	DMR	DMR's Chromosomal Coordinates	Overlapping or Closest Gene (with/to DMR)	Gene Accession	Gene's Chromosomal Coordinates	Strand (Gene)	Distance of Enhancer from Gene TSS (bp)
1702	1	18958671- 18960284	DC1A	228194001- 228195000	Homo sapiens wingless-type MMTV integration site family, member 3A (*WNT3A*)	NM_033131	228194722- 228248972	+ve	209,236,051
1702	1	18958671- 18960284	DC1B	240118001- 240119000	Homo sapiens cholinergic receptor, muscarinic 3 (CHRM3)	NM_000740	239549876- 240078750	+ve	568,125
1944	15	65377669- 65381418	DC15A	65628001- 65629000	Homo sapiens immunoglobulin superfamily, DCC subclass, member 3 (*IGDCC3*)	NM_004884	65619464- 65670378	−ve	288,960

Enhancer-DMR pairs that have a high degree of correlation in their DNA methylation patterns are shown in this table. For the purpose of this study, we have annotated the differentially methylated regions (DMRs) as DC1A, DC1B and DC15A. Two DMRs, DC1A and DC15A, were found to overlap genes, *Wnt3A* and *IGDCC3*, respectively, while the third DMR, DC1B, was found to be in close proximity to the gene, CHRM3. DC1B is located downstream of CHRM3 and so, although it is 568,125 bp from CHRM3′s transcription start site (TSS), it is, in reality, only 39,251 bp from the 3′-end of this gene.

**Figure 1:**
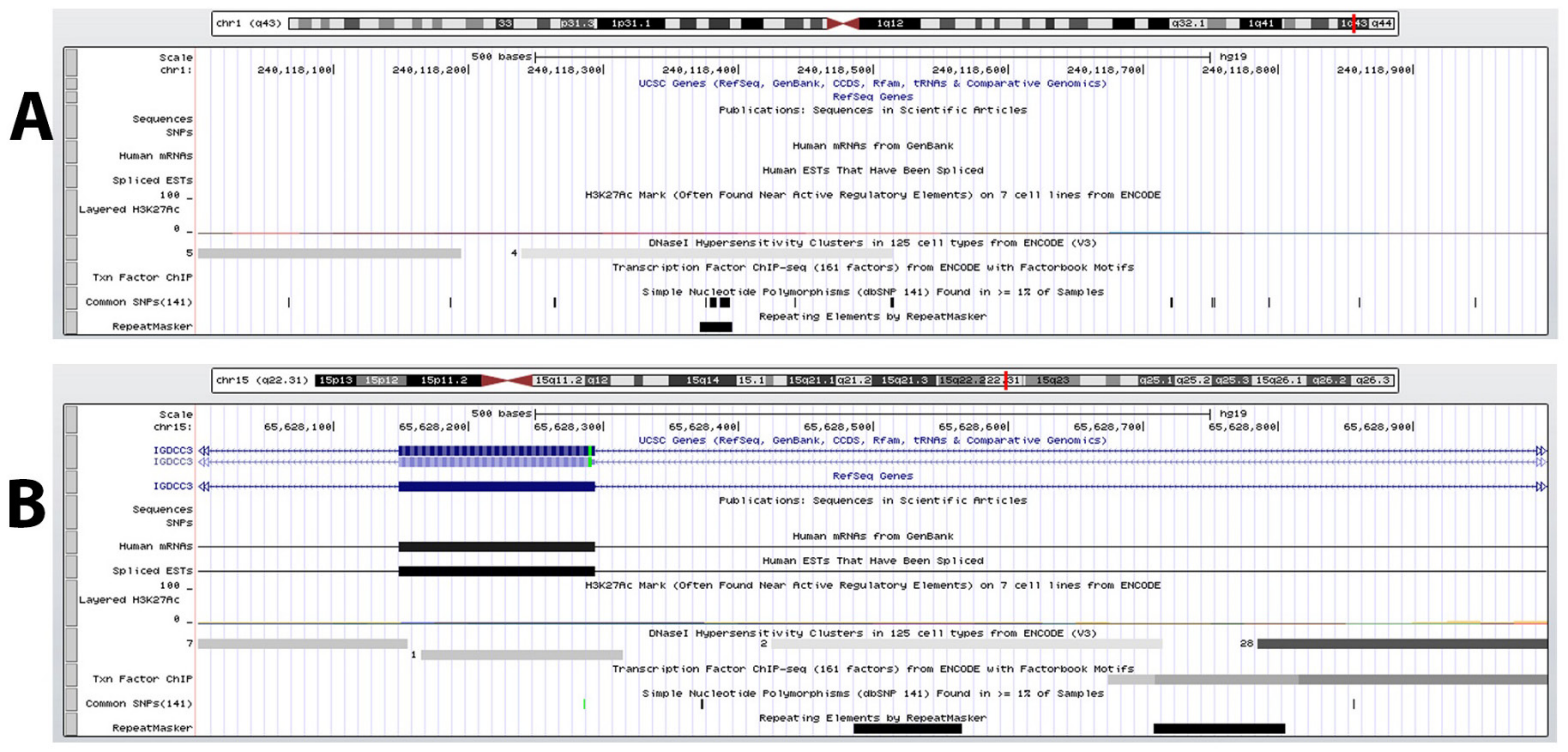
DNase hypersensitive sites identified within DMRs **A.** DC1B (chr1:240118001-240119000), identified as a DMR between CIMP and non-CIMP cells and located in an intergenic region downstream of the *CHRM3* gene, is shown to possess DNase hypersensitive sites in 9 separate cell types by the ENCODE Consortium. **B.** DC15A (chr15: 65628001-65629000) overlaps intron 3, exon 4 and intron 4 of the *IGDCC3* gene and has also be shown by ENCODE to be a highly DNase sensitive site by 38 cell types. Although both these regions are not located within a promoter region but these evidence indicate that DC1B and DC15A are regulatory DNA regions.

The second DMR (chr1:240118001-240119000) (which we will refer to as “DC1B”) also had a methylation state that correlated strongly with those of element 1702 (*r* = −0.855, FDR-adjusted p-value = 0.02) (Supplementary Table 2). However, DC1B is located in an intergenic region. Previous studies by the ENCODE Consortium have shown this to be a DNase hypersensitive area, an indication that this is a gene regulatory region since such regulatory regions tend to be DNase-sensitive [23, 48] (Figure 1A). Since the discovery of DNase hypersensitive sites 30 years ago, they have been used as markers of regulatory DNA regions [23]. In fact, Caco-2 is among the 9 cells that have shown this region to be DNase-sensitive and therefore suggesting that this is potentially a regulatory region in CRC cells. A closer look shows that DC1B is 39,251 bp downstream from the muscarinic 3 cholinergic receptor gene, *CHRM3* (Table 1). The human HT29 and H508 colon cancer cells have been shown to express *CHRM3* and this muscarinic receptor is believed to be responsible for CRC cell migration and invasion [49–51]. In a cell invasion model, acetylcholine-induced HT29 cell invasion could be blocked by atropine [49]. Interestingly, there is evidence to also suggest an interplay of Chrm3 and beta-catenin signaling which is responsible for intestinal mucosal differentiation and neoplasia [51].

The methylation state for enhancer element 1944 correlated well with only one intrachromosomal DMR (chr15: 65628001-65629000; which we will refer to as “DC15A”) (*r* = −0.949, FDR-adjusted p-value = 0.0019) (Supplementary Table 2). DC15A overlaps intron 3, exon 4 and intron 4 of the *IGDCC3* gene (Table 1). A detailed look at the methylation profile for the region covered by DC15A shows that most mapped reads are found between exon 4 and intron 4 (Supplementary Figure 1). Again, the ENCODE Consortium has identified *IGDCC3* exon 4 and intron 4 to contain DNase I hypersensitive sites (Figure 1B) [23, 48]. Moreover, this region was also found to contain binding sites for various transcription factors (namely, SIN3A, CTCF and RAD21) through ENCODE's transcription factor ChIP-seq. The relationship between methylation and gene expression is complex, with high levels of gene expression often associated with low promoter methylation [52] but elevated gene body methylation [53], and the causality relationships have not yet been determined. Therefore, this correlative relationship between element 1944 and DC15A may yet represent a means by which element 1944 exerts control of *IGDCC3* expression. *IGDCC3* (also known as *PUNC*) is most similar to the *Deleted in Colorectal Cancer* gene *DCC*, with 41, 42, and 47% identity in the second, third and fourth lg domains, respectively [54–56]. Interestingly, *IGDCC3* is also one of eight genes whose expression patterns strongly overlapped with known regions of high Wnt activity during embryonic development [57, 58].

For enhancer element 285, we could find no statistically significant correlations with any intrachromosomal DMRs.

### Recurring enhancer-gene methylation signatures found in an independent set of human CRCs

We reanalyzed data from a recent MethylCap-seq of tissues from 24 human patients with primary sporadic CRC (GEO Series: GSE39068) [59]. Here, we aligned the reads with Bowtie 2 [60] and used MEDIPS [61] for analysis of the data. We obtained the methylation levels of the regions covered by enhancers 1702 and 1944, and DC1A, DC1B and DC15A from MEDIPS and performed a Pearson Correlation test to validate the correlations that we observed in our group of 11 CRC cells.

The methylation levels of enhancer 1702 and DC1A again showed good correlation across the 24 human CRC tissues (*r* = 0.634, *p*-value = 0.00088) (Supplementary Table 3). This finding definitely supports our observation in the 11 CRC cell lines and suggests this correlated methylation pattern within CRC cells is maintained because there is a functional relationship between enhancer 1702 and DC1A (within the Wnt3A promoter). A previous study showed that correlation between enhancer and promoter histone modification patterns can be used to infer their interactions [39]. As such, it is possible that other correlated epigenetic changes, such as DNA methylation, between enhancers and promoters may also be indicative of their interaction, especially when such patterns are well conserved within a particular cell type. Enhancer 1702 and DC1A are separated by a distance of 209 Mb. For a long time, the greatest known distance between an enhancer (ZRS) and a gene (*Shh*) was about 1Mb [25] but more recently, lineage-specific enhancers were discovered about 2Mb from the *Myc* promoter [62]. There is increasing evidence to show that distal regulatory elements can function over long distances and even on a different chromosome from their target genes [38, 63, 64]. A closer look at the data from a recent ChIA-PET assay [38] shows that of the 40,000 RNA polymerase II-bound interaction pairs discovered in this study, at least about 250 interacting pairs of loci were separated by a distance of greater than 100 Mb in both embryonic and neural stem cells (FDR ≤ 0.05). Thus, although uncommon, such long-range interactions spanning more than 100 Mb are indeed possible. A second plausible explanation for the well-correlated methylation patterns of enhancer 1702 and DC1A could also be that enhancer 1702 controls the expression of a gene or set of genes that have a functional physiological relationship with Wnt3A and thus, to ensure a coordinated expression of functionally related genes, the regulatory elements for these genes are coordinately regulated by methylation.

The methylation levels of enhancer 1944 and DC15A showed good-to-moderate correlation across the 24 human CRC tissues (*r* = 0.493, *p*-value = 0.014) (Supplementary Table 3). DC15A is located within the gene body of *IGDCC3* and enhancer 1944 is 238,046 bp downstream of *IGDCC3*. Enhancer 1944′s proximity to the *IGDCC3* gene and its coordinated methylation with DC15A within *IGDCC3* leads us to believe that enhancer 1944 controls the transcriptional regulation of *IGDCC3*.

We found no correlation in the DNA methylation pattern between enhancer 1702 and DC1B across the 24 human CRC tissues (*r*=0.089, *p*-value = 0.679) (Supplementary Table 3). In short, of the 3 enhancer-gene pairs, we were able to validate all but one with a second independent dataset.

### Conservative motifs within enhancers and DMRs suggest functional dependencies

**Figure 2:**
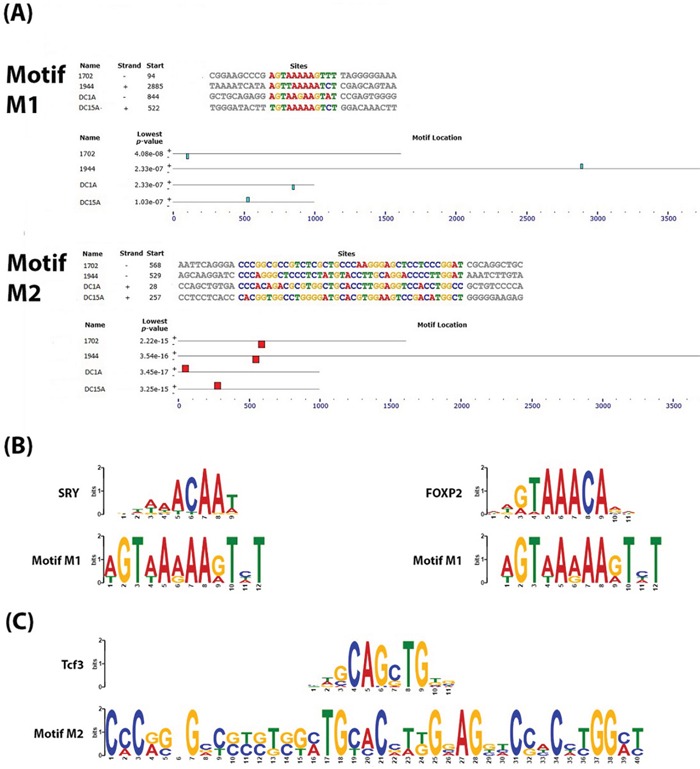
Conserved motifs identified within the DNA sequences of the enhancer-DMR pairs **A.** Output from an MEME analysis showing the relative location of 2 conserved motifs which we called “M1” and “M2” within the DNA sequences of the enhancers and DMRs. **B.** Output of TOMTOM analysis of M1 showing similarity of this motif to known validated transcription factor binding sites for SRY and FOXP2 (E-value >1) **C.** Output of TOMTOM analysis of M2 showing this motif to contain the transcription factor binding site for Tcf3 (E-value >1).

We analyzed the sequences of enhancers 1702 and 1944 along with DMRs, DC1A and DC15A, (see Supplementary File 1 for FASTA sequences) for conserved motifs because we believe that these loci with well-correlated DNA methylation patterns across colon cancer phenotypes must play a functional role in the transcriptional regulation of genes responsible for these phenotypes. Studies have previously shown that functionally-related genes are regulated through a unique combination of conserved transcription factor binding sites (TFBSs) [65–67]. Using MEME (Multiple EM for Motif Elicitation) [68], a Web service available on MEME suite [69], two motifs (*p*-values ranging from 2.33 × 10^−7^ to 3.45 × 10^−17^) were found consistently in all 4 sequences (Figure 2A). For each motif, we used TOMTOM [70] to search against the JASPAR CORE collection of vertebrate TFBS motifs [71] for similarities to known TFBSs. The results of TOMTOM offers an E-value - the expected number of times that the given query would be expected to match a target as well or better than the observed match in a randomized target database of the given size – and we chose a cutoff of E-value < 1, as previously suggested by Vandenbon *et al.* [67].

The first motif identified by MEME (which we will refer to as “M1”) is only 12 bp in length (Figure 2A). A detailed bioinformatics analysis of M1 using TOMTOM showed it to be a good match to the SRY (*p*-value = 0.0025; E-value = 0.52) and FOXP2 (*p*-value = 0.0044; E-value = 0.91) binding sites (Figure 2B). There is evidence to indicate that there is some interplay between FOXP2 and Wnt signaling [72, 73]. Promoters of genes of the Wnt signaling pathway were among 100 most significantly-enriched by FOXP2 ChIP-chip experiments performed on the SH-SY5Y neuronal cell line [73]. Conversely lef1, a member of the Lef/Tcf family of transcription factors activated by Wnt signaling, has also been shown to regulate *FOXP2* during embryogenesis, and novel *FOXP2* enhancers were also found to be lef1-dependent [72]. There are also several studies showing that SRY can antagonize the Wnt/beta-catenin transcriptional activities and repress Wnt target genes during differentiation and development [74–76]. It may be possible that SRY or FOXP2 binds to this site under different cellular conditions to elicit either a repression or expression of Wnt signaling components and other functionally-related genes that contribute to the CIMP or non-CIMP phenotype in CRC.

The second motif (which we will call “M2”) is 40 bp long (Figure 2A). An analysis with TOMTOM shows that this motif contains a putative binding site for Tcf3 (*p*-value = 0.0029; E-value = 0.59) (Figure 2C). Tcf3 is believed to play a role in Wnt-stimulated self-renewal of pluripotent mouse embryonic stem (ES) cells because it was found to bind the same genes as the core stem cell self-renewal circuit transcription factors, Oct4, Sox2 and Nanog [77]. It is generally accepted that the accumulation of stabilized beta-catenin in the presence of the Lef/Tcf family of transcription factors results in their translocation to the nucleus where they activate Wnt-responsive genes [78]. A recent study showed that genetic ablation of Tcf3 in ES cells replaced the requirement of exogenous Wnt3A for self-renewal of ES cells, which the authors suggest demonstrates that inhibition of Tcf3-repressor is the downstream effect of Wnt signaling [79]. Our analysis, however, suggest that Tcf3 may also be able to act upstream through its binding of the Wnt3A promoter and other related enhancers to affect the expression of Wnt3A and other physiologically-related genes. The role of Wnt3A in stimulating self-renewal (shown in pluripotent ES cells) could also therefore suggest its role in tumorigenesis in CRC.

## CONCLUSION

We took a novel approach to analyze DNA methylation patterns in CRCs and our analysis revealed a strong correlation in DNA methylation patterns between specific enhancer-gene pairs that are distinct between phenotypes. To the best of our knowledge, this is the first study to show specific DNA methylation of enhancers and genes that are coordinately regulated in a cell-specific manner. We observed that both element 1702 and DC1A are more highly methylated in CIMP than in non-CIMP cells. DC1A lies within the promoter of *Wnt3A*. On the other hand, the DNA methylation pattern for enhancer 1944 and DC15A is inversely related: in CIMP cells, enhancer 1944 is highly methylated and DC15A is unmethylated while the opposite is true in non-CIMP cells. DC15A lies with the gene body of *IGDCC3*, spanning specifically, intron 3, exon 4 and intron 4. So, what does all this mean? The relationship between methylation and gene expression is complex, with high levels of gene expression often associated with low promoter methylation [52] but elevated gene body methylation [53]. Thus, we postulate from these DNA methylation patterns that perhaps *Wnt3A* and *IGDCC3* are lowly or not expressed in the CIMP phenotype. *IGDCC3* (also known as *PUNC*) is most similar to the *Deleted in Colorectal Cancer* gene *DCC*, with 41, 42, and 47% identity in the second, third and fourth lg domains, respectively [54–56]. Interestingly, *IGDCC3* is also one of eight genes whose expression patterns strongly overlapped with known regions of high Wnt activity during embryonic development [57, 58]. Thus, it is perhaps not surprising if the expression of *IGDCC3* is closely regulated with *Wnt3A* in CRCs with low expression seen in CIMP phenotype and high expression observed in non-CIMP phenotypes.

Enhancer 1702 and *Wnt3A* are separated by a distance of more than 200 Mb while enhancer 1944 is 238 Kb downstream of *IGDCC3*. As these specific enhancer-gene pairs are coordinately regulated through DNA methylation, it would be natural to ask if these enhancers are directly responsible for regulating these genes. The record for the greatest distance between an enhancer and a gene was held by the ZRS enhancer and *Shh* gene (about1Mb) [25] until recently, when lineage-specific enhancers were discovered about 2Mb from the *Myc* promoter [62]. There is increasing evidence to show that distal regulatory elements can function over long distances and even on a different chromosome from their target genes [38, 63, 64]. A close look at the data from a recent ChIA-PET assay [38] shows that of the 40,000 RNA polymerase II-bound interaction pairs discovered, at least about 250 interacting pairs of loci were separated by a distance of greater than 100 Mb in both embryonic and neural stem cells (FDR ≤ 0.05). Thus, although uncommon, such long-range interactions spanning more than 100 Mb are indeed possible. A second plausible explanation for the well-correlated methylation patterns could be that enhancer 1702 and 1944 control the expression of a gene or set of genes that have a functional relationship with *Wnt3A* and *IGDCC3*, respectively. Thus, to ensure a coordinated expression of functionally related genes, the regulatory elements for these genes are coordinately regulated by methylation.

Normally, enhancer regions are predicted by DNAse I hypersensitivity. However, it generally requires a considerable effort to identify just what genes are regulated by these enhancers. If the former argument is true and enhancer 1702 does regulate the expression of *Wnt3A*, then this would be a very exciting find not only because it would be the longest known distance between an interacting enhancer and gene but it also means that by studying the correlation of epigenetic changes between enhancers and genes, one would be able to elucidate the interaction of enhancer-gene pairs.

Gene expression is commonly used as a biomarker to identify various clinical phenotypes. Current classification of CIMP/non-CIMP is based on either the CIMP panel suggested by Issa [80] or by Weisenberger et al. [81] and both panels have selected a subset of genes whose methylation marks are supposed to represent the CIMP phenotype. Through this study, we observe that enhancer-gene pair methylation can also be an effective method for delineating phenotypes and therefore, can be used as a clinical biomarker. The various panels of methylation markers introduced to delineate CIMP and non-CIMP [2, 3, 80, 81] don't always agree on what is CIMP and what is non-CIMP since the methylation markers that were selected for each panel are slightly different. Furthermore, there is also the problem of “grey areas” when not all the markers on a panel are found to be methylated. Not surprisingly, using a combination of two features (the methylation of the enhancer and of the gene) may prove to be a much better predictor of an outcome than using just one feature. We show that by looking at just the methylation level of 1702 and Wnt3A and/or 1944 and IGDCC3, these methylation signatures can potentially be used to delineate CIMP from non-CIMP CRCs.

Finally, mutations in APC and other genes of the Wnt pathway are characteristics of the chromosomal instability (CIN) phenotype of CRC [82]. Here, our results suggest that the Wnt pathway is also responsible in defining the CIMP/non-CIMP phenotype through epigenetic events involving *Wnt3A*. Up until now, involvement of the Wnt pathway in CRC has only been suggested for the CIN phenotype.

## MATERIALS AND METHODS

### Cell Line DNA samples

DNA samples from LIM1215 and LIM2405 colorectal cancer cell lines were provided by John Mariadason. The remaining DNA samples were extracted from cell lines obtained from American Type Culture Collection (Manassas, VA) and cultured under recommended conditions. Genomic DNA was extracted using the DNeasy Blood and Tissue Kit (Qiagen, Hilden, Germany) according to manufacturer's instructions.

### Methyl-binding domain-bisulfite DNA sequencing (MethylCap-BS-seq)

Methylated DNA was captured using the MethylCap kit (Diagenode, Liege, Belgium) according to manufacturer's instructions. Briefly, 2 ug of genomic DNA was randomly sheared by sonication using a Bioruptor UCD-200 (Diagenode) to generate DNA fragments peaking around 300bp. DNA was incubated with H6-GST-MBD (Methyl-DNA Binding Domain) protein and rotated at 4oC for 2 hours before adding magnetic beads. Following an additional one hour incubation, the beads were isolated and subjected to serial washing before the captured methylated DNA was eluted from the beads with low-salt, medium-salt and high-salt buffers in the kit. The medium salt and high salt eluate was combined for each sample and used for bisulfite sequencing. To construct a DNA library for bisulfite sequencing, the Illumina ChIP seq sample preparation kit (Illumina, San Diego, CA) with modification was used. Briefly, methylation enriched DNA was treated as per manufacturer's instruction before ligation with methylated adaptor oligonucleotides (Illumina) instead of the regular unmethylated adaptor oligonucleotides in the kit. DNA was then bisulfite treated using the EZ DNA methylation-gold kit (Zymo Research, Irvine, CA) before being amplified using Illumina PE 1.0 and PE 2.0 primers for 14 cycles. Constructed libraries were quantified using the Illumina library quantification qPCR protocol and sequenced on Genome Analyzer IIx (Illumina) at 36bp.

### Bisulfite-treated DNA methylation sequence analysis

**Figure 3:**
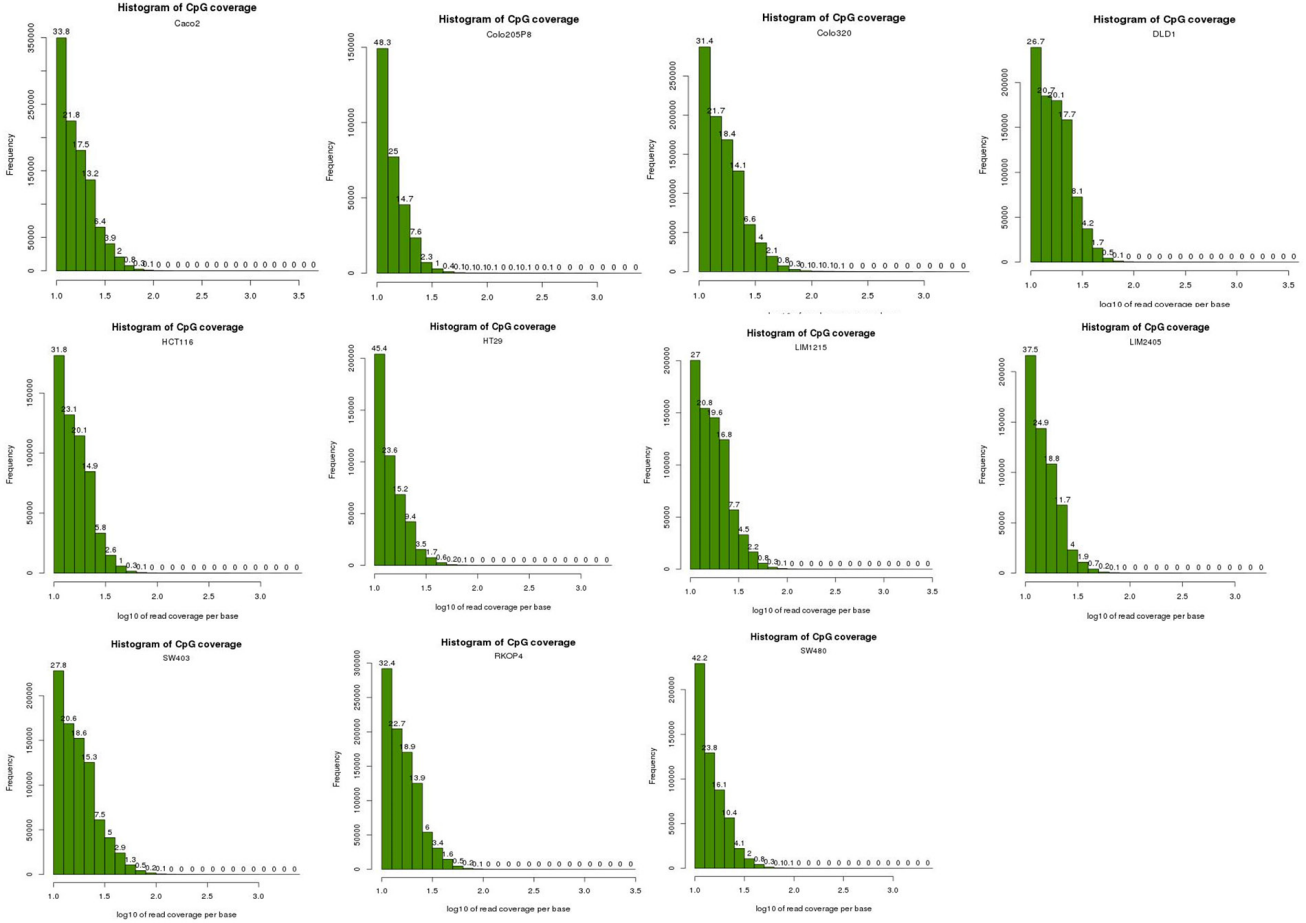
Histogram plots of read coverage per base for the 11 cell lines These histogram plots show that the NGS data for the 11 cell lines do not suffer from PCR amplification bias. Experiments that suffer from PCR duplication bias will have a secondary peak on the right-hand side of the histogram. As no secondary peak is observed in our data for all 11 cell lines, this indicates that our data do not suffer from PCR duplication bias.

Sequencing reads were filtered from the adapter sequences using the wrapper script, Trim Galore! [83] which automates quality and adapter trimming through the use of Cutadapt [84] and FastQC [85]. We performed bisulfite-treated read alignment to hg19 genome using the Bismark Bisulfite Mapper (v0.13.0) alignment software with default parameters, with only uniquely mapped reads kept for DNA methylation calling [42]. The reads from the 11 cell lines were divided into two groups of 6 CIMPs (Colo205, DLD1, HCT116, HT29, LIM2405, RKO) and 5 non-CIMPs (Caco-2, Colo320, LIM1215, SW403, SW480) and input to methylKit [43]. Sequence reads were not de-duplicated to remove potential PCR duplicates because a histogram plot of read coverage per base for each of the 11 cells (Figure 3) indicate that there is no PCR duplication bias present - experiments suffering from PCR duplication bias will have a secondary peak towards the right hand side of the histogram. Furthermore, Bainbridge et al. [86] had previously demonstrated in their study that de-duplicating reads that share the same coordinates could result in a loss of as much as 20% of actual reads that do not represent PCR duplicates for single-ended reads. In addition, a study reported by Illumina (unpublished) suggests that PCR duplicates may represent as little as 1% of all reads. In this study, scientists at lllumina prepared paired-end libraries using Illumina MID-tagged adapters, but instead of a finite set of known MID sequences, these adapters were constructed with random bases where the barcode would be. Thus, for each cluster they had three data points to compare: reads 1 and 2 and their respective alignment positions on the reference genome, plus the random 6bp sequence in the MID position. A read would need to match all three of these to be called a PCR duplicate. When they added these random tags, they found that the number of identified duplicates dropped from 8% to ~1%. Thus, based on our analysis and these studies, we chose not to de-duplicate the reads in our data. methylKit models the methylation per CpG site within a logistic regression between the CIMP and non-CIMP groups. A sliding linear model (SLIM) method is used to determine q-values from *p*-values to correct for multiple hypothesis testing [87] and thus, enabled us to determine the statistically significant DMRs (1000bp windows) between these 2 groups of cells. Methylation values for CpG dinucleotides are calculated by dividing number of methylated Cs by total coverage on that base. The methylation value calculated in this way is sometimes referred to as the beta-value (β-value). Methylation levels for DMRs or enhancers were calculated by taking the mean methylation of CpG dinucleotides within these regions. Intrachromosomal enhancer-to-DMR Pearson Correlation and other statistical calculations were performed in Microsoft Excel. We judged the strength of the correlation based on the following scale: r from +0.5 to +1.0 and from -0.5 to -1.0 constitutes a good to excellent correlation, while r from ±0.49 to 0 was considered moderate to nil. *p*-values were FDR-adjusted with the p.adjust package in R.

### MethylCap-seq DNA methylation analysis

We downloaded the MethylCap-seq data for 24 human patients with primary sporadic CRC (GEO Series: GSE39068) [59] from Gene Expression Omnibus (GEO). The short reads from this study was aligned with Bowtie 2 [60] against the hg19 genome and we used MEDIPS [61] to obtain the methylation levels (measured by reads per kilobase per million mapped reads, rpkm) of the desire regions. Bowtie 2/MEDIPS was used in this case because these are not bisulfite-treated sequences and it would be inappropriate to use the Bismark/methylKit method. Again, Pearson Correlation calculations were performed in Microsoft Excel.

### Motif analysis

The DNA sequence for enhancers 1702 and 1944 along with DMRs, DC1A and DC15A, were entered into MEME (Multiple EM for Motif Elicitation) [68], a Web service available on MEME suite [69]. MEME can be used to discover motifs in a group of related nucleotide or peptide sequences. A MEME motif is a sequence pattern that occurs repeatedly in one or more sequences in the input group. MEME can be used to discover novel patterns because it bases its discoveries only on the input sequences and not on any prior knowledge (such as databases of known motifs). In this case, only motifs that were present in all 4 DNA sequences were taken for further analysis in TOMTOM [70]. TOMTOM was used to search against the JASPAR CORE collection of vertebrate TFBS motifs [71] for similarities of the motifs identified by MEME, M1 and M2, to known TFBSs.

## SUPPLEMENTARY FIGURES AND TABLES












