# Supplementary material for: Distinct epigenetic signatures elucidate enhancer-gene relationships that delineate CIMP and non-CIMP colorectal cancers
Source: Oncotarget. 2016 Mar 30;7(19):28027–39. doi: 10.18632/oncotarget.8473 (PMC5053707; doi:10.18632/oncotarget.8473)
Supplement: Supplementary file 1 [file oncotarget-07-28027-s001.pdf]

SUPPLEMENTARY FIGURE AND TABLES

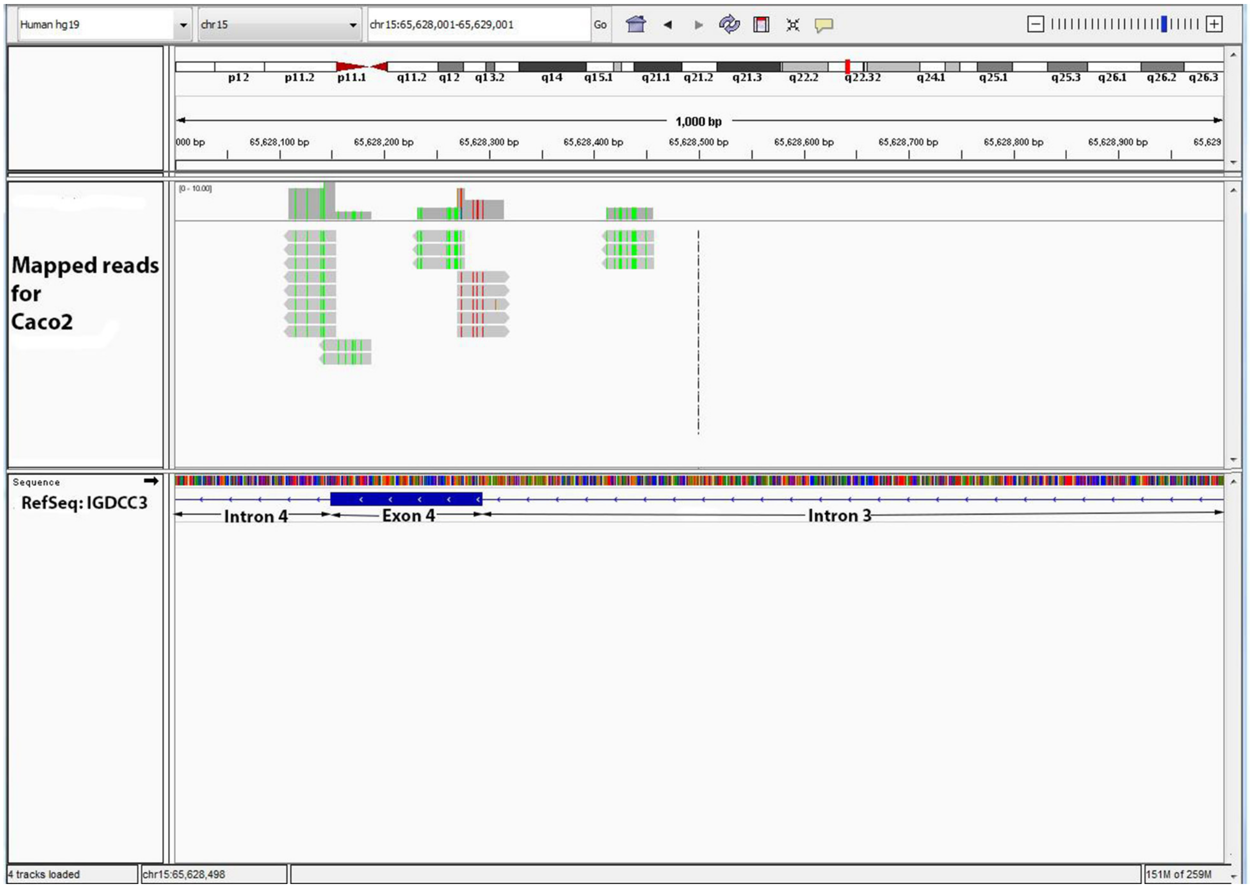

(*Continued*)

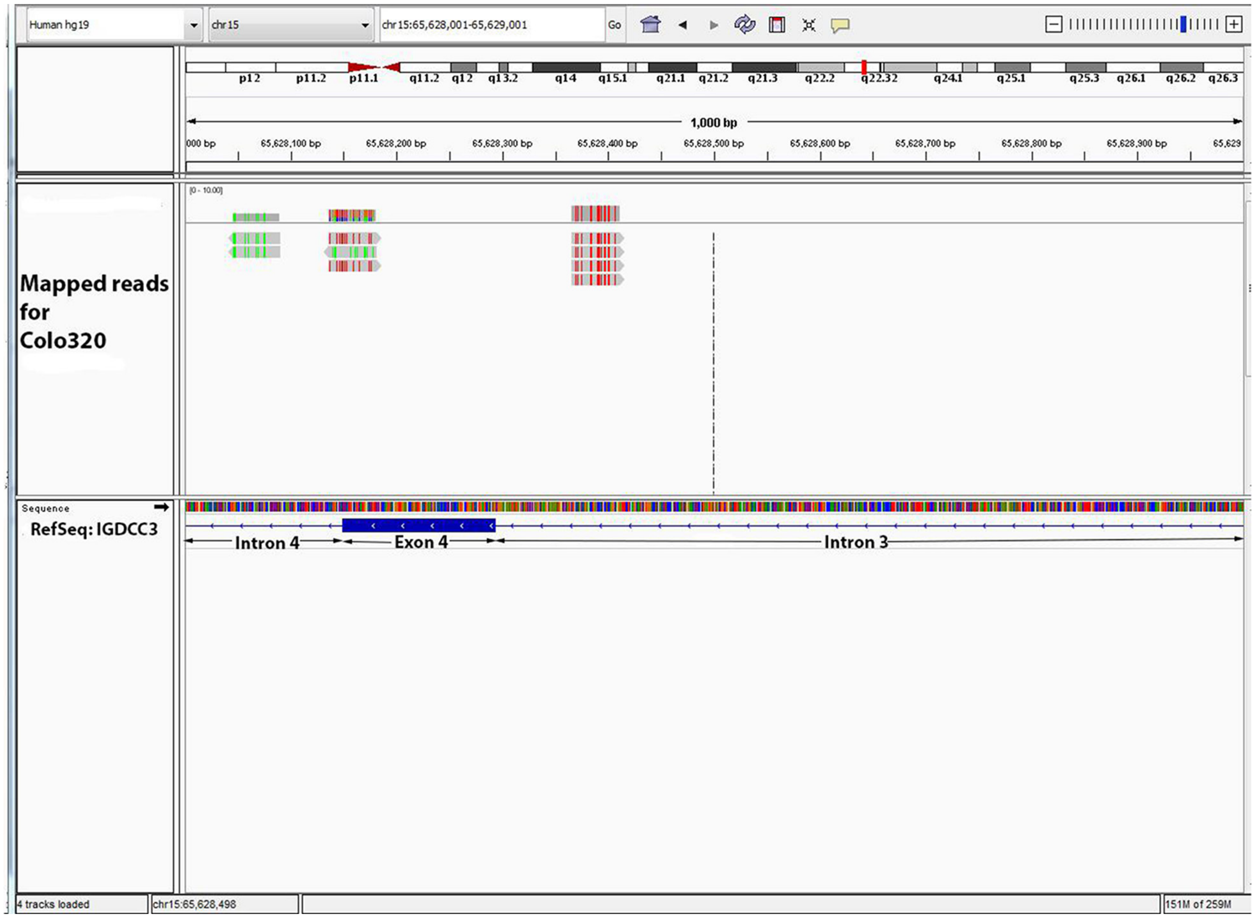
Human hg19
chr15
chr15:65,628,001-65,629,001
Go
p12 p11.2 p11.1 q11.2 q12 q13.2 q14 q15.1 q21.1 q21.2 q21.3 q22.2 q22.32 q24.1 q25.1 q25.3 q26.1 q26.2 q26.3
1,000 bp
000 bp 65,628,100 bp 65,628,200 bp 65,628,300 bp 65,628,400 bp 65,628,500 bp 65,628,600 bp 65,628,700 bp 65,628,800 bp 65,628,900 bp 65,629
Mapped reads for Colo320
Sequence
RefSeq: IGDCC3
Intron 4
Exon 4
Intron 3
4 tracks loaded
chr15:65,628,498
151M of 259M

(*Continued*)

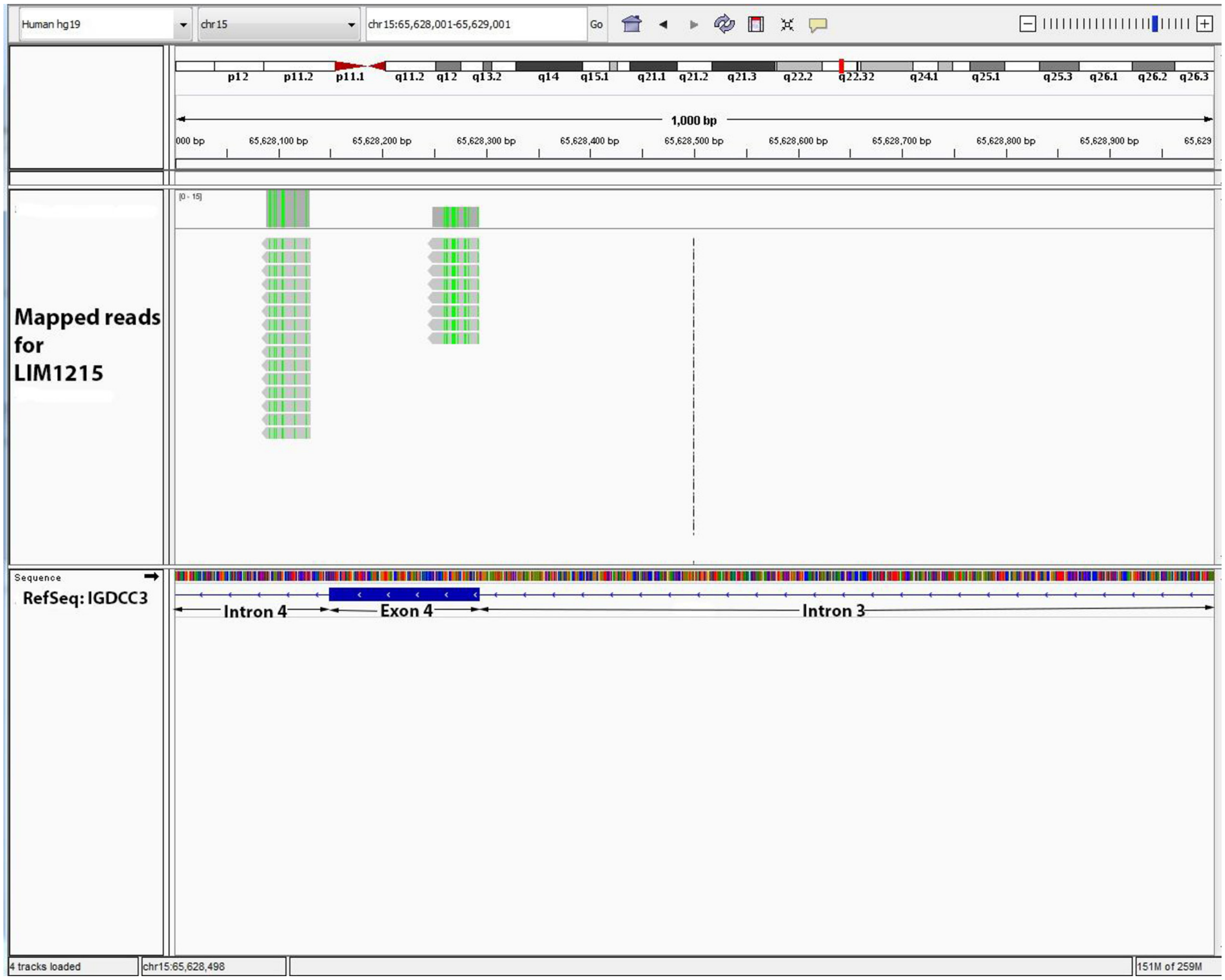
Human hg19
chr15
chr15:65,628,001-65,629,001
Go
1,000 bp
Mapped reads for LIM1215
Sequence
RefSeq: IGDCC3
Intron 4
Exon 4
Intron 3
4 tracks loaded
chr15:65,628,498
151M of 259M

(*Continued*)

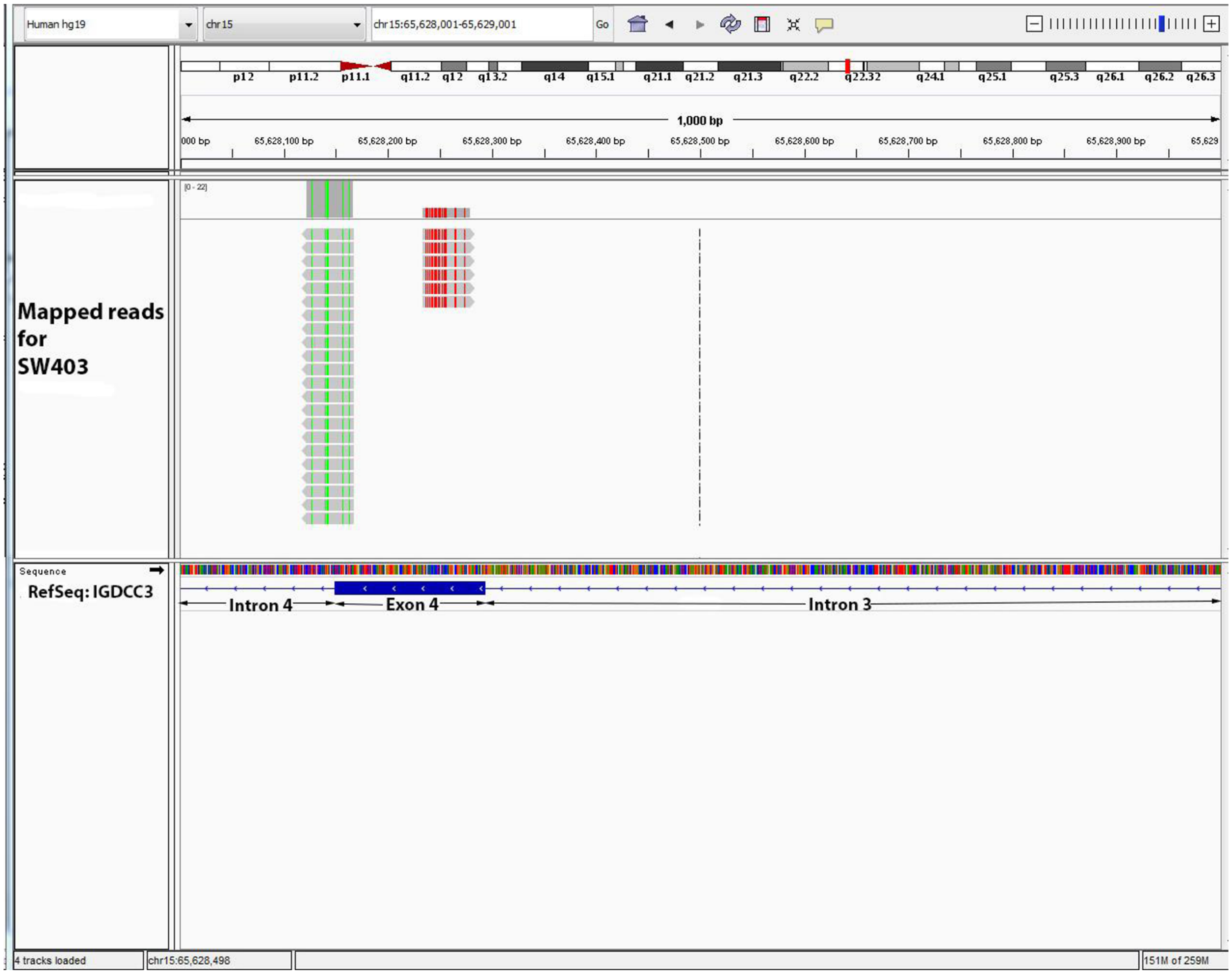
Human hg19
chr15
chr15:65,628,001-65,629,001
Go
p12 p11.2 p11.1 q11.2 q12 q13.2 q14 q15.1 q21.1 q21.2 q21.3 q22.2 q22.32 q24.1 q25.1 q25.3 q26.1 q26.2 q26.3
1,000 bp
000 bp 65,628,100 bp 65,628,200 bp 65,628,300 bp 65,628,400 bp 65,628,500 bp 65,628,600 bp 65,628,700 bp 65,628,800 bp 65,628,900 bp 65,629
Mapped reads for SW403
[0 - 22]
Sequence
RefSeq: IGDCC3
Intron 4
Exon 4
Intron 3
4 tracks loaded
chr15:65,628,498
151M of 259M

(*Continued*)

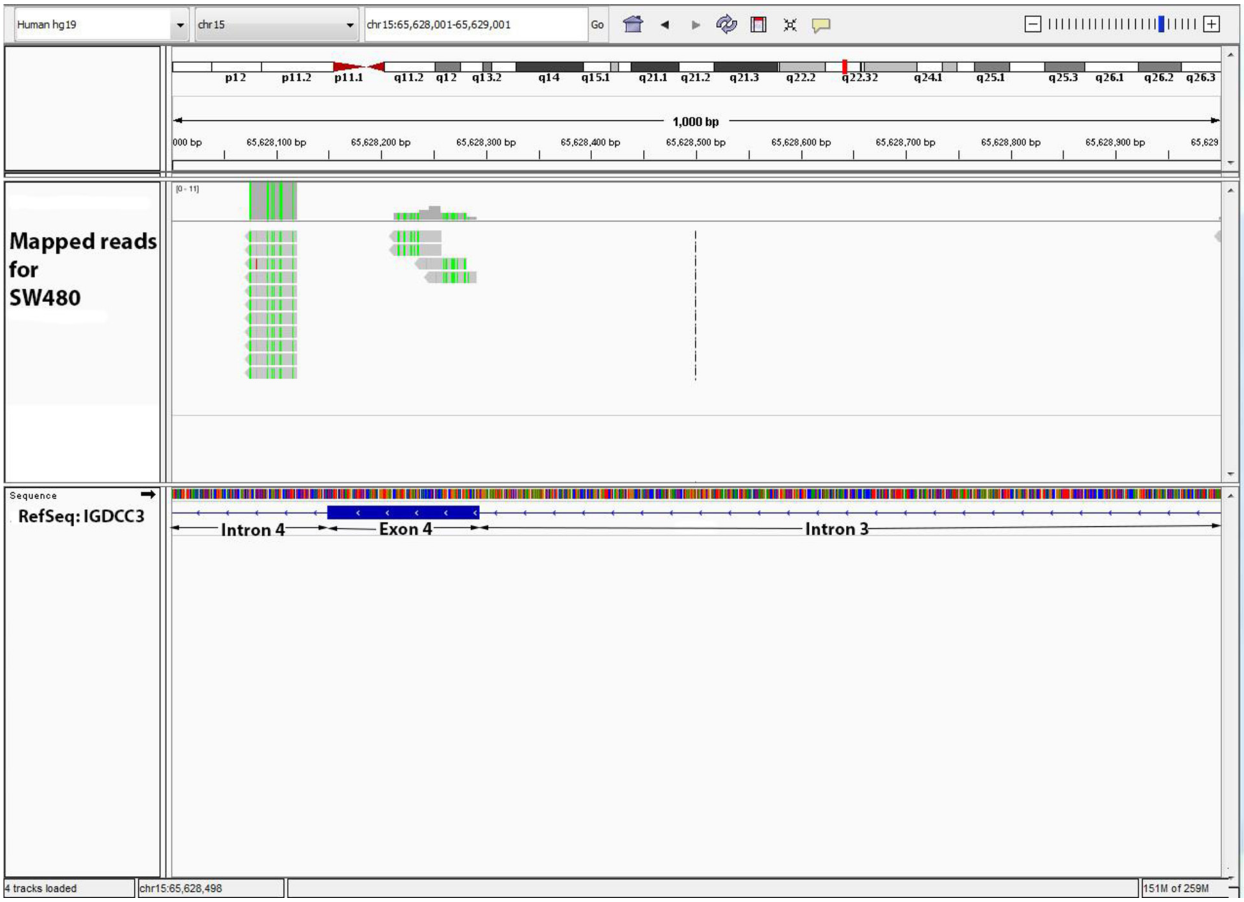
Human hg19
chr15
chr15:65,628,001-65,629,001
Go
1,000 bp
Mapped reads for SW480
Sequence
RefSeq: IGDCC3
Intron 4
Exon 4
Intron 3
4 tracks loaded
chr15:65,628,498
151M of 259M

(*Continued*)

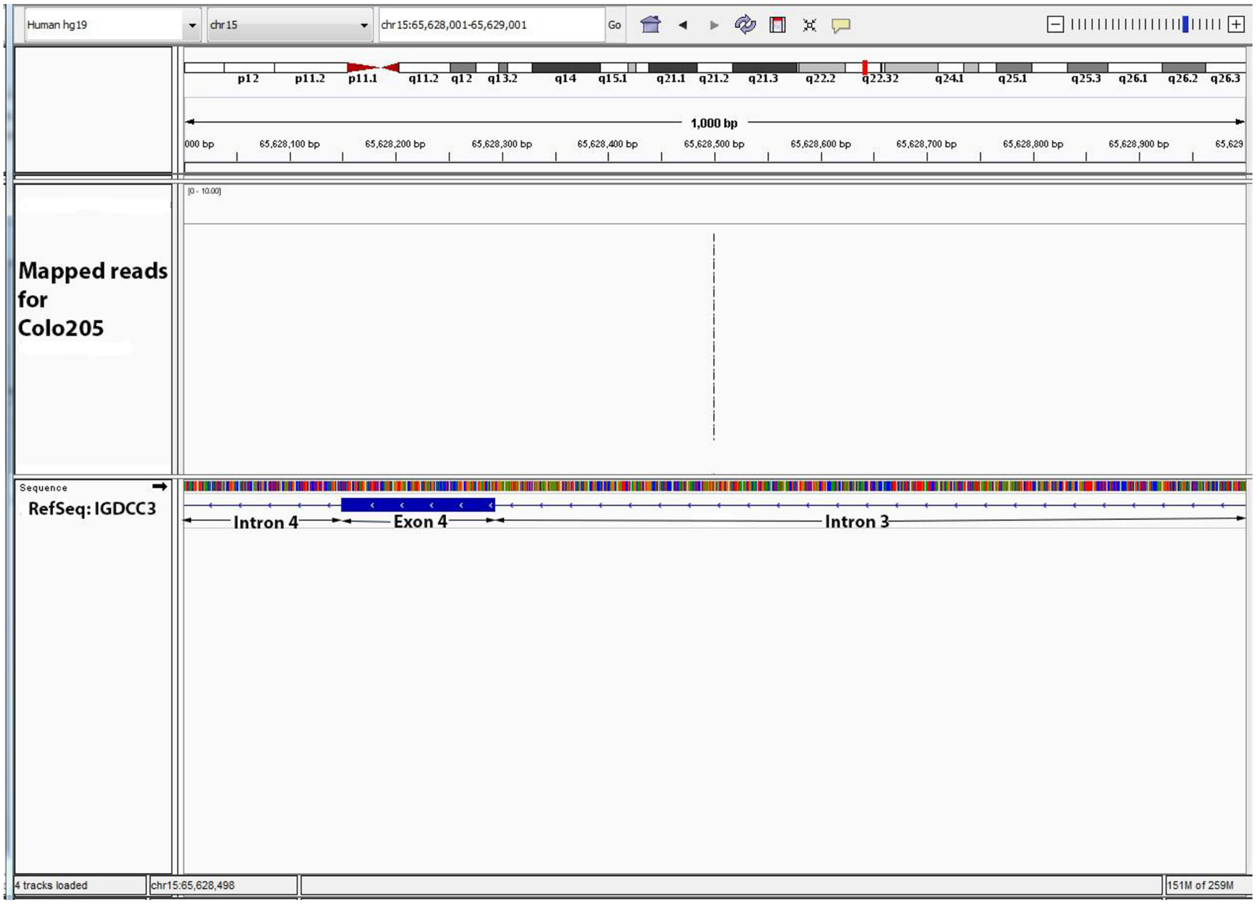
Human hg19
chr15
chr15:65,628,001-65,629,001
Go
p12 p11.2 p11.1 q11.2 q12 q13.2 q14 q15.1 q21.1 q21.2 q21.3 q22.2 q22.32 q24.1 q25.1 q25.3 q26.1 q26.2 q26.3
1,000 bp
Mapped reads for Colo205
Sequence
RefSeq: IGDCC3
Intron 4
Exon 4
Intron 3
4 tracks loaded
chr15:65,628,498
151M of 259M

(*Continued*)

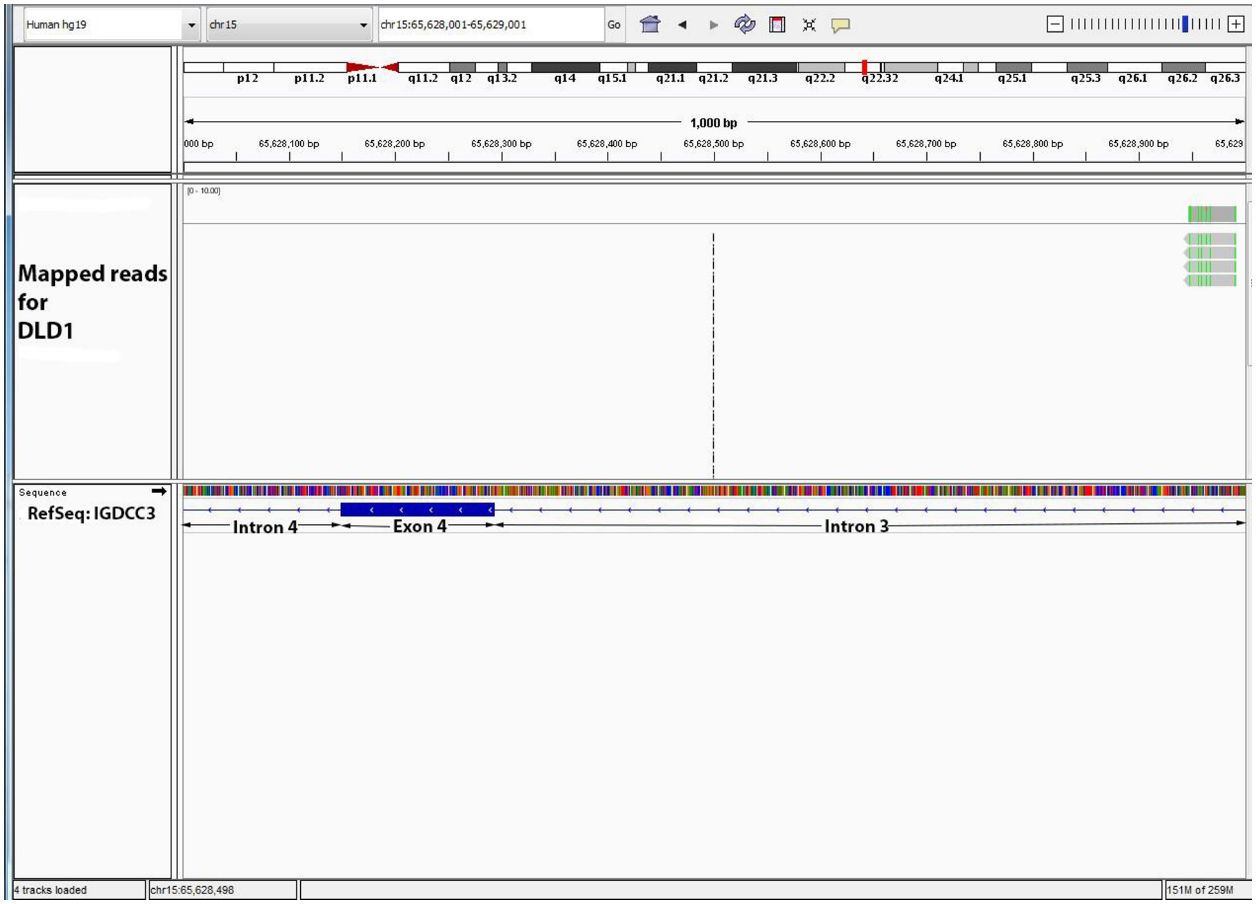
Human hg19
chr15
chr15:65,628,001-65,629,001
Go
p12 p11.2 p11.1 q11.2 q12 q13.2 q14 q15.1 q21.1 q21.2 q21.3 q22.2 q22.32 q24.1 q25.1 q25.3 q26.1 q26.2 q26.3
1,000 bp
000 bp 65,628,100 bp 65,628,200 bp 65,628,300 bp 65,628,400 bp 65,628,500 bp 65,628,600 bp 65,628,700 bp 65,628,800 bp 65,628,900 bp 65,629
[0 - 10.00]
Mapped reads for DLD1
Sequence
RefSeq: IGDCC3
Intron 4
Exon 4
Intron 3
4 tracks loaded
chr15:65,628,498
151M of 259M

(*Continued*)

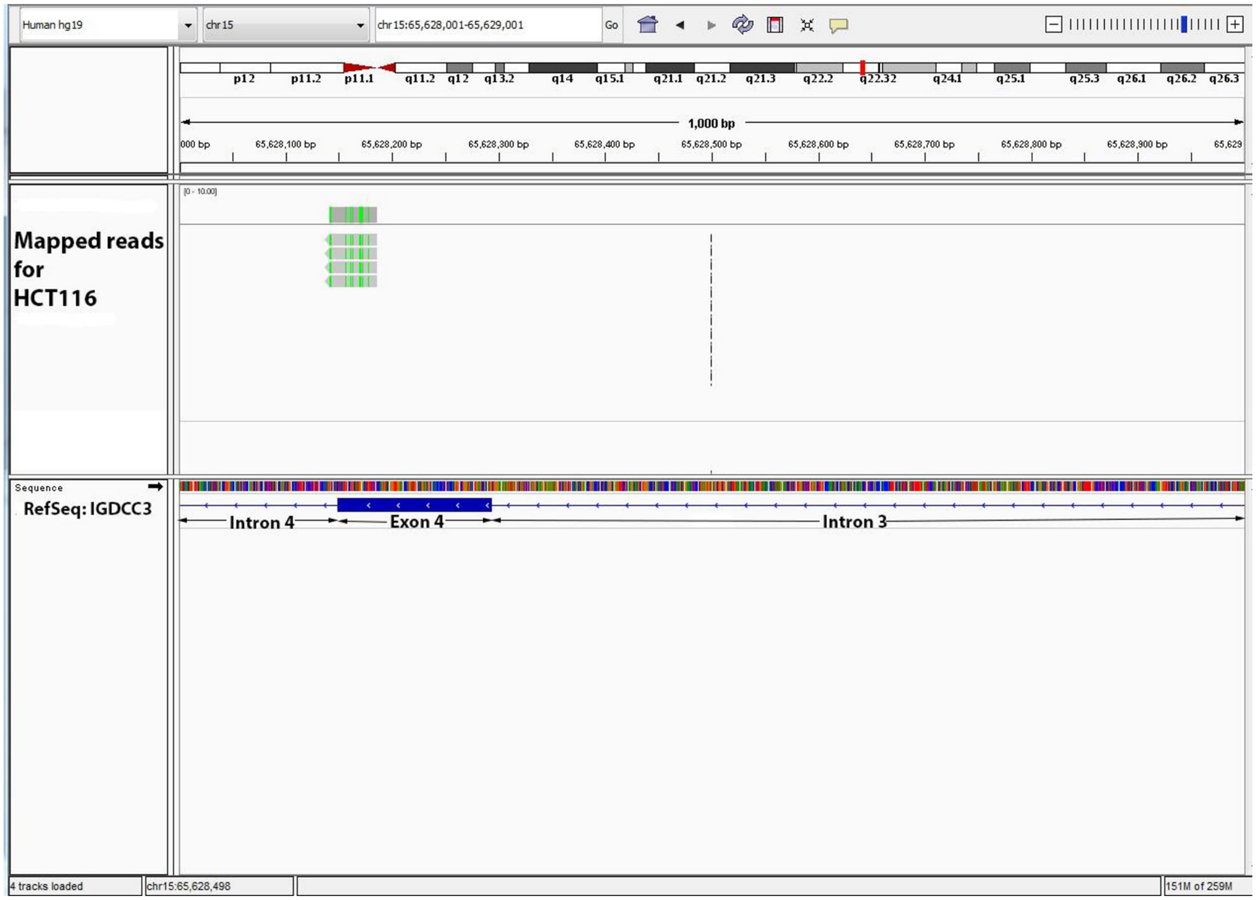
Human hg19
chr15
chr15:65,628,001-65,629,001
Go
1,000 bp
Mapped reads for HCT116
Sequence
RefSeq: IGDCC3
Intron 4
Exon 4
Intron 3
4 tracks loaded
chr15:65,628,498
151M of 259M

(*Continued*)

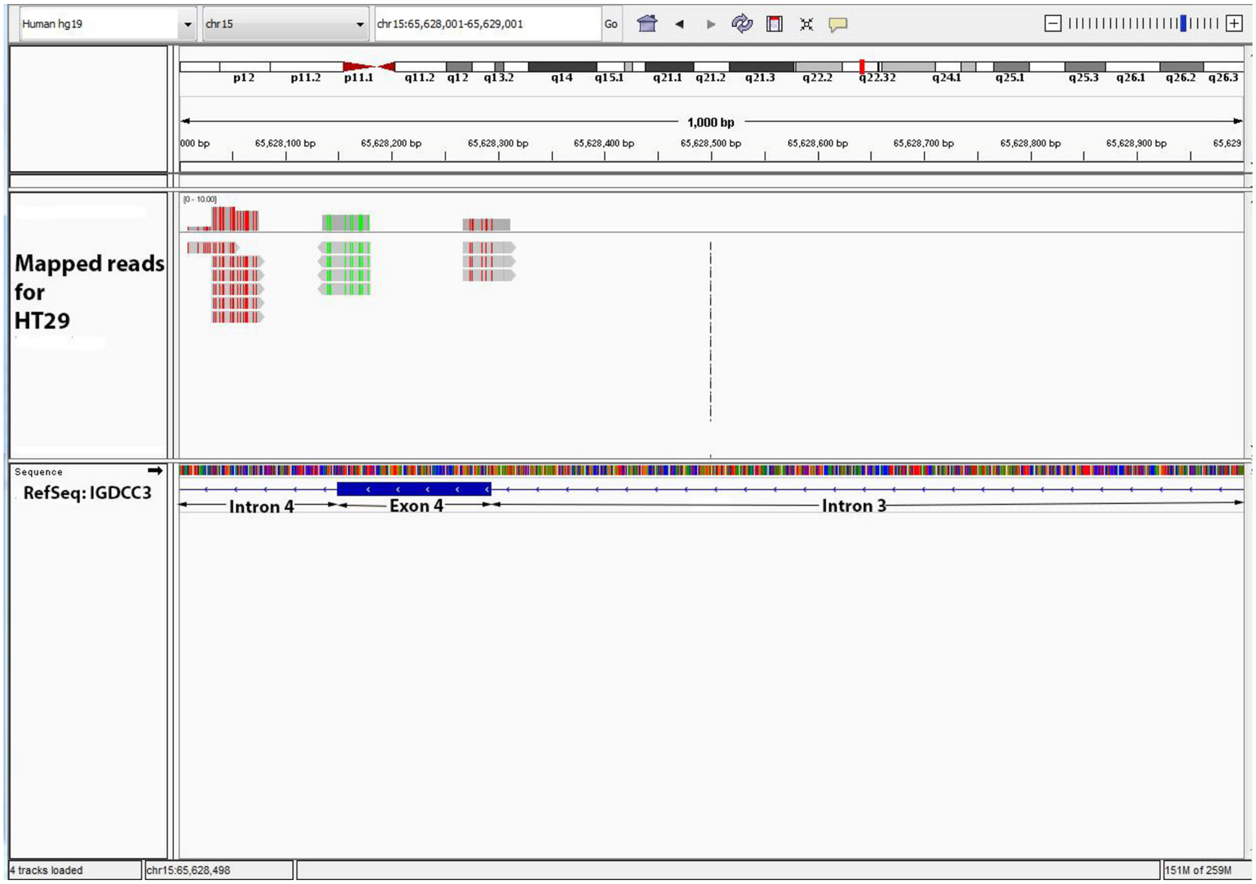
Human hg19
chr15
chr15:65,628,001-65,629,001
Go
p12 p11.2 p11.1 q11.2 q12 q13.2 q14 q15.1 q21.1 q21.2 q21.3 q22.2 q22.32 q24.1 q25.1 q25.3 q26.1 q26.2 q26.3
1,000 bp
000 bp
65,628,100 bp
65,628,200 bp
65,628,300 bp
65,628,400 bp
65,628,500 bp
65,628,600 bp
65,628,700 bp
65,628,800 bp
65,628,900 bp
65,629
Mapped reads for HT29
[0 - 10.00]
Sequence
RefSeq: IGDCC3
Intron 4
Exon 4
Intron 3
4 tracks loaded
chr15:65,628,498
151M of 259M

(*Continued*)

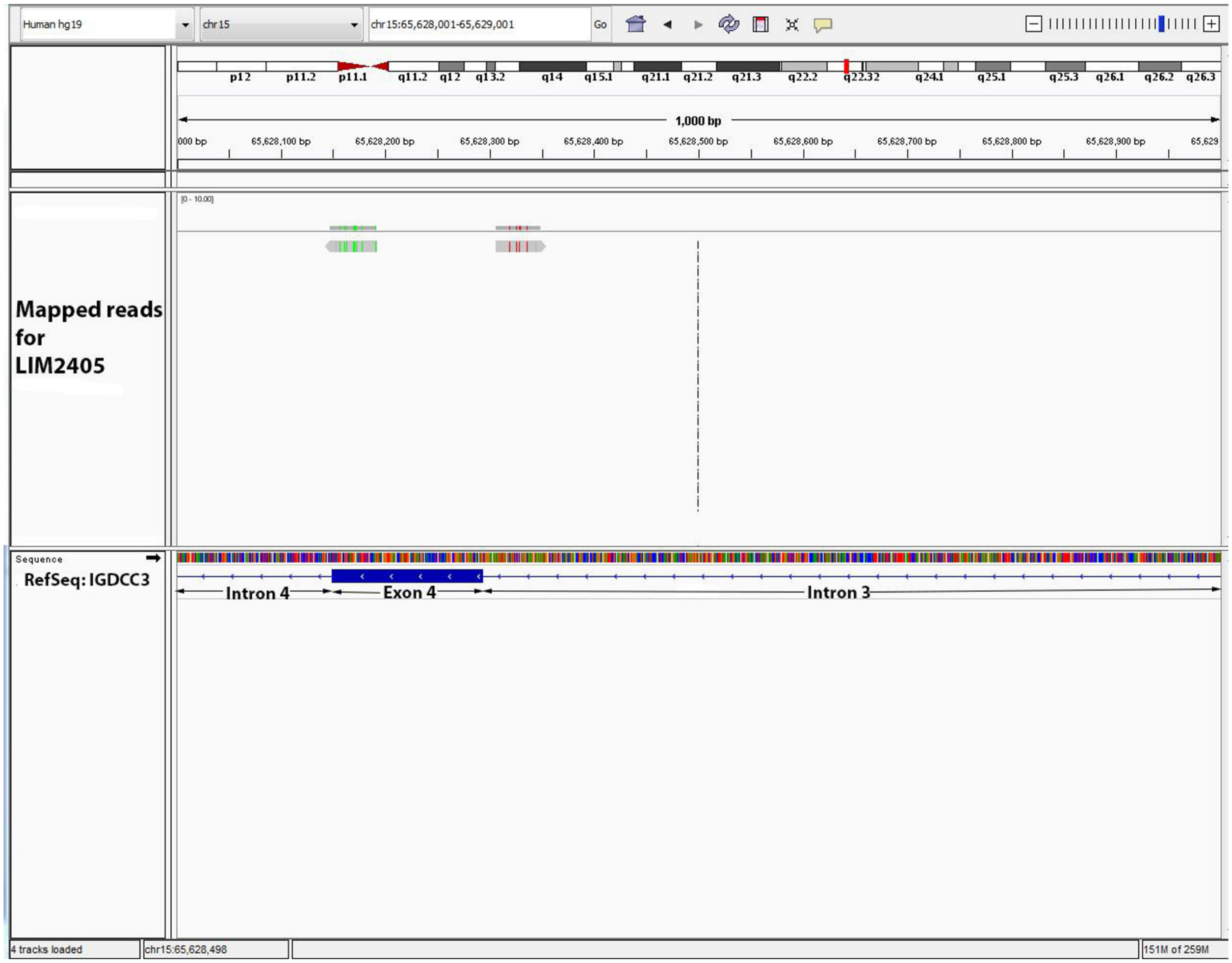
Human hg19
chr15
chr15:65,628,001-65,629,001
Go
p12
p11.2
p11.1
q11.2
q12
q13.2
q14
q15.1
q21.1
q21.2
q21.3
q22.2
q22.32
q24.1
q25.1
q25.3
q26.1
q26.2
q26.3
1,000 bp
000 bp
65,628,100 bp
65,628,200 bp
65,628,300 bp
65,628,400 bp
65,628,500 bp
65,628,600 bp
65,628,700 bp
65,628,800 bp
65,628,900 bp
65,629
[0 - 10.00]
Mapped reads for LIM2405
Sequence
RefSeq: IGDCC3
Intron 4
Exon 4
Intron 3
4 tracks loaded
chr15:65,628,498
151M of 259M

(*Continued*)

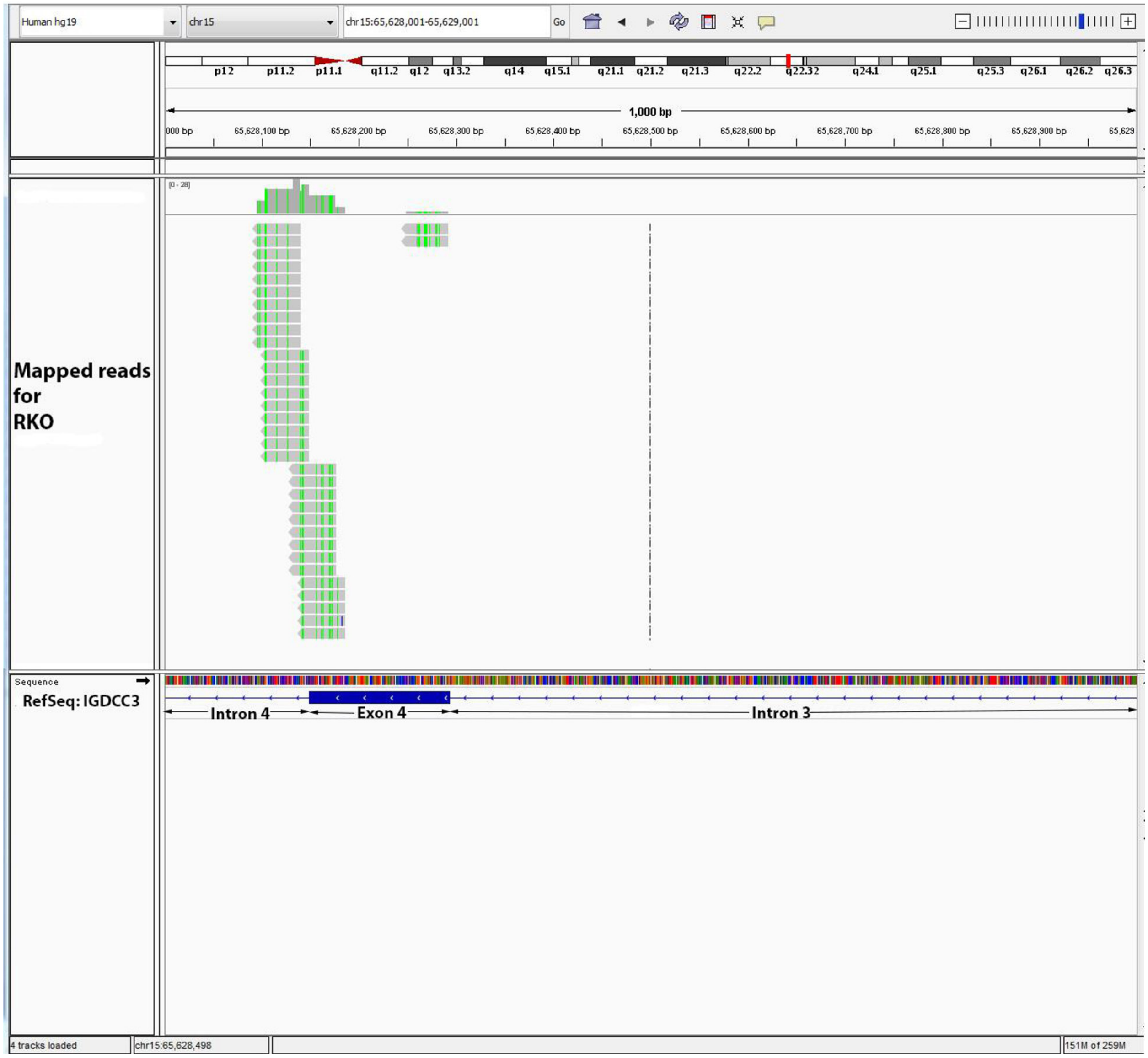

Supplementary Figure S1: Mapped reads within the DMR, DC15A (chr15: 65628001-65629000), across 11 cell lines. DC15A overlaps intron 3, exon 4 and intron 4 of the IGDCC3 gene. Green striped lines within the mapped reads represent methylated cytosines on the reverse strand while red striped lines represent methylated cytosines on the forward strand.

Supplementary File 1: FASTA_enhancers-DMRs

See Supplementary File 1

Supplementary File 2: MethylKit DMR results

See Supplementary File 2

Supplementary Table S1: Beta-values for methylation for each enhancer within each cell type is given above. These 24 enhancers have already been identified by methylKit to contain a region (1000bp) that is differentially methylated between the CIMP and non-CIMP groups. Thus, the average methylation beta-value for each enhancer in the CIMP and non-CIMP groups was calculated and a T-test was also performed to identify the best candidates for further investigation. The p-values of the T-tests are given in the last column.

See Supplementary File 3

Supplementary Table S2: Calculated correlation coefficients for enhancer 1702 against DC1A and DC1B and for enhancer 1944 against DC15A using 11 CRC cell lines

See Supplementary File 4

Supplementary Table S3: Calculated correlation coefficients for enhancer 1702 against DC1A and DC1B and for enhancer 1944 against DC15A using 24 CRCs of the GEO GSE39068 dataset

See Supplementary File 5
