# Supplementary material for: Distinct epigenetic signatures elucidate enhancer-gene relationships that delineate CIMP and non-CIMP colorectal cancers
Source: Oncotarget. 2016 Mar 30;7(19):28027–39. doi: 10.18632/oncotarget.8473 (PMC5053707; doi:10.18632/oncotarget.8473)
Supplement: Supplementary file 2 [file oncotarget-07-28027-s002.pdf]

>Enhancer 1702
tcCaTTTCTTCCGAAGCCACAGTCaaacactcctgcttttccgcctggaaacaaactaat
tggaaataataattagtaatgatttccccctaaaactttttactcgggcttccgcactc
tggaaatctcccgggagagaacctctaaacggcgaggtttagagaacctctaaacggcga
gaggggcacggcaatgtccaaacgagaagaaagagctgattttgtaaagcgtccttccta
aacatccagcgcccgcccggggagacccgataggacgggcggcggcgaaccgggtcctgg
ctggagaaaaggctgggagcgcacagcggagagacccgctcctgtgtttttgttctgttt
tctttttaaactaatggaaggcctcaacctgtcactgagggatcatttggcttctgaggg
taggtagcctagggtgtcacagagaagtcattagaaatatgtgcgacttttttttttttaa
attactcttcctgtgatcgtttgagtccctggcgcgtgaggaaggggagaggaggcaggg
agggggtgggtgggctggcagcctgcgatccgggaggagctcccttgggcagcgagacgg
cgccgggtccctgaattagacagaggcgaagagagcggctccgtgatcaagtgcgcgccg
gcagccacgcaggcgggagagcgcgcaaacccggggatttgcagtggcactctcctgtag
cgaatgcaagtaaaacaggcggctgaggacgcgcggcggattagaacaatatttgcccaa
catgacgcagaatactgaggagagccgagtgccggtcgctaaagaggctcttgaatataa
agttgggcgctcgagagctcttgagcgctaatggcatatcccgccacggagaccgagtga
ctccgactttgcgacgcacgatttctgattaaatccgagagtggcttcagacaccgactt
ttacaaagagaggtgggggaccgggagagcgagggagggggcggtaggaacggcccagc
agcggagcctcttctcgcctcctccctttcaattacagtcagctccctccactcccgcgg
ctgtgaaatcgttctaatcttccagatctccgcagccgctgagtccccaaattgggggga
tcgaggccggattagtgcgctgggtgtccccccaatcttctgtgaggaaattgtgtagac
tttgcattaaggcaaccaggcgttctcccctccccagcacacaccagtgatgcctgtctt
cccggttcccaaagtcacccccacccgggggggactgtgtcccatctgcctaccagcctcg
cagagcctgcaggcacagttcatggatttgtgaggccaagtcgactatttaatttaggag
ctttggggcagtagtgtcctaaaattgtcccaaaacgaaattaggacttcgagggtggtt
agaagcaaggagagtcacccaagggacaaccaggtgcccttggaggcagcagccaacagc
atatgtcacccgtccctcggctgtgcctcagctgttccctgggacagttccacggctcta
gccagagctgtaaatgtagtaatcagccaggcctTTCTAGCCCCCAACAAACTG

>Enhancer 1944
AGACGCAGGAGTGGTGAAGccccaagctcactagcctggtgctatcccttaggggccctc
caccctttgtgaacagcctctttcctcaaccctcctctagtgacctgatttgagtgtgcc
atctctttcctgtcaagaccctgctggacatgtgccctgtcccctctgaccctcagctct
ctgggctgtgttaccaagtggcttggctggggcacagtgtactggaaaatgcccatctca
gtgcctctgcctgatcacacagttatgggaaccagaggaatgtcttacaggcagaggtgg
cgcctccgtgaccggaagccagggccttgcctcccaaggcacttgaatttgagggggaaa
agattgacctttagaagctctccgaggagtggctgggaatggaaacagaaccagatggca
tgagctccagagtgcacagcttctgtcacccccctcccaagtgaggggccaggcaggcctg
cagccccggcctccaccaccctggctggaccggaccattacaagatttatccaaggggtc
ctgcaaggtacatagagggagccctggggatccttgcttcaggcccagccctgccacctg
tttgctgggtgacatggggctggttgcatcacctttctgagccccagtttgtatctgtgg
ttgagatgtgtctggtgcatacatttgctgtggggtcaggtgggggaacgtgtgtgccgg
ctcactgtgaagggagccagtgtagtggcagggaggctccagctgcggctcagacttcct
ggattctaatccagttctaatactttctgactgtgtgatcttgagcagttgcttaacctc
tctgagcctcaggtgggcatctgtcaggacagcagaacctaaactgaaagggctgttggg
agagtaaatgagagagagcgcagacagggcgtagagcagagcctggctgtcagtgtcagt
gaaccagaaacctccctgccctcaggcatgggcagtggagtatcttggcgatagcagtca
gtctgacttcagatggagaaggcaattaatgactgcctggtgtagttgggagccccaaag
agttttggccttgaaaggatctctcatcattcctcctgattagggcattgtctgcagaat
atgactctagggacaaaggctggtgccccctctggtggagggaggacagtgctttgctgg
acaggcaagtgtgtgagtcctctcacctgtgcaggcagcctccccagccctgggtctgac
tgaagatccagatgctaagaacttggcatccctgtgacccatgggccccagctctgtaac
tgaggtccctgcactcctggggagctaaagacgcttctcttaggttcagtctcctacctt
gaacccccaaaacagctctggactgcctctaggcggtctcatgggccacctgcccccacc
aggcctggggttgatatgacaaatcaaagctcctcatcacaggcacggggtggtggcttc
cttgcccagcagcacccactgctctcctcagagttcaatctgctccactgccgagtgtgg
ccactgctgctggcctgctcaaggacagcaacgcacacttgtctctgcttgccttgctgc
catacaaaaggtgccatgtggtttccaaaggagcacagcacagggggtgtgaaccagctg

gaacctcactcccttccatgggcaccagggggcctgtgcagatcaacaccaggaccctcc
cagcaacgacagggatcccttcttcagccccagccctgcagtccttccaggctctgaagt
ctgggtgctgaggaacgggggtggctgccttgtttctcggaaatcacctcactagtagtc
atcccaagtactagaccttccttgaatcccttctggcgggtctttcagctggagttcagt
gacaaccctttggaaggcagctcgctttcagcatgggccactcctcaggtgactctcctg
ggccccctctccccccactctttttcttattcttctttcagcaaacactctgtgcacat
cctctaaccagaagctgtgccaggcgcaggggcagagagacaagccaggttcagcccctg
cccccagggcctcacagtagagcgaaggaggcagccagctcctgcagcagttaggaccac
aaggtgagctccctgatggagatagcgtccctcctggtggcagcctcagcccagggttcc
ctcatccagcttaacataacttcctgtaagttcacagggagacagcacacactctctgcc
ccacttgactgccactgcctttacttatgttctcttgcactcagcagggtgctttggttg
tcagccagggaaacctttggctaagttcaggagaacaggatcttactggaaggactttgg
gtagctcagtgctccctgggaaggccagcacacctggctggaaatggacagtccacagca
tcgggagctgcacacagtcccactgaggctgccccagaacgtgtatactcaagattccag
cgctccagcccggtgttggactggccttgtgtgggcaccatgcgctcacccccctagacag
aggaccacagggcactctaacaacacaagaagatggcccacagctgggggagggtcacct
cccccaacttcagtgggtgtcacttaaggggaaatggcccgcacagggcctgctggagtt
cttagaatcgttcagctgctggagtatttgggatatgtggctaagtcacaccctggggct
gtcagtcctgggtccctggctcccaagcaccctggcattcatccttctgccctcatctgg
cacccagttaatgtctctgctcaacctggaaacattttcttaacgacaaaaaaataaaat
cataagttaaaaatctcgagcagtaaaacagcagatgacgttgtgtgggcagctgcccag
aggacacagaacgcacgtctcctgagcgggtggccttataaggacaggttcaggaggaga
atgggcaggagggagggccacccaccaggagagtaggaggctggggtagtggtgggtgcc
ccaccactgggctgagtgtggagccagcagggtgcctggccaggacctgactccacagag
ggacttggctgcgtgcaagagccagaggcccttgtgggccaaagcctgcccgtccctccg
ccttcactgtgtcactgccagcacaggcactgacctgctctcagccagctgccacgcaaa
cccacctggcagcctcaccctgggaacccccctccctccagggcaggggccatttcctatc
cttcatcccaccttcccccactcctgggcccagcaggcctagcaccagctggcatgacgt
gcagtaaaggtttgtggggtgacagaattaggaaatgaagggttctctggtagaagtttt
ggctcatcacctgggctatagcacctggaactcagggcagcgtcttctctcctcagacgg
cccaacgtggggctgaggaggtgggtagggctgggcagtcagtttttgccctgcccagga
tcacctttgtccccattgtgaagacacacataccatcaacgtcctcctgatagggtggaa
gagtttcactcccccgaggtcaaagtgtgtgtgtgtgacagaatatgtgtgtttctggga
gagtcctgattgtctctgcacttttctgaccccagcatcactaggattaaagacctggcc
cttgggATCTGAGGGTGAGCAGAGGAGcgc

>gi|224589800:228194001-228195000 DC1A
GAGAGTCCCACTCGTCCCCAGCTGTGACCCACAGACGCGTGGCTGCACCTTGGAGGTCCACCTGGCCGCT
GTCCCCATTGCAGGCTGCACCCATCCCGGACCCTTTCTATCCGCCGAAGGCGCAGGCAGCTCTGGTCTCA
GCGGAAGGAGTCCGGCCGCAGGCAGACGGGGCCAAAGGACGTGCACCCGACAACCGTCCGCTCCCTCCCC
AAACCCCCGCCCCGAGAAGGTTGATGGATGAAGGGGAGCCCTAGCCCAGCCACCTCGGGGGCAAGAGACG
GAGCTCGCCACAGACCAGGAGCGAGAGGGGGACTGCGGGTCCCACCGTGAGCGCAGGGCGCGCGGGGCTG
GAAACCCAGGACGCGGCCCGGGAGCTCCAGGAGGGCGCCTGCGGATCGCGCGGCCCCGGGCCGGCCGCCT
GCCCATCTGGCGCACCCCAGCGCGCCGCGCACACCTGGGGGCCCGCACACCAGCACGTCCTCAGACACAC
CGACACCCACGGCAGCAGGCGGGGGCCAACGCCGCCGCCGCGGTTCGGGCTCCCGTGGCCCCGCCTGCCC
CTGCCTGCCCCGCCCGGGCCCCGGCCCCGGCGGGCGCCCCCCGCGGCGCTGCCCCGCCCCCGCCGCCCCG
CGCCGCCCCGGGCCTCGGCCCGCAGCCCGGCCGCCGGCCCACCTGGCGCAGCGCCGCCCTCGGAGCCCGC
GCACACCCGCGCACCCGCGGCCGCAGGAGGGCCCAGCGACGCCGCCGCGCCAGCTCCCAGGGCCCGGCCC
CCCCCGGCGCTCACGCTCTCGGGGCGGACTCCCGGCCCTCCGCGCCCTCTCGCGCGGCGATGGCCCCACT
CGGATACTTCTTACTCCTCTGCAGCCTGAAGCAGGCTCTGGGCAGCTACCCGATCTGGTGGTGAGTGAGC
CTCCTCGCGTTCGCCCCTGCCCCTGTGCGCCGCGCCCGCAGCAGACGGTCCCCTCGGGCAGGGACCCCGC
GGTGGCCCGAGCCCGCGCCC

>gi|224589806:65628001-65629000 DC15A
CAGTCTAAACTGTGGTTGCAGAAACACCACTATCTCAGGCACCTGTGGGCACCAACTGCAGCTCCCAGAG
CCGCGGGCCATCCTTCTCTACGGCCAGAGTCACTGGCCACCTCTTTGTCCTCCCTCTGTCTTTCCCACAC
GTGGCTCACCTCTCATTGTCCGTGTCAATTGGGACTCTGTTCTTCTCCCAAGTGATCAGGGGTTTGGGAA
GCCCATGGATTTGGCACTGGAAGCGGGCCACACCACCCTCCTCACCCACGGTGGCCTGGGGATGCACGTG

GAAGTCCGACATGGCTGGGGGAAGAGAAGTGTATGAGTGCAGTGCGCTGCTGAGGGCAGAAGGTAGGAGA
GAATGGGCTGCAGTGGCGTCACAGGCACGGAGACCTGGAGCCCACTCCATCCAGGGGCAGGAGTTTTCCT
GCAAGGGCAATAGCTTGGGGGACCCATCAGCACTTACCCCAGCCTCCAATAGCTAGGAAACATCTCCCCT
GAGAGGTGGTATAGGCTGGGCTGGGATACTTTGTAAAAAGTCTGGACAAACTTCTGGATAACAAACCTCT
AATAAGGAAATCCTGCACTGTTGGGACTTCTCTTGGAGGCCTTGGTCCCCAAAACTGACCCATGAAGGCC
AATCAGACAAAGCCCTGCTCCCGAGCCCCACACCCCACTTTTTCAGCAGCTTTCAGATTCGTTTTTGTAA
ATTCTCCCTAAGGCAGGCAAGGGCTCAGATCCTCAGTGCTTCACATTTTATAGAATTGGAAGCTGAGGCT
CAGAGAAGGGAAGCAATTAGTCCTTTGTTGCCCAGCAGGGACACTGGTCATCAGGCCCTGGTTACCAGGG
CCCGGGAGCTTTTTTTTGGTCCCTCCTTCTCCTACTCCTCCCACCCCCAGTTCATCTGCAGTCTCTCGAT
GCACCCCAAGAGGGTGTTGCATCCATCCAAGGTGACAAGCCTCTCAGAAGACTGTCAGACCTACCAATCT
ACATTTCAATGTGTCATAAA
