# Supplementary material for: Distinct epigenetic signatures elucidate enhancer-gene relationships that delineate CIMP and non-CIMP colorectal cancers
Source: Oncotarget. 2016 Mar 30;7(19):28027–39. doi: 10.18632/oncotarget.8473 (PMC5053707; doi:10.18632/oncotarget.8473)
Supplement: Supplementary file 3 [file oncotarget-07-28027-s003.pdf]

Results presented by methylKit below can be interpreted as: ("chromosome") (start coordinate of DMR) (end coordinate of DMR) ("strand" - "*" means information not given) (p-value) (q-value) (% methylation difference)

"chr1" 567001 568000 "*" 0 0 -99.2277992277992
"chr1" 808001 809000 "*" 8.7349629751543e-09 1.47505871898511e-08 100
"chr1" 878001 879000 "*" 2.9801938694618e-11 7.13712120080206e-11
53.4942182001006
"chr1" 925001 926000 "*" 3.56775498033812e-10 7.35898629809829e-10 100
"chr1" 937001 938000 "*" 0 0 99.0909090909091
"chr1" 946001 947000 "*" 7.00084434868131e-12 1.82455328913592e-11 80
"chr1" 948001 949000 "*" 7.67056418382595e-11 1.7359448580318e-10 -100
"chr1" 949001 950000 "*" 0 0 -68.3923882989847
"chr1" 950001 951000 "*" 0 0 -56.3505747126437
"chr1" 993001 994000 "*" 0 0 91.4529914529915
"chr1" 996001 997000 "*" 0 0 -53.2166214352449
"chr1" 1006001 1007000 "*" 2.71009259478205e-08 4.26943022775686e-08
74.6987951807229
"chr1" 1143001 1144000 "*" 0 0 -67.3151098440382
"chr1" 1150001 1151000 "*" 0 0 -81.1764705882353
"chr1" 1208001 1209000 "*" 0 0 -59.2964824120603
"chr1" 1242001 1243000 "*" 0 0 57.6628352490421
"chr1" 1245001 1246000 "*" 0 0 -69.9828473413379
"chr1" 1253001 1254000 "*" 4.08209022140227e-11 9.57770690255255e-11 -100
"chr1" 1260001 1261000 "*" 0 0 -100
"chr1" 1261001 1262000 "*" 1.49757983791687e-12 4.26626596017328e-12
55.1136363636364
"chr1" 1280001 1281000 "*" 2.36222152949495e-12 6.56090721848613e-12
-82.6923076923077
"chr1" 1299001 1300000 "*" 2.73625566649116e-12 7.52483363579137e-12 -100
"chr1" 1350001 1351000 "*" 6.6123223829706e-07 8.60613142102833e-07 75
"chr1" 1378001 1379000 "*" 0 0 59.6153846153846
"chr1" 1380001 1381000 "*" 3.530509218308e-14 1.22891039008692e-13 100
"chr1" 1384001 1385000 "*" 8.69011083648452e-07 1.11229659818976e-06
66.6666666666667
"chr1" 1392001 1393000 "*" 0 0 94.300518134715
"chr1" 1446001 1447000 "*" 0 0 97.6190476190476
"chr1" 1514001 1515000 "*" 1.0430847297016e-09 2.01267828368141e-09
-72.2222222222222
"chr1" 1546001 1547000 "*" 1.17905685215192e-13 3.83653245040640e-13 100
"chr1" 1619001 1620000 "*" 0 0 -74.3243243243243
"chr1" 1628001 1629000 "*" 3.66373598126302e-15 1.43441054525534e-14
-65.9090909090909
"chr1" 1694001 1695000 "*" 3.62609997583974e-09 6.49860893215650e-09 56.25
"chr1" 1711001 1712000 "*" 5.94625383376624e-08 8.93214664892267e-08
63.4146341463415
"chr1" 1719001 1720000 "*" 1.99840144432528e-12 5.59476580335607e-12 100
"chr1" 1834001 1835000 "*" 5.220638035075e-05 5.11909765154034e-05 53.125
"chr1" 1837001 1838000 "*" 0 0 100
"chr1" 1915001 1916000 "*" 6.12778716657658e-11 1.40703078182493e-10
66.6666666666667
"chr1" 1946001 1947000 "*" 2.22044604925031e-16 9.81641919380259e-16

-51.8518518518519
"chr1" 1973001 1974000 "*" 1.16836607055859e-09 2.23702437154968e-09
62.8571428571429
"chr1" 2037001 2038000 "*" 1.32174271527674e-11 3.32130954108767e-11
61.2903225806452
"chr1" 2064001 2065000 "*" 0 0 -63.0392826987254
"chr1" 2071001 2072000 "*" 4.71134242729931e-12 1.257615036806e-11 -100
"chr1" 2120001 2121000 "*" 4.30801838646744e-10 8.80013069436791e-10
-67.6470588235294
"chr1" 2136001 2137000 "*" 0 0 60.3849636876964
"chr1" 2161001 2162000 "*" 0 0 -61.4772530704031
"chr1" 2221001 2222000 "*" 0 0 56.6666666666667
"chr1" 2256001 2257000 "*" 3.33066907387547e-16 1.4495649018245e-15
-57.037037037037
"chr1" 2314001 2315000 "*" 7.23310300543289e-13 2.13818392906359e-12
-82.7067669172932
"chr1" 2361001 2362000 "*" 0 0 68.75
"chr1" 2376001 2377000 "*" 2.04554759530851e-06 2.48561368301037e-06
64.8648648648649
"chr1" 2378001 2379000 "*" 6.55031584528842e-15 2.48816226484197e-14
-82.5396825396825
"chr1" 2380001 2381000 "*" 5.06533703870105e-11 1.17449092111009e-10
-50.5733397037745
"chr1" 2395001 2396000 "*" 0 0 54.2372881355932
"chr1" 2398001 2399000 "*" 7.7715611723761e-16 3.26213507634405e-15
61.5384615384615
"chr1" 2403001 2404000 "*" 0 0 54.2027481571602
"chr1" 2413001 2414000 "*" 8.4567405324032e-06 9.36439388842242e-06
55.5555555555556
"chr1" 2461001 2462000 "*" 0 0 58.38766674974
"chr1" 2477001 2478000 "*" 4.01313771103418e-09 7.11882087237587e-09 100
"chr1" 2487001 2488000 "*" 3.86045582855266e-05 3.86136777829505e-05
-52.4691358024691
"chr1" 2507001 2508000 "*" 1.21569421196455e-13 3.95173810577501e-13
64.5424836601307
"chr1" 2512001 2513000 "*" 0.000201461795166558 0.000179722257706492 56
"chr1" 2543001 2544000 "*" 0 0 100
"chr1" 2569001 2570000 "*" 0 0 -100
"chr1" 2583001 2584000 "*" 0 0 97.1428571428571
"chr1" 2624001 2625000 "*" 4.01313771103418e-09 7.11882087237587e-09 100
"chr1" 2704001 2705000 "*" 1.31830011351752e-08 2.1720958608122e-08
56.6666666666667
"chr1" 2732001 2733000 "*" 1.20495169397827e-10 2.66075324129686e-10 60
"chr1" 2737001 2738000 "*" 4.83434240527991e-06 5.5546183284725e-06
-51.5151515151515
"chr1" 2747001 2748000 "*" 7.12547354453363e-10 1.40825483981431e-09 -74
"chr1" 2756001 2757000 "*" 3.34128893442198e-08 5.19772864618523e-08 -100
"chr1" 2763001 2764000 "*" 0 0 100
"chr1" 2773001 2774000 "*" 0 0 -86.4406779661017
"chr1" 2786001 2787000 "*" 3.19230863965458e-10 6.63826178695931e-10
59.4594594594595
"chr1" 2791001 2792000 "*" 6.40709196808587e-09 1.10717513558483e-08

51.6161616161616
"chr1" 2814001 2815000 "*" 1.26554322577022e-12 3.63817817636463e-12
61.6161616161616
"chr1" 2817001 2818000 "*" 1.85573345579115e-09 3.46426411502745e-09
73.3333333333333
"chr1" 2825001 2826000 "*" 2.71381805916349e-11 6.55148978444393e-11
71.0691823899371
"chr1" 2867001 2868000 "*" 6.90747459231034e-12 1.80140148623876e-11 100
"chr1" 2878001 2879000 "*" 0 0 55.5555555555556
"chr1" 2879001 2880000 "*" 1.41959980659578e-07 2.014179973408e-07
76.7123287671233
"chr1" 2919001 2920000 "*" 0 0 56.5514373960561
"chr1" 2921001 2922000 "*" 8.7349629751543e-09 1.47505871898511e-08 -100
"chr1" 2927001 2928000 "*" 2.33028818463765e-10 4.93719283454501e-10 100
"chr1" 2944001 2945000 "*" 7.03213043351525e-10 1.39092749753821e-09
-56.6037735849057
"chr1" 2986001 2987000 "*" 0 0 56.3792390405294
"chr1" 2993001 2994000 "*" 6.52811138479592e-14 2.19810666311838e-13
83.3333333333333
"chr1" 2998001 2999000 "*" 0 0 75.2475247524752
"chr1" 3005001 3006000 "*" 6.10622663543836e-15 2.32593540648772e-14 -75
"chr1" 3014001 3015000 "*" 4.44633077101297e-06 5.13255481329123e-06
54.7619047619048
"chr1" 3023001 3024000 "*" 4.44089209850063e-16 1.91071758245033e-15
82.5396825396825
"chr1" 3026001 3027000 "*" 1.67249547544657e-11 4.14967835815161e-11
-64.5161290322581
"chr1" 3053001 3054000 "*" 2.1094237467878e-15 8.47434540879246e-15 100
"chr1" 3069001 3070000 "*" 0 0 100
"chr1" 3085001 3086000 "*" 1.56539226026098e-11 3.89646050070837e-11
67.0886075949367
"chr1" 3126001 3127000 "*" 0 0 100
"chr1" 3139001 3140000 "*" 0 0 -98.9130434782609
"chr1" 3145001 3146000 "*" 4.10782519111308e-15 1.59859775077462e-14
56.8655941878568
"chr1" 3150001 3151000 "*" 4.49772391331038e-07 5.9806653138081e-07
57.1428571428571
"chr1" 3215001 3216000 "*" 6.60582699651968e-14 2.22176301799e-13 100
"chr1" 3222001 3223000 "*" 2.22044604925031e-16 9.81641919380259e-16
60.944976076555
"chr1" 3250001 3251000 "*" 0 0 100
"chr1" 3297001 3298000 "*" 0 0 -59.6491228070175
"chr1" 3309001 3310000 "*" 0 0 100
"chr1" 3333001 3334000 "*" 2.4535928844216e-14 8.69144396197119e-14 100
"chr1" 3369001 3370000 "*" 5.1223403296774e-10 1.03532645132705e-09
73.9130434782609
"chr1" 3400001 3401000 "*" 0 0 -55.6753355704698
"chr1" 3521001 3522000 "*" 5.45864948797714e-08 8.23916743843603e-08
-61.5384615384615
"chr1" 3528001 3529000 "*" 0 0 51.7902813299233
"chr1" 3631001 3632000 "*" 0 0 54.7079964061096
"chr1" 3637001 3638000 "*" 0 0 53.1878031878032

"chr1" 3657001 3658000 "*" 0 0 -93.9024390243902
"chr1" 3672001 3673000 "*" 0 0 100
"chr1" 3747001 3748000 "*" 0 0 -100
"chr1" 3771001 3772000 "*" 2.40429218167915e-06 2.89170707455451e-06 -76
"chr1" 3795001 3796000 "*" 3.6700841921089e-08 5.65530226072258e-08 100
"chr1" 3818001 3819000 "*" 0 0 -98.5294117647059
"chr1" 3998001 3999000 "*" 2.08814465718632e-09 3.86232567960463e-09 100
"chr1" 4005001 4006000 "*" 1.5512089182157e-05 1.6512049128883e-05
-59.8290598290598
"chr1" 4006001 4007000 "*" 3.49720252756924e-14 1.21818121473274e-13
97.6744186046512
"chr1" 4018001 4019000 "*" 0 0 84.6153846153846
"chr1" 4038001 4039000 "*" 0 0 100
"chr1" 4046001 4047000 "*" 1.29037891483108e-11 3.24582280286866e-11 -100
"chr1" 4052001 4053000 "*" 1.85684474496295e-06 2.26980734744938e-06
53.3333333333333
"chr1" 4057001 4058000 "*" 8.80406858527749e-14 2.91456141063452e-13
63.585768236931
"chr1" 4058001 4059000 "*" 0.000137599100368035 0.000126143016114881
-57.6923076923077
"chr1" 4061001 4062000 "*" 8.32667268468867e-15 3.12210720380152e-14
52.3809523809524
"chr1" 4082001 4083000 "*" 2.62900812231237e-13 8.21294938531841e-13 100
"chr1" 4108001 4109000 "*" 0 0 -74.0384615384615
"chr1" 4122001 4123000 "*" 0 0 -100
"chr1" 4136001 4137000 "*" 2.02327044007689e-12 5.65300384792621e-12 -100
"chr1" 4138001 4139000 "*" 0 0 61.8181818181818
"chr1" 4150001 4151000 "*" 2.88710388929303e-10 6.03399264731139e-10 -100
"chr1" 4168001 4169000 "*" 3.34128893442198e-08 5.19772864618523e-08 100
"chr1" 4233001 4234000 "*" 2.50910403565285e-14 8.87903196465237e-14 100
"chr1" 4288001 4289000 "*" 0 0 -58.4041184041184
"chr1" 4335001 4336000 "*" 1.93720595120794e-11 4.76309924836636e-11 100
"chr1" 4336001 4337000 "*" 1.52466927971773e-12 4.33322475981408e-12 -100
"chr1" 4360001 4361000 "*" 0 0 -100
"chr1" 4361001 4362000 "*" 7.78266340262235e-14 2.59954163987046e-13
79.6296296296296
"chr1" 4384001 4385000 "*" 0 0 88.4210526315789
"chr1" 4385001 4386000 "*" 0 0 100
"chr1" 4388001 4389000 "*" 1.48087875295744e-10 3.22075573380618e-10 100
"chr1" 4391001 4392000 "*" 1.36446409726432e-13 4.41262773670837e-13
-50.9803921568627
"chr1" 4403001 4404000 "*" 2.88779000712225e-11 6.92935809466068e-11 -100
"chr1" 4468001 4469000 "*" 0 0 70.8602923556195
"chr1" 4520001 4521000 "*" 0 0 -53.8700430004778
"chr1" 4567001 4568000 "*" 1.98124849859482e-10 4.25157611354621e-10 100
"chr1" 4580001 4581000 "*" 6.97980105945906e-06 7.83219785087877e-06
57.7777777777778
"chr1" 4621001 4622000 "*" 2.79397076918819e-07 3.82186500677801e-07
75.1295336787565
"chr1" 4645001 4646000 "*" 0 0 -72.6190476190476
"chr1" 4652001 4653000 "*" 1.11022302462516e-16 5.03662826618488e-16
-64.7668393782383

"chr1" 4676001 4677000 "*" 2.16382467499443e-13 6.82775854166559e-13 -100
"chr1" 4687001 4688000 "*" 0 0 -100
"chr1" 4692001 4693000 "*" 1.67299207820548e-08 2.71377429441055e-08 -100
"chr1" 4696001 4697000 "*" 0 0 50.9557401297076
"chr1" 4707001 4708000 "*" 6.02862822451922e-06 6.82630909736564e-06
61.1111111111111
"chr1" 4717001 4718000 "*" 3.10862446895044e-15 1.22466437093774e-14 100
"chr1" 4730001 4731000 "*" 1.14124265593318e-11 2.89138062669327e-11 -100
"chr1" 4747001 4748000 "*" 2.02060590481778e-13 6.40127405465654e-13
-66.6666666666667
"chr1" 4754001 4755000 "*" 1.15666503819334e-07 1.66396436930012e-07
79.5454545454545
"chr1" 4757001 4758000 "*" 2.05269135022945e-12 5.73290098696686e-12
72.2222222222222
"chr1" 4762001 4763000 "*" 1.52794177310511e-09 2.87642564232045e-09 100
"chr1" 4763001 4764000 "*" 0 0 69.2307692307692
"chr1" 4784001 4785000 "*" 1.89700077657395e-06 2.31634779242663e-06
57.8313253012048
"chr1" 4820001 4821000 "*" 0 0 100
"chr1" 4831001 4832000 "*" 8.06688049692639e-13 2.37556391305009e-12 100
"chr1" 4845001 4846000 "*" 0 0 100
"chr1" 4859001 4860000 "*" 1.99840144432528e-12 5.59476580335607e-12 -100
"chr1" 4957001 4958000 "*" 0 0 -100
"chr1" 5037001 5038000 "*" 0 0 -100
"chr1" 5045001 5046000 "*" 0 0 61.3333333333333
"chr1" 5060001 5061000 "*" 2.15125472990962e-10 4.58126659943874e-10 -100
"chr1" 5191001 5192000 "*" 0 0 85.0299401197605
"chr1" 5343001 5344000 "*" 4.44089209850063e-16 1.91071758245033e-15 95
"chr1" 5353001 5354000 "*" 2.00227675550835e-08 3.20459176617961e-08 -100
"chr1" 5391001 5392000 "*" 2.88779000712225e-11 6.92935809466068e-11 -100
"chr1" 5514001 5515000 "*" 4.26951363152739e-10 8.72510873516957e-10 -100
"chr1" 5537001 5538000 "*" 9.34051801460001e-08 1.36032848893872e-07 -80
"chr1" 5539001 5540000 "*" 0 0 68.4210526315789
"chr1" 5547001 5548000 "*" 7.0006535457523e-08 1.03695832512054e-07 100
"chr1" 5705001 5706000 "*" 4.91828799908944e-14 1.68192819018269e-13 -100
"chr1" 5707001 5708000 "*" 5.794809077031e-12 1.52911192757929e-11 100
"chr1" 5720001 5721000 "*" 0 0 -100
"chr1" 5725001 5726000 "*" 4.86753970463383e-11 1.13145908161915e-10 100
"chr1" 5733001 5734000 "*" 1.52794177310511e-09 2.87642564232045e-09 100
"chr1" 5749001 5750000 "*" 0.000379873397011621 0.000323695610297176
52.6315789473684
"chr1" 5766001 5767000 "*" 8.7349629751543e-09 1.47505871898511e-08 100
"chr1" 5790001 5791000 "*" 5.55111512312578e-16 2.36485094870365e-15 100
"chr1" 5794001 5795000 "*" 4.99020824662466e-11 1.15790792586157e-10 100
"chr1" 5802001 5803000 "*" 1.67925686977988e-05 1.77860853586133e-05
57.1428571428571
"chr1" 5806001 5807000 "*" 6.76056877146891e-10 1.33943733804983e-09
-65.9090909090909
"chr1" 5816001 5817000 "*" 5.65614980840845e-06 6.43415947659831e-06
-76.9230769230769
"chr1" 5818001 5819000 "*" 0 0 78.5714285714286
"chr1" 5833001 5834000 "*" 9.80763359414993e-10 1.8980032424715e-09 100

"chr1" 5839001 5840000 "*" 1.0636353062754e-06 1.34530286878699e-06
-52.1739130434783
"chr1" 5844001 5845000 "*" 6.66133814775094e-16 2.81595744474255e-15 100
"chr1" 5846001 5847000 "*" 1.92946192356658e-09 3.59193011611483e-09 -100
"chr1" 5858001 5859000 "*" 4.65375923230926e-06 5.35768484882947e-06
-58.9285714285714
"chr1" 5887001 5888000 "*" 2.00227675550835e-08 3.20459176617961e-08 100
"chr1" 5906001 5907000 "*" 0 0 100
"chr1" 5910001 5911000 "*" 4.20774526332934e-13 1.27917506609538e-12
64.3587069864442
"chr1" 5975001 5976000 "*" 3.03090885722668e-14 1.06210046699299e-13 -100
"chr1" 6080001 6081000 "*" 2.75837855657768e-05 2.82530803747995e-05
66.6666666666667
"chr1" 6114001 6115000 "*" 0 0 100
"chr1" 6119001 6120000 "*" 2.55442333951805e-11 6.18800647355251e-11
-65.5172413793103
"chr1" 6160001 6161000 "*" 6.39714003991543e-09 1.10559284236836e-08
-54.9290780141844
"chr1" 6164001 6165000 "*" 0 0 100
"chr1" 6165001 6166000 "*" 3.88578058618805e-15 1.5152981210988e-14 100
"chr1" 6183001 6184000 "*" 2.19383400335005e-11 5.34788778871231e-11
97.2972972972973
"chr1" 6208001 6209000 "*" 0 0 100
"chr1" 6211001 6212000 "*" 0 0 58.8235294117647
"chr1" 6229001 6230000 "*" 2.40567331610819e-08 3.81152436871569e-08
-65.359477124183
"chr1" 6239001 6240000 "*" 0 0 63.3141591339455
"chr1" 6296001 6297000 "*" 0 0 56.4792317482356
"chr1" 6305001 6306000 "*" 0 0 57.7808978651402
"chr1" 6362001 6363000 "*" 0 0 -100
"chr1" 6376001 6377000 "*" 1.14127990502588e-07 1.64327710092136e-07 54
"chr1" 6438001 6439000 "*" 4.72908490145585e-10 9.6077800007378e-10 -55
"chr1" 6450001 6451000 "*" 7.34939281543667e-07 9.50242626154403e-07
71.7391304347826
"chr1" 6474001 6475000 "*" 1.38486533352022e-09 2.63065104258646e-09
-63.8888888888889
"chr1" 6490001 6491000 "*" 0 0 -100
"chr1" 6568001 6569000 "*" 0 0 -69.811320754717
"chr1" 6579001 6580000 "*" 5.21804821573824e-15 2.00446252136729e-14
67.4418604651163
"chr1" 6580001 6581000 "*" 2.22044604925031e-16 9.81641919380259e-16 -100
"chr1" 6615001 6616000 "*" 6.66133814775094e-16 2.81595744474255e-15
-50.3242835595777
"chr1" 6647001 6648000 "*" 2.82773804372027e-13 8.79129664109438e-13 -100
"chr1" 6672001 6673000 "*" 0 0 70
"chr1" 6698001 6699000 "*" 0 0 -95.2662721893491
"chr1" 6767001 6768000 "*" 4.08209022140227e-11 9.57770690255255e-11 100
"chr1" 6818001 6819000 "*" 0 0 64.4628099173554
"chr1" 6917001 6918000 "*" 5.80137120131941e-06 6.5882135290028e-06
68.4210526315789
"chr1" 6936001 6937000 "*" 4.60677497663431e-05 4.55590100832258e-05
-57.1428571428571

"chr1" 6985001 6986000 "*" 0 0 100
"chr1" 7045001 7046000 "*" 0 0 100
"chr1" 7048001 7049000 "*" 0 0 86.2745098039216
"chr1" 7062001 7063000 "*" 8.3882012447134e-11 1.88922989503423e-10 100
"chr1" 7067001 7068000 "*" 0 0 -71.2309542902967
"chr1" 7078001 7079000 "*" 8.3882012447134e-11 1.88922989503423e-10 100
"chr1" 7108001 7109000 "*" 3.44416961617355e-09 6.18851573747689e-09
85.8333333333333
"chr1" 7117001 7118000 "*" 0 0 -100
"chr1" 7160001 7161000 "*" 1.56863411149288e-12 4.4469621057462e-12 100
"chr1" 7182001 7183000 "*" 3.33066907387547e-16 1.4495649018245e-15
68.2926829268293
"chr1" 7188001 7189000 "*" 5.6621374255883e-15 2.16468580166064e-14 100
"chr1" 7197001 7198000 "*" 5.49904566327086e-12 1.45608864649007e-11 65
"chr1" 7223001 7224000 "*" 1.52701395972343e-09 2.87642564232045e-09 -70
"chr1" 7237001 7238000 "*" 1.97619698383278e-14 7.08978387753544e-14
66.6666666666667
"chr1" 7238001 7239000 "*" 7.68749155333204e-07 9.91437385905438e-07
72.0930232558139
"chr1" 7259001 7260000 "*" 2.17506013200364e-11 5.30460364827628e-11 88.28125
"chr1" 7288001 7289000 "*" 0 0 -100
"chr1" 7339001 7340000 "*" 1.19904086659517e-14 4.40901388691796e-14 -100
"chr1" 7444001 7445000 "*" 0 0 100
"chr1" 7492001 7493000 "*" 4.65637306490407e-10 9.46720524046618e-10 100
"chr1" 7508001 7509000 "*" 0.000792090690089564 0.000639295093468792
52.3809523809524
"chr1" 7509001 7510000 "*" 5.794809077031e-12 1.52911192757929e-11 -100
"chr1" 7535001 7536000 "*" 0 0 50.6607929515419
"chr1" 7539001 7540000 "*" 1.40309586171838e-09 2.66394638337721e-09
-51.685393258427
"chr1" 7541001 7542000 "*" 3.68371999570627e-13 1.13149976091305e-12 -100
"chr1" 7590001 7591000 "*" 0 0 100
"chr1" 7594001 7595000 "*" 2.77146083860202e-11 6.681556500076e-11
-58.8235294117647
"chr1" 7635001 7636000 "*" 0.000220373129685925 0.000195434775679205
-50.9803921568627
"chr1" 7637001 7638000 "*" 2.4535928844216e-14 8.69144396197119e-14 -100
"chr1" 7640001 7641000 "*" 8.57092175010621e-13 2.51149198099779e-12 100
"chr1" 7641001 7642000 "*" 2.71893618730701e-13 8.46692454027087e-13 100
"chr1" 7675001 7676000 "*" 7.67056418382595e-11 1.7359448580318e-10 100
"chr1" 7695001 7696000 "*" 2.66453525910038e-15 1.05861327033776e-14 100
"chr1" 7699001 7700000 "*" 2.07478478841949e-12 5.79266528576294e-12
-66.6666666666667
"chr1" 7714001 7715000 "*" 4.53591360638494e-06 5.23031683672762e-06 -90
"chr1" 7740001 7741000 "*" 0 0 73.6925515055467
"chr1" 7741001 7742000 "*" 1.20470300402076e-12 3.47093205433442e-12
62.5336021505376
"chr1" 7744001 7745000 "*" 1.5588414337131e-06 1.92765052542723e-06
-63.8888888888889
"chr1" 7746001 7747000 "*" 2.22044604925031e-15 8.90449334476539e-15
-62.4848484848485
"chr1" 7758001 7759000 "*" 1.38590361409285e-10 3.02706178892116e-10 -100

"chr1" 7764001 7765000 "*" 0 0 81.3392417528311
"chr1" 7843001 7844000 "*" 0 0 96.5116279069767
"chr1" 7923001 7924000 "*" 3.72545775839672e-06 4.3519872100983e-06
73.6842105263158
"chr1" 7925001 7926000 "*" 2.81117782852114e-06 3.34638163632813e-06
57.7777777777778
"chr1" 7926001 7927000 "*" 1.11022302462516e-16 5.03662826618488e-16
-76.5957446808511
"chr1" 7955001 7956000 "*" 9.98963367582206e-09 1.67533179897223e-08
66.6666666666667
"chr1" 7987001 7988000 "*" 9.65338919911574e-13 2.80678910656113e-12 100
"chr1" 8011001 8012000 "*" 2.00227675550835e-08 3.20459176617961e-08 100
"chr1" 8065001 8066000 "*" 3.37050387599902e-11 8.00975558904068e-11
76.510067114094
"chr1" 8153001 8154000 "*" 0 0 100
"chr1" 8174001 8175000 "*" 0 0 -100
"chr1" 8185001 8186000 "*" 0 0 -100
"chr1" 8193001 8194000 "*" 1.67299207820548e-08 2.71377429441055e-08 -100
"chr1" 8197001 8198000 "*" 1.11022302462516e-16 5.03662826618488e-16 -100
"chr1" 8214001 8215000 "*" 0 0 -62.5
"chr1" 8232001 8233000 "*" 0 0 -100
"chr1" 8258001 8259000 "*" 8.30589819145189e-10 1.62931060806857e-09
-74.5454545454545
"chr1" 8272001 8273000 "*" 0 0 -84.1298287534847
"chr1" 8276001 8277000 "*" 6.30384633382164e-13 1.87665599796749e-12 100
"chr1" 8375001 8376000 "*" 0 0 -71.1111111111111
"chr1" 8408001 8409000 "*" 0 0 -94.8717948717949
"chr1" 8658001 8659000 "*" 3.33066907387547e-16 1.4495649018245e-15 100
"chr1" 8757001 8758000 "*" 0 0 -100
"chr1" 8787001 8788000 "*" 1.52794177310511e-09 2.87642564232045e-09 -100
"chr1" 8893001 8894000 "*" 3.34128893442198e-08 5.19772864618523e-08 -100
"chr1" 8983001 8984000 "*" 1.7962847016495e-06 2.20073010633759e-06 53.125
"chr1" 9031001 9032000 "*" 0 0 93.9759036144578
"chr1" 9032001 9033000 "*" 0 0 100
"chr1" 9033001 9034000 "*" 5.55111512312578e-16 2.36485094870365e-15 -100
"chr1" 9036001 9037000 "*" 0 0 100
"chr1" 9039001 9040000 "*" 9.65729496371637e-10 1.87255912493232e-09 100
"chr1" 9084001 9085000 "*" 9.67004254448511e-14 3.18178990782609e-13 100
"chr1" 9158001 9159000 "*" 6.45894660533664e-07 8.41767429095122e-07
-68.5393258426966
"chr1" 9187001 9188000 "*" 2.44249065417534e-15 9.75180348030994e-15 100
"chr1" 9203001 9204000 "*" 1.55431223447522e-14 5.6388152448458e-14 100
"chr1" 9212001 9213000 "*" 2.21689333557151e-12 6.17227338655116e-12
-58.3333333333333
"chr1" 9214001 9215000 "*" 3.99680288865056e-15 1.5566998858822e-14 100
"chr1" 9294001 9295000 "*" 0 0 -60.2409638554217
"chr1" 9321001 9322000 "*" 9.90563187031057e-12 2.53548354021773e-11
-54.9372146118721
"chr1" 9331001 9332000 "*" 1.37889699658444e-13 4.45602024816198e-13
68.9492325855962
"chr1" 9335001 9336000 "*" 0 0 -69.5121951219512
"chr1" 9340001 9341000 "*" 0 0 -89.8876404494382

"chr1" 9343001 9344000 "*" 3.46090836367274e-06 4.06200733549184e-06
71.4285714285714
"chr1" 9404001 9405000 "*" 0 0 100
"chr1" 9499001 9500000 "*" 8.7349629751543e-09 1.47505871898511e-08 -100
"chr1" 9520001 9521000 "*" 2.88657986402541e-15 1.14155613124573e-14
86.8131868131868
"chr1" 9550001 9551000 "*" 4.14335232790108e-13 1.26153063645578e-12 -100
"chr1" 9711001 9712000 "*" 0 0 68.7546330615271
"chr1" 9733001 9734000 "*" 3.95353005888666e-09 7.03928529283733e-09 100
"chr1" 9770001 9771000 "*" 2.68450373042128e-10 5.64465213127486e-10 100
"chr1" 9798001 9799000 "*" 9.65729496371637e-10 1.87255912493232e-09 100
"chr1" 9885001 9886000 "*" 6.7387999880264e-06 7.57409559911036e-06 56
"chr1" 9938001 9939000 "*" 1.38560607432225e-10 3.02706178892116e-10
65.5172413793103
"chr1" 9965001 9966000 "*" 1.31491162957964e-07 1.87797836048466e-07 60
"chr1" 9989001 9990000 "*" 2.88710388929303e-10 6.03399264731139e-10 100
"chr1" 10057001 10058000 "*" 0 0 53.9705378554299
"chr1" 10193001 10194000 "*" 0.000320710178288164 0.000276619581787338
52.3809523809524
"chr1" 10231001 10232000 "*" 2.88710388929303e-10 6.03399264731139e-10 -100
"chr1" 10391001 10392000 "*" 2.8421709430404e-14 9.99616402633955e-14 100
"chr1" 10482001 10483000 "*" 4.99900121297969e-12 1.32921056339624e-11 -100
"chr1" 10672001 10673000 "*" 1.20591092667155e-10 2.66075324129686e-10 -100
"chr1" 10740001 10741000 "*" 1.4432899320127e-15 5.90750815956055e-15 -100
"chr1" 10741001 10742000 "*" 0 0 -51.5491452991453
"chr1" 10753001 10754000 "*" 0 0 -87.7358490566038
"chr1" 10754001 10755000 "*" 0 0 -57.5545851528384
"chr1" 10773001 10774000 "*" 4.14275443949563e-08 6.34857724979108e-08
-71.4285714285714
"chr1" 10793001 10794000 "*" 5.55111512312578e-16 2.36485094870365e-15
-96.6666666666667
"chr1" 10839001 10840000 "*" 0 0 -64.5161290322581
"chr1" 10856001 10857000 "*" 0 0 -53.4591194968553
"chr1" 10882001 10883000 "*" 0 0 100
"chr1" 10958001 10959000 "*" 0 0 -100
"chr1" 10967001 10968000 "*" 2.70135654997228e-05 2.7705430412866e-05
-57.9124579124579
"chr1" 10968001 10969000 "*" 2.98204186455209e-07 4.06273474085955e-07
55.7522123893805
"chr1" 10969001 10970000 "*" 7.41503747292427e-10 1.46215697670718e-09
-56.5217391304348
"chr1" 11002001 11003000 "*" 1.35890187991095e-11 3.40841971363279e-11 -100
"chr1" 11015001 11016000 "*" 1.11022302462516e-15 4.59817122935606e-15 -100
"chr1" 11022001 11023000 "*" 3.34128893442198e-08 5.19772864618523e-08 -100
"chr1" 11039001 11040000 "*" 1.50623957750895e-12 4.28756717183481e-12 -100
"chr1" 11043001 11044000 "*" 2.88779000712225e-11 6.92935809466068e-11 -100
"chr1" 11064001 11065000 "*" 5.99520433297585e-15 2.28531569014092e-14
-97.2222222222222
"chr1" 11072001 11073000 "*" 0 0 73.9130434782609
"chr1" 11121001 11122000 "*" 7.0006535457523e-08 1.03695832512054e-07 100
"chr1" 11161001 11162000 "*" 7.9747319858825e-12 2.06661344737636e-11
57.1428571428571

"chr1" 11170001 11171000 "*" 4.26951363152739e-10 8.72510873516957e-10 -100
"chr1" 11245001 11246000 "*" 1.29065647058724e-11 3.24642305381743e-11
91.0714285714286
"chr1" 11296001 11297000 "*" 1.98124849859482e-10 4.25157611354621e-10 -100
"chr1" 11368001 11369000 "*" 1.44040334881801e-07 2.04203102566604e-07
62.962962962963
"chr1" 11388001 11389000 "*" 2.22044604925031e-16 9.81641919380259e-16 100
"chr1" 11405001 11406000 "*" 7.53263278910898e-05 7.19956482159304e-05
-61.7647058823529
"chr1" 11407001 11408000 "*" 1.66533453693773e-15 6.7629186866784e-15 100
"chr1" 11412001 11413000 "*" 2.10681916357203e-10 4.50388810476048e-10
-54.5454545454545
"chr1" 11433001 11434000 "*" 3.35389271910458e-10 6.95415534233929e-10
64.2857142857143
"chr1" 11457001 11458000 "*" 6.91004131692097e-09 1.18592913246344e-08
-80.7692307692308
"chr1" 11477001 11478000 "*" 2.19853864003028e-08 3.50003158916803e-08
-66.6666666666667
"chr1" 11490001 11491000 "*" 1.66533453693773e-13 5.33905055233007e-13
95.4128440366973
"chr1" 11502001 11503000 "*" 2.15125472990962e-10 4.58126659943874e-10 100
"chr1" 11511001 11512000 "*" 9.06273666778645e-07 1.15739767942151e-06
52.3809523809524
"chr1" 11512001 11513000 "*" 4.01313771103418e-09 7.11882087237587e-09 -100
"chr1" 11583001 11584000 "*" 0 0 54.2193736998932
"chr1" 11591001 11592000 "*" 2.55121024306249e-09 4.66157024773554e-09
50.7102272727273
"chr1" 11592001 11593000 "*" 8.67402699111786e-07 1.11049351681381e-06
-70.4968944099379
"chr1" 11631001 11632000 "*" 9.61342117022923e-13 2.80092934798378e-12
51.3565426170468
"chr1" 11639001 11640000 "*" 1.12132525487141e-14 4.14352271980498e-14 100
"chr1" 11655001 11656000 "*" 3.6700841921089e-08 5.65530226072258e-08 -100
"chr1" 11750001 11751000 "*" 0 0 67.0924900244178
"chr1" 11768001 11769000 "*" 0 0 68.7652173913043
"chr1" 11769001 11770000 "*" 3.10862446895044e-15 1.22466437093774e-14 100
"chr1" 11914001 11915000 "*" 2.00227675550835e-08 3.20459176617961e-08 100
"chr1" 11943001 11944000 "*" 0 0 -100
"chr1" 11949001 11950000 "*" 1.30021882149833e-10 2.85539026058085e-10
-77.2727272727273
"chr1" 11950001 11951000 "*" 0 0 -54.7619047619048
"chr1" 11971001 11972000 "*" 3.13162829002067e-11 7.46761921480325e-11
-73.0769230769231
"chr1" 12039001 12040000 "*" 0 0 62.5
"chr1" 12145001 12146000 "*" 0.00058133904866009 0.000479945375509325
52.4786324786325
"chr1" 12172001 12173000 "*" 1.26565424807268e-14 4.63923203146481e-14
72.8813559322034
"chr1" 12205001 12206000 "*" 1.89425159169421e-05 1.98874471894124e-05 62.5
"chr1" 12242001 12243000 "*" 2.71560551823313e-13 8.46002737721407e-13 -75
"chr1" 12246001 12247000 "*" 5.48638023900594e-10 1.10374357336421e-09 100
"chr1" 12271001 12272000 "*" 4.95355856333646e-09 8.67483356500546e-09

67.741935483871
"chr1" 12336001 12337000 "*" 8.71525074330748e-14 2.88848349331391e-13 -100
"chr1" 12436001 12437000 "*" 2.08939601620273e-07 2.90467202288664e-07
-93.3333333333333
"chr1" 12644001 12645000 "*" 1.6899581734009e-10 3.65517866989809e-10
64.7887323943662
"chr1" 12650001 12651000 "*" 5.03441732746523e-12 1.3378171545226e-11 -100
"chr1" 12690001 12691000 "*" 4.57183624291702e-10 9.31326369875184e-10
-96.7741935483871
"chr1" 13208001 13209000 "*" 5.08482145278322e-14 1.73432706170696e-13 -100
"chr1" 13794001 13795000 "*" 0 0 100
"chr1" 13795001 13796000 "*" 2.52449542825772e-07 3.47341761878397e-07
66.6666666666667
"chr1" 13828001 13829000 "*" 0 0 100
"chr1" 13830001 13831000 "*" 2.33146835171283e-15 9.32818831833246e-15 100
"chr1" 13955001 13956000 "*" 0 0 100
"chr1" 14026001 14027000 "*" 0 0 67.4977738201247
"chr1" 14155001 14156000 "*" 1.28785870856518e-14 4.71440608755782e-14 -100
"chr1" 14220001 14221000 "*" 4.05163802308373e-06 4.70560416368343e-06
-51.6949152542373
"chr1" 14250001 14251000 "*" 9.65729496371637e-10 1.87255912493232e-09 100
"chr1" 14308001 14309000 "*" 1.5277158427196e-09 2.87642564232045e-09 100
"chr1" 14853001 14854000 "*" 5.10702591327572e-15 1.96271840288234e-14
-66.6666666666667
"chr1" 14908001 14909000 "*" 1.92946192356658e-09 3.59193011611483e-09 -100
"chr1" 14918001 14919000 "*" 0 0 100
"chr1" 14953001 14954000 "*" 3.99680288865056e-15 1.5566998858822e-14 100
"chr1" 14965001 14966000 "*" 3.44672423935322e-09 6.19257304743119e-09
-59.4594594594595
"chr1" 15041001 15042000 "*" 8.3882012447134e-11 1.88922989503423e-10 -100
"chr1" 15052001 15053000 "*" 1.37234668073916e-12 3.92653846299e-12
59.1397849462366
"chr1" 15062001 15063000 "*" 0 0 100
"chr1" 15174001 15175000 "*" 0 0 -100
"chr1" 15220001 15221000 "*" 0 0 -100
"chr1" 15225001 15226000 "*" 0 0 100
"chr1" 15236001 15237000 "*" 1.17964038426344e-06 1.48285273216832e-06
-76.7441860465116
"chr1" 15237001 15238000 "*" 1.07864655669587e-05 1.17574627643031e-05
63.8888888888889
"chr1" 15256001 15257000 "*" 0 0 100
"chr1" 15262001 15263000 "*" 0 0 100
"chr1" 15276001 15277000 "*" 7.54067528419e-08 1.11163710242092e-07
57.8947368421053
"chr1" 15299001 15300000 "*" 2.62834454201055e-09 4.79566202489418e-09
71.4285714285714
"chr1" 15349001 15350000 "*" 0 0 100
"chr1" 15372001 15373000 "*" 1.33226762955019e-14 4.8714125500746e-14
67.741935483871
"chr1" 15396001 15397000 "*" 1.98365768255826e-11 4.85612402473442e-11 100
"chr1" 15410001 15411000 "*" 8.19565414096335e-05 7.7896169099937e-05
54.5454545454545

"chr1" 15430001 15431000 "*" 0 0 100
"chr1" 15455001 15456000 "*" 1.70641278884887e-12 4.81986032810646e-12 -100
"chr1" 15466001 15467000 "*" 8.88178419700125e-16 3.70670032207938e-15 100
"chr1" 15573001 15574000 "*" 0 0 -89.1156462585034
"chr1" 15586001 15587000 "*" 0.000126980445890656 0.000117107414287018 -55
"chr1" 15613001 15614000 "*" 8.7349629751543e-09 1.47505871898511e-08 -100
"chr1" 15704001 15705000 "*" 0 0 -100
"chr1" 15709001 15710000 "*" 0 0 -100
"chr1" 15737001 15738000 "*" 0 0 -78.2371649250341
"chr1" 15742001 15743000 "*" 0 0 -100
"chr1" 15745001 15746000 "*" 7.21644966006352e-15 2.72345776413425e-14 100
"chr1" 15751001 15752000 "*" 0 0 75.8620689655172
"chr1" 15757001 15758000 "*" 1.88737914186277e-15 7.62011598380321e-15 100
"chr1" 15758001 15759000 "*" 1.91740926187567e-05 2.01115878933737e-05
-63.6363636363636
"chr1" 15760001 15761000 "*" 4.0690371269636e-05 4.05550415736729e-05
61.5384615384615
"chr1" 15770001 15771000 "*" 4.41868763800812e-14 1.52055466537903e-13 -93.75
"chr1" 15771001 15772000 "*" 2.79440914852103e-11 6.73046092948135e-11 100
"chr1" 15885001 15886000 "*" 7.93809462606987e-14 2.64679416473686e-13 -100
"chr1" 15909001 15910000 "*" 1.72792472219463e-07 2.4268726721039e-07
-57.1428571428571
"chr1" 15910001 15911000 "*" 2.29234809689949e-07 3.17032749035844e-07
53.6842105263158
"chr1" 15929001 15930000 "*" 0 0 70.3024967989757
"chr1" 15930001 15931000 "*" 0 0 70.0164914450629
"chr1" 15937001 15938000 "*" 1.93720595120794e-11 4.76309924836636e-11 100
"chr1" 16046001 16047000 "*" 1.78335124445539e-12 5.02798204013913e-12
84.9315068493151
"chr1" 16083001 16084000 "*" 0 0 -71.8779707890416
"chr1" 16113001 16114000 "*" 1.55431223447522e-13 4.99540360939345e-13
-57.4361820199778
"chr1" 16119001 16120000 "*" 3.99680288865056e-14 1.37975970265307e-13 -100
"chr1" 16324001 16325000 "*" 5.55111512312578e-16 2.36485094870365e-15 -100
"chr1" 16364001 16365000 "*" 5.72310811678411e-05 5.57606548799911e-05
58.974358974359
"chr1" 16366001 16367000 "*" 0 0 90.4564315352697
"chr1" 16380001 16381000 "*" 3.04324343503026e-11 7.26599935673121e-11 100
"chr1" 16382001 16383000 "*" 1.37828859436695e-10 3.01445544267661e-10 -100
"chr1" 16393001 16394000 "*" 6.59550958292954e-09 1.13456194382773e-08 -100
"chr1" 16400001 16401000 "*" 0 0 -100
"chr1" 16470001 16471000 "*" 6.52733422867868e-12 1.71146951718423e-11 -100
"chr1" 16471001 16472000 "*" 1.20770060618725e-12 3.4790878479756e-12
-77.1929824561404
"chr1" 16479001 16480000 "*" 0 0 -75.5467571644042
"chr1" 16483001 16484000 "*" 6.66133814775094e-16 2.81595744474255e-15 -100
"chr1" 16492001 16493000 "*" 9.3319242466805e-07 1.18962595148735e-06
62.0689655172414
"chr1" 16499001 16500000 "*" 3.34128893442198e-08 5.19772864618523e-08 -100
"chr1" 16502001 16503000 "*" 2.61800248146926e-10 5.51859551681871e-10
-75.3731343283582
"chr1" 16530001 16531000 "*" 2.00227675550835e-08 3.20459176617961e-08 -100

"chr1" 16544001 16545000 "*" 8.87130147120274e-10 1.73467299476368e-09
52.5423728813559
"chr1" 16724001 16725000 "*" 1.34141927565778e-08 2.20765850734954e-08
-54.1666666666667
"chr1" 16744001 16745000 "*" 2.08995487582797e-10 4.46944006972663e-10 100
"chr1" 16811001 16812000 "*" 4.52932612748924e-07 6.02009555421146e-07
-57.396449704142
"chr1" 16828001 16829000 "*" 5.05765823011206e-06 5.79476070757596e-06
64.7058823529412
"chr1" 16863001 16864000 "*" 0 0 -52.3145631067961
"chr1" 17021001 17022000 "*" 0 0 -59.3690579083838
"chr1" 17030001 17031000 "*" 0 0 -51.3541383989145
"chr1" 17034001 17035000 "*" 0 0 60.5038649599549
"chr1" 17043001 17044000 "*" 1.41424205679641e-10 3.08669457816074e-10 -75
"chr1" 17053001 17054000 "*" 8.8491491911924e-10 1.73042253255398e-09
-68.1818181818182
"chr1" 17287001 17288000 "*" 0 0 -74.1757728588774
"chr1" 17290001 17291000 "*" 5.55111512312578e-16 2.36485094870365e-15 -100
"chr1" 17381001 17382000 "*" 7.01366742461573e-11 1.5957245588199e-10
55.7692307692308
"chr1" 17392001 17393000 "*" 1.14130926931466e-13 3.7276674571693e-13 100
"chr1" 17410001 17411000 "*" 0 0 100
"chr1" 17416001 17417000 "*" 1.49765422285952e-09 2.83328583432219e-09
-63.5220125786164
"chr1" 17463001 17464000 "*" 4.55497665141635e-08 6.94836989755125e-08
89.4736842105263
"chr1" 17516001 17517000 "*" 1.95827080107236e-08 3.1498775248764e-08
63.6363636363636
"chr1" 17528001 17529000 "*" 0 0 -100
"chr1" 17550001 17551000 "*" 1.34559030584569e-13 4.35581312463895e-13 100
"chr1" 17552001 17553000 "*" 0 0 -100
"chr1" 17558001 17559000 "*" 0 0 -100
"chr1" 17559001 17560000 "*" 1.27663487082819e-05 1.37621484893738e-05
-54.2857142857143
"chr1" 17572001 17573000 "*" 2.96831905299832e-06 3.52052627123658e-06
68.4210526315789
"chr1" 17603001 17604000 "*" 0 0 59.3023255813954
"chr1" 17678001 17679000 "*" 0.000171200504948343 0.00015450134106212
-50.6493506493507
"chr1" 17701001 17702000 "*" 0 0 67.0068027210884
"chr1" 17704001 17705000 "*" 3.39728245535298e-14 1.18461294664839e-13
79.1666666666667
"chr1" 17770001 17771000 "*" 2.00227675550835e-08 3.20459176617961e-08 -100
"chr1" 17779001 17780000 "*" 1.56863411149288e-12 4.4469621057462e-12 100
"chr1" 17787001 17788000 "*" 2.61335397766516e-12 7.2182660429145e-12
65.7894736842105
"chr1" 17800001 17801000 "*" 7.67056418382595e-11 1.7359448580318e-10 -100
"chr1" 17888001 17889000 "*" 0 0 -100
"chr1" 17896001 17897000 "*" 0 0 -100
"chr1" 17907001 17908000 "*" 1.52701395972343e-09 2.87642564232045e-09
64.7058823529412
"chr1" 17909001 17910000 "*" 5.83442053749827e-05 5.67762044471538e-05

61.3636363636364
"chr1" 18029001 18030000 "*" 8.16442388140715e-08 1.19833628183695e-07
-60.9756097560976
"chr1" 18034001 18035000 "*" 3.88022947106492e-13 1.1867462770515e-12 -100
"chr1" 18043001 18044000 "*" 1.20591092667155e-10 2.66075324129686e-10 100
"chr1" 18062001 18063000 "*" 0 0 -100
"chr1" 18071001 18072000 "*" 7.67056418382595e-11 1.7359448580318e-10 -100
"chr1" 18090001 18091000 "*" 0 0 -57.6470588235294
"chr1" 18092001 18093000 "*" 0 0 -88.3720930232558
"chr1" 18094001 18095000 "*" 2.41076204166024e-06 2.8989450732821e-06
73.9130434782609
"chr1" 18099001 18100000 "*" 0 0 100
"chr1" 18104001 18105000 "*" 1.37828859436695e-10 3.01445544267661e-10 -100
"chr1" 18117001 18118000 "*" 1.72228897810101e-12 4.86272728111482e-12
57.8571428571429
"chr1" 18133001 18134000 "*" 1.12843068222901e-12 3.259512533505e-12 -100
"chr1" 18145001 18146000 "*" 1.20821130877857e-11 3.05248751139931e-11
74.390243902439
"chr1" 18175001 18176000 "*" 1.07882591748876e-11 2.74553537455803e-11 100
"chr1" 18195001 18196000 "*" 6.88338275267597e-15 2.60518659957509e-14 -100
"chr1" 18209001 18210000 "*" 4.02167188440217e-12 1.08266700497926e-11 -100
"chr1" 18306001 18307000 "*" 0 0 -100
"chr1" 18357001 18358000 "*" 0 0 -100
"chr1" 18368001 18369000 "*" 1.78972134889932e-08 2.89243321796634e-08
79.1666666666667
"chr1" 18406001 18407000 "*" 0 0 79.0123456790123
"chr1" 18473001 18474000 "*" 3.87008415980716e-07 5.19019290312119e-07
58.0645161290323
"chr1" 18495001 18496000 "*" 0 0 -100
"chr1" 18496001 18497000 "*" 4.65637306490407e-10 9.46720524046618e-10 100
"chr1" 18548001 18549000 "*" 2.17435180971393e-10 4.62797203928805e-10
66.6666666666667
"chr1" 18609001 18610000 "*" 1.38590361409285e-10 3.02706178892116e-10 100
"chr1" 18610001 18611000 "*" 2.82773804372027e-13 8.79129664109438e-13 100
"chr1" 18614001 18615000 "*" 6.92287338566189e-11 1.57605630124247e-10 -100
"chr1" 18622001 18623000 "*" 1.11022302462516e-15 4.59817122935606e-15 -100
"chr1" 18629001 18630000 "*" 4.73234496034536e-09 8.30627300264826e-09 100
"chr1" 18641001 18642000 "*" 3.56775498033812e-10 7.35898629809829e-10 100
"chr1" 18688001 18689000 "*" 2.43005615629954e-09 4.45014222280278e-09 100
"chr1" 18691001 18692000 "*" 0 0 100
"chr1" 18735001 18736000 "*" 5.2706854991591e-08 7.96829356899468e-08
54.1666666666667
"chr1" 18801001 18802000 "*" 1.66084590524918e-10 3.59444950324497e-10 75
"chr1" 18810001 18811000 "*" 1.89581683684992e-12 5.32311367606535e-12 100
"chr1" 18898001 18899000 "*" 0 0 100
"chr1" 18902001 18903000 "*" 1.28785870856518e-14 4.71440608755782e-14 -100
"chr1" 18960001 18961000 "*" 0 0 66.3265306122449
"chr1" 18961001 18962000 "*" 0 0 72.0588235294118
"chr1" 18970001 18971000 "*" 0 0 76.9652650822669
"chr1" 18973001 18974000 "*" 0 0 67.3659673659674
"chr1" 18999001 19000000 "*" 2.15125472990962e-10 4.58126659943874e-10 100
"chr1" 19037001 19038000 "*" 2.32890674078945e-07 3.21800664964487e-07

-53.8461538461538
"chr1" 19040001 19041000 "*" 4.74065231514942e-14 1.62745413342582e-13
82.1428571428571
"chr1" 19134001 19135000 "*" 3.6700841921089e-08 5.65530226072258e-08 -100
"chr1" 19144001 19145000 "*" 0 0 54.2222222222222
"chr1" 19152001 19153000 "*" 1.12634901405784e-10 2.49563754426169e-10 -100
"chr1" 19178001 19179000 "*" 9.55859280615812e-09 1.60640249482754e-08
64.1509433962264
"chr1" 19188001 19189000 "*" 2.39808173319034e-14 8.51795945223052e-14
93.0555555555556
"chr1" 19200001 19201000 "*" 6.81343870212459e-13 2.02042857428443e-12
-63.2352941176471
"chr1" 19202001 19203000 "*" 2.22044604925031e-16 9.81641919380259e-16
52.112676056338
"chr1" 19210001 19211000 "*" 2.3037127760972e-13 7.24640760143703e-13
-95.1612903225806
"chr1" 19234001 19235000 "*" 1.88737914186277e-15 7.62011598380321e-15 -58
"chr1" 19240001 19241000 "*" 8.26320183389129e-08 1.21195996016357e-07
-82.4561403508772
"chr1" 19247001 19248000 "*" 1.07882591748876e-11 2.74553537455803e-11 100
"chr1" 19249001 19250000 "*" 8.50465424750979e-08 1.24562122074462e-07
-78.9473684210526
"chr1" 19360001 19361000 "*" 0 0 57.2652012560662
"chr1" 19369001 19370000 "*" 9.41103716749492e-05 8.86297231089954e-05 60
"chr1" 19383001 19384000 "*" 3.33274787911453e-06 3.92179778116701e-06
69.7674418604651
"chr1" 19395001 19396000 "*" 6.6724275571417e-07 8.68002540508539e-07
52.2246941045606
"chr1" 19422001 19423000 "*" 2.17381668221606e-13 6.85151076607736e-13 100
"chr1" 19595001 19596000 "*" 3.66373598126302e-14 1.27257562929612e-13
53.5714285714286
"chr1" 19657001 19658000 "*" 1.17017506795491e-13 3.81716338420629e-13
93.0817610062893
"chr1" 19666001 19667000 "*" 9.30777677154992e-12 2.39177677466606e-11
55.009900990099
"chr1" 19740001 19741000 "*" 3.06475578248921e-08 4.79888042921933e-08
-72.2222222222222
"chr1" 19814001 19815000 "*" 7.01606883479755e-07 9.09712121528379e-07
59.7014925373134
"chr1" 19967001 19968000 "*" 6.5536465143623e-13 1.94602017656154e-12 100
"chr1" 19971001 19972000 "*" 0 0 -52.1079258010118
"chr1" 19972001 19973000 "*" 0 0 -64.0569183138843
"chr1" 19981001 19982000 "*" 6.37490060739765e-13 1.89496793587877e-12 -100
"chr1" 19993001 19994000 "*" 1.11022302462516e-16 5.03662826618488e-16
72.2222222222222
"chr1" 20010001 20011000 "*" 1.77892278796499e-07 2.49438289768752e-07
69.6969696969697
"chr1" 20030001 20031000 "*" 0 0 100
"chr1" 20113001 20114000 "*" 2.08037405902139e-06 2.52539366890153e-06
52.3809523809524
"chr1" 20203001 20204000 "*" 3.92132182280847e-08 6.02374149165085e-08
92.8571428571429

"chr1" 20248001 20249000 "*" 4.85364218816287e-08 7.37456306743013e-08 -50.6071871127633
"chr1" 20251001 20252000 "*" 0.000184447926999565 0.00016562323068884 -56.0975609756098
"chr1" 20282001 20283000 "*" 0 0 100
"chr1" 20286001 20287000 "*" 0 0 97.7777777777778
"chr1" 20303001 20304000 "*" 8.01666433236647e-09 1.36392368970109e-08 -100
"chr1" 20304001 20305000 "*" 3.77475828372553e-15 1.47379557022071e-14 -100
"chr1" 20397001 20398000 "*" 6.59550958292954e-09 1.13456194382773e-08 100
"chr1" 20401001 20402000 "*" 8.25450818808804e-13 2.42473843346228e-12 -100
"chr1" 20468001 20469000 "*" 8.98170426921752e-14 2.97124428237056e-13 -77.3584905660377
"chr1" 20474001 20475000 "*" 1.11022302462516e-15 4.59817122935606e-15 -75.609756097561
"chr1" 20479001 20480000 "*" 4.44089209850063e-16 1.91071758245033e-15 60
"chr1" 20493001 20494000 "*" 3.60178553648893e-12 9.77932168110656e-12 -63.3928571428571
"chr1" 20510001 20511000 "*" 3.56775498033812e-10 7.35898629809829e-10 100
"chr1" 20608001 20609000 "*" 0 0 -100
"chr1" 20620001 20621000 "*" 1.5277158427196e-09 2.87642564232045e-09 -100
"chr1" 20678001 20679000 "*" 2.08995487582797e-10 4.46944006972663e-10 100
"chr1" 20687001 20688000 "*" 0 0 -100
"chr1" 20728001 20729000 "*" 0 0 100
"chr1" 20740001 20741000 "*" 2.16382467499443e-13 6.82775854166559e-13 100
"chr1" 20771001 20772000 "*" 1.39779633423487e-07 1.98463037958589e-07 100
"chr1" 20781001 20782000 "*" 2.17381668221606e-13 6.85151076607736e-13 100
"chr1" 20807001 20808000 "*" 7.22205961700695e-10 1.4262284656542e-09 -91.6666666666667
"chr1" 20878001 20879000 "*" 0 0 61.5543751747274
"chr1" 20881001 20882000 "*" 0.000139451549244218 0.000127724714298755 58.3333333333333
"chr1" 20899001 20900000 "*" 1.39779633423487e-07 1.98463037958589e-07 100
"chr1" 20938001 20939000 "*" 0 0 -100
"chr1" 20967001 20968000 "*" 8.88178419700125e-16 3.70670032207938e-15 100
"chr1" 20968001 20969000 "*" 9.19333178561743e-06 1.01330895744113e-05 52.6315789473684
"chr1" 20984001 20985000 "*" 1.46438416948058e-13 4.72057283418642e-13 52.1739130434783
"chr1" 20998001 20999000 "*" 0.000231175672664174 0.000204278487663226 54.5454545454545
"chr1" 21055001 21056000 "*" 0 0 100
"chr1" 21111001 21112000 "*" 1.4432899320127e-15 5.90750815956055e-15 -100
"chr1" 21543001 21544000 "*" 0 0 -100
"chr1" 21609001 21610000 "*" 7.78377362564697e-13 2.29551678017433e-12 -100
"chr1" 21616001 21617000 "*" 0 0 -89.6636977886978
"chr1" 21667001 21668000 "*" 0 0 51.5151515151515
"chr1" 21669001 21670000 "*" 0 0 -100
"chr1" 21687001 21688000 "*" 2.64951482975562e-09 4.82186011375752e-09 -100
"chr1" 21761001 21762000 "*" 1.34559030584569e-13 4.35581312463895e-13 100
"chr1" 21835001 21836000 "*" 0 0 61.2029071150644
"chr1" 21849001 21850000 "*" 0 0 -100
"chr1" 21863001 21864000 "*" 3.61932706027801e-14 1.25777907157994e-13 100

"chr1" 21873001 21874000 "*" 6.90747459231034e-12 1.80140148623876e-11 -100
"chr1" 21887001 21888000 "*" 0 0 100
"chr1" 21894001 21895000 "*" 4.35258160624841e-08 6.65362255908573e-08 54.4303797468354
"chr1" 21899001 21900000 "*" 0 0 100
"chr1" 21913001 21914000 "*" 2.79443135298152e-13 8.69714977320713e-13 -72.6315789473684
"chr1" 21950001 21951000 "*" 8.65973959207622e-14 2.87362558212404e-13 100
"chr1" 21953001 21954000 "*" 0 0 -81.8716577540107
"chr1" 21961001 21962000 "*" 2.12513340258624e-11 5.18784705916391e-11 -51.5384615384615
"chr1" 21985001 21986000 "*" 0 0 70.2040816326531
"chr1" 22110001 22111000 "*" 0 0 -100
"chr1" 22142001 22143000 "*" 0 0 -100
"chr1" 22233001 22234000 "*" 1.94454008450862e-10 4.18033247412119e-10 85.15625
"chr1" 22254001 22255000 "*" 2.61657426647854e-06 3.12843692934624e-06 66.6666666666667
"chr1" 22257001 22258000 "*" 0 0 85.862977602108
"chr1" 22262001 22263000 "*" 2.87792012443333e-12 7.89099990797437e-12 70.2702702702703
"chr1" 22287001 22288000 "*" 0 0 100
"chr1" 22349001 22350000 "*" 8.88178419700125e-16 3.70670032207938e-15 -71.6981132075472
"chr1" 22378001 22379000 "*" 3.78218567576027e-11 8.92022287843478e-11 90.7692307692308
"chr1" 22490001 22491000 "*" 1.27218141301455e-05 1.37167995911245e-05 -60
"chr1" 22505001 22506000 "*" 3.73182817980933e-10 7.68334825632598e-10 -56.1309030654515
"chr1" 22559001 22560000 "*" 1.39779633423487e-07 1.98463037958589e-07 100
"chr1" 22593001 22594000 "*" 0 0 -66.6666666666667
"chr1" 22599001 22600000 "*" 0 0 -100
"chr1" 22604001 22605000 "*" 5.63660229602192e-13 1.68905058935478e-12 100
"chr1" 22618001 22619000 "*" 0 0 52.3809523809524
"chr1" 22652001 22653000 "*" 6.93882705948834e-06 7.78842164960861e-06 -57.8947368421053
"chr1" 22676001 22677000 "*" 2.50742828611372e-07 3.45135856254917e-07 -53.8706256627784
"chr1" 22690001 22691000 "*" 6.60512174028849e-05 6.37369119982594e-05 -54
"chr1" 22702001 22703000 "*" 1.11022302462516e-15 4.59817122935606e-15 100
"chr1" 22712001 22713000 "*" 1.11173455996649e-07 1.60300543120805e-07 -54.4117647058824
"chr1" 22715001 22716000 "*" 1.20681242776755e-13 3.92347864138717e-13 75
"chr1" 22724001 22725000 "*" 0 0 -100
"chr1" 22747001 22748000 "*" 3.10862446895044e-15 1.22466437093774e-14 -100
"chr1" 22774001 22775000 "*" 0 0 100
"chr1" 22870001 22871000 "*" 7.7715611723761e-16 3.26213507634405e-15 -61.7647058823529
"chr1" 22877001 22878000 "*" 9.70334923522387e-14 3.19199340968739e-13 -58.3219178082192
"chr1" 22883001 22884000 "*" 5.70663072352318e-10 1.14567661569139e-09 63.3333333333333
"chr1" 22889001 22890000 "*" 0 0 54.1360520806447

"chr1" 22890001 22891000 "*" 0 0 50.1786921665218
"chr1" 22892001 22893000 "*" 8.54733395172502e-10 1.67383678220771e-09
78.5714285714286
"chr1" 22897001 22898000 "*" 2.07611705604904e-13 6.56815148566745e-13
-54.3350662418246
"chr1" 22918001 22919000 "*" 0 0 -85.7142857142857
"chr1" 22943001 22944000 "*" 7.67056418382595e-11 1.7359448580318e-10 -100
"chr1" 22967001 22968000 "*" 5.99964522507435e-13 1.79332876977318e-12 -97.5
"chr1" 22968001 22969000 "*" 3.90831811358794e-12 1.05610567215048e-11 100
"chr1" 22988001 22989000 "*" 1.12634901405784e-10 2.49563754426169e-10 100
"chr1" 23006001 23007000 "*" 8.77835892658663e-05 8.3025776194848e-05
-58.4905660377358
"chr1" 23038001 23039000 "*" 0 0 -65.8536585365854
"chr1" 23166001 23167000 "*" 6.77335965093562e-12 1.76897011246462e-11 -100
"chr1" 23183001 23184000 "*" 1.66533453693773e-15 6.7629186866784e-15 100
"chr1" 23208001 23209000 "*" 1.14679907792237e-08 1.90241357300708e-08
-52.0956769804696
"chr1" 23415001 23416000 "*" 2.17218465436986e-11 5.29774576328594e-11
52.2727272727273
"chr1" 23419001 23420000 "*" 1.35880195983873e-11 3.40841971363279e-11
54.5454545454545
"chr1" 23495001 23496000 "*" 0 0 -100
"chr1" 23504001 23505000 "*" 0 0 -63.5454296661193
"chr1" 23544001 23545000 "*" 4.34971479668089e-06 5.02694167065183e-06
-68.3168316831683
"chr1" 23671001 23672000 "*" 0 0 71.4285714285714
"chr1" 23719001 23720000 "*" 1.0325074129014e-14 3.83008663711937e-14 100
"chr1" 23764001 23765000 "*" 2.64951482975562e-09 4.82186011375752e-09 -100
"chr1" 23854001 23855000 "*" 3.33066907387547e-16 1.4495649018245e-15 -96.875
"chr1" 23867001 23868000 "*" 7.67056418382595e-11 1.7359448580318e-10 -100
"chr1" 23884001 23885000 "*" 0 0 -100
"chr1" 23917001 23918000 "*" 0 0 -100
"chr1" 23939001 23940000 "*" 0.000166834440645691 0.000150915514744356
-51.3513513513514
"chr1" 23965001 23966000 "*" 3.6700841921089e-08 5.65530226072258e-08 100
"chr1" 24124001 24125000 "*" 6.93889390390723e-14 2.32918211295437e-13
-71.1538461538462
"chr1" 24127001 24128000 "*" 0 0 -59.0421504290762
"chr1" 24195001 24196000 "*" 0 0 -82.5
"chr1" 24238001 24239000 "*" 1.51580216822822e-07 2.14305742156995e-07
-51.0204081632653
"chr1" 24394001 24395000 "*" 1.35888533758788e-09 2.58353624037957e-09
72.8813559322034
"chr1" 24397001 24398000 "*" 1.02273745028469e-12 2.96809159199506e-12
-70.1030927835052
"chr1" 24408001 24409000 "*" 6.98330282489223e-14 2.34267456154807e-13 -100
"chr1" 24426001 24427000 "*" 1.34635635973268e-11 3.38040939191647e-11 80
"chr1" 24429001 24430000 "*" 8.3882012447134e-11 1.88922989503423e-10 100
"chr1" 24463001 24464000 "*" 0 0 -98.5815602836879
"chr1" 24469001 24470000 "*" 6.93112234273485e-13 2.05218842007357e-12 -100
"chr1" 24514001 24515000 "*" 0 0 -71.6417910447761
"chr1" 24525001 24526000 "*" 1.35003119794419e-13 4.3679847476192e-13 100

"chr1" 24546001 24547000 "*" 0 0 67.4418604651163
"chr1" 24686001 24687000 "*" 6.77335965093562e-12 1.76897011246462e-11 -100
"chr1" 24693001 24694000 "*" 1.56863411149288e-12 4.4469621057462e-12 100
"chr1" 24740001 24741000 "*" 0 0 100
"chr1" 25034001 25035000 "*" 0 0 -83.0985915492958
"chr1" 25047001 25048000 "*" 0 0 100
"chr1" 25202001 25203000 "*" 4.78841410966879e-11 1.11468187156588e-10 -100
"chr1" 25239001 25240000 "*" 4.44089209850063e-16 1.91071758245033e-15 -100
"chr1" 25288001 25289000 "*" 7.58546558898843e-11 1.72056111659628e-10
55.0033806626099
"chr1" 25338001 25339000 "*" 1.22457599616155e-12 3.52441478267078e-12 -100
"chr1" 25367001 25368000 "*" 1.14124265593318e-11 2.89138062669327e-11 100
"chr1" 25382001 25383000 "*" 6.5555672723594e-07 8.53810159698914e-07
70.9090909090909
"chr1" 25395001 25396000 "*" 6.43996536897617e-05 6.22599140150143e-05
-52.3809523809524
"chr1" 25522001 25523000 "*" 9.41103768226093e-05 8.86297231089954e-05
-52.3809523809524
"chr1" 25566001 25567000 "*" 0 0 -58.2690582959641
"chr1" 25863001 25864000 "*" 1.11022302462516e-16 5.03662826618488e-16 -100
"chr1" 25868001 25869000 "*" 8.43347613965761e-12 2.17859001990012e-11 -100
"chr1" 25876001 25877000 "*" 2.29329899514852e-09 4.22120358762507e-09
66.6666666666667
"chr1" 25924001 25925000 "*" 2.15125472990962e-10 4.58126659943874e-10 -100
"chr1" 26061001 26062000 "*" 0.000241621856214458 0.000212792315955059
-57.6923076923077
"chr1" 26064001 26065000 "*" 5.73647682022838e-07 7.53130523349164e-07
69.4444444444444
"chr1" 26066001 26067000 "*" 1.11521902823597e-12 3.22514803664301e-12
-58.7179487179487
"chr1" 26081001 26082000 "*" 8.57092175010621e-13 2.51149198099779e-12 -100
"chr1" 26101001 26102000 "*" 0 0 -95.4545454545455
"chr1" 26122001 26123000 "*" 3.35486791236406e-07 4.540031731149e-07 75
"chr1" 26126001 26127000 "*" 0 0 -82.5949367088608
"chr1" 26201001 26202000 "*" 6.66133814775094e-16 2.81595744474255e-15
-50.3649635036496
"chr1" 26319001 26320000 "*" 1.11022302462516e-16 5.03662826618488e-16 -100
"chr1" 26346001 26347000 "*" 7.48072703782299e-08 1.10340422120243e-07
-90.9090909090909
"chr1" 26369001 26370000 "*" 4.44089209850063e-16 1.91071758245033e-15 72.5
"chr1" 26372001 26373000 "*" 0 0 80.2753839060893
"chr1" 26373001 26374000 "*" 0 0 57.1428571428571
"chr1" 26392001 26393000 "*" 0 0 -100
"chr1" 26493001 26494000 "*" 0 0 -100
"chr1" 26516001 26517000 "*" 1.283454974077e-06 1.6057763296708e-06
-59.4594594594595
"chr1" 26536001 26537000 "*" 1.96644922567657e-11 4.82930612417392e-11 -100
"chr1" 26546001 26547000 "*" 9.41469124882133e-14 3.10868561210786e-13 -100
"chr1" 26572001 26573000 "*" 2.4535928844216e-14 8.69144396197119e-14 100
"chr1" 26682001 26683000 "*" 6.83719338923083e-08 1.01840744031014e-07
-66.6666666666667
"chr1" 26703001 26704000 "*" 1.25284005392245e-10 2.75649590272477e-10 100

"chr1" 26758001 26759000 "*" 0 0 63.2911392405063
"chr1" 26797001 26798000 "*" 2.09067207990188e-11 5.1073083948126e-11
66.6666666666667
"chr1" 26855001 26856000 "*" 1.92946192356658e-09 3.59193011611483e-09 -100
"chr1" 27155001 27156000 "*" 2.09609618073703e-05 2.18689338025294e-05
-67.6470588235294
"chr1" 27179001 27180000 "*" 2.02327044007689e-12 5.65300384792621e-12 -100
"chr1" 27189001 27190000 "*" 0 0 -100
"chr1" 27190001 27191000 "*" 0 0 -100
"chr1" 27273001 27274000 "*" 0 0 -100
"chr1" 27282001 27283000 "*" 3.04324343503026e-11 7.26599935673121e-11 -100
"chr1" 27355001 27356000 "*" 0 0 -100
"chr1" 27424001 27425000 "*" 1.0012100370993e-08 1.67889687363554e-08 60
"chr1" 27429001 27430000 "*" 1.93720595120794e-11 4.76309924836636e-11 -100
"chr1" 27557001 27558000 "*" 1.23623818034657e-05 1.33534251888187e-05
-66.6666666666667
"chr1" 27633001 27634000 "*" 1.51992862740258e-11 3.79076276523832e-11
-91.0714285714286
"chr1" 27668001 27669000 "*" 7.7715611723761e-16 3.26213507634405e-15 -100
"chr1" 27675001 27676000 "*" 4.19442258703384e-13 1.27549685387611e-12
-79.7101449275362
"chr1" 27696001 27697000 "*" 8.7937398474125e-09 1.48447329671976e-08
-59.2592592592593
"chr1" 27719001 27720000 "*" 1.27178040321141e-07 1.81964954572368e-07
-52.0833333333333
"chr1" 27822001 27823000 "*" 6.90747459231034e-12 1.80140148623876e-11 -100
"chr1" 27854001 27855000 "*" 0 0 -65.7472249682574
"chr1" 27864001 27865000 "*" 0 0 -58.0427201394943
"chr1" 27867001 27868000 "*" 2.88657986402541e-15 1.14155613124573e-14 100
"chr1" 27869001 27870000 "*" 1.76181497903993e-05 1.86066350745324e-05
61.1940298507463
"chr1" 27887001 27888000 "*" 1.48487888651516e-11 3.70689457767456e-11 -100
"chr1" 27895001 27896000 "*" 5.41788836017076e-14 1.84152775539899e-13
-64.1304347826087
"chr1" 27908001 27909000 "*" 2.33028818463765e-10 4.93719283454501e-10 100
"chr1" 27911001 27912000 "*" 0 0 100
"chr1" 27956001 27957000 "*" 1.67299207820548e-08 2.71377429441055e-08 100
"chr1" 27962001 27963000 "*" 6.93112234273485e-13 2.05218842007357e-12 -100
"chr1" 27973001 27974000 "*" 3.43503003819023e-13 1.05967782244095e-12 100
"chr1" 27981001 27982000 "*" 5.62383823043966e-06 6.40002221520473e-06
72.7272727272727
"chr1" 28286001 28287000 "*" 0 0 -66.9395266197094
"chr1" 28494001 28495000 "*" 9.99200722162641e-16 4.15382497462808e-15 100
"chr1" 28549001 28550000 "*" 3.6700841921089e-08 5.65530226072258e-08 100
"chr1" 28663001 28664000 "*" 0 0 100
"chr1" 28854001 28855000 "*" 0 0 -76.9230769230769
"chr1" 28867001 28868000 "*" 9.08108033215171e-11 2.03407753381119e-10 -100
"chr1" 28892001 28893000 "*" 2.32862840121584e-10 4.93719283454501e-10
-91.6666666666667
"chr1" 28916001 28917000 "*" 7.0006535457523e-08 1.03695832512054e-07 100
"chr1" 28918001 28919000 "*" 0 0 54.0528149642399
"chr1" 28996001 28997000 "*" 6.4152538836737e-10 1.27477697728417e-09 100

"chr1" 29212001 29213000 "*" 6.59550958292954e-09 1.13456194382773e-08 -100
"chr1" 29570001 29571000 "*" 1.66533453693773e-15 6.7629186866784e-15
59.6491228070175
"chr1" 29577001 29578000 "*" 2.88710388929303e-10 6.03399264731139e-10 -100
"chr1" 29652001 29653000 "*" 1.10296338728944e-09 2.12222667509509e-09 -87.5
"chr1" 29743001 29744000 "*" 0 0 100
"chr1" 29775001 29776000 "*" 2.30926389122033e-14 8.22700632271376e-14 82.5
"chr1" 29776001 29777000 "*" 7.0006535457523e-08 1.03695832512054e-07 100
"chr1" 29850001 29851000 "*" 0 0 58.6206896551724
"chr1" 29851001 29852000 "*" 5.55111512312578e-16 2.36485094870365e-15
79.3103448275862
"chr1" 29877001 29878000 "*" 6.62803145701218e-14 2.22896146391892e-13
51.5463917525773
"chr1" 30051001 30052000 "*" 6.24654217240561e-10 1.2462108191303e-09 -100
"chr1" 30108001 30109000 "*" 0 0 57.9710144927536
"chr1" 30170001 30171000 "*" 2.22258290327737e-07 3.07892984126868e-07
-54.1353383458647
"chr1" 30180001 30181000 "*" 0 0 100
"chr1" 30219001 30220000 "*" 6.24654217240561e-10 1.2462108191303e-09 100
"chr1" 30297001 30298000 "*" 1.37828859436695e-10 3.01445544267661e-10 100
"chr1" 30330001 30331000 "*" 6.30384633382164e-13 1.87665599796749e-12 100
"chr1" 30399001 30400000 "*" 6.24654217240561e-10 1.2462108191303e-09 100
"chr1" 30407001 30408000 "*" 2.15125472990962e-10 4.58126659943874e-10 100
"chr1" 30486001 30487000 "*" 0 0 100
"chr1" 30553001 30554000 "*" 8.97451579717767e-09 1.5135808627736e-08 -75
"chr1" 30557001 30558000 "*" 2.4535928844216e-14 8.69144396197119e-14 100
"chr1" 30575001 30576000 "*" 8.38218383591993e-13 2.46085600550496e-12
66.6666666666667
"chr1" 30719001 30720000 "*" 9.76996261670138e-15 3.63402770315216e-14
71.9298245614035
"chr1" 30720001 30721000 "*" 1.11022302462516e-15 4.59817122935606e-15
-78.7878787878788
"chr1" 30727001 30728000 "*" 4.78841410966879e-11 1.11468187156588e-10 100
"chr1" 30730001 30731000 "*" 0 0 100
"chr1" 30738001 30739000 "*" 6.24654217240561e-10 1.2462108191303e-09 -100
"chr1" 30758001 30759000 "*" 1.12812537089724e-10 2.49924141215607e-10
65.1162790697674
"chr1" 30778001 30779000 "*" 5.55111512312578e-16 2.36485094870365e-15 100
"chr1" 30800001 30801000 "*" 3.03050905481328e-08 4.74775180509824e-08
59.4594594594595
"chr1" 30803001 30804000 "*" 5.7835307716303e-06 6.56902676498958e-06
68.2926829268293
"chr1" 30809001 30810000 "*" 0 0 100
"chr1" 30835001 30836000 "*" 1.12055920098442e-11 2.84500040664577e-11 100
"chr1" 30852001 30853000 "*" 0 0 100
"chr1" 30855001 30856000 "*" 0 0 100
"chr1" 30881001 30882000 "*" 9.08108033215171e-11 2.03407753381119e-10 -100
"chr1" 30886001 30887000 "*" 9.14935793794314e-10 1.7831816022306e-09
61.1111111111111
"chr1" 30949001 30950000 "*" 1.39779633423487e-07 1.98463037958589e-07 100
"chr1" 30986001 30987000 "*" 4.73234496034536e-09 8.30627300264826e-09 100
"chr1" 30987001 30988000 "*" 4.73234496034536e-09 8.30627300264826e-09 100

"chr1" 31123001 31124000 "*" 3.43954076331698e-09 6.18113074869787e-09
70.8333333333333
"chr1" 31124001 31125000 "*" 5.1281201507436e-13 1.54461625452838e-12 100
"chr1" 31130001 31131000 "*" 0 0 -69.4779116465863
"chr1" 31159001 31160000 "*" 6.77335965093562e-12 1.76897011246462e-11 100
"chr1" 31177001 31178000 "*" 2.55351295663786e-15 1.01678090381833e-14 100
"chr1" 31188001 31189000 "*" 9.80763359414993e-10 1.8980032424715e-09 100
"chr1" 31190001 31191000 "*" 0 0 100
"chr1" 31218001 31219000 "*" 2.41784279456647e-05 2.49875818383709e-05
67.741935483871
"chr1" 31219001 31220000 "*" 2.08939601620273e-07 2.90467202288664e-07
90.9090909090909
"chr1" 31224001 31225000 "*" 3.34128893442198e-08 5.19772864618523e-08 -100
"chr1" 31229001 31230000 "*" 2.27526614993145e-07 3.14837392572814e-07 65
"chr1" 31247001 31248000 "*" 0 0 -100
"chr1" 31250001 31251000 "*" 1.11022302462516e-16 5.03662826618488e-16 100
"chr1" 31259001 31260000 "*" 3.75729793611335e-06 4.38666293266125e-06
50.6912442396313
"chr1" 31265001 31266000 "*" 4.01313771103418e-09 7.11882087237587e-09 100
"chr1" 31279001 31280000 "*" 1.15345036093562e-05 1.25186825636811e-05
-67.741935483871
"chr1" 31286001 31287000 "*" 0 0 -100
"chr1" 31303001 31304000 "*" 9.67004254448511e-14 3.18178990782609e-13 100
"chr1" 31317001 31318000 "*" 0 0 80
"chr1" 31320001 31321000 "*" 0 0 -100
"chr1" 31616001 31617000 "*" 3.6700841921089e-08 5.65530226072258e-08 -100
"chr1" 31654001 31655000 "*" 0 0 58.2715791671016
"chr1" 31661001 31662000 "*" 4.02167188440217e-12 1.08266700497926e-11 100
"chr1" 31668001 31669000 "*" 3.51358275807456e-10 7.25999680113292e-10
-60.8695652173913
"chr1" 31673001 31674000 "*" 1.30055021196895e-08 2.14458734449053e-08
-62.0253164556962
"chr1" 31695001 31696000 "*" 5.66546809466217e-13 1.69721259969255e-12 80
"chr1" 31707001 31708000 "*" 3.45022391545768e-07 4.66205089083695e-07
-77.2727272727273
"chr1" 31867001 31868000 "*" 0 0 -100
"chr1" 31921001 31922000 "*" 0 0 -100
"chr1" 31976001 31977000 "*" 7.105427357601e-15 2.68362283258525e-14
-73.9130434782609
"chr1" 32012001 32013000 "*" 0.000216481080482001 0.000192267358005665
-51.7241379310345
"chr1" 32015001 32016000 "*" 4.91162666094169e-12 1.30905424892112e-11
63.1578947368421
"chr1" 32043001 32044000 "*" 4.99866814607230e-12 1.32921056339624e-11
85.2941176470588
"chr1" 32079001 32080000 "*" 9.35527815004722e-06 1.03009706123167e-05
51.3513513513514
"chr1" 32082001 32083000 "*" 3.597027969926e-07 4.84612690151399e-07
-90.9090909090909
"chr1" 32121001 32122000 "*" 2.3990809339125e-12 6.65441738642456e-12 -100
"chr1" 32132001 32133000 "*" 6.92287338566189e-11 1.57605630124247e-10 -100
"chr1" 32157001 32158000 "*" 2.08814465718632e-09 3.86232567960463e-09 100

"chr1" 32162001 32163000 "*" 1.07343023358908e-11 2.7370927488588e-11 -62.5
"chr1" 32167001 32168000 "*" 4.86753970463383e-11 1.13145908161915e-10 100
"chr1" 32180001 32181000 "*" 0 0 59.7140551135709
"chr1" 32225001 32226000 "*" 3.63445940010365e-11 8.58565209111204e-11 100
"chr1" 32236001 32237000 "*" 1.11022302462516e-16 5.03662826618488e-16
-66.6666666666667
"chr1" 32281001 32282000 "*" 0 0 54.3010752688172
"chr1" 32304001 32305000 "*" 6.52733422867868e-12 1.71146951718423e-11 100
"chr1" 32313001 32314000 "*" 7.68718422250458e-13 2.26879652449338e-12
95.2380952380952
"chr1" 32314001 32315000 "*" 1.5976109324356e-12 4.52680115687763e-12
-84.6153846153846
"chr1" 32317001 32318000 "*" 4.73234496034536e-09 8.30627300264826e-09 -100
"chr1" 32320001 32321000 "*" 3.10862446895044e-15 1.22466437093774e-14 100
"chr1" 32573001 32574000 "*" 4.38728796638976e-05 4.35324780474722e-05
-55.3846153846154
"chr1" 32670001 32671000 "*" 0 0 82.0524166099387
"chr1" 32706001 32707000 "*" 0 0 -69.5145631067961
"chr1" 32714001 32715000 "*" 2.55684362571174e-13 8.00550138630046e-13
59.1970121381886
"chr1" 32718001 32719000 "*" 5.48638023900594e-10 1.10374357336421e-09 100
"chr1" 32726001 32727000 "*" 6.59550958292954e-09 1.13456194382773e-08 -100
"chr1" 32757001 32758000 "*" 1.88737914186277e-15 7.62011598380321e-15 100
"chr1" 32758001 32759000 "*" 8.11080119056395e-05 7.7152991702048e-05
55.5555555555556
"chr1" 32811001 32812000 "*" 5.37533351163688e-10 1.084227922306e-09
50.3043831168831
"chr1" 32816001 32817000 "*" 1.92379445707047e-11 4.73976539972576e-11
-68.3544303797468
"chr1" 32908001 32909000 "*" 2.33028818463765e-10 4.93719283454501e-10 100
"chr1" 33073001 33074000 "*" 1.65248346117863e-08 2.6910273049009e-08
54.5454545454545
"chr1" 33161001 33162000 "*" 3.574918139293e-14 1.24374547149444e-13
86.6666666666667
"chr1" 33169001 33170000 "*" 0 0 54.2372881355932
"chr1" 33184001 33185000 "*" 1.01145231079514e-08 1.6949472095466e-08
56.0733384262796
"chr1" 33199001 33200000 "*" 3.04324343503026e-11 7.26599935673121e-11 100
"chr1" 33238001 33239000 "*" 8.9865716423887e-08 1.31143716245552e-07
76.9230769230769
"chr1" 33342001 33343000 "*" 2.44548825634183e-12 6.77750390489873e-12 87.5
"chr1" 33350001 33351000 "*" 4.57402116182948e-10 9.31725943020103e-10
-66.6666666666667
"chr1" 33358001 33359000 "*" 0 0 55.8179723502304
"chr1" 33391001 33392000 "*" 0 0 70.0100704934542
"chr1" 33438001 33439000 "*" 0 0 63.7823670137912
"chr1" 33605001 33606000 "*" 2.02327044007689e-12 5.65300384792621e-12 -100
"chr1" 33609001 33610000 "*" 3.6700841921089e-08 5.65530226072258e-08 -100
"chr1" 33634001 33635000 "*" 6.30384633382164e-13 1.87665599796749e-12 100
"chr1" 33642001 33643000 "*" 2.08643580190682e-09 3.86232567960463e-09 -82
"chr1" 33731001 33732000 "*" 5.04263297784746e-13 1.52078941532818e-12 100
"chr1" 33760001 33761000 "*" 0 0 59.5168045412066

"chr1" 33781001 33782000 "*" 4.99020824662466e-11 1.15790792586157e-10 100
"chr1" 33800001 33801000 "*" 6.2680335232046e-05 6.07032716495799e-05
54.8387096774194
"chr1" 33839001 33840000 "*" 1.1669829780292e-07 1.6775923726183e-07
66.6666666666667
"chr1" 33896001 33897000 "*" 0 0 54.3362831858407
"chr1" 33911001 33912000 "*" 1.5277158427196e-09 2.87642564232045e-09 100
"chr1" 33932001 33933000 "*" 1.22124532708767e-14 4.48868324225892e-14
78.2608695652174
"chr1" 34015001 34016000 "*" 3.6892711108294e-13 1.13266255090498e-12 -100
"chr1" 34051001 34052000 "*" 7.0006535457523e-08 1.03695832512054e-07 -100
"chr1" 34068001 34069000 "*" 4.82766049358929e-10 9.79921753062737e-10
83.1325301204819
"chr1" 34073001 34074000 "*" 8.52140580320793e-12 2.19817259951489e-11 -100
"chr1" 34133001 34134000 "*" 1.11022302462516e-16 5.03662826618488e-16 100
"chr1" 34190001 34191000 "*" 2.16149320664272e-11 5.27274971275169e-11
-63.9354838709677
"chr1" 34204001 34205000 "*" 1.53210777398272e-14 5.5628583449216e-14 100
"chr1" 34239001 34240000 "*" 1.36593448846201e-05 1.46569770945072e-05
69.6969696969697
"chr1" 34388001 34389000 "*" 5.6362137179633e-11 1.29963270300065e-10 100
"chr1" 34629001 34630000 "*" 0 0 81.2962931285174
"chr1" 34849001 34850000 "*" 1.88737914186277e-15 7.62011598380321e-15
-93.3333333333333
"chr1" 34962001 34963000 "*" 3.56775498033812e-10 7.35898629809829e-10 100
"chr1" 35105001 35106000 "*" 3.95353005888666e-09 7.03928529283733e-09 -100
"chr1" 35162001 35163000 "*" 8.7349629751543e-09 1.47505871898511e-08 -100
"chr1" 35224001 35225000 "*" 1.11022302462516e-16 5.03662826618488e-16 100
"chr1" 35237001 35238000 "*" 4.73418833024652e-08 7.20303942950589e-08
52.0833333333333
"chr1" 35255001 35256000 "*" 4.64135408995148e-07 6.15986676197051e-07
59.2592592592593
"chr1" 35337001 35338000 "*" 0 0 100
"chr1" 35350001 35351000 "*" 0 0 60.0467020295203
"chr1" 35394001 35395000 "*" 0 0 85.4491235045784
"chr1" 36053001 36054000 "*" 5.1575292392414e-07 6.80463722959395e-07
56.5217391304348
"chr1" 36138001 36139000 "*" 0 0 86.4864864864865
"chr1" 36172001 36173000 "*" 0 0 80.6451612903226
"chr1" 36174001 36175000 "*" 0.000430302178842101 0.000363344149266076
-54.5454545454545
"chr1" 36236001 36237000 "*" 1.08779651952773e-12 3.14977564454477e-12
52.7777777777778
"chr1" 36349001 36350000 "*" 3.87601062357135e-11 9.13375868749433e-11
-55.2631578947368
"chr1" 36554001 36555000 "*" 0 0 81.9672131147541
"chr1" 36586001 36587000 "*" 1.26565424807268e-14 4.63923203146481e-14 100
"chr1" 36687001 36688000 "*" 1.22707838001723e-07 1.75940305659382e-07
-58.3333333333333
"chr1" 36795001 36796000 "*" 2.88710388929303e-10 6.03399264731139e-10 -100
"chr1" 36803001 36804000 "*" 3.09067238468685e-09 5.58438907638716e-09
95.4545454545455

"chr1" 36827001 36828000 "*" 1.87294624254264e-13 5.97285292681429e-13
-95.6521739130435
"chr1" 36952001 36953000 "*" 2.22044604925031e-16 9.81641919380259e-16 -68.75
"chr1" 36959001 36960000 "*" 0.000525299954949099 0.000436849389802675
-54.5454545454545
"chr1" 36982001 36983000 "*" 6.59550958292954e-09 1.13456194382773e-08 100
"chr1" 36983001 36984000 "*" 3.38200635274788e-08 5.25577529078797e-08
56.6666666666667
"chr1" 36987001 36988000 "*" 5.12403258712268e-08 7.7583945583216e-08
66.6666666666667
"chr1" 36992001 36993000 "*" 9.5812247025151e-14 3.15667542866807e-13 -100
"chr1" 37038001 37039000 "*" 2.70956115888676e-06 3.23307899068867e-06
61.9565217391304
"chr1" 37041001 37042000 "*" 2.82773804372027e-13 8.79129664109438e-13 100
"chr1" 37058001 37059000 "*" 8.7349629751543e-09 1.47505871898511e-08 100
"chr1" 37078001 37079000 "*" 9.61897228535236e-13 2.801372325071e-12 -100
"chr1" 37082001 37083000 "*" 6.77335965093562e-12 1.76897011246462e-11 -100
"chr1" 37170001 37171000 "*" 0 0 -100
"chr1" 37246001 37247000 "*" 1.2061079065262e-05 1.30541743073331e-05 -60
"chr1" 37249001 37250000 "*" 1.20591092667155e-10 2.66075324129686e-10 100
"chr1" 37322001 37323000 "*" 7.7715611723761e-16 3.26213507634405e-15
82.4742268041237
"chr1" 37324001 37325000 "*" 5.99503932185996e-06 6.79288714960205e-06
65.2173913043478
"chr1" 37345001 37346000 "*" 0.000525299926963374 0.000436849389802675
54.5454545454545
"chr1" 37346001 37347000 "*" 3.44169137633799e-15 1.3517917469209e-14
-61.2060041407867
"chr1" 37354001 37355000 "*" 6.24654217240561e-10 1.2462108191303e-09 -100
"chr1" 37390001 37391000 "*" 8.93895651399301e-06 9.86692435444536e-06
-53.921568627451
"chr1" 37420001 37421000 "*" 0.000285593490543912 0.000248445226337853
52.1739130434783
"chr1" 37426001 37427000 "*" 0 0 100
"chr1" 37454001 37455000 "*" 1.01562092069685e-11 2.59715130444356e-11 -97.5
"chr1" 37455001 37456000 "*" 2.46432020967191e-06 2.95807013879413e-06
-64.1304347826087
"chr1" 37502001 37503000 "*" 2.22044604925031e-16 9.81641919380259e-16
-66.6666666666667
"chr1" 37513001 37514000 "*" 3.54167384530868e-07 4.77718405230532e-07
-50.6666666666667
"chr1" 37541001 37542000 "*" 5.48638023900594e-10 1.10374357336421e-09 100
"chr1" 37594001 37595000 "*" 1.69751706791121e-06 2.08672866949298e-06 70
"chr1" 37599001 37600000 "*" 0 0 -100
"chr1" 37600001 37601000 "*" 9.80763359414993e-10 1.8980032424715e-09 -100
"chr1" 37709001 37710000 "*" 0 0 100
"chr1" 37764001 37765000 "*" 3.63445940010365e-11 8.58565209111204e-11 100
"chr1" 37776001 37777000 "*" 1.52100632089258e-09 2.8746597910115e-09
66.6666666666667
"chr1" 37857001 37858000 "*" 6.90747459231034e-12 1.80140148623876e-11 100
"chr1" 37899001 37900000 "*" 3.14672510270952e-09 5.68111411923401e-09
87.2093023255814

"chr1" 37951001 37952000 "*" 1.48487888651516e-11 3.70689457767456e-11 100
"chr1" 38079001 38080000 "*" 9.47020240005259e-14 3.12491189162604e-13 100
"chr1" 38082001 38083000 "*" 0.000161156913864868 0.000146123644424559
53.2110091743119
"chr1" 38095001 38096000 "*" 6.25199891857164e-12 1.64423898607046e-11
97.6190476190476
"chr1" 38251001 38252000 "*" 2.4535928844216e-14 8.69144396197119e-14 100
"chr1" 38261001 38262000 "*" 0 0 -66.5872156013001
"chr1" 38264001 38265000 "*" 5.6362137179633e-11 1.29963270300065e-10 -100
"chr1" 38400001 38401000 "*" 2.1763814261333e-05 2.26504601940975e-05
73.3333333333333
"chr1" 38412001 38413000 "*" 5.63714436241369e-08 8.49305890498047e-08
75.7142857142857
"chr1" 38497001 38498000 "*" 2.43005615629954e-09 4.45014222280278e-09 100
"chr1" 38508001 38509000 "*" 2.02884444466811e-07 2.82539375333911e-07 -82.5
"chr1" 38530001 38531000 "*" 0 0 100
"chr1" 38583001 38584000 "*" 1.25399267636439e-08 2.07109062327833e-08
82.7814569536424
"chr1" 38587001 38588000 "*" 0 0 100
"chr1" 38592001 38593000 "*" 0 0 100
"chr1" 38595001 38596000 "*" 2.64951482975562e-09 4.82186011375752e-09 100
"chr1" 38650001 38651000 "*" 4.86832796298131e-13 1.47290845675726e-12 100
"chr1" 38704001 38705000 "*" 2.67660071703313e-05 2.74676737224733e-05
65.7894736842105
"chr1" 38715001 38716000 "*" 8.7349629751543e-09 1.47505871898511e-08 -100
"chr1" 38730001 38731000 "*" 0 0 100
"chr1" 38758001 38759000 "*" 2.87384938069124e-09 5.20898531579295e-09 53.125
"chr1" 39027001 39028000 "*" 0 0 -97.4358974358974
"chr1" 39031001 39032000 "*" 3.33066907387547e-16 1.4495649018245e-15
-69.2307692307692
"chr1" 39035001 39036000 "*" 1.7228118931456e-09 3.22812205428052e-09
85.7142857142857
"chr1" 39042001 39043000 "*" 3.4609084784698e-06 4.06200733549184e-06
-71.4285714285714
"chr1" 39089001 39090000 "*" 9.71063229826541e-11 2.16781365050398e-10 100
"chr1" 39210001 39211000 "*" 0 0 100
"chr1" 39253001 39254000 "*" 2.02060590481778e-13 6.40127405465654e-13 100
"chr1" 39269001 39270000 "*" 0 0 69.3678588598816
"chr1" 39270001 39271000 "*" 0 0 81.6672883252518
"chr1" 39332001 39333000 "*" 1.24344978758018e-14 4.56627092294518e-14 100
"chr1" 39366001 39367000 "*" 2.00227675550835e-08 3.20459176617961e-08 100
"chr1" 39538001 39539000 "*" 7.7715611723761e-16 3.26213507634405e-15 100
"chr1" 39567001 39568000 "*" 2.15162852534867e-07 2.98606087005393e-07
54.5454545454545
"chr1" 39699001 39700000 "*" 5.37504528330679e-07 7.07807059196431e-07
52.7777777777778
"chr1" 40040001 40041000 "*" 7.0006535457523e-08 1.03695832512054e-07 100
"chr1" 40062001 40063000 "*" 0 0 100
"chr1" 40090001 40091000 "*" 5.42290105376431e-07 7.13905997413888e-07
65.2173913043478
"chr1" 40137001 40138000 "*" 0 0 52.8082586906116
"chr1" 40150001 40151000 "*" 0 0 -58.3820882088209

"chr1" 40156001 40157000 "*" 3.77475828372553e-15 1.47379557022071e-14 100
"chr1" 40167001 40168000 "*" 0 0 100
"chr1" 40177001 40178000 "*" 5.01025332333427e-10 1.01358031135889e-09 100
"chr1" 40221001 40222000 "*" 1.11022302462516e-16 5.03662826618488e-16 -100
"chr1" 40241001 40242000 "*" 2.94759772145881e-11 7.06210009640777e-11
-90.9090909090909
"chr1" 40253001 40254000 "*" 0 0 54.2336996544497
"chr1" 40368001 40369000 "*" 0 0 -100
"chr1" 40400001 40401000 "*" 2.3990809339125e-12 6.65441738642456e-12 100
"chr1" 40420001 40421000 "*" 0 0 63.493946132938
"chr1" 40915001 40916000 "*" 0 0 85.9737638748739
"chr1" 40974001 40975000 "*" 0 0 61.1098371933043
"chr1" 41244001 41245000 "*" 1.67299207820548e-08 2.71377429441055e-08 100
"chr1" 41258001 41259000 "*" 1.96644922567657e-11 4.82930612417392e-11 -100
"chr1" 41328001 41329000 "*" 0 0 50.846394984326
"chr1" 41397001 41398000 "*" 3.34128893442198e-08 5.19772864618523e-08 -100
"chr1" 41770001 41771000 "*" 9.08108033215171e-11 2.03407753381119e-10 100
"chr1" 41822001 41823000 "*" 1.89581683684992e-12 5.32311367606535e-12 100
"chr1" 41853001 41854000 "*" 3.77475828372553e-15 1.47379557022071e-14 100
"chr1" 41857001 41858000 "*" 0 0 96.8152866242038
"chr1" 41858001 41859000 "*" 4.86832796298131e-13 1.47290845675726e-12 -100
"chr1" 41860001 41861000 "*" 2.01809635669292e-08 3.22829990076203e-08
52.3809523809524
"chr1" 41862001 41863000 "*" 1.44446195671222e-07 2.04747159889886e-07 70
"chr1" 41869001 41870000 "*" 2.69084754478399e-12 7.41487347763769e-12 100
"chr1" 41922001 41923000 "*" 0.000131993845989276 0.000121371320672996 59.375
"chr1" 41933001 41934000 "*" 8.01666433236647e-09 1.36392368970109e-08 100
"chr1" 41997001 41998000 "*" 2.99419040404869e-05 3.04957583173728e-05
52.9411764705882
"chr1" 42003001 42004000 "*" 3.63445940010365e-11 8.58565209111204e-11 100
"chr1" 42009001 42010000 "*" 7.7715611723761e-16 3.26213507634405e-15 100
"chr1" 42098001 42099000 "*" 6.59976938922613e-05 6.36911591975017e-05
-63.3333333333333
"chr1" 42128001 42129000 "*" 0 0 -73.4239802224969
"chr1" 42175001 42176000 "*" 1.12634901405784e-10 2.49563754426169e-10 100
"chr1" 42198001 42199000 "*" 4.26951363152739e-10 8.72510873516957e-10 100
"chr1" 42201001 42202000 "*" 0 0 100
"chr1" 42273001 42274000 "*" 1.67299207820548e-08 2.71377429441055e-08 100
"chr1" 42317001 42318000 "*" 1.39779633423487e-07 1.98463037958589e-07 -100
"chr1" 42332001 42333000 "*" 2.00227675550835e-08 3.20459176617961e-08 -100
"chr1" 42366001 42367000 "*" 2.00227675550835e-08 3.20459176617961e-08 -100
"chr1" 42512001 42513000 "*" 2.91028312560115e-11 6.97590507139978e-11 100
"chr1" 42623001 42624000 "*" 3.00083254745065e-06 3.55645828461632e-06
67.4418604651163
"chr1" 42629001 42630000 "*" 8.20042478366645e-10 1.60952918762439e-09
52.2727272727273
"chr1" 42796001 42797000 "*" 3.51845019785912e-09 6.31708427526519e-09
-66.6666666666667
"chr1" 43312001 43313000 "*" 0 0 65.6558849955076
"chr1" 43390001 43391000 "*" 0 0 -72.9885057471264
"chr1" 43494001 43495000 "*" 0 0 100
"chr1" 43617001 43618000 "*" 1.54319890199872e-11 3.84569468400407e-11 82.5

"chr1" 43618001 43619000 "*" 3.85540880221136e-06 4.49215246381664e-06 -80
"chr1" 43637001 43638000 "*" 3.10862446895044e-15 1.22466437093774e-14 68.75
"chr1" 43723001 43724000 "*" 0 0 -100
"chr1" 43771001 43772000 "*" 4.71134242729931e-12 1.257615036806e-11 100
"chr1" 43965001 43966000 "*" 3.52704532247117e-11 8.35581292290093e-11 100
"chr1" 43991001 43992000 "*" 7.31254606565201e-05 7.00333519169519e-05
-54.2763157894737
"chr1" 43997001 43998000 "*" 0 0 -55.1351351351351
"chr1" 44158001 44159000 "*" 3.95353005888666e-09 7.03928529283733e-09 100
"chr1" 44379001 44380000 "*" 3.94653087987251e-10 8.10398685199552e-10
-81.294964028777
"chr1" 44385001 44386000 "*" 8.19344592173366e-14 2.72594184001957e-13
66.9047619047619
"chr1" 44390001 44391000 "*" 4.10782519111308e-15 1.59859775077462e-14
-62.0689655172414
"chr1" 44398001 44399000 "*" 0 0 100
"chr1" 44400001 44401000 "*" 2.08814465718632e-09 3.86232567960463e-09 -100
"chr1" 44411001 44412000 "*" 3.34128893442198e-08 5.19772864618523e-08 100
"chr1" 44426001 44427000 "*" 2.40596430023388e-06 2.89363471805774e-06
-54.8387096774194
"chr1" 44567001 44568000 "*" 1.5277158427196e-09 2.87642564232045e-09 -100
"chr1" 44637001 44638000 "*" 8.52140580320793e-12 2.19817259951489e-11 -100
"chr1" 44760001 44761000 "*" 7.03992419914812e-12 1.83313429961219e-11 100
"chr1" 44834001 44835000 "*" 0 0 100
"chr1" 44840001 44841000 "*" 2.37283847792646e-07 3.27577954042927e-07 -60
"chr1" 44871001 44872000 "*" 0 0 69.5221712538226
"chr1" 44889001 44890000 "*" 7.7715611723761e-16 3.26213507634405e-15 100
"chr1" 44972001 44973000 "*" 1.56863411149288e-12 4.4469621057462e-12 100
"chr1" 45036001 45037000 "*" 2.48682099757147e-06 2.98236493157237e-06 60
"chr1" 45063001 45064000 "*" 0 0 100
"chr1" 45089001 45090000 "*" 6.24654217240561e-10 1.2462108191303e-09 100
"chr1" 45184001 45185000 "*" 6.59550958292954e-09 1.13456194382773e-08 100
"chr1" 45253001 45254000 "*" 2.34851418090543e-07 3.24402463696593e-07 -81.25
"chr1" 45266001 45267000 "*" 0 0 -57.4074074074074
"chr1" 45280001 45281000 "*" 1.15592780791651e-07 1.66296116792401e-07
58.5365853658537
"chr1" 46499001 46500000 "*" 0 0 -60
"chr1" 46598001 46599000 "*" 0 0 56.0747663551402
"chr1" 46640001 46641000 "*" 2.64951482975562e-09 4.82186011375752e-09 100
"chr1" 46660001 46661000 "*" 1.12458486967171e-09 2.15740815043925e-09 100
"chr1" 46669001 46670000 "*" 0 0 64.4699140401146
"chr1" 46712001 46713000 "*" 1.23065195811733e-08 2.03407184118012e-08
-66.6666666666667
"chr1" 46716001 46717000 "*" 1.52466927971773e-12 4.33322475981408e-12 -100
"chr1" 46860001 46861000 "*" 3.26350058088565e-12 8.89878450947732e-12
-84.2465753424657
"chr1" 46911001 46912000 "*" 1.37828859436695e-10 3.01445544267661e-10 100
"chr1" 46927001 46928000 "*" 1.25284005392245e-10 2.75649590272477e-10 -100
"chr1" 46935001 46936000 "*" 1.02655276501551e-08 1.71893942776041e-08
-69.2307692307692
"chr1" 47015001 47016000 "*" 6.4399653571745e-05 6.22599140150143e-05
-61.5384615384615

"chr1" 47086001 47087000 "*" 2.08995487582797e-10 4.46944006972663e-10 100
"chr1" 47278001 47279000 "*" 4.65637306490407e-10 9.46720524046618e-10 100
"chr1" 47364001 47365000 "*" 0 0 -100
"chr1" 47488001 47489000 "*" 1.39230795903611e-06 1.73395458284971e-06 -75
"chr1" 47489001 47490000 "*" 0 0 91.2162162162162
"chr1" 47614001 47615000 "*" 9.06552610757672e-11 2.03407753381119e-10
-65.3846153846154
"chr1" 47656001 47657000 "*" 0 0 -100
"chr1" 47697001 47698000 "*" 0 0 95.5704697986577
"chr1" 47705001 47706000 "*" 0 0 81.8181818181818
"chr1" 47912001 47913000 "*" 4.14335232790108e-13 1.26153063645578e-12 100
"chr1" 47935001 47936000 "*" 0.000112744095415507 0.000104888464052018 52.5
"chr1" 47973001 47974000 "*" 0 0 77.1788556271315
"chr1" 48024001 48025000 "*" 5.19151388544969e-12 1.37789477890196e-11
53.5714285714286
"chr1" 48046001 48047000 "*" 7.0006535457523e-08 1.03695832512054e-07 100
"chr1" 48060001 48061000 "*" 2.08814465718632e-09 3.86232567960463e-09 -100
"chr1" 48098001 48099000 "*" 6.4324989779152e-11 1.47230020825092e-10 100
"chr1" 48104001 48105000 "*" 0 0 -55.5986826629028
"chr1" 48110001 48111000 "*" 4.085992681091e-05 4.07152765437708e-05
63.4146341463415
"chr1" 48146001 48147000 "*" 8.24416090949853e-11 1.86044084867924e-10
-57.6923076923077
"chr1" 48152001 48153000 "*" 2.15342185383438e-06 2.60916832739477e-06
-76.9230769230769
"chr1" 48160001 48161000 "*" 1.00808250635964e-13 3.31107370956809e-13 100
"chr1" 48243001 48244000 "*" 0.000231175672664174 0.000204278487663226
-54.5454545454545
"chr1" 48248001 48249000 "*" 2.64951482975562e-09 4.82186011375752e-09 -100
"chr1" 48265001 48266000 "*" 1.92625820849557e-07 2.68993984113541e-07
-73.3333333333333
"chr1" 48276001 48277000 "*" 0 0 -100
"chr1" 48295001 48296000 "*" 1.98325800226939e-11 4.85612402473442e-11 100
"chr1" 48347001 48348000 "*" 5.25790597394327e-07 6.93060157606199e-07
-70.4545454545455
"chr1" 48381001 48382000 "*" 2.37220243448633e-11 5.76533792951891e-11
-55.1020408163265
"chr1" 48484001 48485000 "*" 8.58968451922237e-12 2.213554983903e-11 100
"chr1" 48679001 48680000 "*" 8.7349629751543e-09 1.47505871898511e-08 100
"chr1" 49563001 49564000 "*" 0 0 100
"chr1" 49797001 49798000 "*" 0 0 100
"chr1" 50116001 50117000 "*" 3.77475828372553e-15 1.47379557022071e-14 100
"chr1" 50825001 50826000 "*" 1.16698304242213e-07 1.6775923726183e-07
65.5172413793103
"chr1" 50834001 50835000 "*" 2.71375144578201e-11 6.55148978444393e-11
50.2229033156868
"chr1" 50889001 50890000 "*" 0 0 85.1449465893992
"chr1" 51471001 51472000 "*" 1.17461596005342e-13 3.8279067575877e-13 -100
"chr1" 51760001 51761000 "*" 7.43849426498855e-15 2.80357739563059e-14 -100
"chr1" 51796001 51797000 "*" 0 0 -59.0565068493151
"chr1" 51800001 51801000 "*" 7.78377362564697e-13 2.29551678017433e-12 -100
"chr1" 52310001 52311000 "*" 5.62074521748279e-06 6.39676416941385e-06

-53.8461538461538
"chr1" 52415001 52416000 "*" 1.11022302462516e-16 5.03662826618488e-16
88.5057471264368
"chr1" 52456001 52457000 "*" 0 0 72.7134020618557
"chr1" 52521001 52522000 "*" 0 0 -80
"chr1" 53019001 53020000 "*" 1.71984648744683e-12 4.85615992598945e-12
58.479039393301
"chr1" 53168001 53169000 "*" 0 0 -66.0952380952381
"chr1" 53380001 53381000 "*" 1.88245228138761e-06 2.29966065245188e-06
70.2702702702703
"chr1" 53392001 53393000 "*" 6.35880237354058e-12 1.67127363496107e-11
53.0056104728827
"chr1" 53537001 53538000 "*" 0 0 -55.9322033898305
"chr1" 53539001 53540000 "*" 1.67299207820548e-08 2.71377429441055e-08 100
"chr1" 53545001 53546000 "*" 5.48638023900594e-10 1.10374357336421e-09 100
"chr1" 53547001 53548000 "*" 1.72547645351795e-05 1.82499002990625e-05
-56.231884057971
"chr1" 53548001 53549000 "*" 0 0 -93.1034482758621
"chr1" 53555001 53556000 "*" 8.62155280612065e-07 1.10406361876258e-06
-51.0204081632653
"chr1" 53576001 53577000 "*" 4.99871536718821e-07 6.60722927518948e-07
57.8947368421053
"chr1" 53593001 53594000 "*" 5.63660229602192e-13 1.68905058935478e-12 -100
"chr1" 53601001 53602000 "*" 1.03761443881467e-11 2.64965607418752e-11
63.4615384615385
"chr1" 53791001 53792000 "*" 0 0 -65.9375
"chr1" 53830001 53831000 "*" 2.88779000712225e-11 6.92935809466068e-11 100
"chr1" 53833001 53834000 "*" 1.74075842629406e-09 3.25969881918356e-09
-73.8095238095238
"chr1" 53879001 53880000 "*" 7.49830486590497e-08 1.10574283479921e-07
66.6666666666667
"chr1" 53882001 53883000 "*" 3.20659288011171e-09 5.78369084753962e-09 87.5
"chr1" 53910001 53911000 "*" 4.2478473696872e-05 4.22286970764066e-05 -70
"chr1" 53951001 53952000 "*" 1.90181204118289e-13 6.05298617539364e-13 100
"chr1" 53967001 53968000 "*" 0 0 -58.8235294117647
"chr1" 53980001 53981000 "*" 2.17514209399639e-06 2.63341946444793e-06
-67.3076923076923
"chr1" 53995001 53996000 "*" 4.99599139491824e-06 5.72772651203823e-06
68.9189189189189
"chr1" 54009001 54010000 "*" 2.20901934711026e-06 2.67233943761391e-06
67.3469387755102
"chr1" 54013001 54014000 "*" 2.93866819767175e-08 4.61071141260641e-08
73.7704918032787
"chr1" 54017001 54018000 "*" 3.76365605347928e-14 1.3043487970679e-13 100
"chr1" 54023001 54024000 "*" 8.030686559235e-05 7.64432131329181e-05 -68.75
"chr1" 54024001 54025000 "*" 1.51656465163796e-13 4.87803420672651e-13 100
"chr1" 54043001 54044000 "*" 9.32032229172819e-13 2.7181920310254e-12 100
"chr1" 54100001 54101000 "*" 0 0 100
"chr1" 54109001 54110000 "*" 1.0636353062754e-06 1.34530286878699e-06
52.1739130434783
"chr1" 54152001 54153000 "*" 0 0 -75.5555555555556
"chr1" 54175001 54176000 "*" 1.38590361409285e-10 3.02706178892116e-10 -100

"chr1" 54193001 54194000 "*" 0 0 100
"chr1" 54196001 54197000 "*" 0 0 100
"chr1" 54277001 54278000 "*" 1.0043506959434e-07 1.45708678559813e-07
64.8148148148148
"chr1" 54412001 54413000 "*" 1.60933488757564e-11 3.99998960755348e-11
-90.7692307692308
"chr1" 54634001 54635000 "*" 4.86753970463383e-11 1.13145908161915e-10 100
"chr1" 54690001 54691000 "*" 0 0 100
"chr1" 54714001 54715000 "*" 4.20257521005496e-08 6.43492879463453e-08
-69.0909090909091
"chr1" 54753001 54754000 "*" 4.59744464720302e-11 1.07319214021589e-10
-74.0740740740741
"chr1" 54979001 54980000 "*" 0 0 -100
"chr1" 55008001 55009000 "*" 0 0 -73.0608572713836
"chr1" 55012001 55013000 "*" 3.57367491155003e-09 6.41126695849234e-09 -72.3
"chr1" 55024001 55025000 "*" 2.75827648309468e-05 2.82530803747995e-05
-53.8461538461538
"chr1" 55055001 55056000 "*" 3.74791250290496e-06 4.37656320759567e-06
66.6666666666667
"chr1" 55060001 55061000 "*" 8.40438829641244e-14 2.79367015354627e-13
-74.6894409937888
"chr1" 55088001 55089000 "*" 5.50426926260172e-09 9.58851810531086e-09
81.1320754716981
"chr1" 55093001 55094000 "*" 2.8418941815378e-06 3.38038891121791e-06
56.5217391304348
"chr1" 55207001 55208000 "*" 5.08482145278322e-14 1.73432706170696e-13 -100
"chr1" 55376001 55377000 "*" 0 0 -100
"chr1" 55461001 55462000 "*" 6.87805368215777e-12 1.79496361126149e-11
51.5789473684211
"chr1" 55475001 55476000 "*" 7.00449831481098e-06 7.85758536274046e-06 59.375
"chr1" 55530001 55531000 "*" 1.43218770176645e-14 5.21983361092297e-14 100
"chr1" 55681001 55682000 "*" 0 0 -76.4705882352941
"chr1" 55800001 55801000 "*" 1.13174959848195e-07 1.63017406680248e-07
-53.3333333333333
"chr1" 55984001 55985000 "*" 1.07882591748876e-11 2.74553537455803e-11 100
"chr1" 56547001 56548000 "*" 4.01313771103418e-09 7.11882087237587e-09 100
"chr1" 57110001 57111000 "*" 0 0 87.8638110928061
"chr1" 57378001 57379000 "*" 9.14568420995465e-12 2.35193622575612e-11
-68.9922480620155
"chr1" 58514001 58515000 "*" 0 0 100
"chr1" 58714001 58715000 "*" 8.7349629751543e-09 1.47505871898511e-08 100
"chr1" 58789001 58790000 "*" 3.11594339219567e-09 5.62797727400629e-09
-52.7777777777778
"chr1" 58922001 58923000 "*" 0.000499129428774947 0.000416865202314435
-52.3809523809524
"chr1" 59056001 59057000 "*" 8.31628025166342e-06 9.22073343770347e-06
70.3703703703704
"chr1" 59280001 59281000 "*" 0 0 -56.8947181850408
"chr1" 59281001 59282000 "*" 0 0 -79.646017699115
"chr1" 59369001 59370000 "*" 0 0 -61.3496932515337
"chr1" 59986001 59987000 "*" 2.64951482975562e-09 4.82186011375752e-09 100
"chr1" 60281001 60282000 "*" 5.10702591327572e-15 1.96271840288234e-14 100

"chr1" 60352001 60353000 "*" 0 0 100
"chr1" 60392001 60393000 "*" 1.12243547789603e-13 3.67249514162589e-13 59.2
"chr1" 61124001 61125000 "*" 4.78841410966879e-11 1.11468187156588e-10 -100
"chr1" 61283001 61284000 "*" 4.01313771103418e-09 7.11882087237587e-09 -100
"chr1" 61400001 61401000 "*" 3.34128893442198e-08 5.19772864618523e-08 -100
"chr1" 61519001 61520000 "*" 0 0 59.6560571388947
"chr1" 61523001 61524000 "*" 0 0 -100
"chr1" 61877001 61878000 "*" 4.99900121297969e-12 1.32921056339624e-11 100
"chr1" 61977001 61978000 "*" 3.04628685166008e-07 4.14477782922613e-07
92.3076923076923
"chr1" 62526001 62527000 "*" 1.67299207820548e-08 2.71377429441055e-08 -100
"chr1" 62751001 62752000 "*" 7.82074405236699e-12 2.02866230650976e-11
-82.258064516129
"chr1" 62883001 62884000 "*" 4.13002965160558e-14 1.42427783430954e-13 100
"chr1" 63687001 63688000 "*" 3.34128893442198e-08 5.19772864618523e-08 -100
"chr1" 63789001 63790000 "*" 0 0 54.7476204192379
"chr1" 63792001 63793000 "*" 0 0 59.5545977011494
"chr1" 64058001 64059000 "*" 9.65338919911574e-13 2.80678910656113e-12 -100
"chr1" 64060001 64061000 "*" 0 0 -68.8888888888889
"chr1" 64073001 64074000 "*" 1.30273569709516e-12 3.74058614284377e-12
66.6666666666667
"chr1" 64412001 64413000 "*" 8.61492766013328e-10 1.68640058959895e-09
-58.5365853658537
"chr1" 64814001 64815000 "*" 3.85540880221136e-06 4.49215246381664e-06 80
"chr1" 64936001 64937000 "*" 0 0 58.6854460093897
"chr1" 64958001 64959000 "*" 6.52733422867868e-12 1.71146951718423e-11 -100
"chr1" 65468001 65469000 "*" 0 0 -61.6185070519436
"chr1" 65568001 65569000 "*" 2.60361667936415e-06 3.11419458317291e-06 -80
"chr1" 65583001 65584000 "*" 1.14130926931466e-13 3.7276674571693e-13 -100
"chr1" 65603001 65604000 "*" 4.91828799908944e-14 1.68192819018269e-13 100
"chr1" 65613001 65614000 "*" 0 0 63.8895891807705
"chr1" 65871001 65872000 "*" 6.37490060739765e-13 1.89496793587877e-12 -100
"chr1" 65990001 65991000 "*" 1.13140401492018e-08 1.87801380787728e-08 100
"chr1" 66070001 66071000 "*" 9.56318859657301e-08 1.39100874499781e-07
-53.2051282051282
"chr1" 66210001 66211000 "*" 8.01666433236647e-09 1.36392368970109e-08 100
"chr1" 66744001 66745000 "*" 1.12458486967171e-09 2.15740815043925e-09 100
"chr1" 67519001 67520000 "*" 0 0 -63.921568627451
"chr1" 67840001 67841000 "*" 1.48487888651516e-11 3.70689457767456e-11 -100
"chr1" 67859001 67860000 "*" 2.99760216648792e-15 1.18450729613838e-14
52.6315789473684
"chr1" 67944001 67945000 "*" 6.88338275267597e-15 2.60518659957509e-14 -100
"chr1" 68219001 68220000 "*" 1.00800279234647e-09 1.94796059571093e-09
-64.1025641025641
"chr1" 68615001 68616000 "*" 7.0006535457523e-08 1.03695832512054e-07 100
"chr1" 68962001 68963000 "*" 0 0 -80.5687203791469
"chr1" 68963001 68964000 "*" 1.5277158427196e-09 2.87642564232045e-09 -100
"chr1" 69377001 69378000 "*" 1.17794662912729e-13 3.83653245040640e-13 100
"chr1" 70033001 70034000 "*" 0 0 63.7071203103311
"chr1" 70035001 70036000 "*" 0 0 100
"chr1" 70354001 70355000 "*" 6.04438721296674e-11 1.38849255306602e-10 100
"chr1" 70686001 70687000 "*" 1.33226762955019e-15 5.48133041560118e-15

-52.3809523809524
"chr1" 71032001 71033000 "*" 2.88779000712225e-11 6.92935809466068e-11 100
"chr1" 71211001 71212000 "*" 4.99020824662466e-11 1.15790792586157e-10 100
"chr1" 71509001 71510000 "*" 6.59550958292954e-09 1.13456194382773e-08 100
"chr1" 71519001 71520000 "*" 0 0 100
"chr1" 74412001 74413000 "*" 9.65729496371637e-10 1.87255912493232e-09 -100
"chr1" 75178001 75179000 "*" 2.67283972732457e-11 6.45686178157698e-11 100
"chr1" 75513001 75514000 "*" 2.00227675550835e-08 3.20459176617961e-08 -100
"chr1" 75593001 75594000 "*" 1.78688104160996e-05 1.88505925150618e-05 65.625
"chr1" 75597001 75598000 "*" 0 0 -64.3204868154158
"chr1" 75600001 75601000 "*" 0 0 53.811405985319
"chr1" 76043001 76044000 "*" 2.98788522812288e-08 4.68458304490938e-08
66.6666666666667
"chr1" 76540001 76541000 "*" 4.42741399098168e-10 9.03476165716896e-10
69.4570135746606
"chr1" 76757001 76758000 "*" 4.16111589629509e-12 1.11897841594988e-11
-54.1666666666667
"chr1" 77483001 77484000 "*" 2.52449542825772e-07 3.47341761878397e-07
66.6666666666667
"chr1" 77570001 77571000 "*" 2.09983736660924e-05 2.19044051649657e-05
-66.6666666666667
"chr1" 78022001 78023000 "*" 4.14335232790108e-13 1.26153063645578e-12 100
"chr1" 78354001 78355000 "*" 0 0 75.5017081412063
"chr1" 79263001 79264000 "*" 8.01666433236647e-09 1.36392368970109e-08 -100
"chr1" 79511001 79512000 "*" 2.43005615629954e-09 4.45014222280278e-09 100
"chr1" 80517001 80518000 "*" 2.88710388929303e-10 6.03399264731139e-10 100
"chr1" 81500001 81501000 "*" 1.21523471967588e-05 1.31439160931594e-05
-55.1724137931034
"chr1" 81559001 81560000 "*" 7.4718009557273e-14 2.49961254443042e-13
77.2972972972973
"chr1" 81768001 81769000 "*" 0 0 -66.6666666666667
"chr1" 82266001 82267000 "*" 0 0 55.8077879038939
"chr1" 82267001 82268000 "*" 0 0 73.9652702412669
"chr1" 82268001 82269000 "*" 0 0 82.3529411764706
"chr1" 82664001 82665000 "*" 4.91828799908944e-14 1.68192819018269e-13 -100
"chr1" 83729001 83730000 "*" 6.4152538836737e-10 1.27477697728417e-09 100
"chr1" 84021001 84022000 "*" 9.5812247025151e-14 3.15667542866807e-13 -100
"chr1" 84543001 84544000 "*" 0 0 50.478469501501
"chr1" 84544001 84545000 "*" 0 0 61.9047619047619
"chr1" 84591001 84592000 "*" 2.64951482975562e-09 4.82186011375752e-09 -100
"chr1" 84973001 84974000 "*" 2.66453525910038e-15 1.05861327033776e-14 100
"chr1" 85212001 85213000 "*" 1.61204383175573e-13 5.17337152201509e-13 -100
"chr1" 85246001 85247000 "*" 1.11022302462516e-16 5.03662826618488e-16 100
"chr1" 85358001 85359000 "*" 0 0 53.4026104308192
"chr1" 85404001 85405000 "*" 5.07371922253697e-14 1.73287898516782e-13 -100
"chr1" 85462001 85463000 "*" 0 0 58.5903083700441
"chr1" 85774001 85775000 "*" 2.46431841599559e-09 4.51021250080738e-09
-60.4651162790698
"chr1" 85983001 85984000 "*" 6.30384633382164e-13 1.87665599796749e-12 -100
"chr1" 85995001 85996000 "*" 1.52466927971773e-12 4.33322475981408e-12 100
"chr1" 86577001 86578000 "*" 3.34128893442198e-08 5.19772864618523e-08 -100
"chr1" 86690001 86691000 "*" 1.12458486967171e-09 2.15740815043925e-09 -100

"chr1" 86861001 86862000 "*" 0 0 -90.3765690376569
"chr1" 86882001 86883000 "*" 0 0 100
"chr1" 88928001 88929000 "*" 0 0 57.0712013368481
"chr1" 89509001 89510000 "*" 5.17940296240749e-05 5.08225002622215e-05
60.6060606060606
"chr1" 90098001 90099000 "*" 0 0 88.6323268206039
"chr1" 90227001 90228000 "*" 2.79987144580218e-12 7.68634726811898e-12 -100
"chr1" 90309001 90310000 "*" 0 0 -73.9463601532567
"chr1" 91227001 91228000 "*" 2.76722629255488e-08 4.35425544727557e-08
-67.6470588235294
"chr1" 91316001 91317000 "*" 0 0 50.3909574468085
"chr1" 91568001 91569000 "*" 8.01666433236647e-09 1.36392368970109e-08 100
"chr1" 91870001 91871000 "*" 0 0 95.2380952380952
"chr1" 92032001 92033000 "*" 5.6362137179633e-11 1.29963270300065e-10 -100
"chr1" 92058001 92059000 "*" 1.24696919456824e-11 3.14726122523703e-11
-93.3333333333333
"chr1" 92067001 92068000 "*" 0.000380936249181008 0.000324518373061759
-51.8518518518519
"chr1" 93244001 93245000 "*" 1.22124532708767e-15 5.03921984217778e-15 -100
"chr1" 94167001 94168000 "*" 2.59053889450911e-11 6.27057754546996e-11
-63.4146341463415
"chr1" 94206001 94207000 "*" 9.67004254448511e-14 3.18178990782609e-13 100
"chr1" 94262001 94263000 "*" 3.6700841921089e-08 5.65530226072258e-08 100
"chr1" 94529001 94530000 "*" 4.71134242729931e-12 1.257615036806e-11 -100
"chr1" 94562001 94563000 "*" 0 0 -100
"chr1" 95114001 95115000 "*" 0 0 -100
"chr1" 95408001 95409000 "*" 1.25284005392245e-10 2.75649590272477e-10 -100
"chr1" 95699001 95700000 "*" 0 0 -85.2941176470588
"chr1" 96523001 96524000 "*" 3.40125705378114e-10 7.03784073980162e-10 -100
"chr1" 96981001 96982000 "*" 2.68450373042128e-10 5.64465213127486e-10 100
"chr1" 98386001 98387000 "*" 0 0 94.5205479452055
"chr1" 98514001 98515000 "*" 1.45990208810787e-08 2.39250263613825e-08
62.1621621621622
"chr1" 99571001 99572000 "*" 2.75827648309468e-05 2.82530803747995e-05
-53.8461538461538
"chr1" 99612001 99613000 "*" 7.42862682479029e-09 1.27019980054801e-08
-52.7272727272727
"chr1" 99772001 99773000 "*" 1.87405646556726e-13 5.97285292681429e-13 100
"chr1" 100160001 100161000 "*" 8.43347613965761e-12 2.17859001990012e-11 -100
"chr1" 100884001 100885000 "*" 3.95861121660346e-12 1.0684496262032e-11 -100
"chr1" 100997001 100998000 "*" 3.56775498033812e-10 7.35898629809829e-10 100
"chr1" 101748001 101749000 "*" 2.22044604925031e-16 9.81641919380259e-16 -100
"chr1" 102462001 102463000 "*" 0 0 55.2936311000827
"chr1" 104333001 104334000 "*" 7.2471517675865e-10 1.43077717181584e-09
-92.3076923076923
"chr1" 104434001 104435000 "*" 9.65729496371637e-10 1.87255912493232e-09 100
"chr1" 105548001 105549000 "*" 1.13140401492018e-08 1.87801380787728e-08 100
"chr1" 105826001 105827000 "*" 0 0 100
"chr1" 107596001 107597000 "*" 2.4535928844216e-14 8.69144396197119e-14 100
"chr1" 107598001 107599000 "*" 2.82773804372027e-13 8.79129664109438e-13 -100
"chr1" 107683001 107684000 "*" 0 0 75.589929241402
"chr1" 108037001 108038000 "*" 0 0 100

"chr1" 108275001 108276000 "*" 2.08814465718632e-09 3.86232567960463e-09 -100
"chr1" 108498001 108499000 "*" 1.67299207820548e-08 2.71377429441055e-08 -100
"chr1" 109029001 109030000 "*" 9.06273616041453e-07 1.15739767942151e-06 75
"chr1" 109271001 109272000 "*" 4.3620679157641e-07 5.8099159078825e-07
-67.3076923076923
"chr1" 109408001 109409000 "*" 8.11080236287065e-05 7.7152991702048e-05
-55.5555555555556
"chr1" 109710001 109711000 "*" 3.99546075571244e-05 3.98784360862687e-05
-55.1724137931034
"chr1" 109791001 109792000 "*" 2.22044604925031e-16 9.81641919380259e-16
-55.5555555555556
"chr1" 109792001 109793000 "*" 0 0 -62.4725822532403
"chr1" 109965001 109966000 "*" 2.08995487582797e-10 4.46944006972663e-10 100
"chr1" 110008001 110009000 "*" 0 0 92.3913043478261
"chr1" 110009001 110010000 "*" 0 0 73.0045249331486
"chr1" 110010001 110011000 "*" 4.91579557823085e-05 4.83987297040465e-05
58.3333333333333
"chr1" 110021001 110022000 "*" 3.61899135115085e-06 4.23687359293194e-06
67.7966101694915
"chr1" 110027001 110028000 "*" 0 0 -98.1132075471698
"chr1" 110153001 110154000 "*" 3.34128893442198e-08 5.19772864618523e-08 100
"chr1" 110198001 110199000 "*" 2.91012525188705e-09 5.27221623279015e-09
-81.0888252148997
"chr1" 110248001 110249000 "*" 6.24654217240561e-10 1.2462108191303e-09 100
"chr1" 110310001 110311000 "*" 8.88178419700125e-15 3.31875892636354e-14 -60
"chr1" 110316001 110317000 "*" 2.50057945350157e-07 3.44261326945203e-07
-65.5737704918033
"chr1" 110332001 110333000 "*" 4.26951363152739e-10 8.72510873516957e-10 -100
"chr1" 110430001 110431000 "*" 5.48375232667375e-07 7.21484118943642e-07
71.6981132075472
"chr1" 110453001 110454000 "*" 0 0 -84.4594594594595
"chr1" 110454001 110455000 "*" 0 0 -61.4285714285714
"chr1" 110473001 110474000 "*" 9.53681578153009e-14 3.14577235821553e-13
-53.1914893617021
"chr1" 110524001 110525000 "*" 7.0006535457523e-08 1.03695832512054e-07 -100
"chr1" 110650001 110651000 "*" 4.40227836008278e-05 4.36712678996e-05
-62.7906976744186
"chr1" 110692001 110693000 "*" 7.82695993573057e-08 1.15142528036045e-07
64.7058823529412
"chr1" 110706001 110707000 "*" 5.56833418263736e-08 8.39545433133528e-08
79.1666666666667
"chr1" 110709001 110710000 "*" 2.1094237467878e-15 8.47434540879246e-15
59.9320882852292
"chr1" 110750001 110751000 "*" 9.65729496371637e-10 1.87255912493232e-09 -100
"chr1" 110778001 110779000 "*" 7.85417227922025e-08 1.15517757200783e-07
-66.6666666666667
"chr1" 110792001 110793000 "*" 1.39779633423487e-07 1.98463037958589e-07 -100
"chr1" 110799001 110800000 "*" 6.52733422867868e-12 1.71146951718423e-11 100
"chr1" 110807001 110808000 "*" 9.40325595166769e-12 2.41489792195084e-11
78.5714285714286
"chr1" 110889001 110890000 "*" 0 0 100
"chr1" 110902001 110903000 "*" 1.76253456274367e-11 4.36023524948838e-11

52.3809523809524
"chr1" 110987001 110988000 "*" 0 0 100
"chr1" 110997001 110998000 "*" 2.23385091535633e-08 3.55326371809416e-08
55.5126555126555
"chr1" 110998001 110999000 "*" 1.16836595953629e-09 2.23702437154968e-09
62.8571428571429
"chr1" 111026001 111027000 "*" 5.38014632844863e-10 1.08506747658349e-09
71.4285714285714
"chr1" 111027001 111028000 "*" 4.97868413162905e-12 1.32604901021886e-11
-64.4444444444444
"chr1" 111033001 111034000 "*" 0 0 100
"chr1" 111098001 111099000 "*" 0 0 57.4528045633573
"chr1" 111149001 111150000 "*" 0 0 80.4878048780488
"chr1" 111215001 111216000 "*" 1.09035578343963e-08 1.81960205466234e-08
-61.2244897959184
"chr1" 111506001 111507000 "*" 0 0 82
"chr1" 111747001 111748000 "*" 0 0 59.1529780982906
"chr1" 112281001 112282000 "*" 0 0 -54.739336492891
"chr1" 112282001 112283000 "*" 0 0 54.9019607843137
"chr1" 112458001 112459000 "*" 2.98911478072705e-05 3.04496410249122e-05 -60
"chr1" 112474001 112475000 "*" 3.9190872769268e-14 1.35416418933905e-13 100
"chr1" 112532001 112533000 "*" 0 0 52.9739254969722
"chr1" 112703001 112704000 "*" 2.15795581404166e-08 3.43975819311611e-08
-72.7272727272727
"chr1" 112902001 112903000 "*" 3.13902392495846e-05 3.18712470329053e-05 60
"chr1" 112942001 112943000 "*" 8.01666433236647e-09 1.36392368970109e-08 -100
"chr1" 113008001 113009000 "*" 2.22044604925031e-16 9.81641919380259e-16 -100
"chr1" 113044001 113045000 "*" 0 0 59.7579496915045
"chr1" 113241001 113242000 "*" 6.66133814775094e-16 2.81595744474255e-15
56.1997460854846
"chr1" 113243001 113244000 "*" 2.73625566649116e-12 7.52483363579137e-12 -100
"chr1" 113336001 113337000 "*" 1.0769163338864e-14 3.98631098593833e-14 100
"chr1" 113447001 113448000 "*" 2.79440914852103e-11 6.73046092948135e-11 100
"chr1" 114355001 114356000 "*" 0 0 100
"chr1" 114525001 114526000 "*" 2.22044604925031e-15 8.90449334476539e-15
81.2893081761006
"chr1" 114623001 114624000 "*" 0 0 88.2352941176471
"chr1" 114676001 114677000 "*" 0 0 100
"chr1" 114750001 114751000 "*" 0 0 74.7967479674797
"chr1" 114775001 114776000 "*" 7.7715611723761e-16 3.26213507634405e-15 100
"chr1" 114812001 114813000 "*" 2.69018141096922e-12 7.41487347763769e-12 100
"chr1" 115091001 115092000 "*" 0 0 61.038961038961
"chr1" 115615001 115616000 "*" 5.40384160677698e-05 5.28457203825496e-05
-58.0645161290323
"chr1" 115632001 115633000 "*" 0 0 61.4958448753463
"chr1" 115655001 115656000 "*" 0 0 -100
"chr1" 115659001 115660000 "*" 6.87096021301237e-07 8.92284373915929e-07
76.7857142857143
"chr1" 115678001 115679000 "*" 1.32325304725844e-05 1.42328688846738e-05
66.6666666666667
"chr1" 115763001 115764000 "*" 1.11022302462516e-16 5.03662826618488e-16 100
"chr1" 115764001 115765000 "*" 4.01313771103418e-09 7.11882087237587e-09 100

"chr1" 115822001 115823000 "*" 0 0 100
"chr1" 115845001 115846000 "*" 1.74853465040314e-11 4.32752710900609e-11 -100
"chr1" 116009001 116010000 "*" 8.01666433236647e-09 1.36392368970109e-08 100
"chr1" 116020001 116021000 "*" 1.25284005392245e-10 2.75649590272477e-10 -100
"chr1" 116022001 116023000 "*" 0 0 76.6423357664234
"chr1" 116056001 116057000 "*" 5.08482145278322e-14 1.73432706170696e-13 100
"chr1" 116081001 116082000 "*" 0 0 -100
"chr1" 116214001 116215000 "*" 0 0 100
"chr1" 116370001 116371000 "*" 0 0 60.3794753341033
"chr1" 116371001 116372000 "*" 0 0 58.5846292540004
"chr1" 116380001 116381000 "*" 0 0 52.9736498313189
"chr1" 116382001 116383000 "*" 0 0 64.0262146892655
"chr1" 116416001 116417000 "*" 6.08402217494586e-14 2.05454787937634e-13 100
"chr1" 116526001 116527000 "*" 1.67299207820548e-08 2.71377429441055e-08 100
"chr1" 116697001 116698000 "*" 0 0 100
"chr1" 116856001 116857000 "*" 1.67299207820548e-08 2.71377429441055e-08 100
"chr1" 116860001 116861000 "*" 0 0 100
"chr1" 116914001 116915000 "*" 0 0 100
"chr1" 117317001 117318000 "*" 0 0 90.1960784313726
"chr1" 117350001 117351000 "*" 0 0 -100
"chr1" 117422001 117423000 "*" 1.25316423904565e-11 3.16165323392645e-11
66.6666666666667
"chr1" 117436001 117437000 "*" 3.88022947106492e-13 1.1867462770515e-12 -100
"chr1" 117457001 117458000 "*" 1.11022302462516e-16 5.03662826618488e-16 -100
"chr1" 117714001 117715000 "*" 0 0 -100
"chr1" 117780001 117781000 "*" 0 0 -100
"chr1" 118131001 118132000 "*" 4.64618138407147e-08 7.07959889061992e-08
58.7719298245614
"chr1" 118209001 118210000 "*" 7.25646895127774e-07 9.39130690477135e-07 75
"chr1" 118550001 118551000 "*" 6.88338275267597e-15 2.60518659957509e-14 100
"chr1" 118690001 118691000 "*" 2.00227675550835e-08 3.20459176617961e-08 -100
"chr1" 118728001 118729000 "*" 0 0 -100
"chr1" 119427001 119428000 "*" 8.39695660125317e-05 7.9676887269758e-05
-57.1428571428571
"chr1" 119498001 119499000 "*" 1.83952852950142e-12 5.17499701329218e-12 -100
"chr1" 119541001 119542000 "*" 2.70339306496226e-13 8.42355472617862e-13 -100
"chr1" 119870001 119871000 "*" 0 0 62.3829712065006
"chr1" 120167001 120168000 "*" 7.97140131680862e-14 2.65641277678906e-13 100
"chr1" 121357001 121358000 "*" 4.45047012220812e-06 5.13697753414196e-06
71.7391304347826
"chr1" 142537001 142538000 "*" 2.58681964737661e-14 9.14008479605639e-14 100
"chr1" 142617001 142618000 "*" 8.25450818808804e-13 2.42473843346228e-12 100
"chr1" 142635001 142636000 "*" 0 0 -75.9170653907496
"chr1" 142795001 142796000 "*" 4.86405660193867e-09 8.52579203257057e-09
-58.2278481012658
"chr1" 142797001 142798000 "*" 7.68718422250458e-13 2.26879652449338e-12
-95.2380952380952
"chr1" 144015001 144016000 "*" 4.73234496034536e-09 8.30627300264826e-09 -100
"chr1" 144989001 144990000 "*" 4.10928544795075e-05 4.09327063440139e-05
-66.6666666666667
"chr1" 145072001 145073000 "*" 1.99951166734991e-13 6.34481096739424e-13
97.7272727272727

"chr1" 145077001 145078000 "*" 1.37828859436695e-10 3.01445544267661e-10 -100
"chr1" 145382001 145383000 "*" 0 0 68.4941675503711
"chr1" 145534001 145535000 "*" 4.09676593979835e-05 4.08157989632064e-05 -62.5
"chr1" 145549001 145550000 "*" 1.98124849859482e-10 4.25157611354621e-10 -100
"chr1" 145556001 145557000 "*" 8.69011130277819e-07 1.11229659818976e-06
66.6666666666667
"chr1" 146009001 146010000 "*" 4.99159241718061e-07 6.59844248942709e-07
75.5555555555556
"chr1" 146533001 146534000 "*" 0 0 61.1414031261359
"chr1" 146556001 146557000 "*" 0 0 64.0729181444177
"chr1" 146644001 146645000 "*" 2.34257058195908e-14 8.33889706074077e-14
-52.3364485981308
"chr1" 146670001 146671000 "*" 6.68325839114914e-10 1.32503306197502e-09
-94.1176470588235
"chr1" 146707001 146708000 "*" 9.71063229826541e-11 2.16781365050398e-10 100
"chr1" 146829001 146830000 "*" 3.56775498033812e-10 7.35898629809829e-10 100
"chr1" 147070001 147071000 "*" 0 0 66.2037037037037
"chr1" 147216001 147217000 "*" 1.67299207820548e-08 2.71377429441055e-08 100
"chr1" 147736001 147737000 "*" 1.91489268885903e-10 4.11999548210973e-10
-66.6666666666667
"chr1" 148555001 148556000 "*" 0 0 98.8888888888889
"chr1" 149034001 149035000 "*" 2.50244158728208e-10 5.28595528468656e-10
-63.8888888888889
"chr1" 149193001 149194000 "*" 6.24722495956576e-13 1.86219277227303e-12
65.2652652652653
"chr1" 149231001 149232000 "*" 0 0 100
"chr1" 149232001 149233000 "*" 0 0 51.6129032258064
"chr1" 149379001 149380000 "*" 0 0 100
"chr1" 149883001 149884000 "*" 8.25538495341505e-08 1.2109413635802e-07
-63.8888888888889
"chr1" 149888001 149889000 "*" 0.000145992128289696 0.000133276179989164
65.7142857142857
"chr1" 149914001 149915000 "*" 7.67056418382595e-11 1.7359448580318e-10 100
"chr1" 150073001 150074000 "*" 8.7349629751543e-09 1.47505871898511e-08 -100
"chr1" 150134001 150135000 "*" 2.455369241261e-12 6.8003688121118e-12 100
"chr1" 150153001 150154000 "*" 3.76246992682683e-05 3.77018640183686e-05
64.1025641025641
"chr1" 150280001 150281000 "*" 4.75887536044439e-08 7.23914801846659e-08 75
"chr1" 150474001 150475000 "*" 4.29183910632958e-10 8.76858412948451e-10 -90
"chr1" 150510001 150511000 "*" 2.15339213127663e-09 3.97746339329683e-09
-53.4411915767848
"chr1" 150706001 150707000 "*" 6.99967293460446e-07 9.07727331300434e-07
52.1739130434783
"chr1" 150748001 150749000 "*" 1.13140401492018e-08 1.87801380787728e-08 -100
"chr1" 150898001 150899000 "*" 0 0 79.1666666666667
"chr1" 150976001 150977000 "*" 0 0 -100
"chr1" 150980001 150981000 "*" 0 0 100
"chr1" 151020001 151021000 "*" 0 0 54.6210841024308
"chr1" 151021001 151022000 "*" 5.13401909396638e-06 5.87805132464619e-06
-67.2131147540984
"chr1" 151043001 151044000 "*" 2.20073148593514e-09 4.06099108041962e-09 -65
"chr1" 151044001 151045000 "*" 6.13930017934194e-11 1.40959672355275e-10

51.1979823455233
"chr1" 151110001 151111000 "*" 6.4152538836737e-10 1.27477697728417e-09 -100
"chr1" 151138001 151139000 "*" 0 0 100
"chr1" 151139001 151140000 "*" 1.32849287126646e-12 3.81060776526677e-12
80.1526717557252
"chr1" 151237001 151238000 "*" 1.12634901405784e-10 2.49563754426169e-10 -100
"chr1" 151300001 151301000 "*" 2.38285849374353e-06 2.86729273060702e-06
62.8378378378378
"chr1" 151400001 151401000 "*" 2.81044296890354e-06 3.34565012917882e-06
-84.6153846153846
"chr1" 151408001 151409000 "*" 4.86753970463383e-11 1.13145908161915e-10 -100
"chr1" 151585001 151586000 "*" 1.14352971536391e-14 4.21921674335999e-14 100
"chr1" 151598001 151599000 "*" 8.57092175010621e-13 2.51149198099779e-12 -100
"chr1" 151686001 151687000 "*" 1.11022302462516e-16 5.03662826618488e-16 -100
"chr1" 151801001 151802000 "*" 1.32075461678482e-11 3.31902662305048e-11
-75.6756756756757
"chr1" 151820001 151821000 "*" 4.40306124893652e-10 8.98657408808873e-10
66.6666666666667
"chr1" 151833001 151834000 "*" 5.68123437183488e-10 1.14082514207793e-09
-56.5217391304348
"chr1" 151908001 151909000 "*" 5.01025332333427e-10 1.01358031135889e-09 100
"chr1" 151967001 151968000 "*" 1.20591092667155e-10 2.66075324129686e-10 -100
"chr1" 152008001 152009000 "*" 8.69234906275551e-10 1.70083750589517e-09
-54.8387096774194
"chr1" 152080001 152081000 "*" 0 0 84.743533914103
"chr1" 152081001 152082000 "*" 0 0 94.9193548387097
"chr1" 152083001 152084000 "*" 0 0 79.9546142208775
"chr1" 152084001 152085000 "*" 0 0 69.0907937543612
"chr1" 152085001 152086000 "*" 0 0 54.6994540232364
"chr1" 152104001 152105000 "*" 6.92287338566189e-11 1.57605630124247e-10 100
"chr1" 152281001 152282000 "*" 5.72875080706581e-14 1.93967931988877e-13
57.6086956521739
"chr1" 152648001 152649000 "*" 2.08995487582797e-10 4.46944006972663e-10 -100
"chr1" 152671001 152672000 "*" 2.88710388929303e-10 6.03399264731139e-10 100
"chr1" 152718001 152719000 "*" 6.66133814775094e-16 2.81595744474255e-15 100
"chr1" 152816001 152817000 "*" 7.67056418382595e-11 1.7359448580318e-10 -100
"chr1" 152853001 152854000 "*" 2.88657986402541e-15 1.14155613124573e-14 100
"chr1" 152934001 152935000 "*" 9.65729496371637e-10 1.87255912493232e-09 100
"chr1" 153183001 153184000 "*" 1.13632643961026e-07 1.63651212309773e-07
-69.6969696969697
"chr1" 153234001 153235000 "*" 0 0 59.0965073640813
"chr1" 153270001 153271000 "*" 2.22190110248377e-05 2.30887626988729e-05
-52.3809523809524
"chr1" 153367001 153368000 "*" 0 0 100
"chr1" 153462001 153463000 "*" 2.4535928844216e-14 8.69144396197119e-14 -100
"chr1" 153508001 153509000 "*" 0 0 -66.3569840017218
"chr1" 153540001 153541000 "*" 0 0 -66.6666666666667
"chr1" 153541001 153542000 "*" 0 0 -82.051282051282
"chr1" 153581001 153582000 "*" 2.69673172681451e-13 8.40813882973658e-13 -70
"chr1" 153590001 153591000 "*" 0 0 100
"chr1" 153600001 153601000 "*" 3.34128893442198e-08 5.19772864618523e-08 -100
"chr1" 153602001 153603000 "*" 1.54630752646767e-11 3.85079502648228e-11 -100

"chr1" 153651001 153652000 "*" 0 0 56.7923586639464
"chr1" 153653001 153654000 "*" 9.80763359414993e-10 1.8980032424715e-09 100
"chr1" 153723001 153724000 "*" 1.87405646556726e-13 5.97285292681429e-13 -100
"chr1" 153747001 153748000 "*" 0 0 -77.0240700218818
"chr1" 154055001 154056000 "*" 9.80763359414993e-10 1.8980032424715e-09 100
"chr1" 154438001 154439000 "*" 0 0 100
"chr1" 154442001 154443000 "*" 9.0072393987839e-13 2.63157725646847e-12 100
"chr1" 154465001 154466000 "*" 0 0 100
"chr1" 154505001 154506000 "*" 3.64270735797589e-09 6.5252880340617e-09
-66.6666666666667
"chr1" 154523001 154524000 "*" 5.52403520037714e-07 7.26531864310528e-07
60.5769230769231
"chr1" 154547001 154548000 "*" 4.01313771103418e-09 7.11882087237587e-09 100
"chr1" 154673001 154674000 "*" 5.01025332333427e-10 1.01358031135889e-09 100
"chr1" 154699001 154700000 "*" 1.35447209004269e-14 4.94674708073053e-14 -100
"chr1" 154707001 154708000 "*" 4.55191440096314e-15 1.76090186737198e-14
99.0566037735849
"chr1" 154716001 154717000 "*" 3.95353005888666e-09 7.03928529283733e-09 100
"chr1" 154723001 154724000 "*" 7.63740298781457e-08 1.12492226166431e-07
-63.8095238095238
"chr1" 154745001 154746000 "*" 1.74853465040314e-11 4.32752710900609e-11 100
"chr1" 154761001 154762000 "*" 1.70641278884887e-12 4.81986032810646e-12 -100
"chr1" 154769001 154770000 "*" 1.68531855138099e-13 5.40042040193392e-13
98.3333333333333
"chr1" 154776001 154777000 "*" 1.18468841181496e-07 1.70134378960104e-07
57.1428571428571
"chr1" 154804001 154805000 "*" 0 0 100
"chr1" 154820001 154821000 "*" 2.0590640303908e-11 5.03274647558249e-11
78.8571428571428
"chr1" 154821001 154822000 "*" 1.49902841251048e-08 2.45247885086907e-08
-56.1643835616438
"chr1" 154833001 154834000 "*" 2.33028818463765e-10 4.93719283454501e-10 100
"chr1" 154835001 154836000 "*" 4.14557277395033e-13 1.26216062018055e-12
61.3997113997114
"chr1" 154836001 154837000 "*" 6.66133814775094e-16 2.81595744474255e-15 100
"chr1" 154837001 154838000 "*" 1.45439216225896e-14 5.29728884617264e-14
74.5762711864407
"chr1" 154839001 154840000 "*" 0 0 66.6666666666667
"chr1" 154850001 154851000 "*" 7.84316410568087e-05 7.47772283140318e-05
-58.6206896551724
"chr1" 154851001 154852000 "*" 0 0 73.6842105263158
"chr1" 154853001 154854000 "*" 1.67299207820548e-08 2.71377429441055e-08 100
"chr1" 154854001 154855000 "*" 7.0006535457523e-08 1.03695832512054e-07 100
"chr1" 154856001 154857000 "*" 3.65831983661913e-05 3.6733698352541e-05 -65.625
"chr1" 154880001 154881000 "*" 1.55431223447522e-14 5.6388152448458e-14 -100
"chr1" 154971001 154972000 "*" 0 0 -60.3174603174603
"chr1" 154972001 154973000 "*" 0 0 66.2090662090662
"chr1" 155024001 155025000 "*" 0 0 -69.1558441558442
"chr1" 155042001 155043000 "*" 0 0 83.0065359477124
"chr1" 155051001 155052000 "*" 0 0 71.4285714285714
"chr1" 155060001 155061000 "*" 2.08995487582797e-10 4.46944006972663e-10 -100
"chr1" 155066001 155067000 "*" 4.79616346638068e-14 1.64380445416596e-13 -100

"chr1" 155080001 155081000 "*" 2.33028818463765e-10 4.93719283454501e-10 -100
"chr1" 155084001 155085000 "*" 0.000237820680127232 0.000209721495917721
64.2857142857143
"chr1" 155101001 155102000 "*" 0 0 -100
"chr1" 155197001 155198000 "*" 2.02327044007689e-12 5.65300384792621e-12 -100
"chr1" 155225001 155226000 "*" 0 0 51.5021459227468
"chr1" 155295001 155296000 "*" 4.17443857259059e-13 1.27057803149973e-12
-57.4285714285714
"chr1" 155416001 155417000 "*" 3.90831811358794e-12 1.05610567215048e-11 -100
"chr1" 155534001 155535000 "*" 0 0 -100
"chr1" 155607001 155608000 "*" 4.38208892061809e-07 5.83499808670586e-07
51.4285714285714
"chr1" 155668001 155669000 "*" 1.05373635028716e-06 1.33369268880708e-06
74.8376623376623
"chr1" 155693001 155694000 "*" 5.48638023900594e-10 1.10374357336421e-09 100
"chr1" 155806001 155807000 "*" 2.00721646947954e-06 2.44138389831627e-06
-63.859649122807
"chr1" 155826001 155827000 "*" 0 0 -83.0188679245283
"chr1" 155881001 155882000 "*" 0 0 98.5401459854015
"chr1" 155934001 155935000 "*" 1.48171475089498e-10 3.22240587590086e-10
-86.4077669902913
"chr1" 155945001 155946000 "*" 7.0006535457523e-08 1.03695832512054e-07 100
"chr1" 155996001 155997000 "*" 3.63445940010365e-11 8.58565209111204e-11 100
"chr1" 156036001 156037000 "*" 3.33066907387547e-16 1.4495649018245e-15 -100
"chr1" 156045001 156046000 "*" 7.67056418382595e-11 1.7359448580318e-10 -100
"chr1" 156048001 156049000 "*" 1.30340183090993e-13 4.22662705118657e-13 -100
"chr1" 156060001 156061000 "*" 0 0 -76.5714285714286
"chr1" 156093001 156094000 "*" 0 0 -100
"chr1" 156095001 156096000 "*" 2.95423767674441e-05 3.0118633488738e-05
76.9230769230769
"chr1" 156110001 156111000 "*" 8.71525074330748e-14 2.88848349331391e-13 -100
"chr1" 156128001 156129000 "*" 2.5309083400149e-06 3.03222903732048e-06
-69.4444444444444
"chr1" 156132001 156133000 "*" 6.77335965093562e-12 1.76897011246462e-11 100
"chr1" 156265001 156266000 "*" 1.11022302462516e-16 5.03662826618488e-16 100
"chr1" 156326001 156327000 "*" 2.98916447150077e-12 8.18257131585715e-12
70.3883495145631
"chr1" 156337001 156338000 "*" 1.16155443002919e-05 1.26002437563056e-05
66.6666666666667
"chr1" 156352001 156353000 "*" 2.00227675550835e-08 3.20459176617961e-08 -100
"chr1" 156377001 156378000 "*" 0.000183148280014089 0.000164546600973141 -52
"chr1" 156390001 156391000 "*" 0 0 57.4914807930607
"chr1" 156391001 156392000 "*" 0 0 58.7020844862571
"chr1" 156392001 156393000 "*" 8.86200726135655e-08 1.29452763525729e-07
79.6296296296296
"chr1" 156393001 156394000 "*" 6.15840711759574e-13 1.83676803572802e-12 100
"chr1" 156405001 156406000 "*" 0 0 51.8950201339566
"chr1" 156554001 156555000 "*" 0 0 77.9661016949153
"chr1" 156555001 156556000 "*" 0 0 -100
"chr1" 156571001 156572000 "*" 0 0 -60.9375
"chr1" 156674001 156675000 "*" 0 0 -58.7786259541985
"chr1" 156683001 156684000 "*" 4.34268065951926e-10 8.8691985924363e-10

-65.7142857142857
"chr1" 156693001 156694000 "*" 1.31583632878574e-12 3.77625225968747e-12
-64.2857142857143
"chr1" 156781001 156782000 "*" 3.6117718604789e-07 4.86418348151014e-07
-66.6666666666667
"chr1" 156798001 156799000 "*" 1.48487888651516e-11 3.70689457767456e-11 -100
"chr1" 156802001 156803000 "*" 1.67299207820548e-08 2.71377429441055e-08 100
"chr1" 156808001 156809000 "*" 3.03090885722668e-14 1.06210046699299e-13 100
"chr1" 156816001 156817000 "*" 5.75027003790751e-09 9.99596742033581e-09
81.3333333333333
"chr1" 156830001 156831000 "*" 0 0 63.1772268135905
"chr1" 156837001 156838000 "*" 2.40249107275048e-08 3.80684405385995e-08
58.0645161290323
"chr1" 156848001 156849000 "*" 1.20625731625523e-12 3.47505011746053e-12
-88.5714285714286
"chr1" 156853001 156854000 "*" 6.59550958292954e-09 1.13456194382773e-08 100
"chr1" 156858001 156859000 "*" 8.54034272190773e-08 1.25047521194141e-07
64.367816091954
"chr1" 156871001 156872000 "*" 2.09228207426193e-06 2.53903592891552e-06
63.0434782608696
"chr1" 156877001 156878000 "*" 0 0 -53.7238698121273
"chr1" 156879001 156880000 "*" 3.84137166520304e-14 1.32929369817675e-13 -100
"chr1" 156953001 156954000 "*" 2.64951482975562e-09 4.82186011375752e-09 -100
"chr1" 157051001 157052000 "*" 1.14130926931466e-13 3.7276674571693e-13 100
"chr1" 157059001 157060000 "*" 0 0 -100
"chr1" 157108001 157109000 "*" 0 0 60.6666666666667
"chr1" 157130001 157131000 "*" 0 0 -100
"chr1" 157133001 157134000 "*" 2.89435142519778e-13 8.99169061076996e-13
54.5454545454545
"chr1" 157144001 157145000 "*" 1.48769885299771e-14 5.41245512218755e-14
66.6666666666667
"chr1" 157169001 157170000 "*" 3.34128893442198e-08 5.19772864618523e-08 100
"chr1" 157387001 157388000 "*" 2.08995487582797e-10 4.46944006972663e-10 -100
"chr1" 157415001 157416000 "*" 6.70841160399505e-12 1.75486545176836e-11 -100
"chr1" 157543001 157544000 "*" 7.54951656745106e-14 2.5237892556906e-13 -100
"chr1" 157771001 157772000 "*" 4.26951363152739e-10 8.72510873516957e-10 100
"chr1" 157971001 157972000 "*" 3.73034936274053e-14 1.29420415488591e-13 -100
"chr1" 158037001 158038000 "*" 5.63660229602192e-13 1.68905058935478e-12 -100
"chr1" 158042001 158043000 "*" 1.37263167498958e-09 2.60812517660705e-09 -65
"chr1" 158848001 158849000 "*" 1.12458486967171e-09 2.15740815043925e-09 100
"chr1" 159170001 159171000 "*" 4.18212340480562e-06 4.84642008050785e-06
66.2162162162162
"chr1" 159174001 159175000 "*" 4.44089209850063e-15 1.71914916534614e-14 100
"chr1" 159176001 159177000 "*" 1.52999835023593e-11 3.81439418802985e-11
-73.9130434782609
"chr1" 159750001 159751000 "*" 0 0 94.1588074231822
"chr1" 159795001 159796000 "*" 4.83868500822382e-12 1.290273879976e-11
51.7241379310345
"chr1" 159831001 159832000 "*" 0 0 100
"chr1" 159837001 159838000 "*" 5.37347943918576e-14 1.82732693325353e-13
72.972972972973
"chr1" 159842001 159843000 "*" 9.43689570931383e-15 3.51625644188498e-14

69.6969696969697
"chr1" 159884001 159885000 "*" 0.000150576069463337 0.000137139618444501
53.3333333333333
"chr1" 159887001 159888000 "*" 3.13915560212763e-12 8.57261837307703e-12
-97.4358974358974
"chr1" 159915001 159916000 "*" 0 0 76.5734265734266
"chr1" 159923001 159924000 "*" 4.01313771103418e-09 7.11882087237587e-09 -100
"chr1" 160031001 160032000 "*" 8.57092175010621e-13 2.51149198099779e-12 100
"chr1" 160040001 160041000 "*" 0 0 77.3962106436333
"chr1" 160135001 160136000 "*" 1.29037891483108e-11 3.24582280286866e-11 100
"chr1" 160146001 160147000 "*" 3.18355716233398e-07 4.31991940783722e-07
58.5365853658537
"chr1" 160154001 160155000 "*" 2.88710388929303e-10 6.03399264731139e-10 100
"chr1" 160155001 160156000 "*" 5.01025332333427e-10 1.01358031135889e-09 100
"chr1" 160163001 160164000 "*" 8.37826685806675e-09 1.42187770420251e-08
69.6969696969697
"chr1" 160233001 160234000 "*" 5.2003549414481e-06 5.95024319668787e-06
-66.6666666666667
"chr1" 160333001 160334000 "*" 5.44009282066327e-15 2.08486137681812e-14 100
"chr1" 160390001 160391000 "*" 0 0 66.6666666666667
"chr1" 160599001 160600000 "*" 1.2445600106048e-13 4.04289056263354e-13
65.5172413793103
"chr1" 160639001 160640000 "*" 0 0 70.0930438635357
"chr1" 160650001 160651000 "*" 1.13140401492018e-08 1.87801380787728e-08 -100
"chr1" 160788001 160789000 "*" 2.22044604925031e-16 9.81641919380259e-16 -100
"chr1" 160791001 160792000 "*" 2.17612240449583e-08 3.46699253507917e-08
-51.5151515151515
"chr1" 160851001 160852000 "*" 5.10702591327572e-15 1.96271840288234e-14 -100
"chr1" 160875001 160876000 "*" 1.46776835130424e-09 2.77939521578541e-09
73.5294117647059
"chr1" 160921001 160922000 "*" 8.7349629751543e-09 1.47505871898511e-08 100
"chr1" 161008001 161009000 "*" 0 0 -82.2503961965135
"chr1" 161037001 161038000 "*" 8.3882012447134e-11 1.88922989503423e-10 -100
"chr1" 161044001 161045000 "*" 7.18777826147488e-09 1.23131501832318e-08
-60.655737704918
"chr1" 161048001 161049000 "*" 6.77335965093562e-12 1.76897011246462e-11 -100
"chr1" 161053001 161054000 "*" 1.14130926931466e-13 3.7276674571693e-13 -100
"chr1" 161146001 161147000 "*" 8.52140580320793e-12 2.19817259951489e-11 -100
"chr1" 161229001 161230000 "*" 0 0 -90.3225806451613
"chr1" 161283001 161284000 "*" 0 0 100
"chr1" 161328001 161329000 "*" 3.33066907387547e-16 1.4495649018245e-15
-94.4444444444444
"chr1" 161448001 161449000 "*" 0 0 100
"chr1" 161696001 161697000 "*" 0 0 76.1485826001955
"chr1" 161697001 161698000 "*" 0 0 69.7869101978691
"chr1" 161979001 161980000 "*" 1.571479613105e-09 2.95505264715517e-09
82.5396825396825
"chr1" 161980001 161981000 "*" 0 0 -87.5
"chr1" 162135001 162136000 "*" 3.34128893442198e-08 5.19772864618523e-08 -100
"chr1" 162348001 162349000 "*" 1.39779633423487e-07 1.98463037958589e-07 -100
"chr1" 162357001 162358000 "*" 1.05541186901092e-09 2.0350986189217e-09
86.2068965517241

"chr1" 162809001 162810000 "*" 0 0 -100
"chr1" 162976001 162977000 "*" 6.24654217240561e-10 1.2462108191303e-09 100
"chr1" 163393001 163394000 "*" 0 0 -84.6153846153846
"chr1" 164311001 164312000 "*" 1.17461596005342e-13 3.8279067575877e-13 100
"chr1" 164445001 164446000 "*" 7.67056418382595e-11 1.7359448580318e-10 100
"chr1" 164756001 164757000 "*" 1.11022302462516e-16 5.03662826618488e-16 -100
"chr1" 164760001 164761000 "*" 3.34128893442198e-08 5.19772864618523e-08 -100
"chr1" 164768001 164769000 "*" 1.99840144432528e-15 8.0570469535907e-15 100
"chr1" 164859001 164860000 "*" 1.8011014901731e-08 2.90986632818557e-08
-66.6666666666667
"chr1" 165087001 165088000 "*" 0 0 -67.515923566879
"chr1" 165338001 165339000 "*" 7.94730947717426e-12 2.05982705398163e-11
-97.5609756097561
"chr1" 165372001 165373000 "*" 2.00227675550835e-08 3.20459176617961e-08 -100
"chr1" 165553001 165554000 "*" 1.5277158427196e-09 2.87642564232045e-09 -100
"chr1" 165615001 165616000 "*" 9.47020240005259e-14 3.12491189162604e-13 -100
"chr1" 165707001 165708000 "*" 1.99840144432528e-12 5.59476580335607e-12 -100
"chr1" 165802001 165803000 "*" 5.23665053642208e-06 5.98988252471217e-06
54.5454545454545
"chr1" 166002001 166003000 "*" 4.36618494770169e-06 5.04443830498714e-06
62.962962962963
"chr1" 166125001 166126000 "*" 0 0 81.25
"chr1" 166134001 166135000 "*" 0 0 63.9682074296536
"chr1" 166857001 166858000 "*" 2.08814465718632e-09 3.86232567960463e-09 100
"chr1" 166870001 166871000 "*" 1.11022302462516e-16 5.03662826618488e-16 100
"chr1" 167013001 167014000 "*" 0 0 83.9416058394161
"chr1" 167086001 167087000 "*" 7.50955468031833e-07 9.69898247391516e-07
-63.6363636363636
"chr1" 167131001 167132000 "*" 2.55398147075425e-10 5.39045513624183e-10
57.5581395348837
"chr1" 167189001 167190000 "*" 0 0 66.1971830985916
"chr1" 167423001 167424000 "*" 5.1281201507436e-13 1.54461625452838e-12 -100
"chr1" 167425001 167426000 "*" 0 0 57.2687224669604
"chr1" 167436001 167437000 "*" 6.93722763345228e-07 9.00028246447643e-07
-73.8095238095238
"chr1" 167463001 167464000 "*" 2.69007038866675e-13 8.38831022628993e-13 -100
"chr1" 167624001 167625000 "*" 3.6427075800205e-09 6.5252880340617e-09
-66.6666666666667
"chr1" 167653001 167654000 "*" 1.79480541540045e-10 3.87261073058711e-10
60.6151537884471
"chr1" 167671001 167672000 "*" 5.01025332333427e-10 1.01358031135889e-09 100
"chr1" 167810001 167811000 "*" 1.13140401492018e-08 1.87801380787728e-08 -100
"chr1" 167867001 167868000 "*" 3.66373598126302e-15 1.43441054525534e-14
-66.6666666666667
"chr1" 168286001 168287000 "*" 4.01313771103418e-09 7.11882087237587e-09 -100
"chr1" 168593001 168594000 "*" 1.57873714101697e-13 5.07058104104294e-13
-80.9523809523809
"chr1" 169653001 169654000 "*" 9.99200722162641e-16 4.15382497462808e-15 100
"chr1" 169878001 169879000 "*" 3.73034936274053e-14 1.29420415488591e-13
87.2727272727273
"chr1" 169888001 169889000 "*" 1.14130926931466e-13 3.7276674571693e-13 100
"chr1" 170136001 170137000 "*" 1.35036426485158e-12 3.86629547363774e-12 100

"chr1" 170401001 170402000 "*" 3.40125705378114e-10 7.03784073980162e-10 100
"chr1" 170600001 170601000 "*" 2.66453525910038e-15 1.05861327033776e-14
95.2380952380952
"chr1" 170619001 170620000 "*" 1.11022302462516e-16 5.03662826618488e-16 100
"chr1" 171810001 171811000 "*" 0 0 69.9890791700169
"chr1" 172101001 172102000 "*" 4.86753970463383e-11 1.13145908161915e-10 -100
"chr1" 172809001 172810000 "*" 1.86517468137026e-14 6.70995420767425e-14 -100
"chr1" 172941001 172942000 "*" 1.16573417585641e-14 4.29335582900508e-14 100
"chr1" 173885001 173886000 "*" 0 0 100
"chr1" 174074001 174075000 "*" 3.10862446895044e-15 1.22466437093774e-14 100
"chr1" 175086001 175087000 "*" 0 0 50.7177033492823
"chr1" 175087001 175088000 "*" 1.60982338570648e-14 5.83411082284088e-14 75
"chr1" 175191001 175192000 "*" 1.72791398966865e-08 2.79807450133109e-08
-85.7142857142857
"chr1" 175194001 175195000 "*" 3.40125705378114e-10 7.03784073980162e-10 -100
"chr1" 175311001 175312000 "*" 1.52794177310511e-09 2.87642564232045e-09 -100
"chr1" 175425001 175426000 "*" 2.87128099074607e-11 6.9082285894318e-11
97.2222222222222
"chr1" 175728001 175729000 "*" 6.4324989779152e-11 1.47230020825092e-10 100
"chr1" 176426001 176427000 "*" 3.56131733214582e-07 4.80158368901456e-07
66.6666666666667
"chr1" 176584001 176585000 "*" 4.9960036108132e-15 1.9234186548721e-14
95.2380952380952
"chr1" 176661001 176662000 "*" 1.13140401492018e-08 1.87801380787728e-08 100
"chr1" 176737001 176738000 "*" 3.33066907387547e-16 1.4495649018245e-15 100
"chr1" 176863001 176864000 "*" 2.22044604925031e-16 9.81641919380259e-16 100
"chr1" 177226001 177227000 "*" 1.88737914186277e-15 7.62011598380321e-15 100
"chr1" 177680001 177681000 "*" 2.93692584918404e-06 3.48641648244637e-06
65.7894736842105
"chr1" 177839001 177840000 "*" 1.51323398256409e-13 4.86995056237989e-13 100
"chr1" 177841001 177842000 "*" 4.26951363152739e-10 8.72510873516957e-10 -100
"chr1" 177877001 177878000 "*" 1.66533453693773e-15 6.7629186866784e-15 100
"chr1" 177912001 177913000 "*" 0 0 -100
"chr1" 177921001 177922000 "*" 0 0 -100
"chr1" 178087001 178088000 "*" 4.79616346638068e-14 1.64380445416596e-13 -100
"chr1" 178491001 178492000 "*" 7.0006535457523e-08 1.03695832512054e-07 -100
"chr1" 178501001 178502000 "*" 1.87405646556726e-13 5.97285292681429e-13 100
"chr1" 178928001 178929000 "*" 0 0 72.9186602870814
"chr1" 178971001 178972000 "*" 9.14935904816616e-10 1.7831816022306e-09
61.1111111111111
"chr1" 179062001 179063000 "*" 1.17905685215192e-13 3.83653245040640e-13 100
"chr1" 179262001 179263000 "*" 0 0 -94.5812807881773
"chr1" 179454001 179455000 "*" 1.63883566162148e-07 2.30726441023694e-07
-79.4520547945205
"chr1" 179541001 179542000 "*" 0 0 100
"chr1" 179544001 179545000 "*" 0 0 53.9151247664422
"chr1" 179551001 179552000 "*" 1.91305996340985e-05 2.00697544961136e-05
58.3333333333333
"chr1" 179714001 179715000 "*" 1.0902390101819e-13 3.57149508278892e-13
51.7241379310345
"chr1" 179734001 179735000 "*" 1.38590361409285e-10 3.02706178892116e-10 100
"chr1" 179740001 179741000 "*" 4.55191440096314e-14 1.56510897751783e-13

86.9565217391304
"chr1" 179758001 179759000 "*" 0 0 -100
"chr1" 179779001 179780000 "*" 0 0 -100
"chr1" 179795001 179796000 "*" 4.44089209850063e-16 1.91071758245033e-15 100
"chr1" 180123001 180124000 "*" 0 0 100
"chr1" 180166001 180167000 "*" 4.32209823486573e-12 1.15935290479641e-11 -100
"chr1" 180180001 180181000 "*" 1.65305520560821e-06 2.03582333545644e-06
72.0930232558139
"chr1" 180188001 180189000 "*" 0 0 81.9672131147541
"chr1" 180189001 180190000 "*" 7.105427357601e-15 2.68362283258525e-14 -100
"chr1" 180224001 180225000 "*" 0 0 100
"chr1" 180234001 180235000 "*" 0 0 66.6666666666667
"chr1" 180470001 180471000 "*" 0 0 97.0588235294118
"chr1" 180586001 180587000 "*" 0 0 -100
"chr1" 180663001 180664000 "*" 9.65729496371637e-10 1.87255912493232e-09 -100
"chr1" 180889001 180890000 "*" 1.33959510151271e-12 3.84139613269674e-12
-80.5555555555556
"chr1" 180896001 180897000 "*" 1.11022302462516e-16 5.03662826618488e-16
-52.7777777777778
"chr1" 180938001 180939000 "*" 3.33066907387547e-16 1.4495649018245e-15 -100
"chr1" 180951001 180952000 "*" 9.2148511043888e-15 3.43705279328267e-14 -100
"chr1" 181069001 181070000 "*" 2.43005615629954e-09 4.45014222280278e-09 100
"chr1" 181149001 181150000 "*" 0 0 100
"chr1" 181151001 181152000 "*" 7.7715611723761e-16 3.26213507634405e-15 100
"chr1" 181158001 181159000 "*" 0 0 100
"chr1" 181173001 181174000 "*" 1.66533453693773e-15 6.7629186866784e-15 100
"chr1" 181176001 181177000 "*" 5.12782259987077e-08 7.7634284063037e-08
52.3809523809524
"chr1" 181276001 181277000 "*" 4.98184294817072e-07 6.58670218882383e-07
-69.2307692307692
"chr1" 181288001 181289000 "*" 0 0 100
"chr1" 181356001 181357000 "*" 2.31857695709436e-07 3.2044765709855e-07 -80
"chr1" 181398001 181399000 "*" 2.22044604925031e-16 9.81641919380259e-16 -100
"chr1" 181577001 181578000 "*" 2.68450373042128e-10 5.64465213127486e-10 -100
"chr1" 181616001 181617000 "*" 0 0 -100
"chr1" 181706001 181707000 "*" 4.23772128499422e-13 1.28791235599387e-12
52.8998778998779
"chr1" 181819001 181820000 "*" 1.67299207820548e-08 2.71377429441055e-08 -100
"chr1" 182030001 182031000 "*" 3.6700841921089e-08 5.65530226072258e-08 100
"chr1" 182268001 182269000 "*" 1.37828859436695e-10 3.01445544267661e-10 -100
"chr1" 182639001 182640000 "*" 0 0 100
"chr1" 183221001 183222000 "*" 3.31179528245684e-13 1.02306109629548e-12 72
"chr1" 183244001 183245000 "*" 2.73625566649116e-12 7.52483363579137e-12 -100
"chr1" 183676001 183677000 "*" 3.88217591407169e-09 6.93518778632886e-09
51.6129032258064
"chr1" 183774001 183775000 "*" 0 0 100
"chr1" 183906001 183907000 "*" 1.35036426485158e-12 3.86629547363774e-12 100
"chr1" 184131001 184132000 "*" 7.78377362564697e-13 2.29551678017433e-12 -100
"chr1" 184429001 184430000 "*" 4.25848856178135e-09 7.53209581765839e-09
62.962962962963
"chr1" 184780001 184781000 "*" 2.11835727847998e-07 2.94228547207241e-07
57.1428571428571

"chr1" 185048001 185049000 "*" 4.08209022140227e-11 9.57770690255255e-11 -100
"chr1" 185345001 185346000 "*" 0.000241621869001674 0.000212792315955059
57.6923076923077
"chr1" 186190001 186191000 "*" 2.08814465718632e-09 3.86232567960463e-09 -100
"chr1" 186435001 186436000 "*" 6.22125684301977e-11 1.42751026696934e-10
55.5555555555556
"chr1" 186587001 186588000 "*" 1.67299207820548e-08 2.71377429441055e-08 -100
"chr1" 187318001 187319000 "*" 0.000115274736804838 0.000107060060050355
54.1666666666667
"chr1" 187805001 187806000 "*" 1.49880108324396e-14 5.44951848293151e-14 100
"chr1" 187901001 187902000 "*" 1.17461596005342e-13 3.8279067575877e-13 100
"chr1" 188943001 188944000 "*" 0 0 -86.5979381443299
"chr1" 189724001 189725000 "*" 1.60194080223164e-12 4.53860717499452e-12
-75.3846153846154
"chr1" 191313001 191314000 "*" 1.11022302462516e-16 5.03662826618488e-16
83.5820895522388
"chr1" 191654001 191655000 "*" 9.04570973681018e-10 1.76414389822173e-09 100
"chr1" 192067001 192068000 "*" 4.21884749357559e-15 1.63874445239189e-14 100
"chr1" 192134001 192135000 "*" 4.79616346638068e-14 1.64380445416596e-13 100
"chr1" 193684001 193685000 "*" 3.6700841921089e-08 5.65530226072258e-08 -100
"chr1" 193970001 193971000 "*" 1.79523063081888e-13 5.7367518231213e-13
54.5454545454545
"chr1" 194519001 194520000 "*" 0 0 100
"chr1" 195691001 195692000 "*" 9.2148511043888e-15 3.43705279328267e-14 100
"chr1" 196026001 196027000 "*" 9.80763359414993e-10 1.8980032424715e-09 100
"chr1" 196045001 196046000 "*" 9.32032229172819e-13 2.7181920310254e-12 100
"chr1" 196478001 196479000 "*" 1.13140401492018e-08 1.87801380787728e-08 100
"chr1" 196485001 196486000 "*" 1.67299207820548e-08 2.71377429441055e-08 100
"chr1" 196578001 196579000 "*" 0 0 76.25
"chr1" 196976001 196977000 "*" 0 0 100
"chr1" 197373001 197374000 "*" 2.55351295663786e-15 1.01678090381833e-14 100
"chr1" 197636001 197637000 "*" 0 0 -100
"chr1" 197887001 197888000 "*" 0 0 50.732210916342
"chr1" 197907001 197908000 "*" 0 0 100
"chr1" 198634001 198635000 "*" 4.79874173731076e-09 8.41803010893909e-09
-87.2340425531915
"chr1" 199675001 199676000 "*" 2.43005615629954e-09 4.45014222280278e-09 100
"chr1" 199840001 199841000 "*" 8.7349629751543e-09 1.47505871898511e-08 100
"chr1" 199999001 200000000 "*" 0 0 54.5454545454545
"chr1" 200062001 200063000 "*" 6.92287338566189e-11 1.57605630124247e-10 100
"chr1" 200269001 200270000 "*" 2.25262586361907e-10 4.78773106620275e-10
60.655737704918
"chr1" 200679001 200680000 "*" 0 0 -100
"chr1" 200722001 200723000 "*" 7.43849426498855e-15 2.80357739563059e-14 -100
"chr1" 200975001 200976000 "*" 1.26214766416055e-08 2.08406412460482e-08
67.965367965368
"chr1" 201003001 201004000 "*" 0 0 -100
"chr1" 201009001 201010000 "*" 3.13178021686955e-05 3.18058313017091e-05
-52.3809523809524
"chr1" 201021001 201022000 "*" 1.11022302462516e-16 5.03662826618488e-16
52.6315789473684
"chr1" 201056001 201057000 "*" 1.17793441667402e-10 2.60446848714757e-10

72.089552238806
"chr1" 201075001 201076000 "*" 1.4432899320127e-15 5.90750815956055e-15 -100
"chr1" 201083001 201084000 "*" 0 0 -64.0873015873016
"chr1" 201096001 201097000 "*" 3.33066907387547e-16 1.4495649018245e-15
-84.0909090909091
"chr1" 201178001 201179000 "*" 1.98294731001969e-06 2.41330824932622e-06
56.5217391304348
"chr1" 201184001 201185000 "*" 8.1956545862738e-05 7.7896169099937e-05
54.5454545454545
"chr1" 201219001 201220000 "*" 3.9190872769268e-14 1.35416418933905e-13 100
"chr1" 201220001 201221000 "*" 5.64878919528944e-07 7.42155605113794e-07
64.8648648648649
"chr1" 201221001 201222000 "*" 1.46549439250521e-14 5.33330076973935e-14 -100
"chr1" 201276001 201277000 "*" 7.0006535457523e-08 1.03695832512054e-07 -100
"chr1" 201279001 201280000 "*" 3.40125705378114e-10 7.03784073980162e-10 -100
"chr1" 201285001 201286000 "*" 1.78512946491516e-06 2.1878826958132e-06
65.5172413793103
"chr1" 201290001 201291000 "*" 0 0 100
"chr1" 201294001 201295000 "*" 2.44756081845265e-05 2.52637134962309e-05
64.5161290322581
"chr1" 201322001 201323000 "*" 3.84137166520304e-14 1.32929369817675e-13 -100
"chr1" 201330001 201331000 "*" 7.0006535457523e-08 1.03695832512054e-07 -100
"chr1" 201333001 201334000 "*" 1.39779633423487e-07 1.98463037958589e-07 100
"chr1" 201337001 201338000 "*" 6.51443953014752e-06 7.33754914986653e-06
58.6206896551724
"chr1" 201377001 201378000 "*" 0.000257038227272521 0.000225421852219643
52.2388059701493
"chr1" 201404001 201405000 "*" 0.000301667525735461 0.000261300142255719
-54.0540540540541
"chr1" 201420001 201421000 "*" 5.44568390381528e-10 1.09759499517836e-09
85.0574712643678
"chr1" 201475001 201476000 "*" 0 0 -90.0383141762452
"chr1" 201476001 201477000 "*" 0 0 -77.491961414791
"chr1" 201560001 201561000 "*" 3.99791311167519e-13 1.22027792394232e-12 100
"chr1" 201583001 201584000 "*" 1.75917058697905e-11 4.35257527084094e-11
-91.4285714285714
"chr1" 201666001 201667000 "*" 1.38590361409285e-10 3.02706178892116e-10 -100
"chr1" 201667001 201668000 "*" 4.08209022140227e-11 9.57770690255255e-11 -100
"chr1" 201672001 201673000 "*" 6.12778716657658e-11 1.40703078182493e-10
66.6666666666667
"chr1" 201708001 201709000 "*" 0 0 -81.6993464052288
"chr1" 201709001 201710000 "*" 0 0 -52.6726813128096
"chr1" 201864001 201865000 "*" 4.18887147191072e-13 1.2739946038722e-12 100
"chr1" 201869001 201870000 "*" 0 0 70
"chr1" 201886001 201887000 "*" 6.92287338566189e-11 1.57605630124247e-10 -100
"chr1" 201893001 201894000 "*" 2.91735258173276e-09 5.28473585192998e-09
-92.8571428571429
"chr1" 201900001 201901000 "*" 4.9585470174307e-09 8.68302175679199e-09
88.4615384615385
"chr1" 201968001 201969000 "*" 3.61932706027801e-14 1.25777907157994e-13 -100
"chr1" 201979001 201980000 "*" 1.77635683940025e-15 7.19870740878856e-15
-94.3502824858757

"chr1" 201985001 201986000 "*" 4.99900121297969e-12 1.32921056339624e-11 100
"chr1" 201987001 201988000 "*" 0 0 -98.6754966887417
"chr1" 202037001 202038000 "*" 4.73234496034536e-09 8.30627300264826e-09 -100
"chr1" 202088001 202089000 "*" 6.37490060739765e-13 1.89496793587877e-12 100
"chr1" 202126001 202127000 "*" 1.92946192356658e-09 3.59193011611483e-09 -100
"chr1" 202138001 202139000 "*" 1.39779633423487e-07 1.98463037958589e-07 100
"chr1" 202149001 202150000 "*" 7.105427357601e-15 2.68362283258525e-14
-66.6666666666667
"chr1" 202158001 202159000 "*" 1.11022302462516e-16 5.03662826618488e-16 100
"chr1" 202162001 202163000 "*" 3.96514819867022e-08 6.08792568772522e-08 -70
"chr1" 202187001 202188000 "*" 8.66802341015216e-09 1.4690792207855e-08
76.9230769230769
"chr1" 202205001 202206000 "*" 3.77330389156327e-10 7.76376047338581e-10 -95
"chr1" 202239001 202240000 "*" 4.26951363152739e-10 8.72510873516957e-10 -100
"chr1" 202276001 202277000 "*" 7.7715611723761e-16 3.26213507634405e-15 -100
"chr1" 202611001 202612000 "*" 0 0 59.5134261616452
"chr1" 202613001 202614000 "*" 0 0 58.252427184466
"chr1" 202614001 202615000 "*" 0.000130788944220583 0.000120344323127213 60
"chr1" 202621001 202622000 "*" 8.7349629751543e-09 1.47505871898511e-08 -100
"chr1" 202625001 202626000 "*" 4.26951363152739e-10 8.72510873516957e-10 -100
"chr1" 202784001 202785000 "*" 4.17234969685154e-07 5.57208763360643e-07 80
"chr1" 202814001 202815000 "*" 2.68450373042128e-10 5.64465213127486e-10 -100
"chr1" 202936001 202937000 "*" 0 0 -63.6363636363636
"chr1" 202998001 202999000 "*" 5.25379739713117e-12 1.39362731674666e-11
-66.6666666666667
"chr1" 203016001 203017000 "*" 0 0 -100
"chr1" 203018001 203019000 "*" 2.62088631242108e-07 3.59779054681028e-07
-76.9230769230769
"chr1" 203058001 203059000 "*" 3.87560728731806e-07 5.19714206061417e-07
63.3802816901408
"chr1" 203090001 203091000 "*" 1.52045043222415e-12 4.32624699789741e-12
-59.6330275229358
"chr1" 203099001 203100000 "*" 1.52794177310511e-09 2.87642564232045e-09 -100
"chr1" 203107001 203108000 "*" 8.7349629751543e-09 1.47505871898511e-08 -100
"chr1" 203115001 203116000 "*" 1.38124862658628e-08 2.26917815360148e-08
76.3636363636364
"chr1" 203130001 203131000 "*" 0 0 100
"chr1" 203154001 203155000 "*" 6.60323198964363e-06 7.43143807773833e-06
-61.5384615384615
"chr1" 203155001 203156000 "*" 4.09246636579041e-10 8.38842855496424e-10
57.1428571428571
"chr1" 203192001 203193000 "*" 1.0325074129014e-14 3.83008663711937e-14
80.4421768707483
"chr1" 203195001 203196000 "*" 2.75671430582936e-06 3.28605171729843e-06 -64
"chr1" 203242001 203243000 "*" 0 0 50.1386001386001
"chr1" 203245001 203246000 "*" 5.01025332333427e-10 1.01358031135889e-09 -100
"chr1" 203273001 203274000 "*" 1.94411153842111e-11 4.77923295676325e-11
54.6847888953152
"chr1" 203365001 203366000 "*" 2.342070981598e-11 5.69409842052749e-11
-83.3333333333333
"chr1" 203457001 203458000 "*" 0 0 -54.2970297029703
"chr1" 203490001 203491000 "*" 6.24654217240561e-10 1.2462108191303e-09 100

"chr1" 203693001 203694000 "*" 1.14124265593318e-11 2.89138062669327e-11 -100
"chr1" 203722001 203723000 "*" 8.74401685502235e-08 1.27854605705607e-07
-61.1111111111111
"chr1" 204050001 204051000 "*" 3.51607631898787e-13 1.08255764767511e-12
82.6086956521739
"chr1" 204071001 204072000 "*" 0 0 100
"chr1" 204124001 204125000 "*" 0 0 100
"chr1" 204167001 204168000 "*" 0 0 100
"chr1" 204183001 204184000 "*" 7.11664061014972e-12 1.8526478352197e-11
67.0707070707071
"chr1" 204187001 204188000 "*" 4.01313771103418e-09 7.11882087237587e-09 100
"chr1" 204195001 204196000 "*" 0 0 100
"chr1" 204244001 204245000 "*" 9.41103768226093e-05 8.86297231089954e-05
52.3809523809524
"chr1" 204257001 204258000 "*" 2.69007038866675e-13 8.38831022628993e-13 100
"chr1" 204265001 204266000 "*" 7.0006535457523e-08 1.03695832512054e-07 100
"chr1" 204282001 204283000 "*" 1.11022302462516e-16 5.03662826618488e-16 100
"chr1" 204321001 204322000 "*" 2.88710388929303e-10 6.03399264731139e-10 100
"chr1" 204329001 204330000 "*" 4.26951363152739e-10 8.72510873516957e-10 100
"chr1" 204378001 204379000 "*" 6.58169074796433e-11 1.50487332936514e-10
76.5625
"chr1" 204417001 204418000 "*" 4.44089209850063e-15 1.71914916534614e-14 100
"chr1" 204568001 204569000 "*" 9.04570973681018e-10 1.76414389822173e-09 -100
"chr1" 204586001 204587000 "*" 6.88338275267597e-15 2.60518659957509e-14 100
"chr1" 204595001 204596000 "*" 2.00227675550835e-08 3.20459176617961e-08 -100
"chr1" 204630001 204631000 "*" 1.07882591748876e-11 2.74553537455803e-11 -100
"chr1" 204644001 204645000 "*" 3.63445940010365e-11 8.58565209111204e-11 100
"chr1" 204647001 204648000 "*" 0 0 85.4748603351955
"chr1" 204663001 204664000 "*" 9.0072393987839e-13 2.63157725646847e-12 100
"chr1" 204670001 204671000 "*" 3.33066907387547e-16 1.4495649018245e-15 100
"chr1" 204797001 204798000 "*" 0 0 64.812598338425
"chr1" 204827001 204828000 "*" 7.19313497654639e-13 2.12704747709925e-12
-85.4545454545455
"chr1" 204843001 204844000 "*" 1.47024115104699e-09 2.78363533963333e-09
60.7142857142857
"chr1" 204890001 204891000 "*" 3.95353005888666e-09 7.03928529283733e-09 -100
"chr1" 204942001 204943000 "*" 0 0 73.6842105263158
"chr1" 204944001 204945000 "*" 1.45894769520982e-06 1.81153606811029e-06
-64.5390070921986
"chr1" 204956001 204957000 "*" 8.52140580320793e-12 2.19817259951489e-11 100
"chr1" 204960001 204961000 "*" 4.2068252631644e-05 4.18468260511008e-05 -56
"chr1" 204974001 204975000 "*" 3.12929393508909e-09 5.6513565147891e-09 -90
"chr1" 204975001 204976000 "*" 7.0006535457523e-08 1.03695832512054e-07 -100
"chr1" 205001001 205002000 "*" 5.55111512312578e-15 2.12516350804551e-14 100
"chr1" 205006001 205007000 "*" 0 0 75.7575757575758
"chr1" 205011001 205012000 "*" 0 0 95.7831325301205
"chr1" 205018001 205019000 "*" 1.34373534161902e-05 1.44386492693405e-05
64.7058823529412
"chr1" 205023001 205024000 "*" 2.22044604925031e-16 9.81641919380259e-16
90.9090909090909
"chr1" 205195001 205196000 "*" 2.00227675550835e-08 3.20459176617961e-08 -100
"chr1" 205227001 205228000 "*" 3.40125705378114e-10 7.03784073980162e-10 100

"chr1" 205246001 205247000 "*" 0 0 -67.2413793103448
"chr1" 205290001 205291000 "*" 0 0 55.1814094395422
"chr1" 205455001 205456000 "*" 4.21884749357559e-15 1.63874445239189e-14 100
"chr1" 205470001 205471000 "*" 1.22457599616155e-12 3.52441478267078e-12 -100
"chr1" 205618001 205619000 "*" 5.55111512312578e-16 2.36485094870365e-15 100
"chr1" 205919001 205920000 "*" 6.72795152922845e-13 1.99600116219405e-12
52.1739130434783
"chr1" 206299001 206300000 "*" 0.000235091807712418 0.000207505933348564
51.8518518518519
"chr1" 206310001 206311000 "*" 3.46500605985511e-13 1.06777978272295e-12 100
"chr1" 206320001 206321000 "*" 1.30340183090993e-13 4.22662705118657e-13 -100
"chr1" 206654001 206655000 "*" 3.33389944768836e-05 3.36886820820375e-05 53.125
"chr1" 206669001 206670000 "*" 6.65649757536357e-11 1.52114351480277e-10
-76.1904761904762
"chr1" 206690001 206691000 "*" 0 0 97.7777777777778
"chr1" 206699001 206700000 "*" 7.0006535457523e-08 1.03695832512054e-07 100
"chr1" 206709001 206710000 "*" 2.16382467499443e-13 6.82775854166559e-13 100
"chr1" 206715001 206716000 "*" 5.48638023900594e-10 1.10374357336421e-09 100
"chr1" 206808001 206809000 "*" 0 0 75.9776536312849
"chr1" 206848001 206849000 "*" 9.65729496371637e-10 1.87255912493232e-09 -100
"chr1" 206954001 206955000 "*" 1.48087875295744e-10 3.22075573380618e-10 -100
"chr1" 206973001 206974000 "*" 3.6700841921089e-08 5.65530226072258e-08 -100
"chr1" 207082001 207083000 "*" 0 0 57.8358208955224
"chr1" 207110001 207111000 "*" 6.48495701582874e-11 1.48385397387759e-10
-93.3333333333333
"chr1" 207155001 207156000 "*" 1.52794177310511e-09 2.87642564232045e-09 -100
"chr1" 207198001 207199000 "*" 1.11022302462516e-16 5.03662826618488e-16 -100
"chr1" 207271001 207272000 "*" 9.63829016598083e-12 2.46993429847588e-11 100
"chr1" 207402001 207403000 "*" 6.93112234273485e-13 2.05218842007357e-12 100
"chr1" 207494001 207495000 "*" 1.24342595023696e-05 1.34268907723144e-05
-66.6666666666667
"chr1" 207596001 207597000 "*" 9.58813684004411e-09 1.6110105266243e-08 60
"chr1" 207597001 207598000 "*" 0 0 56.3517915309446
"chr1" 207627001 207628000 "*" 0 0 56.9905533063428
"chr1" 207628001 207629000 "*" 0 0 56.1818495514148
"chr1" 207980001 207981000 "*" 2.75335310107039e-14 9.69727903855348e-14 -100
"chr1" 208000001 208001000 "*" 5.87992721179376e-09 1.02105033599712e-08
-62.962962962963
"chr1" 208004001 208005000 "*" 1.51578287699294e-08 2.47843136032199e-08
59.4427244582043
"chr1" 208164001 208165000 "*" 0 0 100
"chr1" 208204001 208205000 "*" 1.98124849859482e-10 4.25157611354621e-10 -100
"chr1" 208206001 208207000 "*" 2.18594896184143e-07 3.03076599600619e-07
69.8717948717949
"chr1" 208221001 208222000 "*" 3.65923472269714e-05 3.67415597585413e-05
-62.0689655172414
"chr1" 208229001 208230000 "*" 7.67056418382595e-11 1.7359448580318e-10 -100
"chr1" 208239001 208240000 "*" 2.00227675550835e-08 3.20459176617961e-08 100
"chr1" 208263001 208264000 "*" 1.96644922567657e-11 4.82930612417392e-11 100
"chr1" 208315001 208316000 "*" 1.74853465040314e-11 4.32752710900609e-11 -100
"chr1" 208425001 208426000 "*" 0 0 100
"chr1" 208431001 208432000 "*" 1.25284005392245e-10 2.75649590272477e-10 100

"chr1" 208441001 208442000 "*" 2.73625566649116e-12 7.52483363579137e-12 -100
"chr1" 209187001 209188000 "*" 1.54765008828495e-05 1.64771109106961e-05
66.6666666666667
"chr1" 209408001 209409000 "*" 1.13140401492018e-08 1.87801380787728e-08 100
"chr1" 209516001 209517000 "*" 2.04988059859801e-09 3.80458088311340e-09
70.2702702702703
"chr1" 209584001 209585000 "*" 9.65729496371637e-10 1.87255912493232e-09 100
"chr1" 209778001 209779000 "*" 2.15125472990962e-10 4.58126659943874e-10 -100
"chr1" 209835001 209836000 "*" 1.07882591748876e-11 2.74553537455803e-11 -100
"chr1" 209917001 209918000 "*" 4.90274202458174e-06 5.62669742552277e-06
51.5151515151515
"chr1" 210223001 210224000 "*" 2.03463946357729e-07 2.83279970699238e-07
83.3333333333333
"chr1" 210377001 210378000 "*" 5.08482145278322e-14 1.73432706170696e-13 -100
"chr1" 210577001 210578000 "*" 8.86993185567064e-08 1.29554903474019e-07
55.5555555555556
"chr1" 210792001 210793000 "*" 0 0 100
"chr1" 210796001 210797000 "*" 0 0 80.6451612903226
"chr1" 210867001 210868000 "*" 6.4324989779152e-11 1.47230020825092e-10 -100
"chr1" 211120001 211121000 "*" 0 0 88.1188118811881
"chr1" 211435001 211436000 "*" 5.48249515299748e-05 5.35608847348539e-05
51.4705882352941
"chr1" 211573001 211574000 "*" 8.43347613965761e-12 2.17859001990012e-11 -100
"chr1" 211790001 211791000 "*" 6.52733422867868e-12 1.71146951718423e-11 100
"chr1" 211807001 211808000 "*" 3.84137166520304e-14 1.32929369817675e-13 -100
"chr1" 211821001 211822000 "*" 3.10071915601284e-05 3.15124728670996e-05
58.6206896551724
"chr1" 211906001 211907000 "*" 1.39779633423487e-07 1.98463037958589e-07 100
"chr1" 212049001 212050000 "*" 4.26951363152739e-10 8.72510873516957e-10 -100
"chr1" 212097001 212098000 "*" 0 0 -100
"chr1" 212330001 212331000 "*" 8.7349629751543e-09 1.47505871898511e-08 100
"chr1" 212428001 212429000 "*" 1.86821217281752e-05 1.96397758022194e-05 60
"chr1" 212588001 212589000 "*" 0 0 78.2608695652174
"chr1" 212646001 212647000 "*" 7.03992419914812e-12 1.83313429961219e-11 100
"chr1" 212692001 212693000 "*" 3.84479115211889e-11 9.06351965260857e-11
57.3560767590618
"chr1" 212731001 212732000 "*" 0 0 57.578469984485
"chr1" 212780001 212781000 "*" 0 0 54.2579075425791
"chr1" 212841001 212842000 "*" 4.08209022140227e-11 9.57770690255255e-11 -100
"chr1" 212851001 212852000 "*" 2.24562506279113e-05 2.33198893526994e-05 56.25
"chr1" 212877001 212878000 "*" 2.44677114125125e-07 3.37192931864395e-07
-64.7058823529412
"chr1" 212996001 212997000 "*" 2.15125472990962e-10 4.58126659943874e-10 100
"chr1" 213123001 213124000 "*" 0 0 60.2941176470588
"chr1" 213189001 213190000 "*" 1.43642974735902e-05 1.53620283747625e-05
-65.2173913043478
"chr1" 213503001 213504000 "*" 0.000334851753864718 0.000287983064674082
-55.5555555555556
"chr1" 213579001 213580000 "*" 1.11022302462516e-16 5.03662826618488e-16 100
"chr1" 213584001 213585000 "*" 9.80763359414993e-10 1.8980032424715e-09 -100
"chr1" 213621001 213622000 "*" 0 0 -100
"chr1" 213868001 213869000 "*" 2.52953213930596e-12 6.99462031846971e-12

68.4210526315789
"chr1" 213956001 213957000 "*" 2.55351295663786e-15 1.01678090381833e-14 100
"chr1" 214102001 214103000 "*" 2.77555756156289e-14 9.77094403418417e-14
-95.0819672131148
"chr1" 214162001 214163000 "*" 0 0 82.2519083969466
"chr1" 214280001 214281000 "*" 2.22044604925031e-16 9.81641919380259e-16
55.2795031055901
"chr1" 214315001 214316000 "*" 2.82773804372027e-13 8.79129664109438e-13 100
"chr1" 214529001 214530000 "*" 8.90625305727255e-08 1.30046695793726e-07
58.3333333333333
"chr1" 214677001 214678000 "*" 1.31190838992268e-06 1.63923591886211e-06
-66.6666666666667
"chr1" 214927001 214928000 "*" 1.26565424807268e-14 4.63923203146481e-14 -100
"chr1" 215257001 215258000 "*" 1.52940516495725e-06 1.89327104296279e-06
81.8181818181818
"chr1" 215259001 215260000 "*" 2.22044604925031e-16 9.81641919380259e-16
-56.5217391304348
"chr1" 216426001 216427000 "*" 6.59550958292954e-09 1.13456194382773e-08 -100
"chr1" 216948001 216949000 "*" 3.52776696743717e-11 8.35728515355586e-11
57.1428571428571
"chr1" 217263001 217264000 "*" 0 0 50.0199027862523
"chr1" 218138001 218139000 "*" 8.34380342595864e-11 1.88197485153368e-10
-57.5757575757576
"chr1" 218261001 218262000 "*" 4.71134242729931e-12 1.257615036806e-11 100
"chr1" 218330001 218331000 "*" 0 0 100
"chr1" 218338001 218339000 "*" 0 0 50.7216624721199
"chr1" 218458001 218459000 "*" 0 0 72.0388349514563
"chr1" 218899001 218900000 "*" 1.07882591748876e-11 2.74553537455803e-11 -100
"chr1" 218925001 218926000 "*" 1.52794177310511e-09 2.87642564232045e-09 -100
"chr1" 218940001 218941000 "*" 0.000354266918853763 0.000303242292809723
-56.5217391304348
"chr1" 219674001 219675000 "*" 1.37828859436695e-10 3.01445544267661e-10 100
"chr1" 219810001 219811000 "*" 9.65729496371637e-10 1.87255912493232e-09 -100
"chr1" 220700001 220701000 "*" 0 0 60.4412888149912
"chr1" 220702001 220703000 "*" 0 0 94.4944944944945
"chr1" 220880001 220881000 "*" 5.06406028222273e-12 1.34547995232219e-11
-50.0240731824747
"chr1" 220950001 220951000 "*" 4.44089209850063e-16 1.91071758245033e-15 -100
"chr1" 221065001 221066000 "*" 0 0 50.9011656381624
"chr1" 221095001 221096000 "*" 3.40125705378114e-10 7.03784073980162e-10 100
"chr1" 221157001 221158000 "*" 0 0 100
"chr1" 221414001 221415000 "*" 3.85540880221136e-06 4.49215246381664e-06 80
"chr1" 221418001 221419000 "*" 2.68450373042128e-10 5.64465213127486e-10 100
"chr1" 221616001 221617000 "*" 6.44564628715649e-06 7.26663863268796e-06
-52.1739130434783
"chr1" 221626001 221627000 "*" 2.65468175539851e-05 2.72579034500328e-05
-51.8518518518519
"chr1" 221715001 221716000 "*" 3.6700841921089e-08 5.65530226072258e-08 100
"chr1" 221817001 221818000 "*" 0 0 100
"chr1" 222019001 222020000 "*" 0 0 -100
"chr1" 222046001 222047000 "*" 2.00227675550835e-08 3.20459176617961e-08 -100
"chr1" 222052001 222053000 "*" 1.5277158427196e-09 2.87642564232045e-09 -100

"chr1" 222219001 222220000 "*" 3.04324343503026e-11 7.26599935673121e-11 -100
"chr1" 222293001 222294000 "*" 8.79296635503124e-14 2.9138932826348e-13 -66.6666666666667
"chr1" 222648001 222649000 "*" 0 0 100
"chr1" 223202001 223203000 "*" 3.56775498033812e-10 7.35898629809829e-10 -100
"chr1" 223210001 223211000 "*" 1.51072940823838e-08 2.4708951517826e-08 50.9433962264151
"chr1" 223218001 223219000 "*" 8.7349629751543e-09 1.47505871898511e-08 -100
"chr1" 223341001 223342000 "*" 2.22044604925031e-16 9.81641919380259e-16 -62.8571428571429
"chr1" 223404001 223405000 "*" 2.38575468822777e-05 2.46816531377115e-05 -66.6666666666667
"chr1" 223443001 223444000 "*" 5.63660229602192e-13 1.68905058935478e-12 100
"chr1" 223536001 223537000 "*" 2.52614595908085e-11 6.12199493329825e-11 76
"chr1" 223537001 223538000 "*" 0 0 69.9057714958775
"chr1" 223538001 223539000 "*" 0 0 57.3869604932633
"chr1" 223574001 223575000 "*" 1.87294624254264e-13 5.97285292681429e-13 95.6521739130435
"chr1" 223673001 223674000 "*" 1.67299207820548e-08 2.71377429441055e-08 100
"chr1" 223674001 223675000 "*" 0.000150576069463337 0.000137139618444501 53.3333333333333
"chr1" 223675001 223676000 "*" 0 0 -75.8241758241758
"chr1" 223712001 223713000 "*" 1.88737914186277e-14 6.78632187443082e-14 63.3333333333333
"chr1" 223887001 223888000 "*" 4.73234496034536e-09 8.30627300264826e-09 100
"chr1" 223899001 223900000 "*" 0 0 57.6923076923077
"chr1" 224034001 224035000 "*" 2.22044604925031e-15 8.90449334476539e-15 -100
"chr1" 224058001 224059000 "*" 6.99440505513849e-15 2.64480529781883e-14 -100
"chr1" 224425001 224426000 "*" 8.88178419700125e-16 3.70670032207938e-15 -100
"chr1" 224803001 224804000 "*" 0 0 82.9034456592001
"chr1" 224815001 224816000 "*" 1.52794177310511e-09 2.87642564232045e-09 -100
"chr1" 224840001 224841000 "*" 4.58591639107553e-08 6.99288081435725e-08 -74.2857142857143
"chr1" 224884001 224885000 "*" 1.14335407869204e-05 1.24179930779228e-05 -54.5454545454545
"chr1" 224906001 224907000 "*" 7.88303955445713e-09 1.34409574576075e-08 -56.6037735849057
"chr1" 224921001 224922000 "*" 0 0 -94.8717948717949
"chr1" 224923001 224924000 "*" 2.77400968862196e-09 5.0372170400349e-09 62.8571428571429
"chr1" 224954001 224955000 "*" 8.7349629751543e-09 1.47505871898511e-08 -100
"chr1" 225117001 225118000 "*" 0 0 91.0554561717352
"chr1" 225637001 225638000 "*" 0 0 -82.4324324324324
"chr1" 225648001 225649000 "*" 2.70339306496226e-13 8.42355472617862e-13 100
"chr1" 225661001 225662000 "*" 2.455369241261e-12 6.8003688121118e-12 100
"chr1" 225666001 225667000 "*" 9.76709724209712e-09 1.63975827316807e-08 -84.2105263157895
"chr1" 225867001 225868000 "*" 0 0 100
"chr1" 225900001 225901000 "*" 9.04570973681018e-10 1.76414389822173e-09 100
"chr1" 225907001 225908000 "*" 0 0 -100
"chr1" 225961001 225962000 "*" 2.66324193279877e-05 2.73400755089686e-05 63.6363636363636

"chr1" 226000001 226001000 "*" 4.86753970463383e-11 1.13145908161915e-10 100
"chr1" 226036001 226037000 "*" 9.2962427755694e-09 1.56483495475315e-08 -52
"chr1" 226069001 226070000 "*" 0 0 100
"chr1" 226092001 226093000 "*" 1.10134124042816e-12 3.18722464530964e-12
92.3076923076923
"chr1" 226129001 226130000 "*" 7.88258347483861e-15 2.96145301916012e-14 -100
"chr1" 226137001 226138000 "*" 3.04324343503026e-11 7.26599935673121e-11 100
"chr1" 226288001 226289000 "*" 1.36139899148402e-05 1.46162077113887e-05
-63.4615384615385
"chr1" 226297001 226298000 "*" 0 0 62.1794871794872
"chr1" 226315001 226316000 "*" 0 0 -100
"chr1" 226321001 226322000 "*" 5.99520433297585e-15 2.28531569014092e-14 -100
"chr1" 226396001 226397000 "*" 7.0006535457523e-08 1.03695832512054e-07 100
"chr1" 226534001 226535000 "*" 3.95353005888666e-09 7.03928529283733e-09 -100
"chr1" 226556001 226557000 "*" 5.25299859166495e-09 9.17245030097968e-09
52.3809523809524
"chr1" 226616001 226617000 "*" 1.01906444494038e-06 1.29182844104915e-06
68.4719535783366
"chr1" 226643001 226644000 "*" 4.92582776712158e-06 5.65100933800874e-06
58.3333333333333
"chr1" 226649001 226650000 "*" 2.15125472990962e-10 4.58126659943874e-10 100
"chr1" 226670001 226671000 "*" 3.48387985127374e-13 1.07312013500854e-12
90.1639344262295
"chr1" 226722001 226723000 "*" 1.13140401492018e-08 1.87801380787728e-08 100
"chr1" 226736001 226737000 "*" 0 0 60.2932237509044
"chr1" 226737001 226738000 "*" 0 0 62.1360824742268
"chr1" 226749001 226750000 "*" 3.95353005888666e-09 7.03928529283733e-09 100
"chr1" 226755001 226756000 "*" 1.06955344580939e-09 2.06112833509361e-09
-53.125
"chr1" 226774001 226775000 "*" 5.24177368177448e-11 1.21347798372677e-10
52.3809523809524
"chr1" 226799001 226800000 "*" 0 0 -100
"chr1" 226830001 226831000 "*" 0 0 -92.3076923076923
"chr1" 226831001 226832000 "*" 0 0 93.989898989899
"chr1" 226843001 226844000 "*" 8.98110696923027e-10 1.75478641394683e-09
-83.8709677419355
"chr1" 226901001 226902000 "*" 1.99840144432528e-12 5.59476580335607e-12 -100
"chr1" 226950001 226951000 "*" 0 0 100
"chr1" 227026001 227027000 "*" 1.37164501978759e-10 3.00615311518628e-10
67.741935483871
"chr1" 227183001 227184000 "*" 1.35891701003032e-07 1.93757587678022e-07
68.2926829268293
"chr1" 227507001 227508000 "*" 1.39779633423487e-07 1.98463037958589e-07 100
"chr1" 227653001 227654000 "*" 0 0 100
"chr1" 227898001 227899000 "*" 0 0 100
"chr1" 227947001 227948000 "*" 1.75678315228467e-08 2.84222966683147e-08
-81.5789473684211
"chr1" 227956001 227957000 "*" 4.27080593112805e-12 1.14751350282988e-11
-82.967032967033
"chr1" 228026001 228027000 "*" 1.88737914186277e-15 7.62011598380321e-15 100
"chr1" 228027001 228028000 "*" 4.72528449702025e-09 8.30627300264826e-09
95.2380952380952

"chr1" 228086001 228087000 "*" 7.2375438975314e-13 2.13942087404442e-12
-61.7647058823529
"chr1" 228092001 228093000 "*" 0 0 93.1156569630213
"chr1" 228105001 228106000 "*" 1.44236241828022e-06 1.79195636844577e-06
58.8235294117647
"chr1" 228114001 228115000 "*" 0 0 58.6206896551724
"chr1" 228127001 228128000 "*" 0 0 71.5909090909091
"chr1" 228192001 228193000 "*" 3.40394379350073e-13 1.0506709282888e-12
83.3333333333333
"chr1" 228194001 228195000 "*" 0 0 57.4726704926342
"chr1" 228204001 228205000 "*" 1.96644922567657e-11 4.82930612417392e-11 -100
"chr1" 228208001 228209000 "*" 1.48403511701645e-11 3.70689457767456e-11
-52.7179487179487
"chr1" 228220001 228221000 "*" 1.11022302462516e-16 5.03662826618488e-16 100
"chr1" 228225001 228226000 "*" 0 0 51.7458371424095
"chr1" 228233001 228234000 "*" 0 0 53.3834586466165
"chr1" 228236001 228237000 "*" 3.6700841921089e-08 5.65530226072258e-08 100
"chr1" 228237001 228238000 "*" 2.08814465718632e-09 3.86232567960463e-09 100
"chr1" 228253001 228254000 "*" 0 0 -100
"chr1" 228262001 228263000 "*" 0 0 100
"chr1" 228263001 228264000 "*" 9.28996968241336e-09 1.56387369828356e-08
-84.6938775510204
"chr1" 228289001 228290000 "*" 1.95399252334028e-14 7.01253661173697e-14
-57.2745741522113
"chr1" 228365001 228366000 "*" 3.95353005888666e-09 7.03928529283733e-09 -100
"chr1" 228415001 228416000 "*" 0 0 100
"chr1" 228491001 228492000 "*" 2.22044604925031e-16 9.81641919380259e-16 -100
"chr1" 228492001 228493000 "*" 4.08209022140227e-11 9.57770690255255e-11 100
"chr1" 228507001 228508000 "*" 5.40384160677698e-05 5.28457203825496e-05
-58.0645161290323
"chr1" 228511001 228512000 "*" 6.00176575105138e-10 1.20209281144702e-09
91.6666666666667
"chr1" 228523001 228524000 "*" 3.14193115968919e-14 1.09892744201672e-13
-78.2608695652174
"chr1" 228524001 228525000 "*" 0 0 59.8765432098765
"chr1" 228534001 228535000 "*" 1.11022302462516e-16 5.03662826618488e-16
64.1025641025641
"chr1" 228537001 228538000 "*" 1.20281562487889e-12 3.46585342181251e-12 100
"chr1" 228593001 228594000 "*" 0 0 -82.5581395348837
"chr1" 228595001 228596000 "*" 0.000120468649686067 0.00011154597818208
-55.1724137931034
"chr1" 228637001 228638000 "*" 4.14304184901404e-05 4.12507255840686e-05
55.5555555555556
"chr1" 228645001 228646000 "*" 0 0 53.5919540229885
"chr1" 228646001 228647000 "*" 0 0 100
"chr1" 228924001 228925000 "*" 5.13554375691783e-08 7.77455353225249e-08
57.6422764227642
"chr1" 229009001 229010000 "*" 1.07882591748876e-11 2.74553537455803e-11 -100
"chr1" 229030001 229031000 "*" 2.15125472990962e-10 4.58126659943874e-10 100
"chr1" 229068001 229069000 "*" 1.38590361409285e-10 3.02706178892116e-10 100
"chr1" 229079001 229080000 "*" 2.00227675550835e-08 3.20459176617961e-08 -100
"chr1" 229082001 229083000 "*" 1.17461596005342e-13 3.8279067575877e-13 -100

"chr1" 229140001 229141000 "*" 1.51656465163796e-13 4.87803420672651e-13 100
"chr1" 229160001 229161000 "*" 0 0 -100
"chr1" 229193001 229194000 "*" 6.4399653571745e-05 6.22599140150143e-05
61.5384615384615
"chr1" 229244001 229245000 "*" 6.92287338566189e-11 1.57605630124247e-10 100
"chr1" 229300001 229301000 "*" 7.0006535457523e-08 1.03695832512054e-07 100
"chr1" 229340001 229341000 "*" 2.88710388929303e-10 6.03399264731139e-10 -100
"chr1" 229353001 229354000 "*" 1.07864655669587e-05 1.17574627643031e-05
63.8888888888889
"chr1" 229408001 229409000 "*" 2.69007038866675e-13 8.38831022628993e-13 100
"chr1" 229516001 229517000 "*" 6.32651264709239e-10 1.26147121274561e-09
64.7058823529412
"chr1" 229542001 229543000 "*" 2.2494484053226e-10 4.78122138746311e-10
52.7777777777778
"chr1" 229670001 229671000 "*" 0 0 100
"chr1" 229825001 229826000 "*" 9.65729496371637e-10 1.87255912493232e-09 -100
"chr1" 229905001 229906000 "*" 4.08990052408464e-05 4.07512937702815e-05
72.7272727272727
"chr1" 229915001 229916000 "*" 0 0 -74.025974025974
"chr1" 229987001 229988000 "*" 1.92884597183252e-11 4.75178999226159e-11
-54.0983606557377
"chr1" 229996001 229997000 "*" 2.19853864003028e-08 3.50003158916803e-08
66.6666666666667
"chr1" 229998001 229999000 "*" 9.08108033215171e-11 2.03407753381119e-10 -100
"chr1" 230284001 230285000 "*" 2.04292838201781e-09 3.79300264241924e-09
66.6666666666667
"chr1" 230295001 230296000 "*" 3.52704532247117e-11 8.35581292290093e-11 -100
"chr1" 230332001 230333000 "*" 0 0 100
"chr1" 230472001 230473000 "*" 9.34172739164296e-11 2.0898220953406e-10 -80
"chr1" 230478001 230479000 "*" 7.59170504238682e-13 2.24156868839277e-12
-71.0526315789474
"chr1" 230480001 230481000 "*" 1.52992296875709e-06 1.89382781181468e-06
-62.962962962963
"chr1" 230503001 230504000 "*" 3.76365605347928e-14 1.3043487970679e-13 100
"chr1" 230506001 230507000 "*" 0.000235599168805112 0.000207894354164181
55.5555555555556
"chr1" 230516001 230517000 "*" 4.2986978421311e-09 7.59902490718044e-09
68.3333333333333
"chr1" 230561001 230562000 "*" 0 0 50.5808945718404
"chr1" 230562001 230563000 "*" 0 0 51.8095238095238
"chr1" 230691001 230692000 "*" 0 0 -100
"chr1" 230838001 230839000 "*" 0 0 100
"chr1" 230882001 230883000 "*" 0 0 -100
"chr1" 230887001 230888000 "*" 0.000525299954949099 0.000436849389802675
54.5454545454545
"chr1" 230910001 230911000 "*" 0 0 100
"chr1" 230914001 230915000 "*" 0.000471727282945977 0.000395504194557835
-53.8461538461538
"chr1" 230928001 230929000 "*" 1.5277158427196e-09 2.87642564232045e-09 -100
"chr1" 231003001 231004000 "*" 0 0 100
"chr1" 231074001 231075000 "*" 9.22053511209775e-07 1.17627123523267e-06
-62.7450980392157

"chr1" 231177001 231178000 "*" 9.27036225562006e-14 3.06430451936223e-13
-58.7320574162679
"chr1" 231198001 231199000 "*" 1.28785870856518e-14 4.71440608755782e-14 -100
"chr1" 231260001 231261000 "*" 0 0 79.5918367346939
"chr1" 231265001 231266000 "*" 8.02691246803988e-14 2.67352274101148e-13 100
"chr1" 231323001 231324000 "*" 3.45279360658424e-14 1.20341591074277e-13
-85.3333333333333
"chr1" 231348001 231349000 "*" 3.6700841921089e-08 5.65530226072258e-08 100
"chr1" 231463001 231464000 "*" 2.41784279456647e-05 2.49875818383709e-05
67.741935483871
"chr1" 231614001 231615000 "*" 2.43005615629954e-09 4.45014222280278e-09 100
"chr1" 231666001 231667000 "*" 0 0 -91.304347826087
"chr1" 231735001 231736000 "*" 6.42745973644665e-06 7.24848719155575e-06 -60
"chr1" 231822001 231823000 "*" 1.97040500671264e-06 2.39940940694731e-06
-65.3061224489796
"chr1" 232007001 232008000 "*" 9.65729496371637e-10 1.87255912493232e-09 -100
"chr1" 232076001 232077000 "*" 3.56775498033812e-10 7.35898629809829e-10 100
"chr1" 232125001 232126000 "*" 3.04324343503026e-11 7.26599935673121e-11 100
"chr1" 232193001 232194000 "*" 9.06273616041453e-07 1.15739767942151e-06
52.3809523809524
"chr1" 232420001 232421000 "*" 8.7349629751543e-09 1.47505871898511e-08 -100
"chr1" 232735001 232736000 "*" 6.15840711759574e-13 1.83676803572802e-12 -100
"chr1" 232811001 232812000 "*" 1.13140401492018e-08 1.87801380787728e-08 -100
"chr1" 232829001 232830000 "*" 3.6700841921089e-08 5.65530226072258e-08 100
"chr1" 232871001 232872000 "*" 2.64325332233195e-06 3.15856923971574e-06
-53.8461538461538
"chr1" 233011001 233012000 "*" 3.90831811358794e-12 1.05610567215048e-11 -100
"chr1" 233124001 233125000 "*" 3.6700841921089e-08 5.65530226072258e-08 100
"chr1" 233376001 233377000 "*" 2.22044604925031e-16 9.81641919380259e-16 100
"chr1" 233595001 233596000 "*" 3.6700841921089e-08 5.65530226072258e-08 100
"chr1" 233869001 233870000 "*" 0.000168529599185452 0.000152316702506419 60
"chr1" 233927001 233928000 "*" 3.02987580799163e-05 3.08411348302445e-05
57.1428571428571
"chr1" 234234001 234235000 "*" 6.29496454962464e-14 2.12354661990142e-13
-81.8260869565217
"chr1" 234293001 234294000 "*" 7.43054491603168e-07 9.60154664117997e-07 70
"chr1" 234356001 234357000 "*" 0 0 -100
"chr1" 234386001 234387000 "*" 9.5881362849326e-09 1.6110105266243e-08 60
"chr1" 234458001 234459000 "*" 2.35297015205305e-08 3.73363995104836e-08
51.1627906976744
"chr1" 234687001 234688000 "*" 7.21644966006352e-15 2.72345776413425e-14 -100
"chr1" 234695001 234696000 "*" 4.0237924103792e-11 9.46809309014224e-11
-76.0869565217391
"chr1" 234770001 234771000 "*" 3.95353005888666e-09 7.03928529283733e-09 -100
"chr1" 234858001 234859000 "*" 1.07288622430701e-10 2.38383042616132e-10
65.4002713704206
"chr1" 234907001 234908000 "*" 4.04464034668983e-05 4.03345421309266e-05
61.1111111111111
"chr1" 234915001 234916000 "*" 0 0 -100
"chr1" 234917001 234918000 "*" 4.08209022140227e-11 9.57770690255255e-11 100
"chr1" 235005001 235006000 "*" 1.29656123615263e-08 2.13813640920277e-08
51.0695187165775

"chr1" 235059001 235060000 "*" 9.62896429257398e-13 2.80291205576417e-12 -100
"chr1" 235076001 235077000 "*" 5.39636046958947e-09 9.41213844999648e-09
84.2105263157895
"chr1" 235099001 235100000 "*" 2.55351295663786e-15 1.01678090381833e-14
-69.3548387096774
"chr1" 235134001 235135000 "*" 0 0 100
"chr1" 235183001 235184000 "*" 1.52794177310511e-09 2.87642564232045e-09 -100
"chr1" 235267001 235268000 "*" 3.33066907387547e-15 1.30941888440006e-14 100
"chr1" 235313001 235314000 "*" 5.6362137179633e-11 1.29963270300065e-10 100
"chr1" 235675001 235676000 "*" 2.44805405946735e-08 3.87506495191586e-08
-66.6666666666667
"chr1" 235728001 235729000 "*" 9.32032229172819e-13 2.7181920310254e-12 -100
"chr1" 235752001 235753000 "*" 1.95335769781479e-09 3.63430583654299e-09
74.5454545454545
"chr1" 235778001 235779000 "*" 1.12458486967171e-09 2.15740815043925e-09 -100
"chr1" 235800001 235801000 "*" 3.03979064142368e-13 9.42350883309283e-13 -100
"chr1" 235804001 235805000 "*" 1.11022302462516e-16 5.03662826618488e-16
-82.6086956521739
"chr1" 235812001 235813000 "*" 0 0 54.2642924086223
"chr1" 235813001 235814000 "*" 0 0 88.2287245923609
"chr1" 236053001 236054000 "*" 7.77825037712176e-09 1.32690687610048e-08
-92.3076923076923
"chr1" 236087001 236088000 "*" 1.07882591748876e-11 2.74553537455803e-11 -100
"chr1" 236135001 236136000 "*" 1.54085633141676e-11 3.8402011705214e-11
65.7142857142857
"chr1" 236171001 236172000 "*" 5.61876555992269e-05 5.48048639509562e-05
53.3333333333333
"chr1" 236248001 236249000 "*" 3.10862446895044e-15 1.22466437093774e-14 -100
"chr1" 236275001 236276000 "*" 0 0 67.1641791044776
"chr1" 236311001 236312000 "*" 2.28411733971257e-10 4.85120143864209e-10
-62.8571428571429
"chr1" 236331001 236332000 "*" 0 0 100
"chr1" 236333001 236334000 "*" 1.87405646556726e-13 5.97285292681429e-13 -100
"chr1" 236478001 236479000 "*" 3.23373829846929e-06 3.81325174359463e-06
66.6666666666667
"chr1" 236562001 236563000 "*" 6.66133814775094e-16 2.81595744474255e-15 -100
"chr1" 236628001 236629000 "*" 1.39779633423487e-07 1.98463037958589e-07 -100
"chr1" 236686001 236687000 "*" 4.719984403323e-10 9.59022556881712e-10
-90.7894736842105
"chr1" 236687001 236688000 "*" 0 0 -81.9209039548023
"chr1" 236857001 236858000 "*" 0.000245538078540553 0.000215951647556943
54.5454545454545
"chr1" 236898001 236899000 "*" 0.00022586132366087 0.000199925618889989
53.5714285714286
"chr1" 236903001 236904000 "*" 5.21804821573824e-15 2.00446252136729e-14
98.3333333333333
"chr1" 236949001 236950000 "*" 5.5167942081269e-07 7.25648175245512e-07
-58.768115942029
"chr1" 237093001 237094000 "*" 5.55111512312578e-16 2.36485094870365e-15 100
"chr1" 237192001 237193000 "*" 8.7349629751543e-09 1.47505871898511e-08 100
"chr1" 237193001 237194000 "*" 7.98432294013551e-06 8.87935165531163e-06
-55.8333333333333

"chr1" 237415001 237416000 "*" 0.000170900372238547 0.00015425218250759 53.8461538461538
"chr1" 237691001 237692000 "*" 7.7715611723761e-16 3.26213507634405e-15 100
"chr1" 237713001 237714000 "*" 1.06503694752291e-12 3.08687484698138e-12 -66.6666666666667
"chr1" 237791001 237792000 "*" 7.34939306079596e-07 9.50242626154403e-07 -71.7391304347826
"chr1" 237900001 237901000 "*" 2.02060590481778e-13 6.40127405465654e-13 -100
"chr1" 237947001 237948000 "*" 0 0 -65.2266628330458
"chr1" 238101001 238102000 "*" 6.92287338566189e-11 1.57605630124247e-10 100
"chr1" 238648001 238649000 "*" 0 0 88.2352941176471
"chr1" 238730001 238731000 "*" 0.00020976332305167 0.000186660769941092 52.3809523809524
"chr1" 239196001 239197000 "*" 1.21689615051324e-08 2.01249230800208e-08 -66.6666666666667
"chr1" 239551001 239552000 "*" 0 0 100
"chr1" 239806001 239807000 "*" 8.28581159240116e-09 1.40693133083423e-08 -52
"chr1" 239870001 239871000 "*" 6.77335965093562e-12 1.76897011246462e-11 -100
"chr1" 240118001 240119000 "*" 3.36885872623816e-05 3.4020507556965e-05 -53.2258064516129
"chr1" 240298001 240299000 "*" 5.39619190880192e-05 5.27870047241069e-05 52.1739130434783
"chr1" 240308001 240309000 "*" 0 0 54.6511627906977
"chr1" 240367001 240368000 "*" 2.04292838201781e-09 3.79300264241924e-09 -66.6666666666667
"chr1" 240486001 240487000 "*" 0 0 -100
"chr1" 240526001 240527000 "*" 0 0 100
"chr1" 240614001 240615000 "*" 4.57183624291702e-10 9.31326369875184e-10 90.9090909090909
"chr1" 240626001 240627000 "*" 2.03161964800191e-05 2.12374183247922e-05 -70.8333333333333
"chr1" 240641001 240642000 "*" 1.20591092667155e-10 2.66075324129686e-10 -100
"chr1" 240751001 240752000 "*" 0 0 -100
"chr1" 240803001 240804000 "*" 0 0 -100
"chr1" 240817001 240818000 "*" 3.96222721299466e-10 8.13421565824133e-10 52.9411764705882
"chr1" 240887001 240888000 "*" 3.20266586029128e-07 4.343799335014e-07 76.4705882352941
"chr1" 241137001 241138000 "*" 2.62900812231237e-13 8.21294938531841e-13 -100
"chr1" 241506001 241507000 "*" 6.4324989779152e-11 1.47230020825092e-10 -100
"chr1" 241520001 241521000 "*" 0 0 61.9104889634227
"chr1" 241577001 241578000 "*" 3.6700841921089e-08 5.65530226072258e-08 100
"chr1" 241713001 241714000 "*" 1.5277158427196e-09 2.87642564232045e-09 100
"chr1" 241804001 241805000 "*" 0 0 100
"chr1" 241813001 241814000 "*" 1.0551626239419e-10 2.34638843495876e-10 73.469387755102
"chr1" 242160001 242161000 "*" 2.00227675550835e-08 3.20459176617961e-08 100
"chr1" 242222001 242223000 "*" 2.16382467499443e-13 6.82775854166559e-13 -100
"chr1" 242375001 242376000 "*" 0 0 -96.078431372549
"chr1" 242615001 242616000 "*" 2.02327044007689e-12 5.65300384792621e-12 100
"chr1" 242686001 242687000 "*" 0 0 75
"chr1" 242991001 242992000 "*" 3.46104942816616e-07 4.67482227931052e-07

-51.5151515151515
"chr1" 243270001 243271000 "*" 2.82773804372027e-13 8.79129664109438e-13 100
"chr1" 243511001 243512000 "*" 2.88710388929303e-10 6.03399264731139e-10 -100
"chr1" 243644001 243645000 "*" 6.15840711759574e-13 1.83676803572802e-12 100
"chr1" 243982001 243983000 "*" 5.48638023900594e-10 1.10374357336421e-09 -100
"chr1" 244013001 244014000 "*" 0 0 93.187003968254
"chr1" 244058001 244059000 "*" 4.48406133335411e-08 6.84482503893299e-08
-66.6666666666667
"chr1" 244060001 244061000 "*" 2.16382467499443e-13 6.82775854166559e-13 100
"chr1" 244134001 244135000 "*" 0 0 83.4586466165414
"chr1" 244173001 244174000 "*" 7.67056418382595e-11 1.7359448580318e-10 -100
"chr1" 244250001 244251000 "*" 5.77315972805081e-15 2.205310974382e-14
-58.695652173913
"chr1" 244266001 244267000 "*" 5.10702591327572e-15 1.96271840288234e-14 100
"chr1" 244268001 244269000 "*" 0 0 -100
"chr1" 244327001 244328000 "*" 7.0006535457523e-08 1.03695832512054e-07 100
"chr1" 244362001 244363000 "*" 1.72485026261882e-10 3.72764929830203e-10 67
"chr1" 244372001 244373000 "*" 1.48487888651516e-11 3.70689457767456e-11 100
"chr1" 244400001 244401000 "*" 7.75573483213776e-09 1.32322843386823e-08
73.8095238095238
"chr1" 244454001 244455000 "*" 0.000354266937660941 0.000303242292809723
-56.5217391304348
"chr1" 244462001 244463000 "*" 0 0 -100
"chr1" 244500001 244501000 "*" 3.48276962824912e-13 1.07285740703578e-12
55.3191489361702
"chr1" 244738001 244739000 "*" 0 0 -67.7966101694915
"chr1" 244739001 244740000 "*" 8.7349629751543e-09 1.47505871898511e-08 -100
"chr1" 244800001 244801000 "*" 0 0 -91.25
"chr1" 245050001 245051000 "*" 2.15125472990962e-10 4.58126659943874e-10 100
"chr1" 245082001 245083000 "*" 0 0 -100
"chr1" 245318001 245319000 "*" 0 0 67.1663685152057
"chr1" 245381001 245382000 "*" 7.7715611723761e-16 3.26213507634405e-15 -100
"chr1" 245397001 245398000 "*" 1.22124532708767e-15 5.03921984217778e-15 60
"chr1" 245410001 245411000 "*" 4.87564433271359e-11 1.13331141450576e-10
-88.1188118811881
"chr1" 245455001 245456000 "*" 2.00871430600102e-10 4.3076334661872e-10
58.8832487309645
"chr1" 245464001 245465000 "*" 0 0 -100
"chr1" 245496001 245497000 "*" 3.9788609074165e-09 7.08227025555472e-09
55.2197802197802
"chr1" 245581001 245582000 "*" 0 0 76.9275362318841
"chr1" 245644001 245645000 "*" 9.0072393987839e-13 2.63157725646847e-12 -100
"chr1" 245665001 245666000 "*" 9.63829016598083e-12 2.46993429847588e-11 100
"chr1" 245741001 245742000 "*" 1.70588222436763e-08 2.76390027120887e-08 -87.5
"chr1" 245747001 245748000 "*" 0 0 100
"chr1" 245835001 245836000 "*" 0 0 80.9523809523809
"chr1" 245858001 245859000 "*" 1.89848137210902e-14 6.82212372863451e-14 100
"chr1" 246203001 246204000 "*" 1.14237547976792e-07 1.64476938186197e-07
75.609756097561
"chr1" 246315001 246316000 "*" 3.90465437760668e-13 1.19307893943277e-12 -100
"chr1" 246384001 246385000 "*" 0 0 -100
"chr1" 246945001 246946000 "*" 0 0 -100

"chr1" 246950001 246951000 "*" 0 0 68.8888888888889
"chr1" 246998001 246999000 "*" 4.93267648948859e-11 1.14608902952886e-10 96.6666666666667
"chr1" 247021001 247022000 "*" 3.58894225660489e-06 4.20417278477504e-06 63.6363636363636
"chr1" 247139001 247140000 "*" 0 0 -100
"chr1" 247170001 247171000 "*" 0 0 100
"chr1" 247227001 247228000 "*" 1.19904086659517e-14 4.40901388691796e-14 -53.4883720930233
"chr1" 247248001 247249000 "*" 2.13696561385257e-05 2.22671974406952e-05 51.2820512820513
"chr1" 247524001 247525000 "*" 5.794809077031e-12 1.52911192757929e-11 100
"chr1" 247593001 247594000 "*" 0 0 100
"chr1" 247608001 247609000 "*" 3.93190702219215e-10 8.07594596899077e-10 -62.8318584070796
"chr1" 247875001 247876000 "*" 0 0 75.4716981132076
"chr1" 248018001 248019000 "*" 2.88657986402541e-15 1.14155613124573e-14 73.6842105263158
"chr1" 248040001 248041000 "*" 3.95353005888666e-09 7.03928529283733e-09 100
"chr1" 248247001 248248000 "*" 0 0 70.1492537313433
"chr1" 248685001 248686000 "*" 3.25622750985133e-10 6.76191460054273e-10 60.509337860781
"chr1" 248789001 248790000 "*" 1.39779633423487e-07 1.98463037958589e-07 100
"chr1" 248902001 248903000 "*" 1.06363524721154e-06 1.34530286878699e-06 -52.1739130434783
"chr1" 249077001 249078000 "*" 2.43005615629954e-09 4.45014222280278e-09 -100
"chr1" 249131001 249132000 "*" 1.18804928228577e-07 1.70575925561201e-07 70.5882352941177
"chr1" 249135001 249136000 "*" 8.7349629751543e-09 1.47505871898511e-08 -100
"chr1" 249150001 249151000 "*" 7.67056418382595e-11 1.7359448580318e-10 100
"chr10" 94001 95000 "*" 0 0 89.4366197183099
"chr10" 122001 123000 "*" 0 0 73.8080622058523
"chr10" 337001 338000 "*" 0 0 -100
"chr10" 344001 345000 "*" 9.5812247025151e-14 3.15667542866807e-13 -100
"chr10" 458001 459000 "*" 2.04124910127002e-05 2.13316623522606e-05 59.1836734693878
"chr10" 460001 461000 "*" 3.98653221544976e-10 8.18209832067575e-10 -56
"chr10" 517001 518000 "*" 3.58120389308825e-05 3.60126966183746e-05 61.5384615384615
"chr10" 537001 538000 "*" 3.33066907387547e-16 1.4495649018245e-15 68.75
"chr10" 541001 542000 "*" 4.71134242729931e-12 1.257615036806e-11 -100
"chr10" 544001 545000 "*" 7.0006535457523e-08 1.03695832512054e-07 -100
"chr10" 565001 566000 "*" 0 0 70.2702702702703
"chr10" 580001 581000 "*" 1.13140401492018e-08 1.87801380787728e-08 -100
"chr10" 648001 649000 "*" 0 0 -63.3333333333333
"chr10" 649001 650000 "*" 2.22044604925031e-16 9.81641919380259e-16 -100
"chr10" 744001 745000 "*" 0 0 -100
"chr10" 746001 747000 "*" 0 0 -100
"chr10" 773001 774000 "*" 1.09686691580624e-05 1.19451401973408e-05 66.6666666666667
"chr10" 774001 775000 "*" 0 0 -69.6969696969697
"chr10" 782001 783000 "*" 7.29824578549199e-09 1.24913557947419e-08

-90.4761904761905
"chr10" 862001 863000 "*" 0 0 -100
"chr10" 908001 909000 "*" 4.01313771103418e-09 7.11882087237587e-09 100
"chr10" 1009001 1010000 "*" 1.4432899320127e-15 5.90750815956055e-15 -100
"chr10" 1213001 1214000 "*" 1.48766443608395e-10 3.23475468835207e-10
-59.5588235294118
"chr10" 1224001 1225000 "*" 4.73234496034536e-09 8.30627300264826e-09 -100
"chr10" 1258001 1259000 "*" 2.22044604925031e-16 9.81641919380259e-16
-58.8854648176682
"chr10" 1278001 1279000 "*" 1.16235909786155e-11 2.94324191306803e-11
-51.3157894736842
"chr10" 1289001 1290000 "*" 7.9984455014781e-06 8.89173797399106e-06
-66.6666666666667
"chr10" 1357001 1358000 "*" 1.77635683940025e-15 7.19870740878856e-15
91.82156133829
"chr10" 1383001 1384000 "*" 1.19943740162221e-07 1.72145784317353e-07
-51.3636363636364
"chr10" 1390001 1391000 "*" 1.78474365808423e-06 2.18760273061449e-06
-53.2627865961199
"chr10" 1449001 1450000 "*" 3.47943895917524e-13 1.07191058753964e-12
-83.8709677419355
"chr10" 1455001 1456000 "*" 5.57964785485865e-12 1.47649370162352e-11
-83.3333333333333
"chr10" 1462001 1463000 "*" 1.67299207820548e-08 2.71377429441055e-08 100
"chr10" 1465001 1466000 "*" 0 0 -74.390243902439
"chr10" 1492001 1493000 "*" 5.06261699229071e-14 1.7297955084095e-13
62.3655913978495
"chr10" 1516001 1517000 "*" 0 0 59.700332963374
"chr10" 1519001 1520000 "*" 1.22711657357666e-06 1.53909884200857e-06
61.8421052631579
"chr10" 1551001 1552000 "*" 2.47745934878196e-10 5.23491079290299e-10 -75
"chr10" 1558001 1559000 "*" 5.50161563824814e-05 5.37369801477148e-05
-61.9047619047619
"chr10" 1573001 1574000 "*" 0 0 54.9988534739739
"chr10" 1591001 1592000 "*" 0 0 -98.5294117647059
"chr10" 1596001 1597000 "*" 0 0 -100
"chr10" 1607001 1608000 "*" 2.03161960921072e-05 2.12374183247922e-05
58.8235294117647
"chr10" 1616001 1617000 "*" 1.84245397827354e-08 2.97264462436361e-08
53.3333333333333
"chr10" 1621001 1622000 "*" 7.27607307737799e-10 1.436215009599e-09
65.9340659340659
"chr10" 1637001 1638000 "*" 7.48531236993699e-11 1.69844452249602e-10
67.9245283018868
"chr10" 1653001 1654000 "*" 0 0 100
"chr10" 1654001 1655000 "*" 2.46300493667029e-07 3.39295577215992e-07
73.0769230769231
"chr10" 1666001 1667000 "*" 2.30924404043265e-07 3.19221246511306e-07
-70.1492537313433
"chr10" 1696001 1697000 "*" 3.14340396423418e-06 3.71439622574897e-06
-67.1052631578947
"chr10" 1702001 1703000 "*" 3.53400475938681e-06 4.14231950904509e-06 56

"chr10" 1704001 1705000 "*" 1.0708696929207e-08 1.78887310726498e-08
66.6666666666667
"chr10" 1741001 1742000 "*" 2.17381668221606e-13 6.85151076607736e-13 100
"chr10" 1743001 1744000 "*" 2.08814465718632e-09 3.86232567960463e-09 100
"chr10" 1744001 1745000 "*" 1.19904086659517e-14 4.40901388691796e-14
70.3256302521008
"chr10" 1756001 1757000 "*" 1.4432899320127e-15 5.90750815956055e-15 100
"chr10" 1769001 1770000 "*" 3.73034936274053e-14 1.29420415488591e-13 100
"chr10" 1774001 1775000 "*" 1.23651455297136e-07 1.77204761555056e-07
60.377358490566
"chr10" 1822001 1823000 "*" 7.98733127916895e-05 7.60661142575564e-05
52.247191011236
"chr10" 1841001 1842000 "*" 8.61712678990045e-09 1.46086889771042e-08 76
"chr10" 1932001 1933000 "*" 0 0 -100
"chr10" 2053001 2054000 "*" 8.99691432465488e-12 2.31460585246735e-11
58.8785046728972
"chr10" 2054001 2055000 "*" 1.11022302462516e-16 5.03662826618488e-16 -100
"chr10" 2057001 2058000 "*" 4.0497698194919e-08 6.21178940857535e-08
-50.3030303030303
"chr10" 2069001 2070000 "*" 2.38229547200319e-10 5.04200128794648e-10
-66.6666666666667
"chr10" 2220001 2221000 "*" 1.09124644542824e-07 1.57567025821363e-07
-57.7777777777778
"chr10" 2332001 2333000 "*" 0 0 -100
"chr10" 2358001 2359000 "*" 2.91028312560115e-11 6.97590507139978e-11 -100
"chr10" 2374001 2375000 "*" 0 0 -100
"chr10" 2400001 2401000 "*" 1.11022302462516e-16 5.03662826618488e-16 100
"chr10" 2415001 2416000 "*" 9.43986026791244e-07 1.20230128224618e-06
72.1311475409836
"chr10" 2424001 2425000 "*" 9.08108033215171e-11 2.03407753381119e-10 -100
"chr10" 2433001 2434000 "*" 5.04263297784746e-13 1.52078941532818e-12 100
"chr10" 2537001 2538000 "*" 7.0006535457523e-08 1.03695832512054e-07 100
"chr10" 2565001 2566000 "*" 0 0 63.1867514549581
"chr10" 2634001 2635000 "*" 1.99177341286827e-11 4.87470407720531e-11
53.7313432835821
"chr10" 2722001 2723000 "*" 3.52704532247117e-11 8.35581292290093e-11 -100
"chr10" 2777001 2778000 "*" 4.17560012344609e-07 5.57616064179712e-07 -81.25
"chr10" 2808001 2809000 "*" 0 0 -100
"chr10" 2881001 2882000 "*" 2.93627245850914e-07 4.00384496399109e-07
61.5384615384615
"chr10" 2908001 2909000 "*" 5.05555486363107e-10 1.02234808980014e-09
85.7142857142857
"chr10" 2909001 2910000 "*" 1.5277158427196e-09 2.87642564232045e-09 -100
"chr10" 2937001 2938000 "*" 6.66133814775094e-16 2.81595744474255e-15 100
"chr10" 2964001 2965000 "*" 5.55111512312578e-16 2.36485094870365e-15
74.1573033707865
"chr10" 2980001 2981000 "*" 5.98410210272959e-14 2.02227920947152e-13
78.9473684210526
"chr10" 3027001 3028000 "*" 6.31709229370614e-09 1.09250559911453e-08
55.1282051282051
"chr10" 3079001 3080000 "*" 1.14124265593318e-11 2.89138062669327e-11 100
"chr10" 3115001 3116000 "*" 5.35520516820043e-10 1.08035107045551e-09

52.3809523809524
"chr10" 3189001 3190000 "*" 1.11022302462516e-16 5.03662826618488e-16
-53.4883720930233
"chr10" 3220001 3221000 "*" 0 0 -100
"chr10" 3244001 3245000 "*" 1.88737914186277e-15 7.62011598380321e-15 100
"chr10" 3255001 3256000 "*" 1.99634753172973e-10 4.28254476927807e-10
64.0243902439024
"chr10" 3282001 3283000 "*" 7.65054686269195e-13 2.25814319276556e-12
-58.4415584415584
"chr10" 3318001 3319000 "*" 1.42990798535081e-07 2.02801378444991e-07 80
"chr10" 3387001 3388000 "*" 1.22124532708767e-15 5.03921984217778e-15
-83.3333333333333
"chr10" 3396001 3397000 "*" 1.48735728389138e-06 1.84447733364305e-06
73.1707317073171
"chr10" 3422001 3423000 "*" 0 0 100
"chr10" 3456001 3457000 "*" 3.93324872671741e-09 7.02161253526996e-09
-66.2650602409639
"chr10" 3485001 3486000 "*" 5.01025332333427e-10 1.01358031135889e-09 -100
"chr10" 3521001 3522000 "*" 0 0 100
"chr10" 3563001 3564000 "*" 5.87852363453933e-08 8.83677237745202e-08
84.6153846153846
"chr10" 3582001 3583000 "*" 3.94995692043487e-07 5.29118471192652e-07
-73.6842105263158
"chr10" 3594001 3595000 "*" 0 0 67.5324675324675
"chr10" 3631001 3632000 "*" 1.67299207820548e-08 2.71377429441055e-08 100
"chr10" 3692001 3693000 "*" 9.08108033215171e-11 2.03407753381119e-10 100
"chr10" 3704001 3705000 "*" 7.0006535457523e-08 1.03695832512054e-07 -100
"chr10" 3720001 3721000 "*" 4.01313771103418e-09 7.11882087237587e-09 -100
"chr10" 3758001 3759000 "*" 2.37587727269783e-14 8.44268514944447e-14 -100
"chr10" 3769001 3770000 "*" 1.16573417585641e-14 4.29335582900508e-14 -100
"chr10" 3783001 3784000 "*" 0 0 100
"chr10" 3805001 3806000 "*" 0 0 -100
"chr10" 3829001 3830000 "*" 2.08977279925193e-12 5.83255832828878e-12
-52.7272727272727
"chr10" 3876001 3877000 "*" 2.08814465718632e-09 3.86232567960463e-09 100
"chr10" 3957001 3958000 "*" 2.15125472990962e-10 4.58126659943874e-10 100
"chr10" 4011001 4012000 "*" 2.64951482975562e-09 4.82186011375752e-09 100
"chr10" 4034001 4035000 "*" 7.9443175371452e-09 1.35415635126409e-08 -90
"chr10" 4109001 4110000 "*" 3.34128893442198e-08 5.19772864618523e-08 100
"chr10" 5377001 5378000 "*" 1.54967431331698e-06 1.91685454663215e-06
71.4285714285714
"chr10" 5544001 5545000 "*" 1.98729921407903e-14 7.12685498544555e-14
-98.3870967741936
"chr10" 5593001 5594000 "*" 0.000525299926963374 0.000436849389802675
-54.5454545454545
"chr10" 5604001 5605000 "*" 1.41019542931886e-06 1.75486844532563e-06
52.1739130434783
"chr10" 5665001 5666000 "*" 6.66133814775094e-16 2.81595744474255e-15 100
"chr10" 5718001 5719000 "*" 1.06581410364015e-14 3.94854473541761e-14
-66.6666666666667
"chr10" 6018001 6019000 "*" 1.37667655053519e-14 5.0256405417348e-14
-66.2732919254658

"chr10" 6042001 6043000 "*" 7.63213825472064e-10 1.50308314398032e-09 87.0588235294118
"chr10" 6079001 6080000 "*" 2.15125472990962e-10 4.58126659943874e-10 100
"chr10" 6178001 6179000 "*" 2.15125472990962e-10 4.58126659943874e-10 100
"chr10" 6179001 6180000 "*" 4.01313771103418e-09 7.11882087237587e-09 100
"chr10" 6244001 6245000 "*" 0 0 -61.8181818181818
"chr10" 6466001 6467000 "*" 1.72624336913785e-06 2.12026008255153e-06 72.6027397260274
"chr10" 6469001 6470000 "*" 5.6621374255883e-15 2.16468580166064e-14 66.6666666666667
"chr10" 6536001 6537000 "*" 5.26220583330073e-07 6.93615972208185e-07 55
"chr10" 6882001 6883000 "*" 1.38791188838416e-05 1.4877107846787e-05 -65
"chr10" 6942001 6943000 "*" 0 0 -100
"chr10" 6960001 6961000 "*" 9.59247692878806e-05 9.01856948106891e-05 63.6842105263158
"chr10" 7085001 7086000 "*" 9.99200722162641e-16 4.15382497462808e-15 -100
"chr10" 7128001 7129000 "*" 8.57092175010621e-13 2.51149198099779e-12 100
"chr10" 7139001 7140000 "*" 0 0 -64.0756302521008
"chr10" 7231001 7232000 "*" 2.59686716574947e-09 4.74167027675863e-09 -83.5820895522388
"chr10" 7258001 7259000 "*" 0 0 -76.3636363636364
"chr10" 7384001 7385000 "*" 1.82354689659547e-06 2.23185187228314e-06 57.6298701298701
"chr10" 7400001 7401000 "*" 2.00227675550835e-08 3.20459176617961e-08 100
"chr10" 7405001 7406000 "*" 1.24846286975178e-07 1.78815836572989e-07 93.75
"chr10" 7484001 7485000 "*" 8.90920670570949e-11 2.00165418146943e-10 68.9655172413793
"chr10" 7495001 7496000 "*" 4.11539381706127e-06 4.77326970666537e-06 -66.6666666666667
"chr10" 7539001 7540000 "*" 0 0 -96.2962962962963
"chr10" 7550001 7551000 "*" 3.33066907387547e-16 1.4495649018245e-15 100
"chr10" 7564001 7565000 "*" 1.30784272300843e-13 4.24053318659706e-13 -84
"chr10" 7573001 7574000 "*" 5.32907051820075e-15 2.04626240984625e-14 -71.4717741935484
"chr10" 7603001 7604000 "*" 6.44564605500886e-06 7.26663863268796e-06 66.6666666666667
"chr10" 7608001 7609000 "*" 1.11022302462516e-16 5.03662826618488e-16 -56.9444444444444
"chr10" 7631001 7632000 "*" 0 0 -100
"chr10" 7657001 7658000 "*" 0 0 100
"chr10" 7678001 7679000 "*" 3.89583920679115e-11 9.17840974938567e-11 -66.6666666666667
"chr10" 7702001 7703000 "*" 0 0 76.7123287671233
"chr10" 7754001 7755000 "*" 6.37490060739765e-13 1.89496793587877e-12 -100
"chr10" 7763001 7764000 "*" 8.37752089921651e-11 1.88922989503423e-10 -50.8771929824561
"chr10" 7979001 7980000 "*" 1.38590361409285e-10 3.02706178892116e-10 100
"chr10" 8086001 8087000 "*" 0 0 -80.8988764044944
"chr10" 8088001 8089000 "*" 8.72758839535193e-05 8.25802338287769e-05 -60
"chr10" 8106001 8107000 "*" 0.000108193472728613 0.000100892381514582 -50.8196721311475
"chr10" 8123001 8124000 "*" 6.24654217240561e-10 1.2462108191303e-09 -100

"chr10" 8306001 8307000 "*" 5.13960336512298e-08 7.78013436426274e-08
-74.7663551401869
"chr10" 8368001 8369000 "*" 0 0 87.1794871794872
"chr10" 8373001 8374000 "*" 0 0 100
"chr10" 8374001 8375000 "*" 0 0 82.8165374677003
"chr10" 8499001 8500000 "*" 0 0 100
"chr10" 8500001 8501000 "*" 9.67004254448511e-14 3.18178990782609e-13 100
"chr10" 9086001 9087000 "*" 9.08108033215171e-11 2.03407753381119e-10 100
"chr10" 9183001 9184000 "*" 0 0 100
"chr10" 9645001 9646000 "*" 6.52733422867868e-12 1.71146951718423e-11 -100
"chr10" 10056001 10057000 "*" 0 0 100
"chr10" 10202001 10203000 "*" 6.44564627116928e-06 7.26663863268796e-06
-66.6666666666667
"chr10" 10336001 10337000 "*" 9.65818728407841e-05 9.07635808484797e-05
61.8181818181818
"chr10" 10337001 10338000 "*" 4.18792667211676e-10 8.57350458559416e-10
-52.3976023976024
"chr10" 10808001 10809000 "*" 1.67299207820548e-08 2.71377429441055e-08 -100
"chr10" 10959001 10960000 "*" 1.52466927971773e-12 4.33322475981408e-12 -100
"chr10" 11140001 11141000 "*" 7.0006535457523e-08 1.03695832512054e-07 100
"chr10" 11186001 11187000 "*" 0 0 73.5849056603774
"chr10" 11299001 11300000 "*" 3.34128893442198e-08 5.19772864618523e-08 -100
"chr10" 11303001 11304000 "*" 0 0 100
"chr10" 11321001 11322000 "*" 8.64488297613875e-08 1.26480485233459e-07
-63.1578947368421
"chr10" 11348001 11349000 "*" 1.52794177310511e-09 2.87642564232045e-09 100
"chr10" 11370001 11371000 "*" 1.11022302462516e-16 5.03662826618488e-16 100
"chr10" 11409001 11410000 "*" 3.47856766724775e-07 4.6959366769944e-07 60
"chr10" 11418001 11419000 "*" 3.02451547251614e-06 3.58238817268797e-06
66.0377358490566
"chr10" 11423001 11424000 "*" 1.12458486967171e-09 2.15740815043925e-09 -100
"chr10" 11431001 11432000 "*" 4.66562411149951e-07 6.18997949470075e-07
53.968253968254
"chr10" 11852001 11853000 "*" 1.11022302462516e-16 5.03662826618488e-16
50.6493506493507
"chr10" 11865001 11866000 "*" 0 0 77.7124532335649
"chr10" 11917001 11918000 "*" 1.21731014157689e-09 2.32630297043657e-09
60.8695652173913
"chr10" 11963001 11964000 "*" 1.35036426485158e-12 3.86629547363774e-12 100
"chr10" 12111001 12112000 "*" 4.73234496034536e-09 8.30627300264826e-09 -100
"chr10" 12349001 12350000 "*" 1.22707838001723e-07 1.75940305659382e-07
58.3333333333333
"chr10" 12438001 12439000 "*" 6.21724893790088e-15 2.36606385879553e-14
54.8387096774194
"chr10" 12592001 12593000 "*" 1.1340706485008e-08 1.88212211206977e-08
-90.9090909090909
"chr10" 12599001 12600000 "*" 1.11022302462516e-16 5.03662826618488e-16 -100
"chr10" 12613001 12614000 "*" 0 0 -100
"chr10" 12701001 12702000 "*" 2.61701660342339e-10 5.51665677653371e-10
-66.0714285714286
"chr10" 12755001 12756000 "*" 8.7349629751543e-09 1.47505871898511e-08 100
"chr10" 12881001 12882000 "*" 5.07371922253697e-14 1.73287898516782e-13 100

"chr10" 12943001 12944000 "*" 1.11022302462516e-16 5.03662826618488e-16
-69.4117647058823
"chr10" 12951001 12952000 "*" 7.105427357601e-15 2.68362283258525e-14 -100
"chr10" 12958001 12959000 "*" 2.33028818463765e-10 4.93719283454501e-10 100
"chr10" 12977001 12978000 "*" 6.01563132640592e-10 1.20460958326729e-09
-50.0825082508251
"chr10" 13083001 13084000 "*" 2.15125472990962e-10 4.58126659943874e-10 -100
"chr10" 13097001 13098000 "*" 3.65130148338721e-12 9.90602415539447e-12 100
"chr10" 13136001 13137000 "*" 7.0006535457523e-08 1.03695832512054e-07 -100
"chr10" 13305001 13306000 "*" 5.57729418204644e-11 1.28800526662156e-10 56.25
"chr10" 13313001 13314000 "*" 6.10622663543836e-15 2.32593540648772e-14 75
"chr10" 13327001 13328000 "*" 1.08747971072631e-07 1.57061209114014e-07
82.9787234042553
"chr10" 13592001 13593000 "*" 4.26951363152739e-10 8.72510873516957e-10 -100
"chr10" 13624001 13625000 "*" 4.78841410966879e-11 1.11468187156588e-10 100
"chr10" 13741001 13742000 "*" 0 0 89.3939393939394
"chr10" 13745001 13746000 "*" 4.02167188440217e-12 1.08266700497926e-11 100
"chr10" 13772001 13773000 "*" 0.000106420833506649 9.9350448716649e-05
72.7272727272727
"chr10" 13775001 13776000 "*" 0 0 -77.4193548387097
"chr10" 13993001 13994000 "*" 1.99747852374088e-05 2.09001732651667e-05
65.1360544217687
"chr10" 14013001 14014000 "*" 8.19011525265978e-13 2.40989740450878e-12
-68.4265734265734
"chr10" 14016001 14017000 "*" 2.1658988651474e-06 2.62321901854342e-06
66.6666666666667
"chr10" 14051001 14052000 "*" 1.25284005392245e-10 2.75649590272477e-10 -100
"chr10" 14131001 14132000 "*" 0 0 70
"chr10" 14134001 14135000 "*" 0 0 -100
"chr10" 14194001 14195000 "*" 2.1094237467878e-15 8.47434540879246e-15 100
"chr10" 14195001 14196000 "*" 0 0 100
"chr10" 14278001 14279000 "*" 0.000122715633738557 0.000113456589750413
54.1666666666667
"chr10" 14352001 14353000 "*" 6.21724893790088e-15 2.36606385879553e-14
-96.1538461538462
"chr10" 14413001 14414000 "*" 3.40125705378114e-10 7.03784073980162e-10 -100
"chr10" 14414001 14415000 "*" 8.25450818808804e-13 2.42473843346228e-12 -100
"chr10" 14525001 14526000 "*" 1.64457336637724e-12 4.65338642828372e-12
-69.2307692307692
"chr10" 14529001 14530000 "*" 3.6700841921089e-08 5.65530226072258e-08 100
"chr10" 14541001 14542000 "*" 8.26057622305143e-09 1.40281772795055e-08
-61.5384615384615
"chr10" 14627001 14628000 "*" 3.21304205463946e-10 6.67824922105025e-10 -80
"chr10" 14679001 14680000 "*" 1.13140401492018e-08 1.87801380787728e-08 -100
"chr10" 14800001 14801000 "*" 1.36604061395929e-11 3.42333952202875e-11 100
"chr10" 14818001 14819000 "*" 1.46532003270083e-05 1.56543369171236e-05
62.962962962963
"chr10" 14876001 14877000 "*" 2.43005615629954e-09 4.45014222280278e-09 100
"chr10" 15055001 15056000 "*" 8.52140580320793e-12 2.19817259951489e-11 100
"chr10" 15130001 15131000 "*" 0 0 67.1687330492057
"chr10" 15154001 15155000 "*" 3.48211792733366e-10 7.19819214451117e-10
51.1627906976744

"chr10" 15178001 15179000 "*" 1.33122005641084e-08 2.191997641159e-08 -92.3076923076923
"chr10" 15233001 15234000 "*" 7.88258347483861e-15 2.96145301916012e-14 -100
"chr10" 15271001 15272000 "*" 3.94216216873522e-05 3.93770971245002e-05 73.3333333333333
"chr10" 15327001 15328000 "*" 5.01025332333427e-10 1.01358031135889e-09 -100
"chr10" 15334001 15335000 "*" 0 0 -100
"chr10" 15597001 15598000 "*" 0.000234548275865443 0.000207063434072029 -60
"chr10" 15604001 15605000 "*" 2.79447276430034e-09 5.07304969864573e-09 -50.2264492753623
"chr10" 16069001 16070000 "*" 0 0 66.6666666666667
"chr10" 16499001 16500000 "*" 0 0 100
"chr10" 16847001 16848000 "*" 0 0 -63.1578947368421
"chr10" 16954001 16955000 "*" 6.92287338566189e-11 1.57605630124247e-10 100
"chr10" 16993001 16994000 "*" 1.52794177310511e-09 2.87642564232045e-09 100
"chr10" 17028001 17029000 "*" 2.38142838782096e-12 6.61073050618058e-12 83.6363636363636
"chr10" 17156001 17157000 "*" 0 0 -64.8648648648649
"chr10" 17270001 17271000 "*" 0 0 52.2175710323897
"chr10" 17449001 17450000 "*" 1.29037891483108e-11 3.24582280286866e-11 -100
"chr10" 17467001 17468000 "*" 0.000792090690089564 0.000639295093468792 52.3809523809524
"chr10" 17495001 17496000 "*" 0 0 53.7537537537538
"chr10" 17496001 17497000 "*" 0 0 85.3464705589432
"chr10" 18277001 18278000 "*" 6.70841160399505e-12 1.75486545176836e-11 100
"chr10" 18410001 18411000 "*" 1.09618594947847e-05 1.19385035120913e-05 66.6666666666667
"chr10" 18671001 18672000 "*" 3.40125705378114e-10 7.03784073980162e-10 100
"chr10" 18690001 18691000 "*" 1.0325074129014e-14 3.83008663711937e-14 -100
"chr10" 20050001 20051000 "*" 3.90831811358794e-12 1.05610567215048e-11 100
"chr10" 20104001 20105000 "*" 0 0 70.205801328796
"chr10" 20105001 20106000 "*" 0 0 58.5506954243096
"chr10" 20106001 20107000 "*" 0 0 96.3235294117647
"chr10" 20251001 20252000 "*" 0 0 64.6258503401361
"chr10" 20392001 20393000 "*" 0 0 75
"chr10" 20413001 20414000 "*" 3.34128893442198e-08 5.19772864618523e-08 100
"chr10" 21378001 21379000 "*" 0 0 -100
"chr10" 21784001 21785000 "*" 4.44089209850063e-16 1.91071758245033e-15 67.7804295942721
"chr10" 21785001 21786000 "*" 0 0 -66.6666666666667
"chr10" 21805001 21806000 "*" 0 0 50.2824858757062
"chr10" 22018001 22019000 "*" 3.6700841921089e-08 5.65530226072258e-08 100
"chr10" 22118001 22119000 "*" 1.01030295240889e-14 3.75322807935163e-14 66.6666666666667
"chr10" 22302001 22303000 "*" 6.93112234273485e-13 2.05218842007357e-12 100
"chr10" 22497001 22498000 "*" 5.10702591327572e-15 1.96271840288234e-14 100
"chr10" 22569001 22570000 "*" 3.11222049242943e-08 4.86808376361123e-08 54.0540540540541
"chr10" 22771001 22772000 "*" 4.73234496034536e-09 8.30627300264826e-09 -100
"chr10" 22982001 22983000 "*" 2.69007038866675e-13 8.38831022628993e-13 100
"chr10" 23464001 23465000 "*" 4.79394379748754e-09 8.4097903736489e-09 77.5510204081633

"chr10" 23537001 23538000 "*" 1.39779633423487e-07 1.98463037958589e-07 -100
"chr10" 23547001 23548000 "*" 2.43005615629954e-09 4.45014222280278e-09 100
"chr10" 23551001 23552000 "*" 2.08814465718632e-09 3.86232567960463e-09 100
"chr10" 23729001 23730000 "*" 0 0 55.7788944723618
"chr10" 23998001 23999000 "*" 4.01313771103418e-09 7.11882087237587e-09 100
"chr10" 24521001 24522000 "*" 1.37809934086519e-06 1.71725945423483e-06
-66.6666666666667
"chr10" 24544001 24545000 "*" 3.56775498033812e-10 7.35898629809829e-10 -100
"chr10" 24696001 24697000 "*" 1.93409899207353e-09 3.60029218381527e-09
72.2222222222222
"chr10" 24805001 24806000 "*" 9.5812247025151e-14 3.15667542866807e-13 100
"chr10" 24845001 24846000 "*" 8.69011083648452e-07 1.11229659818976e-06
66.6666666666667
"chr10" 25124001 25125000 "*" 2.00227675550835e-08 3.20459176617961e-08 -100
"chr10" 25400001 25401000 "*" 2.64951482975562e-09 4.82186011375752e-09 100
"chr10" 25406001 25407000 "*" 1.5277158427196e-09 2.87642564232045e-09 100
"chr10" 25618001 25619000 "*" 3.77475828372553e-15 1.47379557022071e-14 100
"chr10" 26370001 26371000 "*" 1.52788892648914e-12 4.3419313534645e-12
-69.2307692307692
"chr10" 26538001 26539000 "*" 1.39779633423487e-07 1.98463037958589e-07 100
"chr10" 26566001 26567000 "*" 7.88258347483861e-15 2.96145301916012e-14 -100
"chr10" 26586001 26587000 "*" 2.4535928844216e-14 8.69144396197119e-14 -100
"chr10" 26662001 26663000 "*" 2.02327044007689e-12 5.65300384792621e-12 -100
"chr10" 26767001 26768000 "*" 0 0 100
"chr10" 26883001 26884000 "*" 8.55981951985996e-14 2.84329661419124e-13
-55.4396423248882
"chr10" 27224001 27225000 "*" 4.1696646135847e-12 1.1211326807107e-11
57.1428571428571
"chr10" 27530001 27531000 "*" 1.14463993838854e-13 3.73766761837652e-13
54.5454545454545
"chr10" 27608001 27609000 "*" 0 0 99.0551181102362
"chr10" 27609001 27610000 "*" 0 0 95.7746478873239
"chr10" 27648001 27649000 "*" 1.11022302462516e-16 5.03662826618488e-16 100
"chr10" 28000001 28001000 "*" 0 0 100
"chr10" 28092001 28093000 "*" 1.35890187991095e-11 3.40841971363279e-11 -100
"chr10" 28725001 28726000 "*" 1.35036426485158e-12 3.86629547363774e-12 100
"chr10" 28806001 28807000 "*" 6.66133814775094e-16 2.81595744474255e-15
85.7142857142857
"chr10" 28924001 28925000 "*" 1.28907528396116e-06 1.61243929530756e-06 65.625
"chr10" 28957001 28958000 "*" 0 0 82.6283931122641
"chr10" 29228001 29229000 "*" 2.89268317077429e-07 3.94750540055777e-07
-59.2592592592593
"chr10" 29262001 29263000 "*" 3.33066907387547e-16 1.4495649018245e-15
-51.4285714285714
"chr10" 29275001 29276000 "*" 4.18887147191072e-13 1.2739946038722e-12 100
"chr10" 29480001 29481000 "*" 0 0 -100
"chr10" 29581001 29582000 "*" 4.32986979603811e-15 1.68006959513174e-14 -100
"chr10" 29661001 29662000 "*" 4.93500784792822e-07 6.52819474468471e-07
-66.6666666666667
"chr10" 29670001 29671000 "*" 6.4152538836737e-10 1.27477697728417e-09 -100
"chr10" 29699001 29700000 "*" 7.67056418382595e-11 1.7359448580318e-10 -100
"chr10" 29773001 29774000 "*" 0 0 -66.6666666666667

"chr10" 29824001 29825000 "*" 5.55111512312578e-16 2.36485094870365e-15 -100
"chr10" 29918001 29919000 "*" 9.62896429257398e-13 2.80291205576417e-12 100
"chr10" 30210001 30211000 "*" 0 0 100
"chr10" 30245001 30246000 "*" 2.59712167327564e-08 4.09881271012004e-08 -65.625
"chr10" 30281001 30282000 "*" 6.59550958292954e-09 1.13456194382773e-08 100
"chr10" 30282001 30283000 "*" 1.14352971536391e-14 4.21921674335999e-14
-95.049504950495
"chr10" 30348001 30349000 "*" 0 0 83.8544990427569
"chr10" 30383001 30384000 "*" 1.13140401492018e-08 1.87801380787728e-08 -100
"chr10" 30500001 30501000 "*" 3.6700841921089e-08 5.65530226072258e-08 100
"chr10" 30555001 30556000 "*" 0 0 66.6666666666667
"chr10" 30574001 30575000 "*" 1.64716754680327e-06 2.02893190508151e-06
-54.1666666666667
"chr10" 30676001 30677000 "*" 0 0 -100
"chr10" 30867001 30868000 "*" 1.96509475358653e-13 6.23868565133654e-13 -100
"chr10" 30955001 30956000 "*" 1.53876911213047e-13 4.94754679930307e-13
81.8181818181818
"chr10" 30957001 30958000 "*" 3.66151553521377e-13 1.12588189950301e-12 75
"chr10" 30981001 30982000 "*" 7.7715611723761e-16 3.26213507634405e-15
84.4155844155844
"chr10" 30985001 30986000 "*" 1.40155109740192e-10 3.06019683121616e-10 73.4375
"chr10" 31014001 31015000 "*" 2.22044604925031e-16 9.81641919380259e-16
84.8484848484848
"chr10" 31043001 31044000 "*" 4.21884749357559e-15 1.63874445239189e-14 100
"chr10" 31057001 31058000 "*" 1.11022302462516e-16 5.03662826618488e-16 100
"chr10" 31107001 31108000 "*" 9.54752162884986e-08 1.38880257814482e-07
-57.9310344827586
"chr10" 31345001 31346000 "*" 2.77104695933783e-06 3.30186243034597e-06
-64.8514851485149
"chr10" 31390001 31391000 "*" 0 0 100
"chr10" 31391001 31392000 "*" 1.99240623999231e-12 5.58210390560774e-12
92.8571428571429
"chr10" 31557001 31558000 "*" 0 0 100
"chr10" 31907001 31908000 "*" 1.86821217281752e-05 1.96397758022194e-05 -60
"chr10" 31908001 31909000 "*" 0.000496254900866688 0.000414667937798419
-54.1666666666667
"chr10" 31909001 31910000 "*" 6.88338275267597e-15 2.60518659957509e-14 -100
"chr10" 31922001 31923000 "*" 4.08209022140227e-11 9.57770690255255e-11 100
"chr10" 31938001 31939000 "*" 9.1649421385398e-10 1.7861423045262e-09
63.9344262295082
"chr10" 32048001 32049000 "*" 0 0 -52.3809523809524
"chr10" 32154001 32155000 "*" 0 0 -100
"chr10" 32293001 32294000 "*" 2.26492380372978e-06 2.73465398211104e-06
-54.8235294117647
"chr10" 32427001 32428000 "*" 1.52069024039747e-10 3.30269070920457e-10
-65.7509157509158
"chr10" 32450001 32451000 "*" 1.98365768255826e-11 4.85612402473442e-11 100
"chr10" 32467001 32468000 "*" 0.000183887360524659 0.000165164039707606
58.3333333333333
"chr10" 32735001 32736000 "*" 1.4432899320127e-15 5.90750815956055e-15 -100
"chr10" 33294001 33295000 "*" 0 0 -100
"chr10" 33346001 33347000 "*" 4.18887147191072e-13 1.2739946038722e-12 100

"chr10" 33428001 33429000 "*" 5.44009282066327e-15 2.08486137681812e-14 -100
"chr10" 33472001 33473000 "*" 1.98124849859482e-10 4.25157611354621e-10 100
"chr10" 33515001 33516000 "*" 0 0 100
"chr10" 33626001 33627000 "*" 0 0 74.6268656716418
"chr10" 33752001 33753000 "*" 3.52704532247117e-11 8.35581292290093e-11 -100
"chr10" 34348001 34349000 "*" 7.78377362564697e-13 2.29551678017433e-12 -100
"chr10" 34399001 34400000 "*" 9.0161211829809e-13 2.63398763704587e-12
-51.7241379310345
"chr10" 34943001 34944000 "*" 3.9322656242291e-11 9.26135081558519e-11
-79.3650793650794
"chr10" 35058001 35059000 "*" 2.00227675550835e-08 3.20459176617961e-08 100
"chr10" 35240001 35241000 "*" 2.1094237467878e-15 8.47434540879246e-15 100
"chr10" 35247001 35248000 "*" 1.88737914186277e-15 7.62011598380321e-15 100
"chr10" 35267001 35268000 "*" 0 0 80.7017543859649
"chr10" 35508001 35509000 "*" 4.44089209850063e-16 1.91071758245033e-15 100
"chr10" 35511001 35512000 "*" 1.98365768255826e-11 4.85612402473442e-11 100
"chr10" 35596001 35597000 "*" 1.26565424807268e-14 4.63923203146481e-14 -100
"chr10" 35599001 35600000 "*" 8.1845506383349e-07 1.05155704962166e-06
-66.6666666666667
"chr10" 35839001 35840000 "*" 3.10228708277904e-08 4.85318324024346e-08
-63.1578947368421
"chr10" 35871001 35872000 "*" 1.41109876605761e-06 1.75583535616452e-06 -90
"chr10" 35875001 35876000 "*" 2.05391259555654e-14 7.35435952190685e-14
51.8072289156626
"chr10" 35887001 35888000 "*" 3.04324343503026e-11 7.26599935673121e-11 -100
"chr10" 35930001 35931000 "*" 0 0 61.4945511157239
"chr10" 35990001 35991000 "*" 4.41424674590962e-12 1.18308135228832e-11
-53.6566134437217
"chr10" 36034001 36035000 "*" 8.7349629751543e-09 1.47505871898511e-08 -100
"chr10" 36173001 36174000 "*" 8.25450818808804e-13 2.42473843346228e-12 -100
"chr10" 36771001 36772000 "*" 0 0 96.9135802469136
"chr10" 36966001 36967000 "*" 0 0 100
"chr10" 37232001 37233000 "*" 0 0 58.9041095890411
"chr10" 38105001 38106000 "*" 1.38590361409285e-10 3.02706178892116e-10 100
"chr10" 38633001 38634000 "*" 2.55351295663786e-15 1.01678090381833e-14 100
"chr10" 38804001 38805000 "*" 1.60986557418141e-10 3.48828035630686e-10
52.6315789473684
"chr10" 38883001 38884000 "*" 2.96958361234445e-08 4.65720195903114e-08
-57.0833333333333
"chr10" 38885001 38886000 "*" 7.87098608512338e-10 1.54771001400027e-09
-55.2380952380952
"chr10" 39022001 39023000 "*" 2.84548945517216e-08 4.47293055981002e-08
66.3157894736842
"chr10" 39034001 39035000 "*" 1.91224813761437e-12 5.36527594469447e-12 100
"chr10" 39110001 39111000 "*" 9.88421305481157e-06 1.0841293662501e-05
64.7058823529412
"chr10" 39137001 39138000 "*" 0 0 100
"chr10" 39140001 39141000 "*" 4.78403465731247e-08 7.27476615882735e-08
61.5384615384615
"chr10" 39149001 39150000 "*" 2.79310120476772e-07 3.82080086577431e-07
67.0707070707071
"chr10" 39150001 39151000 "*" 7.7715611723761e-16 3.26213507634405e-15

-81.438127090301
"chr10" 39152001 39153000 "*" 2.62900812231237e-13 8.21294938531841e-13 100
"chr10" 42355001 42356000 "*" 9.71063229826541e-11 2.16781365050398e-10 100
"chr10" 42359001 42360000 "*" 1.67299207820548e-08 2.71377429441055e-08 100
"chr10" 42360001 42361000 "*" 0 0 -76.6666666666667
"chr10" 42371001 42372000 "*" 2.88710388929303e-10 6.03399264731139e-10 100
"chr10" 42378001 42379000 "*" 4.80401245472883e-06 5.52167124178951e-06 68
"chr10" 42532001 42533000 "*" 1.72575644663553e-07 2.42390896527982e-07 80
"chr10" 43248001 43249000 "*" 0 0 84.4884488448845
"chr10" 43249001 43250000 "*" 0 0 87.1439186246125
"chr10" 43271001 43272000 "*" 0 0 -100
"chr10" 43276001 43277000 "*" 6.46149800331841e-14 2.17664365839349e-13 -100
"chr10" 43368001 43369000 "*" 1.79856129989275e-14 6.48457833104701e-14
-50.4950495049505
"chr10" 43369001 43370000 "*" 2.34257058195908e-14 8.33889706074077e-14
58.1395348837209
"chr10" 43376001 43377000 "*" 8.52111456062232e-07 1.09183873929303e-06
72.972972972973
"chr10" 43381001 43382000 "*" 2.33028818463765e-10 4.93719283454501e-10 100
"chr10" 43392001 43393000 "*" 7.5585368286557e-07 9.75771469042378e-07
76.9230769230769
"chr10" 43431001 43432000 "*" 0 0 100
"chr10" 43437001 43438000 "*" 0 0 55.6962025316456
"chr10" 43457001 43458000 "*" 1.21448693324311e-08 2.00882663704464e-08
-79.2682926829268
"chr10" 43476001 43477000 "*" 0 0 75.3086419753086
"chr10" 43481001 43482000 "*" 0.000128040987281364 0.000118003288696945 60
"chr10" 43483001 43484000 "*" 2.52316539550712e-07 3.4718739594317e-07
78.7878787878788
"chr10" 43495001 43496000 "*" 0 0 100
"chr10" 43532001 43533000 "*" 1.18804934778893e-07 1.70575925561201e-07
61.5384615384615
"chr10" 43553001 43554000 "*" 2.8622213765761e-07 3.90913383587999e-07
74.1176470588235
"chr10" 43559001 43560000 "*" 5.48638023900594e-10 1.10374357336421e-09 -100
"chr10" 43563001 43564000 "*" 0 0 100
"chr10" 43591001 43592000 "*" 7.27063789185411e-06 8.13589335437496e-06
50.5376344086022
"chr10" 43633001 43634000 "*" 0 0 -62.0901320901321
"chr10" 43690001 43691000 "*" 2.08814465718632e-09 3.86232567960463e-09 -100
"chr10" 43703001 43704000 "*" 2.1094237467878e-15 8.47434540879246e-15 100
"chr10" 43704001 43705000 "*" 5.39395195175985e-11 1.24735713904846e-10
72.7272727272727
"chr10" 43713001 43714000 "*" 0 0 79.3814432989691
"chr10" 43730001 43731000 "*" 8.99280649946377e-15 3.35888902220833e-14
66.456043956044
"chr10" 43757001 43758000 "*" 7.03992419914812e-12 1.83313429961219e-11 100
"chr10" 43759001 43760000 "*" 0 0 -53.3333333333333
"chr10" 43761001 43762000 "*" 0 0 69.2184795633071
"chr10" 43762001 43763000 "*" 0 0 68.7388248394358
"chr10" 43789001 43790000 "*" 5.966253158185e-09 1.03520018243344e-08
68.0555555555556

"chr10" 43794001 43795000 "*" 7.65188132856309e-08 1.12691547930703e-07
-94.1176470588235
"chr10" 43807001 43808000 "*" 9.67004254448511e-14 3.18178990782609e-13 100
"chr10" 43811001 43812000 "*" 0 0 100
"chr10" 43855001 43856000 "*" 6.4152538836737e-10 1.27477697728417e-09 -100
"chr10" 43856001 43857000 "*" 6.24654217240561e-10 1.2462108191303e-09 100
"chr10" 43867001 43868000 "*" 1.11244347067441e-13 3.64094501191563e-13
-66.6666666666667
"chr10" 43892001 43893000 "*" 0 0 -81.2744796387616
"chr10" 43916001 43917000 "*" 0 0 50.5628150540792
"chr10" 44070001 44071000 "*" 0 0 83.0194681257157
"chr10" 44101001 44102000 "*" 0 0 64.7435897435897
"chr10" 44104001 44105000 "*" 2.88710388929303e-10 6.03399264731139e-10 -100
"chr10" 44150001 44151000 "*" 2.15125472990962e-10 4.58126659943874e-10 -100
"chr10" 44174001 44175000 "*" 0 0 91.6666666666667
"chr10" 44201001 44202000 "*" 2.23999819803922e-08 3.56270211734721e-08
-59.8214285714286
"chr10" 44249001 44250000 "*" 1.2990497566534e-11 3.26684522684866e-11
67.741935483871
"chr10" 44303001 44304000 "*" 8.88178419700125e-16 3.70670032207938e-15 100
"chr10" 44322001 44323000 "*" 5.70765656959793e-13 1.70935986773964e-12
-64.2105263157895
"chr10" 44363001 44364000 "*" 4.00709354586581e-10 8.22085984817432e-10 -68.75
"chr10" 44366001 44367000 "*" 5.29811194915908e-10 1.06932440148915e-09
-63.7254901960784
"chr10" 44438001 44439000 "*" 3.98556572300812e-07 5.33562809588447e-07 80
"chr10" 44680001 44681000 "*" 3.06412817341339e-09 5.5384674208873e-09
-86.734693877551
"chr10" 44738001 44739000 "*" 1.22124532708767e-15 5.03921984217778e-15 100
"chr10" 44757001 44758000 "*" 9.35527675438586e-06 1.03009706123167e-05
-51.3513513513514
"chr10" 44763001 44764000 "*" 1.82790775371533e-06 2.23689371780977e-06
64.4067796610169
"chr10" 44791001 44792000 "*" 2.83731782513286e-09 5.14646116008318e-09
61.9047619047619
"chr10" 44852001 44853000 "*" 2.06183481743505e-06 2.50456553949242e-06
-65.3846153846154
"chr10" 44878001 44879000 "*" 0 0 -76.3636363636364
"chr10" 44879001 44880000 "*" 0 0 71.7409213552725
"chr10" 44918001 44919000 "*" 6.55031584528842e-15 2.48816226484197e-14
-97.6190476190476
"chr10" 45050001 45051000 "*" 5.51780843238703e-14 1.87243792572872e-13 100
"chr10" 45270001 45271000 "*" 3.03090885722668e-14 1.06210046699299e-13 -100
"chr10" 45314001 45315000 "*" 1.07223607770379e-09 2.06601137132775e-09
-57.7777777777778
"chr10" 45372001 45373000 "*" 1.44519793354725e-07 2.04841726501585e-07
53.8461538461538
"chr10" 45404001 45405000 "*" 1.39779633423487e-07 1.98463037958589e-07 -100
"chr10" 45420001 45421000 "*" 5.54235557448379e-09 9.65244565999483e-09
78.6666666666667
"chr10" 45455001 45456000 "*" 0 0 51.5151515151515
"chr10" 45479001 45480000 "*" 1.98250249439091e-06 2.41308381290393e-06

-54.8387096774194
"chr10" 45487001 45488000 "*" 1.64716745554294e-06 2.02893190508151e-06
54.1666666666667
"chr10" 45488001 45489000 "*" 0 0 100
"chr10" 45560001 45561000 "*" 1.87405646556726e-13 5.97285292681429e-13 -100
"chr10" 45647001 45648000 "*" 5.52147216836829e-11 1.27543173283672e-10
-61.1111111111111
"chr10" 45761001 45762000 "*" 3.04324343503026e-11 7.26599935673121e-11 100
"chr10" 45830001 45831000 "*" 7.45486561370967e-10 1.46952418634053e-09
58.3333333333333
"chr10" 45877001 45878000 "*" 0 0 66.5470494417863
"chr10" 45920001 45921000 "*" 3.16660142551939e-10 6.58836758441552e-10
82.1428571428571
"chr10" 45937001 45938000 "*" 8.7349629751543e-09 1.47505871898511e-08 100
"chr10" 46089001 46090000 "*" 0 0 100
"chr10" 46090001 46091000 "*" 0 0 -59.2307692307692
"chr10" 46095001 46096000 "*" 1.5277158427196e-09 2.87642564232045e-09 -100
"chr10" 46167001 46168000 "*" 0 0 74.3298131600325
"chr10" 46168001 46169000 "*" 0 0 78.277047104726
"chr10" 46174001 46175000 "*" 4.65747359568169e-08 7.09486098372231e-08
-78.8235294117647
"chr10" 46975001 46976000 "*" 1.46446613724649e-07 2.07416993351239e-07
-73.3333333333333
"chr10" 46982001 46983000 "*" 3.03090885722668e-14 1.06210046699299e-13
78.2312925170068
"chr10" 46990001 46991000 "*" 0 0 100
"chr10" 46994001 46995000 "*" 0 0 -59.9994323342416
"chr10" 47009001 47010000 "*" 3.18355716233398e-07 4.31991940783722e-07
-58.5365853658537
"chr10" 47033001 47034000 "*" 1.85877979674842e-10 4.00587949461252e-10
-61.5384615384615
"chr10" 47036001 47037000 "*" 7.50402037186237e-06 8.37838983711095e-06
-66.6666666666667
"chr10" 47053001 47054000 "*" 6.99440505513849e-15 2.64480529781883e-14 100
"chr10" 47081001 47082000 "*" 1.04281825397123e-08 1.74477439352264e-08
-78.3783783783784
"chr10" 47082001 47083000 "*" 8.57092175010621e-13 2.51149198099779e-12 -100
"chr10" 47086001 47087000 "*" 1.24172672144596e-10 2.73615878925439e-10
68.6567164179104
"chr10" 47101001 47102000 "*" 0 0 70.2380952380952
"chr10" 47110001 47111000 "*" 7.0006535457523e-08 1.03695832512054e-07 -100
"chr10" 47587001 47588000 "*" 3.66373598126302e-14 1.27257562929612e-13
85.4545454545455
"chr10" 47622001 47623000 "*" 1.30610560145072e-08 2.15310953267345e-08
-51.2820512820513
"chr10" 47623001 47624000 "*" 3.03979064142368e-13 9.42350883309283e-13 100
"chr10" 47636001 47637000 "*" 4.08209022140227e-11 9.57770690255255e-11 100
"chr10" 47639001 47640000 "*" 3.44270512098177e-09 6.18628415640066e-09 58
"chr10" 47670001 47671000 "*" 6.14674399157522e-08 9.2143926124143e-08
-65.9224441833138
"chr10" 47698001 47699000 "*" 4.73234496034536e-09 8.30627300264826e-09 -100
"chr10" 48359001 48360000 "*" 1.25088828184516e-11 3.15648405606829e-11

-54.4602698650675
"chr10" 48363001 48364000 "*" 7.0006535457523e-08 1.03695832512054e-07 -100
"chr10" 48377001 48378000 "*" 6.92287338566189e-11 1.57605630124247e-10 -100
"chr10" 48379001 48380000 "*" 1.75082170983387e-13 5.6014942024636e-13 -100
"chr10" 48397001 48398000 "*" 2.0616746088109e-09 3.8255929181586e-09
-85.7142857142857
"chr10" 48412001 48413000 "*" 0 0 59.5505617977528
"chr10" 48435001 48436000 "*" 5.7065463465733e-14 1.93318145576218e-13 100
"chr10" 48438001 48439000 "*" 0 0 51.5955011241583
"chr10" 48460001 48461000 "*" 3.70571128227581e-08 5.70693202308691e-08
-83.8709677419355
"chr10" 48492001 48493000 "*" 1.70641278884887e-12 4.81986032810646e-12 100
"chr10" 48531001 48532000 "*" 2.43005615629954e-09 4.45014222280278e-09 100
"chr10" 48551001 48552000 "*" 1.71226651746181e-08 2.7738131218886e-08 -60
"chr10" 48632001 48633000 "*" 5.48638023900594e-10 1.10374357336421e-09 -100
"chr10" 48643001 48644000 "*" 2.22044604925031e-16 9.81641919380259e-16
81.9672131147541
"chr10" 48644001 48645000 "*" 2.03876915350065e-11 4.98621062028308e-11 -55
"chr10" 49397001 49398000 "*" 0.000226645703264028 0.000200573098869507
-53.7037037037037
"chr10" 49448001 49449000 "*" 5.76576553168451e-09 1.00214420049533e-08
66.6666666666667
"chr10" 49450001 49451000 "*" 0 0 -84.4155844155844
"chr10" 49656001 49657000 "*" 5.22915044598449e-14 1.78122362258825e-13
52.6315789473684
"chr10" 49703001 49704000 "*" 2.43005615629954e-09 4.45014222280278e-09 100
"chr10" 49749001 49750000 "*" 0 0 100
"chr10" 49756001 49757000 "*" 9.65729496371637e-10 1.87255912493232e-09 -100
"chr10" 49769001 49770000 "*" 7.75573560929388e-09 1.32322843386823e-08
60.7142857142857
"chr10" 49770001 49771000 "*" 9.0072393987839e-13 2.63157725646847e-12 100
"chr10" 49776001 49777000 "*" 4.38208867858947e-07 5.83499808670586e-07
51.4285714285714
"chr10" 49813001 49814000 "*" 1.13140401492018e-08 1.87801380787728e-08 -100
"chr10" 49843001 49844000 "*" 4.44089209850063e-15 1.71914916534614e-14 100
"chr10" 49899001 49900000 "*" 1.00050878804048e-06 1.26940594284362e-06
-65.5172413793103
"chr10" 49905001 49906000 "*" 7.34485790570005e-05 7.03153538894558e-05
54.1666666666667
"chr10" 49969001 49970000 "*" 5.09592368302947e-14 1.73761636562837e-13 100
"chr10" 50019001 50020000 "*" 9.65729496371637e-10 1.87255912493232e-09 -100
"chr10" 50060001 50061000 "*" 1.11022302462516e-15 4.59817122935606e-15 100
"chr10" 50088001 50089000 "*" 5.99520433297585e-15 2.28531569014092e-14
73.1707317073171
"chr10" 50098001 50099000 "*" 4.52432529829405e-06 5.21774801492533e-06
-69.0476190476191
"chr10" 50140001 50141000 "*" 3.34128893442198e-08 5.19772864618523e-08 -100
"chr10" 50147001 50148000 "*" 9.35705957161304e-11 2.09280294419132e-10 -72.5
"chr10" 50158001 50159000 "*" 6.7348775123488e-07 8.75621186218476e-07
53.8461538461538
"chr10" 50178001 50179000 "*" 0 0 60.4790419161677
"chr10" 50210001 50211000 "*" 2.64338018141075e-09 4.82184881095208e-09

58.4615384615385
"chr10" 50220001 50221000 "*" 4.63274163423621e-05 4.57978907776308e-05
61.5384615384615
"chr10" 50279001 50280000 "*" 1.4432899320127e-15 5.90750815956055e-15 100
"chr10" 50289001 50290000 "*" 5.03264097062583e-13 1.51942428521737e-12 -100
"chr10" 50339001 50340000 "*" 0 0 55.4455445544554
"chr10" 50343001 50344000 "*" 0 0 100
"chr10" 50398001 50399000 "*" 0 0 -100
"chr10" 50480001 50481000 "*" 1.66808789003881e-11 4.13898883243923e-11
65.0485436893204
"chr10" 50489001 50490000 "*" 0 0 90.6976744186046
"chr10" 50531001 50532000 "*" 4.73229977426826e-09 8.30627300264826e-09 68
"chr10" 50569001 50570000 "*" 1.59796176291138e-09 3.00261630635878e-09 -62.5
"chr10" 50573001 50574000 "*" 2.43005615629954e-09 4.45014222280278e-09 -100
"chr10" 50594001 50595000 "*" 1.01796349127881e-12 2.95485398379362e-12 -60
"chr10" 50599001 50600000 "*" 1.13140401492018e-08 1.87801380787728e-08 -100
"chr10" 50602001 50603000 "*" 0 0 96.9230769230769
"chr10" 50606001 50607000 "*" 0 0 50.420806615453
"chr10" 50704001 50705000 "*" 1.12687636999453e-13 3.68543487142567e-13 -100
"chr10" 50791001 50792000 "*" 6.59550958292954e-09 1.13456194382773e-08 100
"chr10" 50808001 50809000 "*" 1.0511591597151e-12 3.04845283212741e-12
-50.8196721311475
"chr10" 50856001 50857000 "*" 2.49780196526217e-11 6.05629632802696e-11
-91.8604651162791
"chr10" 50941001 50942000 "*" 8.3882012447134e-11 1.88922989503423e-10 100
"chr10" 50955001 50956000 "*" 1.48087875295744e-10 3.22075573380618e-10 -100
"chr10" 51040001 51041000 "*" 5.6362137179633e-11 1.29963270300065e-10 100
"chr10" 51489001 51490000 "*" 0 0 78.7090558766859
"chr10" 51516001 51517000 "*" 4.69074590281338e-09 8.25845934437494e-09
79.7101449275362
"chr10" 52103001 52104000 "*" 4.57277404830592e-09 8.0616513986018e-09
52.9411764705882
"chr10" 52177001 52178000 "*" 0 0 -78.7126569490547
"chr10" 52374001 52375000 "*" 1.49559882256511e-08 2.44725194116009e-08
66.6666666666667
"chr10" 52441001 52442000 "*" 3.33066907387547e-15 1.30941888440006e-14 -100
"chr10" 52635001 52636000 "*" 2.33028818463765e-10 4.93719283454501e-10 -100
"chr10" 52641001 52642000 "*" 5.01025332333427e-10 1.01358031135889e-09 100
"chr10" 54531001 54532000 "*" 1.98124849859482e-10 4.25157611354621e-10 -100
"chr10" 54649001 54650000 "*" 0 0 -52.2727272727273
"chr10" 57755001 57756000 "*" 2.64951482975562e-09 4.82186011375752e-09 100
"chr10" 58364001 58365000 "*" 0 0 77.3722627737226
"chr10" 58777001 58778000 "*" 2.68450373042128e-10 5.64465213127486e-10 100
"chr10" 58855001 58856000 "*" 0 0 -100
"chr10" 59400001 59401000 "*" 0 0 100
"chr10" 60273001 60274000 "*" 0 0 77.942090771827
"chr10" 60352001 60353000 "*" 0 0 -100
"chr10" 60624001 60625000 "*" 2.00227675550835e-08 3.20459176617961e-08 -100
"chr10" 60769001 60770000 "*" 4.14335232790108e-13 1.26153063645578e-12 -100
"chr10" 60937001 60938000 "*" 0 0 90.9813862173513
"chr10" 61063001 61064000 "*" 2.64951482975562e-09 4.82186011375752e-09 -100
"chr10" 61230001 61231000 "*" 9.04207819729663e-11 2.03063150957007e-10 96.875

"chr10" 61350001 61351000 "*" 4.02167188440217e-12 1.08266700497926e-11 100
"chr10" 61353001 61354000 "*" 1.66464911854902e-08 2.71015083250981e-08 -84
"chr10" 61468001 61469000 "*" 0 0 -76
"chr10" 61636001 61637000 "*" 5.48638023900594e-10 1.10374357336421e-09 -100
"chr10" 62799001 62800000 "*" 1.09648179669186e-06 1.3842235994417e-06
51.5151515151515
"chr10" 63227001 63228000 "*" 6.30384633382164e-13 1.87665599796749e-12 -100
"chr10" 63540001 63541000 "*" 5.794809077031e-12 1.52911192757929e-11 100
"chr10" 63743001 63744000 "*" 1.52466927971773e-12 4.33322475981408e-12 100
"chr10" 64133001 64134000 "*" 0 0 93.2584269662921
"chr10" 64134001 64135000 "*" 0 0 73.6020303865011
"chr10" 64148001 64149000 "*" 2.27215357639921e-10 4.82751291317784e-10 -87.5
"chr10" 64224001 64225000 "*" 1.39927976983145e-08 2.29771440325017e-08 -68.75
"chr10" 64684001 64685000 "*" 0 0 76.9980506822612
"chr10" 64693001 64694000 "*" 1.36604061395929e-11 3.42333952202875e-11 100
"chr10" 65190001 65191000 "*" 0 0 100
"chr10" 65349001 65350000 "*" 9.04570973681018e-10 1.76414389822173e-09 -100
"chr10" 65420001 65421000 "*" 3.88936447237587e-06 4.52924899346346e-06
66.6666666666667
"chr10" 65484001 65485000 "*" 9.93310270702707e-07 1.26111689577963e-06
-65.9090909090909
"chr10" 65556001 65557000 "*" 1.38590361409285e-10 3.02706178892116e-10 -100
"chr10" 65798001 65799000 "*" 6.92287338566189e-11 1.57605630124247e-10 -100
"chr10" 67571001 67572000 "*" 0 0 70.7070707070707
"chr10" 68347001 68348000 "*" 0 0 100
"chr10" 69392001 69393000 "*" 5.44009282066327e-15 2.08486137681812e-14 -100
"chr10" 69571001 69572000 "*" 4.44089209850063e-16 1.91071758245033e-15
-99.2537313432836
"chr10" 69739001 69740000 "*" 4.08209022140227e-11 9.57770690255255e-11 -100
"chr10" 69944001 69945000 "*" 5.01025332333427e-10 1.01358031135889e-09 100
"chr10" 70030001 70031000 "*" 3.40125705378114e-10 7.03784073980162e-10 100
"chr10" 70308001 70309000 "*" 4.21493528968142e-09 7.45822546499483e-09
66.6666666666667
"chr10" 70320001 70321000 "*" 0 0 66.3350785340314
"chr10" 70821001 70822000 "*" 0 0 -100
"chr10" 70956001 70957000 "*" 3.40125705378114e-10 7.03784073980162e-10 100
"chr10" 70993001 70994000 "*" 0 0 -100
"chr10" 71032001 71033000 "*" 1.51656465163796e-13 4.87803420672651e-13 100
"chr10" 71209001 71210000 "*" 2.82773804372027e-13 8.79129664109438e-13 100
"chr10" 71210001 71211000 "*" 0 0 100
"chr10" 71214001 71215000 "*" 0 0 -100
"chr10" 71219001 71220000 "*" 1.77635683940025e-15 7.19870740878856e-15 -100
"chr10" 71220001 71221000 "*" 6.92287338566189e-11 1.57605630124247e-10 100
"chr10" 71229001 71230000 "*" 8.01666433236647e-09 1.36392368970109e-08 100
"chr10" 71237001 71238000 "*" 0.000184006388664448 0.000165249884973406
54.1666666666667
"chr10" 71239001 71240000 "*" 2.22044604925031e-16 9.81641919380259e-16 100
"chr10" 71260001 71261000 "*" 1.55279055280566e-07 2.19249012734611e-07
-76.4705882352941
"chr10" 71277001 71278000 "*" 4.7010173531703e-12 1.25630647180544e-11
-56.3795853269537
"chr10" 71318001 71319000 "*" 8.08271427565899e-08 1.18686874328483e-07

61.9047619047619
"chr10" 71319001 71320000 "*" 6.52733422867868e-12 1.71146951718423e-11 -100
"chr10" 71336001 71337000 "*" 0 0 100
"chr10" 71395001 71396000 "*" 0.000126887439664602 0.000117029410540844
56.5217391304348
"chr10" 71402001 71403000 "*" 8.7349629751543e-09 1.47505871898511e-08 100
"chr10" 71405001 71406000 "*" 5.14294729025977e-09 8.98856693399752e-09
58.6206896551724
"chr10" 71421001 71422000 "*" 0 0 100
"chr10" 71489001 71490000 "*" 6.48628384336547e-09 1.12018676650369e-08
-64.3835616438356
"chr10" 71566001 71567000 "*" 5.92158689083355e-07 7.75918669361872e-07
63.6363636363636
"chr10" 71571001 71572000 "*" 0.000114836247179562 0.000106709856628931
56.6666666666667
"chr10" 71572001 71573000 "*" 4.91579557823085e-05 4.83987297040465e-05
-58.3333333333333
"chr10" 71573001 71574000 "*" 4.01313771103418e-09 7.11882087237587e-09 -100
"chr10" 71595001 71596000 "*" 0.000229234803195366 0.000202707360857361
54.1666666666667
"chr10" 71614001 71615000 "*" 9.65729496371637e-10 1.87255912493232e-09 -100
"chr10" 71676001 71677000 "*" 3.38366751284624e-08 5.25796497446358e-08
-65.7894736842105
"chr10" 71721001 71722000 "*" 0 0 100
"chr10" 71722001 71723000 "*" 1.53680517755994e-07 2.17091175393351e-07 60
"chr10" 71723001 71724000 "*" 1.22949261771588e-07 1.76265240892153e-07 70
"chr10" 71726001 71727000 "*" 5.794809077031e-12 1.52911192757929e-11 100
"chr10" 71812001 71813000 "*" 0 0 61.6521739130435
"chr10" 71814001 71815000 "*" 6.70169475469606e-11 1.53101052789825e-10
-92.4731182795699
"chr10" 71865001 71866000 "*" 2.57717458485018e-09 4.70736216796089e-09
66.1773341162654
"chr10" 71904001 71905000 "*" 0 0 -66.6666666666667
"chr10" 72053001 72054000 "*" 6.30384633382164e-13 1.87665599796749e-12 100
"chr10" 72168001 72169000 "*" 1.62840060813352e-06 2.00704795184366e-06 65
"chr10" 72275001 72276000 "*" 9.52204124201605e-05 8.95780133799817e-05
59.2592592592593
"chr10" 72363001 72364000 "*" 1.05053743482131e-11 2.68109655304185e-11
-60.4651162790698
"chr10" 72410001 72411000 "*" 3.65663700319097e-07 4.9208712465116e-07
73.9130434782609
"chr10" 72421001 72422000 "*" 3.13902409559974e-05 3.18712470329053e-05
-56.5217391304348
"chr10" 72449001 72450000 "*" 1.61810564947018e-11 4.02071084901731e-11
87.1559633027523
"chr10" 72460001 72461000 "*" 1.8757218001042e-12 5.27290364871055e-12
-94.2307692307692
"chr10" 72468001 72469000 "*" 2.05535676878199e-06 2.49709655247574e-06
-56.8421052631579
"chr10" 72514001 72515000 "*" 9.76996261670138e-15 3.63402770315216e-14 100
"chr10" 72523001 72524000 "*" 1.39779633423487e-07 1.98463037958589e-07 100
"chr10" 72537001 72538000 "*" 0 0 100

"chr10" 72651001 72652000 "*" 1.93720595120794e-11 4.76309924836636e-11 100
"chr10" 72670001 72671000 "*" 0 0 -100
"chr10" 72671001 72672000 "*" 1.21922773882455e-05 1.31811221609643e-05
58.8235294117647
"chr10" 72672001 72673000 "*" 6.135425500986e-12 1.61475200538952e-11
51.7241379310345
"chr10" 72679001 72680000 "*" 7.7715611723761e-15 2.92461699448967e-14
50.6666666666667
"chr10" 72683001 72684000 "*" 9.71063229826541e-11 2.16781365050398e-10 -100
"chr10" 72685001 72686000 "*" 2.73625566649116e-12 7.52483363579137e-12 100
"chr10" 72689001 72690000 "*" 2.88710388929303e-10 6.03399264731139e-10 -100
"chr10" 72691001 72692000 "*" 1.95732319241415e-13 6.22064318429587e-13
95.4545454545455
"chr10" 72692001 72693000 "*" 0 0 100
"chr10" 72703001 72704000 "*" 6.04585936869739e-10 1.21048826102541e-09
53.8461538461538
"chr10" 72724001 72725000 "*" 5.7065463465733e-14 1.93318145576218e-13 100
"chr10" 72752001 72753000 "*" 1.35036426485158e-12 3.86629547363774e-12 100
"chr10" 72788001 72789000 "*" 3.61871754783749e-10 7.45986023744022e-10
78.9473684210526
"chr10" 72789001 72790000 "*" 7.23147142167591e-09 1.23834210764063e-08 75
"chr10" 72790001 72791000 "*" 3.52704532247117e-11 8.35581292290093e-11 100
"chr10" 72799001 72800000 "*" 6.39155395276703e-12 1.67951118302464e-11
-57.6772509786864
"chr10" 72837001 72838000 "*" 0 0 100
"chr10" 72850001 72851000 "*" 5.2679309248127e-08 7.96470597000832e-08
50.4132231404959
"chr10" 72886001 72887000 "*" 1.92946192356658e-09 3.59193011611483e-09 100
"chr10" 72934001 72935000 "*" 3.95353005888666e-09 7.03928529283733e-09 100
"chr10" 72941001 72942000 "*" 1.6153745008296e-13 5.18386083482492e-13 65.625
"chr10" 72963001 72964000 "*" 0 0 100
"chr10" 73006001 73007000 "*" 0 0 -100
"chr10" 73026001 73027000 "*" 0 0 -93.7777777777778
"chr10" 73078001 73079000 "*" 1.52100554373646e-14 5.52760130909381e-14
64.2857142857143
"chr10" 73079001 73080000 "*" 0 0 100
"chr10" 73156001 73157000 "*" 0 0 54.4221576712687
"chr10" 73157001 73158000 "*" 0 0 68.8445433826257
"chr10" 73200001 73201000 "*" 4.73234496034536e-09 8.30627300264826e-09 100
"chr10" 73210001 73211000 "*" 0.000525299954949099 0.000436849389802675
-54.5454545454545
"chr10" 73212001 73213000 "*" 8.95384697952029e-06 9.88218350385102e-06
52.1739130434783
"chr10" 73216001 73217000 "*" 4.08209022140227e-11 9.57770690255255e-11 100
"chr10" 73226001 73227000 "*" 1.67299207820548e-08 2.71377429441055e-08 -100
"chr10" 73244001 73245000 "*" 4.83744390378238e-06 5.55756940206571e-06 70
"chr10" 73272001 73273000 "*" 7.45181694128405e-13 2.20182359028443e-12
-74.8502994011976
"chr10" 73286001 73287000 "*" 2.86368005975035e-07 3.91086988294228e-07
53.3333333333333
"chr10" 73293001 73294000 "*" 0 0 100
"chr10" 73296001 73297000 "*" 2.77555756156289e-15 1.10015415195978e-14 100

"chr10" 73328001 73329000 "*" 0 0 100
"chr10" 73336001 73337000 "*" 1.93720595120794e-11 4.76309924836636e-11 -100
"chr10" 73337001 73338000 "*" 0 0 -65.5394524959742
"chr10" 73355001 73356000 "*" 3.59617469136708e-09 6.44844178118722e-09
-52.5252525252525
"chr10" 73378001 73379000 "*" 2.79621881205117e-11 6.73443061609763e-11
53.3333333333333
"chr10" 73388001 73389000 "*" 1.82520665248376e-13 5.82874509614307e-13 -66
"chr10" 73389001 73390000 "*" 1.14352971536391e-14 4.21921674335999e-14 -100
"chr10" 73398001 73399000 "*" 0 0 52.0296888381387
"chr10" 73400001 73401000 "*" 2.52417531321214e-10 5.33037880173179e-10
-58.3333333333333
"chr10" 73406001 73407000 "*" 4.06367051120071e-10 8.33201280581531e-10 -84
"chr10" 73416001 73417000 "*" 0 0 -93.8053097345133
"chr10" 73427001 73428000 "*" 0 0 81.4814814814815
"chr10" 73448001 73449000 "*" 1.04591335592374e-10 2.32706156739909e-10
95.6521739130435
"chr10" 73450001 73451000 "*" 0 0 -84.6153846153846
"chr10" 73488001 73489000 "*" 2.08814465718632e-09 3.86232567960463e-09 -100
"chr10" 73500001 73501000 "*" 1.49880108324396e-14 5.44951848293151e-14 -74
"chr10" 73533001 73534000 "*" 7.36131156031661e-11 1.67158166723342e-10
-52.5132609244759
"chr10" 73538001 73539000 "*" 1.51087968247587e-07 2.13674995855393e-07
57.5757575757576
"chr10" 73585001 73586000 "*" 1.12055920098442e-11 2.84500040664577e-11 100
"chr10" 73638001 73639000 "*" 0 0 -100
"chr10" 73722001 73723000 "*" 9.62896429257398e-13 2.80291205576417e-12 -100
"chr10" 73734001 73735000 "*" 1.40028522332969e-07 1.98806263864739e-07
-77.7777777777778
"chr10" 73765001 73766000 "*" 1.05286133791349e-05 1.1495028000685e-05
58.0645161290323
"chr10" 73769001 73770000 "*" 1.68996275862199e-08 2.7398105072003e-08
52.3809523809524
"chr10" 73802001 73803000 "*" 1.12458486967171e-09 2.15740815043925e-09 100
"chr10" 73821001 73822000 "*" 3.33066907387547e-16 1.4495649018245e-15
-84.6938775510204
"chr10" 73976001 73977000 "*" 1.93178806284777e-14 6.93672913283581e-14
60.655737704918
"chr10" 74003001 74004000 "*" 4.98067509457911e-09 8.71957375821299e-09
54.5454545454545
"chr10" 74046001 74047000 "*" 2.85191870119661e-11 6.86283130534681e-11
-92.0863309352518
"chr10" 74079001 74080000 "*" 0 0 -64.3243243243243
"chr10" 74080001 74081000 "*" 0 0 -100
"chr10" 74081001 74082000 "*" 4.37737760583534e-06 5.05673919921062e-06
-53.7313432835821
"chr10" 74091001 74092000 "*" 0 0 -86.1924686192469
"chr10" 74655001 74656000 "*" 1.03657082917152e-11 2.64747747937042e-11
57.3770491803279
"chr10" 75118001 75119000 "*" 0 0 100
"chr10" 75533001 75534000 "*" 1.81023101157862e-05 1.90701455186207e-05
-58.6666666666667

"chr10" 75610001 75611000 "*" 3.95353005888666e-09 7.03928529283733e-09 100
"chr10" 75633001 75634000 "*" 0 0 -89.4736842105263
"chr10" 75647001 75648000 "*" 9.80763359414993e-10 1.8980032424715e-09 -100
"chr10" 75726001 75727000 "*" 1.11022302462516e-16 5.03662826618488e-16 -100
"chr10" 76516001 76517000 "*" 6.44564592999775e-06 7.26663863268796e-06
-66.6666666666667
"chr10" 76523001 76524000 "*" 6.5536465143623e-13 1.94602017656154e-12 100
"chr10" 76795001 76796000 "*" 2.37926345292294e-11 5.78098267292937e-11
63.8888888888889
"chr10" 76993001 76994000 "*" 0 0 -56.1797752808989
"chr10" 77007001 77008000 "*" 1.25019894436917e-09 2.38631078205631e-09
-79.3103448275862
"chr10" 77021001 77022000 "*" 1.99840144432528e-15 8.0570469535907e-15
77.2727272727273
"chr10" 77054001 77055000 "*" 0 0 -93.1094720496894
"chr10" 77134001 77135000 "*" 1.70641278884887e-12 4.81986032810646e-12 -100
"chr10" 77226001 77227000 "*" 1.6219141030227e-08 2.64300079144354e-08
-66.6666666666667
"chr10" 77474001 77475000 "*" 1.92946192356658e-09 3.59193011611483e-09 -100
"chr10" 77517001 77518000 "*" 6.33547441331039e-06 7.15161785124816e-06
-53.8461538461538
"chr10" 77635001 77636000 "*" 3.28319936359067e-05 3.32125799277005e-05 60
"chr10" 77769001 77770000 "*" 5.34930543194534e-08 8.08173108939779e-08
-64.4444444444444
"chr10" 79034001 79035000 "*" 2.15125472990962e-10 4.58126659943874e-10 -100
"chr10" 79161001 79162000 "*" 2.15125472990962e-10 4.58126659943874e-10 -100
"chr10" 79273001 79274000 "*" 0 0 -100
"chr10" 79341001 79342000 "*" 8.3882012447134e-11 1.88922989503423e-10 -100
"chr10" 79351001 79352000 "*" 2.88710388929303e-10 6.03399264731139e-10 100
"chr10" 79362001 79363000 "*" 2.98248926521305e-05 3.03873442006679e-05
56.4102564102564
"chr10" 79379001 79380000 "*" 2.22044604925031e-16 9.81641919380259e-16 100
"chr10" 79398001 79399000 "*" 0 0 52.9559307681639
"chr10" 79431001 79432000 "*" 1.33476563135559e-11 3.35251871544207e-11
53.4883720930233
"chr10" 79604001 79605000 "*" 2.22044604925031e-16 9.81641919380259e-16 100
"chr10" 79633001 79634000 "*" 0 0 68.7754841997961
"chr10" 79649001 79650000 "*" 1.02158503878513e-10 2.2759083301767e-10
-68.9655172413793
"chr10" 79681001 79682000 "*" 0 0 100
"chr10" 79685001 79686000 "*" 0 0 100
"chr10" 79793001 79794000 "*" 0 0 -100
"chr10" 79822001 79823000 "*" 2.68450373042128e-10 5.64465213127486e-10 100
"chr10" 79940001 79941000 "*" 5.03441732746523e-12 1.3378171545226e-11 -100
"chr10" 79941001 79942000 "*" 1.1409251543526e-08 1.89300825733629e-08 65
"chr10" 79981001 79982000 "*" 0 0 -100
"chr10" 80040001 80041000 "*" 0 0 -100
"chr10" 80050001 80051000 "*" 9.08108033215171e-11 2.03407753381119e-10 -100
"chr10" 80061001 80062000 "*" 9.14679443297928e-12 2.35214920679888e-11
-95.7746478873239
"chr10" 80092001 80093000 "*" 1.26529786648177e-10 2.78280435481264e-10
64.5161290322581

"chr10" 80116001 80117000 "*" 4.02167188440217e-12 1.08266700497926e-11 -100
"chr10" 80122001 80123000 "*" 0.000326726626022067 0.000281450864980403
55.1724137931034
"chr10" 80140001 80141000 "*" 7.73937738340891e-08 1.13929832828137e-07 85
"chr10" 80167001 80168000 "*" 0 0 -80.2325581395349
"chr10" 80182001 80183000 "*" 2.73014943985572e-11 6.58595916221891e-11 100
"chr10" 80207001 80208000 "*" 1.36604061395929e-11 3.42333952202875e-11 100
"chr10" 80209001 80210000 "*" 8.80406858527749e-14 2.91456141063452e-13 100
"chr10" 80288001 80289000 "*" 0 0 100
"chr10" 80291001 80292000 "*" 1.54630752646767e-11 3.85079502648228e-11 -100
"chr10" 80295001 80296000 "*" 2.79440914852103e-11 6.73046092948135e-11 100
"chr10" 80312001 80313000 "*" 2.94254883792533e-08 4.61645267024725e-08
63.1578947368421
"chr10" 80492001 80493000 "*" 6.4152538836737e-10 1.27477697728417e-09 -100
"chr10" 80503001 80504000 "*" 4.03652502123375e-06 4.6892927905593e-06 -68.75
"chr10" 80520001 80521000 "*" 6.76132598590451e-05 6.51054864379892e-05 -60
"chr10" 80526001 80527000 "*" 0 0 100
"chr10" 80562001 80563000 "*" 0 0 -100
"chr10" 80563001 80564000 "*" 0 0 100
"chr10" 80589001 80590000 "*" 1.5277158427196e-09 2.87642564232045e-09 -100
"chr10" 80630001 80631000 "*" 3.17589698983767e-09 5.73129984759112e-09 60
"chr10" 80660001 80661000 "*" 1.35890187991095e-11 3.40841971363279e-11 100
"chr10" 80699001 80700000 "*" 3.37507799486048e-14 1.17696879536064e-13
-52.9411764705882
"chr10" 80733001 80734000 "*" 0 0 -100
"chr10" 80747001 80748000 "*" 2.57009968862576e-11 6.22308980701977e-11 -87.5
"chr10" 80765001 80766000 "*" 2.90132882163618e-08 4.55598361662795e-08
64.2857142857143
"chr10" 80807001 80808000 "*" 9.0072393987839e-13 2.63157725646847e-12 100
"chr10" 80817001 80818000 "*" 0 0 -75.5367599219258
"chr10" 80835001 80836000 "*" 0 0 100
"chr10" 80865001 80866000 "*" 2.04988070962031e-09 3.80458088311340e-09
-66.6666666666667
"chr10" 80886001 80887000 "*" 7.0006535457523e-08 1.03695832512054e-07 100
"chr10" 80887001 80888000 "*" 6.95250634841216e-10 1.37589605662581e-09 -87.5
"chr10" 80911001 80912000 "*" 0 0 -73.3333333333333
"chr10" 80930001 80931000 "*" 1.11022302462516e-16 5.03662826618488e-16 -100
"chr10" 80940001 80941000 "*" 3.597027969926e-07 4.84612690151399e-07
90.9090909090909
"chr10" 80943001 80944000 "*" 3.77475828372553e-15 1.47379557022071e-14 -100
"chr10" 81036001 81037000 "*" 5.63660229602192e-13 1.68905058935478e-12 100
"chr10" 81055001 81056000 "*" 1.47189243016044e-08 2.4110155070176e-08
87.1428571428571
"chr10" 81095001 81096000 "*" 2.20192752919957e-11 5.36667613349327e-11
85.2941176470588
"chr10" 81141001 81142000 "*" 9.75381331258518e-10 1.89030726491126e-09
-64.9484536082474
"chr10" 81169001 81170000 "*" 0 0 65.9574468085106
"chr10" 81179001 81180000 "*" 5.51780843238703e-14 1.87243792572872e-13 100
"chr10" 81184001 81185000 "*" 7.8026474170656e-13 2.30092013574739e-12
59.0909090909091
"chr10" 81191001 81192000 "*" 7.03992419914812e-12 1.83313429961219e-11 100

"chr10" 81263001 81264000 "*" 4.22826804680199e-08 6.47129910406812e-08 81.4814814814815
"chr10" 81318001 81319000 "*" 2.22044604925031e-16 9.81641919380259e-16 68.3333333333333
"chr10" 81741001 81742000 "*" 0 0 68.718179179921
"chr10" 81742001 81743000 "*" 0 0 54.3484645342527
"chr10" 81897001 81898000 "*" 1.48087875295744e-10 3.22075573380618e-10 -100
"chr10" 81966001 81967000 "*" 8.77076189453874e-15 3.27889038038365e-14 -100
"chr10" 82037001 82038000 "*" 7.0006535457523e-08 1.03695832512054e-07 -100
"chr10" 82043001 82044000 "*" 2.14126367292788e-06 2.59560598119355e-06 -70.8333333333333
"chr10" 82146001 82147000 "*" 0 0 -100
"chr10" 82213001 82214000 "*" 0 0 -100
"chr10" 82228001 82229000 "*" 0 0 82.1428571428571
"chr10" 82295001 82296000 "*" 0 0 -50.2124656093802
"chr10" 82300001 82301000 "*" 1.06581410364015e-14 3.94854473541761e-14 -63.6363636363636
"chr10" 82378001 82379000 "*" 3.03090885722668e-14 1.06210046699299e-13 -100
"chr10" 82420001 82421000 "*" 8.58968451922237e-12 2.213554983903e-11 100
"chr10" 82453001 82454000 "*" 4.71134242729931e-12 1.257615036806e-11 -100
"chr10" 82873001 82874000 "*" 1.11022302462516e-16 5.03662826618488e-16 -100
"chr10" 83330001 83331000 "*" 8.3882012447134e-11 1.88922989503423e-10 100
"chr10" 83427001 83428000 "*" 6.49287290599432e-11 1.48554297623062e-10 51.131221719457
"chr10" 85546001 85547000 "*" 9.99200722162641e-16 4.15382497462808e-15 -64.8148148148148
"chr10" 85705001 85706000 "*" 5.295763827462e-14 1.80214784640623e-13 100
"chr10" 85936001 85937000 "*" 1.92946192356658e-09 3.59193011611483e-09 100
"chr10" 85954001 85955000 "*" 0 0 59.5576122829674
"chr10" 85960001 85961000 "*" 5.7065463465733e-14 1.93318145576218e-13 100
"chr10" 85973001 85974000 "*" 0 0 -100
"chr10" 86001001 86002000 "*" 2.18158824338843e-13 6.87314823207509e-13 100
"chr10" 86304001 86305000 "*" 6.88338275267597e-15 2.60518659957509e-14 100
"chr10" 86348001 86349000 "*" 1.25284005392245e-10 2.75649590272477e-10 100
"chr10" 86998001 86999000 "*" 8.86057893723091e-12 2.28051616751041e-11 -66.6666666666667
"chr10" 87407001 87408000 "*" 1.11022302462516e-16 5.03662826618488e-16 -100
"chr10" 87680001 87681000 "*" 8.88178419700125e-16 3.70670032207938e-15 100
"chr10" 87748001 87749000 "*" 8.52111439630931e-07 1.09183873929303e-06 -54.5454545454545
"chr10" 87778001 87779000 "*" 5.04263297784746e-13 1.52078941532818e-12 100
"chr10" 87791001 87792000 "*" 6.66133814775094e-16 2.81595744474255e-15 100
"chr10" 87820001 87821000 "*" 1.14124265593318e-11 2.89138062669327e-11 100
"chr10" 87821001 87822000 "*" 1.4432899320127e-15 5.90750815956055e-15 -100
"chr10" 87829001 87830000 "*" 1.52794177310511e-09 2.87642564232045e-09 -100
"chr10" 87837001 87838000 "*" 8.42555769597908e-09 1.42967189346436e-08 -71.4285714285714
"chr10" 87935001 87936000 "*" 1.90181204118289e-13 6.05298617539364e-13 -100
"chr10" 87941001 87942000 "*" 0 0 -100
"chr10" 87956001 87957000 "*" 3.84444556722485e-06 4.48034154814812e-06 70
"chr10" 87967001 87968000 "*" 0 0 100
"chr10" 87972001 87973000 "*" 0 0 -100

"chr10" 87974001 87975000 "*" 1.16573417585641e-14 4.29335582900508e-14 100
"chr10" 88010001 88011000 "*" 1.34343647317792e-11 3.37348442469965e-11
67.5438596491228
"chr10" 88021001 88022000 "*" 0 0 100
"chr10" 88027001 88028000 "*" 3.56775498033812e-10 7.35898629809829e-10 100
"chr10" 88028001 88029000 "*" 2.22044604925031e-16 9.81641919380259e-16 100
"chr10" 88058001 88059000 "*" 5.37803000546511e-05 5.26241567504211e-05
-54.5454545454545
"chr10" 88073001 88074000 "*" 1.13209705067119e-05 1.23037416520042e-05 -60
"chr10" 88106001 88107000 "*" 6.77335965093562e-12 1.76897011246462e-11 -100
"chr10" 88115001 88116000 "*" 3.66094932147121e-11 8.6460249470872e-11
56.4102564102564
"chr10" 88117001 88118000 "*" 1.25333201705935e-07 1.79460844963063e-07
67.5675675675676
"chr10" 88157001 88158000 "*" 8.58968451922237e-12 2.213554983903e-11 100
"chr10" 88173001 88174000 "*" 1.11022302462516e-16 5.03662826618488e-16 100
"chr10" 88282001 88283000 "*" 0 0 -100
"chr10" 88374001 88375000 "*" 2.88710388929303e-10 6.03399264731139e-10 -100
"chr10" 88390001 88391000 "*" 5.99520433297585e-13 1.79212982023631e-12
98.1132075471698
"chr10" 88436001 88437000 "*" 0 0 100
"chr10" 88442001 88443000 "*" 4.24312575547647e-05 4.21882959593721e-05
-55.8823529411765
"chr10" 88447001 88448000 "*" 6.3948846218409e-14 2.15550998060998e-13
-83.6065573770492
"chr10" 88516001 88517000 "*" 0 0 81.1359026369168
"chr10" 88673001 88674000 "*" 1.39779633423487e-07 1.98463037958589e-07 100
"chr10" 88706001 88707000 "*" 9.21768217310159e-11 2.06312360496437e-10
-61.2903225806452
"chr10" 88726001 88727000 "*" 1.89848137210902e-14 6.82212372863451e-14 -100
"chr10" 88731001 88732000 "*" 0 0 -85.4059609455293
"chr10" 88995001 88996000 "*" 0 0 100
"chr10" 89167001 89168000 "*" 1.11022302462516e-16 5.03662826618488e-16 100
"chr10" 89378001 89379000 "*" 2.73625566649116e-12 7.52483363579137e-12 -100
"chr10" 89675001 89676000 "*" 5.6621374255883e-15 2.16468580166064e-14 -100
"chr10" 89879001 89880000 "*" 1.11022302462516e-16 5.03662826618488e-16
66.6666666666667
"chr10" 90503001 90504000 "*" 5.01025332333427e-10 1.01358031135889e-09 100
"chr10" 90695001 90696000 "*" 1.19904086659517e-14 4.40901388691796e-14 100
"chr10" 90967001 90968000 "*" 0 0 56.4638512301953
"chr10" 91174001 91175000 "*" 0 0 -75
"chr10" 91813001 91814000 "*" 5.8616765863384e-07 7.68442756313584e-07
91.6666666666667
"chr10" 92598001 92599000 "*" 1.11022302462516e-16 5.03662826618488e-16
96.6666666666667
"chr10" 92875001 92876000 "*" 3.34128893442198e-08 5.19772864618523e-08 -100
"chr10" 92920001 92921000 "*" 0 0 92.3076923076923
"chr10" 93667001 93668000 "*" 3.93891363970056e-10 8.08894274732768e-10
-63.4313725490196
"chr10" 93807001 93808000 "*" 6.43996536897617e-05 6.22599140150143e-05
52.3809523809524
"chr10" 93837001 93838000 "*" 2.08814465718632e-09 3.86232567960463e-09 -100

"chr10" 93864001 93865000 "*" 1.14352971536391e-14 4.21921674335999e-14 -100
"chr10" 94244001 94245000 "*" 6.92287338566189e-11 1.57605630124247e-10 100
"chr10" 94920001 94921000 "*" 1.37828859436695e-10 3.01445544267661e-10 -100
"chr10" 95051001 95052000 "*" 2.18158824338843e-13 6.87314823207509e-13 -100
"chr10" 95094001 95095000 "*" 0 0 -62.962962962963
"chr10" 95315001 95316000 "*" 2.69007038866675e-13 8.38831022628993e-13 100
"chr10" 95694001 95695000 "*" 3.56775498033812e-10 7.35898629809829e-10 100
"chr10" 95719001 95720000 "*" 2.68299648054082e-08 4.22993906322019e-08
-74.1935483870968
"chr10" 95754001 95755000 "*" 3.10862446895044e-15 1.22466437093774e-14
64.406779661017
"chr10" 96159001 96160000 "*" 2.40249098393264e-08 3.80684405385995e-08
69.0476190476191
"chr10" 96969001 96970000 "*" 7.85083109633433e-12 2.03627673837864e-11
-52.1739130434783
"chr10" 97050001 97051000 "*" 0 0 80.3448275862069
"chr10" 97164001 97165000 "*" 1.96509475358653e-13 6.23868565133654e-13 100
"chr10" 97369001 97370000 "*" 4.99900121297969e-12 1.32921056339624e-11 -100
"chr10" 97825001 97826000 "*" 1.37828859436695e-10 3.01445544267661e-10 100
"chr10" 97948001 97949000 "*" 1.52794177310511e-09 2.87642564232045e-09 100
"chr10" 97985001 97986000 "*" 2.44093767252096e-05 2.52090772271485e-05
55.5555555555556
"chr10" 98012001 98013000 "*" 2.455369241261e-12 6.8003688121118e-12 -100
"chr10" 98017001 98018000 "*" 2.22044604925031e-16 9.81641919380259e-16 100
"chr10" 98064001 98065000 "*" 1.69376734859839e-11 4.20083199148365e-11
96.2962962962963
"chr10" 98130001 98131000 "*" 0 0 65.1162790697674
"chr10" 98167001 98168000 "*" 7.7715611723761e-16 3.26213507634405e-15 -100
"chr10" 98231001 98232000 "*" 1.52498600525242e-07 2.15509176675961e-07
54.3628013777268
"chr10" 98479001 98480000 "*" 1.59362399383411e-05 1.69352265890339e-05
-59.9557522123894
"chr10" 98773001 98774000 "*" 1.12843068222901e-12 3.259512533505e-12 -100
"chr10" 98799001 98800000 "*" 2.22044604925031e-16 9.81641919380259e-16
58.8923779617471
"chr10" 98819001 98820000 "*" 5.63660229602192e-13 1.68905058935478e-12 100
"chr10" 98825001 98826000 "*" 3.90465437760668e-13 1.19307893943277e-12 100
"chr10" 98838001 98839000 "*" 0.0002426225318366 0.000213538289284657
58.3333333333333
"chr10" 98887001 98888000 "*" 3.06475578248921e-08 4.79888042921933e-08
72.2222222222222
"chr10" 98898001 98899000 "*" 1.19382281837943e-11 3.01860462985606e-11
-52.1739130434783
"chr10" 98919001 98920000 "*" 5.6362137179633e-11 1.29963270300065e-10 100
"chr10" 98939001 98940000 "*" 9.27036225562006e-13 2.70541911008567e-12
-70.5479452054795
"chr10" 98945001 98946000 "*" 0 0 66.4342105263158
"chr10" 98955001 98956000 "*" 0 0 100
"chr10" 99093001 99094000 "*" 0 0 74.6031746031746
"chr10" 99104001 99105000 "*" 2.71362154968813e-10 5.70328936434601e-10
75.4716981132076
"chr10" 99266001 99267000 "*" 5.48638023900594e-10 1.10374357336421e-09 -100

"chr10" 99309001 99310000 "*" 1.29037891483108e-11 3.24582280286866e-11 -100
"chr10" 99497001 99498000 "*" 5.12699245114323e-06 5.87040931072255e-06
68.1818181818182
"chr10" 99516001 99517000 "*" 1.80966353013901e-14 6.52206904171103e-14 100
"chr10" 99522001 99523000 "*" 0.00017933862728825 0.000161381175027113
-51.9230769230769
"chr10" 99530001 99531000 "*" 0 0 77.4517961147488
"chr10" 99570001 99571000 "*" 1.13140401492018e-08 1.87801380787728e-08 -100
"chr10" 99628001 99629000 "*" 1.12143627717387e-12 3.24234095276305e-12
55.5555555555556
"chr10" 99634001 99635000 "*" 1.5785740136165e-06 1.9503745122263e-06
-66.6666666666667
"chr10" 99666001 99667000 "*" 2.52745491202688e-10 5.33716929288583e-10
57.1428571428571
"chr10" 99789001 99790000 "*" 0 0 54.7164253506446
"chr10" 99819001 99820000 "*" 4.18887147191072e-13 1.2739946038722e-12 100
"chr10" 99854001 99855000 "*" 4.01313771103418e-09 7.11882087237587e-09 100
"chr10" 99857001 99858000 "*" 1.78197812061853e-09 3.33284484311841e-09
65.9090909090909
"chr10" 99869001 99870000 "*" 0 0 -100
"chr10" 100030001 100031000 "*" 6.95824159846481e-05 6.68704966898533e-05 -52.5
"chr10" 100080001 100081000 "*" 3.46500605985511e-13 1.06777978272295e-12 100
"chr10" 100111001 100112000 "*" 6.70841160399505e-12 1.75486545176836e-11 100
"chr10" 100120001 100121000 "*" 6.59550958292954e-09 1.13456194382773e-08 100
"chr10" 100151001 100152000 "*" 9.61897228535236e-13 2.801372325071e-12 100
"chr10" 100182001 100183000 "*" 5.89841205486108e-05 5.73627738974644e-05
54.5454545454545
"chr10" 100215001 100216000 "*" 1.13140401492018e-08 1.87801380787728e-08 100
"chr10" 100226001 100227000 "*" 3.77475828372553e-15 1.47379557022071e-14 -100
"chr10" 100368001 100369000 "*" 3.84137166520304e-14 1.32929369817675e-13 -100
"chr10" 100963001 100964000 "*" 2.52449542825772e-07 3.47341761878397e-07
-66.6666666666667
"chr10" 101144001 101145000 "*" 4.88498130835069e-15 1.8824139250584e-14 100
"chr10" 101190001 101191000 "*" 0 0 100
"chr10" 101279001 101280000 "*" 0 0 83.3333333333333
"chr10" 101280001 101281000 "*" 0 0 54.0963295648134
"chr10" 101294001 101295000 "*" 0 0 51.6761928914205
"chr10" 101743001 101744000 "*" 0 0 -71.0526315789474
"chr10" 101863001 101864000 "*" 2.22044604925031e-16 9.81641919380259e-16 100
"chr10" 101871001 101872000 "*" 6.24654217240561e-10 1.2462108191303e-09 -100
"chr10" 102242001 102243000 "*" 1.19110258134381e-06 1.49658444109373e-06
63.6363636363636
"chr10" 102276001 102277000 "*" 3.95353005888666e-09 7.03928529283733e-09 100
"chr10" 102289001 102290000 "*" 4.44089209850063e-16 1.91071758245033e-15
-57.6923076923077
"chr10" 102321001 102322000 "*" 0 0 90.8026755852843
"chr10" 102327001 102328000 "*" 3.34799225010229e-08 5.20728221826256e-08
55.0403225806452
"chr10" 102374001 102375000 "*" 3.6700841921089e-08 5.65530226072258e-08 -100
"chr10" 102395001 102396000 "*" 6.92287338566189e-11 1.57605630124247e-10 100
"chr10" 102413001 102414000 "*" 0 0 62.501630789302
"chr10" 102414001 102415000 "*" 0 0 52.9711971667165

"chr10" 102416001 102417000 "*" 0 0 84.9187935034803
"chr10" 102429001 102430000 "*" 9.27180554555207e-12 2.38297394350244e-11
61.5384615384615
"chr10" 102483001 102484000 "*" 0 0 57.243816254417
"chr10" 102499001 102500000 "*" 0 0 58.6674669867947
"chr10" 102500001 102501000 "*" 0 0 50.8832551743591
"chr10" 102510001 102511000 "*" 0 0 70.1696386829627
"chr10" 102517001 102518000 "*" 5.19395637610387e-12 1.37849917832632e-11
64.9484536082474
"chr10" 102548001 102549000 "*" 9.99200722162641e-16 4.15382497462808e-15 100
"chr10" 102568001 102569000 "*" 4.08209022140227e-11 9.57770690255255e-11 100
"chr10" 102587001 102588000 "*" 0 0 77.710843373494
"chr10" 102588001 102589000 "*" 0 0 86.2337662337662
"chr10" 102619001 102620000 "*" 2.54241072639161e-14 8.9930794199802e-14
89.7959183673469
"chr10" 102639001 102640000 "*" 6.92287338566189e-11 1.57605630124247e-10 100
"chr10" 102759001 102760000 "*" 0 0 54.5454545454545
"chr10" 102774001 102775000 "*" 8.7349629751543e-09 1.47505871898511e-08 -100
"chr10" 102791001 102792000 "*" 0 0 -51.7006802721088
"chr10" 102810001 102811000 "*" 0 0 -54.5387453874539
"chr10" 102822001 102823000 "*" 0 0 -61.6279069767442
"chr10" 102890001 102891000 "*" 0 0 54.0588760035683
"chr10" 102947001 102948000 "*" 1.70641278884887e-12 4.81986032810646e-12 -100
"chr10" 102980001 102981000 "*" 1.92946192356658e-09 3.59193011611483e-09 100
"chr10" 103012001 103013000 "*" 0.000636154581132975 0.000521555309822028 -60
"chr10" 103032001 103033000 "*" 8.65973959207622e-14 2.87362558212404e-13 100
"chr10" 103052001 103053000 "*" 0 0 72.8104575163399
"chr10" 103318001 103319000 "*" 3.25312832227809e-09 5.86318817067582e-09 -80
"chr10" 103375001 103376000 "*" 1.43962619603144e-12 4.11184342893341e-12
-88.8888888888889
"chr10" 103427001 103428000 "*" 2.15795592506396e-08 3.43975819311611e-08
57.1428571428571
"chr10" 103438001 103439000 "*" 0.000808777120367532 0.000651834190530565
-58.3333333333333
"chr10" 103455001 103456000 "*" 0 0 -79.746835443038
"chr10" 103599001 103600000 "*" 0 0 -71.6101694915254
"chr10" 103812001 103813000 "*" 1.4432899320127e-15 5.90750815956055e-15 -100
"chr10" 103871001 103872000 "*" 0 0 100
"chr10" 103985001 103986000 "*" 2.16754614257297e-10 4.61395760603787e-10
-59.349593495935
"chr10" 103990001 103991000 "*" 0 0 56.8285393085008
"chr10" 104001001 104002000 "*" 0 0 56.9320568420478
"chr10" 104135001 104136000 "*" 3.03979064142368e-13 9.42350883309283e-13 100
"chr10" 104168001 104169000 "*" 0 0 65.4262762292187
"chr10" 104211001 104212000 "*" 9.0072393987839e-13 2.63157725646847e-12 -100
"chr10" 104212001 104213000 "*" 5.14477349611298e-13 1.54929660706642e-12
-86.3945578231292
"chr10" 104380001 104381000 "*" 3.95353005888666e-09 7.03928529283733e-09 100
"chr10" 104386001 104387000 "*" 2.00227675550835e-08 3.20459176617961e-08 100
"chr10" 104610001 104611000 "*" 1.07882591748876e-11 2.74553537455803e-11 -100
"chr10" 104678001 104679000 "*" 0 0 51.2043574098329
"chr10" 104948001 104949000 "*" 2.73014943985572e-11 6.58595916221891e-11 -100

"chr10" 104953001 104954000 "*" 1.82076576038526e-14 6.55981396322478e-14
-92.9936305732484
"chr10" 104954001 104955000 "*" 2.15125472990962e-10 4.58126659943874e-10 -100
"chr10" 105038001 105039000 "*" 1.10409141806933e-06 1.39342938100938e-06
71.4285714285714
"chr10" 105043001 105044000 "*" 1.42168360084405e-07 2.01687677516026e-07
63.6363636363636
"chr10" 105249001 105250000 "*" 9.63829016598083e-12 2.46993429847588e-11 100
"chr10" 105254001 105255000 "*" 0 0 74.5393012649869
"chr10" 105319001 105320000 "*" 0 0 78.5714285714286
"chr10" 105320001 105321000 "*" 0 0 100
"chr10" 105360001 105361000 "*" 3.40125705378114e-10 7.03784073980162e-10 100
"chr10" 105378001 105379000 "*" 1.87405646556726e-13 5.97285292681429e-13 100
"chr10" 105419001 105420000 "*" 5.35520627842345e-10 1.08035107045551e-09
52.3809523809524
"chr10" 105526001 105527000 "*" 1.96509475358653e-13 6.23868565133654e-13 100
"chr10" 105532001 105533000 "*" 5.11812814352197e-14 1.74475967443267e-13
-58.7737127371274
"chr10" 105606001 105607000 "*" 4.73234496034536e-09 8.30627300264826e-09 -100
"chr10" 105615001 105616000 "*" 0 0 76.7150395778364
"chr10" 105681001 105682000 "*" 1.92272353130019e-05 2.01632723352912e-05 62.5
"chr10" 105803001 105804000 "*" 8.25450818808804e-13 2.42473843346228e-12 100
"chr10" 105816001 105817000 "*" 1.49880108324396e-14 5.44951848293151e-14
53.2608695652174
"chr10" 105971001 105972000 "*" 0 0 92.9824561403509
"chr10" 106034001 106035000 "*" 0 0 -77.5
"chr10" 106055001 106056000 "*" 2.79987144580218e-12 7.68634726811898e-12 100
"chr10" 106090001 106091000 "*" 8.38194381952428e-05 7.95443938638605e-05
-51.4285714285714
"chr10" 106280001 106281000 "*" 1.52529544550362e-10 3.31234743496174e-10
76.9230769230769
"chr10" 106341001 106342000 "*" 2.15125472990962e-10 4.58126659943874e-10 -100
"chr10" 106440001 106441000 "*" 0 0 -75.0340798442064
"chr10" 106441001 106442000 "*" 1.96644922567657e-11 4.82930612417392e-11 -100
"chr10" 106601001 106602000 "*" 7.67056418382595e-11 1.7359448580318e-10 -100
"chr10" 106621001 106622000 "*" 3.40125705378114e-10 7.03784073980162e-10 100
"chr10" 106934001 106935000 "*" 1.28785870856518e-14 4.71440608755782e-14 -100
"chr10" 108027001 108028000 "*" 1.12804847240966e-07 1.62520779461317e-07 -60
"chr10" 108520001 108521000 "*" 4.01313771103418e-09 7.11882087237587e-09 -100
"chr10" 108973001 108974000 "*" 2.06501482580279e-14 7.3890370570066e-14
-73.9130434782609
"chr10" 109107001 109108000 "*" 2.16382467499443e-13 6.82775854166559e-13 100
"chr10" 109223001 109224000 "*" 0 0 69.5744680851064
"chr10" 110225001 110226000 "*" 2.78665979180914e-13 8.67360958590456e-13
-90.3846153846154
"chr10" 110327001 110328000 "*" 0 0 -100
"chr10" 110379001 110380000 "*" 3.6700841921089e-08 5.65530226072258e-08 100
"chr10" 110800001 110801000 "*" 0 0 -51.6916375652435
"chr10" 111327001 111328000 "*" 1.43679113950856e-06 1.78554011167915e-06
-81.25
"chr10" 111597001 111598000 "*" 0.000412523424090483 0.000349386048469569 52
"chr10" 112033001 112034000 "*" 2.00227675550835e-08 3.20459176617961e-08 -100

"chr10" 112113001 112114000 "*" 1.36557432028894e-14 4.98620244630348e-14
-90.3225806451613
"chr10" 112119001 112120000 "*" 3.33066907387547e-16 1.4495649018245e-15 -100
"chr10" 112125001 112126000 "*" 6.59550958292954e-09 1.13456194382773e-08 -100
"chr10" 112193001 112194000 "*" 3.33066907387547e-16 1.4495649018245e-15
74.5454545454545
"chr10" 112215001 112216000 "*" 9.77933302892531e-06 1.07364240648599e-05
68.8888888888889
"chr10" 112217001 112218000 "*" 3.68371999570627e-13 1.13149976091305e-12 -100
"chr10" 112220001 112221000 "*" 5.98831895182173e-09 1.03874786659479e-08
-85.5670103092783
"chr10" 112255001 112256000 "*" 0 0 -100
"chr10" 112257001 112258000 "*" 0 0 86.2823061630219
"chr10" 112290001 112291000 "*" 9.86988268891764e-14 3.2454957823005e-13
-52.2988505747126
"chr10" 112345001 112346000 "*" 6.33148844464415e-09 1.0949045142633e-08
71.4285714285714
"chr10" 112385001 112386000 "*" 0 0 -96.8253968253968
"chr10" 112404001 112405000 "*" 0 0 62.0700152207001
"chr10" 112418001 112419000 "*" 4.01313771103418e-09 7.11882087237587e-09 100
"chr10" 112493001 112494000 "*" 1.11022302462516e-16 5.03662826618488e-16 -100
"chr10" 112504001 112505000 "*" 1.29037891483108e-11 3.24582280286866e-11 -100
"chr10" 112589001 112590000 "*" 2.88657986402541e-15 1.14155613124573e-14 -100
"chr10" 112818001 112819000 "*" 7.16689363411405e-08 1.06003852859476e-07
-58.3333333333333
"chr10" 112844001 112845000 "*" 4.73234496034536e-09 8.30627300264826e-09 100
"chr10" 112845001 112846000 "*" 0 0 100
"chr10" 112856001 112857000 "*" 1.14124265593318e-11 2.89138062669327e-11 100
"chr10" 112899001 112900000 "*" 6.92287338566189e-11 1.57605630124247e-10 100
"chr10" 113046001 113047000 "*" 1.79320314153131e-09 3.35256095881909e-09
61.8181818181818
"chr10" 113842001 113843000 "*" 1.89581683684992e-12 5.32311367606535e-12 100
"chr10" 113880001 113881000 "*" 7.79172282250329e-11 1.76240809826282e-10
-85.7142857142857
"chr10" 114076001 114077000 "*" 4.86753970463383e-11 1.13145908161915e-10 100
"chr10" 114088001 114089000 "*" 8.01666433236647e-09 1.36392368970109e-08 -100
"chr10" 114596001 114597000 "*" 0 0 100
"chr10" 114755001 114756000 "*" 2.64951482975562e-09 4.82186011375752e-09 100
"chr10" 114872001 114873000 "*" 1.37584737158747e-07 1.96040848961799e-07
52.1739130434783
"chr10" 114940001 114941000 "*" 1.89581683684992e-12 5.32311367606535e-12 -100
"chr10" 115049001 115050000 "*" 4.02167188440217e-12 1.08266700497926e-11 100
"chr10" 115201001 115202000 "*" 2.79440914852103e-11 6.73046092948135e-11 -100
"chr10" 115345001 115346000 "*" 7.0006535457523e-08 1.03695832512054e-07 -100
"chr10" 115353001 115354000 "*" 2.34257058195908e-14 8.33889706074077e-14
52.6315789473684
"chr10" 115354001 115355000 "*" 0 0 87.5
"chr10" 115533001 115534000 "*" 1.39779633423487e-07 1.98463037958589e-07 -100
"chr10" 115580001 115581000 "*" 9.63829016598083e-12 2.46993429847588e-11 100
"chr10" 115743001 115744000 "*" 1.07882591748876e-11 2.74553537455803e-11 100
"chr10" 115835001 115836000 "*" 1.12458486967171e-09 2.15740815043925e-09 -100
"chr10" 115846001 115847000 "*" 1.56245072435723e-09 2.93840574091147e-09

-85.7142857142857
"chr10" 116056001 116057000 "*" 0 0 100
"chr10" 116063001 116064000 "*" 1.96047622580409e-10 4.21317985014898e-10
-51.6129032258064
"chr10" 116071001 116072000 "*" 2.00227675550835e-08 3.20459176617961e-08 100
"chr10" 116073001 116074000 "*" 6.26769901534985e-08 9.38373723255053e-08
-71.0743801652893
"chr10" 116108001 116109000 "*" 1.98325800226939e-11 4.85612402473442e-11 -100
"chr10" 116159001 116160000 "*" 2.08814465718632e-09 3.86232567960463e-09 -100
"chr10" 116163001 116164000 "*" 0 0 67.4641148325359
"chr10" 116245001 116246000 "*" 0 0 100
"chr10" 116298001 116299000 "*" 5.55111512312578e-15 2.12516350804551e-14 -60
"chr10" 116474001 116475000 "*" 3.61932706027801e-14 1.25777907157994e-13 -100
"chr10" 116528001 116529000 "*" 4.2499337382651e-13 1.29138862479083e-12
-52.1082327246254
"chr10" 116636001 116637000 "*" 1.11022302462516e-16 5.03662826618488e-16 100
"chr10" 116776001 116777000 "*" 2.15125472990962e-10 4.58126659943874e-10 100
"chr10" 116852001 116853000 "*" 0 0 64.1908066935271
"chr10" 116853001 116854000 "*" 0 0 66.4024071166928
"chr10" 116854001 116855000 "*" 0 0 75.3271028037383
"chr10" 117477001 117478000 "*" 0 0 -100
"chr10" 117709001 117710000 "*" 1.22124532708767e-15 5.03921984217778e-15 100
"chr10" 117731001 117732000 "*" 0 0 -76.9230769230769
"chr10" 117774001 117775000 "*" 1.99840144432528e-12 5.59476580335607e-12 -100
"chr10" 117809001 117810000 "*" 1.11022302462516e-15 4.59817122935606e-15
71.4285714285714
"chr10" 117830001 117831000 "*" 1.67299207820548e-08 2.71377429441055e-08 -100
"chr10" 117849001 117850000 "*" 1.29037891483108e-11 3.24582280286866e-11 -100
"chr10" 117855001 117856000 "*" 3.64548294884415e-08 5.64230896770432e-08
-66.6666666666667
"chr10" 117885001 117886000 "*" 0 0 -79.5275590551181
"chr10" 118025001 118026000 "*" 0 0 84.6153846153846
"chr10" 118034001 118035000 "*" 1.60375046576178e-11 3.98753559938014e-11
-50.3875968992248
"chr10" 118062001 118063000 "*" 3.04324343503026e-11 7.26599935673121e-11 -100
"chr10" 118084001 118085000 "*" 1.08912878715728e-13 3.56813842709972e-13
66.6666666666667
"chr10" 118239001 118240000 "*" 7.65188132856309e-08 1.12691547930703e-07
94.1176470588235
"chr10" 118371001 118372000 "*" 0 0 -83.8266384778013
"chr10" 118436001 118437000 "*" 1.95915915712774e-08 3.15124566891704e-08
52.1739130434783
"chr10" 118442001 118443000 "*" 1.90181204118289e-13 6.05298617539364e-13 -100
"chr10" 118465001 118466000 "*" 0 0 -70.5882352941177
"chr10" 118482001 118483000 "*" 3.26405569239796e-14 1.13973003849234e-13 100
"chr10" 118516001 118517000 "*" 1.02875828966731e-08 1.72239045994312e-08 -80
"chr10" 118521001 118522000 "*" 1.11022302462516e-16 5.03662826618488e-16 100
"chr10" 118547001 118548000 "*" 4.58850735185479e-11 1.07119583259697e-10
-66.6666666666667
"chr10" 118564001 118565000 "*" 2.22044604925031e-16 9.81641919380259e-16
63.8888888888889
"chr10" 118924001 118925000 "*" 0 0 100

"chr10" 118934001 118935000 "*" 0 0 62.015503875969
"chr10" 118943001 118944000 "*" 1.14352971536391e-13 3.73433475339321e-13 90
"chr10" 118969001 118970000 "*" 2.35972665851847e-08 3.74306455130309e-08
-59.2592592592593
"chr10" 118975001 118976000 "*" 0 0 83.399209486166
"chr10" 118990001 118991000 "*" 0 0 100
"chr10" 118995001 118996000 "*" 4.08209022140227e-11 9.57770690255255e-11 100
"chr10" 119001001 119002000 "*" 0 0 81.5304492345605
"chr10" 119014001 119015000 "*" 5.07219177769969e-09 8.87048018251838e-09
-60.5820105820106
"chr10" 119016001 119017000 "*" 4.34645670899236e-07 5.79023271110259e-07
51.7241379310345
"chr10" 119184001 119185000 "*" 1.88737914186277e-15 7.62011598380321e-15 100
"chr10" 119260001 119261000 "*" 1.39779633423487e-07 1.98463037958589e-07 -100
"chr10" 119302001 119303000 "*" 0 0 70.0593487743325
"chr10" 119380001 119381000 "*" 0 0 -100
"chr10" 119393001 119394000 "*" 3.95353005888666e-09 7.03928529283733e-09 -100
"chr10" 119427001 119428000 "*" 1.39779633423487e-07 1.98463037958589e-07 -100
"chr10" 119445001 119446000 "*" 3.20357620512413e-05 3.24835550088009e-05 -75
"chr10" 119482001 119483000 "*" 3.7274627828765e-12 1.00985250136599e-11 100
"chr10" 119645001 119646000 "*" 1.92946192356658e-09 3.59193011611483e-09 -100
"chr10" 120223001 120224000 "*" 2.41022546365599e-09 4.42480537812895e-09
-58.3333333333333
"chr10" 120367001 120368000 "*" 6.77335965093562e-12 1.76897011246462e-11 100
"chr10" 120428001 120429000 "*" 2.08814465718632e-09 3.86232567960463e-09 100
"chr10" 120430001 120431000 "*" 3.04324343503026e-11 7.26599935673121e-11 100
"chr10" 120515001 120516000 "*" 0 0 56.5573770491803
"chr10" 120535001 120536000 "*" 0.000233299213712068 0.000206023994887949 -55
"chr10" 120553001 120554000 "*" 9.5812247025151e-14 3.15667542866807e-13 100
"chr10" 120558001 120559000 "*" 1.39779633423487e-07 1.98463037958589e-07 -100
"chr10" 120559001 120560000 "*" 1.11022302462516e-15 4.59817122935606e-15 100
"chr10" 120719001 120720000 "*" 8.99170481860612e-06 9.9217343690715e-06
-54.8387096774194
"chr10" 120728001 120729000 "*" 5.04263297784746e-13 1.52078941532818e-12 100
"chr10" 120739001 120740000 "*" 1.87627691161651e-14 6.74814792086625e-14
-55.2380952380952
"chr10" 120741001 120742000 "*" 2.91028312560115e-11 6.97590507139978e-11 100
"chr10" 120786001 120787000 "*" 1.15169651593305e-09 2.2071346337551e-09
71.830985915493
"chr10" 120805001 120806000 "*" 1.29037891483108e-11 3.24582280286866e-11 -100
"chr10" 120847001 120848000 "*" 3.95353005888666e-09 7.03928529283733e-09 100
"chr10" 120965001 120966000 "*" 0 0 100
"chr10" 121074001 121075000 "*" 6.36234953610426e-10 1.26819108887702e-09
62.0689655172414
"chr10" 121205001 121206000 "*" 6.04438721296674e-11 1.38849255306602e-10 100
"chr10" 121368001 121369000 "*" 5.48638023900594e-10 1.10374357336421e-09 100
"chr10" 121633001 121634000 "*" 2.66453525910038e-15 1.05861327033776e-14
-52.0989974937343
"chr10" 121856001 121857000 "*" 3.76365605347928e-13 1.15464976278974e-12
59.375
"chr10" 121864001 121865000 "*" 7.7715611723761e-16 3.26213507634405e-15 100
"chr10" 121887001 121888000 "*" 0 0 74.3424317617866

"chr10" 122394001 122395000 "*" 1.26840350089807e-06 1.58815979843119e-06 58.7092731829574
"chr10" 122649001 122650000 "*" 4.99020824662466e-11 1.15790792586157e-10 100
"chr10" 122703001 122704000 "*" 0 0 -100
"chr10" 122749001 122750000 "*" 1.48780487929656e-09 2.81548293447651e-09 -76.5432098765432
"chr10" 123031001 123032000 "*" 3.85360321381034e-05 3.85553082302386e-05 -65.2173913043478
"chr10" 123060001 123061000 "*" 9.08108033215171e-11 2.03407753381119e-10 100
"chr10" 123179001 123180000 "*" 5.23923820117123e-06 5.99234928575461e-06 -68.1818181818182
"chr10" 123253001 123254000 "*" 0 0 -100
"chr10" 123270001 123271000 "*" 4.99020824662466e-11 1.15790792586157e-10 100
"chr10" 123277001 123278000 "*" 0 0 -66.1764705882353
"chr10" 123330001 123331000 "*" 1.65688228799254e-05 1.75642699767807e-05 67.5
"chr10" 123333001 123334000 "*" 0.000925065456011231 0.000738090308567342 -53.7878787878788
"chr10" 123340001 123341000 "*" 0 0 59.375
"chr10" 123376001 123377000 "*" 1.88737914186277e-15 7.62011598380321e-15 100
"chr10" 123377001 123378000 "*" 3.52704532247117e-11 8.35581292290093e-11 -100
"chr10" 123407001 123408000 "*" 5.01025332333427e-10 1.01358031135889e-09 -100
"chr10" 123422001 123423000 "*" 0 0 -54.4117647058824
"chr10" 123432001 123433000 "*" 1.11022302462516e-16 5.03662826618488e-16 -54.2857142857143
"chr10" 123458001 123459000 "*" 5.23923820117123e-06 5.99234928575461e-06 68.1818181818182
"chr10" 123494001 123495000 "*" 0 0 -100
"chr10" 123735001 123736000 "*" 1.72084568816899e-14 6.21243744137267e-14 81.4285714285714
"chr10" 123781001 123782000 "*" 2.72706959236757e-08 4.29495807172736e-08 80
"chr10" 123873001 123874000 "*" 0 0 -63.0695443645084
"chr10" 123900001 123901000 "*" 3.46104949811021e-07 4.67482227931052e-07 51.5151515151515
"chr10" 123901001 123902000 "*" 1.48487888651516e-11 3.70689457767456e-11 100
"chr10" 123948001 123949000 "*" 0 0 100
"chr10" 123985001 123986000 "*" 0 0 98.6666666666667
"chr10" 124028001 124029000 "*" 6.66133814775094e-16 2.81595744474255e-15 100
"chr10" 124034001 124035000 "*" 5.55111512312578e-16 2.36485094870365e-15 100
"chr10" 124049001 124050000 "*" 7.105427357601e-15 2.68362283258525e-14 100
"chr10" 124097001 124098000 "*" 1.33226762955019e-15 5.48133041560118e-15 -100
"chr10" 124218001 124219000 "*" 2.88710388929303e-10 6.03399264731139e-10 100
"chr10" 124223001 124224000 "*" 0 0 74.5762711864407
"chr10" 124224001 124225000 "*" 2.63075117246103e-11 6.36292794319057e-11 86.2068965517241
"chr10" 124235001 124236000 "*" 1.34559030584569e-13 4.35581312463895e-13 100
"chr10" 124271001 124272000 "*" 0.000141752611761192 0.000129667082920112 -55.2238805970149
"chr10" 124304001 124305000 "*" 1.26397892152852e-09 2.41117847690952e-09 76.9230769230769
"chr10" 124306001 124307000 "*" 4.09383971167188e-10 8.39098267966717e-10 -89.622641509434
"chr10" 124323001 124324000 "*" 1.5277158427196e-09 2.87642564232045e-09 100

"chr10" 124337001 124338000 "*" 0 0 77.2727272727273
"chr10" 124339001 124340000 "*" 5.52236589790311e-10 1.11050095193809e-09 66.40625
"chr10" 124447001 124448000 "*" 1.40781186708239e-09 2.67223111923349e-09 -54.5454545454545
"chr10" 124488001 124489000 "*" 2.30926389122033e-14 8.22700632271376e-14 78.2608695652174
"chr10" 124527001 124528000 "*" 1.27184721643303e-08 2.09920728853124e-08 -52.8255528255528
"chr10" 124539001 124540000 "*" 1.43600098478913e-09 2.72263987861654e-09 77.6699029126214
"chr10" 124561001 124562000 "*" 1.17767795515533e-10 2.60403951073233e-10 83.8983050847458
"chr10" 124859001 124860000 "*" 3.77475828372553e-15 1.47379557022071e-14 100
"chr10" 124866001 124867000 "*" 1.14124265593318e-11 2.89138062669327e-11 100
"chr10" 124879001 124880000 "*" 4.71134242729931e-12 1.257615036806e-11 100
"chr10" 124899001 124900000 "*" 0 0 64.0759930915371
"chr10" 124906001 124907000 "*" 4.41983707007987e-05 4.38318975644086e-05 -62.5190839694657
"chr10" 125034001 125035000 "*" 0 0 100
"chr10" 125041001 125042000 "*" 2.23653187492179e-06 2.70311306704149e-06 -66.6666666666667
"chr10" 125150001 125151000 "*" 0 0 100
"chr10" 125154001 125155000 "*" 0 0 100
"chr10" 125155001 125156000 "*" 0 0 100
"chr10" 125195001 125196000 "*" 8.77076189453874e-15 3.27889038038365e-14 100
"chr10" 125202001 125203000 "*" 3.05311331771918e-12 8.34639368772922e-12 100
"chr10" 125206001 125207000 "*" 2.35932436143393e-07 3.25820146356964e-07 70.5882352941177
"chr10" 125233001 125234000 "*" 1.11022302462516e-16 5.03662826618488e-16 80.3571428571429
"chr10" 125252001 125253000 "*" 0 0 79.2207792207792
"chr10" 125255001 125256000 "*" 4.01313771103418e-09 7.11882087237587e-09 100
"chr10" 125275001 125276000 "*" 6.57587648589253e-06 7.40204991240724e-06 55
"chr10" 125317001 125318000 "*" 1.35036426485158e-12 3.86629547363774e-12 -100
"chr10" 125330001 125331000 "*" 2.08814465718632e-09 3.86232567960463e-09 100
"chr10" 125393001 125394000 "*" 1.11022302462516e-16 5.03662826618488e-16 67.6470588235294
"chr10" 125503001 125504000 "*" 0 0 -100
"chr10" 125514001 125515000 "*" 1.48487888651516e-11 3.70689457767456e-11 -100
"chr10" 125667001 125668000 "*" 0 0 -90.6976744186046
"chr10" 125722001 125723000 "*" 0 0 100
"chr10" 125728001 125729000 "*" 8.43769498715119e-15 3.16245570171727e-14 -62.280701754386
"chr10" 125729001 125730000 "*" 5.76601100199525e-10 1.15695738313808e-09 56.5972222222222
"chr10" 125758001 125759000 "*" 3.52704532247117e-11 8.35581292290093e-11 100
"chr10" 125771001 125772000 "*" 6.60113408379459e-10 1.30965383680779e-09 53.3333333333333
"chr10" 125776001 125777000 "*" 1.4432899320127e-15 5.90750815956055e-15 56.9767441860465
"chr10" 125787001 125788000 "*" 1.55806589852148e-10 3.38069521194183e-10 60

"chr10" 125793001 125794000 "*" 2.38452912970644e-09 4.38023416065507e-09 71.4285714285714
"chr10" 125810001 125811000 "*" 9.71063229826541e-11 2.16781365050398e-10 -100
"chr10" 125843001 125844000 "*" 1.11022302462516e-16 5.03662826618488e-16 100
"chr10" 125849001 125850000 "*" 2.88710388929303e-10 6.03399264731139e-10 100
"chr10" 125866001 125867000 "*" 2.88710388929303e-10 6.03399264731139e-10 -100
"chr10" 125923001 125924000 "*" 8.08496325443997e-09 1.37487066796077e-08 50.695134061569
"chr10" 125958001 125959000 "*" 1.41579290036109e-06 1.76122957885034e-06 60.7142857142857
"chr10" 125988001 125989000 "*" 5.57964785485865e-12 1.47649370162352e-11 83.3333333333333
"chr10" 125997001 125998000 "*" 0 0 100
"chr10" 126011001 126012000 "*" 1.98365768255826e-11 4.85612402473442e-11 -100
"chr10" 126023001 126024000 "*" 8.7349629751543e-09 1.47505871898511e-08 100
"chr10" 126027001 126028000 "*" 7.0006535457523e-08 1.03695832512054e-07 100
"chr10" 126030001 126031000 "*" 2.22044604925031e-16 9.81641919380259e-16 100
"chr10" 126052001 126053000 "*" 2.98606583903904e-10 6.23067230856289e-10 86.4197530864197
"chr10" 126064001 126065000 "*" 2.98649993624167e-14 1.04825628627798e-13 73.2142857142857
"chr10" 126220001 126221000 "*" 4.43011537168969e-05 4.39265220551362e-05 63.8888888888889
"chr10" 126300001 126301000 "*" 3.58757468177373e-12 9.74359218740287e-12 73.6842105263158
"chr10" 126315001 126316000 "*" 2.38353757842091e-07 3.28978743080408e-07 72.2222222222222
"chr10" 126399001 126400000 "*" 1.81099579776856e-12 5.10299046364922e-12 -78
"chr10" 126421001 126422000 "*" 1.11022302462516e-16 5.03662826618488e-16 75
"chr10" 126482001 126483000 "*" 3.63445940010365e-11 8.58565209111204e-11 100
"chr10" 126733001 126734000 "*" 0 0 -100
"chr10" 126745001 126746000 "*" 6.31272811801864e-13 1.87896475668436e-12 -62.9699248120301
"chr10" 126750001 126751000 "*" 4.41291688746404e-08 6.74202392576976e-08 -51.1857707509881
"chr10" 126751001 126752000 "*" 4.83380002691547e-12 1.28906532984872e-11 90
"chr10" 126755001 126756000 "*" 0 0 65.8536585365854
"chr10" 126772001 126773000 "*" 8.08115796502307e-11 1.8255483805226e-10 -64.7058823529412
"chr10" 126826001 126827000 "*" 0 0 100
"chr10" 126837001 126838000 "*" 0 0 54.1176470588235
"chr10" 126877001 126878000 "*" 1.11382901392121e-05 1.21160977238229e-05 66.6666666666667
"chr10" 126905001 126906000 "*" 1.12843068222901e-12 3.259512533505e-12 -100
"chr10" 126950001 126951000 "*" 3.597027969926e-07 4.84612690151399e-07 92.8571428571429
"chr10" 126960001 126961000 "*" 2.11424519735992e-06 2.56432546771576e-06 -54.5454545454545
"chr10" 126988001 126989000 "*" 1.93720595120794e-11 4.76309924836636e-11 -100
"chr10" 127203001 127204000 "*" 1.56863411149288e-12 4.4469621057462e-12 100
"chr10" 127222001 127223000 "*" 1.40595757258666e-10 3.06957700166362e-10 54.8387096774194

"chr10" 127231001 127232000 "*" 4.32209823486573e-12 1.15935290479641e-11 -100
"chr10" 127232001 127233000 "*" 6.17506046296512e-13 1.84147154303754e-12
76.9230769230769
"chr10" 127264001 127265000 "*" 4.44089209850063e-15 1.71914916534614e-14 100
"chr10" 127267001 127268000 "*" 6.92526036516483e-11 1.57651365092085e-10
-72.2222222222222
"chr10" 127269001 127270000 "*" 4.99900121297969e-12 1.32921056339624e-11 100
"chr10" 127327001 127328000 "*" 0 0 100
"chr10" 127349001 127350000 "*" 6.77335965093562e-12 1.76897011246462e-11 100
"chr10" 127678001 127679000 "*" 8.11055667071514e-11 1.83159426809419e-10
88.8888888888889
"chr10" 127702001 127703000 "*" 3.95353005888666e-09 7.03928529283733e-09 -100
"chr10" 127751001 127752000 "*" 0 0 -100
"chr10" 127792001 127793000 "*" 3.6700841921089e-08 5.65530226072258e-08 -100
"chr10" 127793001 127794000 "*" 2.68450373042128e-10 5.64465213127486e-10 100
"chr10" 127812001 127813000 "*" 2.88657986402541e-15 1.14155613124573e-14
51.8518518518519
"chr10" 127863001 127864000 "*" 1.8125231115329e-05 1.90935684500041e-05
53.8461538461538
"chr10" 127924001 127925000 "*" 0 0 81.25
"chr10" 127938001 127939000 "*" 0 0 100
"chr10" 127947001 127948000 "*" 3.86282006381578e-10 7.9401066099168e-10
94.7368421052632
"chr10" 127959001 127960000 "*" 5.72875080706581e-14 1.93967931988877e-13 100
"chr10" 127980001 127981000 "*" 2.02327044007689e-12 5.65300384792621e-12 100
"chr10" 128151001 128152000 "*" 0 0 86.8421052631579
"chr10" 128208001 128209000 "*" 5.14294751230437e-09 8.98856693399752e-09
58.6206896551724
"chr10" 128224001 128225000 "*" 1.40303574980294e-07 1.99179817877998e-07
69.6969696969697
"chr10" 128487001 128488000 "*" 2.27584839862516e-10 4.83462411214229e-10
-85.5072463768116
"chr10" 128530001 128531000 "*" 2.72646106247443e-07 3.73405126957647e-07
66.6666666666667
"chr10" 128548001 128549000 "*" 6.60582699651968e-14 2.22176301799e-13 100
"chr10" 128559001 128560000 "*" 6.16805551856281e-07 8.06313781087357e-07
73.1707317073171
"chr10" 128560001 128561000 "*" 1.77980741256079e-10 3.84134306400813e-10
66.6666666666667
"chr10" 128581001 128582000 "*" 2.88657986402541e-15 1.14155613124573e-14 100
"chr10" 128591001 128592000 "*" 0 0 100
"chr10" 128783001 128784000 "*" 0 0 -80.8219178082192
"chr10" 128833001 128834000 "*" 1.55431223447522e-14 5.6388152448458e-14 -100
"chr10" 128837001 128838000 "*" 3.56714459415208e-06 4.1798126523543e-06
-51.1363636363636
"chr10" 128920001 128921000 "*" 4.88498130835069e-15 1.8824139250584e-14 -100
"chr10" 129013001 129014000 "*" 0 0 53.3333333333333
"chr10" 129225001 129226000 "*" 3.33066907387547e-16 1.4495649018245e-15
97.3684210526316
"chr10" 129237001 129238000 "*" 2.88710388929303e-10 6.03399264731139e-10 100
"chr10" 129352001 129353000 "*" 9.0072393987839e-13 2.63157725646847e-12 -100
"chr10" 129632001 129633000 "*" 6.04438721296674e-11 1.38849255306602e-10 100

"chr10" 129647001 129648000 "*" 2.22044604925031e-16 9.81641919380259e-16 -100
"chr10" 129649001 129650000 "*" 0 0 80.6451612903226
"chr10" 129650001 129651000 "*" 1.46929405098195e-05 1.56951838495452e-05
52.9411764705882
"chr10" 129670001 129671000 "*" 8.59101678685192e-12 2.2138012905973e-11
54.5454545454545
"chr10" 129671001 129672000 "*" 0 0 93.8461538461538
"chr10" 129704001 129705000 "*" 0 0 90.1234567901235
"chr10" 129707001 129708000 "*" 8.57092175010621e-13 2.51149198099779e-12 -100
"chr10" 129740001 129741000 "*" 1.52794177310511e-09 2.87642564232045e-09 100
"chr10" 129777001 129778000 "*" 0 0 -100
"chr10" 129790001 129791000 "*" 4.01313771103418e-09 7.11882087237587e-09 -100
"chr10" 129811001 129812000 "*" 5.7065463465733e-14 1.93318145576218e-13 100
"chr10" 129850001 129851000 "*" 8.25450818808804e-13 2.42473843346228e-12 100
"chr10" 129882001 129883000 "*" 1.52794177310511e-09 2.87642564232045e-09 -100
"chr10" 130067001 130068000 "*" 7.0006535457523e-08 1.03695832512054e-07 100
"chr10" 130122001 130123000 "*" 0 0 100
"chr10" 130123001 130124000 "*" 0 0 -100
"chr10" 130157001 130158000 "*" 2.08814465718632e-09 3.86232567960463e-09 100
"chr10" 130167001 130168000 "*" 5.63660229602192e-13 1.68905058935478e-12 100
"chr10" 130182001 130183000 "*" 2.22044604925031e-16 9.81641919380259e-16 100
"chr10" 130198001 130199000 "*" 2.69084754478399e-12 7.41487347763769e-12 100
"chr10" 130227001 130228000 "*" 1.12132525487141e-14 4.14352271980498e-14 100
"chr10" 130229001 130230000 "*" 0 0 -100
"chr10" 130232001 130233000 "*" 8.97060203897126e-14 2.96780698277594e-13
71.830985915493
"chr10" 130259001 130260000 "*" 4.73234496034536e-09 8.30627300264826e-09 100
"chr10" 130260001 130261000 "*" 3.47530892952364e-11 8.24871042481877e-11
-54.6052631578947
"chr10" 130267001 130268000 "*" 7.38969840874404e-09 1.26393223618283e-08
58.8235294117647
"chr10" 130271001 130272000 "*" 3.63015936200028e-08 5.62046932076955e-08
51.8518518518519
"chr10" 130286001 130287000 "*" 0 0 -100
"chr10" 130316001 130317000 "*" 6.54132303878896e-12 1.71481392009472e-11
-60.7843137254902
"chr10" 130364001 130365000 "*" 0 0 -66.6666666666667
"chr10" 130366001 130367000 "*" 0 0 100
"chr10" 130409001 130410000 "*" 7.0006535457523e-08 1.03695832512054e-07 100
"chr10" 130522001 130523000 "*" 0 0 -100
"chr10" 130526001 130527000 "*" 9.65729496371637e-10 1.87255912493232e-09 100
"chr10" 130541001 130542000 "*" 2.38575468822777e-05 2.46816531377115e-05
66.6666666666667
"chr10" 130666001 130667000 "*" 1.37354119189581e-08 2.2573164010542e-08
65.9574468085106
"chr10" 130721001 130722000 "*" 2.33146835171283e-15 9.32818831833246e-15 100
"chr10" 130730001 130731000 "*" 1.17964038426344e-06 1.48285273216832e-06
76.7441860465116
"chr10" 130833001 130834000 "*" 1.11022302462516e-13 3.63439078563186e-13
-66.1016949152542
"chr10" 130874001 130875000 "*" 2.66855949559996e-08 4.20800812311105e-08
64.5640074211503

"chr10" 130898001 130899000 "*" 5.55111512312578e-16 2.36485094870365e-15 -84.375
"chr10" 130911001 130912000 "*" 7.0006535457523e-08 1.03695832512054e-07 100
"chr10" 131040001 131041000 "*" 1.17461596005342e-13 3.8279067575877e-13 -100
"chr10" 131043001 131044000 "*" 6.66133814775094e-16 2.81595744474255e-15 100
"chr10" 131076001 131077000 "*" 2.15218347587864e-08 3.43114716982286e-08 54.1125541125541
"chr10" 131114001 131115000 "*" 7.0006535457523e-08 1.03695832512054e-07 -100
"chr10" 131158001 131159000 "*" 0.000148679623408254 0.000135545631461674 51.2820512820513
"chr10" 131160001 131161000 "*" 0 0 100
"chr10" 131171001 131172000 "*" 7.93809462606987e-14 2.64679416473686e-13 100
"chr10" 131223001 131224000 "*" 0 0 -67.5925925925926
"chr10" 131232001 131233000 "*" 0.000223064486262303 0.000197655528181121 -53.125
"chr10" 131269001 131270000 "*" 1.52422983346057e-07 2.1540961543467e-07 75
"chr10" 131279001 131280000 "*" 1.14124265593318e-11 2.89138062669327e-11 -100
"chr10" 131300001 131301000 "*" 1.14130926931466e-13 3.7276674571693e-13 100
"chr10" 131304001 131305000 "*" 3.7274627828765e-12 1.00985250136599e-11 -100
"chr10" 131307001 131308000 "*" 1.76489045600192e-10 3.81090594900825e-10 78.4313725490196
"chr10" 131330001 131331000 "*" 3.15636405900932e-13 9.77000305862078e-13 -82.0512820512821
"chr10" 131354001 131355000 "*" 0 0 77.1428571428571
"chr10" 131448001 131449000 "*" 0 0 -100
"chr10" 131516001 131517000 "*" 4.8884896131085e-11 1.13620221515027e-10 56.3380281690141
"chr10" 131525001 131526000 "*" 1.81489157036197e-10 3.91412743121759e-10 94.1176470588235
"chr10" 131529001 131530000 "*" 6.90747459231034e-12 1.80140148623876e-11 100
"chr10" 131572001 131573000 "*" 6.66133814775094e-16 2.81595744474255e-15 100
"chr10" 131579001 131580000 "*" 0 0 100
"chr10" 131631001 131632000 "*" 0 0 75
"chr10" 131645001 131646000 "*" 3.83510710333379e-06 4.47018399725337e-06 52.7777777777778
"chr10" 131648001 131649000 "*" 1.11022302462516e-16 5.03662826618488e-16 -100
"chr10" 131660001 131661000 "*" 1.94671057052176e-10 4.18489066862024e-10 69.1176470588235
"chr10" 131664001 131665000 "*" 4.34121769643525e-07 5.78408567117475e-07 -71.4285714285714
"chr10" 131672001 131673000 "*" 2.88710388929303e-10 6.03399264731139e-10 100
"chr10" 131685001 131686000 "*" 3.96462775770212e-05 3.95891713722014e-05 58.3333333333333
"chr10" 131687001 131688000 "*" 0 0 -100
"chr10" 131688001 131689000 "*" 0 0 100
"chr10" 131719001 131720000 "*" 0 0 100
"chr10" 131725001 131726000 "*" 0 0 -100
"chr10" 131730001 131731000 "*" 8.43347613965761e-12 2.17859001990012e-11 100
"chr10" 131732001 131733000 "*" 2.22044604925031e-16 9.81641919380259e-16 100
"chr10" 131762001 131763000 "*" 0 0 72.9271240268683
"chr10" 131764001 131765000 "*" 0 0 81.1320754716981
"chr10" 131812001 131813000 "*" 0 0 68.9320388349515

"chr10" 131815001 131816000 "*" 9.1699505433418e-08 1.33679473255088e-07 91.6666666666667
"chr10" 131820001 131821000 "*" 1.78718817522849e-09 3.3417648909279e-09 58.6206896551724
"chr10" 131821001 131822000 "*" 0 0 100
"chr10" 131872001 131873000 "*" 1.0769163338864e-14 3.98631098593833e-14 -100
"chr10" 131991001 131992000 "*" 0 0 -71.4285714285714
"chr10" 132019001 132020000 "*" 5.41788836017076e-14 1.84152775539899e-13 79.2452830188679
"chr10" 132030001 132031000 "*" 1.68753899743024e-14 6.09826805967439e-14 -100
"chr10" 132060001 132061000 "*" 0 0 -55.1181102362205
"chr10" 132071001 132072000 "*" 2.00227675550835e-08 3.20459176617961e-08 -100
"chr10" 132133001 132134000 "*" 0 0 -100
"chr10" 132167001 132168000 "*" 4.17169709443499e-07 5.5713945238012e-07 -77.9661016949153
"chr10" 132173001 132174000 "*" 4.99900121297969e-12 1.32921056339624e-11 100
"chr10" 132229001 132230000 "*" 3.65631191989735e-08 5.65530226072258e-08 -55.5555555555556
"chr10" 132252001 132253000 "*" 0 0 55.9101654846336
"chr10" 132262001 132263000 "*" 1.17563885293492e-08 1.94773738020317e-08 68.5714285714286
"chr10" 132268001 132269000 "*" 4.44089209850063e-16 1.91071758245033e-15 100
"chr10" 132280001 132281000 "*" 1.85021421517106e-08 2.98453000964175e-08 -67.6470588235294
"chr10" 132282001 132283000 "*" 1.67299207820548e-08 2.71377429441055e-08 100
"chr10" 132291001 132292000 "*" 2.31563546115865e-09 4.25940344033825e-09 -90.3846153846154
"chr10" 132310001 132311000 "*" 1.20591092667155e-10 2.66075324129686e-10 -100
"chr10" 132368001 132369000 "*" 4.86753970463383e-11 1.13145908161915e-10 -100
"chr10" 132446001 132447000 "*" 2.36924924124082e-11 5.75866400531606e-11 86.2068965517241
"chr10" 132475001 132476000 "*" 4.91828799908944e-14 1.68192819018269e-13 -100
"chr10" 132527001 132528000 "*" 1.11022302462516e-15 4.59817122935606e-15 100
"chr10" 132540001 132541000 "*" 0 0 -84.2105263157895
"chr10" 132556001 132557000 "*" 4.03360781053852e-05 4.02351074444744e-05 -58.0645161290323
"chr10" 132573001 132574000 "*" 5.55111512312578e-16 2.36485094870365e-15 100
"chr10" 132577001 132578000 "*" 3.4284545671337e-06 4.02641014303533e-06 -52.1739130434783
"chr10" 132586001 132587000 "*" 0 0 100
"chr10" 132589001 132590000 "*" 3.56775498033812e-10 7.35898629809829e-10 100
"chr10" 132608001 132609000 "*" 0 0 -60.655737704918
"chr10" 132634001 132635000 "*" 8.71525074330748e-14 2.88848349331391e-13 -100
"chr10" 132637001 132638000 "*" 3.6892711108294e-13 1.13266255090498e-12 -100
"chr10" 132682001 132683000 "*" 0 0 -100
"chr10" 132687001 132688000 "*" 2.62900812231237e-13 8.21294938531841e-13 -100
"chr10" 132728001 132729000 "*" 0 0 69.2307692307692
"chr10" 132729001 132730000 "*" 4.77395900588817e-15 1.84201484337652e-14 -62.1212121212121
"chr10" 132791001 132792000 "*" 4.99900121297969e-12 1.32921056339624e-11 -100
"chr10" 132797001 132798000 "*" 4.68514116391816e-14 1.60932470540441e-13 83.8709677419355

"chr10" 132810001 132811000 "*" 6.15840711759574e-13 1.83676803572802e-12 100
"chr10" 132831001 132832000 "*" 0 0 -100
"chr10" 132877001 132878000 "*" 0 0 78.2608695652174
"chr10" 132884001 132885000 "*" 1.55431223447522e-14 5.6388152448458e-14 -100
"chr10" 132910001 132911000 "*" 0 0 -72
"chr10" 132957001 132958000 "*" 0 0 52.8337236533958
"chr10" 132958001 132959000 "*" 4.82301134585583e-08 7.33042782554184e-08
57.3770491803279
"chr10" 132977001 132978000 "*" 0 0 100
"chr10" 132994001 132995000 "*" 0 0 50.9803921568627
"chr10" 133012001 133013000 "*" 4.73234496034536e-09 8.30627300264826e-09 100
"chr10" 133017001 133018000 "*" 0 0 -73.6111111111111
"chr10" 133045001 133046000 "*" 3.57842807219289e-06 4.19279847255992e-06
-61.4285714285714
"chr10" 133051001 133052000 "*" 2.94128055244869e-11 7.04817975111009e-11
-83.3256563795486
"chr10" 133075001 133076000 "*" 0 0 100
"chr10" 133114001 133115000 "*" 2.16382467499443e-13 6.82775854166559e-13 100
"chr10" 133142001 133143000 "*" 2.91330993351657e-05 2.97351770785225e-05 -56
"chr10" 133147001 133148000 "*" 1.70344255479016e-07 2.39410014315768e-07
-72.972972972973
"chr10" 133171001 133172000 "*" 1.10460185442918e-09 2.12523217987942e-09 75
"chr10" 133174001 133175000 "*" 0 0 84.8
"chr10" 133190001 133191000 "*" 0 0 -100
"chr10" 133223001 133224000 "*" 3.95353005888666e-09 7.03928529283733e-09 100
"chr10" 133229001 133230000 "*" 1.87510396266743e-05 1.97080033495427e-05
-66.6666666666667
"chr10" 133273001 133274000 "*" 2.32200592087395e-10 4.92853465422976e-10
-87.719298245614
"chr10" 133292001 133293000 "*" 7.12914709044554e-07 9.23469497775327e-07
-56.5217391304348
"chr10" 133343001 133344000 "*" 0 0 77.2727272727273
"chr10" 133353001 133354000 "*" 1.08213438210214e-12 3.13370700285384e-12
55.1932367149758
"chr10" 133355001 133356000 "*" 0 0 -77.5700934579439
"chr10" 133358001 133359000 "*" 0 0 -59.3220338983051
"chr10" 133375001 133376000 "*" 6.37412345128041e-12 1.67524765514662e-11 90
"chr10" 133454001 133455000 "*" 0 0 100
"chr10" 133486001 133487000 "*" 2.22044604925031e-16 9.81641919380259e-16
95.4545454545455
"chr10" 133495001 133496000 "*" 0 0 96
"chr10" 133500001 133501000 "*" 1.14352971536391e-14 4.21921674335999e-14 -100
"chr10" 133540001 133541000 "*" 0 0 56.8627450980392
"chr10" 133564001 133565000 "*" 6.4324989779152e-11 1.47230020825092e-10 100
"chr10" 133570001 133571000 "*" 4.45909498214547e-09 7.8732217500796e-09
69.5652173913043
"chr10" 133595001 133596000 "*" 5.59352619777798e-09 9.73696874145814e-09
-51.8518518518519
"chr10" 133605001 133606000 "*" 6.59550958292954e-09 1.13456194382773e-08 100
"chr10" 133606001 133607000 "*" 0 0 58.743961352657
"chr10" 133637001 133638000 "*" 2.00227675550835e-08 3.20459176617961e-08 100
"chr10" 133663001 133664000 "*" 1.70974345792274e-14 6.17369338139741e-14

-85.0574712643678
"chr10" 133666001 133667000 "*" 5.79959180679879e-08 8.72614105767205e-08 70.7317073170732
"chr10" 133675001 133676000 "*" 0 0 100
"chr10" 133765001 133766000 "*" 3.530509218308e-14 1.22891039008692e-13 100
"chr10" 133869001 133870000 "*" 3.53310225520431e-09 6.34187411210968e-09 56.5217391304348
"chr10" 133914001 133915000 "*" 4.07229805432507e-13 1.24220948757928e-12 -67.4157303370787
"chr10" 133915001 133916000 "*" 0 0 -54.1866028708134
"chr10" 133936001 133937000 "*" 3.78436175729746e-08 5.82257329712716e-08 -67.5438596491228
"chr10" 133939001 133940000 "*" 9.65729496371637e-10 1.87255912493232e-09 100
"chr10" 133943001 133944000 "*" 0 0 91.4285714285714
"chr10" 133966001 133967000 "*" 0 0 100
"chr10" 133991001 133992000 "*" 4.83434240527991e-06 5.5546183284725e-06 51.5151515151515
"chr10" 134011001 134012000 "*" 4.74065231514942e-14 1.62745413342582e-13 53.1929824561403
"chr10" 134013001 134014000 "*" 2.17549311898324e-11 5.30550448494011e-11 57.5342465753425
"chr10" 134025001 134026000 "*" 6.59550958292954e-09 1.13456194382773e-08 100
"chr10" 134035001 134036000 "*" 6.41637853959764e-11 1.47017896707011e-10 50.2653655196028
"chr10" 134048001 134049000 "*" 1.62360409518003e-05 1.72320836269876e-05 -55.5555555555556
"chr10" 134087001 134088000 "*" 8.62063320727913e-08 1.26143381606989e-07 -76.1904761904762
"chr10" 134121001 134122000 "*" 0 0 76.1321909424725
"chr10" 134125001 134126000 "*" 1.63417335219407e-09 3.06796318526677e-09 -53.7313432835821
"chr10" 134127001 134128000 "*" 2.25101378081849e-05 2.3372357989545e-05 -56
"chr10" 134133001 134134000 "*" 5.40034683638169e-12 1.43090827942664e-11 -66.6666666666667
"chr10" 134191001 134192000 "*" 0 0 100
"chr10" 134193001 134194000 "*" 3.03090885722668e-14 1.06210046699299e-13 -52.2820512820513
"chr10" 134201001 134202000 "*" 0 0 80.2197802197802
"chr10" 134205001 134206000 "*" 5.1281201507436e-13 1.54461625452838e-12 100
"chr10" 134211001 134212000 "*" 2.8966828935495e-12 7.94035734501975e-12 -64.4628099173554
"chr10" 134213001 134214000 "*" 3.5527136788005e-15 1.39277426410519e-14 55.5555555555556
"chr10" 134261001 134262000 "*" 0 0 -87.2679045092838
"chr10" 134312001 134313000 "*" 3.87851270866602e-06 4.51736814105762e-06 81.8181818181818
"chr10" 134320001 134321000 "*" 2.82773804372027e-13 8.79129664109438e-13 100
"chr10" 134325001 134326000 "*" 0 0 -100
"chr10" 134341001 134342000 "*" 7.7715611723761e-16 3.26213507634405e-15 82.8571428571429
"chr10" 134385001 134386000 "*" 1.11022302462516e-16 5.03662826618488e-16 -58.3054626532887

"chr10" 134389001 134390000 "*" 0 0 -100
"chr10" 134449001 134450000 "*" 5.48006084954977e-13 1.64669751139903e-12
66.6666666666667
"chr10" 134531001 134532000 "*" 0 0 100
"chr10" 134601001 134602000 "*" 0 0 57.9607067609138
"chr10" 134602001 134603000 "*" 0 0 58.5380702849379
"chr10" 134627001 134628000 "*" 0 0 -55.1417004048583
"chr10" 134645001 134646000 "*" 6.04438721296674e-11 1.38849255306602e-10 100
"chr10" 134655001 134656000 "*" 3.7274627828765e-12 1.00985250136599e-11 100
"chr10" 134696001 134697000 "*" 0 0 65.9391665020739
"chr10" 134700001 134701000 "*" 0 0 95.8333333333333
"chr10" 134766001 134767000 "*" 6.59550958292954e-09 1.13456194382773e-08 100
"chr10" 134787001 134788000 "*" 0 0 -52.2051282051282
"chr10" 134799001 134800000 "*" 1.0483280705742e-06 1.32725038313729e-06
54.1742286751361
"chr10" 134841001 134842000 "*" 1.00882455722484e-08 1.69091746438156e-08
-83.8709677419355
"chr10" 134849001 134850000 "*" 9.04570973681018e-10 1.76414389822173e-09 100
"chr10" 134851001 134852000 "*" 3.57708107578958e-09 6.41627394521332e-09
-55.1724137931034
"chr10" 134906001 134907000 "*" 2.38209452163574e-11 5.78735560918541e-11
-63.4146341463415
"chr10" 134925001 134926000 "*" 1.63199453950824e-11 4.05413522907475e-11
55.625
"chr10" 134964001 134965000 "*" 6.30585006433648e-09 1.09081013492641e-08
-71.4285714285714
"chr10" 134973001 134974000 "*" 0 0 -64.7058823529412
"chr10" 135005001 135006000 "*" 1.46549439250521e-14 5.33330076973935e-14 -100
"chr10" 135046001 135047000 "*" 0 0 100
"chr10" 135050001 135051000 "*" 0 0 53.2720398990033
"chr10" 135059001 135060000 "*" 8.00445265625171e-11 1.80856342217256e-10
56.8542568542569
"chr10" 135060001 135061000 "*" 0 0 -84.5549738219895
"chr10" 135169001 135170000 "*" 5.55111512312578e-16 2.36485094870365e-15 100
"chr10" 135186001 135187000 "*" 0 0 -59.3406593406593
"chr10" 135187001 135188000 "*" 0 0 -100
"chr10" 135217001 135218000 "*" 0 0 -94.1520467836257
"chr10" 135234001 135235000 "*" 3.33066907387547e-16 1.4495649018245e-15
63.6363636363636
"chr10" 135244001 135245000 "*" 4.18509116251187e-10 8.56791005535884e-10
57.1428571428571
"chr10" 135252001 135253000 "*" 3.32729332974679e-09 5.99038474059895e-09
-66.6666666666667
"chr10" 135257001 135258000 "*" 1.83304482703761e-11 4.52714667747443e-11
92.3076923076923
"chr10" 135348001 135349000 "*" 5.47647851512068e-06 6.24246074187317e-06
71.1111111111111
"chr10" 135404001 135405000 "*" 1.12458486967171e-09 2.15740815043925e-09 100
"chr10" 135426001 135427000 "*" 9.04570973681018e-10 1.76414389822173e-09 -100
"chr10" 135438001 135439000 "*" 1.41585265733823e-09 2.68657653467126e-09
55.8479532163743
"chr11" 187001 188000 "*" 0 0 -95.679012345679

"chr11" 193001 194000 "*" 0 0 75.5905511811024
"chr11" 194001 195000 "*" 0 0 65.5263157894737
"chr11" 202001 203000 "*" 3.69553054824223e-10 7.61181060967148e-10
55.1724137931034
"chr11" 259001 260000 "*" 6.04438721296674e-11 1.38849255306602e-10 100
"chr11" 313001 314000 "*" 9.63382706942184e-11 2.15239597116267e-10
71.4285714285714
"chr11" 320001 321000 "*" 0 0 -100
"chr11" 321001 322000 "*" 3.54922704115523e-07 4.78613366753507e-07
-52.7272727272727
"chr11" 368001 369000 "*" 9.82131853621837e-09 1.64839671385566e-08
59.0163934426229
"chr11" 369001 370000 "*" 0 0 81.782334384858
"chr11" 390001 391000 "*" 1.3949399857438e-08 2.29108424825089e-08
50.6024096385542
"chr11" 444001 445000 "*" 0 0 -100
"chr11" 445001 446000 "*" 2.67753066385268e-07 3.67210293236612e-07
-63.1578947368421
"chr11" 449001 450000 "*" 0 0 -56.5776736924278
"chr11" 481001 482000 "*" 3.33066907387547e-16 1.4495649018245e-15 100
"chr11" 509001 510000 "*" 1.98474570112239e-12 5.5612019076505e-12
-62.7906976744186
"chr11" 526001 527000 "*" 5.19910781093813e-11 1.20390153275629e-10
-55.7496360989811
"chr11" 527001 528000 "*" 8.69304628281498e-14 2.88410421981304e-13
-66.6666666666667
"chr11" 547001 548000 "*" 2.82075643731616e-07 3.85584889618316e-07
84.2105263157895
"chr11" 606001 607000 "*" 0 0 -58.206691199191
"chr11" 650001 651000 "*" 5.21499377015289e-09 9.11009415448405e-09
-93.3333333333333
"chr11" 699001 700000 "*" 3.88169574261354e-09 6.93447853396753e-09
-66.6666666666667
"chr11" 706001 707000 "*" 0 0 -68.5890880373489
"chr11" 728001 729000 "*" 2.1830970364789e-10 4.64540017336377e-10
-89.4039735099338
"chr11" 730001 731000 "*" 3.20273041154451e-06 3.77989342157269e-06
-53.5714285714286
"chr11" 745001 746000 "*" 0 0 -100
"chr11" 776001 777000 "*" 8.93226825504634e-07 1.14160391339096e-06
-76.9230769230769
"chr11" 796001 797000 "*" 0 0 -84.4249084249084
"chr11" 815001 816000 "*" 1.33898878196259e-05 1.43911695897696e-05
62.962962962963
"chr11" 834001 835000 "*" 1.88763449315843e-11 4.65493886320087e-11
-52.3375834851245
"chr11" 841001 842000 "*" 0 0 -99.009900990099
"chr11" 911001 912000 "*" 0 0 -79.0243902439024
"chr11" 941001 942000 "*" 6.92287338566189e-11 1.57605630124247e-10 100
"chr11" 1054001 1055000 "*" 1.26553976742549e-07 1.81087584534879e-07 71.875
"chr11" 1070001 1071000 "*" 5.07371922253697e-14 1.73287898516782e-13 100
"chr11" 1091001 1092000 "*" 0 0 86.4197530864197

"chr11" 1111001 1112000 "*" 0 0 100
"chr11" 1114001 1115000 "*" 0 0 76.1904761904762
"chr11" 1116001 1117000 "*" 4.42277339196551e-08 6.75621687967191e-08
72.0338983050847
"chr11" 1134001 1135000 "*" 0 0 -100
"chr11" 1160001 1161000 "*" 0 0 84.3373493975904
"chr11" 1377001 1378000 "*" 0 0 89.9159663865546
"chr11" 1408001 1409000 "*" 0 0 -56.140350877193
"chr11" 1440001 1441000 "*" 0 0 100
"chr11" 1454001 1455000 "*" 6.92287338566189e-11 1.57605630124247e-10 100
"chr11" 1462001 1463000 "*" 1.12492475334847e-08 1.87365463358949e-08
59.6153846153846
"chr11" 1533001 1534000 "*" 1.15296661107323e-11 2.92016820876116e-11
55.3735024665257
"chr11" 1575001 1576000 "*" 0.000361273490986247 0.000308833981928707
52.3809523809524
"chr11" 1627001 1628000 "*" 4.27954982562539e-09 7.56742156628225e-09
90.9090909090909
"chr11" 1678001 1679000 "*" 3.15216716617606e-07 4.28122559072529e-07
81.8181818181818
"chr11" 1714001 1715000 "*" 0 0 -56.8885859274407
"chr11" 1721001 1722000 "*" 2.09973149978282e-11 5.12816915084879e-11
60.7594936708861
"chr11" 1737001 1738000 "*" 0 0 -100
"chr11" 1754001 1755000 "*" 0 0 88.5869565217391
"chr11" 1770001 1771000 "*" 0 0 50.2583812637882
"chr11" 1822001 1823000 "*" 1.88261159994596e-08 3.03420719188995e-08
-94.4444444444444
"chr11" 1829001 1830000 "*" 0 0 100
"chr11" 1832001 1833000 "*" 9.20205661136819e-06 1.01420076485268e-05 60.9375
"chr11" 1848001 1849000 "*" 0 0 -71.6535433070866
"chr11" 1852001 1853000 "*" 0 0 51.7110964235882
"chr11" 1879001 1880000 "*" 8.8770301109875e-09 1.4977150394023e-08
-60.4395604395604
"chr11" 1897001 1898000 "*" 0 0 -58.4187697160883
"chr11" 1905001 1906000 "*" 0 0 100
"chr11" 1909001 1910000 "*" 2.00227675550835e-08 3.20459176617961e-08 100
"chr11" 1916001 1917000 "*" 3.10862446895044e-15 1.22466437093774e-14
63.6363636363636
"chr11" 1923001 1924000 "*" 1.16573417585641e-14 4.29335582900508e-14 -100
"chr11" 1943001 1944000 "*" 3.46722650590436e-11 8.23046357215889e-11
-50.4761904761905
"chr11" 1955001 1956000 "*" 2.88657986402541e-15 1.14155613124573e-14
53.1914893617021
"chr11" 2008001 2009000 "*" 1.9373391779709e-13 6.15971322898373e-13 -100
"chr11" 2047001 2048000 "*" 1.25122134875255e-13 4.06357992793329e-13
-65.8508158508158
"chr11" 2093001 2094000 "*" 0.000146916150864596 0.000134059546618682
-54.6666666666667
"chr11" 2112001 2113000 "*" 9.1699505433418e-08 1.33679473255088e-07
93.3333333333333
"chr11" 2149001 2150000 "*" 8.60046860173824e-07 1.10151582611715e-06

69.3548387096774
"chr11" 2160001 2161000 "*" 0 0 67.2304860247481
"chr11" 2177001 2178000 "*" 0 0 -50.4522648576072
"chr11" 2182001 2183000 "*" 1.11345116793338e-07 1.6052031035535e-07
71.0843373493976
"chr11" 2200001 2201000 "*" 0 0 -71.0858585858586
"chr11" 2226001 2227000 "*" 0 0 72.9885057471264
"chr11" 2232001 2233000 "*" 4.04388723362903e-06 4.69732188124514e-06
59.8198198198198
"chr11" 2237001 2238000 "*" 6.982525668775e-12 1.82000658619443e-11 -78.125
"chr11" 2238001 2239000 "*" 0 0 -80.6383508439974
"chr11" 2284001 2285000 "*" 4.03031179485591e-06 4.68311825421348e-06
54.7619047619048
"chr11" 2290001 2291000 "*" 0 0 64.7798742138365
"chr11" 2311001 2312000 "*" 1.27009514017118e-13 4.12294955270516e-13
-62.1951219512195
"chr11" 2329001 2330000 "*" 0 0 65.2173913043478
"chr11" 2330001 2331000 "*" 3.34830736470337e-08 5.20767521184995e-08
-50.5444832545716
"chr11" 2346001 2347000 "*" 8.25450818808804e-13 2.42473843346228e-12 -100
"chr11" 2350001 2351000 "*" 5.87913273619733e-10 1.17866290330822e-09
82.1052631578947
"chr11" 2367001 2368000 "*" 1.39779633423487e-07 1.98463037958589e-07 -100
"chr11" 2368001 2369000 "*" 8.3882012447134e-11 1.88922989503423e-10 100
"chr11" 2384001 2385000 "*" 7.4451556031363e-13 2.20001121864162e-12
-58.294930875576
"chr11" 2396001 2397000 "*" 2.71562928921831e-07 3.71994900847726e-07
58.4158415841584
"chr11" 2410001 2411000 "*" 1.11022302462516e-15 4.59817122935606e-15
68.1818181818182
"chr11" 2444001 2445000 "*" 0 0 100
"chr11" 2448001 2449000 "*" 0 0 59.7826086956522
"chr11" 2449001 2450000 "*" 2.44249065417534e-15 9.75180348030994e-15
-52.6193870277976
"chr11" 2456001 2457000 "*" 1.15727342153704e-08 1.91891148635522e-08
85.7142857142857
"chr11" 2461001 2462000 "*" 3.04324343503026e-11 7.26599935673121e-11 -100
"chr11" 2496001 2497000 "*" 9.99200722162641e-16 4.15382497462808e-15 100
"chr11" 2520001 2521000 "*" 5.08650899178065e-11 1.17900668424206e-10 60
"chr11" 2726001 2727000 "*" 1.02140518265514e-13 3.35337968436403e-13
-65.7657657657658
"chr11" 2741001 2742000 "*" 1.07634446333549e-06 1.36049041286493e-06
63.6363636363636
"chr11" 2766001 2767000 "*" 0 0 75.6756756756757
"chr11" 2789001 2790000 "*" 1.5277158427196e-09 2.87642564232045e-09 100
"chr11" 2845001 2846000 "*" 3.99709487730604e-10 8.20216270012864e-10
52.2727272727273
"chr11" 2882001 2883000 "*" 7.57374707394121e-09 1.29387284589791e-08 88
"chr11" 2900001 2901000 "*" 1.0636353062754e-06 1.34530286878699e-06
52.1739130434783
"chr11" 2901001 2902000 "*" 9.3319242466805e-07 1.18962595148735e-06
62.0689655172414

"chr11" 2913001 2914000 "*" 3.33066907387547e-16 1.4495649018245e-15
70.446735395189
"chr11" 2927001 2928000 "*" 9.65729496371637e-10 1.87255912493232e-09 100
"chr11" 2934001 2935000 "*" 0 0 -67.6910299003322
"chr11" 2954001 2955000 "*" 0 0 -84.5070422535211
"chr11" 3007001 3008000 "*" 0 0 100
"chr11" 3152001 3153000 "*" 0 0 100
"chr11" 3189001 3190000 "*" 3.6427075800205e-09 6.5252880340617e-09
72.2222222222222
"chr11" 3190001 3191000 "*" 1.22014620629329e-11 3.08180024257396e-11
-55.9299191374663
"chr11" 3191001 3192000 "*" 3.67483821150927e-14 1.27627245811088e-13
61.5600086188321
"chr11" 3215001 3216000 "*" 0 0 -58.9473684210526
"chr11" 3218001 3219000 "*" 0 0 89.6551724137931
"chr11" 3236001 3237000 "*" 2.2573304221396e-06 2.7265120493757e-06
90.9090909090909
"chr11" 3237001 3238000 "*" 1.51592506325571e-08 2.47861525308496e-08
69.1176470588235
"chr11" 3252001 3253000 "*" 2.31651631210639e-08 3.67879115279518e-08
52.1646859083192
"chr11" 3376001 3377000 "*" 7.88258347483861e-15 2.96145301916012e-14
57.6923076923077
"chr11" 3388001 3389000 "*" 0 0 -100
"chr11" 3602001 3603000 "*" 0 0 58.4821428571429
"chr11" 3627001 3628000 "*" 6.60582699651968e-14 2.22176301799e-13 100
"chr11" 3690001 3691000 "*" 2.92902573195697e-07 3.99494321134604e-07
56.8627450980392
"chr11" 3819001 3820000 "*" 0 0 64.491858506457
"chr11" 4189001 4190000 "*" 1.67299207820548e-08 2.71377429441055e-08 100
"chr11" 4233001 4234000 "*" 0 0 52.983603154836
"chr11" 4245001 4246000 "*" 9.65729496371637e-10 1.87255912493232e-09 100
"chr11" 4517001 4518000 "*" 1.52794177310511e-09 2.87642564232045e-09 100
"chr11" 4663001 4664000 "*" 1.29037891483108e-11 3.24582280286866e-11 100
"chr11" 5058001 5059000 "*" 8.7349629751543e-09 1.47505871898511e-08 -100
"chr11" 5166001 5167000 "*" 1.96644922567657e-11 4.82930612417392e-11 100
"chr11" 5335001 5336000 "*" 4.71134242729931e-12 1.257615036806e-11 100
"chr11" 5443001 5444000 "*" 3.90831811358794e-12 1.05610567215048e-11 100
"chr11" 5482001 5483000 "*" 0 0 83.8150289017341
"chr11" 5616001 5617000 "*" 2.66453525910038e-15 1.05861327033776e-14 -100
"chr11" 5617001 5618000 "*" 0 0 -100
"chr11" 5674001 5675000 "*" 1.74853465040314e-11 4.32752710900609e-11 -100
"chr11" 6099001 6100000 "*" 0 0 -100
"chr11" 6136001 6137000 "*" 0.000173919515143783 0.000156793955027816 60
"chr11" 6519001 6520000 "*" 9.62896429257398e-13 2.80291205576417e-12 -100
"chr11" 6619001 6620000 "*" 6.4152538836737e-10 1.27477697728417e-09 100
"chr11" 6632001 6633000 "*" 1.78830027453003e-08 2.89047247595699e-08
-82.4427480916031
"chr11" 6646001 6647000 "*" 2.83445384940961e-08 4.45608721148354e-08
-55.8265582655827
"chr11" 6672001 6673000 "*" 5.55111512312578e-16 2.36485094870365e-15 100
"chr11" 6910001 6911000 "*" 9.65729496371637e-10 1.87255912493232e-09 -100

"chr11" 7041001 7042000 "*" 0 0 71.3498622589532
"chr11" 7368001 7369000 "*" 1.35036426485158e-12 3.86629547363774e-12 -100
"chr11" 7533001 7534000 "*" 4.02167188440217e-12 1.08266700497926e-11 100
"chr11" 7559001 7560000 "*" 0 0 -100
"chr11" 7598001 7599000 "*" 0 0 -90.7407407407407
"chr11" 7687001 7688000 "*" 3.08364290768637e-08 4.82618915528272e-08
-82.9268292682927
"chr11" 7882001 7883000 "*" 4.46405532761318e-05 4.42311628182131e-05
52.3809523809524
"chr11" 8008001 8009000 "*" 1.11022302462516e-16 5.03662826618488e-16 100
"chr11" 8042001 8043000 "*" 4.05156765747039e-05 4.03939637160074e-05
55.2631578947368
"chr11" 8090001 8091000 "*" 1.04471986617227e-13 3.42749557361457e-13
-52.3830834638622
"chr11" 8093001 8094000 "*" 3.5853098268035e-11 8.48637259564962e-11
93.3333333333333
"chr11" 8107001 8108000 "*" 5.96128786334305e-08 8.95279496227116e-08
51.6507703595011
"chr11" 8118001 8119000 "*" 0 0 -100
"chr11" 8122001 8123000 "*" 7.67056418382595e-11 1.7359448580318e-10 -100
"chr11" 8123001 8124000 "*" 0 0 100
"chr11" 8244001 8245000 "*" 5.51850422814137e-06 6.28718511088478e-06
69.4444444444444
"chr11" 8254001 8255000 "*" 0 0 -100
"chr11" 8258001 8259000 "*" 6.59550958292954e-09 1.13456194382773e-08 100
"chr11" 8265001 8266000 "*" 3.95353005888666e-09 7.03928529283733e-09 100
"chr11" 8273001 8274000 "*" 2.06246131284615e-12 5.7592229204274e-12
53.8461538461538
"chr11" 8314001 8315000 "*" 4.44089209850063e-15 1.71914916534614e-14
-64.5161290322581
"chr11" 8318001 8319000 "*" 6.92287338566189e-11 1.57605630124247e-10 -100
"chr11" 8334001 8335000 "*" 3.90831811358794e-12 1.05610567215048e-11 -100
"chr11" 8357001 8358000 "*" 1.11022302462516e-16 5.03662826618488e-16
57.4468085106383
"chr11" 8375001 8376000 "*" 9.87358834704244e-05 9.26448142584499e-05
-53.7313432835821
"chr11" 8384001 8385000 "*" 3.52704532247117e-11 8.35581292290093e-11 100
"chr11" 8394001 8395000 "*" 1.22457599616155e-12 3.52441478267078e-12 100
"chr11" 8404001 8405000 "*" 7.23269108293678e-06 8.09603765127355e-06
-65.7142857142857
"chr11" 8615001 8616000 "*" 0 0 58.7598010550871
"chr11" 8623001 8624000 "*" 3.72374575619006e-10 7.66765403560727e-10 80
"chr11" 8853001 8854000 "*" 1.52100632089258e-09 2.8746597910115e-09
66.6666666666667
"chr11" 8864001 8865000 "*" 4.26951363152739e-10 8.72510873516957e-10 -100
"chr11" 8879001 8880000 "*" 9.99200722162641e-16 4.15382497462808e-15 -100
"chr11" 8959001 8960000 "*" 0 0 100
"chr11" 9055001 9056000 "*" 9.08108033215171e-11 2.03407753381119e-10 100
"chr11" 9094001 9095000 "*" 1.37001521238744e-13 4.42851918810123e-13 100
"chr11" 9164001 9165000 "*" 4.44089209850063e-15 1.71914916534614e-14 100
"chr11" 9594001 9595000 "*" 0 0 -77.3913043478261
"chr11" 9635001 9636000 "*" 0 0 -86.1777150916784

"chr11" 9685001 9686000 "*" 0 0 68.2352941176471
"chr11" 9686001 9687000 "*" 0 0 -100
"chr11" 9777001 9778000 "*" 1.11022302462516e-16 5.03662826618488e-16 -100
"chr11" 9780001 9781000 "*" 0 0 -72.0529645494751
"chr11" 9912001 9913000 "*" 6.51096655479577e-08 9.72647149904707e-08
58.3333333333333
"chr11" 10325001 10326000 "*" 6.66133814775094e-16 2.81595744474255e-15 -100
"chr11" 10407001 10408000 "*" 1.52466927971773e-12 4.33322475981408e-12 100
"chr11" 10416001 10417000 "*" 4.75841588354342e-11 1.10912272200105e-10
-74.2268041237113
"chr11" 10464001 10465000 "*" 1.07882591748876e-11 2.74553537455803e-11 -100
"chr11" 10509001 10510000 "*" 3.32505045719245e-09 5.98673443123549e-09
-75.7142857142857
"chr11" 10511001 10512000 "*" 4.78841410966879e-11 1.11468187156588e-10 -100
"chr11" 10670001 10671000 "*" 1.88737914186277e-15 7.62011598380321e-15 -100
"chr11" 10680001 10681000 "*" 3.39935207760433e-07 4.59748212171356e-07
-63.3333333333333
"chr11" 10724001 10725000 "*" 2.69018141096922e-12 7.41487347763769e-12 100
"chr11" 10814001 10815000 "*" 2.22044604925031e-16 9.81641919380259e-16
-51.6129032258064
"chr11" 11165001 11166000 "*" 1.00808250635964e-13 3.31107370956809e-13 100
"chr11" 11174001 11175000 "*" 0.000496254900866688 0.000414667937798419
-54.1666666666667
"chr11" 11184001 11185000 "*" 5.6621374255883e-15 2.16468580166064e-14 -100
"chr11" 11311001 11312000 "*" 5.48638023900594e-10 1.10374357336421e-09 -100
"chr11" 11313001 11314000 "*" 4.01313771103418e-09 7.11882087237587e-09 -100
"chr11" 11410001 11411000 "*" 2.73625566649116e-12 7.52483363579137e-12 100
"chr11" 11426001 11427000 "*" 8.01666433236647e-09 1.36392368970109e-08 100
"chr11" 11450001 11451000 "*" 1.17794662912729e-13 3.83653245040640e-13 100
"chr11" 11468001 11469000 "*" 4.78841410966879e-11 1.11468187156588e-10 -100
"chr11" 11522001 11523000 "*" 6.15840711759574e-13 1.83676803572802e-12 100
"chr11" 11576001 11577000 "*" 0 0 -100
"chr11" 11594001 11595000 "*" 1.37828859436695e-10 3.01445544267661e-10 100
"chr11" 11642001 11643000 "*" 0 0 77.0956734790304
"chr11" 11643001 11644000 "*" 0 0 74.6494066882416
"chr11" 11644001 11645000 "*" 0.00020976332305167 0.000186660769941092
-52.3809523809524
"chr11" 11997001 11998000 "*" 5.28789737668234e-05 5.17954580201457e-05
57.6923076923077
"chr11" 12006001 12007000 "*" 8.96610757861183e-08 1.30861118541309e-07 75
"chr11" 12142001 12143000 "*" 9.2861067158001e-07 1.18407475076477e-06
59.4594594594595
"chr11" 12289001 12290000 "*" 1.004450997244e-05 1.1004376066266e-05
-83.3333333333333
"chr11" 12309001 12310000 "*" 6.66133814775094e-16 2.81595744474255e-15
85.0931677018634
"chr11" 12546001 12547000 "*" 0 0 53.8461538461538
"chr11" 13136001 13137000 "*" 4.81571154065108e-07 6.37949233437604e-07 87.5
"chr11" 13156001 13157000 "*" 2.00227675550835e-08 3.20459176617961e-08 100
"chr11" 13205001 13206000 "*" 7.0006535457523e-08 1.03695832512054e-07 100
"chr11" 13953001 13954000 "*" 2.43005615629954e-09 4.45014222280278e-09 -100
"chr11" 13984001 13985000 "*" 0 0 58.9821693907875

"chr11" 14076001 14077000 "*" 2.1094237467878e-15 8.47434540879246e-15 100
"chr11" 14226001 14227000 "*" 1.11022302462516e-15 4.59817122935606e-15 -100
"chr11" 14282001 14283000 "*" 4.44089209850063e-16 1.91071758245033e-15 -100
"chr11" 14558001 14559000 "*" 5.55111512312578e-16 2.36485094870365e-15 -100
"chr11" 15199001 15200000 "*" 1.52794177310511e-09 2.87642564232045e-09 100
"chr11" 15394001 15395000 "*" 3.23373829846929e-06 3.81325174359463e-06 66.6666666666667
"chr11" 15434001 15435000 "*" 1.48214773787458e-13 4.77488235285846e-13 100
"chr11" 15591001 15592000 "*" 0 0 -100
"chr11" 15613001 15614000 "*" 1.13140401492018e-08 1.87801380787728e-08 100
"chr11" 15671001 15672000 "*" 0 0 -100
"chr11" 15805001 15806000 "*" 2.33028818463765e-10 4.93719283454501e-10 100
"chr11" 15920001 15921000 "*" 1.67299207820548e-08 2.71377429441055e-08 -100
"chr11" 15932001 15933000 "*" 0 0 -86.3970588235294
"chr11" 15957001 15958000 "*" 1.39761168738151e-06 1.74009179262251e-06 57.5757575757576
"chr11" 16340001 16341000 "*" 8.02691246803988e-14 2.67352274101148e-13 100
"chr11" 16505001 16506000 "*" 2.00227675550835e-08 3.20459176617961e-08 -100
"chr11" 16847001 16848000 "*" 1.2421974560084e-10 2.73697911880706e-10 -87.6623376623377
"chr11" 16901001 16902000 "*" 8.43347613965761e-12 2.17859001990012e-11 -100
"chr11" 16946001 16947000 "*" 0 0 -60.4026845637584
"chr11" 17003001 17004000 "*" 2.37587727269783e-14 8.44268514944447e-14 -56.1016949152542
"chr11" 17103001 17104000 "*" 0 0 100
"chr11" 17460001 17461000 "*" 2.88710388929303e-10 6.03399264731139e-10 -100
"chr11" 17462001 17463000 "*" 5.48638023900594e-10 1.10374357336421e-09 -100
"chr11" 17491001 17492000 "*" 8.25450818808804e-13 2.42473843346228e-12 -100
"chr11" 17528001 17529000 "*" 3.56775498033812e-10 7.35898629809829e-10 -100
"chr11" 17547001 17548000 "*" 6.48370246381091e-14 2.18368252806999e-13 -81.2925170068027
"chr11" 17613001 17614000 "*" 4.26951363152739e-10 8.72510873516957e-10 -100
"chr11" 17633001 17634000 "*" 0 0 100
"chr11" 17675001 17676000 "*" 0 0 -100
"chr11" 17707001 17708000 "*" 3.84137166520304e-14 1.32929369817675e-13 100
"chr11" 17755001 17756000 "*" 0 0 100
"chr11" 17763001 17764000 "*" 0 0 92.1985815602837
"chr11" 17766001 17767000 "*" 1.85331353597107e-08 2.98924036132437e-08 59.6774193548387
"chr11" 17767001 17768000 "*" 0 0 72.7496310870635
"chr11" 18742001 18743000 "*" 0 0 -58.6319218241042
"chr11" 18874001 18875000 "*" 1.12458486967171e-09 2.15740815043925e-09 100
"chr11" 18994001 18995000 "*" 0 0 -51.0869565217391
"chr11" 19094001 19095000 "*" 3.95353005888666e-09 7.03928529283733e-09 100
"chr11" 19111001 19112000 "*" 0 0 -88.1818181818182
"chr11" 19352001 19353000 "*" 8.43347613965761e-12 2.17859001990012e-11 100
"chr11" 19428001 19429000 "*" 9.04570973681018e-10 1.76414389822173e-09 100
"chr11" 19597001 19598000 "*" 2.64951482975562e-09 4.82186011375752e-09 100
"chr11" 19691001 19692000 "*" 2.4535928844216e-14 8.69144396197119e-14 100
"chr11" 19698001 19699000 "*" 1.4432899320127e-15 5.90750815956055e-15 100
"chr11" 19733001 19734000 "*" 0 0 -69.1943127962085
"chr11" 19798001 19799000 "*" 0 0 66.8877833056938

"chr11" 19860001 19861000 "*" 3.34128893442198e-08 5.19772864618523e-08 100
"chr11" 19891001 19892000 "*" 0 0 -100
"chr11" 19979001 19980000 "*" 4.44089209850063e-16 1.91071758245033e-15 -100
"chr11" 19990001 19991000 "*" 8.3882012447134e-11 1.88922989503423e-10 100
"chr11" 20134001 20135000 "*" 0 0 -100
"chr11" 20183001 20184000 "*" 1.12458486967171e-09 2.15740815043925e-09 100
"chr11" 20260001 20261000 "*" 1.5277158427196e-09 2.87642564232045e-09 -100
"chr11" 20298001 20299000 "*" 0 0 51.7241379310345
"chr11" 20385001 20386000 "*" 0 0 -65.4654255319149
"chr11" 20648001 20649000 "*" 1.0320741705705e-08 1.72756056497744e-08 -60
"chr11" 20778001 20779000 "*" 8.01666433236647e-09 1.36392368970109e-08 -100
"chr11" 20905001 20906000 "*" 4.73234496034536e-09 8.30627300264826e-09 -100
"chr11" 21538001 21539000 "*" 0 0 -100
"chr11" 21773001 21774000 "*" 6.59550958292954e-09 1.13456194382773e-08 -100
"chr11" 21832001 21833000 "*" 0 0 -83.3333333333333
"chr11" 22317001 22318000 "*" 0 0 100
"chr11" 22337001 22338000 "*" 1.11022302462516e-16 5.03662826618488e-16
78.858024691358
"chr11" 22359001 22360000 "*" 1.10234044115032e-12 3.1900055895149e-12
57.3033707865169
"chr11" 22364001 22365000 "*" 1.44564747206477e-06 1.79577013319455e-06
70.7317073170732
"chr11" 22528001 22529000 "*" 1.11022302462516e-16 5.03662826618488e-16 100
"chr11" 22588001 22589000 "*" 1.96509475358653e-13 6.23868565133654e-13 -100
"chr11" 23225001 23226000 "*" 6.08402217494586e-14 2.05454787937634e-13 -100
"chr11" 23701001 23702000 "*" 5.01337193981044e-10 1.0141620111296e-09 -65
"chr11" 24349001 24350000 "*" 0 0 100
"chr11" 26291001 26292000 "*" 0 0 100
"chr11" 26353001 26354000 "*" 0 0 53.0401910067648
"chr11" 26608001 26609000 "*" 9.67004254448511e-14 3.18178990782609e-13 100
"chr11" 26904001 26905000 "*" 0.000354677012256444 0.00030353099993751
-51.7241379310345
"chr11" 27164001 27165000 "*" 3.772968241611e-06 4.40310894836477e-06 71.875
"chr11" 27253001 27254000 "*" 0 0 -100
"chr11" 27403001 27404000 "*" 1.67299207820548e-08 2.71377429441055e-08 -100
"chr11" 27631001 27632000 "*" 2.08814465718632e-09 3.86232567960463e-09 100
"chr11" 27721001 27722000 "*" 0 0 76.4179104477612
"chr11" 27740001 27741000 "*" 0 0 73.0805817881023
"chr11" 28526001 28527000 "*" 1.11022302462516e-16 5.03662826618488e-16 100
"chr11" 29778001 29779000 "*" 0 0 -97.6744186046512
"chr11" 29955001 29956000 "*" 0 0 -82.967032967033
"chr11" 29997001 29998000 "*" 1.11022302462516e-16 5.03662826618488e-16 -100
"chr11" 30033001 30034000 "*" 4.79616346638068e-14 1.64380445416596e-13 -100
"chr11" 30034001 30035000 "*" 8.65973959207622e-15 3.24130830964561e-14 -100
"chr11" 30200001 30201000 "*" 1.54360015880428e-07 2.18010413179814e-07
75.9036144578313
"chr11" 30470001 30471000 "*" 1.86882903141505e-06 2.28378747398741e-06
54.9771167048055
"chr11" 30557001 30558000 "*" 0 0 -100
"chr11" 30915001 30916000 "*" 0 0 100
"chr11" 31831001 31832000 "*" 0 0 93.3611082384111
"chr11" 31833001 31834000 "*" 0 0 57.6479301473454

"chr11" 31837001 31838000 "*" 0 0 87.4453091079122
"chr11" 31844001 31845000 "*" 3.10862446895044e-15 1.22466437093774e-14 100
"chr11" 32396001 32397000 "*" 0 0 59.3220338983051
"chr11" 32402001 32403000 "*" 9.5587315840362e-11 2.13641933875083e-10
-55.1724137931034
"chr11" 32404001 32405000 "*" 1.14124265593318e-11 2.89138062669327e-11 100
"chr11" 32451001 32452000 "*" 1.25033317033285e-12 3.59581578793415e-12
-80.9523809523809
"chr11" 32491001 32492000 "*" 9.04570973681018e-10 1.76414389822173e-09 100
"chr11" 32517001 32518000 "*" 4.79616346638068e-14 1.64380445416596e-13 100
"chr11" 32580001 32581000 "*" 8.80406858527749e-14 2.91456141063452e-13 100
"chr11" 32907001 32908000 "*" 2.62900812231237e-13 8.21294938531841e-13 100
"chr11" 33036001 33037000 "*" 4.08209022140227e-11 9.57770690255255e-11 100
"chr11" 33428001 33429000 "*" 3.04628685166008e-07 4.14477782922613e-07
91.6666666666667
"chr11" 33640001 33641000 "*" 0 0 100
"chr11" 33652001 33653000 "*" 1.04823927316033e-11 2.6754517807651e-11
-53.5714285714286
"chr11" 33703001 33704000 "*" 4.65637306490407e-10 9.46720524046618e-10 100
"chr11" 33756001 33757000 "*" 6.22406126637998e-10 1.24422590507714e-09 56.25
"chr11" 33851001 33852000 "*" 3.46090829739243e-06 4.06200733549184e-06
52.3809523809524
"chr11" 33890001 33891000 "*" 0 0 56.5717746253178
"chr11" 33940001 33941000 "*" 0 0 -100
"chr11" 34010001 34011000 "*" 2.84888113100124e-09 5.16575001433463e-09
63.6363636363636
"chr11" 34244001 34245000 "*" 0 0 100
"chr11" 34328001 34329000 "*" 3.56775498033812e-10 7.35898629809829e-10 100
"chr11" 34367001 34368000 "*" 0.000354266937660941 0.000303242292809723
-56.5217391304348
"chr11" 34791001 34792000 "*" 1.5277158427196e-09 2.87642564232045e-09 -100
"chr11" 35302001 35303000 "*" 4.73234496034536e-09 8.30627300264826e-09 100
"chr11" 35338001 35339000 "*" 5.48638023900594e-10 1.10374357336421e-09 100
"chr11" 35439001 35440000 "*" 0 0 95.2662721893491
"chr11" 36043001 36044000 "*" 3.63445940010365e-11 8.58565209111204e-11 100
"chr11" 36443001 36444000 "*" 9.65338919911574e-13 2.80678910656113e-12 -100
"chr11" 36477001 36478000 "*" 0 0 -72.3268072289157
"chr11" 36823001 36824000 "*" 1.20591092667155e-10 2.66075324129686e-10 -100
"chr11" 37463001 37464000 "*" 3.6700841921089e-08 5.65530226072258e-08 100
"chr11" 37833001 37834000 "*" 8.57092175010621e-13 2.51149198099779e-12 100
"chr11" 38778001 38779000 "*" 1.37828859436695e-10 3.01445544267661e-10 100
"chr11" 39386001 39387000 "*" 0.000105586747396802 9.86490749407008e-05
-57.6923076923077
"chr11" 40302001 40303000 "*" 1.13140401492018e-08 1.87801380787728e-08 -100
"chr11" 40444001 40445000 "*" 9.71063229826541e-11 2.16781365050398e-10 100
"chr11" 41523001 41524000 "*" 4.78841410966879e-11 1.11468187156588e-10 -100
"chr11" 42972001 42973000 "*" 0 0 100
"chr11" 43087001 43088000 "*" 0 0 -100
"chr11" 43180001 43181000 "*" 3.40125705378114e-10 7.03784073980162e-10 100
"chr11" 43538001 43539000 "*" 4.99900121297969e-12 1.32921056339624e-11 -100
"chr11" 43579001 43580000 "*" 1.11022302462516e-16 5.03662826618488e-16
65.8119658119658

"chr11" 43598001 43599000 "*" 1.14305231946332e-11 2.89576137164939e-11 53.3855987216618
"chr11" 43600001 43601000 "*" 0 0 66.726618705036
"chr11" 43955001 43956000 "*" 2.73625566649116e-12 7.52483363579137e-12 100
"chr11" 43963001 43964000 "*" 0 0 61.8896631823461
"chr11" 43964001 43965000 "*" 0 0 78.5173280130053
"chr11" 43965001 43966000 "*" 0 0 53.995824017443
"chr11" 43969001 43970000 "*" 4.01313771103418e-09 7.11882087237587e-09 100
"chr11" 44023001 44024000 "*" 1.77635683940025e-15 7.19870740878856e-15 100
"chr11" 44029001 44030000 "*" 4.01313771103418e-09 7.11882087237587e-09 -100
"chr11" 44035001 44036000 "*" 6.66133814775094e-16 2.81595744474255e-15 -100
"chr11" 44057001 44058000 "*" 2.00227675550835e-08 3.20459176617961e-08 100
"chr11" 44083001 44084000 "*" 0.000106471233016214 9.93930494825975e-05 -51.4285714285714
"chr11" 44105001 44106000 "*" 1.55431223447522e-15 6.33705141101682e-15 96.1538461538462
"chr11" 44349001 44350000 "*" 3.9458181166907e-10 8.10272273225146e-10 -64
"chr11" 44355001 44356000 "*" 2.15125472990962e-10 4.58126659943874e-10 -100
"chr11" 44356001 44357000 "*" 0 0 100
"chr11" 44381001 44382000 "*" 5.18172116326099e-09 9.05310785400106e-09 88.4615384615385
"chr11" 44489001 44490000 "*" 1.39779633423487e-07 1.98463037958589e-07 -100
"chr11" 44526001 44527000 "*" 3.28181926079196e-13 1.01417672179719e-12 -63.6469221835076
"chr11" 44546001 44547000 "*" 2.43897124718728e-11 5.92070968402812e-11 67.5
"chr11" 44589001 44590000 "*" 3.65130148338721e-12 9.90602415539447e-12 100
"chr11" 44609001 44610000 "*" 3.61431756745745e-08 5.59729275811955e-08 -75
"chr11" 44612001 44613000 "*" 8.00762784913811e-05 7.62332949242677e-05 -57.6923076923077
"chr11" 44636001 44637000 "*" 0 0 62.0085322264499
"chr11" 44638001 44639000 "*" 1.12376774552558e-12 3.24761818527664e-12 -82.7586206896552
"chr11" 44649001 44650000 "*" 1.22457599616155e-12 3.52441478267078e-12 -100
"chr11" 44673001 44674000 "*" 1.11022302462516e-14 4.10577217300208e-14 62.8571428571429
"chr11" 44675001 44676000 "*" 5.33790485035546e-07 7.03071689168268e-07 66.6666666666667
"chr11" 44680001 44681000 "*" 9.65729496371637e-10 1.87255912493232e-09 -100
"chr11" 44717001 44718000 "*" 2.03358707651091e-09 3.77658330244668e-09 50.1937150236763
"chr11" 44720001 44721000 "*" 4.7110316747645e-07 6.24717132053794e-07 54.1666666666667
"chr11" 44724001 44725000 "*" 4.01313771103418e-09 7.11882087237587e-09 100
"chr11" 44739001 44740000 "*" 4.01313771103418e-09 7.11882087237587e-09 -100
"chr11" 44746001 44747000 "*" 6.26201313025376e-11 1.43646686881909e-10 90
"chr11" 44764001 44765000 "*" 5.55111512312578e-16 2.36485094870365e-15 -100
"chr11" 44891001 44892000 "*" 2.3990809339125e-12 6.65441738642456e-12 -100
"chr11" 44917001 44918000 "*" 6.37490060739765e-13 1.89496793587877e-12 -100
"chr11" 44951001 44952000 "*" 1.89893459845436e-08 3.05909619306108e-08 -70.5882352941177
"chr11" 44954001 44955000 "*" 1.13519194044898e-11 2.88066083575206e-11 -86.7647058823529

"chr11" 44964001 44965000 "*" 2.61346499996762e-13 8.17572402487652e-13 73.3333333333333
"chr11" 45002001 45003000 "*" 9.65729496371637e-10 1.87255912493232e-09 -100
"chr11" 45047001 45048000 "*" 1.55431223447522e-15 6.33705141101682e-15 -100
"chr11" 45056001 45057000 "*" 4.02167188440217e-12 1.08266700497926e-11 -100
"chr11" 45070001 45071000 "*" 8.77555699674293e-06 9.69721453514667e-06 64.1025641025641
"chr11" 45101001 45102000 "*" 0.000193419911714732 0.000173088784454713 -52.9411764705882
"chr11" 45119001 45120000 "*" 4.08209022140227e-11 9.57770690255255e-11 100
"chr11" 45127001 45128000 "*" 1.35447209004269e-14 4.94674708073053e-14 100
"chr11" 45255001 45256000 "*" 0 0 75
"chr11" 45274001 45275000 "*" 6.66133814775094e-16 2.81595744474255e-15 -82.03125
"chr11" 45290001 45291000 "*" 1.83952852950142e-12 5.17499701329218e-12 -100
"chr11" 45390001 45391000 "*" 0 0 100
"chr11" 45397001 45398000 "*" 1.8125231115329e-05 1.90935684500041e-05 -53.8461538461538
"chr11" 45400001 45401000 "*" 1.50623957750895e-12 4.28756717183481e-12 100
"chr11" 45403001 45404000 "*" 7.0006535457523e-08 1.03695832512054e-07 100
"chr11" 45406001 45407000 "*" 4.07525843182377e-07 5.44861882277266e-07 -60
"chr11" 45422001 45423000 "*" 1.19904086659517e-14 4.40901388691796e-14 100
"chr11" 45424001 45425000 "*" 7.0006535457523e-08 1.03695832512054e-07 -100
"chr11" 45483001 45484000 "*" 0 0 -100
"chr11" 45489001 45490000 "*" 8.77076189453874e-15 3.27889038038365e-14 100
"chr11" 45529001 45530000 "*" 6.59550958292954e-09 1.13456194382773e-08 -100
"chr11" 45535001 45536000 "*" 4.01313771103418e-09 7.11882087237587e-09 100
"chr11" 45585001 45586000 "*" 0 0 100
"chr11" 45586001 45587000 "*" 9.67229976001072e-07 1.22970859507725e-06 60.7142857142857
"chr11" 45595001 45596000 "*" 2.64951482975562e-09 4.82186011375752e-09 100
"chr11" 45598001 45599000 "*" 1.01030295240889e-14 3.75322807935163e-14 -55.3894571203777
"chr11" 45679001 45680000 "*" 8.12104199277019e-06 9.01864929849813e-06 61.2903225806452
"chr11" 45685001 45686000 "*" 3.54686058301468e-10 7.32639797597011e-10 68.75
"chr11" 45686001 45687000 "*" 0 0 65.7156597940281
"chr11" 45687001 45688000 "*" 0 0 50.5390941917996
"chr11" 45751001 45752000 "*" 7.67056418382595e-11 1.7359448580318e-10 -100
"chr11" 45772001 45773000 "*" 8.65973959207622e-14 2.87362558212404e-13 100
"chr11" 45776001 45777000 "*" 6.24654217240561e-10 1.2462108191303e-09 -100
"chr11" 45780001 45781000 "*" 5.46136436074818e-09 9.51795202365188e-09 66.6666666666667
"chr11" 45842001 45843000 "*" 1.4432899320127e-15 5.90750815956055e-15 100
"chr11" 45882001 45883000 "*" 1.9373391779709e-13 6.15971322898373e-13 -100
"chr11" 45902001 45903000 "*" 1.32791699858359e-09 2.52771267630847e-09 -53.3333333333333
"chr11" 45927001 45928000 "*" 0 0 55.9368191721133
"chr11" 46158001 46159000 "*" 2.6165741273676e-06 3.12843692934624e-06 -66.6666666666667
"chr11" 46256001 46257000 "*" 1.66533453693773e-15 6.7629186866784e-15 -100
"chr11" 46258001 46259000 "*" 0 0 -83.6734693877551

"chr11" 46260001 46261000 "*" 0 0 66.5198237885463
"chr11" 46265001 46266000 "*" 0 0 100
"chr11" 46340001 46341000 "*" 2.05280237253191e-13 6.49882890260434e-13
-89.8550724637681
"chr11" 46354001 46355000 "*" 0 0 51.679817635675
"chr11" 46363001 46364000 "*" 1.59172675040509e-11 3.95858417680319e-11 84.375
"chr11" 46402001 46403000 "*" 0 0 -70.3125
"chr11" 46404001 46405000 "*" 0 0 65.2985074626866
"chr11" 46633001 46634000 "*" 3.77475828372553e-15 1.47379557022071e-14 -100
"chr11" 46725001 46726000 "*" 0.000106439195992625 9.93653667048631e-05
-55.5555555555556
"chr11" 46914001 46915000 "*" 6.21724893790088e-15 2.36606385879553e-14
-65.6904761904762
"chr11" 46925001 46926000 "*" 4.53653781207208e-11 1.05953797461759e-10
-64.7887323943662
"chr11" 46940001 46941000 "*" 4.6337429338017e-08 7.06141993600112e-08
-72.0156555772994
"chr11" 47174001 47175000 "*" 4.02167188440217e-12 1.08266700497926e-11 100
"chr11" 47179001 47180000 "*" 4.61508942173339e-10 9.39563721989063e-10
63.2911392405063
"chr11" 47207001 47208000 "*" 0 0 78.7037037037037
"chr11" 47261001 47262000 "*" 0 0 100
"chr11" 47279001 47280000 "*" 3.53310214418201e-09 6.34187411210968e-09
81.8181818181818
"chr11" 47292001 47293000 "*" 7.84707385736994e-08 1.15419461196667e-07 -68.75
"chr11" 47376001 47377000 "*" 0 0 -63.6122920862236
"chr11" 47390001 47391000 "*" 8.29425417236962e-12 2.14541630375412e-11
92.8571428571429
"chr11" 47471001 47472000 "*" 2.08748758279143e-07 2.90229945171996e-07
-68.5714285714286
"chr11" 47481001 47482000 "*" 1.92946192356658e-09 3.59193011611483e-09 100
"chr11" 47489001 47490000 "*" 0 0 95.2380952380952
"chr11" 47531001 47532000 "*" 1.12458486967171e-09 2.15740815043925e-09 100
"chr11" 47541001 47542000 "*" 1.26426137336821e-08 2.08734765987202e-08 -62.5
"chr11" 47557001 47558000 "*" 3.63445940010365e-11 8.58565209111204e-11 100
"chr11" 47609001 47610000 "*" 0 0 67.6470588235294
"chr11" 47736001 47737000 "*" 0 0 58.9078129224115
"chr11" 47914001 47915000 "*" 0 0 68.75
"chr11" 47988001 47989000 "*" 3.34128893442198e-08 5.19772864618523e-08 -100
"chr11" 48028001 48029000 "*" 4.26951363152739e-10 8.72510873516957e-10 -100
"chr11" 48125001 48126000 "*" 3.73034936274053e-14 1.29420415488591e-13 -100
"chr11" 48875001 48876000 "*" 0 0 100
"chr11" 48988001 48989000 "*" 4.79616346638068e-14 1.64380445416596e-13 -100
"chr11" 49582001 49583000 "*" 9.08108033215171e-11 2.03407753381119e-10 75
"chr11" 50123001 50124000 "*" 0.00010969170336006 0.000102204943716034
54.006968641115
"chr11" 50237001 50238000 "*" 1.38590361409285e-10 3.02706178892116e-10 100
"chr11" 50238001 50239000 "*" 0 0 -64.4194162436548
"chr11" 51269001 51270000 "*" 3.88022947106492e-13 1.1867462770515e-12 -100
"chr11" 51569001 51570000 "*" 1.34353794756237e-09 2.55598898770358e-09 80
"chr11" 55268001 55269000 "*" 0 0 100
"chr11" 55906001 55907000 "*" 0 0 100

"chr11" 55919001 55920000 "*" 1.11022302462516e-16 5.03662826618488e-16 100
"chr11" 55920001 55921000 "*" 7.55981943711959e-11 1.71483724541256e-10
-51.5387611998442
"chr11" 56390001 56391000 "*" 2.4535928844216e-14 8.69144396197119e-14 -100
"chr11" 56613001 56614000 "*" 5.52131564579561e-07 7.26208539874537e-07
-58.3333333333333
"chr11" 56860001 56861000 "*" 5.04263297784746e-13 1.52078941532818e-12 -100
"chr11" 57060001 57061000 "*" 5.04263297784746e-13 1.52078941532818e-12 -100
"chr11" 57061001 57062000 "*" 3.31690230837012e-12 9.03347040781321e-12 -100
"chr11" 57074001 57075000 "*" 6.93268023523874e-05 6.66409676902322e-05
-55.5555555555556
"chr11" 57078001 57079000 "*" 8.28330626312379e-10 1.62507001848458e-09
-50.9677419354839
"chr11" 57117001 57118000 "*" 0 0 -79.200717560295
"chr11" 57141001 57142000 "*" 2.23154827949656e-14 7.95931582053377e-14 -100
"chr11" 57147001 57148000 "*" 9.08108033215171e-11 2.03407753381119e-10 100
"chr11" 57226001 57227000 "*" 0 0 80.8446455505279
"chr11" 57231001 57232000 "*" 1.96436548294265e-06 2.39268376948828e-06
66.1764705882353
"chr11" 57236001 57237000 "*" 7.0006535457523e-08 1.03695832512054e-07 -100
"chr11" 57275001 57276000 "*" 2.43005615629954e-09 4.45014222280278e-09 100
"chr11" 57365001 57366000 "*" 4.44089209850063e-16 1.91071758245033e-15
78.3333333333333
"chr11" 57401001 57402000 "*" 1.4432899320127e-15 5.90750815956055e-15 100
"chr11" 57417001 57418000 "*" 1.10400577568726e-12 3.19438145819067e-12
-70.5882352941177
"chr11" 57421001 57422000 "*" 0 0 -100
"chr11" 58158001 58159000 "*" 1.66351331376546e-07 2.34054541106703e-07
54.5454545454545
"chr11" 58395001 58396000 "*" 9.71063229826541e-11 2.16781365050398e-10 -100
"chr11" 58406001 58407000 "*" 5.03264097062583e-13 1.51942428521737e-12 100
"chr11" 58442001 58443000 "*" 1.07014397343619e-12 3.10081482967372e-12
63.2893226176808
"chr11" 58694001 58695000 "*" 0.000722189611975566 0.000586999420811564
57.1428571428571
"chr11" 58912001 58913000 "*" 0 0 -87.8419452887538
"chr11" 59031001 59032000 "*" 4.71103186350241e-07 6.24717132053794e-07
54.1666666666667
"chr11" 59294001 59295000 "*" 0 0 100
"chr11" 60218001 60219000 "*" 0 0 52.7272727272727
"chr11" 60516001 60517000 "*" 7.49583342840587e-07 9.68216039365942e-07
-76.9230769230769
"chr11" 60520001 60521000 "*" 2.23154827949656e-14 7.95931582053377e-14 -100
"chr11" 60524001 60525000 "*" 0 0 100
"chr11" 60539001 60540000 "*" 1.6189272145084e-12 4.58346316902745e-12
-93.2432432432432
"chr11" 60570001 60571000 "*" 5.01025332333427e-10 1.01358031135889e-09 -100
"chr11" 60609001 60610000 "*" 3.10862446895044e-15 1.22466437093774e-14 100
"chr11" 60632001 60633000 "*" 1.11809308478428e-08 1.86309449457567e-08
76.0869565217391
"chr11" 60643001 60644000 "*" 0 0 -100
"chr11" 60666001 60667000 "*" 7.78672069268183e-07 1.00356652327738e-06

-73.2142857142857
"chr11" 60725001 60726000 "*" 0 0 -100
"chr11" 60781001 60782000 "*" 1.99325966843134e-09 3.70507367363734e-09
54.5454545454545
"chr11" 60808001 60809000 "*" 6.59550958292954e-09 1.13456194382773e-08 100
"chr11" 60809001 60810000 "*" 5.61418600497632e-09 9.7699953204495e-09 -52
"chr11" 60820001 60821000 "*" 9.49633749414147e-09 1.59645467782879e-08
50.328407224959
"chr11" 60830001 60831000 "*" 1.40857882779954e-06 1.75301363857165e-06
54.5454545454545
"chr11" 60836001 60837000 "*" 0 0 100
"chr11" 60837001 60838000 "*" 7.67056418382595e-11 1.7359448580318e-10 -100
"chr11" 60878001 60879000 "*" 0.000318501135539329 0.00027486487815337
-53.8461538461538
"chr11" 60888001 60889000 "*" 5.48638023900594e-10 1.10374357336421e-09 100
"chr11" 60894001 60895000 "*" 8.6615080138186e-06 9.57765940311424e-06
-58.2089552238806
"chr11" 60931001 60932000 "*" 1.92946192356658e-09 3.59193011611483e-09 100
"chr11" 60942001 60943000 "*" 1.20591092667155e-10 2.66075324129686e-10 -100
"chr11" 60969001 60970000 "*" 8.7349629751543e-09 1.47505871898511e-08 -100
"chr11" 61041001 61042000 "*" 1.70776726093891e-11 4.23416908925427e-11 74
"chr11" 61108001 61109000 "*" 7.45486561370967e-10 1.46952418634053e-09
58.3333333333333
"chr11" 61114001 61115000 "*" 5.04263297784746e-13 1.52078941532818e-12 100
"chr11" 61163001 61164000 "*" 2.38871089919268e-06 2.87375451142406e-06 62
"chr11" 61273001 61274000 "*" 5.8059563268209e-08 8.73477170489365e-08
-62.0689655172414
"chr11" 61276001 61277000 "*" 0 0 64.1983932937478
"chr11" 61341001 61342000 "*" 1.67299207820548e-08 2.71377429441055e-08 100
"chr11" 61345001 61346000 "*" 4.01313771103418e-09 7.11882087237587e-09 -100
"chr11" 61367001 61368000 "*" 9.93161875307846e-05 9.31526365269394e-05
60.6060606060606
"chr11" 61369001 61370000 "*" 2.0677379763967e-06 2.51126077066295e-06
-73.5849056603774
"chr11" 61388001 61389000 "*" 2.22044604925031e-16 9.81641919380259e-16
-50.4950495049505
"chr11" 61446001 61447000 "*" 6.59550958292954e-09 1.13456194382773e-08 -100
"chr11" 61524001 61525000 "*" 0 0 100
"chr11" 61582001 61583000 "*" 0 0 85.8630952380952
"chr11" 61583001 61584000 "*" 0 0 93.2671863926293
"chr11" 61584001 61585000 "*" 0 0 72.5249169435216
"chr11" 61609001 61610000 "*" 0.000209763311656563 0.000186660769941092
52.3809523809524
"chr11" 61659001 61660000 "*" 0 0 -100
"chr11" 61691001 61692000 "*" 3.95861121660346e-12 1.0684496262032e-11 100
"chr11" 61713001 61714000 "*" 1.10851106072118e-10 2.46003552493985e-10
-50.6329113924051
"chr11" 61729001 61730000 "*" 1.94250699458998e-06 2.36785742582199e-06
54.5454545454545
"chr11" 61781001 61782000 "*" 4.51329196238248e-10 9.2001884476189e-10
-70.5882352941177
"chr11" 61819001 61820000 "*" 1.93720595120794e-11 4.76309924836636e-11 -100

"chr11" 61844001 61845000 "*" 2.08995487582797e-10 4.46944006972663e-10 -100
"chr11" 61972001 61973000 "*" 1.29037891483108e-11 3.24582280286866e-11 100
"chr11" 62105001 62106000 "*" 0 0 58.206106870229
"chr11" 62149001 62150000 "*" 4.44089209850063e-16 1.91071758245033e-15
83.3333333333333
"chr11" 62182001 62183000 "*" 3.77475828372553e-15 1.47379557022071e-14 -100
"chr11" 62207001 62208000 "*" 1.88220047991905e-07 2.63185232394914e-07
-51.0422870756403
"chr11" 62228001 62229000 "*" 1.14130926931466e-13 3.7276674571693e-13 -100
"chr11" 62230001 62231000 "*" 0.000194200384562526 0.000173742486291534
-55.5555555555556
"chr11" 62280001 62281000 "*" 0.000808777120367532 0.000651834190530565
58.3333333333333
"chr11" 62311001 62312000 "*" 0 0 -97.1698113207547
"chr11" 62340001 62341000 "*" 0 0 -100
"chr11" 62361001 62362000 "*" 1.93720595120794e-11 4.76309924836636e-11 -100
"chr11" 62436001 62437000 "*" 4.98198101661629e-08 7.55785595670105e-08 -75
"chr11" 62438001 62439000 "*" 2.48536746738637e-11 6.02772417720813e-11
-58.0645161290323
"chr11" 62457001 62458000 "*" 0.000499129428774947 0.000416865202314435
-52.3809523809524
"chr11" 62538001 62539000 "*" 3.26072502332408e-13 1.00814361287029e-12
63.5135135135135
"chr11" 62558001 62559000 "*" 2.62697641417731e-11 6.35453517794948e-11
-73.1543624161074
"chr11" 62573001 62574000 "*" 0 0 100
"chr11" 62585001 62586000 "*" 1.33226762955019e-15 5.48133041560118e-15 100
"chr11" 62609001 62610000 "*" 3.03273406387916e-10 6.32393575985756e-10
-53.8461538461538
"chr11" 62648001 62649000 "*" 0 0 -69.8564593301435
"chr11" 62760001 62761000 "*" 5.72126176558996e-05 5.57465732173144e-05 62.5
"chr11" 62906001 62907000 "*" 1.25284005392245e-10 2.75649590272477e-10 -100
"chr11" 63226001 63227000 "*" 6.22552779994123e-07 8.13304131452572e-07 -75
"chr11" 63227001 63228000 "*" 3.95353005888666e-09 7.03928529283733e-09 100
"chr11" 63278001 63279000 "*" 1.9625394301892e-06 2.39070396570886e-06
78.5714285714286
"chr11" 63325001 63326000 "*" 2.38031816479634e-13 7.47299807843568e-13 100
"chr11" 63357001 63358000 "*" 0 0 51.5873015873016
"chr11" 63607001 63608000 "*" 0 0 62.1621621621622
"chr11" 63653001 63654000 "*" 1.13140401492018e-08 1.87801380787728e-08 -100
"chr11" 63753001 63754000 "*" 0 0 78.7945921381504
"chr11" 63754001 63755000 "*" 0 0 -59.5611285266458
"chr11" 63834001 63835000 "*" 2.22044604925031e-16 9.81641919380259e-16
-60.5054418004058
"chr11" 63841001 63842000 "*" 0 0 -71.8432510885341
"chr11" 63861001 63862000 "*" 1.56863411149288e-12 4.4469621057462e-12 -100
"chr11" 63880001 63881000 "*" 9.20794250323098e-05 8.68455135226072e-05
-64.2857142857143
"chr11" 63949001 63950000 "*" 9.08108033215171e-11 2.03407753381119e-10 100
"chr11" 63994001 63995000 "*" 0 0 -90.9871244635193
"chr11" 64001001 64002000 "*" 0 0 61.8879603837821
"chr11" 64036001 64037000 "*" 6.87450130154588e-09 1.18026679265499e-08

-83.6734693877551
"chr11" 64051001 64052000 "*" 3.42845475753695e-06 4.02641014303533e-06
-52.1739130434783
"chr11" 64094001 64095000 "*" 1.68753899743024e-14 6.09826805967439e-14 100
"chr11" 64125001 64126000 "*" 4.26951363152739e-10 8.72510873516957e-10 -100
"chr11" 64140001 64141000 "*" 3.66373598126302e-15 1.43441054525534e-14 100
"chr11" 64207001 64208000 "*" 2.79987144580218e-12 7.68634726811898e-12 100
"chr11" 64302001 64303000 "*" 4.56087558764473e-06 5.25706510588819e-06
-56.1904761904762
"chr11" 64307001 64308000 "*" 0 0 100
"chr11" 64334001 64335000 "*" 9.48574552239734e-13 2.76469609591165e-12
-53.7735849056604
"chr11" 64350001 64351000 "*" 1.42219569454483e-13 4.58954156292913e-13
-58.10635538262
"chr11" 64372001 64373000 "*" 4.2706999048292e-09 7.55257312576703e-09
-81.0344827586207
"chr11" 64385001 64386000 "*" 8.7349629751543e-09 1.47505871898511e-08 -100
"chr11" 64388001 64389000 "*" 1.93986546104252e-06 2.36484486104086e-06
63.6363636363636
"chr11" 64420001 64421000 "*" 1.48487888651516e-11 3.70689457767456e-11 100
"chr11" 64432001 64433000 "*" 9.59851414483737e-06 1.05512425142076e-05
70.2702702702703
"chr11" 64435001 64436000 "*" 3.25073301610246e-13 1.0052033636326e-12
-54.1666666666667
"chr11" 64443001 64444000 "*" 1.68244879361623e-07 2.36602987949447e-07
-52.2935779816514
"chr11" 64484001 64485000 "*" 1.14908083048704e-13 3.75128751466461e-13
-84.8484848484848
"chr11" 64491001 64492000 "*" 2.41460746319699e-06 2.90324230109513e-06
-53.030303030303
"chr11" 64493001 64494000 "*" 0 0 -100
"chr11" 64502001 64503000 "*" 6.0619731456768e-10 1.21353972626925e-09
-81.4285714285714
"chr11" 64506001 64507000 "*" 1.41115674701098e-10 3.08036370911628e-10
-62.962962962963
"chr11" 64555001 64556000 "*" 4.01313771103418e-09 7.11882087237587e-09 100
"chr11" 64565001 64566000 "*" 2.14030093015349e-05 2.22974941352435e-05 -59.375
"chr11" 64590001 64591000 "*" 1.38586475628699e-10 3.02706178892116e-10
-60.7142857142857
"chr11" 64656001 64657000 "*" 1.46575529491599e-10 3.19335997447865e-10
77.4193548387097
"chr11" 64661001 64662000 "*" 3.93285404243215e-12 1.06228833182929e-11
-69.6969696969697
"chr11" 64684001 64685000 "*" 0 0 -100
"chr11" 64782001 64783000 "*" 2.15125472990962e-10 4.58126659943874e-10 -100
"chr11" 64822001 64823000 "*" 0 0 -97.5845410628019
"chr11" 64828001 64829000 "*" 4.44089209850063e-16 1.91071758245033e-15
53.968253968254
"chr11" 64843001 64844000 "*" 8.43347613965761e-12 2.17859001990012e-11 100
"chr11" 64844001 64845000 "*" 2.00227675550835e-08 3.20459176617961e-08 100
"chr11" 64853001 64854000 "*" 1.66533453693773e-15 6.7629186866784e-15 100
"chr11" 64904001 64905000 "*" 1.66533453693773e-15 6.7629186866784e-15 -100

"chr11" 64905001 64906000 "*" 2.78719380908399e-10 5.85054631700465e-10 -53.8461538461538
"chr11" 64919001 64920000 "*" 1.92946192356658e-09 3.59193011611483e-09 -100
"chr11" 65141001 65142000 "*" 1.97131200252443e-12 5.52523171646948e-12 -55.8139534883721
"chr11" 65150001 65151000 "*" 3.33066907387547e-16 1.4495649018245e-15 -100
"chr11" 65190001 65191000 "*" 0 0 -56.8027210884354
"chr11" 65222001 65223000 "*" 0 0 77.731673582296
"chr11" 65233001 65234000 "*" 0 0 -68.4782608695652
"chr11" 65268001 65269000 "*" 0 0 -100
"chr11" 65308001 65309000 "*" 0 0 -57.3117236024845
"chr11" 65318001 65319000 "*" 0 0 -72.3633407843934
"chr11" 65339001 65340000 "*" 1.66533453693773e-15 6.7629186866784e-15 50.5154639175258
"chr11" 65342001 65343000 "*" 0 0 -93.3333333333333
"chr11" 65357001 65358000 "*" 6.59550958292954e-09 1.13456194382773e-08 100
"chr11" 65467001 65468000 "*" 1.36604061395929e-11 3.42333952202875e-11 100
"chr11" 65478001 65479000 "*" 3.88022947106492e-13 1.1867462770515e-12 -100
"chr11" 65525001 65526000 "*" 9.0072393987839e-13 2.63157725646847e-12 100
"chr11" 65614001 65615000 "*" 7.67056418382595e-11 1.7359448580318e-10 -100
"chr11" 65652001 65653000 "*" 0 0 100
"chr11" 65656001 65657000 "*" 6.98496116502412e-09 1.19804756703193e-08 61.2903225806452
"chr11" 65660001 65661000 "*" 2.37587727269783e-14 8.44268514944447e-14 100
"chr11" 65676001 65677000 "*" 1.88737914186277e-15 7.62011598380321e-15 100
"chr11" 65702001 65703000 "*" 2.00227675550835e-08 3.20459176617961e-08 100
"chr11" 65728001 65729000 "*" 3.33955085807247e-13 1.03144416964409e-12 -97.9166666666667
"chr11" 65757001 65758000 "*" 6.38322283919024e-09 1.10339339031813e-08 -63.1578947368421
"chr11" 65774001 65775000 "*" 1.02497708653893e-07 1.48527997003363e-07 51.8518518518519
"chr11" 65780001 65781000 "*" 4.99020824662466e-11 1.15790792586157e-10 -100
"chr11" 65782001 65783000 "*" 8.62854232508425e-12 2.22278438222256e-11 -64.7058823529412
"chr11" 66032001 66033000 "*" 0 0 -50.8771929824561
"chr11" 66044001 66045000 "*" 0 0 -63.6363636363636
"chr11" 66057001 66058000 "*" 2.98950849630941e-08 4.68668757286857e-08 51.219512195122
"chr11" 66080001 66081000 "*" 0 0 -55.9375833555615
"chr11" 66094001 66095000 "*" 6.65742801331248e-07 8.66145851016855e-07 53.8461538461538
"chr11" 66127001 66128000 "*" 1.54434221734112e-05 1.64448325229152e-05 55.1724137931034
"chr11" 66173001 66174000 "*" 9.65729496371637e-10 1.87255912493232e-09 100
"chr11" 66178001 66179000 "*" 5.21445020209566e-05 5.11375164526832e-05 54.2372881355932
"chr11" 66179001 66180000 "*" 1.83186799063151e-14 6.59696304569048e-14 61.1111111111111
"chr11" 66444001 66445000 "*" 1.11022302462516e-16 5.03662826618488e-16 87.1794871794872
"chr11" 66446001 66447000 "*" 1.06659125975739e-12 3.09116497360924e-12

52.7472527472527
"chr11" 66503001 66504000 "*" 2.00227675550835e-08 3.20459176617961e-08 -100
"chr11" 66511001 66512000 "*" 0 0 -94.0594059405941
"chr11" 66635001 66636000 "*" 0 0 100
"chr11" 66646001 66647000 "*" 0 0 55.1282051282051
"chr11" 66654001 66655000 "*" 6.99652558111552e-11 1.59208509953711e-10 90
"chr11" 66725001 66726000 "*" 0 0 61.9612882171776
"chr11" 66770001 66771000 "*" 0 0 100
"chr11" 66823001 66824000 "*" 0 0 -100
"chr11" 66824001 66825000 "*" 0 0 -75.3208292201382
"chr11" 66846001 66847000 "*" 2.99102884682156e-08 4.68889476666579e-08
-52.7514231499051
"chr11" 67152001 67153000 "*" 5.1188540040048e-07 6.75724515456281e-07
-70.9677419354839
"chr11" 67157001 67158000 "*" 5.03264097062583e-13 1.51942428521737e-12 100
"chr11" 67167001 67168000 "*" 0 0 -100
"chr11" 67168001 67169000 "*" 1.11022302462516e-16 5.03662826618488e-16
-54.0350096424863
"chr11" 67169001 67170000 "*" 0 0 -100
"chr11" 67231001 67232000 "*" 0 0 -67.258064516129
"chr11" 67273001 67274000 "*" 0 0 -91.3793103448276
"chr11" 67396001 67397000 "*" 0 0 -54.4658150157008
"chr11" 67442001 67443000 "*" 0 0 100
"chr11" 67468001 67469000 "*" 0 0 100
"chr11" 67483001 67484000 "*" 4.97892771456065e-09 8.71688060868859e-09 -75
"chr11" 67511001 67512000 "*" 0 0 -100
"chr11" 67542001 67543000 "*" 1.11022302462516e-16 5.03662826618488e-16 100
"chr11" 67559001 67560000 "*" 0 0 -90.4975347377858
"chr11" 67573001 67574000 "*" 0 0 51.3513513513514
"chr11" 67600001 67601000 "*" 0 0 -100
"chr11" 67746001 67747000 "*" 5.08482145278322e-14 1.73432706170696e-13 -100
"chr11" 67788001 67789000 "*" 0 0 -66.0698412698413
"chr11" 67806001 67807000 "*" 0 0 -59.2046939156402
"chr11" 67875001 67876000 "*" 7.99010846375126e-09 1.36162773751599e-08
53.3333333333333
"chr11" 67910001 67911000 "*" 1.71295392281667e-06 2.10492413684395e-06
-52.1645021645022
"chr11" 67972001 67973000 "*" 4.08209022140227e-11 9.57770690255255e-11 100
"chr11" 68049001 68050000 "*" 2.22044604925031e-16 9.81641919380259e-16 -100
"chr11" 68202001 68203000 "*" 7.0006535457523e-08 1.03695832512054e-07 -100
"chr11" 68227001 68228000 "*" 1.11298015248451e-09 2.1402656313269e-09
-74.0740740740741
"chr11" 68392001 68393000 "*" 1.39779633423487e-07 1.98463037958589e-07 100
"chr11" 68403001 68404000 "*" 0.000412523424090483 0.000349386048469569 52
"chr11" 68421001 68422000 "*" 2.51081377911078e-11 6.08607606414268e-11
91.7159763313609
"chr11" 68449001 68450000 "*" 2.17990273942981e-07 3.02303637836045e-07
75.7575757575758
"chr11" 68480001 68481000 "*" 7.552484970752e-09 1.29058417082711e-08
70.5882352941177
"chr11" 68500001 68501000 "*" 3.65130148338721e-12 9.90602415539447e-12 100
"chr11" 68504001 68505000 "*" 0 0 -66.6666666666667

"chr11" 68528001 68529000 "*" 5.91870164878827e-07 7.75564979754954e-07
-57.6923076923077
"chr11" 68565001 68566000 "*" 0 0 -50.2976190476191
"chr11" 68607001 68608000 "*" 0 0 -65.5769230769231
"chr11" 68611001 68612000 "*" 0 0 -67.021087398374
"chr11" 68743001 68744000 "*" 0 0 -99.2424242424242
"chr11" 68748001 68749000 "*" 2.88710388929303e-10 6.03399264731139e-10 100
"chr11" 68797001 68798000 "*" 0 0 100
"chr11" 68805001 68806000 "*" 3.69941410838237e-10 7.6190570445393e-10
66.6666666666667
"chr11" 68817001 68818000 "*" 4.65637306490407e-10 9.46720524046618e-10 100
"chr11" 68856001 68857000 "*" 0 0 -67.453591606134
"chr11" 68876001 68877000 "*" 1.25284005392245e-10 2.75649590272477e-10 100
"chr11" 68882001 68883000 "*" 2.64233079860787e-13 8.2527143103169e-13
-61.9402985074627
"chr11" 68890001 68891000 "*" 4.71844785465692e-14 1.6202982653006e-13
-59.2592592592593
"chr11" 68898001 68899000 "*" 4.05686595428278e-11 9.5434927396784e-11
-50.3658536585366
"chr11" 68900001 68901000 "*" 1.07882591748876e-11 2.74553537455803e-11 100
"chr11" 68916001 68917000 "*" 0 0 -100
"chr11" 68957001 68958000 "*" 3.10862446895044e-15 1.22466437093774e-14 100
"chr11" 68974001 68975000 "*" 6.57719248994271e-05 6.34967922170184e-05
-55.1724137931034
"chr11" 68979001 68980000 "*" 1.9616218649432e-09 3.64894905157263e-09
57.6271186440678
"chr11" 69042001 69043000 "*" 1.33959510151271e-12 3.84139613269674e-12
80.5555555555556
"chr11" 69056001 69057000 "*" 0 0 70.2702702702703
"chr11" 69061001 69062000 "*" 2.90641954947546e-11 6.97245551884382e-11
-55.1615445232467
"chr11" 69062001 69063000 "*" 1.82742709853301e-13 5.83561257720885e-13
-51.5444348576359
"chr11" 69185001 69186000 "*" 9.53776946310825e-10 1.85490304253222e-09
51.1506730351715
"chr11" 69188001 69189000 "*" 3.91786603159971e-12 1.05861711160927e-11
-87.9120879120879
"chr11" 69230001 69231000 "*" 6.32827124036339e-15 2.40666772484995e-14
-64.3681318681319
"chr11" 69235001 69236000 "*" 0 0 -100
"chr11" 69299001 69300000 "*" 4.65183447317941e-14 1.59847652445645e-13
-51.6237799467613
"chr11" 69326001 69327000 "*" 0 0 -100
"chr11" 69337001 69338000 "*" 6.39165064653113e-07 8.33531059852563e-07
92.3076923076923
"chr11" 69339001 69340000 "*" 5.89528426075958e-14 1.99347557647516e-13
-66.3133640552995
"chr11" 69343001 69344000 "*" 8.83138007168327e-12 2.27349215415833e-11
-86.8131868131868
"chr11" 69348001 69349000 "*" 0 0 -63.3333333333333
"chr11" 69394001 69395000 "*" 4.78841410966879e-11 1.11468187156588e-10 100
"chr11" 69483001 69484000 "*" 1.40520262092991e-09 2.66770339781019e-09

-92.5925925925926
"chr11" 69520001 69521000 "*" 6.41930952838266e-13 1.90789668483421e-12
-83.9506172839506
"chr11" 69529001 69530000 "*" 2.04697370165263e-11 5.00495553786191e-11
-64.5833333333333
"chr11" 69550001 69551000 "*" 3.33066907387547e-16 1.4495649018245e-15 60
"chr11" 69558001 69559000 "*" 0 0 100
"chr11" 69566001 69567000 "*" 2.3850007524473e-09 4.3810040091482e-09
71.8181818181818
"chr11" 69620001 69621000 "*" 2.05391259555654e-14 7.35435952190685e-14 -100
"chr11" 69628001 69629000 "*" 2.00227675550835e-08 3.20459176617961e-08 100
"chr11" 69630001 69631000 "*" 4.57411886145564e-14 1.57248434075695e-13
73.469387755102
"chr11" 69639001 69640000 "*" 0 0 -100
"chr11" 69802001 69803000 "*" 0 0 67.2619047619048
"chr11" 69812001 69813000 "*" 2.53555687557139e-09 4.63418676869216e-09
-61.5384615384615
"chr11" 69826001 69827000 "*" 1.21884147219475e-09 2.32917600248869e-09
63.6363636363636
"chr11" 69843001 69844000 "*" 2.02718650754719e-08 3.24159766118952e-08
92.8571428571429
"chr11" 69847001 69848000 "*" 3.12164156484052e-05 3.17090411597004e-05
-59.2592592592593
"chr11" 69850001 69851000 "*" 0.000199851252180694 0.000178453492150885
54.5454545454545
"chr11" 69866001 69867000 "*" 1.52794177310511e-09 2.87642564232045e-09 -100
"chr11" 69878001 69879000 "*" 3.33066907387547e-16 1.4495649018245e-15
55.5058750129978
"chr11" 69883001 69884000 "*" 7.19048581787618e-08 1.06341483652509e-07
-75.3246753246753
"chr11" 69933001 69934000 "*" 2.45832546541536e-08 3.89080666465499e-08
-54.5454545454545
"chr11" 69937001 69938000 "*" 6.92287338566189e-11 1.57605630124247e-10 -100
"chr11" 69945001 69946000 "*" 0 0 -52.3809523809524
"chr11" 69988001 69989000 "*" 6.70463462526527e-10 1.32889205920977e-09
-83.3333333333333
"chr11" 69999001 70000000 "*" 3.50322780229284e-07 4.72754385845506e-07
66.6666666666667
"chr11" 70030001 70031000 "*" 3.80251385934116e-13 1.1658847325686e-12 55
"chr11" 70043001 70044000 "*" 1.7656157980106e-08 2.85552257475162e-08
-85.4838709677419
"chr11" 70101001 70102000 "*" 1.96509475358653e-13 6.23868565133654e-13 100
"chr11" 70109001 70110000 "*" 9.08108033215171e-11 2.03407753381119e-10 100
"chr11" 70323001 70324000 "*" 0 0 -91.6666666666667
"chr11" 70349001 70350000 "*" 5.77315972805081e-15 2.205310974382e-14
58.695652173913
"chr11" 70375001 70376000 "*" 1.74853465040314e-11 4.32752710900609e-11 100
"chr11" 70387001 70388000 "*" 0 0 -91.4634146341463
"chr11" 70413001 70414000 "*" 1.47770684577608e-13 4.76186317586715e-13
-53.5064935064935
"chr11" 70420001 70421000 "*" 6.04438721296674e-11 1.38849255306602e-10 100
"chr11" 70449001 70450000 "*" 1.09068643006083e-08 1.82000817211812e-08

-64.5161290322581
"chr11" 70456001 70457000 "*" 2.22044604925031e-15 8.90449334476539e-15
-50.4545454545455
"chr11" 70459001 70460000 "*" 3.81916720471054e-14 1.32265263149517e-13
-89.0243902439024
"chr11" 70470001 70471000 "*" 7.3404803079824e-05 7.02790923864156e-05
58.3333333333333
"chr11" 70471001 70472000 "*" 5.31368837819457e-10 1.07239035504224e-09
-57.1428571428571
"chr11" 70493001 70494000 "*" 2.96028846991092e-08 4.64323581179945e-08
-56.5217391304348
"chr11" 70527001 70528000 "*" 0 0 57.8125
"chr11" 70549001 70550000 "*" 1.8650636590678e-12 5.24400273275934e-12
-53.5714285714286
"chr11" 70603001 70604000 "*" 2.49922305073369e-12 6.91585468376262e-12
64.8648648648649
"chr11" 70609001 70610000 "*" 0 0 100
"chr11" 70671001 70672000 "*" 3.04126768213564e-09 5.50000746712767e-09
-61.5483870967742
"chr11" 70677001 70678000 "*" 0 0 -62.8192898781134
"chr11" 70678001 70679000 "*" 0 0 -100
"chr11" 70764001 70765000 "*" 1.5277158427196e-09 2.87642564232045e-09 -100
"chr11" 70920001 70921000 "*" 8.21565038222616e-15 3.08158744293689e-14 100
"chr11" 70993001 70994000 "*" 0 0 -100
"chr11" 71022001 71023000 "*" 0 0 84.6153846153846
"chr11" 71092001 71093000 "*" 2.64951482975562e-09 4.82186011375752e-09 -100
"chr11" 71098001 71099000 "*" 2.24043006369357e-13 7.05213725935634e-13 -60
"chr11" 71107001 71108000 "*" 0 0 -71.4285714285714
"chr11" 71159001 71160000 "*" 0 0 76.2237762237762
"chr11" 71167001 71168000 "*" 1.49613654798486e-12 4.26244554429779e-12 -100
"chr11" 71198001 71199000 "*" 0 0 100
"chr11" 71254001 71255000 "*" 1.00156549720509e-11 2.56246581163586e-11
-59.2592592592593
"chr11" 71283001 71284000 "*" 9.19333264171041e-06 1.01330895744113e-05
52.6315789473684
"chr11" 71309001 71310000 "*" 4.44089209850063e-16 1.91071758245033e-15 -100
"chr11" 71481001 71482000 "*" 1.39779633423487e-07 1.98463037958589e-07 100
"chr11" 71486001 71487000 "*" 1.0325074129014e-14 3.83008663711937e-14
-91.8238993710692
"chr11" 71511001 71512000 "*" 9.65729496371637e-10 1.87255912493232e-09 100
"chr11" 71757001 71758000 "*" 6.04438721296674e-11 1.38849255306602e-10 100
"chr11" 71874001 71875000 "*" 0 0 -100
"chr11" 71932001 71933000 "*" 1.37828859436695e-10 3.01445544267661e-10 -100
"chr11" 72045001 72046000 "*" 5.96089844151493e-12 1.57055375177837e-11
68.6274509803922
"chr11" 72160001 72161000 "*" 1.77079773067135e-09 3.31334585803845e-09
92.3076923076923
"chr11" 72172001 72173000 "*" 0 0 100
"chr11" 72275001 72276000 "*" 1.52794177310511e-09 2.87642564232045e-09 -100
"chr11" 72293001 72294000 "*" 2.95423767674441e-05 3.0118633488738e-05
-76.9230769230769
"chr11" 72294001 72295000 "*" 2.15125472990962e-10 4.58126659943874e-10 -100

"chr11" 72296001 72297000 "*" 3.11867195612603e-06 3.68689547054812e-06 60.9756097560976
"chr11" 72297001 72298000 "*" 1.29037891483108e-11 3.24582280286866e-11 100
"chr11" 72299001 72300000 "*" 2.69018141096922e-12 7.41487347763769e-12 100
"chr11" 72308001 72309000 "*" 1.11022302462516e-16 5.03662826618488e-16 -100
"chr11" 72317001 72318000 "*" 4.73234496034536e-09 8.30627300264826e-09 100
"chr11" 72332001 72333000 "*" 1.52794177310511e-09 2.87642564232045e-09 100
"chr11" 72346001 72347000 "*" 8.65973959207622e-14 2.87362558212404e-13 100
"chr11" 72354001 72355000 "*" 0 0 65.3862426400592
"chr11" 72368001 72369000 "*" 0 0 -100
"chr11" 72374001 72375000 "*" 0.00034686637909298 0.000297539755126761 -55
"chr11" 72491001 72492000 "*" 1.19904086659517e-14 4.40901388691796e-14 -100
"chr11" 72496001 72497000 "*" 4.90718576884319e-14 1.6794399166207e-13 100
"chr11" 72535001 72536000 "*" 6.15840711759574e-13 1.83676803572802e-12 -100
"chr11" 72538001 72539000 "*" 2.06501482580279e-14 7.3890370570066e-14 100
"chr11" 72864001 72865000 "*" 5.48638023900594e-10 1.10374357336421e-09 100
"chr11" 72896001 72897000 "*" 1.563860152487e-12 4.439917847241e-12 -84.2105263157895
"chr11" 72925001 72926000 "*" 8.57092175010621e-13 2.51149198099779e-12 100
"chr11" 72939001 72940000 "*" 5.41645372020838e-06 6.17919206863303e-06 -60.4838709677419
"chr11" 73081001 73082000 "*" 0.000471727282945977 0.000395504194557835 -53.8461538461538
"chr11" 73085001 73086000 "*" 1.5277158427196e-09 2.87642564232045e-09 100
"chr11" 73308001 73309000 "*" 9.3221530494958e-10 1.81534057529218e-09 -86.5168539325843
"chr11" 73380001 73381000 "*" 6.54408749412028e-11 1.49667780508112e-10 -86.3636363636364
"chr11" 73401001 73402000 "*" 8.7349629751543e-09 1.47505871898511e-08 100
"chr11" 73686001 73687000 "*" 0 0 -100
"chr11" 73966001 73967000 "*" 7.0006535457523e-08 1.03695832512054e-07 100
"chr11" 73970001 73971000 "*" 6.88338275267597e-15 2.60518659957509e-14 100
"chr11" 74302001 74303000 "*" 4.08209022140227e-11 9.57770690255255e-11 -100
"chr11" 74404001 74405000 "*" 7.3482275819714e-10 1.44983984345485e-09 53.8823529411765
"chr11" 74418001 74419000 "*" 1.11022302462516e-16 5.03662826618488e-16 -100
"chr11" 74547001 74548000 "*" 3.24330162726483e-09 5.8464880516327e-09 -61.2903225806452
"chr11" 74660001 74661000 "*" 0 0 -100
"chr11" 74842001 74843000 "*" 8.00692845359663e-13 2.35924187817154e-12 82
"chr11" 74850001 74851000 "*" 1.11022302462516e-16 5.03662826618488e-16 100
"chr11" 74870001 74871000 "*" 0 0 100
"chr11" 74906001 74907000 "*" 6.43823017454359e-07 8.39277382550671e-07 71.7647058823529
"chr11" 74952001 74953000 "*" 0 0 61.0040776454845
"chr11" 74957001 74958000 "*" 1.20591092667155e-10 2.66075324129686e-10 -100
"chr11" 74959001 74960000 "*" 9.32032229172819e-13 2.7181920310254e-12 100
"chr11" 74972001 74973000 "*" 9.65729496371637e-10 1.87255912493232e-09 100
"chr11" 75029001 75030000 "*" 1.62548530191486e-10 3.52020480106197e-10 64.0350877192982
"chr11" 75141001 75142000 "*" 0 0 51.0438528793112
"chr11" 75143001 75144000 "*" 9.0072393987839e-13 2.63157725646847e-12 100

"chr11" 75235001 75236000 "*" 3.31956684362922e-14 1.15814343608523e-13
66.6666666666667
"chr11" 75258001 75259000 "*" 1.46549439250521e-14 5.33330076973935e-14 100
"chr11" 75296001 75297000 "*" 3.33066907387547e-16 1.4495649018245e-15
-94.7826086956522
"chr11" 75300001 75301000 "*" 3.99680288865056e-15 1.5566998858822e-14 -100
"chr11" 75340001 75341000 "*" 8.51117620825903e-10 1.66722573233912e-09
-93.3333333333333
"chr11" 75378001 75379000 "*" 0 0 80.242623850693
"chr11" 75379001 75380000 "*" 0 0 80.6917692124659
"chr11" 75417001 75418000 "*" 0 0 -90.3703703703704
"chr11" 75870001 75871000 "*" 0 0 97.4358974358974
"chr11" 75877001 75878000 "*" 0.000726374156166165 0.000590081342527627
52.1739130434783
"chr11" 75885001 75886000 "*" 9.93582993658038e-12 2.54290097794575e-11
-79.1666666666667
"chr11" 75919001 75920000 "*" 0 0 55.9220505437437
"chr11" 75920001 75921000 "*" 0 0 78.2407407407407
"chr11" 75928001 75929000 "*" 1.52794177310511e-09 2.87642564232045e-09 -100
"chr11" 75946001 75947000 "*" 0 0 -64.667060677699
"chr11" 75947001 75948000 "*" 0 0 -74.8490945674044
"chr11" 75959001 75960000 "*" 1.12634901405784e-10 2.49563754426169e-10 100
"chr11" 75992001 75993000 "*" 0 0 -77.2727272727273
"chr11" 75994001 75995000 "*" 3.52704532247117e-11 8.35581292290093e-11 -100
"chr11" 75995001 75996000 "*" 3.00165781119688e-10 6.26252476865071e-10
-50.5154639175258
"chr11" 76002001 76003000 "*" 4.30544488949636e-11 1.00779428725404e-10 -72.5
"chr11" 76008001 76009000 "*" 3.99546075571244e-05 3.98784360862687e-05
55.1724137931034
"chr11" 76013001 76014000 "*" 1.53210777398272e-14 5.5628583449216e-14 -100
"chr11" 76018001 76019000 "*" 3.6700841921089e-08 5.65530226072258e-08 100
"chr11" 76316001 76317000 "*" 8.98453977882241e-10 1.75541601346051e-09
70.8333333333333
"chr11" 76318001 76319000 "*" 1.48487888651516e-11 3.70689457767456e-11 100
"chr11" 76365001 76366000 "*" 2.11221972812314e-05 2.20233781915554e-05 60
"chr11" 76368001 76369000 "*" 1.08604236714882e-11 2.76325361843958e-11
-56.344991759751
"chr11" 76377001 76378000 "*" 1.65312208366686e-13 5.30173174211934e-13
-54.5454545454545
"chr11" 76404001 76405000 "*" 3.2085445411667e-14 1.12142674960866e-13
68.5714285714286
"chr11" 76411001 76412000 "*" 8.22675261247241e-13 2.42016543801578e-12
-84.1726618705036
"chr11" 76412001 76413000 "*" 4.73234496034536e-09 8.30627300264826e-09 100
"chr11" 76427001 76428000 "*" 1.80023693285847e-07 2.52295437591977e-07
64.1025641025641
"chr11" 76432001 76433000 "*" 7.0006535457523e-08 1.03695832512054e-07 -100
"chr11" 76480001 76481000 "*" 3.597027969926e-07 4.84612690151399e-07
-92.8571428571429
"chr11" 76518001 76519000 "*" 0 0 -98.6486486486486
"chr11" 76762001 76763000 "*" 4.90718576884319e-14 1.6794399166207e-13 -100
"chr11" 76778001 76779000 "*" 0 0 -99.7347480106101

"chr11" 76783001 76784000 "*" 1.11022302462516e-16 5.03662826618488e-16 -100
"chr11" 76802001 76803000 "*" 2.75335310107039e-14 9.69727903855348e-14 100
"chr11" 76806001 76807000 "*" 4.7110316747645e-07 6.24717132053794e-07
54.1666666666667
"chr11" 76824001 76825000 "*" 3.5527136788005e-15 1.39277426410519e-14 -100
"chr11" 76835001 76836000 "*" 1.50623957750895e-12 4.28756717183481e-12 -100
"chr11" 76842001 76843000 "*" 7.7715611723761e-16 3.26213507634405e-15 -100
"chr11" 76869001 76870000 "*" 0 0 -54.0625
"chr11" 76873001 76874000 "*" 5.55111512312578e-16 2.36485094870365e-15 -100
"chr11" 76892001 76893000 "*" 9.37810478607304e-07 1.19512938606036e-06
68.3181818181818
"chr11" 76898001 76899000 "*" 6.59550958292954e-09 1.13456194382773e-08 -100
"chr11" 76997001 76998000 "*" 3.95353005888666e-09 7.03928529283733e-09 100
"chr11" 77122001 77123000 "*" 0 0 -54.4498704405023
"chr11" 77347001 77348000 "*" 0 0 100
"chr11" 77712001 77713000 "*" 9.08108033215171e-11 2.03407753381119e-10 -100
"chr11" 77720001 77721000 "*" 8.71525074330748e-14 2.88848349331391e-13 -100
"chr11" 77733001 77734000 "*" 8.77076189453874e-15 3.27889038038365e-14
-95.2380952380952
"chr11" 77734001 77735000 "*" 0 0 -67.4191207667022
"chr11" 77900001 77901000 "*" 3.95353005888666e-09 7.03928529283733e-09 100
"chr11" 78094001 78095000 "*" 1.67299207820548e-08 2.71377429441055e-08 100
"chr11" 78131001 78132000 "*" 8.43457970134409e-10 1.65295915064162e-09
53.8461538461538
"chr11" 78316001 78317000 "*" 4.14335232790108e-13 1.26153063645578e-12 -100
"chr11" 78436001 78437000 "*" 2.37587727269783e-14 8.44268514944447e-14 100
"chr11" 78541001 78542000 "*" 1.48087875295744e-10 3.22075573380618e-10 -100
"chr11" 78624001 78625000 "*" 1.26565424807268e-14 4.63923203146481e-14 -100
"chr11" 78652001 78653000 "*" 2.28981325012256e-08 3.63818631112906e-08
71.7948717948718
"chr11" 78867001 78868000 "*" 8.7349629751543e-09 1.47505871898511e-08 -100
"chr11" 78926001 78927000 "*" 5.794809077031e-12 1.52911192757929e-11 -100
"chr11" 78938001 78939000 "*" 6.42233020664573e-06 7.24338804013403e-06
-81.8181818181818
"chr11" 78990001 78991000 "*" 1.11022302462516e-16 5.03662826618488e-16 -100
"chr11" 79041001 79042000 "*" 1.14124265593318e-11 2.89138062669327e-11 -100
"chr11" 79199001 79200000 "*" 4.08209022140227e-11 9.57770690255255e-11 -100
"chr11" 79204001 79205000 "*" 2.29294694342741e-10 4.8692105004935e-10 -84
"chr11" 79223001 79224000 "*" 1.92946192356658e-09 3.59193011611483e-09 100
"chr11" 79321001 79322000 "*" 7.0006535457523e-08 1.03695832512054e-07 -100
"chr11" 79368001 79369000 "*" 3.91047837348113e-05 3.90826169390821e-05
-59.5744680851064
"chr11" 79438001 79439000 "*" 1.96451743761372e-11 4.8282584872431e-11
-53.7931034482759
"chr11" 79608001 79609000 "*" 1.52794177310511e-09 2.87642564232045e-09 -100
"chr11" 79904001 79905000 "*" 1.52794177310511e-09 2.87642564232045e-09 -100
"chr11" 80243001 80244000 "*" 0 0 -57.4780058651026
"chr11" 81492001 81493000 "*" 3.87601062357135e-11 9.13375868749433e-11
-55.2631578947368
"chr11" 81738001 81739000 "*" 1.37828859436695e-10 3.01445544267661e-10 -100
"chr11" 81866001 81867000 "*" 0 0 100
"chr11" 82288001 82289000 "*" 7.67056418382595e-11 1.7359448580318e-10 100

"chr11" 82430001 82431000 "*" 6.59550958292954e-09 1.13456194382773e-08 -100
"chr11" 82708001 82709000 "*" 3.5182823321378e-10 7.26934717295833e-10 -75
"chr11" 82790001 82791000 "*" 4.73234496034536e-09 8.30627300264826e-09 100
"chr11" 83392001 83393000 "*" 9.24470944241307e-10 1.80105792131285e-09 -70.5882352941177
"chr11" 84807001 84808000 "*" 0 0 -99.0291262135922
"chr11" 86317001 86318000 "*" 2.91028312560115e-11 6.97590507139978e-11 -100
"chr11" 86523001 86524000 "*" 1.55431223447522e-15 6.33705141101682e-15 -100
"chr11" 86665001 86666000 "*" 0 0 88.3211678832117
"chr11" 87088001 87089000 "*" 1.65267703956529e-05 1.75228091743656e-05 -64.2857142857143
"chr11" 87480001 87481000 "*" 3.04324343503026e-11 7.26599935673121e-11 -100
"chr11" 87997001 87998000 "*" 2.16382467499443e-13 6.82775854166559e-13 100
"chr11" 88300001 88301000 "*" 7.6800471981997e-08 1.13086385785668e-07 54.3940795559667
"chr11" 88796001 88797000 "*" 0 0 68.9655172413793
"chr11" 90796001 90797000 "*" 5.38980003775835e-05 5.27300440686403e-05 57.1428571428571
"chr11" 91374001 91375000 "*" 1.06503694752291e-12 3.08687484698138e-12 66.6666666666667
"chr11" 91409001 91410000 "*" 2.68450373042128e-10 5.64465213127486e-10 -100
"chr11" 92642001 92643000 "*" 1.98365768255826e-11 4.85612402473442e-11 -100
"chr11" 93063001 93064000 "*" 0 0 63.9354446441789
"chr11" 93281001 93282000 "*" 3.40125705378114e-10 7.03784073980162e-10 100
"chr11" 93343001 93344000 "*" 1.38590361409285e-10 3.02706178892116e-10 -100
"chr11" 93348001 93349000 "*" 1.5277158427196e-09 2.87642564232045e-09 -100
"chr11" 93686001 93687000 "*" 3.04324343503026e-11 7.26599935673121e-11 -100
"chr11" 93704001 93705000 "*" 0 0 100
"chr11" 93823001 93824000 "*" 0 0 -98.6301369863014
"chr11" 94277001 94278000 "*" 0 0 -56.3769197692626
"chr11" 94385001 94386000 "*" 9.80763359414993e-10 1.8980032424715e-09 -100
"chr11" 94532001 94533000 "*" 0 0 -82
"chr11" 94594001 94595000 "*" 3.14672488066492e-09 5.68111411923401e-09 87.2093023255814
"chr11" 94609001 94610000 "*" 1.53379531298015e-11 3.82340311777501e-11 -51.2820512820513
"chr11" 94757001 94758000 "*" 2.35737007692194e-09 4.33234850335597e-09 -78.5714285714286
"chr11" 94888001 94889000 "*" 0 0 100
"chr11" 95746001 95747000 "*" 0 0 57.6923076923077
"chr11" 96046001 96047000 "*" 3.04324343503026e-11 7.26599935673121e-11 100
"chr11" 96947001 96948000 "*" 0 0 100
"chr11" 97332001 97333000 "*" 0 0 98
"chr11" 98607001 98608000 "*" 1.58671964456403e-11 3.94719096379376e-11 66.6666666666667
"chr11" 99650001 99651000 "*" 2.08814465718632e-09 3.86232567960463e-09 100
"chr11" 99881001 99882000 "*" 5.01025332333427e-10 1.01358031135889e-09 100
"chr11" 99980001 99981000 "*" 1.12458486967171e-09 2.15740815043925e-09 -100
"chr11" 100557001 100558000 "*" 5.44009282066327e-15 2.08486137681812e-14 100
"chr11" 100997001 100998000 "*" 1.05621067447714e-11 2.69475072343073e-11 81.8181818181818
"chr11" 101000001 101001000 "*" 0 0 56.1910377358491

"chr11" 101210001 101211000 "*" 7.0006535457523e-08 1.03695832512054e-07 100
"chr11" 102261001 102262000 "*" 2.46634490608244e-10 5.21234787357618e-10
-68.0851063829787
"chr11" 102534001 102535000 "*" 0 0 100
"chr11" 102915001 102916000 "*" 1.52466927971773e-12 4.33322475981408e-12 100
"chr11" 102980001 102981000 "*" 0 0 61.9385178814194
"chr11" 103407001 103408000 "*" 3.68371999570627e-13 1.13149976091305e-12 100
"chr11" 103521001 103522000 "*" 3.04529172823198e-06 3.60576760240498e-06
-85.7142857142857
"chr11" 103797001 103798000 "*" 1.31610942394333e-05 1.4161144785177e-05
66.6666666666667
"chr11" 104618001 104619000 "*" 6.5536465143623e-13 1.94602017656154e-12 -100
"chr11" 104887001 104888000 "*" 3.88022947106492e-13 1.1867462770515e-12 -100
"chr11" 105422001 105423000 "*" 5.7065463465733e-14 1.93318145576218e-13 -100
"chr11" 105475001 105476000 "*" 1.90115718723405e-07 2.65666667785906e-07
57.8947368421053
"chr11" 105570001 105571000 "*" 2.69007038866675e-13 8.38831022628993e-13 100
"chr11" 105759001 105760000 "*" 1.56863411149288e-12 4.4469621057462e-12 -100
"chr11" 106368001 106369000 "*" 5.01025332333427e-10 1.01358031135889e-09 100
"chr11" 107099001 107100000 "*" 1.5277158427196e-09 2.87642564232045e-09 100
"chr11" 107361001 107362000 "*" 1.83952852950142e-12 5.17499701329218e-12 100
"chr11" 107461001 107462000 "*" 0 0 91.3850231941683
"chr11" 107505001 107506000 "*" 0 0 100
"chr11" 107620001 107621000 "*" 0 0 100
"chr11" 107842001 107843000 "*" 1.12458486967171e-09 2.15740815043925e-09 -100
"chr11" 108258001 108259000 "*" 2.64951482975562e-09 4.82186011375752e-09 -100
"chr11" 108890001 108891000 "*" 1.11022302462516e-16 5.03662826618488e-16 -100
"chr11" 109564001 109565000 "*" 3.33066907387547e-16 1.4495649018245e-15 100
"chr11" 109615001 109616000 "*" 0 0 100
"chr11" 109929001 109930000 "*" 3.95353005888666e-09 7.03928529283733e-09 -100
"chr11" 109974001 109975000 "*" 1.39779633423487e-07 1.98463037958589e-07 100
"chr11" 110043001 110044000 "*" 6.37490060739765e-13 1.89496793587877e-12 -100
"chr11" 110065001 110066000 "*" 3.530509218308e-14 1.22891039008692e-13 -100
"chr11" 110166001 110167000 "*" 0 0 93.8356164383562
"chr11" 110167001 110168000 "*" 0 0 78.1591852131271
"chr11" 110581001 110582000 "*" 0 0 55.1960046521174
"chr11" 110879001 110880000 "*" 1.52466927971773e-12 4.33322475981408e-12 100
"chr11" 111224001 111225000 "*" 1.84303333417013e-06 2.25408060488287e-06
-72.0930232558139
"chr11" 111231001 111232000 "*" 3.33066907387547e-16 1.4495649018245e-15 -100
"chr11" 111306001 111307000 "*" 1.46049838889439e-12 4.16860313336453e-12
92.8571428571429
"chr11" 111308001 111309000 "*" 3.6700841921089e-08 5.65530226072258e-08 100
"chr11" 111324001 111325000 "*" 6.59550958292954e-09 1.13456194382773e-08 -100
"chr11" 111664001 111665000 "*" 0 0 -100
"chr11" 111738001 111739000 "*" 4.01313771103418e-09 7.11882087237587e-09 -100
"chr11" 111798001 111799000 "*" 0 0 -100
"chr11" 111812001 111813000 "*" 0 0 -100
"chr11" 111828001 111829000 "*" 4.44089209850063e-16 1.91071758245033e-15
-74.3801652892562
"chr11" 112263001 112264000 "*" 1.13140401492018e-08 1.87801380787728e-08 -100
"chr11" 112435001 112436000 "*" 1.14124265593318e-11 2.89138062669327e-11 -100

"chr11" 112564001 112565000 "*" 1.5277158427196e-09 2.87642564232045e-09 -100
"chr11" 112617001 112618000 "*" 2.79987144580218e-12 7.68634726811898e-12 -100
"chr11" 112644001 112645000 "*" 1.5277158427196e-09 2.87642564232045e-09 100
"chr11" 112752001 112753000 "*" 6.62569998866047e-12 1.73578764556744e-11 63.75
"chr11" 112828001 112829000 "*" 7.67056418382595e-11 1.7359448580318e-10 100
"chr11" 112949001 112950000 "*" 0 0 -66.1290322580645
"chr11" 113113001 113114000 "*" 0 0 -100
"chr11" 113134001 113135000 "*" 1.67299207820548e-08 2.71377429441055e-08 -100
"chr11" 113300001 113301000 "*" 2.82773804372027e-13 8.79129664109438e-13 -100
"chr11" 113302001 113303000 "*" 4.78841410966879e-11 1.11468187156588e-10 -100
"chr11" 113345001 113346000 "*" 0 0 66.3067328483325
"chr11" 113346001 113347000 "*" 1.98721039623706e-11 4.86396466724183e-11
73.991935483871
"chr11" 113383001 113384000 "*" 4.01313771103418e-09 7.11882087237587e-09 -100
"chr11" 113385001 113386000 "*" 7.105427357601e-15 2.68362283258525e-14 -100
"chr11" 113403001 113404000 "*" 5.63660229602192e-13 1.68905058935478e-12 -100
"chr11" 113514001 113515000 "*" 0 0 -100
"chr11" 113723001 113724000 "*" 3.74147421511317e-06 4.36959554745124e-06
53.3333333333333
"chr11" 113788001 113789000 "*" 0 0 -74.2857142857143
"chr11" 113800001 113801000 "*" 1.37839755709468e-06 1.7175538899633e-06
-71.4285714285714
"chr11" 113823001 113824000 "*" 4.9293902293357e-14 1.68558663699143e-13
72.2222222222222
"chr11" 113882001 113883000 "*" 1.88261159994596e-08 3.03420719188995e-08
-91.6666666666667
"chr11" 113907001 113908000 "*" 0 0 -100
"chr11" 113931001 113932000 "*" 0 0 64.3615751789976
"chr11" 113932001 113933000 "*" 0 0 84.4357976653696
"chr11" 113939001 113940000 "*" 1.57738322403844e-09 2.96575274523453e-09
76.9230769230769
"chr11" 114011001 114012000 "*" 7.95831178734829e-10 1.56414372853608e-09
-56.0117302052786
"chr11" 114041001 114042000 "*" 0 0 -75
"chr11" 114048001 114049000 "*" 0 0 -100
"chr11" 114072001 114073000 "*" 2.4535928844216e-14 8.69144396197119e-14 -100
"chr11" 114118001 114119000 "*" 6.62958576924666e-12 1.73620572057889e-11 100
"chr11" 114122001 114123000 "*" 0 0 -71.7948717948718
"chr11" 114130001 114131000 "*" 0 0 64.9157660521297
"chr11" 114133001 114134000 "*" 1.11022302462516e-15 4.59817122935606e-15 -100
"chr11" 114238001 114239000 "*" 0.000220373109395711 0.000195434775679205
50.9803921568627
"chr11" 114479001 114480000 "*" 4.21884749357559e-15 1.63874445239189e-14 -100
"chr11" 114493001 114494000 "*" 0 0 -92.8571428571429
"chr11" 114495001 114496000 "*" 3.9190872769268e-14 1.35416418933905e-13 -100
"chr11" 114860001 114861000 "*" 8.88178419700125e-16 3.70670032207938e-15 100
"chr11" 114931001 114932000 "*" 4.73234496034536e-09 8.30627300264826e-09 -100
"chr11" 114939001 114940000 "*" 8.77076189453874e-15 3.27889038038365e-14 100
"chr11" 114949001 114950000 "*" 0 0 -100
"chr11" 114952001 114953000 "*" 2.00227675550835e-08 3.20459176617961e-08 100
"chr11" 115010001 115011000 "*" 1.12458486967171e-09 2.15740815043925e-09 100
"chr11" 115016001 115017000 "*" 4.07050848361123e-07 5.44261714716217e-07

-57.6923076923077
"chr11" 115333001 115334000 "*" 3.40547257060564e-09 6.12294196308574e-09
-63.0952380952381
"chr11" 115374001 115375000 "*" 0 0 92.4290220820189
"chr11" 115436001 115437000 "*" 6.59550958292954e-09 1.13456194382773e-08 100
"chr11" 115568001 115569000 "*" 5.48638023900594e-10 1.10374357336421e-09 100
"chr11" 115648001 115649000 "*" 4.87230505630265e-06 5.59369037847916e-06
55.2631578947368
"chr11" 115740001 115741000 "*" 2.68450373042128e-10 5.64465213127486e-10 -100
"chr11" 115803001 115804000 "*" 2.43005615629954e-09 4.45014222280278e-09 100
"chr11" 115860001 115861000 "*" 1.88737914186277e-15 7.62011598380321e-15 100
"chr11" 115943001 115944000 "*" 6.81765754961816e-12 1.78014899764838e-11
-52.1739130434783
"chr11" 116014001 116015000 "*" 2.1094237467878e-15 8.47434540879246e-15 -100
"chr11" 116064001 116065000 "*" 5.30686605770825e-14 1.8057048995867e-13
79.7752808988764
"chr11" 116070001 116071000 "*" 0 0 -71.4285714285714
"chr11" 116093001 116094000 "*" 0 0 -100
"chr11" 116183001 116184000 "*" 0 0 -73.4429400386847
"chr11" 116199001 116200000 "*" 2.22044604925031e-16 9.81641919380259e-16 -100
"chr11" 116274001 116275000 "*" 2.43005615629954e-09 4.45014222280278e-09 -100
"chr11" 116291001 116292000 "*" 8.87810713834369e-10 1.73584096752978e-09
-75.609756097561
"chr11" 116377001 116378000 "*" 3.34128893442198e-08 5.19772864618523e-08 -100
"chr11" 116402001 116403000 "*" 6.24654217240561e-10 1.2462108191303e-09 -100
"chr11" 116406001 116407000 "*" 3.77475828372553e-15 1.47379557022071e-14 -100
"chr11" 116430001 116431000 "*" 1.20989991125864e-05 1.30911385395102e-05
-56.078431372549
"chr11" 116659001 116660000 "*" 2.02060590481778e-13 6.40127405465654e-13 -100
"chr11" 116708001 116709000 "*" 0 0 76.9230769230769
"chr11" 116726001 116727000 "*" 8.19387890871326e-11 1.84980973898163e-10
-52.9411764705882
"chr11" 117170001 117171000 "*" 6.36960155975252e-06 7.18732018484993e-06
-53.6585365853659
"chr11" 117199001 117200000 "*" 3.40125705378114e-10 7.03784073980162e-10 -100
"chr11" 117294001 117295000 "*" 0.000138951373611085 0.000127295950042963 70
"chr11" 117302001 117303000 "*" 2.75593159404508e-09 5.00656986090238e-09
59.4936708860759
"chr11" 117331001 117332000 "*" 0 0 -72.005772005772
"chr11" 117344001 117345000 "*" 4.71134242729931e-12 1.257615036806e-11 -100
"chr11" 117373001 117374000 "*" 0 0 80.3571428571429
"chr11" 117396001 117397000 "*" 1.52794177310511e-09 2.87642564232045e-09 100
"chr11" 117398001 117399000 "*" 8.12104155656357e-06 9.01864929849813e-06
53.8461538461538
"chr11" 117408001 117409000 "*" 4.99900121297969e-12 1.32921056339624e-11 -100
"chr11" 117422001 117423000 "*" 1.51264178960275e-09 2.85982317045676e-09 -80
"chr11" 117456001 117457000 "*" 0 0 100
"chr11" 117464001 117465000 "*" 9.5812247025151e-14 3.15667542866807e-13 -100
"chr11" 117511001 117512000 "*" 7.0006535457523e-08 1.03695832512054e-07 -100
"chr11" 117520001 117521000 "*" 5.2326543098502e-10 1.05667558460774e-09
-52.7272727272727
"chr11" 117647001 117648000 "*" 0 0 -76.6666666666667

"chr11" 117669001 117670000 "*" 6.77335965093562e-12 1.76897011246462e-11 100
"chr11" 117685001 117686000 "*" 1.2179146580138e-13 3.95849303694312e-13
-83.3333333333333
"chr11" 117707001 117708000 "*" 1.11022302462516e-16 5.03662826618488e-16
-61.5384615384615
"chr11" 117740001 117741000 "*" 4.75887544926223e-08 7.23914801846659e-08 -75
"chr11" 117746001 117747000 "*" 0 0 100
"chr11" 117747001 117748000 "*" 0 0 57.1621621621622
"chr11" 117771001 117772000 "*" 6.13509243407862e-13 1.83243557273814e-12
65.9090909090909
"chr11" 117800001 117801000 "*" 1.64371621091952e-07 2.31378405365712e-07
92.8571428571429
"chr11" 117901001 117902000 "*" 6.15840711759574e-13 1.83676803572802e-12 100
"chr11" 117928001 117929000 "*" 0.000231175672664174 0.000204278487663226
-54.5454545454545
"chr11" 117959001 117960000 "*" 3.34128893442198e-08 5.19772864618523e-08 100
"chr11" 117981001 117982000 "*" 3.95353005888666e-09 7.03928529283733e-09 100
"chr11" 118003001 118004000 "*" 6.15840711759574e-13 1.83676803572802e-12 100
"chr11" 118011001 118012000 "*" 3.65107943878229e-12 9.90602415539447e-12 -87.5
"chr11" 118186001 118187000 "*" 1.13564713188907e-12 3.27956240689702e-12
-94.3661971830986
"chr11" 118489001 118490000 "*" 2.9917416156211e-05 3.0473422476587e-05
-60.8695652173913
"chr11" 118493001 118494000 "*" 5.9313019051821e-08 8.91097063444377e-08 -75
"chr11" 118566001 118567000 "*" 2.22044604925031e-16 9.81641919380259e-16
58.0645161290323
"chr11" 118615001 118616000 "*" 4.01313771103418e-09 7.11882087237587e-09 100
"chr11" 118666001 118667000 "*" 3.76365605347928e-14 1.3043487970679e-13 100
"chr11" 118705001 118706000 "*" 1.52466927971773e-12 4.33322475981408e-12 100
"chr11" 118711001 118712000 "*" 1.86444082395099e-10 4.01761559073664e-10
56.1422413793103
"chr11" 118727001 118728000 "*" 3.95353005888666e-09 7.03928529283733e-09 100
"chr11" 118782001 118783000 "*" 3.29350413608154e-09 5.93352213988679e-09
-73.2142857142857
"chr11" 118783001 118784000 "*" 1.39307460789162e-07 1.98370229491695e-07
-72.1311475409836
"chr11" 118788001 118789000 "*" 3.02357913151141e-08 4.73793946854452e-08
70.8333333333333
"chr11" 118790001 118791000 "*" 3.25818815749557e-05 3.29735679006858e-05
66.6666666666667
"chr11" 118811001 118812000 "*" 4.16111589629509e-12 1.11897841594988e-11
-89.2156862745098
"chr11" 118831001 118832000 "*" 2.84573726472281e-07 3.8881484784083e-07 75
"chr11" 118889001 118890000 "*" 2.89801516117905e-12 7.94270405097209e-12
-66.6666666666667
"chr11" 119027001 119028000 "*" 4.69446703732501e-12 1.25467656726221e-11
77.6119402985075
"chr11" 119039001 119040000 "*" 0 0 -68.3453237410072
"chr11" 119048001 119049000 "*" 4.28478353331485e-06 4.95677832652837e-06
-71.1538461538462
"chr11" 119187001 119188000 "*" 0 0 59.7866630276565
"chr11" 119197001 119198000 "*" 4.83744390378238e-06 5.55756940206571e-06 70

"chr11" 119205001 119206000 "*" 0 0 -100
"chr11" 119221001 119222000 "*" 1.93720595120794e-11 4.76309924836636e-11 100
"chr11" 119227001 119228000 "*" 0 0 -91.0127431254192
"chr11" 119249001 119250000 "*" 1.0769163338864e-14 3.98631098593833e-14 -100
"chr11" 119288001 119289000 "*" 5.55111512312578e-16 2.36485094870365e-15 -100
"chr11" 119302001 119303000 "*" 1.21610397307848e-08 2.01130186095469e-08 53.315649867374
"chr11" 119317001 119318000 "*" 5.45032019516611e-10 1.09847636109535e-09 -59.1836734693878
"chr11" 119341001 119342000 "*" 4.84712270321097e-12 1.29241155965803e-11 84.9056603773585
"chr11" 119344001 119345000 "*" 0 0 79.3548387096774
"chr11" 119347001 119348000 "*" 0 0 100
"chr11" 119364001 119365000 "*" 2.02060590481778e-13 6.40127405465654e-13 -100
"chr11" 119367001 119368000 "*" 6.58243449525031e-07 8.57004003654891e-07 81.8181818181818
"chr11" 119373001 119374000 "*" 3.15936166117581e-12 8.62553897215534e-12 58.3333333333333
"chr11" 119392001 119393000 "*" 0 0 66.6666666666667
"chr11" 119394001 119395000 "*" 1.268418703404e-11 3.19888127950687e-11 68.75
"chr11" 119396001 119397000 "*" 2.17104112465449e-12 6.04925641273751e-12 90.1408450704225
"chr11" 119402001 119403000 "*" 7.00597672653736e-07 9.08488310757339e-07 67.8571428571429
"chr11" 119496001 119497000 "*" 5.55111512312578e-16 2.36485094870365e-15 58.3636363636364
"chr11" 119499001 119500000 "*" 1.50938729071015e-07 2.13471175245767e-07 70.3703703703704
"chr11" 119509001 119510000 "*" 1.55431223447522e-15 6.33705141101682e-15 87.2340425531915
"chr11" 119514001 119515000 "*" 6.83142431512351e-12 1.78352026663651e-11 -66.6666666666667
"chr11" 119540001 119541000 "*" 2.93841121545846e-07 4.00649931094887e-07 -70.4941860465116
"chr11" 119552001 119553000 "*" 4.01313771103418e-09 7.11882087237587e-09 100
"chr11" 119567001 119568000 "*" 8.05772648604375e-09 1.37043457986262e-08 -65.4471544715447
"chr11" 119571001 119572000 "*" 0 0 -100
"chr11" 119585001 119586000 "*" 0 0 -71.4285714285714
"chr11" 119588001 119589000 "*" 3.21964677141295e-15 1.26720521345953e-14 87.5
"chr11" 119618001 119619000 "*" 1.88737914186277e-15 7.62011598380321e-15 97.6190476190476
"chr11" 119619001 119620000 "*" 3.46500605985511e-13 1.06777978272295e-12 -100
"chr11" 119633001 119634000 "*" 2.64951482975562e-09 4.82186011375752e-09 -100
"chr11" 119660001 119661000 "*" 9.3543617296632e-11 2.09236788060014e-10 -77.2727272727273
"chr11" 119668001 119669000 "*" 2.78703282674542e-10 5.85035564742876e-10 67.6515151515151
"chr11" 119694001 119695000 "*" 3.95353005888666e-09 7.03928529283733e-09 -100
"chr11" 119723001 119724000 "*" 4.01313771103418e-09 7.11882087237587e-09 -100
"chr11" 119734001 119735000 "*" 1.36471001166427e-09 2.59372372847633e-09 -71.7391304347826

"chr11" 119744001 119745000 "*" 2.22044604925031e-16 9.81641919380259e-16
-56.5664160401003
"chr11" 119747001 119748000 "*" 2.71893618730701e-13 8.46692454027087e-13 -100
"chr11" 119764001 119765000 "*" 0 0 100
"chr11" 119792001 119793000 "*" 1.5277158427196e-09 2.87642564232045e-09 -100
"chr11" 119812001 119813000 "*" 6.92287338566189e-11 1.57605630124247e-10 -100
"chr11" 119847001 119848000 "*" 1.11022302462516e-16 5.03662826618488e-16
67.741935483871
"chr11" 119848001 119849000 "*" 9.2148511043888e-15 3.43705279328267e-14
63.4033613445378
"chr11" 119853001 119854000 "*" 1.50354173555911e-11 3.75146379072152e-11
55.267947421638
"chr11" 119902001 119903000 "*" 0 0 -84.8837209302326
"chr11" 119911001 119912000 "*" 2.08995487582797e-10 4.46944006972663e-10 -100
"chr11" 119952001 119953000 "*" 0 0 -79.3248945147679
"chr11" 119955001 119956000 "*" 6.40784549155171e-05 6.19674252924143e-05
-53.5714285714286
"chr11" 119985001 119986000 "*" 7.67056418382595e-11 1.7359448580318e-10 100
"chr11" 119988001 119989000 "*" 1.38590361409285e-10 3.02706178892116e-10 -100
"chr11" 119993001 119994000 "*" 2.08995487582797e-10 4.46944006972663e-10 100
"chr11" 120003001 120004000 "*" 0 0 76.271186440678
"chr11" 120039001 120040000 "*" 0 0 60.4364900534346
"chr11" 120043001 120044000 "*" 8.68395256592436e-07 1.11166185180334e-06
-63.4328358208955
"chr11" 120050001 120051000 "*" 3.98556564640273e-07 5.33562809588447e-07 80
"chr11" 120057001 120058000 "*" 0 0 -95.5882352941177
"chr11" 120059001 120060000 "*" 1.83952852950142e-12 5.17499701329218e-12 -100
"chr11" 120063001 120064000 "*" 0 0 -100
"chr11" 120070001 120071000 "*" 0 0 -100
"chr11" 120397001 120398000 "*" 0 0 66.304347826087
"chr11" 120418001 120419000 "*" 0 0 -71.4739229024943
"chr11" 120419001 120420000 "*" 0.000896607310246877 0.000717057981883268
-53.8461538461538
"chr11" 120422001 120423000 "*" 8.71683656367139e-08 1.27477291339692e-07 -75
"chr11" 120495001 120496000 "*" 0 0 100
"chr11" 120518001 120519000 "*" 2.32869279415127e-12 6.47187850513267e-12
-52.9411764705882
"chr11" 120563001 120564000 "*" 0 0 -92.0353982300885
"chr11" 120577001 120578000 "*" 2.44249065417534e-15 9.75180348030994e-15 100
"chr11" 120585001 120586000 "*" 1.98365768255826e-11 4.85612402473442e-11 100
"chr11" 120589001 120590000 "*" 0 0 -100
"chr11" 120655001 120656000 "*" 8.02691246803988e-14 2.67352274101148e-13 -100
"chr11" 120699001 120700000 "*" 0 0 100
"chr11" 120730001 120731000 "*" 0.000265908371390844 0.000232535857460809
-53.5714285714286
"chr11" 120753001 120754000 "*" 1.07429442008922e-07 1.55283286341352e-07
86.6666666666667
"chr11" 120754001 120755000 "*" 4.70147631936868e-09 8.27630288582799e-09
-55.1724137931034
"chr11" 120765001 120766000 "*" 4.73234496034536e-09 8.30627300264826e-09 -100
"chr11" 120769001 120770000 "*" 2.11601514131798e-05 2.20604694656489e-05
-54.3478260869565

"chr11" 120775001 120776000 "*" 1.39230815476843e-06 1.73395458284971e-06 75
"chr11" 120789001 120790000 "*" 3.90090636348539e-07 5.22942647884215e-07 72.7272727272727
"chr11" 120793001 120794000 "*" 1.11022302462516e-16 5.03662826618488e-16 -100
"chr11" 120838001 120839000 "*" 4.6087075733503e-07 6.11994751690818e-07 -54.0740740740741
"chr11" 120968001 120969000 "*" 1.83952852950142e-12 5.17499701329218e-12 100
"chr11" 120976001 120977000 "*" 7.0006535457523e-08 1.03695832512054e-07 -100
"chr11" 121084001 121085000 "*" 0 0 -100
"chr11" 121213001 121214000 "*" 0 0 -100
"chr11" 121220001 121221000 "*" 2.91366930582626e-12 7.98429606851069e-12 -63.8297872340426
"chr11" 121298001 121299000 "*" 0 0 100
"chr11" 121446001 121447000 "*" 1.33445109407049e-08 2.19697088944398e-08 -82.0512820512821
"chr11" 121487001 121488000 "*" 5.99520433297585e-15 2.28531569014092e-14 -60
"chr11" 121567001 121568000 "*" 2.22044604925031e-16 9.81641919380259e-16 100
"chr11" 121593001 121594000 "*" 0 0 62.0809655962292
"chr11" 121917001 121918000 "*" 6.52733422867868e-12 1.71146951718423e-11 100
"chr11" 121972001 121973000 "*" 8.01051639498507e-06 8.90361414875024e-06 64.8648648648649
"chr11" 122073001 122074000 "*" 6.733526292102e-09 1.15706370487096e-08 -65
"chr11" 122083001 122084000 "*" 1.18302034834983e-11 2.99192532922064e-11 94.7368421052632
"chr11" 122100001 122101000 "*" 3.07531777821168e-14 1.07626052665081e-13 100
"chr11" 122495001 122496000 "*" 1.20591092667155e-10 2.66075324129686e-10 -100
"chr11" 122697001 122698000 "*" 5.03642644456015e-06 5.77170324987535e-06 -55.5555555555556
"chr11" 122727001 122728000 "*" 3.6700841921089e-08 5.65530226072258e-08 100
"chr11" 122753001 122754000 "*" 0 0 54.8165153238826
"chr11" 122783001 122784000 "*" 0 0 100
"chr11" 122800001 122801000 "*" 1.12634901405784e-10 2.49563754426169e-10 100
"chr11" 122851001 122852000 "*" 0.000243367436658426 0.000214153219492301 -52.6315789473684
"chr11" 122861001 122862000 "*" 3.99036463960556e-06 4.63960979386448e-06 57.8947368421053
"chr11" 122978001 122979000 "*" 1.95399252334028e-14 7.01253661173697e-14 -100
"chr11" 123087001 123088000 "*" 2.88657986402541e-15 1.14155613124573e-14 -100
"chr11" 123172001 123173000 "*" 0 0 68.3574685332648
"chr11" 123272001 123273000 "*" 4.2478473696872e-05 4.22286970764066e-05 -70
"chr11" 123359001 123360000 "*" 1.98365768255826e-11 4.85612402473442e-11 -100
"chr11" 123412001 123413000 "*" 2.55351295663786e-15 1.01678090381833e-14 100
"chr11" 123461001 123462000 "*" 1.24364827591705e-06 1.55889581418267e-06 53.1746031746032
"chr11" 123498001 123499000 "*" 2.22044604925031e-16 9.81641919380259e-16 100
"chr11" 123524001 123525000 "*" 0 0 54.0956749672346
"chr11" 123809001 123810000 "*" 0 0 -100
"chr11" 123986001 123987000 "*" 1.39779633423487e-07 1.98463037958589e-07 -100
"chr11" 124006001 124007000 "*" 3.19300141882195e-13 9.87864167952596e-13 -66.4322250639386
"chr11" 124072001 124073000 "*" 3.24001809826058e-07 4.39253174013075e-07 58.974358974359

"chr11" 124246001 124247000 "*" 2.43005615629954e-09 4.45014222280278e-09 -100
"chr11" 124295001 124296000 "*" 6.99440505513849e-15 2.64480529781883e-14 100
"chr11" 124333001 124334000 "*" 1.06793954346429e-07 1.54420926950348e-07 -80
"chr11" 124342001 124343000 "*" 1.39779633423487e-07 1.98463037958589e-07 -100
"chr11" 124406001 124407000 "*" 6.59550958292954e-09 1.13456194382773e-08 100
"chr11" 124709001 124710000 "*" 0 0 55.6515787232781
"chr11" 124713001 124714000 "*" 0 0 76.7799490229397
"chr11" 124716001 124717000 "*" 1.35036426485158e-12 3.86629547363774e-12 84
"chr11" 124745001 124746000 "*" 0 0 -73.5849056603774
"chr11" 124757001 124758000 "*" 1.27221598700267e-06 1.59262255729358e-06
53.3333333333333
"chr11" 124805001 124806000 "*" 2.0335187977949e-09 3.77654059492906e-09
-54.8780487804878
"chr11" 124910001 124911000 "*" 1.20591092667155e-10 2.66075324129686e-10 -100
"chr11" 124918001 124919000 "*" 2.22044604925031e-16 9.81641919380259e-16 -100
"chr11" 124932001 124933000 "*" 0 0 -70.3999408590227
"chr11" 125034001 125035000 "*" 0 0 69.0019193857965
"chr11" 125091001 125092000 "*" 8.37541491716109e-09 1.42148034493616e-08
56.7567567567568
"chr11" 125098001 125099000 "*" 2.00227675550835e-08 3.20459176617961e-08 -100
"chr11" 125145001 125146000 "*" 7.0006535457523e-08 1.03695832512054e-07 100
"chr11" 125160001 125161000 "*" 1.29037891483108e-11 3.24582280286866e-11 -100
"chr11" 125176001 125177000 "*" 8.01666433236647e-09 1.36392368970109e-08 100
"chr11" 125216001 125217000 "*" 1.67299207820548e-08 2.71377429441055e-08 100
"chr11" 125237001 125238000 "*" 1.12055920098442e-11 2.84500040664577e-11 -100
"chr11" 125255001 125256000 "*" 9.0072393987839e-13 2.63157725646847e-12 100
"chr11" 125263001 125264000 "*" 1.15727342153704e-08 1.91891148635522e-08
-85.7142857142857
"chr11" 125757001 125758000 "*" 0 0 -62.3764564537335
"chr11" 125781001 125782000 "*" 1.52794177310511e-09 2.87642564232045e-09 -100
"chr11" 125796001 125797000 "*" 5.38014632844863e-10 1.08506747658349e-09
-70.5882352941177
"chr11" 125956001 125957000 "*" 1.10442103402608e-05 1.20217549185931e-05
59.2592592592593
"chr11" 125980001 125981000 "*" 2.08995487582797e-10 4.46944006972663e-10 100
"chr11" 125983001 125984000 "*" 0 0 -95.1923076923077
"chr11" 125985001 125986000 "*" 3.40125705378114e-10 7.03784073980162e-10 100
"chr11" 126009001 126010000 "*" 1.63847824197205e-11 4.0693933100105e-11
81.8181818181818
"chr11" 126031001 126032000 "*" 1.70344253147547e-07 2.39410014315768e-07
-58.3333333333333
"chr11" 126139001 126140000 "*" 0 0 -100
"chr11" 126152001 126153000 "*" 1.05582209641852e-13 3.46296597126441e-13
60.1449275362319
"chr11" 126174001 126175000 "*" 0 0 100
"chr11" 126254001 126255000 "*" 7.44056039003738e-10 1.46691232861555e-09
-69.2307692307692
"chr11" 126256001 126257000 "*" 4.75366453119852e-05 4.6905450034535e-05
57.6923076923077
"chr11" 126295001 126296000 "*" 1.48639434094378e-10 3.23216154785743e-10
-55.8823529411765
"chr11" 126300001 126301000 "*" 7.67056418382595e-11 1.7359448580318e-10 -100

"chr11" 126324001 126325000 "*" 0.000354266937660941 0.000303242292809723 56.5217391304348
"chr11" 126327001 126328000 "*" 0 0 -97.5757575757576
"chr11" 126330001 126331000 "*" 2.08995487582797e-10 4.46944006972663e-10 100
"chr11" 126346001 126347000 "*" 2.30824880542713e-07 3.19088957836092e-07 -69.2307692307692
"chr11" 126352001 126353000 "*" 2.68450373042128e-10 5.64465213127486e-10 -100
"chr11" 126368001 126369000 "*" 3.6700841921089e-08 5.65530226072258e-08 100
"chr11" 126369001 126370000 "*" 1.3326482173559e-05 1.43296704756074e-05 -54.5454545454545
"chr11" 126388001 126389000 "*" 5.18047281738987e-08 7.83858639675763e-08 -67.1641791044776
"chr11" 126390001 126391000 "*" 1.11022302462516e-15 4.59817122935606e-15 100
"chr11" 126399001 126400000 "*" 9.4325578403609e-07 1.20146295354564e-06 -70
"chr11" 126411001 126412000 "*" 1.54630752646767e-11 3.85079502648228e-11 100
"chr11" 126413001 126414000 "*" 3.12379488263126e-05 3.1728595486819e-05 51.6129032258064
"chr11" 126448001 126449000 "*" 2.47154741117583e-10 5.22281340577481e-10 -53.760593220339
"chr11" 126453001 126454000 "*" 1.22124532708767e-15 5.03921984217778e-15 -100
"chr11" 126462001 126463000 "*" 2.83981232693797e-05 2.90326224386187e-05 54.7169811320755
"chr11" 126499001 126500000 "*" 2.58011042203465e-05 2.65382822081383e-05 -51.4705882352941
"chr11" 126569001 126570000 "*" 2.00227675550835e-08 3.20459176617961e-08 -100
"chr11" 126618001 126619000 "*" 1.95399252334028e-13 6.21053118598154e-13 -74.025974025974
"chr11" 126813001 126814000 "*" 2.00227675550835e-08 3.20459176617961e-08 100
"chr11" 127007001 127008000 "*" 2.1094237467878e-15 8.47434540879246e-15 100
"chr11" 127082001 127083000 "*" 3.95353005888666e-09 7.03928529283733e-09 -100
"chr11" 127102001 127103000 "*" 8.7349629751543e-09 1.47505871898511e-08 100
"chr11" 127116001 127117000 "*" 0 0 -100
"chr11" 127282001 127283000 "*" 1.56631929648654e-10 3.39771540189748e-10 -57.6923076923077
"chr11" 127718001 127719000 "*" 1.5277158427196e-09 2.87642564232045e-09 100
"chr11" 127902001 127903000 "*" 1.67299207820548e-08 2.71377429441055e-08 100
"chr11" 127921001 127922000 "*" 4.08209022140227e-11 9.57770690255255e-11 -100
"chr11" 128023001 128024000 "*" 6.46149800331841e-14 2.17664365839349e-13 100
"chr11" 128024001 128025000 "*" 1.46913487153455e-07 2.0800406109474e-07 81.8181818181818
"chr11" 128350001 128351000 "*" 0 0 67.0731707317073
"chr11" 128438001 128439000 "*" 1.56863411149288e-12 4.4469621057462e-12 100
"chr11" 128488001 128489000 "*" 2.08472959117856e-07 2.8990062675722e-07 62.5
"chr11" 128559001 128560000 "*" 0 0 60.2660497397339
"chr11" 128596001 128597000 "*" 1.92946192356658e-09 3.59193011611483e-09 100
"chr11" 128604001 128605000 "*" 4.2542187578043e-05 4.2288507820221e-05 53.8461538461538
"chr11" 128635001 128636000 "*" 2.33146835171283e-15 9.32818831833246e-15 63.6363636363636
"chr11" 128702001 128703000 "*" 0 0 67.7835610496638
"chr11" 128763001 128764000 "*" 6.11406148820492e-07 7.99681626961299e-07 -68.5185185185185

"chr11" 128768001 128769000 "*" 2.00227675550835e-08 3.20459176617961e-08 -100
"chr11" 128773001 128774000 "*" 1.35036426485158e-12 3.86629547363774e-12 -100
"chr11" 128783001 128784000 "*" 7.0006535457523e-08 1.03695832512054e-07 100
"chr11" 128796001 128797000 "*" 5.79425174507264e-10 1.16220450687095e-09 56.1643835616438
"chr11" 128803001 128804000 "*" 5.39619190149665e-05 5.27870047241069e-05 59.2592592592593
"chr11" 128805001 128806000 "*" 0 0 69.5652173913043
"chr11" 128912001 128913000 "*" 1.67299207820548e-08 2.71377429441055e-08 100
"chr11" 129244001 129245000 "*" 0 0 100
"chr11" 129250001 129251000 "*" 8.59201598757409e-13 2.51731933442864e-12 97.7272727272727
"chr11" 129261001 129262000 "*" 1.12458486967171e-09 2.15740815043925e-09 -100
"chr11" 129298001 129299000 "*" 8.63487059632462e-12 2.22427719052604e-11 -77.9069767441861
"chr11" 129315001 129316000 "*" 0 0 77.8552746294682
"chr11" 129458001 129459000 "*" 9.65729496371637e-10 1.87255912493232e-09 -100
"chr11" 129459001 129460000 "*" 2.00227675550835e-08 3.20459176617961e-08 100
"chr11" 129479001 129480000 "*" 2.16589898094366e-06 2.62321901854342e-06 66.6666666666667
"chr11" 129590001 129591000 "*" 2.33028818463765e-10 4.93719283454501e-10 -100
"chr11" 129601001 129602000 "*" 1.88737914186277e-15 7.62011598380321e-15 100
"chr11" 129673001 129674000 "*" 2.16382467499443e-13 6.82775854166559e-13 -100
"chr11" 129696001 129697000 "*" 6.2111909815421e-07 8.11552637786282e-07 57.1428571428571
"chr11" 129871001 129872000 "*" 1.77635683940025e-15 7.19870740878856e-15 55.5555555555556
"chr11" 129990001 129991000 "*" 2.79987144580218e-12 7.68634726811898e-12 100
"chr11" 130051001 130052000 "*" 0 0 100
"chr11" 130083001 130084000 "*" 0 0 -60.4195804195804
"chr11" 130232001 130233000 "*" 3.33066907387547e-16 1.4495649018245e-15 100
"chr11" 130261001 130262000 "*" 0 0 100
"chr11" 130298001 130299000 "*" 0 0 55.3994514242477
"chr11" 130322001 130323000 "*" 5.08482145278322e-14 1.73432706170696e-13 100
"chr11" 130457001 130458000 "*" 0 0 100
"chr11" 130478001 130479000 "*" 4.73234496034536e-09 8.30627300264826e-09 -100
"chr11" 130542001 130543000 "*" 2.88710388929303e-10 6.03399264731139e-10 100
"chr11" 130635001 130636000 "*" 1.67299207820548e-08 2.71377429441055e-08 100
"chr11" 130641001 130642000 "*" 1.23694509277517e-05 1.3360368613825e-05 69.4444444444444
"chr11" 130643001 130644000 "*" 2.53228154811769e-06 3.03378696866317e-06 -61.5384615384615
"chr11" 130655001 130656000 "*" 0 0 100
"chr11" 130694001 130695000 "*" 6.59550958292954e-09 1.13456194382773e-08 -100
"chr11" 130700001 130701000 "*" 1.48087875295744e-10 3.22075573380618e-10 -100
"chr11" 130702001 130703000 "*" 6.4152538836737e-10 1.27477697728417e-09 -100
"chr11" 130738001 130739000 "*" 3.04324343503026e-11 7.26599935673121e-11 -100
"chr11" 130786001 130787000 "*" 0 0 68.1214421252372
"chr11" 130863001 130864000 "*" 7.0006535457523e-08 1.03695832512054e-07 100
"chr11" 131067001 131068000 "*" 9.41469124882133e-14 3.10868561210786e-13 -100
"chr11" 131105001 131106000 "*" 1.0661817333002e-05 1.16323879104036e-05 61.7647058823529

"chr11" 131143001 131144000 "*" 1.07967738094406e-06 1.36441369284731e-06
-70.3703703703704
"chr11" 131167001 131168000 "*" 3.46706228993021e-06 4.06854288553042e-06
-66.2790697674419
"chr11" 131289001 131290000 "*" 8.7349629751543e-09 1.47505871898511e-08 -100
"chr11" 131317001 131318000 "*" 1.11022302462516e-16 5.03662826618488e-16
79.6875
"chr11" 131318001 131319000 "*" 7.57266626394415e-06 8.44903872709532e-06
-62.962962962963
"chr11" 131454001 131455000 "*" 6.02187189002734e-11 1.38461709497824e-10
-75.5555555555556
"chr11" 131480001 131481000 "*" 1.14352971536391e-14 4.21921674335999e-14 -100
"chr11" 131483001 131484000 "*" 0 0 -100
"chr11" 131526001 131527000 "*" 2.06528016910568e-10 4.42529623978385e-10
-51.3736263736264
"chr11" 131533001 131534000 "*" 2.1094237467878e-15 8.47434540879246e-15 -100
"chr11" 131552001 131553000 "*" 0 0 -93.75
"chr11" 131559001 131560000 "*" 5.794809077031e-12 1.52911192757929e-11 -100
"chr11" 131754001 131755000 "*" 8.7349629751543e-09 1.47505871898511e-08 -100
"chr11" 131779001 131780000 "*" 4.99900121297969e-12 1.32921056339624e-11 -100
"chr11" 131788001 131789000 "*" 1.52794177310511e-09 2.87642564232045e-09 -100
"chr11" 131803001 131804000 "*" 3.6700841921089e-08 5.65530226072258e-08 100
"chr11" 132125001 132126000 "*" 8.7349629751543e-09 1.47505871898511e-08 -100
"chr11" 132126001 132127000 "*" 0 0 65.6862745098039
"chr11" 132177001 132178000 "*" 5.77032743809269e-09 1.00287438281263e-08
-61.9047619047619
"chr11" 132307001 132308000 "*" 1.02859987194392e-09 1.98601565512361e-09
84.0909090909091
"chr11" 132360001 132361000 "*" 5.08482145278322e-14 1.73432706170696e-13 -100
"chr11" 132642001 132643000 "*" 1.49972523288966e-09 2.83643172292987e-09
-66.6666666666667
"chr11" 132707001 132708000 "*" 1.11022302462516e-16 5.03662826618488e-16 100
"chr11" 132778001 132779000 "*" 1.89828819330273e-10 4.08627292924826e-10
-73.6111111111111
"chr11" 132780001 132781000 "*" 0 0 -100
"chr11" 132874001 132875000 "*" 1.5277158427196e-09 2.87642564232045e-09 100
"chr11" 132887001 132888000 "*" 2.33028818463765e-10 4.93719283454501e-10 -100
"chr11" 132928001 132929000 "*" 6.66133814775094e-16 2.81595744474255e-15 -100
"chr11" 132945001 132946000 "*" 1.3748299054761e-08 2.25930087708667e-08
-82.6086956521739
"chr11" 132948001 132949000 "*" 0 0 67.3469387755102
"chr11" 133023001 133024000 "*" 0 0 -57.2439400308253
"chr11" 133053001 133054000 "*" 4.01313771103418e-09 7.11882087237587e-09 -100
"chr11" 133131001 133132000 "*" 3.77475828372553e-15 1.47379557022071e-14 -100
"chr11" 133184001 133185000 "*" 1.43857914025602e-07 2.03958356044912e-07
-60.2941176470588
"chr11" 133188001 133189000 "*" 1.22228082877207e-07 1.75285564709212e-07
-64.5161290322581
"chr11" 133196001 133197000 "*" 3.49116291431528e-11 8.28516120674536e-11
-70.4545454545455
"chr11" 133212001 133213000 "*" 7.65537620739387e-07 9.87630651953536e-07
-83.3333333333333

"chr11" 133232001 133233000 "*" 0 0 -77.6190476190476
"chr11" 133416001 133417000 "*" 1.49383446723661e-08 2.444556877867e-08
-66.6666666666667
"chr11" 133445001 133446000 "*" 1.79186101640916e-05 1.88985869234961e-05
63.0434782608696
"chr11" 133466001 133467000 "*" 0 0 -60.4761904761905
"chr11" 133563001 133564000 "*" 5.55111512312578e-16 2.36485094870365e-15
-72.3684210526316
"chr11" 133584001 133585000 "*" 1.90181204118289e-13 6.05298617539364e-13 -100
"chr11" 133700001 133701000 "*" 9.65338919911574e-13 2.80678910656113e-12 100
"chr11" 133704001 133705000 "*" 1.93616595708379e-05 2.02976042071257e-05
-81.8181818181818
"chr11" 133773001 133774000 "*" 8.19565414096335e-05 7.7896169099937e-05
54.5454545454545
"chr11" 133785001 133786000 "*" 0 0 100
"chr11" 133793001 133794000 "*" 0 0 67.6470588235294
"chr11" 133798001 133799000 "*" 0 0 91.4110429447853
"chr11" 133803001 133804000 "*" 4.44089209850063e-16 1.91071758245033e-15
51.5151515151515
"chr11" 133816001 133817000 "*" 0 0 65.0645994832041
"chr11" 133817001 133818000 "*" 0 0 61.2352941176471
"chr11" 133819001 133820000 "*" 9.65729496371637e-10 1.87255912493232e-09 100
"chr11" 133836001 133837000 "*" 1.55852886152275e-09 2.93142674496256e-09
57.1428571428571
"chr11" 133842001 133843000 "*" 3.43758097542946e-08 5.33736664322045e-08
58.1900452488688
"chr11" 133866001 133867000 "*" 0 0 100
"chr11" 133894001 133895000 "*" 3.69469364791541e-06 4.31919521916483e-06
73.1707317073171
"chr11" 133920001 133921000 "*" 0 0 -52.2980812137439
"chr11" 134153001 134154000 "*" 2.54373622166071e-09 4.64852477745664e-09
-91.1764705882353
"chr11" 134227001 134228000 "*" 0 0 -100
"chr11" 134259001 134260000 "*" 0 0 54.7004947889252
"chr11" 134264001 134265000 "*" 2.44249065417534e-15 9.75180348030994e-15
-70.1174215192907
"chr11" 134271001 134272000 "*" 6.18660678242122e-11 1.41987208654994e-10
68.1818181818182
"chr11" 134300001 134301000 "*" 2.72870614992371e-12 7.51299048328333e-12 60
"chr11" 134310001 134311000 "*" 9.08108033215171e-11 2.03407753381119e-10 100
"chr11" 134337001 134338000 "*" 3.77475828372553e-15 1.47379557022071e-14 -100
"chr11" 134374001 134375000 "*" 3.09730587336743e-05 3.14813986602943e-05
65.7142857142857
"chr11" 134413001 134414000 "*" 4.73234496034536e-09 8.30627300264826e-09 -100
"chr11" 134429001 134430000 "*" 0 0 -82.0987654320988
"chr11" 134444001 134445000 "*" 1.11022302462516e-15 4.59817122935606e-15
65.9574468085106
"chr11" 134483001 134484000 "*" 0 0 -77.4193548387097
"chr11" 134493001 134494000 "*" 0.000240479789304304 0.000211878237500588
57.1428571428571
"chr11" 134501001 134502000 "*" 0 0 100
"chr11" 134505001 134506000 "*" 4.98181667085706e-06 5.71226053845047e-06

-56.1604584527221
"chr11" 134595001 134596000 "*" 0 0 -66.1654135338346
"chr11" 134596001 134597000 "*" 3.67594843453389e-13 1.13011155066644e-12
-92.5925925925926
"chr11" 134608001 134609000 "*" 0 0 -61.4814814814815
"chr11" 134624001 134625000 "*" 0 0 -74.1379310344828
"chr11" 134629001 134630000 "*" 0 0 -72.2222222222222
"chr11" 134713001 134714000 "*" 0 0 -100
"chr11" 134714001 134715000 "*" 0 0 100
"chr11" 134724001 134725000 "*" 3.33066907387547e-16 1.4495649018245e-15 100
"chr11" 134725001 134726000 "*" 0 0 -98.0392156862745
"chr11" 134784001 134785000 "*" 1.48769885299771e-14 5.41245512218755e-14 -60
"chr11" 134806001 134807000 "*" 1.98931358053045e-08 3.19617290079798e-08
73.3333333333333
"chr11" 134807001 134808000 "*" 5.1281201507436e-13 1.54461625452838e-12 100
"chr11" 134828001 134829000 "*" 1.65722990885797e-12 4.6888804640154e-12
-60.8974358974359
"chr11" 134841001 134842000 "*" 1.4432899320127e-15 5.90750815956055e-15 -100
"chr11" 134852001 134853000 "*" 1.09146802707016e-10 2.42369652744533e-10
-88.3720930232558
"chr11" 134857001 134858000 "*" 3.6700841921089e-08 5.65530226072258e-08 -100
"chr11" 134863001 134864000 "*" 2.30599364963169e-05 2.39042293936956e-05
54.8387096774194
"chr11_gl000202_random" 22001 23000 "*" 0 0 100
"chr12" 160001 161000 "*" 2.44805401505843e-08 3.87506495191586e-08
-66.6666666666667
"chr12" 211001 212000 "*" 2.52731169325671e-12 6.98871209142155e-12
62.962962962963
"chr12" 212001 213000 "*" 2.455369241261e-12 6.8003688121118e-12 100
"chr12" 232001 233000 "*" 5.6621374255883e-14 1.91985285860987e-13
-96.1538461538462
"chr12" 252001 253000 "*" 0.000226645707655515 0.000200573098869507
-53.7037037037037
"chr12" 270001 271000 "*" 2.00227675550835e-08 3.20459176617961e-08 100
"chr12" 273001 274000 "*" 6.66133814775094e-16 2.81595744474255e-15 -100
"chr12" 283001 284000 "*" 2.53672860495158e-10 5.35566751071889e-10
-65.2459016393443
"chr12" 285001 286000 "*" 7.88258347483861e-15 2.96145301916012e-14
-87.5813241484883
"chr12" 302001 303000 "*" 1.17461596005342e-13 3.8279067575877e-13
80.4878048780488
"chr12" 316001 317000 "*" 2.87547763377916e-14 1.01086173823114e-13
53.0484952440355
"chr12" 445001 446000 "*" 7.0006535457523e-08 1.03695832512054e-07 -100
"chr12" 473001 474000 "*" 1.89352503676687e-07 2.64641376365253e-07
-53.8461538461538
"chr12" 502001 503000 "*" 1.00808250635964e-13 3.31107370956809e-13 100
"chr12" 667001 668000 "*" 0 0 -73.6111111111111
"chr12" 680001 681000 "*" 0 0 -91.304347826087
"chr12" 692001 693000 "*" 0 0 -98.8888888888889
"chr12" 719001 720000 "*" 5.6362137179633e-11 1.29963270300065e-10 100
"chr12" 738001 739000 "*" 6.16959703788611e-05 5.98208095078624e-05 70

"chr12" 752001 753000 "*" 0 0 -68.2953278605453
"chr12" 771001 772000 "*" 1.52538993880569e-07 2.15558954974681e-07
-63.0434782608696
"chr12" 1057001 1058000 "*" 1.11022302462516e-16 5.03662826618488e-16 100
"chr12" 1610001 1611000 "*" 9.80723946497619e-10 1.8980032424715e-09 65.625
"chr12" 1643001 1644000 "*" 4.2232883856741e-13 1.2837130799229e-12
-64.8648648648649
"chr12" 1719001 1720000 "*" 5.55111512312578e-16 2.36485094870365e-15 -100
"chr12" 1771001 1772000 "*" 0 0 -68.4563758389262
"chr12" 1978001 1979000 "*" 2.15125472990962e-10 4.58126659943874e-10 -100
"chr12" 1999001 2000000 "*" 2.00227675550835e-08 3.20459176617961e-08 -100
"chr12" 2016001 2017000 "*" 0 0 69.7368421052632
"chr12" 2038001 2039000 "*" 0 0 59.0909090909091
"chr12" 2134001 2135000 "*" 2.3574878160737e-08 3.73979775094372e-08
-84.6153846153846
"chr12" 2161001 2162000 "*" 0 0 67.3011318348478
"chr12" 2162001 2163000 "*" 0 0 54.625452104851
"chr12" 2163001 2164000 "*" 0 0 55.7847575310262
"chr12" 2206001 2207000 "*" 1.13140401492018e-08 1.87801380787728e-08 -100
"chr12" 2219001 2220000 "*" 2.00227675550835e-08 3.20459176617961e-08 100
"chr12" 2225001 2226000 "*" 1.22124532708767e-15 5.03921984217778e-15 -100
"chr12" 2317001 2318000 "*" 2.69018141096922e-12 7.41487347763769e-12 -100
"chr12" 2377001 2378000 "*" 2.69007038866675e-13 8.38831022628993e-13 -100
"chr12" 2410001 2411000 "*" 1.33379262079458e-08 2.19593016142501e-08
-54.0983606557377
"chr12" 2413001 2414000 "*" 5.56820467512154e-09 9.69503446294489e-09 -60
"chr12" 2420001 2421000 "*" 8.00762784913811e-05 7.62332949242677e-05
-57.6923076923077
"chr12" 2431001 2432000 "*" 2.0874876538457e-07 2.90229945171996e-07
-68.5714285714286
"chr12" 2481001 2482000 "*" 7.0006535457523e-08 1.03695832512054e-07 100
"chr12" 2486001 2487000 "*" 2.84200885047881e-10 5.96020717471281e-10
66.6666666666667
"chr12" 2518001 2519000 "*" 0 0 100
"chr12" 2521001 2522000 "*" 2.1094237467878e-15 8.47434540879246e-15 -100
"chr12" 2563001 2564000 "*" 0 0 -69.7079236552921
"chr12" 2576001 2577000 "*" 0 0 -90.3225806451613
"chr12" 2609001 2610000 "*" 0 0 -100
"chr12" 2628001 2629000 "*" 0 0 100
"chr12" 2664001 2665000 "*" 0 0 -77.2727272727273
"chr12" 2670001 2671000 "*" 4.44089209850063e-16 1.91071758245033e-15
-88.5245901639344
"chr12" 2694001 2695000 "*" 4.43136073150496e-05 4.39299853239263e-05
65.5172413793103
"chr12" 2697001 2698000 "*" 4.13089673578781e-10 8.46277737348438e-10
-65.5172413793103
"chr12" 2730001 2731000 "*" 5.72471036908162e-07 7.51633057935261e-07 -78
"chr12" 2746001 2747000 "*" 1.38590361409285e-10 3.02706178892116e-10 100
"chr12" 2762001 2763000 "*" 0 0 -51.8496225923998
"chr12" 2783001 2784000 "*" 0 0 59.7222222222222
"chr12" 2789001 2790000 "*" 2.6445412526499e-10 5.57143970586599e-10
-68.9320388349515

"chr12" 2798001 2799000 "*" 2.79987144580218e-12 7.68634726811898e-12 100
"chr12" 2859001 2860000 "*" 4.79272978137768e-06 5.5096142490499e-06
-51.7241379310345
"chr12" 2871001 2872000 "*" 1.93720595120794e-11 4.76309924836636e-11 100
"chr12" 2879001 2880000 "*" 8.45376425440136e-06 9.36283854341238e-06
67.5675675675676
"chr12" 2886001 2887000 "*" 0 0 79.6875
"chr12" 2892001 2893000 "*" 2.73625566649116e-12 7.52483363579137e-12 100
"chr12" 2930001 2931000 "*" 9.62896429257398e-13 2.80291205576417e-12 -100
"chr12" 2962001 2963000 "*" 0 0 -100
"chr12" 3041001 3042000 "*" 5.48638023900594e-10 1.10374357336421e-09 -100
"chr12" 3045001 3046000 "*" 5.82384140912495e-11 1.34089510134998e-10
90.5701754385965
"chr12" 3053001 3054000 "*" 5.55111512312578e-16 2.36485094870365e-15
-55.0548589341693
"chr12" 3096001 3097000 "*" 3.65130148338721e-12 9.90602415539447e-12 -100
"chr12" 3178001 3179000 "*" 3.67513797172592e-11 8.67756832290752e-11
90.9090909090909
"chr12" 3185001 3186000 "*" 1.11022302462516e-16 5.03662826618488e-16
89.5238095238095
"chr12" 3222001 3223000 "*" 9.67004254448511e-14 3.18178990782609e-13 -100
"chr12" 3300001 3301000 "*" 4.36618494992214e-06 5.04443830498714e-06 60
"chr12" 3309001 3310000 "*" 0 0 79.0802361737065
"chr12" 3318001 3319000 "*" 0 0 56.7164179104478
"chr12" 3327001 3328000 "*" 2.00227675550835e-08 3.20459176617961e-08 100
"chr12" 3378001 3379000 "*" 0 0 74.3589743589744
"chr12" 3388001 3389000 "*" 1.63746793901964e-12 4.63391019577792e-12
-97.9166666666667
"chr12" 3400001 3401000 "*" 7.82712499494131e-05 7.46478152805681e-05
56.5217391304348
"chr12" 3407001 3408000 "*" 7.72616432165307e-07 9.96131354566251e-07
70.1754385964912
"chr12" 3423001 3424000 "*" 5.00779943068963e-08 7.59467500961879e-08
-73.4177215189873
"chr12" 3425001 3426000 "*" 8.61307206667661e-08 1.26041587136976e-07 90
"chr12" 3432001 3433000 "*" 4.75814901257365e-07 6.30674396767931e-07
-70.2702702702703
"chr12" 3440001 3441000 "*" 4.35069721360648e-08 6.65086386566157e-08
-50.2857142857143
"chr12" 3446001 3447000 "*" 3.52704532247117e-11 8.35581292290093e-11 100
"chr12" 3475001 3476000 "*" 0 0 57.9407843065627
"chr12" 3547001 3548000 "*" 7.50599582488576e-12 1.94998997989941e-11 65.625
"chr12" 3579001 3580000 "*" 6.39957897785592e-09 1.10592268512008e-08
66.6666666666667
"chr12" 3582001 3583000 "*" 7.7715611723761e-16 3.26213507634405e-15 -100
"chr12" 3625001 3626000 "*" 4.02167188440217e-12 1.08266700497926e-11 -100
"chr12" 3649001 3650000 "*" 5.88729065498228e-12 1.5519443197696e-11
-55.8752997601918
"chr12" 3653001 3654000 "*" 1.77635683940025e-15 7.19870740878856e-15 100
"chr12" 3663001 3664000 "*" 0.000191016562678725 0.000171120574224663
53.968253968254
"chr12" 3665001 3666000 "*" 1.39779633423487e-07 1.98463037958589e-07 -100

"chr12" 3693001 3694000 "*" 0 0 -100
"chr12" 3711001 3712000 "*" 4.99020824662466e-11 1.15790792586157e-10 -100
"chr12" 3748001 3749000 "*" 3.68371999570627e-13 1.13149976091305e-12 100
"chr12" 3779001 3780000 "*" 2.91028312560115e-11 6.97590507139978e-11 100
"chr12" 3787001 3788000 "*" 1.11022302462516e-15 4.59817122935606e-15 100
"chr12" 3857001 3858000 "*" 1.96253939310775e-06 2.39070396570886e-06
78.5714285714286
"chr12" 3861001 3862000 "*" 3.61932706027801e-14 1.25777907157994e-13
-57.3770491803279
"chr12" 3982001 3983000 "*" 0 0 100
"chr12" 4000001 4001000 "*" 4.48240397021848e-08 6.84329675653433e-08
-52.1739130434783
"chr12" 4140001 4141000 "*" 0 0 89.8412698412698
"chr12" 4148001 4149000 "*" 2.15125472990962e-10 4.58126659943874e-10 -100
"chr12" 4215001 4216000 "*" 2.64951482975562e-09 4.82186011375752e-09 100
"chr12" 4245001 4246000 "*" 0 0 98
"chr12" 4252001 4253000 "*" 3.78051111527e-08 5.81707800293723e-08
-79.8076923076923
"chr12" 4273001 4274000 "*" 0 0 74.0686702201758
"chr12" 4303001 4304000 "*" 3.44169137633799e-15 1.3517917469209e-14
95.4545454545455
"chr12" 4349001 4350000 "*" 0.000299166032473375 0.000259267996470385
-54.1666666666667
"chr12" 4378001 4379000 "*" 0 0 88.8276947285602
"chr12" 4406001 4407000 "*" 3.68589603283453e-11 8.70173799349621e-11
-54.8888888888889
"chr12" 4416001 4417000 "*" 1.17794662912729e-13 3.83653245040640e-13 100
"chr12" 4500001 4501000 "*" 0 0 100
"chr12" 4501001 4502000 "*" 3.24866192502604e-07 4.40374918758373e-07
-51.5873015873016
"chr12" 4526001 4527000 "*" 1.29037891483108e-11 3.24582280286866e-11 -100
"chr12" 4533001 4534000 "*" 8.67505319801509e-08 1.26895177832038e-07
52.1739130434783
"chr12" 4545001 4546000 "*" 6.15840711759574e-13 1.83676803572802e-12 -100
"chr12" 4705001 4706000 "*" 3.40125705378114e-10 7.03784073980162e-10 100
"chr12" 4714001 4715000 "*" 0 0 -86.9565217391304
"chr12" 4814001 4815000 "*" 6.66133814775094e-16 2.81595744474255e-15 100
"chr12" 4880001 4881000 "*" 5.08737141302618e-09 8.89609430096227e-09 62.5
"chr12" 4939001 4940000 "*" 5.56277031105168e-08 8.38843041611454e-08
52.3404255319149
"chr12" 4990001 4991000 "*" 1.11022302462516e-15 4.59817122935606e-15 -100
"chr12" 5097001 5098000 "*" 9.63829016598083e-12 2.46993429847588e-11 -100
"chr12" 5129001 5130000 "*" 9.65338919911574e-13 2.80678910656113e-12 100
"chr12" 5169001 5170000 "*" 6.75015598972095e-12 1.76534247161148e-11
-83.5820895522388
"chr12" 5320001 5321000 "*" 2.46613396370776e-10 5.21216622472487e-10
-64.406779661017
"chr12" 5342001 5343000 "*" 5.91639255365095e-08 8.89001265712045e-08
76.4705882352941
"chr12" 5414001 5415000 "*" 1.11022302462516e-16 5.03662826618488e-16
-69.1176470588235
"chr12" 5541001 5542000 "*" 0 0 85.0948509485095

"chr12" 5545001 5546000 "*" 0 0 100
"chr12" 5631001 5632000 "*" 0 0 -51.5151515151515
"chr12" 5632001 5633000 "*" 4.8168136146387e-12 1.28494630448437e-11 -93.75
"chr12" 5699001 5700000 "*" 1.9829472005517e-06 2.41330824932622e-06 -68.75
"chr12" 5769001 5770000 "*" 0 0 -90.6976744186046
"chr12" 5852001 5853000 "*" 0 0 -100
"chr12" 5936001 5937000 "*" 0 0 -52.9824561403509
"chr12" 5970001 5971000 "*" 1.4432899320127e-15 5.90750815956055e-15 100
"chr12" 5978001 5979000 "*" 2.22044604925031e-16 9.81641919380259e-16 100
"chr12" 6031001 6032000 "*" 1.53909440747668e-10 3.34135636937e-10
-67.2222222222222
"chr12" 6054001 6055000 "*" 0 0 63.0903508469314
"chr12" 6079001 6080000 "*" 1.23966884502114e-05 1.33880538279549e-05
-73.3333333333333
"chr12" 6094001 6095000 "*" 2.61495269882062e-11 6.326551335125e-11
-89.6551724137931
"chr12" 6151001 6152000 "*" 1.77635683940025e-15 7.19870740878856e-15
-62.790070156503
"chr12" 6168001 6169000 "*" 2.08995487582797e-10 4.46944006972663e-10 -100
"chr12" 6183001 6184000 "*" 1.16573417585641e-14 4.29335582900508e-14 100
"chr12" 6232001 6233000 "*" 1.36247679805024e-11 3.41697532889583e-11
-84.0579710144928
"chr12" 6269001 6270000 "*" 2.16098569039147e-08 3.44432428850266e-08
61.7647058823529
"chr12" 6273001 6274000 "*" 2.54927622046486e-05 2.62418431936613e-05
67.7777777777778
"chr12" 6299001 6300000 "*" 0 0 56.7164179104478
"chr12" 6310001 6311000 "*" 0.000449815378086083 0.000378571446274996
-63.6363636363636
"chr12" 6334001 6335000 "*" 2.37032615757471e-13 7.44555209171911e-13
63.4146341463415
"chr12" 6373001 6374000 "*" 9.99200722162641e-16 4.15382497462808e-15
73.0769230769231
"chr12" 6377001 6378000 "*" 4.14335232790108e-13 1.26153063645578e-12 100
"chr12" 6399001 6400000 "*" 3.99846228349432e-08 6.13647647811993e-08 90
"chr12" 6435001 6436000 "*" 2.22044604925031e-16 9.81641919380259e-16
-56.8085922516302
"chr12" 6457001 6458000 "*" 6.81010803305071e-13 2.01951271652453e-12
69.3452380952381
"chr12" 6463001 6464000 "*" 1.35036426485158e-12 3.86629547363774e-12 100
"chr12" 6473001 6474000 "*" 0 0 56.9060773480663
"chr12" 6494001 6495000 "*" 0 0 -84.7094801223242
"chr12" 6503001 6504000 "*" 3.6427075800205e-09 6.5252880340617e-09
66.6666666666667
"chr12" 6641001 6642000 "*" 1.92946192356658e-09 3.59193011611483e-09 100
"chr12" 6651001 6652000 "*" 4.42354207408346e-06 5.10752148706029e-06 68
"chr12" 6794001 6795000 "*" 1.12634901405784e-10 2.49563754426169e-10 -100
"chr12" 6804001 6805000 "*" 9.12428294176371e-05 8.61061372448753e-05
-53.3333333333333
"chr12" 6810001 6811000 "*" 0 0 57.4252072569987
"chr12" 6829001 6830000 "*" 7.59327470246651e-05 7.25486479096441e-05 60
"chr12" 6854001 6855000 "*" 0 0 -100

"chr12" 6900001 6901000 "*" 2.08814465718632e-09 3.86232567960463e-09 100
"chr12" 6918001 6919000 "*" 2.00227675550835e-08 3.20459176617961e-08 100
"chr12" 6937001 6938000 "*" 0 0 89.9501661129568
"chr12" 6955001 6956000 "*" 8.09581290894812e-11 1.82866083238778e-10
-88.8888888888889
"chr12" 7014001 7015000 "*" 0 0 -100
"chr12" 7028001 7029000 "*" 0 0 -100
"chr12" 7055001 7056000 "*" 0 0 50.9419190683564
"chr12" 7058001 7059000 "*" 2.05946371067967e-13 6.51843297360463e-13
-65.1041666666667
"chr12" 7071001 7072000 "*" 0 0 -73.323754789272
"chr12" 7073001 7074000 "*" 1.31583632878574e-12 3.77625225968747e-12
-64.2857142857143
"chr12" 7074001 7075000 "*" 5.06208408523889e-11 1.17376929723124e-10
-83.1858407079646
"chr12" 7077001 7078000 "*" 1.90181204118289e-13 6.05298617539364e-13 -100
"chr12" 7169001 7170000 "*" 2.79440914852103e-11 6.73046092948135e-11 -100
"chr12" 7260001 7261000 "*" 0 0 -100
"chr12" 7746001 7747000 "*" 3.7274627828765e-12 1.00985250136599e-11 100
"chr12" 7853001 7854000 "*" 3.1276351653009e-07 4.2499136916602e-07 90
"chr12" 7902001 7903000 "*" 1.52466927971773e-12 4.33322475981408e-12 100
"chr12" 7933001 7934000 "*" 1.00069952324588e-10 2.23170408578095e-10
55.1020408163265
"chr12" 7980001 7981000 "*" 7.54951656745106e-14 2.5237892556906e-13 -80
"chr12" 8023001 8024000 "*" 1.74853465040314e-11 4.32752710900609e-11 -100
"chr12" 8036001 8037000 "*" 1.67299207820548e-08 2.71377429441055e-08 100
"chr12" 8117001 8118000 "*" 3.05172401682263e-07 4.15176924395495e-07
-53.3333333333333
"chr12" 8123001 8124000 "*" 0 0 77.6073619631902
"chr12" 8124001 8125000 "*" 0 0 100
"chr12" 8167001 8168000 "*" 0 0 -97.6190476190476
"chr12" 8214001 8215000 "*" 3.67990513883143e-06 4.30329479768314e-06
61.3636363636364
"chr12" 8333001 8334000 "*" 0 0 81.7307692307692
"chr12" 8384001 8385000 "*" 0 0 -100
"chr12" 8542001 8543000 "*" 0 0 57.5213675213675
"chr12" 8832001 8833000 "*" 1.88737914186277e-15 7.62011598380321e-15 -100
"chr12" 8833001 8834000 "*" 2.08995487582797e-10 4.46944006972663e-10 -100
"chr12" 8850001 8851000 "*" 0 0 95.4531583765639
"chr12" 8851001 8852000 "*" 0 0 73.0981608799178
"chr12" 8936001 8937000 "*" 6.84617402358922e-05 6.5862557691964e-05
54.5454545454545
"chr12" 8971001 8972000 "*" 2.16015490900334e-05 2.24925309279237e-05
-63.8888888888889
"chr12" 9246001 9247000 "*" 0 0 -81.0810810810811
"chr12" 9795001 9796000 "*" 0 0 -95.6521739130435
"chr12" 9800001 9801000 "*" 0 0 -85.7086167800453
"chr12" 9869001 9870000 "*" 2.15125472990962e-10 4.58126659943874e-10 100
"chr12" 10517001 10518000 "*" 1.13140401492018e-08 1.87801380787728e-08 -100
"chr12" 11386001 11387000 "*" 1.92946192356658e-09 3.59193011611483e-09 100
"chr12" 11497001 11498000 "*" 1.29037891483108e-11 3.24582280286866e-11 100
"chr12" 11767001 11768000 "*" 0 0 -100

"chr12" 11827001 11828000 "*" 8.25538581938901e-08 1.2109413635802e-07 -60.6060606060606
"chr12" 11872001 11873000 "*" 8.75292891500123e-08 1.27973699145787e-07 -51.25
"chr12" 12102001 12103000 "*" 1.9829472005517e-06 2.41330824932622e-06 68.75
"chr12" 12159001 12160000 "*" 0.00014439803701094 0.000131924792739962 -51.1627906976744
"chr12" 12164001 12165000 "*" 1.48087875295744e-10 3.22075573380618e-10 -100
"chr12" 12503001 12504000 "*" 0 0 93.2294243773709
"chr12" 12939001 12940000 "*" 0 0 65.3918361541744
"chr12" 13044001 13045000 "*" 0 0 -93.613707165109
"chr12" 13068001 13069000 "*" 1.72931349240457e-08 2.80012329480734e-08 -77.4193548387097
"chr12" 13197001 13198000 "*" 0 0 86.0321384425216
"chr12" 13275001 13276000 "*" 4.02167188440217e-12 1.08266700497926e-11 -100
"chr12" 13288001 13289000 "*" 0 0 -75
"chr12" 13295001 13296000 "*" 4.49238402033814e-09 7.92830603617938e-09 -85.7142857142857
"chr12" 13350001 13351000 "*" 1.44519793021658e-07 2.04841726501585e-07 -72.0930232558139
"chr12" 13557001 13558000 "*" 6.00276495177354e-10 1.20226406840177e-09 60
"chr12" 13603001 13604000 "*" 3.6700841921089e-08 5.65530226072258e-08 -100
"chr12" 13625001 13626000 "*" 1.13140401492018e-08 1.87801380787728e-08 100
"chr12" 13736001 13737000 "*" 0 0 65.3846153846154
"chr12" 14033001 14034000 "*" 4.73234496034536e-09 8.30627300264826e-09 100
"chr12" 14109001 14110000 "*" 0 0 -92.7710843373494
"chr12" 14474001 14475000 "*" 0 0 86.3636363636364
"chr12" 14668001 14669000 "*" 5.44009282066327e-15 2.08486137681812e-14 100
"chr12" 14849001 14850000 "*" 1.39779633423487e-07 1.98463037958589e-07 100
"chr12" 15091001 15092000 "*" 2.62900812231237e-13 8.21294938531841e-13 100
"chr12" 17697001 17698000 "*" 8.01666433236647e-09 1.36392368970109e-08 100
"chr12" 18281001 18282000 "*" 2.22044604925031e-16 9.81641919380259e-16 -100
"chr12" 18788001 18789000 "*" 3.47557093960393e-06 4.07806861000566e-06 54.5454545454545
"chr12" 18906001 18907000 "*" 1.11022302462516e-16 5.03662826618488e-16 56
"chr12" 19049001 19050000 "*" 0.000923822137060548 0.000737225242626394 60
"chr12" 19558001 19559000 "*" 1.20591092667155e-10 2.66075324129686e-10 100
"chr12" 19794001 19795000 "*" 9.61897228535236e-13 2.801372325071e-12 -100
"chr12" 19814001 19815000 "*" 1.29037891483108e-11 3.24582280286866e-11 100
"chr12" 19854001 19855000 "*" 7.04457048250617e-10 1.39315702524552e-09 67.5675675675676
"chr12" 19879001 19880000 "*" 1.03886985030055e-05 1.13522277663458e-05 -70.5882352941177
"chr12" 19937001 19938000 "*" 7.40308501394615e-05 7.08426979701479e-05 -51.0204081632653
"chr12" 20482001 20483000 "*" 3.6700841921089e-08 5.65530226072258e-08 -100
"chr12" 20521001 20522000 "*" 0 0 77.8999094788568
"chr12" 20820001 20821000 "*" 1.07634446333549e-06 1.36049041286493e-06 63.6363636363636
"chr12" 20984001 20985000 "*" 6.4152538836737e-10 1.27477697728417e-09 100
"chr12" 21445001 21446000 "*" 6.11364625591193e-10 1.22332619666323e-09 -96.6666666666667
"chr12" 21866001 21867000 "*" 6.4324989779152e-11 1.47230020825092e-10 100

"chr12" 22409001 22410000 "*" 2.22044604925031e-16 9.81641919380259e-16 -100
"chr12" 22441001 22442000 "*" 5.778251227051e-06 6.56428173920586e-06
59.7014925373134
"chr12" 22488001 22489000 "*" 0 0 52.576164016095
"chr12" 22777001 22778000 "*" 4.73234496034536e-09 8.30627300264826e-09 100
"chr12" 22866001 22867000 "*" 2.08814465718632e-09 3.86232567960463e-09 100
"chr12" 22932001 22933000 "*" 3.34128893442198e-08 5.19772864618523e-08 -100
"chr12" 23977001 23978000 "*" 0.000235599134865705 0.000207894354164181
55.5555555555556
"chr12" 24549001 24550000 "*" 1.3988810110277e-14 5.10290212559087e-14
71.4285714285714
"chr12" 24716001 24717000 "*" 0 0 87.1728033018356
"chr12" 25498001 25499000 "*" 6.59550958292954e-09 1.13456194382773e-08 100
"chr12" 25509001 25510000 "*" 1.07882591748876e-11 2.74553537455803e-11 -100
"chr12" 25576001 25577000 "*" 9.65729496371637e-10 1.87255912493232e-09 100
"chr12" 25818001 25819000 "*" 0 0 100
"chr12" 26007001 26008000 "*" 0 0 85
"chr12" 26111001 26112000 "*" 0 0 65.3908794788274
"chr12" 26112001 26113000 "*" 0 0 70.3174603174603
"chr12" 26392001 26393000 "*" 3.56775498033812e-10 7.35898629809829e-10 100
"chr12" 26547001 26548000 "*" 1.12798659301916e-13 3.68863190206523e-13
92.3076923076923
"chr12" 27176001 27177000 "*" 9.80763359414993e-10 1.8980032424715e-09 -100
"chr12" 27255001 27256000 "*" 3.46500605985511e-13 1.06777978272295e-12 100
"chr12" 27483001 27484000 "*" 0 0 100
"chr12" 27852001 27853000 "*" 6.24654217240561e-10 1.2462108191303e-09 -100
"chr12" 28122001 28123000 "*" 0 0 60.0242130750605
"chr12" 28127001 28128000 "*" 0 0 -62.1412109784203
"chr12" 28128001 28129000 "*" 0 0 97.3377703826955
"chr12" 28340001 28341000 "*" 6.77335965093562e-12 1.76897011246462e-11 100
"chr12" 29683001 29684000 "*" 4.01313771103418e-09 7.11882087237587e-09 100
"chr12" 29943001 29944000 "*" 0 0 100
"chr12" 30169001 30170000 "*" 4.33877644923086e-08 6.63361344592129e-08
-86.9565217391304
"chr12" 30247001 30248000 "*" 0.000447727107318552 0.000376920426399982 -60
"chr12" 30757001 30758000 "*" 0 0 100
"chr12" 30969001 30970000 "*" 1.12634901405784e-10 2.49563754426169e-10 100
"chr12" 30977001 30978000 "*" 2.80374033989617e-08 4.40846684644989e-08
54.3650793650794
"chr12" 31106001 31107000 "*" 3.61871754783749e-10 7.45986023744022e-10
-78.9473684210526
"chr12" 31123001 31124000 "*" 1.19904086659517e-14 4.40901388691796e-14 -100
"chr12" 31125001 31126000 "*" 4.44089209850063e-15 1.71914916534614e-14 100
"chr12" 31142001 31143000 "*" 4.27032853522746e-11 9.99995440481693e-11
-70.5882352941177
"chr12" 31270001 31271000 "*" 0 0 86.6028708133971
"chr12" 31276001 31277000 "*" 1.48087875295744e-10 3.22075573380618e-10 100
"chr12" 31373001 31374000 "*" 1.15463194561016e-14 4.25829614430727e-14 -100
"chr12" 31387001 31388000 "*" 9.65729496371637e-10 1.87255912493232e-09 -100
"chr12" 31390001 31391000 "*" 5.09085811284393e-08 7.71124436587394e-08
-53.3333333333333
"chr12" 31882001 31883000 "*" 0 0 69.3693693693694

"chr12" 31947001 31948000 "*" 0 0 59.4202898550725
"chr12" 32080001 32081000 "*" 1.22457599616155e-12 3.52441478267078e-12 100
"chr12" 32658001 32659000 "*" 9.08108033215171e-11 2.03407753381119e-10 -100
"chr12" 32701001 32702000 "*" 1.22457599616155e-12 3.52441478267078e-12 100
"chr12" 32810001 32811000 "*" 7.67056418382595e-11 1.7359448580318e-10 -100
"chr12" 32995001 32996000 "*" 6.92287338566189e-11 1.57605630124247e-10 -100
"chr12" 33097001 33098000 "*" 2.00227675550835e-08 3.20459176617961e-08 -100
"chr12" 33419001 33420000 "*" 0 0 100
"chr12" 33593001 33594000 "*" 0 0 50.7692307692308
"chr12" 34277001 34278000 "*" 3.59925459658683e-07 4.84897008249163e-07
71.4285714285714
"chr12" 34317001 34318000 "*" 3.90831811358794e-12 1.05610567215048e-11 100
"chr12" 34369001 34370000 "*" 3.63445940010365e-11 8.58565209111204e-11 100
"chr12" 34440001 34441000 "*" 1.56735245870898e-06 1.93725608775872e-06
70.4918032786885
"chr12" 34441001 34442000 "*" 1.01779795949053e-05 1.11391868137953e-05
56.7901234567901
"chr12" 34443001 34444000 "*" 0 0 98.3333333333333
"chr12" 34463001 34464000 "*" 6.74676726708512e-08 1.00590849220071e-07
-74.7252747252747
"chr12" 34464001 34465000 "*" 6.62803145701218e-14 2.22896146391892e-13
50.8690440515433
"chr12" 34467001 34468000 "*" 5.80646641878957e-14 1.96527473015782e-13
56.5217391304348
"chr12" 34472001 34473000 "*" 7.7715611723761e-16 3.26213507634405e-15
70.5882352941177
"chr12" 34479001 34480000 "*" 4.44089209850063e-16 1.91071758245033e-15
68.6648983200707
"chr12" 34482001 34483000 "*" 6.0285110237146e-14 2.03679165917479e-13
64.4384546271339
"chr12" 34513001 34514000 "*" 1.14130926931466e-13 3.7276674571693e-13 100
"chr12" 34522001 34523000 "*" 5.23868415314155e-09 9.14898812888254e-09
86.8421052631579
"chr12" 34527001 34528000 "*" 0 0 74.2465753424657
"chr12" 34529001 34530000 "*" 0 0 51.6816516816517
"chr12" 34530001 34531000 "*" 1.49831795859257e-08 2.45146078449471e-08
72.5563909774436
"chr12" 34533001 34534000 "*" 0 0 76.4705882352941
"chr12" 34534001 34535000 "*" 0 0 65
"chr12" 34551001 34552000 "*" 1.08092630179968e-07 1.56163398945001e-07
54.0300546448087
"chr12" 34557001 34558000 "*" 1.88737914186277e-15 7.62011598380321e-15 -100
"chr12" 34559001 34560000 "*" 6.22970933373823e-05 6.03552460796889e-05
-55.5555555555556
"chr12" 34752001 34753000 "*" 5.01025332333427e-10 1.01358031135889e-09 100
"chr12" 34754001 34755000 "*" 1.01996189272313e-12 2.96034566680495e-12
-50.3597122302158
"chr12" 34755001 34756000 "*" 9.65338919911574e-13 2.80678910656113e-12 100
"chr12" 38445001 38446000 "*" 4.21107593240322e-13 1.28014094315562e-12
87.962962962963
"chr12" 38447001 38448000 "*" 2.22044604925031e-16 9.81641919380259e-16
-54.2852456327401

"chr12" 38482001 38483000 "*" 5.01025332333427e-10 1.01358031135889e-09 -100
"chr12" 39299001 39300000 "*" 0 0 51.8536585365854
"chr12" 40013001 40014000 "*" 2.62900812231237e-13 8.21294938531841e-13 100
"chr12" 40499001 40500000 "*" 0 0 54.3018335684062
"chr12" 42537001 42538000 "*" 2.64951482975562e-09 4.82186011375752e-09 -100
"chr12" 42570001 42571000 "*" 2.44249065417534e-15 9.75180348030994e-15 -100
"chr12" 42666001 42667000 "*" 3.95353005888666e-09 7.03928529283733e-09 -100
"chr12" 42720001 42721000 "*" 0 0 100
"chr12" 42983001 42984000 "*" 0 0 87.8623094807543
"chr12" 43068001 43069000 "*" 2.68450373042128e-10 5.64465213127486e-10 -100
"chr12" 43900001 43901000 "*" 1.19160237233018e-11 3.01317289318662e-11
66.6666666666667
"chr12" 45116001 45117000 "*" 1.29738864318441e-07 1.85457145691328e-07 90
"chr12" 45609001 45610000 "*" 0 0 81.3842482100239
"chr12" 45863001 45864000 "*" 3.20423422239813e-08 5.00515746557833e-08
-82.4175824175824
"chr12" 45924001 45925000 "*" 6.15840711759574e-13 1.83676803572802e-12 -100
"chr12" 46496001 46497000 "*" 3.40502548379362e-09 6.12227009912616e-09
78.5714285714286
"chr12" 47219001 47220000 "*" 0 0 85.0746268656716
"chr12" 47372001 47373000 "*" 1.13140401492018e-08 1.87801380787728e-08 100
"chr12" 47472001 47473000 "*" 3.07317337100432e-06 3.63687035006489e-06 -60
"chr12" 47473001 47474000 "*" 0 0 51.0011123470523
"chr12" 47820001 47821000 "*" 1.84540160930169e-11 4.55658526230159e-11
-56.1151079136691
"chr12" 47932001 47933000 "*" 2.00227675550835e-08 3.20459176617961e-08 100
"chr12" 47937001 47938000 "*" 1.94826821342531e-10 4.18791532986873e-10
-61.1111111111111
"chr12" 47940001 47941000 "*" 1.51958176486389e-07 2.14810580080327e-07
75.4716981132076
"chr12" 48111001 48112000 "*" 1.66533453693773e-15 6.7629186866784e-15
-78.4615384615385
"chr12" 48140001 48141000 "*" 2.22044604925031e-16 9.81641919380259e-16
98.7341772151899
"chr12" 48145001 48146000 "*" 0 0 -78.2608695652174
"chr12" 48152001 48153000 "*" 3.5527136788005e-15 1.39277426410519e-14
-51.4285714285714
"chr12" 48153001 48154000 "*" 2.37017283577501e-09 4.35520548255659e-09
-84.3137254901961
"chr12" 48214001 48215000 "*" 1.10141499254368e-08 1.83702859833246e-08
-62.1951219512195
"chr12" 48351001 48352000 "*" 2.08814465718632e-09 3.86232567960463e-09 -100
"chr12" 48374001 48375000 "*" 0 0 73.5923423423423
"chr12" 48396001 48397000 "*" 0 0 80.8807710511596
"chr12" 48411001 48412000 "*" 0 0 81.5384615384615
"chr12" 48419001 48420000 "*" 1.73394842928332e-09 3.24782166625656e-09
54.3984962406015
"chr12" 48433001 48434000 "*" 3.6700841921089e-08 5.65530226072258e-08 100
"chr12" 48508001 48509000 "*" 9.660920470278e-07 1.22837421766042e-06
-61.5384615384615
"chr12" 48719001 48720000 "*" 1.23234755733392e-14 4.52749417164674e-14 -78
"chr12" 48780001 48781000 "*" 8.7349629751543e-09 1.47505871898511e-08 -100

"chr12" 48894001 48895000 "*" 1.54321000422897e-14 5.6024372055589e-14 -84.7826086956522
"chr12" 49018001 49019000 "*" 5.01111496662432e-05 4.92883234243094e-05 61.7021276595745
"chr12" 49108001 49109000 "*" 3.33066907387547e-16 1.4495649018245e-15 100
"chr12" 49159001 49160000 "*" 1.93720595120794e-11 4.76309924836636e-11 -100
"chr12" 49257001 49258000 "*" 6.66133814775094e-16 2.81595744474255e-15 -98.3870967741936
"chr12" 49259001 49260000 "*" 1.78845827036866e-12 5.04152863685409e-12 -52.4390243902439
"chr12" 49275001 49276000 "*" 2.02327044007689e-12 5.65300384792621e-12 100
"chr12" 49280001 49281000 "*" 2.08814465718632e-09 3.86232567960463e-09 -100
"chr12" 49294001 49295000 "*" 2.18713935851156e-14 7.80893646430581e-14 -100
"chr12" 49318001 49319000 "*" 1.01529910478959e-07 1.47209980654302e-07 72
"chr12" 49365001 49366000 "*" 1.1230558272235e-05 1.22105725326657e-05 60.233918128655
"chr12" 49379001 49380000 "*" 5.08482145278322e-14 1.73432706170696e-13 -100
"chr12" 49439001 49440000 "*" 8.07468134134126e-07 1.03833672799554e-06 -52.1739130434783
"chr12" 49483001 49484000 "*" 0 0 59.485449695404
"chr12" 49503001 49504000 "*" 1.11022302462516e-16 5.03662826618488e-16 100
"chr12" 49518001 49519000 "*" 0 0 -90
"chr12" 49606001 49607000 "*" 7.67056418382595e-11 1.7359448580318e-10 -100
"chr12" 49691001 49692000 "*" 0 0 63.4146341463415
"chr12" 49931001 49932000 "*" 0 0 65.2944250093989
"chr12" 49956001 49957000 "*" 7.69917077061422e-05 7.35006305210438e-05 -51.6129032258064
"chr12" 49959001 49960000 "*" 5.07371922253697e-14 1.73287898516782e-13 -100
"chr12" 50022001 50023000 "*" 6.30273611079701e-13 1.87665599796749e-12 81.25
"chr12" 50027001 50028000 "*" 3.74791257395923e-06 4.37656320759567e-06 78.2608695652174
"chr12" 50051001 50052000 "*" 1.12634901405784e-10 2.49563754426169e-10 100
"chr12" 50093001 50094000 "*" 1.37828859436695e-10 3.01445544267661e-10 -100
"chr12" 50099001 50100000 "*" 4.01352826597812e-05 4.00482226663819e-05 53.0434782608696
"chr12" 50100001 50101000 "*" 0 0 50.7935123822811
"chr12" 50232001 50233000 "*" 9.99200722162641e-16 4.15382497462808e-15 -62.0689655172414
"chr12" 50266001 50267000 "*" 8.33810798184231e-12 2.15649212979267e-11 -83.8709677419355
"chr12" 50293001 50294000 "*" 5.53979861983578e-10 1.11379168305788e-09 -90.3846153846154
"chr12" 50303001 50304000 "*" 0 0 -67.0719351570415
"chr12" 50314001 50315000 "*" 6.66133814775094e-15 2.52791665956799e-14 58.974358974359
"chr12" 50366001 50367000 "*" 0 0 -51.5873015873016
"chr12" 50419001 50420000 "*" 0 0 -85.7142857142857
"chr12" 50446001 50447000 "*" 4.08209022140227e-11 9.57770690255255e-11 -100
"chr12" 50476001 50477000 "*" 6.52733422867868e-12 1.71146951718423e-11 100
"chr12" 50706001 50707000 "*" 9.41469124882133e-14 3.10868561210786e-13 100
"chr12" 51457001 51458000 "*" 4.60763649456908e-11 1.07551104122606e-10 -60
"chr12" 51566001 51567000 "*" 1.16034810537791e-07 1.66884737210566e-07

76.2749445676275
"chr12" 51754001 51755000 "*" 0 0 -100
"chr12" 51799001 51800000 "*" 0 0 -100
"chr12" 51903001 51904000 "*" 9.04831765069503e-14 2.99233117281087e-13 -81.25
"chr12" 51931001 51932000 "*" 5.48638023900594e-10 1.10374357336421e-09 100
"chr12" 51984001 51985000 "*" 0 0 68.0209698558322
"chr12" 52186001 52187000 "*" 1.96644922567657e-11 4.82930612417392e-11 100
"chr12" 52212001 52213000 "*" 1.2404663962684e-09 2.36887234713147e-09
56.5217391304348
"chr12" 52224001 52225000 "*" 3.04324343503026e-11 7.26599935673121e-11 -100
"chr12" 52257001 52258000 "*" 3.88316623300966e-09 6.93665974730547e-09
65.6542056074766
"chr12" 52332001 52333000 "*" 6.04438721296674e-11 1.38849255306602e-10 -100
"chr12" 52341001 52342000 "*" 1.12055920098442e-11 2.84500040664577e-11 -100
"chr12" 52359001 52360000 "*" 4.32209823486573e-12 1.15935290479641e-11 -100
"chr12" 52399001 52400000 "*" 2.4535928844216e-14 8.69144396197119e-14 -100
"chr12" 52413001 52414000 "*" 1.96644922567657e-11 4.82930612417392e-11 100
"chr12" 52427001 52428000 "*" 0 0 100
"chr12" 52431001 52432000 "*" 0 0 87.2361947675755
"chr12" 52443001 52444000 "*" 2.53719267817587e-12 7.0151053476422e-12
92.3076923076923
"chr12" 52444001 52445000 "*" 0 0 -87.4358974358974
"chr12" 52445001 52446000 "*" 0 0 -71.6596617293157
"chr12" 52455001 52456000 "*" 1.33898878196259e-05 1.43911695897696e-05
62.962962962963
"chr12" 52513001 52514000 "*" 0 0 -79.6407185628743
"chr12" 52514001 52515000 "*" 4.44089209850063e-16 1.91071758245033e-15
-51.8072289156626
"chr12" 52537001 52538000 "*" 0 0 -71.0144927536232
"chr12" 52542001 52543000 "*" 2.61211179231058e-06 3.12359423291548e-06
72.2222222222222
"chr12" 52597001 52598000 "*" 7.0006535457523e-08 1.03695832512054e-07 100
"chr12" 52603001 52604000 "*" 8.7349629751543e-09 1.47505871898511e-08 -100
"chr12" 52607001 52608000 "*" 0 0 -80.7017543859649
"chr12" 52635001 52636000 "*" 0 0 100
"chr12" 52673001 52674000 "*" 2.05391259555654e-14 7.35435952190685e-14 -100
"chr12" 52713001 52714000 "*" 4.44089209850063e-15 1.71914916534614e-14 100
"chr12" 52719001 52720000 "*" 0 0 -100
"chr12" 52732001 52733000 "*" 7.97140131680862e-14 2.65641277678906e-13 100
"chr12" 52757001 52758000 "*" 8.47100167788994e-14 2.81502746928913e-13
74.7126436781609
"chr12" 52764001 52765000 "*" 2.00227675550835e-08 3.20459176617961e-08 100
"chr12" 52785001 52786000 "*" 7.37177208165463e-09 1.26096956234335e-08
-61.2244897959184
"chr12" 52799001 52800000 "*" 1.11022302462516e-16 5.03662826618488e-16
-50.9863331614234
"chr12" 52805001 52806000 "*" 6.66133814775094e-16 2.81595744474255e-15 -100
"chr12" 52809001 52810000 "*" 7.08248345016038e-06 7.93982511214607e-06
58.2278481012658
"chr12" 52818001 52819000 "*" 2.22044604925031e-16 9.81641919380259e-16
-71.1864406779661
"chr12" 52863001 52864000 "*" 0 0 100

"chr12" 52933001 52934000 "*" 2.71893618730701e-13 8.46692454027087e-13 -100
"chr12" 52942001 52943000 "*" 2.30698760095294e-09 4.24471350644025e-09 61.7021276595745
"chr12" 52961001 52962000 "*" 0 0 82.8125
"chr12" 53012001 53013000 "*" 4.79772954177227e-05 4.73172966813196e-05 -57.3770491803279
"chr12" 53041001 53042000 "*" 1.27409880423812e-08 2.10279873116017e-08 -56.25
"chr12" 53053001 53054000 "*" 5.72875080706581e-14 1.93967931988877e-13 100
"chr12" 53059001 53060000 "*" 7.0006535457523e-08 1.03695832512054e-07 100
"chr12" 53085001 53086000 "*" 0 0 71.3218424613213
"chr12" 53167001 53168000 "*" 5.295763827462e-14 1.80214784640623e-13 100
"chr12" 53170001 53171000 "*" 3.68371999570627e-13 1.13149976091305e-12 -100
"chr12" 53178001 53179000 "*" 0 0 100
"chr12" 53199001 53200000 "*" 0 0 100
"chr12" 53221001 53222000 "*" 3.34128893442198e-08 5.19772864618523e-08 -100
"chr12" 53225001 53226000 "*" 3.03979064142368e-13 9.42350883309283e-13 100
"chr12" 53228001 53229000 "*" 0 0 83.0769230769231
"chr12" 53230001 53231000 "*" 7.67056418382595e-11 1.7359448580318e-10 100
"chr12" 53244001 53245000 "*" 3.25678226653636e-06 3.838579860199e-06 65
"chr12" 53269001 53270000 "*" 3.33066907387547e-16 1.4495649018245e-15 100
"chr12" 53293001 53294000 "*" 1.88737914186277e-15 7.62011598380321e-15 -83.1683168316832
"chr12" 53294001 53295000 "*" 1.5277158427196e-09 2.87642564232045e-09 100
"chr12" 53318001 53319000 "*" 7.0006535457523e-08 1.03695832512054e-07 -100
"chr12" 53336001 53337000 "*" 1.19915188889763e-12 3.45720766408293e-12 80
"chr12" 53488001 53489000 "*" 1.01171097055541e-07 1.46715237217593e-07 -76.6666666666667
"chr12" 53512001 53513000 "*" 1.12055920098442e-11 2.84500040664577e-11 -100
"chr12" 53599001 53600000 "*" 2.82155461461819e-06 3.35763180330011e-06 -59.7014925373134
"chr12" 53607001 53608000 "*" 0 0 -100
"chr12" 53618001 53619000 "*" 5.01025332333427e-10 1.01358031135889e-09 100
"chr12" 53734001 53735000 "*" 2.04175676365992e-10 4.37646655354365e-10 80.672268907563
"chr12" 54005001 54006000 "*" 1.83238091366889e-10 3.95102886098283e-10 -90
"chr12" 54070001 54071000 "*" 1.93178806284777e-14 6.93672913283581e-14 80.4878048780488
"chr12" 54091001 54092000 "*" 0 0 -70.8955223880597
"chr12" 54152001 54153000 "*" 3.33066907387547e-16 1.4495649018245e-15 100
"chr12" 54201001 54202000 "*" 1.11022302462516e-16 5.03662826618488e-16 100
"chr12" 54235001 54236000 "*" 1.13140401492018e-08 1.87801380787728e-08 100
"chr12" 54330001 54331000 "*" 0 0 -67.6535087719298
"chr12" 54429001 54430000 "*" 3.83582835494778e-08 5.89882098092988e-08 -77.7777777777778
"chr12" 54476001 54477000 "*" 0 0 85.7142857142857
"chr12" 54528001 54529000 "*" 5.59980003478344e-08 8.43969181184147e-08 51.7746913580247
"chr12" 54598001 54599000 "*" 2.64951482975562e-09 4.82186011375752e-09 -100
"chr12" 54682001 54683000 "*" 5.52669021658403e-13 1.66011098865014e-12 52.3809523809524
"chr12" 54718001 54719000 "*" 0 0 -61.8556701030928
"chr12" 54749001 54750000 "*" 1.5277158427196e-09 2.87642564232045e-09 -100

"chr12" 54787001 54788000 "*" 3.03090885722668e-14 1.06210046699299e-13 100
"chr12" 54793001 54794000 "*" 4.01313771103418e-09 7.11882087237587e-09 100
"chr12" 54981001 54982000 "*" 9.99200722162641e-16 4.15382497462808e-15 100
"chr12" 54982001 54983000 "*" 0 0 53.2661977834612
"chr12" 55040001 55041000 "*" 0 0 87.5
"chr12" 55052001 55053000 "*" 9.52586898250729e-11 2.12930265171967e-10
-84.1666666666667
"chr12" 55179001 55180000 "*" 4.69101480993217e-08 7.14269369853421e-08
-77.2727272727273
"chr12" 55749001 55750000 "*" 7.0006535457523e-08 1.03695832512054e-07 100
"chr12" 55758001 55759000 "*" 7.0006535457523e-08 1.03695832512054e-07 -100
"chr12" 55808001 55809000 "*" 1.69112918113612e-07 2.37775599528879e-07
-67.741935483871
"chr12" 56011001 56012000 "*" 2.43005615629954e-09 4.45014222280278e-09 -100
"chr12" 56051001 56052000 "*" 5.48638023900594e-10 1.10374357336421e-09 -100
"chr12" 56060001 56061000 "*" 1.98241423277068e-12 5.55504245861376e-12
-94.9152542372881
"chr12" 56087001 56088000 "*" 2.15795584734835e-08 3.43975819311611e-08
-72.7272727272727
"chr12" 56120001 56121000 "*" 7.7715611723761e-16 3.26213507634405e-15
-80.6451612903226
"chr12" 56123001 56124000 "*" 6.51096689896491e-08 9.72647149904707e-08 75
"chr12" 56209001 56210000 "*" 2.35955699423585e-12 6.55416146074912e-12 81.25
"chr12" 56242001 56243000 "*" 9.76996261670138e-15 3.63402770315216e-14 -100
"chr12" 56495001 56496000 "*" 6.72119371269986e-09 1.15513474994851e-08
79.1044776119403
"chr12" 56531001 56532000 "*" 7.77825037712176e-09 1.32690687610048e-08
-94.4444444444444
"chr12" 56545001 56546000 "*" 0 0 -100
"chr12" 56553001 56554000 "*" 2.15125472990962e-10 4.58126659943874e-10 100
"chr12" 56602001 56603000 "*" 1.50623957750895e-12 4.28756717183481e-12 100
"chr12" 56651001 56652000 "*" 0 0 100
"chr12" 56661001 56662000 "*" 0 0 89.5424836601307
"chr12" 56842001 56843000 "*" 0 0 -100
"chr12" 56848001 56849000 "*" 5.07087083434499e-09 8.86854198896302e-09
-55.5555555555556
"chr12" 56902001 56903000 "*" 6.7390537594747e-14 2.26447131529591e-13 100
"chr12" 56975001 56976000 "*" 3.4639957569027e-11 8.22349663902986e-11 90
"chr12" 57083001 57084000 "*" 5.0011373464609e-05 4.91954071919253e-05
-56.8352059925094
"chr12" 57177001 57178000 "*" 2.22044604925031e-16 9.81641919380259e-16
-70.4545454545455
"chr12" 57319001 57320000 "*" 0 0 100
"chr12" 57323001 57324000 "*" 1.11022302462516e-16 5.03662826618488e-16
68.1818181818182
"chr12" 57481001 57482000 "*" 0 0 -73.3668341708543
"chr12" 57483001 57484000 "*" 0 0 -58.8235294117647
"chr12" 57490001 57491000 "*" 7.0006535457523e-08 1.03695832512054e-07 100
"chr12" 57492001 57493000 "*" 0 0 -85.593220338983
"chr12" 57504001 57505000 "*" 0 0 -100
"chr12" 57505001 57506000 "*" 3.26405569239796e-14 1.13973003849234e-13 -100
"chr12" 57543001 57544000 "*" 4.67611505072796e-11 1.09076258416177e-10

64.2857142857143
"chr12" 57549001 57550000 "*" 4.12718748066254e-11 9.67725582422511e-11
53.8461538461538
"chr12" 57576001 57577000 "*" 4.71134242729931e-12 1.257615036806e-11 100
"chr12" 57611001 57612000 "*" 0 0 -61.9954648526077
"chr12" 57612001 57613000 "*" 3.32659383628364e-06 3.914998084932e-06
-56.4102564102564
"chr12" 57624001 57625000 "*" 2.68673971959288e-14 9.47266815431045e-14 100
"chr12" 57625001 57626000 "*" 4.44089209850063e-15 1.71914916534614e-14 100
"chr12" 57633001 57634000 "*" 0 0 50.2792715558673
"chr12" 57636001 57637000 "*" 0 0 -57.3851294903927
"chr12" 57748001 57749000 "*" 7.9984455014781e-06 8.89173797399106e-06
66.6666666666667
"chr12" 57838001 57839000 "*" 2.72200032513936e-08 4.28754106422082e-08
69.3548387096774
"chr12" 57849001 57850000 "*" 4.99020824662466e-11 1.15790792586157e-10 100
"chr12" 57863001 57864000 "*" 2.67283972732457e-11 6.45686178157698e-11 100
"chr12" 57943001 57944000 "*" 0 0 78.0856423173803
"chr12" 57955001 57956000 "*" 3.34128893442198e-08 5.19772864618523e-08 -100
"chr12" 57984001 57985000 "*" 7.66053886991358e-15 2.88426834415713e-14
-67.9738562091503
"chr12" 58015001 58016000 "*" 0 0 56.9355680238907
"chr12" 58119001 58120000 "*" 0 0 -100
"chr12" 58133001 58134000 "*" 0 0 64
"chr12" 58146001 58147000 "*" 7.53841433720481e-14 2.52139165738615e-13
-54.2372881355932
"chr12" 58159001 58160000 "*" 0 0 76.9759708890144
"chr12" 58259001 58260000 "*" 0 0 -82.0571428571429
"chr12" 58266001 58267000 "*" 1.07792772406912e-05 1.17513174008031e-05
-55.5555555555556
"chr12" 58630001 58631000 "*" 4.01313771103418e-09 7.11882087237587e-09 100
"chr12" 59245001 59246000 "*" 6.60582699651968e-14 2.22176301799e-13 -100
"chr12" 59448001 59449000 "*" 3.40125705378114e-10 7.03784073980162e-10 -100
"chr12" 59989001 59990000 "*" 0 0 56.9002630949534
"chr12" 60138001 60139000 "*" 3.6700841921089e-08 5.65530226072258e-08 -100
"chr12" 60144001 60145000 "*" 1.95399252334028e-14 7.01253661173697e-14 100
"chr12" 60767001 60768000 "*" 8.57092175010621e-13 2.51149198099779e-12 100
"chr12" 60988001 60989000 "*" 2.15125472990962e-10 4.58126659943874e-10 100
"chr12" 61390001 61391000 "*" 6.4324989779152e-11 1.47230020825092e-10 100
"chr12" 63149001 63150000 "*" 3.6700841921089e-08 5.65530226072258e-08 -100
"chr12" 63905001 63906000 "*" 1.12376774552558e-12 3.24761818527664e-12 -100
"chr12" 64635001 64636000 "*" 8.7349629751543e-09 1.47505871898511e-08 -100
"chr12" 64926001 64927000 "*" 1.15972064318459e-06 1.45972698357591e-06
51.7241379310345
"chr12" 65408001 65409000 "*" 2.08814465718632e-09 3.86232567960463e-09 100
"chr12" 65888001 65889000 "*" 0 0 -100
"chr12" 65904001 65905000 "*" 1.14686038443779e-13 3.74462496140022e-13
59.058231488138
"chr12" 65940001 65941000 "*" 2.17381668221606e-13 6.85151076607736e-13 100
"chr12" 66135001 66136000 "*" 0 0 96.9465648854962
"chr12" 66136001 66137000 "*" 0 0 54.6745565654964
"chr12" 66636001 66637000 "*" 2.38575468822777e-05 2.46816531377115e-05

-66.6666666666667
"chr12" 66643001 66644000 "*" 1.54714019373614e-10 3.35812436152993e-10
72.9166666666667
"chr12" 67463001 67464000 "*" 0 0 83.0508474576271
"chr12" 68006001 68007000 "*" 3.63445940010365e-11 8.58565209111204e-11 -100
"chr12" 68172001 68173000 "*" 9.2800211959343e-12 2.38493848032318e-11
-55.5555555555556
"chr12" 69184001 69185000 "*" 7.67056418382595e-11 1.7359448580318e-10 100
"chr12" 69234001 69235000 "*" 0 0 100
"chr12" 69328001 69329000 "*" 3.03272448265446e-08 4.75110065895966e-08
66.6666666666667
"chr12" 69367001 69368000 "*" 3.6700841921089e-08 5.65530226072258e-08 -100
"chr12" 69481001 69482000 "*" 1.67299207820548e-08 2.71377429441055e-08 100
"chr12" 69493001 69494000 "*" 6.4152538836737e-10 1.27477697728417e-09 -100
"chr12" 69754001 69755000 "*" 0 0 -100
"chr12" 69863001 69864000 "*" 1.43550171749496e-09 2.72175514902758e-09 75
"chr12" 69900001 69901000 "*" 9.5812247025151e-14 3.15667542866807e-13 100
"chr12" 69999001 70000000 "*" 0 0 -100
"chr12" 70759001 70760000 "*" 0 0 74.6556473829201
"chr12" 70760001 70761000 "*" 0 0 78.6347517730496
"chr12" 70799001 70800000 "*" 2.00227675550835e-08 3.20459176617961e-08 100
"chr12" 71003001 71004000 "*" 0 0 72.6449275362319
"chr12" 72345001 72346000 "*" 1.96509475358653e-13 6.23868565133654e-13 -100
"chr12" 72540001 72541000 "*" 0 0 -67.3358526216344
"chr12" 72712001 72713000 "*" 7.67056418382595e-11 1.7359448580318e-10 -100
"chr12" 73092001 73093000 "*" 6.4152538836737e-10 1.27477697728417e-09 100
"chr12" 73666001 73667000 "*" 6.04438721296674e-11 1.38849255306602e-10 100
"chr12" 74219001 74220000 "*" 6.4324989779152e-11 1.47230020825092e-10 -100
"chr12" 74645001 74646000 "*" 1.64488768272797e-07 2.31523769558304e-07
-81.8181818181818
"chr12" 74903001 74904000 "*" 0 0 87.2832369942196
"chr12" 75784001 75785000 "*" 0 0 -100
"chr12" 75917001 75918000 "*" 6.95250634841216e-10 1.37589605662581e-09
-52.3809523809524
"chr12" 76645001 76646000 "*" 8.46938562615307e-08 1.24078250443771e-07
-53.880266075388
"chr12" 76709001 76710000 "*" 7.67056418382595e-11 1.7359448580318e-10 100
"chr12" 76742001 76743000 "*" 0 0 80
"chr12" 76787001 76788000 "*" 8.88178419700125e-16 3.70670032207938e-15 -100
"chr12" 77311001 77312000 "*" 5.58892692403834e-06 6.36220245656122e-06 53.125
"chr12" 77412001 77413000 "*" 9.08108033215171e-11 2.03407753381119e-10 -100
"chr12" 77423001 77424000 "*" 6.92287338566189e-11 1.57605630124247e-10 100
"chr12" 78430001 78431000 "*" 4.08209022140227e-11 9.57770690255255e-11 100
"chr12" 80683001 80684000 "*" 0 0 -100
"chr12" 81330001 81331000 "*" 0 0 75
"chr12" 82496001 82497000 "*" 4.91828799908944e-14 1.68192819018269e-13 100
"chr12" 82610001 82611000 "*" 4.59076887615595e-10 9.34931929815876e-10
55.5555555555556
"chr12" 83238001 83239000 "*" 1.98124849859482e-10 4.25157611354621e-10 100
"chr12" 83399001 83400000 "*" 7.0006535457523e-08 1.03695832512054e-07 100
"chr12" 83501001 83502000 "*" 6.4324989779152e-11 1.47230020825092e-10 100
"chr12" 83959001 83960000 "*" 8.21565038222616e-15 3.08158744293689e-14 100

"chr12" 87565001 87566000 "*" 0 0 100
"chr12" 87662001 87663000 "*" 1.75082170983387e-13 5.6014942024636e-13 100
"chr12" 89746001 89747000 "*" 7.14428516346288e-13 2.11335164213332e-12
-73.8255033557047
"chr12" 89747001 89748000 "*" 6.45096853713056e-09 1.11431852890575e-08
-82.1490467937608
"chr12" 89748001 89749000 "*" 0 0 -79.6934865900383
"chr12" 90758001 90759000 "*" 9.08108033215171e-11 2.03407753381119e-10 100
"chr12" 90804001 90805000 "*" 0 0 89.0710382513661
"chr12" 91130001 91131000 "*" 6.30384633382164e-13 1.87665599796749e-12 100
"chr12" 92305001 92306000 "*" 2.455369241261e-12 6.8003688121118e-12 -100
"chr12" 93592001 93593000 "*" 9.04570973681018e-10 1.76414389822173e-09 100
"chr12" 93925001 93926000 "*" 1.59301718808402e-05 1.69294262236803e-05
66.6666666666667
"chr12" 94204001 94205000 "*" 2.79440914852103e-11 6.73046092948135e-11 100
"chr12" 94223001 94224000 "*" 3.84137166520304e-14 1.32929369817675e-13 100
"chr12" 94286001 94287000 "*" 2.22044604925031e-16 9.81641919380259e-16 100
"chr12" 94509001 94510000 "*" 2.15125472990962e-10 4.58126659943874e-10 100
"chr12" 94547001 94548000 "*" 0 0 -100
"chr12" 94603001 94604000 "*" 1.59301718808402e-05 1.69294262236803e-05
-66.6666666666667
"chr12" 94651001 94652000 "*" 5.6621374255883e-15 2.16468580166064e-14 100
"chr12" 94929001 94930000 "*" 1.91224813761437e-12 5.36527594469447e-12 -100
"chr12" 94954001 94955000 "*" 0 0 -84.4036697247706
"chr12" 94972001 94973000 "*" 1.83952852950142e-12 5.17499701329218e-12 100
"chr12" 95009001 95010000 "*" 9.65729496371637e-10 1.87255912493232e-09 -100
"chr12" 95043001 95044000 "*" 0 0 66.7977900811987
"chr12" 95044001 95045000 "*" 0 0 86.7866847826087
"chr12" 95268001 95269000 "*" 0 0 -100
"chr12" 95376001 95377000 "*" 2.06501482580279e-14 7.3890370570066e-14 100
"chr12" 95438001 95439000 "*" 1.19904086659517e-14 4.40901388691796e-14 -100
"chr12" 96185001 96186000 "*" 0 0 96.6101694915254
"chr12" 96409001 96410000 "*" 8.01666433236647e-09 1.36392368970109e-08 -100
"chr12" 96472001 96473000 "*" 1.55229628806541e-06 1.91995539564183e-06
-60.7142857142857
"chr12" 96525001 96526000 "*" 3.40125705378114e-10 7.03784073980162e-10 -100
"chr12" 96624001 96625000 "*" 2.15537587777703e-11 5.25828849348495e-11
-94.6666666666667
"chr12" 97380001 97381000 "*" 3.79304698761018e-10 7.80322551567583e-10
85.7142857142857
"chr12" 97391001 97392000 "*" 5.01025332333427e-10 1.01358031135889e-09 -100
"chr12" 97718001 97719000 "*" 5.72875080706581e-14 1.93967931988877e-13 100
"chr12" 98464001 98465000 "*" 9.65729496371637e-10 1.87255912493232e-09 -100
"chr12" 98522001 98523000 "*" 3.6700841921089e-08 5.65530226072258e-08 100
"chr12" 98704001 98705000 "*" 3.95353005888666e-09 7.03928529283733e-09 -100
"chr12" 98862001 98863000 "*" 9.65729496371637e-10 1.87255912493232e-09 100
"chr12" 98952001 98953000 "*" 2.88779000712225e-11 6.92935809466068e-11 -100
"chr12" 99251001 99252000 "*" 0 0 -100
"chr12" 99287001 99288000 "*" 0 0 63.448275862069
"chr12" 99674001 99675000 "*" 1.4432899320127e-15 5.90750815956055e-15 100
"chr12" 99776001 99777000 "*" 3.61932706027801e-14 1.25777907157994e-13 100
"chr12" 100042001 100043000 "*" 4.73234496034536e-09 8.30627300264826e-09 100

"chr12" 100338001 100339000 "*" 4.02167188440217e-12 1.08266700497926e-11 -100
"chr12" 100774001 100775000 "*" 2.00227675550835e-08 3.20459176617961e-08 -100
"chr12" 100891001 100892000 "*" 7.93809462606987e-14 2.64679416473686e-13 100
"chr12" 100910001 100911000 "*" 0 0 -100
"chr12" 101540001 101541000 "*" 1.11022302462516e-16 5.03662826618488e-16 -100
"chr12" 101669001 101670000 "*" 0 0 100
"chr12" 101828001 101829000 "*" 3.03090885722668e-14 1.06210046699299e-13 100
"chr12" 101871001 101872000 "*" 1.07882591748876e-11 2.74553537455803e-11 100
"chr12" 102260001 102261000 "*" 4.59412452524788e-09 8.0974087529323e-09
78.5714285714286
"chr12" 102349001 102350000 "*" 1.36604061395929e-11 3.42333952202875e-11 -100
"chr12" 103204001 103205000 "*" 0 0 -100
"chr12" 103351001 103352000 "*" 2.36712810464113e-08 3.75416231661109e-08
77.5784753363229
"chr12" 103356001 103357000 "*" 0 0 84.5714285714286
"chr12" 104119001 104120000 "*" 1.48487888651516e-11 3.70689457767456e-11 100
"chr12" 104136001 104137000 "*" 2.13762341161328e-12 5.95992653318919e-12
90.5405405405405
"chr12" 104175001 104176000 "*" 0 0 -100
"chr12" 104185001 104186000 "*" 0 0 -100
"chr12" 104444001 104445000 "*" 0 0 -70.7865168539326
"chr12" 104532001 104533000 "*" 1.63202784619898e-12 4.61929884772104e-12
-73.9130434782609
"chr12" 104756001 104757000 "*" 6.66133814775094e-16 2.81595744474255e-15
-72.972972972973
"chr12" 104847001 104848000 "*" 5.68195006989125e-06 6.46183399435396e-06
69.941348973607
"chr12" 105028001 105029000 "*" 4.71060745965701e-10 9.57233919609297e-10
87.9518072289157
"chr12" 105086001 105087000 "*" 6.99440505513849e-15 2.64480529781883e-14
72.093023255814
"chr12" 105096001 105097000 "*" 0 0 78.6590304292491
"chr12" 105102001 105103000 "*" 4.44089209850063e-16 1.91071758245033e-15 -100
"chr12" 105113001 105114000 "*" 8.3882012447134e-11 1.88922989503423e-10 -100
"chr12" 105135001 105136000 "*" 0 0 100
"chr12" 105955001 105956000 "*" 1.12055920098442e-11 2.84500040664577e-11 -100
"chr12" 106134001 106135000 "*" 1.13140401492018e-08 1.87801380787728e-08 -100
"chr12" 106489001 106490000 "*" 3.45309336680089e-11 8.19878134409357e-11
68.4210526315789
"chr12" 106517001 106518000 "*" 1.56863411149288e-12 4.4469621057462e-12 100
"chr12" 106542001 106543000 "*" 8.67652620750725e-07 1.1107452797228e-06
70.2702702702703
"chr12" 106639001 106640000 "*" 0 0 -100
"chr12" 106754001 106755000 "*" 2.64951482975562e-09 4.82186011375752e-09 -100
"chr12" 106945001 106946000 "*" 1.11022302462516e-15 4.59817122935606e-15
65.7894736842105
"chr12" 106975001 106976000 "*" 0 0 52.6780523255814
"chr12" 107621001 107622000 "*" 1.93720595120794e-11 4.76309924836636e-11 100
"chr12" 107809001 107810000 "*" 3.77475828372553e-15 1.47379557022071e-14 -100
"chr12" 107819001 107820000 "*" 8.42958795810311e-06 9.33879080874541e-06
-52.3809523809524
"chr12" 107839001 107840000 "*" 3.6700841921089e-08 5.65530226072258e-08 100

"chr12" 107849001 107850000 "*" 1.52794177310511e-09 2.87642564232045e-09 100
"chr12" 107856001 107857000 "*" 3.34128893442198e-08 5.19772864618523e-08 100
"chr12" 107903001 107904000 "*" 8.02691246803988e-14 2.67352274101148e-13 -100
"chr12" 107914001 107915000 "*" 9.80763359414993e-10 1.8980032424715e-09 -100
"chr12" 107937001 107938000 "*" 1.88737914186277e-15 7.62011598380321e-15 -100
"chr12" 108040001 108041000 "*" 1.07025499573865e-13 3.50892415205218e-13 -89.4736842105263
"chr12" 108064001 108065000 "*" 1.54630752646767e-11 3.85079502648228e-11 -100
"chr12" 108168001 108169000 "*" 0 0 76.7857142857143
"chr12" 108267001 108268000 "*" 1.88737914186277e-15 7.62011598380321e-15 85
"chr12" 108297001 108298000 "*" 0 0 83.1553973902728
"chr12" 108343001 108344000 "*" 4.36756633549473e-05 4.33501227512163e-05 -62
"chr12" 108448001 108449000 "*" 3.56775498033812e-10 7.35898629809829e-10 -100
"chr12" 108514001 108515000 "*" 0 0 -100
"chr12" 108686001 108687000 "*" 7.7715611723761e-13 2.29280702649121e-12 82.5396825396825
"chr12" 108722001 108723000 "*" 6.24654217240561e-10 1.2462108191303e-09 -100
"chr12" 108727001 108728000 "*" 6.24654217240561e-10 1.2462108191303e-09 -100
"chr12" 108735001 108736000 "*" 3.6700841921089e-08 5.65530226072258e-08 100
"chr12" 108756001 108757000 "*" 1.82510129231872e-09 3.40967518972438e-09 -60.5392156862745
"chr12" 108908001 108909000 "*" 4.39648317751562e-14 1.5131009830411e-13 85.1351351351351
"chr12" 108987001 108988000 "*" 3.01410632563837e-07 4.10360390967729e-07 76.9230769230769
"chr12" 108990001 108991000 "*" 3.46146957430626e-07 4.67482227931052e-07 -79.5918367346939
"chr12" 109007001 109008000 "*" 5.6362137179633e-11 1.29963270300065e-10 100
"chr12" 109055001 109056000 "*" 4.19442258703384e-13 1.27549685387611e-12 -79.7101449275362
"chr12" 109136001 109137000 "*" 6.59550958292954e-09 1.13456194382773e-08 -100
"chr12" 109289001 109290000 "*" 7.31858089020321e-07 9.46552240977687e-07 -68.75
"chr12" 109431001 109432000 "*" 9.65729496371637e-10 1.87255912493232e-09 100
"chr12" 109534001 109535000 "*" 2.22044604925031e-16 9.81641919380259e-16 66.6666666666667
"chr12" 109535001 109536000 "*" 0 0 -59.1397849462366
"chr12" 109554001 109555000 "*" 1.0099698855015e-12 2.93256945198031e-12 55
"chr12" 109654001 109655000 "*" 1.12055920098442e-11 2.84500040664577e-11 100
"chr12" 109669001 109670000 "*" 7.48072703782299e-08 1.10340422120243e-07 90.9090909090909
"chr12" 109696001 109697000 "*" 1.66533453693773e-15 6.7629186866784e-15 80.2083333333333
"chr12" 109711001 109712000 "*" 5.400901947894e-12 1.43100990157687e-11 55.0489139446195
"chr12" 109797001 109798000 "*" 1.51170954421076e-06 1.87292218199121e-06 72.972972972973
"chr12" 109855001 109856000 "*" 8.57092175010621e-13 2.51149198099779e-12 100
"chr12" 109907001 109908000 "*" 4.9560355819267e-13 1.49846585607346e-12 98.1481481481482
"chr12" 109981001 109982000 "*" 1.11022302462516e-16 5.03662826618488e-16 65.9582999198075

"chr12" 110053001 110054000 "*" 1.39779633423487e-07 1.98463037958589e-07 100
"chr12" 110076001 110077000 "*" 1.77635683940025e-15 7.19870740878856e-15 100
"chr12" 110080001 110081000 "*" 0 0 -77.9661016949153
"chr12" 110101001 110102000 "*" 3.42280648268911e-11 8.12872163131922e-11 -73.6842105263158
"chr12" 110153001 110154000 "*" 5.18474152499948e-14 1.76689027414982e-13 -55.0994575045208
"chr12" 110162001 110163000 "*" 5.90114479503967e-09 1.02452139728008e-08 -84.3971631205674
"chr12" 110178001 110179000 "*" 0 0 -100
"chr12" 110192001 110193000 "*" 9.65729496371637e-10 1.87255912493232e-09 -100
"chr12" 110261001 110262000 "*" 0 0 -80.3149606299213
"chr12" 110283001 110284000 "*" 1.02029495963052e-13 3.34986657538201e-13 -95.6331877729258
"chr12" 110293001 110294000 "*" 0 0 52.2307786334189
"chr12" 110357001 110358000 "*" 1.24984033345044e-07 1.79000832707579e-07 66.6666666666667
"chr12" 110360001 110361000 "*" 0.000188913003384639 0.000169331787295583 54.5454545454545
"chr12" 110365001 110366000 "*" 2.98699205369957e-08 4.68327071459571e-08 60
"chr12" 110490001 110491000 "*" 2.62900812231237e-13 8.21294938531841e-13 100
"chr12" 110787001 110788000 "*" 4.25916968360696e-10 8.71293679327487e-10 -56.8965517241379
"chr12" 110806001 110807000 "*" 2.22044604925031e-16 9.81641919380259e-16 -100
"chr12" 110842001 110843000 "*" 3.88022947106492e-13 1.1867462770515e-12 -100
"chr12" 111093001 111094000 "*" 1.12804847018921e-07 1.62520779461317e-07 72.2222222222222
"chr12" 111140001 111141000 "*" 8.80406858527749e-14 2.91456141063452e-13 -100
"chr12" 111182001 111183000 "*" 1.13140401492018e-08 1.87801380787728e-08 100
"chr12" 111291001 111292000 "*" 8.15347789284715e-13 2.39962502290376e-12 -66.6666666666667
"chr12" 111325001 111326000 "*" 0 0 79.2857142857143
"chr12" 111346001 111347000 "*" 3.33066907387547e-16 1.4495649018245e-15 -100
"chr12" 111352001 111353000 "*" 6.4152538836737e-10 1.27477697728417e-09 -100
"chr12" 111404001 111405000 "*" 2.33590924381133e-13 7.3418676838825e-13 55.2083333333333
"chr12" 111462001 111463000 "*" 1.48087875295744e-10 3.22075573380618e-10 -100
"chr12" 111478001 111479000 "*" 5.398668037504e-05 5.2808752078579e-05 -57.5757575757576
"chr12" 111486001 111487000 "*" 0 0 87.3563218390805
"chr12" 111515001 111516000 "*" 2.47782997270241e-05 2.55562250124639e-05 -55.2380952380952
"chr12" 111529001 111530000 "*" 1.48487888651516e-11 3.70689457767456e-11 -100
"chr12" 111531001 111532000 "*" 1.83982384882597e-10 3.96625717407801e-10 91.4285714285714
"chr12" 111540001 111541000 "*" 1.74217518729147e-05 1.84143748638769e-05 60.4938271604938
"chr12" 111616001 111617000 "*" 0 0 70
"chr12" 111624001 111625000 "*" 2.96184077264172e-10 6.18229013794136e-10 -80.4347826086957
"chr12" 111625001 111626000 "*" 2.22044604925031e-16 9.81641919380259e-16 84.2105263157895

"chr12" 111629001 111630000 "*" 3.10077874622339e-05 3.15124728670996e-05
64.7058823529412
"chr12" 111638001 111639000 "*" 0 0 -100
"chr12" 111641001 111642000 "*" 7.93809462606987e-14 2.64679416473686e-13 -100
"chr12" 111643001 111644000 "*" 1.32325323474181e-05 1.42328688846738e-05
66.6666666666667
"chr12" 111649001 111650000 "*" 1.36604061395929e-11 3.42333952202875e-11 100
"chr12" 111684001 111685000 "*" 0 0 -97.1428571428571
"chr12" 111732001 111733000 "*" 2.83626316543995e-07 3.87577540941096e-07
88.2352941176471
"chr12" 111735001 111736000 "*" 2.52225684782559e-10 5.32659726135026e-10
-68.75
"chr12" 111785001 111786000 "*" 0 0 100
"chr12" 111802001 111803000 "*" 2.58681964737661e-14 9.14008479605639e-14 100
"chr12" 111807001 111808000 "*" 0 0 -85.9154929577465
"chr12" 111823001 111824000 "*" 1.52794177310511e-09 2.87642564232045e-09 100
"chr12" 112213001 112214000 "*" 2.23421303680027e-09 4.11871828003941e-09
66.6666666666667
"chr12" 112338001 112339000 "*" 1.03761443881467e-11 2.64965607418752e-11
-64.1509433962264
"chr12" 112819001 112820000 "*" 1.85296222809939e-13 5.91421130049794e-13
-54.2222222222222
"chr12" 112829001 112830000 "*" 5.34443600486156e-11 1.2362835960376e-10
-61.7647058823529
"chr12" 112847001 112848000 "*" 0 0 -81.8181818181818
"chr12" 113110001 113111000 "*" 0 0 100
"chr12" 113197001 113198000 "*" 0 0 75
"chr12" 113205001 113206000 "*" 1.01091713888835e-08 1.69415251299883e-08
93.3333333333333
"chr12" 113336001 113337000 "*" 5.55111512312578e-16 2.36485094870365e-15
78.3783783783784
"chr12" 113376001 113377000 "*" 8.80406858527749e-14 2.91456141063452e-13 -100
"chr12" 113424001 113425000 "*" 1.41019544774856e-06 1.75486844532563e-06
71.0526315789474
"chr12" 113500001 113501000 "*" 0 0 51.5984843999763
"chr12" 113516001 113517000 "*" 1.92946192356658e-09 3.59193011611483e-09 100
"chr12" 113519001 113520000 "*" 6.4152538836737e-10 1.27477697728417e-09 -100
"chr12" 113524001 113525000 "*" 3.42522743501661e-09 6.15633712685039e-09 54
"chr12" 113527001 113528000 "*" 1.32075309233759e-06 1.64949652474337e-06
54.1666666666667
"chr12" 113533001 113534000 "*" 0 0 92.5
"chr12" 113567001 113568000 "*" 1.52794177310511e-09 2.87642564232045e-09 100
"chr12" 113591001 113592000 "*" 5.60042358044299e-08 8.44047905479906e-08
56.964656964657
"chr12" 113613001 113614000 "*" 2.55870880039311e-11 6.19748779645345e-11
95.8333333333333
"chr12" 113727001 113728000 "*" 0 0 -100
"chr12" 113728001 113729000 "*" 3.34128893442198e-08 5.19772864618523e-08 -100
"chr12" 113752001 113753000 "*" 4.90718576884319e-14 1.6794399166207e-13 -100
"chr12" 113766001 113767000 "*" 8.77076189453874e-15 3.27889038038365e-14 -100
"chr12" 113772001 113773000 "*" 0 0 -93.3333333333333
"chr12" 113775001 113776000 "*" 9.0072393987839e-13 2.63157725646847e-12 100

"chr12" 113809001 113810000 "*" 5.43117762319412e-06 6.19387126034938e-06 -84.6153846153846
"chr12" 113821001 113822000 "*" 9.47020240005259e-14 3.12491189162604e-13 -100
"chr12" 113831001 113832000 "*" 0 0 75
"chr12" 113870001 113871000 "*" 2.08814465718632e-09 3.86232567960463e-09 100
"chr12" 113894001 113895000 "*" 2.22044604925031e-16 9.81641919380259e-16 100
"chr12" 113903001 113904000 "*" 0 0 50.2510332159804
"chr12" 113905001 113906000 "*" 0 0 54.9745761970192
"chr12" 113912001 113913000 "*" 7.2055397093429e-08 1.06545231128777e-07 75
"chr12" 113917001 113918000 "*" 0 0 53.9078077741683
"chr12" 113943001 113944000 "*" 6.04438721296674e-11 1.38849255306602e-10 100
"chr12" 114004001 114005000 "*" 7.94730947717426e-12 2.05982705398163e-11 97.5609756097561
"chr12" 114029001 114030000 "*" 0 0 58.2042672080936
"chr12" 114048001 114049000 "*" 1.48214773787458e-13 4.77488235285846e-13 100
"chr12" 114075001 114076000 "*" 0 0 78.7835767276379
"chr12" 114077001 114078000 "*" 0 0 92.1212121212121
"chr12" 114087001 114088000 "*" 0 0 54.5454545454545
"chr12" 114104001 114105000 "*" 2.4535928844216e-14 8.69144396197119e-14 100
"chr12" 114105001 114106000 "*" 6.92287338566189e-11 1.57605630124247e-10 100
"chr12" 114112001 114113000 "*" 4.77395900588817e-15 1.84201484337652e-14 100
"chr12" 114122001 114123000 "*" 0 0 51.1627906976744
"chr12" 114238001 114239000 "*" 1.22124532708767e-15 5.03921984217778e-15 100
"chr12" 114242001 114243000 "*" 1.07449546427052e-06 1.35827151898915e-06 70.2380952380952
"chr12" 114287001 114288000 "*" 0 0 100
"chr12" 114350001 114351000 "*" 1.43443135147692e-09 2.71978753395078e-09 57.1428571428571
"chr12" 114404001 114405000 "*" 5.88418203051333e-15 2.245355428447e-14 -100
"chr12" 114793001 114794000 "*" 2.19380069665931e-13 6.90875305343023e-13 -56.8421052631579
"chr12" 115069001 115070000 "*" 1.74853465040314e-11 4.32752710900609e-11 -100
"chr12" 115129001 115130000 "*" 1.11022302462516e-16 5.03662826618488e-16 100
"chr12" 115131001 115132000 "*" 4.73234496034536e-09 8.30627300264826e-09 100
"chr12" 115139001 115140000 "*" 5.01025332333427e-10 1.01358031135889e-09 -100
"chr12" 115212001 115213000 "*" 7.0006535457523e-08 1.03695832512054e-07 100
"chr12" 115225001 115226000 "*" 6.88338275267597e-15 2.60518659957509e-14 100
"chr12" 115256001 115257000 "*" 1.07813388586786e-05 1.1753410651612e-05 -60.9022556390977
"chr12" 115269001 115270000 "*" 3.33066907387547e-16 1.4495649018245e-15 66.6666666666667
"chr12" 115775001 115776000 "*" 1.07864652044709e-05 1.17574627643031e-05 -63.8888888888889
"chr12" 115949001 115950000 "*" 3.52704532247117e-11 8.35581292290093e-11 100
"chr12" 116037001 116038000 "*" 0 0 100
"chr12" 116152001 116153000 "*" 1.35890187991095e-11 3.40841971363279e-11 100
"chr12" 116220001 116221000 "*" 7.0006535457523e-08 1.03695832512054e-07 100
"chr12" 116384001 116385000 "*" 3.33066907387547e-16 1.4495649018245e-15 100
"chr12" 116724001 116725000 "*" 5.01025332333427e-10 1.01358031135889e-09 -100
"chr12" 116749001 116750000 "*" 0 0 -100
"chr12" 116806001 116807000 "*" 2.68450373042128e-10 5.64465213127486e-10 100
"chr12" 116873001 116874000 "*" 1.06183120962555e-07 1.53590937852418e-07

-86.9565217391304
"chr12" 116985001 116986000 "*" 1.14551701457799e-11 2.90182903764696e-11
-88.5906040268456
"chr12" 117065001 117066000 "*" 2.88710388929303e-10 6.03399264731139e-10 100
"chr12" 117135001 117136000 "*" 7.7715611723761e-16 3.26213507634405e-15 100
"chr12" 117256001 117257000 "*" 0 0 -88.695652173913
"chr12" 117271001 117272000 "*" 6.81765754961816e-12 1.78014899764838e-11
52.1739130434783
"chr12" 117298001 117299000 "*" 2.51786181793001e-07 3.46503358726054e-07
52.9411764705882
"chr12" 117319001 117320000 "*" 0 0 67.4317750845082
"chr12" 117384001 117385000 "*" 1.13140401492018e-08 1.87801380787728e-08 100
"chr12" 117599001 117600000 "*" 8.44949322398314e-07 1.08322745972012e-06
53.3333333333333
"chr12" 117613001 117614000 "*" 1.11022302462516e-16 5.03662826618488e-16 -100
"chr12" 117658001 117659000 "*" 1.68753899743024e-14 6.09826805967439e-14 100
"chr12" 117675001 117676000 "*" 2.16382467499443e-13 6.82775854166559e-13 100
"chr12" 117732001 117733000 "*" 0 0 60.655737704918
"chr12" 117755001 117756000 "*" 3.34128893442198e-08 5.19772864618523e-08 -100
"chr12" 117803001 117804000 "*" 8.32667268468867e-15 3.12210720380152e-14
53.7313432835821
"chr12" 117807001 117808000 "*" 9.65729496371637e-10 1.87255912493232e-09 100
"chr12" 117831001 117832000 "*" 1.47472950695615e-06 1.83001485994317e-06
66.6666666666667
"chr12" 118019001 118020000 "*" 6.37490060739765e-13 1.89496793587877e-12 100
"chr12" 118100001 118101000 "*" 2.22044604925031e-16 9.81641919380259e-16 100
"chr12" 118346001 118347000 "*" 1.66533453693773e-15 6.7629186866784e-15 100
"chr12" 118350001 118351000 "*" 2.96028862534214e-08 4.64323581179945e-08
-78.2608695652174
"chr12" 118418001 118419000 "*" 6.52733422867868e-12 1.71146951718423e-11 100
"chr12" 118427001 118428000 "*" 1.38590361409285e-10 3.02706178892116e-10 100
"chr12" 118461001 118462000 "*" 3.05311331771918e-12 8.34639368772922e-12 100
"chr12" 118539001 118540000 "*" 1.03448583033128e-10 2.30280358871788e-10 -80
"chr12" 118564001 118565000 "*" 2.77555756156289e-15 1.10015415195978e-14
-50.8771929824561
"chr12" 118574001 118575000 "*" 0 0 -97.4093264248705
"chr12" 118576001 118577000 "*" 1.98365768255826e-11 4.85612402473442e-11 -100
"chr12" 118747001 118748000 "*" 8.06688049692639e-13 2.37556391305009e-12 -100
"chr12" 119211001 119212000 "*" 3.212705657063e-10 6.67801238585205e-10
60.2150537634409
"chr12" 119446001 119447000 "*" 3.5527136788005e-15 1.39277426410519e-14 70
"chr12" 119632001 119633000 "*" 0 0 -89.8550724637681
"chr12" 120313001 120314000 "*" 5.30231414330729e-12 1.40618384350073e-11
92.8571428571429
"chr12" 120490001 120491000 "*" 1.67299207820548e-08 2.71377429441055e-08 100
"chr12" 120672001 120673000 "*" 0 0 -100
"chr12" 120693001 120694000 "*" 6.58243449525031e-07 8.57004003654891e-07
-81.8181818181818
"chr12" 120694001 120695000 "*" 2.77788437808013e-08 4.36995426069457e-08
-85.7142857142857
"chr12" 120699001 120700000 "*" 4.03546211114048e-07 5.39887313983092e-07
-60.2409638554217

"chr12" 120729001 120730000 "*" 3.19300141882195e-13 9.87864167952596e-13
-66.6666666666667
"chr12" 120786001 120787000 "*" 7.48072703782299e-08 1.10340422120243e-07
83.3333333333333
"chr12" 120858001 120859000 "*" 1.23694513355366e-05 1.3360368613825e-05
-69.4444444444444
"chr12" 120933001 120934000 "*" 0 0 100
"chr12" 121020001 121021000 "*" 3.91921461950773e-09 6.99820649668721e-09
56.5217391304348
"chr12" 121086001 121087000 "*" 2.88779000712225e-11 6.92935809466068e-11 -100
"chr12" 121097001 121098000 "*" 0 0 100
"chr12" 121105001 121106000 "*" 1.05687417228939e-06 1.33727857294019e-06
63.659793814433
"chr12" 121204001 121205000 "*" 0 0 51.3274336283186
"chr12" 121375001 121376000 "*" 4.01313771103418e-09 7.11882087237587e-09 100
"chr12" 121387001 121388000 "*" 3.34307824245705e-06 3.93328803212408e-06
-55.8823529411765
"chr12" 121394001 121395000 "*" 6.15840711759574e-13 1.83676803572802e-12 100
"chr12" 121433001 121434000 "*" 2.56300714840396e-06 3.06861399840947e-06
62.962962962963
"chr12" 121479001 121480000 "*" 1.90181204118289e-13 6.05298617539364e-13 100
"chr12" 121491001 121492000 "*" 3.03979064142368e-13 9.42350883309283e-13 100
"chr12" 121568001 121569000 "*" 2.43005615629954e-09 4.45014222280278e-09 100
"chr12" 121668001 121669000 "*" 3.25439675208372e-12 8.87454084945396e-12
-64.8148148148148
"chr12" 121975001 121976000 "*" 0 0 -93.4348239771646
"chr12" 121976001 121977000 "*" 0 0 -94.25
"chr12" 121996001 121997000 "*" 5.01831909360817e-12 1.3340363656552e-11
60.8695652173913
"chr12" 122007001 122008000 "*" 2.36695339073112e-06 2.84951084027545e-06
-74.4186046511628
"chr12" 122017001 122018000 "*" 0 0 63.3058189508152
"chr12" 122020001 122021000 "*" 2.66453525910038e-14 9.40074020513994e-14 100
"chr12" 122045001 122046000 "*" 7.13039608468691e-08 1.05488346247008e-07 72.5
"chr12" 122059001 122060000 "*" 4.44089209850063e-16 1.91071758245033e-15 -100
"chr12" 122098001 122099000 "*" 0 0 75
"chr12" 122112001 122113000 "*" 5.45632428128329e-11 1.26115136754623e-10
64.4444444444444
"chr12" 122116001 122117000 "*" 1.4432899320127e-15 5.90750815956055e-15 -100
"chr12" 122124001 122125000 "*" 5.55111512312578e-16 2.36485094870365e-15
-76.9911504424779
"chr12" 122214001 122215000 "*" 6.04072347698548e-13 1.80508974626227e-12
57.5757575757576
"chr12" 122235001 122236000 "*" 0 0 -78.8793103448276
"chr12" 122278001 122279000 "*" 4.92385296679831e-06 5.64921544609157e-06
-60.4651162790698
"chr12" 122303001 122304000 "*" 8.65973959207622e-14 2.87362558212404e-13 -100
"chr12" 122402001 122403000 "*" 1.17683640610267e-14 4.33290554866129e-14
-94.1176470588235
"chr12" 122414001 122415000 "*" 1.03245623161996e-09 1.99309236182236e-09
84.6153846153846
"chr12" 122434001 122435000 "*" 3.6892711108294e-13 1.13266255090498e-12 100

"chr12" 122446001 122447000 "*" 3.33066907387547e-16 1.4495649018245e-15
97.3684210526316
"chr12" 122448001 122449000 "*" 5.72875080706581e-14 1.93967931988877e-13 100
"chr12" 122485001 122486000 "*" 3.33066907387547e-16 1.4495649018245e-15
-53.9204545454545
"chr12" 122487001 122488000 "*" 8.88178419700125e-16 3.70670032207938e-15 -100
"chr12" 122500001 122501000 "*" 1.12055920098442e-11 2.84500040664577e-11 100
"chr12" 122590001 122591000 "*" 9.5812247025151e-14 3.15667542866807e-13 100
"chr12" 122599001 122600000 "*" 4.86753970463383e-11 1.13145908161915e-10 100
"chr12" 122606001 122607000 "*" 0.000325412457621521 0.000280394175399686
59.2307692307692
"chr12" 122675001 122676000 "*" 0 0 94.0860215053764
"chr12" 122681001 122682000 "*" 9.65729496371637e-10 1.87255912493232e-09 -100
"chr12" 122687001 122688000 "*" 0 0 -100
"chr12" 122944001 122945000 "*" 3.25818826539814e-05 3.29735679006858e-05
-66.6666666666667
"chr12" 123041001 123042000 "*" 2.43005615629954e-09 4.45014222280278e-09 100
"chr12" 123236001 123237000 "*" 1.63746793901964e-12 4.63391019577792e-12
97.9166666666667
"chr12" 123270001 123271000 "*" 0 0 100
"chr12" 123319001 123320000 "*" 4.44089209850063e-16 1.91071758245033e-15 -100
"chr12" 123322001 123323000 "*" 0 0 80
"chr12" 123374001 123375000 "*" 1.2534417948018e-13 4.07063273616362e-13
61.7021276595745
"chr12" 123393001 123394000 "*" 6.59550958292954e-09 1.13456194382773e-08 -100
"chr12" 123435001 123436000 "*" 0 0 70.2127659574468
"chr12" 123450001 123451000 "*" 0 0 100
"chr12" 123451001 123452000 "*" 1.21430643318376e-11 3.06723613975142e-11
64.6153846153846
"chr12" 123458001 123459000 "*" 0 0 -75
"chr12" 123547001 123548000 "*" 1.11022302462516e-16 5.03662826618488e-16
71.2328767123288
"chr12" 123555001 123556000 "*" 1.11022302462516e-16 5.03662826618488e-16
-79.5918367346939
"chr12" 123556001 123557000 "*" 6.0326408313216e-09 1.04608792810228e-08
61.5384615384615
"chr12" 123582001 123583000 "*" 2.62900812231237e-13 8.21294938531841e-13 100
"chr12" 123718001 123719000 "*" 0 0 100
"chr12" 123767001 123768000 "*" 1.67299207820548e-08 2.71377429441055e-08 -100
"chr12" 123783001 123784000 "*" 2.61211192886801e-06 3.12359423291548e-06
-72.2222222222222
"chr12" 123877001 123878000 "*" 2.00227675550835e-08 3.20459176617961e-08 100
"chr12" 124011001 124012000 "*" 0.000416904518971917 0.000352860108725752
53.125
"chr12" 124045001 124046000 "*" 4.45565806472814e-12 1.19345148234248e-11
94.4444444444444
"chr12" 124156001 124157000 "*" 8.88178419700125e-16 3.70670032207938e-15 -100
"chr12" 124223001 124224000 "*" 4.9960036108132e-15 1.9234186548721e-14
97.1311475409836
"chr12" 124313001 124314000 "*" 5.6362137179633e-11 1.29963270300065e-10 100
"chr12" 124354001 124355000 "*" 0 0 92.3076923076923
"chr12" 124360001 124361000 "*" 1.39779633423487e-07 1.98463037958589e-07 100

"chr12" 124378001 124379000 "*" 0 0 100
"chr12" 124401001 124402000 "*" 5.48638023900594e-10 1.10374357336421e-09 100
"chr12" 124410001 124411000 "*" 1.93720595120794e-11 4.76309924836636e-11 -100
"chr12" 124414001 124415000 "*" 1.70280504141473e-08 2.75934340951488e-08
-65.5172413793103
"chr12" 124433001 124434000 "*" 3.33066907387547e-16 1.4495649018245e-15
-52.5423728813559
"chr12" 124441001 124442000 "*" 3.88022947106492e-13 1.1867462770515e-12 100
"chr12" 124457001 124458000 "*" 0 0 -65.625
"chr12" 124514001 124515000 "*" 1.4432899320127e-15 5.90750815956055e-15 -100
"chr12" 124524001 124525000 "*" 2.06349917359905e-05 2.15496010912152e-05
70.7070707070707
"chr12" 124538001 124539000 "*" 4.91828799908944e-14 1.68192819018269e-13 100
"chr12" 124565001 124566000 "*" 2.82773804372027e-13 8.79129664109438e-13 100
"chr12" 124601001 124602000 "*" 0 0 -100
"chr12" 124640001 124641000 "*" 0 0 100
"chr12" 124644001 124645000 "*" 5.53306662154363e-07 7.27639394821453e-07
-76.1904761904762
"chr12" 124683001 124684000 "*" 1.35003119794419e-13 4.3679847476192e-13 100
"chr12" 124692001 124693000 "*" 5.23142215591577e-05 5.12900895932851e-05
-58.8888888888889
"chr12" 124734001 124735000 "*" 0 0 100
"chr12" 124771001 124772000 "*" 0 0 100
"chr12" 124776001 124777000 "*" 1.92946192356658e-09 3.59193011611483e-09 100
"chr12" 124778001 124779000 "*" 0 0 68.8
"chr12" 124796001 124797000 "*" 1.11022302462516e-16 5.03662826618488e-16 100
"chr12" 124884001 124885000 "*" 0.000171200504948343 0.00015450134106212
-50.6493506493507
"chr12" 124919001 124920000 "*" 3.29638145335487e-07 4.46502486012762e-07
79.2452830188679
"chr12" 125026001 125027000 "*" 6.10622663543836e-15 2.32593540648772e-14
-57.3529411764706
"chr12" 125042001 125043000 "*" 1.01030295240889e-14 3.75322807935163e-14
53.3333333333333
"chr12" 125050001 125051000 "*" 0 0 -86.5384615384615
"chr12" 125090001 125091000 "*" 0 0 100
"chr12" 125103001 125104000 "*" 0 0 54.2779156327543
"chr12" 125125001 125126000 "*" 7.7715611723761e-16 3.26213507634405e-15
96.1538461538462
"chr12" 125136001 125137000 "*" 0 0 100
"chr12" 125171001 125172000 "*" 1.55431223447522e-14 5.6388152448458e-14 100
"chr12" 125184001 125185000 "*" 4.4374375285372e-09 7.83647530257679e-09
50.6897910918408
"chr12" 125192001 125193000 "*" 1.50623957750895e-12 4.28756717183481e-12 -100
"chr12" 125199001 125200000 "*" 5.66247740672043e-05 5.52079777294684e-05
51.2820512820513
"chr12" 125230001 125231000 "*" 1.69864122767649e-14 6.13572966736024e-14
-85.8974358974359
"chr12" 125245001 125246000 "*" 2.53841405080113e-05 2.61400314900154e-05
-60.3448275862069
"chr12" 125252001 125253000 "*" 1.97840450022468e-07 2.75860297995596e-07
70.2702702702703

"chr12" 125254001 125255000 "*" 0 0 56.9620253164557
"chr12" 125255001 125256000 "*" 1.66533453693773e-15 6.7629186866784e-15 100
"chr12" 125256001 125257000 "*" 1.97760696707405e-11 4.85442074953215e-11
-58.3333333333333
"chr12" 125294001 125295000 "*" 6.79894552468863e-06 7.63840273607792e-06
59.0909090909091
"chr12" 125295001 125296000 "*" 1.86517468137026e-14 6.70995420767425e-14 -100
"chr12" 125312001 125313000 "*" 1.18804934778893e-07 1.70575925561201e-07
61.5384615384615
"chr12" 125347001 125348000 "*" 0 0 75.8620689655172
"chr12" 125383001 125384000 "*" 0 0 -87.2
"chr12" 125412001 125413000 "*" 0 0 -93.6046511627907
"chr12" 125427001 125428000 "*" 2.88710388929303e-10 6.03399264731139e-10 100
"chr12" 125498001 125499000 "*" 2.09777617943274e-07 2.91559310498972e-07 -60
"chr12" 125563001 125564000 "*" 5.21804821573824e-14 1.7777322631293e-13 -73.75
"chr12" 125571001 125572000 "*" 1.97583605032747e-08 3.17635466557112e-08
61.3333333333333
"chr12" 125579001 125580000 "*" 0 0 -61.8181818181818
"chr12" 125583001 125584000 "*" 4.08209022140227e-11 9.57770690255255e-11 100
"chr12" 125602001 125603000 "*" 2.00227675550835e-08 3.20459176617961e-08 100
"chr12" 125613001 125614000 "*" 9.69224700497762e-14 3.18871856180509e-13
83.3333333333333
"chr12" 125646001 125647000 "*" 5.94302385081846e-13 1.77716863128645e-12
53.125
"chr12" 125661001 125662000 "*" 0 0 100
"chr12" 125668001 125669000 "*" 0 0 90
"chr12" 125675001 125676000 "*" 0.000654030243051218 0.000535279413851719
-55.1515151515151
"chr12" 125691001 125692000 "*" 1.48087875295744e-10 3.22075573380618e-10 -100
"chr12" 125714001 125715000 "*" 0 0 55.5238095238095
"chr12" 125724001 125725000 "*" 7.105427357601e-14 2.38163167708172e-13
-62.4708624708625
"chr12" 125781001 125782000 "*" 4.32986979603811e-15 1.68006959513174e-14
63.2183908045977
"chr12" 125816001 125817000 "*" 1.90181204118289e-13 6.05298617539364e-13 100
"chr12" 125866001 125867000 "*" 6.4324989779152e-11 1.47230020825092e-10 -100
"chr12" 125896001 125897000 "*" 6.92287338566189e-11 1.57605630124247e-10 100
"chr12" 125977001 125978000 "*" 0 0 -100
"chr12" 126010001 126011000 "*" 0 0 -100
"chr12" 126018001 126019000 "*" 0 0 100
"chr12" 126049001 126050000 "*" 1.53210777398272e-14 5.5628583449216e-14 100
"chr12" 126169001 126170000 "*" 0 0 -57.1146953405018
"chr12" 126391001 126392000 "*" 7.0006535457523e-08 1.03695832512054e-07 100
"chr12" 126515001 126516000 "*" 6.4152538836737e-10 1.27477697728417e-09 -100
"chr12" 126637001 126638000 "*" 3.34128893442198e-08 5.19772864618523e-08 -100
"chr12" 126789001 126790000 "*" 0 0 -65.8602150537634
"chr12" 126942001 126943000 "*" 8.71525074330748e-14 2.88848349331391e-13 100
"chr12" 127385001 127386000 "*" 0 0 100
"chr12" 127421001 127422000 "*" 2.44946890037845e-06 2.94150378099288e-06
-66.6666666666667
"chr12" 127519001 127520000 "*" 7.0006535457523e-08 1.03695832512054e-07 100
"chr12" 127638001 127639000 "*" 6.49103928385131e-08 9.69861335645993e-08

58.3333333333333
"chr12" 127779001 127780000 "*" 9.9077693768157e-06 1.08644157860742e-05
52.6315789473684
"chr12" 127789001 127790000 "*" 0 0 -100
"chr12" 127856001 127857000 "*" 7.0006535457523e-08 1.03695832512054e-07 -100
"chr12" 127865001 127866000 "*" 0 0 96.4601769911504
"chr12" 127966001 127967000 "*" 3.47832873615062e-13 1.07168732086404e-12
61.5384615384615
"chr12" 128055001 128056000 "*" 8.7349629751543e-09 1.47505871898511e-08 -100
"chr12" 128081001 128082000 "*" 1.92946192356658e-09 3.59193011611483e-09 -100
"chr12" 128095001 128096000 "*" 3.33066907387547e-16 1.4495649018245e-15 100
"chr12" 128291001 128292000 "*" 2.62900812231237e-13 8.21294938531841e-13 -100
"chr12" 128318001 128319000 "*" 0 0 -69.8275862068966
"chr12" 128461001 128462000 "*" 0 0 100
"chr12" 128544001 128545000 "*" 0 0 -73.3333333333333
"chr12" 128552001 128553000 "*" 6.55384191361463e-10 1.30082815732091e-09
-96.1538461538462
"chr12" 128594001 128595000 "*" 2.52020626589911e-14 8.9164280780292e-14
66.1290322580645
"chr12" 128673001 128674000 "*" 0 0 52.309705561614
"chr12" 128690001 128691000 "*" 0 0 100
"chr12" 128773001 128774000 "*" 1.87392993900026e-07 2.62077108671233e-07
72.9166666666667
"chr12" 128850001 128851000 "*" 0 0 -55.6390977443609
"chr12" 128875001 128876000 "*" 0 0 -100
"chr12" 128887001 128888000 "*" 5.39866832600655e-05 5.2808752078579e-05
57.5757575757576
"chr12" 128960001 128961000 "*" 1.80989033669032e-05 1.90667975357288e-05 68.75
"chr12" 129079001 129080000 "*" 0 0 -73.8738738738739
"chr12" 129085001 129086000 "*" 5.44009282066327e-15 2.08486137681812e-14
-92.5925925925926
"chr12" 129112001 129113000 "*" 1.80989033669032e-05 1.90667975357288e-05 68.75
"chr12" 129144001 129145000 "*" 7.0006535457523e-08 1.03695832512054e-07 -100
"chr12" 129167001 129168000 "*" 6.95837509834263e-09 1.1939049806171e-08
-70.2702702702703
"chr12" 129232001 129233000 "*" 6.65796640042515e-10 1.32039529699025e-09
71.5909090909091
"chr12" 129332001 129333000 "*" 0.000134107235349457 0.000123177464126781
-56.6666666666667
"chr12" 129393001 129394000 "*" 4.2632564145606e-14 1.46882536594739e-13
56.0975609756098
"chr12" 129416001 129417000 "*" 1.92956761679852e-13 6.13874820107395e-13
-81.25
"chr12" 129455001 129456000 "*" 7.3404803079824e-05 7.02790923864156e-05
58.3333333333333
"chr12" 129477001 129478000 "*" 4.73234496034536e-09 8.30627300264826e-09 100
"chr12" 129509001 129510000 "*" 8.10196857639056e-05 7.70782322467687e-05
-51.4726507713885
"chr12" 129513001 129514000 "*" 1.98365768255826e-11 4.85612402473442e-11 -100
"chr12" 129528001 129529000 "*" 0 0 100
"chr12" 129563001 129564000 "*" 2.08814465718632e-09 3.86232567960463e-09 100
"chr12" 129615001 129616000 "*" 0 0 -100

"chr12" 129634001 129635000 "*" 5.80383285431196e-07 7.61326389445009e-07 51.4705882352941
"chr12" 129786001 129787000 "*" 6.59484211684713e-09 1.13456194382773e-08 76.4705882352941
"chr12" 129852001 129853000 "*" 0 0 -100
"chr12" 129981001 129982000 "*" 4.46405508218728e-05 4.42311628182131e-05 -52.3809523809524
"chr12" 130070001 130071000 "*" 2.80853118539426e-10 5.89385180219943e-10 87.5
"chr12" 130145001 130146000 "*" 0 0 100
"chr12" 130268001 130269000 "*" 2.17381668221606e-13 6.85151076607736e-13 -100
"chr12" 130324001 130325000 "*" 1.10074171999486e-11 2.79911658478501e-11 -66.3157894736842
"chr12" 130340001 130341000 "*" 0 0 81.8181818181818
"chr12" 130420001 130421000 "*" 0 0 -85.1351351351351
"chr12" 130427001 130428000 "*" 0 0 -68.7203371035706
"chr12" 130430001 130431000 "*" 5.794809077031e-12 1.52911192757929e-11 100
"chr12" 130499001 130500000 "*" 3.34128893442198e-08 5.19772864618523e-08 100
"chr12" 130503001 130504000 "*" 1.37828859436695e-10 3.01445544267661e-10 100
"chr12" 130513001 130514000 "*" 7.38587191406737e-10 1.4565780689575e-09 62.2222222222222
"chr12" 130529001 130530000 "*" 0 0 -100
"chr12" 130548001 130549000 "*" 4.44089209850063e-16 1.91071758245033e-15 -100
"chr12" 130557001 130558000 "*" 7.7715611723761e-16 3.26213507634405e-15 -100
"chr12" 130577001 130578000 "*" 0 0 -100
"chr12" 130589001 130590000 "*" 1.31228361510694e-13 4.25443607859404e-13 -57.1428571428571
"chr12" 130594001 130595000 "*" 2.30168661907726e-10 4.88677430469091e-10 86.6666666666667
"chr12" 130607001 130608000 "*" 1.67299207820548e-08 2.71377429441055e-08 100
"chr12" 130617001 130618000 "*" 0 0 -71.09375
"chr12" 130619001 130620000 "*" 2.22044604925031e-16 9.81641919380259e-16 -100
"chr12" 130630001 130631000 "*" 7.78672028745042e-07 1.00356652327738e-06 73.2142857142857
"chr12" 130646001 130647000 "*" 0 0 51.3732997481108
"chr12" 130659001 130660000 "*" 4.52970994047064e-12 1.21219626538044e-11 70.5882352941177
"chr12" 130660001 130661000 "*" 1.49880108324396e-14 5.44951848293151e-14 100
"chr12" 130683001 130684000 "*" 0 0 52.7674453206368
"chr12" 130719001 130720000 "*" 3.63445940010365e-11 8.58565209111204e-11 100
"chr12" 130792001 130793000 "*" 2.69018141096922e-12 7.41487347763769e-12 -100
"chr12" 130819001 130820000 "*" 1.77635683940025e-15 7.19870740878856e-15 -57.4468085106383
"chr12" 130821001 130822000 "*" 0 0 -57.8125
"chr12" 130842001 130843000 "*" 0.000184006388664448 0.000165249884973406 -54.1666666666667
"chr12" 130893001 130894000 "*" 1.4432899320127e-14 5.25937808621513e-14 63.8888888888889
"chr12" 130899001 130900000 "*" 4.02167188440217e-12 1.08266700497926e-11 100
"chr12" 130919001 130920000 "*" 0 0 -75.8748778103617
"chr12" 130938001 130939000 "*" 8.44449166148564e-09 1.43279588664747e-08 57.1428571428571
"chr12" 130949001 130950000 "*" 0 0 -55.2238805970149

"chr12" 130978001 130979000 "*" 4.99020824662466e-11 1.15790792586157e-10 100
"chr12" 130981001 130982000 "*" 6.60865823065571e-07 8.60163088894937e-07
-50.3663003663004
"chr12" 130984001 130985000 "*" 8.58968451922237e-12 2.213554983903e-11 -100
"chr12" 130986001 130987000 "*" 0 0 -100
"chr12" 130994001 130995000 "*" 3.05157635864806e-05 3.10468679297701e-05
-59.6491228070175
"chr12" 131007001 131008000 "*" 0 0 80
"chr12" 131010001 131011000 "*" 3.62924807872922e-07 4.88693057852253e-07
-58.9285714285714
"chr12" 131041001 131042000 "*" 2.22044604925031e-16 9.81641919380259e-16 -100
"chr12" 131059001 131060000 "*" 7.16197878780633e-10 1.41503369322759e-09 -85
"chr12" 131066001 131067000 "*" 0 0 -100
"chr12" 131075001 131076000 "*" 1.20591092667155e-10 2.66075324129686e-10 -100
"chr12" 131092001 131093000 "*" 7.0006535457523e-08 1.03695832512054e-07 -100
"chr12" 131118001 131119000 "*" 1.99944172329936e-10 4.2888514511833e-10
-69.6969696969697
"chr12" 131128001 131129000 "*" 7.1886979419733e-05 6.89454132022215e-05
-55.0724637681159
"chr12" 131131001 131132000 "*" 6.32827124036339e-15 2.40666772484995e-14
-77.1929824561404
"chr12" 131140001 131141000 "*" 4.85008477824067e-10 9.84186011842796e-10
71.830985915493
"chr12" 131144001 131145000 "*" 0 0 -100
"chr12" 131154001 131155000 "*" 0.000451115314036077 0.000379477851858741
52.1739130434783
"chr12" 131161001 131162000 "*" 8.71525074330748e-14 2.88848349331391e-13 100
"chr12" 131163001 131164000 "*" 1.10442103432584e-05 1.20217549185931e-05
-57.6923076923077
"chr12" 131186001 131187000 "*" 4.07009934355607e-06 4.72553174708673e-06
58.8235294117647
"chr12" 131190001 131191000 "*" 0 0 96
"chr12" 131196001 131197000 "*" 7.95908594686257e-06 8.85270264440226e-06
-71.4285714285714
"chr12" 131221001 131222000 "*" 1.0547118733939e-14 3.91001935116974e-14
64.4444444444444
"chr12" 131243001 131244000 "*" 0 0 -76.7441860465116
"chr12" 131245001 131246000 "*" 2.62811994389267e-12 7.25760852424122e-12
59.2532467532468
"chr12" 131310001 131311000 "*" 0 0 100
"chr12" 131324001 131325000 "*" 8.34186836873307e-06 9.24761438950058e-06
-60.6060606060606
"chr12" 131338001 131339000 "*" 1.12458486967171e-09 2.15740815043925e-09 100
"chr12" 131348001 131349000 "*" 8.57092175010621e-13 2.51149198099779e-12 100
"chr12" 131351001 131352000 "*" 9.30051856173897e-06 1.02447332143295e-05 56.25
"chr12" 131355001 131356000 "*" 4.88498130835069e-15 1.8824139250584e-14
-59.7147950089127
"chr12" 131372001 131373000 "*" 7.30193934072032e-05 6.99373915024258e-05
54.7945205479452
"chr12" 131408001 131409000 "*" 3.5527136788005e-15 1.39277426410519e-14 -100
"chr12" 131434001 131435000 "*" 5.61306556789987e-12 1.48514825195728e-11
-67.8899082568807

"chr12" 131441001 131442000 "*" 0 0 -50.1532010965973
"chr12" 131451001 131452000 "*" 0 0 -99.6282527881041
"chr12" 131468001 131469000 "*" 4.54129254400248e-05 4.49481935503239e-05 56.9444444444444
"chr12" 131471001 131472000 "*" 0.000499129428774947 0.000416865202314435 52.3809523809524
"chr12" 131490001 131491000 "*" 0 0 52.6717557251908
"chr12" 131494001 131495000 "*" 2.26488587894424e-05 2.35038252445516e-05 -64.2857142857143
"chr12" 131498001 131499000 "*" 0 0 -100
"chr12" 131501001 131502000 "*" 1.10246192175367e-08 1.83859090677293e-08 -80
"chr12" 131502001 131503000 "*" 0 0 83.6734693877551
"chr12" 131506001 131507000 "*" 1.91175019359502e-06 2.33265021579581e-06 -68
"chr12" 131509001 131510000 "*" 5.55111512312578e-16 2.36485094870365e-15 -73.6842105263158
"chr12" 131520001 131521000 "*" 5.7065463465733e-14 1.93318145576218e-13 100
"chr12" 131544001 131545000 "*" 1.11022302462516e-16 5.03662826618488e-16 92.3076923076923
"chr12" 131571001 131572000 "*" 3.33066907387547e-16 1.4495649018245e-15 58.5106382978723
"chr12" 131578001 131579000 "*" 0 0 84.7222222222222
"chr12" 131579001 131580000 "*" 0 0 -100
"chr12" 131581001 131582000 "*" 1.66533453693773e-15 6.7629186866784e-15 -70
"chr12" 131617001 131618000 "*" 1.22124532708767e-15 5.03921984217778e-15 100
"chr12" 131625001 131626000 "*" 1.29037891483108e-11 3.24582280286866e-11 100
"chr12" 131692001 131693000 "*" 0 0 63.4920634920635
"chr12" 131695001 131696000 "*" 0 0 100
"chr12" 131742001 131743000 "*" 5.31904520428839e-11 1.23064908938601e-10 66.6666666666667
"chr12" 131757001 131758000 "*" 2.81963186465894e-07 3.8544380054175e-07 69.2307692307692
"chr12" 131758001 131759000 "*" 2.36801344088988e-08 3.75542359436053e-08 -67.741935483871
"chr12" 131772001 131773000 "*" 0 0 64.8241206030151
"chr12" 131775001 131776000 "*" 2.23086262651773e-06 2.69712099930746e-06 61.4035087719298
"chr12" 131852001 131853000 "*" 1.16155443002919e-05 1.26002437563056e-05 66.6666666666667
"chr12" 131855001 131856000 "*" 0 0 73.469387755102
"chr12" 131876001 131877000 "*" 3.64270735797589e-09 6.5252880340617e-09 -66.6666666666667
"chr12" 131880001 131881000 "*" 2.25259898423147e-06 2.72138877153708e-06 -63.7931034482759
"chr12" 131889001 131890000 "*" 1.39779633423487e-07 1.98463037958589e-07 -100
"chr12" 131896001 131897000 "*" 7.94356751088188e-06 8.83803450951692e-06 -69.5652173913043
"chr12" 131920001 131921000 "*" 2.33028818463765e-10 4.93719283454501e-10 100
"chr12" 131922001 131923000 "*" 7.0006535457523e-08 1.03695832512054e-07 100
"chr12" 131977001 131978000 "*" 1.02162887645196e-05 1.11796468287273e-05 65.3061224489796
"chr12" 131998001 131999000 "*" 2.11424531426641e-06 2.56432546771576e-06 54.5454545454545

"chr12" 132021001 132022000 "*" 2.15125472990962e-10 4.58126659943874e-10 100
"chr12" 132024001 132025000 "*" 2.22044604925031e-16 9.81641919380259e-16 100
"chr12" 132036001 132037000 "*" 1.93720595120794e-11 4.76309924836636e-11 100
"chr12" 132042001 132043000 "*" 8.19373111804467e-07 1.05252749372156e-06
51.3513513513514
"chr12" 132050001 132051000 "*" 3.76933895207543e-09 6.74300198876295e-09
-55.8823529411765
"chr12" 132051001 132052000 "*" 0 0 -72.3404255319149
"chr12" 132055001 132056000 "*" 1.52047929802279e-10 3.30231857247646e-10
57.1428571428571
"chr12" 132057001 132058000 "*" 1.11022302462516e-16 5.03662826618488e-16
94.4444444444444
"chr12" 132058001 132059000 "*" 0 0 -56.7796610169492
"chr12" 132071001 132072000 "*" 5.03264097062583e-13 1.51942428521737e-12 100
"chr12" 132083001 132084000 "*" 4.76815253946938e-11 1.11123692239864e-10 -70
"chr12" 132089001 132090000 "*" 8.52140580320793e-12 2.19817259951489e-11 100
"chr12" 132097001 132098000 "*" 0 0 62.5862068965517
"chr12" 132105001 132106000 "*" 6.01276806122542e-11 1.38263819521722e-10
52.9505582137161
"chr12" 132109001 132110000 "*" 1.70776726093891e-11 4.23416908925427e-11 74
"chr12" 132120001 132121000 "*" 0 0 100
"chr12" 132157001 132158000 "*" 1.36051803156789e-09 2.58646346187833e-09
82.9347826086957
"chr12" 132160001 132161000 "*" 1.24691762692919e-07 1.7860064772304e-07
54.6218487394958
"chr12" 132161001 132162000 "*" 1.75068215479968e-09 3.27732513036079e-09
60.7142857142857
"chr12" 132167001 132168000 "*" 6.53033183084517e-13 1.93971873401198e-12
-70.4819277108434
"chr12" 132176001 132177000 "*" 1.69482203856708e-05 1.79420595392413e-05
-66.6666666666667
"chr12" 132177001 132178000 "*" 2.45921890320577e-05 2.53771384328844e-05
61.3445378151261
"chr12" 132186001 132187000 "*" 1.37828859436695e-10 3.01445544267661e-10 100
"chr12" 132264001 132265000 "*" 0 0 100
"chr12" 132288001 132289000 "*" 3.05637626318855e-10 6.37020893543828e-10
-66.6666666666667
"chr12" 132297001 132298000 "*" 2.88779000712225e-11 6.92935809466068e-11 -100
"chr12" 132312001 132313000 "*" 0 0 57.5474049675065
"chr12" 132331001 132332000 "*" 0.000182056957508503 0.000163624466431919
-56.7567567567568
"chr12" 132366001 132367000 "*" 6.83719338923083e-08 1.01840744031014e-07
66.6666666666667
"chr12" 132367001 132368000 "*" 5.61876586454568e-05 5.48048639509562e-05
53.3333333333333
"chr12" 132371001 132372000 "*" 2.69084754478399e-12 7.41487347763769e-12 100
"chr12" 132433001 132434000 "*" 5.72471026027976e-07 7.51633057935261e-07 78
"chr12" 132470001 132471000 "*" 1.07315135122743e-06 1.35672165178098e-06
52.9411764705882
"chr12" 132535001 132536000 "*" 9.67229923154456e-07 1.22970859507725e-06
-63.3333333333333
"chr12" 132667001 132668000 "*" 8.70081784398735e-13 2.54812231334686e-12

-86.1111111111111
"chr12" 132677001 132678000 "*" 0 0 54.3193690037668
"chr12" 132701001 132702000 "*" 0 0 82.4324324324324
"chr12" 132833001 132834000 "*" 3.67868684835182e-05 3.69217322449025e-05
59.8214285714286
"chr12" 132904001 132905000 "*" 0 0 -79.3388429752066
"chr12" 132912001 132913000 "*" 0 0 68.3271066832711
"chr12" 132925001 132926000 "*" 1.11022302462516e-16 5.03662826618488e-16
86.046511627907
"chr12" 132953001 132954000 "*" 0 0 100
"chr12" 133022001 133023000 "*" 0 0 -62.1738621738622
"chr12" 133064001 133065000 "*" 0 0 50.1154401154401
"chr12" 133174001 133175000 "*" 0 0 62.8053853717571
"chr12" 133213001 133214000 "*" 0 0 100
"chr12" 133288001 133289000 "*" 1.89626092605977e-13 6.04131266215094e-13 -65
"chr12" 133306001 133307000 "*" 1.75928160928152e-11 4.35257527084094e-11
56.5217391304348
"chr12" 133415001 133416000 "*" 0 0 -100
"chr12" 133427001 133428000 "*" 0 0 -67.175572519084
"chr12" 133448001 133449000 "*" 1.37390099297363e-12 3.93044627933178e-12
-64.5161290322581
"chr12" 133463001 133464000 "*" 0 0 54.9702772537768
"chr12" 133473001 133474000 "*" 8.80406858527749e-14 2.91456141063452e-13 -100
"chr12" 133706001 133707000 "*" 0 0 78.3603918412219
"chr12" 133758001 133759000 "*" 0 0 100
"chr13" 19173001 19174000 "*" 2.37387887125351e-12 6.59108969918702e-12
91.0569105691057
"chr13" 19524001 19525000 "*" 9.2148511043888e-15 3.43705279328267e-14
-86.8421052631579
"chr13" 19526001 19527000 "*" 4.52265242856242e-06 5.21603543073849e-06
-66.6666666666667
"chr13" 19528001 19529000 "*" 4.94337903944597e-12 1.31719251525618e-11
-97.6744186046512
"chr13" 19623001 19624000 "*" 1.35729887329461e-08 2.23194274279259e-08
63.1550802139037
"chr13" 19642001 19643000 "*" 7.0006535457523e-08 1.03695832512054e-07 -100
"chr13" 19659001 19660000 "*" 1.29936490345095e-07 1.85720545956764e-07
60.8695652173913
"chr13" 19660001 19661000 "*" 6.4152538836737e-10 1.27477697728417e-09
66.6666666666667
"chr13" 19690001 19691000 "*" 2.68450373042128e-10 5.64465213127486e-10 100
"chr13" 19729001 19730000 "*" 0 0 73.469387755102
"chr13" 19733001 19734000 "*" 0 0 100
"chr13" 19879001 19880000 "*" 0 0 66.0700969425802
"chr13" 19938001 19939000 "*" 0 0 -100
"chr13" 19939001 19940000 "*" 3.10862446895044e-15 1.22466437093774e-14 -100
"chr13" 20235001 20236000 "*" 0 0 98.6666666666667
"chr13" 20438001 20439000 "*" 0 0 -53.1617561430069
"chr13" 20687001 20688000 "*" 2.84328116606503e-13 8.83797215648449e-13
60.9756097560976
"chr13" 20734001 20735000 "*" 0 0 59.4594594594595
"chr13" 20743001 20744000 "*" 1.67377583903949e-07 2.35442859751953e-07 -90

"chr13" 20766001 20767000 "*" 0 0 63.0544628026925
"chr13" 20778001 20779000 "*" 5.55111512312578e-16 2.36485094870365e-15 -100
"chr13" 20797001 20798000 "*" 0 0 71.2765957446808
"chr13" 20814001 20815000 "*" 0 0 100
"chr13" 20832001 20833000 "*" 1.11355369369903e-13 3.64429266079421e-13
96.9230769230769
"chr13" 20842001 20843000 "*" 4.56037858279634e-05 4.51247837423645e-05
69.2307692307692
"chr13" 20872001 20873000 "*" 6.93112234273485e-13 2.05218842007357e-12 100
"chr13" 20874001 20875000 "*" 2.77688982919244e-12 7.63054091010879e-12
68.4863523573201
"chr13" 20918001 20919000 "*" 0 0 85.1851851851852
"chr13" 20924001 20925000 "*" 2.68451927354363e-13 8.38071414718782e-13
-56.2280701754386
"chr13" 20973001 20974000 "*" 9.08108033215171e-11 2.03407753381119e-10 100
"chr13" 21001001 21002000 "*" 3.99680288865056e-15 1.5566998858822e-14 100
"chr13" 21069001 21070000 "*" 1.67299207820548e-08 2.71377429441055e-08 100
"chr13" 21278001 21279000 "*" 0 0 -100
"chr13" 21494001 21495000 "*" 1.51323398256409e-13 4.86995056237989e-13 100
"chr13" 21654001 21655000 "*" 5.99520433297585e-15 2.28531569014092e-14 100
"chr13" 21827001 21828000 "*" 2.8421709430404e-14 9.99616402633955e-14 100
"chr13" 21904001 21905000 "*" 3.04324343503026e-11 7.26599935673121e-11 100
"chr13" 22047001 22048000 "*" 1.35036426485158e-12 3.86629547363774e-12 -100
"chr13" 22057001 22058000 "*" 0 0 85.7142857142857
"chr13" 22196001 22197000 "*" 1.54630752646767e-11 3.85079502648228e-11 100
"chr13" 22246001 22247000 "*" 3.6700841921089e-08 5.65530226072258e-08 100
"chr13" 22295001 22296000 "*" 6.7390537594747e-14 2.26447131529591e-13 100
"chr13" 22301001 22302000 "*" 4.01313771103418e-09 7.11882087237587e-09 -100
"chr13" 22304001 22305000 "*" 7.105427357601e-15 2.68362283258525e-14 100
"chr13" 22311001 22312000 "*" 6.59550958292954e-09 1.13456194382773e-08 -100
"chr13" 22341001 22342000 "*" 3.95353005888666e-09 7.03928529283733e-09 100
"chr13" 22357001 22358000 "*" 3.68371999570627e-13 1.13149976091305e-12 -100
"chr13" 22452001 22453000 "*" 1.96509475358653e-13 6.23868565133654e-13 100
"chr13" 22480001 22481000 "*" 1.29037891483108e-11 3.24582280286866e-11 -100
"chr13" 22487001 22488000 "*" 1.73701222061506e-06 2.13292170979784e-06 62.5
"chr13" 22504001 22505000 "*" 1.5277158427196e-09 2.87642564232045e-09 -100
"chr13" 22509001 22510000 "*" 5.6621374255883e-15 2.16468580166064e-14 -100
"chr13" 22522001 22523000 "*" 0 0 -100
"chr13" 22573001 22574000 "*" 0 0 100
"chr13" 22574001 22575000 "*" 0 0 -100
"chr13" 22582001 22583000 "*" 5.01025332333427e-10 1.01358031135889e-09 -100
"chr13" 22620001 22621000 "*" 6.37490060739765e-13 1.89496793587877e-12 100
"chr13" 22621001 22622000 "*" 8.53472802708755e-06 9.44560983446889e-06 -60
"chr13" 22627001 22628000 "*" 2.38575468822777e-05 2.46816531377115e-05
-66.6666666666667
"chr13" 22679001 22680000 "*" 3.38202243987951e-10 7.01073816650772e-10
92.8571428571429
"chr13" 22814001 22815000 "*" 0 0 98
"chr13" 22860001 22861000 "*" 7.0006535457523e-08 1.03695832512054e-07 100
"chr13" 23030001 23031000 "*" 0.000792090690089564 0.000639295093468792
52.3809523809524
"chr13" 23126001 23127000 "*" 1.07864655669587e-05 1.17574627643031e-05

-63.8888888888889
"chr13" 23253001 23254000 "*" 1.39779633423487e-07 1.98463037958589e-07 100
"chr13" 23319001 23320000 "*" 1.60982338570648e-14 5.83411082284088e-14
-76.5957446808511
"chr13" 23336001 23337000 "*" 9.65338919911574e-13 2.80678910656113e-12 -100
"chr13" 23404001 23405000 "*" 3.33066907387547e-16 1.4495649018245e-15 100
"chr13" 23409001 23410000 "*" 0 0 -100
"chr13" 23511001 23512000 "*" 2.4463169601141e-08 3.87275614977233e-08
-78.3783783783784
"chr13" 23514001 23515000 "*" 7.16689364521628e-08 1.06003852859476e-07
52.3809523809524
"chr13" 23794001 23795000 "*" 1.34559030584569e-13 4.35581312463895e-13 100
"chr13" 23817001 23818000 "*" 2.22044604925031e-16 9.81641919380259e-16 100
"chr13" 23981001 23982000 "*" 3.63763963395769e-09 6.51810504554469e-09
-82.9787234042553
"chr13" 24027001 24028000 "*" 0.000126118798739694 0.000116368097213291
-51.7241379310345
"chr13" 24134001 24135000 "*" 7.12914707268197e-07 9.23469497775327e-07
71.4285714285714
"chr13" 24153001 24154000 "*" 0 0 75.5708408259613
"chr13" 24227001 24228000 "*" 1.5746981163467e-08 2.57012701657925e-08
73.4939759036145
"chr13" 24512001 24513000 "*" 7.03992419914812e-12 1.83313429961219e-11 -100
"chr13" 24588001 24589000 "*" 0 0 -100
"chr13" 24626001 24627000 "*" 8.65973959207622e-15 3.24130830964561e-14 -100
"chr13" 24633001 24634000 "*" 4.79616346638068e-14 1.64380445416596e-13 100
"chr13" 24645001 24646000 "*" 8.96610840017686e-08 1.30861118541309e-07 -75
"chr13" 24704001 24705000 "*" 1.46549439250521e-14 5.33330076973935e-14 -100
"chr13" 24724001 24725000 "*" 9.97657512158412e-12 2.55278062990321e-11
63.6363636363636
"chr13" 24758001 24759000 "*" 0 0 100
"chr13" 24773001 24774000 "*" 5.44009282066327e-15 2.08486137681812e-14 100
"chr13" 24865001 24866000 "*" 1.98365768255826e-11 4.85612402473442e-11 100
"chr13" 24877001 24878000 "*" 3.86896332082998e-07 5.18885661575616e-07 -60
"chr13" 24942001 24943000 "*" 1.87405646556726e-13 5.97285292681429e-13 100
"chr13" 25259001 25260000 "*" 3.1835571678851e-07 4.31991940783722e-07
54.0540540540541
"chr13" 25317001 25318000 "*" 2.08814465718632e-09 3.86232567960463e-09 100
"chr13" 25318001 25319000 "*" 1.38590361409285e-10 3.02706178892116e-10 -100
"chr13" 25556001 25557000 "*" 1.67521431504447e-07 2.35637255360457e-07
52.485180118559
"chr13" 25561001 25562000 "*" 1.40321088082374e-12 4.01085631147308e-12
-53.3333333333333
"chr13" 25630001 25631000 "*" 1.51656465163796e-13 4.87803420672651e-13 100
"chr13" 25671001 25672000 "*" 0 0 81.8181818181818
"chr13" 25689001 25690000 "*" 2.33146835171283e-15 9.32818831833246e-15 100
"chr13" 25723001 25724000 "*" 9.04570973681018e-10 1.76414389822173e-09 100
"chr13" 25726001 25727000 "*" 0 0 53.030303030303
"chr13" 25743001 25744000 "*" 2.01283434364541e-13 6.38489986096795e-13
63.6363636363636
"chr13" 25867001 25868000 "*" 0 0 100
"chr13" 25931001 25932000 "*" 1.13625101549886e-08 1.88562811654589e-08

55.9322033898305
"chr13" 25945001 25946000 "*" 1.91089366552433e-11 4.70965050798509e-11
-94.1860465116279
"chr13" 26049001 26050000 "*" 2.00227675550835e-08 3.20459176617961e-08 -100
"chr13" 26093001 26094000 "*" 1.45446377164404e-09 2.75539006615226e-09
-77.2727272727273
"chr13" 26262001 26263000 "*" 4.01313771103418e-09 7.11882087237587e-09 -100
"chr13" 26394001 26395000 "*" 2.28663749046731e-07 3.16284944821387e-07 -75
"chr13" 26436001 26437000 "*" 4.73234496034536e-09 8.30627300264826e-09 100
"chr13" 26444001 26445000 "*" 0 0 -68.3933676386507
"chr13" 26489001 26490000 "*" 5.36237720893951e-14 1.82370018611088e-13
-65.8536585365854
"chr13" 26523001 26524000 "*" 8.88178419700125e-16 3.70670032207938e-15 100
"chr13" 26542001 26543000 "*" 0 0 87.5
"chr13" 26545001 26546000 "*" 9.63829016598083e-12 2.46993429847588e-11 -100
"chr13" 26553001 26554000 "*" 7.22470026470745e-06 8.0885042751598e-06
67.0588235294118
"chr13" 26595001 26596000 "*" 1.92946192356658e-09 3.59193011611483e-09 100
"chr13" 26981001 26982000 "*" 0 0 -100
"chr13" 27000001 27001000 "*" 6.24654217240561e-10 1.2462108191303e-09 -100
"chr13" 27036001 27037000 "*" 1.5277158427196e-09 2.87642564232045e-09 -100
"chr13" 27048001 27049000 "*" 1.11022302462516e-16 5.03662826618488e-16
-57.3148148148148
"chr13" 27054001 27055000 "*" 2.33892193368668e-06 2.81787965947533e-06
59.2592592592593
"chr13" 27060001 27061000 "*" 1.01952880582346e-07 1.47797557814672e-07 -65.625
"chr13" 27070001 27071000 "*" 2.22044604925031e-16 9.81641919380259e-16 -100
"chr13" 27072001 27073000 "*" 5.09996640252197e-07 6.73329253665808e-07
-56.9230769230769
"chr13" 27256001 27257000 "*" 0 0 -54.3438258008755
"chr13" 27259001 27260000 "*" 0 0 -50.6850998463902
"chr13" 27296001 27297000 "*" 3.17155016827941e-05 3.21795553318075e-05
59.3220338983051
"chr13" 27307001 27308000 "*" 0 0 100
"chr13" 27312001 27313000 "*" 4.14335232790108e-13 1.26153063645578e-12 100
"chr13" 27360001 27361000 "*" 0 0 90.5982905982906
"chr13" 27366001 27367000 "*" 2.38353745185549e-07 3.28978743080408e-07
-58.3333333333333
"chr13" 27371001 27372000 "*" 1.11022302462516e-16 5.03662826618488e-16
64.5161290322581
"chr13" 27427001 27428000 "*" 4.08209022140227e-11 9.57770690255255e-11 -100
"chr13" 27457001 27458000 "*" 0 0 83.3333333333333
"chr13" 27472001 27473000 "*" 0.00011867343116434 0.00010998140802395
53.8461538461538
"chr13" 27525001 27526000 "*" 0 0 -66.6666666666667
"chr13" 27550001 27551000 "*" 4.41633042761458e-05 4.38007677054066e-05 60
"chr13" 27627001 27628000 "*" 9.32032229172819e-13 2.7181920310254e-12
58.3333333333333
"chr13" 27854001 27855000 "*" 2.62900812231237e-13 8.21294938531841e-13 -100
"chr13" 27910001 27911000 "*" 0 0 100
"chr13" 27911001 27912000 "*" 0 0 -100
"chr13" 27926001 27927000 "*" 2.43173259306673e-11 5.903653046479e-11

59.2592592592593
"chr13" 27936001 27937000 "*" 0 0 90.4411764705882
"chr13" 27947001 27948000 "*" 1.14352971536391e-14 4.21921674335999e-14 100
"chr13" 27956001 27957000 "*" 5.78353024927036e-06 6.56902676498958e-06
68.2926829268293
"chr13" 27957001 27958000 "*" 0 0 79.5918367346939
"chr13" 27960001 27961000 "*" 3.05311331771918e-12 8.34639368772922e-12 100
"chr13" 27999001 28000000 "*" 0 0 -86.8367346938775
"chr13" 28051001 28052000 "*" 8.7349629751543e-09 1.47505871898511e-08 -100
"chr13" 28103001 28104000 "*" 3.95353005888666e-09 7.03928529283733e-09 -100
"chr13" 28193001 28194000 "*" 7.0006535457523e-08 1.03695832512054e-07 -100
"chr13" 28304001 28305000 "*" 2.15125472990962e-10 4.58126659943874e-10 100
"chr13" 28335001 28336000 "*" 3.90465437760668e-13 1.19307893943277e-12 -100
"chr13" 28363001 28364000 "*" 0 0 85.0600679028467
"chr13" 28376001 28377000 "*" 0 0 100
"chr13" 28384001 28385000 "*" 3.59625337398306e-07 4.84570954293995e-07
68.9922480620155
"chr13" 28399001 28400000 "*" 1.11475197961397e-08 1.85774986608361e-08
60.4166666666667
"chr13" 28477001 28478000 "*" 0 0 100
"chr13" 28504001 28505000 "*" 1.70641278884887e-12 4.81986032810646e-12 100
"chr13" 28510001 28511000 "*" 7.13039608468691e-08 1.05488346247008e-07
57.6923076923077
"chr13" 28546001 28547000 "*" 3.98459845118992e-07 5.33450443003747e-07
62.2222222222222
"chr13" 28552001 28553000 "*" 0 0 63.9491007916713
"chr13" 28566001 28567000 "*" 6.59550958292954e-09 1.13456194382773e-08 -100
"chr13" 28594001 28595000 "*" 6.04438721296674e-11 1.38849255306602e-10 -100
"chr13" 28605001 28606000 "*" 4.08209022140227e-11 9.57770690255255e-11 -100
"chr13" 28630001 28631000 "*" 8.16498746392114e-08 1.19839791147926e-07
73.3333333333333
"chr13" 28631001 28632000 "*" 6.14916653047803e-05 5.96331133051861e-05
-53.030303030303
"chr13" 28884001 28885000 "*" 1.26565424807268e-14 4.63923203146481e-14 -100
"chr13" 28982001 28983000 "*" 0 0 100
"chr13" 28991001 28992000 "*" 4.18887147191072e-13 1.2739946038722e-12 -100
"chr13" 29183001 29184000 "*" 3.19541565430015e-08 4.99241102033518e-08
64.2857142857143
"chr13" 29337001 29338000 "*" 0 0 100
"chr13" 29392001 29393000 "*" 2.61177746097019e-11 6.31941920492787e-11
59.0909090909091
"chr13" 29394001 29395000 "*" 0 0 54.5793777135663
"chr13" 29415001 29416000 "*" 1.61548552313207e-12 4.57449558868942e-12
-93.3333333333333
"chr13" 29435001 29436000 "*" 4.65637306490407e-10 9.46720524046618e-10 -100
"chr13" 29465001 29466000 "*" 6.65782864395226e-08 9.93571823373348e-08 -90
"chr13" 29586001 29587000 "*" 2.63798982658159e-11 6.3791170118387e-11
-65.7142857142857
"chr13" 29674001 29675000 "*" 2.67283972732457e-11 6.45686178157698e-11 -100
"chr13" 29790001 29791000 "*" 9.5812247025151e-14 3.15667542866807e-13 100
"chr13" 29798001 29799000 "*" 3.34128893442198e-08 5.19772864618523e-08 -100
"chr13" 29818001 29819000 "*" 3.99680288865056e-14 1.37975970265307e-13 -100

"chr13" 30015001 30016000 "*" 5.03441732746523e-12 1.3378171545226e-11 100
"chr13" 30016001 30017000 "*" 1.39779633423487e-07 1.98463037958589e-07 100
"chr13" 30055001 30056000 "*" 5.02641261945769e-11 1.16588696106366e-10
-64.1509433962264
"chr13" 30074001 30075000 "*" 0 0 100
"chr13" 30112001 30113000 "*" 1.11022302462516e-16 5.03662826618488e-16
66.6666666666667
"chr13" 30136001 30137000 "*" 7.93809462606987e-14 2.64679416473686e-13 100
"chr13" 30221001 30222000 "*" 0 0 100
"chr13" 30481001 30482000 "*" 7.0006535457523e-08 1.03695832512054e-07 -100
"chr13" 30492001 30493000 "*" 2.68673971959288e-14 9.47266815431045e-14 100
"chr13" 30498001 30499000 "*" 9.63829016598083e-12 2.46993429847588e-11 -100
"chr13" 30512001 30513000 "*" 0 0 54.6875
"chr13" 30590001 30591000 "*" 1.77806292118721e-05 1.8763499200702e-05
52.1739130434783
"chr13" 30688001 30689000 "*" 0 0 -93.006993006993
"chr13" 30690001 30691000 "*" 6.37490060739765e-13 1.89496793587877e-12 -100
"chr13" 30722001 30723000 "*" 4.33877644923086e-08 6.63361344592129e-08
-86.9565217391304
"chr13" 30731001 30732000 "*" 3.10862446895044e-15 1.22466437093774e-14 -100
"chr13" 30926001 30927000 "*" 1.87811988183739e-11 4.6329825531037e-11
90.7894736842105
"chr13" 30937001 30938000 "*" 1.03834827450688e-05 1.13502457227881e-05
57.1428571428571
"chr13" 31120001 31121000 "*" 6.4152538836737e-10 1.27477697728417e-09 -100
"chr13" 31320001 31321000 "*" 5.6362137179633e-11 1.29963270300065e-10 100
"chr13" 31446001 31447000 "*" 2.1094237467878e-15 8.47434540879246e-15
-53.8461538461538
"chr13" 31529001 31530000 "*" 2.32862840121584e-10 4.93719283454501e-10
-91.6666666666667
"chr13" 31661001 31662000 "*" 1.98365768255826e-11 4.85612402473442e-11 -100
"chr13" 31692001 31693000 "*" 3.10862446895044e-15 1.22466437093774e-14 100
"chr13" 31921001 31922000 "*" 0 0 -100
"chr13" 32326001 32327000 "*" 7.64977431955138e-07 9.86938478298795e-07
57.1428571428571
"chr13" 32605001 32606000 "*" 0 0 -85.3324263724082
"chr13" 32823001 32824000 "*" 2.12785544739802e-09 3.93264697433322e-09
-74.7474747474748
"chr13" 32847001 32848000 "*" 0 0 100
"chr13" 32869001 32870000 "*" 2.96843660763102e-11 7.11039353170169e-11
-66.6666666666667
"chr13" 33002001 33003000 "*" 0 0 -58.4160756501182
"chr13" 33282001 33283000 "*" 6.62958576924666e-12 1.73620572057889e-11 -100
"chr13" 33401001 33402000 "*" 0.000499129401911658 0.000416865202314435
-52.3809523809524
"chr13" 33467001 33468000 "*" 5.1223403296774e-10 1.03532645132705e-09
-73.9130434782609
"chr13" 33477001 33478000 "*" 2.08814465718632e-09 3.86232567960463e-09 100
"chr13" 33492001 33493000 "*" 2.66639721686612e-05 2.73687601798495e-05
-53.1645569620253
"chr13" 33546001 33547000 "*" 2.33028818463765e-10 4.93719283454501e-10 -100
"chr13" 33616001 33617000 "*" 3.04628685166008e-07 4.14477782922613e-07

-92.3076923076923
"chr13" 33838001 33839000 "*" 5.6362137179633e-11 1.29963270300065e-10 100
"chr13" 33957001 33958000 "*" 4.14335232790108e-13 1.26153063645578e-12 -100
"chr13" 33962001 33963000 "*" 8.55919767679403e-05 8.10918052683734e-05 53.125
"chr13" 34026001 34027000 "*" 1.67299207820548e-08 2.71377429441055e-08 -100
"chr13" 34188001 34189000 "*" 1.46549439250521e-14 5.33330076973935e-14 -100
"chr13" 34239001 34240000 "*" 1.12458486967171e-09 2.15740815043925e-09 100
"chr13" 34302001 34303000 "*" 3.34128893442198e-08 5.19772864618523e-08 100
"chr13" 34822001 34823000 "*" 1.92946192356658e-09 3.59193011611483e-09 100
"chr13" 35093001 35094000 "*" 1.69109171110904e-12 4.78063274849742e-12 84.375
"chr13" 35353001 35354000 "*" 3.03090885722668e-14 1.06210046699299e-13 -100
"chr13" 35516001 35517000 "*" 0 0 56.9574025340555
"chr13" 35821001 35822000 "*" 3.6700841921089e-08 5.65530226072258e-08 100
"chr13" 36083001 36084000 "*" 6.4152538836737e-10 1.27477697728417e-09 -100
"chr13" 36271001 36272000 "*" 1.11022302462516e-16 5.03662826618488e-16 -100
"chr13" 36280001 36281000 "*" 2.56822341171414e-11 6.21944953427370e-11
-55.3359683794466
"chr13" 36286001 36287000 "*" 4.26951363152739e-10 8.72510873516957e-10 -100
"chr13" 36287001 36288000 "*" 2.3990809339125e-12 6.65441738642456e-12 -100
"chr13" 36304001 36305000 "*" 0 0 100
"chr13" 36339001 36340000 "*" 2.60792099027185e-08 4.11523334967881e-08
56.3492063492063
"chr13" 36386001 36387000 "*" 4.99900121297969e-12 1.32921056339624e-11 -100
"chr13" 36484001 36485000 "*" 9.99200722162641e-16 4.15382497462808e-15 100
"chr13" 36490001 36491000 "*" 9.04570973681018e-10 1.76414389822173e-09 -100
"chr13" 36609001 36610000 "*" 5.76576564270681e-09 1.00214420049533e-08
71.4285714285714
"chr13" 36664001 36665000 "*" 0 0 -100
"chr13" 36732001 36733000 "*" 0.000184006378754042 0.000165249884973406
54.1666666666667
"chr13" 36954001 36955000 "*" 3.34128893442198e-08 5.19772864618523e-08 -100
"chr13" 37288001 37289000 "*" 2.43005615629954e-09 4.45014222280278e-09 100
"chr13" 37430001 37431000 "*" 1.29037891483108e-11 3.24582280286866e-11 -100
"chr13" 37477001 37478000 "*" 0 0 100
"chr13" 37491001 37492000 "*" 3.6700841921089e-08 5.65530226072258e-08 -100
"chr13" 39260001 39261000 "*" 2.26195986463562e-06 2.7315498780547e-06
62.3376623376623
"chr13" 39727001 39728000 "*" 1.90181204118289e-13 6.05298617539364e-13 -100
"chr13" 40176001 40177000 "*" 0 0 68.561872909699
"chr13" 40177001 40178000 "*" 0 0 64.5109993991001
"chr13" 40215001 40216000 "*" 1.51656465163796e-13 4.87803420672651e-13 -100
"chr13" 40431001 40432000 "*" 1.02140518265514e-14 3.79278290836596e-14 87.5
"chr13" 40519001 40520000 "*" 2.43005615629954e-09 4.45014222280278e-09 100
"chr13" 40601001 40602000 "*" 7.57266585726946e-06 8.44903872709532e-06
62.962962962963
"chr13" 40641001 40642000 "*" 2.64951482975562e-09 4.82186011375752e-09 100
"chr13" 40648001 40649000 "*" 0 0 -100
"chr13" 40671001 40672000 "*" 9.67004254448511e-14 3.18178990782609e-13 100
"chr13" 40810001 40811000 "*" 1.21580523426701e-12 3.50134682034786e-12
66.6666666666667
"chr13" 40912001 40913000 "*" 2.88710388929303e-10 6.03399264731139e-10 -100
"chr13" 40954001 40955000 "*" 3.90831811358794e-12 1.05610567215048e-11 -100

"chr13" 41044001 41045000 "*" 5.85087533977457e-14 1.9792613012899e-13 -70
"chr13" 41108001 41109000 "*" 3.7274627828765e-12 1.00985250136599e-11 -100
"chr13" 41137001 41138000 "*" 0 0 -100
"chr13" 41148001 41149000 "*" 2.00227675550835e-08 3.20459176617961e-08 -100
"chr13" 41232001 41233000 "*" 1.12960025666631e-07 1.62731564018923e-07
50.6568144499179
"chr13" 41238001 41239000 "*" 0 0 -84.5833333333333
"chr13" 41495001 41496000 "*" 0 0 -62.3655913978495
"chr13" 41647001 41648000 "*" 1.13140401492018e-08 1.87801380787728e-08 100
"chr13" 41741001 41742000 "*" 0 0 -100
"chr13" 41964001 41965000 "*" 6.4152538836737e-10 1.27477697728417e-09 100
"chr13" 42030001 42031000 "*" 0 0 98.1884057971015
"chr13" 42046001 42047000 "*" 0 0 100
"chr13" 42232001 42233000 "*" 1.15463194561016e-14 4.25829614430727e-14 100
"chr13" 42586001 42587000 "*" 1.10184765755861e-08 1.83760323537865e-08
50.7246376811594
"chr13" 42589001 42590000 "*" 4.78841410966879e-11 1.11468187156588e-10 100
"chr13" 42638001 42639000 "*" 8.52140580320793e-12 2.19817259951489e-11 100
"chr13" 42845001 42846000 "*" 0 0 100
"chr13" 42943001 42944000 "*" 9.0072393987839e-13 2.63157725646847e-12 -100
"chr13" 42969001 42970000 "*" 1.7803536422889e-11 4.40222759700651e-11
62.962962962963
"chr13" 43100001 43101000 "*" 1.84048428297778e-05 1.93665920541667e-05
-59.2592592592593
"chr13" 43701001 43702000 "*" 1.06363524698949e-06 1.34530286878699e-06
-52.1739130434783
"chr13" 43836001 43837000 "*" 4.01313771103418e-09 7.11882087237587e-09 100
"chr13" 44160001 44161000 "*" 4.71134242729931e-12 1.257615036806e-11 -100
"chr13" 44360001 44361000 "*" 0 0 72.3854173691915
"chr13" 44361001 44362000 "*" 0 0 79.1714667831936
"chr13" 44454001 44455000 "*" 0 0 -77.9591836734694
"chr13" 44550001 44551000 "*" 1.82217324784517e-07 2.55249620191849e-07 52.5
"chr13" 44619001 44620000 "*" 2.20956036933728e-06 2.67287762240877e-06
-58.6538461538462
"chr13" 44627001 44628000 "*" 3.25818809512324e-05 3.29735679006858e-05
66.6666666666667
"chr13" 44793001 44794000 "*" 0 0 56.25
"chr13" 44881001 44882000 "*" 7.6518013396587e-05 7.30785292812216e-05
-52.7777777777778
"chr13" 44887001 44888000 "*" 9.65729496371637e-10 1.87255912493232e-09 -100
"chr13" 44912001 44913000 "*" 0 0 -67.741935483871
"chr13" 45152001 45153000 "*" 0 0 100
"chr13" 45356001 45357000 "*" 3.66373598126302e-15 1.43441054525534e-14
-92.3076923076923
"chr13" 45375001 45376000 "*" 4.50215679970167e-06 5.19390556817063e-06
62.0689655172414
"chr13" 45418001 45419000 "*" 2.73625566649116e-12 7.52483363579137e-12 -100
"chr13" 45456001 45457000 "*" 5.82080730282541e-08 8.75553486242363e-08 90
"chr13" 45816001 45817000 "*" 2.08995487582797e-10 4.46944006972663e-10 -100
"chr13" 45959001 45960000 "*" 3.9190872769268e-14 1.35416418933905e-13 -100
"chr13" 45992001 45993000 "*" 0 0 85
"chr13" 46019001 46020000 "*" 7.0006535457523e-08 1.03695832512054e-07 -100

"chr13" 46021001 46022000 "*" 8.25450818808804e-13 2.42473843346228e-12 100
"chr13" 46323001 46324000 "*" 1.35036426485158e-12 3.86629547363774e-12 100
"chr13" 46365001 46366000 "*" 0.000126887439664713 0.000117029410540844
-54.5454545454545
"chr13" 46384001 46385000 "*" 1.17461596005342e-13 3.8279067575877e-13 100
"chr13" 46453001 46454000 "*" 0.000525299954949099 0.000436849389802675
-54.5454545454545
"chr13" 46785001 46786000 "*" 0 0 79.5762248800923
"chr13" 46930001 46931000 "*" 7.4951922419686e-06 8.36942869052848e-06
-71.0526315789474
"chr13" 47571001 47572000 "*" 2.50077736296817e-11 6.0631580870835e-11
-94.4444444444444
"chr13" 47840001 47841000 "*" 1.93209892529467e-11 4.75910088850756e-11
-58.8235294117647
"chr13" 48470001 48471000 "*" 6.92287338566189e-11 1.57605630124247e-10 -100
"chr13" 48575001 48576000 "*" 0 0 58
"chr13" 48751001 48752000 "*" 6.4324989779152e-11 1.47230020825092e-10 100
"chr13" 48859001 48860000 "*" 4.3704954100221e-09 7.72201689084812e-09
-66.6666666666667
"chr13" 49005001 49006000 "*" 2.00227675550835e-08 3.20459176617961e-08 -100
"chr13" 49077001 49078000 "*" 5.6362137179633e-11 1.29963270300065e-10 -100
"chr13" 49107001 49108000 "*" 0 0 -97.8260869565217
"chr13" 49254001 49255000 "*" 1.13140401492018e-08 1.87801380787728e-08 -100
"chr13" 49442001 49443000 "*" 1.67299207820548e-08 2.71377429441055e-08 -100
"chr13" 49657001 49658000 "*" 8.01666433236647e-09 1.36392368970109e-08 100
"chr13" 49884001 49885000 "*" 0.000215362416299381 0.000191330857945016
52.6315789473684
"chr13" 50017001 50018000 "*" 0 0 100
"chr13" 50128001 50129000 "*" 0 0 90.4761904761905
"chr13" 50413001 50414000 "*" 2.08995487582797e-10 4.46944006972663e-10 -100
"chr13" 50415001 50416000 "*" 3.95353005888666e-09 7.03928529283733e-09 100
"chr13" 50570001 50571000 "*" 0 0 -50.1638191661393
"chr13" 50801001 50802000 "*" 1.67299207820548e-08 2.71377429441055e-08 -100
"chr13" 51576001 51577000 "*" 0.000105773217436211 9.87995681627625e-05
-53.8461538461538
"chr13" 51619001 51620000 "*" 2.88779000712225e-11 6.92935809466068e-11 100
"chr13" 51689001 51690000 "*" 2.33028818463765e-10 4.93719283454501e-10 100
"chr13" 51711001 51712000 "*" 1.07882591748876e-11 2.74553537455803e-11 100
"chr13" 51792001 51793000 "*" 3.7607964253894e-07 5.05231943813116e-07
72.972972972973
"chr13" 51796001 51797000 "*" 0 0 70.6649282920469
"chr13" 51797001 51798000 "*" 0 0 90.5660377358491
"chr13" 51817001 51818000 "*" 9.67004254448511e-14 3.18178990782609e-13 100
"chr13" 51854001 51855000 "*" 1.88737914186277e-15 7.62011598380321e-15
51.624506846136
"chr13" 51866001 51867000 "*" 1.37828859436695e-10 3.01445544267661e-10 -100
"chr13" 52339001 52340000 "*" 0 0 100
"chr13" 52355001 52356000 "*" 8.92637370020211e-06 9.85381833193747e-06
-64.2857142857143
"chr13" 52371001 52372000 "*" 6.37523367430504e-11 1.46123322005585e-10
-81.8181818181818
"chr13" 52378001 52379000 "*" 0 0 56.4102564102564

"chr13" 52390001 52391000 "*" 0 0 -94.4444444444444
"chr13" 52392001 52393000 "*" 2.33028818463765e-10 4.93719283454501e-10 100
"chr13" 52415001 52416000 "*" 0 0 -100
"chr13" 52467001 52468000 "*" 9.65729496371637e-10 1.87255912493232e-09 100
"chr13" 52639001 52640000 "*" 0.000320710195636176 0.000276619581787338
-52.3809523809524
"chr13" 52657001 52658000 "*" 5.44009282066327e-15 2.08486137681812e-14 100
"chr13" 52742001 52743000 "*" 3.63445940010365e-11 8.58565209111204e-11 -100
"chr13" 52969001 52970000 "*" 8.10054090472079e-09 1.37737926825267e-08
60.5797101449275
"chr13" 53015001 53016000 "*" 1.68753899743024e-14 6.09826805967439e-14 -100
"chr13" 53185001 53186000 "*" 1.08682025601325e-08 1.81388338901142e-08
80.9523809523809
"chr13" 53282001 53283000 "*" 1.30850885682321e-12 3.7561282663343e-12
53.8461538461538
"chr13" 53363001 53364000 "*" 5.55111512312578e-16 2.36485094870365e-15
85.7142857142857
"chr13" 53411001 53412000 "*" 0.000432672693284286 0.000365157266029702
-53.3333333333333
"chr13" 53579001 53580000 "*" 2.22044604925031e-16 9.81641919380259e-16 100
"chr13" 53582001 53583000 "*" 3.90465437760668e-13 1.19307893943277e-12 100
"chr13" 53589001 53590000 "*" 3.33066907387547e-16 1.4495649018245e-15 100
"chr13" 53649001 53650000 "*" 1.48487888651516e-11 3.70689457767456e-11 100
"chr13" 54447001 54448000 "*" 0 0 63.6363636363636
"chr13" 54887001 54888000 "*" 0.000234316146434987 0.000206869454871927
-54.4385026737968
"chr13" 54890001 54891000 "*" 9.13713549266504e-14 3.02110477479709e-13
55.5555555555556
"chr13" 54892001 54893000 "*" 1.11022302462516e-16 5.03662826618488e-16 100
"chr13" 55216001 55217000 "*" 5.6362137179633e-11 1.29963270300065e-10 100
"chr13" 55232001 55233000 "*" 0 0 -100
"chr13" 55373001 55374000 "*" 0 0 -50.8542875564152
"chr13" 56551001 56552000 "*" 3.56775498033812e-10 7.35898629809829e-10 100
"chr13" 57460001 57461000 "*" 2.75868217158859e-12 7.58275709670968e-12
64.9136577708006
"chr13" 57693001 57694000 "*" 7.0006535457523e-08 1.03695832512054e-07 100
"chr13" 59068001 59069000 "*" 0 0 -100
"chr13" 59077001 59078000 "*" 2.16382467499443e-13 6.82775854166559e-13 100
"chr13" 59185001 59186000 "*" 0 0 -78.8461538461538
"chr13" 59219001 59220000 "*" 3.85360313920335e-05 3.85553082302386e-05
57.8947368421053
"chr13" 59435001 59436000 "*" 1.55431223447522e-15 6.33705141101682e-15
-85.7142857142857
"chr13" 59693001 59694000 "*" 8.41756960467954e-07 1.07946670534514e-06
-77.0833333333333
"chr13" 59831001 59832000 "*" 6.77335965093562e-12 1.76897011246462e-11 -100
"chr13" 59985001 59986000 "*" 1.11022302462516e-16 5.03662826618488e-16 -100
"chr13" 60013001 60014000 "*" 5.43117762319412e-06 6.19387126034938e-06
84.6153846153846
"chr13" 60191001 60192000 "*" 5.48638023900594e-10 1.10374357336421e-09 100
"chr13" 60222001 60223000 "*" 1.01313477069986e-05 1.10909163221897e-05 -52
"chr13" 60232001 60233000 "*" 2.30404601619938e-07 3.18550210701194e-07

-85.7142857142857
"chr13" 61161001 61162000 "*" 1.24422019909254e-07 1.78238773919326e-07
54.6296296296296
"chr13" 61331001 61332000 "*" 6.59550958292954e-09 1.13456194382773e-08 -100
"chr13" 61463001 61464000 "*" 1.81731929504991e-09 3.3959735570263e-09
61.5384615384615
"chr13" 61790001 61791000 "*" 0.000560161430107065 0.0004636271528737 55
"chr13" 62340001 62341000 "*" 0 0 100
"chr13" 62516001 62517000 "*" 0 0 96.6666666666667
"chr13" 62723001 62724000 "*" 4.73234496034536e-09 8.30627300264826e-09 100
"chr13" 62891001 62892000 "*" 0 0 -100
"chr13" 62969001 62970000 "*" 0 0 100
"chr13" 63304001 63305000 "*" 4.44089209850063e-16 1.91071758245033e-15 100
"chr13" 63373001 63374000 "*" 2.16382467499443e-13 6.82775854166559e-13 100
"chr13" 63811001 63812000 "*" 2.8519409056571e-12 7.82336806394733e-12 -60
"chr13" 63971001 63972000 "*" 0 0 76.9230769230769
"chr13" 64780001 64781000 "*" 3.34128893442198e-08 5.19772864618523e-08 -100
"chr13" 65800001 65801000 "*" 2.91028312560115e-11 6.97590507139978e-11 -100
"chr13" 65905001 65906000 "*" 6.83991506766901e-07 8.88460267349551e-07
67.5675675675676
"chr13" 66296001 66297000 "*" 6.62958576924666e-12 1.73620572057889e-11 100
"chr13" 66357001 66358000 "*" 0 0 100
"chr13" 66358001 66359000 "*" 0 0 100
"chr13" 66584001 66585000 "*" 8.58713663065558e-06 9.50083815296748e-06
63.0434782608696
"chr13" 66704001 66705000 "*" 3.00652418849801e-07 4.0944173474794e-07 75
"chr13" 66808001 66809000 "*" 4.01313771103418e-09 7.11882087237587e-09 100
"chr13" 67411001 67412000 "*" 6.24654217240561e-10 1.2462108191303e-09 100
"chr13" 67483001 67484000 "*" 5.6362137179633e-11 1.29963270300065e-10 -100
"chr13" 67568001 67569000 "*" 2.15125472990962e-10 4.58126659943874e-10 -100
"chr13" 68040001 68041000 "*" 0 0 -100
"chr13" 68939001 68940000 "*" 1.12055920098442e-11 2.84500040664577e-11 100
"chr13" 69270001 69271000 "*" 2.88779000712225e-11 6.92935809466068e-11 100
"chr13" 70830001 70831000 "*" 1.73072419040743e-06 2.12560713538065e-06
-61.9047619047619
"chr13" 70961001 70962000 "*" 0 0 -100
"chr13" 72438001 72439000 "*" 0 0 100
"chr13" 72439001 72440000 "*" 0 0 54.5336742075728
"chr13" 72440001 72441000 "*" 0 0 60.1336302895323
"chr13" 72596001 72597000 "*" 1.56863411149288e-12 4.4469621057462e-12 100
"chr13" 72848001 72849000 "*" 4.08097999837764e-11 9.57770690255255e-11
-60.462962962963
"chr13" 73128001 73129000 "*" 3.9190872769268e-14 1.35416418933905e-13 -100
"chr13" 73337001 73338000 "*" 1.65267695177995e-05 1.75228091743656e-05
54.5454545454545
"chr13" 73615001 73616000 "*" 0 0 -100
"chr13" 73678001 73679000 "*" 6.4324989779152e-11 1.47230020825092e-10 -100
"chr13" 73940001 73941000 "*" 7.43849426498855e-15 2.80357739563059e-14 -100
"chr13" 73984001 73985000 "*" 8.24873502835999e-11 1.86128689830788e-10
-59.2592592592593
"chr13" 74655001 74656000 "*" 3.34128893442198e-08 5.19772864618523e-08 100
"chr13" 74833001 74834000 "*" 1.13140401492018e-08 1.87801380787728e-08 -100

"chr13" 74875001 74876000 "*" 0 0 -80
"chr13" 74987001 74988000 "*" 1.60669759718957e-08 2.61933029878312e-08
80.7017543859649
"chr13" 75958001 75959000 "*" 6.15840711759574e-13 1.83676803572802e-12 100
"chr13" 75991001 75992000 "*" 2.33028818463765e-10 4.93719283454501e-10 -100
"chr13" 76210001 76211000 "*" 0 0 -77.7915632754342
"chr13" 76279001 76280000 "*" 1.98325800226939e-11 4.85612402473442e-11 -100
"chr13" 76765001 76766000 "*" 4.32209823486573e-12 1.15935290479641e-11 -100
"chr13" 76793001 76794000 "*" 1.12634901405784e-10 2.49563754426169e-10 -100
"chr13" 77423001 77424000 "*" 0 0 -86.6666666666667
"chr13" 77459001 77460000 "*" 0 0 76.1070003328655
"chr13" 77460001 77461000 "*" 0 0 80.7963763614782
"chr13" 77486001 77487000 "*" 1.39779633423487e-07 1.98463037958589e-07 100
"chr13" 77565001 77566000 "*" 0 0 -57.1428571428571
"chr13" 78125001 78126000 "*" 0.000405843218347801 0.000344123133324164
-51.0204081632653
"chr13" 78640001 78641000 "*" 1.94160798550058e-10 4.17456713670218e-10
-53.8461538461538
"chr13" 79030001 79031000 "*" 1.98365768255826e-11 4.85612402473442e-11 100
"chr13" 79179001 79180000 "*" 0 0 76.4150943396226
"chr13" 79326001 79327000 "*" 1.39779633423487e-07 1.98463037958589e-07 100
"chr13" 79427001 79428000 "*" 3.5527136788005e-15 1.39277426410519e-14 -100
"chr13" 79508001 79509000 "*" 6.47593090263854e-13 1.92390250706249e-12
78.2608695652174
"chr13" 80015001 80016000 "*" 1.60310431596145e-10 3.47435209361035e-10 75
"chr13" 80413001 80414000 "*" 2.88779000712225e-11 6.92935809466068e-11 100
"chr13" 80414001 80415000 "*" 1.52794177310511e-09 2.87642564232045e-09 100
"chr13" 80914001 80915000 "*" 0 0 73.6636687595452
"chr13" 81048001 81049000 "*" 2.88779000712225e-11 6.92935809466068e-11 100
"chr13" 81279001 81280000 "*" 1.74013364573344e-06 2.1364085133039e-06
66.6666666666667
"chr13" 81688001 81689000 "*" 1.49613654798486e-12 4.26244554429779e-12 -100
"chr13" 83397001 83398000 "*" 8.9381607670802e-09 1.50769337945046e-08
54.5454545454545
"chr13" 84027001 84028000 "*" 0 0 100
"chr13" 85444001 85445000 "*" 9.63829016598083e-12 2.46993429847588e-11 100
"chr13" 85991001 85992000 "*" 6.59550958292954e-09 1.13456194382773e-08 100
"chr13" 86082001 86083000 "*" 4.50904817972386e-06 5.20135215572493e-06
59.2592592592593
"chr13" 87395001 87396000 "*" 0 0 100
"chr13" 87478001 87479000 "*" 0 0 100
"chr13" 88629001 88630000 "*" 2.89780532902739e-10 6.05499283768567e-10
72.7272727272727
"chr13" 88789001 88790000 "*" 1.09648181845223e-06 1.3842235994417e-06 60
"chr13" 89099001 89100000 "*" 0 0 -100
"chr13" 89547001 89548000 "*" 7.0006535457523e-08 1.03695832512054e-07 100
"chr13" 89548001 89549000 "*" 0 0 100
"chr13" 90756001 90757000 "*" 1.98365768255826e-11 4.85612402473442e-11 100
"chr13" 91080001 91081000 "*" 6.88338275267597e-15 2.60518659957509e-14 -100
"chr13" 91094001 91095000 "*" 1.5277158427196e-09 2.87642564232045e-09 -100
"chr13" 91139001 91140000 "*" 3.6700841921089e-08 5.65530226072258e-08 -100
"chr13" 91383001 91384000 "*" 4.79616346638068e-14 1.64380445416596e-13 -100

"chr13" 91384001 91385000 "*" 4.13329948045771e-09 7.31905277636459e-09 -80
"chr13" 91401001 91402000 "*" 6.24386464753712e-09 1.08049105382103e-08 75.4491017964072
"chr13" 91519001 91520000 "*" 0 0 100
"chr13" 91779001 91780000 "*" 1.96509475358653e-13 6.23868565133654e-13 -100
"chr13" 91811001 91812000 "*" 2.43005615629954e-09 4.45014222280278e-09 100
"chr13" 92050001 92051000 "*" 0 0 54.8387096774194
"chr13" 92416001 92417000 "*" 0 0 -100
"chr13" 92504001 92505000 "*" 9.65729496371637e-10 1.87255912493232e-09 -100
"chr13" 92573001 92574000 "*" 1.36593444340916e-05 1.46569770945072e-05 -69.6969696969697
"chr13" 94148001 94149000 "*" 8.7349629751543e-09 1.47505871898511e-08 100
"chr13" 94925001 94926000 "*" 5.63660229602192e-13 1.68905058935478e-12 100
"chr13" 95061001 95062000 "*" 8.14264211612681e-11 1.83849160122308e-10 -65.8227848101266
"chr13" 95318001 95319000 "*" 0.00020976332305167 0.000186660769941092 52.3809523809524
"chr13" 95335001 95336000 "*" 0 0 100
"chr13" 95486001 95487000 "*" 0 0 100
"chr13" 95686001 95687000 "*" 5.04196684403269e-12 1.33978063114264e-11 62.5
"chr13" 95852001 95853000 "*" 2.77555756156289e-15 1.10015415195978e-14 -100
"chr13" 95993001 95994000 "*" 5.51780843238703e-14 1.87243792572872e-13 -100
"chr13" 96056001 96057000 "*" 0 0 70.2564102564103
"chr13" 96095001 96096000 "*" 5.58071477918531e-10 1.12161263408564e-09 -61.5384615384615
"chr13" 96256001 96257000 "*" 3.33066907387547e-16 1.4495649018245e-15 100
"chr13" 96266001 96267000 "*" 3.56775498033812e-10 7.35898629809829e-10 100
"chr13" 96454001 96455000 "*" 1.98973839449224e-06 2.42104327047281e-06 51.4285714285714
"chr13" 96742001 96743000 "*" 0 0 52.6688144831294
"chr13" 96810001 96811000 "*" 1.8125231100341e-05 1.90935684500041e-05 -58.6206896551724
"chr13" 97075001 97076000 "*" 1.74853465040314e-11 4.32752710900609e-11 -100
"chr13" 97228001 97229000 "*" 1.18454067443707e-07 1.70116091534804e-07 -74.8201438848921
"chr13" 97332001 97333000 "*" 8.7349629751543e-09 1.47505871898511e-08 -100
"chr13" 97374001 97375000 "*" 7.0006535457523e-08 1.03695832512054e-07 100
"chr13" 97579001 97580000 "*" 5.03264097062583e-13 1.51942428521737e-12 -100
"chr13" 98019001 98020000 "*" 7.0006535457523e-08 1.03695832512054e-07 -100
"chr13" 98085001 98086000 "*" 0 0 87.3239436619718
"chr13" 98274001 98275000 "*" 7.17204073907851e-14 2.40299371819945e-13 -51.0582010582011
"chr13" 98427001 98428000 "*" 3.51676460952355e-07 4.74504334939215e-07 71.6450216450216
"chr13" 98520001 98521000 "*" 4.73234496034536e-09 8.30627300264826e-09 -100
"chr13" 98527001 98528000 "*" 1.8918767263898e-07 2.64436276666986e-07 79.4117647058823
"chr13" 98713001 98714000 "*" 1.98365768255826e-11 4.85612402473442e-11 100
"chr13" 98721001 98722000 "*" 6.66133814775094e-16 2.81595744474255e-15 100
"chr13" 98771001 98772000 "*" 9.08108033215171e-11 2.03407753381119e-10 -100
"chr13" 98783001 98784000 "*" 0 0 100
"chr13" 98807001 98808000 "*" 0 0 -100

"chr13" 98824001 98825000 "*" 0 0 -68.75
"chr13" 98968001 98969000 "*" 0.000139451556673498 0.000127724714298755
58.3333333333333
"chr13" 99002001 99003000 "*" 0 0 94.4444444444444
"chr13" 99072001 99073000 "*" 4.08209022140227e-11 9.57770690255255e-11 -100
"chr13" 99113001 99114000 "*" 3.90831811358794e-12 1.05610567215048e-11 100
"chr13" 99158001 99159000 "*" 3.31956684362922e-14 1.15814343608523e-13
53.8461538461538
"chr13" 99305001 99306000 "*" 6.59550958292954e-09 1.13456194382773e-08 100
"chr13" 99404001 99405000 "*" 0 0 65.232628216368
"chr13" 99444001 99445000 "*" 1.39779633423487e-07 1.98463037958589e-07 100
"chr13" 99537001 99538000 "*" 5.77315972805081e-15 2.205310974382e-14 60
"chr13" 99630001 99631000 "*" 0 0 -79.5180722891566
"chr13" 99650001 99651000 "*" 1.00808250635964e-13 3.31107370956809e-13 -100
"chr13" 99740001 99741000 "*" 3.92655274961129e-08 6.03144370578987e-08
73.5632183908046
"chr13" 99807001 99808000 "*" 2.51121345939964e-11 6.08686795959118e-11
-69.2307692307692
"chr13" 99855001 99856000 "*" 2.88710388929303e-10 6.03399264731139e-10 -100
"chr13" 100057001 100058000 "*" 8.06688049692639e-13 2.37556391305009e-12 100
"chr13" 100059001 100060000 "*" 2.77788440028459e-08 4.36995426069457e-08 -90
"chr13" 100075001 100076000 "*" 1.11022302462516e-16 5.03662826618488e-16 100
"chr13" 100234001 100235000 "*" 8.01666433236647e-09 1.36392368970109e-08 -100
"chr13" 100478001 100479000 "*" 3.04324343503026e-11 7.26599935673121e-11 -100
"chr13" 100482001 100483000 "*" 4.99020824662466e-11 1.15790792586157e-10 -100
"chr13" 100533001 100534000 "*" 5.96089844151493e-12 1.57055375177837e-11
-68.6274509803922
"chr13" 100550001 100551000 "*" 0 0 -100
"chr13" 100631001 100632000 "*" 0 0 -89.622641509434
"chr13" 100882001 100883000 "*" 0 0 100
"chr13" 101108001 101109000 "*" 0 0 -56.25
"chr13" 101134001 101135000 "*" 9.48348621854223e-10 1.84507747294874e-09
50.0398247710076
"chr13" 101135001 101136000 "*" 0.000354266937660941 0.000303242292809723
-56.5217391304348
"chr13" 101314001 101315000 "*" 1.07185123543463e-06 1.35516016195071e-06
58.7301587301587
"chr13" 101390001 101391000 "*" 4.08209022140227e-11 9.57770690255255e-11 100
"chr13" 101426001 101427000 "*" 0 0 100
"chr13" 101454001 101455000 "*" 8.7349629751543e-09 1.47505871898511e-08 -100
"chr13" 101490001 101491000 "*" 9.62896429257398e-13 2.80291205576417e-12 -100
"chr13" 101711001 101712000 "*" 2.16382467499443e-13 6.82775854166559e-13 -100
"chr13" 101796001 101797000 "*" 1.07882591748876e-11 2.74553537455803e-11 100
"chr13" 101832001 101833000 "*" 4.36607293374802e-06 5.04443830498714e-06
-65.9090909090909
"chr13" 101959001 101960000 "*" 0 0 -100
"chr13" 102433001 102434000 "*" 1.61204383175573e-13 5.17337152201509e-13
88.1720430107527
"chr13" 102453001 102454000 "*" 4.73234496034536e-09 8.30627300264826e-09 100
"chr13" 103363001 103364000 "*" 4.14335232790108e-13 1.26153063645578e-12 -100
"chr13" 103452001 103453000 "*" 0 0 56.6617862371889
"chr13" 103522001 103523000 "*" 3.6700841921089e-08 5.65530226072258e-08 100

"chr13" 104114001 104115000 "*" 2.22044604925031e-16 9.81641919380259e-16
-60.1176470588235
"chr13" 104606001 104607000 "*" 6.59550958292954e-09 1.13456194382773e-08 100
"chr13" 105136001 105137000 "*" 3.151751082342e-10 6.55981225680216e-10
52.5210084033613
"chr13" 105256001 105257000 "*" 0 0 100
"chr13" 105415001 105416000 "*" 2.50910403565285e-14 8.87903196465237e-14 100
"chr13" 105507001 105508000 "*" 3.77475828372553e-15 1.47379557022071e-14 100
"chr13" 105689001 105690000 "*" 3.95353005888666e-09 7.03928529283733e-09 100
"chr13" 106475001 106476000 "*" 2.49798024487546e-08 3.95034497779869e-08
59.3220338983051
"chr13" 106542001 106543000 "*" 9.08108033215171e-11 2.03407753381119e-10 100
"chr13" 106726001 106727000 "*" 4.70376358387981e-05 4.64559371515489e-05 -60
"chr13" 106812001 106813000 "*" 3.72381421254175e-08 5.73375243628994e-08
-56.25
"chr13" 106889001 106890000 "*" 0 0 100
"chr13" 106911001 106912000 "*" 1.33634214805056e-11 3.35607415271157e-11
93.9393939393939
"chr13" 107181001 107182000 "*" 1.10991671409266e-09 2.13466994589161e-09
-76.5625
"chr13" 107343001 107344000 "*" 4.71134242729931e-12 1.257615036806e-11 100
"chr13" 107354001 107355000 "*" 2.16337618150764e-05 2.25235415157106e-05
58.8235294117647
"chr13" 107356001 107357000 "*" 0 0 100
"chr13" 107474001 107475000 "*" 1.78368872013612e-05 1.88207237302589e-05
-50.7462686567164
"chr13" 107506001 107507000 "*" 3.25818809512324e-05 3.29735679006858e-05
-66.6666666666667
"chr13" 107526001 107527000 "*" 1.12458486967171e-09 2.15740815043925e-09 -100
"chr13" 107537001 107538000 "*" 2.69084754478399e-12 7.41487347763769e-12 -100
"chr13" 107724001 107725000 "*" 0 0 100
"chr13" 107856001 107857000 "*" 2.75891978362974e-06 3.28830469657595e-06
-51.6129032258064
"chr13" 107891001 107892000 "*" 2.22044604925031e-16 9.81641919380259e-16 100
"chr13" 108388001 108389000 "*" 3.52704532247117e-11 8.35581292290093e-11 100
"chr13" 108402001 108403000 "*" 5.08482145278322e-14 1.73432706170696e-13 -100
"chr13" 108723001 108724000 "*" 1.13140401492018e-08 1.87801380787728e-08 100
"chr13" 108871001 108872000 "*" 0 0 100
"chr13" 108913001 108914000 "*" 0 0 100
"chr13" 109003001 109004000 "*" 0 0 76.7123287671233
"chr13" 109038001 109039000 "*" 1.89651602311436e-07 2.65035872152575e-07
-64.4067796610169
"chr13" 109040001 109041000 "*" 8.25450818808804e-13 2.42473843346228e-12 100
"chr13" 109083001 109084000 "*" 0 0 81.5126050420168
"chr13" 109149001 109150000 "*" 2.18713935851156e-14 7.80893646430581e-14 100
"chr13" 109200001 109201000 "*" 7.22200077518664e-13 2.13505334148473e-12
60.7142857142857
"chr13" 109501001 109502000 "*" 6.04438721296674e-11 1.38849255306602e-10 100
"chr13" 109616001 109617000 "*" 5.62926395586416e-06 6.40552376199523e-06
66.6666666666667
"chr13" 109659001 109660000 "*" 2.2345949035607e-06 2.70100686099647e-06 -56.25
"chr13" 109740001 109741000 "*" 1.02840624904843e-10 2.29012597294218e-10

-67.4418604651163
"chr13" 109746001 109747000 "*" 2.88779000712225e-11 6.92935809466068e-11 100
"chr13" 109757001 109758000 "*" 1.14352971536391e-14 4.21921674335999e-14 -100
"chr13" 109939001 109940000 "*" 0 0 -100
"chr13" 109956001 109957000 "*" 6.4152538836737e-10 1.27477697728417e-09 -100
"chr13" 110037001 110038000 "*" 2.35305307461076e-08 3.73363995104836e-08
78.5714285714286
"chr13" 110171001 110172000 "*" 8.34776692215655e-12 2.15892328035363e-11
-89.0625
"chr13" 110172001 110173000 "*" 0 0 -71.2121212121212
"chr13" 110297001 110298000 "*" 6.59550958292954e-09 1.13456194382773e-08 100
"chr13" 110315001 110316000 "*" 0 0 -83.0508474576271
"chr13" 110356001 110357000 "*" 2.68450373042128e-10 5.64465213127486e-10 100
"chr13" 110358001 110359000 "*" 7.0006535457523e-08 1.03695832512054e-07 -100
"chr13" 110446001 110447000 "*" 2.11067031075096e-07 2.93248775554536e-07
-76.6666666666667
"chr13" 110496001 110497000 "*" 9.98963367582206e-09 1.67533179897223e-08
64.7058823529412
"chr13" 110528001 110529000 "*" 8.67652622971171e-07 1.1107452797228e-06
54.1666666666667
"chr13" 110552001 110553000 "*" 1.31610944930083e-05 1.4161144785177e-05
66.6666666666667
"chr13" 110553001 110554000 "*" 3.26405569239796e-14 1.13973003849234e-13 100
"chr13" 110616001 110617000 "*" 2.22044604925031e-16 9.81641919380259e-16
73.6842105263158
"chr13" 110714001 110715000 "*" 1.88737914186277e-15 7.62011598380321e-15
82.6446280991736
"chr13" 110726001 110727000 "*" 9.08108033215171e-11 2.03407753381119e-10 -100
"chr13" 110757001 110758000 "*" 5.99711072836184e-05 5.82540228222811e-05
54.5454545454545
"chr13" 110767001 110768000 "*" 0 0 -100
"chr13" 110773001 110774000 "*" 6.32827124036339e-15 2.40666772484995e-14
50.1276813074566
"chr13" 110802001 110803000 "*" 3.85641518718671e-11 9.08938008805696e-11
64.8648648648649
"chr13" 110862001 110863000 "*" 2.4174136876276e-05 2.49875818383709e-05
54.5454545454545
"chr13" 110889001 110890000 "*" 1.99977812087582e-10 4.28946270937178e-10
59.6153846153846
"chr13" 110892001 110893000 "*" 2.23154827949656e-14 7.95931582053377e-14 -100
"chr13" 110898001 110899000 "*" 0.000321268697584531 0.000277075512580754 60
"chr13" 110908001 110909000 "*" 0 0 100
"chr13" 110922001 110923000 "*" 0 0 70.3703703703704
"chr13" 110937001 110938000 "*" 4.08209022140227e-11 9.57770690255255e-11 -100
"chr13" 111055001 111056000 "*" 2.00227675550835e-08 3.20459176617961e-08 100
"chr13" 111062001 111063000 "*" 5.03495578563218e-10 1.01838003039562e-09
81.6901408450704
"chr13" 111071001 111072000 "*" 3.33066907387547e-16 1.4495649018245e-15
-90.9090909090909
"chr13" 111106001 111107000 "*" 1.04606567852272e-09 2.01786993558168e-09
77.6470588235294
"chr13" 111112001 111113000 "*" 0 0 -100

"chr13" 111135001 111136000 "*" 2.96451752035409e-12 8.11696741222615e-12 86.8852459016394
"chr13" 111170001 111171000 "*" 6.5536465143623e-13 1.94602017656154e-12 -100
"chr13" 111183001 111184000 "*" 0.000158379004406961 0.000143762727275916 -51.2820512820513
"chr13" 111195001 111196000 "*" 3.34128893442198e-08 5.19772864618523e-08 -100
"chr13" 111202001 111203000 "*" 0 0 62.962962962963
"chr13" 111212001 111213000 "*" 0 0 61.7886178861789
"chr13" 111230001 111231000 "*" 1.00027888860765e-07 1.45153300634997e-07 51.0204081632653
"chr13" 111267001 111268000 "*" 2.39432311754939e-08 3.79462254435764e-08 -60.2409638554217
"chr13" 111337001 111338000 "*" 4.01313771103418e-09 7.11882087237587e-09 100
"chr13" 111412001 111413000 "*" 1.11022302462516e-16 5.03662826618488e-16 61.7647058823529
"chr13" 111453001 111454000 "*" 4.99900121297969e-12 1.32921056339624e-11 100
"chr13" 111481001 111482000 "*" 4.93398655265764e-11 1.14636148130922e-10 -65.5172413793103
"chr13" 111524001 111525000 "*" 1.36372868553281e-09 2.59203543496899e-09 50.8474576271186
"chr13" 111527001 111528000 "*" 1.31006316905768e-14 4.79358493281887e-14 51.1627906976744
"chr13" 111531001 111532000 "*" 0 0 100
"chr13" 111602001 111603000 "*" 2.73625566649116e-12 7.52483363579137e-12 100
"chr13" 111698001 111699000 "*" 0 0 100
"chr13" 111703001 111704000 "*" 8.52140580320793e-12 2.19817259951489e-11 100
"chr13" 111735001 111736000 "*" 6.4152538836737e-10 1.27477697728417e-09 -100
"chr13" 111764001 111765000 "*" 3.90465437760668e-13 1.19307893943277e-12 -100
"chr13" 111766001 111767000 "*" 0 0 86.9033646322379
"chr13" 111767001 111768000 "*" 0 0 70.9421511037787
"chr13" 111856001 111857000 "*" 0.000285593505977677 0.000248445226337853 52.1739130434783
"chr13" 111975001 111976000 "*" 4.7310932949074e-11 1.10311661064467e-10 56.989247311828
"chr13" 111976001 111977000 "*" 1.19273531109521e-05 1.29198322266291e-05 -75
"chr13" 111979001 111980000 "*" 0 0 61.0644257703081
"chr13" 112010001 112011000 "*" 1.67299207820548e-08 2.71377429441055e-08 100
"chr13" 112013001 112014000 "*" 8.7349629751543e-09 1.47505871898511e-08 -100
"chr13" 112042001 112043000 "*" 1.96587190970376e-12 5.5110953755443e-12 73.4042553191489
"chr13" 112043001 112044000 "*" 3.86908316065693e-05 3.86920828116388e-05 -60.3413654618474
"chr13" 112057001 112058000 "*" 0 0 -64.5833333333333
"chr13" 112060001 112061000 "*" 0 0 -73.8095238095238
"chr13" 112066001 112067000 "*" 1.38033362517831e-10 3.01869064376388e-10 66.6666666666667
"chr13" 112077001 112078000 "*" 6.01080296647183e-11 1.38222443132037e-10 58.0645161290323
"chr13" 112121001 112122000 "*" 4.65771448077135e-08 7.09509833757406e-08 60.9090909090909
"chr13" 112128001 112129000 "*" 5.11368725142347e-13 1.54127093655369e-12 69.5652173913043

"chr13" 112131001 112132000 "*" 1.11022302462516e-16 5.03662826618488e-16 82.258064516129
"chr13" 112137001 112138000 "*" 3.15303338993544e-14 1.1025793806681e-13 70.1754385964912
"chr13" 112147001 112148000 "*" 1.83466277617894e-05 1.93097202505878e-05 65
"chr13" 112158001 112159000 "*" 3.42280648268911e-11 8.12872163131922e-11 72.2222222222222
"chr13" 112159001 112160000 "*" 0 0 100
"chr13" 112161001 112162000 "*" 0 0 63.6012636012636
"chr13" 112167001 112168000 "*" 0 0 99.236641221374
"chr13" 112169001 112170000 "*" 0 0 59.609796596098
"chr13" 112207001 112208000 "*" 9.86151402972979e-05 9.25440119785655e-05 -53.4246575342466
"chr13" 112214001 112215000 "*" 1.44362299892009e-12 4.12255313375858e-12 80
"chr13" 112219001 112220000 "*" 9.99200722162641e-16 4.15382497462808e-15 100
"chr13" 112221001 112222000 "*" 2.16015464068464e-05 2.24925309279237e-05 -63.8888888888889
"chr13" 112236001 112237000 "*" 1.17579279645952e-11 2.97490940498961e-11 62.0915032679739
"chr13" 112243001 112244000 "*" 0 0 58.1539933046389
"chr13" 112247001 112248000 "*" 0 0 100
"chr13" 112263001 112264000 "*" 0 0 -100
"chr13" 112281001 112282000 "*" 1.08473570126222e-08 1.81076666826488e-08 55.1020408163265
"chr13" 112308001 112309000 "*" 0 0 -100
"chr13" 112315001 112316000 "*" 1.4751422305892e-11 3.6855663449439e-11 66.6666666666667
"chr13" 112316001 112317000 "*" 1.71229154376507e-05 1.8115731087825e-05 63.6363636363636
"chr13" 112324001 112325000 "*" 2.52475818030007e-12 6.98257699684869e-12 71.4285714285714
"chr13" 112328001 112329000 "*" 6.99440505513849e-15 2.64480529781883e-14 -100
"chr13" 112331001 112332000 "*" 8.67677041327397e-11 1.95137714354733e-10 -80
"chr13" 112334001 112335000 "*" 1.91069382537989e-13 6.08032649314634e-13 -51.219512195122
"chr13" 112341001 112342000 "*" 0 0 -100
"chr13" 112353001 112354000 "*" 0 0 -75.5555555555556
"chr13" 112538001 112539000 "*" 1.66533453693773e-15 6.7629186866784e-15 -100
"chr13" 112555001 112556000 "*" 1.22124532708767e-15 5.03921984217778e-15 -100
"chr13" 112568001 112569000 "*" 4.13225009765483e-13 1.25975998171404e-12 81.0810810810811
"chr13" 112593001 112594000 "*" 0 0 100
"chr13" 112596001 112597000 "*" 3.10862446895044e-15 1.22466437093774e-14 100
"chr13" 112597001 112598000 "*" 7.67035336135535e-07 9.8945573498661e-07 65.4320987654321
"chr13" 112599001 112600000 "*" 4.29056790096638e-12 1.1526376335526e-11 59.0909090909091
"chr13" 112617001 112618000 "*" 1.97625914188926e-07 2.75598009909371e-07 -50.3205128205128
"chr13" 112635001 112636000 "*" 1.93508449353175e-06 2.35946484584504e-06 57.5268817204301
"chr13" 112782001 112783000 "*" 0 0 -93.4426229508197

"chr13" 112808001 112809000 "*" 4.73959188673767e-07 6.28368274925326e-07
-73.8095238095238
"chr13" 112814001 112815000 "*" 4.1451987620178e-08 6.35197269375315e-08
77.3955773955774
"chr13" 112827001 112828000 "*" 1.51094193590051e-05 1.61080928716407e-05
64.2857142857143
"chr13" 112859001 112860000 "*" 2.61776622600962e-09 4.77711122078405e-09
65.1162790697674
"chr13" 112883001 112884000 "*" 1.26565424807268e-14 4.63923203146481e-14 100
"chr13" 112884001 112885000 "*" 0.000194225706850348 0.000173761410290447
-52.5252525252525
"chr13" 112893001 112894000 "*" 1.63068881064987e-07 2.29679668525646e-07
71.4285714285714
"chr13" 112899001 112900000 "*" 6.87628248341454e-07 8.92892153029571e-07
69.6969696969697
"chr13" 112903001 112904000 "*" 1.27881660816342e-09 2.43825605635895e-09
-55.6133056133056
"chr13" 112926001 112927000 "*" 2.21417562062953e-08 3.52391637833357e-08
78.3950617283951
"chr13" 112977001 112978000 "*" 1.39779633423487e-07 1.98463037958589e-07 100
"chr13" 112978001 112979000 "*" 1.86616252451088e-08 3.00920815921843e-08
-64.5161290322581
"chr13" 113004001 113005000 "*" 1.11022302462516e-16 5.03662826618488e-16
86.2068965517241
"chr13" 113028001 113029000 "*" 0 0 -100
"chr13" 113044001 113045000 "*" 6.92287338566189e-11 1.57605630124247e-10 -100
"chr13" 113098001 113099000 "*" 0 0 86
"chr13" 113111001 113112000 "*" 2.54787302367276e-11 6.17249712323817e-11
86.6666666666667
"chr13" 113112001 113113000 "*" 0 0 100
"chr13" 113116001 113117000 "*" 0 0 -100
"chr13" 113123001 113124000 "*" 2.76029128443156e-08 4.34408078199365e-08
-79.3103448275862
"chr13" 113152001 113153000 "*" 1.52794177310511e-09 2.87642564232045e-09 100
"chr13" 113255001 113256000 "*" 0 0 100
"chr13" 113270001 113271000 "*" 4.99020824662466e-11 1.15790792586157e-10 -100
"chr13" 113273001 113274000 "*" 3.33066907387547e-16 1.4495649018245e-15
-64.8648648648649
"chr13" 113357001 113358000 "*" 0 0 -96.0526315789474
"chr13" 113527001 113528000 "*" 2.32036612146658e-14 8.26479401047126e-14
-59.4857872884063
"chr13" 113583001 113584000 "*" 1.24344978758018e-14 4.56627092294518e-14 100
"chr13" 113585001 113586000 "*" 0 0 65.0602409638554
"chr13" 113609001 113610000 "*" 0 0 58.7301587301587
"chr13" 113611001 113612000 "*" 0 0 51.3919413919414
"chr13" 113615001 113616000 "*" 1.92068583260152e-14 6.89775382589628e-14 100
"chr13" 113711001 113712000 "*" 8.58968451922237e-12 2.213554983903e-11 100
"chr13" 113769001 113770000 "*" 0 0 -56.9620253164557
"chr13" 113800001 113801000 "*" 2.97547997352154e-09 5.38628893061017e-09
-73.6842105263158
"chr13" 113881001 113882000 "*" 0 0 100
"chr13" 113944001 113945000 "*" 5.55111512312578e-16 2.36485094870365e-15

50.5747126436782
"chr13" 113981001 113982000 "*" 0 0 67.7966101694915
"chr13" 114027001 114028000 "*" 5.6843418860808e-14 1.92706831069135e-13
54.2253521126761
"chr13" 114037001 114038000 "*" 0 0 100
"chr13" 114047001 114048000 "*" 6.2349386986682e-07 8.14445235079435e-07
51.0344827586207
"chr13" 114065001 114066000 "*" 0 0 -77.8135048231511
"chr13" 114075001 114076000 "*" 0 0 -56.9723277265328
"chr13" 114146001 114147000 "*" 1.97619698383278e-14 7.08978387753544e-14
-57.9487179487179
"chr13" 114248001 114249000 "*" 1.11022302462516e-16 5.03662826618488e-16
-53.7553891536192
"chr13" 114319001 114320000 "*" 0 0 -90
"chr13" 114440001 114441000 "*" 1.7311759359373e-07 2.43114264397939e-07
-51.0760401721664
"chr13" 114456001 114457000 "*" 3.23395754620037e-11 7.7026020483071e-11
69.3877551020408
"chr13" 114462001 114463000 "*" 0 0 51.2971698113208
"chr13" 114476001 114477000 "*" 9.65729496371637e-10 1.87255912493232e-09 100
"chr13" 114486001 114487000 "*" 1.86395714457666e-05 1.95989970724601e-05
69.2307692307692
"chr13" 114540001 114541000 "*" 0 0 -92.5465838509317
"chr13" 114552001 114553000 "*" 1.98325800226939e-11 4.85612402473442e-11 100
"chr13" 114553001 114554000 "*" 1.90181204118289e-13 6.05298617539364e-13 100
"chr13" 114563001 114564000 "*" 9.2341220447878e-08 1.34572624316514e-07
66.2921348314607
"chr13" 114582001 114583000 "*" 1.11022302462516e-16 5.03662826618488e-16 100
"chr13" 114583001 114584000 "*" 1.13882125951648e-10 2.521329619527e-10
73.6842105263158
"chr13" 114616001 114617000 "*" 0 0 -56.7194052611514
"chr13" 114630001 114631000 "*" 6.33112674508496e-08 9.47206714761097e-08
-79.5620437956204
"chr13" 114923001 114924000 "*" 5.28422483547786e-08 7.98774825047459e-08
78.5714285714286
"chr13" 114950001 114951000 "*" 0 0 81.5789473684211
"chr13" 114962001 114963000 "*" 6.24654217240561e-10 1.2462108191303e-09 -100
"chr13" 115047001 115048000 "*" 0 0 59.6685082872928
"chr13" 115093001 115094000 "*" 5.794809077031e-12 1.52911192757929e-11 100
"chr14" 19500001 19501000 "*" 6.10366818198926e-08 9.15379983945833e-08
-62.962962962963
"chr14" 19961001 19962000 "*" 5.88418203051333e-15 2.245355428447e-14 100
"chr14" 20838001 20839000 "*" 1.12458486967171e-09 2.15740815043925e-09 100
"chr14" 21070001 21071000 "*" 2.07117875350438e-06 2.51507333310658e-06
-73.5294117647059
"chr14" 21233001 21234000 "*" 3.66373598126302e-15 1.43441054525534e-14
66.6666666666667
"chr14" 21237001 21238000 "*" 1.88261159994596e-08 3.03420719188995e-08
91.6666666666667
"chr14" 21254001 21255000 "*" 2.58681964737661e-14 9.14008479605639e-14 -100
"chr14" 21298001 21299000 "*" 9.80763359414993e-10 1.8980032424715e-09 100
"chr14" 21551001 21552000 "*" 0 0 -100

"chr14" 21565001 21566000 "*" 2.81191514517332e-09 5.10293735854033e-09 -80
"chr14" 21809001 21810000 "*" 2.91988655476416e-14 1.02578169224843e-13
-80.2325581395349
"chr14" 21993001 21994000 "*" 1.5710649408085e-06 1.94167201695392e-06
52.112676056338
"chr14" 22144001 22145000 "*" 7.80423503599081e-11 1.76499889176712e-10
-51.4018691588785
"chr14" 22855001 22856000 "*" 4.47974990436251e-13 1.35889381055418e-12
-94.3060498220641
"chr14" 23071001 23072000 "*" 8.7349629751543e-09 1.47505871898511e-08 100
"chr14" 23107001 23108000 "*" 0 0 -50.7462686567164
"chr14" 23199001 23200000 "*" 2.69007038866675e-13 8.38831022628993e-13 -100
"chr14" 23265001 23266000 "*" 1.5277158427196e-09 2.87642564232045e-09 -100
"chr14" 23290001 23291000 "*" 4.44089209850063e-16 1.91071758245033e-15
-77.4193548387097
"chr14" 23316001 23317000 "*" 0 0 -94.1908713692946
"chr14" 23356001 23357000 "*" 0 0 -77.992277992278
"chr14" 23357001 23358000 "*" 3.95861121660346e-12 1.0684496262032e-11 100
"chr14" 23399001 23400000 "*" 0 0 62.1052631578947
"chr14" 23525001 23526000 "*" 4.1014197593725e-09 7.26553281681452e-09
-62.962962962963
"chr14" 23584001 23585000 "*" 0 0 -100
"chr14" 23590001 23591000 "*" 2.25751195515045e-10 4.79787142543627e-10
86.7469879518072
"chr14" 23596001 23597000 "*" 9.08785219855268e-07 1.16030312500671e-06
-76.4705882352941
"chr14" 23707001 23708000 "*" 0 0 75.8928571428571
"chr14" 23746001 23747000 "*" 6.52733422867868e-12 1.71146951718423e-11 100
"chr14" 23776001 23777000 "*" 0 0 -100
"chr14" 23787001 23788000 "*" 3.95353005888666e-09 7.03928529283733e-09 100
"chr14" 23822001 23823000 "*" 0 0 100
"chr14" 23828001 23829000 "*" 0 0 87.1705572289157
"chr14" 23868001 23869000 "*" 2.15125472990962e-10 4.58126659943874e-10 -100
"chr14" 23880001 23881000 "*" 1.01597855683977e-08 1.70219007506586e-08
81.6326530612245
"chr14" 23905001 23906000 "*" 5.2583356327407e-05 5.15382873150132e-05
-54.7169811320755
"chr14" 23912001 23913000 "*" 0 0 -100
"chr14" 24001001 24002000 "*" 2.67338817749874e-10 5.6303645776248e-10
83.3333333333333
"chr14" 24007001 24008000 "*" 3.10336944800982e-05 3.15338093902317e-05
51.8518518518519
"chr14" 24018001 24019000 "*" 2.41784284078506e-05 2.49875818383709e-05
-67.741935483871
"chr14" 24021001 24022000 "*" 0 0 59.1836734693878
"chr14" 24423001 24424000 "*" 0 0 100
"chr14" 24507001 24508000 "*" 0 0 -90.2097902097902
"chr14" 24512001 24513000 "*" 2.198362416439e-06 2.6598331334668e-06
-58.2089552238806
"chr14" 24528001 24529000 "*" 8.86200736127662e-08 1.29452763525729e-07
79.6296296296296
"chr14" 24538001 24539000 "*" 2.38575468822777e-05 2.46816531377115e-05

66.6666666666667
"chr14" 24604001 24605000 "*" 6.4152538836737e-10 1.27477697728417e-09 100
"chr14" 24647001 24648000 "*" 5.99520433297585e-15 2.28531569014092e-14 100
"chr14" 24680001 24681000 "*" 2.41774710252329e-08 3.8295626817888e-08
-64.2857142857143
"chr14" 24734001 24735000 "*" 0 0 95.5555555555556
"chr14" 24765001 24766000 "*" 4.44089209850063e-15 1.71914916534614e-14 100
"chr14" 24814001 24815000 "*" 4.90718576884319e-14 1.6794399166207e-13 -100
"chr14" 24836001 24837000 "*" 1.96644922567657e-11 4.82930612417392e-11 -100
"chr14" 24889001 24890000 "*" 6.4324989779152e-11 1.47230020825092e-10 100
"chr14" 24899001 24900000 "*" 0 0 -55.5555555555556
"chr14" 24942001 24943000 "*" 3.89943710654705e-09 6.96378605330768e-09
-59.6153846153846
"chr14" 25148001 25149000 "*" 4.44089209850063e-16 1.91071758245033e-15 -100
"chr14" 25155001 25156000 "*" 2.66324188131772e-05 2.73400755089686e-05
-63.6363636363636
"chr14" 25166001 25167000 "*" 3.60623352069211e-07 4.85790136098343e-07
62.2950819672131
"chr14" 25195001 25196000 "*" 1.39794525955139e-08 2.2957489213758e-08
58.5641025641026
"chr14" 25395001 25396000 "*" 9.04570973681018e-10 1.76414389822173e-09 -100
"chr14" 25519001 25520000 "*" 0 0 59.0214067278287
"chr14" 26006001 26007000 "*" 1.56863411149288e-12 4.4469621057462e-12 -100
"chr14" 26315001 26316000 "*" 3.46500605985511e-13 1.06777978272295e-12 -100
"chr14" 26633001 26634000 "*" 1.29037891483108e-11 3.24582280286866e-11 -100
"chr14" 30400001 30401000 "*" 1.0030847508169e-07 1.45542740671197e-07
-63.9344262295082
"chr14" 30555001 30556000 "*" 8.17846470546613e-08 1.20022814812237e-07 -70
"chr14" 30708001 30709000 "*" 9.65338919911574e-13 2.80678910656113e-12 100
"chr14" 31926001 31927000 "*" 0 0 -80
"chr14" 32670001 32671000 "*" 0 0 50.6457739791073
"chr14" 32979001 32980000 "*" 2.05391259555654e-14 7.35435952190685e-14 -100
"chr14" 33408001 33409000 "*" 0 0 64.7909886515016
"chr14" 33409001 33410000 "*" 0 0 84.3076923076923
"chr14" 34035001 34036000 "*" 1.4432899320127e-15 5.90750815956055e-15 -100
"chr14" 34320001 34321000 "*" 1.30129695907755e-09 2.47885143099023e-09
66.6666666666667
"chr14" 34419001 34420000 "*" 0 0 74.2236024844721
"chr14" 34556001 34557000 "*" 2.70339306496226e-13 8.42355472617862e-13 -100
"chr14" 34735001 34736000 "*" 9.43689570931383e-15 3.51625644188498e-14 -100
"chr14" 35186001 35187000 "*" 0 0 100
"chr14" 35348001 35349000 "*" 0.000153647307420512 0.000139750611014767
-51.3888888888889
"chr14" 35806001 35807000 "*" 8.77076189453874e-15 3.27889038038365e-14
83.3333333333333
"chr14" 35809001 35810000 "*" 0.000102952062572492 9.63396776775729e-05 -58
"chr14" 35872001 35873000 "*" 4.71134242729931e-12 1.257615036806e-11 100
"chr14" 35882001 35883000 "*" 0 0 -100
"chr14" 36002001 36003000 "*" 0 0 64.6532438478747
"chr14" 36380001 36381000 "*" 6.77335965093562e-12 1.76897011246462e-11 100
"chr14" 36418001 36419000 "*" 2.52448983939502e-09 4.61486995810145e-09
-64.1215106732348

"chr14" 36484001 36485000 "*" 7.98734522489131e-06 8.88247584795875e-06 -55.7692307692308
"chr14" 36650001 36651000 "*" 4.3412176120583e-07 5.78408567117475e-07 -71.4285714285714
"chr14" 36663001 36664000 "*" 2.02327044007689e-12 5.65300384792621e-12 -100
"chr14" 36989001 36990000 "*" 0 0 73.6932348697055
"chr14" 37218001 37219000 "*" 1.11022302462516e-16 5.03662826618488e-16 -100
"chr14" 37666001 37667000 "*" 1.41135214626331e-10 3.08070960709898e-10 53.5714285714286
"chr14" 38072001 38073000 "*" 2.00128236205188e-08 3.20459176617961e-08 78.8732394366197
"chr14" 38343001 38344000 "*" 4.43807576466781e-07 5.9054934342361e-07 65.2777777777778
"chr14" 39304001 39305000 "*" 9.00643751564623e-05 8.50604330368962e-05 52.7836504580691
"chr14" 40283001 40284000 "*" 0 0 -100
"chr14" 42079001 42080000 "*" 4.44089209850063e-16 1.91071758245033e-15 56.0975609756098
"chr14" 42352001 42353000 "*" 0 0 100
"chr14" 42388001 42389000 "*" 3.6700841921089e-08 5.65530226072258e-08 -100
"chr14" 42480001 42481000 "*" 7.0006535457523e-08 1.03695832512054e-07 100
"chr14" 42698001 42699000 "*" 1.61237689866311e-12 4.56615813671789e-12 100
"chr14" 42848001 42849000 "*" 8.06688049692639e-13 2.37556391305009e-12 100
"chr14" 44067001 44068000 "*" 1.87510396266743e-05 1.97080033495427e-05 -66.6666666666667
"chr14" 44135001 44136000 "*" 9.88421286296504e-06 1.0841293662501e-05 -64.7058823529412
"chr14" 44442001 44443000 "*" 1.13140401492018e-08 1.87801380787728e-08 100
"chr14" 45996001 45997000 "*" 1.11022302462516e-16 5.03662826618488e-16 -56
"chr14" 46237001 46238000 "*" 2.42908371861361e-07 3.34866070167804e-07 -79.3103448275862
"chr14" 46977001 46978000 "*" 0 0 96.078431372549
"chr14" 47711001 47712000 "*" 3.46389583683049e-13 1.06777978272295e-12 -63.4920634920635
"chr14" 48009001 48010000 "*" 4.22251122955686e-12 1.13508583241713e-11 90
"chr14" 48736001 48737000 "*" 0 0 67.0103092783505
"chr14" 49717001 49718000 "*" 0 0 -100
"chr14" 49840001 49841000 "*" 3.6700841921089e-08 5.65530226072258e-08 -100
"chr14" 50088001 50089000 "*" 0 0 54.4715447154472
"chr14" 50159001 50160000 "*" 0 0 -81.9862557054208
"chr14" 50161001 50162000 "*" 7.0006535457523e-08 1.03695832512054e-07 100
"chr14" 50466001 50467000 "*" 5.55111512312578e-15 2.12516350804551e-14 -55.8132956152758
"chr14" 50470001 50471000 "*" 0 0 89.7297297297297
"chr14" 50512001 50513000 "*" 4.86832796298131e-13 1.47290845675726e-12 100
"chr14" 51298001 51299000 "*" 0 0 -100
"chr14" 51326001 51327000 "*" 4.04332123338236e-12 1.08828441533745e-11 -52.6785714285714
"chr14" 51410001 51411000 "*" 0 0 93.1706104181343
"chr14" 51562001 51563000 "*" 0 0 77.1048002010555
"chr14" 51832001 51833000 "*" 6.60582699651968e-14 2.22176301799e-13 100
"chr14" 51892001 51893000 "*" 0 0 100

"chr14" 51938001 51939000 "*" 1.70641278884887e-12 4.81986032810646e-12 -100
"chr14" 51975001 51976000 "*" 5.01025332333427e-10 1.01358031135889e-09 -100
"chr14" 52233001 52234000 "*" 1.17905685215192e-13 3.83653245040640e-13 -100
"chr14" 52712001 52713000 "*" 4.46405532761318e-05 4.42311628182131e-05
-52.3809523809524
"chr14" 53411001 53412000 "*" 3.2700897545368e-10 6.78798757234889e-10
66.6666666666667
"chr14" 53417001 53418000 "*" 0 0 75.9982925731878
"chr14" 53418001 53419000 "*" 0 0 86.530612244898
"chr14" 53684001 53685000 "*" 0 0 81.25
"chr14" 54295001 54296000 "*" 2.43005615629954e-09 4.45014222280278e-09 -100
"chr14" 54421001 54422000 "*" 0 0 -82.5059101654846
"chr14" 54423001 54424000 "*" 0 0 -85.9569648924122
"chr14" 54782001 54783000 "*" 3.34128893442198e-08 5.19772864618523e-08 100
"chr14" 55119001 55120000 "*" 3.34128893442198e-08 5.19772864618523e-08 -100
"chr14" 55138001 55139000 "*" 8.77076189453874e-15 3.27889038038365e-14 -100
"chr14" 55286001 55287000 "*" 8.01666433236647e-09 1.36392368970109e-08 -100
"chr14" 55337001 55338000 "*" 2.08614236996141e-11 5.09698923781446e-11
66.6666666666667
"chr14" 55595001 55596000 "*" 0 0 -70.6199460916442
"chr14" 55596001 55597000 "*" 0 0 -88.8524590163934
"chr14" 55605001 55606000 "*" 1.45991774225251e-10 3.18130685397763e-10
58.4415584415584
"chr14" 56614001 56615000 "*" 1.39779633423487e-07 1.98463037958589e-07 100
"chr14" 56691001 56692000 "*" 0 0 100
"chr14" 56777001 56778000 "*" 4.67611505072796e-11 1.09076258416177e-10
64.2857142857143
"chr14" 56879001 56880000 "*" 1.13140401492018e-08 1.87801380787728e-08 100
"chr14" 57001001 57002000 "*" 0 0 -100
"chr14" 57023001 57024000 "*" 8.3882012447134e-11 1.88922989503423e-10 100
"chr14" 57191001 57192000 "*" 0 0 -100
"chr14" 57242001 57243000 "*" 0 0 -100
"chr14" 57270001 57271000 "*" 5.63660229602192e-13 1.68905058935478e-12 100
"chr14" 58222001 58223000 "*" 1.93720595120794e-11 4.76309924836636e-11 100
"chr14" 58427001 58428000 "*" 2.95652391457679e-13 9.17765865497751e-13
-55.2941176470588
"chr14" 58528001 58529000 "*" 2.26502835752873e-05 2.35038252445516e-05
55.1724137931034
"chr14" 58862001 58863000 "*" 0 0 -55.3263882063882
"chr14" 59098001 59099000 "*" 6.97978544184075e-07 9.05274980247362e-07 -68.75
"chr14" 59249001 59250000 "*" 1.12132525487141e-14 4.14352271980498e-14 -100
"chr14" 60097001 60098000 "*" 0 0 64.283900246133
"chr14" 60230001 60231000 "*" 6.15840711759574e-13 1.83676803572802e-12 100
"chr14" 60366001 60367000 "*" 5.10702591327572e-15 1.96271840288234e-14
66.6666666666667
"chr14" 60810001 60811000 "*" 6.4324989779152e-11 1.47230020825092e-10 -100
"chr14" 60981001 60982000 "*" 0 0 53.5644842123376
"chr14" 61117001 61118000 "*" 4.16429224436854e-10 8.52658537675143e-10
70.8333333333333
"chr14" 61155001 61156000 "*" 8.52140580320793e-12 2.19817259951489e-11 100
"chr14" 61190001 61191000 "*" 0 0 66.9527821660523
"chr14" 61546001 61547000 "*" 1.39779633423487e-07 1.98463037958589e-07 -100

"chr14" 61615001 61616000 "*" 1.9519538673185e-06 2.378434283772e-06
-54.2857142857143
"chr14" 61748001 61749000 "*" 0 0 76.9784172661871
"chr14" 62020001 62021000 "*" 0 0 -100
"chr14" 62034001 62035000 "*" 0 0 -80.5555555555556
"chr14" 62081001 62082000 "*" 3.55123774831156e-08 5.50471549649875e-08
-76.1904761904762
"chr14" 62217001 62218000 "*" 0 0 -100
"chr14" 62330001 62331000 "*" 2.22996458265623e-06 2.6961916005055e-06
77.7777777777778
"chr14" 62805001 62806000 "*" 5.01025332333427e-10 1.01358031135889e-09 100
"chr14" 63174001 63175000 "*" 2.88710388929303e-10 6.03399264731139e-10 -100
"chr14" 63445001 63446000 "*" 1.5277158427196e-09 2.87642564232045e-09 -100
"chr14" 63561001 63562000 "*" 8.01666433236647e-09 1.36392368970109e-08 100
"chr14" 63671001 63672000 "*" 3.08941761062442e-12 8.44231919910001e-12
-56.8957871396896
"chr14" 64204001 64205000 "*" 0 0 -100
"chr14" 64319001 64320000 "*" 0 0 -100
"chr14" 64611001 64612000 "*" 2.88710388929303e-10 6.03399264731139e-10 -100
"chr14" 65006001 65007000 "*" 0 0 -57.261738443783
"chr14" 65072001 65073000 "*" 0 0 -100
"chr14" 65190001 65191000 "*" 2.69007038866675e-13 8.38831022628993e-13 100
"chr14" 65226001 65227000 "*" 8.7349629751543e-09 1.47505871898511e-08 100
"chr14" 65236001 65237000 "*" 2.19834594972212e-10 4.6764164767468e-10
62.2641509433962
"chr14" 65289001 65290000 "*" 0 0 -80
"chr14" 65619001 65620000 "*" 1.37828859436695e-10 3.01445544267661e-10 100
"chr14" 65641001 65642000 "*" 2.22044604925031e-16 9.81641919380259e-16 100
"chr14" 65696001 65697000 "*" 2.29329899514852e-09 4.22120358762507e-09
-66.6666666666667
"chr14" 65711001 65712000 "*" 2.15125472990962e-10 4.58126659943874e-10 100
"chr14" 65718001 65719000 "*" 1.13140401492018e-08 1.87801380787728e-08 -100
"chr14" 65772001 65773000 "*" 8.65973959207622e-14 2.87362558212404e-13 100
"chr14" 65996001 65997000 "*" 2.64951482975562e-09 4.82186011375752e-09 100
"chr14" 66265001 66266000 "*" 1.10777798045802e-09 2.13104801535919e-09
-82.089552238806
"chr14" 66295001 66296000 "*" 2.64951482975562e-09 4.82186011375752e-09 100
"chr14" 66296001 66297000 "*" 2.08814465718632e-09 3.86232567960463e-09 -100
"chr14" 66413001 66414000 "*" 6.59550958292954e-09 1.13456194382773e-08 -100
"chr14" 67674001 67675000 "*" 1.97619698383278e-14 7.08978387753544e-14
-66.6666666666667
"chr14" 67878001 67879000 "*" 0 0 -81.6124469589816
"chr14" 67894001 67895000 "*" 0 0 -53.0216247808299
"chr14" 67913001 67914000 "*" 0 0 100
"chr14" 67931001 67932000 "*" 2.40918396343659e-14 8.55630122551753e-14 -80
"chr14" 67999001 68000000 "*" 0 0 -60
"chr14" 68024001 68025000 "*" 1.74853465040314e-11 4.32752710900609e-11 -100
"chr14" 68041001 68042000 "*" 0 0 -100
"chr14" 68284001 68285000 "*" 2.02060590481778e-13 6.40127405465654e-13 100
"chr14" 68994001 68995000 "*" 8.58968451922237e-12 2.213554983903e-11 -100
"chr14" 69041001 69042000 "*" 1.12634901405784e-10 2.49563754426169e-10 100
"chr14" 69144001 69145000 "*" 2.43005615629954e-09 4.45014222280278e-09 -100

"chr14" 69152001 69153000 "*" 4.21884749357559e-15 1.63874445239189e-14 -100
"chr14" 69174001 69175000 "*" 4.73234496034536e-09 8.30627300264826e-09 100
"chr14" 69261001 69262000 "*" 4.49550383363473e-07 5.9779038721134e-07
-74.6268656716418
"chr14" 69408001 69409000 "*" 1.13140401492018e-08 1.87801380787728e-08 100
"chr14" 69415001 69416000 "*" 7.506772981003e-12 1.95000965118339e-11
66.6666666666667
"chr14" 69437001 69438000 "*" 2.89801516117905e-12 7.94270405097209e-12
66.6666666666667
"chr14" 69522001 69523000 "*" 2.22044604925031e-16 9.81641919380259e-16
-61.4035087719298
"chr14" 69726001 69727000 "*" 0 0 78.3229825988611
"chr14" 69727001 69728000 "*" 0 0 68.6545976819533
"chr14" 69993001 69994000 "*" 4.65637306490407e-10 9.46720524046618e-10 100
"chr14" 70009001 70010000 "*" 1.11022302462516e-16 5.03662826618488e-16
65.7142857142857
"chr14" 70010001 70011000 "*" 1.41502588846265e-06 1.76038045507763e-06
58.3333333333333
"chr14" 70015001 70016000 "*" 1.15190845750845e-09 2.20743935574765e-09
67.0588235294118
"chr14" 70070001 70071000 "*" 0 0 100
"chr14" 70152001 70153000 "*" 3.61932706027801e-14 1.25777907157994e-13 100
"chr14" 70170001 70171000 "*" 2.00227675550835e-08 3.20459176617961e-08 -100
"chr14" 70317001 70318000 "*" 0 0 71.8367346938775
"chr14" 70358001 70359000 "*" 4.01313771103418e-09 7.11882087237587e-09 100
"chr14" 70415001 70416000 "*" 2.03553217629215e-05 2.12753843906822e-05
56.5217391304348
"chr14" 70449001 70450000 "*" 6.77335965093562e-12 1.76897011246462e-11 -100
"chr14" 70493001 70494000 "*" 2.08000963120014e-08 3.32173431651681e-08
-88.8888888888889
"chr14" 70544001 70545000 "*" 6.4324989779152e-11 1.47230020825092e-10 100
"chr14" 70655001 70656000 "*" 0 0 65.9998714611652
"chr14" 70656001 70657000 "*" 0 0 100
"chr14" 70692001 70693000 "*" 4.73234496034536e-09 8.30627300264826e-09 100
"chr14" 70777001 70778000 "*" 1.67299207820548e-08 2.71377429441055e-08 -100
"chr14" 70913001 70914000 "*" 2.1094237467878e-15 8.47434540879246e-15 -100
"chr14" 71137001 71138000 "*" 1.98365768255826e-11 4.85612402473442e-11 100
"chr14" 71166001 71167000 "*" 0.000184006388740388 0.000165249884973406
52.1739130434783
"chr14" 71247001 71248000 "*" 4.99900121297969e-12 1.32921056339624e-11 -100
"chr14" 71338001 71339000 "*" 1.11022302462516e-16 5.03662826618488e-16 100
"chr14" 71351001 71352000 "*" 1.70641278884887e-12 4.81986032810646e-12 -100
"chr14" 71632001 71633000 "*" 0 0 100
"chr14" 71682001 71683000 "*" 4.62050709360007e-07 6.13385849410841e-07
-64.2857142857143
"chr14" 71745001 71746000 "*" 5.6621374255883e-15 2.16468580166064e-14 100
"chr14" 72315001 72316000 "*" 5.51780843238703e-14 1.87243792572872e-13 -100
"chr14" 72462001 72463000 "*" 0.000131523009637924 0.000120961047510062 -55
"chr14" 72776001 72777000 "*" 2.68673971959288e-14 9.47266815431045e-14 100
"chr14" 73005001 73006000 "*" 5.27066615907401e-08 7.96829356899468e-08
91.6666666666667
"chr14" 73028001 73029000 "*" 5.81201753391269e-13 1.73949010668347e-12

-66.6666666666667
"chr14" 73068001 73069000 "*" 2.88710388929303e-10 6.03399264731139e-10 100
"chr14" 73071001 73072000 "*" 1.20762375233063e-06 1.51606374274134e-06
68.2926829268293
"chr14" 73080001 73081000 "*" 4.7867338970331e-07 6.34301921289258e-07
58.3333333333333
"chr14" 73085001 73086000 "*" 7.90449091736534e-08 1.16206605825357e-07
-72.972972972973
"chr14" 73114001 73115000 "*" 0 0 -79.1666666666667
"chr14" 73117001 73118000 "*" 6.46149800331841e-14 2.17664365839349e-13 100
"chr14" 73155001 73156000 "*" 0 0 100
"chr14" 73206001 73207000 "*" 2.67283972732457e-11 6.45686178157698e-11 -100
"chr14" 73207001 73208000 "*" 1.96509475358653e-13 6.23868565133654e-13 100
"chr14" 73250001 73251000 "*" 1.63823263366059e-05 1.73784991598845e-05
64.8648648648649
"chr14" 73283001 73284000 "*" 7.0006535457523e-08 1.03695832512054e-07 100
"chr14" 73299001 73300000 "*" 2.84189433263915e-06 3.38038891121791e-06
67.741935483871
"chr14" 73302001 73303000 "*" 0 0 -72.7415966386555
"chr14" 73418001 73419000 "*" 1.5819080489976e-09 2.9737238837933e-09
58.8235294117647
"chr14" 73458001 73459000 "*" 1.11022302462516e-16 5.03662826618488e-16 100
"chr14" 73524001 73525000 "*" 0 0 -100
"chr14" 73526001 73527000 "*" 0 0 -100
"chr14" 73604001 73605000 "*" 0 0 -68.3235867446394
"chr14" 73733001 73734000 "*" 1.37828859436695e-10 3.01445544267661e-10 100
"chr14" 73743001 73744000 "*" 4.65637306490407e-10 9.46720524046618e-10 -100
"chr14" 73957001 73958000 "*" 0 0 -65.1162790697674
"chr14" 74036001 74037000 "*" 0 0 -59.1836734693878
"chr14" 74428001 74429000 "*" 0 0 -100
"chr14" 74556001 74557000 "*" 2.08100203735739e-12 5.80885669425549e-12
-68.4210526315789
"chr14" 74601001 74602000 "*" 0 0 -80
"chr14" 74730001 74731000 "*" 0.000199851262950967 0.000178453492150885
54.5454545454545
"chr14" 74761001 74762000 "*" 1.87405646556726e-13 5.97285292681429e-13 -100
"chr14" 74770001 74771000 "*" 1.88737914186277e-15 7.62011598380321e-15 -100
"chr14" 74870001 74871000 "*" 3.6700841921089e-08 5.65530226072258e-08 100
"chr14" 74889001 74890000 "*" 2.43005615629954e-09 4.45014222280278e-09 100
"chr14" 74895001 74896000 "*" 0 0 100
"chr14" 74922001 74923000 "*" 3.05311331771918e-12 8.34639368772922e-12 -100
"chr14" 74979001 74980000 "*" 9.65729496371637e-10 1.87255912493232e-09 100
"chr14" 74982001 74983000 "*" 1.66533453693773e-15 6.7629186866784e-15 100
"chr14" 74984001 74985000 "*" 1.13140401492018e-08 1.87801380787728e-08 100
"chr14" 74987001 74988000 "*" 0 0 100
"chr14" 74988001 74989000 "*" 0 0 100
"chr14" 74989001 74990000 "*" 2.70339306496226e-13 8.42355472617862e-13 100
"chr14" 74991001 74992000 "*" 6.12778716657658e-11 1.40703078182493e-10
66.6666666666667
"chr14" 75013001 75014000 "*" 5.7835307716303e-06 6.56902676498958e-06
68.2926829268293
"chr14" 75018001 75019000 "*" 1.92512672470002e-13 6.1250871931855e-13

-56.1904761904762
"chr14" 75035001 75036000 "*" 0 0 100
"chr14" 75071001 75072000 "*" 0 0 75
"chr14" 75097001 75098000 "*" 1.2166162521865e-09 2.32508348709932e-09
57.4509803921569
"chr14" 75113001 75114000 "*" 1.92946192356658e-09 3.59193011611483e-09 -100
"chr14" 75361001 75362000 "*" 1.25235662118417e-06 1.56893911947026e-06 80
"chr14" 75377001 75378000 "*" 3.86282006381578e-10 7.9401066099168e-10
94.7368421052632
"chr14" 75421001 75422000 "*" 2.1197392729988e-09 3.91842836984148e-09
-62.6506024096386
"chr14" 75428001 75429000 "*" 1.65423230669148e-14 5.98698695225911e-14 -100
"chr14" 75469001 75470000 "*" 0 0 100
"chr14" 75518001 75519000 "*" 0 0 -58.5464303432102
"chr14" 75536001 75537000 "*" 0 0 -84.2857142857143
"chr14" 75760001 75761000 "*" 0 0 78.3726481766498
"chr14" 75777001 75778000 "*" 4.08209022140227e-11 9.57770690255255e-11 100
"chr14" 76009001 76010000 "*" 3.34128893442198e-08 5.19772864618523e-08 -100
"chr14" 76014001 76015000 "*" 1.41264777653305e-12 4.03686171442689e-12
-57.1428571428571
"chr14" 76330001 76331000 "*" 2.62927690508619e-07 3.60853669669655e-07
54.1666666666667
"chr14" 76530001 76531000 "*" 6.44564605500886e-06 7.26663863268796e-06
66.6666666666667
"chr14" 76584001 76585000 "*" 8.7349629751543e-09 1.47505871898511e-08 -100
"chr14" 76734001 76735000 "*" 0 0 -76.4705882352941
"chr14" 76770001 76771000 "*" 8.01666433236647e-09 1.36392368970109e-08 100
"chr14" 76781001 76782000 "*" 7.82696032430863e-08 1.15142528036045e-07 62.5
"chr14" 76806001 76807000 "*" 6.59550958292954e-09 1.13456194382773e-08 100
"chr14" 76819001 76820000 "*" 0 0 85.4401805869074
"chr14" 76842001 76843000 "*" 0 0 78.87760857304
"chr14" 76858001 76859000 "*" 3.17523785042795e-13 9.82732956433936e-13
51.2602662135372
"chr14" 76879001 76880000 "*" 6.73683331342545e-13 1.99842288354385e-12
97.7777777777778
"chr14" 76929001 76930000 "*" 2.57016630200724e-13 8.04510061985557e-13
69.2307692307692
"chr14" 76939001 76940000 "*" 6.66133814775094e-16 2.81595744474255e-15 -100
"chr14" 76940001 76941000 "*" 1.13140401492018e-08 1.87801380787728e-08 -100
"chr14" 76975001 76976000 "*" 7.19069981336418e-09 1.2316637081482e-08
72.3404255319149
"chr14" 77026001 77027000 "*" 0 0 100
"chr14" 77041001 77042000 "*" 1.11022302462516e-16 5.03662826618488e-16
93.801652892562
"chr14" 77057001 77058000 "*" 0 0 100
"chr14" 77161001 77162000 "*" 2.22044604925031e-16 9.81641919380259e-16
-62.8571428571429
"chr14" 77177001 77178000 "*" 7.91722243320692e-12 2.05253922596697e-11
-68.4210526315789
"chr14" 77228001 77229000 "*" 0 0 64.5461662554565
"chr14" 77234001 77235000 "*" 1.26565424807268e-14 4.63923203146481e-14
-96.1538461538462

"chr14" 77235001 77236000 "*" 4.99020824662466e-11 1.15790792586157e-10 -100
"chr14" 77237001 77238000 "*" 1.48452907744456e-08 2.4306645411127e-08
53.5714285714286
"chr14" 77240001 77241000 "*" 1.5225265492802e-11 3.79701499207093e-11
-88.3720930232558
"chr14" 77330001 77331000 "*" 5.93070037524512e-12 1.56289353138867e-11
55.2631578947368
"chr14" 77333001 77334000 "*" 3.07531777821168e-14 1.07626052665081e-13 100
"chr14" 77356001 77357000 "*" 1.28785870856518e-14 4.71440608755782e-14 100
"chr14" 77385001 77386000 "*" 0 0 -96
"chr14" 77390001 77391000 "*" 3.92023507610473e-05 3.91707405990543e-05
-58.3333333333333
"chr14" 77396001 77397000 "*" 1.41019906862994e-09 2.67639689581636e-09
-55.672514619883
"chr14" 77399001 77400000 "*" 0 0 100
"chr14" 77415001 77416000 "*" 1.87405646556726e-13 5.97285292681429e-13 100
"chr14" 77423001 77424000 "*" 0 0 -64.0096618357488
"chr14" 77437001 77438000 "*" 9.65338919911574e-13 2.80678910656113e-12 100
"chr14" 77500001 77501000 "*" 0 0 -67.2131147540984
"chr14" 77590001 77591000 "*" 2.3990809339125e-12 6.65441738642456e-12 100
"chr14" 77647001 77648000 "*" 6.82787160144471e-14 2.29357680997297e-13 -75
"chr14" 77709001 77710000 "*" 1.75559566883976e-12 4.95274168312475e-12
-70.2755905511811
"chr14" 77710001 77711000 "*" 7.80486786311485e-14 2.60664512069585e-13
-56.140350877193
"chr14" 77786001 77787000 "*" 0 0 100
"chr14" 77793001 77794000 "*" 0 0 -100
"chr14" 77941001 77942000 "*" 5.63420975430162e-08 8.488944423918e-08
-51.4285714285714
"chr14" 77943001 77944000 "*" 8.7349629751543e-09 1.47505871898511e-08 100
"chr14" 77964001 77965000 "*" 0 0 55.117239614745
"chr14" 78254001 78255000 "*" 7.93809462606987e-14 2.64679416473686e-13 -100
"chr14" 78283001 78284000 "*" 1.18882348409954e-10 2.627708765712e-10
-54.5454545454545
"chr14" 78313001 78314000 "*" 2.68673971959288e-14 9.47266815431045e-14 100
"chr14" 78406001 78407000 "*" 2.94198342021268e-07 4.01110767715142e-07
-61.9047619047619
"chr14" 78480001 78481000 "*" 0 0 -54.1288339172088
"chr14" 78593001 78594000 "*" 3.46500605985511e-13 1.06777978272295e-12 -100
"chr14" 78668001 78669000 "*" 1.69864122767649e-14 6.13572966736024e-14 60
"chr14" 78704001 78705000 "*" 3.90831811358794e-12 1.05610567215048e-11 100
"chr14" 78915001 78916000 "*" 6.41059381134568e-08 9.58562859580824e-08
-72.7272727272727
"chr14" 79463001 79464000 "*" 9.04570973681018e-10 1.76414389822173e-09 -100
"chr14" 79608001 79609000 "*" 5.84976281159388e-06 6.64027514691778e-06
-71.4285714285714
"chr14" 79745001 79746000 "*" 0 0 72.4252141259863
"chr14" 80760001 80761000 "*" 3.56775498033812e-10 7.35898629809829e-10 100
"chr14" 81452001 81453000 "*" 3.84137166520304e-14 1.32929369817675e-13 -100
"chr14" 81687001 81688000 "*" 0 0 80
"chr14" 81714001 81715000 "*" 6.90747459231034e-12 1.80140148623876e-11 -100
"chr14" 81731001 81732000 "*" 3.63445940010365e-11 8.58565209111204e-11 -100

"chr14" 81799001 81800000 "*" 1.19904086659517e-14 4.40901388691796e-14 100
"chr14" 81902001 81903000 "*" 0 0 69.0260265291405
"chr14" 81927001 81928000 "*" 6.24654217240561e-10 1.2462108191303e-09 100
"chr14" 82134001 82135000 "*" 0.000124234819681268 0.000114751114635044
52.6315789473684
"chr14" 82143001 82144000 "*" 2.79440914852103e-11 6.73046092948135e-11 -100
"chr14" 84144001 84145000 "*" 1.98365768255826e-11 4.85612402473442e-11 100
"chr14" 84556001 84557000 "*" 2.79776202205539e-14 9.84620232065018e-14
66.6666666666667
"chr14" 84769001 84770000 "*" 8.7349629751543e-09 1.47505871898511e-08 100
"chr14" 86051001 86052000 "*" 5.48638023900594e-10 1.10374357336421e-09 -100
"chr14" 86089001 86090000 "*" 0 0 -93.5483870967742
"chr14" 86204001 86205000 "*" 1.22862220064412e-07 1.76152573195251e-07
54.1666666666667
"chr14" 86647001 86648000 "*" 5.295763827462e-14 1.80214784640623e-13 -100
"chr14" 87556001 87557000 "*" 5.55111512312578e-16 2.36485094870365e-15 -100
"chr14" 88383001 88384000 "*" 1.504943045183e-06 1.86503757716367e-06
59.1549295774648
"chr14" 88477001 88478000 "*" 3.04324343503026e-11 7.26599935673121e-11 -100
"chr14" 88613001 88614000 "*" 1.07306019625497e-09 2.06750377161448e-09
-73.6842105263158
"chr14" 88651001 88652000 "*" 0 0 -100
"chr14" 88708001 88709000 "*" 3.6700841921089e-08 5.65530226072258e-08 -100
"chr14" 88741001 88742000 "*" 1.27649570478283e-05 1.37608261433093e-05
-59.0909090909091
"chr14" 88746001 88747000 "*" 2.88779000712225e-11 6.92935809466068e-11 -100
"chr14" 88755001 88756000 "*" 7.0006535457523e-08 1.03695832512054e-07 -100
"chr14" 89467001 89468000 "*" 1.91224813761437e-12 5.36527594469447e-12 -100
"chr14" 89494001 89495000 "*" 0 0 87.7777777777778
"chr14" 89653001 89654000 "*" 6.39173269867399e-10 1.27371442682169e-09
-68.5714285714286
"chr14" 89721001 89722000 "*" 9.65338919911574e-13 2.80678910656113e-12 -100
"chr14" 89894001 89895000 "*" 4.99020824662466e-11 1.15790792586157e-10 -100
"chr14" 89970001 89971000 "*" 5.01025332333427e-10 1.01358031135889e-09 -100
"chr14" 90011001 90012000 "*" 1.13140401492018e-08 1.87801380787728e-08 100
"chr14" 90027001 90028000 "*" 6.75256256532641e-06 7.58854169420589e-06
-61.9047619047619
"chr14" 90043001 90044000 "*" 1.56863411149288e-12 4.4469621057462e-12 -100
"chr14" 90076001 90077000 "*" 0 0 -100
"chr14" 90149001 90150000 "*" 1.48087875295744e-10 3.22075573380618e-10 100
"chr14" 90161001 90162000 "*" 9.08108033215171e-11 2.03407753381119e-10 100
"chr14" 90167001 90168000 "*" 0 0 89.9628252788104
"chr14" 90528001 90529000 "*" 0 0 88.8041498131966
"chr14" 90605001 90606000 "*" 4.02167188440217e-12 1.08266700497926e-11 100
"chr14" 90888001 90889000 "*" 7.67056418382595e-11 1.7359448580318e-10 100
"chr14" 90892001 90893000 "*" 7.0995120893258e-10 1.40352301987475e-09 -64.0625
"chr14" 90893001 90894000 "*" 9.51515422009663e-09 1.59952128218453e-08
-80.1204819277108
"chr14" 90922001 90923000 "*" 1.85549925313389e-07 2.59651891706434e-07
-78.9473684210526
"chr14" 90949001 90950000 "*" 9.88098491916389e-15 3.67401261259622e-14
-73.6842105263158

"chr14" 90978001 90979000 "*" 0 0 -100
"chr14" 90984001 90985000 "*" 0 0 -54.2372881355932
"chr14" 91014001 91015000 "*" 7.67056418382595e-11 1.7359448580318e-10 -100
"chr14" 91015001 91016000 "*" 2.78677081411161e-12 7.656935764371e-12
71.7948717948718
"chr14" 91018001 91019000 "*" 1.67334377465522e-08 2.71418641479927e-08
-53.8461538461538
"chr14" 91023001 91024000 "*" 1.36604061395929e-11 3.42333952202875e-11 -100
"chr14" 91035001 91036000 "*" 4.43136073150496e-05 4.39299853239263e-05
65.5172413793103
"chr14" 91061001 91062000 "*" 1.67299207820548e-08 2.71377429441055e-08 100
"chr14" 91085001 91086000 "*" 7.0006535457523e-08 1.03695832512054e-07 -100
"chr14" 91097001 91098000 "*" 1.28141512499846e-05 1.3811181398921e-05
66.1290322580645
"chr14" 91252001 91253000 "*" 7.56965417014754e-08 1.115633055789e-07
60.377358490566
"chr14" 91272001 91273000 "*" 0 0 62.7450980392157
"chr14" 91279001 91280000 "*" 2.68450373042128e-10 5.64465213127486e-10 -100
"chr14" 91670001 91671000 "*" 1.74853465040314e-11 4.32752710900609e-11 100
"chr14" 91688001 91689000 "*" 6.15840711759574e-13 1.83676803572802e-12 100
"chr14" 91708001 91709000 "*" 1.57871920936081e-06 1.9504960058684e-06
56.6666666666667
"chr14" 91751001 91752000 "*" 4.95355900742567e-09 8.67483356500546e-09
67.741935483871
"chr14" 91797001 91798000 "*" 0 0 67.741935483871
"chr14" 91820001 91821000 "*" 8.67505306478833e-08 1.26895177832038e-07
52.1739130434783
"chr14" 92041001 92042000 "*" 6.59550958292954e-09 1.13456194382773e-08 100
"chr14" 92045001 92046000 "*" 2.08814465718632e-09 3.86232567960463e-09 -100
"chr14" 92048001 92049000 "*" 1.29037891483108e-11 3.24582280286866e-11 100
"chr14" 92329001 92330000 "*" 2.06501482580279e-14 7.3890370570066e-14 100
"chr14" 92355001 92356000 "*" 9.67004254448511e-14 3.18178990782609e-13 100
"chr14" 92377001 92378000 "*" 2.68450373042128e-10 5.64465213127486e-10 100
"chr14" 92392001 92393000 "*" 4.2708164887939e-06 4.94144268742661e-06
54.5454545454545
"chr14" 92418001 92419000 "*" 1.67299207820548e-08 2.71377429441055e-08 100
"chr14" 92587001 92588000 "*" 7.24664772633332e-11 1.64639590037884e-10
-58.9285714285714
"chr14" 92616001 92617000 "*" 4.86832796298131e-13 1.47290845675726e-12 -100
"chr14" 92648001 92649000 "*" 1.14124265593318e-11 2.89138062669327e-11 -100
"chr14" 92673001 92674000 "*" 2.88710388929303e-10 6.03399264731139e-10 -100
"chr14" 92721001 92722000 "*" 0 0 74.2857142857143
"chr14" 92743001 92744000 "*" 1.24984033345044e-07 1.79000832707579e-07
66.6666666666667
"chr14" 92755001 92756000 "*" 1.76990644362718e-11 4.37795269358674e-11
81.7204301075269
"chr14" 92788001 92789000 "*" 1.08156346101396e-08 1.80590495236184e-08
-80.5970149253731
"chr14" 92844001 92845000 "*" 7.0006535457523e-08 1.03695832512054e-07 -100
"chr14" 92851001 92852000 "*" 8.88178419700125e-16 3.70670032207938e-15 100
"chr14" 92854001 92855000 "*" 0 0 100
"chr14" 92908001 92909000 "*" 6.92287338566189e-11 1.57605630124247e-10 -100

"chr14" 92913001 92914000 "*" 0 0 98.2456140350877
"chr14" 92914001 92915000 "*" 0 0 -100
"chr14" 92924001 92925000 "*" 0 0 -73.0769230769231
"chr14" 92925001 92926000 "*" 5.80312464748545e-11 1.33619900113832e-10 76.5957446808511
"chr14" 92931001 92932000 "*" 0 0 80
"chr14" 92979001 92980000 "*" 0 0 -71.0185524838541
"chr14" 93020001 93021000 "*" 0.00026000927797809 0.000227826229278078 -51.219512195122
"chr14" 93048001 93049000 "*" 7.88258347483861e-15 2.96145301916012e-14 100
"chr14" 93080001 93081000 "*" 4.89385171487111e-06 5.61726783723613e-06 63.7931034482759
"chr14" 93126001 93127000 "*" 0 0 100
"chr14" 93162001 93163000 "*" 1.20591092667155e-10 2.66075324129686e-10 100
"chr14" 93226001 93227000 "*" 0 0 -66.6666666666667
"chr14" 93327001 93328000 "*" 3.80977693836826e-10 7.83571028258916e-10 72.2222222222222
"chr14" 93364001 93365000 "*" 3.33066907387547e-16 1.4495649018245e-15 100
"chr14" 93383001 93384000 "*" 0 0 -100
"chr14" 93391001 93392000 "*" 3.04324343503026e-11 7.26599935673121e-11 100
"chr14" 93453001 93454000 "*" 8.12683254025615e-14 2.70475078021219e-13 -69.2307692307692
"chr14" 93502001 93503000 "*" 0 0 -82.521645021645
"chr14" 93510001 93511000 "*" 5.7397198105491e-11 1.32229333354669e-10 67.8571428571429
"chr14" 93511001 93512000 "*" 0 0 -100
"chr14" 93542001 93543000 "*" 4.79389640362093e-06 5.5104996383779e-06 -70
"chr14" 93580001 93581000 "*" 0 0 91.7197452229299
"chr14" 93898001 93899000 "*" 0 0 -100
"chr14" 94169001 94170000 "*" 6.4152538836737e-10 1.27477697728417e-09 100
"chr14" 94201001 94202000 "*" 0 0 -55.3956834532374
"chr14" 94204001 94205000 "*" 0 0 -91.6666666666667
"chr14" 94229001 94230000 "*" 2.90434343241941e-13 9.02138795169982e-13 -96.1538461538462
"chr14" 94231001 94232000 "*" 2.08814465718632e-09 3.86232567960463e-09 100
"chr14" 94236001 94237000 "*" 3.00535152319981e-10 6.26981543572063e-10 77.3809523809524
"chr14" 94245001 94246000 "*" 7.16924630772553e-10 1.41628603646556e-09 66.6666666666667
"chr14" 94299001 94300000 "*" 7.0006535457523e-08 1.03695832512054e-07 -100
"chr14" 94300001 94301000 "*" 2.58488785931377e-11 6.25744366234981e-11 65.8536585365854
"chr14" 94320001 94321000 "*" 0 0 100
"chr14" 94358001 94359000 "*" 1.42176913353609e-07 2.01691647814992e-07 80.3030303030303
"chr14" 94362001 94363000 "*" 3.84614562420893e-12 1.04062035914347e-11 66.6666666666667
"chr14" 94381001 94382000 "*" 6.07653830722032e-05 5.89767869178676e-05 55.8823529411765
"chr14" 94390001 94391000 "*" 0 0 100
"chr14" 94394001 94395000 "*" 4.53321524762629e-09 7.99529069650621e-09 -75.5102040816327

"chr14" 94398001 94399000 "*" 6.67017106771306e-05 6.43065783242906e-05 60.7142857142857
"chr14" 94414001 94415000 "*" 2.61776633703192e-09 4.77711122078405e-09 65.1162790697674
"chr14" 94422001 94423000 "*" 5.12901089710915e-07 6.76904585220232e-07 -60
"chr14" 94425001 94426000 "*" 1.11022302462516e-16 5.03662826618488e-16 100
"chr14" 94506001 94507000 "*" 2.3990809339125e-12 6.65441738642456e-12 100
"chr14" 94604001 94605000 "*" 1.68753899743024e-14 6.09826805967439e-14 100
"chr14" 94640001 94641000 "*" 0 0 -74.4082840236686
"chr14" 94665001 94666000 "*" 4.44089209850063e-16 1.91071758245033e-15 -100
"chr14" 94756001 94757000 "*" 2.79554157600614e-13 8.70028046297026e-13 -74.4949494949495
"chr14" 94914001 94915000 "*" 1.51536347914316e-08 2.47779418740862e-08 -51.3888888888889
"chr14" 94941001 94942000 "*" 4.11205047767815e-06 4.77032042248001e-06 67.9245283018868
"chr14" 94952001 94953000 "*" 4.44089209850063e-16 1.91071758245033e-15 60.377358490566
"chr14" 94957001 94958000 "*" 1.12634901405784e-10 2.49563754426169e-10 100
"chr14" 94971001 94972000 "*" 5.72875658022554e-09 9.96023038963802e-09 72.2222222222222
"chr14" 94988001 94989000 "*" 2.69018141096922e-12 7.41487347763769e-12 100
"chr14" 95022001 95023000 "*" 1.67299207820548e-08 2.71377429441055e-08 -100
"chr14" 95051001 95052000 "*" 2.33028818463765e-10 4.93719283454501e-10 -100
"chr14" 95065001 95066000 "*" 2.00548466722239e-10 4.30081817969627e-10 56
"chr14" 95086001 95087000 "*" 3.6700841921089e-08 5.65530226072258e-08 -100
"chr14" 95151001 95152000 "*" 5.75735936703126e-09 1.00076653588041e-08 78.8461538461538
"chr14" 95152001 95153000 "*" 3.6700841921089e-08 5.65530226072258e-08 -100
"chr14" 95156001 95157000 "*" 0 0 -67.2646352118829
"chr14" 95160001 95161000 "*" 0 0 100
"chr14" 95178001 95179000 "*" 5.03441732746523e-12 1.3378171545226e-11 100
"chr14" 95200001 95201000 "*" 1.02332222140511e-07 1.48311384985254e-07 -68.0412371134021
"chr14" 95211001 95212000 "*" 2.05391259555654e-14 7.35435952190685e-14 100
"chr14" 95233001 95234000 "*" 1.35152055102949e-08 2.22296682689876e-08 73.5135135135135
"chr14" 95236001 95237000 "*" 0 0 68.3582089552239
"chr14" 95289001 95290000 "*" 1.14756237101776e-06 1.44529600663061e-06 63.6363636363636
"chr14" 95347001 95348000 "*" 3.40125705378114e-10 7.03784073980162e-10 100
"chr14" 95352001 95353000 "*" 8.57092175010621e-13 2.51149198099779e-12 100
"chr14" 95386001 95387000 "*" 9.06273616041453e-07 1.15739767942151e-06 75
"chr14" 95390001 95391000 "*" 0 0 98.3050847457627
"chr14" 95463001 95464000 "*" 2.43005615629954e-09 4.45014222280278e-09 100
"chr14" 95478001 95479000 "*" 0 0 -100
"chr14" 95518001 95519000 "*" 1.27738147837064e-08 2.10788281973632e-08 -59.8360655737705
"chr14" 95544001 95545000 "*" 8.3882012447134e-11 1.88922989503423e-10 -100
"chr14" 95657001 95658000 "*" 9.04570973681018e-10 1.76414389822173e-09 -100
"chr14" 95688001 95689000 "*" 1.11022302462516e-16 5.03662826618488e-16 98.3193277310924

"chr14" 95726001 95727000 "*" 6.21414031343193e-12 1.63459166469347e-11 -61.1111111111111
"chr14" 95759001 95760000 "*" 4.44089209850063e-16 1.91071758245033e-15 100
"chr14" 95781001 95782000 "*" 2.5131008385415e-12 6.95264252270586e-12 53.8461538461538
"chr14" 95827001 95828000 "*" 2.47148078891257e-07 3.40451936347986e-07 52.9761904761905
"chr14" 95888001 95889000 "*" 0 0 100
"chr14" 95891001 95892000 "*" 5.48638023900594e-10 1.10374357336421e-09 -100
"chr14" 95910001 95911000 "*" 4.01313771103418e-09 7.11882087237587e-09 -100
"chr14" 95933001 95934000 "*" 2.15125472990962e-10 4.58126659943874e-10 100
"chr14" 95940001 95941000 "*" 6.54429075375162e-08 9.77502704411541e-08 -56.5217391304348
"chr14" 96016001 96017000 "*" 7.12117476098229e-10 1.40750530455241e-09 -65.3846153846154
"chr14" 96021001 96022000 "*" 4.72282942753921e-08 7.18654355900973e-08 -71.7948717948718
"chr14" 96086001 96087000 "*" 9.08108033215171e-11 2.03407753381119e-10 -100
"chr14" 96090001 96091000 "*" 1.67299207820548e-08 2.71377429441055e-08 -100
"chr14" 96143001 96144000 "*" 2.88779000712225e-11 6.92935809466068e-11 100
"chr14" 96151001 96152000 "*" 1.29037891483108e-11 3.24582280286866e-11 100
"chr14" 96181001 96182000 "*" 9.04570973681018e-10 1.76414389822173e-09 100
"chr14" 96355001 96356000 "*" 2.15950257675956e-10 4.59742233591841e-10 91.2280701754386
"chr14" 96358001 96359000 "*" 1.83297821365613e-13 5.8528910199162e-13 61.8955512572534
"chr14" 96505001 96506000 "*" 0 0 71.160870956706
"chr14" 96548001 96549000 "*" 1.13140401492018e-08 1.87801380787728e-08 100
"chr14" 96553001 96554000 "*" 0 0 -100
"chr14" 96558001 96559000 "*" 1.93720595120794e-11 4.76309924836636e-11 -100
"chr14" 96592001 96593000 "*" 6.92287338566189e-11 1.57605630124247e-10 100
"chr14" 96627001 96628000 "*" 6.15840711759574e-13 1.83676803572802e-12 -100
"chr14" 96655001 96656000 "*" 1.59872115546023e-14 5.79614235026873e-14 -54.8136645962733
"chr14" 96658001 96659000 "*" 1.67088565206086e-13 5.35643557574347e-13 82.7586206896552
"chr14" 96687001 96688000 "*" 7.00293898059368e-06 7.85615355394095e-06 57.6923076923077
"chr14" 96735001 96736000 "*" 0 0 -66.6666666666667
"chr14" 97048001 97049000 "*" 5.5291465187457e-06 6.29827733433899e-06 -53.8461538461538
"chr14" 97049001 97050000 "*" 2.00227675550835e-08 3.20459176617961e-08 100
"chr14" 97069001 97070000 "*" 2.00227675550835e-08 3.20459176617961e-08 -100
"chr14" 97093001 97094000 "*" 2.96843660763102e-11 7.11039353170169e-11 66.6666666666667
"chr14" 97220001 97221000 "*" 3.00837132982679e-12 8.23244629232561e-12 77.4193548387097
"chr14" 97360001 97361000 "*" 1.50154333411479e-11 3.74672949165617e-11 73.5849056603774
"chr14" 97390001 97391000 "*" 6.4324989779152e-11 1.47230020825092e-10 100
"chr14" 97431001 97432000 "*" 2.55351295663786e-15 1.01678090381833e-14 100
"chr14" 97467001 97468000 "*" 6.04438721296674e-11 1.38849255306602e-10 100

"chr14" 97477001 97478000 "*" 1.37828859436695e-10 3.01445544267661e-10 100
"chr14" 97512001 97513000 "*" 0.000412523446254087 0.000349386048469569 -52
"chr14" 97527001 97528000 "*" 7.97140131680862e-14 2.65641277678906e-13 -100
"chr14" 97674001 97675000 "*" 0 0 -100
"chr14" 97726001 97727000 "*" 6.88338275267597e-15 2.60518659957509e-14 -100
"chr14" 97843001 97844000 "*" 1.14124265593318e-11 2.89138062669327e-11 100
"chr14" 97900001 97901000 "*" 1.52794177310511e-09 2.87642564232045e-09 100
"chr14" 97906001 97907000 "*" 8.58968451922237e-12 2.213554983903e-11 100
"chr14" 98012001 98013000 "*" 3.43946745240409e-06 4.03871797278586e-06
-54.0540540540541
"chr14" 98119001 98120000 "*" 7.23857003226414e-08 1.0699758706795e-07
-66.6666666666667
"chr14" 98134001 98135000 "*" 4.65637306490407e-10 9.46720524046618e-10 -100
"chr14" 98354001 98355000 "*" 0 0 -100
"chr14" 98524001 98525000 "*" 1.12458486967171e-09 2.15740815043925e-09 -100
"chr14" 98843001 98844000 "*" 2.02227318224502e-07 2.81656956190631e-07
-66.6666666666667
"chr14" 98983001 98984000 "*" 2.88710388929303e-10 6.03399264731139e-10 -100
"chr14" 99308001 99309000 "*" 4.44089209850063e-16 1.91071758245033e-15 -100
"chr14" 99368001 99369000 "*" 5.7562294069391e-05 5.60610907415495e-05
-62.8571428571429
"chr14" 99370001 99371000 "*" 0.000321268697584531 0.000277075512580754 60
"chr14" 99404001 99405000 "*" 1.98124849859482e-10 4.25157611354621e-10 -100
"chr14" 99424001 99425000 "*" 5.61115034880544e-07 7.3743121634436e-07 59.375
"chr14" 99464001 99465000 "*" 8.99330487857952e-09 1.5165043858425e-08
-95.4545454545455
"chr14" 99482001 99483000 "*" 2.15125472990962e-10 4.58126659943874e-10 -100
"chr14" 99509001 99510000 "*" 1.37643085817984e-09 2.61498651228495e-09
-62.9032258064516
"chr14" 99533001 99534000 "*" 2.88710388929303e-10 6.03399264731139e-10 100
"chr14" 99585001 99586000 "*" 0 0 -66.6666666666667
"chr14" 99595001 99596000 "*" 5.10702591327572e-15 1.96271840288234e-14 -100
"chr14" 99644001 99645000 "*" 5.6362137179633e-11 1.29963270300065e-10 -100
"chr14" 99649001 99650000 "*" 6.03040628543994e-09 1.04574393316449e-08
-83.3333333333333
"chr14" 99650001 99651000 "*" 4.35340596305345e-06 5.03092867450971e-06 -58.4
"chr14" 99656001 99657000 "*" 2.26663132707472e-12 6.30759534372307e-12
-61.7647058823529
"chr14" 99658001 99659000 "*" 6.00713747633819e-08 9.01889203095212e-08
78.8177339901478
"chr14" 99665001 99666000 "*" 2.22349350043061e-09 4.10086040786164e-09
-73.4584450402145
"chr14" 99669001 99670000 "*" 1.69162905905296e-10 3.65860283541333e-10
90.5172413793103
"chr14" 99678001 99679000 "*" 5.378030005454e-05 5.26241567504211e-05 60
"chr14" 99681001 99682000 "*" 8.88178419700125e-16 3.70670032207938e-15 -98
"chr14" 99685001 99686000 "*" 0 0 -100
"chr14" 99690001 99691000 "*" 0 0 100
"chr14" 99720001 99721000 "*" 8.71677421021566e-07 1.11555243887433e-06
67.2727272727273
"chr14" 99722001 99723000 "*" 0 0 69.6969696969697
"chr14" 99728001 99729000 "*" 4.59974724975609e-10 9.36623190864066e-10

-52.1739130434783
"chr14" 99736001 99737000 "*" 0 0 79.96632996633
"chr14" 99796001 99797000 "*" 7.0006535457523e-08 1.03695832512054e-07 -100
"chr14" 99846001 99847000 "*" 1.12055920098442e-11 2.84500040664577e-11 -100
"chr14" 99931001 99932000 "*" 1.80989033669032e-05 1.90667975357288e-05 68.75
"chr14" 99934001 99935000 "*" 4.87054078179838e-08 7.39916020002763e-08
78.5714285714286
"chr14" 100017001 100018000 "*" 1.0406453476719e-11 2.65698904926094e-11
63.4146341463415
"chr14" 100030001 100031000 "*" 8.3758206703699e-08 1.22770045449312e-07
61.0665737190659
"chr14" 100036001 100037000 "*" 8.57092175010621e-13 2.51149198099779e-12 100
"chr14" 100040001 100041000 "*" 6.25943642473814e-08 9.37203979140189e-08
54.5454545454545
"chr14" 100102001 100103000 "*" 2.05391259555654e-14 7.35435952190685e-14 -100
"chr14" 100110001 100111000 "*" 2.17278466330129e-08 3.46207166872483e-08
-56.056338028169
"chr14" 100129001 100130000 "*" 8.61533067109121e-14 2.86139374215847e-13
66.2650602409639
"chr14" 100137001 100138000 "*" 1.49613654798486e-12 4.26244554429779e-12 100
"chr14" 100138001 100139000 "*" 1.26397892152852e-09 2.41117847690952e-09
92.8571428571429
"chr14" 100139001 100140000 "*" 0 0 98.6111111111111
"chr14" 100143001 100144000 "*" 1.29998126596753e-07 1.85795907100183e-07
-54.5454545454545
"chr14" 100149001 100150000 "*" 7.07212066686225e-14 2.37065833066764e-13
78.2101167315175
"chr14" 100155001 100156000 "*" 0 0 57.3734409391049
"chr14" 100156001 100157000 "*" 1.48487888651516e-11 3.70689457767456e-11 100
"chr14" 100165001 100166000 "*" 7.50889320721981e-08 1.10720624980668e-07
51.2368972746331
"chr14" 100170001 100171000 "*" 3.63445940010365e-11 8.58565209111204e-11 100
"chr14" 100174001 100175000 "*" 0 0 100
"chr14" 100195001 100196000 "*" 1.22124532708767e-15 5.03921984217778e-15
77.4193548387097
"chr14" 100213001 100214000 "*" 2.80053757961696e-12 7.68792291333011e-12
53.1060606060606
"chr14" 100217001 100218000 "*" 8.09791678157978e-10 1.59023287870581e-09
54.1666666666667
"chr14" 100220001 100221000 "*" 2.22044604925031e-16 9.81641919380259e-16
-85.8974358974359
"chr14" 100234001 100235000 "*" 7.7715611723761e-16 3.26213507634405e-15
-58.3708145927037
"chr14" 100240001 100241000 "*" 0 0 59.2592592592593
"chr14" 100260001 100261000 "*" 3.44169137633799e-15 1.3517917469209e-14
96.0988296488947
"chr14" 100268001 100269000 "*" 0 0 -100
"chr14" 100295001 100296000 "*" 1.67299207820548e-08 2.71377429441055e-08 100
"chr14" 100383001 100384000 "*" 1.29037891483108e-11 3.24582280286866e-11 100
"chr14" 100396001 100397000 "*" 1.31190845942264e-06 1.63923591886211e-06
-53.8461538461538
"chr14" 100400001 100401000 "*" 1.11022302462516e-16 5.03662826618488e-16 100

"chr14" 100439001 100440000 "*" 1.67299207820548e-08 2.71377429441055e-08 -100
"chr14" 100525001 100526000 "*" 1.92946192356658e-09 3.59193011611483e-09 -100
"chr14" 100583001 100584000 "*" 0 0 -100
"chr14" 100625001 100626000 "*" 0 0 55.9134850217119
"chr14" 100675001 100676000 "*" 6.37490060739765e-13 1.89496793587877e-12 -100
"chr14" 100681001 100682000 "*" 0 0 65.2294154619736
"chr14" 100720001 100721000 "*" 4.01313771103418e-09 7.11882087237587e-09 100
"chr14" 100788001 100789000 "*" 2.91192730594858e-07 3.97285685748504e-07 -74
"chr14" 100806001 100807000 "*" 1.12634901405784e-10 2.49563754426169e-10 -100
"chr14" 100813001 100814000 "*" 2.15125472990962e-10 4.58126659943874e-10 100
"chr14" 100919001 100920000 "*" 1.12634901405784e-10 2.49563754426169e-10 -100
"chr14" 100958001 100959000 "*" 4.08209022140227e-11 9.57770690255255e-11 100
"chr14" 101044001 101045000 "*" 0 0 71.7171717171717
"chr14" 101061001 101062000 "*" 2.00227675550835e-08 3.20459176617961e-08 100
"chr14" 101070001 101071000 "*" 5.9433680199561e-11 1.36739595920481e-10
-63.4408602150538
"chr14" 101076001 101077000 "*" 0 0 72.7272727272727
"chr14" 101104001 101105000 "*" 0 0 100
"chr14" 101106001 101107000 "*" 1.11022302462516e-16 5.03662826618488e-16
57.8947368421053
"chr14" 101107001 101108000 "*" 6.08402217494586e-14 2.05454787937634e-13 -100
"chr14" 101120001 101121000 "*" 0 0 73.9130434782609
"chr14" 101139001 101140000 "*" 4.08209022140227e-11 9.57770690255255e-11 -100
"chr14" 101155001 101156000 "*" 0 0 100
"chr14" 101159001 101160000 "*" 0 0 -75.1159913393133
"chr14" 101165001 101166000 "*" 1.64880934461209e-07 2.32052259590606e-07
81.1320754716981
"chr14" 101167001 101168000 "*" 9.47730782741019e-12 2.43309141846448e-11
68.5714285714286
"chr14" 101181001 101182000 "*" 2.69284570431072e-06 3.21451361994916e-06
-69.7247706422018
"chr14" 101185001 101186000 "*" 0 0 95.0819672131148
"chr14" 101243001 101244000 "*" 7.2748190915739e-08 1.07497206386442e-07
70.8333333333333
"chr14" 101251001 101252000 "*" 5.44268425960448e-05 5.31987507485423e-05
-54.3478260869565
"chr14" 101317001 101318000 "*" 2.80937484387067e-09 5.09865996492029e-09
-91.304347826087
"chr14" 101326001 101327000 "*" 0 0 -62.1621621621622
"chr14" 101328001 101329000 "*" 5.18375477590594e-05 5.08604172540523e-05
51.7241379310345
"chr14" 101359001 101360000 "*" 0 0 55.3594351732991
"chr14" 101370001 101371000 "*" 1.94443504930586e-06 2.36999982936668e-06
54.0616246498599
"chr14" 101377001 101378000 "*" 2.85044210457386e-11 6.8594759556e-11
60.4054859868813
"chr14" 101489001 101490000 "*" 7.31718281987703e-06 8.1817202364377e-06
68.5714285714286
"chr14" 101494001 101495000 "*" 6.66133814775094e-16 2.81595744474255e-15 100
"chr14" 101500001 101501000 "*" 1.5438052531902e-06 1.91018981220019e-06
55.045871559633
"chr14" 101502001 101503000 "*" 3.29846731039751e-08 5.14512638859322e-08

70.8333333333333
"chr14" 101510001 101511000 "*" 1.11382907246327e-05 1.21160977238229e-05
66.6666666666667
"chr14" 101517001 101518000 "*" 4.10120271077119e-10 8.40504151596823e-10
86.3636363636364
"chr14" 101518001 101519000 "*" 7.95622282807606e-05 7.57887419325014e-05
61.1111111111111
"chr14" 101521001 101522000 "*" 4.32986979603811e-15 1.68006959513174e-14
-66.6666666666667
"chr14" 101580001 101581000 "*" 1.54343982039506e-09 2.90468356240933e-09
58.0116959064327
"chr14" 101585001 101586000 "*" 1.98124849859482e-10 4.25157611354621e-10 100
"chr14" 101644001 101645000 "*" 3.13730930123768e-10 6.53056911927157e-10
51.7118863049096
"chr14" 101650001 101651000 "*" 0 0 81.8181818181818
"chr14" 101667001 101668000 "*" 3.35769860273505e-05 3.39131450192894e-05
-54.5454545454545
"chr14" 101686001 101687000 "*" 1.77385000688091e-07 2.48772998744819e-07
77.7777777777778
"chr14" 101687001 101688000 "*" 9.04207819729663e-11 2.03063150957007e-10
96.875
"chr14" 101691001 101692000 "*" 1.76508386795504e-08 2.85482852963876e-08
-61.9718309859155
"chr14" 101709001 101710000 "*" 1.20591092667155e-10 2.66075324129686e-10 -100
"chr14" 101717001 101718000 "*" 3.26184634857896e-11 7.76392759152788e-11
69.7674418604651
"chr14" 101799001 101800000 "*" 2.85418778611657e-08 4.48575831312927e-08 75
"chr14" 101828001 101829000 "*" 1.4210854715202e-14 5.18140765840233e-14
70.4918032786885
"chr14" 101839001 101840000 "*" 8.71525074330748e-14 2.88848349331391e-13
-75.5555555555556
"chr14" 101909001 101910000 "*" 0 0 -65.0485436893204
"chr14" 101938001 101939000 "*" 0 0 100
"chr14" 101967001 101968000 "*" 1.2061079065262e-05 1.30541743073331e-05
-53.8461538461538
"chr14" 101983001 101984000 "*" 1.13277408231305e-07 1.6315651418575e-07
-51.9675925925926
"chr14" 101993001 101994000 "*" 2.76201284066246e-12 7.59166186436745e-12 76
"chr14" 102001001 102002000 "*" 0 0 -75.2380952380952
"chr14" 102026001 102027000 "*" 0 0 68.4643819696857
"chr14" 102038001 102039000 "*" 4.03101203194645e-07 5.3936046097731e-07
-71.1111111111111
"chr14" 102045001 102046000 "*" 1.29037891483108e-11 3.24582280286866e-11 100
"chr14" 102048001 102049000 "*" 7.67056418382595e-11 1.7359448580318e-10 -100
"chr14" 102095001 102096000 "*" 1.55431223447522e-15 6.33705141101682e-15 -100
"chr14" 102171001 102172000 "*" 1.17148188816252e-07 1.68359536049301e-07
-54.6875
"chr14" 102173001 102174000 "*" 0 0 -100
"chr14" 102198001 102199000 "*" 5.55111512312578e-16 2.36485094870365e-15 100
"chr14" 102242001 102243000 "*" 0 0 100
"chr14" 102274001 102275000 "*" 9.08108033215171e-11 2.03407753381119e-10 -100
"chr14" 102401001 102402000 "*" 0 0 54.2553191489362

"chr14" 102406001 102407000 "*" 9.0072393987839e-13 2.63157725646847e-12 100
"chr14" 102423001 102424000 "*" 0.000455208012464681 0.000382689032955743
-51.219512195122
"chr14" 102543001 102544000 "*" 1.14484027091688e-06 1.44210721372388e-06
-54.5454545454545
"chr14" 102653001 102654000 "*" 1.55431223447522e-15 6.33705141101682e-15 100
"chr14" 102861001 102862000 "*" 3.41034267159479e-07 4.61167338008112e-07
75.9259259259259
"chr14" 102921001 102922000 "*" 4.0705084769499e-07 5.44261714716217e-07
-68.5714285714286
"chr14" 102922001 102923000 "*" 1.12067016078132e-06 1.41298429985191e-06
53.8461538461538
"chr14" 102958001 102959000 "*" 5.10702591327572e-15 1.96271840288234e-14
64.0669240669241
"chr14" 102976001 102977000 "*" 0 0 -57.2365136220558
"chr14" 103026001 103027000 "*" 1.08113518137998e-12 3.13124835551985e-12
-53.1914893617021
"chr14" 103057001 103058000 "*" 1.06515109210559e-06 1.34717919745894e-06
-66.6666666666667
"chr14" 103236001 103237000 "*" 2.08157258096975e-09 3.86053957272538e-09
-66.6666666666667
"chr14" 103334001 103335000 "*" 7.0006535457523e-08 1.03695832512054e-07 100
"chr14" 103541001 103542000 "*" 0 0 -65.9637678981173
"chr14" 103566001 103567000 "*" 0 0 -54.1528662420382
"chr14" 103595001 103596000 "*" 2.80581375911026e-07 3.83712098183096e-07
59.3220338983051
"chr14" 103603001 103604000 "*" 0 0 -85.6697819314642
"chr14" 103618001 103619000 "*" 4.78841410966879e-11 1.11468187156588e-10 100
"chr14" 103628001 103629000 "*" 4.71134242729931e-12 1.257615036806e-11 -100
"chr14" 103631001 103632000 "*" 1.05065132616211e-06 1.33001006138239e-06 87.5
"chr14" 103633001 103634000 "*" 1.75415237890775e-14 6.3304859797472e-14 -67.5
"chr14" 103643001 103644000 "*" 2.22044604925031e-16 9.81641919380259e-16 -100
"chr14" 103654001 103655000 "*" 5.295763827462e-14 1.80214784640623e-13 100
"chr14" 103668001 103669000 "*" 1.98124849859482e-10 4.25157611354621e-10 100
"chr14" 103734001 103735000 "*" 4.71134242729931e-12 1.257615036806e-11 -100
"chr14" 103742001 103743000 "*" 6.24654217240561e-10 1.2462108191303e-09 -100
"chr14" 103764001 103765000 "*" 2.64951482975562e-09 4.82186011375752e-09 100
"chr14" 103851001 103852000 "*" 0 0 92.5925925925926
"chr14" 103867001 103868000 "*" 6.4152538836737e-10 1.27477697728417e-09 100
"chr14" 103972001 103973000 "*" 1.81891146588953e-08 2.93670613897249e-08
-62.962962962963
"chr14" 104094001 104095000 "*" 0 0 -96.0526315789474
"chr14" 104139001 104140000 "*" 6.92287338566189e-11 1.57605630124247e-10 100
"chr14" 104170001 104171000 "*" 2.18249862626863e-11 5.32196674364684e-11
85.8823529411765
"chr14" 104188001 104189000 "*" 9.08108033215171e-11 2.03407753381119e-10 -100
"chr14" 104470001 104471000 "*" 6.4152538836737e-10 1.27477697728417e-09 100
"chr14" 104486001 104487000 "*" 0 0 87.8787878787879
"chr14" 104500001 104501000 "*" 6.15840711759574e-13 1.83676803572802e-12 100
"chr14" 104504001 104505000 "*" 6.08402217494586e-14 2.05454787937634e-13 -100
"chr14" 104510001 104511000 "*" 0 0 100
"chr14" 104521001 104522000 "*" 0 0 100

"chr14" 104602001 104603000 "*" 0 0 52.4166489288733
"chr14" 104617001 104618000 "*" 0 0 -58.8361006082525
"chr14" 104631001 104632000 "*" 0 0 100
"chr14" 104669001 104670000 "*" 1.55431223447522e-15 6.33705141101682e-15 100
"chr14" 104694001 104695000 "*" 3.33066907387547e-16 1.4495649018245e-15 100
"chr14" 104698001 104699000 "*" 2.44681541672342e-05 2.52637134962309e-05
-50.5494505494505
"chr14" 104718001 104719000 "*" 0 0 -50.2196882519092
"chr14" 104748001 104749000 "*" 0 0 -100
"chr14" 104752001 104753000 "*" 0.000339409657744816 0.000291590294462647
-52.0833333333333
"chr14" 104760001 104761000 "*" 5.18477993050048e-06 5.9332362765949e-06
-54.4592030360531
"chr14" 104764001 104765000 "*" 2.03553217438257e-05 2.12753843906822e-05
-61.5384615384615
"chr14" 104786001 104787000 "*" 3.08642000845794e-14 1.08000998509109e-13
-57.565011820331
"chr14" 104792001 104793000 "*" 3.42236393280348e-06 4.01993630240642e-06 -67.5
"chr14" 104829001 104830000 "*" 5.6362137179633e-11 1.29963270300065e-10 100
"chr14" 104834001 104835000 "*" 0 0 55.2070636786794
"chr14" 104838001 104839000 "*" 1.3051448810586e-11 3.281480592286e-11
97.4358974358974
"chr14" 104859001 104860000 "*" 0 0 57.8512396694215
"chr14" 104864001 104865000 "*" 5.6621374255883e-15 2.16468580166064e-14 -100
"chr14" 104871001 104872000 "*" 1.37828859436695e-10 3.01445544267661e-10 100
"chr14" 104888001 104889000 "*" 3.29736238313671e-14 1.15087809548132e-13
57.2258064516129
"chr14" 104892001 104893000 "*" 0 0 91.1504424778761
"chr14" 104895001 104896000 "*" 0 0 64.6825396825397
"chr14" 104912001 104913000 "*" 2.22044604925031e-16 9.81641919380259e-16
78.1609195402299
"chr14" 104933001 104934000 "*" 1.13140401492018e-08 1.87801380787728e-08 -100
"chr14" 104937001 104938000 "*" 0 0 -60.7142857142857
"chr14" 104947001 104948000 "*" 4.01313771103418e-09 7.11882087237587e-09 -100
"chr14" 104980001 104981000 "*" 6.70766542310020e-09 1.15299979662832e-08
-55.5555555555556
"chr14" 104981001 104982000 "*" 1.06955355683169e-09 2.06112833509361e-09
52.3809523809524
"chr14" 104983001 104984000 "*" 1.96644922567657e-11 4.82930612417392e-11 -100
"chr14" 105010001 105011000 "*" 1.5246004458902e-10 3.31092437734489e-10
-84.375
"chr14" 105011001 105012000 "*" 1.21123264773537e-06 1.52031976843376e-06
69.7674418604651
"chr14" 105021001 105022000 "*" 0 0 97.8494623655914
"chr14" 105028001 105029000 "*" 1.92060986003995e-08 3.09216248280382e-08
-72.7272727272727
"chr14" 105043001 105044000 "*" 0 0 61.9684789507087
"chr14" 105059001 105060000 "*" 0 0 50.9090909090909
"chr14" 105062001 105063000 "*" 1.09615555864417e-05 1.193832842474e-05
-81.8181818181818
"chr14" 105068001 105069000 "*" 1.21065293012812e-07 1.7369567892842e-07
80.9523809523809

"chr14" 105116001 105117000 "*" 0 0 -66.5871121718377
"chr14" 105124001 105125000 "*" 0 0 -54.1363144608727
"chr14" 105147001 105148000 "*" 0 0 -51.2366998081284
"chr14" 105191001 105192000 "*" 5.68545210910543e-13 1.70301572563023e-12 -50.886132033673
"chr14" 105276001 105277000 "*" 8.10795874883752e-13 2.38732360337017e-12 89.5833333333333
"chr14" 105389001 105390000 "*" 7.30615568045323e-12 1.90008567750447e-11 -65.5172413793103
"chr14" 105392001 105393000 "*" 6.10622663543836e-15 2.32593540648772e-14 67.5605739825923
"chr14" 105406001 105407000 "*" 2.00227675550835e-08 3.20459176617961e-08 100
"chr14" 105417001 105418000 "*" 7.26463333933225e-12 1.89005329339158e-11 68.9655172413793
"chr14" 105418001 105419000 "*" 0 0 85.5769230769231
"chr14" 105446001 105447000 "*" 0 0 -59.4387755102041
"chr14" 105485001 105486000 "*" 3.31690230837012e-12 9.03347040781321e-12 -100
"chr14" 105526001 105527000 "*" 1.1550078671263e-09 2.21275873837915e-09 -55.3324555628703
"chr14" 105534001 105535000 "*" 3.15303338993544e-14 1.1025793806681e-13 58
"chr14" 105555001 105556000 "*" 0 0 -68.75
"chr14" 105560001 105561000 "*" 6.05482330939822e-12 1.59429337637235e-11 53.4320104098894
"chr14" 105576001 105577000 "*" 5.54966150811964e-09 9.66456406679912e-09 90
"chr14" 105586001 105587000 "*" 4.79408238662771e-08 7.28903061847097e-08 -70.9090909090909
"chr14" 105589001 105590000 "*" 8.6590385351637e-05 8.19846342120331e-05 53.3333333333333
"chr14" 105593001 105594000 "*" 3.5527136788005e-15 1.39277426410519e-14 -64.2156862745098
"chr14" 105654001 105655000 "*" 5.54914349533142e-05 5.41707559020162e-05 54.5454545454545
"chr14" 105887001 105888000 "*" 9.43689570931383e-15 3.51625644188498e-14 -75
"chr14" 105944001 105945000 "*" 0 0 66.6160178488998
"chr14" 105947001 105948000 "*" 3.17423864970579e-12 8.66331944936225e-12 -54.1062801932367
"chr14" 105948001 105949000 "*" 0 0 -100
"chr14" 105969001 105970000 "*" 0 0 87.6106194690265
"chr14" 105977001 105978000 "*" 1.88737914186277e-15 7.62011598380321e-15 100
"chr14" 106002001 106003000 "*" 1.26453902904444e-09 2.41208143008691e-09 72.5490196078431
"chr14" 106024001 106025000 "*" 0 0 66.6666666666667
"chr14" 106053001 106054000 "*" 7.54922790946466e-11 1.71248128497101e-10 -61.7117117117117
"chr14" 106072001 106073000 "*" 3.2602306354601e-07 4.41864123893867e-07 58.75
"chr14" 106084001 106085000 "*" 2.75276401673352e-08 4.33321576363063e-08 -85.7142857142857
"chr14" 106087001 106088000 "*" 8.71359051579645e-09 1.47505871898511e-08 -77.2727272727273
"chr14" 106110001 106111000 "*" 0 0 51.0461225083661
"chr14" 106113001 106114000 "*" 3.21409565628983e-13 9.94169112716859e-13 88.8888888888889

"chr14" 106114001 106115000 "*" 1.20792265079217e-11 3.05185068044685e-11
71.4975845410628
"chr14" 106117001 106118000 "*" 0 0 73.469387755102
"chr14" 106126001 106127000 "*" 1.93720595120794e-11 4.76309924836636e-11 100
"chr14" 106158001 106159000 "*" 5.84350455801186e-08 8.78650619990244e-08
51.219512195122
"chr14" 106185001 106186000 "*" 5.57921436827868e-08 8.41094622135478e-08
-54.8387096774194
"chr14" 106205001 106206000 "*" 2.76182587910512e-09 5.01673135048814e-09
-54.7619047619048
"chr14" 106234001 106235000 "*" 0 0 55.3846153846154
"chr14" 106239001 106240000 "*" 8.35233711493633e-06 9.25812704126149e-06 66
"chr14" 106286001 106287000 "*" 3.6700841921089e-08 5.65530226072258e-08 100
"chr14" 106302001 106303000 "*" 0 0 -77.2727272727273
"chr14" 106303001 106304000 "*" 4.71028438475685e-10 9.57191590395459e-10
51.219512195122
"chr14" 106307001 106308000 "*" 0 0 72.0245398773006
"chr14" 106316001 106317000 "*" 2.23200036231219e-09 4.11527580699148e-09
59.8039215686275
"chr14" 106330001 106331000 "*" 2.04980477036543e-12 5.72522258875706e-12
-65.1162790697674
"chr14" 106334001 106335000 "*" 2.96785992781601e-07 4.04526355638833e-07
72.8813559322034
"chr14" 106342001 106343000 "*" 1.11022302462516e-16 5.03662826618488e-16 100
"chr14" 106344001 106345000 "*" 6.43929354282591e-15 2.44732760419317e-14
-86.6666666666667
"chr14" 106347001 106348000 "*" 1.13140401492018e-08 1.87801380787728e-08 100
"chr14" 106349001 106350000 "*" 0 0 100
"chr14" 106351001 106352000 "*" 0 0 87.0967741935484
"chr14" 106371001 106372000 "*" 9.95929220315439e-07 1.26390320926982e-06
-66.1290322580645
"chr14" 106374001 106375000 "*" 0 0 -75.9036144578313
"chr14" 106379001 106380000 "*" 0 0 -100
"chr14" 106380001 106381000 "*" 5.04263297784746e-13 1.52078941532818e-12 100
"chr14" 106385001 106386000 "*" 0 0 57.1428571428571
"chr14" 106393001 106394000 "*" 0 0 81.5384615384615
"chr14" 106396001 106397000 "*" 3.61932706027801e-14 1.25777907157994e-13 100
"chr14" 106573001 106574000 "*" 0 0 100
"chr14" 106623001 106624000 "*" 1.49547041417009e-13 4.81631428944031e-13
-85.2459016393443
"chr14" 106659001 106660000 "*" 1.74853465040314e-11 4.32752710900609e-11 -100
"chr14" 106667001 106668000 "*" 2.18158824338843e-13 6.87314823207509e-13 -100
"chr14" 106725001 106726000 "*" 0 0 -66.8114143920595
"chr14" 106775001 106776000 "*" 9.19190198400699e-08 1.33983087534006e-07
52.8301886792453
"chr14" 106846001 106847000 "*" 0 0 87.7551020408163
"chr14" 106873001 106874000 "*" 1.68753899743024e-14 6.09826805967439e-14 -100
"chr14" 106930001 106931000 "*" 3.34128893442198e-08 5.19772864618523e-08 100
"chr14" 106939001 106940000 "*" 6.90747459231034e-12 1.80140148623876e-11 -100
"chr14" 107218001 107219000 "*" 0 0 -100
"chr15" 20422001 20423000 "*" 2.88657986402541e-15 1.14155613124573e-14 -100
"chr15" 20461001 20462000 "*" 2.87847523594564e-12 7.89226250535469e-12

70.8333333333333
"chr15" 20474001 20475000 "*" 2.66453525910038e-14 9.40074020513994e-14 100
"chr15" 20478001 20479000 "*" 5.14294773434898e-09 8.98856693399752e-09
73.9130434782609
"chr15" 21360001 21361000 "*" 0 0 61.8961038961039
"chr15" 21941001 21942000 "*" 1.12458486967171e-09 2.15740815043925e-09 100
"chr15" 21968001 21969000 "*" 3.33066907387547e-16 1.4495649018245e-15
-58.7837837837838
"chr15" 22386001 22387000 "*" 1.91814265713131e-05 2.01190274029802e-05
-55.2147239263804
"chr15" 22435001 22436000 "*" 3.5527136788005e-15 1.39277426410519e-14
-66.6666666666667
"chr15" 22511001 22512000 "*" 3.62393763997382e-06 4.24189028853679e-06
67.1641791044776
"chr15" 22512001 22513000 "*" 2.04942107728812e-09 3.80429446972369e-09 87.5
"chr15" 22522001 22523000 "*" 1.37828859436695e-10 3.01445544267661e-10 -100
"chr15" 22550001 22551000 "*" 5.34709920785303e-09 9.32914523871505e-09
78.9855072463768
"chr15" 22771001 22772000 "*" 2.77555756156289e-15 1.10015415195978e-14 100
"chr15" 22778001 22779000 "*" 1.19622536484121e-05 1.29551129297337e-05
-69.2307692307692
"chr15" 22826001 22827000 "*" 6.4152538836737e-10 1.27477697728417e-09 100
"chr15" 22882001 22883000 "*" 4.26951363152739e-10 8.72510873516957e-10 -100
"chr15" 22894001 22895000 "*" 5.36518000042729e-05 5.25070408481107e-05
56.4102564102564
"chr15" 22928001 22929000 "*" 3.88811075879847e-06 4.52797803782963e-06
-69.8630136986301
"chr15" 22990001 22991000 "*" 2.41022546365599e-09 4.42480537812895e-09
-58.3333333333333
"chr15" 23085001 23086000 "*" 2.29171737142764e-09 4.21885100729795e-09 78.4
"chr15" 23104001 23105000 "*" 1.68931535426964e-12 4.77577295507603e-12
63.1578947368421
"chr15" 23184001 23185000 "*" 1.37828859436695e-10 3.01445544267661e-10 -100
"chr15" 23208001 23209000 "*" 0 0 92.5
"chr15" 23655001 23656000 "*" 8.7349629751543e-09 1.47505871898511e-08 -100
"chr15" 23662001 23663000 "*" 0 0 50.4347826086956
"chr15" 23665001 23666000 "*" 0.00109523382873278 0.000862983165008511
-56.6666666666667
"chr15" 23689001 23690000 "*" 2.08995487582797e-10 4.46944006972663e-10 -100
"chr15" 23810001 23811000 "*" 0 0 82.3529411764706
"chr15" 23878001 23879000 "*" 9.04570973681018e-10 1.76414389822173e-09 -100
"chr15" 23969001 23970000 "*" 6.90205219200379e-07 8.95750219153074e-07
-53.2051282051282
"chr15" 23991001 23992000 "*" 1.67299207820548e-08 2.71377429441055e-08 -100
"chr15" 24043001 24044000 "*" 1.66533453693773e-15 6.7629186866784e-15
85.8064516129032
"chr15" 24105001 24106000 "*" 4.21656366933476e-07 5.62707974781376e-07
-64.8148148148148
"chr15" 24300001 24301000 "*" 3.95353005888666e-09 7.03928529283733e-09 100
"chr15" 24992001 24993000 "*" 4.01313771103418e-09 7.11882087237587e-09 100
"chr15" 25081001 25082000 "*" 1.19904086659517e-14 4.40901388691796e-14 100
"chr15" 25295001 25296000 "*" 4.65637306490407e-10 9.46720524046618e-10 -100

"chr15" 25298001 25299000 "*" 0 0 -100
"chr15" 25317001 25318000 "*" 0 0 -98.7341772151899
"chr15" 25329001 25330000 "*" 7.68947794327346e-10 1.51366082846044e-09 -67.741935483871
"chr15" 25417001 25418000 "*" 1.25284005392245e-10 2.75649590272477e-10 100
"chr15" 25429001 25430000 "*" 3.08536707294138e-08 4.82861579423663e-08 55.7377049180328
"chr15" 25431001 25432000 "*" 9.99200722162641e-16 4.15382497462808e-15 100
"chr15" 25435001 25436000 "*" 4.9960036108132e-15 1.9234186548721e-14 -80.7692307692308
"chr15" 25463001 25464000 "*" 1.49436019114546e-13 4.81292455938601e-13 -84.6153846153846
"chr15" 25496001 25497000 "*" 7.67164110015983e-14 2.56348497397613e-13 -90.9090909090909
"chr15" 25842001 25843000 "*" 0 0 -57.9487179487179
"chr15" 25889001 25890000 "*" 0 0 66.2650602409639
"chr15" 25900001 25901000 "*" 0 0 100
"chr15" 25902001 25903000 "*" 0 0 -81.6326530612245
"chr15" 25921001 25922000 "*" 0 0 100
"chr15" 25927001 25928000 "*" 0 0 -100
"chr15" 25938001 25939000 "*" 0 0 77.4193548387097
"chr15" 25941001 25942000 "*" 0 0 81.0344827586207
"chr15" 25957001 25958000 "*" 1.52794177310511e-09 2.87642564232045e-09 -100
"chr15" 25962001 25963000 "*" 9.08108033215171e-11 2.03407753381119e-10 100
"chr15" 25988001 25989000 "*" 1.39779633423487e-07 1.98463037958589e-07 -100
"chr15" 26004001 26005000 "*" 1.03740349643999e-11 2.64927964486177e-11 -55.4971620769392
"chr15" 26024001 26025000 "*" 0.000125810433746043 0.000116104599953562 -54.7619047619048
"chr15" 26039001 26040000 "*" 3.94923686519721e-06 4.59512133508005e-06 64.9122807017544
"chr15" 26050001 26051000 "*" 5.04263297784746e-13 1.52078941532818e-12 -100
"chr15" 26056001 26057000 "*" 2.70290456683142e-11 6.52665464305038e-11 85.0746268656716
"chr15" 26104001 26105000 "*" 0.000183148306629688 0.000164546600973141 -52
"chr15" 26106001 26107000 "*" 0 0 100
"chr15" 26111001 26112000 "*" 0 0 -66.0606060606061
"chr15" 26147001 26148000 "*" 8.19565414096335e-05 7.7896169099937e-05 -54.5454545454545
"chr15" 26364001 26365000 "*" 5.44009282066327e-15 2.08486137681812e-14 100
"chr15" 26421001 26422000 "*" 3.33066907387547e-16 1.4495649018245e-15 100
"chr15" 26442001 26443000 "*" 2.66453525910038e-14 9.40074020513994e-14 100
"chr15" 26607001 26608000 "*" 0 0 100
"chr15" 26761001 26762000 "*" 0 0 80
"chr15" 26893001 26894000 "*" 1.67299207820548e-08 2.71377429441055e-08 -100
"chr15" 27182001 27183000 "*" 0 0 82.4175824175824
"chr15" 27190001 27191000 "*" 4.93050045236032e-13 1.49112366876838e-12 -65.4761904761905
"chr15" 27312001 27313000 "*" 3.34128893442198e-08 5.19772864618523e-08 100
"chr15" 27332001 27333000 "*" 6.75926776649849e-06 7.59587203032783e-06 -52
"chr15" 27586001 27587000 "*" 7.25646895127774e-07 9.39130690477135e-07 75
"chr15" 27587001 27588000 "*" 0 0 -100

"chr15" 27595001 27596000 "*" 0.00159187604110156 0.00122164572966646
54.5454545454545
"chr15" 27613001 27614000 "*" 2.91028312560115e-11 6.97590507139978e-11 100
"chr15" 27713001 27714000 "*" 8.06688049692639e-13 2.37556391305009e-12 100
"chr15" 27772001 27773000 "*" 4.21493506763682e-09 7.45822546499483e-09
-66.6666666666667
"chr15" 27999001 28000000 "*" 7.68311852472614e-07 9.90964461140931e-07 -60
"chr15" 28037001 28038000 "*" 0 0 -100
"chr15" 28116001 28117000 "*" 1.40723093031703e-05 1.50698562385407e-05 -62.5
"chr15" 28190001 28191000 "*" 4.01313771103418e-09 7.11882087237587e-09 -100
"chr15" 28230001 28231000 "*" 3.15206049672501e-06 3.72388648210208e-06 53.125
"chr15" 28233001 28234000 "*" 9.08108033215171e-11 2.03407753381119e-10 -100
"chr15" 28260001 28261000 "*" 0 0 -100
"chr15" 28261001 28262000 "*" 5.62926395586416e-06 6.40552376199523e-06
-66.6666666666667
"chr15" 28266001 28267000 "*" 2.79987144580218e-12 7.68634726811898e-12 -100
"chr15" 28273001 28274000 "*" 1.85178816725085e-09 3.4572860197658e-09
78.5714285714286
"chr15" 28327001 28328000 "*" 0 0 -52.7272727272727
"chr15" 28344001 28345000 "*" 0 0 59.7578613003975
"chr15" 28491001 28492000 "*" 2.22044604925031e-16 9.81641919380259e-16 -100
"chr15" 28983001 28984000 "*" 0 0 96
"chr15" 29075001 29076000 "*" 2.38353757842091e-07 3.28978743080408e-07
72.2222222222222
"chr15" 29124001 29125000 "*" 7.66886776304432e-10 1.50988884892903e-09
63.6363636363636
"chr15" 29229001 29230000 "*" 2.30662478006849e-09 4.24423320622348e-09
64.1025641025641
"chr15" 29253001 29254000 "*" 0 0 60.1548801870251
"chr15" 29271001 29272000 "*" 1.98808166185227e-06 2.41923918804664e-06
63.9344262295082
"chr15" 29285001 29286000 "*" 5.794809077031e-12 1.52911192757929e-11 100
"chr15" 29294001 29295000 "*" 3.34128893442198e-08 5.19772864618523e-08 100
"chr15" 29317001 29318000 "*" 1.4432899320127e-15 5.90750815956055e-15 100
"chr15" 29321001 29322000 "*" 6.82957261022299e-06 7.67084757316439e-06
63.6363636363636
"chr15" 29382001 29383000 "*" 1.98365768255826e-11 4.85612402473442e-11 100
"chr15" 29383001 29384000 "*" 1.48087875295744e-10 3.22075573380618e-10 -100
"chr15" 29384001 29385000 "*" 4.9171752436461e-07 6.50759850268532e-07
64.2857142857143
"chr15" 29388001 29389000 "*" 9.80763359414993e-10 1.8980032424715e-09 100
"chr15" 29390001 29391000 "*" 4.73909800291494e-12 1.26482151893118e-11
-72.0430107526882
"chr15" 29391001 29392000 "*" 1.11022302462516e-16 5.03662826618488e-16 100
"chr15" 29427001 29428000 "*" 1.82133530479689e-10 3.9274151003329e-10
54.5454545454545
"chr15" 29488001 29489000 "*" 2.44249065417534e-15 9.75180348030994e-15
66.6666666666667
"chr15" 29519001 29520000 "*" 0 0 100
"chr15" 29535001 29536000 "*" 1.07884812194925e-11 2.74553537455803e-11
81.8181818181818
"chr15" 29595001 29596000 "*" 3.6700841921089e-08 5.65530226072258e-08 -100

"chr15" 29633001 29634000 "*" 2.22044604925031e-16 9.81641919380259e-16 100
"chr15" 29674001 29675000 "*" 0 0 100
"chr15" 29675001 29676000 "*" 1.05112252235529e-10 2.33797528638982e-10 53.4351145038168
"chr15" 29677001 29678000 "*" 3.95353005888666e-09 7.03928529283733e-09 -100
"chr15" 29678001 29679000 "*" 6.12778716657658e-11 1.40703078182493e-10 66.6666666666667
"chr15" 29698001 29699000 "*" 6.67167043832961e-09 1.14714352027381e-08 76.9230769230769
"chr15" 29750001 29751000 "*" 0 0 100
"chr15" 29831001 29832000 "*" 4.86753970463383e-11 1.13145908161915e-10 -100
"chr15" 29862001 29863000 "*" 0 0 53.7090030386496
"chr15" 29863001 29864000 "*" 0 0 76.8018018018018
"chr15" 29905001 29906000 "*" 2.02327044007689e-12 5.65300384792621e-12 100
"chr15" 29928001 29929000 "*" 1.22124532708767e-15 5.03921984217778e-15 57.5342465753425
"chr15" 29954001 29955000 "*" 3.45838701010237e-08 5.36757270793292e-08 75
"chr15" 29973001 29974000 "*" 1.35003119794419e-13 4.3679847476192e-13 100
"chr15" 30159001 30160000 "*" 1.37001521238744e-13 4.42851918810123e-13 100
"chr15" 30260001 30261000 "*" 0 0 51.7016331212001
"chr15" 30339001 30340000 "*" 8.7349629751543e-09 1.47505871898511e-08 100
"chr15" 31295001 31296000 "*" 0 0 -100
"chr15" 31328001 31329000 "*" 2.64951482975562e-09 4.82186011375752e-09 100
"chr15" 31448001 31449000 "*" 1.67299207820548e-08 2.71377429441055e-08 100
"chr15" 31454001 31455000 "*" 3.27589178006349e-09 5.90332226301497e-09 -68.2888540031397
"chr15" 31505001 31506000 "*" 0 0 100
"chr15" 31510001 31511000 "*" 3.34128893442198e-08 5.19772864618523e-08 100
"chr15" 31533001 31534000 "*" 1.11022302462516e-16 5.03662826618488e-16 100
"chr15" 31553001 31554000 "*" 1.26565424807268e-14 4.63923203146481e-14 100
"chr15" 31568001 31569000 "*" 1.96509475358653e-13 6.23868565133654e-13 100
"chr15" 31572001 31573000 "*" 0 0 100
"chr15" 31589001 31590000 "*" 1.84705462036305e-08 2.97954860987945e-08 64.1025641025641
"chr15" 31601001 31602000 "*" 0 0 -100
"chr15" 31606001 31607000 "*" 2.15800732839e-08 3.4397743870466e-08 -79.0697674418605
"chr15" 31609001 31610000 "*" 6.4152538836737e-10 1.27477697728417e-09 -100
"chr15" 31621001 31622000 "*" 0 0 96.7741935483871
"chr15" 31670001 31671000 "*" 1.39779633423487e-07 1.98463037958589e-07 100
"chr15" 31686001 31687000 "*" 1.26565424807268e-14 4.63923203146481e-14 -100
"chr15" 31690001 31691000 "*" 1.75910032096382e-08 2.84564731181628e-08 -79.4117647058823
"chr15" 31691001 31692000 "*" 1.52794177310511e-09 2.87642564232045e-09 -100
"chr15" 31692001 31693000 "*" 3.51940698806175e-14 1.22560856705654e-13 -52.2875816993464
"chr15" 31724001 31725000 "*" 1.57585056115295e-12 4.46681287157134e-12 -92.3976608187134
"chr15" 31729001 31730000 "*" 3.31690230837012e-12 9.03347040781321e-12 -100
"chr15" 31731001 31732000 "*" 0 0 -100
"chr15" 31753001 31754000 "*" 9.04570973681018e-10 1.76414389822173e-09 -100
"chr15" 31756001 31757000 "*" 0 0 100

"chr15" 31761001 31762000 "*" 1.76488379466377e-10 3.81090594900825e-10 -63.6752136752137
"chr15" 31762001 31763000 "*" 5.01025332333427e-10 1.01358031135889e-09 -100
"chr15" 31778001 31779000 "*" 1.11022302462516e-16 5.03662826618488e-16 100
"chr15" 31791001 31792000 "*" 1.11022302462516e-15 4.59817122935606e-15 -100
"chr15" 31818001 31819000 "*" 2.17148521386434e-12 6.05008954017764e-12 -59.6250755895989
"chr15" 32014001 32015000 "*" 1.7159362819541e-10 3.7093460996491e-10 -95.4545454545455
"chr15" 32085001 32086000 "*" 2.99540392489916e-11 7.17128186480525e-11 -74.6835443037975
"chr15" 32107001 32108000 "*" 1.67299207820548e-08 2.71377429441055e-08 -100
"chr15" 32149001 32150000 "*" 0 0 72
"chr15" 32407001 32408000 "*" 0 0 100
"chr15" 32413001 32414000 "*" 0 0 85.2941176470588
"chr15" 32933001 32934000 "*" 0 0 -95.852534562212
"chr15" 32934001 32935000 "*" 0 0 -57.8947368421053
"chr15" 33010001 33011000 "*" 0 0 70.6539542519175
"chr15" 33131001 33132000 "*" 0 0 -100
"chr15" 33586001 33587000 "*" 2.09983736660924e-05 2.19044051649657e-05 -66.6666666666667
"chr15" 33759001 33760000 "*" 6.88338275267597e-15 2.60518659957509e-14 -100
"chr15" 33806001 33807000 "*" 8.7349629751543e-09 1.47505871898511e-08 100
"chr15" 33808001 33809000 "*" 2.64951482975562e-09 4.82186011375752e-09 100
"chr15" 33815001 33816000 "*" 0 0 100
"chr15" 33849001 33850000 "*" 0 0 -96.1538461538462
"chr15" 33993001 33994000 "*" 2.73625566649116e-12 7.52483363579137e-12 100
"chr15" 33994001 33995000 "*" 3.99546075805501e-05 3.98784360862687e-05 51.8518518518519
"chr15" 34019001 34020000 "*" 3.23373829846929e-06 3.81325174359463e-06 66.6666666666667
"chr15" 34159001 34160000 "*" 5.55111512312578e-16 2.36485094870365e-15 -100
"chr15" 34453001 34454000 "*" 1.36604061395929e-11 3.42333952202875e-11 100
"chr15" 34555001 34556000 "*" 7.26194548938963e-10 1.43349423709122e-09 88.1889763779528
"chr15" 34626001 34627000 "*" 1.12458486967171e-09 2.15740815043925e-09 -100
"chr15" 34665001 34666000 "*" 4.68339034220833e-10 9.52027929027301e-10 55.1724137931034
"chr15" 34875001 34876000 "*" 4.06701672339693e-10 8.33846377203198e-10 66.2251655629139
"chr15" 35674001 35675000 "*" 6.18676998520584e-10 1.23706767863191e-09 64.2857142857143
"chr15" 35762001 35763000 "*" 3.6700841921089e-08 5.65530226072258e-08 -100
"chr15" 37311001 37312000 "*" 1.17461596005342e-13 3.8279067575877e-13 -100
"chr15" 37776001 37777000 "*" 5.794809077031e-12 1.52911192757929e-11 -100
"chr15" 38083001 38084000 "*" 1.37828859436695e-10 3.01445544267661e-10 100
"chr15" 38192001 38193000 "*" 0.000105894811617713 9.88981208988924e-05 54.8387096774194
"chr15" 38269001 38270000 "*" 1.39779633423487e-07 1.98463037958589e-07 100
"chr15" 38365001 38366000 "*" 0 0 -75.4074074074074
"chr15" 38919001 38920000 "*" 8.43347613965761e-12 2.17859001990012e-11 100
"chr15" 39045001 39046000 "*" 8.80406858527749e-14 2.91456141063452e-13 100

"chr15" 39681001 39682000 "*" 0 0 100
"chr15" 40069001 40070000 "*" 9.63829016598083e-12 2.46993429847588e-11 100
"chr15" 40211001 40212000 "*" 0 0 71.9122023809524
"chr15" 40213001 40214000 "*" 0 0 64.408990567931
"chr15" 40361001 40362000 "*" 0 0 -68.5840707964602
"chr15" 40395001 40396000 "*" 0 0 -65.1878293387727
"chr15" 40396001 40397000 "*" 3.69346791928571e-06 4.31806494110551e-06 -54.5454545454545
"chr15" 40397001 40398000 "*" 0 0 -60
"chr15" 40403001 40404000 "*" 7.0006535457523e-08 1.03695832512054e-07 100
"chr15" 40409001 40410000 "*" 7.0006535457523e-08 1.03695832512054e-07 100
"chr15" 40421001 40422000 "*" 0 0 77.1428571428571
"chr15" 40531001 40532000 "*" 0 0 -52.0426383561792
"chr15" 40545001 40546000 "*" 0 0 -73.6644116130207
"chr15" 40569001 40570000 "*" 0 0 -100
"chr15" 40600001 40601000 "*" 7.10831393746503e-12 1.85065348274956e-11 -97.9591836734694
"chr15" 40631001 40632000 "*" 1.46948680834758e-06 1.82372619460965e-06 -76.1904761904762
"chr15" 40734001 40735000 "*" 0 0 65.4075546719682
"chr15" 40738001 40739000 "*" 0 0 100
"chr15" 40759001 40760000 "*" 1.4432899320127e-15 5.90750815956055e-15 -100
"chr15" 40772001 40773000 "*" 3.35605987444865e-11 7.97679422614068e-11 60.6060606060606
"chr15" 40804001 40805000 "*" 1.06013764433754e-09 2.04373857938985e-09 -72.5714285714286
"chr15" 40979001 40980000 "*" 1.52466927971773e-12 4.33322475981408e-12 -100
"chr15" 41044001 41045000 "*" 3.40746653115787e-10 7.05033947602166e-10 60
"chr15" 41046001 41047000 "*" 0 0 74
"chr15" 41061001 41062000 "*" 0 0 -64.7619047619048
"chr15" 41102001 41103000 "*" 9.03216962433451e-06 9.96387981558718e-06 60
"chr15" 41136001 41137000 "*" 0 0 -81.7456359102244
"chr15" 41137001 41138000 "*" 0 0 -96.1538461538462
"chr15" 41172001 41173000 "*" 2.72646106247443e-07 3.73405126957647e-07 66.6666666666667
"chr15" 41215001 41216000 "*" 4.66461858028566e-09 8.21436741329438e-09 74.1379310344828
"chr15" 41233001 41234000 "*" 2.22044604925031e-16 9.81641919380259e-16 -100
"chr15" 41245001 41246000 "*" 0 0 64.7058823529412
"chr15" 41836001 41837000 "*" 1.06513734110525e-06 1.34717919745894e-06 64.8648648648649
"chr15" 41913001 41914000 "*" 0 0 54.5032268824537
"chr15" 42083001 42084000 "*" 8.29789400635939e-07 1.06506939918901e-06 -90
"chr15" 42108001 42109000 "*" 4.02167188440217e-12 1.08266700497926e-11 100
"chr15" 42210001 42211000 "*" 5.14603117895973e-08 7.78901629649248e-08 -55.7692307692308
"chr15" 42337001 42338000 "*" 0 0 100
"chr15" 42363001 42364000 "*" 1.05392605387333e-05 1.15062005769659e-05 62.5
"chr15" 42380001 42381000 "*" 2.11481063772112e-08 3.37401589947146e-08 66.6666666666667
"chr15" 42475001 42476000 "*" 2.73625566649116e-12 7.52483363579137e-12 100
"chr15" 42486001 42487000 "*" 2.08995487582797e-10 4.46944006972663e-10 100

"chr15" 42672001 42673000 "*" 2.16382467499443e-13 6.82775854166559e-13 -100
"chr15" 42743001 42744000 "*" 7.105427357601e-15 2.68362283258525e-14 -100
"chr15" 43093001 43094000 "*" 6.7390537594747e-14 2.26447131529591e-13 100
"chr15" 43129001 43130000 "*" 8.57092175010621e-13 2.51149198099779e-12 100
"chr15" 43512001 43513000 "*" 1.3063817694281e-08 2.15343710008517e-08
76.9230769230769
"chr15" 43527001 43528000 "*" 0 0 -100
"chr15" 43530001 43531000 "*" 2.37976084072056e-06 2.86393721212228e-06
-67.9487179487179
"chr15" 43553001 43554000 "*" 2.30593322214645e-13 7.25311853262654e-13
53.9858156028369
"chr15" 43560001 43561000 "*" 0 0 -53
"chr15" 43574001 43575000 "*" 1.4751422305892e-11 3.6855663449439e-11
66.6666666666667
"chr15" 43587001 43588000 "*" 6.62958576924666e-12 1.73620572057889e-11 100
"chr15" 43662001 43663000 "*" 0 0 -61.525974025974
"chr15" 43818001 43819000 "*" 2.87558865608162e-12 7.8848664556602e-12
58.8235294117647
"chr15" 43820001 43821000 "*" 0 0 69.421768707483
"chr15" 44022001 44023000 "*" 0 0 100
"chr15" 44069001 44070000 "*" 0 0 -92.5064599483204
"chr15" 44085001 44086000 "*" 0 0 -100
"chr15" 44105001 44106000 "*" 3.58120389308825e-05 3.60126966183746e-05
-61.5384615384615
"chr15" 44117001 44118000 "*" 1.66533453693773e-15 6.7629186866784e-15 -100
"chr15" 44119001 44120000 "*" 0 0 -88.3495145631068
"chr15" 44460001 44461000 "*" 9.80763359414993e-10 1.8980032424715e-09 100
"chr15" 44871001 44872000 "*" 6.92287338566189e-11 1.57605630124247e-10 -100
"chr15" 45402001 45403000 "*" 0 0 -100
"chr15" 45406001 45407000 "*" 0 0 55.88666534575
"chr15" 45458001 45459000 "*" 6.41819930535803e-12 1.6860878046974e-11
-51.9449499545041
"chr15" 45491001 45492000 "*" 4.86753970463383e-11 1.13145908161915e-10 -100
"chr15" 45740001 45741000 "*" 3.95353005888666e-09 7.03928529283733e-09 -100
"chr15" 45748001 45749000 "*" 1.11022302462516e-16 5.03662826618488e-16
-78.2608695652174
"chr15" 45839001 45840000 "*" 0 0 -71.40625
"chr15" 45927001 45928000 "*" 0 0 -98.0551053484603
"chr15" 46006001 46007000 "*" 1.28785870856518e-14 4.71440608755782e-14 -100
"chr15" 46133001 46134000 "*" 3.33066907387547e-16 1.4495649018245e-15 -100
"chr15" 46236001 46237000 "*" 4.01567668006919e-12 1.08266700497926e-11
61.0169491525424
"chr15" 46582001 46583000 "*" 3.99680288865056e-15 1.5566998858822e-14 100
"chr15" 46997001 46998000 "*" 2.15125472990962e-10 4.58126659943874e-10 100
"chr15" 48009001 48010000 "*" 0 0 64.995652173913
"chr15" 48022001 48023000 "*" 0 0 -100
"chr15" 48112001 48113000 "*" 9.47020240005259e-14 3.12491189162604e-13 -100
"chr15" 48402001 48403000 "*" 8.7349629751543e-09 1.47505871898511e-08 -100
"chr15" 48470001 48471000 "*" 0 0 80.119926199262
"chr15" 48610001 48611000 "*" 3.6700841921089e-08 5.65530226072258e-08 -100
"chr15" 48813001 48814000 "*" 0 0 100
"chr15" 48829001 48830000 "*" 1.86517468137026e-14 6.70995420767425e-14 100

"chr15" 49170001 49171000 "*" 0 0 56.441717791411
"chr15" 49216001 49217000 "*" 6.92287338566189e-11 1.57605630124247e-10 -100
"chr15" 49568001 49569000 "*" 9.04570973681018e-10 1.76414389822173e-09 -100
"chr15" 49688001 49689000 "*" 1.5277158427196e-09 2.87642564232045e-09 -100
"chr15" 49996001 49997000 "*" 4.14335232790108e-13 1.26153063645578e-12 100
"chr15" 50541001 50542000 "*" 8.01666433236647e-09 1.36392368970109e-08 100
"chr15" 50688001 50689000 "*" 3.4609876564673e-07 4.67482227931052e-07 60
"chr15" 51058001 51059000 "*" 0 0 -72.8813559322034
"chr15" 51061001 51062000 "*" 2.22044604925031e-16 9.81641919380259e-16 100
"chr15" 51330001 51331000 "*" 1.56863411149288e-12 4.4469621057462e-12 -100
"chr15" 51447001 51448000 "*" 8.51552162117741e-12 2.19817259951489e-11
60.7142857142857
"chr15" 51476001 51477000 "*" 1.56863411149288e-12 4.4469621057462e-12 -100
"chr15" 51543001 51544000 "*" 0 0 87.126047068209
"chr15" 51973001 51974000 "*" 0 0 80
"chr15" 52432001 52433000 "*" 6.15840711759574e-13 1.83676803572802e-12 -100
"chr15" 52471001 52472000 "*" 0 0 -100
"chr15" 52580001 52581000 "*" 0 0 100
"chr15" 52585001 52586000 "*" 1.16573417585641e-14 4.29335582900508e-14 100
"chr15" 52603001 52604000 "*" 1.12055920098442e-11 2.84500040664577e-11 -100
"chr15" 52753001 52754000 "*" 6.77335965093562e-12 1.76897011246462e-11 100
"chr15" 52811001 52812000 "*" 3.6700841921089e-08 5.65530226072258e-08 100
"chr15" 53082001 53083000 "*" 3.66862096257137e-10 7.55862410623019e-10
52.5233644859813
"chr15" 53088001 53089000 "*" 1.99397165445703e-11 4.87965451054975e-11
70.5882352941177
"chr15" 53304001 53305000 "*" 9.65729496371637e-10 1.87255912493232e-09 -100
"chr15" 54202001 54203000 "*" 1.9748950252918e-08 3.17496427224634e-08
53.5353535353535
"chr15" 54203001 54204000 "*" 2.31332867017819e-06 2.78986658105478e-06
60.9090909090909
"chr15" 54459001 54460000 "*" 0 0 56.4102564102564
"chr15" 55073001 55074000 "*" 0 0 100
"chr15" 55707001 55708000 "*" 7.0006535457523e-08 1.03695832512054e-07 -100
"chr15" 56058001 56059000 "*" 2.76302612900459e-07 3.78189313644252e-07
-73.6842105263158
"chr15" 56078001 56079000 "*" 1.07556424766564e-07 1.55425048898003e-07
-59.2592592592593
"chr15" 56081001 56082000 "*" 2.90317550000196e-08 4.55845441590445e-08
82.1428571428571
"chr15" 56536001 56537000 "*" 0 0 -66.6666666666667
"chr15" 56701001 56702000 "*" 9.94253306352277e-08 1.44301508273363e-07
57.6923076923077
"chr15" 56900001 56901000 "*" 2.43005615629954e-09 4.45014222280278e-09 -100
"chr15" 57209001 57210000 "*" 2.31469803679829e-06 2.79034791839104e-06 75
"chr15" 57573001 57574000 "*" 6.60582699651968e-14 2.22176301799e-13 100
"chr15" 57601001 57602000 "*" 2.71855036981794e-06 3.24296987141407e-06 -60
"chr15" 57630001 57631000 "*" 0 0 -72.0779220779221
"chr15" 57883001 57884000 "*" 5.02641261945769e-11 1.16588696106366e-10
64.1509433962264
"chr15" 57896001 57897000 "*" 3.5527136788005e-15 1.39277426410519e-14
-50.8771929824561

"chr15" 58046001 58047000 "*" 1.76253456274367e-11 4.36023524948838e-11
72.972972972973
"chr15" 58058001 58059000 "*" 6.59550958292954e-09 1.13456194382773e-08 -100
"chr15" 58159001 58160000 "*" 1.86517468137026e-14 6.70995420767425e-14 100
"chr15" 58282001 58283000 "*" 2.02060590481778e-13 6.40127405465654e-13 100
"chr15" 58358001 58359000 "*" 0 0 57.5714434787794
"chr15" 58480001 58481000 "*" 0 0 100
"chr15" 58537001 58538000 "*" 3.6700841921089e-08 5.65530226072258e-08 -100
"chr15" 58539001 58540000 "*" 8.24340606886409e-09 1.40010144091621e-08
-71.1111111111111
"chr15" 58721001 58722000 "*" 0 0 -100
"chr15" 58752001 58753000 "*" 9.08108033215171e-11 2.03407753381119e-10 -100
"chr15" 58753001 58754000 "*" 9.65729496371637e-10 1.87255912493232e-09 100
"chr15" 58841001 58842000 "*" 7.0006535457523e-08 1.03695832512054e-07 -100
"chr15" 58856001 58857000 "*" 1.11022302462516e-16 5.03662826618488e-16 100
"chr15" 59095001 59096000 "*" 9.67004254448511e-14 3.18178990782609e-13 100
"chr15" 59615001 59616000 "*" 0 0 -100
"chr15" 59821001 59822000 "*" 0 0 -100
"chr15" 60237001 60238000 "*" 1.38590361409285e-10 3.02706178892116e-10 100
"chr15" 60289001 60290000 "*" 0 0 80.9703894431928
"chr15" 60291001 60292000 "*" 8.58968451922237e-12 2.213554983903e-11 100
"chr15" 60292001 60293000 "*" 0 0 54.5684523809524
"chr15" 60443001 60444000 "*" 1.30340183090993e-13 4.22662705118657e-13 100
"chr15" 60690001 60691000 "*" 0 0 -74.6350364963504
"chr15" 60883001 60884000 "*" 0 0 80.3370786516854
"chr15" 60884001 60885000 "*" 0 0 92.5531914893617
"chr15" 60912001 60913000 "*" 6.51493836656414e-09 1.12484832601884e-08
-61.9047619047619
"chr15" 60987001 60988000 "*" 2.68450373042128e-10 5.64465213127486e-10 100
"chr15" 60993001 60994000 "*" 0 0 96.1038961038961
"chr15" 61238001 61239000 "*" 1.59656732279245e-11 3.97026738266842e-11 -55
"chr15" 61265001 61266000 "*" 2.91028312560115e-11 6.97590507139978e-11 100
"chr15" 61312001 61313000 "*" 6.2962250968468e-08 9.42238581293952e-08
-58.0645161290323
"chr15" 61342001 61343000 "*" 3.34128893442198e-08 5.19772864618523e-08 -100
"chr15" 61354001 61355000 "*" 8.7349629751543e-09 1.47505871898511e-08 -100
"chr15" 61520001 61521000 "*" 0 0 72.1315885385029
"chr15" 61601001 61602000 "*" 4.79616346638068e-14 1.64380445416596e-13 100
"chr15" 61695001 61696000 "*" 1.4873571501095e-06 1.84447733364305e-06
73.1707317073171
"chr15" 62046001 62047000 "*" 7.34456939710526e-12 1.90959942220558e-11
97.2222222222222
"chr15" 62099001 62100000 "*" 0 0 100
"chr15" 62122001 62123000 "*" 4.01313771103418e-09 7.11882087237587e-09 100
"chr15" 62123001 62124000 "*" 1.07504030788519e-07 1.55356097428857e-07
79.2452830188679
"chr15" 62456001 62457000 "*" 0 0 55.2595375516845
"chr15" 62533001 62534000 "*" 1.14124265593318e-11 2.89138062669327e-11 -100
"chr15" 62546001 62547000 "*" 3.10862446895044e-15 1.22466437093774e-14 100
"chr15" 62967001 62968000 "*" 5.55111512312578e-16 2.36485094870365e-15 -100
"chr15" 63088001 63089000 "*" 5.51681911264978e-09 9.60937655961104e-09
-56.5656565656566

"chr15" 63227001 63228000 "*" 3.6700841921089e-08 5.65530226072258e-08 100
"chr15" 63246001 63247000 "*" 0 0 -100
"chr15" 63282001 63283000 "*" 7.27481803686203e-08 1.07497206386442e-07
-51.7241379310345
"chr15" 63307001 63308000 "*" 1.54319890199872e-11 3.84569468400407e-11 -82.5
"chr15" 63335001 63336000 "*" 0 0 54.3766578249337
"chr15" 63641001 63642000 "*" 5.77808698665194e-08 8.6944120419714e-08
52.7777777777778
"chr15" 63650001 63651000 "*" 3.03979064142368e-13 9.42350883309283e-13 100
"chr15" 63657001 63658000 "*" 1.67299207820548e-08 2.71377429441055e-08 100
"chr15" 63662001 63663000 "*" 2.23154827949656e-14 7.95931582053377e-14 -100
"chr15" 63674001 63675000 "*" 0 0 -67.7099236641221
"chr15" 63795001 63796000 "*" 0 0 -88.2352941176471
"chr15" 63894001 63895000 "*" 3.72755218738252e-05 3.73766697227076e-05
51.3513513513514
"chr15" 64180001 64181000 "*" 0 0 -100
"chr15" 64193001 64194000 "*" 1.98365768255826e-11 4.85612402473442e-11 -100
"chr15" 64369001 64370000 "*" 3.7733050017863e-10 7.76376047338581e-10 95
"chr15" 64438001 64439000 "*" 6.59550958292954e-09 1.13456194382773e-08 -100
"chr15" 64448001 64449000 "*" 8.3882012447134e-11 1.88922989503423e-10 -100
"chr15" 65029001 65030000 "*" 4.99900121297969e-12 1.32921056339624e-11 -100
"chr15" 65044001 65045000 "*" 2.05391259555654e-14 7.35435952190685e-14 -100
"chr15" 65054001 65055000 "*" 3.20357620512413e-05 3.24835550088009e-05 -75
"chr15" 65190001 65191000 "*" 3.15231470208177e-05 3.19968409343818e-05
61.9047619047619
"chr15" 65204001 65205000 "*" 0 0 58.28125
"chr15" 65223001 65224000 "*" 1.55431223447522e-15 6.33705141101682e-15 -100
"chr15" 65341001 65342000 "*" 0 0 -84.2105263157895
"chr15" 65378001 65379000 "*" 1.91224813761437e-12 5.36527594469447e-12 100
"chr15" 65628001 65629000 "*" 2.10831352376317e-13 6.66722850310871e-13
-68.5714285714286
"chr15" 65629001 65630000 "*" 1.4432899320127e-14 5.25937808621513e-14
66.6666666666667
"chr15" 65632001 65633000 "*" 8.71525074330748e-14 2.88848349331391e-13 -100
"chr15" 65637001 65638000 "*" 3.6700841921089e-08 5.65530226072258e-08 -100
"chr15" 65639001 65640000 "*" 2.75057754350883e-12 7.56147683426174e-12
77.7777777777778
"chr15" 65670001 65671000 "*" 0 0 75.2801608809135
"chr15" 65678001 65679000 "*" 6.52250242705321e-09 1.12602204181904e-08
-84.1379310344828
"chr15" 65703001 65704000 "*" 4.21884749357559e-15 1.63874445239189e-14 100
"chr15" 65849001 65850000 "*" 1.04861119787358e-10 2.33275286083984e-10 83.75
"chr15" 66235001 66236000 "*" 1.50623957750895e-12 4.28756717183481e-12 -100
"chr15" 66310001 66311000 "*" 0 0 -75
"chr15" 66370001 66371000 "*" 1.47803887560194e-05 1.5780310930685e-05
-65.7142857142857
"chr15" 66446001 66447000 "*" 7.93809462606987e-14 2.64679416473686e-13 -100
"chr15" 66448001 66449000 "*" 0 0 100
"chr15" 66449001 66450000 "*" 3.34128893442198e-08 5.19772864618523e-08 100
"chr15" 66460001 66461000 "*" 9.41469124882133e-14 3.10868561210786e-13 -100
"chr15" 66472001 66473000 "*" 1.08878606130958e-09 2.09576985700032e-09
-72.7272727272727

"chr15" 66477001 66478000 "*" 0.000354266937660941 0.000303242292809723
-56.5217391304348
"chr15" 66526001 66527000 "*" 6.4324989779152e-11 1.47230020825092e-10 -100
"chr15" 66535001 66536000 "*" 1.33226762955019e-15 5.48133041560118e-15 100
"chr15" 66545001 66546000 "*" 0 0 61.8762434787754
"chr15" 66651001 66652000 "*" 4.73234496034536e-09 8.30627300264826e-09 100
"chr15" 66859001 66860000 "*" 4.22826752499716e-08 6.47129910406812e-08
81.4814814814815
"chr15" 66886001 66887000 "*" 8.88178419700125e-16 3.70670032207938e-15 -100
"chr15" 66887001 66888000 "*" 4.01313771103418e-09 7.11882087237587e-09 -100
"chr15" 66896001 66897000 "*" 1.98365768255826e-11 4.85612402473442e-11 -100
"chr15" 66940001 66941000 "*" 0 0 -100
"chr15" 66964001 66965000 "*" 8.7349629751543e-09 1.47505871898511e-08 100
"chr15" 66972001 66973000 "*" 8.10462807976364e-15 3.04213344674586e-14
66.6666666666667
"chr15" 66979001 66980000 "*" 1.67299207820548e-08 2.71377429441055e-08 100
"chr15" 66992001 66993000 "*" 4.71134242729931e-12 1.257615036806e-11 -100
"chr15" 67020001 67021000 "*" 4.01313771103418e-09 7.11882087237587e-09 -100
"chr15" 67033001 67034000 "*" 0 0 100
"chr15" 67053001 67054000 "*" 0.000125720250141348 0.000116027315919275
55.5555555555556
"chr15" 67058001 67059000 "*" 1.73450143137188e-12 4.89521939262129e-12
-55.9322033898305
"chr15" 67063001 67064000 "*" 1.88737914186277e-15 7.62011598380321e-15
63.4615384615385
"chr15" 67065001 67066000 "*" 0 0 100
"chr15" 67134001 67135000 "*" 0 0 -50.2085978596046
"chr15" 67193001 67194000 "*" 6.75324018928336e-10 1.33808072457949e-09
-59.0909090909091
"chr15" 67246001 67247000 "*" 1.08878595028727e-09 2.09576985700032e-09 -62.5
"chr15" 67938001 67939000 "*" 1.98124849859482e-10 4.25157611354621e-10 100
"chr15" 68079001 68080000 "*" 1.0769163338864e-14 3.98631098593833e-14 100
"chr15" 68101001 68102000 "*" 8.51587852457314e-08 1.24715583872536e-07 56
"chr15" 68116001 68117000 "*" 0 0 60.1874325782093
"chr15" 68124001 68125000 "*" 0 0 85.1851851851852
"chr15" 68156001 68157000 "*" 0 0 50.7692307692308
"chr15" 68183001 68184000 "*" 6.52733422867868e-12 1.71146951718423e-11 -100
"chr15" 68220001 68221000 "*" 1.12458486967171e-09 2.15740815043925e-09 100
"chr15" 68225001 68226000 "*" 1.12634901405784e-10 2.49563754426169e-10 -100
"chr15" 68491001 68492000 "*" 0 0 59.3333333333333
"chr15" 68597001 68598000 "*" 2.78853268730339e-05 2.85426570500594e-05
61.6792929292929
"chr15" 68608001 68609000 "*" 4.22939461230953e-12 1.13678964332236e-11
78.7128712871287
"chr15" 68620001 68621000 "*" 2.72004641033163e-14 9.58564042979718e-14
-57.4468085106383
"chr15" 68628001 68629000 "*" 0 0 73.1852315394243
"chr15" 68657001 68658000 "*" 0 0 59.4594594594595
"chr15" 68658001 68659000 "*" 3.19721360497738e-10 6.64796417392446e-10
-64.2857142857143
"chr15" 68680001 68681000 "*" 2.61122070988051e-06 3.12270776186565e-06
68.2926829268293

"chr15" 68698001 68699000 "*" 1.47803887560194e-05 1.5780310930685e-05
-65.7142857142857
"chr15" 68714001 68715000 "*" 6.83206121676605e-08 1.01782477799636e-07
-61.7021276595745
"chr15" 68772001 68773000 "*" 9.63829016598083e-12 2.46993429847588e-11 100
"chr15" 68865001 68866000 "*" 4.32986979603811e-15 1.68006959513174e-14 100
"chr15" 68882001 68883000 "*" 2.44389475323459e-09 4.47479693825001e-09
52.3809523809524
"chr15" 68892001 68893000 "*" 1.07882591748876e-11 2.74553537455803e-11 100
"chr15" 68907001 68908000 "*" 7.97140131680862e-14 2.65641277678906e-13 -100
"chr15" 68986001 68987000 "*" 1.11022302462516e-16 5.03662826618488e-16 100
"chr15" 69019001 69020000 "*" 1.13140401492018e-08 1.87801380787728e-08 100
"chr15" 69103001 69104000 "*" 1.35890187991095e-11 3.40841971363279e-11 100
"chr15" 69167001 69168000 "*" 0 0 100
"chr15" 69379001 69380000 "*" 3.04324343503026e-11 7.26599935673121e-11 -100
"chr15" 69514001 69515000 "*" 6.4152538836737e-10 1.27477697728417e-09 100
"chr15" 69572001 69573000 "*" 1.69482203856708e-05 1.79420595392413e-05
-66.6666666666667
"chr15" 69574001 69575000 "*" 2.88779000712225e-11 6.92935809466068e-11 -100
"chr15" 69586001 69587000 "*" 2.68673971959288e-14 9.47266815431045e-14 100
"chr15" 69587001 69588000 "*" 9.65729496371637e-10 1.87255912493232e-09 100
"chr15" 69645001 69646000 "*" 2.08995487582797e-10 4.46944006972663e-10 100
"chr15" 69680001 69681000 "*" 7.60712492997584e-10 1.49844005927147e-09
-53.2258064516129
"chr15" 69769001 69770000 "*" 3.6700841921089e-08 5.65530226072258e-08 100
"chr15" 69842001 69843000 "*" 1.39779633423487e-07 1.98463037958589e-07 100
"chr15" 69843001 69844000 "*" 6.04438721296674e-11 1.38849255306602e-10 -100
"chr15" 69885001 69886000 "*" 0 0 -100
"chr15" 69886001 69887000 "*" 1.41775480244632e-13 4.57645000023835e-13
70.5479452054795
"chr15" 69921001 69922000 "*" 5.55111512312578e-16 2.36485094870365e-15 100
"chr15" 70015001 70016000 "*" 0 0 75.5555555555556
"chr15" 70040001 70041000 "*" 1.29142252447423e-10 2.83711873728409e-10
-94.1176470588235
"chr15" 70086001 70087000 "*" 8.40400387669415e-06 9.31267396532594e-06
69.7674418604651
"chr15" 70114001 70115000 "*" 6.59401644398372e-10 1.30839733359793e-09
-51.1111111111111
"chr15" 70135001 70136000 "*" 3.29977374713408e-06 3.88628562647627e-06
-52.3809523809524
"chr15" 70178001 70179000 "*" 6.4152538836737e-10 1.27477697728417e-09 -100
"chr15" 70308001 70309000 "*" 1.01030295240889e-14 3.75322807935163e-14
51.1363636363636
"chr15" 70393001 70394000 "*" 5.55111512312578e-16 2.36485094870365e-15 54.6875
"chr15" 70446001 70447000 "*" 0 0 64.1221374045801
"chr15" 70478001 70479000 "*" 1.67299207820548e-08 2.71377429441055e-08 100
"chr15" 70483001 70484000 "*" 1.11022302462516e-16 5.03662826618488e-16 -100
"chr15" 70488001 70489000 "*" 1.32230588700821e-08 2.17848078113936e-08
50.5941213258286
"chr15" 70490001 70491000 "*" 1.19904086659517e-14 4.40901388691796e-14 100
"chr15" 70505001 70506000 "*" 7.90449076193411e-08 1.16206605825357e-07 -60
"chr15" 70542001 70543000 "*" 4.54081217071689e-14 1.56142038363268e-13

52.248579194465
"chr15" 70545001 70546000 "*" 2.87572371648892e-06 3.41877556551342e-06
61.6666666666667
"chr15" 70550001 70551000 "*" 9.94253306352277e-08 1.44301508273363e-07
57.6923076923077
"chr15" 70570001 70571000 "*" 6.66133814775094e-16 2.81595744474255e-15 75
"chr15" 70629001 70630000 "*" 4.56412685423402e-13 1.38373390781645e-12
-81.8181818181818
"chr15" 70644001 70645000 "*" 2.64951482975562e-09 4.82186011375752e-09 -100
"chr15" 70667001 70668000 "*" 3.04324343503026e-11 7.26599935673121e-11 -100
"chr15" 70671001 70672000 "*" 1.17905685215192e-13 3.83653245040640e-13 -100
"chr15" 70752001 70753000 "*" 2.22044604925031e-16 9.81641919380259e-16
-51.8518518518519
"chr15" 70776001 70777000 "*" 1.13140401492018e-08 1.87801380787728e-08 100
"chr15" 70785001 70786000 "*" 1.75909864452706e-09 3.29219426412058e-09
-51.3440860215054
"chr15" 70795001 70796000 "*" 9.7002308407923e-09 1.62892582883122e-08
-65.7142857142857
"chr15" 70805001 70806000 "*" 4.50215679970167e-06 5.19390556817063e-06
62.0689655172414
"chr15" 70819001 70820000 "*" 2.08814465718632e-09 3.86232567960463e-09 100
"chr15" 70877001 70878000 "*" 0 0 73.3898305084746
"chr15" 71150001 71151000 "*" 2.00227675550835e-08 3.20459176617961e-08 -100
"chr15" 72102001 72103000 "*" 1.25717547483362e-10 2.76566987983258e-10
-52.3809523809524
"chr15" 72108001 72109000 "*" 1.60460105126381e-05 1.70453625649539e-05
68.5714285714286
"chr15" 72491001 72492000 "*" 0 0 -100
"chr15" 72564001 72565000 "*" 0 0 52.058432934927
"chr15" 72565001 72566000 "*" 0 0 75.625
"chr15" 72597001 72598000 "*" 1.55431223447522e-15 6.33705141101682e-15 100
"chr15" 72612001 72613000 "*" 0 0 53.3762346096604
"chr15" 73306001 73307000 "*" 3.43591821660993e-12 9.34753307659663e-12 -92
"chr15" 73502001 73503000 "*" 1.11022302462516e-16 5.03662826618488e-16 100
"chr15" 73613001 73614000 "*" 1.29037891483108e-11 3.24582280286866e-11 100
"chr15" 73619001 73620000 "*" 2.08814465718632e-09 3.86232567960463e-09 100
"chr15" 73630001 73631000 "*" 1.01313471478903e-05 1.10909163221897e-05 -52
"chr15" 73633001 73634000 "*" 0 0 91.588785046729
"chr15" 73646001 73647000 "*" 1.39779633423487e-07 1.98463037958589e-07 100
"chr15" 73660001 73661000 "*" 0 0 56.6269281545385
"chr15" 73667001 73668000 "*" 6.62979444576717e-06 7.45931896423396e-06
59.5238095238095
"chr15" 73680001 73681000 "*" 2.62900812231237e-13 8.21294938531841e-13
66.6666666666667
"chr15" 73975001 73976000 "*" 4.81463713342123e-09 8.44502592293080e-09
-61.5384615384615
"chr15" 74033001 74034000 "*" 1.14124265593318e-11 2.89138062669327e-11 100
"chr15" 74037001 74038000 "*" 0 0 100
"chr15" 74044001 74045000 "*" 0 0 52.9006510579692
"chr15" 74109001 74110000 "*" 0 0 -80
"chr15" 74111001 74112000 "*" 1.39779633423487e-07 1.98463037958589e-07 100
"chr15" 74168001 74169000 "*" 2.43005615629954e-09 4.45014222280278e-09 100

"chr15" 74178001 74179000 "*" 1.49623857748082e-09 2.83092877030055e-09 -63.6363636363636
"chr15" 74181001 74182000 "*" 3.07531777821168e-14 1.07626052665081e-13 100
"chr15" 74186001 74187000 "*" 4.02167188440217e-12 1.08266700497926e-11 100
"chr15" 74192001 74193000 "*" 6.44564617913179e-06 7.26663863268796e-06 52.1739130434783
"chr15" 74215001 74216000 "*" 8.98110696923027e-10 1.75478641394683e-09 83.8709677419355
"chr15" 74248001 74249000 "*" 5.55111512312578e-16 2.36485094870365e-15 100
"chr15" 74284001 74285000 "*" 0 0 -59.4082840236686
"chr15" 74321001 74322000 "*" 7.0006535457523e-08 1.03695832512054e-07 100
"chr15" 74392001 74393000 "*" 0 0 75.4098360655738
"chr15" 74410001 74411000 "*" 8.58968451922237e-12 2.213554983903e-11 100
"chr15" 74423001 74424000 "*" 0 0 68.3097390414464
"chr15" 74428001 74429000 "*" 0 0 73.8102103857731
"chr15" 74434001 74435000 "*" 1.54630752646767e-11 3.85079502648228e-11 100
"chr15" 74476001 74477000 "*" 0 0 83.3333333333333
"chr15" 74480001 74481000 "*" 2.8345201741331e-09 5.14161089815519e-09 61.2903225806452
"chr15" 74481001 74482000 "*" 0 0 97.7777777777778
"chr15" 74487001 74488000 "*" 4.01313771103418e-09 7.11882087237587e-09 -100
"chr15" 74488001 74489000 "*" 3.90831811358794e-12 1.05610567215048e-11 100
"chr15" 74501001 74502000 "*" 0 0 86.144578313253
"chr15" 74504001 74505000 "*" 1.36967270858435e-09 2.60268075908651e-09 50.3496503496503
"chr15" 74533001 74534000 "*" 3.8746783559418e-14 1.34043004570659e-13 60.1941747572816
"chr15" 74535001 74536000 "*" 1.35890187991095e-11 3.40841971363279e-11 -100
"chr15" 74548001 74549000 "*" 2.88923949398612e-06 3.43376683307184e-06 68.2539682539683
"chr15" 74549001 74550000 "*" 2.3990809339125e-12 6.65441738642456e-12 100
"chr15" 74555001 74556000 "*" 1.5277158427196e-09 2.87642564232045e-09 -100
"chr15" 74569001 74570000 "*" 6.26566576400478e-11 1.43726522827759e-10 76.3636363636364
"chr15" 74611001 74612000 "*" 1.29037891483108e-11 3.24582280286866e-11 -100
"chr15" 74616001 74617000 "*" 0 0 -100
"chr15" 74640001 74641000 "*" 1.78631553993114e-11 4.4159220155336e-11 59.0070921985816
"chr15" 74681001 74682000 "*" 1.98654724725689e-06 2.41747782100063e-06 70.2290076335878
"chr15" 74695001 74696000 "*" 0 0 100
"chr15" 74705001 74706000 "*" 2.8689339294119e-05 2.93084297927469e-05 -62.8571428571429
"chr15" 74715001 74716000 "*" 0.000120483972667174 0.000111549020346707 56
"chr15" 74725001 74726000 "*" 0 0 72.8695652173913
"chr15" 74831001 74832000 "*" 5.12450964440525e-07 6.76396157331262e-07 -78.7234042553192
"chr15" 74842001 74843000 "*" 3.34128893442198e-08 5.19772864618523e-08 -100
"chr15" 74890001 74891000 "*" 1.22124532708767e-15 5.03921984217778e-15 51.1737932686549
"chr15" 74894001 74895000 "*" 0.000297440250456416 0.000257992216380177 51.4285714285714

"chr15" 74904001 74905000 "*" 3.0099678305362e-10 6.27863110718699e-10
-95.1219512195122
"chr15" 75030001 75031000 "*" 1.38590361409285e-10 3.02706178892116e-10 100
"chr15" 75078001 75079000 "*" 8.43347613965761e-12 2.17859001990012e-11 -100
"chr15" 75082001 75083000 "*" 8.52140580320793e-12 2.19817259951489e-11 -100
"chr15" 75107001 75108000 "*" 9.00459595776226e-10 1.75921098832928e-09
54.9019607843137
"chr15" 75116001 75117000 "*" 4.73234496034536e-09 8.30627300264826e-09 -100
"chr15" 75125001 75126000 "*" 3.07314299563544e-08 4.81129071584046e-08
-94.7368421052632
"chr15" 75228001 75229000 "*" 5.55061641094312e-09 9.66582334128355e-09
-51.7241379310345
"chr15" 75263001 75264000 "*" 1.20543575121701e-11 3.04593655104189e-11 -75
"chr15" 75277001 75278000 "*" 8.80406858527749e-14 2.91456141063452e-13 -100
"chr15" 75312001 75313000 "*" 4.99900121297969e-12 1.32921056339624e-11 -100
"chr15" 75335001 75336000 "*" 4.66293670342566e-15 1.80217883586487e-14
82.7586206896552
"chr15" 75369001 75370000 "*" 9.65729496371637e-10 1.87255912493232e-09 100
"chr15" 75390001 75391000 "*" 1.78390280325402e-09 3.33629495262998e-09
-77.6595744680851
"chr15" 75438001 75439000 "*" 8.80406858527749e-14 2.91456141063452e-13 100
"chr15" 75446001 75447000 "*" 4.08839628818214e-12 1.09999064430838e-11
-90.9090909090909
"chr15" 75473001 75474000 "*" 2.91028312560115e-11 6.97590507139978e-11 -100
"chr15" 75487001 75488000 "*" 1.11022302462516e-16 5.03662826618488e-16
81.4102564102564
"chr15" 75488001 75489000 "*" 1.11022302462516e-16 5.03662826618488e-16
56.6666666666667
"chr15" 75493001 75494000 "*" 3.33066907387547e-16 1.4495649018245e-15 -82.8125
"chr15" 75507001 75508000 "*" 0.000241617109017045 0.000212792315955059
51.1111111111111
"chr15" 75514001 75515000 "*" 0 0 100
"chr15" 75516001 75517000 "*" 2.08814465718632e-09 3.86232567960463e-09 -100
"chr15" 75652001 75653000 "*" 8.12683254025615e-14 2.70475078021219e-13
69.2307692307692
"chr15" 75876001 75877000 "*" 2.00227675550835e-08 3.20459176617961e-08 100
"chr15" 75918001 75919000 "*" 0 0 -100
"chr15" 75940001 75941000 "*" 0 0 81.4345991561181
"chr15" 75955001 75956000 "*" 1.7329047197201e-08 2.80544804345704e-08
83.9080459770115
"chr15" 75973001 75974000 "*" 5.55111512312578e-15 2.12516350804551e-14 100
"chr15" 75979001 75980000 "*" 0 0 58.7905783896875
"chr15" 75985001 75986000 "*" 0 0 -100
"chr15" 75996001 75997000 "*" 8.02691246803988e-14 2.67352274101148e-13 100
"chr15" 76013001 76014000 "*" 5.37980771042612e-12 1.42569258241711e-11
-50.0915750915751
"chr15" 76068001 76069000 "*" 3.52704532247117e-11 8.35581292290093e-11 100
"chr15" 76083001 76084000 "*" 0 0 51.3668036448097
"chr15" 76084001 76085000 "*" 3.43213901743411e-10 7.09874726957059e-10 -68
"chr15" 76282001 76283000 "*" 2.58681964737661e-14 9.14008479605639e-14 100
"chr15" 76351001 76352000 "*" 0 0 88.8888888888889
"chr15" 76352001 76353000 "*" 0 0 68.8912809472551

"chr15" 76373001 76374000 "*" 2.00227675550835e-08 3.20459176617961e-08 100
"chr15" 76447001 76448000 "*" 1.66533453693773e-15 6.7629186866784e-15 100
"chr15" 76471001 76472000 "*" 2.7574353859694e-05 2.82493161653342e-05
-51.5384615384615
"chr15" 76478001 76479000 "*" 1.06229123386647e-07 1.53654814147424e-07
67.8571428571429
"chr15" 76628001 76629000 "*" 0 0 72.8245476003147
"chr15" 76629001 76630000 "*" 0 0 52.9363517060367
"chr15" 77251001 77252000 "*" 1.12376774552558e-12 3.24761818527664e-12 -100
"chr15" 77256001 77257000 "*" 0 0 100
"chr15" 77801001 77802000 "*" 8.7349629751543e-09 1.47505871898511e-08 -100
"chr15" 77838001 77839000 "*" 4.65637306490407e-10 9.46720524046618e-10 -100
"chr15" 77846001 77847000 "*" 5.06492790153246e-07 6.69018874072016e-07
53.6969696969697
"chr15" 77849001 77850000 "*" 4.48752146553488e-13 1.36105323256995e-12
-72.9166666666667
"chr15" 77860001 77861000 "*" 0.000216481080482001 0.000192267358005665
-51.7241379310345
"chr15" 77884001 77885000 "*" 2.95516167092558e-10 6.16896633493509e-10 -56.25
"chr15" 77917001 77918000 "*" 4.26951363152739e-10 8.72510873516957e-10 -100
"chr15" 77924001 77925000 "*" 0 0 51.7963217026957
"chr15" 77942001 77943000 "*" 7.67056418382595e-11 1.7359448580318e-10 -100
"chr15" 77968001 77969000 "*" 7.0006535457523e-08 1.03695832512054e-07 100
"chr15" 77969001 77970000 "*" 2.76259015663527e-11 6.66074830172604e-11
67.741935483871
"chr15" 77978001 77979000 "*" 8.3882012447134e-11 1.88922989503423e-10 -100
"chr15" 77980001 77981000 "*" 1.65423230669148e-14 5.98698695225911e-14 100
"chr15" 77981001 77982000 "*" 8.21565038222616e-15 3.08158744293689e-14 100
"chr15" 77984001 77985000 "*" 9.08108033215171e-11 2.03407753381119e-10 -100
"chr15" 77988001 77989000 "*" 3.52273765713562e-12 9.57342291526305e-12
66.6666666666667
"chr15" 77994001 77995000 "*" 3.6700841921089e-08 5.65530226072258e-08 100
"chr15" 78007001 78008000 "*" 1.68517089171871e-10 3.64529702716602e-10
54.3859649122807
"chr15" 78015001 78016000 "*" 1.01706421062886e-11 2.60036370415026e-11
54.4687380130418
"chr15" 78042001 78043000 "*" 1.62499419475992e-08 2.64755413105723e-08
53.4246575342466
"chr15" 78066001 78067000 "*" 3.4325259023249e-05 3.46019675789391e-05
64.8648648648649
"chr15" 78079001 78080000 "*" 3.33318339595934e-09 6.00008281430369e-09
60.6060606060606
"chr15" 78087001 78088000 "*" 2.64325346821526e-06 3.15856923971574e-06
-53.8461538461538
"chr15" 78103001 78104000 "*" 1.11184395024111e-11 2.82614246355336e-11
86.6666666666667
"chr15" 78109001 78110000 "*" 9.63829016598083e-12 2.46993429847588e-11 -100
"chr15" 78112001 78113000 "*" 0 0 51.1333319588479
"chr15" 78132001 78133000 "*" 8.6159741230496e-08 1.26079635525357e-07
80.7692307692308
"chr15" 78151001 78152000 "*" 1.67299207820548e-08 2.71377429441055e-08 100
"chr15" 78159001 78160000 "*" 0 0 100

"chr15" 78183001 78184000 "*" 3.23169073015439e-05 3.27487222859242e-05 -53.125
"chr15" 78186001 78187000 "*" 6.43929354282591e-15 2.44732760419317e-14
71.1087420042644
"chr15" 78277001 78278000 "*" 0 0 100
"chr15" 78329001 78330000 "*" 1.65423230669148e-14 5.98698695225911e-14 100
"chr15" 78385001 78386000 "*" 8.7349629751543e-09 1.47505871898511e-08 -100
"chr15" 78458001 78459000 "*" 1.29037891483108e-11 3.24582280286866e-11 -100
"chr15" 78484001 78485000 "*" 2.47944188014326e-06 2.97424262282235e-06
73.0769230769231
"chr15" 78487001 78488000 "*" 1.85452941892095e-09 3.46224892191735e-09
-58.6538461538462
"chr15" 78495001 78496000 "*" 1.84519325374666e-08 2.97677628114308e-08
-70.4545454545455
"chr15" 78537001 78538000 "*" 3.61932706027801e-14 1.25777907157994e-13 -100
"chr15" 78599001 78600000 "*" 9.61897228535236e-13 2.801372325071e-12 -100
"chr15" 78601001 78602000 "*" 9.80763359414993e-10 1.8980032424715e-09 100
"chr15" 78608001 78609000 "*" 8.39094571603027e-10 1.64494937720704e-09
-52.1739130434783
"chr15" 78832001 78833000 "*" 0 0 -81.6176470588235
"chr15" 78974001 78975000 "*" 1.11022302462516e-15 4.59817122935606e-15 100
"chr15" 78984001 78985000 "*" 1.66533453693773e-15 6.7629186866784e-15 -100
"chr15" 79012001 79013000 "*" 1.14124265593318e-11 2.89138062669327e-11 100
"chr15" 79043001 79044000 "*" 0 0 -100
"chr15" 79053001 79054000 "*" 2.24739431258225e-05 2.33373905291779e-05
70.3225806451613
"chr15" 79058001 79059000 "*" 1.77635683940025e-15 7.19870740878856e-15
88.3333333333333
"chr15" 79103001 79104000 "*" 0 0 60.7666290868095
"chr15" 79164001 79165000 "*" 1.72417635724287e-13 5.51984606705033e-13
66.6666666666667
"chr15" 79168001 79169000 "*" 0.000320710195636176 0.000276619581787338
52.3809523809524
"chr15" 79236001 79237000 "*" 0 0 100
"chr15" 79272001 79273000 "*" 5.62609180132512e-07 7.39313308405962e-07
-74.3589743589744
"chr15" 79320001 79321000 "*" 0 0 100
"chr15" 79341001 79342000 "*" 1.39779633423487e-07 1.98463037958589e-07 100
"chr15" 79343001 79344000 "*" 6.59550958292954e-09 1.13456194382773e-08 100
"chr15" 79358001 79359000 "*" 9.06977959402866e-09 1.52893628876485e-08
67.6470588235294
"chr15" 79465001 79466000 "*" 2.62900812231237e-13 8.21294938531841e-13 -100
"chr15" 79744001 79745000 "*" 0 0 100
"chr15" 79749001 79750000 "*" 0.00203458288042468 0.0015345749967898
50.377358490566
"chr15" 79750001 79751000 "*" 1.11022302462516e-16 5.03662826618488e-16
60.2222222222222
"chr15" 79784001 79785000 "*" 3.91446555448871e-05 3.91201221655578e-05 80
"chr15" 79851001 79852000 "*" 0 0 50.1230251230251
"chr15" 79884001 79885000 "*" 4.01313771103418e-09 7.11882087237587e-09 -100
"chr15" 80109001 80110000 "*" 7.84927678409986e-14 2.62032243726529e-13
70.3703703703704
"chr15" 80233001 80234000 "*" 0 0 100

"chr15" 80467001 80468000 "*" 9.94196613923748e-10 1.92279504037644e-09
-73.469387755102
"chr15" 80563001 80564000 "*" 4.01313771103418e-09 7.11882087237587e-09 -100
"chr15" 80569001 80570000 "*" 7.0006535457523e-08 1.03695832512054e-07 100
"chr15" 80619001 80620000 "*" 2.64951482975562e-09 4.82186011375752e-09 100
"chr15" 80627001 80628000 "*" 4.03652523450759e-06 4.6892927905593e-06 68.75
"chr15" 80697001 80698000 "*" 0 0 69.0374813972063
"chr15" 80745001 80746000 "*" 2.02327044007689e-12 5.65300384792621e-12 100
"chr15" 80758001 80759000 "*" 2.22044604925031e-16 9.81641919380259e-16 100
"chr15" 80782001 80783000 "*" 0 0 100
"chr15" 80799001 80800000 "*" 1.49880108324396e-14 5.44951848293151e-14 100
"chr15" 80860001 80861000 "*" 0 0 100
"chr15" 81080001 81081000 "*" 5.01025332333427e-10 1.01358031135889e-09 -100
"chr15" 81131001 81132000 "*" 0 0 70.1298701298701
"chr15" 81144001 81145000 "*" 0.000126887439664713 0.000117029410540844
54.5454545454545
"chr15" 81163001 81164000 "*" 5.03264097062583e-13 1.51942428521737e-12 100
"chr15" 81185001 81186000 "*" 6.4152538836737e-10 1.27477697728417e-09 100
"chr15" 81490001 81491000 "*" 9.63829016598083e-12 2.46993429847588e-11 -100
"chr15" 81573001 81574000 "*" 0 0 -96.551724137931
"chr15" 81592001 81593000 "*" 2.33227125390201e-08 3.70226018953189e-08
57.1428571428571
"chr15" 81648001 81649000 "*" 1.39779633423487e-07 1.98463037958589e-07 100
"chr15" 81857001 81858000 "*" 1.58041458031555e-07 2.22975787896719e-07
58.7301587301587
"chr15" 82134001 82135000 "*" 5.55111512312578e-16 2.36485094870365e-15 100
"chr15" 82144001 82145000 "*" 8.43559920105097e-06 9.34507800883669e-06
66.6666666666667
"chr15" 82302001 82303000 "*" 3.12379488263126e-05 3.1728595486819e-05
51.6129032258064
"chr15" 82337001 82338000 "*" 0 0 50.872034638505
"chr15" 82344001 82345000 "*" 0 0 -100
"chr15" 82386001 82387000 "*" 1.46663061251218e-07 2.07681232118119e-07
-66.3793103448276
"chr15" 82402001 82403000 "*" 8.7349629751543e-09 1.47505871898511e-08 -100
"chr15" 82404001 82405000 "*" 3.90898459824429e-05 3.90690942172519e-05
-58.8235294117647
"chr15" 82407001 82408000 "*" 1.26565424807268e-14 4.63923203146481e-14 100
"chr15" 82462001 82463000 "*" 2.21097817831861e-09 4.07913109294465e-09 -81.25
"chr15" 83240001 83241000 "*" 0 0 62.2327044025157
"chr15" 83248001 83249000 "*" 2.26485497023532e-14 8.07362297199981e-14 75
"chr15" 83324001 83325000 "*" 6.59550958292954e-09 1.13456194382773e-08 -100
"chr15" 83331001 83332000 "*" 2.81191525619562e-09 5.10293735854033e-09 -80
"chr15" 83344001 83345000 "*" 2.79440914852103e-11 6.73046092948135e-11 100
"chr15" 83378001 83379000 "*" 0 0 66.1668839634941
"chr15" 83440001 83441000 "*" 2.54696427282042e-08 4.02292707681792e-08
58.3333333333333
"chr15" 83451001 83452000 "*" 9.63829016598083e-12 2.46993429847588e-11 100
"chr15" 83498001 83499000 "*" 1.54630752646767e-11 3.85079502648228e-11 100
"chr15" 83518001 83519000 "*" 0 0 -56.2840702604482
"chr15" 83549001 83550000 "*" 2.69084754478399e-12 7.41487347763769e-12 -100
"chr15" 83592001 83593000 "*" 2.87422404430249e-05 2.93567189445702e-05 56

"chr15" 83602001 83603000 "*" 1.16573417585641e-14 4.29335582900508e-14 100
"chr15" 83621001 83622000 "*" 0 0 55.6409648508361
"chr15" 83630001 83631000 "*" 1.98729921407903e-14 7.12685498544555e-14
-88.2352941176471
"chr15" 83654001 83655000 "*" 1.11022302462516e-16 5.03662826618488e-16 -100
"chr15" 83736001 83737000 "*" 0 0 51.6129032258064
"chr15" 83747001 83748000 "*" 0 0 100
"chr15" 83785001 83786000 "*" 5.41848229618225e-08 8.18120462768728e-08
54.3478260869565
"chr15" 83954001 83955000 "*" 4.32209823486573e-12 1.15935290479641e-11 100
"chr15" 84029001 84030000 "*" 1.66533453693773e-15 6.7629186866784e-15
63.8297872340426
"chr15" 84065001 84066000 "*" 1.19251930552622e-09 2.28133180178698e-09
70.9677419354839
"chr15" 84068001 84069000 "*" 7.67056418382595e-11 1.7359448580318e-10 100
"chr15" 84162001 84163000 "*" 9.62896429257398e-13 2.80291205576417e-12 -100
"chr15" 84198001 84199000 "*" 4.43136073150496e-05 4.39299853239263e-05
65.5172413793103
"chr15" 84225001 84226000 "*" 0 0 100
"chr15" 84324001 84325000 "*" 1.74853465040314e-11 4.32752710900609e-11 100
"chr15" 84326001 84327000 "*" 6.93112234273485e-13 2.05218842007357e-12 100
"chr15" 84341001 84342000 "*" 6.4152538836737e-10 1.27477697728417e-09 100
"chr15" 84435001 84436000 "*" 0 0 100
"chr15" 84445001 84446000 "*" 8.7349629751543e-09 1.47505871898511e-08 100
"chr15" 84541001 84542000 "*" 0 0 100
"chr15" 84611001 84612000 "*" 3.6700841921089e-08 5.65530226072258e-08 100
"chr15" 85424001 85425000 "*" 1.61467534276483e-06 1.99188026338058e-06
-50.8771929824561
"chr15" 85617001 85618000 "*" 1.56863411149288e-12 4.4469621057462e-12 100
"chr15" 85875001 85876000 "*" 1.02437835991509e-10 2.28170470255037e-10
-66.6666666666667
"chr15" 86087001 86088000 "*" 0.000193557836068559 0.000173202918736337
52.8735632183908
"chr15" 86122001 86123000 "*" 3.88936503170623e-06 4.52924899346346e-06
-66.6666666666667
"chr15" 86299001 86300000 "*" 3.90831811358794e-12 1.05610567215048e-11 -100
"chr15" 86397001 86398000 "*" 1.35036426485158e-12 3.86629547363774e-12 100
"chr15" 86417001 86418000 "*" 2.88710388929303e-10 6.03399264731139e-10 -100
"chr15" 86441001 86442000 "*" 0.000139451556673498 0.000127724714298755
58.3333333333333
"chr15" 86740001 86741000 "*" 7.0006535457523e-08 1.03695832512054e-07 -100
"chr15" 87116001 87117000 "*" 2.14109840968035e-10 4.57328463015937e-10
-80.7692307692308
"chr15" 87415001 87416000 "*" 4.07050848361123e-07 5.44261714716217e-07
-57.6923076923077
"chr15" 88533001 88534000 "*" 8.58968451922237e-12 2.213554983903e-11 -100
"chr15" 88604001 88605000 "*" 1.57823754065589e-11 3.92691043510968e-11
-73.8095238095238
"chr15" 88622001 88623000 "*" 5.55111512312578e-16 2.36485094870365e-15
58.960974397216
"chr15" 88652001 88653000 "*" 2.68141064907468e-12 7.40011637970864e-12
-66.6666666666667

"chr15" 88727001 88728000 "*" 3.90465437760668e-13 1.19307893943277e-12 100
"chr15" 88765001 88766000 "*" 2.43005615629954e-09 4.45014222280278e-09 -100
"chr15" 88766001 88767000 "*" 2.02327044007689e-12 5.65300384792621e-12 100
"chr15" 88783001 88784000 "*" 0.000127939268828592 0.000117929091402497
-52.1821631878558
"chr15" 88941001 88942000 "*" 0.0001808540354854 0.000162597451724947
-61.5384615384615
"chr15" 88945001 88946000 "*" 6.15840711759574e-13 1.83676803572802e-12 -100
"chr15" 89103001 89104000 "*" 2.02718650754719e-08 3.24159766118952e-08
-93.3333333333333
"chr15" 89104001 89105000 "*" 1.14124265593318e-11 2.89138062669327e-11 100
"chr15" 89155001 89156000 "*" 0 0 82.2033898305085
"chr15" 89248001 89249000 "*" 0 0 62.5738230137539
"chr15" 89309001 89310000 "*" 2.69007038866675e-13 8.38831022628993e-13 -100
"chr15" 89317001 89318000 "*" 9.5812247025151e-14 3.15667542866807e-13 100
"chr15" 89325001 89326000 "*" 2.88322787866946e-09 5.22518936556917e-09
-84.9056603773585
"chr15" 89336001 89337000 "*" 8.25538539750426e-08 1.2109413635802e-07
-63.8888888888889
"chr15" 89357001 89358000 "*" 4.58850735185479e-11 1.07119583259697e-10
54.1666666666667
"chr15" 89560001 89561000 "*" 4.79673107900425e-08 7.29274309401268e-08
-57.5757575757576
"chr15" 89561001 89562000 "*" 6.9939528390961e-08 1.03695832512054e-07
-54.5454545454545
"chr15" 89583001 89584000 "*" 1.10615960835503e-11 2.81212224277682e-11
77.1428571428571
"chr15" 89584001 89585000 "*" 8.99758045846966e-12 2.31470582460068e-11
-79.5918367346939
"chr15" 89637001 89638000 "*" 1.11022302462516e-16 5.03662826618488e-16
-82.1428571428571
"chr15" 89761001 89762000 "*" 3.56775498033812e-10 7.35898629809829e-10 -100
"chr15" 89877001 89878000 "*" 0 0 -100
"chr15" 89908001 89909000 "*" 0 0 82.089552238806
"chr15" 89913001 89914000 "*" 0 0 -62.5448028673835
"chr15" 89962001 89963000 "*" 2.94254883792533e-08 4.61645267024725e-08
61.1111111111111
"chr15" 89991001 89992000 "*" 2.34257058195908e-14 8.33889706074077e-14 100
"chr15" 90021001 90022000 "*" 1.39521727504643e-12 3.98923834080818e-12
78.6885245901639
"chr15" 90029001 90030000 "*" 0 0 100
"chr15" 90036001 90037000 "*" 3.33066907387547e-15 1.30941888440006e-14
96.9135802469136
"chr15" 90046001 90047000 "*" 5.40314903929584e-10 1.08933786764431e-09
55.8823529411765
"chr15" 90206001 90207000 "*" 0.000792090690089564 0.000639295093468792
52.3809523809524
"chr15" 90233001 90234000 "*" 0 0 -100
"chr15" 90371001 90372000 "*" 0 0 -100
"chr15" 90436001 90437000 "*" 1.27542421068938e-12 3.66506798413395e-12
60.2409638554217
"chr15" 90455001 90456000 "*" 0 0 -73.469387755102

"chr15" 90456001 90457000 "*" 0 0 -55.5428571428571
"chr15" 90546001 90547000 "*" 0 0 -96.6442953020134
"chr15" 90648001 90649000 "*" 1.83186799063151e-14 6.59696304569048e-14 -100
"chr15" 90660001 90661000 "*" 2.88710388929303e-10 6.03399264731139e-10 -100
"chr15" 90744001 90745000 "*" 0 0 100
"chr15" 90755001 90756000 "*" 0 0 -86.9047619047619
"chr15" 90789001 90790000 "*" 4.23745471722636e-06 4.90563267622047e-06
65.3061224489796
"chr15" 90792001 90793000 "*" 0 0 100
"chr15" 90800001 90801000 "*" 0 0 100
"chr15" 90883001 90884000 "*" 0 0 100
"chr15" 91004001 91005000 "*" 1.26565424807268e-14 4.63923203146481e-14 -100
"chr15" 91208001 91209000 "*" 0 0 -54.2880704978676
"chr15" 91209001 91210000 "*" 0 0 -100
"chr15" 91317001 91318000 "*" 1.20591092667155e-10 2.66075324129686e-10 100
"chr15" 91487001 91488000 "*" 4.4545922506245e-11 1.04124467045374e-10
57.6923076923077
"chr15" 91498001 91499000 "*" 0 0 -83.4437086092715
"chr15" 91557001 91558000 "*" 1.28115100750392e-08 2.11368493104501e-08
61.5384615384615
"chr15" 91643001 91644000 "*" 0 0 79.2294865559647
"chr15" 91829001 91830000 "*" 4.08209022140227e-11 9.57770690255255e-11 -100
"chr15" 92031001 92032000 "*" 1.92946192356658e-09 3.59193011611483e-09 100
"chr15" 92113001 92114000 "*" 3.04324343503026e-11 7.26599935673121e-11 100
"chr15" 92460001 92461000 "*" 1.11022302462516e-16 5.03662826618488e-16 -100
"chr15" 92516001 92517000 "*" 9.32032229172819e-13 2.7181920310254e-12 100
"chr15" 92543001 92544000 "*" 1.13140401492018e-08 1.87801380787728e-08 -100
"chr15" 92561001 92562000 "*" 9.47020240005259e-14 3.12491189162604e-13
-52.5423728813559
"chr15" 92575001 92576000 "*" 4.14335232790108e-13 1.26153063645578e-12 -100
"chr15" 92577001 92578000 "*" 5.37201338870474e-05 5.25702894772176e-05
51.5151515151515
"chr15" 92705001 92706000 "*" 1.98124849859482e-10 4.25157611354621e-10 100
"chr15" 92887001 92888000 "*" 0 0 -100
"chr15" 92938001 92939000 "*" 0 0 50.568177906127
"chr15" 92940001 92941000 "*" 0 0 -100
"chr15" 92979001 92980000 "*" 1.0163536678931e-11 2.59878604010155e-11
62.0689655172414
"chr15" 92987001 92988000 "*" 3.33066907387547e-16 1.4495649018245e-15 -65
"chr15" 93007001 93008000 "*" 5.5300029124794e-06 6.29908078812909e-06
63.6363636363636
"chr15" 93071001 93072000 "*" 3.90802242102861e-05 3.90618161178078e-05
-64.2857142857143
"chr15" 93122001 93123000 "*" 2.02327044007689e-12 5.65300384792621e-12 -100
"chr15" 93124001 93125000 "*" 1.11022302462516e-16 5.03662826618488e-16 75
"chr15" 93129001 93130000 "*" 3.34128893442198e-08 5.19772864618523e-08 -100
"chr15" 93161001 93162000 "*" 2.22044604925031e-16 9.81641919380259e-16
92.8571428571429
"chr15" 93176001 93177000 "*" 1.72216563232297e-09 3.22698362203137e-09
51.9334049409237
"chr15" 93181001 93182000 "*" 1.56442647725186e-08 2.55431203133619e-08
-57.1428571428571

"chr15" 93188001 93189000 "*" 2.88710388929303e-10 6.03399264731139e-10 100
"chr15" 93198001 93199000 "*" 0 0 53.1584062196307
"chr15" 93330001 93331000 "*" 8.7349629751543e-09 1.47505871898511e-08 -100
"chr15" 93576001 93577000 "*" 1.80745751698907e-10 3.89869951273778e-10
-72.4137931034483
"chr15" 93581001 93582000 "*" 6.94999613415348e-14 2.33262715411123e-13
70.5882352941177
"chr15" 93593001 93594000 "*" 6.75492994872684e-12 1.76648020100745e-11
79.2452830188679
"chr15" 93608001 93609000 "*" 2.22044604925031e-16 9.81641919380259e-16
-51.1111111111111
"chr15" 93618001 93619000 "*" 2.78563838662649e-11 6.71437770440712e-11
-59.4476744186046
"chr15" 93630001 93631000 "*" 3.68352487728485e-05 3.69658679362228e-05
-52.1739130434783
"chr15" 93632001 93633000 "*" 0 0 56.1103772859012
"chr15" 93670001 93671000 "*" 1.99840144432528e-12 5.59476580335607e-12 100
"chr15" 93705001 93706000 "*" 1.16408228700848e-06 1.46479698533027e-06
66.6666666666667
"chr15" 93722001 93723000 "*" 0 0 -69.5121951219512
"chr15" 93743001 93744000 "*" 0 0 -100
"chr15" 93783001 93784000 "*" 0 0 -100
"chr15" 93842001 93843000 "*" 2.00227675550835e-08 3.20459176617961e-08 100
"chr15" 93850001 93851000 "*" 7.43206607367597e-11 1.68700530855537e-10
-83.3333333333333
"chr15" 93887001 93888000 "*" 3.61932706027801e-14 1.25777907157994e-13 -100
"chr15" 93902001 93903000 "*" 3.63445940010365e-11 8.58565209111204e-11 100
"chr15" 93957001 93958000 "*" 8.53897275110427e-09 1.44811964465278e-08
-53.0612244897959
"chr15" 94115001 94116000 "*" 9.04570973681018e-10 1.76414389822173e-09 -100
"chr15" 94247001 94248000 "*" 0 0 100
"chr15" 94367001 94368000 "*" 8.7349629751543e-09 1.47505871898511e-08 -100
"chr15" 94544001 94545000 "*" 1.14124265593318e-11 2.89138062669327e-11 100
"chr15" 94576001 94577000 "*" 2.38031816479634e-13 7.47299807843568e-13 -100
"chr15" 94837001 94838000 "*" 6.71435517740626e-07 8.73129962575465e-07
66.6666666666667
"chr15" 95014001 95015000 "*" 2.1094237467878e-15 8.47434540879246e-15 100
"chr15" 95194001 95195000 "*" 2.00227675550835e-08 3.20459176617961e-08 100
"chr15" 95217001 95218000 "*" 4.95459229199469e-12 1.32001166097957e-11
56.4356435643564
"chr15" 95576001 95577000 "*" 8.71525074330748e-14 2.88848349331391e-13 100
"chr15" 96084001 96085000 "*" 8.7349629751543e-09 1.47505871898511e-08 -100
"chr15" 96114001 96115000 "*" 2.54010507072522e-08 4.0129289879085e-08
61.9047619047619
"chr15" 96211001 96212000 "*" 2.88779000712225e-11 6.92935809466068e-11 100
"chr15" 96450001 96451000 "*" 3.10071932616562e-05 3.15124728670996e-05 -52
"chr15" 96712001 96713000 "*" 3.52704532247117e-11 8.35581292290093e-11 -100
"chr15" 96754001 96755000 "*" 5.794809077031e-12 1.52911192757929e-11 100
"chr15" 96846001 96847000 "*" 4.78841410966879e-11 1.11468187156588e-10 100
"chr15" 96876001 96877000 "*" 0 0 -63.3838383838384
"chr15" 96890001 96891000 "*" 0 0 -87.8048780487805
"chr15" 96953001 96954000 "*" 0 0 51.8269021482232

"chr15" 97012001 97013000 "*" 4.73234496034536e-09 8.30627300264826e-09 100
"chr15" 97043001 97044000 "*" 3.39440209273789e-09 6.10422192745839e-09
-61.5384615384615
"chr15" 97125001 97126000 "*" 0.000216683727490286 0.000192426836197816 -70
"chr15" 97430001 97431000 "*" 0 0 59.5238095238095
"chr15" 98237001 98238000 "*" 9.32032229172819e-13 2.7181920310254e-12 100
"chr15" 98379001 98380000 "*" 1.67299207820548e-08 2.71377429441055e-08 100
"chr15" 98543001 98544000 "*" 4.83744390433749e-06 5.55756940206571e-06 -70
"chr15" 98707001 98708000 "*" 8.69011083648452e-07 1.11229659818976e-06
66.6666666666667
"chr15" 98829001 98830000 "*" 0 0 97.2222222222222
"chr15" 98863001 98864000 "*" 3.35319686006752e-06 3.94441403675924e-06
61.4285714285714
"chr15" 98970001 98971000 "*" 1.41109876605761e-06 1.75583535616452e-06 -90
"chr15" 98977001 98978000 "*" 2.88710388929303e-10 6.03399264731139e-10 100
"chr15" 98987001 98988000 "*" 0 0 86.3013698630137
"chr15" 99046001 99047000 "*" 4.80936401814347e-11 1.11934004323867e-10 66.25
"chr15" 99049001 99050000 "*" 2.32758257112664e-12 6.46922413725066e-12 96.875
"chr15" 99085001 99086000 "*" 1.64320779205696e-11 4.08065373760084e-11
92.3076923076923
"chr15" 99091001 99092000 "*" 0 0 81.3047711781889
"chr15" 99237001 99238000 "*" 1.92946192356658e-09 3.59193011611483e-09 -100
"chr15" 99248001 99249000 "*" 5.295763827462e-14 1.80214784640623e-13 -100
"chr15" 99271001 99272000 "*" 3.9190872769268e-14 1.35416418933905e-13 -100
"chr15" 99298001 99299000 "*" 2.88779000712225e-11 6.92935809466068e-11 -100
"chr15" 99309001 99310000 "*" 1.07897108138078e-05 1.17605389980271e-05
-65.8536585365854
"chr15" 99418001 99419000 "*" 3.25818809512324e-05 3.29735679006858e-05
-66.6666666666667
"chr15" 99458001 99459000 "*" 1.88737914186277e-15 7.62011598380321e-15 100
"chr15" 99484001 99485000 "*" 5.71347714695136e-07 7.50240854262333e-07
72.7477477477478
"chr15" 99601001 99602000 "*" 0 0 50.0890560063329
"chr15" 99608001 99609000 "*" 0 0 100
"chr15" 99632001 99633000 "*" 6.59550958292954e-09 1.13456194382773e-08 100
"chr15" 99645001 99646000 "*" 0 0 90.2463156798417
"chr15" 99663001 99664000 "*" 2.42094122526737e-11 5.8788232787879e-11
-56.4685314685315
"chr15" 100034001 100035000 "*" 0 0 -60.377358490566
"chr15" 100266001 100267000 "*" 2.09721129351692e-12 5.85233993281462e-12
55.5555555555556
"chr15" 100448001 100449000 "*" 4.01313771103418e-09 7.11882087237587e-09 100
"chr15" 100449001 100450000 "*" 7.97140131680862e-14 2.65641277678906e-13 100
"chr15" 100457001 100458000 "*" 0 0 100
"chr15" 100513001 100514000 "*" 0 0 100
"chr15" 100521001 100522000 "*" 6.31650287630237e-12 1.66067947258909e-11
-90.9090909090909
"chr15" 100523001 100524000 "*" 4.44089209850063e-16 1.91071758245033e-15 -100
"chr15" 100543001 100544000 "*" 0 0 80
"chr15" 100593001 100594000 "*" 3.73757139926489e-06 4.36534316905481e-06
-68.2926829268293
"chr15" 100594001 100595000 "*" 1.17905685215192e-13 3.83653245040640e-13 100

"chr15" 100640001 100641000 "*" 3.02691205433803e-12 8.28046626423617e-12
71.2765957446808
"chr15" 100641001 100642000 "*" 2.00227675550835e-08 3.20459176617961e-08 -100
"chr15" 100651001 100652000 "*" 0 0 -100
"chr15" 100695001 100696000 "*" 2.66453525910038e-14 9.40074020513994e-14 -100
"chr15" 100701001 100702000 "*" 0 0 66.6666666666667
"chr15" 100708001 100709000 "*" 4.01313771103418e-09 7.11882087237587e-09 -100
"chr15" 100739001 100740000 "*" 1.11022302462516e-16 5.03662826618488e-16 100
"chr15" 100741001 100742000 "*" 0.000241621869001674 0.000212792315955059
57.6923076923077
"chr15" 100746001 100747000 "*" 0 0 88.3720930232558
"chr15" 100754001 100755000 "*" 0 0 -100
"chr15" 100760001 100761000 "*" 3.02470549384815e-07 4.11709339200624e-07
57.4468085106383
"chr15" 100800001 100801000 "*" 2.08814465718632e-09 3.86232567960463e-09 100
"chr15" 100820001 100821000 "*" 1.60460105126381e-05 1.70453625649539e-05
-68.5714285714286
"chr15" 100844001 100845000 "*" 8.8342340305303e-06 9.75817539917938e-06
-78.5714285714286
"chr15" 100855001 100856000 "*" 1.14059791278542e-07 1.64232348483281e-07
54.5454545454545
"chr15" 101107001 101108000 "*" 1.93720595120794e-11 4.76309924836636e-11 100
"chr15" 101141001 101142000 "*" 0 0 -100
"chr15" 101183001 101184000 "*" 3.68371999570627e-13 1.13149976091305e-12 100
"chr15" 101254001 101255000 "*" 2.3990809339125e-12 6.65441738642456e-12 100
"chr15" 101260001 101261000 "*" 1.0774605917474e-06 1.36177735814262e-06
-53.125
"chr15" 101329001 101330000 "*" 0 0 100
"chr15" 101365001 101366000 "*" 1.89581683684992e-12 5.32311367606535e-12 -100
"chr15" 101390001 101391000 "*" 0 0 94.2821628340584
"chr15" 101611001 101612000 "*" 3.03979064142368e-13 9.42350883309283e-13 100
"chr15" 101625001 101626000 "*" 8.66760219153662e-10 1.69631365441799e-09
-71.7948717948718
"chr15" 101643001 101644000 "*" 6.21724893790088e-15 2.36606385879553e-14
52.3264137437366
"chr15" 101647001 101648000 "*" 5.01025332333427e-10 1.01358031135889e-09 100
"chr15" 101667001 101668000 "*" 2.08814465718632e-09 3.86232567960463e-09 -100
"chr15" 101687001 101688000 "*" 0 0 100
"chr15" 101688001 101689000 "*" 5.7065463465733e-14 1.93318145576218e-13 100
"chr15" 101773001 101774000 "*" 2.33590924381133e-13 7.3418676838825e-13 -100
"chr15" 101777001 101778000 "*" 0 0 100
"chr15" 101808001 101809000 "*" 4.14335232790108e-13 1.26153063645578e-12 100
"chr15" 101947001 101948000 "*" 0 0 90.728476821192
"chr15" 101965001 101966000 "*" 0 0 100
"chr15" 102010001 102011000 "*" 1.39331806092713e-07 1.98398036179707e-07
77.2727272727273
"chr15" 102034001 102035000 "*" 1.87627691161651e-14 6.74814792086625e-14
74.2857142857143
"chr15" 102062001 102063000 "*" 7.105427357601e-15 2.68362283258525e-14 100
"chr15" 102064001 102065000 "*" 0 0 -69.2307692307692
"chr15" 102083001 102084000 "*" 0 0 -79.3793385055125
"chr15" 102315001 102316000 "*" 9.80763359414993e-10 1.8980032424715e-09 100

"chr15" 102319001 102320000 "*" 1.88737914186277e-15 7.62011598380321e-15 -100
"chr16" 109001 110000 "*" 2.68673971959288e-14 9.47266815431045e-14 -100
"chr16" 115001 116000 "*" 2.01809636779515e-08 3.22829990076203e-08
52.3809523809524
"chr16" 148001 149000 "*" 2.52242671194836e-13 7.90130125188633e-13
-53.1631097560976
"chr16" 227001 228000 "*" 0 0 64.4444444444444
"chr16" 300001 301000 "*" 0 0 -100
"chr16" 307001 308000 "*" 2.64951482975562e-09 4.82186011375752e-09 -100
"chr16" 330001 331000 "*" 0 0 71.7271660654464
"chr16" 408001 409000 "*" 2.45559128586592e-11 5.95984124534912e-11
51.063829787234
"chr16" 447001 448000 "*" 0 0 58.3333333333333
"chr16" 474001 475000 "*" 5.95079541199084e-14 2.01110497157026e-13
-54.4642857142857
"chr16" 476001 477000 "*" 0 0 57.6023391812866
"chr16" 477001 478000 "*" 0 0 -100
"chr16" 500001 501000 "*" 1.07119524361821e-09 2.06414893159545e-09
-66.6666666666667
"chr16" 749001 750000 "*" 0 0 54.1329380914377
"chr16" 754001 755000 "*" 5.38346356382391e-09 9.39082247461634e-09
51.2731481481482
"chr16" 757001 758000 "*" 0 0 100
"chr16" 759001 760000 "*" 0 0 50.5996691480563
"chr16" 762001 763000 "*" 9.08108033215171e-11 2.03407753381119e-10 -100
"chr16" 769001 770000 "*" 0 0 76.25
"chr16" 791001 792000 "*" 0 0 80
"chr16" 826001 827000 "*" 1.11022302462516e-16 5.03662826618488e-16
83.8235294117647
"chr16" 856001 857000 "*" 0 0 -98.7096774193548
"chr16" 864001 865000 "*" 3.12578478622871e-06 3.69509475058822e-06
52.8985507246377
"chr16" 871001 872000 "*" 3.56678575563762e-10 7.35898629809829e-10
-51.4814814814815
"chr16" 872001 873000 "*" 1.11022302462516e-16 5.03662826618488e-16
79.4285714285714
"chr16" 884001 885000 "*" 2.78682632526284e-11 6.71704692966937e-11 70
"chr16" 890001 891000 "*" 1.07525099934946e-12 3.11507165357469e-12
53.3533105022831
"chr16" 914001 915000 "*" 1.52794177310511e-09 2.87642564232045e-09 100
"chr16" 947001 948000 "*" 0 0 74.7076023391813
"chr16" 1078001 1079000 "*" 0 0 53.0841121495327
"chr16" 1085001 1086000 "*" 4.98299776274802e-06 5.71345770314862e-06
-59.4936708860759
"chr16" 1086001 1087000 "*" 0 0 73.6196319018405
"chr16" 1102001 1103000 "*" 3.91687831358123e-06 4.55931529118571e-06
61.6666666666667
"chr16" 1108001 1109000 "*" 0 0 67.2413793103448
"chr16" 1184001 1185000 "*" 0 0 -58.8888888888889
"chr16" 1202001 1203000 "*" 0 0 67.848759830611
"chr16" 1229001 1230000 "*" 1.4432899320127e-15 5.90750815956055e-15 100
"chr16" 1247001 1248000 "*" 8.79629702410512e-13 2.57472818784556e-12

78.0821917808219
"chr16" 1291001 1292000 "*" 9.32587340685131e-15 3.47706353427762e-14
-57.7697689209243
"chr16" 1293001 1294000 "*" 2.39599229345799e-10 5.06957473192833e-10
66.5266106442577
"chr16" 1298001 1299000 "*" 2.20712337295481e-13 6.94992175498525e-13
-61.2244897959184
"chr16" 1315001 1316000 "*" 0 0 53.0043092934385
"chr16" 1320001 1321000 "*" 2.44249065417534e-15 9.75180348030994e-15 100
"chr16" 1352001 1353000 "*" 7.84268325837445e-08 1.15357846405799e-07
54.8387096774194
"chr16" 1353001 1354000 "*" 0 0 90
"chr16" 1358001 1359000 "*" 0 0 -100
"chr16" 1387001 1388000 "*" 6.65961096515399e-05 6.42248145283162e-05
51.7647058823529
"chr16" 1392001 1393000 "*" 0 0 -50.411376333164
"chr16" 1465001 1466000 "*" 0 0 98.7951807228916
"chr16" 1473001 1474000 "*" 1.26453891802214e-09 2.41208143008691e-09
57.5757575757576
"chr16" 1666001 1667000 "*" 6.92287338566189e-11 1.57605630124247e-10 -100
"chr16" 1767001 1768000 "*" 8.7349629751543e-09 1.47505871898511e-08 100
"chr16" 1772001 1773000 "*" 0 0 -100
"chr16" 1820001 1821000 "*" 0 0 -60.6306306306306
"chr16" 1824001 1825000 "*" 0 0 -62.7151102481581
"chr16" 1857001 1858000 "*" 2.06926697998711e-11 5.05664708451183e-11
-89.1891891891892
"chr16" 1863001 1864000 "*" 5.00231644995353e-09 8.75507251377598e-09
-52.9411764705882
"chr16" 1889001 1890000 "*" 0.000722189611975566 0.000586999420811564
57.1428571428571
"chr16" 1949001 1950000 "*" 1.55828960846094e-08 2.54469062159849e-08
-77.5700934579439
"chr16" 1967001 1968000 "*" 0 0 63.2777916400972
"chr16" 2001001 2002000 "*" 1.68292180480201e-06 2.06973267269277e-06
73.3333333333333
"chr16" 2238001 2239000 "*" 1.20679412018987e-08 1.99701324873047e-08
-64.2857142857143
"chr16" 2244001 2245000 "*" 1.52331027236663e-07 2.15297901653729e-07 -75
"chr16" 2246001 2247000 "*" 0 0 58.5682898952393
"chr16" 2302001 2303000 "*" 5.84981698081855e-07 7.67032732489292e-07 60
"chr16" 2342001 2343000 "*" 1.39779633423487e-07 1.98463037958589e-07 100
"chr16" 2353001 2354000 "*" 2.66453525910038e-15 1.05861327033776e-14 100
"chr16" 2384001 2385000 "*" 0 0 -100
"chr16" 2416001 2417000 "*" 0 0 -100
"chr16" 2571001 2572000 "*" 1.11022302462516e-15 4.59817122935606e-15
-78.2608695652174
"chr16" 2637001 2638000 "*" 9.80763359414993e-10 1.8980032424715e-09 -100
"chr16" 2673001 2674000 "*" 6.63913368725844e-14 2.23251502617671e-13
69.6969696969697
"chr16" 2714001 2715000 "*" 0 0 -100
"chr16" 2775001 2776000 "*" 0 0 98.1818181818182
"chr16" 2788001 2789000 "*" 1.54967423182661e-06 1.91685454663215e-06

54.5454545454545
"chr16" 2790001 2791000 "*" 5.95544724646402e-11 1.37002386252927e-10
65.7142857142857
"chr16" 2818001 2819000 "*" 0 0 -61.6013723769249
"chr16" 2828001 2829000 "*" 4.34108304858682e-12 1.16410839476376e-11 55.2
"chr16" 2858001 2859000 "*" 3.76365605347928e-14 1.3043487970679e-13 100
"chr16" 2863001 2864000 "*" 3.80806497446429e-14 1.31902680029991e-13
86.1386138613861
"chr16" 2877001 2878000 "*" 0 0 50.4273504273504
"chr16" 2888001 2889000 "*" 1.15463194561016e-14 4.25829614430727e-14 100
"chr16" 2908001 2909000 "*" 1.80966353013901e-14 6.52206904171103e-14
-62.6865671641791
"chr16" 2940001 2941000 "*" 4.87947221516905e-08 7.41205346767756e-08 -75
"chr16" 2949001 2950000 "*" 9.65729496371637e-10 1.87255912493232e-09 100
"chr16" 2963001 2964000 "*" 1.02853281447324e-11 2.62799404378762e-11
63.1578947368421
"chr16" 3029001 3030000 "*" 6.32733077043923e-09 1.09425359504913e-08
-51.9047619047619
"chr16" 3033001 3034000 "*" 0 0 -100
"chr16" 3038001 3039000 "*" 2.35593766717557e-11 5.72697669589915e-11
-54.7877358490566
"chr16" 3041001 3042000 "*" 0 0 -85.5421686746988
"chr16" 3047001 3048000 "*" 0 0 72.5
"chr16" 3061001 3062000 "*" 0 0 -80.3571428571429
"chr16" 3086001 3087000 "*" 4.21884749357559e-15 1.63874445239189e-14
50.5589879376287
"chr16" 3090001 3091000 "*" 4.26951363152739e-10 8.72510873516957e-10 -100
"chr16" 3161001 3162000 "*" 9.67229975556982e-07 1.22970859507725e-06
63.3333333333333
"chr16" 3285001 3286000 "*" 6.88338275267597e-15 2.60518659957509e-14 100
"chr16" 3318001 3319000 "*" 0.000394212930368454 0.000335045023868536
58.8235294117647
"chr16" 3423001 3424000 "*" 0 0 100
"chr16" 3479001 3480000 "*" 4.01313771103418e-09 7.11882087237587e-09 -100
"chr16" 3492001 3493000 "*" 1.21332608793434e-06 1.52278699490218e-06
-66.6666666666667
"chr16" 3598001 3599000 "*" 0 0 75.8896151053014
"chr16" 3615001 3616000 "*" 7.7715611723761e-16 3.26213507634405e-15
-86.2595419847328
"chr16" 3651001 3652000 "*" 1.47041824516414e-06 1.82482785714399e-06
65.7894736842105
"chr16" 3701001 3702000 "*" 1.42687766047089e-08 2.34072760373255e-08
57.5757575757576
"chr16" 3772001 3773000 "*" 3.56775498033812e-10 7.35898629809829e-10 100
"chr16" 3775001 3776000 "*" 4.18887147191072e-13 1.2739946038722e-12 -100
"chr16" 3925001 3926000 "*" 1.12055920098442e-11 2.84500040664577e-11 100
"chr16" 3960001 3961000 "*" 2.08814465718632e-09 3.86232567960463e-09 -100
"chr16" 4071001 4072000 "*" 1.74375081041234e-09 3.26500904606579e-09
-80.9523809523809
"chr16" 4162001 4163000 "*" 2.27169172362096e-09 4.18380845023679e-09
-75.7575757575758
"chr16" 4186001 4187000 "*" 3.90465437760668e-13 1.19307893943277e-12 100

"chr16" 4219001 4220000 "*" 0 0 -100
"chr16" 4267001 4268000 "*" 8.17124146124115e-14 2.71877137546954e-13 67.1428571428571
"chr16" 4268001 4269000 "*" 0 0 64.2857142857143
"chr16" 4275001 4276000 "*" 6.99440505513849e-15 2.64480529781883e-14 100
"chr16" 4278001 4279000 "*" 1.98325800226939e-11 4.85612402473442e-11 -100
"chr16" 4289001 4290000 "*" 4.44089209850063e-16 1.91071758245033e-15 -93.75
"chr16" 4315001 4316000 "*" 1.12376774552558e-12 3.24761818527664e-12 100
"chr16" 4324001 4325000 "*" 0 0 100
"chr16" 4338001 4339000 "*" 4.7115035605505e-05 4.65252173536101e-05 -60.2941176470588
"chr16" 4340001 4341000 "*" 1.22228082877207e-07 1.75285564709212e-07 64.5161290322581
"chr16" 4357001 4358000 "*" 0 0 -100
"chr16" 4366001 4367000 "*" 0 0 -61.9205298013245
"chr16" 4367001 4368000 "*" 0 0 -71.4285714285714
"chr16" 4419001 4420000 "*" 0 0 -100
"chr16" 4421001 4422000 "*" 0 0 -81.283422459893
"chr16" 4452001 4453000 "*" 0 0 71.5328467153285
"chr16" 4460001 4461000 "*" 1.09092727074156e-08 1.820302037906e-08 81.3559322033898
"chr16" 4465001 4466000 "*" 0.000182145353712748 0.000163691361803338 54.8387096774194
"chr16" 4466001 4467000 "*" 0 0 -65.3878672585077
"chr16" 4524001 4525000 "*" 0 0 61.1111111111111
"chr16" 4561001 4562000 "*" 3.85641518718671e-11 9.08938008805696e-11 64.8648648648649
"chr16" 4588001 4589000 "*" 0 0 52.1735322964831
"chr16" 4648001 4649000 "*" 3.52704532247117e-11 8.35581292290093e-11 100
"chr16" 4653001 4654000 "*" 0 0 73.8461538461538
"chr16" 4744001 4745000 "*" 0 0 82.046568627451
"chr16" 4787001 4788000 "*" 8.52140580320793e-12 2.19817259951489e-11 100
"chr16" 4828001 4829000 "*" 4.60677497663431e-05 4.55590100832258e-05 -57.1428571428571
"chr16" 4834001 4835000 "*" 1.11022302462516e-16 5.03662826618488e-16 -75.8620689655172
"chr16" 4866001 4867000 "*" 2.64325346821526e-06 3.15856923971574e-06 53.8461538461538
"chr16" 4974001 4975000 "*" 0 0 100
"chr16" 4997001 4998000 "*" 6.37490060739765e-13 1.89496793587877e-12 -100
"chr16" 5017001 5018000 "*" 6.73690652419623e-06 7.57247768858545e-06 -59.2592592592593
"chr16" 5042001 5043000 "*" 1.15463194561016e-14 4.25829614430727e-14 100
"chr16" 5051001 5052000 "*" 0 0 100
"chr16" 5060001 5061000 "*" 0 0 100
"chr16" 5082001 5083000 "*" 4.02167188440217e-12 1.08266700497926e-11 100
"chr16" 5150001 5151000 "*" 0 0 73.5849056603774
"chr16" 5198001 5199000 "*" 0 0 84.375
"chr16" 5272001 5273000 "*" 2.07389660999979e-13 6.56162453403164e-13 -65.7894736842105
"chr16" 5289001 5290000 "*" 1.36557432028894e-14 4.98620244630348e-14 52.6881720430108

"chr16" 5301001 5302000 "*" 3.46706228993021e-06 4.06854288553042e-06
66.2790697674419
"chr16" 5356001 5357000 "*" 3.62240519058421e-06 4.24051313235319e-06
-66.6666666666667
"chr16" 5364001 5365000 "*" 8.7349629751543e-09 1.47505871898511e-08 -100
"chr16" 5424001 5425000 "*" 5.89841205486108e-05 5.73627738974644e-05
-54.5454545454545
"chr16" 5633001 5634000 "*" 2.88710388929303e-10 6.03399264731139e-10 100
"chr16" 5638001 5639000 "*" 1.34559030584569e-13 4.35581312463895e-13 -100
"chr16" 5785001 5786000 "*" 2.70847788641504e-11 6.53935510679517e-11
66.6666666666667
"chr16" 5912001 5913000 "*" 2.39607067674674e-05 2.47819195194925e-05 56.25
"chr16" 5946001 5947000 "*" 2.73625566649116e-12 7.52483363579137e-12 100
"chr16" 5970001 5971000 "*" 1.61755827587928e-06 1.99516227329402e-06
69.2307692307692
"chr16" 6053001 6054000 "*" 3.88169574261354e-09 6.93447853396753e-09
66.6666666666667
"chr16" 6071001 6072000 "*" 9.47029188735904e-07 1.2058457158965e-06
-71.0526315789474
"chr16" 6473001 6474000 "*" 8.89428530825853e-11 1.99846326244974e-10
66.6666666666667
"chr16" 6934001 6935000 "*" 4.08209022140227e-11 9.57770690255255e-11 -100
"chr16" 7136001 7137000 "*" 0 0 100
"chr16" 7368001 7369000 "*" 5.8292493054779e-10 1.16899936252519e-09
-75.3623188405797
"chr16" 7414001 7415000 "*" 0 0 100
"chr16" 7444001 7445000 "*" 4.71438321714146e-09 8.29832308503069e-09
64.7058823529412
"chr16" 7469001 7470000 "*" 2.42062481170535e-10 5.12000498093754e-10
-68.4210526315789
"chr16" 7489001 7490000 "*" 3.18729389880978e-07 4.32442708000648e-07
-59.4594594594595
"chr16" 7867001 7868000 "*" 2.93503829018604e-07 4.00242383025006e-07
-73.5849056603774
"chr16" 8497001 8498000 "*" 0 0 100
"chr16" 8526001 8527000 "*" 0 0 -100
"chr16" 8573001 8574000 "*" 6.66133814775094e-16 2.81595744474255e-15 100
"chr16" 8700001 8701000 "*" 1.11022302462516e-16 5.03662826618488e-16 100
"chr16" 8788001 8789000 "*" 0 0 -58.75
"chr16" 8805001 8806000 "*" 1.06720694625917e-06 1.34971798994475e-06
-70.3703703703704
"chr16" 8807001 8808000 "*" 5.87773837679162e-06 6.66985247129035e-06
56.8627450980392
"chr16" 8971001 8972000 "*" 4.79616346638068e-14 1.64380445416596e-13 100
"chr16" 9046001 9047000 "*" 1.8357537712177e-12 5.17048205005927e-12
-90.1098901098901
"chr16" 9052001 9053000 "*" 1.53549306614043e-06 1.90032782662227e-06 -70.3125
"chr16" 9076001 9077000 "*" 1.74202673397517e-08 2.81935902938494e-08
-66.6666666666667
"chr16" 9078001 9079000 "*" 1.33226762955019e-15 5.48133041560118e-15
59.0909090909091
"chr16" 9232001 9233000 "*" 0 0 68.75

"chr16" 9264001 9265000 "*" 1.53482426767937e-09 2.88886061048214e-09
-66.0852713178295
"chr16" 9368001 9369000 "*" 3.77475828372553e-15 1.47379557022071e-14 -100
"chr16" 9575001 9576000 "*" 1.13140401492018e-08 1.87801380787728e-08 -100
"chr16" 9630001 9631000 "*" 3.52736506670226e-10 7.28757196367245e-10
80.5970149253731
"chr16" 9675001 9676000 "*" 8.57092175010621e-13 2.51149198099779e-12 -100
"chr16" 9825001 9826000 "*" 0 0 -100
"chr16" 9944001 9945000 "*" 1.12458486967171e-09 2.15740815043925e-09 100
"chr16" 10045001 10046000 "*" 6.4152538836737e-10 1.27477697728417e-09 100
"chr16" 10084001 10085000 "*" 2.1094237467878e-15 8.47434540879246e-15 100
"chr16" 10309001 10310000 "*" 5.69544411632705e-14 1.93067515690621e-13
-66.6666666666667
"chr16" 10332001 10333000 "*" 0 0 100
"chr16" 10343001 10344000 "*" 2.15125472990962e-10 4.58126659943874e-10 -100
"chr16" 10350001 10351000 "*" 4.01313771103418e-09 7.11882087237587e-09 -100
"chr16" 10609001 10610000 "*" 2.88779000712225e-11 6.92935809466068e-11 100
"chr16" 10626001 10627000 "*" 0 0 100
"chr16" 10632001 10633000 "*" 1.19148486860121e-05 1.29076284610434e-05 53.75
"chr16" 10672001 10673000 "*" 0 0 -100
"chr16" 10860001 10861000 "*" 6.35280716920761e-11 1.45637313256185e-10
-64.2857142857143
"chr16" 10916001 10917000 "*" 0 0 100
"chr16" 10962001 10963000 "*" 2.37587727269783e-14 8.44268514944447e-14 100
"chr16" 10988001 10989000 "*" 1.12458486967171e-09 2.15740815043925e-09 100
"chr16" 11292001 11293000 "*" 1.67299207820548e-08 2.71377429441055e-08 100
"chr16" 11304001 11305000 "*" 4.01319387371979e-05 4.00453665662627e-05
57.8947368421053
"chr16" 11398001 11399000 "*" 7.0006535457523e-08 1.03695832512054e-07 -100
"chr16" 11420001 11421000 "*" 2.74912315134657e-11 6.62942856376559e-11
52.7272727272727
"chr16" 11427001 11428000 "*" 1.66533453693773e-15 6.7629186866784e-15 -100
"chr16" 11461001 11462000 "*" 8.00762784913811e-05 7.62332949242677e-05
-57.6923076923077
"chr16" 11480001 11481000 "*" 0 0 100
"chr16" 11490001 11491000 "*" 0 0 75.3164556962025
"chr16" 11505001 11506000 "*" 0 0 100
"chr16" 11528001 11529000 "*" 8.42958793312309e-06 9.33879080874541e-06 68.75
"chr16" 11529001 11530000 "*" 1.23234755733392e-14 4.52749417164674e-14
-96.4664310954064
"chr16" 11542001 11543000 "*" 6.82826595266306e-09 1.17261842797601e-08
-66.6666666666667
"chr16" 11557001 11558000 "*" 2.22044604925031e-16 9.81641919380259e-16
71.4285714285714
"chr16" 11594001 11595000 "*" 0 0 96.6666666666667
"chr16" 11610001 11611000 "*" 7.85057341357032e-09 1.33886120340988e-08
51.063829787234
"chr16" 11611001 11612000 "*" 5.37803029284634e-05 5.26241567504211e-05 -60
"chr16" 11621001 11622000 "*" 1.55431223447522e-15 6.33705141101682e-15
-56.059009483667
"chr16" 11637001 11638000 "*" 3.20966742578532e-05 3.25417578837137e-05
52.7777777777778

"chr16" 11681001 11682000 "*" 3.59523522064364e-12 9.7631259172858e-12
63.2352941176471
"chr16" 11689001 11690000 "*" 0 0 100
"chr16" 11693001 11694000 "*" 5.60662627435704e-14 1.90149460749864e-13 -95
"chr16" 11706001 11707000 "*" 1.33226762955019e-15 5.48133041560118e-15 100
"chr16" 11707001 11708000 "*" 9.80882042256326e-13 2.85039184561802e-12
-61.0169491525424
"chr16" 11715001 11716000 "*" 2.62206354484373e-06 3.13437095470483e-06
62.0689655172414
"chr16" 11724001 11725000 "*" 4.2476976002459e-05 4.2228214892602e-05
-55.5555555555556
"chr16" 11731001 11732000 "*" 2.15705897588236e-10 4.59280628014627e-10
63.6363636363636
"chr16" 11734001 11735000 "*" 3.83548748317253e-12 1.03787135172492e-11
58.1395348837209
"chr16" 11769001 11770000 "*" 0 0 -56.5229416157314
"chr16" 11784001 11785000 "*" 1.98124849859482e-10 4.25157611354621e-10 100
"chr16" 11835001 11836000 "*" 0 0 -84.3227424749164
"chr16" 12071001 12072000 "*" 1.16478958478439e-06 1.46555427642147e-06
73.8095238095238
"chr16" 12094001 12095000 "*" 3.90831811358794e-12 1.05610567215048e-11 100
"chr16" 12120001 12121000 "*" 6.59550958292954e-09 1.13456194382773e-08 100
"chr16" 12178001 12179000 "*" 1.11022302462516e-16 5.03662826618488e-16
57.4468085106383
"chr16" 12205001 12206000 "*" 1.12458486967171e-09 2.15740815043925e-09 -100
"chr16" 12207001 12208000 "*" 1.11444718742248e-06 1.40552060619325e-06
53.7220843672457
"chr16" 12217001 12218000 "*" 0 0 100
"chr16" 12296001 12297000 "*" 0 0 100
"chr16" 12361001 12362000 "*" 9.99200722162641e-16 4.15382497462808e-15 79.6875
"chr16" 12401001 12402000 "*" 2.27472434237708e-05 2.3595924872599e-05
57.1428571428571
"chr16" 12431001 12432000 "*" 2.48931644186712e-08 3.93716649086578e-08
66.6666666666667
"chr16" 12450001 12451000 "*" 6.95242419190834e-10 1.37589605662581e-09
90.4109589041096
"chr16" 12462001 12463000 "*" 9.63829016598083e-12 2.46993429847588e-11 100
"chr16" 12468001 12469000 "*" 2.19383400335005e-11 5.34788778871231e-11
91.6666666666667
"chr16" 12515001 12516000 "*" 1.32899343752158e-08 2.18863374913517e-08
-85.5072463768116
"chr16" 12535001 12536000 "*" 1.11022302462516e-16 5.03662826618488e-16 -100
"chr16" 12567001 12568000 "*" 2.15125472990962e-10 4.58126659943874e-10 -100
"chr16" 12572001 12573000 "*" 1.39779633423487e-07 1.98463037958589e-07 100
"chr16" 12632001 12633000 "*" 9.08108033215171e-11 2.03407753381119e-10 -100
"chr16" 12649001 12650000 "*" 1.19043681579001e-08 1.97111834928772e-08
69.3877551020408
"chr16" 12666001 12667000 "*" 5.01025332333427e-10 1.01358031135889e-09 -100
"chr16" 12675001 12676000 "*" 0 0 100
"chr16" 12678001 12679000 "*" 4.86753970463383e-11 1.13145908161915e-10 100
"chr16" 12971001 12972000 "*" 3.04324343503026e-11 7.26599935673121e-11 100
"chr16" 13164001 13165000 "*" 7.0006535457523e-08 1.03695832512054e-07 100

"chr16" 13305001 13306000 "*" 6.59550958292954e-09 1.13456194382773e-08 100
"chr16" 13334001 13335000 "*" 4.73234496034536e-09 8.30627300264826e-09 -100
"chr16" 13491001 13492000 "*" 2.00227675550835e-08 3.20459176617961e-08 100
"chr16" 13673001 13674000 "*" 5.6621374255883e-15 2.16468580166064e-14 100
"chr16" 13923001 13924000 "*" 6.92287338566189e-11 1.57605630124247e-10 100
"chr16" 14094001 14095000 "*" 2.00227675550835e-08 3.20459176617961e-08 100
"chr16" 14390001 14391000 "*" 6.24654217240561e-10 1.2462108191303e-09 -100
"chr16" 14438001 14439000 "*" 0 0 100
"chr16" 14468001 14469000 "*" 0 0 -100
"chr16" 14474001 14475000 "*" 4.93267648948859e-11 1.14608902952886e-10
96.6666666666667
"chr16" 14493001 14494000 "*" 0 0 -100
"chr16" 14500001 14501000 "*" 5.55111512312578e-16 2.36485094870365e-15 100
"chr16" 14639001 14640000 "*" 2.79440914852103e-11 6.73046092948135e-11 -100
"chr16" 14773001 14774000 "*" 7.67056418382595e-11 1.7359448580318e-10 100
"chr16" 15138001 15139000 "*" 1.48735707250491e-06 1.84447733364305e-06
73.1707317073171
"chr16" 15150001 15151000 "*" 2.3715474029018e-12 6.58505481084979e-12
81.3084112149533
"chr16" 15489001 15490000 "*" 0 0 50.8413867860872
"chr16" 15502001 15503000 "*" 8.57092175010621e-13 2.51149198099779e-12 100
"chr16" 15529001 15530000 "*" 0 0 100
"chr16" 15603001 15604000 "*" 9.27670293693339e-07 1.18298419064093e-06
64.2857142857143
"chr16" 15609001 15610000 "*" 1.50623957750895e-12 4.28756717183481e-12 100
"chr16" 15657001 15658000 "*" 6.59550958292954e-09 1.13456194382773e-08 100
"chr16" 15731001 15732000 "*" 1.68753899743024e-14 6.09826805967439e-14 100
"chr16" 15745001 15746000 "*" 2.05374284245607e-09 3.81146853417661e-09
-91.6666666666667
"chr16" 15835001 15836000 "*" 3.73423514332671e-12 1.01162156045665e-11
58.8235294117647
"chr16" 15850001 15851000 "*" 8.3909468262533e-10 1.64494937720704e-09
85.1351351351351
"chr16" 15922001 15923000 "*" 6.24654217240561e-10 1.2462108191303e-09 -100
"chr16" 15958001 15959000 "*" 6.32827124036339e-15 2.40666772484995e-14 -100
"chr16" 16255001 16256000 "*" 3.95861121660346e-12 1.0684496262032e-11 100
"chr16" 17200001 17201000 "*" 1.87268534013185e-10 4.03417958341548e-10
-85.3333333333333
"chr16" 17255001 17256000 "*" 5.01025332333427e-10 1.01358031135889e-09 100
"chr16" 17439001 17440000 "*" 1.39779633423487e-07 1.98463037958589e-07 -100
"chr16" 17442001 17443000 "*" 5.07371922253697e-14 1.73287898516782e-13 100
"chr16" 17448001 17449000 "*" 3.87502474552548e-10 7.96362339341696e-10 75
"chr16" 17476001 17477000 "*" 7.0006535457523e-08 1.03695832512054e-07 -100
"chr16" 17518001 17519000 "*" 1.66533453693773e-15 6.7629186866784e-15
97.8723404255319
"chr16" 17529001 17530000 "*" 5.44009282066327e-15 2.08486137681812e-14 -100
"chr16" 17547001 17548000 "*" 5.83049822866499e-08 8.76853029340816e-08
66.6666666666667
"chr16" 17554001 17555000 "*" 2.16382467499443e-13 6.82775854166559e-13 -100
"chr16" 17637001 17638000 "*" 2.6569935140941e-09 4.83462661545569e-09
66.6666666666667
"chr16" 17655001 17656000 "*" 7.43849426498855e-15 2.80357739563059e-14

-71.1538461538462
"chr16" 18802001 18803000 "*" 0 0 78.6885245901639
"chr16" 18813001 18814000 "*" 0 0 -63.433570256971
"chr16" 19018001 19019000 "*" 3.6700841921089e-08 5.65530226072258e-08 -100
"chr16" 19097001 19098000 "*" 5.06261699229071e-14 1.7297955084095e-13 -68
"chr16" 19147001 19148000 "*" 1.78392687033568e-06 2.18669798687467e-06
58.8235294117647
"chr16" 19215001 19216000 "*" 0 0 71.5201465201465
"chr16" 19249001 19250000 "*" 0.000199851252180694 0.000178453492150885
-54.5454545454545
"chr16" 19381001 19382000 "*" 3.31690230837012e-12 9.03347040781321e-12 100
"chr16" 19421001 19422000 "*" 3.33066907387547e-16 1.4495649018245e-15 -100
"chr16" 19746001 19747000 "*" 1.83186799063151e-14 6.59696304569048e-14 -100
"chr16" 19795001 19796000 "*" 1.52794177310511e-09 2.87642564232045e-09 100
"chr16" 19893001 19894000 "*" 0.000198360190729274 0.000177211240630969
-63.6363636363636
"chr16" 19904001 19905000 "*" 2.73625566649116e-12 7.52483363579137e-12 -100
"chr16" 19914001 19915000 "*" 2.22044604925031e-16 9.81641919380259e-16 100
"chr16" 19928001 19929000 "*" 0 0 100
"chr16" 20013001 20014000 "*" 1.66533453693773e-14 6.02507639546739e-14
83.4437086092715
"chr16" 20059001 20060000 "*" 4.08209022140227e-11 9.57770690255255e-11 100
"chr16" 20070001 20071000 "*" 7.20239060689742e-05 6.90632392773323e-05
58.8235294117647
"chr16" 20352001 20353000 "*" 0 0 -62.3523093447905
"chr16" 20621001 20622000 "*" 2.22044604925031e-16 9.81641919380259e-16 100
"chr16" 20636001 20637000 "*" 0 0 51.7006802721088
"chr16" 20683001 20684000 "*" 4.65637306490407e-10 9.46720524046618e-10 -100
"chr16" 20786001 20787000 "*" 2.91028312560115e-11 6.97590507139978e-11 100
"chr16" 20974001 20975000 "*" 7.43849426498855e-15 2.80357739563059e-14 -100
"chr16" 20975001 20976000 "*" 1.64457336637724e-12 4.65338642828372e-12
-69.2307692307692
"chr16" 20996001 20997000 "*" 1.92989607405991e-07 2.69461390302816e-07
-54.4117647058824
"chr16" 21069001 21070000 "*" 2.73625566649116e-12 7.52483363579137e-12 -100
"chr16" 21156001 21157000 "*" 0 0 100
"chr16" 21295001 21296000 "*" 0 0 51.0026143254082
"chr16" 21523001 21524000 "*" 0 0 -62.3289106145251
"chr16" 21635001 21636000 "*" 2.91028312560115e-11 6.97590507139978e-11 -100
"chr16" 21959001 21960000 "*" 4.12196810017917e-09 7.30038251097473e-09
92.5925925925926
"chr16" 22020001 22021000 "*" 0 0 100
"chr16" 22232001 22233000 "*" 0 0 -100
"chr16" 22250001 22251000 "*" 5.83442053749827e-05 5.67762044471538e-05
-61.3636363636364
"chr16" 22683001 22684000 "*" 0 0 55.3191489361702
"chr16" 22710001 22711000 "*" 2.4535928844216e-14 8.69144396197119e-14 100
"chr16" 22799001 22800000 "*" 7.0006535457523e-08 1.03695832512054e-07 100
"chr16" 23059001 23060000 "*" 8.43347613965761e-12 2.17859001990012e-11 100
"chr16" 23083001 23084000 "*" 0 0 -91.8604651162791
"chr16" 23170001 23171000 "*" 9.65729496371637e-10 1.87255912493232e-09 100
"chr16" 23186001 23187000 "*" 4.99020824662466e-11 1.15790792586157e-10 100

"chr16" 23194001 23195000 "*" 0 0 66.145447644812
"chr16" 23221001 23222000 "*" 0 0 100
"chr16" 23291001 23292000 "*" 8.57092175010621e-13 2.51149198099779e-12 100
"chr16" 23328001 23329000 "*" 3.10862446895044e-15 1.22466437093774e-14 100
"chr16" 23360001 23361000 "*" 3.7295500021628e-11 8.80106019930144e-11
-86.8131868131868
"chr16" 23385001 23386000 "*" 8.98170426921752e-14 2.97124428237056e-13
62.8571428571429
"chr16" 23386001 23387000 "*" 5.80965558993896e-08 8.73986409124991e-08
70.3703703703704
"chr16" 23398001 23399000 "*" 3.34128893442198e-08 5.19772864618523e-08 100
"chr16" 23715001 23716000 "*" 4.96478846385706e-05 4.8856288723899e-05 -60
"chr16" 23821001 23822000 "*" 5.03264097062583e-13 1.51942428521737e-12 100
"chr16" 23846001 23847000 "*" 0 0 100
"chr16" 23849001 23850000 "*" 1.47024104002469e-09 2.78363533963333e-09
-76.5957446808511
"chr16" 23924001 23925000 "*" 6.24654217240561e-10 1.2462108191303e-09 100
"chr16" 24057001 24058000 "*" 1.26192389870994e-11 3.18327181281005e-11
74.5098039215686
"chr16" 24083001 24084000 "*" 2.22044604925031e-16 9.81641919380259e-16 -100
"chr16" 24363001 24364000 "*" 4.0595467210025e-08 6.22586980586337e-08
-83.3333333333333
"chr16" 24506001 24507000 "*" 3.79924262539078e-06 4.4315391800754e-06 -62.5
"chr16" 24840001 24841000 "*" 5.05277286677597e-09 8.83911439367989e-09 52
"chr16" 24856001 24857000 "*" 2.64951482975562e-09 4.82186011375752e-09 -100
"chr16" 24887001 24888000 "*" 2.84224053181958e-08 4.46799188203104e-08
-62.8571428571429
"chr16" 24921001 24922000 "*" 0 0 100
"chr16" 24928001 24929000 "*" 1.92068583260152e-14 6.89775382589628e-14 -100
"chr16" 25046001 25047000 "*" 4.01313771103418e-09 7.11882087237587e-09 -100
"chr16" 25072001 25073000 "*" 1.12132525487141e-14 4.14352271980498e-14 -100
"chr16" 25080001 25081000 "*" 0 0 -98.4848484848485
"chr16" 25082001 25083000 "*" 1.56863411149288e-12 4.4469621057462e-12 -100
"chr16" 25115001 25116000 "*" 0 0 100
"chr16" 25118001 25119000 "*" 0 0 -67.6567656765677
"chr16" 25137001 25138000 "*" 0 0 -54.9088009568424
"chr16" 25217001 25218000 "*" 6.4152538836737e-10 1.27477697728417e-09 -100
"chr16" 25234001 25235000 "*" 7.0006535457523e-08 1.03695832512054e-07 100
"chr16" 25705001 25706000 "*" 0 0 100
"chr16" 25906001 25907000 "*" 1.5277158427196e-09 2.87642564232045e-09 -100
"chr16" 25925001 25926000 "*" 1.49613654798486e-12 4.26244554429779e-12 100
"chr16" 26044001 26045000 "*" 0 0 -100
"chr16" 26060001 26061000 "*" 3.79304698761018e-10 7.80322551567583e-10
80.9523809523809
"chr16" 26147001 26148000 "*" 2.69007038866675e-13 8.38831022628993e-13 -100
"chr16" 26656001 26657000 "*" 2.64951482975562e-09 4.82186011375752e-09 -100
"chr16" 26828001 26829000 "*" 0 0 56.9230769230769
"chr16" 26974001 26975000 "*" 7.0006535457523e-08 1.03695832512054e-07 100
"chr16" 27074001 27075000 "*" 0 0 100
"chr16" 27075001 27076000 "*" 8.52140580320793e-12 2.19817259951489e-11 -100
"chr16" 27079001 27080000 "*" 1.4255263636187e-13 4.59957800504786e-13
61.0619469026549

"chr16" 27095001 27096000 "*" 1.67299207820548e-08 2.71377429441055e-08 100
"chr16" 27119001 27120000 "*" 0.000198360190729274 0.000177211240630969
-63.6363636363636
"chr16" 27210001 27211000 "*" 2.73014943985572e-11 6.58595916221891e-11 100
"chr16" 27242001 27243000 "*" 1.02606811935857e-12 2.97755032334345e-12
-66.1764705882353
"chr16" 27359001 27360000 "*" 4.01313771103418e-09 7.11882087237587e-09 -100
"chr16" 27448001 27449000 "*" 2.22044604925031e-16 9.81641919380259e-16 -100
"chr16" 27496001 27497000 "*" 1.35036426485158e-12 3.86629547363774e-12 100
"chr16" 27533001 27534000 "*" 2.43005615629954e-09 4.45014222280278e-09 -100
"chr16" 27646001 27647000 "*" 0 0 100
"chr16" 27734001 27735000 "*" 3.6700841921089e-08 5.65530226072258e-08 100
"chr16" 27787001 27788000 "*" 0 0 -100
"chr16" 27803001 27804000 "*" 1.39779633423487e-07 1.98463037958589e-07 100
"chr16" 27812001 27813000 "*" 1.14124265593318e-11 2.89138062669327e-11 100
"chr16" 27824001 27825000 "*" 1.89581683684992e-12 5.32311367606535e-12 100
"chr16" 27853001 27854000 "*" 0 0 95.8333333333333
"chr16" 27882001 27883000 "*" 3.68371999570627e-13 1.13149976091305e-12 -100
"chr16" 27936001 27937000 "*" 5.70958169632263e-10 1.14621387656155e-09
74.4186046511628
"chr16" 27962001 27963000 "*" 0.000235599168805112 0.000207894354164181
55.5555555555556
"chr16" 27974001 27975000 "*" 0.000277688249054053 0.00024207626296996
-51.219512195122
"chr16" 27988001 27989000 "*" 1.17340581695657e-11 2.96941053380085e-11
60.7594936708861
"chr16" 27989001 27990000 "*" 3.58120389308825e-05 3.60126966183746e-05
61.5384615384615
"chr16" 27992001 27993000 "*" 0 0 100
"chr16" 28021001 28022000 "*" 8.65973959207622e-14 2.87362558212404e-13 100
"chr16" 28063001 28064000 "*" 1.13140401492018e-08 1.87801380787728e-08 100
"chr16" 28073001 28074000 "*" 0 0 71.6666666666667
"chr16" 28076001 28077000 "*" 4.32209823486573e-12 1.15935290479641e-11 100
"chr16" 28263001 28264000 "*" 8.3882012447134e-11 1.88922989503423e-10 100
"chr16" 28267001 28268000 "*" 2.30274654122731e-07 3.18396940354316e-07 63
"chr16" 28293001 28294000 "*" 1.49952494865602e-10 3.25875982597286e-10
81.4159292035398
"chr16" 28302001 28303000 "*" 3.66002350649097e-09 6.55475770076275e-09
52.1739130434783
"chr16" 28312001 28313000 "*" 1.92451518943315e-07 2.68768579727545e-07
69.2307692307692
"chr16" 28503001 28504000 "*" 0 0 -100
"chr16" 28545001 28546000 "*" 1.48087875295744e-10 3.22075573380618e-10 100
"chr16" 28550001 28551000 "*" 4.46509496043745e-12 1.19571035106719e-11
56.3942307692308
"chr16" 28603001 28604000 "*" 1.04861119787358e-10 2.33275286083984e-10
-51.8518518518519
"chr16" 28617001 28618000 "*" 2.92612156727046e-10 6.11125079829759e-10
-79.3650793650794
"chr16" 28634001 28635000 "*" 0 0 71.4575515511796
"chr16" 28635001 28636000 "*" 8.7349629751543e-09 1.47505871898511e-08 100
"chr16" 28862001 28863000 "*" 2.88657986402541e-15 1.14155613124573e-14 -100

"chr16" 28963001 28964000 "*" 6.66133814775094e-16 2.81595744474255e-15 -100
"chr16" 29004001 29005000 "*" 0 0 70
"chr16" 29009001 29010000 "*" 1.90958360235527e-13 6.07725724484217e-13 72
"chr16" 29032001 29033000 "*" 0.000183332150308524 0.000164701876483498 56.4102564102564
"chr16" 29035001 29036000 "*" 2.5942625825337e-10 5.47131894059214e-10 86.5853658536585
"chr16" 29118001 29119000 "*" 0 0 -71.0884353741497
"chr16" 29180001 29181000 "*" 3.56775498033812e-10 7.35898629809829e-10 100
"chr16" 29192001 29193000 "*" 0 0 64.4368858654573
"chr16" 29200001 29201000 "*" 1.72285234512071e-06 2.11650026306093e-06 71.875
"chr16" 29214001 29215000 "*" 3.73490127714149e-12 1.01173629214827e-11 54.7619047619048
"chr16" 29278001 29279000 "*" 6.59550958292954e-09 1.13456194382773e-08 100
"chr16" 29315001 29316000 "*" 0.000114256469000651 0.00010620542277845 51.5151515151515
"chr16" 29338001 29339000 "*" 6.24005042082487e-08 9.34435715465956e-08 -52.3809523809524
"chr16" 29346001 29347000 "*" 0 0 -78.5046728971963
"chr16" 29787001 29788000 "*" 1.12376774552558e-12 3.24761818527664e-12 100
"chr16" 29801001 29802000 "*" 0 0 66.6666666666667
"chr16" 29819001 29820000 "*" 0 0 -53.7820512820513
"chr16" 29831001 29832000 "*" 0 0 -92.156862745098
"chr16" 29836001 29837000 "*" 2.68450373042128e-10 5.64465213127486e-10 -100
"chr16" 29873001 29874000 "*" 0 0 -100
"chr16" 29903001 29904000 "*" 4.02167188440217e-12 1.08266700497926e-11 -100
"chr16" 29909001 29910000 "*" 0 0 100
"chr16" 29938001 29939000 "*" 0 0 79.4326241134752
"chr16" 30006001 30007000 "*" 0 0 60.5932203389831
"chr16" 30042001 30043000 "*" 0 0 68.7306501547988
"chr16" 30048001 30049000 "*" 1.06192832305396e-12 3.07839980099276e-12 75
"chr16" 30065001 30066000 "*" 3.65130148338721e-12 9.90602415539447e-12 100
"chr16" 30085001 30086000 "*" 6.66133814775094e-15 2.52791665956799e-14 -89.4736842105263
"chr16" 30392001 30393000 "*" 5.62883073484954e-13 1.68905058935478e-12 100
"chr16" 30429001 30430000 "*" 0 0 -76.4683779150325
"chr16" 30437001 30438000 "*" 3.77475828372553e-15 1.47379557022071e-14 -100
"chr16" 30528001 30529000 "*" 0 0 63.4408602150538
"chr16" 30537001 30538000 "*" 0 0 -93.4210526315789
"chr16" 30624001 30625000 "*" 3.03979064142368e-13 9.42350883309283e-13 -100
"chr16" 30645001 30646000 "*" 0 0 77.6595744680851
"chr16" 30663001 30664000 "*" 1.77036163506727e-12 4.99287820414658e-12 -91.5254237288136
"chr16" 30681001 30682000 "*" 0.000447727107318552 0.000376920426399982 -60
"chr16" 30774001 30775000 "*" 4.03051019459699e-06 4.68321829238973e-06 -58.3333333333333
"chr16" 30786001 30787000 "*" 1.16408228700848e-06 1.46479698533027e-06 -66.6666666666667
"chr16" 30918001 30919000 "*" 2.41602046069378e-06 2.90480762826846e-06 63.4146341463415
"chr16" 30924001 30925000 "*" 3.01644265121581e-11 7.21999252326135e-11 92.6829268292683

"chr16" 30927001 30928000 "*" 9.62896429257398e-13 2.80291205576417e-12 100
"chr16" 30960001 30961000 "*" 0 0 -67.0570955575748
"chr16" 30965001 30966000 "*" 0 0 100
"chr16" 31023001 31024000 "*" 9.30051856606884e-06 1.02447332143295e-05 53.3333333333333
"chr16" 31059001 31060000 "*" 3.77475828372553e-15 1.47379557022071e-14 100
"chr16" 31060001 31061000 "*" 1.59301718808402e-05 1.69294262236803e-05 66.6666666666667
"chr16" 31106001 31107000 "*" 0 0 -61.038961038961
"chr16" 31146001 31147000 "*" 0 0 -98.7654320987654
"chr16" 31150001 31151000 "*" 0 0 -71.1409395973154
"chr16" 31162001 31163000 "*" 8.94783367988428e-06 9.87593937733781e-06 -70
"chr16" 31211001 31212000 "*" 2.3990809339125e-12 6.65441738642456e-12 -100
"chr16" 31277001 31278000 "*" 1.49972523288966e-09 2.83643172292987e-09 73.6842105263158
"chr16" 31367001 31368000 "*" 3.45977298437106e-05 3.48617813738808e-05 -62.5
"chr16" 31384001 31385000 "*" 5.15398834721736e-12 1.36858832250262e-11 63.0434782608696
"chr16" 31404001 31405000 "*" 9.99200722162641e-16 4.15382497462808e-15 100
"chr16" 31454001 31455000 "*" 0 0 67.3227749207361
"chr16" 31469001 31470000 "*" 0 0 91.8918918918919
"chr16" 31483001 31484000 "*" 5.02251573664125e-11 1.16504787499903e-10 -90.4761904761905
"chr16" 31490001 31491000 "*" 5.6362137179633e-11 1.29963270300065e-10 -100
"chr16" 31568001 31569000 "*" 9.63829016598083e-12 2.46993429847588e-11 -100
"chr16" 31579001 31580000 "*" 7.7715611723761e-16 3.26213507634405e-15 -62.962962962963
"chr16" 31849001 31850000 "*" 8.11321854143898e-09 1.37928190725433e-08 -68.75
"chr16" 31990001 31991000 "*" 4.81571154065108e-07 6.37949233437604e-07 84.6153846153846
"chr16" 32297001 32298000 "*" 0 0 64.4550342130987
"chr16" 32484001 32485000 "*" 3.90831811358794e-12 1.05610567215048e-11 100
"chr16" 32486001 32487000 "*" 1.0991207943789e-14 4.06651762510369e-14 55.8139534883721
"chr16" 32488001 32489000 "*" 6.98726632109015e-10 1.38244699331437e-09 86.6666666666667
"chr16" 32628001 32629000 "*" 1.67299207820548e-08 2.71377429441055e-08 -100
"chr16" 32878001 32879000 "*" 1.05426778418405e-11 2.69012293081375e-11 -65.6716417910448
"chr16" 33038001 33039000 "*" 3.30369065437708e-12 9.00336905360004e-12 -80
"chr16" 33298001 33299000 "*" 1.16129328375791e-12 3.35130343431273e-12 69.8529411764706
"chr16" 33351001 33352000 "*" 1.11022302462516e-16 5.03662826618488e-16 -71.9785138764548
"chr16" 33380001 33381000 "*" 2.27583171619195e-06 2.74694923536694e-06 -50.609756097561
"chr16" 33408001 33409000 "*" 5.21804821573824e-14 1.7777322631293e-13 -53.921568627451
"chr16" 33412001 33413000 "*" 8.01666433236647e-09 1.36392368970109e-08 -100
"chr16" 33416001 33417000 "*" 2.95345469225605e-06 3.50503984009585e-06 56.2901744719926
"chr16" 33572001 33573000 "*" 0 0 65.1086956521739

"chr16" 33761001 33762000 "*" 1.26565424807268e-14 4.63923203146481e-14 100
"chr16" 33797001 33798000 "*" 7.54951656745106e-14 2.5237892556906e-13 100
"chr16" 33818001 33819000 "*" 0.000231175651597026 0.000204278487663226 54.5454545454545
"chr16" 33862001 33863000 "*" 6.88338275267597e-15 2.60518659957509e-14 -100
"chr16" 33886001 33887000 "*" 0 0 100
"chr16" 33952001 33953000 "*" 4.99900121297969e-12 1.32921056339624e-11 -100
"chr16" 34182001 34183000 "*" 3.73387165630845e-09 6.68213416585887e-09 -74.468085106383
"chr16" 34183001 34184000 "*" 4.44089209850063e-15 1.71914916534614e-14 -61.7283950617284
"chr16" 34194001 34195000 "*" 6.66133814775094e-16 2.81595744474255e-15 93.75
"chr16" 34265001 34266000 "*" 0 0 -100
"chr16" 34294001 34295000 "*" 3.98384134303598e-06 4.63286451783947e-06 57.4074074074074
"chr16" 34642001 34643000 "*" 3.6700841921089e-08 5.65530226072258e-08 100
"chr16" 34968001 34969000 "*" 1.69482179817049e-05 1.79420595392413e-05 66.6666666666667
"chr16" 34969001 34970000 "*" 4.44089209850063e-15 1.71914916534614e-14 -100
"chr16" 46396001 46397000 "*" 1.75967979332281e-06 2.15875356125722e-06 -52.1739130434783
"chr16" 46410001 46411000 "*" 0 0 81.5086206896552
"chr16" 46411001 46412000 "*" 1.46854417515385e-10 3.19885101699278e-10 -55.3719008264463
"chr16" 46424001 46425000 "*" 7.68773577464188e-07 9.91437385905438e-07 -52
"chr16" 46425001 46426000 "*" 0 0 87.9518072289157
"chr16" 46431001 46432000 "*" 8.74470433387398e-06 9.6651705086143e-06 -63.5135135135135
"chr16" 46435001 46436000 "*" 4.21884749357559e-15 1.63874445239189e-14 62.3251748251748
"chr16" 46448001 46449000 "*" 3.34081444730572e-08 5.19772864618523e-08 -81.3008130081301
"chr16" 46449001 46450000 "*" 3.30402372128447e-13 1.02073596517831e-12 63.7476459510358
"chr16" 46451001 46452000 "*" 9.65338919911574e-13 2.80678910656113e-12 100
"chr16" 46766001 46767000 "*" 0 0 -59.6311475409836
"chr16" 46790001 46791000 "*" 1.24847021609753e-10 2.75043680609774e-10 -54.9019607843137
"chr16" 46955001 46956000 "*" 3.84137166520304e-14 1.32929369817675e-13 100
"chr16" 47102001 47103000 "*" 3.58862350946509e-09 6.43614687220395e-09 -56.25
"chr16" 47176001 47177000 "*" 0 0 66.8587896253602
"chr16" 47177001 47178000 "*" 0 0 79.5722713864307
"chr16" 47178001 47179000 "*" 0 0 76.4705882352941
"chr16" 47599001 47600000 "*" 0 0 100
"chr16" 47744001 47745000 "*" 5.1281201507436e-13 1.54461625452838e-12 -100
"chr16" 47980001 47981000 "*" 9.80763359414993e-10 1.8980032424715e-09 100
"chr16" 48089001 48090000 "*" 7.57172102794357e-14 2.5308063922355e-13 -62.5
"chr16" 48090001 48091000 "*" 2.62900812231237e-13 8.21294938531841e-13 100
"chr16" 48143001 48144000 "*" 0 0 -100
"chr16" 48151001 48152000 "*" 9.34530230978226e-12 2.40075381496491e-11 70.7317073170732
"chr16" 48152001 48153000 "*" 4.6029846600959e-13 1.3952103535992e-12

94.2307692307692
"chr16" 48168001 48169000 "*" 9.61897228535236e-13 2.801372325071e-12 100
"chr16" 48178001 48179000 "*" 8.55919767679403e-05 8.10918052683734e-05 53.125
"chr16" 48190001 48191000 "*" 0 0 100
"chr16" 48248001 48249000 "*" 4.01313771103418e-09 7.11882087237587e-09 -100
"chr16" 48292001 48293000 "*" 3.10862446895044e-15 1.22466437093774e-14 -100
"chr16" 48368001 48369000 "*" 4.88498130835069e-15 1.8824139250584e-14 100
"chr16" 48384001 48385000 "*" 3.6700841921089e-08 5.65530226072258e-08 100
"chr16" 48535001 48536000 "*" 4.02994859705075e-09 7.14727318970939e-09
-64.9350649350649
"chr16" 48887001 48888000 "*" 0 0 69.7674418604651
"chr16" 48896001 48897000 "*" 2.02327044007689e-12 5.65300384792621e-12 100
"chr16" 48941001 48942000 "*" 0 0 100
"chr16" 49000001 49001000 "*" 8.25450818808804e-13 2.42473843346228e-12 100
"chr16" 49057001 49058000 "*" 8.01666433236647e-09 1.36392368970109e-08 100
"chr16" 49315001 49316000 "*" 0 0 66.274049834411
"chr16" 49316001 49317000 "*" 0 0 57.2025815429702
"chr16" 49358001 49359000 "*" 2.02327044007689e-12 5.65300384792621e-12 100
"chr16" 49392001 49393000 "*" 4.71134242729931e-12 1.257615036806e-11 100
"chr16" 49496001 49497000 "*" 2.38031816479634e-13 7.47299807843568e-13 100
"chr16" 49527001 49528000 "*" 1.51323398256409e-13 4.86995056237989e-13 100
"chr16" 49541001 49542000 "*" 1.29037891483108e-11 3.24582280286866e-11 100
"chr16" 49545001 49546000 "*" 4.79616346638068e-14 1.64380445416596e-13 -100
"chr16" 49591001 49592000 "*" 0 0 -97.6190476190476
"chr16" 49594001 49595000 "*" 2.22044604925031e-16 9.81641919380259e-16 100
"chr16" 49598001 49599000 "*" 1.44251914191429e-07 2.04485674657138e-07
54.5454545454545
"chr16" 49609001 49610000 "*" 5.63240443174351e-08 8.48653084301593e-08
-64.9484536082474
"chr16" 49654001 49655000 "*" 9.8862888640916e-05 9.27525037076316e-05
54.3859649122807
"chr16" 49673001 49674000 "*" 3.31909070383496e-06 3.90760147196615e-06
69.5652173913043
"chr16" 49704001 49705000 "*" 9.63829016598083e-12 2.46993429847588e-11 100
"chr16" 49712001 49713000 "*" 4.99900121297969e-12 1.32921056339624e-11 -100
"chr16" 49716001 49717000 "*" 1.83952852950142e-12 5.17499701329218e-12 75
"chr16" 49721001 49722000 "*" 5.04263297784746e-13 1.52078941532818e-12 100
"chr16" 49740001 49741000 "*" 6.4152538836737e-10 1.27477697728417e-09 100
"chr16" 49741001 49742000 "*" 2.23154827949656e-14 7.95931582053377e-14
-80.1801801801802
"chr16" 49761001 49762000 "*" 0 0 91.1764705882353
"chr16" 49804001 49805000 "*" 1.9125552408461e-07 2.67187792223674e-07
52.5373134328358
"chr16" 49806001 49807000 "*" 1.86821217281752e-05 1.96397758022194e-05 60
"chr16" 49814001 49815000 "*" 0 0 67.0967741935484
"chr16" 49914001 49915000 "*" 2.38575473383573e-05 2.46816531377115e-05
66.6666666666667
"chr16" 49917001 49918000 "*" 4.02167188440217e-12 1.08266700497926e-11 100
"chr16" 49920001 49921000 "*" 0 0 -100
"chr16" 49945001 49946000 "*" 1.12634901405784e-10 2.49563754426169e-10 100
"chr16" 49952001 49953000 "*" 0 0 -100
"chr16" 49968001 49969000 "*" 0 0 100

"chr16" 49993001 49994000 "*" 4.10812271045558e-06 4.76629426978236e-06
55.1724137931034
"chr16" 50001001 50002000 "*" 2.64666066840391e-12 7.30832514608382e-12
-76.9230769230769
"chr16" 50007001 50008000 "*" 7.07661325094477e-08 1.04737273899807e-07
65.5172413793103
"chr16" 50012001 50013000 "*" 3.6700841921089e-08 5.65530226072258e-08 -100
"chr16" 50033001 50034000 "*" 8.19565414095225e-05 7.7896169099937e-05
58.3333333333333
"chr16" 50060001 50061000 "*" 0 0 -97.7272727272727
"chr16" 50255001 50256000 "*" 1.23694513355366e-05 1.3360368613825e-05
-69.4444444444444
"chr16" 50279001 50280000 "*" 0 0 -100
"chr16" 50296001 50297000 "*" 1.16573417585641e-14 4.29335582900508e-14 -100
"chr16" 50436001 50437000 "*" 1.33794086920602e-10 2.93459922669956e-10
59.2592592592593
"chr16" 50472001 50473000 "*" 4.44089209850063e-15 1.71914916534614e-14 -100
"chr16" 50485001 50486000 "*" 1.07882591748876e-11 2.74553537455803e-11 100
"chr16" 50499001 50500000 "*" 4.38219849374644e-06 5.06174739125268e-06
64.1025641025641
"chr16" 50512001 50513000 "*" 0 0 -81.25
"chr16" 50516001 50517000 "*" 5.01025332333427e-10 1.01358031135889e-09 100
"chr16" 50537001 50538000 "*" 4.35984581770299e-13 1.32372558111634e-12 60
"chr16" 50644001 50645000 "*" 1.36900479841273e-09 2.60147085471260e-09
-50.9547190398254
"chr16" 50652001 50653000 "*" 0 0 100
"chr16" 50653001 50654000 "*" 7.99634872761246e-08 1.17484551589118e-07
-53.5714285714286
"chr16" 50666001 50667000 "*" 7.34753580200476e-09 1.25713325858446e-08
-68.8405797101449
"chr16" 50678001 50679000 "*" 1.10442103402608e-05 1.20217549185931e-05
-59.2592592592593
"chr16" 50743001 50744000 "*" 1.70641278884887e-12 4.81986032810646e-12 100
"chr16" 50911001 50912000 "*" 7.88258347483861e-15 2.96145301916012e-14 -100
"chr16" 50918001 50919000 "*" 0.000226767320649301 0.000200676463139324
51.0204081632653
"chr16" 50940001 50941000 "*" 1.11895673710061e-05 1.21690188495108e-05
-64.7619047619048
"chr16" 50951001 50952000 "*" 1.46624235863868e-07 2.07629778995107e-07
69.7674418604651
"chr16" 50963001 50964000 "*" 2.1094237467878e-15 8.47434540879246e-15 100
"chr16" 50969001 50970000 "*" 1.11022302462516e-16 5.03662826618488e-16
67.741935483871
"chr16" 51001001 51002000 "*" 8.93729534823251e-14 2.95749181076308e-13
-85.3658536585366
"chr16" 51009001 51010000 "*" 0 0 100
"chr16" 51010001 51011000 "*" 1.39779633423487e-07 1.98463037958589e-07 100
"chr16" 51126001 51127000 "*" 3.23373847044284e-06 3.81325174359463e-06
66.6666666666667
"chr16" 51271001 51272000 "*" 2.08814465718632e-09 3.86232567960463e-09 -100
"chr16" 51273001 51274000 "*" 2.77555756156289e-15 1.10015415195978e-14 -100
"chr16" 51393001 51394000 "*" 1.93768068257327e-09 3.60637771230225e-09

72.2222222222222
"chr16" 51429001 51430000 "*" 1.15463194561016e-14 4.25829614430727e-14 100
"chr16" 51561001 51562000 "*" 0 0 100
"chr16" 51685001 51686000 "*" 1.11022302462516e-16 5.03662826618488e-16
-80.6122448979592
"chr16" 51799001 51800000 "*" 1.67299207820548e-08 2.71377429441055e-08 -100
"chr16" 51910001 51911000 "*" 1.11022302462516e-16 5.03662826618488e-16 -100
"chr16" 52228001 52229000 "*" 3.95353005888666e-09 7.03928529283733e-09 -100
"chr16" 52473001 52474000 "*" 0 0 -100
"chr16" 52580001 52581000 "*" 0 0 71.3178294573643
"chr16" 52689001 52690000 "*" 2.22044604925031e-16 9.81641919380259e-16
-74.1565452091768
"chr16" 53392001 53393000 "*" 1.9373391779709e-13 6.15971322898373e-13 100
"chr16" 53393001 53394000 "*" 6.46149800331841e-14 2.17664365839349e-13 -100
"chr16" 53558001 53559000 "*" 5.0406012697124e-11 1.16891797086073e-10
-87.6106194690265
"chr16" 53563001 53564000 "*" 7.94730947717426e-12 2.05982705398163e-11
-91.6666666666667
"chr16" 53613001 53614000 "*" 1.48087875295744e-10 3.22075573380618e-10 100
"chr16" 54148001 54149000 "*" 1.4432899320127e-12 4.12202541809485e-12 -62.5
"chr16" 54149001 54150000 "*" 3.68371999570627e-13 1.13149976091305e-12 -100
"chr16" 54188001 54189000 "*" 7.03992419914812e-12 1.83313429961219e-11 -100
"chr16" 54189001 54190000 "*" 1.951953760293e-06 2.378434283772e-06
-54.2857142857143
"chr16" 54234001 54235000 "*" 8.7349629751543e-09 1.47505871898511e-08 -100
"chr16" 54365001 54366000 "*" 0 0 100
"chr16" 54376001 54377000 "*" 4.43011560358197e-05 4.39265220551362e-05
-63.8888888888889
"chr16" 54466001 54467000 "*" 2.80889212778135e-07 3.840942150991e-07
52.762568442011
"chr16" 54471001 54472000 "*" 0 0 -66
"chr16" 54518001 54519000 "*" 1.12458486967171e-09 2.15740815043925e-09 -100
"chr16" 54536001 54537000 "*" 4.01313771103418e-09 7.11882087237587e-09 -100
"chr16" 54538001 54539000 "*" 2.88779000712225e-11 6.92935809466068e-11 100
"chr16" 54545001 54546000 "*" 0 0 -82.5
"chr16" 54623001 54624000 "*" 2.81044296890354e-06 3.34565012917882e-06
84.6153846153846
"chr16" 54650001 54651000 "*" 0.00063975764960944 0.00052432375414322
52.9411764705882
"chr16" 54962001 54963000 "*" 0 0 62.0081411126187
"chr16" 54963001 54964000 "*" 0 0 74.6987951807229
"chr16" 54964001 54965000 "*" 0 0 64.7273262276859
"chr16" 54965001 54966000 "*" 0 0 63.5677947356779
"chr16" 55032001 55033000 "*" 0 0 -100
"chr16" 55078001 55079000 "*" 0 0 -100
"chr16" 55164001 55165000 "*" 1.72175296242472e-09 3.22628284973177e-09
-55.5555555555556
"chr16" 55210001 55211000 "*" 5.36903854708726e-13 1.61444172513789e-12
-64.1025641025641
"chr16" 55335001 55336000 "*" 3.34128893442198e-08 5.19772864618523e-08 100
"chr16" 55405001 55406000 "*" 0 0 54.9658434051498
"chr16" 55532001 55533000 "*" 4.21884749357559e-15 1.63874445239189e-14 -100

"chr16" 55677001 55678000 "*" 5.08650899178065e-11 1.17900668424206e-10 -84.375
"chr16" 55686001 55687000 "*" 1.22457599616155e-12 3.52441478267078e-12 100
"chr16" 55691001 55692000 "*" 0 0 100
"chr16" 55698001 55699000 "*" 1.13140401492018e-08 1.87801380787728e-08 100
"chr16" 55703001 55704000 "*" 0 0 100
"chr16" 55755001 55756000 "*" 1.95399252334028e-14 7.01253661173697e-14 100
"chr16" 55808001 55809000 "*" 1.39777078800307e-13 4.51491316511768e-13
-58.2417582417582
"chr16" 55815001 55816000 "*" 1.54321000422897e-14 5.6024372055589e-14 -70
"chr16" 55855001 55856000 "*" 5.48638023900594e-10 1.10374357336421e-09 100
"chr16" 55867001 55868000 "*" 0 0 100
"chr16" 55883001 55884000 "*" 0 0 100
"chr16" 55906001 55907000 "*" 6.2400505429494e-08 9.34435715465956e-08
-80.7692307692308
"chr16" 56077001 56078000 "*" 8.02691246803988e-14 2.67352274101148e-13 -100
"chr16" 56140001 56141000 "*" 2.88710388929303e-10 6.03399264731139e-10 100
"chr16" 56225001 56226000 "*" 0 0 93.6300417246175
"chr16" 56283001 56284000 "*" 3.84340814729711e-09 6.86961098612525e-09
85.2941176470588
"chr16" 56322001 56323000 "*" 0 0 100
"chr16" 56323001 56324000 "*" 3.46500605985511e-13 1.06777978272295e-12 -100
"chr16" 56333001 56334000 "*" 2.64951482975562e-09 4.82186011375752e-09 -100
"chr16" 56339001 56340000 "*" 3.14570343329379e-07 4.27293435984224e-07
-51.8518518518519
"chr16" 56340001 56341000 "*" 1.11022302462516e-16 5.03662826618488e-16
65.0793650793651
"chr16" 56350001 56351000 "*" 0.000432672693284286 0.000365157266029702
-53.3333333333333
"chr16" 56380001 56381000 "*" 1.11022302462516e-16 5.03662826618488e-16 100
"chr16" 56384001 56385000 "*" 1.02840624904843e-10 2.29012597294218e-10
67.4418604651163
"chr16" 56486001 56487000 "*" 2.30129248990352e-11 5.5982206740486e-11
66.6666666666667
"chr16" 56710001 56711000 "*" 1.22124532708767e-15 5.03921984217778e-15
-63.7731481481482
"chr16" 56743001 56744000 "*" 2.16382467499443e-13 6.82775854166559e-13 100
"chr16" 56761001 56762000 "*" 6.24654217240561e-10 1.2462108191303e-09 100
"chr16" 56778001 56779000 "*" 2.08814465718632e-09 3.86232567960463e-09 100
"chr16" 56889001 56890000 "*" 1.43443135147692e-09 2.71978753395078e-09
57.1428571428571
"chr16" 56900001 56901000 "*" 1.93720595120794e-11 4.76309924836636e-11 -100
"chr16" 56908001 56909000 "*" 3.36266410477792e-05 3.39604182576453e-05
63.265306122449
"chr16" 56915001 56916000 "*" 3.530509218308e-14 1.22891039008692e-13 100
"chr16" 56957001 56958000 "*" 7.0006535457523e-08 1.03695832512054e-07 -100
"chr16" 56965001 56966000 "*" 0 0 100
"chr16" 57005001 57006000 "*" 1.52466927971773e-12 4.33322475981408e-12 -100
"chr16" 57016001 57017000 "*" 7.53552707521976e-06 8.41108968411447e-06
-65.4135338345865
"chr16" 57032001 57033000 "*" 7.0006535457523e-08 1.03695832512054e-07 100
"chr16" 57081001 57082000 "*" 1.39779633423487e-07 1.98463037958589e-07 100
"chr16" 57091001 57092000 "*" 8.01666433236647e-09 1.36392368970109e-08 100

"chr16" 57112001 57113000 "*" 2.08814465718632e-09 3.86232567960463e-09 100
"chr16" 57238001 57239000 "*" 4.88498130835069e-15 1.8824139250584e-14 -52
"chr16" 57278001 57279000 "*" 8.15680856192103e-13 2.40052055158123e-12
54.5454545454545
"chr16" 57300001 57301000 "*" 2.22044604925031e-16 9.81641919380259e-16
70.2127659574468
"chr16" 57334001 57335000 "*" 0 0 100
"chr16" 57335001 57336000 "*" 2.27080243497824e-10 4.82488805048582e-10
-89.0173410404624
"chr16" 57398001 57399000 "*" 5.01025332333427e-10 1.01358031135889e-09 100
"chr16" 57415001 57416000 "*" 0 0 -100
"chr16" 57435001 57436000 "*" 0 0 100
"chr16" 57447001 57448000 "*" 6.57252030578093e-14 2.21243451061355e-13 -75
"chr16" 57512001 57513000 "*" 0 0 -100
"chr16" 57541001 57542000 "*" 1.06707975788822e-11 2.72139916611012e-11
68.6274509803922
"chr16" 57553001 57554000 "*" 4.08209022140227e-11 9.57770690255255e-11 100
"chr16" 57573001 57574000 "*" 2.22044604925031e-16 9.81641919380259e-16 100
"chr16" 57599001 57600000 "*" 1.03687058938817e-11 2.64813760686657e-11
77.5862068965517
"chr16" 57650001 57651000 "*" 9.80763359414993e-10 1.8980032424715e-09 100
"chr16" 57657001 57658000 "*" 1.35003119794419e-13 4.3679847476192e-13 -100
"chr16" 57674001 57675000 "*" 1.06363524698949e-06 1.34530286878699e-06
-71.7948717948718
"chr16" 57682001 57683000 "*" 1.39779633423487e-07 1.98463037958589e-07 -100
"chr16" 57686001 57687000 "*" 0 0 -58.3333333333333
"chr16" 57707001 57708000 "*" 8.52140580320793e-12 2.19817259951489e-11 100
"chr16" 57724001 57725000 "*" 0.000173919515143783 0.000156793955027816 -60
"chr16" 57727001 57728000 "*" 4.08209022140227e-11 9.57770690255255e-11 100
"chr16" 57820001 57821000 "*" 0 0 -54.9510585021625
"chr16" 57926001 57927000 "*" 3.60355445483407e-09 6.46028594313868e-09
-87.3417721518987
"chr16" 57997001 57998000 "*" 5.88418203051333e-15 2.245355428447e-14
-72.4137931034483
"chr16" 58028001 58029000 "*" 7.54067528419e-08 1.11163710242092e-07
57.8947368421053
"chr16" 58057001 58058000 "*" 0 0 -100
"chr16" 58061001 58062000 "*" 4.82947015711943e-14 1.65487972841165e-13
-53.9001200036924
"chr16" 58067001 58068000 "*" 0 0 -100
"chr16" 58130001 58131000 "*" 1.11022302462516e-16 5.03662826618488e-16 100
"chr16" 58131001 58132000 "*" 0 0 -100
"chr16" 58327001 58328000 "*" 0 0 87.3684210526316
"chr16" 58329001 58330000 "*" 0 0 52.6881720430108
"chr16" 58355001 58356000 "*" 0 0 100
"chr16" 58464001 58465000 "*" 1.76943237839566e-10 3.82013703336647e-10
52.8368794326241
"chr16" 58483001 58484000 "*" 2.15125472990962e-10 4.58126659943874e-10 -100
"chr16" 58505001 58506000 "*" 1.1640823099901e-06 1.46479698533027e-06
66.6666666666667
"chr16" 58522001 58523000 "*" 2.06294411553287e-08 3.29580631467495e-08
-75.609756097561

"chr16" 58528001 58529000 "*" 2.94986257642904e-13 9.15868493928254e-13 61.9047619047619
"chr16" 58548001 58549000 "*" 5.46136436074818e-09 9.51795202365188e-09 -66.6666666666667
"chr16" 58699001 58700000 "*" 0 0 -100
"chr16" 58710001 58711000 "*" 1.30638158069019e-08 2.15343710008517e-08 53.8461538461538
"chr16" 58788001 58789000 "*" 1.6219141030227e-08 2.64300079144354e-08 66.6666666666667
"chr16" 58880001 58881000 "*" 2.8199664825479e-14 9.92183471463377e-14 51.1494252873563
"chr16" 59020001 59021000 "*" 0.000499129401911658 0.000416865202314435 52.3809523809524
"chr16" 59122001 59123000 "*" 0 0 -100
"chr16" 60317001 60318000 "*" 3.46500605985511e-13 1.06777978272295e-12 -100
"chr16" 60393001 60394000 "*" 1.11022302462516e-16 5.03662826618488e-16 -96.9849246231156
"chr16" 60901001 60902000 "*" 0 0 57.6086956521739
"chr16" 61500001 61501000 "*" 6.59550958292954e-09 1.13456194382773e-08 -100
"chr16" 61590001 61591000 "*" 0 0 -81.5384615384615
"chr16" 61835001 61836000 "*" 1.41274921680035e-05 1.51258433455559e-05 53.3333333333333
"chr16" 62132001 62133000 "*" 1.55431223447522e-15 6.33705141101682e-15 100
"chr16" 63153001 63154000 "*" 1.25708423448501e-07 1.79960077649726e-07 56.7567567567568
"chr16" 63325001 63326000 "*" 8.72351752190248e-06 9.64290370122903e-06 -66.6666666666667
"chr16" 63389001 63390000 "*" 7.00065354575233e-08 1.03695832512054e-07 -100
"chr16" 64734001 64735000 "*" 1.14124265593318e-11 2.89138062669327e-11 -100
"chr16" 64805001 64806000 "*" 4.44089209850063e-16 1.91071758245033e-15 -81.7567567567568
"chr16" 65032001 65033000 "*" 1.11022302462516e-16 5.03662826618488e-16 -66.6666666666667
"chr16" 65105001 65106000 "*" 0 0 -70
"chr16" 65486001 65487000 "*" 1.68753899743024e-14 6.09826805967439e-14 100
"chr16" 65506001 65507000 "*" 0 0 -100
"chr16" 65788001 65789000 "*" 1.28785870856518e-14 4.71440608755782e-14 100
"chr16" 65789001 65790000 "*" 0 0 100
"chr16" 66265001 66266000 "*" 0 0 92.8571428571429
"chr16" 66266001 66267000 "*" 9.21510640083945e-06 1.01557468217126e-05 62.5
"chr16" 66303001 66304000 "*" 3.6700841921089e-08 5.65530226072258e-08 100
"chr16" 66347001 66348000 "*" 0 0 100
"chr16" 66382001 66383000 "*" 9.21768217310159e-11 2.06312360496437e-10 -55.5555555555556
"chr16" 66384001 66385000 "*" 2.16382467499443e-13 6.82775854166559e-13 100
"chr16" 66435001 66436000 "*" 3.38366762386855e-08 5.25796497446358e-08 65.7894736842105
"chr16" 66452001 66453000 "*" 2.08814465718632e-09 3.86232567960463e-09 100
"chr16" 66480001 66481000 "*" 5.68635138975537e-11 1.31046911801985e-10 61.2903225806452
"chr16" 66504001 66505000 "*" 3.04324343503026e-11 7.26599935673121e-11 100
"chr16" 66550001 66551000 "*" 1.44576572935762e-11 3.61628736116508e-11 -83.75

"chr16" 66563001 66564000 "*" 0 0 100
"chr16" 66584001 66585000 "*" 0 0 100
"chr16" 66619001 66620000 "*" 2.15125472990962e-10 4.58126659943874e-10 100
"chr16" 66906001 66907000 "*" 2.02327044007689e-12 5.65300384792621e-12 -100
"chr16" 66909001 66910000 "*" 0 0 100
"chr16" 66914001 66915000 "*" 0 0 98.8636363636364
"chr16" 66966001 66967000 "*" 9.76427261267787e-09 1.63933049617869e-08
53.1914893617021
"chr16" 66995001 66996000 "*" 0 0 100
"chr16" 66996001 66997000 "*" 2.88657986402541e-15 1.14155613124573e-14 100
"chr16" 67044001 67045000 "*" 6.59550958292954e-09 1.13456194382773e-08 -100
"chr16" 67175001 67176000 "*" 6.15096862333075e-11 1.41204258250625e-10
-78.3333333333333
"chr16" 67212001 67213000 "*" 0 0 -71.8013513713606
"chr16" 67245001 67246000 "*" 2.80631073934501e-12 7.70351759511944e-12
-51.3513513513514
"chr16" 67251001 67252000 "*" 9.0116802908824e-11 2.02402288295498e-10
51.219512195122
"chr16" 67274001 67275000 "*" 3.77475828372553e-15 1.47379557022071e-14 -100
"chr16" 67292001 67293000 "*" 2.11708428565771e-12 5.90503009823905e-12
51.2329656067489
"chr16" 67335001 67336000 "*" 5.39619190149665e-05 5.27870047241069e-05
-59.2592592592593
"chr16" 67450001 67451000 "*" 0 0 -90.4029692470838
"chr16" 67552001 67553000 "*" 3.61290469097497e-07 4.86547393947888e-07
-54.5454545454545
"chr16" 67563001 67564000 "*" 1.11022302462516e-16 5.03662826618488e-16 100
"chr16" 67686001 67687000 "*" 0 0 67.1358390517779
"chr16" 67687001 67688000 "*" 0 0 67.0558695729873
"chr16" 67699001 67700000 "*" 2.98824465427838e-06 3.54254647244229e-06
66.6666666666667
"chr16" 67777001 67778000 "*" 4.27954982562539e-09 7.56742156628225e-09
-90.9090909090909
"chr16" 67826001 67827000 "*" 0 0 100
"chr16" 67850001 67851000 "*" 0 0 69.2307692307692
"chr16" 67871001 67872000 "*" 5.65418315445765e-06 6.4320100371832e-06
54.5454545454545
"chr16" 67875001 67876000 "*" 0 0 64.2857142857143
"chr16" 67880001 67881000 "*" 0 0 50.7349270619557
"chr16" 67917001 67918000 "*" 2.03170813506404e-14 7.28047926885552e-14 -53.75
"chr16" 67988001 67989000 "*" 9.04570973681018e-10 1.76414389822173e-09 -100
"chr16" 68046001 68047000 "*" 4.73234496034536e-09 8.30627300264826e-09 -100
"chr16" 68050001 68051000 "*" 7.0006535457523e-08 1.03695832512054e-07 100
"chr16" 68074001 68075000 "*" 2.22044604925031e-16 9.81641919380259e-16
84.8484848484848
"chr16" 68269001 68270000 "*" 0 0 -88.271186440678
"chr16" 68270001 68271000 "*" 0 0 -96.4239271781534
"chr16" 68276001 68277000 "*" 0 0 -83.3333333333333
"chr16" 68345001 68346000 "*" 0 0 -100
"chr16" 68480001 68481000 "*" 3.51663143050018e-11 8.34133023205418e-11
60.8923884514436
"chr16" 68518001 68519000 "*" 5.55111512312578e-16 2.36485094870365e-15 100

"chr16" 68526001 68527000 "*" 0 0 100
"chr16" 68540001 68541000 "*" 5.48638023900594e-10 1.10374357336421e-09 100
"chr16" 68545001 68546000 "*" 2.4535928844216e-14 8.69144396197119e-14 100
"chr16" 68672001 68673000 "*" 3.6700841921089e-08 5.65530226072258e-08 100
"chr16" 68677001 68678000 "*" 0 0 86.25
"chr16" 68680001 68681000 "*" 2.44992914844033e-12 6.78890914397779e-12
78.9473684210526
"chr16" 68734001 68735000 "*" 1.12002908059239e-08 1.86609676863843e-08 -60
"chr16" 68770001 68771000 "*" 0 0 -80.3921568627451
"chr16" 68795001 68796000 "*" 1.59614987893519e-10 3.45981942954809e-10
54.7169811320755
"chr16" 68804001 68805000 "*" 1.38590361409285e-10 3.02706178892116e-10 100
"chr16" 68826001 68827000 "*" 0 0 100
"chr16" 68876001 68877000 "*" 6.34375885155691e-11 1.45441875258413e-10
66.6666666666667
"chr16" 69146001 69147000 "*" 3.76365605347928e-14 1.3043487970679e-13 100
"chr16" 69271001 69272000 "*" 9.08108033215171e-11 2.03407753381119e-10 -100
"chr16" 69362001 69363000 "*" 0 0 -100
"chr16" 69443001 69444000 "*" 0 0 -77.319587628866
"chr16" 69662001 69663000 "*" 1.52794177310511e-09 2.87642564232045e-09 100
"chr16" 69761001 69762000 "*" 0 0 -62.5698324022346
"chr16" 69779001 69780000 "*" 0 0 80.6451612903226
"chr16" 69788001 69789000 "*" 0 0 100
"chr16" 69924001 69925000 "*" 1.11022302462516e-16 5.03662826618488e-16 -100
"chr16" 70222001 70223000 "*" 0 0 63.716814159292
"chr16" 70400001 70401000 "*" 0 0 -100
"chr16" 70462001 70463000 "*" 0 0 100
"chr16" 70595001 70596000 "*" 4.78841410966879e-11 1.11468187156588e-10 -100
"chr16" 70626001 70627000 "*" 0 0 92.8571428571429
"chr16" 70636001 70637000 "*" 0 0 100
"chr16" 70673001 70674000 "*" 1.03420177421931e-07 1.49799664421382e-07
-51.1627906976744
"chr16" 70773001 70774000 "*" 0 0 100
"chr16" 70780001 70781000 "*" 0 0 -75.6756756756757
"chr16" 70794001 70795000 "*" 1.17905685215192e-13 3.83653245040640e-13 -100
"chr16" 70829001 70830000 "*" 4.78841410966879e-11 1.11468187156588e-10 -100
"chr16" 71405001 71406000 "*" 9.08108033215171e-11 2.03407753381119e-10 100
"chr16" 71446001 71447000 "*" 1.35447209004269e-14 4.94674708073053e-14 -100
"chr16" 71458001 71459000 "*" 0 0 100
"chr16" 71466001 71467000 "*" 1.11022302462516e-16 5.03662826618488e-16 75
"chr16" 71565001 71566000 "*" 1.14130926931466e-13 3.7276674571693e-13 -100
"chr16" 71586001 71587000 "*" 9.65729496371637e-10 1.87255912493232e-09 -100
"chr16" 72047001 72048000 "*" 0 0 -84.2592592592593
"chr16" 72154001 72155000 "*" 2.0370805042802e-09 3.78281826327819e-09
-54.0540540540541
"chr16" 72165001 72166000 "*" 5.48638023900594e-10 1.10374357336421e-09 100
"chr16" 72191001 72192000 "*" 6.99440505513849e-14 2.34592719571672e-13 -65.625
"chr16" 72279001 72280000 "*" 4.08209022140227e-11 9.57770690255255e-11 100
"chr16" 73030001 73031000 "*" 2.44805401505843e-08 3.87506495191586e-08
-66.6666666666667
"chr16" 73063001 73064000 "*" 2.72420863645095e-10 5.72438646940985e-10
56.140350877193

"chr16" 73081001 73082000 "*" 0 0 -83.7126631118047
"chr16" 73082001 73083000 "*" 0 0 -72.1649484536082
"chr16" 73104001 73105000 "*" 0 0 97.5308641975309
"chr16" 73260001 73261000 "*" 1.56863411149288e-12 4.4469621057462e-12 -100
"chr16" 73299001 73300000 "*" 6.52733422867868e-12 1.71146951718423e-11 -100
"chr16" 73380001 73381000 "*" 1.39779633423487e-07 1.98463037958589e-07 -100
"chr16" 73385001 73386000 "*" 8.7349629751543e-09 1.47505871898511e-08 100
"chr16" 73437001 73438000 "*" 1.29644872615131e-08 2.13799314548267e-08
-75.6653992395437
"chr16" 73536001 73537000 "*" 5.44009282066327e-15 2.08486137681812e-14 100
"chr16" 73575001 73576000 "*" 1.90817864564874e-06 2.32887155826907e-06 75
"chr16" 73577001 73578000 "*" 2.73625566649116e-12 7.52483363579137e-12 100
"chr16" 73578001 73579000 "*" 7.0006535457523e-08 1.03695832512054e-07 100
"chr16" 73589001 73590000 "*" 1.39779633423487e-07 1.98463037958589e-07 100
"chr16" 73593001 73594000 "*" 0 0 100
"chr16" 73840001 73841000 "*" 0 0 -100
"chr16" 73857001 73858000 "*" 1.68348002205221e-10 3.64173385832118e-10
96.6666666666667
"chr16" 73983001 73984000 "*" 5.01025332333427e-10 1.01358031135889e-09 -100
"chr16" 74024001 74025000 "*" 0.00062881353774058 0.000515972945757495
-51.8518518518519
"chr16" 74322001 74323000 "*" 1.76764438033494e-05 1.86641818672063e-05
-57.6923076923077
"chr16" 74483001 74484000 "*" 2.35030150896876e-07 3.24627842630129e-07
65.8479532163743
"chr16" 74514001 74515000 "*" 6.88338275267597e-15 2.60518659957509e-14 -100
"chr16" 74525001 74526000 "*" 1.11022302462516e-16 5.03662826618488e-16
-84.6153846153846
"chr16" 74759001 74760000 "*" 6.252366219206e-07 8.16567308913579e-07
-69.5652173913043
"chr16" 74774001 74775000 "*" 1.88737914186277e-15 7.62011598380321e-15 -100
"chr16" 74787001 74788000 "*" 1.11022302462516e-16 5.03662826618488e-16 -100
"chr16" 74847001 74848000 "*" 4.91828799908944e-14 1.68192819018269e-13 -100
"chr16" 74882001 74883000 "*" 7.67056418382595e-11 1.7359448580318e-10 -100
"chr16" 74918001 74919000 "*" 1.45439216225896e-14 5.29728884617264e-14
-50.4862461794943
"chr16" 75097001 75098000 "*" 7.0006535457523e-08 1.03695832512054e-07 -100
"chr16" 75172001 75173000 "*" 4.71134242729931e-12 1.257615036806e-11 100
"chr16" 75226001 75227000 "*" 3.07963307077941e-08 4.82018501272284e-08
66.6666666666667
"chr16" 75253001 75254000 "*" 1.67299207820548e-08 2.71377429441055e-08 100
"chr16" 75257001 75258000 "*" 1.1163110824608e-07 1.60902031425516e-07
54.6218487394958
"chr16" 75302001 75303000 "*" 1.93720595120794e-11 4.76309924836636e-11 -100
"chr16" 75363001 75364000 "*" 2.33028818463765e-10 4.93719283454501e-10 100
"chr16" 75432001 75433000 "*" 1.48087875295744e-10 3.22075573380618e-10 100
"chr16" 75551001 75552000 "*" 0 0 100
"chr16" 75679001 75680000 "*" 0 0 -100
"chr16" 75683001 75684000 "*" 1.24493265585279e-07 1.78331646419667e-07
-72.9166666666667
"chr16" 76870001 76871000 "*" 1.10219389171107e-10 2.44660222411491e-10
82.1428571428571

"chr16" 77146001 77147000 "*" 0 0 -100
"chr16" 77319001 77320000 "*" 2.02060590481778e-13 6.40127405465654e-13 -100
"chr16" 77862001 77863000 "*" 1.92946192356658e-09 3.59193011611483e-09 100
"chr16" 77872001 77873000 "*" 0 0 100
"chr16" 77981001 77982000 "*" 4.73234496034536e-09 8.30627300264826e-09 100
"chr16" 77997001 77998000 "*" 5.794809077031e-12 1.52911192757929e-11 100
"chr16" 78080001 78081000 "*" 0 0 65.6462585034014
"chr16" 78134001 78135000 "*" 7.0006535457523e-08 1.03695832512054e-07 100
"chr16" 78247001 78248000 "*" 0.000144402658994736 0.000131924792739962
66.6666666666667
"chr16" 78460001 78461000 "*" 1.43950407149873e-11 3.60116512249416e-11 -80
"chr16" 78571001 78572000 "*" 2.51135823137183e-07 3.45648271894177e-07 -52
"chr16" 78627001 78628000 "*" 1.13140401492018e-08 1.87801380787728e-08 -100
"chr16" 78697001 78698000 "*" 4.99020824662466e-11 1.15790792586157e-10 100
"chr16" 78714001 78715000 "*" 0 0 -100
"chr16" 78804001 78805000 "*" 1.11022302462516e-16 5.03662826618488e-16
73.0769230769231
"chr16" 78825001 78826000 "*" 4.85906552771809e-08 7.38253423265473e-08 -60
"chr16" 78843001 78844000 "*" 4.86753970463383e-11 1.13145908161915e-10 100
"chr16" 79002001 79003000 "*" 0 0 -66.2162162162162
"chr16" 79034001 79035000 "*" 0 0 -100
"chr16" 79040001 79041000 "*" 3.63445940010365e-11 8.58565209111204e-11 100
"chr16" 79044001 79045000 "*" 9.98963478604509e-09 1.67533179897223e-08
-66.6666666666667
"chr16" 79124001 79125000 "*" 0 0 -100
"chr16" 79263001 79264000 "*" 0 0 -51.1052631578947
"chr16" 79286001 79287000 "*" 0 0 -100
"chr16" 79293001 79294000 "*" 1.51914612489534e-06 1.88121349342686e-06
-52.3809523809524
"chr16" 79321001 79322000 "*" 3.77475828372553e-15 1.47379557022071e-14 -100
"chr16" 79335001 79336000 "*" 6.82121026329696e-13 2.0226612036401e-12
-81.6901408450704
"chr16" 79357001 79358000 "*" 1.11022302462516e-16 5.03662826618488e-16
-53.4883720930233
"chr16" 79364001 79365000 "*" 2.00227675550835e-08 3.20459176617961e-08 -100
"chr16" 79372001 79373000 "*" 6.90747459231034e-12 1.80140148623876e-11 -100
"chr16" 79423001 79424000 "*" 0 0 78.7878787878788
"chr16" 79553001 79554000 "*" 0 0 -100
"chr16" 79624001 79625000 "*" 0 0 -74.5098039215686
"chr16" 79632001 79633000 "*" 0 0 83.0028328611898
"chr16" 79633001 79634000 "*" 0 0 63.1988154773596
"chr16" 79634001 79635000 "*" 0 0 84.964200477327
"chr16" 79635001 79636000 "*" 0 0 59.8705501618123
"chr16" 79741001 79742000 "*" 4.02167188440217e-12 1.08266700497926e-11 -100
"chr16" 79828001 79829000 "*" 6.04438721296674e-11 1.38849255306602e-10 -100
"chr16" 79909001 79910000 "*" 6.4152538836737e-10 1.27477697728417e-09 100
"chr16" 79919001 79920000 "*" 8.01666433236647e-09 1.36392368970109e-08 100
"chr16" 80012001 80013000 "*" 1.17461596005342e-13 3.8279067575877e-13 -100
"chr16" 80095001 80096000 "*" 9.5812247025151e-14 3.15667542866807e-13 100
"chr16" 80276001 80277000 "*" 1.71171965490657e-11 4.24308551748959e-11
69.0140845070423
"chr16" 80387001 80388000 "*" 2.53090843860271e-06 3.03222903732048e-06

-52.1739130434783
"chr16" 80574001 80575000 "*" 0 0 94.1734417344173
"chr16" 80773001 80774000 "*" 1.13140401492018e-08 1.87801380787728e-08 100
"chr16" 80838001 80839000 "*" 0 0 59.9056603773585
"chr16" 80844001 80845000 "*" 2.81154688464014e-06 3.34643884318492e-06 -55
"chr16" 80941001 80942000 "*" 4.99900121297969e-12 1.32921056339624e-11 100
"chr16" 81128001 81129000 "*" 0 0 -52.9411764705882
"chr16" 81144001 81145000 "*" 3.57708129783418e-09 6.41627394521332e-09
55.1724137931034
"chr16" 81154001 81155000 "*" 6.14507200680237e-09 1.06432265548624e-08
-66.6666666666667
"chr16" 81191001 81192000 "*" 9.52204174803351e-05 8.95780133799817e-05
-59.2592592592593
"chr16" 81197001 81198000 "*" 1.11022302462516e-16 5.03662826618488e-16
83.3333333333333
"chr16" 81206001 81207000 "*" 0 0 100
"chr16" 81208001 81209000 "*" 2.242854002521e-08 3.56690414193825e-08
86.1111111111111
"chr16" 81211001 81212000 "*" 3.34128893442198e-08 5.19772864618523e-08 -100
"chr16" 81216001 81217000 "*" 7.0006535457523e-08 1.03695832512054e-07 -100
"chr16" 81219001 81220000 "*" 0 0 100
"chr16" 81242001 81243000 "*" 1.98365768255826e-11 4.85612402473442e-11 100
"chr16" 81295001 81296000 "*" 2.9972135884293e-11 7.17540839458191e-11
66.6666666666667
"chr16" 81323001 81324000 "*" 3.33066907387547e-16 1.4495649018245e-15 -100
"chr16" 81352001 81353000 "*" 4.71134242729931e-12 1.257615036806e-11 100
"chr16" 81437001 81438000 "*" 1.35579991678014e-10 2.97275437103139e-10
-83.7837837837838
"chr16" 81481001 81482000 "*" 6.4152538836737e-10 1.27477697728417e-09 -100
"chr16" 81485001 81486000 "*" 2.62927728367224e-07 3.60853669669655e-07
-73.1707317073171
"chr16" 81523001 81524000 "*" 4.51551129820871e-09 7.9662089949093e-09 56
"chr16" 81527001 81528000 "*" 0 0 -56.669783387876
"chr16" 81602001 81603000 "*" 0 0 -100
"chr16" 81619001 81620000 "*" 1.11022302462516e-16 5.03662826618488e-16 -100
"chr16" 81632001 81633000 "*" 6.59550958292954e-09 1.13456194382773e-08 -100
"chr16" 81655001 81656000 "*" 0 0 75
"chr16" 81773001 81774000 "*" 1.62171751072471e-06 1.99994633409834e-06
-71.4285714285714
"chr16" 81789001 81790000 "*" 0 0 100
"chr16" 81843001 81844000 "*" 3.88022947106492e-13 1.1867462770515e-12 -100
"chr16" 81878001 81879000 "*" 0 0 -100
"chr16" 81887001 81888000 "*" 3.90465437760668e-13 1.19307893943277e-12 100
"chr16" 81900001 81901000 "*" 1.48532297572501e-10 3.22991607959143e-10
72.1311475409836
"chr16" 81950001 81951000 "*" 0 0 100
"chr16" 81962001 81963000 "*" 0 0 71.4285714285714
"chr16" 82297001 82298000 "*" 4.73234496034536e-09 8.30627300264826e-09 -100
"chr16" 82308001 82309000 "*" 8.65973959207622e-14 2.87362558212404e-13 100
"chr16" 82521001 82522000 "*" 0 0 -100
"chr16" 82701001 82702000 "*" 6.65646821215171e-05 6.41959941692905e-05
53.8461538461538

"chr16" 82737001 82738000 "*" 8.8118947028093e-08 1.28770353209608e-07 75.5555555555556
"chr16" 82848001 82849000 "*" 5.01025332333427e-10 1.01358031135889e-09 -100
"chr16" 83176001 83177000 "*" 1.11022302462516e-16 5.03662826618488e-16 -100
"chr16" 83247001 83248000 "*" 1.11022302462516e-16 5.03662826618488e-16 -100
"chr16" 83331001 83332000 "*" 5.8616765863384e-07 7.68442756313584e-07 -91.6666666666667
"chr16" 83353001 83354000 "*" 6.93112234273485e-13 2.05218842007357e-12 -100
"chr16" 83469001 83470000 "*" 2.73625566649116e-12 7.52483363579137e-12 -100
"chr16" 83654001 83655000 "*" 4.73234496034536e-09 8.30627300264826e-09 -100
"chr16" 83656001 83657000 "*" 3.6700841921089e-08 5.65530226072258e-08 -100
"chr16" 83712001 83713000 "*" 0.000189884794687623 0.000170160803519512 -54.3859649122807
"chr16" 83744001 83745000 "*" 0 0 100
"chr16" 83761001 83762000 "*" 1.78174519582797e-09 3.33248393519906e-09 -85.3932584269663
"chr16" 83795001 83796000 "*" 3.56775498033812e-10 7.35898629809829e-10 100
"chr16" 83850001 83851000 "*" 4.44089209850063e-16 1.91071758245033e-15 -100
"chr16" 83921001 83922000 "*" 1.00293011964236e-05 1.09889190312666e-05 -73.0769230769231
"chr16" 83973001 83974000 "*" 6.84545080620325e-08 1.01954634767777e-07 63.6363636363636
"chr16" 84034001 84035000 "*" 1.11022302462516e-16 5.03662826618488e-16 -100
"chr16" 84035001 84036000 "*" 2.72004641033163e-14 9.58564042979718e-14 84.7161572052402
"chr16" 84061001 84062000 "*" 0 0 70.6533776301218
"chr16" 84076001 84077000 "*" 0 0 68.0851063829787
"chr16" 84172001 84173000 "*" 1.14352971536391e-14 4.21921674335999e-14 100
"chr16" 84194001 84195000 "*" 0 0 83.7837837837838
"chr16" 84238001 84239000 "*" 0 0 -80.5827067669173
"chr16" 84252001 84253000 "*" 1.11022302462516e-16 5.03662826618488e-16 100
"chr16" 84265001 84266000 "*" 1.11022302462516e-15 4.59817122935606e-15 100
"chr16" 84269001 84270000 "*" 0.000110450472473933 0.000102865956280942 57.1428571428571
"chr16" 84316001 84317000 "*" 6.98330282489223e-14 2.34267456154807e-13 100
"chr16" 84332001 84333000 "*" 5.01025332333427e-10 1.01358031135889e-09 -100
"chr16" 84335001 84336000 "*" 1.11022302462516e-16 5.03662826618488e-16 70
"chr16" 84361001 84362000 "*" 1.25284005392245e-10 2.75649590272477e-10 -100
"chr16" 84402001 84403000 "*" 0 0 72.459499263623
"chr16" 84469001 84470000 "*" 4.14335232790108e-13 1.26153063645578e-12 100
"chr16" 84529001 84530000 "*" 3.4639957569027e-11 8.22349663902986e-11 90
"chr16" 84544001 84545000 "*" 0 0 -100
"chr16" 84619001 84620000 "*" 0 0 100
"chr16" 84636001 84637000 "*" 0 0 -96.5317919075144
"chr16" 84712001 84713000 "*" 1.49880108324396e-14 5.44951848293151e-14 -54.9019607843137
"chr16" 84718001 84719000 "*" 1.98365768255826e-11 4.85612402473442e-11 100
"chr16" 84764001 84765000 "*" 3.33066907387547e-16 1.4495649018245e-15 -66.8918918918919
"chr16" 84841001 84842000 "*" 1.33226762955019e-15 5.48133041560118e-15 100
"chr16" 84883001 84884000 "*" 3.33066907387547e-16 1.4495649018245e-15 95
"chr16" 84886001 84887000 "*" 6.16459217006593e-10 1.23295830479732e-09

54.5454545454545
"chr16" 84964001 84965000 "*" 4.99900121297969e-12 1.32921056339624e-11 100
"chr16" 84984001 84985000 "*" 5.14563234778054e-05 5.05166828508704e-05
-50.6666666666667
"chr16" 85038001 85039000 "*" 1.05428354124637e-06 1.33429012030175e-06
-51.1111111111111
"chr16" 85063001 85064000 "*" 0 0 86.1313868613139
"chr16" 85075001 85076000 "*" 1.33988231620918e-09 2.54938331917089e-09
65.6862745098039
"chr16" 85084001 85085000 "*" 1.51656465163796e-13 4.87803420672651e-13 100
"chr16" 85124001 85125000 "*" 1.94308505176988e-05 2.03668117319786e-05
-54.320987654321
"chr16" 85133001 85134000 "*" 0.000624048363117358 0.000512350616097199
-53.8461538461538
"chr16" 85140001 85141000 "*" 5.99520433297585e-15 2.28531569014092e-14
67.0212765957447
"chr16" 85142001 85143000 "*" 5.55111512312578e-16 2.36485094870365e-15
57.1428571428571
"chr16" 85190001 85191000 "*" 1.11022302462516e-16 5.03662826618488e-16 100
"chr16" 85208001 85209000 "*" 1.29055433006897e-10 2.83528609148655e-10
56.4102564102564
"chr16" 85244001 85245000 "*" 0 0 100
"chr16" 85281001 85282000 "*" 0 0 -79.6610169491525
"chr16" 85352001 85353000 "*" 0 0 51.1442611442611
"chr16" 85381001 85382000 "*" 6.90747459231034e-12 1.80140148623876e-11 -100
"chr16" 85384001 85385000 "*" 2.41741355513359e-05 2.49875818383709e-05
62.962962962963
"chr16" 85421001 85422000 "*" 1.97630800613524e-11 4.85166058748038e-11 -60
"chr16" 85425001 85426000 "*" 4.87079154787295e-10 9.88099362440924e-10
-57.6923076923077
"chr16" 85431001 85432000 "*" 0 0 -91.7948717948718
"chr16" 85437001 85438000 "*" 8.81691275544938e-10 1.72436138477978e-09
-88.7755102040816
"chr16" 85461001 85462000 "*" 1.12458486967171e-09 2.15740815043925e-09 -100
"chr16" 85693001 85694000 "*" 2.17381668221606e-13 6.85151076607736e-13 -100
"chr16" 85711001 85712000 "*" 0 0 69.0476190476191
"chr16" 85740001 85741000 "*" 0 0 -100
"chr16" 85783001 85784000 "*" 2.18269846641306e-13 6.87638625669691e-13
-52.5641025641026
"chr16" 85859001 85860000 "*" 0 0 -100
"chr16" 85861001 85862000 "*" 0 0 -70.2702702702703
"chr16" 85876001 85877000 "*" 6.4152538836737e-10 1.27477697728417e-09 100
"chr16" 85893001 85894000 "*" 5.33495114529714e-06 6.09237962218305e-06
-66.6666666666667
"chr16" 85894001 85895000 "*" 1.51914609258785e-06 1.88121349342686e-06
73.6842105263158
"chr16" 85907001 85908000 "*" 0 0 -66.6666666666667
"chr16" 85932001 85933000 "*" 0 0 59.7994308366128
"chr16" 85933001 85934000 "*" 1.35880195983873e-11 3.40841971363279e-11
-89.8989898989899
"chr16" 86067001 86068000 "*" 0 0 -87.6288659793814
"chr16" 86068001 86069000 "*" 3.6700841921089e-08 5.65530226072258e-08 -100

"chr16" 86078001 86079000 "*" 1.53526191759568e-10 3.33329638295662e-10
67.3469387755102
"chr16" 86079001 86080000 "*" 4.32209823486573e-12 1.15935290479641e-11 -100
"chr16" 86093001 86094000 "*" 1.13140401492018e-08 1.87801380787728e-08 100
"chr16" 86094001 86095000 "*" 6.12868581397752e-05 5.94427906677698e-05 -60
"chr16" 86096001 86097000 "*" 0 0 -67.5889328063241
"chr16" 86099001 86100000 "*" 0 0 100
"chr16" 86108001 86109000 "*" 1.38925759773922e-06 1.7304914035083e-06
-64.8854961832061
"chr16" 86113001 86114000 "*" 0 0 -59.5238095238095
"chr16" 86122001 86123000 "*" 1.15990550497713e-11 2.937207455111e-11
64.5161290322581
"chr16" 86134001 86135000 "*" 0 0 100
"chr16" 86140001 86141000 "*" 1.80770820534804e-09 3.37846860013928e-09 -52
"chr16" 86168001 86169000 "*" 0.000173919515143783 0.000156793955027816 60
"chr16" 86234001 86235000 "*" 1.51809678017312e-06 1.88016527773109e-06 -52
"chr16" 86235001 86236000 "*" 0 0 64.8351648351648
"chr16" 86244001 86245000 "*" 1.14124265593318e-11 2.89138062669327e-11 100
"chr16" 86247001 86248000 "*" 1.21324639223985e-09 2.31901530283899e-09
72.972972972973
"chr16" 86248001 86249000 "*" 8.23023486473673e-07 1.05693812103436e-06
-66.6666666666667
"chr16" 86252001 86253000 "*" 1.39779633423487e-07 1.98463037958589e-07 100
"chr16" 86261001 86262000 "*" 5.40034683638169e-12 1.43090827942664e-11
66.6666666666667
"chr16" 86263001 86264000 "*" 2.22044604925031e-16 9.81641919380259e-16 100
"chr16" 86264001 86265000 "*" 0 0 100
"chr16" 86276001 86277000 "*" 8.62854232508425e-12 2.22278438222256e-11
64.7058823529412
"chr16" 86277001 86278000 "*" 1.66533453693773e-15 6.7629186866784e-15 100
"chr16" 86279001 86280000 "*" 1.37828859436695e-10 3.01445544267661e-10 100
"chr16" 86282001 86283000 "*" 9.76996261670138e-15 3.63402770315216e-14 -100
"chr16" 86284001 86285000 "*" 5.62883073484954e-13 1.68905058935478e-12 100
"chr16" 86304001 86305000 "*" 8.3882012447134e-11 1.88922989503423e-10 -100
"chr16" 86319001 86320000 "*" 1.89581683684992e-12 5.32311367606535e-12 100
"chr16" 86328001 86329000 "*" 8.14276024385663e-09 1.38399372229876e-08
-59.4594594594595
"chr16" 86338001 86339000 "*" 0 0 62.5
"chr16" 86358001 86359000 "*" 0 0 51.9480519480519
"chr16" 86391001 86392000 "*" 6.27559182508897e-07 8.19254190448222e-07
54.8387096774194
"chr16" 86406001 86407000 "*" 1.17905685215192e-13 3.83653245040640e-13 100
"chr16" 86407001 86408000 "*" 2.87908585860919e-11 6.92560835405971e-11
-54.5454545454545
"chr16" 86437001 86438000 "*" 0 0 100
"chr16" 86460001 86461000 "*" 6.92287338566189e-11 1.57605630124247e-10 100
"chr16" 86462001 86463000 "*" 1.5277158427196e-09 2.87642564232045e-09 -100
"chr16" 86464001 86465000 "*" 3.6700841921089e-08 5.65530226072258e-08 100
"chr16" 86474001 86475000 "*" 0 0 100
"chr16" 86492001 86493000 "*" 0 0 -94.8717948717949
"chr16" 86522001 86523000 "*" 5.03264097062583e-13 1.51942428521737e-12 -100
"chr16" 86523001 86524000 "*" 1.83952852950142e-12 5.17499701329218e-12 100

"chr16" 86535001 86536000 "*" 2.62900812231237e-13 8.21294938531841e-13 100
"chr16" 86543001 86544000 "*" 0 0 71.1333236644007
"chr16" 86551001 86552000 "*" 0 0 100
"chr16" 86593001 86594000 "*" 1.84928616864255e-09 3.45276937834312e-09 76.7123287671233
"chr16" 86610001 86611000 "*" 0 0 59.8289136013687
"chr16" 86629001 86630000 "*" 4.96817325845722e-06 5.69716498812773e-06 69.2307692307692
"chr16" 86653001 86654000 "*" 9.44333224728311e-07 1.20270675453519e-06 74.0740740740741
"chr16" 86671001 86672000 "*" 5.58502334380151e-08 8.41879077739806e-08 73.6842105263158
"chr16" 86674001 86675000 "*" 0 0 100
"chr16" 86697001 86698000 "*" 7.88258347483861e-15 2.96145301916012e-14 -100
"chr16" 86706001 86707000 "*" 3.25818809512324e-05 3.29735679006858e-05 66.6666666666667
"chr16" 86714001 86715000 "*" 0 0 90.4761904761905
"chr16" 86752001 86753000 "*" 5.51850422814137e-06 6.28718511088478e-06 69.4444444444444
"chr16" 86763001 86764000 "*" 6.29532538907185e-05 6.09541335910211e-05 -53.3333333333333
"chr16" 86768001 86769000 "*" 9.63229496164786e-13 2.80378372615919e-12 72.1052631578947
"chr16" 86771001 86772000 "*" 5.80595602706069e-08 8.73477170489365e-08 68.5714285714286
"chr16" 86785001 86786000 "*" 6.4152538836737e-10 1.27477697728417e-09 100
"chr16" 86786001 86787000 "*" 0 0 66.6442460560108
"chr16" 86806001 86807000 "*" 7.97140131680862e-14 2.65641277678906e-13 100
"chr16" 86828001 86829000 "*" 1.8893420161703e-10 4.06754022981325e-10 72.972972972973
"chr16" 86860001 86861000 "*" 4.44089209850063e-16 1.91071758245033e-15 57.1428571428571
"chr16" 86877001 86878000 "*" 0 0 73.3333333333333
"chr16" 86881001 86882000 "*" 9.04570973681018e-10 1.76414389822173e-09 100
"chr16" 86885001 86886000 "*" 1.17905685215192e-13 3.83653245040640e-13 100
"chr16" 86886001 86887000 "*" 1.11022302462516e-16 5.03662826618488e-16 -100
"chr16" 86890001 86891000 "*" 2.44859916675999e-07 3.37428127603975e-07 -74.1935483870968
"chr16" 86897001 86898000 "*" 2.43005615629954e-09 4.45014222280278e-09 -100
"chr16" 86914001 86915000 "*" 1.44296796733556e-11 3.60961410260146e-11 87.2727272727273
"chr16" 86916001 86917000 "*" 4.14535072934541e-12 1.11495465689902e-11 65.625
"chr16" 86943001 86944000 "*" 0 0 57.7319587628866
"chr16" 86969001 86970000 "*" 0 0 -100
"chr16" 86975001 86976000 "*" 0 0 100
"chr16" 86985001 86986000 "*" 1.29596333664495e-12 3.7222938155926e-12 -65.9574468085106
"chr16" 86997001 86998000 "*" 2.22044604925031e-16 9.81641919380259e-16 -52.061754934532
"chr16" 87003001 87004000 "*" 1.92946192356658e-09 3.59193011611483e-09 100
"chr16" 87004001 87005000 "*" 3.6700841921089e-08 5.65530226072258e-08 -100
"chr16" 87018001 87019000 "*" 1.17794662912729e-13 3.83653245040640e-13 100

"chr16" 87032001 87033000 "*" 0 0 90.523598820059
"chr16" 87044001 87045000 "*" 1.34559030584569e-13 4.35581312463895e-13 100
"chr16" 87062001 87063000 "*" 1.17816723044228e-09 2.25485869667672e-09
81.8181818181818
"chr16" 87076001 87077000 "*" 0 0 84.7826086956522
"chr16" 87105001 87106000 "*" 0 0 100
"chr16" 87108001 87109000 "*" 2.51078840070029e-05 2.58712012060582e-05 -62.5
"chr16" 87123001 87124000 "*" 2.73014943985572e-11 6.58595916221891e-11 100
"chr16" 87125001 87126000 "*" 6.52733422867868e-12 1.71146951718423e-11 100
"chr16" 87194001 87195000 "*" 0 0 84.5730027548209
"chr16" 87215001 87216000 "*" 2.15125472990962e-10 4.58126659943874e-10 100
"chr16" 87229001 87230000 "*" 0 0 100
"chr16" 87262001 87263000 "*" 1.21779554407286e-06 1.52805147129053e-06
66.0714285714286
"chr16" 87271001 87272000 "*" 2.00227675550835e-08 3.20459176617961e-08 100
"chr16" 87280001 87281000 "*" 0 0 100
"chr16" 87290001 87291000 "*" 1.12055920098442e-11 2.84500040664577e-11 -100
"chr16" 87307001 87308000 "*" 2.67283972732457e-11 6.45686178157698e-11 100
"chr16" 87313001 87314000 "*" 1.11022302462516e-16 5.03662826618488e-16 100
"chr16" 87354001 87355000 "*" 9.39626154661255e-12 2.41317596199128e-11
87.1212121212121
"chr16" 87524001 87525000 "*" 0 0 63.6455893832943
"chr16" 87530001 87531000 "*" 8.25450818808804e-13 2.42473843346228e-12 100
"chr16" 87575001 87576000 "*" 1.35890187991095e-11 3.40841971363279e-11 -100
"chr16" 87603001 87604000 "*" 6.06847905260111e-13 1.81305886631896e-12
59.2920353982301
"chr16" 87608001 87609000 "*" 3.90465437760668e-13 1.19307893943277e-12 100
"chr16" 87621001 87622000 "*" 2.22044604925031e-16 9.81641919380259e-16
52.6315789473684
"chr16" 87625001 87626000 "*" 1.11022302462516e-16 5.03662826618488e-16
81.4569536423841
"chr16" 87627001 87628000 "*" 0 0 -77.7777777777778
"chr16" 87642001 87643000 "*" 1.05066907396534e-08 1.75709911877311e-08
-63.1578947368421
"chr16" 87647001 87648000 "*" 3.2337384772152e-06 3.81325174359463e-06
-54.1666666666667
"chr16" 87663001 87664000 "*" 8.21565038222616e-15 3.08158744293689e-14 -100
"chr16" 87683001 87684000 "*" 0 0 70.5882352941177
"chr16" 87687001 87688000 "*" 1.11022302462516e-16 5.03662826618488e-16
62.9032258064516
"chr16" 87726001 87727000 "*" 1.8158710455296e-06 2.2229188794026e-06 60
"chr16" 87757001 87758000 "*" 0 0 -100
"chr16" 87798001 87799000 "*" 0 0 -100
"chr16" 87810001 87811000 "*" 3.17554433859613e-08 4.96229433769447e-08
-56.4102564102564
"chr16" 87850001 87851000 "*" 2.52275977885574e-12 6.97751289526057e-12
73.1707317073171
"chr16" 87887001 87888000 "*" 0 0 63.0090497737557
"chr16" 87901001 87902000 "*" 6.77335965093562e-12 1.76897011246462e-11 -100
"chr16" 87951001 87952000 "*" 2.61131560819194e-10 5.50561320957241e-10
-57.4603174603175
"chr16" 87968001 87969000 "*" 0.000258832568460265 0.000226861893436355

-58.3333333333333
"chr16" 87972001 87973000 "*" 1.49972523288966e-09 2.83643172292987e-09
-66.6666666666667
"chr16" 87979001 87980000 "*" 3.62299079625927e-12 9.83529579854242e-12
70.9090909090909
"chr16" 88000001 88001000 "*" 5.23026122412062e-08 7.90932950309586e-08
61.5384615384615
"chr16" 88134001 88135000 "*" 0 0 64.327485380117
"chr16" 88141001 88142000 "*" 0.000175834889814652 0.000158411134466825
-51.8518518518519
"chr16" 88148001 88149000 "*" 0 0 -70.6832298136646
"chr16" 88190001 88191000 "*" 3.95353005888666e-09 7.03928529283733e-09 100
"chr16" 88221001 88222000 "*" 8.88178419700125e-16 3.70670032207938e-15
85.7142857142857
"chr16" 88254001 88255000 "*" 1.94073368486869e-09 3.61162401710587e-09
81.8181818181818
"chr16" 88314001 88315000 "*" 4.73959188673767e-07 6.28368274925326e-07
52.1739130434783
"chr16" 88320001 88321000 "*" 1.11890252618707e-09 2.15115838001743e-09
62.1621621621622
"chr16" 88321001 88322000 "*" 4.11987440829265e-07 5.50491688288806e-07
-67.741935483871
"chr16" 88338001 88339000 "*" 6.98330282489223e-14 2.34267456154807e-13 100
"chr16" 88350001 88351000 "*" 8.36868352394049e-11 1.88738247327057e-10
51.1627906976744
"chr16" 88357001 88358000 "*" 1.33615341013638e-12 3.83192206814796e-12
53.5714285714286
"chr16" 88359001 88360000 "*" 3.58602036953926e-14 1.24750395498083e-13
90.8496732026144
"chr16" 88449001 88450000 "*" 0 0 52.9135492290505
"chr16" 88461001 88462000 "*" 2.62097644804227e-05 2.69333738920509e-05
-64.2857142857143
"chr16" 88462001 88463000 "*" 0 0 68.8524590163934
"chr16" 88486001 88487000 "*" 0 0 -64.4814814814815
"chr16" 88487001 88488000 "*" 1.96817614717126e-05 2.06135581725463e-05
60.4048582995951
"chr16" 88509001 88510000 "*" 0 0 -52.7620968155953
"chr16" 88607001 88608000 "*" 0 0 61.8054077070471
"chr16" 88621001 88622000 "*" 2.62186938826403e-11 6.34254943568664e-11
53.7313432835821
"chr16" 88623001 88624000 "*" 0 0 99
"chr16" 88717001 88718000 "*" 0 0 -63.3042499296369
"chr16" 88754001 88755000 "*" 5.34132904572715e-09 9.31927292646869e-09
-60.2272727272727
"chr16" 88899001 88900000 "*" 0 0 -100
"chr16" 89012001 89013000 "*" 8.25450818808804e-13 2.42473843346228e-12 -100
"chr16" 89028001 89029000 "*" 0.000189884811966357 0.000170160803519512
54.3859649122807
"chr16" 89045001 89046000 "*" 0 0 -90.4
"chr16" 89052001 89053000 "*" 0 0 50.3030303030303
"chr16" 89053001 89054000 "*" 0 0 100
"chr16" 89060001 89061000 "*" 6.97602285604226e-06 7.82816889037512e-06 -60

"chr16" 89070001 89071000 "*" 0 0 66.4703622149645
"chr16" 89071001 89072000 "*" 8.63753513158372e-14 2.86865422934981e-13
62.6506024096386
"chr16" 89072001 89073000 "*" 0 0 67.3101839854885
"chr16" 89076001 89077000 "*" 0 0 -100
"chr16" 89079001 89080000 "*" 2.79440914852103e-11 6.73046092948135e-11 -100
"chr16" 89090001 89091000 "*" 4.55191440096314e-15 1.76090186737198e-14
75.7446808510638
"chr16" 89101001 89102000 "*" 0 0 54.2056074766355
"chr16" 89105001 89106000 "*" 3.53568296862505e-09 6.34595527715708e-09
82.0359281437126
"chr16" 89110001 89111000 "*" 0 0 -78.2608695652174
"chr16" 89113001 89114000 "*" 0 0 97.5609756097561
"chr16" 89123001 89124000 "*" 3.77475828372553e-15 1.47379557022071e-14
55.8139534883721
"chr16" 89136001 89137000 "*" 1.12132525487141e-14 4.14352271980498e-14 -100
"chr16" 89145001 89146000 "*" 1.05049302590032e-12 3.04673287390084e-12
-88.4955752212389
"chr16" 89156001 89157000 "*" 3.19867168363519e-06 3.77542382949913e-06
62.043795620438
"chr16" 89159001 89160000 "*" 2.88657986402541e-15 1.14155613124573e-14 100
"chr16" 89215001 89216000 "*" 1.39779633423487e-07 1.98463037958589e-07 -100
"chr16" 89237001 89238000 "*" 0 0 60.458452722063
"chr16" 89239001 89240000 "*" 8.52140580320793e-12 2.19817259951489e-11 100
"chr16" 89241001 89242000 "*" 1.33226762955019e-15 5.48133041560118e-15
76.9303201506591
"chr16" 89287001 89288000 "*" 9.08108033215171e-11 2.03407753381119e-10 100
"chr16" 89309001 89310000 "*" 1.26565424807268e-14 4.63923203146481e-14
-76.8115942028985
"chr16" 89313001 89314000 "*" 0 0 75.6785243741765
"chr16" 89392001 89393000 "*" 5.55111512312578e-16 2.36485094870365e-15
-74.3589743589744
"chr16" 89394001 89395000 "*" 0.000128743268926979 0.000118607263594326
55.2631578947368
"chr16" 89395001 89396000 "*" 0 0 -78.9356854956481
"chr16" 89396001 89397000 "*" 0 0 -51.9377417572297
"chr16" 89399001 89400000 "*" 3.06365044444459e-10 6.38473181084758e-10
-58.4615384615385
"chr16" 89427001 89428000 "*" 0 0 100
"chr16" 89462001 89463000 "*" 1.63614483406072e-07 2.30409839933864e-07
-79.1891891891892
"chr16" 89466001 89467000 "*" 1.73416836446449e-13 5.55119560371298e-13
-52.9411764705882
"chr16" 89525001 89526000 "*" 1.91224813761437e-12 5.36527594469447e-12 -100
"chr16" 89540001 89541000 "*" 2.37587727269783e-14 8.44268514944447e-14 -100
"chr16" 89565001 89566000 "*" 8.00762784916031e-05 7.62332949242677e-05
52.1739130434783
"chr16" 89572001 89573000 "*" 6.66133814775094e-16 2.81595744474255e-15 -100
"chr16" 89606001 89607000 "*" 1.54316870393245e-09 2.90430440605204e-09
96.2962962962963
"chr16" 89624001 89625000 "*" 5.8662206425808e-08 8.81939088887157e-08
-63.5036496350365

"chr16" 89641001 89642000 "*" 0 0 -85.9872611464968
"chr16" 89673001 89674000 "*" 0.000118603937450446 0.000109926776654688
-61.5384615384615
"chr16" 89710001 89711000 "*" 1.78760792057808e-07 2.50605547872388e-07
65.7142857142857
"chr16" 89723001 89724000 "*" 0 0 -51.9089688758468
"chr16" 89801001 89802000 "*" 1.04120045918421e-11 2.65824360918148e-11
55.1724137931034
"chr16" 89875001 89876000 "*" 2.82454060140935e-11 6.79949934411161e-11
-64.5390070921986
"chr16" 89984001 89985000 "*" 0 0 62.5441696113074
"chr16" 89992001 89993000 "*" 0 0 50.7454273877632
"chr16" 90004001 90005000 "*" 0 0 85
"chr16" 90014001 90015000 "*" 0 0 -51.4285714285714
"chr16" 90074001 90075000 "*" 1.38624201007076e-09 2.63314619100769e-09
54.9453883495146
"chr16" 90118001 90119000 "*" 1.73091857513796e-08 2.80261343773516e-08 56.25
"chr16" 90119001 90120000 "*" 1.30340183090993e-13 4.22662705118657e-13 100
"chr16" 90232001 90233000 "*" 1.16573417585641e-14 4.29335582900508e-14 -100
"chr17" 1 1000 "*" 7.87891973885735e-12 2.04330793118257e-11 -57.8947368421053
"chr17" 8001 9000 "*" 3.03979064142368e-13 9.42350883309283e-13 100
"chr17" 14001 15000 "*" 1.33226762955019e-15 5.48133041560118e-15
93.1712962962963
"chr17" 23001 24000 "*" 3.56115137378765e-12 9.67466388444195e-12
-58.3333333333333
"chr17" 26001 27000 "*" 1.93720595120794e-11 4.76309924836636e-11 100
"chr17" 51001 52000 "*" 4.46405532761318e-05 4.42311628182131e-05
-52.3809523809524
"chr17" 97001 98000 "*" 1.50990331349021e-13 4.86129511665914e-13 60
"chr17" 236001 237000 "*" 4.21332968514321e-11 9.873131755333e-11
67.6470588235294
"chr17" 238001 239000 "*" 1.07882591748876e-11 2.74553537455803e-11 100
"chr17" 244001 245000 "*" 7.76179120975939e-12 2.01393397202252e-11
-77.1428571428571
"chr17" 254001 255000 "*" 0 0 100
"chr17" 263001 264000 "*" 0 0 -56.8132660418169
"chr17" 266001 267000 "*" 0.000296795293984187 0.000257472949048318
-53.8461538461538
"chr17" 408001 409000 "*" 1.97126177603479e-08 3.16979535786496e-08 -90
"chr17" 570001 571000 "*" 1.25284005392245e-10 2.75649590272477e-10 100
"chr17" 631001 632000 "*" 0 0 69.3548387096774
"chr17" 804001 805000 "*" 5.51780843238703e-14 1.87243792572872e-13 -100
"chr17" 853001 854000 "*" 3.39368507185078e-07 4.59033880354872e-07 78.125
"chr17" 865001 866000 "*" 2.62127575090254e-07 3.59814753919398e-07
-66.6666666666667
"chr17" 882001 883000 "*" 0 0 -53.6043533930858
"chr17" 883001 884000 "*" 0 0 55.6451612903226
"chr17" 978001 979000 "*" 1.67421632113474e-13 5.36608155184961e-13
58.6206896551724
"chr17" 984001 985000 "*" 1.58749557943594e-08 2.58908887422551e-08
-58.3333333333333
"chr17" 1012001 1013000 "*" 0 0 -71.8181818181818

"chr17" 1014001 1015000 "*" 1.88737914186277e-15 7.62011598380321e-15
-85.3658536585366
"chr17" 1048001 1049000 "*" 1.10467190950203e-13 3.61721268846938e-13
-57.8947368421053
"chr17" 1074001 1075000 "*" 1.69468217237068e-10 3.66482586521377e-10 -78.75
"chr17" 1132001 1133000 "*" 0 0 52.8504424985133
"chr17" 1154001 1155000 "*" 1.11288755988426e-12 3.21896365898773e-12
-51.5151515151515
"chr17" 1157001 1158000 "*" 0 0 -71.4285714285714
"chr17" 1187001 1188000 "*" 0 0 84.393063583815
"chr17" 1200001 1201000 "*" 2.74846672088103e-08 4.3268595828283e-08 -52
"chr17" 1217001 1218000 "*" 0 0 -100
"chr17" 1219001 1220000 "*" 3.1696723024055e-10 6.59431448778094e-10
-70.5882352941177
"chr17" 1342001 1343000 "*" 1.47501282299345e-08 2.41569981692117e-08 -78.125
"chr17" 1388001 1389000 "*" 0 0 -92.1458625525947
"chr17" 1389001 1390000 "*" 0 0 -57.1676827053637
"chr17" 1391001 1392000 "*" 2.45137243837235e-13 7.68739123491479e-13
-77.9369627507163
"chr17" 1470001 1471000 "*" 0 0 -80.7692307692308
"chr17" 1546001 1547000 "*" 0 0 62.992125984252
"chr17" 1577001 1578000 "*" 5.55111512312578e-16 2.36485094870365e-15
-64.7619047619048
"chr17" 1585001 1586000 "*" 2.88779000712225e-11 6.92935809466068e-11 100
"chr17" 1653001 1654000 "*" 1.70588222436763e-08 2.76390027120887e-08 87.5
"chr17" 1656001 1657000 "*" 0 0 -96.7741935483871
"chr17" 1665001 1666000 "*" 1.11943232461442e-10 2.48331803872384e-10
-55.8118899733807
"chr17" 1819001 1820000 "*" 0 0 64.6464646464646
"chr17" 1872001 1873000 "*" 4.05746613862945e-07 5.42648338506479e-07
-51.010101010101
"chr17" 1892001 1893000 "*" 0 0 -100
"chr17" 1899001 1900000 "*" 7.67056418382595e-11 1.7359448580318e-10 -100
"chr17" 1915001 1916000 "*" 5.55111512312578e-16 2.36485094870365e-15
-72.4137931034483
"chr17" 1928001 1929000 "*" 0 0 53.07237573308
"chr17" 1957001 1958000 "*" 0 0 50.1118298372527
"chr17" 1970001 1971000 "*" 0 0 -100
"chr17" 2297001 2298000 "*" 0 0 -57.6970825574178
"chr17" 2320001 2321000 "*" 0 0 -100
"chr17" 2390001 2391000 "*" 2.15125472990962e-10 4.58126659943874e-10 100
"chr17" 2398001 2399000 "*" 7.7715611723761e-16 3.26213507634405e-15 100
"chr17" 2434001 2435000 "*" 5.62577462392966e-09 9.78995794829878e-09
52.9411764705882
"chr17" 2486001 2487000 "*" 2.48184182993771e-06 2.97686444206053e-06 -90
"chr17" 2638001 2639000 "*" 3.95353005888666e-09 7.03928529283733e-09 100
"chr17" 2669001 2670000 "*" 7.66732899393219e-10 1.50965716509256e-09
81.7204301075269
"chr17" 2679001 2680000 "*" 0 0 66.5933259992666
"chr17" 2680001 2681000 "*" 0 0 75.5309325946445
"chr17" 2694001 2695000 "*" 1.11022302462516e-16 5.03662826618488e-16
83.3333333333333

"chr17" 2708001 2709000 "*" 0 0 -100
"chr17" 2731001 2732000 "*" 0 0 -86.5079365079365
"chr17" 2751001 2752000 "*" 0 0 100
"chr17" 2822001 2823000 "*" 2.07063230461824e-06 2.51448299149857e-06 55.5555555555556
"chr17" 2834001 2835000 "*" 2.88779000712225e-11 6.92935809466068e-11 100
"chr17" 2838001 2839000 "*" 0 0 55.2631578947368
"chr17" 2863001 2864000 "*" 0 0 83.3333333333333
"chr17" 2883001 2884000 "*" 1.11022302462516e-16 5.03662826618488e-16 100
"chr17" 2935001 2936000 "*" 6.93311584809564e-08 1.03197650079882e-07 52.1739130434783
"chr17" 2952001 2953000 "*" 0 0 70.0854700854701
"chr17" 3057001 3058000 "*" 5.04263297784746e-13 1.52078941532818e-12 -100
"chr17" 3065001 3066000 "*" 1.4432899320127e-15 5.90750815956055e-15 100
"chr17" 3131001 3132000 "*" 0 0 -80.3571428571429
"chr17" 3281001 3282000 "*" 4.01313771103418e-09 7.11882087237587e-09 100
"chr17" 3419001 3420000 "*" 7.33857419277228e-14 2.45681680491746e-13 -84.2105263157895
"chr17" 3565001 3566000 "*" 0 0 -100
"chr17" 3570001 3571000 "*" 8.25450818808804e-13 2.42473843346228e-12 -100
"chr17" 3604001 3605000 "*" 5.6362137179633e-11 1.29963270300065e-10 -100
"chr17" 3642001 3643000 "*" 3.61932706027801e-14 1.25777907157994e-13 -100
"chr17" 3654001 3655000 "*" 0 0 67.1428571428571
"chr17" 3680001 3681000 "*" 2.55351295663786e-15 1.01678090381833e-14 -100
"chr17" 3689001 3690000 "*" 1.87405646556726e-13 5.97285292681429e-13 100
"chr17" 3748001 3749000 "*" 8.02931499066517e-10 1.57731825794538e-09 -71.7948717948718
"chr17" 3768001 3769000 "*" 0 0 -77.1739130434783
"chr17" 3782001 3783000 "*" 4.44089209850063e-16 1.91071758245033e-15 -66.6666666666667
"chr17" 3790001 3791000 "*" 0 0 58.3333333333333
"chr17" 3791001 3792000 "*" 0 0 -77.4891774891775
"chr17" 3794001 3795000 "*" 1.11022302462516e-16 5.03662826618488e-16 -100
"chr17" 3811001 3812000 "*" 1.11022302462516e-16 5.03662826618488e-16 -85
"chr17" 3812001 3813000 "*" 3.95750099357883e-12 1.0684496262032e-11 -59.1135232926278
"chr17" 3816001 3817000 "*" 2.56182366770163e-05 2.63622194064996e-05 -56.25
"chr17" 3829001 3830000 "*" 0 0 100
"chr17" 3836001 3837000 "*" 1.11022302462516e-16 5.03662826618488e-16 100
"chr17" 3905001 3906000 "*" 8.7349629751543e-09 1.47505871898511e-08 100
"chr17" 4051001 4052000 "*" 5.21493959126929e-12 1.38371594733623e-11 -75
"chr17" 4057001 4058000 "*" 1.76436462107077e-08 2.85377575210863e-08 -63.6363636363636
"chr17" 4151001 4152000 "*" 9.63382706942184e-11 2.15239597116267e-10 -67.5675675675676
"chr17" 4163001 4164000 "*" 1.09716713757591e-05 1.19480980750523e-05 -62.2222222222222
"chr17" 4212001 4213000 "*" 0 0 100
"chr17" 4316001 4317000 "*" 1.11022302462516e-16 5.03662826618488e-16 100
"chr17" 4343001 4344000 "*" 1.79709469527722e-10 3.87734963411371e-10 -91.6666666666667
"chr17" 4347001 4348000 "*" 3.92685883809918e-13 1.19951200966592e-12 -100

"chr17" 4383001 4384000 "*" 0 0 100
"chr17" 4384001 4385000 "*" 0 0 100
"chr17" 4387001 4388000 "*" 3.95353005888666e-09 7.03928529283733e-09 -100
"chr17" 4403001 4404000 "*" 0 0 74.3405275779376
"chr17" 4487001 4488000 "*" 0 0 61.3967462162009
"chr17" 4495001 4496000 "*" 1.7430501486615e-14 6.29150858008753e-14
-74.0963855421687
"chr17" 4506001 4507000 "*" 6.24654217240561e-10 1.2462108191303e-09 -100
"chr17" 4614001 4615000 "*" 0 0 -100
"chr17" 4641001 4642000 "*" 0 0 -62.2222222222222
"chr17" 4690001 4691000 "*" 1.17153376945067e-05 1.27015577375004e-05
-52.9411764705882
"chr17" 4692001 4693000 "*" 0 0 73.2139337800552
"chr17" 4696001 4697000 "*" 6.4324989779152e-11 1.47230020825092e-10 -100
"chr17" 4707001 4708000 "*" 0.000525299954949099 0.000436849389802675
54.5454545454545
"chr17" 4753001 4754000 "*" 3.12283532366564e-12 8.52972563422097e-12
-51.7241379310345
"chr17" 4781001 4782000 "*" 0 0 -100
"chr17" 4801001 4802000 "*" 1.07882591748876e-11 2.74553537455803e-11 100
"chr17" 4812001 4813000 "*" 2.88313817264907e-12 7.90426787610106e-12
62.7272727272727
"chr17" 4825001 4826000 "*" 1.96509475358653e-13 6.23868565133654e-13 -100
"chr17" 4842001 4843000 "*" 0 0 -93.75
"chr17" 4848001 4849000 "*" 1.25284005392245e-10 2.75649590272477e-10 100
"chr17" 4935001 4936000 "*" 0 0 -100
"chr17" 5015001 5016000 "*" 0 0 -100
"chr17" 5095001 5096000 "*" 0 0 81.083725653097
"chr17" 5131001 5132000 "*" 4.10560474506383e-13 1.25200298811809e-12
95.8333333333333
"chr17" 5161001 5162000 "*" 3.68371999570627e-13 1.13149976091305e-12 -100
"chr17" 5176001 5177000 "*" 1.59474655703207e-11 3.96585783899868e-11 -75
"chr17" 5415001 5416000 "*" 1.99840144432528e-12 5.59476580335607e-12 -100
"chr17" 5424001 5425000 "*" 5.99234319376141e-06 6.79020205076211e-06
65.7894736842105
"chr17" 5485001 5486000 "*" 9.65729496371637e-10 1.87255912493232e-09 100
"chr17" 5547001 5548000 "*" 3.52704532247117e-11 8.35581292290093e-11 100
"chr17" 5591001 5592000 "*" 2.69007038866675e-13 8.38831022628993e-13 100
"chr17" 5606001 5607000 "*" 0 0 -69.4117647058823
"chr17" 5684001 5685000 "*" 3.75377506856012e-12 1.01651879013373e-11
-71.1111111111111
"chr17" 5693001 5694000 "*" 4.95129315325471e-09 8.6712296812971e-09
65.2173913043478
"chr17" 5701001 5702000 "*" 2.88710388929303e-10 6.03399264731139e-10 -100
"chr17" 5726001 5727000 "*" 0 0 100
"chr17" 5775001 5776000 "*" 6.37490060739765e-13 1.89496793587877e-12 100
"chr17" 5917001 5918000 "*" 9.08108033215171e-11 2.03407753381119e-10 -100
"chr17" 5944001 5945000 "*" 0 0 -72.6114649681529
"chr17" 5945001 5946000 "*" 8.52140580320793e-12 2.19817259951489e-11 100
"chr17" 6035001 6036000 "*" 2.28981344996271e-08 3.63818631112906e-08
60.7142857142857
"chr17" 6070001 6071000 "*" 8.69011131499065e-07 1.11229659818976e-06

-57.6923076923077
"chr17" 6111001 6112000 "*" 2.04184160813581e-05 2.1337586860998e-05
-63.2911392405063
"chr17" 6143001 6144000 "*" 2.37587727269783e-14 8.44268514944447e-14 100
"chr17" 6188001 6189000 "*" 6.92287338566189e-11 1.57605630124247e-10 100
"chr17" 6221001 6222000 "*" 0 0 100
"chr17" 6325001 6326000 "*" 5.55111512312578e-16 2.36485094870365e-15
-76.0869565217391
"chr17" 6330001 6331000 "*" 0 0 100
"chr17" 6383001 6384000 "*" 4.79616346638068e-14 1.64380445416596e-13 -100
"chr17" 6427001 6428000 "*" 0 0 86.3636363636364
"chr17" 6456001 6457000 "*" 0 0 100
"chr17" 6555001 6556000 "*" 0 0 66.560165601656
"chr17" 6572001 6573000 "*" 4.44089209850063e-16 1.91071758245033e-15 100
"chr17" 6593001 6594000 "*" 4.88203187765102e-07 6.46385771339354e-07
69.2307692307692
"chr17" 6610001 6611000 "*" 1.11022302462516e-16 5.03662826618488e-16
-62.8571428571429
"chr17" 6639001 6640000 "*" 2.88657986402541e-15 1.14155613124573e-14
70.8333333333333
"chr17" 6817001 6818000 "*" 9.54791801177635e-14 3.14918538634687e-13
91.6417910447761
"chr17" 6822001 6823000 "*" 6.59550958292954e-09 1.13456194382773e-08 -100
"chr17" 6969001 6970000 "*" 2.05391259555654e-14 7.35435952190685e-14 100
"chr17" 6980001 6981000 "*" 0 0 100
"chr17" 7005001 7006000 "*" 0 0 -85.5072463768116
"chr17" 7027001 7028000 "*" 3.6700841921089e-08 5.65530226072258e-08 100
"chr17" 7083001 7084000 "*" 2.18626894366025e-10 4.65191227840769e-10
53.8803240135877
"chr17" 7117001 7118000 "*" 1.21036112021855e-07 1.73659787041099e-07
-78.7037037037037
"chr17" 7125001 7126000 "*" 5.1236792586451e-13 1.54417092913736e-12
-58.5714285714286
"chr17" 7200001 7201000 "*" 0 0 -100
"chr17" 7247001 7248000 "*" 0.000298482767503438 0.000258818394743619
51.5151515151515
"chr17" 7252001 7253000 "*" 0 0 100
"chr17" 7253001 7254000 "*" 0 0 -54.5741551266413
"chr17" 7284001 7285000 "*" 0 0 -67.965367965368
"chr17" 7298001 7299000 "*" 0 0 -75.8333333333333
"chr17" 7339001 7340000 "*" 0 0 -50.4859335038363
"chr17" 7342001 7343000 "*" 0 0 56.3847235238988
"chr17" 7346001 7347000 "*" 9.63829016598083e-12 2.46993429847588e-11 -100
"chr17" 7349001 7350000 "*" 0 0 -80
"chr17" 7359001 7360000 "*" 0 0 86.6141732283465
"chr17" 7451001 7452000 "*" 9.43689570931383e-15 3.51625644188498e-14 100
"chr17" 7485001 7486000 "*" 5.65418284215191e-06 6.4320100371832e-06
54.5454545454545
"chr17" 7528001 7529000 "*" 7.105427357601e-15 2.68362283258525e-14 59.375
"chr17" 7539001 7540000 "*" 2.88431064698891e-07 3.93691762673522e-07
56.6037735849057
"chr17" 7558001 7559000 "*" 1.47471479472472e-09 2.79172511602426e-09

-51.3129440840284
"chr17" 7599001 7600000 "*" 2.88779000712225e-11 6.92935809466068e-11 -100
"chr17" 7608001 7609000 "*" 0 0 90.0069881201957
"chr17" 7611001 7612000 "*" 2.78987943858056e-12 7.66471949267203e-12
-73.7704918032787
"chr17" 7656001 7657000 "*" 1.51656465163796e-13 4.87803420672651e-13
-51.4285714285714
"chr17" 7674001 7675000 "*" 0.000240479789304304 0.000211878237500588
-57.1428571428571
"chr17" 7680001 7681000 "*" 0 0 100
"chr17" 7697001 7698000 "*" 8.88178419700125e-16 3.70670032207938e-15
-56.3385826771653
"chr17" 7700001 7701000 "*" 1.5277158427196e-09 2.87642564232045e-09 -100
"chr17" 7703001 7704000 "*" 2.55351295663786e-15 1.01678090381833e-14 100
"chr17" 7761001 7762000 "*" 7.70383756787396e-13 2.27347023283685e-12
50.8379888268156
"chr17" 7789001 7790000 "*" 0 0 -86.685552407932
"chr17" 7791001 7792000 "*" 0 0 100
"chr17" 7825001 7826000 "*" 0 0 100
"chr17" 7836001 7837000 "*" 0 0 100
"chr17" 7893001 7894000 "*" 2.22044604925031e-16 9.81641919380259e-16
53.7084398976982
"chr17" 7895001 7896000 "*" 3.25818815749557e-05 3.29735679006858e-05
-66.6666666666667
"chr17" 7915001 7916000 "*" 0 0 -94.3548387096774
"chr17" 7960001 7961000 "*" 5.50265094378943e-07 7.23847575893666e-07
63.4408602150538
"chr17" 7967001 7968000 "*" 4.50750547997814e-14 1.55028701647451e-13
-84.3137254901961
"chr17" 7991001 7992000 "*" 1.67299207820548e-08 2.71377429441055e-08 100
"chr17" 7994001 7995000 "*" 0.000241621869001674 0.000212792315955059
-57.6923076923077
"chr17" 8011001 8012000 "*" 1.43850042988447e-10 3.13701334702367e-10
91.1764705882353
"chr17" 8021001 8022000 "*" 0 0 -56.4596511405022
"chr17" 8027001 8028000 "*" 3.97170748256404e-06 4.61946241884362e-06
66.0714285714286
"chr17" 8028001 8029000 "*" 0 0 58.5526315789474
"chr17" 8043001 8044000 "*" 0 0 90
"chr17" 8056001 8057000 "*" 0 0 100
"chr17" 8059001 8060000 "*" 2.66453525910038e-15 1.05861327033776e-14
-69.8082698082698
"chr17" 8159001 8160000 "*" 4.78417305771472e-12 1.27644316016844e-11
-64.2857142857143
"chr17" 8165001 8166000 "*" 2.00227675550835e-08 3.20459176617961e-08 -100
"chr17" 8198001 8199000 "*" 4.0001140511059e-08 6.13867257609592e-08
56.3758389261745
"chr17" 8279001 8280000 "*" 6.94600703177173e-07 9.01090120559002e-07
-69.7368421052632
"chr17" 8300001 8301000 "*" 0 0 -59.5455533811698
"chr17" 8328001 8329000 "*" 0 0 100
"chr17" 8368001 8369000 "*" 1.67299207820548e-08 2.71377429441055e-08 -100

"chr17" 8404001 8405000 "*" 0 0 100
"chr17" 8448001 8449000 "*" 9.19692810130712e-07 1.17343911762874e-06 70
"chr17" 8494001 8495000 "*" 3.40125705378114e-10 7.03784073980162e-10 100
"chr17" 8534001 8535000 "*" 0 0 78.6100897190763
"chr17" 8552001 8553000 "*" 1.96509475358653e-13 6.23868565133654e-13 100
"chr17" 8553001 8554000 "*" 0 0 100
"chr17" 8645001 8646000 "*" 2.67621480531943e-10 5.63603302874254e-10 72
"chr17" 8658001 8659000 "*" 4.21551682450172e-13 1.28144423841277e-12
-61.500754147813
"chr17" 8659001 8660000 "*" 4.88798519771017e-07 6.47132903034509e-07
-82.7586206896552
"chr17" 8725001 8726000 "*" 6.77335965093562e-12 1.76897011246462e-11 100
"chr17" 8738001 8739000 "*" 0 0 78.1818181818182
"chr17" 8770001 8771000 "*" 0 0 -53.2467532467532
"chr17" 8798001 8799000 "*" 4.63299953956664e-10 9.43002783795344e-10
-53.5714285714286
"chr17" 8931001 8932000 "*" 0.00063975764960944 0.00052432375414322
52.9411764705882
"chr17" 8959001 8960000 "*" 3.6700841921089e-08 5.65530226072258e-08 100
"chr17" 8974001 8975000 "*" 7.0006535457523e-08 1.03695832512054e-07 100
"chr17" 8985001 8986000 "*" 0 0 58.3333333333333
"chr17" 8994001 8995000 "*" 2.16382467499443e-13 6.82775854166559e-13 100
"chr17" 9019001 9020000 "*" 0 0 96.640826873385
"chr17" 9031001 9032000 "*" 9.08108033215171e-11 2.03407753381119e-10 100
"chr17" 9032001 9033000 "*" 0 0 100
"chr17" 9046001 9047000 "*" 0 0 100
"chr17" 9057001 9058000 "*" 1.42307277073428e-11 3.56166194273734e-11
73.1707317073171
"chr17" 9064001 9065000 "*" 0.000337498912639123 0.000290065278296537
-50.7246376811594
"chr17" 9070001 9071000 "*" 1.11022302462516e-16 5.03662826618488e-16 100
"chr17" 9141001 9142000 "*" 6.88338275267597e-15 2.60518659957509e-14 100
"chr17" 9175001 9176000 "*" 2.8421709430404e-14 9.99616402633955e-14 -100
"chr17" 9178001 9179000 "*" 1.12458486967171e-09 2.15740815043925e-09 -100
"chr17" 9227001 9228000 "*" 3.03090885722668e-14 1.06210046699299e-13 100
"chr17" 9479001 9480000 "*" 0 0 -70.5882352941177
"chr17" 9573001 9574000 "*" 1.14124265593318e-11 2.89138062669327e-11 -100
"chr17" 9674001 9675000 "*" 0 0 68.75
"chr17" 9705001 9706000 "*" 1.92510479779529e-08 3.09892070789102e-08
-51.6569200779727
"chr17" 9725001 9726000 "*" 5.42899059041702e-14 1.84507579088307e-13
57.2916666666667
"chr17" 9747001 9748000 "*" 0 0 81.6091954022989
"chr17" 9809001 9810000 "*" 1.39779633423487e-07 1.98463037958589e-07 -100
"chr17" 9810001 9811000 "*" 1.55133461632317e-09 2.91855334268503e-09
-80.4347826086957
"chr17" 9818001 9819000 "*" 1.12458486967171e-09 2.15740815043925e-09 100
"chr17" 9828001 9829000 "*" 1.11022302462516e-16 5.03662826618488e-16
-50.8620689655172
"chr17" 9861001 9862000 "*" 6.24654217240561e-10 1.2462108191303e-09 -100
"chr17" 9862001 9863000 "*" 2.46469511466785e-14 8.7281767390389e-14
-79.3103448275862

"chr17" 9870001 9871000 "*" 0 0 79.5918367346939
"chr17" 9880001 9881000 "*" 6.59550958292954e-09 1.13456194382773e-08 100
"chr17" 9956001 9957000 "*" 0 0 100
"chr17" 9965001 9966000 "*" 1.11022302462516e-16 5.03662826618488e-16 100
"chr17" 9998001 9999000 "*" 0 0 100
"chr17" 10013001 10014000 "*" 1.12055920098442e-11 2.84500040664577e-11 100
"chr17" 10027001 10028000 "*" 9.94782034524633e-12 2.54589158623688e-11 58.7719298245614
"chr17" 10100001 10101000 "*" 4.08645228167082e-06 4.7426694371739e-06 -70.4918032786885
"chr17" 10102001 10103000 "*" 0 0 62.8469305692074
"chr17" 10178001 10179000 "*" 6.4324989779152e-11 1.47230020825092e-10 100
"chr17" 10180001 10181000 "*" 4.99787471408553e-07 6.6062228116197e-07 -52.962962962963
"chr17" 10209001 10210000 "*" 3.77475828372553e-15 1.47379557022071e-14 100
"chr17" 10222001 10223000 "*" 1.85311495009e-05 1.9493842032551e-05 -57.7464788732394
"chr17" 10243001 10244000 "*" 2.70339306496226e-13 8.42355472617862e-13 100
"chr17" 10301001 10302000 "*" 3.02636461446681e-08 4.7418824327602e-08 77.1084337349398
"chr17" 10549001 10550000 "*" 3.60169446167458e-06 4.21798537352463e-06 69
"chr17" 10643001 10644000 "*" 4.6812846129507e-06 5.38800552240891e-06 52.1739130434783
"chr17" 10710001 10711000 "*" 1.43218770176645e-14 5.21983361092297e-14 100
"chr17" 10740001 10741000 "*" 7.88258347483861e-15 2.96145301916012e-14 -91.812865497076
"chr17" 10744001 10745000 "*" 2.88710388929303e-10 6.03399264731139e-10 -100
"chr17" 11175001 11176000 "*" 1.48487888651516e-11 3.70689457767456e-11 100
"chr17" 11637001 11638000 "*" 2.47944188014326e-06 2.97424262282235e-06 73.0769230769231
"chr17" 11772001 11773000 "*" 1.43631318505477e-07 2.03671717589617e-07 79.7297297297297
"chr17" 12225001 12226000 "*" 8.10964051467522e-09 1.37881408088721e-08 -77.2727272727273
"chr17" 12449001 12450000 "*" 1.14124265593318e-11 2.89138062669327e-11 -100
"chr17" 12526001 12527000 "*" 1.19451015745398e-09 2.28482591782588e-09 -52.3809523809524
"chr17" 12569001 12570000 "*" 1.58024170415771e-08 2.57781284488e-08 80.2424242424242
"chr17" 12777001 12778000 "*" 7.21644966006352e-15 2.72345776413425e-14 100
"chr17" 12779001 12780000 "*" 5.794809077031e-12 1.52911192757929e-11 100
"chr17" 12826001 12827000 "*" 1.93720595120794e-11 4.76309924836636e-11 100
"chr17" 12850001 12851000 "*" 2.00227675550835e-08 3.20459176617961e-08 -100
"chr17" 12860001 12861000 "*" 1.1483329143136e-07 1.65266213593465e-07 -54.1666666666667
"chr17" 12903001 12904000 "*" 6.92287338566189e-11 1.57605630124247e-10 -100
"chr17" 13458001 13459000 "*" 1.98365768255826e-11 4.85612402473442e-11 100
"chr17" 13477001 13478000 "*" 1.4432899320127e-15 5.90750815956055e-15 100
"chr17" 13569001 13570000 "*" 1.77635683940025e-13 5.67970397457708e-13 62.1645796064401
"chr17" 13678001 13679000 "*" 8.7349629751543e-09 1.47505871898511e-08 100
"chr17" 13874001 13875000 "*" 1.11022302462516e-16 5.03662826618488e-16 100

"chr17" 13903001 13904000 "*" 3.04324343503026e-11 7.26599935673121e-11 100
"chr17" 14144001 14145000 "*" 0 0 -100
"chr17" 14158001 14159000 "*" 7.93809462606987e-14 2.64679416473686e-13 100
"chr17" 14200001 14201000 "*" 0 0 69.6161976483023
"chr17" 14204001 14205000 "*" 0 0 62.4789170191535
"chr17" 14214001 14215000 "*" 8.88178419700125e-16 3.70670032207938e-15 -100
"chr17" 14338001 14339000 "*" 1.91224813761437e-12 5.36527594469447e-12 100
"chr17" 14374001 14375000 "*" 1.25284005392245e-10 2.75649590272477e-10 100
"chr17" 14936001 14937000 "*" 6.4152538836737e-10 1.27477697728417e-09 100
"chr17" 15157001 15158000 "*" 1.0212497514317e-11 2.61074488258745e-11
85.8490566037736
"chr17" 15181001 15182000 "*" 6.59550958292954e-09 1.13456194382773e-08 100
"chr17" 15193001 15194000 "*" 2.22044604925031e-16 9.81641919380259e-16 100
"chr17" 15207001 15208000 "*" 2.68673971959288e-14 9.47266815431045e-14 100
"chr17" 15220001 15221000 "*" 2.00227675550835e-08 3.20459176617961e-08 100
"chr17" 15245001 15246000 "*" 6.66133814775094e-16 2.81595744474255e-15
59.4155844155844
"chr17" 15306001 15307000 "*" 8.77076189453874e-15 3.27889038038365e-14 -100
"chr17" 15308001 15309000 "*" 0 0 82.5396825396825
"chr17" 15673001 15674000 "*" 0 0 66.6666666666667
"chr17" 15686001 15687000 "*" 0 0 51.8627450980392
"chr17" 15689001 15690000 "*" 0 0 53.8717010971632
"chr17" 15720001 15721000 "*" 1.69354419377044e-10 3.66245990170978e-10
53.763440860215
"chr17" 15812001 15813000 "*" 5.6621374255883e-15 2.16468580166064e-14 100
"chr17" 15845001 15846000 "*" 3.76365605347928e-14 1.3043487970679e-13 100
"chr17" 16314001 16315000 "*" 1.98124849859482e-10 4.25157611354621e-10 100
"chr17" 16368001 16369000 "*" 1.22668426605355e-06 1.53860290181864e-06
-53.6645962732919
"chr17" 16394001 16395000 "*" 1.54726781986803e-06 1.91404818487039e-06
-69.7674418604651
"chr17" 16520001 16521000 "*" 6.32827124036339e-15 2.40666772484995e-14 100
"chr17" 16773001 16774000 "*" 1.55431223447522e-15 6.33705141101682e-15 -58
"chr17" 16785001 16786000 "*" 3.6700841921089e-08 5.65530226072258e-08 -100
"chr17" 16799001 16800000 "*" 3.29762030015779e-07 4.4664129931678e-07
57.1428571428571
"chr17" 16800001 16801000 "*" 5.63660229602192e-13 1.68905058935478e-12 100
"chr17" 16820001 16821000 "*" 4.31678991719142e-06 4.99131135124808e-06
69.2307692307692
"chr17" 16826001 16827000 "*" 1.44236241883533e-06 1.79195636844577e-06 56.25
"chr17" 16835001 16836000 "*" 0 0 100
"chr17" 16842001 16843000 "*" 2.79440914852103e-11 6.73046092948135e-11 100
"chr17" 16868001 16869000 "*" 0 0 100
"chr17" 16894001 16895000 "*" 0 0 -65.9685863874346
"chr17" 16931001 16932000 "*" 9.0072393987839e-13 2.63157725646847e-12 100
"chr17" 16935001 16936000 "*" 5.46229728115577e-14 1.85594149883513e-13
-56.6371681415929
"chr17" 17040001 17041000 "*" 1.67299207820548e-08 2.71377429441055e-08 100
"chr17" 17109001 17110000 "*" 0 0 -70.6292438034993
"chr17" 17193001 17194000 "*" 1.13140401492018e-08 1.87801380787728e-08 -100
"chr17" 17217001 17218000 "*" 1.37828859436695e-10 3.01445544267661e-10 -100
"chr17" 17225001 17226000 "*" 0 0 56.6080384262202

"chr17" 17372001 17373000 "*" 3.76365605347928e-14 1.3043487970679e-13 100
"chr17" 17400001 17401000 "*" 0 0 62.5
"chr17" 17437001 17438000 "*" 2.18158824338843e-13 6.87314823207509e-13 -100
"chr17" 17441001 17442000 "*" 4.88498130835069e-15 1.8824139250584e-14 100
"chr17" 17484001 17485000 "*" 1.5277158427196e-09 2.87642564232045e-09 -100
"chr17" 17503001 17504000 "*" 5.55111512312578e-16 2.36485094870365e-15 81.25
"chr17" 17567001 17568000 "*" 6.7390537594747e-14 2.26447131529591e-13 -100
"chr17" 17589001 17590000 "*" 3.35287353436797e-14 1.1695189862716e-13
-58.0582524271845
"chr17" 17594001 17595000 "*" 4.77395900588817e-15 1.84201484337652e-14
66.6666666666667
"chr17" 17597001 17598000 "*" 9.23447760370344e-07 1.17794173977535e-06
-60.8695652173913
"chr17" 17602001 17603000 "*" 8.39328606616618e-14 2.79009090750649e-13 84
"chr17" 17614001 17615000 "*" 2.69836322177586e-05 2.76764322277499e-05
53.4246575342466
"chr17" 17655001 17656000 "*" 0 0 -68.6439061605951
"chr17" 17682001 17683000 "*" 1.72816431639466e-05 1.82764748691336e-05
-52.5220680958386
"chr17" 17709001 17710000 "*" 0 0 99.290780141844
"chr17" 17786001 17787000 "*" 8.49470134567376e-07 1.08878884380982e-06
-55.8823529411765
"chr17" 17839001 17840000 "*" 8.52140580320793e-12 2.19817259951489e-11 100
"chr17" 17902001 17903000 "*" 1.22124532708767e-15 5.03921984217778e-15 100
"chr17" 17974001 17975000 "*" 3.63445940010365e-11 8.58565209111204e-11 100
"chr17" 18006001 18007000 "*" 2.22044604925031e-16 9.81641919380259e-16 100
"chr17" 18031001 18032000 "*" 0 0 76.9230769230769
"chr17" 18057001 18058000 "*" 6.88338275267597e-15 2.60518659957509e-14 100
"chr17" 18085001 18086000 "*" 0 0 62.5
"chr17" 18087001 18088000 "*" 0 0 79.6583850931677
"chr17" 18106001 18107000 "*" 1.65900401183361e-06 2.04236557510993e-06
66.6666666666667
"chr17" 18170001 18171000 "*" 1.13140401492018e-08 1.87801380787728e-08 100
"chr17" 18171001 18172000 "*" 9.01894892102462e-10 1.76197382512895e-09
53.8461538461538
"chr17" 18178001 18179000 "*" 6.59550958292954e-09 1.13456194382773e-08 -100
"chr17" 18230001 18231000 "*" 2.22044604925031e-16 9.81641919380259e-16 -100
"chr17" 18764001 18765000 "*" 0 0 -100
"chr17" 18836001 18837000 "*" 3.90796963123385e-07 5.23839005739906e-07
65.7894736842105
"chr17" 18901001 18902000 "*" 1.39779633423487e-07 1.98463037958589e-07 -100
"chr17" 19035001 19036000 "*" 2.64951482975562e-09 4.82186011375752e-09 100
"chr17" 19152001 19153000 "*" 1.77191594730175e-13 5.66680822826469e-13
-64.7058823529412
"chr17" 19211001 19212000 "*" 3.17946891037479e-10 6.613375975627e-10
-53.4584980237154
"chr17" 19230001 19231000 "*" 0 0 -100
"chr17" 19323001 19324000 "*" 6.95591910737647e-08 1.0351489064112e-07
-80.4123711340206
"chr17" 19391001 19392000 "*" 2.7649948897146e-07 3.78440142643551e-07 75
"chr17" 19409001 19410000 "*" 8.52140580320793e-12 2.19817259951489e-11 -100
"chr17" 19427001 19428000 "*" 3.6700841921089e-08 5.65530226072258e-08 -100

"chr17" 19437001 19438000 "*" 0 0 60.6467597451543
"chr17" 19551001 19552000 "*" 0 0 -100
"chr17" 19640001 19641000 "*" 5.78426195829707e-14 1.95807719599496e-13
59.6491228070175
"chr17" 19669001 19670000 "*" 1.13140401492018e-08 1.87801380787728e-08 100
"chr17" 19776001 19777000 "*" 4.78841410966879e-11 1.11468187156588e-10 100
"chr17" 20539001 20540000 "*" 6.93112234273485e-13 2.05218842007357e-12 -100
"chr17" 20659001 20660000 "*" 1.61237689866311e-12 4.56615813671789e-12 100
"chr17" 20717001 20718000 "*" 1.11022302462516e-16 5.03662826618488e-16 100
"chr17" 20747001 20748000 "*" 0 0 66.4376145260703
"chr17" 20755001 20756000 "*" 0 0 58.588850174216
"chr17" 20929001 20930000 "*" 1.13140401492018e-08 1.87801380787728e-08 -100
"chr17" 20960001 20961000 "*" 2.08814465718632e-09 3.86232567960463e-09 -100
"chr17" 20999001 21000000 "*" 7.0006535457523e-08 1.03695832512054e-07 -100
"chr17" 21183001 21184000 "*" 7.7715611723761e-15 2.92461699448967e-14
65.8119658119658
"chr17" 21210001 21211000 "*" 3.6437852735105e-11 8.60646364822127e-11
-88.4057971014493
"chr17" 21222001 21223000 "*" 4.99900121297969e-12 1.32921056339624e-11 -100
"chr17" 21223001 21224000 "*" 1.14124265593318e-11 2.89138062669327e-11 100
"chr17" 21250001 21251000 "*" 0.000726374156166165 0.000590081342527627
-52.1739130434783
"chr17" 21251001 21252000 "*" 1.54876003133353e-08 2.53011960456056e-08
83.3333333333333
"chr17" 21252001 21253000 "*" 4.05497857514092e-12 1.0912459725854e-11
-53.2558139534884
"chr17" 21284001 21285000 "*" 8.8982377022262e-10 1.73940988202906e-09
62.0689655172414
"chr17" 21292001 21293000 "*" 4.18887147191072e-13 1.2739946038722e-12 100
"chr17" 21337001 21338000 "*" 0.000188913003384639 0.000169331787295583
54.5454545454545
"chr17" 21357001 21358000 "*" 1.22124532708767e-15 5.03921984217778e-15 -100
"chr17" 21359001 21360000 "*" 2.77555756156289e-15 1.10015415195978e-14 -100
"chr17" 21361001 21362000 "*" 1.00522586921059e-08 1.68522429251694e-08
76.2886597938144
"chr17" 21425001 21426000 "*" 9.27575020126525e-08 1.35148028825722e-07
73.6842105263158
"chr17" 21427001 21428000 "*" 6.63625931984768e-10 1.31621558275135e-09 68
"chr17" 21438001 21439000 "*" 6.52733422867868e-12 1.71146951718423e-11 -100
"chr17" 21454001 21455000 "*" 0 0 90.1098901098901
"chr17" 21482001 21483000 "*" 3.88022947106492e-13 1.1867462770515e-12 100
"chr17" 21514001 21515000 "*" 0 0 100
"chr17" 21517001 21518000 "*" 3.79989023580052e-08 5.84571032914815e-08
-76.9662921348315
"chr17" 21558001 21559000 "*" 2.21876961248313e-11 5.40646075017609e-11
92.8571428571429
"chr17" 21826001 21827000 "*" 0 0 100
"chr17" 21848001 21849000 "*" 9.95295942662899e-07 1.26313797719273e-06
-71.4285714285714
"chr17" 22068001 22069000 "*" 0 0 100
"chr17" 25263001 25264000 "*" 2.55351295663786e-15 1.01678090381833e-14
-70.5882352941177

"chr17" 25322001 25323000 "*" 5.27068596545277e-08 7.96829356899468e-08
76.5957446808511
"chr17" 25417001 25418000 "*" 4.00564319436025e-06 4.65588073919783e-06
-65.2173913043478
"chr17" 25461001 25462000 "*" 1.11022302462516e-15 4.59817122935606e-15 100
"chr17" 25656001 25657000 "*" 2.79440914852103e-11 6.73046092948135e-11 100
"chr17" 25681001 25682000 "*" 0 0 -100
"chr17" 25775001 25776000 "*" 0 0 -100
"chr17" 25782001 25783000 "*" 0 0 87.0588235294118
"chr17" 25788001 25789000 "*" 9.2148511043888e-15 3.43705279328267e-14
61.6341991341991
"chr17" 25813001 25814000 "*" 0 0 -100
"chr17" 25878001 25879000 "*" 2.64951482975562e-09 4.82186011375752e-09 -100
"chr17" 25937001 25938000 "*" 6.43929354282591e-15 2.44732760419317e-14
-63.4146341463415
"chr17" 25971001 25972000 "*" 0 0 -50.1764705882353
"chr17" 25978001 25979000 "*" 0 0 57.0469798657718
"chr17" 26079001 26080000 "*" 2.94209101525666e-14 1.03319062369232e-13
-82.6923076923077
"chr17" 26080001 26081000 "*" 4.33653113418586e-13 1.31683859101362e-12
-89.5287958115183
"chr17" 26093001 26094000 "*" 1.19904086659517e-14 4.40901388691796e-14 -100
"chr17" 26127001 26128000 "*" 6.59550958292954e-09 1.13456194382773e-08 -100
"chr17" 26173001 26174000 "*" 5.03264097062583e-13 1.51942428521737e-12 -100
"chr17" 26203001 26204000 "*" 0.000120562093188115 0.000111613913217382
-50.9433962264151
"chr17" 26212001 26213000 "*" 0.000496254927290996 0.000414667937798419
-54.1666666666667
"chr17" 26224001 26225000 "*" 7.49519199538806e-06 8.36942869052848e-06
-71.0526315789474
"chr17" 26276001 26277000 "*" 4.73234496034536e-09 8.30627300264826e-09 100
"chr17" 26279001 26280000 "*" 7.90700838138036e-12 2.05001868301528e-11
-55.5555555555556
"chr17" 26311001 26312000 "*" 1.65507296756573e-08 2.69503375826245e-08
-84.7826086956522
"chr17" 26319001 26320000 "*" 2.00227675550835e-08 3.20459176617961e-08 -100
"chr17" 26323001 26324000 "*" 2.00227675550835e-08 3.20459176617961e-08 -100
"chr17" 26561001 26562000 "*" 5.63660229602192e-13 1.68905058935478e-12 -100
"chr17" 26650001 26651000 "*" 1.59227557805508e-08 2.59658003984163e-08
61.5384615384615
"chr17" 26697001 26698000 "*" 3.49194319015922e-08 5.41783859504158e-08
-88.2352941176471
"chr17" 26750001 26751000 "*" 3.7274627828765e-12 1.00985250136599e-11 100
"chr17" 26812001 26813000 "*" 4.79616346638068e-14 1.64380445416596e-13 -100
"chr17" 26820001 26821000 "*" 7.03881397612349e-14 2.35996780481906e-13
-67.741935483871
"chr17" 26822001 26823000 "*" 7.67056418382595e-11 1.7359448580318e-10 100
"chr17" 26943001 26944000 "*" 1.11022302462516e-16 5.03662826618488e-16 -100
"chr17" 27037001 27038000 "*" 9.71063229826541e-11 2.16781365050398e-10 100
"chr17" 27049001 27050000 "*" 0 0 -83.5616438356164
"chr17" 27051001 27052000 "*" 7.67056418382595e-11 1.7359448580318e-10 -100
"chr17" 27058001 27059000 "*" 1.99396055222678e-13 6.3281593586436e-13

58.8235294117647
"chr17" 27131001 27132000 "*" 1.07174135122179e-08 1.79014992015265e-08
52.4324324324324
"chr17" 27210001 27211000 "*" 0 0 66.6666666666667
"chr17" 27218001 27219000 "*" 0 0 -100
"chr17" 27225001 27226000 "*" 0 0 -63.8297872340426
"chr17" 27302001 27303000 "*" 4.01313771103418e-09 7.11882087237587e-09 100
"chr17" 27347001 27348000 "*" 0 0 67.3323092677931
"chr17" 27366001 27367000 "*" 2.00227675550835e-08 3.20459176617961e-08 -100
"chr17" 27423001 27424000 "*" 1.93720595120794e-11 4.76309924836636e-11 -100
"chr17" 27444001 27445000 "*" 8.1845506383349e-07 1.05155704962166e-06
-66.6666666666667
"chr17" 27466001 27467000 "*" 3.46500605985511e-13 1.06777978272295e-12 -100
"chr17" 27484001 27485000 "*" 9.89811471076019e-05 9.28582300971213e-05 65
"chr17" 27523001 27524000 "*" 6.15840711759574e-13 1.83676803572802e-12 100
"chr17" 27551001 27552000 "*" 1.28785870856518e-14 4.71440608755782e-14 -100
"chr17" 27561001 27562000 "*" 5.63660229602192e-13 1.68905058935478e-12 -100
"chr17" 27575001 27576000 "*" 1.88737914186277e-15 7.62011598380321e-15 -100
"chr17" 27612001 27613000 "*" 2.15125472990962e-10 4.58126659943874e-10 -100
"chr17" 27718001 27719000 "*" 0 0 100
"chr17" 27748001 27749000 "*" 0 0 100
"chr17" 27760001 27761000 "*" 6.25199891857164e-12 1.64423898607046e-11
-97.6190476190476
"chr17" 27784001 27785000 "*" 7.7715611723761e-16 3.26213507634405e-15 100
"chr17" 27863001 27864000 "*" 1.13140401492018e-08 1.87801380787728e-08 -100
"chr17" 27949001 27950000 "*" 0 0 58.8335200475776
"chr17" 28193001 28194000 "*" 1.12458486967171e-09 2.15740815043925e-09 -100
"chr17" 28195001 28196000 "*" 1.54630752646767e-11 3.85079502648228e-11 100
"chr17" 28474001 28475000 "*" 2.17381668221606e-13 6.85151076607736e-13 100
"chr17" 28648001 28649000 "*" 4.18887147191072e-13 1.2739946038722e-12 -100
"chr17" 29025001 29026000 "*" 0 0 -100
"chr17" 29238001 29239000 "*" 2.32862640281439e-08 3.69689652174363e-08
56.5217391304348
"chr17" 29297001 29298000 "*" 0 0 -51.7238386599839
"chr17" 29298001 29299000 "*" 0 0 -74.1773563859453
"chr17" 29302001 29303000 "*" 0 0 98.0769230769231
"chr17" 29382001 29383000 "*" 1.48087875295744e-10 3.22075573380618e-10 -100
"chr17" 29413001 29414000 "*" 3.63445940010365e-11 8.58565209111204e-11 -100
"chr17" 29425001 29426000 "*" 6.59550958292954e-09 1.13456194382773e-08 100
"chr17" 29741001 29742000 "*" 0 0 100
"chr17" 29742001 29743000 "*" 5.18375477590594e-05 5.08604172540523e-05
-51.7241379310345
"chr17" 29755001 29756000 "*" 1.14130926931466e-13 3.7276674571693e-13 -100
"chr17" 29758001 29759000 "*" 1.20987220242341e-10 2.66914043292706e-10
82.051282051282
"chr17" 29760001 29761000 "*" 1.02005275781636e-07 1.47868372842969e-07
-71.0084033613445
"chr17" 29764001 29765000 "*" 0 0 -77.0833333333333
"chr17" 29800001 29801000 "*" 7.50998191856311e-07 9.69923424620037e-07
-53.5714285714286
"chr17" 29840001 29841000 "*" 3.63445940010365e-11 8.58565209111204e-11 100
"chr17" 29866001 29867000 "*" 1.4432899320127e-15 5.90750815956055e-15 100

"chr17" 29888001 29889000 "*" 3.84137166520304e-14 1.32929369817675e-13 -100
"chr17" 29923001 29924000 "*" 5.95545834869426e-12 1.56921956607829e-11
-54.5454545454545
"chr17" 29988001 29989000 "*" 6.3800790395252e-07 8.32178207554581e-07
66.6666666666667
"chr17" 30064001 30065000 "*" 3.6700841921089e-08 5.65530226072258e-08 100
"chr17" 30078001 30079000 "*" 4.73898942310313e-09 8.31723528659139e-09
-66.6666666666667
"chr17" 30132001 30133000 "*" 0 0 -100
"chr17" 30153001 30154000 "*" 4.55497665141635e-08 6.94836989755125e-08
85.7142857142857
"chr17" 30165001 30166000 "*" 1.5277158427196e-09 2.87642564232045e-09 100
"chr17" 30186001 30187000 "*" 0 0 75
"chr17" 30481001 30482000 "*" 3.77475828372553e-15 1.47379557022071e-14 -100
"chr17" 30594001 30595000 "*" 2.91028312560115e-11 6.97590507139978e-11 -100
"chr17" 30604001 30605000 "*" 0 0 -100
"chr17" 30612001 30613000 "*" 1.78512946646947e-06 2.1878826958132e-06 60
"chr17" 30814001 30815000 "*" 0 0 -59.7399647987282
"chr17" 31236001 31237000 "*" 3.52704532247117e-11 8.35581292290093e-11 100
"chr17" 31258001 31259000 "*" 2.63525867794101e-11 6.37327563971936e-11
-52.1739130434783
"chr17" 31273001 31274000 "*" 3.34128893442198e-08 5.19772864618523e-08 -100
"chr17" 31319001 31320000 "*" 0 0 -69.6969696969697
"chr17" 31346001 31347000 "*" 3.12694314885675e-12 8.54066605532247e-12
-50.65164923572
"chr17" 31382001 31383000 "*" 9.68114477473137e-14 3.1851916118077e-13
-64.1025641025641
"chr17" 31491001 31492000 "*" 3.90465437760668e-13 1.19307893943277e-12 -100
"chr17" 31532001 31533000 "*" 3.6700841921089e-08 5.65530226072258e-08 -100
"chr17" 31545001 31546000 "*" 5.99234307763208e-06 6.79020205076211e-06
-65.7894736842105
"chr17" 31556001 31557000 "*" 0.000166002052559566 0.00015019676146835 -60
"chr17" 31560001 31561000 "*" 0 0 -100
"chr17" 31573001 31574000 "*" 1.11022302462516e-16 5.03662826618488e-16 -100
"chr17" 31577001 31578000 "*" 0 0 -81.1320754716981
"chr17" 31607001 31608000 "*" 3.24885605962333e-06 3.82972480568202e-06
-65.7407407407407
"chr17" 31837001 31838000 "*" 2.88779000712225e-11 6.92935809466068e-11 100
"chr17" 31994001 31995000 "*" 3.66373598126302e-15 1.43441054525534e-14
84.0579710144928
"chr17" 32000001 32001000 "*" 2.08995487582797e-10 4.46944006972663e-10 -100
"chr17" 32007001 32008000 "*" 2.08814465718632e-09 3.86232567960463e-09 100
"chr17" 32046001 32047000 "*" 9.04570973681018e-10 1.76414389822173e-09 -100
"chr17" 32256001 32257000 "*" 6.59550958292954e-09 1.13456194382773e-08 -100
"chr17" 32308001 32309000 "*" 0 0 100
"chr17" 32452001 32453000 "*" 3.34465788398575e-12 9.10668272826883e-12
71.4285714285714
"chr17" 32702001 32703000 "*" 1.10633724403897e-12 3.2010163828754e-12 75
"chr17" 32756001 32757000 "*" 1.86894943965399e-12 5.254396961681e-12
53.1914893617021
"chr17" 32759001 32760000 "*" 1.80848842568082e-08 2.92117822252068e-08 -59.375
"chr17" 32786001 32787000 "*" 1.12055920098442e-11 2.84500040664577e-11 100

"chr17" 32854001 32855000 "*" 2.10205709357814e-05 2.19253650839195e-05 61.1111111111111
"chr17" 32868001 32869000 "*" 6.93112234273485e-13 2.05218842007357e-12 100
"chr17" 32919001 32920000 "*" 2.19853864003028e-08 3.50003158916803e-08 -66.6666666666667
"chr17" 32924001 32925000 "*" 1.90520139431438e-06 2.32564618288801e-06 52
"chr17" 32929001 32930000 "*" 8.44866073321793e-05 8.01219179790309e-05 62.962962962963
"chr17" 32947001 32948000 "*" 2.4535928844216e-14 8.69144396197119e-14 100
"chr17" 32956001 32957000 "*" 6.01185767834522e-13 1.79678595397461e-12 -56.701030927835
"chr17" 32962001 32963000 "*" 0.000320710195636176 0.000276619581787338 -52.3809523809524
"chr17" 32980001 32981000 "*" 2.00227675550835e-08 3.20459176617961e-08 100
"chr17" 32993001 32994000 "*" 1.16573417585641e-14 4.29335582900508e-14 100
"chr17" 33001001 33002000 "*" 5.75885202191984e-07 7.5590152878305e-07 -52
"chr17" 33108001 33109000 "*" 1.61212432292501e-08 2.62761226785254e-08 -63.3333333333333
"chr17" 33176001 33177000 "*" 3.95353005888666e-09 7.03928529283733e-09 100
"chr17" 33202001 33203000 "*" 9.5812247025151e-14 3.15667542866807e-13 100
"chr17" 33474001 33475000 "*" 6.73487749680568e-07 8.75621186218476e-07 -68.4210526315789
"chr17" 33475001 33476000 "*" 4.60954045378159e-07 6.12056624724009e-07 76.0869565217391
"chr17" 33569001 33570000 "*" 0 0 67.2638436482085
"chr17" 33718001 33719000 "*" 0 0 -100
"chr17" 33720001 33721000 "*" 1.87405646556726e-13 5.97285292681429e-13 -100
"chr17" 33976001 33977000 "*" 8.57092175010621e-13 2.51149198099779e-12 100
"chr17" 34089001 34090000 "*" 0 0 -100
"chr17" 34211001 34212000 "*" 8.94783367988428e-06 9.87593937733781e-06 70
"chr17" 34308001 34309000 "*" 6.85672693068184e-08 1.02115284539518e-07 54.6875
"chr17" 34339001 34340000 "*" 3.77475828372553e-15 1.47379557022071e-14 100
"chr17" 34416001 34417000 "*" 0 0 100
"chr17" 34476001 34477000 "*" 7.94253551816837e-13 2.34068197145517e-12 -70.2702702702703
"chr17" 34839001 34840000 "*" 0 0 52.6819923371647
"chr17" 34980001 34981000 "*" 1.87405646556726e-13 5.97285292681429e-13 100
"chr17" 34989001 34990000 "*" 0 0 -100
"chr17" 34990001 34991000 "*" 2.22044604925031e-16 9.81641919380259e-16 -100
"chr17" 35015001 35016000 "*" 1.35890187991095e-11 3.40841971363279e-11 100
"chr17" 35021001 35022000 "*" 9.06149599799733e-11 2.03407753381119e-10 -63.2352941176471
"chr17" 35062001 35063000 "*" 0 0 71.5909090909091
"chr17" 35108001 35109000 "*" 0.000144400851692206 0.000131924792739962 -53.8461538461538
"chr17" 35120001 35121000 "*" 1.50623957750895e-12 4.28756717183481e-12 100
"chr17" 35226001 35227000 "*" 1.85407245112401e-13 5.91752840136453e-13 71.0144927536232
"chr17" 35227001 35228000 "*" 1.45439216225896e-14 5.29728884617264e-14 -73.4177215189873
"chr17" 35229001 35230000 "*" 4.3582252806651e-07 5.80526138692936e-07 76.7441860465116

"chr17" 35270001 35271000 "*" 0 0 74.3589743589744
"chr17" 35281001 35282000 "*" 9.65338919911574e-13 2.80678910656113e-12 -100
"chr17" 35418001 35419000 "*" 1.06924274989595e-08 1.78629843698404e-08 -80
"chr17" 35425001 35426000 "*" 0 0 -100
"chr17" 35716001 35717000 "*" 7.24764692705548e-12 1.88592806809993e-11
-57.3770491803279
"chr17" 35845001 35846000 "*" 1.14352971536391e-14 4.21921674335999e-14 -100
"chr17" 35947001 35948000 "*" 2.88710388929303e-10 6.03399264731139e-10 -100
"chr17" 36073001 36074000 "*" 2.79987144580218e-12 7.68634726811898e-12 -100
"chr17" 36130001 36131000 "*" 6.59039323247512e-06 7.41758959369498e-06
54.1666666666667
"chr17" 36131001 36132000 "*" 5.42206413101098e-09 9.45360888783521e-09
-64.2857142857143
"chr17" 36154001 36155000 "*" 2.69018141096922e-12 7.41487347763769e-12 -100
"chr17" 36199001 36200000 "*" 1.11022302462516e-16 5.03662826618488e-16 100
"chr17" 36207001 36208000 "*" 2.86081418776618e-07 3.90746789539144e-07 80
"chr17" 36297001 36298000 "*" 2.88657986402541e-15 1.14155613124573e-14 -100
"chr17" 36396001 36397000 "*" 0 0 100
"chr17" 36476001 36477000 "*" 2.54696427282042e-08 4.02292707681792e-08
-58.3333333333333
"chr17" 36489001 36490000 "*" 1.11022302462516e-16 5.03662826618488e-16
73.2142857142857
"chr17" 36567001 36568000 "*" 1.06964709312152e-08 1.7869381463042e-08 62.5
"chr17" 36571001 36572000 "*" 1.76764428451159e-05 1.86641818672063e-05
57.6923076923077
"chr17" 36580001 36581000 "*" 1.31006316905768e-14 4.79358493281887e-14
-73.3333333333333
"chr17" 36618001 36619000 "*" 3.04324343503026e-11 7.26599935673121e-11 100
"chr17" 36624001 36625000 "*" 7.35548041363998e-08 1.08588645363448e-07
59.0909090909091
"chr17" 36639001 36640000 "*" 1.68753899743024e-14 6.09826805967439e-14 -100
"chr17" 36664001 36665000 "*" 1.20281562487889e-12 3.46585342181251e-12 100
"chr17" 36684001 36685000 "*" 1.80538108178752e-05 1.90327565720297e-05
57.9710144927536
"chr17" 36696001 36697000 "*" 0 0 100
"chr17" 36710001 36711000 "*" 6.66249388991957e-10 1.32123034519602e-09
-64.7058823529412
"chr17" 36716001 36717000 "*" 4.57399425002336e-08 6.97610424423192e-08
-50.9090909090909
"chr17" 36736001 36737000 "*" 1.22124532708767e-15 5.03921984217778e-15 -100
"chr17" 36737001 36738000 "*" 4.01313771103418e-09 7.11882087237587e-09 100
"chr17" 36743001 36744000 "*" 7.57216511715342e-12 1.96644511393499e-11
56.8181818181818
"chr17" 36853001 36854000 "*" 0 0 100
"chr17" 36858001 36859000 "*" 0 0 -100
"chr17" 36862001 36863000 "*" 0 0 -73.8636363636364
"chr17" 36905001 36906000 "*" 0 0 -71.7660292463442
"chr17" 36956001 36957000 "*" 7.89035503601099e-13 2.32571517703366e-12
-55.8823529411765
"chr17" 36969001 36970000 "*" 2.23154827949656e-14 7.95931582053377e-14 100
"chr17" 37010001 37011000 "*" 3.2463032262342e-11 7.73024073900491e-11 65
"chr17" 37022001 37023000 "*" 1.11022302462516e-16 5.03662826618488e-16 -100

"chr17" 37235001 37236000 "*" 3.6700841921089e-08 5.65530226072258e-08 -100
"chr17" 37303001 37304000 "*" 7.0006535457523e-08 1.03695832512054e-07 -100
"chr17" 37308001 37309000 "*" 3.33066907387547e-16 1.4495649018245e-15
54.2910798122066
"chr17" 37313001 37314000 "*" 1.02645253519107e-07 1.48731465376225e-07
-71.4285714285714
"chr17" 37347001 37348000 "*" 1.65423230669148e-14 5.98698695225911e-14 100
"chr17" 37357001 37358000 "*" 7.0006535457523e-08 1.03695832512054e-07 -100
"chr17" 37358001 37359000 "*" 0 0 100
"chr17" 37362001 37363000 "*" 3.763962768355e-06 4.39370607931469e-06 -56
"chr17" 37382001 37383000 "*" 0 0 72.7272727272727
"chr17" 37387001 37388000 "*" 4.99412455501336e-07 6.60168434415759e-07 60
"chr17" 37404001 37405000 "*" 0 0 100
"chr17" 37456001 37457000 "*" 2.06501482580279e-14 7.3890370570066e-14 100
"chr17" 37687001 37688000 "*" 1.75319547768282e-09 3.28173549013282e-09 80
"chr17" 37753001 37754000 "*" 0 0 52.2546419098143
"chr17" 37755001 37756000 "*" 0 0 71.8855218855219
"chr17" 37757001 37758000 "*" 0 0 71.4285714285714
"chr17" 37792001 37793000 "*" 6.83719301175501e-08 1.01840744031014e-07
-66.6666666666667
"chr17" 37821001 37822000 "*" 4.65637306490407e-10 9.46720524046618e-10 100
"chr17" 37844001 37845000 "*" 0 0 -100
"chr17" 37859001 37860000 "*" 1.37828859436695e-10 3.01445544267661e-10 -100
"chr17" 37873001 37874000 "*" 2.00227675550835e-08 3.20459176617961e-08 -100
"chr17" 37893001 37894000 "*" 2.04292871508471e-09 3.79300264241924e-09
56.4102564102564
"chr17" 37908001 37909000 "*" 9.61897228535236e-13 2.801372325071e-12 -100
"chr17" 38020001 38021000 "*" 0 0 100
"chr17" 38075001 38076000 "*" 3.64270735797589e-09 6.5252880340617e-09
-66.6666666666667
"chr17" 38084001 38085000 "*" 1.31456705981803e-05 1.41498424153002e-05
68.2926829268293
"chr17" 38102001 38103000 "*" 6.88338275267597e-15 2.60518659957509e-14 -100
"chr17" 38218001 38219000 "*" 0 0 84.8484848484848
"chr17" 38221001 38222000 "*" 1.90181204118289e-13 6.05298617539364e-13 -100
"chr17" 38231001 38232000 "*" 3.33066907387547e-16 1.4495649018245e-15
-58.6206896551724
"chr17" 38346001 38347000 "*" 0 0 -72.2222222222222
"chr17" 38376001 38377000 "*" 1.45328193923433e-13 4.6865952604445e-13
-62.589928057554
"chr17" 38463001 38464000 "*" 0 0 100
"chr17" 38468001 38469000 "*" 0.000792090732462114 0.000639295093468792
-52.3809523809524
"chr17" 38472001 38473000 "*" 3.94957647698035e-08 6.06558079982472e-08
79.5918367346939
"chr17" 38473001 38474000 "*" 0 0 -77.9411764705882
"chr17" 38481001 38482000 "*" 3.01980662698043e-14 1.0593227230447e-13 -100
"chr17" 38502001 38503000 "*" 0 0 100
"chr17" 38519001 38520000 "*" 0 0 60.7228914938582
"chr17" 38574001 38575000 "*" 1.11022302462516e-16 5.03662826618488e-16 -80
"chr17" 38580001 38581000 "*" 3.04324343503026e-11 7.26599935673121e-11 100
"chr17" 38600001 38601000 "*" 0 0 81.5476190476191

"chr17" 38636001 38637000 "*" 2.27436958155636e-11 5.53531015140883e-11
-52.7777777777778
"chr17" 38637001 38638000 "*" 3.04324343503026e-11 7.26599935673121e-11 -100
"chr17" 38650001 38651000 "*" 1.67299207820548e-08 2.71377429441055e-08 -100
"chr17" 38666001 38667000 "*" 0 0 -100
"chr17" 38769001 38770000 "*" 1.93720595120794e-11 4.76309924836636e-11 100
"chr17" 38775001 38776000 "*" 4.57214266447181e-11 1.06761453890818e-10
-54.491833030853
"chr17" 39084001 39085000 "*" 2.91028312560115e-11 6.97590507139978e-11 100
"chr17" 39137001 39138000 "*" 3.40125705378114e-10 7.03784073980162e-10 100
"chr17" 39410001 39411000 "*" 6.57121363989432e-08 9.81365919068788e-08
-63.1205673758865
"chr17" 39455001 39456000 "*" 8.23110912762104e-10 1.61528562572585e-09
-93.1818181818182
"chr17" 39485001 39486000 "*" 0 0 -100
"chr17" 39593001 39594000 "*" 9.63829016598083e-12 2.46993429847588e-11 -100
"chr17" 39595001 39596000 "*" 1.96644922567657e-11 4.82930612417392e-11 -100
"chr17" 39728001 39729000 "*" 1.11022302462516e-16 5.03662826618488e-16
-79.1666666666667
"chr17" 39766001 39767000 "*" 1.3532508447156e-12 3.87402820252333e-12
68.4210526315789
"chr17" 39769001 39770000 "*" 3.81434206565556e-05 3.81886133293927e-05
-59.0604026845638
"chr17" 39789001 39790000 "*" 2.39808173319034e-14 8.51795945223052e-14
51.196661272464
"chr17" 39797001 39798000 "*" 1.58097868406815e-05 1.68087711747143e-05
-52.1739130434783
"chr17" 39799001 39800000 "*" 1.4432899320127e-15 5.90750815956055e-15 100
"chr17" 39819001 39820000 "*" 0 0 100
"chr17" 39883001 39884000 "*" 8.7349629751543e-09 1.47505871898511e-08 -100
"chr17" 39890001 39891000 "*" 0 0 52.5837625513761
"chr17" 39891001 39892000 "*" 0 0 94.1275167785235
"chr17" 39903001 39904000 "*" 1.48087875295744e-10 3.22075573380618e-10 -100
"chr17" 39917001 39918000 "*" 3.13902392496956e-05 3.18712470329053e-05
-56.5217391304348
"chr17" 39940001 39941000 "*" 1.93720595120794e-11 4.76309924836636e-11 -100
"chr17" 39969001 39970000 "*" 6.08662136042382e-05 5.90595322645893e-05
64.2857142857143
"chr17" 39992001 39993000 "*" 0 0 57.8389830508475
"chr17" 40127001 40128000 "*" 1.12634901405784e-10 2.49563754426169e-10 -100
"chr17" 40130001 40131000 "*" 2.73625566649116e-12 7.52483363579137e-12 -100
"chr17" 40173001 40174000 "*" 1.75934489199392e-10 3.79963721165209e-10
81.8181818181818
"chr17" 40194001 40195000 "*" 9.31511801027796e-09 1.56779071784182e-08
72.1311475409836
"chr17" 40206001 40207000 "*" 0 0 100
"chr17" 40225001 40226000 "*" 2.02060590481778e-13 6.40127405465654e-13 -100
"chr17" 40227001 40228000 "*" 5.10702591327572e-15 1.96271840288234e-14 -100
"chr17" 40312001 40313000 "*" 1.24197541140347e-10 2.73656217617171e-10 56
"chr17" 40319001 40320000 "*" 9.41103766758378e-05 8.86297231089954e-05 -60
"chr17" 40327001 40328000 "*" 1.23348664615719e-10 2.71864814990284e-10
-70.5128205128205

"chr17" 40331001 40332000 "*" 0 0 79.2207792207792
"chr17" 40437001 40438000 "*" 4.73234496034536e-09 8.30627300264826e-09 100
"chr17" 40443001 40444000 "*" 1.74853465040314e-11 4.32752710900609e-11 -100
"chr17" 40445001 40446000 "*" 1.38486533352022e-09 2.63065104258646e-09
95.8333333333333
"chr17" 40543001 40544000 "*" 8.64488408636177e-08 1.26480485233459e-07
63.1578947368421
"chr17" 40562001 40563000 "*" 3.04324343503026e-11 7.26599935673121e-11 -100
"chr17" 40610001 40611000 "*" 7.66053886991358e-15 2.88426834415713e-14
-67.3469387755102
"chr17" 40715001 40716000 "*" 0 0 -100
"chr17" 40808001 40809000 "*" 4.17456301745611e-05 4.15477058163393e-05 64
"chr17" 40820001 40821000 "*" 4.44089209850063e-15 1.71914916534614e-14 -100
"chr17" 40897001 40898000 "*" 0 0 68.4210526315789
"chr17" 40912001 40913000 "*" 0 0 50.9126065684235
"chr17" 40931001 40932000 "*" 4.32986979603811e-13 1.31491154927582e-12
-60.5263157894737
"chr17" 41030001 41031000 "*" 1.50879309046559e-13 4.85790817001478e-13
65.3092006033182
"chr17" 41086001 41087000 "*" 3.6700841921089e-08 5.65530226072258e-08 -100
"chr17" 41142001 41143000 "*" 0 0 82.9268292682927
"chr17" 41465001 41466000 "*" 1.98365768255826e-11 4.85612402473442e-11 -100
"chr17" 41476001 41477000 "*" 0 0 72.8316326530612
"chr17" 41555001 41556000 "*" 1.98294730591186e-06 2.41330824932622e-06 -68.75
"chr17" 41610001 41611000 "*" 1.92946192356658e-09 3.59193011611483e-09 -100
"chr17" 41622001 41623000 "*" 1.4894709426061e-05 1.58966670159526e-05
-53.416149068323
"chr17" 41640001 41641000 "*" 0 0 100
"chr17" 41662001 41663000 "*" 6.92287338566189e-11 1.57605630124247e-10 100
"chr17" 41683001 41684000 "*" 1.66755498298699e-13 5.34596378306383e-13
-73.6842105263158
"chr17" 41705001 41706000 "*" 4.02955446787701e-12 1.08468402381402e-11
-66.6666666666667
"chr17" 41726001 41727000 "*" 4.08209022140227e-11 9.57770690255255e-11 100
"chr17" 41728001 41729000 "*" 1.2228967838368e-09 2.33644378479772e-09
-68.4210526315789
"chr17" 41755001 41756000 "*" 1.87405646556726e-13 5.97285292681429e-13 100
"chr17" 41757001 41758000 "*" 6.59550958292954e-09 1.13456194382773e-08 -100
"chr17" 41775001 41776000 "*" 2.90317555551312e-08 4.55845441590445e-08
52.3809523809524
"chr17" 41807001 41808000 "*" 4.53658743904128e-09 8.00106907206934e-09
-79.4520547945205
"chr17" 41904001 41905000 "*" 1.10442103402608e-05 1.20217549185931e-05
59.2592592592593
"chr17" 41929001 41930000 "*" 0 0 100
"chr17" 42029001 42030000 "*" 0 0 53.7387157213422
"chr17" 42054001 42055000 "*" 4.4495740425532e-11 1.04021745302651e-10
92.1739130434783
"chr17" 42088001 42089000 "*" 2.43005615629954e-09 4.45014222280278e-09 -100
"chr17" 42134001 42135000 "*" 3.51407791754355e-11 8.33574753346092e-11
92.3076923076923
"chr17" 42149001 42150000 "*" 3.04324343503026e-11 7.26599935673121e-11 100

"chr17" 42219001 42220000 "*" 0 0 -100
"chr17" 42246001 42247000 "*" 5.88418203051333e-15 2.245355428447e-14
-71.1538461538462
"chr17" 42256001 42257000 "*" 0 0 -54.5454545454545
"chr17" 42279001 42280000 "*" 2.68450373042128e-10 5.64465213127486e-10 100
"chr17" 42306001 42307000 "*" 2.22044604925031e-16 9.81641919380259e-16 -100
"chr17" 42324001 42325000 "*" 1.11022302462516e-16 5.03662826618488e-16 -100
"chr17" 42332001 42333000 "*" 3.88022947106492e-13 1.1867462770515e-12 100
"chr17" 42334001 42335000 "*" 3.00737212910462e-12 8.23025206282417e-12
88.0794701986755
"chr17" 42350001 42351000 "*" 5.82015994288199e-08 8.75487691565306e-08
52.4844720496894
"chr17" 42377001 42378000 "*" 2.1094237467878e-15 8.47434540879246e-15 100
"chr17" 42385001 42386000 "*" 0 0 69.5852534562212
"chr17" 42401001 42402000 "*" 0 0 96.1538461538462
"chr17" 42402001 42403000 "*" 0 0 77.3406884909869
"chr17" 42403001 42404000 "*" 0 0 70.5479452054795
"chr17" 42428001 42429000 "*" 1.46509471221634e-11 3.66210873720135e-11
-85.7142857142857
"chr17" 42458001 42459000 "*" 1.98365768255826e-11 4.85612402473442e-11 -100
"chr17" 42467001 42468000 "*" 3.7274627828765e-12 1.00985250136599e-11 100
"chr17" 42708001 42709000 "*" 4.35807989696002e-09 7.7004067891801e-09
-63.6363636363636
"chr17" 42789001 42790000 "*" 3.52704532247117e-11 8.35581292290093e-11 100
"chr17" 42836001 42837000 "*" 0 0 60.2294730292576
"chr17" 42838001 42839000 "*" 9.46416009328654e-06 1.04143968019011e-05
56.8627450980392
"chr17" 42850001 42851000 "*" 8.71525074330748e-14 2.88848349331391e-13 -100
"chr17" 42851001 42852000 "*" 8.77076189453874e-15 3.27889038038365e-14 100
"chr17" 42902001 42903000 "*" 4.99900121297969e-12 1.32921056339624e-11 -100
"chr17" 42906001 42907000 "*" 0 0 68.1596956756858
"chr17" 42908001 42909000 "*" 0 0 100
"chr17" 42998001 42999000 "*" 1.89581683684992e-12 5.32311367606535e-12 100
"chr17" 43002001 43003000 "*" 1.21380683282268e-12 3.49595385723632e-12 -53.125
"chr17" 43043001 43044000 "*" 6.69497790539708e-12 1.7526706745773e-11
62.8571428571429
"chr17" 43056001 43057000 "*" 0.000134368092775117 0.000123398369467587
51.7241379310345
"chr17" 43066001 43067000 "*" 0 0 98.4848484848485
"chr17" 43078001 43079000 "*" 4.44089209850063e-16 1.91071758245033e-15 100
"chr17" 43171001 43172000 "*" 2.79987144580218e-12 7.68634726811898e-12 100
"chr17" 43180001 43181000 "*" 3.33066907387547e-16 1.4495649018245e-15 -100
"chr17" 43201001 43202000 "*" 1.36604061395929e-11 3.42333952202875e-11 -100
"chr17" 43204001 43205000 "*" 1.78972142661493e-08 2.89243321796634e-08
-56.5217391304348
"chr17" 43239001 43240000 "*" 1.29542274924788e-05 1.39536802611071e-05
-59.1549295774648
"chr17" 43240001 43241000 "*" 3.66373598126302e-15 1.43441054525534e-14 -56.25
"chr17" 43265001 43266000 "*" 1.49613654798486e-12 4.26244554429779e-12 100
"chr17" 43299001 43300000 "*" 0 0 59.4826189125679
"chr17" 43308001 43309000 "*" 3.88428432307464e-07 5.20823182914617e-07
-80.4878048780488

"chr17" 43335001 43336000 "*" 0 0 100
"chr17" 43971001 43972000 "*" 0 0 97.4063400576369
"chr17" 43972001 43973000 "*" 0 0 100
"chr17" 43973001 43974000 "*" 0 0 78.7878787878788
"chr17" 44848001 44849000 "*" 0 0 -50.4697009102731
"chr17" 44854001 44855000 "*" 2.43005615629954e-09 4.45014222280278e-09 100
"chr17" 44928001 44929000 "*" 0 0 51.5579414856549
"chr17" 44929001 44930000 "*" 0 0 62.6905218819899
"chr17" 44954001 44955000 "*" 2.61139809776267e-09 4.76642858684585e-09 60
"chr17" 44987001 44988000 "*" 1.31610949317684e-05 1.4161144785177e-05
66.6666666666667
"chr17" 44988001 44989000 "*" 3.7159164634204e-13 1.14054917904619e-12
-79.3650793650794
"chr17" 45035001 45036000 "*" 4.26951363152739e-10 8.72510873516957e-10 100
"chr17" 45047001 45048000 "*" 8.25450818808804e-13 2.42473843346228e-12 100
"chr17" 45055001 45056000 "*" 0 0 51.4112124150308
"chr17" 45178001 45179000 "*" 1.61204383175573e-13 5.17337152201509e-13 -100
"chr17" 45372001 45373000 "*" 5.55111512312578e-16 2.36485094870365e-15 -100
"chr17" 45373001 45374000 "*" 2.52324385829894e-08 3.98810423951785e-08
56.221198156682
"chr17" 45380001 45381000 "*" 5.20693488326174e-11 1.20568048847758e-10
76.9230769230769
"chr17" 45400001 45401000 "*" 0 0 63.8053097345133
"chr17" 45726001 45727000 "*" 0 0 51.7799352750809
"chr17" 45790001 45791000 "*" 6.4152538836737e-10 1.27477697728417e-09 -100
"chr17" 45794001 45795000 "*" 3.95353005888666e-09 7.03928529283733e-09 100
"chr17" 45803001 45804000 "*" 3.10586911744792e-09 5.61087486660776e-09
-77.6315789473684
"chr17" 45823001 45824000 "*" 2.70610380592728e-06 3.22927704626886e-06
60.6060606060606
"chr17" 45920001 45921000 "*" 1.37828859436695e-10 3.01445544267661e-10 100
"chr17" 45928001 45929000 "*" 0 0 -67.9455678063192
"chr17" 45960001 45961000 "*" 4.08209022140227e-11 9.57770690255255e-11 -100
"chr17" 45961001 45962000 "*" 2.78284798538309e-08 4.37734975842904e-08
-75.5102040816327
"chr17" 46018001 46019000 "*" 2.93101620751912e-09 5.30844957624644e-09
82.962962962963
"chr17" 46027001 46028000 "*" 7.0006535457523e-08 1.03695832512054e-07 100
"chr17" 46038001 46039000 "*" 2.72646146104449e-07 3.73405126957647e-07
-66.6666666666667
"chr17" 46056001 46057000 "*" 1.53680526193689e-07 2.17091175393351e-07 -60
"chr17" 46070001 46071000 "*" 1.11022302462516e-16 5.03662826618488e-16 100
"chr17" 46100001 46101000 "*" 0 0 -62.5794191059678
"chr17" 46104001 46105000 "*" 0 0 -59.4936708860759
"chr17" 46123001 46124000 "*" 0 0 100
"chr17" 46406001 46407000 "*" 1.34170119459043e-08 2.20794819956426e-08
54.5454545454545
"chr17" 46559001 46560000 "*" 4.99900121297969e-12 1.32921056339624e-11 100
"chr17" 46583001 46584000 "*" 3.95353005888666e-09 7.03928529283733e-09 100
"chr17" 46648001 46649000 "*" 0 0 -74.1167434715822
"chr17" 46655001 46656000 "*" 0 0 -50.1411022576361
"chr17" 46680001 46681000 "*" 1.66533453693773e-14 6.02507639546739e-14

-98.6666666666667
"chr17" 46681001 46682000 "*" 0 0 -81.8518518518518
"chr17" 46690001 46691000 "*" 0 0 -80.0578034682081
"chr17" 46696001 46697000 "*" 0 0 93.9947780678851
"chr17" 46724001 46725000 "*" 0 0 -86.2450592885375
"chr17" 46756001 46757000 "*" 3.01110192335585e-09 5.44769836120163e-09
54.0229885057471
"chr17" 46805001 46806000 "*" 0 0 -78.3216783216783
"chr17" 46897001 46898000 "*" 9.71063229826541e-11 2.16781365050398e-10 100
"chr17" 47044001 47045000 "*" 4.08990052408464e-05 4.07512937702815e-05
-72.7272727272727
"chr17" 47073001 47074000 "*" 0 0 59.6692739124889
"chr17" 47074001 47075000 "*" 0 0 85.5555107472678
"chr17" 47075001 47076000 "*" 0 0 76.4160091263428
"chr17" 47119001 47120000 "*" 2.00227675550835e-08 3.20459176617961e-08 -100
"chr17" 47166001 47167000 "*" 1.11022302462516e-16 5.03662826618488e-16
-65.2173913043478
"chr17" 47216001 47217000 "*" 6.70841160399505e-12 1.75486545176836e-11 -100
"chr17" 47268001 47269000 "*" 2.88710388929303e-10 6.03399264731139e-10 -100
"chr17" 47307001 47308000 "*" 0 0 56.5933170334148
"chr17" 47335001 47336000 "*" 2.50910403565285e-14 8.87903196465237e-14 -100
"chr17" 47337001 47338000 "*" 0 0 -75.2495543672014
"chr17" 47477001 47478000 "*" 0 0 -100
"chr17" 47479001 47480000 "*" 8.01666433236647e-09 1.36392368970109e-08 -100
"chr17" 47517001 47518000 "*" 4.11974563130357e-07 5.50491688288806e-07
74.2424242424242
"chr17" 47519001 47520000 "*" 0.000174526632750749 0.000157303882021212 53.125
"chr17" 47544001 47545000 "*" 2.43005615629954e-09 4.45014222280278e-09 100
"chr17" 47562001 47563000 "*" 0 0 -90.0990099009901
"chr17" 47569001 47570000 "*" 2.29877117341459e-10 4.88095720452722e-10
-51.7241379310345
"chr17" 47572001 47573000 "*" 0 0 53.113209293354
"chr17" 47575001 47576000 "*" 0 0 51.7612997673385
"chr17" 47587001 47588000 "*" 0 0 -88.2539682539683
"chr17" 47602001 47603000 "*" 4.32209823486573e-12 1.15935290479641e-11 -100
"chr17" 47622001 47623000 "*" 1.39999123405232e-13 4.5217351355987e-13
-71.4285714285714
"chr17" 47634001 47635000 "*" 2.99556568439385e-09 5.42076605400438e-09
-83.3333333333333
"chr17" 47645001 47646000 "*" 0 0 55.3911205073996
"chr17" 47652001 47653000 "*" 0 0 71.4285714285714
"chr17" 47653001 47654000 "*" 0 0 77.9301120458416
"chr17" 47654001 47655000 "*" 0 0 74.477419701076
"chr17" 47660001 47661000 "*" 8.41938408424614e-06 9.32872554744457e-06
-54.3859649122807
"chr17" 47759001 47760000 "*" 3.1216415677715e-05 3.17090411597004e-05
54.1666666666667
"chr17" 47772001 47773000 "*" 3.63986951690265e-10 7.50142469577035e-10
69.4444444444444
"chr17" 47796001 47797000 "*" 0 0 -77.2727272727273
"chr17" 47840001 47841000 "*" 0 0 73.1060606060606
"chr17" 47935001 47936000 "*" 2.41784292074332e-05 2.49875818383709e-05

67.741935483871
"chr17" 47938001 47939000 "*" 8.01666433236647e-09 1.36392368970109e-08 100
"chr17" 47958001 47959000 "*" 0 0 96
"chr17" 47977001 47978000 "*" 1.27147854810383e-05 1.37108165154609e-05
67.3469387755102
"chr17" 47978001 47979000 "*" 2.91028312560115e-11 6.97590507139978e-11 -100
"chr17" 47991001 47992000 "*" 2.80939082775156e-07 3.840942150991e-07
-69.5652173913043
"chr17" 47997001 47998000 "*" 1.22707844552039e-07 1.75940305659382e-07
-73.6842105263158
"chr17" 47998001 47999000 "*" 4.44089209850063e-16 1.91071758245033e-15
70.7317073170732
"chr17" 48006001 48007000 "*" 1.96644922567657e-11 4.82930612417392e-11 100
"chr17" 48012001 48013000 "*" 5.42852462503962e-06 6.19160768640719e-06
62.6865671641791
"chr17" 48027001 48028000 "*" 1.53210777398272e-14 5.5628583449216e-14 100
"chr17" 48071001 48072000 "*" 0 0 57.2259992937355
"chr17" 48076001 48077000 "*" 4.09721145899766e-11 9.61048181214516e-11
55.4054054054054
"chr17" 48113001 48114000 "*" 9.04570973681018e-10 1.76414389822173e-09 100
"chr17" 48125001 48126000 "*" 1.02210118146928e-09 1.97387928745632e-09
52.4621212121212
"chr17" 48139001 48140000 "*" 5.6621374255883e-15 2.16468580166064e-14 -100
"chr17" 48142001 48143000 "*" 0 0 -100
"chr17" 48145001 48146000 "*" 0 0 67.3469387755102
"chr17" 48151001 48152000 "*" 0 0 100
"chr17" 48168001 48169000 "*" 1.91840648624542e-05 2.01207824673728e-05
63.4146341463415
"chr17" 48184001 48185000 "*" 2.99523939578661e-05 3.0506069730201e-05
63.4615384615385
"chr17" 48227001 48228000 "*" 0 0 53.7100381528746
"chr17" 48247001 48248000 "*" 1.81077375316363e-13 5.78398288273767e-13
69.1176470588235
"chr17" 48277001 48278000 "*" 0 0 -61.7521367521367
"chr17" 48284001 48285000 "*" 0 0 -86.4197530864197
"chr17" 48287001 48288000 "*" 0 0 -100
"chr17" 48333001 48334000 "*" 0 0 84.5360824742268
"chr17" 48334001 48335000 "*" 2.15833517724917e-09 3.98588754314856e-09
52.0833333333333
"chr17" 48351001 48352000 "*" 3.33066907387547e-16 1.4495649018245e-15
-52.1739130434783
"chr17" 48359001 48360000 "*" 1.11022302462516e-16 5.03662826618488e-16
93.3333333333333
"chr17" 48424001 48425000 "*" 0 0 -60.145725388601
"chr17" 48469001 48470000 "*" 9.63829016598083e-12 2.46993429847588e-11 -100
"chr17" 48511001 48512000 "*" 3.34128893442198e-08 5.19772864618523e-08 100
"chr17" 48536001 48537000 "*" 0 0 -100
"chr17" 48557001 48558000 "*" 3.66373598126302e-15 1.43441054525534e-14
-89.247311827957
"chr17" 48589001 48590000 "*" 1.83952852950142e-12 5.17499701329218e-12
66.6666666666667
"chr17" 48606001 48607000 "*" 4.01313771103418e-09 7.11882087237587e-09 100

"chr17" 48625001 48626000 "*" 0.000591775622736268 0.000487833560499899
51.7241379310345
"chr17" 48633001 48634000 "*" 1.09615555864417e-05 1.193832842474e-05
81.8181818181818
"chr17" 48640001 48641000 "*" 1.24467103290726e-12 3.58051985521724e-12
-71.1538461538462
"chr17" 48662001 48663000 "*" 8.25450818808804e-13 2.42473843346228e-12 100
"chr17" 48694001 48695000 "*" 4.63429294939033e-12 1.23918968240111e-11
54.8672566371681
"chr17" 48735001 48736000 "*" 6.87627732531837e-12 1.79455618953117e-11
-55.5555555555556
"chr17" 48762001 48763000 "*" 3.68352487728485e-05 3.69658679362228e-05
52.1739130434783
"chr17" 48845001 48846000 "*" 0 0 -100
"chr17" 48860001 48861000 "*" 1.36223143434311e-05 1.46230742870329e-05
63.6363636363636
"chr17" 48907001 48908000 "*" 3.6700841921089e-08 5.65530226072258e-08 -100
"chr17" 48997001 48998000 "*" 0 0 -74.3362831858407
"chr17" 49019001 49020000 "*" 0 0 -88.9830508474576
"chr17" 49027001 49028000 "*" 0 0 -56.1615548132402
"chr17" 49034001 49035000 "*" 0.000209763322864043 0.000186660769941092
-56.5217391304348
"chr17" 49413001 49414000 "*" 6.77335965093562e-12 1.76897011246462e-11 100
"chr17" 49439001 49440000 "*" 6.81955830694747e-08 1.01608917521198e-07
56.6666666666667
"chr17" 49452001 49453000 "*" 6.25915924112874e-06 7.06891604472024e-06
-57.3770491803279
"chr17" 49485001 49486000 "*" 8.7349629751543e-09 1.47505871898511e-08 100
"chr17" 49874001 49875000 "*" 1.11022302462516e-16 5.03662826618488e-16
-55.6717881660673
"chr17" 49959001 49960000 "*" 8.7349629751543e-09 1.47505871898511e-08 -100
"chr17" 50919001 50920000 "*" 2.17936779733918e-13 6.86796876068017e-13
-72.6315789473684
"chr17" 52284001 52285000 "*" 3.03979064142368e-13 9.42350883309283e-13 100
"chr17" 53303001 53304000 "*" 6.66133814775094e-16 2.81595744474255e-15 100
"chr17" 53314001 53315000 "*" 1.45550238528358e-12 4.15462774227212e-12
54.5454545454545
"chr17" 53551001 53552000 "*" 2.64233079860787e-14 9.32753027293626e-14
95.6521739130435
"chr17" 53588001 53589000 "*" 2.22044604925031e-16 9.81641919380259e-16 100
"chr17" 53632001 53633000 "*" 3.34128893442198e-08 5.19772864618523e-08 -100
"chr17" 53633001 53634000 "*" 2.561585055183e-09 4.67991191564998e-09
61.7977528089888
"chr17" 53707001 53708000 "*" 2.15125472990962e-10 4.58126659943874e-10 100
"chr17" 53922001 53923000 "*" 0 0 55.2376980817348
"chr17" 54141001 54142000 "*" 3.07531777821168e-14 1.07626052665081e-13 100
"chr17" 54669001 54670000 "*" 0 0 54.2105263157895
"chr17" 54670001 54671000 "*" 0 0 73.6916548797737
"chr17" 54671001 54672000 "*" 0 0 64.5202168755025
"chr17" 54672001 54673000 "*" 0 0 54.6458893910499
"chr17" 54674001 54675000 "*" 0 0 61.6999895137894
"chr17" 54678001 54679000 "*" 9.32032229172819e-13 2.7181920310254e-12 -100

"chr17" 54716001 54717000 "*" 0 0 -100
"chr17" 54789001 54790000 "*" 3.7274627828765e-12 1.00985250136599e-11 100
"chr17" 54829001 54830000 "*" 8.7349629751543e-09 1.47505871898511e-08 100
"chr17" 54874001 54875000 "*" 2.38808972596871e-13 7.49683244521259e-13 52.5
"chr17" 54988001 54989000 "*" 2.02060590481778e-13 6.40127405465654e-13 100
"chr17" 55207001 55208000 "*" 2.4535928844216e-14 8.69144396197119e-14 100
"chr17" 55227001 55228000 "*" 2.16382467499443e-13 6.82775854166559e-13 -100
"chr17" 55566001 55567000 "*" 1.63283805876269e-06 2.01223257972618e-06
-51.7241379310345
"chr17" 55717001 55718000 "*" 0 0 -100
"chr17" 55729001 55730000 "*" 6.17334590091723e-06 6.9782376326089e-06
-53.1914893617021
"chr17" 55740001 55741000 "*" 1.0737348875578e-06 1.35741403538047e-06
-64.2028985507246
"chr17" 55760001 55761000 "*" 0 0 91.5343915343915
"chr17" 55807001 55808000 "*" 6.52733422867868e-12 1.71146951718423e-11 -100
"chr17" 55813001 55814000 "*" 2.8421709430404e-14 9.99616402633955e-14 -100
"chr17" 55840001 55841000 "*" 3.61177185936867e-07 4.86418348151014e-07
66.6666666666667
"chr17" 55910001 55911000 "*" 1.2228966728145e-09 2.33644378479772e-09
-66.6666666666667
"chr17" 55940001 55941000 "*" 3.10862446895044e-15 1.22466437093774e-14 -100
"chr17" 55947001 55948000 "*" 1.20762364197446e-06 1.51606374274134e-06
-68.2926829268293
"chr17" 55954001 55955000 "*" 5.51780843238703e-14 1.87243792572872e-13 100
"chr17" 55969001 55970000 "*" 3.77475828372553e-15 1.47379557022071e-14 -100
"chr17" 55977001 55978000 "*" 2.35400459125401e-08 3.73491151109009e-08
-63.0952380952381
"chr17" 55991001 55992000 "*" 1.00578004369467e-07 1.45905892474209e-07 -60
"chr17" 56002001 56003000 "*" 1.4189075137061e-08 2.32852316126461e-08 52
"chr17" 56016001 56017000 "*" 7.88258347483861e-15 2.96145301916012e-14 -100
"chr17" 56160001 56161000 "*" 1.42310706999771e-05 1.52300938663303e-05
-57.4074074074074
"chr17" 56281001 56282000 "*" 1.11022302462516e-16 5.03662826618488e-16
-69.7674418604651
"chr17" 56284001 56285000 "*" 3.44722487444216e-07 4.6586018916251e-07 80
"chr17" 56395001 56396000 "*" 7.43486582099884e-06 8.30651437189353e-06 67.5
"chr17" 56563001 56564000 "*" 6.88338275267597e-15 2.60518659957509e-14 100
"chr17" 56591001 56592000 "*" 0 0 100
"chr17" 56602001 56603000 "*" 1.01313471477793e-05 1.10909163221897e-05 -62.5
"chr17" 56702001 56703000 "*" 5.6621374255883e-15 2.16468580166064e-14 100
"chr17" 57171001 57172000 "*" 0 0 100
"chr17" 57285001 57286000 "*" 4.34121761316852e-07 5.78408567117475e-07 -52
"chr17" 57295001 57296000 "*" 7.0006535457523e-08 1.03695832512054e-07 100
"chr17" 57507001 57508000 "*" 2.50910403565285e-14 8.87903196465237e-14 100
"chr17" 57531001 57532000 "*" 5.6621374255883e-15 2.16468580166064e-14 100
"chr17" 57635001 57636000 "*" 3.6700841921089e-08 5.65530226072258e-08 -100
"chr17" 57643001 57644000 "*" 0 0 93.5483870967742
"chr17" 57932001 57933000 "*" 9.75745124698335e-08 1.41765802248358e-07
59.2592592592593
"chr17" 57970001 57971000 "*" 0 0 100
"chr17" 58212001 58213000 "*" 0 0 94.1605839416058

"chr17" 58223001 58224000 "*" 0 0 100
"chr17" 58238001 58239000 "*" 0 0 100
"chr17" 58491001 58492000 "*" 2.66453525910038e-14 9.40074020513994e-14 -100
"chr17" 58750001 58751000 "*" 4.46539891729714e-08 6.81870813025817e-08
53.5714285714286
"chr17" 59159001 59160000 "*" 1.36214064472284e-08 2.23946300151075e-08
-66.6666666666667
"chr17" 59362001 59363000 "*" 6.54429038737803e-08 9.77502704411541e-08
56.5217391304348
"chr17" 59366001 59367000 "*" 1.5277158427196e-09 2.87642564232045e-09 100
"chr17" 59388001 59389000 "*" 5.794809077031e-12 1.52911192757929e-11 -100
"chr17" 59434001 59435000 "*" 2.2035949465149e-07 3.0539117362908e-07 65.625
"chr17" 59436001 59437000 "*" 0 0 -100
"chr17" 59439001 59440000 "*" 1.48087875295744e-10 3.22075573380618e-10 -100
"chr17" 59446001 59447000 "*" 3.83080972332639e-06 4.46567428273108e-06
-59.3220338983051
"chr17" 59476001 59477000 "*" 0 0 55.2369933473345
"chr17" 59544001 59545000 "*" 2.09012653296092e-07 2.90554228858733e-07
-71.1864406779661
"chr17" 59545001 59546000 "*" 8.65973959207622e-14 2.87362558212404e-13 -100
"chr17" 59572001 59573000 "*" 1.83431048128568e-12 5.16659144201439e-12
-78.2608695652174
"chr17" 59591001 59592000 "*" 6.02950546157999e-08 9.04921644553472e-08
-71.4285714285714
"chr17" 60198001 60199000 "*" 3.84590465030143e-08 5.91355325062938e-08 -85
"chr17" 60290001 60291000 "*" 2.43005615629954e-09 4.45014222280278e-09 -100
"chr17" 60545001 60546000 "*" 1.74853465040314e-11 4.32752710900609e-11 100
"chr17" 60555001 60556000 "*" 0 0 -100
"chr17" 60698001 60699000 "*" 8.57092175010621e-13 2.51149198099779e-12 100
"chr17" 60704001 60705000 "*" 0 0 54.5202380214467
"chr17" 60707001 60708000 "*" 0 0 56.9875776397516
"chr17" 60726001 60727000 "*" 2.02327044007689e-12 5.65300384792621e-12 100
"chr17" 60735001 60736000 "*" 1.38333788868295e-13 4.46985177097929e-13
53.3333333333333
"chr17" 60751001 60752000 "*" 4.44089209850063e-16 1.91071758245033e-15
-53.4883720930233
"chr17" 60773001 60774000 "*" 1.38358435153307e-07 1.97065834231448e-07
-55.4824561403509
"chr17" 60780001 60781000 "*" 8.31955678459728e-07 1.06757059776961e-06
57.6086956521739
"chr17" 60790001 60791000 "*" 0 0 -100
"chr17" 60904001 60905000 "*" 1.13140401492018e-08 1.87801380787728e-08 100
"chr17" 60918001 60919000 "*" 2.10793207910953e-05 2.19814212751768e-05
58.8235294117647
"chr17" 61323001 61324000 "*" 9.65729496371637e-10 1.87255912493232e-09 -100
"chr17" 61530001 61531000 "*" 1.4432899320127e-15 5.90750815956055e-15 100
"chr17" 61531001 61532000 "*" 1.96644922567657e-11 4.82930612417392e-11 100
"chr17" 61578001 61579000 "*" 0 0 71.363204696538
"chr17" 61587001 61588000 "*" 0 0 100
"chr17" 61593001 61594000 "*" 7.0006535457523e-08 1.03695832512054e-07 -100
"chr17" 61765001 61766000 "*" 1.10219389171107e-10 2.44660222411491e-10
-61.5384615384615

"chr17" 61959001 61960000 "*" 0 0 83.75
"chr17" 61987001 61988000 "*" 2.22044604925031e-16 9.81641919380259e-16 -56.25
"chr17" 62014001 62015000 "*" 7.105427357601e-15 2.68362283258525e-14 100
"chr17" 62015001 62016000 "*" 1.48087875295744e-10 3.22075573380618e-10 100
"chr17" 62041001 62042000 "*" 0 0 -100
"chr17" 62052001 62053000 "*" 0 0 -100
"chr17" 62069001 62070000 "*" 8.07436562055841e-10 1.58586939298384e-09 63.6363636363636
"chr17" 62212001 62213000 "*" 1.64217528464405e-11 4.07821109745182e-11 71.2121212121212
"chr17" 62461001 62462000 "*" 1.23623818034657e-05 1.33534251888187e-05 -66.6666666666667
"chr17" 62595001 62596000 "*" 1.34665347317764e-07 1.92087838409547e-07 -71.4285714285714
"chr17" 62651001 62652000 "*" 7.20239098899178e-05 6.90632392773323e-05 -58.8235294117647
"chr17" 62676001 62677000 "*" 0 0 -80.9523809523809
"chr17" 62709001 62710000 "*" 0 0 -100
"chr17" 62746001 62747000 "*" 6.92287338566189e-11 1.57605630124247e-10 100
"chr17" 62770001 62771000 "*" 1.99840144432528e-12 5.59476580335607e-12 -100
"chr17" 62772001 62773000 "*" 4.2927328358644e-10 8.76998041338936e-10 58.2391713747646
"chr17" 62777001 62778000 "*" 0 0 -82.9721362229102
"chr17" 62778001 62779000 "*" 4.34180936759176e-08 6.63788545546839e-08 -65.625
"chr17" 62811001 62812000 "*" 3.33066907387547e-16 1.4495649018245e-15 100
"chr17" 63082001 63083000 "*" 0 0 59.7014925373134
"chr17" 63117001 63118000 "*" 4.32209823486573e-12 1.15935290479641e-11 -100
"chr17" 63127001 63128000 "*" 1.33226762955019e-15 5.48133041560118e-15 100
"chr17" 63133001 63134000 "*" 0 0 -66.2337662337662
"chr17" 63167001 63168000 "*" 0 0 100
"chr17" 63245001 63246000 "*" 4.77395900588817e-15 1.84201484337652e-14 -100
"chr17" 63367001 63368000 "*" 1.4095391520641e-12 4.02839242300806e-12 79.746835443038
"chr17" 63399001 63400000 "*" 2.158880544334e-07 2.99532767832466e-07 66.8639053254438
"chr17" 63528001 63529000 "*" 2.22044604925031e-16 9.81641919380259e-16 100
"chr17" 63529001 63530000 "*" 8.88178419700125e-16 3.70670032207938e-15 94.1176470588235
"chr17" 63550001 63551000 "*" 0 0 100
"chr17" 63554001 63555000 "*" 0 0 81.25
"chr17" 63555001 63556000 "*" 0 0 89.3048128342246
"chr17" 63558001 63559000 "*" 0 0 61.3924050632911
"chr17" 63563001 63564000 "*" 6.15840711759574e-13 1.83676803572802e-12 -100
"chr17" 63582001 63583000 "*" 3.33066907387547e-16 1.4495649018245e-15 -60.7142857142857
"chr17" 63639001 63640000 "*" 3.04324343503026e-11 7.26599935673121e-11 -100
"chr17" 63678001 63679000 "*" 8.7349629751543e-09 1.47505871898511e-08 100
"chr17" 63689001 63690000 "*" 3.04324343503026e-11 7.26599935673121e-11 -100
"chr17" 63772001 63773000 "*" 6.32827124036339e-15 2.40666772484995e-14 55.1724137931034
"chr17" 64235001 64236000 "*" 4.02167188440217e-12 1.08266700497926e-11 -100
"chr17" 64264001 64265000 "*" 2.06501482580279e-14 7.3890370570066e-14 100

"chr17" 64346001 64347000 "*" 2.67283972732457e-11 6.45686178157698e-11 -100
"chr17" 64370001 64371000 "*" 0 0 -51.685393258427
"chr17" 64465001 64466000 "*" 2.43005615629954e-09 4.45014222280278e-09 100
"chr17" 64728001 64729000 "*" 0 0 -51.4705882352941
"chr17" 64776001 64777000 "*" 5.44009282066327e-15 2.08486137681812e-14 -100
"chr17" 64934001 64935000 "*" 1.07472154509125e-08 1.79491210743456e-08 58.8235294117647
"chr17" 64945001 64946000 "*" 1.6410484970919e-08 2.67292767007535e-08 64.7058823529412
"chr17" 64960001 64961000 "*" 0 0 88.1750465549348
"chr17" 64961001 64962000 "*" 0 0 84.9341186952433
"chr17" 64979001 64980000 "*" 2.4980018054066e-14 8.8434965448031e-14 -51.4285714285714
"chr17" 64991001 64992000 "*" 1.13140401492018e-08 1.87801380787728e-08 100
"chr17" 64996001 64997000 "*" 0.000792090732462114 0.000639295093468792 -52.3809523809524
"chr17" 65017001 65018000 "*" 3.33066907387547e-16 1.4495649018245e-15 -67.4418604651163
"chr17" 65030001 65031000 "*" 1.89848137210902e-14 6.82212372863451e-14 100
"chr17" 65342001 65343000 "*" 0 0 100
"chr17" 65375001 65376000 "*" 0 0 -91.9308357348703
"chr17" 65487001 65488000 "*" 0 0 -64.281365875256
"chr17" 65516001 65517000 "*" 9.67004254448511e-14 3.18178990782609e-13 100
"chr17" 65525001 65526000 "*" 0 0 -86.1224489795918
"chr17" 65615001 65616000 "*" 0 0 50.9551243177683
"chr17" 65649001 65650000 "*" 4.73234496034536e-09 8.30627300264826e-09 100
"chr17" 65650001 65651000 "*" 0 0 100
"chr17" 65652001 65653000 "*" 5.47326179045626e-07 7.20206131209137e-07 63.0434782608696
"chr17" 65662001 65663000 "*" 4.01313771103418e-09 7.11882087237587e-09 100
"chr17" 65728001 65729000 "*" 1.92946192356658e-09 3.59193011611483e-09 100
"chr17" 65747001 65748000 "*" 4.16429224436854e-10 8.52658537675143e-10 70.8333333333333
"chr17" 65795001 65796000 "*" 4.08209022140227e-11 9.57770690255255e-11 100
"chr17" 65816001 65817000 "*" 9.67004254448511e-14 3.18178990782609e-13 -100
"chr17" 65992001 65993000 "*" 0 0 -58.3663384324698
"chr17" 66012001 66013000 "*" 1.11022302462516e-16 5.03662826618488e-16 -100
"chr17" 66049001 66050000 "*" 1.39779633423487e-07 1.98463037958589e-07 100
"chr17" 66063001 66064000 "*" 3.34128893442198e-08 5.19772864618523e-08 100
"chr17" 66129001 66130000 "*" 1.06026298851702e-13 3.47698459474259e-13 -85.1851851851852
"chr17" 66148001 66149000 "*" 0 0 77.9310344827586
"chr17" 66192001 66193000 "*" 0 0 76.3140161725067
"chr17" 66195001 66196000 "*" 0 0 53.3249871437101
"chr17" 66230001 66231000 "*" 3.52704532247117e-11 8.35581292290093e-11 -100
"chr17" 66287001 66288000 "*" 0 0 92.485549132948
"chr17" 66385001 66386000 "*" 7.0006535457523e-08 1.03695832512054e-07 -100
"chr17" 66391001 66392000 "*" 0 0 69.4202898550725
"chr17" 66421001 66422000 "*" 6.4152538836737e-10 1.27477697728417e-09 100
"chr17" 66587001 66588000 "*" 9.22717424689523e-09 1.55374184879414e-08 66.6666666666667
"chr17" 66682001 66683000 "*" 4.9960036108132e-14 1.70780466925544e-13

56.0515636190022
"chr17" 66705001 66706000 "*" 1.67299207820548e-08 2.71377429441055e-08 100
"chr17" 66813001 66814000 "*" 1.48087875295744e-10 3.22075573380618e-10 100
"chr17" 66965001 66966000 "*" 0 0 100
"chr17" 67793001 67794000 "*" 1.13140401492018e-08 1.87801380787728e-08 -100
"chr17" 68165001 68166000 "*" 0 0 70.1986754966887
"chr17" 68239001 68240000 "*" 1.14124265593318e-11 2.89138062669327e-11 100
"chr17" 68665001 68666000 "*" 0.000135810753319365 0.000124644681570312
-57.1428571428571
"chr17" 69778001 69779000 "*" 3.25628413122558e-13 1.00684523811062e-12
52.0833333333333
"chr17" 70105001 70106000 "*" 1.37001521238744e-13 4.42851918810123e-13 -100
"chr17" 70157001 70158000 "*" 3.25818809512324e-05 3.29735679006858e-05
66.6666666666667
"chr17" 70220001 70221000 "*" 3.88578058618805e-15 1.5152981210988e-14 100
"chr17" 70233001 70234000 "*" 0 0 -100
"chr17" 70315001 70316000 "*" 8.80406858527749e-14 2.91456141063452e-13 100
"chr17" 70349001 70350000 "*" 1.43568240329461e-06 1.78424201379716e-06 -60
"chr17" 70375001 70376000 "*" 8.49320613838245e-14 2.82195654136649e-13
-57.3186528497409
"chr17" 70391001 70392000 "*" 0 0 96.4285714285714
"chr17" 70407001 70408000 "*" 7.93809462606987e-14 2.64679416473686e-13 -100
"chr17" 70442001 70443000 "*" 1.06091555540644e-08 1.7728129983348e-08
-77.7777777777778
"chr17" 70461001 70462000 "*" 0 0 -100
"chr17" 70497001 70498000 "*" 8.5158797569207e-08 1.24715583872536e-07
73.8095238095238
"chr17" 70513001 70514000 "*" 0 0 100
"chr17" 70560001 70561000 "*" 9.71063229826541e-11 2.16781365050398e-10 100
"chr17" 70571001 70572000 "*" 9.1699505433418e-08 1.33679473255088e-07
-93.3333333333333
"chr17" 70587001 70588000 "*" 1.11022302462516e-16 5.03662826618488e-16
65.9574468085106
"chr17" 70600001 70601000 "*" 1.04002551015725e-09 2.00719332654647e-09
-58.030303030303
"chr17" 70619001 70620000 "*" 0 0 -93.9393939393939
"chr17" 70630001 70631000 "*" 2.02327044007689e-12 5.65300384792621e-12 -100
"chr17" 70631001 70632000 "*" 3.04467695499966e-09 5.50545751863268e-09
-53.8461538461538
"chr17" 70646001 70647000 "*" 2.29066964285707e-08 3.63920002936616e-08
-51.3513513513514
"chr17" 70649001 70650000 "*" 2.94818018886644e-08 4.62476549944916e-08
-67.2131147540984
"chr17" 70671001 70672000 "*" 1.87405646556726e-13 5.97285292681429e-13 -100
"chr17" 70675001 70676000 "*" 2.22044604925031e-16 9.81641919380259e-16
-61.2903225806452
"chr17" 70690001 70691000 "*" 0 0 59.6491228070175
"chr17" 70734001 70735000 "*" 0 0 -100
"chr17" 70745001 70746000 "*" 5.05457490085348e-07 6.67735965305576e-07
-54.5454545454545
"chr17" 70787001 70788000 "*" 2.17381668221606e-13 6.85151076607736e-13 100
"chr17" 70969001 70970000 "*" 9.99200722162641e-16 4.15382497462808e-15 -100

"chr17" 70976001 70977000 "*" 1.13140401492018e-08 1.87801380787728e-08 100
"chr17" 71007001 71008000 "*" 0 0 100
"chr17" 71047001 71048000 "*" 1.19534143864231e-05 1.29468849991476e-05
-60.7142857142857
"chr17" 71152001 71153000 "*" 2.02233485513403e-09 3.75702529178912e-09
-87.7777777777778
"chr17" 71257001 71258000 "*" 2.15162852534867e-07 2.98606087005393e-07
-54.5454545454545
"chr17" 71263001 71264000 "*" 0 0 90.5511811023622
"chr17" 71295001 71296000 "*" 7.74953223814379e-09 1.32223084306055e-08 60
"chr17" 71333001 71334000 "*" 3.04324343503026e-11 7.26599935673121e-11 -100
"chr17" 71341001 71342000 "*" 8.86993185567064e-08 1.29554903474019e-07
-55.5555555555556
"chr17" 71352001 71353000 "*" 0 0 69.6428571428571
"chr17" 71367001 71368000 "*" 1.11022302462516e-16 5.03662826618488e-16 100
"chr17" 71381001 71382000 "*" 0 0 -55.7728011139476
"chr17" 71392001 71393000 "*" 8.43347613965761e-12 2.17859001990012e-11 100
"chr17" 71436001 71437000 "*" 2.32063907467106e-05 2.4047079113211e-05
65.2173913043478
"chr17" 71439001 71440000 "*" 8.28337398672829e-13 2.43270358389251e-12
86.1111111111111
"chr17" 71446001 71447000 "*" 5.55111512312578e-16 2.36485094870365e-15
-78.1818181818182
"chr17" 71467001 71468000 "*" 0.000496254927290996 0.000414667937798419
54.1666666666667
"chr17" 71473001 71474000 "*" 2.7284623449475e-09 4.9588280682559e-09
75.9507829977629
"chr17" 71477001 71478000 "*" 0.000115274730568937 0.000107060060050355
-54.1666666666667
"chr17" 71483001 71484000 "*" 0.000129561109398435 0.000119284304019944
55.5555555555556
"chr17" 71486001 71487000 "*" 0 0 100
"chr17" 71491001 71492000 "*" 3.86647271599649e-06 4.50435143635178e-06
63.768115942029
"chr17" 71503001 71504000 "*" 1.48087875295744e-10 3.22075573380618e-10 100
"chr17" 71515001 71516000 "*" 1.65900401183361e-06 2.04236557510993e-06
-66.6666666666667
"chr17" 71528001 71529000 "*" 8.11041367398957e-09 1.3788893142866e-08
85.8974358974359
"chr17" 71536001 71537000 "*" 9.28610585204659e-07 1.18407475076477e-06
54.5454545454545
"chr17" 71558001 71559000 "*" 2.88247958835086e-09 5.22417381987932e-09
62.962962962963
"chr17" 71567001 71568000 "*" 1.11022302462516e-16 5.03662826618488e-16
53.6796536796537
"chr17" 71571001 71572000 "*" 3.1457036075988e-07 4.27293435984224e-07
51.8518518518519
"chr17" 71575001 71576000 "*" 3.95353005888666e-09 7.03928529283733e-09 -100
"chr17" 71605001 71606000 "*" 0 0 100
"chr17" 71608001 71609000 "*" 5.81391379483875e-10 1.16600808611588e-09
69.3333333333333
"chr17" 71610001 71611000 "*" 4.01313771103418e-09 7.11882087237587e-09 100

"chr17" 71621001 71622000 "*" 3.3734396609475e-08 5.2442256253974e-08
57.8947368421053
"chr17" 71640001 71641000 "*" 0 0 55.1551292023161
"chr17" 71706001 71707000 "*" 4.32986979603811e-14 1.49109818638045e-13 75
"chr17" 71730001 71731000 "*" 0 0 100
"chr17" 71737001 71738000 "*" 8.58397502967456e-07 1.0995215865911e-06
55.5555555555556
"chr17" 71743001 71744000 "*" 0 0 100
"chr17" 71750001 71751000 "*" 4.3327895901113e-05 4.30223604219843e-05
61.4035087719298
"chr17" 71760001 71761000 "*" 1.98325800226939e-11 4.85612402473442e-11 100
"chr17" 71762001 71763000 "*" 4.07152041598247e-05 4.0578340745223e-05
60.5263157894737
"chr17" 71773001 71774000 "*" 1.52100632089258e-09 2.8746597910115e-09
-63.1578947368421
"chr17" 71778001 71779000 "*" 0 0 86.4
"chr17" 71780001 71781000 "*" 1.98365768255826e-11 4.85612402473442e-11 100
"chr17" 71789001 71790000 "*" 1.11022302462516e-15 4.59817122935606e-15 -100
"chr17" 71797001 71798000 "*" 0 0 100
"chr17" 71815001 71816000 "*" 2.37587727269783e-14 8.44268514944447e-14 100
"chr17" 71819001 71820000 "*" 1.46882506157908e-13 4.73433947293732e-13
-59.5238095238095
"chr17" 71845001 71846000 "*" 9.22717469098444e-09 1.55374184879414e-08
70.5882352941177
"chr17" 71939001 71940000 "*" 4.48406142217195e-08 6.84482503893299e-08
-66.6666666666667
"chr17" 71945001 71946000 "*" 3.88022947106492e-13 1.1867462770515e-12 100
"chr17" 71970001 71971000 "*" 5.32140997933084e-12 1.4110686161061e-11
62.8877005347594
"chr17" 71973001 71974000 "*" 2.24771978807325e-10 4.77766895832731e-10
-52.3809523809524
"chr17" 71983001 71984000 "*" 5.63660229602192e-13 1.68905058935478e-12 -100
"chr17" 72024001 72025000 "*" 0 0 70.2970297029703
"chr17" 72027001 72028000 "*" 1.60913504743121e-11 3.99985051484648e-11
-84.5528455284553
"chr17" 72044001 72045000 "*" 4.01313771103418e-09 7.11882087237587e-09 -100
"chr17" 72046001 72047000 "*" 5.794809077031e-12 1.52911192757929e-11 100
"chr17" 72059001 72060000 "*" 1.33898878355021e-05 1.43911695897696e-05
56.5217391304348
"chr17" 72108001 72109000 "*" 2.64951482975562e-09 4.82186011375752e-09 100
"chr17" 72116001 72117000 "*" 1.39779633423487e-07 1.98463037958589e-07 100
"chr17" 72126001 72127000 "*" 1.54321000422897e-14 5.6024372055589e-14
66.6666666666667
"chr17" 72149001 72150000 "*" 5.56368978665844e-08 8.38936188628635e-08
78.4313725490196
"chr17" 72159001 72160000 "*" 2.44805405946735e-08 3.87506495191586e-08 68.75
"chr17" 72160001 72161000 "*" 3.04216651869638e-11 7.26599935673121e-11 -65
"chr17" 72179001 72180000 "*" 0 0 75
"chr17" 72191001 72192000 "*" 4.41652459048925e-09 7.80086469207946e-09
59.4202898550725
"chr17" 72211001 72212000 "*" 0 0 100
"chr17" 72264001 72265000 "*" 9.06273663336954e-07 1.15739767942151e-06 -75

"chr17" 72281001 72282000 "*" 4.44089209850063e-16 1.91071758245033e-15
-50.7262474367737
"chr17" 72318001 72319000 "*" 0 0 100
"chr17" 72319001 72320000 "*" 1.11023489957063e-07 1.60098147216736e-07
66.6666666666667
"chr17" 72322001 72323000 "*" 0 0 59.9722513613995
"chr17" 72333001 72334000 "*" 3.25818809512324e-05 3.29735679006858e-05
-66.6666666666667
"chr17" 72334001 72335000 "*" 7.0006535457523e-08 1.03695832512054e-07 100
"chr17" 72335001 72336000 "*" 0 0 87.9227053140097
"chr17" 72344001 72345000 "*" 0 0 78.3783783783784
"chr17" 72349001 72350000 "*" 0 0 66.6666666666667
"chr17" 72355001 72356000 "*" 1.48665846300133e-09 2.81344116111208e-09 -90.625
"chr17" 72372001 72373000 "*" 1.70641278884887e-12 4.81986032810646e-12 100
"chr17" 72375001 72376000 "*" 7.9984455014781e-06 8.89173797399106e-06
66.6666666666667
"chr17" 72376001 72377000 "*" 8.16788192636864e-10 1.60340601488839e-09
-55.4123711340206
"chr17" 72380001 72381000 "*" 3.51507812190732e-07 4.74284456278014e-07
75.3846153846154
"chr17" 72385001 72386000 "*" 4.73234496034536e-09 8.30627300264826e-09 100
"chr17" 72387001 72388000 "*" 6.08402217494586e-14 2.05454787937634e-13 100
"chr17" 72426001 72427000 "*" 0 0 52.6296398636824
"chr17" 72431001 72432000 "*" 2.57998289399097e-10 5.44285461585871e-10
-81.6091954022989
"chr17" 72439001 72440000 "*" 0 0 -75
"chr17" 72463001 72464000 "*" 1.22202233359126e-05 1.32089367705288e-05
-60.9090909090909
"chr17" 72473001 72474000 "*" 6.24654217240561e-10 1.2462108191303e-09 100
"chr17" 72522001 72523000 "*" 5.07028752316785e-10 1.02527764830995e-09
62.4084249084249
"chr17" 72530001 72531000 "*" 3.04324343503026e-11 7.26599935673121e-11 100
"chr17" 72535001 72536000 "*" 1.46831780067913e-09 2.78024670726801e-09 -80
"chr17" 72538001 72539000 "*" 0 0 59.5533498759305
"chr17" 72546001 72547000 "*" 6.39294062132478e-10 1.27392469716478e-09
64.7058823529412
"chr17" 72557001 72558000 "*" 2.09024626711685e-05 2.18111747761494e-05
57.5757575757576
"chr17" 72564001 72565000 "*" 1.43710821021159e-10 3.13414116292349e-10
61.7647058823529
"chr17" 72600001 72601000 "*" 4.26951363152739e-10 8.72510873516957e-10 -100
"chr17" 72615001 72616000 "*" 0 0 100
"chr17" 72616001 72617000 "*" 1.29037891483108e-11 3.24582280286866e-11 -100
"chr17" 72642001 72643000 "*" 7.54951656745106e-14 2.5237892556906e-13 100
"chr17" 72691001 72692000 "*" 0 0 -55.8602588469545
"chr17" 72694001 72695000 "*" 1.12458486967171e-09 2.15740815043925e-09 100
"chr17" 72717001 72718000 "*" 1.37828859436695e-10 3.01445544267661e-10 -100
"chr17" 72728001 72729000 "*" 1.89848137210902e-14 6.82212372863451e-14
-86.3013698630137
"chr17" 72733001 72734000 "*" 0 0 63.1934032983508
"chr17" 72767001 72768000 "*" 8.92637418925535e-06 9.85381833193747e-06
-56.5217391304348

"chr17" 72773001 72774000 "*" 9.87908865823783e-10 1.91116624416613e-09 54.3735224586288
"chr17" 72776001 72777000 "*" 3.0115909765982e-12 8.24098649635605e-12 56.5217391304348
"chr17" 72778001 72779000 "*" 9.99200722162641e-16 4.15382497462808e-15 -98.9795918367347
"chr17" 72787001 72788000 "*" 0 0 100
"chr17" 72847001 72848000 "*" 9.8411390148101e-11 2.19600763293869e-10 83.6065573770492
"chr17" 72867001 72868000 "*" 2.22044604925031e-16 9.81641919380259e-16 -100
"chr17" 72874001 72875000 "*" 1.3933182574366e-07 1.98398036179707e-07 -77.2727272727273
"chr17" 72876001 72877000 "*" 0 0 100
"chr17" 72886001 72887000 "*" 6.92287338566189e-11 1.57605630124247e-10 100
"chr17" 72912001 72913000 "*" 4.32961111407337e-10 8.84315589159417e-10 60.7594936708861
"chr17" 72923001 72924000 "*" 0 0 70.5882352941177
"chr17" 72929001 72930000 "*" 3.44169137633799e-15 1.3517917469209e-14 63.0952380952381
"chr17" 72930001 72931000 "*" 1.38590361409285e-10 3.02706178892116e-10 100
"chr17" 72934001 72935000 "*" 1.11022302462516e-16 5.03662826618488e-16 -52.0979020979021
"chr17" 72945001 72946000 "*" 2.18561220233227e-08 3.481380123874e-08 -81.8840579710145
"chr17" 72970001 72971000 "*" 2.68450373042128e-10 5.64465213127486e-10 100
"chr17" 72977001 72978000 "*" 0 0 100
"chr17" 73075001 73076000 "*" 0 0 -100
"chr17" 73084001 73085000 "*" 0 0 -92.202462380301
"chr17" 73120001 73121000 "*" 0 0 -100
"chr17" 73130001 73131000 "*" 0.000128040980536093 0.000118003288696945 60
"chr17" 73147001 73148000 "*" 9.98963367582206e-09 1.67533179897223e-08 66.6666666666667
"chr17" 73179001 73180000 "*" 0 0 -56.5560263653484
"chr17" 73226001 73227000 "*" 7.00224100924629e-09 1.20091258409783e-08 -65.0793650793651
"chr17" 73233001 73234000 "*" 1.51323398256409e-13 4.86995056237989e-13 -100
"chr17" 73370001 73371000 "*" 1.38590361409285e-10 3.02706178892116e-10 100
"chr17" 73510001 73511000 "*" 7.99132354145637e-06 8.88654452816447e-06 51.5151515151515
"chr17" 73520001 73521000 "*" 0 0 100
"chr17" 73526001 73527000 "*" 4.19153467035027e-06 4.85557211020648e-06 70.2702702702703
"chr17" 73527001 73528000 "*" 0 0 -66.7189952904239
"chr17" 73549001 73550000 "*" 5.62883073484954e-13 1.68905058935478e-12 100
"chr17" 73553001 73554000 "*" 5.04263297784746e-13 1.52078941532818e-12 -100
"chr17" 73572001 73573000 "*" 0 0 100
"chr17" 73607001 73608000 "*" 0 0 63.855421686747
"chr17" 73628001 73629000 "*" 4.97124563736406e-12 1.32423696369647e-11 64.1025641025641
"chr17" 73655001 73656000 "*" 0.000136199988891583 0.000124970284403136 63.6363636363636
"chr17" 73658001 73659000 "*" 0 0 62.8571428571429

"chr17" 73685001 73686000 "*" 3.94216216874632e-05 3.93770971245002e-05
-71.4285714285714
"chr17" 73697001 73698000 "*" 0.000299166032473375 0.000259267996470385
-54.1666666666667
"chr17" 73760001 73761000 "*" 0 0 97.2222222222222
"chr17" 73774001 73775000 "*" 7.9984455014781e-06 8.89173797399106e-06
-66.6666666666667
"chr17" 73781001 73782000 "*" 8.87728492215967e-06 9.80274272388281e-06
-61.2903225806452
"chr17" 73844001 73845000 "*" 0 0 -60.8108108108108
"chr17" 73852001 73853000 "*" 0 0 100
"chr17" 73855001 73856000 "*" 1.12458486967171e-09 2.15740815043925e-09 100
"chr17" 73861001 73862000 "*" 1.5277158427196e-09 2.87642564232045e-09 100
"chr17" 73868001 73869000 "*" 7.99154248332101e-08 1.17422211196405e-07
-53.6379769299024
"chr17" 73869001 73870000 "*" 2.57151299221103e-06 3.07817914855153e-06
-51.1111111111111
"chr17" 73873001 73874000 "*" 0 0 -74.3779421654338
"chr17" 74023001 74024000 "*" 0 0 -74.8898678414097
"chr17" 74044001 74045000 "*" 4.55191440096314e-15 1.76090186737198e-14
68.5714285714286
"chr17" 74076001 74077000 "*" 2.72323275041231e-10 5.72248001700952e-10
52.6960784313725
"chr17" 74111001 74112000 "*" 2.00227675550835e-08 3.20459176617961e-08 -100
"chr17" 74137001 74138000 "*" 0 0 83.1476323119777
"chr17" 74194001 74195000 "*" 7.88258347483861e-15 2.96145301916012e-14 100
"chr17" 74302001 74303000 "*" 2.88710388929303e-10 6.03399264731139e-10 -100
"chr17" 74419001 74420000 "*" 1.37828859436695e-10 3.01445544267661e-10 100
"chr17" 74502001 74503000 "*" 2.71893618730701e-13 8.46692454027087e-13 -100
"chr17" 74515001 74516000 "*" 6.66133814775094e-16 2.81595744474255e-15
63.4146341463415
"chr17" 74575001 74576000 "*" 0 0 100
"chr17" 74580001 74581000 "*" 0 0 -81.4432989690722
"chr17" 74674001 74675000 "*" 0 0 100
"chr17" 74690001 74691000 "*" 4.18887147191072e-13 1.2739946038722e-12 100
"chr17" 74694001 74695000 "*" 1.06467442639868e-08 1.77888030305838e-08
-56.3194077207826
"chr17" 74810001 74811000 "*" 1.97126177603479e-08 3.16979535786496e-08 -90
"chr17" 74830001 74831000 "*" 1.37001521238744e-13 4.42851918810123e-13 100
"chr17" 74831001 74832000 "*" 0 0 100
"chr17" 74840001 74841000 "*" 5.44949974035092e-10 1.09833754587107e-09
-65.9090909090909
"chr17" 74862001 74863000 "*" 1.49613654798486e-12 4.26244554429779e-12 100
"chr17" 74863001 74864000 "*" 8.07476308040123e-12 2.09149622277287e-11
50.7453416149068
"chr17" 74873001 74874000 "*" 4.14701606388235e-11 9.72238235993468e-11
-56.1728395061728
"chr17" 74874001 74875000 "*" 1.5277158427196e-09 2.87642564232045e-09 -100
"chr17" 74876001 74877000 "*" 7.0006535457523e-08 1.03695832512054e-07 -100
"chr17" 74879001 74880000 "*" 9.04570973681018e-10 1.76414389822173e-09 100
"chr17" 74881001 74882000 "*" 5.01025332333427e-10 1.01358031135889e-09 100
"chr17" 74895001 74896000 "*" 1.3968096701511e-07 1.98463037958589e-07

62.0689655172414
"chr17" 74897001 74898000 "*" 3.46167539078124e-13 1.06762125683752e-12
58.1818181818182
"chr17" 74898001 74899000 "*" 0 0 -52.5179856115108
"chr17" 74901001 74902000 "*" 0.000103130229750614 9.6490161990646e-05
55.5555555555556
"chr17" 74953001 74954000 "*" 0 0 100
"chr17" 74965001 74966000 "*" 0 0 56.5074584236696
"chr17" 74966001 74967000 "*" 3.6700841921089e-08 5.65530226072258e-08 100
"chr17" 74977001 74978000 "*" 0 0 100
"chr17" 74986001 74987000 "*" 0 0 100
"chr17" 75030001 75031000 "*" 5.99520433297585e-15 2.28531569014092e-14
-73.0769230769231
"chr17" 75099001 75100000 "*" 9.63829016598083e-12 2.46993429847588e-11 -100
"chr17" 75151001 75152000 "*" 1.35447209004269e-14 4.94674708073053e-14 -100
"chr17" 75188001 75189000 "*" 2.22044604925031e-16 9.81641919380259e-16 -100
"chr17" 75193001 75194000 "*" 0 0 -100
"chr17" 75220001 75221000 "*" 7.21991201602101e-07 9.34805623305556e-07
-64.7619047619048
"chr17" 75276001 75277000 "*" 0 0 -61.9834710743802
"chr17" 75281001 75282000 "*" 4.92986762523628e-11 1.1455002254285e-10
-90.9090909090909
"chr17" 75313001 75314000 "*" 2.75335310107039e-14 9.69727903855348e-14 100
"chr17" 75366001 75367000 "*" 4.9960036108132e-15 1.9234186548721e-14 96.875
"chr17" 75437001 75438000 "*" 1.1207701433591e-12 3.24052733919139e-12 75
"chr17" 75447001 75448000 "*" 0 0 84.0956340956341
"chr17" 75521001 75522000 "*" 9.08108033215171e-11 2.03407753381119e-10 100
"chr17" 75527001 75528000 "*" 1.14124265593318e-11 2.89138062669327e-11 100
"chr17" 75530001 75531000 "*" 3.38408899026588e-05 3.41565268180454e-05
63.3333333333333
"chr17" 75537001 75538000 "*" 2.16382467499443e-13 6.82775854166559e-13 100
"chr17" 75560001 75561000 "*" 0 0 -100
"chr17" 75580001 75581000 "*" 7.105427357601e-15 2.68362283258525e-14 100
"chr17" 75607001 75608000 "*" 1.55431223447522e-14 5.6388152448458e-14 -100
"chr17" 75610001 75611000 "*" 1.28785870856518e-14 4.71440608755782e-14 -100
"chr17" 75673001 75674000 "*" 1.36169530096097e-08 2.2387749553592e-08
-70.5882352941177
"chr17" 75689001 75690000 "*" 0 0 100
"chr17" 75693001 75694000 "*" 1.11022302462516e-16 5.03662826618488e-16
50.758389261745
"chr17" 75719001 75720000 "*" 1.5683010445855e-12 4.4469621057462e-12
-52.8571428571429
"chr17" 75731001 75732000 "*" 1.11022302462516e-16 5.03662826618488e-16 100
"chr17" 75732001 75733000 "*" 1.36146649509783e-12 3.89647762918394e-12
70.1408450704225
"chr17" 75736001 75737000 "*" 2.68450373042128e-10 5.64465213127486e-10 100
"chr17" 75739001 75740000 "*" 7.03992419914812e-12 1.83313429961219e-11 -100
"chr17" 75752001 75753000 "*" 4.25088853006628e-11 9.95750663262604e-11
66.6666666666667
"chr17" 75764001 75765000 "*" 0 0 -81.9277108433735
"chr17" 75770001 75771000 "*" 0 0 52.0833333333333
"chr17" 75777001 75778000 "*" 4.10798062233653e-11 9.63411638942903e-11

87.8260869565217
"chr17" 75780001 75781000 "*" 1.86339288443804e-09 3.47809554833057e-09
55.2795031055901
"chr17" 75788001 75789000 "*" 1.05065132616211e-06 1.33001006138239e-06 87.5
"chr17" 75801001 75802000 "*" 1.89519511195613e-11 4.67247822013431e-11
57.4786324786325
"chr17" 75820001 75821000 "*" 0 0 100
"chr17" 75830001 75831000 "*" 1.48087875295744e-10 3.22075573380618e-10 100
"chr17" 75847001 75848000 "*" 1.1140205091742e-08 1.8567534703664e-08
61.7647058823529
"chr17" 75852001 75853000 "*" 0 0 60.9375
"chr17" 75865001 75866000 "*" 6.30384633382164e-13 1.87665599796749e-12 100
"chr17" 75883001 75884000 "*" 0 0 89.4413407821229
"chr17" 75884001 75885000 "*" 0 0 61.6683293684291
"chr17" 75885001 75886000 "*" 8.80406858527749e-14 2.91456141063452e-13 -100
"chr17" 75890001 75891000 "*" 4.77395900588817e-15 1.84201484337652e-14
98.1132075471698
"chr17" 75896001 75897000 "*" 0 0 70.3125
"chr17" 75899001 75900000 "*" 0 0 -100
"chr17" 75904001 75905000 "*" 2.41824383806488e-06 2.90722934768982e-06
52.9411764705882
"chr17" 75906001 75907000 "*" 6.24654217240561e-10 1.2462108191303e-09 -100
"chr17" 75920001 75921000 "*" 9.65338919911574e-13 2.80678910656113e-12 100
"chr17" 75923001 75924000 "*" 0 0 100
"chr17" 75932001 75933000 "*" 2.02060590481778e-13 6.40127405465654e-13
-81.9672131147541
"chr17" 75936001 75937000 "*" 0 0 77.7777777777778
"chr17" 75938001 75939000 "*" 3.41306332307667e-07 4.61505309661803e-07
-60.6060606060606
"chr17" 75942001 75943000 "*" 1.11022302462516e-15 4.59817122935606e-15
-75.609756097561
"chr17" 75953001 75954000 "*" 0 0 87.3968070146414
"chr17" 76124001 76125000 "*" 0 0 -100
"chr17" 76136001 76137000 "*" 0 0 -58.1168831168831
"chr17" 76209001 76210000 "*" 0 0 -100
"chr17" 76221001 76222000 "*" 1.94637639339135e-11 4.78437760541728e-11
51.219512195122
"chr17" 76237001 76238000 "*" 1.56863411149288e-12 4.4469621057462e-12 -100
"chr17" 76250001 76251000 "*" 0 0 65.8682634730539
"chr17" 76285001 76286000 "*" 1.11022302462516e-16 5.03662826618488e-16
77.4193548387097
"chr17" 76300001 76301000 "*" 0 0 65.8730158730159
"chr17" 76302001 76303000 "*" 3.6700841921089e-08 5.65530226072258e-08 100
"chr17" 76306001 76307000 "*" 3.530509218308e-14 1.22891039008692e-13 100
"chr17" 76341001 76342000 "*" 1.37001521238744e-13 4.42851918810123e-13 100
"chr17" 76383001 76384000 "*" 9.65729496371637e-10 1.87255912493232e-09 100
"chr17" 76429001 76430000 "*" 0 0 56.25
"chr17" 76439001 76440000 "*" 0 0 55.7320099255583
"chr17" 76454001 76455000 "*" 0 0 52.2935779816514
"chr17" 76456001 76457000 "*" 0 0 53.2638136511376
"chr17" 76459001 76460000 "*" 1.83466277617894e-05 1.93097202505878e-05 65
"chr17" 76463001 76464000 "*" 0 0 51.1518717916615

"chr17" 76478001 76479000 "*" 7.05910274123589e-07 9.14808391757987e-07 75
"chr17" 76479001 76480000 "*" 2.18935980456081e-13 6.89554885263635e-13 60.9375
"chr17" 76502001 76503000 "*" 0 0 75.8620689655172
"chr17" 76527001 76528000 "*" 0 0 100
"chr17" 76540001 76541000 "*" 0 0 99.236641221374
"chr17" 76547001 76548000 "*" 0 0 100
"chr17" 76560001 76561000 "*" 4.18887147191072e-13 1.2739946038722e-12 100
"chr17" 76577001 76578000 "*" 1.32893696047631e-13 4.30574836768249e-13
60.7142857142857
"chr17" 76599001 76600000 "*" 7.0006535457523e-08 1.03695832512054e-07 -100
"chr17" 76606001 76607000 "*" 3.37330363642252e-09 6.06824283611688e-09
-66.6666666666667
"chr17" 76614001 76615000 "*" 3.60139912947144e-06 4.21783752267897e-06
68.6274509803922
"chr17" 76718001 76719000 "*" 0 0 -90
"chr17" 76777001 76778000 "*" 0 0 -100
"chr17" 76832001 76833000 "*" 4.01313771103418e-09 7.11882087237587e-09 100
"chr17" 76835001 76836000 "*" 3.39864003328083e-05 3.42934337751028e-05
-53.469387755102
"chr17" 76912001 76913000 "*" 4.80888995291195e-10 9.76301786084297e-10
-53.0577956989247
"chr17" 76913001 76914000 "*" 1.98294731001969e-06 2.41330824932622e-06
-56.5217391304348
"chr17" 76958001 76959000 "*" 3.34128893442198e-08 5.19772864618523e-08 -100
"chr17" 76965001 76966000 "*" 4.57347782978346e-08 6.97544417869494e-08 60
"chr17" 77020001 77021000 "*" 0 0 64.850136239782
"chr17" 77039001 77040000 "*" 1.0711986186962e-08 1.78931505522321e-08
-85.1851851851852
"chr17" 77054001 77055000 "*" 4.01960244311717e-07 5.38015800508855e-07
88.8888888888889
"chr17" 77073001 77074000 "*" 2.56220224814641e-07 3.52192789782665e-07
64.7058823529412
"chr17" 77083001 77084000 "*" 0 0 -83.8888888888889
"chr17" 77092001 77093000 "*" 0 0 82.7067669172932
"chr17" 77098001 77099000 "*" 2.92777766650598e-05 2.98674924724064e-05 -60
"chr17" 77106001 77107000 "*" 0 0 100
"chr17" 77108001 77109000 "*" 0 0 56.25
"chr17" 77120001 77121000 "*" 2.23598917159507e-13 7.03895577426956e-13
-86.6666666666667
"chr17" 77123001 77124000 "*" 0.000451115314036077 0.000379477851858741
-52.1739130434783
"chr17" 77131001 77132000 "*" 0 0 66.6666666666667
"chr17" 77132001 77133000 "*" 0 0 63.4146341463415
"chr17" 77142001 77143000 "*" 2.88710388929303e-10 6.03399264731139e-10 -100
"chr17" 77143001 77144000 "*" 2.55351295663786e-15 1.01678090381833e-14
-80.9523809523809
"chr17" 77145001 77146000 "*" 0 0 88.6363636363636
"chr17" 77146001 77147000 "*" 3.28779502212218e-05 3.32562456142348e-05
59.0163934426229
"chr17" 77148001 77149000 "*" 0 0 100
"chr17" 77166001 77167000 "*" 0 0 69.6416497633536
"chr17" 77167001 77168000 "*" 0 0 100

"chr17" 77181001 77182000 "*" 5.03149832227212e-05 4.94695573148981e-05 58.3333333333333
"chr17" 77189001 77190000 "*" 6.59550958292954e-09 1.13456194382773e-08 100
"chr17" 77197001 77198000 "*" 2.07416611541777e-06 2.51848093026003e-06 66.4383561643836
"chr17" 77204001 77205000 "*" 0 0 100
"chr17" 77208001 77209000 "*" 8.95782606414874e-05 8.46224089124224e-05 51.6129032258064
"chr17" 77215001 77216000 "*" 0 0 66.0714285714286
"chr17" 77220001 77221000 "*" 4.99900121297969e-12 1.32921056339624e-11 100
"chr17" 77229001 77230000 "*" 6.4152538836737e-10 1.27477697728417e-09 100
"chr17" 77235001 77236000 "*" 0.000808777120367532 0.000651834190530565 -58.3333333333333
"chr17" 77240001 77241000 "*" 1.67299207820548e-08 2.71377429441055e-08 100
"chr17" 77247001 77248000 "*" 4.44089209850063e-16 1.91071758245033e-15 79.1666666666667
"chr17" 77251001 77252000 "*" 3.33066907387547e-16 1.4495649018245e-15 80.8510638297872
"chr17" 77261001 77262000 "*" 7.50202344690365e-10 1.47864532815326e-09 67.816091954023
"chr17" 77266001 77267000 "*" 1.76568204501848e-10 3.81233627207447e-10 72.7272727272727
"chr17" 77270001 77271000 "*" 2.99760216648792e-14 1.05188753281006e-13 75
"chr17" 77282001 77283000 "*" 2.50661865708324e-06 3.00507055123819e-06 72.7272727272727
"chr17" 77285001 77286000 "*" 2.23421281475567e-09 4.11871828003941e-09 66.6666666666667
"chr17" 77306001 77307000 "*" 2.55029330986645e-12 7.05015875633984e-12 95.9183673469388
"chr17" 77322001 77323000 "*" 4.75401816801835e-08 7.23228683045732e-08 -62.5
"chr17" 77332001 77333000 "*" 2.20637952352831e-11 5.37689828600441e-11 75.5102040816327
"chr17" 77356001 77357000 "*" 0 0 81.9718309859155
"chr17" 77357001 77358000 "*" 1.22124532708767e-15 5.03921984217778e-15 57.3728354978355
"chr17" 77358001 77359000 "*" 3.48832074337224e-13 1.07440867586637e-12 51.2
"chr17" 77371001 77372000 "*" 3.51451824642801e-07 4.74216585123463e-07 71.2328767123288
"chr17" 77388001 77389000 "*" 6.80929423957366e-10 1.34883472781556e-09 66.1971830985916
"chr17" 77390001 77391000 "*" 0 0 -60.8098458436594
"chr17" 77398001 77399000 "*" 0 0 61.1950873878129
"chr17" 77400001 77401000 "*" 1.24300569837033e-11 3.13782739818966e-11 62.9353233830846
"chr17" 77405001 77406000 "*" 1.16651133197365e-12 3.36577955750396e-12 60.2593440122044
"chr17" 77412001 77413000 "*" 1.1465273175304e-12 3.3099511016938e-12 86.7816091954023
"chr17" 77427001 77428000 "*" 8.94966323272683e-11 2.0105270067434e-10 53.0120481927711
"chr17" 77429001 77430000 "*" 1.83952852950142e-12 5.17499701329218e-12 -100
"chr17" 77432001 77433000 "*" 5.55111512312578e-16 2.36485094870365e-15

60.4651162790698
"chr17" 77439001 77440000 "*" 0.000555237812773224 0.00045982126931809 -56.1290322580645
"chr17" 77445001 77446000 "*" 3.8350339248705e-06 4.47016142430037e-06 -56.5217391304348
"chr17" 77447001 77448000 "*" 7.7715611723761e-16 3.26213507634405e-15 63.7305699481865
"chr17" 77450001 77451000 "*" 2.1919348114352e-08 3.4905844914427e-08 81.8181818181818
"chr17" 77534001 77535000 "*" 9.04570973681018e-10 1.76414389822173e-09 100
"chr17" 77667001 77668000 "*" 1.03992122135743e-07 1.50583642361949e-07 -54.4444444444444
"chr17" 77695001 77696000 "*" 1.5277158427196e-09 2.87642564232045e-09 100
"chr17" 77715001 77716000 "*" 0 0 -75.609756097561
"chr17" 77718001 77719000 "*" 3.34128893442198e-08 5.19772864618523e-08 100
"chr17" 77726001 77727000 "*" 0 0 -61.9718309859155
"chr17" 77734001 77735000 "*" 0 0 -83.3333333333333
"chr17" 77774001 77775000 "*" 0 0 53.1476449275362
"chr17" 77783001 77784000 "*" 1.01230135385322e-12 2.93913449798872e-12 50.509337860781
"chr17" 77785001 77786000 "*" 0 0 -51.6433579879761
"chr17" 77792001 77793000 "*" 4.32209823486573e-12 1.15935290479641e-11 100
"chr17" 77825001 77826000 "*" 4.21884749357559e-15 1.63874445239189e-14 52.3809523809524
"chr17" 77833001 77834000 "*" 0 0 -98.0582524271845
"chr17" 77866001 77867000 "*" 0 0 -100
"chr17" 77892001 77893000 "*" 0 0 -63.1578947368421
"chr17" 77908001 77909000 "*" 1.61204383175573e-13 5.17337152201509e-13 -59.2592592592593
"chr17" 77962001 77963000 "*" 0 0 -70.0476585163697
"chr17" 77965001 77966000 "*" 2.22044604925031e-16 9.81641919380259e-16 54.3666666666667
"chr17" 78000001 78001000 "*" 6.37490060739765e-13 1.89496793587877e-12 -100
"chr17" 78134001 78135000 "*" 0 0 100
"chr17" 78140001 78141000 "*" 2.62123656113999e-13 8.19942075318906e-13 67.5
"chr17" 78141001 78142000 "*" 4.7482617837602e-10 9.64533069050774e-10 95.8333333333333
"chr17" 78203001 78204000 "*" 0.000628813571299403 0.000515972945757495 51.8518518518519
"chr17" 78233001 78234000 "*" 0 0 -55.308608597465
"chr17" 78235001 78236000 "*" 0 0 74.4186046511628
"chr17" 78266001 78267000 "*" 7.3323102589562e-08 1.08271916783818e-07 70.5882352941177
"chr17" 78407001 78408000 "*" 1.01816888253836e-10 2.26854013775798e-10 78
"chr17" 78412001 78413000 "*" 0 0 -68
"chr17" 78440001 78441000 "*" 1.16709751862842e-08 1.93439349917952e-08 51.0143493320139
"chr17" 78448001 78449000 "*" 0 0 -100
"chr17" 78449001 78450000 "*" 0 0 51.0124329911887
"chr17" 78450001 78451000 "*" 0 0 62.4350873745913
"chr17" 78464001 78465000 "*" 2.74357321194962e-07 3.75674653680942e-07 -78.0821917808219

"chr17" 78483001 78484000 "*" 0 0 100
"chr17" 78494001 78495000 "*" 5.66546809466217e-13 1.69721259969255e-12
-58.3333333333333
"chr17" 78495001 78496000 "*" 0 0 100
"chr17" 78708001 78709000 "*" 0 0 -100
"chr17" 78785001 78786000 "*" 0 0 -100
"chr17" 78794001 78795000 "*" 0 0 100
"chr17" 78830001 78831000 "*" 5.10702591327572e-15 1.96271840288234e-14 -100
"chr17" 78865001 78866000 "*" 0 0 -100
"chr17" 78978001 78979000 "*" 0 0 70.0669184485259
"chr17" 79019001 79020000 "*" 2.74421438906103e-08 4.32073612765642e-08
78.8135593220339
"chr17" 79023001 79024000 "*" 0 0 -52.3281272806889
"chr17" 79075001 79076000 "*" 0 0 54.0740740740741
"chr17" 79099001 79100000 "*" 0 0 -61.6402116402116
"chr17" 79127001 79128000 "*" 9.76996261670138e-15 3.63402770315216e-14
-57.6923076923077
"chr17" 79140001 79141000 "*" 0 0 91.7864476386037
"chr17" 79141001 79142000 "*" 2.22065652533132e-08 3.53369143773134e-08
-75.4716981132076
"chr17" 79179001 79180000 "*" 1.20591092667155e-10 2.66075324129686e-10 100
"chr17" 79212001 79213000 "*" 0 0 -97.8723404255319
"chr17" 79303001 79304000 "*" 0 0 -100
"chr17" 79309001 79310000 "*" 8.43293023078395e-07 1.08128691154327e-06
-55.8139534883721
"chr17" 79339001 79340000 "*" 0 0 -81.371087928465
"chr17" 79370001 79371000 "*" 0 0 64.0711902113459
"chr17" 79373001 79374000 "*" 0 0 -73.8662002607988
"chr17" 79393001 79394000 "*" 0 0 -58.5806525779319
"chr17" 79399001 79400000 "*" 2.13750933140133e-05 2.22723064452095e-05
60.3174603174603
"chr17" 79459001 79460000 "*" 0 0 -88.030888030888
"chr17" 79486001 79487000 "*" 0 0 -71.7631995179634
"chr17" 79520001 79521000 "*" 0 0 -79.4642857142857
"chr17" 79597001 79598000 "*" 2.04988059859801e-09 3.80458088311340e-09
66.6666666666667
"chr17" 79604001 79605000 "*" 0 0 100
"chr17" 79609001 79610000 "*" 5.44009282066327e-15 2.08486137681812e-14 100
"chr17" 79617001 79618000 "*" 4.53591360638494e-06 5.23031683672762e-06 90
"chr17" 79678001 79679000 "*" 0 0 83.0028328611898
"chr17" 79680001 79681000 "*" 0 0 -60.9375
"chr17" 79691001 79692000 "*" 0 0 100
"chr17" 79760001 79761000 "*" 1.32993616119848e-12 3.81448527302964e-12
55.1448551448551
"chr17" 79788001 79789000 "*" 8.60029936156081e-10 1.6836556051411e-09 90
"chr17" 79796001 79797000 "*" 9.0649709960644e-13 2.64779497454508e-12
56.5217391304348
"chr17" 79817001 79818000 "*" 2.23154827949656e-14 7.95931582053377e-14 100
"chr17" 79824001 79825000 "*" 5.20672394088706e-12 1.381711867121e-11
60.7142857142857
"chr17" 79876001 79877000 "*" 3.43665623292466e-07 4.64522312106388e-07
74.025974025974

"chr17" 79894001 79895000 "*" 0 0 59.1865357643759
"chr17" 79961001 79962000 "*" 0 0 -54.8670178316998
"chr17" 79979001 79980000 "*" 0 0 -88.6075949367089
"chr17" 80059001 80060000 "*" 0 0 100
"chr17" 80111001 80112000 "*" 1.11022302462516e-16 5.03662826618488e-16 -100
"chr17" 80163001 80164000 "*" 2.22044604925031e-16 9.81641919380259e-16
-57.681420483788
"chr17" 80187001 80188000 "*" 0 0 -50.1904761904762
"chr17" 80188001 80189000 "*" 0 0 -78.6192598565918
"chr17" 80225001 80226000 "*" 3.46600526057728e-12 9.42600366624647e-12
-55.5555555555556
"chr17" 80226001 80227000 "*" 6.25236642792792e-07 8.16567308913579e-07
69.5652173913043
"chr17" 80230001 80231000 "*" 1.56751860173054e-05 1.66739549642615e-05
51.6129032258064
"chr17" 80255001 80256000 "*" 0 0 -57.5531914893617
"chr17" 80290001 80291000 "*" 0 0 -88.9570552147239
"chr17" 80291001 80292000 "*" 0 0 -54.5372518690384
"chr17" 80297001 80298000 "*" 0 0 -97.0588235294118
"chr17" 80310001 80311000 "*" 0 0 100
"chr17" 80312001 80313000 "*" 0.000179338653346295 0.000161381175027113
51.9230769230769
"chr17" 80372001 80373000 "*" 0 0 60.4703147353362
"chr17" 80417001 80418000 "*" 2.12790891473968e-06 2.57994252743677e-06
-71.1111111111111
"chr17" 80454001 80455000 "*" 0 0 90.4564315352697
"chr17" 80455001 80456000 "*" 0 0 59.4032805429864
"chr17" 80460001 80461000 "*" 2.64672483929473e-09 4.82186011375752e-09
-58.2089552238806
"chr17" 80527001 80528000 "*" 0 0 -50.3649635036496
"chr17" 80539001 80540000 "*" 2.77555756156289e-15 1.10015415195978e-14
-64.2122487143525
"chr17" 80550001 80551000 "*" 2.59139457114976e-07 3.55953214680203e-07
71.7948717948718
"chr17" 80612001 80613000 "*" 1.30817578991582e-12 3.7554306979851e-12
80.7692307692308
"chr17" 80667001 80668000 "*" 0 0 59.0990990990991
"chr17" 80674001 80675000 "*" 0 0 52.4281609195402
"chr17" 80675001 80676000 "*" 6.66133814775094e-16 2.81595744474255e-15 76
"chr17" 80693001 80694000 "*" 0 0 53.0132517056413
"chr17" 80727001 80728000 "*" 1.83952852950142e-12 5.17499701329218e-12 -100
"chr17" 80805001 80806000 "*" 8.43495051583432e-10 1.65299297881828e-09
-54.8387096774194
"chr17" 80970001 80971000 "*" 0 0 -56.1956777073056
"chr17" 81034001 81035000 "*" 0 0 73.8466059359389
"chr17" 81037001 81038000 "*" 3.5527136788005e-15 1.39277426410519e-14
-57.341650671785
"chr17" 81107001 81108000 "*" 0 0 92
"chr17" 81121001 81122000 "*" 2.1094237467878e-15 8.47434540879246e-15
-93.3333333333333
"chr17_ctg5_hap1" 806001 807000 "*" 0 0 -61.1764705882353
"chr17_gl000204_random" 33001 34000 "*" 3.05836419522976e-07

4.15998867274448e-07 66.6666666666667
"chr17_gl000204_random" 47001 48000 "*" 1.88667836908962e-08
3.03999789296224e-08 72.9411764705882
"chr17_gl000204_random" 74001 75000 "*" 6.90747459231034e-12
1.80140148623876e-11 -100
"chr17_gl000205_random" 88001 89000 "*" 3.76964459647411e-10
7.75680310562266e-10 77.5862068965517
"chr17_gl000205_random" 98001 99000 "*" 0 0 -100
"chr17_gl000205_random" 133001 134000 "*" 9.65338919911574e-13
2.80678910656113e-12 100
"chr17_gl000205_random" 144001 145000 "*" 8.51433988646733e-07
1.09113827927329e-06 52.3809523809524
"chr17_gl000205_random" 166001 167000 "*" 0.000144235317824704
0.000131787791518504 57.5
"chr17_gl000205_random" 167001 168000 "*" 7.05328740713274e-06
7.90880605658123e-06 62.1078037007241
"chr18" 317001 318000 "*" 2.4535928844216e-14 8.69144396197119e-14 100
"chr18" 331001 332000 "*" 8.23991908038835e-09 1.39956619746302e-08 74
"chr18" 346001 347000 "*" 4.08209022140227e-11 9.57770690255255e-11 -100
"chr18" 375001 376000 "*" 1.12634901405784e-10 2.49563754426169e-10 -100
"chr18" 480001 481000 "*" 8.8883287545638e-06 9.81402829224479e-06
-55.1020408163265
"chr18" 499001 500000 "*" 0 0 72.2689075630252
"chr18" 813001 814000 "*" 0 0 -93.4782608695652
"chr18" 1296001 1297000 "*" 8.7349629751543e-09 1.47505871898511e-08 -100
"chr18" 2273001 2274000 "*" 0 0 100
"chr18" 2643001 2644000 "*" 2.69084754478399e-12 7.41487347763769e-12 -100
"chr18" 2655001 2656000 "*" 0 0 100
"chr18" 2846001 2847000 "*" 0 0 71.1800688161081
"chr18" 2870001 2871000 "*" 2.56325038971994e-09 4.68274925546504e-09
-73.1707317073171
"chr18" 2892001 2893000 "*" 6.6778889247221e-05 6.43705713226498e-05
-56.3636363636364
"chr18" 3219001 3220000 "*" 8.71525074330748e-14 2.88848349331391e-13 100
"chr18" 3246001 3247000 "*" 5.16831022423503e-12 1.37195453197788e-11
-76.4814814814815
"chr18" 3279001 3280000 "*" 3.31147522847353e-07 4.48459595110263e-07
64.2857142857143
"chr18" 3415001 3416000 "*" 7.7715611723761e-16 3.26213507634405e-15
69.7674418604651
"chr18" 3452001 3453000 "*" 1.19856347069458e-11 3.03004027942852e-11
56.4516129032258
"chr18" 3515001 3516000 "*" 1.3767929174513e-07 1.96165523017648e-07 -71.875
"chr18" 3576001 3577000 "*" 2.38031816479634e-13 7.47299807843568e-13 100
"chr18" 3784001 3785000 "*" 0 0 -62.3745819397993
"chr18" 3801001 3802000 "*" 1.48087875295744e-10 3.22075573380618e-10 100
"chr18" 3807001 3808000 "*" 3.95353005888666e-09 7.03928529283733e-09 100
"chr18" 4498001 4499000 "*" 8.40730016249402e-06 9.31620287501922e-06
-66.6666666666667
"chr18" 4750001 4751000 "*" 1.29037891483108e-11 3.24582280286866e-11 100
"chr18" 5238001 5239000 "*" 0 0 68.5248518011856
"chr18" 5410001 5411000 "*" 4.02167188440217e-12 1.08266700497926e-11 -100

"chr18" 5432001 5433000 "*" 4.79888864202138e-08 7.29535840441379e-08 -90.9090909090909
"chr18" 5433001 5434000 "*" 2.22385682313586e-07 3.08045655982239e-07 79.3650793650794
"chr18" 5506001 5507000 "*" 0.000320710195636176 0.000276619581787338 -52.3809523809524
"chr18" 5631001 5632000 "*" 6.4152538836737e-10 1.27477697728417e-09 100
"chr18" 5894001 5895000 "*" 1.4432899320127e-15 5.90750815956055e-15 81.1111111111111
"chr18" 5896001 5897000 "*" 0 0 61.8474045811045
"chr18" 5917001 5918000 "*" 2.10681916357203e-10 4.50388810476048e-10 -87.1794871794872
"chr18" 5938001 5939000 "*" 0 0 -100
"chr18" 5987001 5988000 "*" 2.00227675550835e-08 3.20459176617961e-08 -100
"chr18" 6042001 6043000 "*" 9.80763359414993e-10 1.8980032424715e-09 -100
"chr18" 6079001 6080000 "*" 4.08209022140227e-11 9.57770690255255e-11 100
"chr18" 6145001 6146000 "*" 0 0 -100
"chr18" 6271001 6272000 "*" 0 0 -51.3025951121189
"chr18" 6413001 6414000 "*" 2.33146835171283e-15 9.32818831833246e-15 88.6363636363636
"chr18" 6539001 6540000 "*" 0 0 100
"chr18" 6727001 6728000 "*" 1.72084568816899e-13 5.50960611810785e-13 94.1176470588235
"chr18" 6729001 6730000 "*" 0 0 68.0897529448556
"chr18" 6855001 6856000 "*" 3.67990547833763e-06 4.30329479768314e-06 61.3636363636364
"chr18" 6930001 6931000 "*" 0 0 83.75
"chr18" 6982001 6983000 "*" 0 0 -100
"chr18" 6990001 6991000 "*" 3.23685522829464e-12 8.82988198834855e-12 75
"chr18" 7016001 7017000 "*" 1.0547118733939e-14 3.91001935116974e-14 64.1791044776119
"chr18" 7038001 7039000 "*" 6.84466255007621e-07 8.8903539510783e-07 -66.9064748201439
"chr18" 7061001 7062000 "*" 1.67299207820548e-08 2.71377429441055e-08 -100
"chr18" 7071001 7072000 "*" 8.06688049692639e-13 2.37556391305009e-12 -100
"chr18" 7272001 7273000 "*" 5.16164888608728e-12 1.37043381386669e-11 57.0821959356991
"chr18" 7307001 7308000 "*" 2.12648565423024e-10 4.54336840422348e-10 -96.1538461538462
"chr18" 7385001 7386000 "*" 2.88779000712225e-11 6.92935809466068e-11 -100
"chr18" 7395001 7396000 "*" 1.37828859436695e-10 3.01445544267661e-10 100
"chr18" 7459001 7460000 "*" 9.80763359414993e-10 1.8980032424715e-09 -100
"chr18" 7566001 7567000 "*" 0 0 69.432536208645
"chr18" 7828001 7829000 "*" 0 0 -100
"chr18" 8138001 8139000 "*" 2.14487926530893e-07 2.97733806535428e-07 -78.8732394366197
"chr18" 8208001 8209000 "*" 6.92287338566189e-11 1.57605630124247e-10 100
"chr18" 8211001 8212000 "*" 0 0 -100
"chr18" 8279001 8280000 "*" 3.9190872769268e-14 1.35416418933905e-13 -100
"chr18" 8359001 8360000 "*" 1.35447209004269e-14 4.94674708073053e-14 100
"chr18" 8367001 8368000 "*" 0 0 -70.2352941176471
"chr18" 8416001 8417000 "*" 2.22044604925031e-16 9.81641919380259e-16

-68.4210526315789
"chr18" 8423001 8424000 "*" 9.68119528210742e-07 1.23074573810667e-06
65.7142857142857
"chr18" 8428001 8429000 "*" 1.69905867153375e-10 3.67390035933181e-10
67.6470588235294
"chr18" 8463001 8464000 "*" 4.71134242729931e-12 1.257615036806e-11 100
"chr18" 8466001 8467000 "*" 2.15125472990962e-10 4.58126659943874e-10 -100
"chr18" 8493001 8494000 "*" 2.88710388929303e-10 6.03399264731139e-10 100
"chr18" 8545001 8546000 "*" 8.63475957402215e-12 2.22427719052604e-11
55.1724137931034
"chr18" 8572001 8573000 "*" 0 0 100
"chr18" 8657001 8658000 "*" 3.21072057829497e-11 7.64900405635135e-11
59.6153846153846
"chr18" 8662001 8663000 "*" 0 0 100
"chr18" 8704001 8705000 "*" 0 0 64.6808510638298
"chr18" 8868001 8869000 "*" 3.6700841921089e-08 5.65530226072258e-08 -100
"chr18" 8952001 8953000 "*" 5.08482145278322e-14 1.73432706170696e-13 -100
"chr18" 8965001 8966000 "*" 1.24847021609753e-10 2.75043680609774e-10
62.9032258064516
"chr18" 8979001 8980000 "*" 3.6700841921089e-08 5.65530226072258e-08 -100
"chr18" 9017001 9018000 "*" 0 0 -70.5156831472621
"chr18" 9138001 9139000 "*" 6.15840711759574e-13 1.83676803572802e-12 100
"chr18" 9321001 9322000 "*" 2.69007038866675e-13 8.38831022628993e-13 100
"chr18" 9448001 9449000 "*" 1.23694509277517e-05 1.3360368613825e-05
69.4444444444444
"chr18" 9474001 9475000 "*" 3.43834122285003e-08 5.33844765815273e-08 -76
"chr18" 9615001 9616000 "*" 8.21565038222616e-15 3.08158744293689e-14 -100
"chr18" 9706001 9707000 "*" 4.73234496034536e-09 8.30627300264826e-09 -100
"chr18" 9714001 9715000 "*" 0.000245538078540553 0.000215951647556943
-54.5454545454545
"chr18" 9732001 9733000 "*" 0 0 -100
"chr18" 9734001 9735000 "*" 0 0 -100
"chr18" 9885001 9886000 "*" 1.20101260392413e-06 1.50833165035332e-06
70.4545454545455
"chr18" 9992001 9993000 "*" 1.66533453693773e-15 6.7629186866784e-15 100
"chr18" 10004001 10005000 "*" 1.12458486967171e-09 2.15740815043925e-09 100
"chr18" 10056001 10057000 "*" 1.96050553569194e-09 3.6470351769825e-09
-52.4952987125705
"chr18" 10128001 10129000 "*" 1.11022302462516e-16 5.03662826618488e-16 -100
"chr18" 10140001 10141000 "*" 6.59550958292954e-09 1.13456194382773e-08 -100
"chr18" 10167001 10168000 "*" 0 0 -58.4836022355795
"chr18" 10182001 10183000 "*" 6.35350224276188e-06 7.1700277597973e-06 -62.5
"chr18" 10237001 10238000 "*" 2.62900812231237e-13 8.21294938531841e-13 100
"chr18" 10400001 10401000 "*" 0 0 -100
"chr18" 10405001 10406000 "*" 9.94196613923748e-10 1.92279504037644e-09
-73.469387755102
"chr18" 10474001 10475000 "*" 1.67299207820548e-08 2.71377429441055e-08 -100
"chr18" 10665001 10666000 "*" 1.24026557135615e-07 1.77707997653412e-07
59.0909090909091
"chr18" 10667001 10668000 "*" 4.1153940362193e-06 4.77326970666537e-06
66.6666666666667
"chr18" 10668001 10669000 "*" 1.99784633281297e-12 5.59476580335607e-12

77.1428571428571
"chr18" 10682001 10683000 "*" 6.92287338566189e-11 1.57605630124247e-10 100
"chr18" 10927001 10928000 "*" 9.99200722162641e-16 4.15382497462808e-15 100
"chr18" 10935001 10936000 "*" 3.07111464037391e-08 4.80838592552796e-08
74.5664739884393
"chr18" 10962001 10963000 "*" 6.4152538836737e-10 1.27477697728417e-09 100
"chr18" 11056001 11057000 "*" 2.43005615629954e-09 4.45014222280278e-09 -100
"chr18" 11225001 11226000 "*" 8.73678272839129e-07 1.11791004728543e-06
50.5681818181818
"chr18" 11564001 11565000 "*" 5.03264097062583e-13 1.51942428521737e-12 -100
"chr18" 11609001 11610000 "*" 7.08114522834924e-05 6.79693915501506e-05 62.5
"chr18" 11654001 11655000 "*" 3.18015203837341e-07 4.31600104911059e-07 60
"chr18" 11667001 11668000 "*" 2.22190110248377e-05 2.30887626988729e-05
-52.3809523809524
"chr18" 11689001 11690000 "*" 0 0 59.2424242424242
"chr18" 11745001 11746000 "*" 1.39779633423487e-07 1.98463037958589e-07 -100
"chr18" 11775001 11776000 "*" 1.38486533352022e-09 2.63065104258646e-09
95.8333333333333
"chr18" 11799001 11800000 "*" 0.000792090690089564 0.000639295093468792
-52.3809523809524
"chr18" 11930001 11931000 "*" 5.01025332333427e-10 1.01358031135889e-09 100
"chr18" 11973001 11974000 "*" 8.3882012447134e-11 1.88922989503423e-10 -100
"chr18" 11980001 11981000 "*" 0 0 74.1690739976509
"chr18" 12007001 12008000 "*" 1.45137446505572e-09 2.74997482676393e-09
78.5714285714286
"chr18" 12027001 12028000 "*" 1.11022302462516e-16 5.03662826618488e-16
-72.2222222222222
"chr18" 12089001 12090000 "*" 9.08108033215171e-11 2.03407753381119e-10 -100
"chr18" 12092001 12093000 "*" 1.20063958775063e-11 3.03464492007794e-11
-58.5526315789474
"chr18" 12141001 12142000 "*" 4.73234496034536e-09 8.30627300264826e-09 -100
"chr18" 12230001 12231000 "*" 9.86433157379452e-12 2.52545471633917e-11
-53.030303030303
"chr18" 12237001 12238000 "*" 0 0 -100
"chr18" 12245001 12246000 "*" 3.53670426278541e-11 8.37777000754774e-11
-91.9117647058823
"chr18" 12278001 12279000 "*" 0 0 -100
"chr18" 12288001 12289000 "*" 4.84057238736568e-13 1.46509566626226e-12
60.126582278481
"chr18" 12420001 12421000 "*" 0 0 -56.3876513378137
"chr18" 12658001 12659000 "*" 2.52020626589911e-14 8.9164280780292e-14
-53.7515375153752
"chr18" 13139001 13140000 "*" 9.76996261670138e-15 3.63402770315216e-14 -100
"chr18" 13214001 13215000 "*" 0 0 -100
"chr18" 13222001 13223000 "*" 1.1492538287694e-07 1.65393032707173e-07
79.5918367346939
"chr18" 13230001 13231000 "*" 1.29037891483108e-11 3.24582280286866e-11 -100
"chr18" 13242001 13243000 "*" 2.88779000712225e-11 6.92935809466068e-11 -100
"chr18" 13284001 13285000 "*" 0 0 -74.0694789081886
"chr18" 13401001 13402000 "*" 0 0 100
"chr18" 13418001 13419000 "*" 2.08814465718632e-09 3.86232567960463e-09 -100
"chr18" 13432001 13433000 "*" 0 0 -100

"chr18" 13437001 13438000 "*" 6.52733422867868e-12 1.71146951718423e-11 -100
"chr18" 13491001 13492000 "*" 2.22044604925031e-16 9.81641919380259e-16
76.0536398467433
"chr18" 13516001 13517000 "*" 1.13140401492018e-08 1.87801380787728e-08 100
"chr18" 13528001 13529000 "*" 0 0 -100
"chr18" 13529001 13530000 "*" 2.02327044007689e-12 5.65300384792621e-12 100
"chr18" 13530001 13531000 "*" 5.14294773434898e-09 8.98856693399752e-09
73.9130434782609
"chr18" 13531001 13532000 "*" 3.9057646006313e-12 1.05610567215048e-11
-81.4814814814815
"chr18" 13542001 13543000 "*" 6.13931405712975e-09 1.06334746486227e-08
-63.7254901960784
"chr18" 13551001 13552000 "*" 9.82547376793264e-14 3.23127508005936e-13
-70.5585585585586
"chr18" 13554001 13555000 "*" 8.7349629751543e-09 1.47505871898511e-08 100
"chr18" 13560001 13561000 "*" 4.32209823486573e-12 1.15935290479641e-11 100
"chr18" 13566001 13567000 "*" 6.93112234273485e-13 2.05218842007357e-12 100
"chr18" 13574001 13575000 "*" 1.38531659567009e-05 1.48504331108706e-05
64.9350649350649
"chr18" 13584001 13585000 "*" 0 0 100
"chr18" 13619001 13620000 "*" 1.51312296026163e-12 4.30613217384394e-12
53.7378114842903
"chr18" 13622001 13623000 "*" 0 0 72.6936026936027
"chr18" 13623001 13624000 "*" 9.46902556364648e-11 2.11693695652591e-10
-51.5151515151515
"chr18" 13633001 13634000 "*" 5.33566007820951e-07 7.02842608769088e-07
-61.9047619047619
"chr18" 13655001 13656000 "*" 1.57113100307527e-10 3.40762543071219e-10
66.1290322580645
"chr18" 13801001 13802000 "*" 0 0 62.6740947075209
"chr18" 13887001 13888000 "*" 1.53210777398272e-14 5.5628583449216e-14 -100
"chr18" 13920001 13921000 "*" 1.54967429266684e-06 1.91685454663215e-06
54.5454545454545
"chr18" 13970001 13971000 "*" 1.28635010715472e-05 1.38618620931529e-05
-62.3188405797101
"chr18" 14086001 14087000 "*" 2.69018141096922e-12 7.41487347763769e-12 100
"chr18" 14173001 14174000 "*" 1.19073046100926e-06 1.49613945244789e-06
-69.4444444444444
"chr18" 14174001 14175000 "*" 6.32266461408904e-11 1.44998112442782e-10
82.1428571428571
"chr18" 14449001 14450000 "*" 4.50565140752701e-11 1.05258954535084e-10
-57.8947368421053
"chr18" 14633001 14634000 "*" 8.4821039081362e-14 2.81837999086832e-13
70.9090909090909
"chr18" 14831001 14832000 "*" 3.45022391545768e-07 4.66205089083695e-07
-77.2727272727273
"chr18" 14879001 14880000 "*" 0 0 66.6666666666667
"chr18" 14943001 14944000 "*" 0 0 -100
"chr18" 14946001 14947000 "*" 0 0 -100
"chr18" 14965001 14966000 "*" 1.68753899743024e-14 6.09826805967439e-14 -100
"chr18" 14966001 14967000 "*" 1.39779633423487e-07 1.98463037958589e-07 -100
"chr18" 14969001 14970000 "*" 1.17794662912729e-13 3.83653245040640e-13 -100

"chr18" 18515001 18516000 "*" 4.77658128605896e-08 7.26449192876705e-08
-56.7164179104478
"chr18" 18797001 18798000 "*" 1.66533453693773e-15 6.7629186866784e-15 -100
"chr18" 19232001 19233000 "*" 5.22872563767374e-05 5.12666630353878e-05
54.3859649122807
"chr18" 19314001 19315000 "*" 1.39779633423487e-07 1.98463037958589e-07 -100
"chr18" 19319001 19320000 "*" 1.99840144432528e-15 8.0570469535907e-15
54.4303797468354
"chr18" 19491001 19492000 "*" 5.6362137179633e-11 1.29963270300065e-10 100
"chr18" 19562001 19563000 "*" 7.0006535457523e-08 1.03695832512054e-07 100
"chr18" 19599001 19600000 "*" 4.21884749357559e-15 1.63874445239189e-14 100
"chr18" 19646001 19647000 "*" 2.58681964737661e-14 9.14008479605639e-14 -100
"chr18" 19659001 19660000 "*" 7.57374707394121e-09 1.29387284589791e-08 81.25
"chr18" 19754001 19755000 "*" 3.77475828372553e-15 1.47379557022071e-14 100
"chr18" 19756001 19757000 "*" 0 0 -52.9122952655063
"chr18" 19803001 19804000 "*" 0 0 -72.3809523809524
"chr18" 19810001 19811000 "*" 8.88178419700125e-16 3.70670032207938e-15 100
"chr18" 19847001 19848000 "*" 6.24654217240561e-10 1.2462108191303e-09 100
"chr18" 19927001 19928000 "*" 1.87405646556726e-13 5.97285292681429e-13 -100
"chr18" 19928001 19929000 "*" 0 0 54.2520779720176
"chr18" 19937001 19938000 "*" 1.20591092667155e-10 2.66075324129686e-10 100
"chr18" 19991001 19992000 "*" 1.12055920098442e-11 2.84500040664577e-11 -100
"chr18" 20193001 20194000 "*" 5.48053716853403e-08 8.27055729786969e-08
57.0607553366174
"chr18" 20377001 20378000 "*" 2.68450373042128e-10 5.64465213127486e-10 100
"chr18" 20635001 20636000 "*" 2.83622270114137e-09 5.14458665992009e-09
52.3809523809524
"chr18" 20714001 20715000 "*" 1.41331391034782e-13 4.56246816953652e-13
-72.972972972973
"chr18" 20728001 20729000 "*" 1.52466927971773e-12 4.33322475981408e-12 100
"chr18" 20803001 20804000 "*" 5.34028377074947e-12 1.41575825199932e-11
-85.1851851851852
"chr18" 20811001 20812000 "*" 1.16198698785208e-06 1.46238097234879e-06
54.5454545454545
"chr18" 20888001 20889000 "*" 1.4432899320127e-15 5.90750815956055e-15 100
"chr18" 21212001 21213000 "*" 4.88498130835069e-15 1.8824139250584e-14 100
"chr18" 21315001 21316000 "*" 0 0 -65.7894736842105
"chr18" 21386001 21387000 "*" 6.59550958292954e-09 1.13456194382773e-08 -100
"chr18" 22212001 22213000 "*" 1.56863411149288e-12 4.4469621057462e-12 100
"chr18" 22314001 22315000 "*" 1.77635683940025e-15 7.19870740878856e-15
-70.3703703703704
"chr18" 22402001 22403000 "*" 4.99900121297969e-12 1.32921056339624e-11 -100
"chr18" 22756001 22757000 "*" 8.24674165743611e-05 7.83478005558066e-05
-59.6153846153846
"chr18" 22931001 22932000 "*" 0 0 72.8410088715294
"chr18" 23436001 23437000 "*" 5.63660229602192e-13 1.68905058935478e-12 100
"chr18" 24047001 24048000 "*" 3.91325427617772e-09 6.98801246259140e-09
-66.6666666666667
"chr18" 24131001 24132000 "*" 0 0 87.2473604826546
"chr18" 24513001 24514000 "*" 1.14124265593318e-11 2.89138062669327e-11 -100
"chr18" 24765001 24766000 "*" 0 0 79.0840167842838
"chr18" 25706001 25707000 "*" 2.08814465718632e-09 3.86232567960463e-09 -100

"chr18" 25740001 25741000 "*" 6.24654217240561e-10 1.2462108191303e-09 -100
"chr18" 28621001 28622000 "*" 0 0 62.0259699624531
"chr18" 28681001 28682000 "*" 0 0 -69.4619837576038
"chr18" 28682001 28683000 "*" 0 0 -91.2751677852349
"chr18" 28868001 28869000 "*" 4.32209823486573e-12 1.15935290479641e-11 -100
"chr18" 29300001 29301000 "*" 6.24654217240561e-10 1.2462108191303e-09 -100
"chr18" 29320001 29321000 "*" 2.08219982200664e-06 2.52753654819169e-06
58.3333333333333
"chr18" 29387001 29388000 "*" 4.32209823486573e-12 1.15935290479641e-11 100
"chr18" 29502001 29503000 "*" 0.000240479789304304 0.000211878237500588
-57.1428571428571
"chr18" 29601001 29602000 "*" 5.03264097062583e-13 1.51942428521737e-12 -100
"chr18" 30209001 30210000 "*" 1.53210777398272e-14 5.5628583449216e-14 -100
"chr18" 30862001 30863000 "*" 2.96831899559979e-06 3.52052627123658e-06
68.4210526315789
"chr18" 31293001 31294000 "*" 3.90831811358794e-12 1.05610567215048e-11 -100
"chr18" 31326001 31327000 "*" 0 0 100
"chr18" 31802001 31803000 "*" 0 0 62.7996739547372
"chr18" 32073001 32074000 "*" 0 0 62.4471336476236
"chr18" 32334001 32335000 "*" 8.57092175010621e-13 2.51149198099779e-12 -100
"chr18" 32724001 32725000 "*" 7.12914669520615e-07 9.23469497775327e-07
-56.5217391304348
"chr18" 32936001 32937000 "*" 1.66533453693773e-15 6.7629186866784e-15 100
"chr18" 32991001 32992000 "*" 6.15840711759574e-13 1.83676803572802e-12 100
"chr18" 33025001 33026000 "*" 2.88779000712225e-11 6.92935809466068e-11 100
"chr18" 33096001 33097000 "*" 6.4152538836737e-10 1.27477697728417e-09 -100
"chr18" 33430001 33431000 "*" 7.0006535457523e-08 1.03695832512054e-07 100
"chr18" 33761001 33762000 "*" 2.33028818463765e-10 4.93719283454501e-10 -100
"chr18" 33767001 33768000 "*" 0 0 -54.0983606557377
"chr18" 33768001 33769000 "*" 0 0 -81.8553254712601
"chr18" 33877001 33878000 "*" 0 0 73.1391585760518
"chr18" 33968001 33969000 "*" 1.11022302462516e-16 5.03662826618488e-16 100
"chr18" 33989001 33990000 "*" 4.02167188440217e-12 1.08266700497926e-11 -100
"chr18" 34018001 34019000 "*" 5.08482145278322e-14 1.73432706170696e-13 100
"chr18" 34096001 34097000 "*" 1.46831802272374e-09 2.78024670726801e-09 -80
"chr18" 34232001 34233000 "*" 3.70575692354436e-09 6.63310104785764e-09
-76.4044943820225
"chr18" 34313001 34314000 "*" 1.19904086659517e-14 4.40901388691796e-14 -100
"chr18" 34578001 34579000 "*" 5.51780843238703e-14 1.87243792572872e-13 100
"chr18" 34829001 34830000 "*" 4.79616346638068e-14 1.64380445416596e-13 100
"chr18" 34834001 34835000 "*" 0 0 83.2953871694533
"chr18" 34842001 34843000 "*" 0 0 100
"chr18" 34869001 34870000 "*" 2.22044604925031e-16 9.81641919380259e-16 -100
"chr18" 34871001 34872000 "*" 4.78841410966879e-11 1.11468187156588e-10 100
"chr18" 34877001 34878000 "*" 2.54058807325208e-09 4.64317868304603e-09 60
"chr18" 34918001 34919000 "*" 0 0 100
"chr18" 34964001 34965000 "*" 3.6700841921089e-08 5.65530226072258e-08 100
"chr18" 34966001 34967000 "*" 2.66453525910038e-15 1.05861327033776e-14
-70.8333333333333
"chr18" 34973001 34974000 "*" 1.53501792388155e-08 2.50894768770232e-08
67.5675675675676
"chr18" 34990001 34991000 "*" 2.01216820983063e-12 5.63141715168262e-12

53.3333333333333
"chr18" 35001001 35002000 "*" 8.88178419700125e-16 3.70670032207938e-15 96.875
"chr18" 35002001 35003000 "*" 5.48638023900594e-10 1.10374357336421e-09 100
"chr18" 35027001 35028000 "*" 1.26570642855484e-10 2.7836295105875e-10
71.6417910447761
"chr18" 35028001 35029000 "*" 1.11022302462516e-15 4.59817122935606e-15
58.8172043010753
"chr18" 35065001 35066000 "*" 0 0 58.7254500818331
"chr18" 35071001 35072000 "*" 0 0 58.5059819083747
"chr18" 35092001 35093000 "*" 8.06688049692639e-13 2.37556391305009e-12 100
"chr18" 35101001 35102000 "*" 1.44362299892009e-12 4.12255313375858e-12 80
"chr18" 35119001 35120000 "*" 2.52275977885574e-12 6.97751289526057e-12
73.1707317073171
"chr18" 35134001 35135000 "*" 6.37490060739765e-13 1.89496793587877e-12 -100
"chr18" 35250001 35251000 "*" 0 0 100
"chr18" 35251001 35252000 "*" 3.30369065437708e-12 9.00336905360004e-12 -80
"chr18" 35344001 35345000 "*" 3.597027969926e-07 4.84612690151399e-07
-92.8571428571429
"chr18" 35360001 35361000 "*" 0.000219315854348978 0.000194579908186008 55
"chr18" 37423001 37424000 "*" 0 0 -100
"chr18" 38529001 38530000 "*" 3.31690230837012e-12 9.03347040781321e-12 -100
"chr18" 39211001 39212000 "*" 4.99020824662466e-11 1.15790792586157e-10 -100
"chr18" 41175001 41176000 "*" 1.92946192356658e-09 3.59193011611483e-09 100
"chr18" 41641001 41642000 "*" 7.28134592620222e-08 1.07586026241957e-07
-66.6666666666667
"chr18" 42259001 42260000 "*" 0 0 84.7775175644028
"chr18" 42589001 42590000 "*" 1.98365768255826e-11 4.85612402473442e-11 -100
"chr18" 43200001 43201000 "*" 4.73234496034536e-09 8.30627300264826e-09 -100
"chr18" 43425001 43426000 "*" 0 0 -100
"chr18" 43502001 43503000 "*" 1.11022302462516e-16 5.03662826618488e-16 100
"chr18" 43732001 43733000 "*" 4.99020824662466e-11 1.15790792586157e-10 100
"chr18" 43748001 43749000 "*" 0 0 -71.0144927536232
"chr18" 43881001 43882000 "*" 1.6753644027645e-09 3.14260624508072e-09
70.8333333333333
"chr18" 43925001 43926000 "*" 2.41690790714344e-08 3.82830615477435e-08
82.4561403508772
"chr18" 44017001 44018000 "*" 1.91224813761437e-12 5.36527594469447e-12 100
"chr18" 44036001 44037000 "*" 2.82880385782391e-11 6.80917263140965e-11
82.2429906542056
"chr18" 44057001 44058000 "*" 0 0 57.591706539075
"chr18" 44085001 44086000 "*" 1.11022302462516e-16 5.03662826618488e-16 100
"chr18" 44109001 44110000 "*" 2.69018141096922e-12 7.41487347763769e-12 100
"chr18" 44135001 44136000 "*" 6.66133814775094e-16 2.81595744474255e-15 100
"chr18" 44146001 44147000 "*" 2.22044604925031e-16 9.81641919380259e-16 -100
"chr18" 44155001 44156000 "*" 9.65729496371637e-10 1.87255912493232e-09 -100
"chr18" 44241001 44242000 "*" 0 0 100
"chr18" 44245001 44246000 "*" 1.39927984754706e-08 2.29771440325017e-08
-67.741935483871
"chr18" 44295001 44296000 "*" 1.11022302462516e-16 5.03662826618488e-16 100
"chr18" 44300001 44301000 "*" 7.63388113500518e-07 9.84994673705853e-07
70.8333333333333
"chr18" 44335001 44336000 "*" 3.26405569239796e-14 1.13973003849234e-13 100

"chr18" 44644001 44645000 "*" 8.7349629751543e-09 1.47505871898511e-08 -100
"chr18" 44778001 44779000 "*" 3.33066907387547e-16 1.4495649018245e-15
-95.2380952380952
"chr18" 44932001 44933000 "*" 2.33028818463765e-10 4.93719283454501e-10 100
"chr18" 44991001 44992000 "*" 0 0 100
"chr18" 45083001 45084000 "*" 6.89796697539435e-09 1.18397870636312e-08
80.0982800982801
"chr18" 45163001 45164000 "*" 0 0 100
"chr18" 45326001 45327000 "*" 0 0 100
"chr18" 45327001 45328000 "*" 7.78377362564697e-13 2.29551678017433e-12 100
"chr18" 45328001 45329000 "*" 1.98365768255826e-11 4.85612402473442e-11 100
"chr18" 45573001 45574000 "*" 0 0 100
"chr18" 45576001 45577000 "*" 0 0 70.2702702702703
"chr18" 45661001 45662000 "*" 6.24654217240561e-10 1.2462108191303e-09 -100
"chr18" 45664001 45665000 "*" 7.86301024291447e-10 1.54632397495563e-09
-57.6923076923077
"chr18" 45666001 45667000 "*" 0 0 -100
"chr18" 45686001 45687000 "*" 2.64951482975562e-09 4.82186011375752e-09 100
"chr18" 45728001 45729000 "*" 4.41730974021226e-09 7.80208625902143e-09
60.377358490566
"chr18" 45802001 45803000 "*" 1.13140401492018e-08 1.87801380787728e-08 100
"chr18" 45805001 45806000 "*" 9.08108033215171e-11 2.03407753381119e-10 -100
"chr18" 45820001 45821000 "*" 2.84472445599704e-12 7.80485565882277e-12
-55.5555555555556
"chr18" 45823001 45824000 "*" 3.29217383177038e-06 3.87771819332901e-06 -62.5
"chr18" 45847001 45848000 "*" 0 0 100
"chr18" 45882001 45883000 "*" 3.95861121660346e-12 1.0684496262032e-11 100
"chr18" 45929001 45930000 "*" 5.01025332333427e-10 1.01358031135889e-09 100
"chr18" 45982001 45983000 "*" 6.59401644398372e-10 1.30839733359793e-09
-51.1111111111111
"chr18" 46045001 46046000 "*" 2.01809627897731e-08 3.22829990076203e-08
82.7586206896552
"chr18" 46187001 46188000 "*" 2.69007038866675e-13 8.38831022628993e-13 -100
"chr18" 46203001 46204000 "*" 1.13140401492018e-08 1.87801380787728e-08 -100
"chr18" 46213001 46214000 "*" 3.10862446895044e-15 1.22466437093774e-14 100
"chr18" 46227001 46228000 "*" 2.38440361965964e-06 2.86906919056847e-06
-58.3333333333333
"chr18" 46242001 46243000 "*" 0 0 -100
"chr18" 46257001 46258000 "*" 3.28190852272314e-09 5.9136536283007e-09 60
"chr18" 46295001 46296000 "*" 0.000143144970250364 0.000130844579592364
-57.8947368421053
"chr18" 46303001 46304000 "*" 2.37587727269783e-14 8.44268514944447e-14 100
"chr18" 46361001 46362000 "*" 3.33066907387547e-16 1.4495649018245e-15 -100
"chr18" 46456001 46457000 "*" 7.0006535457523e-08 1.03695832512054e-07 100
"chr18" 46469001 46470000 "*" 0 0 -100
"chr18" 46476001 46477000 "*" 0 0 59.2814371257485
"chr18" 46503001 46504000 "*" 0 0 100
"chr18" 46513001 46514000 "*" 1.48087875295744e-10 3.22075573380618e-10 100
"chr18" 46539001 46540000 "*" 1.51874335330149e-09 2.87096838512746e-09 -65.625
"chr18" 46543001 46544000 "*" 6.55373100233447e-09 1.13117908391268e-08
66.6666666666667
"chr18" 46555001 46556000 "*" 0 0 100

"chr18" 46558001 46559000 "*" 2.08814465718632e-09 3.86232567960463e-09 100
"chr18" 47088001 47089000 "*" 0 0 95.2569169960474
"chr18" 47428001 47429000 "*" 7.0006535457523e-08 1.03695832512054e-07 100
"chr18" 47740001 47741000 "*" 1.29037891483108e-11 3.24582280286866e-11 -100
"chr18" 47931001 47932000 "*" 0 0 -100
"chr18" 48085001 48086000 "*" 0 0 56.6280566280566
"chr18" 48086001 48087000 "*" 0 0 76.0790051207023
"chr18" 48087001 48088000 "*" 0 0 86.9387755102041
"chr18" 48184001 48185000 "*" 6.29093539750158e-06 7.10329156043758e-06
-53.6585365853659
"chr18" 48301001 48302000 "*" 5.45864978773736e-08 8.23916743843603e-08
72.2222222222222
"chr18" 48637001 48638000 "*" 0 0 -59.3220338983051
"chr18" 48648001 48649000 "*" 1.71751501909512e-13 5.49936459663128e-13
-68.4210526315789
"chr18" 49019001 49020000 "*" 1.74853465040314e-11 4.32752710900609e-11 -100
"chr18" 49189001 49190000 "*" 4.89590999142031e-06 5.61947518077339e-06 80
"chr18" 50396001 50397000 "*" 9.69457847332933e-12 2.48374835346384e-11
70.2702702702703
"chr18" 50671001 50672000 "*" 1.25284005392245e-10 2.75649590272477e-10 -100
"chr18" 51152001 51153000 "*" 0 0 -100
"chr18" 51210001 51211000 "*" 3.90593728960553e-06 4.54759527414781e-06
61.5384615384615
"chr18" 52452001 52453000 "*" 5.50057949630922e-05 5.37293766519188e-05 -65
"chr18" 52495001 52496000 "*" 0 0 -62.1052631578947
"chr18" 52588001 52589000 "*" 1.11022302462516e-16 5.03662826618488e-16 100
"chr18" 52626001 52627000 "*" 0 0 -53.7629037629038
"chr18" 53255001 53256000 "*" 0 0 77.426013962054
"chr18" 53619001 53620000 "*" 4.73234496034536e-09 8.30627300264826e-09 -100
"chr18" 53620001 53621000 "*" 4.73234496034536e-09 8.30627300264826e-09 -100
"chr18" 54719001 54720000 "*" 1.67299207820548e-08 2.71377429441055e-08 -100
"chr18" 55138001 55139000 "*" 8.21565038222616e-15 3.08158744293689e-14 -100
"chr18" 55289001 55290000 "*" 1.56985535681997e-13 5.04283137158255e-13
-61.1111111111111
"chr18" 55397001 55398000 "*" 4.23745479916082e-06 4.90563267622047e-06
-65.3061224489796
"chr18" 55469001 55470000 "*" 0 0 72.0338983050847
"chr18" 55795001 55796000 "*" 5.1281201507436e-13 1.54461625452838e-12 -100
"chr18" 55894001 55895000 "*" 1.12458486967171e-09 2.15740815043925e-09 100
"chr18" 55902001 55903000 "*" 5.24204809504436e-06 5.9949875857084e-06 -56
"chr18" 55994001 55995000 "*" 3.33066907387547e-16 1.4495649018245e-15 100
"chr18" 56121001 56122000 "*" 2.15125472990962e-10 4.58126659943874e-10 -100
"chr18" 56151001 56152000 "*" 2.69018141096922e-12 7.41487347763769e-12 -100
"chr18" 56159001 56160000 "*" 2.33028818463765e-10 4.93719283454501e-10 100
"chr18" 56160001 56161000 "*" 2.69574645921367e-05 2.7651973231347e-05
-63.6363636363636
"chr18" 56265001 56266000 "*" 8.4295874905882e-06 9.33879080874541e-06
52.3809523809524
"chr18" 56422001 56423000 "*" 0 0 100
"chr18" 56434001 56435000 "*" 7.0006535457523e-08 1.03695832512054e-07 -100
"chr18" 56452001 56453000 "*" 0 0 -100
"chr18" 56515001 56516000 "*" 5.10702591327572e-15 1.96271840288234e-14 -100

"chr18" 56530001 56531000 "*" 0 0 64.5021645021645
"chr18" 56708001 56709000 "*" 1.55018292946618e-06 1.91745523608112e-06
-72.7272727272727
"chr18" 56762001 56763000 "*" 8.25450818808804e-13 2.42473843346228e-12 100
"chr18" 56975001 56976000 "*" 4.29629665177345e-11 1.00570937225351e-10
59.6638655462185
"chr18" 56976001 56977000 "*" 0 0 98.7551867219917
"chr18" 57014001 57015000 "*" 8.60711502070899e-12 2.21781256102041e-11
90.9090909090909
"chr18" 57063001 57064000 "*" 2.4535928844216e-14 8.69144396197119e-14 100
"chr18" 57122001 57123000 "*" 9.2917025962258e-08 1.35366826656484e-07
71.6981132075472
"chr18" 57136001 57137000 "*" 0 0 100
"chr18" 57163001 57164000 "*" 0 0 100
"chr18" 57292001 57293000 "*" 1.98365768255826e-11 4.85612402473442e-11 100
"chr18" 57311001 57312000 "*" 4.01313771103418e-09 7.11882087237587e-09 100
"chr18" 57458001 57459000 "*" 0 0 64.7058823529412
"chr18" 57677001 57678000 "*" 0 0 62.4113475177305
"chr18" 58329001 58330000 "*" 0 0 57.1051293639137
"chr18" 58352001 58353000 "*" 5.04263297784746e-13 1.52078941532818e-12 -100
"chr18" 58813001 58814000 "*" 4.14335232790108e-13 1.26153063645578e-12 100
"chr18" 59526001 59527000 "*" 4.60606257490781e-06 5.30621621406359e-06
69.6969696969697
"chr18" 59590001 59591000 "*" 1.93720595120794e-11 4.76309924836636e-11 -100
"chr18" 59658001 59659000 "*" 1.12458486967171e-09 2.15740815043925e-09 100
"chr18" 59991001 59992000 "*" 1.16108678227533e-10 2.5687839054584e-10
52.9411764705882
"chr18" 59995001 59996000 "*" 1.74013373976933e-06 2.1364085133039e-06 62.5
"chr18" 60002001 60003000 "*" 3.95861121660346e-12 1.0684496262032e-11 100
"chr18" 60199001 60200000 "*" 8.43347613965761e-12 2.17859001990012e-11 -100
"chr18" 60301001 60302000 "*" 8.08883973910657e-05 7.69625089176508e-05
-58.3333333333333
"chr18" 60383001 60384000 "*" 0 0 66.4627930682977
"chr18" 60408001 60409000 "*" 1.39779633423487e-07 1.98463037958589e-07 100
"chr18" 60414001 60415000 "*" 1.39779633423487e-07 1.98463037958589e-07 -100
"chr18" 60672001 60673000 "*" 7.41628980449605e-14 2.48193787819199e-13
88.0952380952381
"chr18" 60731001 60732000 "*" 3.76365605347928e-14 1.3043487970679e-13 -100
"chr18" 60822001 60823000 "*" 0 0 -57.1428571428571
"chr18" 60867001 60868000 "*" 2.03170813506404e-14 7.28047926885552e-14
-84.7826086956522
"chr18" 60893001 60894000 "*" 9.65729496371637e-10 1.87255912493232e-09 100
"chr18" 61112001 61113000 "*" 9.65729496371637e-10 1.87255912493232e-09 100
"chr18" 61603001 61604000 "*" 0 0 57.5065866194446
"chr18" 61616001 61617000 "*" 5.38680211548126e-13 1.61966634533483e-12 -80
"chr18" 61634001 61635000 "*" 7.0006535457523e-08 1.03695832512054e-07 100
"chr18" 61638001 61639000 "*" 0 0 -51.214953271028
"chr18" 62080001 62081000 "*" 1.42169380490387e-07 2.01687677516026e-07
54.0540540540541
"chr18" 63250001 63251000 "*" 0 0 100
"chr18" 63417001 63418000 "*" 0 0 56.9497904370884
"chr18" 63466001 63467000 "*" 3.88022947106492e-13 1.1867462770515e-12 -100

"chr18" 64616001 64617000 "*" 4.44089209850063e-16 1.91071758245033e-15
-66.6666666666667
"chr18" 64831001 64832000 "*" 1.52466927971773e-12 4.33322475981408e-12 100
"chr18" 65338001 65339000 "*" 0 0 100
"chr18" 65456001 65457000 "*" 0.000138951373611085 0.000127295950042963 70
"chr18" 65630001 65631000 "*" 3.68371999570627e-13 1.13149976091305e-12 100
"chr18" 66065001 66066000 "*" 3.04324343503026e-11 7.26599935673121e-11 100
"chr18" 66829001 66830000 "*" 3.6700841921089e-08 5.65530226072258e-08 100
"chr18" 67508001 67509000 "*" 7.7715611723761e-16 3.26213507634405e-15 100
"chr18" 67616001 67617000 "*" 0 0 100
"chr18" 68083001 68084000 "*" 7.68773577464188e-07 9.91437385905438e-07 -52
"chr18" 68103001 68104000 "*" 7.7715611723761e-16 3.26213507634405e-15 -100
"chr18" 68245001 68246000 "*" 9.63829016598083e-12 2.46993429847588e-11 100
"chr18" 68623001 68624000 "*" 7.66568364340969e-10 1.50944011004503e-09
-66.6666666666667
"chr18" 69224001 69225000 "*" 1.12458486967171e-09 2.15740815043925e-09 -100
"chr18" 70269001 70270000 "*" 3.56775498033812e-10 7.35898629809829e-10 100
"chr18" 71359001 71360000 "*" 2.73625566649116e-12 7.52483363579137e-12 100
"chr18" 71382001 71383000 "*" 5.55555601522428e-13 1.66842117217204e-12
96.8421052631579
"chr18" 71568001 71569000 "*" 0 0 100
"chr18" 71741001 71742000 "*" 0 0 100
"chr18" 71745001 71746000 "*" 0 0 100
"chr18" 71750001 71751000 "*" 8.65751914602697e-13 2.53597615484386e-12
64.1379310344828
"chr18" 71933001 71934000 "*" 9.41469124882133e-14 3.10868561210786e-13 100
"chr18" 71956001 71957000 "*" 1.36604061395929e-11 3.42333952202875e-11 -100
"chr18" 72079001 72080000 "*" 3.17634807345257e-13 9.83003611059031e-13 -81.25
"chr18" 72118001 72119000 "*" 2.55351295663786e-15 1.01678090381833e-14
73.9130434782609
"chr18" 72153001 72154000 "*" 4.72282917218791e-08 7.18654355900973e-08
-59.2592592592593
"chr18" 72176001 72177000 "*" 2.88779000712225e-11 6.92935809466068e-11 100
"chr18" 72178001 72179000 "*" 9.08108033215171e-11 2.03407753381119e-10 100
"chr18" 72206001 72207000 "*" 0 0 -100
"chr18" 72254001 72255000 "*" 5.6362137179633e-11 1.29963270300065e-10 -100
"chr18" 72824001 72825000 "*" 7.9984455014781e-06 8.89173797399106e-06
-66.6666666666667
"chr18" 72844001 72845000 "*" 0.000471727282945977 0.000395504194557835
-53.8461538461538
"chr18" 72849001 72850000 "*" 1.31610942394333e-05 1.4161144785177e-05
-66.6666666666667
"chr18" 73011001 73012000 "*" 2.49341319791263e-05 2.57048700295864e-05
64.1509433962264
"chr18" 73013001 73014000 "*" 5.88418203051333e-15 2.245355428447e-14 -100
"chr18" 73017001 73018000 "*" 3.41356554134364e-09 6.13669911406708e-09
-66.6666666666667
"chr18" 73053001 73054000 "*" 4.26951363152739e-10 8.72510873516957e-10 100
"chr18" 73076001 73077000 "*" 2.22044604925031e-16 9.81641919380259e-16 -100
"chr18" 73104001 73105000 "*" 3.9409849827976e-09 7.03483718242869e-09
63.4146341463415
"chr18" 73186001 73187000 "*" 2.62900812231237e-13 8.21294938531841e-13 100

"chr18" 73263001 73264000 "*" 0.000131667642706557 0.00012108605420816
-56.3636363636364
"chr18" 73266001 73267000 "*" 0 0 65.9574468085106
"chr18" 73267001 73268000 "*" 1.49310831476512e-08 2.44375206748162e-08
55.5555555555556
"chr18" 73332001 73333000 "*" 1.11022302462516e-16 5.03662826618488e-16 100
"chr18" 73424001 73425000 "*" 1.22124532708767e-15 5.03921984217778e-15
-66.6666666666667
"chr18" 73572001 73573000 "*" 1.12687636999453e-13 3.68543487142567e-13 -100
"chr18" 73587001 73588000 "*" 3.07963307077941e-08 4.82018501272284e-08
-66.6666666666667
"chr18" 73718001 73719000 "*" 1.52466927971773e-12 4.33322475981408e-12 -100
"chr18" 73767001 73768000 "*" 1.55133461632317e-09 2.91855334268503e-09
80.4347826086957
"chr18" 73793001 73794000 "*" 1.13140401492018e-08 1.87801380787728e-08 -100
"chr18" 73842001 73843000 "*" 0 0 66.6666666666667
"chr18" 73843001 73844000 "*" 0 0 -73.3333333333333
"chr18" 73867001 73868000 "*" 2.43005615629954e-09 4.45014222280278e-09 100
"chr18" 73888001 73889000 "*" 3.77475828372553e-15 1.47379557022071e-14 100
"chr18" 73918001 73919000 "*" 0 0 84.6153846153846
"chr18" 73935001 73936000 "*" 3.68371999570627e-13 1.13149976091305e-12 100
"chr18" 74023001 74024000 "*" 2.75335310107039e-14 9.69727903855348e-14 100
"chr18" 74074001 74075000 "*" 2.88657986402541e-15 1.14155613124573e-14
78.5714285714286
"chr18" 74208001 74209000 "*" 0 0 100
"chr18" 74218001 74219000 "*" 5.61766966278299e-10 1.128740399975e-09
-52.5519421860885
"chr18" 74261001 74262000 "*" 1.15498388630897e-09 2.21275873837915e-09
56.6308243727599
"chr18" 74292001 74293000 "*" 2.68562949656825e-13 8.38386629046894e-13
63.3802816901408
"chr18" 74304001 74305000 "*" 2.64795836368847e-08 4.17690999234936e-08
-76.7441860465116
"chr18" 74312001 74313000 "*" 0 0 -100
"chr18" 74324001 74325000 "*" 0 0 89.8989898989899
"chr18" 74379001 74380000 "*" 1.49972523288966e-09 2.83643172292987e-09
-73.6842105263158
"chr18" 74448001 74449000 "*" 2.6625853588591e-06 3.18053072473338e-06
68.9655172413793
"chr18" 74484001 74485000 "*" 2.1658988651474e-06 2.62321901854342e-06
-66.6666666666667
"chr18" 74490001 74491000 "*" 1.89581683684992e-12 5.32311367606535e-12 -100
"chr18" 74634001 74635000 "*" 4.26465529557163e-11 9.98891296616345e-11
72.0588235294118
"chr18" 74705001 74706000 "*" 1.12055920098442e-11 2.84500040664577e-11 100
"chr18" 74728001 74729000 "*" 3.53316902401701e-08 5.47915134092157e-08
51.0989010989011
"chr18" 74732001 74733000 "*" 0 0 100
"chr18" 74767001 74768000 "*" 0 0 94.4444444444444
"chr18" 74782001 74783000 "*" 0 0 100
"chr18" 74813001 74814000 "*" 5.794809077031e-12 1.52911192757929e-11 -100
"chr18" 74849001 74850000 "*" 4.6666426878339e-07 6.19106422769246e-07

62.7450980392157
"chr18" 74857001 74858000 "*" 4.2854608750531e-14 1.47617059233354e-13
90.3614457831325
"chr18" 74873001 74874000 "*" 1.86517468137026e-14 6.70995420767425e-14 100
"chr18" 74915001 74916000 "*" 0 0 75.968992248062
"chr18" 74922001 74923000 "*" 7.99844592513921e-06 8.89173797399106e-06
66.6666666666667
"chr18" 75035001 75036000 "*" 1.37828859436695e-10 3.01445544267661e-10 -100
"chr18" 75078001 75079000 "*" 1.07882591748876e-11 2.74553537455803e-11 100
"chr18" 75154001 75155000 "*" 5.09592368302947e-14 1.73761636562837e-13 -100
"chr18" 75258001 75259000 "*" 6.53921361504217e-14 2.20157828648574e-13 -74
"chr18" 75271001 75272000 "*" 9.08108033215171e-11 2.03407753381119e-10 -100
"chr18" 75273001 75274000 "*" 5.06907016362135e-09 8.86595052081024e-09
-61.5384615384615
"chr18" 75301001 75302000 "*" 6.07647486094987e-06 6.87749858892424e-06
-56.0975609756098
"chr18" 75370001 75371000 "*" 1.99283367585679e-10 4.27533678485245e-10
76.4705882352941
"chr18" 75669001 75670000 "*" 4.75366453437376e-05 4.6905450034535e-05
54.1666666666667
"chr18" 75689001 75690000 "*" 6.27176088840997e-12 1.64922824227733e-11 80
"chr18" 75705001 75706000 "*" 3.10862446895044e-15 1.22466437093774e-14 -100
"chr18" 75831001 75832000 "*" 2.06540895497653e-10 4.42545849276785e-10
-53.1055900621118
"chr18" 75883001 75884000 "*" 2.33146835171283e-15 9.32818831833246e-15
-63.2824143070045
"chr18" 75973001 75974000 "*" 0 0 -100
"chr18" 75989001 75990000 "*" 4.4227737916458e-08 6.75621687967191e-08
72.0338983050847
"chr18" 76071001 76072000 "*" 5.30635868578599e-10 1.07093701666285e-09
51.0314247156352
"chr18" 76133001 76134000 "*" 9.96306577349948e-07 1.26432438560702e-06
-50.4901960784314
"chr18" 76177001 76178000 "*" 0 0 100
"chr18" 76252001 76253000 "*" 2.87956325450978e-11 6.92655694492512e-11
-61.4035087719298
"chr18" 76261001 76262000 "*" 1.40068135777716e-05 1.50039561086948e-05
-54.1666666666667
"chr18" 76265001 76266000 "*" 3.64153152077051e-14 1.26507379876445e-13
-70.3125
"chr18" 76267001 76268000 "*" 4.21884749357559e-15 1.63874445239189e-14 100
"chr18" 76275001 76276000 "*" 3.93566290668446e-11 9.26856693554149e-11
71.9298245614035
"chr18" 76298001 76299000 "*" 5.26494595221028e-06 6.01996330880252e-06
54.7945205479452
"chr18" 76344001 76345000 "*" 1.99840144432528e-15 8.0570469535907e-15
73.8775510204082
"chr18" 76347001 76348000 "*" 4.27693436222398e-11 1.00142987367171e-10
90.3846153846154
"chr18" 76349001 76350000 "*" 7.0006535457523e-08 1.03695832512054e-07 100
"chr18" 76373001 76374000 "*" 0 0 79.4392523364486
"chr18" 76380001 76381000 "*" 2.00227675550835e-08 3.20459176617961e-08 -100

"chr18" 76384001 76385000 "*" 1.11022302462516e-16 5.03662826618488e-16 66.6666666666667
"chr18" 76416001 76417000 "*" 0 0 52.9716599190283
"chr18" 76425001 76426000 "*" 0 0 -100
"chr18" 76441001 76442000 "*" 0 0 75
"chr18" 76471001 76472000 "*" 3.10862446895044e-15 1.22466437093774e-14 100
"chr18" 76489001 76490000 "*" 0 0 83.4645669291339
"chr18" 76492001 76493000 "*" 5.36517932488989e-05 5.25070408481107e-05 56.4102564102564
"chr18" 76500001 76501000 "*" 1.88737914186277e-15 7.62011598380321e-15 -100
"chr18" 76511001 76512000 "*" 3.77475828372553e-15 1.47379557022071e-14 87.0967741935484
"chr18" 76525001 76526000 "*" 0 0 91.6666666666667
"chr18" 76528001 76529000 "*" 4.73962023184171e-07 6.28368274925326e-07 -63.6363636363636
"chr18" 76582001 76583000 "*" 6.41564579240139e-12 1.68562936898522e-11 -66.6666666666667
"chr18" 76590001 76591000 "*" 0 0 100
"chr18" 76635001 76636000 "*" 0 0 74.3697478991597
"chr18" 76658001 76659000 "*" 2.35305319673529e-08 3.73363995104836e-08 78.5714285714286
"chr18" 76668001 76669000 "*" 1.29275812277285e-10 2.83967889940194e-10 88.4318766066838
"chr18" 76687001 76688000 "*" 1.68305258618773e-10 3.6409036579438e-10 -54.9295774647887
"chr18" 76692001 76693000 "*" 0.000387553810620944 0.000329814340956859 -56.1904761904762
"chr18" 76697001 76698000 "*" 0 0 -100
"chr18" 76699001 76700000 "*" 5.93969318174459e-14 2.00775970146984e-13 95.6521739130435
"chr18" 76709001 76710000 "*" 4.40787094824202e-07 5.86780801607064e-07 73.6677115987461
"chr18" 76726001 76727000 "*" 0 0 100
"chr18" 76735001 76736000 "*" 5.05858217758615e-07 6.68233593552183e-07 57.1428571428571
"chr18" 76768001 76769000 "*" 7.43849426498855e-15 2.80357739563059e-14 100
"chr18" 76769001 76770000 "*" 0 0 97.3484848484848
"chr18" 76790001 76791000 "*" 0 0 100
"chr18" 77160001 77161000 "*" 0 0 70.8238009851811
"chr18" 77165001 77166000 "*" 0 0 65.04884004884
"chr18" 77210001 77211000 "*" 0 0 -50.534079626417
"chr18" 77213001 77214000 "*" 3.00082181325934e-12 8.21259535038715e-12 -78.9473684210526
"chr18" 77242001 77243000 "*" 1.29268928894533e-10 2.83960248803703e-10 69.4444444444444
"chr18" 77287001 77288000 "*" 0 0 55.9196172248804
"chr18" 77304001 77305000 "*" 3.33066907387547e-16 1.4495649018245e-15 67.3913043478261
"chr18" 77317001 77318000 "*" 3.84137166520304e-14 1.32929369817675e-13 56.9237385321101
"chr18" 77333001 77334000 "*" 6.9058092577734e-11 1.57605630124247e-10 -64.2276422764228

"chr18" 77342001 77343000 "*" 1.319500064767e-12 3.78585472179468e-12
94.3502824858757
"chr18" 77344001 77345000 "*" 0 0 69.7368421052632
"chr18" 77357001 77358000 "*" 0.000911789639360894 0.00072838976448704
-51.6438356164384
"chr18" 77388001 77389000 "*" 1.46549439250521e-14 5.33330076973935e-14
55.7046979865772
"chr18" 77394001 77395000 "*" 1.93720595120794e-11 4.76309924836636e-11 100
"chr18" 77402001 77403000 "*" 0 0 75.5905511811024
"chr18" 77463001 77464000 "*" 0 0 100
"chr18" 77566001 77567000 "*" 2.99760216648792e-15 1.18450729613838e-14
81.4814814814815
"chr18" 77579001 77580000 "*" 0 0 62.5
"chr18" 77582001 77583000 "*" 6.9408128910986e-08 1.03304833167463e-07
58.5714285714286
"chr18" 77583001 77584000 "*" 0 0 -100
"chr18" 77588001 77589000 "*" 3.03979064142368e-13 9.42350883309283e-13 100
"chr18" 77592001 77593000 "*" 9.86770945484716e-05 9.2596942519293e-05
55.5555555555556
"chr18" 77593001 77594000 "*" 1.51534340631088e-12 4.31230409918318e-12
73.3333333333333
"chr18" 77615001 77616000 "*" 1.35036426485158e-12 3.86629547363774e-12 100
"chr18" 77616001 77617000 "*" 4.08112432737084e-11 9.57770690255255e-11
88.8888888888889
"chr18" 77718001 77719000 "*" 0 0 -100
"chr18" 77832001 77833000 "*" 0.00019185659259191 0.000171804085901073
52.1739130434783
"chr18" 77868001 77869000 "*" 0 0 100
"chr18" 77920001 77921000 "*" 8.88178419700125e-16 3.70670032207938e-15
57.5757575757576
"chr18" 77958001 77959000 "*" 5.37803029284634e-05 5.26241567504211e-05 -60
"chr18" 77986001 77987000 "*" 7.63857592178674e-07 9.85554696369057e-07 -60
"chr18" 78005001 78006000 "*" 0 0 91.8854415274463
"chr19" 281001 282000 "*" 2.21822560320106e-13 6.98435372028823e-13
52.7272727272727
"chr19" 291001 292000 "*" 0 0 -61.2614080075953
"chr19" 298001 299000 "*" 7.30328020281945e-11 1.65890094256102e-10
-89.6551724137931
"chr19" 365001 366000 "*" 1.92512672470002e-13 6.1250871931855e-13
74.5098039215686
"chr19" 372001 373000 "*" 1.72032388334742e-11 4.26365370671867e-11
54.5454545454545
"chr19" 380001 381000 "*" 1.67299207820548e-08 2.71377429441055e-08 -100
"chr19" 382001 383000 "*" 8.11575139758602e-07 1.04319404043546e-06
68.8888888888889
"chr19" 391001 392000 "*" 1.58048615861439e-07 2.22982114523461e-07
52.9411764705882
"chr19" 399001 400000 "*" 0 0 61.1904761904762
"chr19" 443001 444000 "*" 0.000170906249498448 0.000154255817718553
-57.9326923076923
"chr19" 488001 489000 "*" 0 0 75.7009345794392
"chr19" 526001 527000 "*" 3.23574500527002e-11 7.70663930821415e-11

-55.1724137931034
"chr19" 564001 565000 "*" 1.74784686723939e-08 2.8282657869282e-08
51.8518518518519
"chr19" 588001 589000 "*" 0 0 -59.8614875037639
"chr19" 589001 590000 "*" 0 0 55.3625147878993
"chr19" 605001 606000 "*" 0 0 51.5151515151515
"chr19" 661001 662000 "*" 0 0 -80.5194805194805
"chr19" 665001 666000 "*" 2.22044604925031e-16 9.81641919380259e-16 -62.5
"chr19" 666001 667000 "*" 1.11022302462516e-16 5.03662826618488e-16
69.8795180722892
"chr19" 677001 678000 "*" 0.000236762667221169 0.000208837069819571
-54.0832049306626
"chr19" 688001 689000 "*" 3.27515792264421e-14 1.14341518453829e-13
-56.6820276497696
"chr19" 702001 703000 "*" 6.66133814775094e-16 2.81595744474255e-15 -100
"chr19" 716001 717000 "*" 8.25450818808804e-13 2.42473843346228e-12 -100
"chr19" 741001 742000 "*" 0 0 -100
"chr19" 751001 752000 "*" 6.96065245442412e-07 9.02919815321495e-07
63.6363636363636
"chr19" 775001 776000 "*" 8.0999675677873e-07 1.04131538148627e-06 -56
"chr19" 799001 800000 "*" 0 0 -73.1707317073171
"chr19" 809001 810000 "*" 8.65973959207622e-14 2.87362558212404e-13 -100
"chr19" 817001 818000 "*" 1.17816734146459e-09 2.25485869667672e-09
58.3333333333333
"chr19" 821001 822000 "*" 0 0 57.1313541373974
"chr19" 833001 834000 "*" 3.84336960645992e-06 4.47937651296003e-06 75
"chr19" 837001 838000 "*" 4.73234496034536e-09 8.30627300264826e-09 100
"chr19" 841001 842000 "*" 0 0 73.6842105263158
"chr19" 844001 845000 "*" 5.31855880447907e-08 8.03731742136787e-08
-52.3444626892903
"chr19" 859001 860000 "*" 7.07223168916471e-12 1.84143190036999e-11
63.4562211981567
"chr19" 913001 914000 "*" 5.55111512312578e-16 2.36485094870365e-15
-62.2222222222222
"chr19" 916001 917000 "*" 1.69503522329251e-10 3.66549430888533e-10
54.395325203252
"chr19" 917001 918000 "*" 0 0 55.1645104804339
"chr19" 930001 931000 "*" 8.88178419700125e-16 3.70670032207938e-15 -100
"chr19" 952001 953000 "*" 0 0 -100
"chr19" 957001 958000 "*" 6.36157793110215e-14 2.14489017335447e-13
57.0606844981306
"chr19" 1041001 1042000 "*" 0 0 -58.1821665815023
"chr19" 1094001 1095000 "*" 6.82826595266306e-09 1.17261842797601e-08
-66.6666666666667
"chr19" 1102001 1103000 "*" 1.1330425486733e-10 2.5092017593116e-10
-53.3333333333333
"chr19" 1131001 1132000 "*" 6.67910171614494e-13 1.98207279482631e-12
-57.5757575757576
"chr19" 1148001 1149000 "*" 0.000499129428774947 0.000416865202314435
52.3809523809524
"chr19" 1252001 1253000 "*" 0 0 -73.568281938326
"chr19" 1260001 1261000 "*" 0 0 -91.5254237288136

"chr19" 1337001 1338000 "*" 1.12463371948479e-11 2.8549106970431e-11
52.8571428571429
"chr19" 1339001 1340000 "*" 0 0 63.3802816901408
"chr19" 1349001 1350000 "*" 9.14157638476354e-13 2.66886212024714e-12
-98.3606557377049
"chr19" 1400001 1401000 "*" 1.66533453693773e-15 6.7629186866784e-15
-55.5229716520039
"chr19" 1406001 1407000 "*" 3.58595664737837e-05 3.60565776953974e-05
52.1739130434783
"chr19" 1415001 1416000 "*" 0 0 -100
"chr19" 1451001 1452000 "*" 3.17257701476503e-06 3.74647977658681e-06
-63.1578947368421
"chr19" 1455001 1456000 "*" 0 0 -59.1000871011165
"chr19" 1514001 1515000 "*" 0 0 100
"chr19" 1538001 1539000 "*" 2.88667978409762e-11 6.92935809466068e-11
-60.3174603174603
"chr19" 1670001 1671000 "*" 1.16573417585641e-14 4.29335582900508e-14 -100
"chr19" 1674001 1675000 "*" 0 0 59.1517857142857
"chr19" 1679001 1680000 "*" 0 0 66.0725964682799
"chr19" 1680001 1681000 "*" 4.75175454539567e-14 1.63113121878055e-13
56.6666666666667
"chr19" 1701001 1702000 "*" 8.49319448104069e-08 1.24403017126745e-07
77.3584905660377
"chr19" 1716001 1717000 "*" 1.56445055243815e-07 2.2081686035738e-07
63.6363636363636
"chr19" 1721001 1722000 "*" 8.42659275690494e-14 2.80071617186335e-13
63.4146341463415
"chr19" 1723001 1724000 "*" 3.83310166940376e-08 5.89495391451193e-08
83.0985915492958
"chr19" 1737001 1738000 "*" 0 0 56.6037735849057
"chr19" 1739001 1740000 "*" 4.02167188440217e-12 1.08266700497926e-11 100
"chr19" 1756001 1757000 "*" 8.88178419700125e-16 3.70670032207938e-15
-95.6521739130435
"chr19" 1759001 1760000 "*" 1.4432899320127e-15 5.90750815956055e-15 100
"chr19" 1768001 1769000 "*" 6.87183643321987e-12 1.79350946015426e-11
58.0528846153846
"chr19" 1773001 1774000 "*" 2.22044604925031e-16 9.81641919380259e-16 -100
"chr19" 1779001 1780000 "*" 0 0 90.5511811023622
"chr19" 1793001 1794000 "*" 2.44635490953549e-06 2.93814467663915e-06
-56.6666666666667
"chr19" 1813001 1814000 "*" 2.66251465319556e-11 6.43639355850841e-11
59.0909090909091
"chr19" 1860001 1861000 "*" 1.0769163338864e-14 3.98631098593833e-14
-53.2984859408796
"chr19" 1885001 1886000 "*" 0 0 80
"chr19" 1897001 1898000 "*" 0 0 -68.2634730538922
"chr19" 1929001 1930000 "*" 2.07406070096283e-09 3.84712114444672e-09
55.0847457627119
"chr19" 1932001 1933000 "*" 1.56863411149288e-12 4.4469621057462e-12 100
"chr19" 1939001 1940000 "*" 0 0 -91.3978494623656
"chr19" 1942001 1943000 "*" 0 0 -67.2413793103448
"chr19" 1992001 1993000 "*" 0 0 -100

"chr19" 2020001 2021000 "*" 0 0 100
"chr19" 2030001 2031000 "*" 0 0 -59.5281782437746
"chr19" 2050001 2051000 "*" 1.48487888651516e-11 3.70689457767456e-11 -100
"chr19" 2119001 2120000 "*" 0 0 58.4158415841584
"chr19" 2149001 2150000 "*" 0 0 -100
"chr19" 2150001 2151000 "*" 1.98365768255826e-11 4.85612402473442e-11 -100
"chr19" 2172001 2173000 "*" 0 0 56
"chr19" 2261001 2262000 "*" 2.22044604925031e-16 9.81641919380259e-16 100
"chr19" 2271001 2272000 "*" 0 0 -82.8125
"chr19" 2281001 2282000 "*" 4.88089546557546e-09 8.55440851648019e-09 54.9019607843137
"chr19" 2292001 2293000 "*" 0 0 -50.7042253521127
"chr19" 2331001 2332000 "*" 0 0 -100
"chr19" 2392001 2393000 "*" 2.29329866208161e-09 4.22120358762507e-09 -66.6666666666667
"chr19" 2455001 2456000 "*" 7.105427357601e-15 2.68362283258525e-14 -100
"chr19" 2541001 2542000 "*" 2.10789163901381e-11 5.14756476429927e-11 -56.2061403508772
"chr19" 2578001 2579000 "*" 0 0 58.5365853658537
"chr19" 2580001 2581000 "*" 3.99846228349432e-08 6.13647647811993e-08 -90
"chr19" 2588001 2589000 "*" 0 0 100
"chr19" 2612001 2613000 "*" 3.10862446895044e-15 1.22466437093774e-14 -85
"chr19" 2628001 2629000 "*" 1.11022302462516e-16 5.03662826618488e-16 100
"chr19" 2641001 2642000 "*" 1.99840144432528e-15 8.0570469535907e-15 75
"chr19" 2693001 2694000 "*" 1.67299207820548e-08 2.71377429441055e-08 -100
"chr19" 2696001 2697000 "*" 1.62092561595273e-14 5.87307003677525e-14 80.1652892561983
"chr19" 2702001 2703000 "*" 0 0 67.0861396667848
"chr19" 2714001 2715000 "*" 1.61330021475337e-06 1.99027220773611e-06 -75
"chr19" 2721001 2722000 "*" 0 0 53.0023669890601
"chr19" 2726001 2727000 "*" 1.5806816966446e-09 2.97155250174472e-09 74
"chr19" 2749001 2750000 "*" 0 0 98.7179487179487
"chr19" 2752001 2753000 "*" 1.20281562487889e-12 3.46585342181251e-12 100
"chr19" 2842001 2843000 "*" 0 0 -66.9100982120373
"chr19" 2858001 2859000 "*" 0 0 100
"chr19" 2974001 2975000 "*" 2.14414552779374e-09 3.96082288080217e-09 -71.7948717948718
"chr19" 2978001 2979000 "*" 4.18299724858073e-08 6.40718628356732e-08 -74.8148148148148
"chr19" 2981001 2982000 "*" 0 0 -100
"chr19" 2996001 2997000 "*" 0 0 57.4279379157428
"chr19" 3002001 3003000 "*" 4.8527482587879e-08 7.37333916927297e-08 58.5365853658537
"chr19" 3020001 3021000 "*" 8.07333775534325e-05 7.68281644181083e-05 -52.9411764705882
"chr19" 3032001 3033000 "*" 8.08953612985164e-08 1.18784631746042e-07 79.0322580645161
"chr19" 3047001 3048000 "*" 0 0 66.6666666666667
"chr19" 3066001 3067000 "*" 3.33066907387547e-16 1.4495649018245e-15 -100
"chr19" 3076001 3077000 "*" 8.7349629751543e-09 1.47505871898511e-08 -100
"chr19" 3142001 3143000 "*" 2.30662478006849e-09 4.24423320622348e-09 -64.1025641025641

"chr19" 3145001 3146000 "*" 7.0006535457523e-08 1.03695832512054e-07 -100
"chr19" 3162001 3163000 "*" 0 0 66.6666666666667
"chr19" 3163001 3164000 "*" 3.36957017843531e-09 6.06165745712583e-09
-50.1623376623377
"chr19" 3219001 3220000 "*" 4.9960036108132e-15 1.9234186548721e-14
62.3711340206186
"chr19" 3225001 3226000 "*" 0 0 51.5338110165696
"chr19" 3227001 3228000 "*" 0 0 66.6666666666667
"chr19" 3229001 3230000 "*" 0 0 82.8947368421053
"chr19" 3239001 3240000 "*" 1.06621183004751e-06 1.34850034489853e-06
-50.9090909090909
"chr19" 3266001 3267000 "*" 0 0 64.2857142857143
"chr19" 3267001 3268000 "*" 1.52794177310511e-09 2.87642564232045e-09 100
"chr19" 3282001 3283000 "*" 0 0 75
"chr19" 3319001 3320000 "*" 1.11022302462516e-15 4.59817122935606e-15
-73.3333333333333
"chr19" 3343001 3344000 "*" 2.1094237467878e-15 8.47434540879246e-15 -100
"chr19" 3344001 3345000 "*" 1.59089033269222e-06 1.96437659485627e-06 -67.1875
"chr19" 3347001 3348000 "*" 2.65343302885412e-14 9.36553235299198e-14
69.4444444444444
"chr19" 3350001 3351000 "*" 2.91028312560115e-11 6.97590507139978e-11 100
"chr19" 3360001 3361000 "*" 0 0 -52.4949795368464
"chr19" 3367001 3368000 "*" 0 0 -62.3209610349607
"chr19" 3371001 3372000 "*" 0.00122932663261099 0.000960680434050461
-51.1904761904762
"chr19" 3413001 3414000 "*" 2.69018141096922e-12 7.41487347763769e-12 -100
"chr19" 3415001 3416000 "*" 4.86753970463383e-11 1.13145908161915e-10 -100
"chr19" 3429001 3430000 "*" 0 0 -74.4444444444444
"chr19" 3458001 3459000 "*" 0 0 59.2233009708738
"chr19" 3470001 3471000 "*" 0.000412523446254087 0.000349386048469569 -52
"chr19" 3477001 3478000 "*" 2.00227675550835e-08 3.20459176617961e-08 100
"chr19" 3556001 3557000 "*" 3.34128893442198e-08 5.19772864618523e-08 -100
"chr19" 3606001 3607000 "*" 0 0 -67.3913043478261
"chr19" 3627001 3628000 "*" 0 0 100
"chr19" 3703001 3704000 "*" 1.45734879719583e-09 2.76054203852193e-09
-83.6363636363636
"chr19" 3706001 3707000 "*" 2.4535928844216e-14 8.69144396197119e-14 -100
"chr19" 3718001 3719000 "*" 0 0 -100
"chr19" 3732001 3733000 "*" 0.000361273490986247 0.000308833981928707
52.3809523809524
"chr19" 3811001 3812000 "*" 0 0 68.4957924263675
"chr19" 3832001 3833000 "*" 1.89581683684992e-12 5.32311367606535e-12 -100
"chr19" 3841001 3842000 "*" 5.14355180669668e-09 8.98943431639241e-09 -76.25
"chr19" 3862001 3863000 "*" 1.54065649127233e-12 4.3771699936297e-12
53.4883720930233
"chr19" 3899001 3900000 "*" 2.22044604925031e-16 9.81641919380259e-16 70
"chr19" 3905001 3906000 "*" 3.27812577083364e-09 5.90709275634358e-09
-88.2352941176471
"chr19" 3927001 3928000 "*" 0 0 54.163364259406
"chr19" 3936001 3937000 "*" 0 0 85.3174603174603
"chr19" 3937001 3938000 "*" 4.59785987061423e-10 9.36261732495609e-10
58.0246913580247

"chr19" 3941001 3942000 "*" 1.84915416312492e-11 4.56517512311393e-11
71.5909090909091
"chr19" 3969001 3970000 "*" 3.08261416392952e-10 6.42152487162507e-10
-71.4285714285714
"chr19" 4081001 4082000 "*" 1.12687636999453e-13 3.68543487142567e-13
-92.3076923076923
"chr19" 4158001 4159000 "*" 2.79041212691844e-07 3.81718497248826e-07
65.7142857142857
"chr19" 4189001 4190000 "*" 2.63805643996307e-11 6.3791170118387e-11 75
"chr19" 4193001 4194000 "*" 2.08814465718632e-09 3.86232567960463e-09 -100
"chr19" 4194001 4195000 "*" 1.49983359065686e-11 3.74265952963154e-11
52.7777777777778
"chr19" 4264001 4265000 "*" 1.99652748777979e-08 3.20459176617961e-08
54.1666666666667
"chr19" 4302001 4303000 "*" 0 0 53.8461538461538
"chr19" 4304001 4305000 "*" 0 0 55.6164383561644
"chr19" 4312001 4313000 "*" 1.26529675625875e-10 2.78280435481264e-10
76.5957446808511
"chr19" 4317001 4318000 "*" 3.76365605347928e-14 1.3043487970679e-13 -100
"chr19" 4338001 4339000 "*" 9.65729496371637e-10 1.87255912493232e-09 -100
"chr19" 4392001 4393000 "*" 9.43689570931383e-15 3.51625644188498e-14
-51.8166383701188
"chr19" 4399001 4400000 "*" 2.0231327613196e-08 3.23573586384923e-08
52.2088353413655
"chr19" 4466001 4467000 "*" 6.88338275267597e-15 2.60518659957509e-14 60
"chr19" 4470001 4471000 "*" 2.00227675550835e-08 3.20459176617961e-08 100
"chr19" 4553001 4554000 "*" 9.41103766758378e-05 8.86297231089954e-05 -60
"chr19" 4575001 4576000 "*" 7.0006535457523e-08 1.03695832512054e-07 100
"chr19" 4578001 4579000 "*" 3.40340631888036e-06 3.99960927822812e-06
66.6666666666667
"chr19" 4584001 4585000 "*" 0 0 55.425219941349
"chr19" 4598001 4599000 "*" 0 0 90.1578813343519
"chr19" 4599001 4600000 "*" 7.7715611723761e-16 3.26213507634405e-15 100
"chr19" 4612001 4613000 "*" 5.3780302986417e-05 5.26241567504211e-05
54.5454545454545
"chr19" 4626001 4627000 "*" 0.000525299954949099 0.000436849389802675
54.5454545454545
"chr19" 4726001 4727000 "*" 1.29037891483108e-11 3.24582280286866e-11 -100
"chr19" 4745001 4746000 "*" 1.46359212694902e-05 1.56374814385865e-05
-62.8571428571429
"chr19" 4868001 4869000 "*" 8.61100080129518e-12 2.21874526827309e-11
-62.8571428571429
"chr19" 4872001 4873000 "*" 5.794809077031e-12 1.52911192757929e-11 100
"chr19" 4874001 4875000 "*" 1.13140401492018e-08 1.87801380787728e-08 100
"chr19" 4913001 4914000 "*" 9.65338919911574e-13 2.80678910656113e-12 100
"chr19" 4958001 4959000 "*" 0.000144235310220231 0.000131787791518504 -57.5
"chr19" 4968001 4969000 "*" 0 0 -66.6666666666667
"chr19" 5046001 5047000 "*" 0 0 52.7777777777778
"chr19" 5170001 5171000 "*" 1.20591092667155e-10 2.66075324129686e-10 100
"chr19" 5194001 5195000 "*" 3.90465437760668e-13 1.19307893943277e-12 100
"chr19" 5267001 5268000 "*" 0 0 -100
"chr19" 5282001 5283000 "*" 0 0 -72.3404255319149

"chr19" 5288001 5289000 "*" 7.68311825383172e-07 9.90964461140931e-07 67.741935483871
"chr19" 5301001 5302000 "*" 5.7065463465733e-14 1.93318145576218e-13 -100
"chr19" 5311001 5312000 "*" 9.50350909079134e-13 2.76967984704842e-12 -62.5
"chr19" 5326001 5327000 "*" 1.7814786912318e-07 2.49779877419423e-07 -66.6666666666667
"chr19" 5330001 5331000 "*" 0.000334851736096708 0.000287983064674082 55.5555555555556
"chr19" 5340001 5341000 "*" 0 0 70.0392402114066
"chr19" 5341001 5342000 "*" 0 0 70.2364018153492
"chr19" 5353001 5354000 "*" 0 0 100
"chr19" 5368001 5369000 "*" 1.92946192356658e-09 3.59193011611483e-09 -100
"chr19" 5392001 5393000 "*" 5.3804560806725e-10 1.08510370736308e-09 70.9677419354839
"chr19" 5409001 5410000 "*" 0 0 66.6666666666667
"chr19" 5432001 5433000 "*" 6.62958576924666e-12 1.73620572057889e-11 -100
"chr19" 5459001 5460000 "*" 1.83186799063151e-14 6.59696304569048e-14 100
"chr19" 5465001 5466000 "*" 1.95399252334028e-14 7.01253661173697e-14 -100
"chr19" 5524001 5525000 "*" 7.7715611723761e-15 2.92461699448967e-14 70.8333333333333
"chr19" 5530001 5531000 "*" 4.73652181920414e-08 7.20632688643672e-08 -61.1111111111111
"chr19" 5554001 5555000 "*" 3.6700841921089e-08 5.65530226072258e-08 -100
"chr19" 5557001 5558000 "*" 0 0 66.6666666666667
"chr19" 5567001 5568000 "*" 0 0 57.4024910008454
"chr19" 5578001 5579000 "*" 0 0 -100
"chr19" 5623001 5624000 "*" 0 0 -100
"chr19" 5673001 5674000 "*" 4.48240380368503e-08 6.84329675653433e-08 78.8461538461538
"chr19" 5710001 5711000 "*" 4.77395900588817e-15 1.84201484337652e-14 -85.7142857142857
"chr19" 5751001 5752000 "*" 0 0 -100
"chr19" 5766001 5767000 "*" 4.09752232144456e-11 9.61094067152364e-11 -77.7777777777778
"chr19" 5771001 5772000 "*" 3.31723681645801e-06 3.90563925837338e-06 -69.6629213483146
"chr19" 5805001 5806000 "*" 2.49878417957206e-09 4.57068411345896e-09 -76.0517799352751
"chr19" 5814001 5815000 "*" 1.29037891483108e-11 3.24582280286866e-11 100
"chr19" 5823001 5824000 "*" 2.66453525910038e-15 1.05861327033776e-14 -65.3603603603604
"chr19" 5875001 5876000 "*" 2.44249065417534e-15 9.75180348030994e-15 100
"chr19" 5954001 5955000 "*" 0 0 100
"chr19" 5961001 5962000 "*" 0 0 64.2857142857143
"chr19" 5983001 5984000 "*" 3.05311331771918e-12 8.34639368772922e-12 -100
"chr19" 6001001 6002000 "*" 0 0 78.5714285714286
"chr19" 6009001 6010000 "*" 1.57726276484027e-10 3.42003519985517e-10 -54.5454545454545
"chr19" 6075001 6076000 "*" 1.75415237890775e-14 6.3304859797472e-14 -60.2564102564103
"chr19" 6132001 6133000 "*" 3.02680103203556e-12 8.28043415536192e-12 -60
"chr19" 6133001 6134000 "*" 1.52233781136601e-12 4.33146951730515e-12

-59.0909090909091
"chr19" 6138001 6139000 "*" 2.64951482975562e-09 4.82186011375752e-09 -100
"chr19" 6239001 6240000 "*" 0 0 -51.3787463867336
"chr19" 6279001 6280000 "*" 0 0 -64.5161290322581
"chr19" 6287001 6288000 "*" 3.31690230837012e-12 9.03347040781321e-12 -100
"chr19" 6307001 6308000 "*" 1.45326750633501e-10 3.16772575693255e-10
51.5151515151515
"chr19" 6340001 6341000 "*" 1.29037891483108e-11 3.24582280286866e-11 100
"chr19" 6348001 6349000 "*" 3.41968636075052e-07 4.62340891767081e-07
-54.656862745098
"chr19" 6476001 6477000 "*" 0 0 81.0606060606061
"chr19" 6485001 6486000 "*" 3.00432619007029e-05 3.05952547348071e-05
58.3333333333333
"chr19" 6502001 6503000 "*" 4.21118695470568e-11 9.86866523622886e-11
89.1637220259128
"chr19" 6539001 6540000 "*" 6.45987785041768e-06 7.28081397894206e-06
68.4210526315789
"chr19" 6587001 6588000 "*" 0 0 76.3888888888889
"chr19" 6588001 6589000 "*" 1.11022302462516e-16 5.03662826618488e-16
84.4444444444444
"chr19" 6591001 6592000 "*" 5.55111512312578e-16 2.36485094870365e-15
89.8305084745763
"chr19" 6604001 6605000 "*" 0 0 100
"chr19" 6632001 6633000 "*" 3.36302354964069e-07 4.55012026436444e-07
-71.4285714285714
"chr19" 6668001 6669000 "*" 0 0 52.2507775413325
"chr19" 6696001 6697000 "*" 0 0 71.7647058823529
"chr19" 6706001 6707000 "*" 4.04588473834622e-10 8.29656529313599e-10 60
"chr19" 6711001 6712000 "*" 9.84656800540051e-13 2.86076302657953e-12
-59.5744680851064
"chr19" 6740001 6741000 "*" 3.33066907387547e-16 1.4495649018245e-15 75
"chr19" 6758001 6759000 "*" 6.88338275267597e-15 2.60518659957509e-14 100
"chr19" 6877001 6878000 "*" 0.000120483966197349 0.000111549020346707
-52.1739130434783
"chr19" 6933001 6934000 "*" 9.63829016598083e-12 2.46993429847588e-11 -100
"chr19" 6937001 6938000 "*" 8.57092175010621e-13 2.51149198099779e-12 -100
"chr19" 6965001 6966000 "*" 1.54630752646767e-11 3.85079502648228e-11 100
"chr19" 7005001 7006000 "*" 1.14130926931466e-13 3.7276674571693e-13 -100
"chr19" 7017001 7018000 "*" 1.4432899320127e-15 5.90750815956055e-15 100
"chr19" 7101001 7102000 "*" 3.03090885722668e-14 1.06210046699299e-13 100
"chr19" 7119001 7120000 "*" 1.0325074129014e-14 3.83008663711937e-14
-80.4347826086957
"chr19" 7159001 7160000 "*" 6.59550958292954e-09 1.13456194382773e-08 100
"chr19" 7216001 7217000 "*" 4.34485171507681e-07 5.78846442177264e-07
-77.2727272727273
"chr19" 7252001 7253000 "*" 6.17623951981727e-10 1.23513974823649e-09
79.4117647058823
"chr19" 7253001 7254000 "*" 3.58894225660489e-06 4.20417278477504e-06
-63.6363636363636
"chr19" 7401001 7402000 "*" 3.65831971539388e-05 3.6733698352541e-05 -65.625
"chr19" 7404001 7405000 "*" 2.73625566649116e-12 7.52483363579137e-12 100
"chr19" 7420001 7421000 "*" 4.87331952570003e-10 9.8856410225085e-10

-80.8510638297872
"chr19" 7429001 7430000 "*" 1.5277158427196e-09 2.87642564232045e-09 -100
"chr19" 7448001 7449000 "*" 5.15087635877265e-08 7.79620845570711e-08
57.6923076923077
"chr19" 7454001 7455000 "*" 1.17702637125738e-06 1.48016917543092e-06
90.9090909090909
"chr19" 7485001 7486000 "*" 9.65729496371637e-10 1.87255912493232e-09 -100
"chr19" 7523001 7524000 "*" 0 0 -100
"chr19" 7545001 7546000 "*" 1.22124532708767e-15 5.03921984217778e-15 100
"chr19" 7565001 7566000 "*" 0 0 53.0317457089991
"chr19" 7569001 7570000 "*" 1.9373391779709e-13 6.15971322898373e-13 100
"chr19" 7572001 7573000 "*" 0.00018629777499668 0.000167167295482563
-55.5555555555556
"chr19" 7662001 7663000 "*" 1.4086509736444e-12 4.02599201497498e-12
-62.1621621621622
"chr19" 7722001 7723000 "*" 1.19777055275216e-05 1.29698262693693e-05
58.3333333333333
"chr19" 7737001 7738000 "*" 0 0 -100
"chr19" 7744001 7745000 "*" 0 0 -50.1541911970844
"chr19" 7766001 7767000 "*" 1.6741612744009e-05 1.7735042630379e-05
64.3835616438356
"chr19" 7790001 7791000 "*" 1.98325800226939e-11 4.85612402473442e-11 100
"chr19" 7846001 7847000 "*" 0 0 100
"chr19" 7896001 7897000 "*" 2.51376697235628e-12 6.95425473559287e-12
52.9941860465116
"chr19" 7904001 7905000 "*" 2.96999909110696e-08 4.65750338512475e-08
81.6666666666667
"chr19" 7909001 7910000 "*" 4.87370939161735e-08 7.40383898157822e-08 60
"chr19" 7936001 7937000 "*" 2.70334867649158e-05 2.77251800014148e-05
-53.393665158371
"chr19" 7953001 7954000 "*" 0 0 -66.9444444444444
"chr19" 7986001 7987000 "*" 5.46573797333139e-09 9.5245791147007e-09
-79.4871794871795
"chr19" 8001001 8002000 "*" 1.89581683684992e-12 5.32311367606535e-12 100
"chr19" 8009001 8010000 "*" 2.81785705880111e-12 7.73317679055585e-12
-85.4838709677419
"chr19" 8035001 8036000 "*" 4.44089209850063e-15 1.71914916534614e-14
-73.6842105263158
"chr19" 8072001 8073000 "*" 1.13140401492018e-08 1.87801380787728e-08 100
"chr19" 8091001 8092000 "*" 1.53099755095809e-13 4.92350824434347e-13
-73.5294117647059
"chr19" 8151001 8152000 "*" 0 0 -100
"chr19" 8165001 8166000 "*" 6.98330282489223e-14 2.34267456154807e-13 100
"chr19" 8214001 8215000 "*" 0 0 82.3308270676692
"chr19" 8223001 8224000 "*" 1.37828859436695e-10 3.01445544267661e-10 -100
"chr19" 8236001 8237000 "*" 1.83952852950142e-12 5.17499701329218e-12 100
"chr19" 8271001 8272000 "*" 0 0 100
"chr19" 8318001 8319000 "*" 0 0 -83.5820895522388
"chr19" 8323001 8324000 "*" 2.15307771611606e-11 5.25298918072789e-11
-53.5714285714286
"chr19" 8326001 8327000 "*" 4.44089209850063e-16 1.91071758245033e-15
-51.3513513513514

"chr19" 8475001 8476000 "*" 4.79616346638068e-14 1.64380445416596e-13 100
"chr19" 8477001 8478000 "*" 1.07882591748876e-11 2.74553537455803e-11 -100
"chr19" 8590001 8591000 "*" 0 0 61.025641025641
"chr19" 8591001 8592000 "*" 0 0 53.5084500658307
"chr19" 8625001 8626000 "*" 3.25818809512324e-05 3.29735679006858e-05
66.6666666666667
"chr19" 8662001 8663000 "*" 1.11022302462516e-16 5.03662826618488e-16 100
"chr19" 8666001 8667000 "*" 0 0 88.8888888888889
"chr19" 8671001 8672000 "*" 1.9373391779709e-13 6.15971322898373e-13 -100
"chr19" 8771001 8772000 "*" 1.98124849859482e-10 4.25157611354621e-10 100
"chr19" 8786001 8787000 "*" 3.90465437760668e-13 1.19307893943277e-12 -100
"chr19" 8806001 8807000 "*" 2.94992988902898e-06 3.50095638154591e-06
73.8095238095238
"chr19" 8815001 8816000 "*" 2.4535928844216e-14 8.69144396197119e-14 -100
"chr19" 8942001 8943000 "*" 0 0 51.7786561264822
"chr19" 9006001 9007000 "*" 3.03090885722668e-14 1.06210046699299e-13 -100
"chr19" 9033001 9034000 "*" 0 0 53.476080769752
"chr19" 9133001 9134000 "*" 2.91028312560115e-11 6.97590507139978e-11 -100
"chr19" 9420001 9421000 "*" 6.66133814775094e-16 2.81595744474255e-15
-73.134328358209
"chr19" 9476001 9477000 "*" 1.83952852950142e-12 5.17499701329218e-12 -100
"chr19" 9477001 9478000 "*" 7.03541702673505e-09 1.20625513126693e-08
65.6217345872518
"chr19" 9786001 9787000 "*" 0 0 -100
"chr19" 9841001 9842000 "*" 1.13140401492018e-08 1.87801380787728e-08 100
"chr19" 9849001 9850000 "*" 2.09067207990188e-11 5.1073083948126e-11
66.6666666666667
"chr19" 10009001 10010000 "*" 5.54573065530972e-05 5.41431433256124e-05
-54.0540540540541
"chr19" 10019001 10020000 "*" 1.55572221771649e-11 3.87331533968589e-11 -90
"chr19" 10041001 10042000 "*" 0 0 -100
"chr19" 10114001 10115000 "*" 1.93720595120794e-11 4.76309924836636e-11 100
"chr19" 10116001 10117000 "*" 3.06085157220082e-11 7.30553056241137e-11
61.8729096989967
"chr19" 10119001 10120000 "*" 6.99440505513849e-14 2.34592719571672e-13
-83.8235294117647
"chr19" 10121001 10122000 "*" 0 0 71.5203426124197
"chr19" 10144001 10145000 "*" 3.77475828372553e-15 1.47379557022071e-14 100
"chr19" 10163001 10164000 "*" 0 0 100
"chr19" 10166001 10167000 "*" 2.72646106247443e-07 3.73405126957647e-07
66.6666666666667
"chr19" 10177001 10178000 "*" 6.24654217240561e-10 1.2462108191303e-09 -100
"chr19" 10196001 10197000 "*" 0 0 73.9757480088972
"chr19" 10223001 10224000 "*" 0 0 -64.3485537722084
"chr19" 10229001 10230000 "*" 2.14348194749192e-09 3.9597724795727e-09 60
"chr19" 10339001 10340000 "*" 3.6700841921089e-08 5.65530226072258e-08 100
"chr19" 10360001 10361000 "*" 0 0 -100
"chr19" 10387001 10388000 "*" 2.28007638325423e-08 3.623267715404e-08 -75
"chr19" 10400001 10401000 "*" 0 0 72.2893569844789
"chr19" 10401001 10402000 "*" 0 0 52.7306138487735
"chr19" 10410001 10411000 "*" 2.66827121397917e-07 3.65988551657854e-07
77.9220779220779

"chr19" 10414001 10415000 "*" 0 0 -94.2028985507246
"chr19" 10499001 10500000 "*" 5.01111496662432e-05 4.92883234243094e-05 61.7021276595745
"chr19" 10501001 10502000 "*" 0 0 -100
"chr19" 10508001 10509000 "*" 0.000168529599185452 0.000152316702506419 -60
"chr19" 10527001 10528000 "*" 0 0 65.0157310813049
"chr19" 10530001 10531000 "*" 0 0 70.5203120194593
"chr19" 10589001 10590000 "*" 0 0 84.8214285714286
"chr19" 10614001 10615000 "*" 0 0 68.7222715173026
"chr19" 10628001 10629000 "*" 0 0 80.6366712620629
"chr19" 10631001 10632000 "*" 1.35336186701807e-13 4.37825147251811e-13 52.34375
"chr19" 10645001 10646000 "*" 6.75992928300673e-10 1.33934245657011e-09 85.7142857142857
"chr19" 10646001 10647000 "*" 1.50623957750895e-12 4.28756717183481e-12 -100
"chr19" 10692001 10693000 "*" 0 0 -52.2261198057205
"chr19" 10697001 10698000 "*" 0 0 62.4588174093983
"chr19" 10704001 10705000 "*" 3.6700841921089e-08 5.65530226072258e-08 -100
"chr19" 10705001 10706000 "*" 2.27526627316621e-07 3.14837392572814e-07 65
"chr19" 10727001 10728000 "*" 3.33066907387547e-16 1.4495649018245e-15 100
"chr19" 10763001 10764000 "*" 3.99791311167519e-13 1.22027792394232e-12 -100
"chr19" 10812001 10813000 "*" 0 0 100
"chr19" 10838001 10839000 "*" 1.26565424807268e-14 4.63923203146481e-14 100
"chr19" 10860001 10861000 "*" 1.48753432295257e-05 1.58772171415432e-05 -56.1616161616162
"chr19" 10963001 10964000 "*" 6.77335965093562e-12 1.76897011246462e-11 100
"chr19" 10975001 10976000 "*" 6.4152538836737e-10 1.27477697728417e-09 -100
"chr19" 11038001 11039000 "*" 2.8590463330147e-12 7.84182776472793e-12 54.7169811320755
"chr19" 11210001 11211000 "*" 3.97779587046898e-11 9.36276770243261e-11 -85.7142857142857
"chr19" 11401001 11402000 "*" 0 0 -56.2881562881563
"chr19" 11407001 11408000 "*" 0 0 -100
"chr19" 11492001 11493000 "*" 0 0 63.9372822299652
"chr19" 11493001 11494000 "*" 5.10009012966162e-05 5.01055555267402e-05 53.2994923857868
"chr19" 11494001 11495000 "*" 0 0 73.2605502136752
"chr19" 11575001 11576000 "*" 0 0 -100
"chr19" 11583001 11584000 "*" 3.9190872769268e-14 1.35416418933905e-13 100
"chr19" 11638001 11639000 "*" 8.25450818808804e-13 2.42473843346228e-12 100
"chr19" 11640001 11641000 "*" 0 0 100
"chr19" 11641001 11642000 "*" 2.08995487582797e-10 4.46944006972663e-10 55.5555555555556
"chr19" 11646001 11647000 "*" 0 0 100
"chr19" 11651001 11652000 "*" 0.00020976332305167 0.000186660769941092 -52.3809523809524
"chr19" 11673001 11674000 "*" 1.4432899320127e-15 5.90750815956055e-15 100
"chr19" 11685001 11686000 "*" 0.000184006388740388 0.000165249884973406 -52.1739130434783
"chr19" 11878001 11879000 "*" 0 0 60.3448275862069
"chr19" 11909001 11910000 "*" 0 0 80.5555555555556
"chr19" 11999001 12000000 "*" 0 0 67.1232876712329

"chr19" 12034001 12035000 "*" 2.59712167327564e-08 4.09881271012004e-08 -65.625
"chr19" 12076001 12077000 "*" 6.76399873567046e-06 7.60077808447808e-06
-65.9420289855072
"chr19" 12369001 12370000 "*" 3.92685883809918e-13 1.19951200966592e-12 100
"chr19" 12476001 12477000 "*" 0 0 64.8604269293924
"chr19" 12664001 12665000 "*" 1.98325800226939e-11 4.85612402473442e-11 100
"chr19" 12757001 12758000 "*" 2.06340833308616e-10 4.42151263103378e-10
-57.8947368421053
"chr19" 12793001 12794000 "*" 0 0 -100
"chr19" 12860001 12861000 "*" 1.50723877823111e-12 4.29011858312785e-12
70.2702702702703
"chr19" 12861001 12862000 "*" 0 0 68.75
"chr19" 12862001 12863000 "*" 0 0 71.8181818181818
"chr19" 12863001 12864000 "*" 0 0 75.5122143420016
"chr19" 12864001 12865000 "*" 0 0 100
"chr19" 12867001 12868000 "*" 4.76285677564192e-14 1.63474041999827e-13
68.9655172413793
"chr19" 12871001 12872000 "*" 5.38014632844863e-10 1.08506747658349e-09
-70.5882352941177
"chr19" 12889001 12890000 "*" 0 0 50.6393861892583
"chr19" 12890001 12891000 "*" 2.02327044007689e-12 5.65300384792621e-12 100
"chr19" 12897001 12898000 "*" 0.000232511352137754 0.000205376073095269
-50.5555555555556
"chr19" 12900001 12901000 "*" 0 0 -58.6531088805242
"chr19" 12937001 12938000 "*" 5.22915044598449e-14 1.78122362258825e-13
-70.2702702702703
"chr19" 12992001 12993000 "*" 0 0 -76.4397905759162
"chr19" 12998001 12999000 "*" 0 0 85.4166666666667
"chr19" 13048001 13049000 "*" 1.04360964314765e-14 3.87023752069057e-14
75.7575757575758
"chr19" 13051001 13052000 "*" 0 0 -100
"chr19" 13099001 13100000 "*" 0 0 51.225873761085
"chr19" 13147001 13148000 "*" 0 0 100
"chr19" 13204001 13205000 "*" 1.2228967838368e-09 2.33644378479772e-09
66.6666666666667
"chr19" 13251001 13252000 "*" 2.17492690524068e-13 6.85423291094387e-13
90.9090909090909
"chr19" 13264001 13265000 "*" 0 0 -87.7777777777778
"chr19" 13265001 13266000 "*" 4.35375180352082e-10 8.89072096292233e-10
-70.8333333333333
"chr19" 13294001 13295000 "*" 6.4152538836737e-10 1.27477697728417e-09 100
"chr19" 13306001 13307000 "*" 2.44249065417534e-15 9.75180348030994e-15 -80
"chr19" 13335001 13336000 "*" 2.22044604925031e-16 9.81641919380259e-16
57.843137254902
"chr19" 13510001 13511000 "*" 1.07882591748876e-11 2.74553537455803e-11 100
"chr19" 13540001 13541000 "*" 5.72310759449079e-05 5.57606548799911e-05
-58.974358974359
"chr19" 13717001 13718000 "*" 2.79440914852103e-11 6.73046092948135e-11 -100
"chr19" 13789001 13790000 "*" 2.43005615629954e-09 4.45014222280278e-09 -100
"chr19" 13884001 13885000 "*" 4.01313771103418e-09 7.11882087237587e-09 -100
"chr19" 13886001 13887000 "*" 3.34128893442198e-08 5.19772864618523e-08 -100
"chr19" 13896001 13897000 "*" 1.98365768255826e-11 4.85612402473442e-11 -100

"chr19" 13899001 13900000 "*" 1.48087875295744e-10 3.22075573380618e-10 100
"chr19" 13926001 13927000 "*" 2.88779000712225e-11 6.92935809466068e-11 100
"chr19" 13951001 13952000 "*" 0 0 -83.1460674157303
"chr19" 13952001 13953000 "*" 0 0 -94.0928270042194
"chr19" 13954001 13955000 "*" 0 0 -85.1851851851852
"chr19" 13957001 13958000 "*" 0 0 -78.7878787878788
"chr19" 13960001 13961000 "*" 4.08209022140227e-11 9.57770690255255e-11 -100
"chr19" 13962001 13963000 "*" 3.6472261467746e-05 3.66333312434789e-05
-51.8987341772152
"chr19" 13982001 13983000 "*" 1.63283829768268e-06 2.01223257972618e-06
63.1578947368421
"chr19" 13985001 13986000 "*" 0.00108442561824407 0.000855136979599194
-55.7142857142857
"chr19" 13992001 13993000 "*" 0 0 -62.7118644067797
"chr19" 14064001 14065000 "*" 0.000176914307783993 0.000159330245150163
-58.0645161290323
"chr19" 14108001 14109000 "*" 1.69905867153375e-10 3.67390035933181e-10
67.6470588235294
"chr19" 14137001 14138000 "*" 7.93809462606987e-14 2.64679416473686e-13 -100
"chr19" 14143001 14144000 "*" 0 0 -100
"chr19" 14162001 14163000 "*" 5.48638023900594e-10 1.10374357336421e-09 -100
"chr19" 14169001 14170000 "*" 0 0 -50.5090475243587
"chr19" 14179001 14180000 "*" 1.11022302462516e-16 5.03662826618488e-16
71.4285714285714
"chr19" 14186001 14187000 "*" 1.11022302462516e-16 5.03662826618488e-16 100
"chr19" 14190001 14191000 "*" 5.01025332333427e-10 1.01358031135889e-09 100
"chr19" 14239001 14240000 "*" 0 0 100
"chr19" 14264001 14265000 "*" 8.51541059887495e-14 2.82899609197464e-13 -75
"chr19" 14459001 14460000 "*" 0 0 -100
"chr19" 14462001 14463000 "*" 1.63039848288804e-09 3.06128978817594e-09 -75
"chr19" 14495001 14496000 "*" 2.91028312560115e-11 6.97590507139978e-11 -100
"chr19" 14543001 14544000 "*" 2.77555756156289e-15 1.10015415195978e-14
69.3069306930693
"chr19" 14617001 14618000 "*" 0 0 -93.3333333333333
"chr19" 14625001 14626000 "*" 1.5277158427196e-09 2.87642564232045e-09 100
"chr19" 14653001 14654000 "*" 0 0 -100
"chr19" 14667001 14668000 "*" 0 0 -63.3449477351916
"chr19" 14854001 14855000 "*" 3.88022947106492e-13 1.1867462770515e-12 -100
"chr19" 14887001 14888000 "*" 0 0 70.0737618545838
"chr19" 14955001 14956000 "*" 2.88710388929303e-10 6.03399264731139e-10 100
"chr19" 15064001 15065000 "*" 9.72048715186524e-07 1.23551481856642e-06
-75.9259259259259
"chr19" 15164001 15165000 "*" 3.69853481174687e-10 7.61762233142457e-10
87.9120879120879
"chr19" 15251001 15252000 "*" 3.04324343503026e-11 7.26599935673121e-11 100
"chr19" 15309001 15310000 "*" 0 0 -62.6393570132227
"chr19" 15331001 15332000 "*" 1.35016442470715e-11 3.38925638226179e-11
78.4313725490196
"chr19" 15337001 15338000 "*" 4.78841410966879e-11 1.11468187156588e-10 -100
"chr19" 15444001 15445000 "*" 3.57390039784633e-09 6.41139565671457e-09
52.9411764705882
"chr19" 15619001 15620000 "*" 0 0 56.6134522268565

"chr19" 15623001 15624000 "*" 1.39779633423487e-07 1.98463037958589e-07 100
"chr19" 15629001 15630000 "*" 1.1304871979867e-05 1.22875265834422e-05
-61.0169491525424
"chr19" 15630001 15631000 "*" 5.295763827462e-14 1.80214784640623e-13 -100
"chr19" 15641001 15642000 "*" 3.6700841921089e-08 5.65530226072258e-08 -100
"chr19" 15659001 15660000 "*" 3.33066907387547e-16 1.4495649018245e-15 83.75
"chr19" 15672001 15673000 "*" 1.50138567134306e-08 2.45599831107531e-08 60
"chr19" 15696001 15697000 "*" 1.98124849859482e-10 4.25157611354621e-10 -100
"chr19" 15942001 15943000 "*" 3.6700841921089e-08 5.65530226072258e-08 -100
"chr19" 16014001 16015000 "*" 2.4144371718382e-05 2.49591887405302e-05 -54.6875
"chr19" 16045001 16046000 "*" 3.00432619007029e-05 3.05952547348071e-05
58.3333333333333
"chr19" 16132001 16133000 "*" 8.96610711231816e-08 1.30861118541309e-07 -75
"chr19" 16147001 16148000 "*" 2.7586291362347e-07 3.77618449362166e-07
68.2926829268293
"chr19" 16149001 16150000 "*" 0 0 71.6814159292035
"chr19" 16178001 16179000 "*" 0 0 -65.1689288565786
"chr19" 16179001 16180000 "*" 1.11022302462516e-16 5.03662826618488e-16
-61.9047619047619
"chr19" 16214001 16215000 "*" 4.78841410966879e-11 1.11468187156588e-10 -100
"chr19" 16267001 16268000 "*" 1.35036426485158e-12 3.86629547363774e-12 100
"chr19" 16298001 16299000 "*" 7.0006535457523e-08 1.03695832512054e-07 100
"chr19" 16302001 16303000 "*" 0 0 72.9166666666667
"chr19" 16364001 16365000 "*" 7.45486561370967e-10 1.46952418634053e-09
58.3333333333333
"chr19" 16388001 16389000 "*" 1.5320073147973e-05 1.63222378406705e-05
63.6363636363636
"chr19" 16391001 16392000 "*" 1.11022302462516e-16 5.03662826618488e-16 100
"chr19" 16398001 16399000 "*" 0 0 -99.1803278688525
"chr19" 16412001 16413000 "*" 0 0 -69.6498054474708
"chr19" 16413001 16414000 "*" 0 0 -60
"chr19" 16417001 16418000 "*" 0 0 100
"chr19" 16430001 16431000 "*" 7.99844594256971e-06 8.89173797399106e-06
54.5454545454545
"chr19" 16439001 16440000 "*" 0 0 100
"chr19" 16450001 16451000 "*" 8.7349629751543e-09 1.47505871898511e-08 -100
"chr19" 16460001 16461000 "*" 6.04036487494852e-10 1.20941720008158e-09
-80.2325581395349
"chr19" 16476001 16477000 "*" 1.12997596835029e-08 1.87801380787728e-08
51.063829787234
"chr19" 16552001 16553000 "*" 0 0 93.7759336099585
"chr19" 16606001 16607000 "*" 1.11022302462516e-15 4.59817122935606e-15
-84.180790960452
"chr19" 16670001 16671000 "*" 3.90465437760668e-13 1.19307893943277e-12 100
"chr19" 16700001 16701000 "*" 2.64951482975562e-09 4.82186011375752e-09 100
"chr19" 16706001 16707000 "*" 6.24654217240561e-10 1.2462108191303e-09 100
"chr19" 16762001 16763000 "*" 3.69056931814882e-06 4.31497859976197e-06
57.5757575757576
"chr19" 16772001 16773000 "*" 0 0 78.0322862129145
"chr19" 16797001 16798000 "*" 3.6700841921089e-08 5.65530226072258e-08 -100
"chr19" 16800001 16801000 "*" 0 0 98.780487804878
"chr19" 16939001 16940000 "*" 6.13977297891921e-08 9.20427346498613e-08

78.4810126582279
"chr19" 17017001 17018000 "*" 0 0 70
"chr19" 17049001 17050000 "*" 9.04570973681018e-10 1.76414389822173e-09 100
"chr19" 17085001 17086000 "*" 8.01495584346057e-08 1.17737191285945e-07
87.0967741935484
"chr19" 17087001 17088000 "*" 1.46184566574004e-06 1.81497227178446e-06
-58.3333333333333
"chr19" 17119001 17120000 "*" 9.0072393987839e-13 2.63157725646847e-12 -100
"chr19" 17126001 17127000 "*" 1.12458486967171e-09 2.15740815043925e-09 100
"chr19" 17301001 17302000 "*" 1.1144021427878e-07 1.60649074243831e-07
58.0645161290323
"chr19" 17312001 17313000 "*" 0 0 50.6700616456714
"chr19" 17346001 17347000 "*" 0 0 -72.8146853146853
"chr19" 17392001 17393000 "*" 0 0 50.9332217735579
"chr19" 17393001 17394000 "*" 0 0 63.6369608580882
"chr19" 17400001 17401000 "*" 2.13922224379104e-07 2.96983147907201e-07 56
"chr19" 17413001 17414000 "*" 9.33704780159417e-10 1.8181137360611e-09
-54.5454545454545
"chr19" 17422001 17423000 "*" 8.36652969127272e-12 2.16357448596883e-11
86.7647058823529
"chr19" 17457001 17458000 "*" 0 0 84.7826086956522
"chr19" 17481001 17482000 "*" 1.96509475358653e-13 6.23868565133654e-13 100
"chr19" 17494001 17495000 "*" 9.47020240005259e-14 3.12491189162604e-13 -100
"chr19" 17505001 17506000 "*" 0 0 52.5403935370341
"chr19" 17508001 17509000 "*" 2.0874876538457e-07 2.90229945171996e-07
68.5714285714286
"chr19" 17513001 17514000 "*" 2.41051623106614e-12 6.68546884798253e-12
-63.1578947368421
"chr19" 17516001 17517000 "*" 0 0 -100
"chr19" 17558001 17559000 "*" 0 0 91.1392405063291
"chr19" 17559001 17560000 "*" 0 0 62.7197039777983
"chr19" 17564001 17565000 "*" 0 0 -100
"chr19" 17599001 17600000 "*" 0 0 52.8888404691869
"chr19" 17603001 17604000 "*" 0.000334851753864718 0.000287983064674082
55.5555555555556
"chr19" 17607001 17608000 "*" 1.28141512499846e-05 1.3811181398921e-05
-66.1290322580645
"chr19" 17623001 17624000 "*" 5.44009282066327e-15 2.08486137681812e-14
-70.7692307692308
"chr19" 17712001 17713000 "*" 5.55111512312578e-16 2.36485094870365e-15
71.4285714285714
"chr19" 17715001 17716000 "*" 1.39779633423487e-07 1.98463037958589e-07 -100
"chr19" 17726001 17727000 "*" 0 0 100
"chr19" 17729001 17730000 "*" 3.95353005888666e-09 7.03928529283733e-09 100
"chr19" 17735001 17736000 "*" 2.36019946919797e-08 3.74360093415148e-08
-63.1578947368421
"chr19" 17743001 17744000 "*" 3.88022947106492e-13 1.1867462770515e-12 100
"chr19" 17763001 17764000 "*" 0.000672965876117981 0.000549698110587813 52
"chr19" 17771001 17772000 "*" 2.08037405902139e-06 2.52539366890153e-06
52.3809523809524
"chr19" 17778001 17779000 "*" 1.13140401492018e-08 1.87801380787728e-08 100
"chr19" 17796001 17797000 "*" 4.37740464850345e-05 4.3441456827013e-05

-53.9473684210526
"chr19" 17805001 17806000 "*" 8.88178419700125e-16 3.70670032207938e-15
-97.5609756097561
"chr19" 17816001 17817000 "*" 9.65729496371637e-10 1.87255912493232e-09 -100
"chr19" 17885001 17886000 "*" 5.32907051820075e-15 2.04626240984625e-14
54.1666666666667
"chr19" 17914001 17915000 "*" 6.7518801979416e-06 7.58814969368226e-06
53.8461538461538
"chr19" 17950001 17951000 "*" 7.03066994844903e-07 9.11435281289134e-07 -65.625
"chr19" 17952001 17953000 "*" 0 0 -52.8054298642534
"chr19" 17959001 17960000 "*" 6.59550958292954e-09 1.13456194382773e-08 100
"chr19" 17972001 17973000 "*" 1.16540110894903e-12 3.36292522093995e-12 -60
"chr19" 17980001 17981000 "*" 1.55431223447522e-15 6.33705141101682e-15
58.8888888888889
"chr19" 17984001 17985000 "*" 0 0 62.3162393162393
"chr19" 17985001 17986000 "*" 0 0 63.9122213946027
"chr19" 17993001 17994000 "*" 5.63660229602192e-13 1.68905058935478e-12 -100
"chr19" 17999001 18000000 "*" 1.92557081390987e-12 5.40156563669536e-12
60.6060606060606
"chr19" 18027001 18028000 "*" 6.15840711759574e-13 1.83676803572802e-12 100
"chr19" 18067001 18068000 "*" 1.98325800226939e-11 4.85612402473442e-11 100
"chr19" 18075001 18076000 "*" 0 0 67.5675675675676
"chr19" 18096001 18097000 "*" 1.05025862562336e-07 1.51993443189061e-07 70
"chr19" 18099001 18100000 "*" 1.33226762955019e-15 5.48133041560118e-15
55.4385964912281
"chr19" 18169001 18170000 "*" 5.44009282066327e-15 2.08486137681812e-14
66.6666666666667
"chr19" 18181001 18182000 "*" 1.51745493948141e-09 2.86872788949267e-09
53.2796317606444
"chr19" 18183001 18184000 "*" 2.33028818463765e-10 4.93719283454501e-10 100
"chr19" 18207001 18208000 "*" 2.43005615629954e-09 4.45014222280278e-09 100
"chr19" 18208001 18209000 "*" 0 0 -91.6666666666667
"chr19" 18220001 18221000 "*" 7.26425190101798e-05 6.96129177828238e-05
-58.5749385749386
"chr19" 18228001 18229000 "*" 0 0 65.0793650793651
"chr19" 18324001 18325000 "*" 8.47300007933427e-12 2.18815101835634e-11 68.75
"chr19" 18369001 18370000 "*" 9.50946665856378e-09 1.59859741017156e-08
-85.3658536585366
"chr19" 18370001 18371000 "*" 0 0 100
"chr19" 18385001 18386000 "*" 5.47654960270094e-08 8.26483884363011e-08
-61.1940298507463
"chr19" 18410001 18411000 "*" 7.54951656745106e-14 2.5237892556906e-13 -100
"chr19" 18411001 18412000 "*" 4.43136049954607e-05 4.39299853239263e-05
-65.5172413793103
"chr19" 18452001 18453000 "*" 1.4432899320127e-15 5.90750815956055e-15 -100
"chr19" 18485001 18486000 "*" 6.57859990038645e-07 8.56558242696799e-07
-67.4418604651163
"chr19" 18491001 18492000 "*" 8.71525074330748e-14 2.88848349331391e-13 -100
"chr19" 18494001 18495000 "*" 7.65188132856309e-08 1.12691547930703e-07
-90.9090909090909
"chr19" 18496001 18497000 "*" 3.95930676022616e-08 6.0795810434514e-08 75
"chr19" 18513001 18514000 "*" 3.96347399345132e-11 9.32984740262792e-11

-66.6666666666667
"chr19" 18583001 18584000 "*" 1.07504017687887e-07 1.55356097428857e-07
-79.2452830188679
"chr19" 18599001 18600000 "*" 8.88432767354175e-08 1.29751535754657e-07
52.5490196078431
"chr19" 18615001 18616000 "*" 3.10862446895044e-15 1.22466437093774e-14 100
"chr19" 18633001 18634000 "*" 1.53310697470488e-12 4.3560177910091e-12 -78
"chr19" 18683001 18684000 "*" 1.77831194214662e-10 3.83841340108602e-10
69.5652173913043
"chr19" 18699001 18700000 "*" 0 0 -72
"chr19" 18715001 18716000 "*" 0 0 -83.6206896551724
"chr19" 18723001 18724000 "*" 0 0 62.5486840344539
"chr19" 18724001 18725000 "*" 1.33226762955019e-15 5.48133041560118e-15
-68.1818181818182
"chr19" 18738001 18739000 "*" 9.65338919911574e-13 2.80678910656113e-12 100
"chr19" 18781001 18782000 "*" 5.45864971002175e-08 8.23916743843603e-08
-61.5384615384615
"chr19" 18795001 18796000 "*" 3.6700841921089e-08 5.65530226072258e-08 100
"chr19" 18848001 18849000 "*" 4.77710127233522e-07 6.33085831703398e-07
-60.377358490566
"chr19" 18887001 18888000 "*" 0 0 -76.7441860465116
"chr19" 18889001 18890000 "*" 0 0 100
"chr19" 18891001 18892000 "*" 2.97528668369296e-12 8.14618637289158e-12
85.3403141361257
"chr19" 18914001 18915000 "*" 3.07531777821168e-14 1.07626052665081e-13 -100
"chr19" 19189001 19190000 "*" 8.53858295180032e-09 1.44808302221037e-08
-84.1269841269841
"chr19" 19205001 19206000 "*" 9.67004254448511e-14 3.18178990782609e-13 -100
"chr19" 19218001 19219000 "*" 7.71058427362359e-06 8.5933759499658e-06
-51.5151515151515
"chr19" 19224001 19225000 "*" 5.12901061511251e-07 6.76904585220232e-07 60
"chr19" 19229001 19230000 "*" 6.15840711759574e-13 1.83676803572802e-12 100
"chr19" 19248001 19249000 "*" 0 0 84.1530054644809
"chr19" 19252001 19253000 "*" 1.28785870856518e-14 4.71440608755782e-14
-66.8463611859838
"chr19" 19266001 19267000 "*" 8.96610728995384e-08 1.30861118541309e-07 -75
"chr19" 19276001 19277000 "*" 1.45061740397523e-12 4.14153438750877e-12
-51.6129032258064
"chr19" 19280001 19281000 "*" 1.05908241794239e-05 1.15588635632259e-05
-58.4905660377358
"chr19" 19283001 19284000 "*" 0 0 100
"chr19" 19318001 19319000 "*" 0 0 100
"chr19" 19335001 19336000 "*" 0 0 73.462783171521
"chr19" 19336001 19337000 "*" 0 0 100
"chr19" 19337001 19338000 "*" 0.000187069196050049 0.000167814363725717
55.3191489361702
"chr19" 19340001 19341000 "*" 0 0 100
"chr19" 19379001 19380000 "*" 1.92068583260152e-14 6.89775382589628e-14 -100
"chr19" 19383001 19384000 "*" 0 0 52.7910354845404
"chr19" 19385001 19386000 "*" 4.01313771103418e-09 7.11882087237587e-09 100
"chr19" 19444001 19445000 "*" 6.93112234273485e-13 2.05218842007357e-12 -100
"chr19" 19453001 19454000 "*" 0 0 50.2197802197802

"chr19" 19482001 19483000 "*" 1.49383437841877e-08 2.444556877867e-08 66.6666666666667
"chr19" 19496001 19497000 "*" 0 0 67.0212765957447
"chr19" 19528001 19529000 "*" 1.98310022181403e-07 2.76478060647682e-07 73.8095238095238
"chr19" 19547001 19548000 "*" 1.81642185118625e-05 1.91318178670518e-05 -53.125
"chr19" 19572001 19573000 "*" 7.43486490173417e-06 8.30651437189353e-06 67.5
"chr19" 19601001 19602000 "*" 3.44672468344243e-09 6.19257304743119e-09 -59.4594594594595
"chr19" 19618001 19619000 "*" 5.08482145278322e-14 1.73432706170696e-13 100
"chr19" 19649001 19650000 "*" 0 0 66.684413777437
"chr19" 19679001 19680000 "*" 4.99900121297969e-12 1.32921056339624e-11 100
"chr19" 19701001 19702000 "*" 2.18713935851156e-14 7.80893646430581e-14 100
"chr19" 19755001 19756000 "*" 6.5536465143623e-13 1.94602017656154e-12 100
"chr19" 19756001 19757000 "*" 4.21884749357559e-15 1.63874445239189e-14 -53.0890804597701
"chr19" 19773001 19774000 "*" 4.05744147502496e-06 4.71181941756713e-06 51.8518518518519
"chr19" 20150001 20151000 "*" 0 0 69.4851331784355
"chr19" 20168001 20169000 "*" 0 0 85.2941176470588
"chr19" 20175001 20176000 "*" 0 0 -70.2380952380952
"chr19" 20348001 20349000 "*" 0 0 81.6666666666667
"chr19" 20843001 20844000 "*" 0 0 54.7550547430672
"chr19" 20959001 20960000 "*" 6.3238185548653e-05 6.12087074932446e-05 57.8947368421053
"chr19" 21082001 21083000 "*" 9.08108033215171e-11 2.03407753381119e-10 100
"chr19" 21203001 21204000 "*" 4.32209823486573e-12 1.15935290479641e-11 100
"chr19" 21442001 21443000 "*" 2.71893618730701e-13 8.46692454027087e-13 100
"chr19" 21512001 21513000 "*" 0 0 -100
"chr19" 21579001 21580000 "*" 6.07958128284736e-13 1.81631077616203e-12 -66.6666666666667
"chr19" 21591001 21592000 "*" 0 0 -100
"chr19" 21626001 21627000 "*" 2.24265050974282e-14 7.99720436001354e-14 53
"chr19" 22004001 22005000 "*" 1.8757218001042e-12 5.27290364871055e-12 -94.2307692307692
"chr19" 22286001 22287000 "*" 6.15840711759574e-13 1.83676803572802e-12 100
"chr19" 22323001 22324000 "*" 1.32697596244569e-08 2.18565656423431e-08 57.1153846153846
"chr19" 22516001 22517000 "*" 9.04570973681018e-10 1.76414389822173e-09 -100
"chr19" 22725001 22726000 "*" 4.61597577161577e-06 5.31682843575882e-06 -55.5555555555556
"chr19" 22783001 22784000 "*" 5.794809077031e-12 1.52911192757929e-11 100
"chr19" 22809001 22810000 "*" 0 0 100
"chr19" 22816001 22817000 "*" 0 0 -71.4285714285714
"chr19" 22835001 22836000 "*" 0 0 100
"chr19" 23297001 23298000 "*" 0 0 -100
"chr19" 23375001 23376000 "*" 1.79824821699981e-10 3.87973806452552e-10 64.7058823529412
"chr19" 23378001 23379000 "*" 0 0 -100
"chr19" 24216001 24217000 "*" 0 0 100
"chr19" 24345001 24346000 "*" 5.55217207875991e-06 6.32277870260892e-06 53.3653846153846

"chr19" 28231001 28232000 "*" 0 0 100
"chr19" 28254001 28255000 "*" 1.4432899320127e-15 5.90750815956055e-15 100
"chr19" 28285001 28286000 "*" 1.29037891483108e-11 3.24582280286866e-11 -100
"chr19" 28402001 28403000 "*" 3.30069305221059e-13 1.01978254811217e-12 61.5384615384615
"chr19" 28794001 28795000 "*" 3.56775498033812e-10 7.35898629809829e-10 100
"chr19" 28960001 28961000 "*" 0 0 100
"chr19" 28980001 28981000 "*" 3.34128893442198e-08 5.19772864618523e-08 -100
"chr19" 28994001 28995000 "*" 1.77635683940025e-15 7.19870740878856e-15 100
"chr19" 29041001 29042000 "*" 5.51780843238703e-14 1.87243792572872e-13 -100
"chr19" 29145001 29146000 "*" 8.7349629751543e-09 1.47505871898511e-08 -100
"chr19" 29365001 29366000 "*" 1.83952852950142e-12 5.17499701329218e-12 100
"chr19" 29412001 29413000 "*" 2.70848504735355e-08 4.26721967434805e-08 87.0967741935484
"chr19" 29467001 29468000 "*" 1.28961938561378e-06 1.61298054297059e-06 61.1764705882353
"chr19" 29500001 29501000 "*" 1.41959555222115e-10 3.09813577051749e-10 -63.3333333333333
"chr19" 29506001 29507000 "*" 3.1151192736445e-11 7.43058913641707e-11 54.3859649122807
"chr19" 29513001 29514000 "*" 2.86262472615206e-08 4.4985942854623e-08 80.5555555555556
"chr19" 29535001 29536000 "*" 1.80365037882879e-05 1.90157129274617e-05 51.5151515151515
"chr19" 29565001 29566000 "*" 2.64951482975562e-09 4.82186011375752e-09 -100
"chr19" 29595001 29596000 "*" 3.09694846101216e-07 4.20986194852157e-07 -59.2592592592593
"chr19" 29716001 29717000 "*" 4.44089209850063e-15 1.71914916534614e-14 100
"chr19" 29724001 29725000 "*" 9.55097179122788e-09 1.60521875768559e-08 72.972972972973
"chr19" 29735001 29736000 "*" 1.14989052169001e-07 1.65478950456906e-07 73.1707317073171
"chr19" 29737001 29738000 "*" 6.08402217494586e-14 2.05454787937634e-13 -100
"chr19" 29791001 29792000 "*" 2.69018141096922e-12 7.41487347763769e-12 100
"chr19" 29800001 29801000 "*" 7.24555637710012e-10 1.43056379295798e-09 66.6666666666667
"chr19" 29895001 29896000 "*" 9.62807085691519e-06 1.05815014619344e-05 -63.6363636363636
"chr19" 29919001 29920000 "*" 0 0 100
"chr19" 29932001 29933000 "*" 4.91828799908944e-14 1.68192819018269e-13 100
"chr19" 29969001 29970000 "*" 5.00985586349145e-10 1.01358031135889e-09 -66.2337662337662
"chr19" 29987001 29988000 "*" 1.42567912925218e-05 1.52550383843778e-05 66.6666666666667
"chr19" 29995001 29996000 "*" 2.8590463330147e-12 7.84182776472793e-12 54.7169811320755
"chr19" 30006001 30007000 "*" 2.6013813325676e-09 4.74908099597444e-09 -93.75
"chr19" 30022001 30023000 "*" 0 0 -100
"chr19" 30053001 30054000 "*" 2.55071468613366e-07 3.50723571996354e-07 -68
"chr19" 30057001 30058000 "*" 0 0 77.0929635336415
"chr19" 30060001 30061000 "*" 0 0 100
"chr19" 30085001 30086000 "*" 4.08209022140227e-11 9.57770690255255e-11 100

"chr19" 30100001 30101000 "*" 3.530509218308e-14 1.22891039008692e-13 -100
"chr19" 30111001 30112000 "*" 4.07187450424118e-08 6.24351323038044e-08
-57.843137254902
"chr19" 30155001 30156000 "*" 3.24185900346663e-10 6.73391199139756e-10
-57.1428571428571
"chr19" 30156001 30157000 "*" 0 0 85.3354134165367
"chr19" 30176001 30177000 "*" 6.92287338566189e-11 1.57605630124247e-10 100
"chr19" 30185001 30186000 "*" 0 0 -79.4642857142857
"chr19" 30243001 30244000 "*" 1.80062161847516e-08 2.90920375744928e-08
80.4878048780488
"chr19" 30273001 30274000 "*" 0 0 -59.6153846153846
"chr19" 30349001 30350000 "*" 0 0 -73.6842105263158
"chr19" 30376001 30377000 "*" 1.11022302462516e-16 5.03662826618488e-16 100
"chr19" 30433001 30434000 "*" 0 0 -80.605937598022
"chr19" 30564001 30565000 "*" 0 0 -100
"chr19" 30580001 30581000 "*" 4.81873330127058e-09 8.45167747969663e-09
70.3703703703704
"chr19" 30588001 30589000 "*" 2.15125472990962e-10 4.58126659943874e-10 -100
"chr19" 30602001 30603000 "*" 2.40807374041196e-13 7.55672304266029e-13
51.1627906976744
"chr19" 30635001 30636000 "*" 6.54429038737803e-08 9.77502704411541e-08
56.5217391304348
"chr19" 30637001 30638000 "*" 0 0 56.2874251497006
"chr19" 30663001 30664000 "*" 3.95861121660346e-12 1.0684496262032e-11 -100
"chr19" 30804001 30805000 "*" 0 0 -100
"chr19" 30805001 30806000 "*" 0 0 -80.7692307692308
"chr19" 30835001 30836000 "*" 1.17461596005342e-13 3.8279067575877e-13 100
"chr19" 30852001 30853000 "*" 2.00227675550835e-08 3.20459176617961e-08 100
"chr19" 30896001 30897000 "*" 0 0 100
"chr19" 30897001 30898000 "*" 5.01025332333427e-10 1.01358031135889e-09 100
"chr19" 30959001 30960000 "*" 7.7715611723761e-16 3.26213507634405e-15 100
"chr19" 30989001 30990000 "*" 0 0 100
"chr19" 31031001 31032000 "*" 3.03090885722668e-14 1.06210046699299e-13 100
"chr19" 31085001 31086000 "*" 1.35003119794419e-13 4.3679847476192e-13 -100
"chr19" 31161001 31162000 "*" 1.2827964246398e-09 2.44562053411042e-09
-50.2267573696145
"chr19" 31177001 31178000 "*" 1.47803887560194e-05 1.5780310930685e-05
65.7142857142857
"chr19" 31218001 31219000 "*" 0 0 -65.0793650793651
"chr19" 31233001 31234000 "*" 2.00062189037453e-13 6.34809237673369e-13
-64.5161290322581
"chr19" 31270001 31271000 "*" 2.33590924381133e-13 7.3418676838825e-13 -100
"chr19" 31564001 31565000 "*" 4.99020824662466e-11 1.15790792586157e-10 -100
"chr19" 31580001 31581000 "*" 2.33028818463765e-10 4.93719283454501e-10 100
"chr19" 31654001 31655000 "*" 0 0 -100
"chr19" 31742001 31743000 "*" 7.0006535457523e-08 1.03695832512054e-07 -100
"chr19" 31780001 31781000 "*" 8.77076189453874e-14 2.90665046077958e-13
83.3333333333333
"chr19" 31787001 31788000 "*" 1.12634901405784e-10 2.49563754426169e-10 100
"chr19" 31794001 31795000 "*" 1.16462395283179e-13 3.80054190138805e-13
-70.2702702702703
"chr19" 31799001 31800000 "*" 2.84214862755761e-09 5.15421462588383e-09 95

"chr19" 31821001 31822000 "*" 0 0 100
"chr19" 31994001 31995000 "*" 0 0 100
"chr19" 32056001 32057000 "*" 9.5812247025151e-14 3.15667542866807e-13 100
"chr19" 32119001 32120000 "*" 2.43005615629954e-09 4.45014222280278e-09 -100
"chr19" 32131001 32132000 "*" 2.383537544004e-07 3.28978743080408e-07
-58.3333333333333
"chr19" 32374001 32375000 "*" 0 0 -100
"chr19" 32438001 32439000 "*" 2.83974510573159e-10 5.95605877261594e-10
70.7317073170732
"chr19" 32483001 32484000 "*" 1.67299207820548e-08 2.71377429441055e-08 -100
"chr19" 32516001 32517000 "*" 0 0 -81.4814814814815
"chr19" 32517001 32518000 "*" 7.94897592193422e-10 1.56245612086885e-09
86.4583333333333
"chr19" 32561001 32562000 "*" 0 0 79.8245614035088
"chr19" 32580001 32581000 "*" 1.20792265079217e-13 3.926781951337e-13
83.0508474576271
"chr19" 32690001 32691000 "*" 1.37828859436695e-10 3.01445544267661e-10 100
"chr19" 32745001 32746000 "*" 3.78583553395373e-09 6.77106049933274e-09
66.9278996865204
"chr19" 32754001 32755000 "*" 6.39710506789015e-13 1.90150053906343e-12
90.2439024390244
"chr19" 32817001 32818000 "*" 2.08941686175024e-09 3.86426039539209e-09
-57.1428571428571
"chr19" 32897001 32898000 "*" 0 0 87.9120879120879
"chr19" 33038001 33039000 "*" 1.35890187991095e-11 3.40841971363279e-11 -100
"chr19" 33336001 33337000 "*" 4.5915737878488e-10 9.35073020522015e-10
91.6666666666667
"chr19" 33371001 33372000 "*" 8.85353236934616e-06 9.77806753952514e-06
-55.8823529411765
"chr19" 33380001 33381000 "*" 4.01313771103418e-09 7.11882087237587e-09 -100
"chr19" 33462001 33463000 "*" 2.08460693151835e-10 4.46464320373107e-10
-81.1320754716981
"chr19" 33468001 33469000 "*" 3.07314299563544e-08 4.81129071584046e-08
-94.7368421052632
"chr19" 33558001 33559000 "*" 1.52794177310511e-09 2.87642564232045e-09 -100
"chr19" 33561001 33562000 "*" 7.0006535457523e-08 1.03695832512054e-07 100
"chr19" 33571001 33572000 "*" 8.59666722519137e-06 9.51075198245685e-06
-56.3636363636364
"chr19" 33632001 33633000 "*" 3.52704532247117e-11 8.35581292290093e-11 100
"chr19" 33682001 33683000 "*" 0.000235091795027231 0.000207505933348564
51.8518518518519
"chr19" 33707001 33708000 "*" 1.48087875295744e-10 3.22075573380618e-10 100
"chr19" 33712001 33713000 "*" 7.90290055618925e-12 2.04914476750626e-11
-71.7948717948718
"chr19" 33723001 33724000 "*" 0 0 -55.5774823431658
"chr19" 33740001 33741000 "*" 0 0 -71.1324570273003
"chr19" 33765001 33766000 "*" 6.45299735868576e-08 9.64436459075686e-08 -75
"chr19" 33839001 33840000 "*" 6.37490060739765e-13 1.89496793587877e-12 -100
"chr19" 33841001 33842000 "*" 4.88498130835069e-15 1.8824139250584e-14 -100
"chr19" 33929001 33930000 "*" 2.22044604925031e-16 9.81641919380259e-16
56.3380281690141
"chr19" 33941001 33942000 "*" 3.63445940010365e-11 8.58565209111204e-11 -100

"chr19" 33961001 33962000 "*" 1.30914612483934e-09 2.49334777909977e-09
-74.1496598639456
"chr19" 33975001 33976000 "*" 1.77391434874608e-11 4.38747606909752e-11
57.1428571428571
"chr19" 34014001 34015000 "*" 2.67283972732457e-11 6.45686178157698e-11 100
"chr19" 34026001 34027000 "*" 1.06837294566731e-09 2.05899591663134e-09
73.3333333333333
"chr19" 34029001 34030000 "*" 4.68626781824355e-09 8.25092364686129e-09
82.6086956521739
"chr19" 34044001 34045000 "*" 0 0 69.4444444444444
"chr19" 34049001 34050000 "*" 3.82027742773516e-13 1.17115897846246e-12
75.2941176470588
"chr19" 34093001 34094000 "*" 2.62900812231237e-13 8.21294938531841e-13 100
"chr19" 34181001 34182000 "*" 1.33226762955019e-15 5.48133041560118e-15
53.3333333333333
"chr19" 34190001 34191000 "*" 0 0 -72.8571428571429
"chr19" 34193001 34194000 "*" 3.15303338993544e-14 1.1025793806681e-13
-53.2608695652174
"chr19" 34194001 34195000 "*" 1.28115100750392e-08 2.11368493104501e-08
-61.5384615384615
"chr19" 34214001 34215000 "*" 1.66533453693773e-15 6.7629186866784e-15
-75.4716981132076
"chr19" 34223001 34224000 "*" 5.99442670129458e-05 5.82299856529221e-05
-56.0606060606061
"chr19" 34257001 34258000 "*" 9.22717435791753e-09 1.55374184879414e-08
-66.6666666666667
"chr19" 34266001 34267000 "*" 2.90229696942035e-08 4.5573322927098e-08
-53.6363636363636
"chr19" 34278001 34279000 "*" 7.67056418382595e-11 1.7359448580318e-10 100
"chr19" 34295001 34296000 "*" 1.03124175865332e-11 2.6346734902568e-11
51.4860456687205
"chr19" 34369001 34370000 "*" 1.17839071833714e-11 2.98103030853977e-11
84.6153846153846
"chr19" 34373001 34374000 "*" 7.93809462606987e-14 2.64679416473686e-13 100
"chr19" 34379001 34380000 "*" 0 0 100
"chr19" 34394001 34395000 "*" 0 0 100
"chr19" 34396001 34397000 "*" 0 0 55.4259259259259
"chr19" 34432001 34433000 "*" 6.18972780808136e-08 9.27382759807238e-08
-73.9130434782609
"chr19" 34441001 34442000 "*" 3.07531777821168e-14 1.07626052665081e-13 -100
"chr19" 34530001 34531000 "*" 6.4324989779152e-11 1.47230020825092e-10 100
"chr19" 34531001 34532000 "*" 0 0 -100
"chr19" 34598001 34599000 "*" 2.17177471806274e-06 2.62953347968408e-06
-57.1428571428571
"chr19" 34625001 34626000 "*" 0 0 -83.6065573770492
"chr19" 34641001 34642000 "*" 1.52466927971773e-12 4.33322475981408e-12 -100
"chr19" 34650001 34651000 "*" 7.0006535457523e-08 1.03695832512054e-07 100
"chr19" 34744001 34745000 "*" 4.49843613470335e-08 6.86525964185201e-08
-54.6666666666667
"chr19" 34749001 34750000 "*" 2.33028818463765e-10 4.93719283454501e-10 100
"chr19" 34855001 34856000 "*" 0 0 100
"chr19" 34857001 34858000 "*" 3.07531777821168e-14 1.07626052665081e-13 100

"chr19" 34970001 34971000 "*" 5.03264097062583e-13 1.51942428521737e-12 -100
"chr19" 34992001 34993000 "*" 0 0 -56.4009661835749
"chr19" 34995001 34996000 "*" 0 0 100
"chr19" 35064001 35065000 "*" 1.37025183205575e-05 1.46995212257477e-05 58
"chr19" 35082001 35083000 "*" 5.99234394071946e-06 6.79020205076211e-06
65.7894736842105
"chr19" 35083001 35084000 "*" 1.72186709335165e-11 4.26735158905008e-11
87.9120879120879
"chr19" 35086001 35087000 "*" 9.28725311467593e-05 8.75598718868157e-05
66.6666666666667
"chr19" 35138001 35139000 "*" 2.91759527648594e-09 5.28506069664913e-09
64.5161290322581
"chr19" 35156001 35157000 "*" 1.12843068222901e-12 3.259512533505e-12 -100
"chr19" 35160001 35161000 "*" 7.105427357601e-15 2.68362283258525e-14 100
"chr19" 35168001 35169000 "*" 0 0 80.1892371377883
"chr19" 35264001 35265000 "*" 0 0 88.8888888888889
"chr19" 35337001 35338000 "*" 5.00287589133563e-11 1.16065352635758e-10
89.7435897435897
"chr19" 35338001 35339000 "*" 2.66891642775047e-06 3.18759115320439e-06
57.1428571428571
"chr19" 35381001 35382000 "*" 4.79888864202138e-08 7.29535840441379e-08
90.9090909090909
"chr19" 35486001 35487000 "*" 4.70123939777523e-12 1.25632555479461e-11
52.1739130434783
"chr19" 35546001 35547000 "*" 2.98824465383429e-06 3.54254647244229e-06
-66.6666666666667
"chr19" 35564001 35565000 "*" 2.19747824600214e-05 2.28520576680913e-05
-52.3076923076923
"chr19" 35572001 35573000 "*" 0 0 -100
"chr19" 35598001 35599000 "*" 0 0 -100
"chr19" 35604001 35605000 "*" 3.34128893442198e-08 5.19772864618523e-08 -100
"chr19" 35607001 35608000 "*" 6.67013488931945e-05 6.43065783242906e-05
52.3809523809524
"chr19" 35692001 35693000 "*" 3.64270735797589e-09 6.5252880340617e-09
66.6666666666667
"chr19" 35713001 35714000 "*" 2.54484454444981e-05 2.62004281959145e-05
-62.0689655172414
"chr19" 35723001 35724000 "*" 0 0 100
"chr19" 35744001 35745000 "*" 1.49613654798486e-12 4.26244554429779e-12 -100
"chr19" 35758001 35759000 "*" 0 0 -56.8553007370923
"chr19" 35773001 35774000 "*" 1.22124532708767e-14 4.48868324225892e-14
91.7197452229299
"chr19" 35821001 35822000 "*" 3.56775498033812e-10 7.35898629809829e-10 100
"chr19" 35836001 35837000 "*" 1.32844938823817e-05 1.42863633262883e-05 -60
"chr19" 35850001 35851000 "*" 0 0 100
"chr19" 35861001 35862000 "*" 0 0 100
"chr19" 35888001 35889000 "*" 5.25841424847684e-08 7.95060575196433e-08
64.8648648648649
"chr19" 35899001 35900000 "*" 4.64366234353975e-09 8.17936336444593e-09
-55.5555555555556
"chr19" 35924001 35925000 "*" 6.24140372718074e-09 1.0801637484149e-08
82.258064516129

"chr19" 35951001 35952000 "*" 1.31190832008965e-06 1.63923591886211e-06
53.8461538461538
"chr19" 35959001 35960000 "*" 1.38590361409285e-10 3.02706178892116e-10 -100
"chr19" 35982001 35983000 "*" 4.32209823486573e-12 1.15935290479641e-11 100
"chr19" 36028001 36029000 "*" 6.93311593691348e-08 1.03197650079882e-07 78
"chr19" 36037001 36038000 "*" 0 0 100
"chr19" 36083001 36084000 "*" 0 0 -100
"chr19" 36102001 36103000 "*" 2.79617107246111e-09 5.07591201160347e-09
-78.4313725490196
"chr19" 36107001 36108000 "*" 2.02327044007689e-12 5.65300384792621e-12 -100
"chr19" 36114001 36115000 "*" 2.69784194983913e-14 9.51020326009845e-14
60.377358490566
"chr19" 36130001 36131000 "*" 2.14925055530557e-09 3.97007739258012e-09
-68.4210526315789
"chr19" 36169001 36170000 "*" 2.84189433807924e-06 3.38038891121791e-06
56.5217391304348
"chr19" 36178001 36179000 "*" 2.57571741713036e-14 9.10510014774126e-14
-66.0377358490566
"chr19" 36193001 36194000 "*" 0 0 86.9649805447471
"chr19" 36194001 36195000 "*" 0 0 76.6355140186916
"chr19" 36202001 36203000 "*" 6.30384633382164e-13 1.87665599796749e-12 100
"chr19" 36217001 36218000 "*" 2.08814465718632e-09 3.86232567960463e-09 -100
"chr19" 36235001 36236000 "*" 0 0 67.7918752182516
"chr19" 36243001 36244000 "*" 6.59742260822327e-11 1.50809824237209e-10
65.9090909090909
"chr19" 36248001 36249000 "*" 1.11022302462516e-16 5.03662826618488e-16
70.2702702702703
"chr19" 36249001 36250000 "*" 5.7529598873618e-07 7.55181675919392e-07
67.741935483871
"chr19" 36282001 36283000 "*" 0 0 56.4516129032258
"chr19" 36334001 36335000 "*" 1.11022302462516e-16 5.03662826618488e-16
79.646017699115
"chr19" 36354001 36355000 "*" 9.5812247025151e-14 3.15667542866807e-13 -100
"chr19" 36355001 36356000 "*" 3.46500605985511e-13 1.06777978272295e-12 -100
"chr19" 36366001 36367000 "*" 2.02060590481778e-14 7.24287363058787e-14
70.3703703703704
"chr19" 36369001 36370000 "*" 2.79620770982092e-11 6.73443061609763e-11 78.125
"chr19" 36376001 36377000 "*" 0 0 100
"chr19" 36393001 36394000 "*" 8.58968451922237e-12 2.213554983903e-11 -100
"chr19" 36445001 36446000 "*" 1.50623957750895e-12 4.28756717183481e-12 100
"chr19" 36449001 36450000 "*" 0 0 82.4772313296903
"chr19" 36477001 36478000 "*" 3.43606965890197e-05 3.46346556590872e-05
55.8823529411765
"chr19" 36486001 36487000 "*" 0 0 65
"chr19" 36490001 36491000 "*" 2.08814465718632e-09 3.86232567960463e-09 -100
"chr19" 36493001 36494000 "*" 2.55351295663786e-15 1.01678090381833e-14 -100
"chr19" 36500001 36501000 "*" 0 0 -73.1636185745465
"chr19" 36502001 36503000 "*" 0 0 -100
"chr19" 36518001 36519000 "*" 1.11022302462516e-16 5.03662826618488e-16 -100
"chr19" 36524001 36525000 "*" 1.50440135904262e-08 2.46078651158753e-08
53.5567715458276
"chr19" 36749001 36750000 "*" 6.03184169278848e-13 1.80250027299235e-12

-54.6468401486989
"chr19" 36819001 36820000 "*" 5.55111512312578e-16 2.36485094870365e-15 100
"chr19" 36908001 36909000 "*" 1.52629020533368e-11 3.80571833600964e-11
55.9931871407281
"chr19" 37019001 37020000 "*" 0 0 83.2923832923833
"chr19" 37052001 37053000 "*" 3.72134329185148e-06 4.34876449114335e-06 -70
"chr19" 37157001 37158000 "*" 0 0 95.0854700854701
"chr19" 37263001 37264000 "*" 0 0 63.7931034482759
"chr19" 37355001 37356000 "*" 7.03992419914812e-12 1.83313429961219e-11 100
"chr19" 37501001 37502000 "*" 0 0 -51.5797889350816
"chr19" 37502001 37503000 "*" 1.46438416948058e-13 4.72057283418642e-13
50.3620803159974
"chr19" 37506001 37507000 "*" 1.85761853455091e-07 2.59935372110621e-07 60
"chr19" 37551001 37552000 "*" 0 0 79.0697674418605
"chr19" 37666001 37667000 "*" 1.35890187991095e-11 3.40841971363279e-11 100
"chr19" 37855001 37856000 "*" 6.64447386000688e-11 1.51847906757688e-10
61.5384615384615
"chr19" 38085001 38086000 "*" 0 0 71.5076071922545
"chr19" 38145001 38146000 "*" 0 0 78.9655172413793
"chr19" 38146001 38147000 "*" 0 0 75.3008895866039
"chr19" 38148001 38149000 "*" 2.13421858630269e-07 2.96327963080103e-07 -60
"chr19" 38303001 38304000 "*" 6.44564592999775e-06 7.26663863268796e-06
66.6666666666667
"chr19" 38411001 38412000 "*" 0 0 -100
"chr19" 38423001 38424000 "*" 6.97508717451001e-12 1.81818137764816e-11
-57.7731092436975
"chr19" 38432001 38433000 "*" 1.98124849859482e-10 4.25157611354621e-10 -100
"chr19" 38552001 38553000 "*" 2.22044604925031e-15 8.90449334476539e-15
-83.3333333333333
"chr19" 38560001 38561000 "*" 1.11022302462516e-16 5.03662826618488e-16
-51.6129032258064
"chr19" 38598001 38599000 "*" 2.26498236277628e-07 3.13481982242673e-07
77.9069767441861
"chr19" 38633001 38634000 "*" 6.99440505513849e-14 2.34592719571672e-13
-96.2962962962963
"chr19" 38680001 38681000 "*" 6.59550958292954e-09 1.13456194382773e-08 -100
"chr19" 38685001 38686000 "*" 3.530509218308e-14 1.22891039008692e-13 -100
"chr19" 38717001 38718000 "*" 9.13071840358270e-11 2.04420777057026e-10
54.1666666666667
"chr19" 38719001 38720000 "*" 0 0 61.5758671192799
"chr19" 38720001 38721000 "*" 0 0 83.0729166666667
"chr19" 38735001 38736000 "*" 1.49161705209622e-09 2.82244082395655e-09 76.8
"chr19" 38741001 38742000 "*" 0 0 59.1374084364739
"chr19" 38758001 38759000 "*" 7.61612994892857e-14 2.54524180939423e-13
-69.7674418604651
"chr19" 38785001 38786000 "*" 9.76996261670138e-15 3.63402770315216e-14 100
"chr19" 38794001 38795000 "*" 4.51860771022439e-14 1.55391321278806e-13
-55.7017543859649
"chr19" 38833001 38834000 "*" 1.61204383175573e-13 5.17337152201509e-13 100
"chr19" 38845001 38846000 "*" 3.6700841921089e-08 5.65530226072258e-08 -100
"chr19" 38900001 38901000 "*" 9.41876687754473e-10 1.83329867152656e-09 -62.5
"chr19" 38908001 38909000 "*" 0 0 63.4407248930279

"chr19" 38916001 38917000 "*" 2.00227675550835e-08 3.20459176617961e-08 100
"chr19" 38917001 38918000 "*" 3.04324343503026e-11 7.26599935673121e-11 100
"chr19" 38927001 38928000 "*" 7.28830468088582e-07 9.43045938490413e-07 64.4444444444444
"chr19" 38942001 38943000 "*" 6.39165064653113e-07 8.33531059852563e-07 90.9090909090909
"chr19" 38969001 38970000 "*" 1.39779633423487e-07 1.98463037958589e-07 100
"chr19" 38973001 38974000 "*" 0 0 56.4543215405284
"chr19" 38974001 38975000 "*" 0 0 81.1594202898551
"chr19" 39014001 39015000 "*" 0 0 95.2380952380952
"chr19" 39065001 39066000 "*" 8.7349629751543e-09 1.47505871898511e-08 100
"chr19" 39105001 39106000 "*" 1.37924116572208e-11 3.45569364948834e-11 94.4444444444444
"chr19" 39115001 39116000 "*" 8.7349629751543e-09 1.47505871898511e-08 -100
"chr19" 39156001 39157000 "*" 4.6558954913678e-08 7.09271611226448e-08 60.5263157894737
"chr19" 39222001 39223000 "*" 1.21821383036735e-06 1.5285073905752e-06 -66.6666666666667
"chr19" 39236001 39237000 "*" 9.20707954321642e-13 2.68760929249829e-12 60
"chr19" 39282001 39283000 "*" 0 0 -88.0952380952381
"chr19" 39414001 39415000 "*" 2.76076186178642e-06 3.29045321100886e-06 69.2307692307692
"chr19" 39455001 39456000 "*" 9.79505365705791e-12 2.5087194111544e-11 -93.75
"chr19" 39480001 39481000 "*" 1.20281562487889e-12 3.46585342181251e-12 100
"chr19" 39506001 39507000 "*" 1.67299207820548e-08 2.71377429441055e-08 -100
"chr19" 39518001 39519000 "*" 8.88178419700125e-16 3.70670032207938e-15 100
"chr19" 39522001 39523000 "*" 0 0 62.7640245287304
"chr19" 39544001 39545000 "*" 2.22044604925031e-16 9.81641919380259e-16 -54.2641446822632
"chr19" 39574001 39575000 "*" 0 0 -62.8953335720253
"chr19" 39575001 39576000 "*" 0 0 -72.3256755389131
"chr19" 39596001 39597000 "*" 0 0 100
"chr19" 39600001 39601000 "*" 0.000451115289779591 0.000379477851858741 -52.1739130434783
"chr19" 39616001 39617000 "*" 0 0 -54.9019607843137
"chr19" 39636001 39637000 "*" 4.47153425398028e-12 1.19731939931881e-11 -52.3809523809524
"chr19" 39643001 39644000 "*" 1.0636353062754e-06 1.34530286878699e-06 -52.1739130434783
"chr19" 39702001 39703000 "*" 2.17381668221606e-13 6.85151076607736e-13 100
"chr19" 39710001 39711000 "*" 7.35820626651673e-10 1.45160263760594e-09 -57.1428571428571
"chr19" 39728001 39729000 "*" 0 0 -100
"chr19" 39811001 39812000 "*" 0 0 53.1197894086788
"chr19" 39818001 39819000 "*" 7.54951656745106e-14 2.5237892556906e-13 -100
"chr19" 39826001 39827000 "*" 1.0727581012393e-07 1.55085410510047e-07 54.4061302681992
"chr19" 39888001 39889000 "*" 3.56775498033812e-10 7.35898629809829e-10 -100
"chr19" 39893001 39894000 "*" 0 0 -52.790346907994
"chr19" 39898001 39899000 "*" 0 0 -70.4228281835029
"chr19" 39900001 39901000 "*" 0 0 -81.6091954022989
"chr19" 39901001 39902000 "*" 0 0 -100

"chr19" 39930001 39931000 "*" 8.65973959207622e-15 3.24130830964561e-14 100
"chr19" 40042001 40043000 "*" 0 0 100
"chr19" 40357001 40358000 "*" 0 0 100
"chr19" 40389001 40390000 "*" 6.24654217240561e-10 1.2462108191303e-09 100
"chr19" 40718001 40719000 "*" 7.06767977476375e-13 2.09135859206644e-12
81.8181818181818
"chr19" 40746001 40747000 "*" 2.34590125103296e-13 7.3721617000728e-13
62.0689655172414
"chr19" 40841001 40842000 "*" 8.52140580320793e-12 2.19817259951489e-11 100
"chr19" 40879001 40880000 "*" 1.49613654798486e-12 4.26244554429779e-12 -100
"chr19" 40891001 40892000 "*" 1.44584344496934e-12 4.12818729953819e-12
90.8496732026144
"chr19" 40898001 40899000 "*" 0 0 -60.4651162790698
"chr19" 40939001 40940000 "*" 6.4152538836737e-10 1.27477697728417e-09 -100
"chr19" 40972001 40973000 "*" 0 0 53.6261631089217
"chr19" 41010001 41011000 "*" 9.89197612710768e-12 2.53214359107573e-11
-54.754922496858
"chr19" 41015001 41016000 "*" 1.19073046100926e-06 1.49613945244789e-06
69.4444444444444
"chr19" 41031001 41032000 "*" 3.05532965594324e-09 5.52316244966729e-09
-73.0769230769231
"chr19" 41038001 41039000 "*" 0 0 64.2857142857143
"chr19" 41067001 41068000 "*" 6.59550958292954e-09 1.13456194382773e-08 100
"chr19" 41082001 41083000 "*" 0 0 100
"chr19" 41102001 41103000 "*" 0 0 72.1896383186706
"chr19" 41107001 41108000 "*" 0 0 -61.0657730240311
"chr19" 41111001 41112000 "*" 0 0 -81.2991329798578
"chr19" 41115001 41116000 "*" 0 0 -89.9598393574297
"chr19" 41121001 41122000 "*" 1.14352971536391e-14 4.21921674335999e-14 -75
"chr19" 41169001 41170000 "*" 0 0 -59.1836734693878
"chr19" 41297001 41298000 "*" 1.17702637125738e-06 1.48016917543092e-06
90.9090909090909
"chr19" 41304001 41305000 "*" 1.11022302462516e-16 5.03662826618488e-16
57.9365079365079
"chr19" 41329001 41330000 "*" 5.81201753391269e-13 1.73949010668347e-12
-66.6666666666667
"chr19" 41381001 41382000 "*" 0 0 50.5679974034404
"chr19" 41382001 41383000 "*" 8.7349629751543e-09 1.47505871898511e-08 100
"chr19" 41414001 41415000 "*" 1.11022302462516e-16 5.03662826618488e-16
-95.7894736842105
"chr19" 41497001 41498000 "*" 1.51656465163796e-13 4.87803420672651e-13 -100
"chr19" 41618001 41619000 "*" 2.64951482975562e-09 4.82186011375752e-09 -100
"chr19" 41673001 41674000 "*" 0 0 75
"chr19" 41700001 41701000 "*" 1.49998246046223e-08 2.45384717139466e-08
73.7179487179487
"chr19" 41743001 41744000 "*" 2.55351295663786e-15 1.01678090381833e-14 100
"chr19" 41764001 41765000 "*" 1.37828859436695e-10 3.01445544267661e-10 -100
"chr19" 41879001 41880000 "*" 0 0 100
"chr19" 41883001 41884000 "*" 1.45599865497559e-10 3.17334716489586e-10
-76.9230769230769
"chr19" 41884001 41885000 "*" 1.34559030584569e-13 4.35581312463895e-13 100
"chr19" 41922001 41923000 "*" 0 0 -64.7154471544715

"chr19" 42021001 42022000 "*" 0 0 55.9567901234568
"chr19" 42052001 42053000 "*" 6.63806998257854e-08 9.90835922040079e-08
-79.1044776119403
"chr19" 42123001 42124000 "*" 1.52794177310511e-09 2.87642564232045e-09 100
"chr19" 42131001 42132000 "*" 5.6300528805675e-07 7.39798856492168e-07 72
"chr19" 42203001 42204000 "*" 0 0 -100
"chr19" 42211001 42212000 "*" 0 0 -100
"chr19" 42238001 42239000 "*" 6.24005129790106e-08 9.34435715465956e-08
80.7692307692308
"chr19" 42262001 42263000 "*" 1.61068774429118e-07 2.27001166795264e-07
-55.6650246305419
"chr19" 42266001 42267000 "*" 1.12458486967171e-09 2.15740815043925e-09 100
"chr19" 42313001 42314000 "*" 0 0 75.5102040816327
"chr19" 42347001 42348000 "*" 6.88338275267597e-15 2.60518659957509e-14 100
"chr19" 42385001 42386000 "*" 9.30637172047444e-06 1.02500975640718e-05
65.3061224489796
"chr19" 42391001 42392000 "*" 2.47746045900499e-10 5.23491079290299e-10 75
"chr19" 42415001 42416000 "*" 3.75177666711579e-12 1.01604360602709e-11
-69.4736842105263
"chr19" 42448001 42449000 "*" 2.64951482975562e-09 4.82186011375752e-09 100
"chr19" 42475001 42476000 "*" 0 0 99.1071428571429
"chr19" 42500001 42501000 "*" 8.88178419700125e-16 3.70670032207938e-15
89.2857142857143
"chr19" 42502001 42503000 "*" 0 0 55.8400471735616
"chr19" 42506001 42507000 "*" 4.08209022140227e-11 9.57770690255255e-11 100
"chr19" 42527001 42528000 "*" 2.78418621491028e-10 5.84496868167592e-10 -65.625
"chr19" 42556001 42557000 "*" 2.64951482975562e-09 4.82186011375752e-09 100
"chr19" 42560001 42561000 "*" 3.31491103455761e-07 4.48888447535355e-07
55.5555555555556
"chr19" 42567001 42568000 "*" 1.93303231454944e-06 2.35716937594482e-06
64.5161290322581
"chr19" 42581001 42582000 "*" 4.73234496034536e-09 8.30627300264826e-09 -100
"chr19" 42633001 42634000 "*" 1.07882591748876e-11 2.74553537455803e-11 100
"chr19" 42635001 42636000 "*" 0 0 100
"chr19" 42637001 42638000 "*" 0 0 -76.3975155279503
"chr19" 42642001 42643000 "*" 4.73234496034536e-09 8.30627300264826e-09 100
"chr19" 42664001 42665000 "*" 4.90996751774109e-05 4.83476248645988e-05
63.6363636363636
"chr19" 42699001 42700000 "*" 5.09592368302947e-14 1.73761636562837e-13 100
"chr19" 42725001 42726000 "*" 7.34939306079596e-07 9.50242626154403e-07
-71.7391304347826
"chr19" 42749001 42750000 "*" 0 0 -100
"chr19" 42758001 42759000 "*" 0 0 -66.6666666666667
"chr19" 42774001 42775000 "*" 0 0 -55.9322033898305
"chr19" 42779001 42780000 "*" 1.36593441736332e-05 1.46569770945072e-05
-69.6969696969697
"chr19" 42782001 42783000 "*" 6.06239325406932e-09 1.05089753636051e-08 -63.75
"chr19" 42824001 42825000 "*" 2.3990809339125e-12 6.65441738642456e-12 100
"chr19" 42830001 42831000 "*" 0 0 68.9814814814815
"chr19" 42863001 42864000 "*" 6.29919238992471e-09 1.08977148121874e-08
-66.8831168831169
"chr19" 42883001 42884000 "*" 3.95353005888666e-09 7.03928529283733e-09 -100

"chr19" 42901001 42902000 "*" 6.90114632106997e-13 2.04534617294632e-12
-70.1298701298701
"chr19" 42927001 42928000 "*" 0 0 -53.6927521271944
"chr19" 43070001 43071000 "*" 1.67299207820548e-08 2.71377429441055e-08 100
"chr19" 43100001 43101000 "*" 8.88178419700125e-16 3.70670032207938e-15
-56.6037735849057
"chr19" 43135001 43136000 "*" 8.5318418996394e-12 2.20066048390298e-11
73.8095238095238
"chr19" 43384001 43385000 "*" 9.63829016598083e-12 2.46993429847588e-11 100
"chr19" 43441001 43442000 "*" 1.07882591748876e-11 2.74553537455803e-11 100
"chr19" 43491001 43492000 "*" 4.01313771103418e-09 7.11882087237587e-09 100
"chr19" 43496001 43497000 "*" 5.04263297784746e-13 1.52078941532818e-12
63.7362637362637
"chr19" 43657001 43658000 "*" 3.82652398656091e-09 6.84089874708887e-09
-68.4210526315789
"chr19" 43711001 43712000 "*" 7.93714094449172e-10 1.56020337210645e-09
-90.2777777777778
"chr19" 43864001 43865000 "*" 3.40125705378114e-10 7.03784073980162e-10 -100
"chr19" 43991001 43992000 "*" 0 0 100
"chr19" 44037001 44038000 "*" 0 0 -81.25
"chr19" 44059001 44060000 "*" 8.7349629751543e-09 1.47505871898511e-08 100
"chr19" 44115001 44116000 "*" 1.43019312889292e-05 1.52998435464261e-05
53.3333333333333
"chr19" 44123001 44124000 "*" 0 0 58.0381941119007
"chr19" 44124001 44125000 "*" 6.21911411258225e-11 1.42709712896805e-10
56.4885496183206
"chr19" 44135001 44136000 "*" 3.6700841921089e-08 5.65530226072258e-08 100
"chr19" 44140001 44141000 "*" 1.43743161817866e-09 2.72522843378903e-09
-64.2857142857143
"chr19" 44144001 44145000 "*" 0 0 51.7837837837838
"chr19" 44152001 44153000 "*" 4.04946233650838e-05 4.0376350196346e-05
64.7058823529412
"chr19" 44216001 44217000 "*" 1.07882591748876e-11 2.74553537455803e-11 100
"chr19" 44237001 44238000 "*" 3.56131733214582e-07 4.80158368901456e-07
-62.962962962963
"chr19" 44266001 44267000 "*" 0 0 100
"chr19" 44413001 44414000 "*" 2.02060590481778e-13 6.40127405465654e-13 100
"chr19" 44529001 44530000 "*" 0 0 84.5528455284553
"chr19" 44576001 44577000 "*" 0 0 -64.3617021276596
"chr19" 44645001 44646000 "*" 0 0 100
"chr19" 44646001 44647000 "*" 1.04037820496394e-05 1.1367667257137e-05
-66.6666666666667
"chr19" 44708001 44709000 "*" 1.15552012402986e-12 3.33522006252296e-12
-90.2654867256637
"chr19" 44809001 44810000 "*" 1.98124849859482e-10 4.25157611354621e-10 100
"chr19" 44813001 44814000 "*" 1.67299207820548e-08 2.71377429441055e-08 100
"chr19" 44832001 44833000 "*" 1.99840144432528e-12 5.59476580335607e-12 100
"chr19" 44921001 44922000 "*" 9.63829016598083e-12 2.46993429847588e-11 -100
"chr19" 45017001 45018000 "*" 2.38031816479634e-13 7.47299807843568e-13 100
"chr19" 45055001 45056000 "*" 7.03992419914812e-12 1.83313429961219e-11 -100
"chr19" 45066001 45067000 "*" 0 0 72.9032258064516
"chr19" 45099001 45100000 "*" 8.44866073321793e-05 8.01219179790309e-05

62.962962962963
"chr19" 45192001 45193000 "*" 0 0 -100
"chr19" 45193001 45194000 "*" 3.40125705378114e-10 7.03784073980162e-10 100
"chr19" 45229001 45230000 "*" 8.69637695188885e-13 2.54691118762067e-12
55.4682159945318
"chr19" 45245001 45246000 "*" 1.37828859436695e-10 3.01445544267661e-10 -100
"chr19" 45249001 45250000 "*" 7.55356609793978e-06 8.42908128629145e-06
60.7594936708861
"chr19" 45279001 45280000 "*" 3.33066907387547e-16 1.4495649018245e-15
-95.4545454545455
"chr19" 45287001 45288000 "*" 3.33066907387547e-16 1.4495649018245e-15 -100
"chr19" 45300001 45301000 "*" 9.04570973681018e-10 1.76414389822173e-09 -100
"chr19" 45311001 45312000 "*" 6.76028137913676e-08 1.00779726290333e-07
71.0526315789474
"chr19" 45354001 45355000 "*" 1.86117573131028e-07 2.60389440338124e-07
66.6666666666667
"chr19" 45380001 45381000 "*" 2.81441575045172e-07 3.84774904759053e-07
-54.5454545454545
"chr19" 45409001 45410000 "*" 0 0 66.4429530201342
"chr19" 45414001 45415000 "*" 2.38575468822777e-05 2.46816531377115e-05
66.6666666666667
"chr19" 45430001 45431000 "*" 5.28933352195171e-06 6.04525163399597e-06
-53.9325842696629
"chr19" 45441001 45442000 "*" 3.40125705378114e-10 7.03784073980162e-10 100
"chr19" 45457001 45458000 "*" 1.96509475358653e-13 6.23868565133654e-13 -100
"chr19" 45483001 45484000 "*" 0 0 53.7037037037037
"chr19" 45502001 45503000 "*" 0 0 62.0689655172414
"chr19" 45594001 45595000 "*" 0 0 -60.6707317073171
"chr19" 45602001 45603000 "*" 5.22862601770591e-07 6.89451341049503e-07
-74.5454545454545
"chr19" 45624001 45625000 "*" 0 0 98.7421383647799
"chr19" 45634001 45635000 "*" 5.44009282066327e-15 2.08486137681812e-14
-93.3884297520661
"chr19" 45654001 45655000 "*" 8.52140580320793e-12 2.19817259951489e-11 100
"chr19" 45658001 45659000 "*" 0 0 100
"chr19" 45686001 45687000 "*" 7.9984455014781e-06 8.89173797399106e-06
-66.6666666666667
"chr19" 45728001 45729000 "*" 0 0 68.1481481481482
"chr19" 45730001 45731000 "*" 2.44575202312802e-08 3.87204709479012e-08 76
"chr19" 45750001 45751000 "*" 4.24312552729234e-05 4.21882959593721e-05
-55.8823529411765
"chr19" 45752001 45753000 "*" 2.08814465718632e-09 3.86232567960463e-09 100
"chr19" 45811001 45812000 "*" 0 0 -72.9757085020243
"chr19" 45847001 45848000 "*" 2.44249065417534e-15 9.75180348030994e-15 -100
"chr19" 45850001 45851000 "*" 0 0 -52.4324324324324
"chr19" 45902001 45903000 "*" 8.00170272263756e-08 1.1755907199822e-07
51.8518518518519
"chr19" 45905001 45906000 "*" 1.11022302462516e-16 5.03662826618488e-16
-55.518018018018
"chr19" 45908001 45909000 "*" 0 0 61.6
"chr19" 45923001 45924000 "*" 2.70339306496226e-13 8.42355472617862e-13 -100
"chr19" 45942001 45943000 "*" 1.39779633423487e-07 1.98463037958589e-07 -100

"chr19" 45943001 45944000 "*" 1.2171708085873e-10 2.6844610278289e-10
-89.1089108910891
"chr19" 45966001 45967000 "*" 9.47973900710108e-05 8.92323920772727e-05
61.2903225806452
"chr19" 45969001 45970000 "*" 3.31767792238757e-07 4.49226630918757e-07
-52.5773195876289
"chr19" 46008001 46009000 "*" 1.13140401492018e-08 1.87801380787728e-08 -100
"chr19" 46012001 46013000 "*" 1.02140518265514e-14 3.79278290836596e-14
-69.0909090909091
"chr19" 46017001 46018000 "*" 6.66133814775094e-16 2.81595744474255e-15 -100
"chr19" 46029001 46030000 "*" 6.92287338566189e-11 1.57605630124247e-10 100
"chr19" 46030001 46031000 "*" 7.0006535457523e-08 1.03695832512054e-07 -100
"chr19" 46080001 46081000 "*" 5.62883073484954e-13 1.68905058935478e-12 -100
"chr19" 46093001 46094000 "*" 7.0006535457523e-08 1.03695832512054e-07 -100
"chr19" 46099001 46100000 "*" 8.57092175010621e-13 2.51149198099779e-12 100
"chr19" 46110001 46111000 "*" 0 0 83.3333333333333
"chr19" 46113001 46114000 "*" 1.98325800226939e-11 4.85612402473442e-11 100
"chr19" 46143001 46144000 "*" 1.4432899320127e-15 5.90750815956055e-15 -87.5
"chr19" 46149001 46150000 "*" 2.08253949253168e-05 2.17364676983994e-05
-64.8148148148148
"chr19" 46156001 46157000 "*" 5.66652058608952e-10 1.13800752810875e-09
-77.6119402985075
"chr19" 46181001 46182000 "*" 0 0 67.6989129441856
"chr19" 46234001 46235000 "*" 0 0 -59.8958333333333
"chr19" 46303001 46304000 "*" 0 0 -68.75
"chr19" 46305001 46306000 "*" 4.0365252444996e-06 4.6892927905593e-06
-54.5454545454545
"chr19" 46320001 46321000 "*" 0 0 67.1764705882353
"chr19" 46342001 46343000 "*" 8.58968451922237e-12 2.213554983903e-11 -100
"chr19" 46378001 46379000 "*" 5.58071588940834e-10 1.12161263408564e-09 80
"chr19" 46385001 46386000 "*" 7.86685565423717e-06 8.75794273207872e-06
62.0689655172414
"chr19" 46390001 46391000 "*" 1.4432899320127e-15 5.90750815956055e-15 -100
"chr19" 46409001 46410000 "*" 1.49613654798486e-12 4.26244554429779e-12 -100
"chr19" 46431001 46432000 "*" 0 0 -100
"chr19" 46436001 46437000 "*" 6.38007938813523e-07 8.32178207554581e-07
61.5384615384615
"chr19" 46437001 46438000 "*" 3.25124442309699e-05 3.2934466660002e-05
61.5384615384615
"chr19" 46457001 46458000 "*" 4.41613184445355e-05 4.37993190316921e-05
-52.2774327122153
"chr19" 46459001 46460000 "*" 1.87510396266743e-05 1.97080033495427e-05
66.6666666666667
"chr19" 46462001 46463000 "*" 6.24654217240561e-10 1.2462108191303e-09 -100
"chr19" 46471001 46472000 "*" 1.43064449176222e-11 3.58007524390384e-11 -60
"chr19" 46475001 46476000 "*" 5.86988235795616e-11 1.35097353473983e-10
-60.5801605801606
"chr19" 46690001 46691000 "*" 4.32209823486573e-12 1.15935290479641e-11 -100
"chr19" 46692001 46693000 "*" 1.4432899320127e-15 5.90750815956055e-15 -100
"chr19" 46706001 46707000 "*" 9.97010987802138e-06 1.09290395054275e-05
61.1111111111111
"chr19" 46727001 46728000 "*" 0 0 -100

"chr19" 46773001 46774000 "*" 6.48213926979224e-10 1.28745451536922e-09 -62.5
"chr19" 46781001 46782000 "*" 0 0 -100
"chr19" 46903001 46904000 "*" 1.71853075436879e-07 2.41422393347956e-07
-61.5384615384615
"chr19" 46920001 46921000 "*" 1.45465417489277e-10 3.17058258509136e-10
54.5454545454545
"chr19" 46959001 46960000 "*" 0 0 100
"chr19" 46972001 46973000 "*" 5.33431965266118e-10 1.07644989448352e-09
64.8648648648649
"chr19" 46991001 46992000 "*" 0 0 100
"chr19" 46995001 46996000 "*" 0 0 83.3333333333333
"chr19" 47016001 47017000 "*" 3.16660031529636e-10 6.58836758441552e-10 -60
"chr19" 47037001 47038000 "*" 5.55111512312578e-15 2.12516350804551e-14 100
"chr19" 47046001 47047000 "*" 5.81211284256256e-06 6.59969314258037e-06
-69.6428571428571
"chr19" 47086001 47087000 "*" 2.88779000712225e-11 6.92935809466068e-11 -100
"chr19" 47106001 47107000 "*" 2.88710388929303e-10 6.03399264731139e-10 100
"chr19" 47107001 47108000 "*" 1.76052916600611e-06 2.15976346611539e-06
-76.3157894736842
"chr19" 47115001 47116000 "*" 0 0 100
"chr19" 47138001 47139000 "*" 0 0 91.9642857142857
"chr19" 47142001 47143000 "*" 0 0 67.7139761646804
"chr19" 47170001 47171000 "*" 6.59550958292954e-09 1.13456194382773e-08 -100
"chr19" 47217001 47218000 "*" 0 0 -68.2126636387924
"chr19" 47219001 47220000 "*" 0 0 92.4914675767918
"chr19" 47250001 47251000 "*" 5.03264097062583e-13 1.51942428521737e-12 100
"chr19" 47365001 47366000 "*" 3.99680288865056e-14 1.37975970265307e-13 -100
"chr19" 47366001 47367000 "*" 2.80939082775156e-07 3.840942150991e-07
69.5652173913043
"chr19" 47524001 47525000 "*" 0 0 54.9907488557795
"chr19" 47526001 47527000 "*" 9.08108033215171e-11 2.03407753381119e-10 100
"chr19" 47531001 47532000 "*" 3.55123785933387e-08 5.50471549649875e-08
-76.1904761904762
"chr19" 47535001 47536000 "*" 3.55123785933387e-08 5.50471549649875e-08
76.1904761904762
"chr19" 47579001 47580000 "*" 1.48487888651516e-11 3.70689457767456e-11 -100
"chr19" 47599001 47600000 "*" 2.86016765826957e-11 6.88228436670133e-11
77.2727272727273
"chr19" 47691001 47692000 "*" 7.80438248471071e-08 1.14830008757999e-07
64.5833333333333
"chr19" 47730001 47731000 "*" 3.53716922418812e-09 6.34823280819993e-09
-59.2105263157895
"chr19" 47740001 47741000 "*" 9.1506337806968e-05 8.63421053552552e-05 70
"chr19" 47744001 47745000 "*" 4.44089209850063e-16 1.91071758245033e-15
-73.5294117647059
"chr19" 47761001 47762000 "*" 8.65973959207622e-15 3.24130830964561e-14 -100
"chr19" 47806001 47807000 "*" 2.55351295663786e-14 9.03120140387382e-14 87.5
"chr19" 47834001 47835000 "*" 8.92637417682085e-06 9.85381833193747e-06
-64.2857142857143
"chr19" 47843001 47844000 "*" 1.98365768255826e-11 4.85612402473442e-11 100
"chr19" 47853001 47854000 "*" 2.82773804372027e-13 8.79129664109438e-13 100
"chr19" 47862001 47863000 "*" 3.61568774920329e-08 5.5992067422079e-08

-66.6666666666667
"chr19" 47895001 47896000 "*" 9.10727911396236e-06 1.00428848699875e-05
-62.7906976744186
"chr19" 47901001 47902000 "*" 0 0 78.8888888888889
"chr19" 47907001 47908000 "*" 1.11022302462516e-16 5.03662826618488e-16
54.8780487804878
"chr19" 47925001 47926000 "*" 1.1010618527818e-05 1.19878496551981e-05
-59.7222222222222
"chr19" 47927001 47928000 "*" 4.32209823486573e-12 1.15935290479641e-11 100
"chr19" 47948001 47949000 "*" 3.75255382323303e-14 1.30174529687187e-13 62.5
"chr19" 47972001 47973000 "*" 7.67056418382595e-11 1.7359448580318e-10 100
"chr19" 47977001 47978000 "*" 0.000150576069463337 0.000137139618444501
53.3333333333333
"chr19" 47992001 47993000 "*" 1.14106069037945e-09 2.1877573475797e-09 56
"chr19" 48017001 48018000 "*" 1.24344978758018e-14 4.56627092294518e-14
66.6666666666667
"chr19" 48024001 48025000 "*" 0 0 -100
"chr19" 48032001 48033000 "*" 0 0 50.618856934838
"chr19" 48058001 48059000 "*" 0 0 -100
"chr19" 48103001 48104000 "*" 0 0 -100
"chr19" 48137001 48138000 "*" 1.84829929139596e-12 5.19844363507975e-12 -66
"chr19" 48163001 48164000 "*" 1.56863411149288e-12 4.4469621057462e-12 100
"chr19" 48217001 48218000 "*" 0 0 -100
"chr19" 48233001 48234000 "*" 0 0 51.3877922866687
"chr19" 48290001 48291000 "*" 2.38031816479634e-13 7.47299807843568e-13 100
"chr19" 48325001 48326000 "*" 1.77635683940025e-15 7.19870740878856e-15 100
"chr19" 48344001 48345000 "*" 4.65637306490407e-10 9.46720524046618e-10 -100
"chr19" 48468001 48469000 "*" 2.25689266608597e-07 3.12408998070319e-07
55.3191489361702
"chr19" 48491001 48492000 "*" 8.42703684611479e-12 2.17859001990012e-11
83.3333333333333
"chr19" 48587001 48588000 "*" 0 0 100
"chr19" 48607001 48608000 "*" 0 0 -100
"chr19" 48639001 48640000 "*" 0 0 -65.8536585365854
"chr19" 48794001 48795000 "*" 0 0 79.0816326530612
"chr19" 48805001 48806000 "*" 0 0 -51.1363636363636
"chr19" 48858001 48859000 "*" 1.55431223447522e-15 6.33705141101682e-15 -68.75
"chr19" 48902001 48903000 "*" 0 0 -95.3703703703704
"chr19" 48924001 48925000 "*" 3.90465437760668e-13 1.19307893943277e-12 -100
"chr19" 48936001 48937000 "*" 3.68971520003925e-12 1.00056842563147e-11 -56.25
"chr19" 48989001 48990000 "*" 1.11130698088324e-07 1.60244431530545e-07
58.4415584415584
"chr19" 48994001 48995000 "*" 0 0 -51.4240145819093
"chr19" 48996001 48997000 "*" 0 0 -80
"chr19" 49045001 49046000 "*" 5.1281201507436e-13 1.54461625452838e-12 -100
"chr19" 49078001 49079000 "*" 3.77475828372553e-15 1.47379557022071e-14 -100
"chr19" 49105001 49106000 "*" 0 0 100
"chr19" 49127001 49128000 "*" 1.11022302462516e-15 4.59817122935606e-15
81.404174573055
"chr19" 49128001 49129000 "*" 2.4743138316019e-07 3.40830935284511e-07
-70.1612903225806
"chr19" 49170001 49171000 "*" 3.95353005888666e-09 7.03928529283733e-09 -100

"chr19" 49172001 49173000 "*" 5.0875319446142e-07 6.71813087869187e-07
-77.7777777777778
"chr19" 49228001 49229000 "*" 1.98365768255826e-11 4.85612402473442e-11 100
"chr19" 49255001 49256000 "*" 0 0 -72.1591210657608
"chr19" 49256001 49257000 "*" 0 0 -54.1921808761187
"chr19" 49277001 49278000 "*" 3.04324343503026e-11 7.26599935673121e-11 -100
"chr19" 49297001 49298000 "*" 7.78377362564697e-13 2.29551678017433e-12 100
"chr19" 49313001 49314000 "*" 2.64951482975562e-09 4.82186011375752e-09 -100
"chr19" 49315001 49316000 "*" 0 0 58.498789346247
"chr19" 49322001 49323000 "*" 0 0 -86.2068965517241
"chr19" 49371001 49372000 "*" 0 0 -77.7777777777778
"chr19" 49378001 49379000 "*" 0 0 -56.3386727688787
"chr19" 49408001 49409000 "*" 6.04130079295828e-11 1.38849255306602e-10
-62.4675324675325
"chr19" 49447001 49448000 "*" 4.27032853522746e-11 9.99995440481693e-11
70.5882352941177
"chr19" 49534001 49535000 "*" 0 0 100
"chr19" 49542001 49543000 "*" 2.85689027990088e-10 5.98900664864482e-10
71.6417910447761
"chr19" 49552001 49553000 "*" 3.91359648821421e-06 4.55568593885524e-06
-53.9583333333333
"chr19" 49553001 49554000 "*" 0 0 -52.2691705790297
"chr19" 49569001 49570000 "*" 1.13140401492018e-08 1.87801380787728e-08 -100
"chr19" 49589001 49590000 "*" 0 0 -54.1990291262136
"chr19" 49609001 49610000 "*" 2.72163402925685e-11 6.56902746242013e-11
-55.5555555555556
"chr19" 49639001 49640000 "*" 0 0 92.3076923076923
"chr19" 49649001 49650000 "*" 0 0 -100
"chr19" 49729001 49730000 "*" 0 0 -100
"chr19" 49783001 49784000 "*" 8.60915992743072e-05 8.15336677459667e-05 -60
"chr19" 49814001 49815000 "*" 6.08291195192123e-13 1.8172407364528e-12
63.1578947368421
"chr19" 49846001 49847000 "*" 4.72585619581967e-05 4.66476159461294e-05
-62.0689655172414
"chr19" 49911001 49912000 "*" 2.05391259555654e-14 7.35435952190685e-14 -56.25
"chr19" 49915001 49916000 "*" 1.97138194657498e-10 4.23519817464338e-10
71.6216216216216
"chr19" 49924001 49925000 "*" 0 0 60.669588080631
"chr19" 49930001 49931000 "*" 7.06177855858137e-07 9.15126718716161e-07
52.3809523809524
"chr19" 49942001 49943000 "*" 1.12458486967171e-09 2.15740815043925e-09 100
"chr19" 49943001 49944000 "*" 1.13209705067119e-05 1.23037416520042e-05 60
"chr19" 50009001 50010000 "*" 7.86622250670277e-08 1.15684789204325e-07
92.8571428571429
"chr19" 50016001 50017000 "*" 0 0 81.25
"chr19" 50029001 50030000 "*" 9.45910016980633e-13 2.7575082906359e-12
51.5151515151515
"chr19" 50095001 50096000 "*" 1.57423962754422e-10 3.4138350486103e-10
-58.2417582417582
"chr19" 50096001 50097000 "*" 0 0 -66.2783171521036
"chr19" 50268001 50269000 "*" 3.88022947106492e-13 1.1867462770515e-12 100
"chr19" 50433001 50434000 "*" 0 0 63.9344262295082

"chr19" 50456001 50457000 "*" 3.33066907387547e-16 1.4495649018245e-15 100
"chr19" 50475001 50476000 "*" 2.23998597448372e-11 5.45560819654491e-11
88.8888888888889
"chr19" 50486001 50487000 "*" 7.09814422484856e-05 6.81223663184311e-05
-52.112676056338
"chr19" 50490001 50491000 "*" 0 0 -94.6428571428571
"chr19" 50526001 50527000 "*" 0 0 100
"chr19" 50543001 50544000 "*" 2.67283972732457e-11 6.45686178157698e-11 100
"chr19" 50550001 50551000 "*" 5.44009282066327e-15 2.08486137681812e-14 -100
"chr19" 50554001 50555000 "*" 0 0 54.4917344722212
"chr19" 50563001 50564000 "*" 1.11022302462516e-16 5.03662826618488e-16
68.1159420289855
"chr19" 50640001 50641000 "*" 1.34504629656362e-11 3.37732345596615e-11 92.5
"chr19" 50706001 50707000 "*" 0 0 -54.8313321898228
"chr19" 50767001 50768000 "*" 1.03173281096325e-06 1.30703337972536e-06
75.4385964912281
"chr19" 50770001 50771000 "*" 0 0 -62.5483870967742
"chr19" 50801001 50802000 "*" 1.34559030584569e-13 4.35581312463895e-13 100
"chr19" 50837001 50838000 "*" 0 0 52.3809523809524
"chr19" 50929001 50930000 "*" 3.52704532247117e-11 8.35581292290093e-11 100
"chr19" 50965001 50966000 "*" 3.64660357465851e-09 6.53198624782448e-09
50.8324382384533
"chr19" 50972001 50973000 "*" 1.95676808090184e-12 5.48631149584043e-12
70.7317073170732
"chr19" 51000001 51001000 "*" 2.81359380238655e-11 6.77451596826195e-11
-64.2857142857143
"chr19" 51039001 51040000 "*" 4.38316050122012e-12 1.17505188277661e-11
79.7297297297297
"chr19" 51085001 51086000 "*" 0 0 100
"chr19" 51141001 51142000 "*" 0 0 100
"chr19" 51152001 51153000 "*" 2.60109279670573e-08 4.10484695973576e-08
73.6842105263158
"chr19" 51232001 51233000 "*" 2.29774586024689e-06 2.77211598900355e-06
67.6470588235294
"chr19" 51239001 51240000 "*" 3.6700841921089e-08 5.65530226072258e-08 -100
"chr19" 51240001 51241000 "*" 6.51518050620581e-09 1.12485114706444e-08
-80.3571428571429
"chr19" 51259001 51260000 "*" 1.52794177310511e-09 2.87642564232045e-09 100
"chr19" 51269001 51270000 "*" 0 0 100
"chr19" 51356001 51357000 "*" 4.65637306490407e-10 9.46720524046618e-10 100
"chr19" 51362001 51363000 "*" 1.03886988452873e-05 1.13522277663458e-05
70.5882352941177
"chr19" 51363001 51364000 "*" 1.13140401492018e-08 1.87801380787728e-08 100
"chr19" 51374001 51375000 "*" 9.41163845049608e-05 8.8633379222919e-05
-51.8518518518519
"chr19" 51377001 51378000 "*" 1.14124265593318e-11 2.89138062669327e-11 -100
"chr19" 51379001 51380000 "*" 3.90831811358794e-12 1.05610567215048e-11 -100
"chr19" 51381001 51382000 "*" 5.9810565322671e-07 7.83169710025017e-07
67.741935483871
"chr19" 51388001 51389000 "*" 5.08482145278322e-14 1.73432706170696e-13 100
"chr19" 51410001 51411000 "*" 3.6700841921089e-08 5.65530226072258e-08 -100
"chr19" 51416001 51417000 "*" 9.51572154406222e-13 2.77304521102886e-12 -70

"chr19" 51425001 51426000 "*" 1.78590020549763e-09 3.33980585817043e-09 67.1232876712329
"chr19" 51426001 51427000 "*" 2.22044604925031e-16 9.81641919380259e-16 -100
"chr19" 51432001 51433000 "*" 5.18932674609118e-11 1.20190360028741e-10 61.5384615384615
"chr19" 51446001 51447000 "*" 8.7349629751543e-09 1.47505871898511e-08 100
"chr19" 51447001 51448000 "*" 3.63445940010365e-11 8.58565209111204e-11 -100
"chr19" 51456001 51457000 "*" 0 0 93.5945945945946
"chr19" 51461001 51462000 "*" 5.48638023900594e-10 1.10374357336421e-09 -100
"chr19" 51468001 51469000 "*" 1.49613654798486e-12 4.26244554429779e-12 100
"chr19" 51526001 51527000 "*" 1.6789981627241e-09 3.14899741346654e-09 86.25
"chr19" 51538001 51539000 "*" 9.0616525394438e-10 1.76692260704862e-09 51.5151515151515
"chr19" 51569001 51570000 "*" 1.54522650230859e-09 2.90752114584838e-09 54.9019607843137
"chr19" 51586001 51587000 "*" 0 0 88.4210526315789
"chr19" 51588001 51589000 "*" 0 0 100
"chr19" 51621001 51622000 "*" 5.33495114529714e-06 6.09237962218305e-06 -66.6666666666667
"chr19" 51650001 51651000 "*" 1.48533999100309e-07 2.10202108083997e-07 73.9130434782609
"chr19" 51687001 51688000 "*" 8.28387716665935e-05 7.8680886628866e-05 56.5656565656566
"chr19" 51689001 51690000 "*" 0 0 100
"chr19" 51694001 51695000 "*" 1.91605042942911e-09 3.57254507586573e-09 87.5
"chr19" 51728001 51729000 "*" 4.99900121297969e-12 1.32921056339624e-11 100
"chr19" 51795001 51796000 "*" 8.88178419700125e-16 3.70670032207938e-15 -97.5609756097561
"chr19" 51866001 51867000 "*" 2.22044604925031e-16 9.81641919380259e-16 -100
"chr19" 51873001 51874000 "*" 8.7349629751543e-09 1.47505871898511e-08 100
"chr19" 51918001 51919000 "*" 1.66989437833109e-08 2.71377429441055e-08 69.7674418604651
"chr19" 51924001 51925000 "*" 3.40852091440524e-07 4.60928462394503e-07 -71.3513513513514
"chr19" 51930001 51931000 "*" 1.11022302462516e-16 5.03662826618488e-16 100
"chr19" 51983001 51984000 "*" 2.66385726545026e-05 2.73460547451894e-05 -58.1632653061224
"chr19" 52002001 52003000 "*" 0 0 -100
"chr19" 52046001 52047000 "*" 0 0 -100
"chr19" 52068001 52069000 "*" 1.37828859436695e-10 3.01445544267661e-10 -100
"chr19" 52131001 52132000 "*" 0 0 -99.4252873563218
"chr19" 52201001 52202000 "*" 4.45158532258461e-09 7.86029510138311e-09 61.9565217391304
"chr19" 52213001 52214000 "*" 2.62061988554052e-07 3.59760235362752e-07 71.4285714285714
"chr19" 52350001 52351000 "*" 0 0 -62.0915032679739
"chr19" 52383001 52384000 "*" 1.67299207820548e-08 2.71377429441055e-08 -100
"chr19" 52430001 52431000 "*" 0 0 -62.4365482233503
"chr19" 52490001 52491000 "*" 0 0 100
"chr19" 52511001 52512000 "*" 0 0 79.7402597402597
"chr19" 52512001 52513000 "*" 0 0 -56.4102564102564
"chr19" 52531001 52532000 "*" 0 0 70.8933717579251

"chr19" 52597001 52598000 "*" 4.44089209850063e-16 1.91071758245033e-15
-95.4545454545455
"chr19" 52598001 52599000 "*" 0 0 54.7312340966921
"chr19" 52599001 52600000 "*" 2.22044604925031e-16 9.81641919380259e-16
81.6901408450704
"chr19" 52674001 52675000 "*" 0 0 74.5070422535211
"chr19" 52692001 52693000 "*" 0.000221844876828414 0.000196668874836782
-52.9411764705882
"chr19" 52694001 52695000 "*" 0 0 -100
"chr19" 52800001 52801000 "*" 0 0 97.3244147157191
"chr19" 52868001 52869000 "*" 0 0 -100
"chr19" 52910001 52911000 "*" 4.86753970463383e-11 1.13145908161915e-10 100
"chr19" 52917001 52918000 "*" 2.02060590481778e-13 6.40127405465654e-13 -100
"chr19" 52929001 52930000 "*" 1.46658822863799e-08 2.40261015608166e-08 -55
"chr19" 53115001 53116000 "*" 6.79707058304224e-07 8.83197669203001e-07
-62.8571428571429
"chr19" 53144001 53145000 "*" 1.77424741565346e-12 5.00299121148805e-12
-80.9523809523809
"chr19" 53149001 53150000 "*" 7.26085858104852e-14 2.43197054722028e-13
97.9166666666667
"chr19" 53187001 53188000 "*" 1.92068583260152e-14 6.89775382589628e-14 -100
"chr19" 53237001 53238000 "*" 0 0 100
"chr19" 53242001 53243000 "*" 6.21724893790088e-15 2.36606385879553e-14
-77.2727272727273
"chr19" 53244001 53245000 "*" 4.99020824662466e-11 1.15790792586157e-10 -100
"chr19" 53290001 53291000 "*" 2.60913513017158e-11 6.31349963963769e-11 87.5
"chr19" 53401001 53402000 "*" 0 0 59.5238095238095
"chr19" 53447001 53448000 "*" 0 0 -100
"chr19" 53495001 53496000 "*" 5.12782262207523e-08 7.7634284063037e-08
52.3809523809524
"chr19" 53501001 53502000 "*" 5.21804821573824e-14 1.7777322631293e-13
53.6919831223629
"chr19" 53548001 53549000 "*" 9.35208294028378e-07 1.19203178160239e-06
-61.2903225806452
"chr19" 53574001 53575000 "*" 2.43005615629954e-09 4.45014222280278e-09 100
"chr19" 53636001 53637000 "*" 1.69437797126193e-11 4.2020965159043e-11
53.015873015873
"chr19" 53806001 53807000 "*" 0 0 55.9633027522936
"chr19" 53843001 53844000 "*" 3.10862446895044e-15 1.22466437093774e-14 -100
"chr19" 53846001 53847000 "*" 0 0 -100
"chr19" 53904001 53905000 "*" 4.64135408995148e-07 6.15986676197051e-07
59.2592592592593
"chr19" 53926001 53927000 "*" 0 0 -100
"chr19" 53955001 53956000 "*" 2.00227675550835e-08 3.20459176617961e-08 100
"chr19" 54015001 54016000 "*" 5.22692999993524e-13 1.57301484865015e-12
65.5172413793103
"chr19" 54049001 54050000 "*" 0 0 -100
"chr19" 54099001 54100000 "*" 0 0 100
"chr19" 54101001 54102000 "*" 1.17905685215192e-13 3.83653245040640e-13 100
"chr19" 54186001 54187000 "*" 2.88710388929303e-10 6.03399264731139e-10 -100
"chr19" 54187001 54188000 "*" 0 0 100
"chr19" 54224001 54225000 "*" 7.7715611723761e-16 3.26213507634405e-15 -100

"chr19" 54247001 54248000 "*" 0 0 100
"chr19" 54268001 54269000 "*" 5.11424287705786e-05 5.02363135762244e-05 53.3333333333333
"chr19" 54272001 54273000 "*" 0 0 100
"chr19" 54287001 54288000 "*" 1.72385206109738e-09 3.22992593835751e-09 63.1578947368421
"chr19" 54299001 54300000 "*" 3.60632809426331e-06 4.22293748596603e-06 61.5384615384615
"chr19" 54307001 54308000 "*" 3.04161140718406e-11 7.26599935673121e-11 59.2592592592593
"chr19" 54346001 54347000 "*" 1.50623957750895e-12 4.28756717183481e-12 100
"chr19" 54349001 54350000 "*" 5.78292191910634e-10 1.16015519718754e-09 66.6666666666667
"chr19" 54350001 54351000 "*" 1.32615249803791e-06 1.65582211629877e-06 55.8823529411765
"chr19" 54369001 54370000 "*" 0 0 -73.4351741436737
"chr19" 54370001 54371000 "*" 0 0 -100
"chr19" 54382001 54383000 "*" 0 0 97.9591836734694
"chr19" 54404001 54405000 "*" 1.13140401492018e-08 1.87801380787728e-08 100
"chr19" 54410001 54411000 "*" 0 0 51.1129235766917
"chr19" 54417001 54418000 "*" 9.0072393987839e-13 2.63157725646847e-12 -100
"chr19" 54441001 54442000 "*" 5.01025332333427e-10 1.01358031135889e-09 100
"chr19" 54464001 54465000 "*" 0 0 66.6666666666667
"chr19" 54470001 54471000 "*" 0 0 100
"chr19" 54486001 54487000 "*" 0 0 76.2431776834445
"chr19" 54495001 54496000 "*" 0 0 52.3526326830292
"chr19" 54499001 54500000 "*" 9.47020240005259e-14 3.12491189162604e-13 100
"chr19" 54502001 54503000 "*" 0.000299166032473375 0.000259267996470385 -54.1666666666667
"chr19" 54511001 54512000 "*" 3.88065629408629e-07 5.2036181749383e-07 -52.6717557251908
"chr19" 54512001 54513000 "*" 2.70339306496226e-13 8.42355472617862e-13 -100
"chr19" 54547001 54548000 "*" 3.3742579085283e-06 3.96739780813697e-06 51.9480519480519
"chr19" 54565001 54566000 "*" 1.05632791402854e-09 2.03677080174149e-09 68.5714285714286
"chr19" 54568001 54569000 "*" 9.04570973681018e-10 1.76414389822173e-09 -100
"chr19" 54599001 54600000 "*" 0 0 69.1903095446403
"chr19" 54600001 54601000 "*" 0 0 -100
"chr19" 54605001 54606000 "*" 0 0 67.741935483871
"chr19" 54617001 54618000 "*" 0 0 60.0283837224136
"chr19" 54619001 54620000 "*" 0 0 -100
"chr19" 54658001 54659000 "*" 7.32747196252603e-15 2.76435639707144e-14 -56.4102564102564
"chr19" 54665001 54666000 "*" 6.39165064653113e-07 8.33531059852563e-07 -92.3076923076923
"chr19" 54677001 54678000 "*" 0 0 -54.3458544327715
"chr19" 54755001 54756000 "*" 0 0 -69.2741469057258
"chr19" 54757001 54758000 "*" 1.34431188403283e-09 2.55734467902157e-09 52.1739130434783
"chr19" 54759001 54760000 "*" 0 0 100
"chr19" 54785001 54786000 "*" 2.22044604925031e-16 9.81641919380259e-16 -100

"chr19" 54828001 54829000 "*" 0 0 -64.9671052631579
"chr19" 54837001 54838000 "*" 6.59550958292954e-09 1.13456194382773e-08 100
"chr19" 54849001 54850000 "*" 1.13140401492018e-08 1.87801380787728e-08 100
"chr19" 54900001 54901000 "*" 4.44089209850063e-16 1.91071758245033e-15 100
"chr19" 54932001 54933000 "*" 0 0 74.0588235294118
"chr19" 54938001 54939000 "*" 1.29671928750241e-09 2.47092150319297e-09
52.6315789473684
"chr19" 54941001 54942000 "*" 1.05529375238334e-08 1.76419648926669e-08
62.7450980392157
"chr19" 54942001 54943000 "*" 0 0 83.9285714285714
"chr19" 54961001 54962000 "*" 0 0 100
"chr19" 54984001 54985000 "*" 8.60915975843257e-05 8.15336677459667e-05 60
"chr19" 54985001 54986000 "*" 3.95861121660346e-12 1.0684496262032e-11 -100
"chr19" 54987001 54988000 "*" 4.11539381706127e-06 4.77326970666537e-06
66.6666666666667
"chr19" 54992001 54993000 "*" 8.3882012447134e-11 1.88922989503423e-10 -100
"chr19" 55019001 55020000 "*" 4.66582591007736e-07 6.1900791047296e-07
-52.6315789473684
"chr19" 55045001 55046000 "*" 2.79440914852103e-11 6.73046092948135e-11 100
"chr19" 55061001 55062000 "*" 5.55111512312578e-16 2.36485094870365e-15
-84.6153846153846
"chr19" 55066001 55067000 "*" 0 0 81.6091954022989
"chr19" 55107001 55108000 "*" 2.15125472990962e-10 4.58126659943874e-10 100
"chr19" 55109001 55110000 "*" 0 0 80
"chr19" 55136001 55137000 "*" 8.88178419700125e-16 3.70670032207938e-15
83.0769230769231
"chr19" 55211001 55212000 "*" 4.10141998141711e-09 7.26553281681452e-09
75.609756097561
"chr19" 55398001 55399000 "*" 1.35036426485158e-12 3.86629547363774e-12 100
"chr19" 55434001 55435000 "*" 4.44089209850063e-16 1.91071758245033e-15
80.7142857142857
"chr19" 55486001 55487000 "*" 8.80053807605918e-11 1.97819855335604e-10
-94.7368421052632
"chr19" 55500001 55501000 "*" 6.7390537594747e-14 2.26447131529591e-13 -100
"chr19" 55657001 55658000 "*" 4.99900121297969e-12 1.32921056339624e-11 -100
"chr19" 55667001 55668000 "*" 0 0 80.1857585139319
"chr19" 55675001 55676000 "*" 1.77635683940025e-15 7.19870740878856e-15
62.962962962963
"chr19" 55685001 55686000 "*" 0 0 55.6222031800438
"chr19" 55690001 55691000 "*" 0 0 57.8962703962704
"chr19" 55711001 55712000 "*" 9.70024884860976e-07 1.23311158070565e-06
64.2857142857143
"chr19" 55718001 55719000 "*" 0 0 -100
"chr19" 55720001 55721000 "*" 1.36604061395929e-11 3.42333952202875e-11 -100
"chr19" 55722001 55723000 "*" 1.39779633423487e-07 1.98463037958589e-07 -100
"chr19" 55726001 55727000 "*" 4.14335232790108e-13 1.26153063645578e-12 -100
"chr19" 55806001 55807000 "*" 0.000533283099452109 0.000442910457246435
-51.5151515151515
"chr19" 55840001 55841000 "*" 0 0 100
"chr19" 55845001 55846000 "*" 1.64506686384236e-07 2.31545082199018e-07
-61.9047619047619
"chr19" 55881001 55882000 "*" 0 0 55.0725263795565

"chr19" 55884001 55885000 "*" 3.11736206937141e-06 3.68539916214203e-06 -75
"chr19" 55885001 55886000 "*" 1.11022302462516e-16 5.03662826618488e-16
68.1818181818182
"chr19" 55908001 55909000 "*" 3.63445940010365e-11 8.58565209111204e-11 -100
"chr19" 55933001 55934000 "*" 1.5277158427196e-09 2.87642564232045e-09 100
"chr19" 55950001 55951000 "*" 4.95936625100057e-13 1.49941853130847e-12
54.8827292110874
"chr19" 55955001 55956000 "*" 0.000115288900086497 0.00010706844483635
-52.3809523809524
"chr19" 55978001 55979000 "*" 0 0 100
"chr19" 55987001 55988000 "*" 0 0 76.1904761904762
"chr19" 55996001 55997000 "*" 0 0 78.5617686216803
"chr19" 56010001 56011000 "*" 0 0 -97.6744186046512
"chr19" 56023001 56024000 "*" 1.52466927971773e-12 4.33322475981408e-12 100
"chr19" 56046001 56047000 "*" 6.90747459231034e-12 1.80140148623876e-11 100
"chr19" 56061001 56062000 "*" 0 0 68.8685321691834
"chr19" 56080001 56081000 "*" 3.10862446895044e-15 1.22466437093774e-14 -100
"chr19" 56098001 56099000 "*" 1.03112849738629e-05 1.1275907396131e-05
63.5555555555556
"chr19" 56179001 56180000 "*" 0 0 -56.8
"chr19" 56213001 56214000 "*" 0 0 99.3150684931507
"chr19" 56237001 56238000 "*" 2.06377137601521e-10 4.42217694737626e-10
73.6842105263158
"chr19" 56477001 56478000 "*" 2.08614236996141e-11 5.09698923781446e-11
-66.6666666666667
"chr19" 56519001 56520000 "*" 3.07531777821168e-14 1.07626052665081e-13 -100
"chr19" 56548001 56549000 "*" 8.14194944798174e-09 1.38388411725303e-08
85.1063829787234
"chr19" 56561001 56562000 "*" 2.08814465718632e-09 3.86232567960463e-09 -100
"chr19" 56567001 56568000 "*" 9.2148511043888e-15 3.43705279328267e-14 -100
"chr19" 56685001 56686000 "*" 4.71134242729931e-12 1.257615036806e-11 -100
"chr19" 56735001 56736000 "*" 0 0 73.1818181818182
"chr19" 56756001 56757000 "*" 6.80745044778774e-08 1.01435763508276e-07
57.1428571428571
"chr19" 56802001 56803000 "*" 2.22044604925031e-16 9.81641919380259e-16
-54.3478260869565
"chr19" 56974001 56975000 "*" 2.68450373042128e-10 5.64465213127486e-10 -100
"chr19" 57141001 57142000 "*" 9.41977680080086e-07 1.19996326635982e-06 -60
"chr19" 57271001 57272000 "*" 1.25516490534494e-07 1.79704779093716e-07 63.75
"chr19" 57307001 57308000 "*" 7.31885595572912e-07 9.46573137518221e-07
61.3636363636364
"chr19" 57423001 57424000 "*" 2.82773804372027e-13 8.79129664109438e-13 -100
"chr19" 57462001 57463000 "*" 3.50322792663782e-07 4.72754385845506e-07
57.1428571428571
"chr19" 57498001 57499000 "*" 3.6700841921089e-08 5.65530226072258e-08 -100
"chr19" 57506001 57507000 "*" 1.64313007644523e-14 5.95016254558654e-14
82.5396825396825
"chr19" 57513001 57514000 "*" 3.10640402290119e-13 9.61892999849369e-13
-55.5555555555556
"chr19" 57514001 57515000 "*" 0 0 80.3571428571429
"chr19" 57521001 57522000 "*" 0 0 100
"chr19" 57605001 57606000 "*" 3.84023590704885e-10 7.89524111758443e-10

61.8181818181818
"chr19" 57615001 57616000 "*" 3.44722487444216e-07 4.6586018916251e-07 -80
"chr19" 57702001 57703000 "*" 0 0 59.0935587292335
"chr19" 57831001 57832000 "*" 0 0 -75
"chr19" 57999001 58000000 "*" 0 0 -60.3809523809524
"chr19" 58115001 58116000 "*" 1.54630752646767e-11 3.85079502648228e-11 -100
"chr19" 58144001 58145000 "*" 1.55997437190081e-12 4.42903659136655e-12
55.0173010380623
"chr19" 58145001 58146000 "*" 6.66133814775094e-16 2.81595744474255e-15 -100
"chr19" 58361001 58362000 "*" 0 0 -78.3333333333333
"chr19" 58513001 58514000 "*" 0 0 69.3519606209276
"chr19" 58514001 58515000 "*" 0 0 72.0419515206441
"chr19" 58530001 58531000 "*" 2.22044604925031e-16 9.81641919380259e-16 -100
"chr19" 58558001 58559000 "*" 0 0 -67.032967032967
"chr19" 58567001 58568000 "*" 0 0 70.1754385964912
"chr19" 58604001 58605000 "*" 3.0991022979876e-05 3.14977384116983e-05
-62.7118644067797
"chr19" 58661001 58662000 "*" 0 0 59.8943233891331
"chr19" 58692001 58693000 "*" 1.83952852950142e-12 5.17499701329218e-12 -100
"chr19" 58704001 58705000 "*" 3.77475828372553e-15 1.47379557022071e-14 -100
"chr19" 58865001 58866000 "*" 1.77402315060249e-10 3.82975091762547e-10
-57.4816487859966
"chr19" 59117001 59118000 "*" 0 0 63.8772991714168
"chr2" 46001 47000 "*" 0 0 52.7916142557652
"chr2" 217001 218000 "*" 2.12052597703405e-14 7.58083691658395e-14
84.8837209302326
"chr2" 264001 265000 "*" 0 0 -51.2548262548263
"chr2" 288001 289000 "*" 0 0 64.866489806594
"chr2" 289001 290000 "*" 0 0 79.2250578709262
"chr2" 306001 307000 "*" 5.21804821573824e-15 2.00446252136729e-14
-92.8571428571429
"chr2" 395001 396000 "*" 0 0 -100
"chr2" 424001 425000 "*" 9.2148511043888e-15 3.43705279328267e-14 -100
"chr2" 429001 430000 "*" 2.5681843762726e-08 4.05529231753584e-08 53
"chr2" 444001 445000 "*" 0 0 78.9446589446589
"chr2" 453001 454000 "*" 4.915795842797e-05 4.83987297040465e-05
-58.3333333333333
"chr2" 489001 490000 "*" 1.4619805333016e-07 2.07082533005235e-07
50.1108647450111
"chr2" 502001 503000 "*" 8.15780776264319e-12 2.11202226717963e-11
56.5829871771717
"chr2" 553001 554000 "*" 1.90520132559158e-06 2.32564618288801e-06
-67.5675675675676
"chr2" 564001 565000 "*" 1.4432899320127e-15 5.90750815956055e-15
56.3829787234043
"chr2" 567001 568000 "*" 3.78053144345358e-12 1.02353175555878e-11
-93.8775510204082
"chr2" 573001 574000 "*" 0 0 -100
"chr2" 584001 585000 "*" 0.000591775622736268 0.000487833560499899
-51.7241379310345
"chr2" 622001 623000 "*" 0 0 56.6265060240964
"chr2" 629001 630000 "*" 0 0 70.1298701298701

"chr2" 650001 651000 "*" 3.61865315490206e-10 7.45986023744022e-10 61.3636363636364
"chr2" 740001 741000 "*" 6.17773610045447e-11 1.41796774935e-10 53.0386740331492
"chr2" 751001 752000 "*" 1.73731917740838e-10 3.75401275370312e-10 -84.2105263157895
"chr2" 791001 792000 "*" 4.01313771103418e-09 7.11882087237587e-09 -100
"chr2" 797001 798000 "*" 2.88779000712225e-11 6.92935809466068e-11 -100
"chr2" 838001 839000 "*" 0 0 -65.1719901719902
"chr2" 851001 852000 "*" 2.15125472990962e-10 4.58126659943874e-10 -100
"chr2" 856001 857000 "*" 4.88498130835069e-15 1.8824139250584e-14 -63.1578947368421
"chr2" 910001 911000 "*" 8.06688049692639e-13 2.37556391305009e-12 100
"chr2" 916001 917000 "*" 6.06172749773037e-08 9.09463010273961e-08 66.6666666666667
"chr2" 956001 957000 "*" 1.75415237890775e-13 5.61171963912381e-13 -78.8461538461538
"chr2" 961001 962000 "*" 7.88097879887983e-05 7.51124636744888e-05 -52.7272727272727
"chr2" 1004001 1005000 "*" 7.18203274630014e-11 1.63247175597028e-10 72.7272727272727
"chr2" 1049001 1050000 "*" 3.16908455033627e-09 5.71937722353377e-09 -61.8181818181818
"chr2" 1066001 1067000 "*" 1.67299207820548e-08 2.71377429441055e-08 100
"chr2" 1071001 1072000 "*" 0 0 97.5206611570248
"chr2" 1076001 1077000 "*" 1.11022302462516e-16 5.03662826618488e-16 100
"chr2" 1080001 1081000 "*" 0 0 -90.1960784313726
"chr2" 1098001 1099000 "*" 2.17666781932024e-07 3.01870085309906e-07 53.2258064516129
"chr2" 1112001 1113000 "*" 0 0 -66.995709942105
"chr2" 1133001 1134000 "*" 8.65973959207622e-14 2.87362558212404e-13 -100
"chr2" 1134001 1135000 "*" 0 0 70.7142294668872
"chr2" 1152001 1153000 "*" 0.000199851252180694 0.000178453492150885 54.5454545454545
"chr2" 1159001 1160000 "*" 3.16746628925557e-13 9.80364047175588e-13 83.6734693877551
"chr2" 1204001 1205000 "*" 1.17300613666771e-11 2.9684891779287e-11 69.8529411764706
"chr2" 1231001 1232000 "*" 4.16036094463834e-11 9.75284600804856e-11 55.0310559006211
"chr2" 1299001 1300000 "*" 2.25024332500823e-10 4.78278910413349e-10 90
"chr2" 1304001 1305000 "*" 7.67056418382595e-11 1.7359448580318e-10 -100
"chr2" 1382001 1383000 "*" 6.36093400174786e-11 1.45815603629271e-10 -70.8333333333333
"chr2" 1415001 1416000 "*" 7.94730947717426e-12 2.05982705398163e-11 -91.6666666666667
"chr2" 1425001 1426000 "*" 0 0 55.5944055944056
"chr2" 1484001 1485000 "*" 0 0 -60.362317242596
"chr2" 1490001 1491000 "*" 4.48240371486719e-08 6.84329675653433e-08 -52.1739130434783
"chr2" 1496001 1497000 "*" 0 0 -59.375
"chr2" 1503001 1504000 "*" 0 0 100

"chr2" 1509001 1510000 "*" 3.6700841921089e-08 5.65530226072258e-08 -100
"chr2" 1529001 1530000 "*" 1.48487888651516e-11 3.70689457767456e-11 -100
"chr2" 1542001 1543000 "*" 7.87321319251078e-11 1.7801644733677e-10 -88.2352941176471
"chr2" 1565001 1566000 "*" 5.55111512312578e-16 2.36485094870365e-15 -62.9993112947658
"chr2" 1568001 1569000 "*" 8.41964529207928e-07 1.07968980226462e-06 54.1666666666667
"chr2" 1611001 1612000 "*" 6.25199891857164e-12 1.64423898607046e-11 91.6666666666667
"chr2" 1612001 1613000 "*" 1.78084136326362e-09 3.33101755828139e-09 67.6470588235294
"chr2" 1628001 1629000 "*" 2.23154827949656e-14 7.95931582053377e-14 -100
"chr2" 1655001 1656000 "*" 1.01763042437142e-12 2.95398999861281e-12 -63.0665380906461
"chr2" 1663001 1664000 "*" 0 0 -84.7619047619048
"chr2" 1691001 1692000 "*" 1.64716754680327e-06 2.02893190508151e-06 54.1666666666667
"chr2" 1723001 1724000 "*" 4.22541494016215e-08 6.467525895531e-08 81.0344827586207
"chr2" 1724001 1725000 "*" 1.0991207943789e-14 4.06651762510369e-14 71.830985915493
"chr2" 1731001 1732000 "*" 2.26596519325994e-13 7.13089866526877e-13 50.1067008109262
"chr2" 1749001 1750000 "*" 0 0 100
"chr2" 1770001 1771000 "*" 7.6213924060653e-11 1.72819314449538e-10 71.2121212121212
"chr2" 1822001 1823000 "*" 0 0 100
"chr2" 1826001 1827000 "*" 0.000168512691140155 0.000152316702506419 54.0540540540541
"chr2" 1847001 1848000 "*" 3.68657326887956e-11 8.70284412393242e-11 50.4081632653061
"chr2" 1849001 1850000 "*" 3.50164341966774e-13 1.07835278686819e-12 54.3859649122807
"chr2" 1858001 1859000 "*" 0 0 100
"chr2" 1859001 1860000 "*" 0 0 81.4814814814815
"chr2" 1870001 1871000 "*" 9.5812247025151e-14 3.15667542866807e-13 100
"chr2" 1878001 1879000 "*" 0 0 -86.5087040618956
"chr2" 1906001 1907000 "*" 2.1183055309848e-13 6.69755696712775e-13 68.8888888888889
"chr2" 1908001 1909000 "*" 4.08209022140227e-11 9.57770690255255e-11 100
"chr2" 1914001 1915000 "*" 1.25628071057093e-07 1.79852181494098e-07 -56.3636363636364
"chr2" 1918001 1919000 "*" 1.05207310746591e-05 1.14877756514443e-05 -54.3147208121827
"chr2" 1943001 1944000 "*" 3.11792591745075e-10 6.49135511658488e-10 68.75
"chr2" 1969001 1970000 "*" 5.02931030155196e-14 1.71897866655592e-13 -65.1162790697674
"chr2" 1977001 1978000 "*" 2.00227675550835e-08 3.20459176617961e-08 -100
"chr2" 1983001 1984000 "*" 4.3578849417969e-09 7.7002254546527e-09 -84.7682119205298
"chr2" 1998001 1999000 "*" 8.01666433236647e-09 1.36392368970109e-08 100

"chr2" 2017001 2018000 "*" 2.5448444090026e-05 2.62004281959145e-05
52.1739130434783
"chr2" 2028001 2029000 "*" 0 0 74.5762711864407
"chr2" 2046001 2047000 "*" 2.85679702116681e-10 5.98896168379121e-10
-54.0277777777778
"chr2" 2063001 2064000 "*" 0 0 95.360824742268
"chr2" 2112001 2113000 "*" 2.3990809339125e-12 6.65441738642456e-12 100
"chr2" 2118001 2119000 "*" 6.92287338566189e-11 1.57605630124247e-10 100
"chr2" 2119001 2120000 "*" 6.31515562066198e-09 1.09226126137559e-08
-83.5443037974684
"chr2" 2125001 2126000 "*" 0 0 50.6410256410256
"chr2" 2162001 2163000 "*" 2.16382467499443e-13 6.82775854166559e-13 100
"chr2" 2165001 2166000 "*" 9.08326895143752e-07 1.15980672920235e-06
64.2857142857143
"chr2" 2176001 2177000 "*" 0 0 88.2352941176471
"chr2" 2245001 2246000 "*" 2.48184182993771e-06 2.97686444206053e-06 90
"chr2" 2257001 2258000 "*" 1.55109347588223e-09 2.91823133735765e-09 68.75
"chr2" 2270001 2271000 "*" 7.09291175793325e-08 1.0496172468571e-07
-51.2820512820513
"chr2" 2271001 2272000 "*" 8.67505395296675e-08 1.26895177832038e-07
77.5510204081633
"chr2" 2273001 2274000 "*" 6.19948536950687e-13 1.84855702778088e-12
83.3333333333333
"chr2" 2278001 2279000 "*" 0 0 100
"chr2" 2290001 2291000 "*" 1.83952852950142e-12 5.17499701329218e-12 -100
"chr2" 2296001 2297000 "*" 1.765254609154e-14 6.36724611765475e-14
66.6666666666667
"chr2" 2307001 2308000 "*" 0 0 100
"chr2" 2328001 2329000 "*" 0 0 85.7142857142857
"chr2" 2329001 2330000 "*" 0 0 100
"chr2" 2506001 2507000 "*" 0 0 -100
"chr2" 2511001 2512000 "*" 5.04892794239709e-11 1.17078382179195e-10
-51.4285714285714
"chr2" 2533001 2534000 "*" 3.04324343503026e-11 7.26599935673121e-11 100
"chr2" 2565001 2566000 "*" 1.37828859436695e-10 3.01445544267661e-10 100
"chr2" 2570001 2571000 "*" 0 0 -69.0140845070423
"chr2" 2574001 2575000 "*" 5.49874151989371e-07 7.23387714075565e-07
-59.4594594594595
"chr2" 2590001 2591000 "*" 0 0 100
"chr2" 2610001 2611000 "*" 2.1094237467878e-15 8.47434540879246e-15 100
"chr2" 2612001 2613000 "*" 1.52794177310511e-09 2.87642564232045e-09 100
"chr2" 2665001 2666000 "*" 7.88724484612757e-08 1.15977593945077e-07
51.2345679012346
"chr2" 2667001 2668000 "*" 1.4432899320127e-15 5.90750815956055e-15 -60
"chr2" 2699001 2700000 "*" 9.47020240005259e-14 3.12491189162604e-13 100
"chr2" 2700001 2701000 "*" 5.15661957578573e-11 1.19459366858729e-10
63.8888888888889
"chr2" 2701001 2702000 "*" 4.32209823486573e-12 1.15935290479641e-11 -100
"chr2" 2702001 2703000 "*" 8.54871728961371e-15 3.20277157428094e-14
74.1573033707865
"chr2" 2710001 2711000 "*" 1.12634901405784e-10 2.49563754426169e-10 100
"chr2" 2716001 2717000 "*" 0 0 86.3013698630137

"chr2" 2719001 2720000 "*" 2.03528637277017e-07 2.83360546911656e-07 75.6756756756757
"chr2" 2721001 2722000 "*" 3.53400475439081e-06 4.14231950904509e-06 -64.5161290322581
"chr2" 2752001 2753000 "*" 2.82538422879952e-07 3.86166853308865e-07 69.4444444444444
"chr2" 2772001 2773000 "*" 1.19904086659517e-14 4.40901388691796e-14 100
"chr2" 2776001 2777000 "*" 1.15092973151931e-07 1.6561816717757e-07 73.5849056603774
"chr2" 2784001 2785000 "*" 6.24642559898803e-11 1.43308827169057e-10 -54.1666666666667
"chr2" 2793001 2794000 "*" 7.11652958784725e-14 2.3851612587684e-13 77.2727272727273
"chr2" 2794001 2795000 "*" 1.81731929504991e-09 3.3959735570263e-09 61.5384615384615
"chr2" 2843001 2844000 "*" 7.64680227882675e-06 8.52593734901427e-06 75
"chr2" 2844001 2845000 "*" 0 0 62.962962962963
"chr2" 2867001 2868000 "*" 2.40302466814057e-09 4.41236283125202e-09 70.2127659574468
"chr2" 2895001 2896000 "*" 8.57092175010621e-13 2.51149198099779e-12 -100
"chr2" 2909001 2910000 "*" 1.51448252283526e-06 1.87605118233228e-06 51.4285714285714
"chr2" 2915001 2916000 "*" 0 0 66.6666666666667
"chr2" 2917001 2918000 "*" 0 0 58.2505841121495
"chr2" 3013001 3014000 "*" 2.00794936233706e-12 5.62017573241497e-12 -88.2352941176471
"chr2" 3052001 3053000 "*" 8.43769498715119e-15 3.16245570171727e-14 -77.027027027027
"chr2" 3108001 3109000 "*" 2.00227675550835e-08 3.20459176617961e-08 100
"chr2" 3110001 3111000 "*" 1.30168276084586e-05 1.40182141257152e-05 63.1578947368421
"chr2" 3129001 3130000 "*" 2.4736644054224e-06 2.96786695267589e-06 -60
"chr2" 3134001 3135000 "*" 0 0 78.9473684210526
"chr2" 3243001 3244000 "*" 1.51423382721561e-08 2.47643259402449e-08 -75.4098360655738
"chr2" 3353001 3354000 "*" 0 0 -55.5581723975506
"chr2" 3431001 3432000 "*" 3.33066907387547e-16 1.4495649018245e-15 95.2380952380952
"chr2" 3584001 3585000 "*" 3.53754581183807e-10 7.30788136008935e-10 -60.4166666666667
"chr2" 3600001 3601000 "*" 4.08209022140227e-11 9.57770690255255e-11 -100
"chr2" 3622001 3623000 "*" 0 0 -74.5833333333333
"chr2" 3635001 3636000 "*" 0 0 -100
"chr2" 3676001 3677000 "*" 1.90181204118289e-13 6.05298617539364e-13 -100
"chr2" 3693001 3694000 "*" 5.69716140930154e-09 9.90984279067739e-09 -63.3333333333333
"chr2" 3694001 3695000 "*" 2.43005615629954e-09 4.45014222280278e-09 -100
"chr2" 3774001 3775000 "*" 3.30512284207884e-11 7.86244754841384e-11 72.4770642201835
"chr2" 3779001 3780000 "*" 6.24654217240561e-10 1.2462108191303e-09 100
"chr2" 3793001 3794000 "*" 4.6664101471805e-09 8.2173488444357e-09 -64.8648648648649

"chr2" 3797001 3798000 "*" 3.3002561794504e-09 5.94497676880652e-09
-63.9344262295082
"chr2" 3801001 3802000 "*" 8.7349629751543e-09 1.47505871898511e-08 -100
"chr2" 3802001 3803000 "*" 2.62723176547297e-12 7.2556362906511e-12
80.5555555555556
"chr2" 3965001 3966000 "*" 0 0 85
"chr2" 3975001 3976000 "*" 0 0 -100
"chr2" 4000001 4001000 "*" 1.24344978758018e-14 4.56627092294518e-14 100
"chr2" 4023001 4024000 "*" 1.98365768255826e-11 4.85612402473442e-11 -100
"chr2" 4072001 4073000 "*" 0 0 100
"chr2" 4102001 4103000 "*" 3.6700841921089e-08 5.65530226072258e-08 100
"chr2" 4158001 4159000 "*" 2.15125472990962e-10 4.58126659943874e-10 -100
"chr2" 4245001 4246000 "*" 3.63445940010365e-11 8.58565209111204e-11 -100
"chr2" 4320001 4321000 "*" 6.59550958292954e-09 1.13456194382773e-08 100
"chr2" 4465001 4466000 "*" 3.6700841921089e-08 5.65530226072258e-08 -100
"chr2" 4470001 4471000 "*" 2.02060590481778e-13 6.40127405465654e-13 -100
"chr2" 4601001 4602000 "*" 1.36113342819044e-13 4.40236859457983e-13
66.6666666666667
"chr2" 4942001 4943000 "*" 0 0 -77.0833333333333
"chr2" 5466001 5467000 "*" 6.09507309701218e-06 6.8968637727707e-06
-57.1428571428571
"chr2" 5517001 5518000 "*" 5.62926395586416e-06 6.40552376199523e-06
66.6666666666667
"chr2" 5524001 5525000 "*" 0 0 100
"chr2" 5593001 5594000 "*" 9.61675183930311e-13 2.801372325071e-12
-59.5588235294118
"chr2" 5634001 5635000 "*" 4.51727544259484e-12 1.20906264543166e-11
-53.7037037037037
"chr2" 5647001 5648000 "*" 2.43065123584074e-10 5.13977858470558e-10
-58.3333333333333
"chr2" 5666001 5667000 "*" 4.01313771103418e-09 7.11882087237587e-09 100
"chr2" 5830001 5831000 "*" 0 0 100
"chr2" 5899001 5900000 "*" 1.26565424807268e-14 4.63923203146481e-14 -100
"chr2" 5922001 5923000 "*" 6.63913368725844e-14 2.23251502617671e-13
-82.7586206896552
"chr2" 6024001 6025000 "*" 0 0 -58.4615384615385
"chr2" 6072001 6073000 "*" 3.23685744874069e-10 6.72402528685186e-10
-70.8333333333333
"chr2" 6073001 6074000 "*" 0 0 -66.6666666666667
"chr2" 6125001 6126000 "*" 1.1988837312904e-06 1.5057940841428e-06
-58.3333333333333
"chr2" 6308001 6309000 "*" 2.25462126746123e-09 4.15404464960385e-09
-87.6404494382023
"chr2" 6353001 6354000 "*" 2.62900812231237e-13 8.21294938531841e-13 -100
"chr2" 6423001 6424000 "*" 1.19571019752129e-13 3.88829351764533e-13
-96.2732919254658
"chr2" 6598001 6599000 "*" 4.7840345573924e-08 7.27476615882735e-08
-61.5384615384615
"chr2" 6599001 6600000 "*" 0 0 -96.7532467532468
"chr2" 6704001 6705000 "*" 8.01666433236647e-09 1.36392368970109e-08 -100
"chr2" 6897001 6898000 "*" 4.71839415316921e-08 7.18031860144247e-08
-60.9523809523809

"chr2" 6900001 6901000 "*" 1.6509434286327e-07 2.32329085641623e-07
-60.7142857142857
"chr2" 7044001 7045000 "*" 1.55431223447522e-15 6.33705141101682e-15 100
"chr2" 7062001 7063000 "*" 5.51780843238703e-14 1.87243792572872e-13 -100
"chr2" 7084001 7085000 "*" 0 0 100
"chr2" 7097001 7098000 "*" 5.04263297784746e-13 1.52078941532818e-12 -100
"chr2" 7100001 7101000 "*" 1.67299207820548e-08 2.71377429441055e-08 -100
"chr2" 7147001 7148000 "*" 1.11022302462516e-16 5.03662826618488e-16 100
"chr2" 7149001 7150000 "*" 0 0 100
"chr2" 7231001 7232000 "*" 3.40125705378114e-10 7.03784073980162e-10 100
"chr2" 7234001 7235000 "*" 0 0 -100
"chr2" 7249001 7250000 "*" 1.96644922567657e-11 4.82930612417392e-11 -100
"chr2" 7348001 7349000 "*" 2.88657986402541e-15 1.14155613124573e-14 100
"chr2" 7386001 7387000 "*" 1.67299207820548e-08 2.71377429441055e-08 100
"chr2" 7405001 7406000 "*" 3.94939636549907e-12 1.06661833973061e-11
55.3191489361702
"chr2" 7408001 7409000 "*" 6.4152538836737e-10 1.27477697728417e-09 100
"chr2" 7497001 7498000 "*" 1.83952852950142e-12 5.17499701329218e-12 100
"chr2" 7568001 7569000 "*" 2.00227675550835e-08 3.20459176617961e-08 -100
"chr2" 7660001 7661000 "*" 0 0 100
"chr2" 7740001 7741000 "*" 8.43347613965761e-12 2.17859001990012e-11 -100
"chr2" 7872001 7873000 "*" 2.08814465718632e-09 3.86232567960463e-09 -100
"chr2" 7981001 7982000 "*" 0 0 100
"chr2" 8218001 8219000 "*" 7.0006535457523e-08 1.03695832512054e-07 -100
"chr2" 8359001 8360000 "*" 8.80406858527749e-14 2.91456141063452e-13 100
"chr2" 8362001 8363000 "*" 4.32209823486573e-12 1.15935290479641e-11 100
"chr2" 8364001 8365000 "*" 2.77555756156289e-15 1.10015415195978e-14
86.5853658536585
"chr2" 8532001 8533000 "*" 1.58346331602388e-08 2.58271480686597e-08
78.8461538461538
"chr2" 8540001 8541000 "*" 0 0 -56.3829787234043
"chr2" 8581001 8582000 "*" 0 0 100
"chr2" 8634001 8635000 "*" 2.46348476062863e-08 3.89838047190206e-08
-51.7241379310345
"chr2" 8665001 8666000 "*" 1.92068583260152e-14 6.89775382589628e-14 100
"chr2" 8707001 8708000 "*" 5.220638035075e-05 5.11909765154034e-05 -53.125
"chr2" 8723001 8724000 "*" 5.88418203051333e-15 2.245355428447e-14
58.6206896551724
"chr2" 8738001 8739000 "*" 4.01313771103418e-09 7.11882087237587e-09 -100
"chr2" 8791001 8792000 "*" 1.5017558308017e-09 2.84007897884734e-09
64.2857142857143
"chr2" 8799001 8800000 "*" 5.7065463465733e-12 1.50892470940709e-11
55.6445461479786
"chr2" 8818001 8819000 "*" 0 0 64.0102918370224
"chr2" 8822001 8823000 "*" 0 0 65.2777777777778
"chr2" 8846001 8847000 "*" 5.48625589402718e-11 1.26771829805218e-10
-89.6551724137931
"chr2" 8849001 8850000 "*" 1.11022302462516e-16 5.03662826618488e-16
-63.9880952380952
"chr2" 8968001 8969000 "*" 0 0 100
"chr2" 9134001 9135000 "*" 0 0 100
"chr2" 9252001 9253000 "*" 0 0 -51.5781637717122

"chr2" 9276001 9277000 "*" 9.99200722162641e-16 4.15382497462808e-15 -100
"chr2" 9307001 9308000 "*" 4.20108392518159e-13 1.27728966274775e-12
-97.8723404255319
"chr2" 9439001 9440000 "*" 8.77076189453874e-15 3.27889038038365e-14 -100
"chr2" 9480001 9481000 "*" 9.80763359414993e-10 1.8980032424715e-09 100
"chr2" 9624001 9625000 "*" 0 0 100
"chr2" 9752001 9753000 "*" 1.4432899320127e-15 5.90750815956055e-15 -100
"chr2" 9865001 9866000 "*" 2.22044604925031e-16 9.81641919380259e-16
-67.1390013495277
"chr2" 9891001 9892000 "*" 7.01360969301845e-10 1.38739578442437e-09
78.8732394366197
"chr2" 9893001 9894000 "*" 0 0 -100
"chr2" 9914001 9915000 "*" 3.33066907387547e-16 1.4495649018245e-15
-90.6542056074766
"chr2" 9928001 9929000 "*" 4.78841410966879e-11 1.11468187156588e-10 100
"chr2" 9942001 9943000 "*" 0 0 -93.9393939393939
"chr2" 10148001 10149000 "*" 0 0 -73.4526112185687
"chr2" 10157001 10158000 "*" 0.000726374156166165 0.000590081342527627
52.1739130434783
"chr2" 10165001 10166000 "*" 0 0 -100
"chr2" 10170001 10171000 "*" 8.09996743567076e-07 1.04131538148627e-06 56
"chr2" 10188001 10189000 "*" 0 0 100
"chr2" 10197001 10198000 "*" 0 0 53.7313432835821
"chr2" 10208001 10209000 "*" 2.33028818463765e-10 4.93719283454501e-10 -100
"chr2" 10213001 10214000 "*" 0 0 -73.1707317073171
"chr2" 10224001 10225000 "*" 5.15657918086498e-05 5.06175998528116e-05 -62.5
"chr2" 10233001 10234000 "*" 2.15125472990962e-10 4.58126659943874e-10 -100
"chr2" 10240001 10241000 "*" 1.35447209004269e-14 4.94674708073053e-14 -100
"chr2" 10272001 10273000 "*" 9.04570973681018e-10 1.76414389822173e-09 -100
"chr2" 10408001 10409000 "*" 0 0 -85.8108108108108
"chr2" 10423001 10424000 "*" 0 0 -100
"chr2" 10425001 10426000 "*" 6.54550080803062e-10 1.29935812583121e-09
-52.9281277728483
"chr2" 10429001 10430000 "*" 8.7349629751543e-09 1.47505871898511e-08 -100
"chr2" 10470001 10471000 "*" 2.66453525910038e-15 1.05861327033776e-14
-80.7453416149068
"chr2" 10577001 10578000 "*" 2.69018141096922e-12 7.41487347763769e-12 100
"chr2" 10590001 10591000 "*" 5.77038417048925e-11 1.32902726133415e-10
52.9411764705882
"chr2" 10638001 10639000 "*" 0 0 -70.7650273224044
"chr2" 10742001 10743000 "*" 3.33807043118028e-08 5.19772864618523e-08
60.7142857142857
"chr2" 10749001 10750000 "*" 3.45248512001461e-08 5.35891134143653e-08 -52
"chr2" 10847001 10848000 "*" 5.51988590481445e-05 5.39040714316344e-05
-58.8235294117647
"chr2" 10866001 10867000 "*" 7.67056418382595e-11 1.7359448580318e-10 100
"chr2" 10905001 10906000 "*" 5.63660229602192e-13 1.68905058935478e-12 100
"chr2" 10968001 10969000 "*" 3.12929393508909e-09 5.6513565147891e-09 90
"chr2" 10977001 10978000 "*" 3.26405569239796e-14 1.13973003849234e-13 -100
"chr2" 10987001 10988000 "*" 1.77635683940025e-15 7.19870740878856e-15
75.8620689655172
"chr2" 11004001 11005000 "*" 2.15447659712709e-11 5.25624834884597e-11 -86.25

"chr2" 11006001 11007000 "*" 4.99020824662466e-11 1.15790792586157e-10 100
"chr2" 11017001 11018000 "*" 2.59712167327564e-08 4.09881271012004e-08 -65.625
"chr2" 11024001 11025000 "*" 7.67056418382595e-11 1.7359448580318e-10 100
"chr2" 11047001 11048000 "*" 0 0 -100
"chr2" 11058001 11059000 "*" 3.6700841921089e-08 5.65530226072258e-08 -100
"chr2" 11059001 11060000 "*" 0 0 61.5384615384615
"chr2" 11065001 11066000 "*" 4.26951363152739e-10 8.72510873516957e-10 100
"chr2" 11071001 11072000 "*" 1.55431223447522e-15 6.33705141101682e-15
63.1147540983607
"chr2" 11074001 11075000 "*" 0 0 77.5510204081633
"chr2" 11096001 11097000 "*" 2.06390460277817e-13 6.53199310681027e-13
54.3157894736842
"chr2" 11098001 11099000 "*" 2.08814465718632e-09 3.86232567960463e-09 -100
"chr2" 11124001 11125000 "*" 7.90449119492109e-08 1.16206605825357e-07 60
"chr2" 11133001 11134000 "*" 6.59550958292954e-09 1.13456194382773e-08 -100
"chr2" 11210001 11211000 "*" 5.12252717532746e-06 5.865457620008e-06
-51.5151515151515
"chr2" 11262001 11263000 "*" 1.88737914186277e-15 7.62011598380321e-15 100
"chr2" 11273001 11274000 "*" 0 0 -80.8080808080808
"chr2" 11274001 11275000 "*" 6.4152538836737e-10 1.27477697728417e-09 100
"chr2" 11491001 11492000 "*" 0 0 -66.6666666666667
"chr2" 11526001 11527000 "*" 0 0 -75
"chr2" 11555001 11556000 "*" 1.11022302462516e-16 5.03662826618488e-16 100
"chr2" 11635001 11636000 "*" 1.11873183361588e-06 1.41066833954357e-06
-66.6666666666667
"chr2" 11638001 11639000 "*" 2.61657427258477e-06 3.12843692934624e-06 52
"chr2" 11645001 11646000 "*" 9.65729496371637e-10 1.87255912493232e-09 -100
"chr2" 11676001 11677000 "*" 4.73234496034536e-09 8.30627300264826e-09 100
"chr2" 11696001 11697000 "*" 2.23154827949656e-14 7.95931582053377e-14 -100
"chr2" 11702001 11703000 "*" 1.12376774552558e-12 3.24761818527664e-12 100
"chr2" 11740001 11741000 "*" 8.88178419700125e-16 3.70670032207938e-15 -100
"chr2" 11816001 11817000 "*" 2.34871788684643e-10 4.97421809924291e-10
58.1395348837209
"chr2" 11827001 11828000 "*" 4.47368257310288e-05 4.43153009261903e-05
56.6666666666667
"chr2" 11836001 11837000 "*" 8.8742654780205e-08 1.29615930712427e-07
-53.3333333333333
"chr2" 11860001 11861000 "*" 9.80763359414993e-10 1.8980032424715e-09 -100
"chr2" 11894001 11895000 "*" 2.76310766611498e-06 3.29291968157958e-06
-66.6666666666667
"chr2" 11970001 11971000 "*" 0 0 -100
"chr2" 11997001 11998000 "*" 3.95861121660346e-12 1.0684496262032e-11 100
"chr2" 12042001 12043000 "*" 0 0 98.5074626865672
"chr2" 12084001 12085000 "*" 3.40125705378114e-10 7.03784073980162e-10 100
"chr2" 12125001 12126000 "*" 2.79440914852103e-11 6.73046092948135e-11 -100
"chr2" 12134001 12135000 "*" 3.33066907387547e-16 1.4495649018245e-15 100
"chr2" 12606001 12607000 "*" 1.33634214805056e-11 3.35607415271157e-11
-93.9393939393939
"chr2" 12782001 12783000 "*" 4.02167188440217e-12 1.08266700497926e-11 -100
"chr2" 12877001 12878000 "*" 9.80763359414993e-10 1.8980032424715e-09 -100
"chr2" 12879001 12880000 "*" 3.34128893442198e-08 5.19772864618523e-08 100
"chr2" 12958001 12959000 "*" 3.28319936359067e-05 3.32125799277005e-05 60

"chr2" 13084001 13085000 "*" 3.10862446895044e-15 1.22466437093774e-14 -100
"chr2" 13156001 13157000 "*" 7.0006535457523e-08 1.03695832512054e-07 100
"chr2" 13180001 13181000 "*" 4.99654712049491e-08 7.57871259964003e-08
56.5217391304348
"chr2" 14322001 14323000 "*" 0 0 -90.4977375565611
"chr2" 14695001 14696000 "*" 2.15125472990962e-10 4.58126659943874e-10 -100
"chr2" 14772001 14773000 "*" 0 0 96.0902255639098
"chr2" 14773001 14774000 "*" 0 0 92.6100902603377
"chr2" 14809001 14810000 "*" 7.0006535457523e-08 1.03695832512054e-07 100
"chr2" 14891001 14892000 "*" 5.04263297784746e-13 1.52078941532818e-12 -100
"chr2" 14988001 14989000 "*" 1.16573417585641e-14 4.29335582900508e-14
65.7142857142857
"chr2" 15228001 15229000 "*" 2.08995487582797e-10 4.46944006972663e-10 -100
"chr2" 15268001 15269000 "*" 6.42352513291478e-07 8.37465176000401e-07
83.3333333333333
"chr2" 15551001 15552000 "*" 1.83952852950142e-12 5.17499701329218e-12 100
"chr2" 15838001 15839000 "*" 1.41574309364678e-06 1.76119388950473e-06
67.741935483871
"chr2" 15857001 15858000 "*" 2.33776331626245e-11 5.68379121145318e-11
-61.2903225806452
"chr2" 15859001 15860000 "*" 9.34677655606286e-07 1.19141004630311e-06
-60.6060606060606
"chr2" 15864001 15865000 "*" 2.33982833108826e-09 4.30200663260379e-09 -60
"chr2" 15870001 15871000 "*" 0 0 -100
"chr2" 16052001 16053000 "*" 6.30606677987089e-14 2.12703374819495e-13
83.9506172839506
"chr2" 16077001 16078000 "*" 1.25284005392245e-10 2.75649590272477e-10 -100
"chr2" 16080001 16081000 "*" 0 0 68.3333333333333
"chr2" 16081001 16082000 "*" 0 0 -56.390977443609
"chr2" 16133001 16134000 "*" 9.34325938839464e-09 1.57224152568657e-08
69.2307692307692
"chr2" 16148001 16149000 "*" 6.88338275267597e-15 2.60518659957509e-14 -100
"chr2" 16153001 16154000 "*" 0 0 -52.1917040358744
"chr2" 16154001 16155000 "*" 9.78961356423724e-12 2.50740309047781e-11
-54.3841999364609
"chr2" 16193001 16194000 "*" 2.15125472990962e-10 4.58126659943874e-10 100
"chr2" 16198001 16199000 "*" 0 0 -100
"chr2" 16233001 16234000 "*" 4.71134242729931e-12 1.257615036806e-11 100
"chr2" 16251001 16252000 "*" 1.87620141645084e-10 4.04133644431758e-10
-53.6585365853659
"chr2" 16263001 16264000 "*" 2.70339306496226e-13 8.42355472617862e-13 -100
"chr2" 16269001 16270000 "*" 5.16242723014315e-08 7.81284076811498e-08
-61.0778443113772
"chr2" 16383001 16384000 "*" 2.88779000712225e-11 6.92935809466068e-11 100
"chr2" 16484001 16485000 "*" 0 0 -100
"chr2" 16621001 16622000 "*" 7.0006535457523e-08 1.03695832512054e-07 -100
"chr2" 16847001 16848000 "*" 1.23037811050608e-08 2.03365951786567e-08
-77.027027027027
"chr2" 16917001 16918000 "*" 1.74853465040314e-11 4.32752710900609e-11 100
"chr2" 16928001 16929000 "*" 1.96509475358653e-13 6.23868565133654e-13 100
"chr2" 17019001 17020000 "*" 1.13140401492018e-08 1.87801380787728e-08 -100
"chr2" 17044001 17045000 "*" 2.22044604925031e-16 9.81641919380259e-16 -100

"chr2" 17721001 17722000 "*" 0 0 -70.7686622320769
"chr2" 17816001 17817000 "*" 1.66533453693773e-15 6.7629186866784e-15 100
"chr2" 17998001 17999000 "*" 0 0 100
"chr2" 18059001 18060000 "*" 0 0 85.9986728599867
"chr2" 18060001 18061000 "*" 0 0 50.7044164098986
"chr2" 18142001 18143000 "*" 0 0 64.7058823529412
"chr2" 18349001 18350000 "*" 5.65418314124599e-06 6.4320100371832e-06
67.741935483871
"chr2" 18513001 18514000 "*" 2.73625566649116e-12 7.52483363579137e-12 100
"chr2" 18879001 18880000 "*" 1.13140401492018e-08 1.87801380787728e-08 100
"chr2" 19143001 19144000 "*" 2.76301204138463e-12 7.59415798797596e-12
-72.3076923076923
"chr2" 19874001 19875000 "*" 1.48087875295744e-10 3.22075573380618e-10 100
"chr2" 19927001 19928000 "*" 1.12458486967171e-09 2.15740815043925e-09 100
"chr2" 19928001 19929000 "*" 1.10223382088215e-07 1.59057131040995e-07
-66.6666666666667
"chr2" 19987001 19988000 "*" 3.66692343156672e-10 7.555499911732e-10
-85.9649122807018
"chr2" 20005001 20006000 "*" 1.39779633423487e-07 1.98463037958589e-07 100
"chr2" 20060001 20061000 "*" 5.17553910839297e-09 9.04249646661562e-09 -62.5
"chr2" 20074001 20075000 "*" 1.54630752646767e-11 3.85079502648228e-11 -100
"chr2" 20189001 20190000 "*" 0 0 75.2596830985916
"chr2" 20190001 20191000 "*" 0 0 79.0361445783133
"chr2" 20422001 20423000 "*" 5.55111512312578e-15 2.12516350804551e-14
-75.4385964912281
"chr2" 20442001 20443000 "*" 0 0 60.4026845637584
"chr2" 20619001 20620000 "*" 2.08814465718632e-09 3.86232567960463e-09 -100
"chr2" 20677001 20678000 "*" 0 0 100
"chr2" 20687001 20688000 "*" 0 0 100
"chr2" 20730001 20731000 "*" 3.61932706027801e-14 1.25777907157994e-13 -100
"chr2" 20751001 20752000 "*" 2.06501482580279e-14 7.3890370570066e-14 100
"chr2" 20767001 20768000 "*" 2.68450373042128e-10 5.64465213127486e-10 -100
"chr2" 20814001 20815000 "*" 2.68450373042128e-10 5.64465213127486e-10 -100
"chr2" 20821001 20822000 "*" 4.44089209850063e-16 1.91071758245033e-15
98.8505747126437
"chr2" 20835001 20836000 "*" 0 0 -89.4736842105263
"chr2" 20851001 20852000 "*" 0 0 -97.6744186046512
"chr2" 20866001 20867000 "*" 0 0 63.632587756231
"chr2" 20911001 20912000 "*" 7.00453028912307e-11 1.5937761446451e-10
56.5957446808511
"chr2" 21180001 21181000 "*" 6.92287338566189e-11 1.57605630124247e-10 -100
"chr2" 21181001 21182000 "*" 1.35890187991095e-11 3.40841971363279e-11 -100
"chr2" 21264001 21265000 "*" 9.65729496371637e-10 1.87255912493232e-09 -100
"chr2" 21347001 21348000 "*" 2.34523511721818e-12 6.51654983620872e-12
-62.8484848484848
"chr2" 21499001 21500000 "*" 0 0 -80
"chr2" 21510001 21511000 "*" 9.80763359414993e-10 1.8980032424715e-09 -100
"chr2" 21558001 21559000 "*" 8.51861281514488e-09 1.44501941585257e-08
-66.0377358490566
"chr2" 21654001 21655000 "*" 1.39779633423487e-07 1.98463037958589e-07 -100
"chr2" 23292001 23293000 "*" 7.03992419914812e-12 1.83313429961219e-11 100
"chr2" 23428001 23429000 "*" 2.06501482580279e-14 7.3890370570066e-14 100

"chr2" 23435001 23436000 "*" 8.25450818808804e-13 2.42473843346228e-12 -100
"chr2" 23605001 23606000 "*" 0 0 66.6666666666667
"chr2" 23606001 23607000 "*" 2.05280237253191e-13 6.49882890260434e-13
69.5652173913043
"chr2" 23607001 23608000 "*" 0 0 97.3190348525469
"chr2" 23634001 23635000 "*" 9.65729496371637e-10 1.87255912493232e-09 100
"chr2" 23636001 23637000 "*" 1.10411929599152e-08 1.84128129458287e-08
-73.1707317073171
"chr2" 23679001 23680000 "*" 0 0 -57.2368421052632
"chr2" 23690001 23691000 "*" 7.73825448163734e-14 2.58522599382084e-13
90.983606557377
"chr2" 23724001 23725000 "*" 6.88338275267597e-15 2.60518659957509e-14 100
"chr2" 23726001 23727000 "*" 6.38733017912507e-05 6.1778263544122e-05
59.6491228070175
"chr2" 23734001 23735000 "*" 7.7715611723761e-16 3.26213507634405e-15
60.7142857142857
"chr2" 23756001 23757000 "*" 1.8609336294162e-11 4.59262477681417e-11
-81.8181818181818
"chr2" 23774001 23775000 "*" 2.88779000712225e-11 6.92935809466068e-11 100
"chr2" 23780001 23781000 "*" 4.1153938973304e-06 4.77326970666537e-06
-66.6666666666667
"chr2" 23815001 23816000 "*" 7.33877137948369e-08 1.0835581346709e-07
-63.6363636363636
"chr2" 23816001 23817000 "*" 3.99680288865056e-15 1.5566998858822e-14 100
"chr2" 23827001 23828000 "*" 1.48487888651516e-11 3.70689457767456e-11 100
"chr2" 23842001 23843000 "*" 0 0 -100
"chr2" 23869001 23870000 "*" 4.73234496034536e-09 8.30627300264826e-09 100
"chr2" 23872001 23873000 "*" 8.88178419700125e-15 3.31875892636354e-14
-57.3529411764706
"chr2" 23889001 23890000 "*" 0 0 -69.4444444444444
"chr2" 23920001 23921000 "*" 4.02167188440217e-12 1.08266700497926e-11 100
"chr2" 24037001 24038000 "*" 4.65637306490407e-10 9.46720524046618e-10 -100
"chr2" 24238001 24239000 "*" 1.50266566401047e-06 1.86246459132231e-06
53.6585365853659
"chr2" 24278001 24279000 "*" 1.35036426485158e-12 3.86629547363774e-12 -100
"chr2" 24303001 24304000 "*" 1.67299207820548e-08 2.71377429441055e-08 100
"chr2" 24327001 24328000 "*" 0 0 100
"chr2" 24368001 24369000 "*" 0 0 -55.1724137931034
"chr2" 24422001 24423000 "*" 8.3882012447134e-11 1.88922989503423e-10 100
"chr2" 24443001 24444000 "*" 1.36069056022592e-09 2.58673249032228e-09
58.9285714285714
"chr2" 24623001 24624000 "*" 5.11793074586819e-08 7.74971842202054e-08
-83.3333333333333
"chr2" 24715001 24716000 "*" 0 0 80
"chr2" 25220001 25221000 "*" 0 0 -100
"chr2" 25352001 25353000 "*" 1.20071275144795e-09 2.29605815405974e-09
74.6987951807229
"chr2" 25372001 25373000 "*" 2.08814465718632e-09 3.86232567960463e-09 -100
"chr2" 25397001 25398000 "*" 1.17794662912729e-13 3.83653245040640e-13 100
"chr2" 25400001 25401000 "*" 5.794809077031e-12 1.52911192757929e-11 100
"chr2" 25510001 25511000 "*" 1.1477700645468e-07 1.6519945352344e-07
-51.7857142857143

"chr2" 25528001 25529000 "*" 0 0 -100
"chr2" 25560001 25561000 "*" 5.03264097062583e-13 1.51942428521737e-12 -100
"chr2" 25577001 25578000 "*" 6.24654217240561e-10 1.2462108191303e-09 100
"chr2" 25606001 25607000 "*" 0 0 -59.375
"chr2" 25775001 25776000 "*" 4.73234496034536e-09 8.30627300264826e-09 100
"chr2" 25916001 25917000 "*" 0 0 -100
"chr2" 25921001 25922000 "*" 0 0 -72.2222222222222
"chr2" 25938001 25939000 "*" 9.04570973681018e-10 1.76414389822173e-09 -100
"chr2" 25964001 25965000 "*" 0 0 -100
"chr2" 26107001 26108000 "*" 1.39779633423487e-07 1.98463037958589e-07 100
"chr2" 26249001 26250000 "*" 3.6700841921089e-08 5.65530226072258e-08 100
"chr2" 26655001 26656000 "*" 0 0 100
"chr2" 26708001 26709000 "*" 2.33028818463765e-10 4.93719283454501e-10 100
"chr2" 26718001 26719000 "*" 2.08814465718632e-09 3.86232567960463e-09 -100
"chr2" 26721001 26722000 "*" 1.52794177310511e-09 2.87642564232045e-09 -100
"chr2" 26754001 26755000 "*" 6.52733422867868e-12 1.71146951718423e-11 -100
"chr2" 26792001 26793000 "*" 8.62854232508425e-12 2.22278438222256e-11
64.7058823529412
"chr2" 26793001 26794000 "*" 4.7922689434543e-06 5.50916092610823e-06
-58.6206896551724
"chr2" 26808001 26809000 "*" 9.50128864474209e-13 2.76912949078053e-12
68.1481481481482
"chr2" 26821001 26822000 "*" 2.20268248085631e-13 6.93619989074409e-13
-53.4500253678336
"chr2" 26828001 26829000 "*" 5.88418203051333e-15 2.245355428447e-14 100
"chr2" 26839001 26840000 "*" 3.47255955102099e-08 5.38886714221253e-08
70.1754385964912
"chr2" 26876001 26877000 "*" 1.11022302462516e-16 5.03662826618488e-16 80
"chr2" 26909001 26910000 "*" 3.4735535558994e-09 6.23916237774764e-09
-55.9006211180124
"chr2" 26922001 26923000 "*" 4.79616346638068e-14 1.64380445416596e-13 -100
"chr2" 26981001 26982000 "*" 6.75492994872684e-12 1.76648020100745e-11
-79.2452830188679
"chr2" 26995001 26996000 "*" 3.04467739908887e-09 5.50545751863268e-09
-57.1428571428571
"chr2" 27040001 27041000 "*" 0 0 -100
"chr2" 27128001 27129000 "*" 1.07708972341136e-06 1.36134988793805e-06
64.5161290322581
"chr2" 27194001 27195000 "*" 7.73825448163734e-14 2.58522599382084e-13
57.7263444520082
"chr2" 27294001 27295000 "*" 0 0 -100
"chr2" 27307001 27308000 "*" 1.14124265593318e-11 2.89138062669327e-11 100
"chr2" 27308001 27309000 "*" 0 0 -61.376404494382
"chr2" 27319001 27320000 "*" 0 0 -100
"chr2" 27356001 27357000 "*" 6.83009204749396e-13 2.0251508714739e-12
-54.9678768862991
"chr2" 27461001 27462000 "*" 0 0 -100
"chr2" 27486001 27487000 "*" 0 0 86.0328638497653
"chr2" 27487001 27488000 "*" 0 0 68.1093189964158
"chr2" 27513001 27514000 "*" 2.91028312560115e-11 6.97590507139978e-11 100
"chr2" 27603001 27604000 "*" 0 0 68.7664022029347
"chr2" 27633001 27634000 "*" 1.14833297870653e-07 1.65266213593465e-07

-54.1666666666667
"chr2" 27718001 27719000 "*" 0 0 88.9583333333333
"chr2" 27719001 27720000 "*" 2.38229547200319e-10 5.04200128794648e-10
61.9047619047619
"chr2" 27762001 27763000 "*" 4.44089209850063e-16 1.91071758245033e-15
-52.4444444444444
"chr2" 27944001 27945000 "*" 1.25284005392245e-10 2.75649590272477e-10 -100
"chr2" 27976001 27977000 "*" 7.7715611723761e-15 2.92461699448967e-14
68.3544303797468
"chr2" 28350001 28351000 "*" 3.33817973263706e-10 6.92243563359806e-10 53.125
"chr2" 28559001 28560000 "*" 0 0 -73.0019493177388
"chr2" 28574001 28575000 "*" 5.44961853421455e-11 1.2597410792219e-10
-94.1176470588235
"chr2" 28577001 28578000 "*" 9.04570973681018e-10 1.76414389822173e-09 100
"chr2" 28631001 28632000 "*" 4.44089209850063e-15 1.71914916534614e-14 -100
"chr2" 28660001 28661000 "*" 0 0 -72.2751322751323
"chr2" 28713001 28714000 "*" 2.88710388929303e-10 6.03399264731139e-10 100
"chr2" 28752001 28753000 "*" 1.93723754260411e-07 2.70423069619759e-07
-74.5454545454545
"chr2" 28766001 28767000 "*" 1.20591092667155e-10 2.66075324129686e-10 -100
"chr2" 28783001 28784000 "*" 9.80763359414993e-10 1.8980032424715e-09 100
"chr2" 28806001 28807000 "*" 1.16573417585641e-14 4.29335582900508e-14 100
"chr2" 28821001 28822000 "*" 8.48157100108438e-11 1.90922788903753e-10
77.2727272727273
"chr2" 28906001 28907000 "*" 6.83719311167508e-08 1.01840744031014e-07
66.6666666666667
"chr2" 29196001 29197000 "*" 7.0006535457523e-08 1.03695832512054e-07 -100
"chr2" 29210001 29211000 "*" 2.04612993215392e-11 5.00303915190111e-11
-92.3076923076923
"chr2" 29221001 29222000 "*" 1.55871404672325e-09 2.93170894125605e-09
-79.3193717277487
"chr2" 29226001 29227000 "*" 0 0 100
"chr2" 29228001 29229000 "*" 4.65395529336554e-07 6.17619723339161e-07
65.8653846153846
"chr2" 29267001 29268000 "*" 2.08814465718632e-09 3.86232567960463e-09 -100
"chr2" 29294001 29295000 "*" 3.88022947106492e-13 1.1867462770515e-12 100
"chr2" 29296001 29297000 "*" 2.37587727269783e-14 8.44268514944447e-14 -100
"chr2" 29299001 29300000 "*" 1.65325531042981e-11 4.10438307908254e-11
88.7878787878788
"chr2" 29440001 29441000 "*" 1.93728699748874e-10 4.16570633272037e-10
-81.8181818181818
"chr2" 29455001 29456000 "*" 5.55111512312578e-16 2.36485094870365e-15 -100
"chr2" 29464001 29465000 "*" 8.01666433236647e-09 1.36392368970109e-08 -100
"chr2" 29471001 29472000 "*" 0.000168529599185452 0.000152316702506419 60
"chr2" 29494001 29495000 "*" 6.24654217240561e-10 1.2462108191303e-09 100
"chr2" 29556001 29557000 "*" 1.17794662912729e-13 3.83653245040640e-13 100
"chr2" 29767001 29768000 "*" 4.73234496034536e-09 8.30627300264826e-09 100
"chr2" 29803001 29804000 "*" 0 0 68.5185185185185
"chr2" 29824001 29825000 "*" 4.17289697496326e-06 4.83646732947935e-06
59.2592592592593
"chr2" 29873001 29874000 "*" 8.57092175010621e-13 2.51149198099779e-12 100
"chr2" 30067001 30068000 "*" 1.39779633423487e-07 1.98463037958589e-07 100

"chr2" 30073001 30074000 "*" 2.88779000712225e-11 6.92935809466068e-11 100
"chr2" 30173001 30174000 "*" 2.98024489469029e-05 3.03663320819956e-05 70
"chr2" 30334001 30335000 "*" 3.02856923983796e-08 4.74498074566709e-08 51.6129032258064
"chr2" 30335001 30336000 "*" 3.34128893442198e-08 5.19772864618523e-08 100
"chr2" 30340001 30341000 "*" 1.84163795324821e-12 5.1805817885438e-12 -78.7234042553192
"chr2" 30390001 30391000 "*" 6.66133814775094e-16 2.81595744474255e-15 77.4193548387097
"chr2" 30418001 30419000 "*" 4.03652502123375e-06 4.6892927905593e-06 -68.75
"chr2" 30454001 30455000 "*" 0 0 70.8855309075632
"chr2" 30455001 30456000 "*" 0 0 57.4607927003136
"chr2" 30506001 30507000 "*" 3.52704532247117e-11 8.35581292290093e-11 -100
"chr2" 30518001 30519000 "*" 3.99546075805501e-05 3.98784360862687e-05 -51.8518518518519
"chr2" 30569001 30570000 "*" 9.0072393987839e-13 2.63157725646847e-12 100
"chr2" 30901001 30902000 "*" 2.88779000712225e-11 6.92935809466068e-11 -100
"chr2" 30915001 30916000 "*" 4.75366453437376e-05 4.6905450034535e-05 -54.1666666666667
"chr2" 30932001 30933000 "*" 3.25818809512324e-05 3.29735679006858e-05 -66.6666666666667
"chr2" 30986001 30987000 "*" 3.63445940010365e-11 8.58565209111204e-11 -100
"chr2" 30987001 30988000 "*" 1.25284005392245e-10 2.75649590272477e-10 -100
"chr2" 30989001 30990000 "*" 0 0 -100
"chr2" 31120001 31121000 "*" 1.7402912444453e-09 3.25889715780136e-09 -58.2608695652174
"chr2" 31139001 31140000 "*" 0 0 -100
"chr2" 31188001 31189000 "*" 0 0 100
"chr2" 31200001 31201000 "*" 0 0 100
"chr2" 31242001 31243000 "*" 2.00227675550835e-08 3.20459176617961e-08 100
"chr2" 31289001 31290000 "*" 1.35890187991095e-11 3.40841971363279e-11 -100
"chr2" 31290001 31291000 "*" 0 0 100
"chr2" 31292001 31293000 "*" 3.07531777821168e-14 1.07626052665081e-13 100
"chr2" 31302001 31303000 "*" 2.88657986402541e-15 1.14155613124573e-14 100
"chr2" 31354001 31355000 "*" 1.67299207820548e-08 2.71377429441055e-08 100
"chr2" 31364001 31365000 "*" 3.40125705378114e-10 7.03784073980162e-10 -100
"chr2" 31391001 31392000 "*" 5.58109114479066e-13 1.67566740343777e-12 -62.5
"chr2" 31428001 31429000 "*" 0 0 -100
"chr2" 31432001 31433000 "*" 2.33028818463765e-10 4.93719283454501e-10 100
"chr2" 31502001 31503000 "*" 3.29235250728921e-07 4.46007461671159e-07 72.0930232558139
"chr2" 31525001 31526000 "*" 3.38662431431658e-12 9.21643373322861e-12 96.7741935483871
"chr2" 31588001 31589000 "*" 8.00762828668811e-05 7.62332949242677e-05 52.1739130434783
"chr2" 31719001 31720000 "*" 0 0 56.1702127659574
"chr2" 31793001 31794000 "*" 3.27896043650355e-10 6.80572360112631e-10 80
"chr2" 32048001 32049000 "*" 0 0 93.6170212765958
"chr2" 32675001 32676000 "*" 0 0 -92.8571428571429
"chr2" 33367001 33368000 "*" 7.43849426498855e-15 2.80357739563059e-14 -65.3846153846154
"chr2" 33476001 33477000 "*" 1.83466277617894e-05 1.93097202505878e-05 65

"chr2" 33500001 33501000 "*" 2.22044604925031e-16 9.81641919380259e-16 -100
"chr2" 33664001 33665000 "*" 6.70841160399505e-12 1.75486545176836e-11 -100
"chr2" 34207001 34208000 "*" 0 0 100
"chr2" 34386001 34387000 "*" 1.48087875295744e-10 3.22075573380618e-10 -100
"chr2" 34921001 34922000 "*" 5.95860027985395e-11 1.37063578427242e-10
69.8795180722892
"chr2" 34950001 34951000 "*" 6.4152538836737e-10 1.27477697728417e-09 -100
"chr2" 35725001 35726000 "*" 2.33442109968118e-05 2.41802782933016e-05
71.4285714285714
"chr2" 36013001 36014000 "*" 0 0 100
"chr2" 36704001 36705000 "*" 0 0 -100
"chr2" 36824001 36825000 "*" 3.93756138805657e-11 9.27277605887009e-11
-71.7948717948718
"chr2" 36826001 36827000 "*" 2.15125472990962e-10 4.58126659943874e-10 -100
"chr2" 36966001 36967000 "*" 1.44187550787933e-10 3.14396251668466e-10
-75.4716981132076
"chr2" 37005001 37006000 "*" 1.50623957750895e-12 4.28756717183481e-12 -100
"chr2" 37018001 37019000 "*" 1.87405646556726e-13 5.97285292681429e-13 -100
"chr2" 37034001 37035000 "*" 1.29037891483108e-11 3.24582280286866e-11 100
"chr2" 37194001 37195000 "*" 6.77236045021345e-15 2.56946402364576e-14
-66.6666666666667
"chr2" 37595001 37596000 "*" 1.32615242542933e-06 1.65582211629877e-06
-55.8823529411765
"chr2" 37876001 37877000 "*" 3.6700841921089e-08 5.65530226072258e-08 100
"chr2" 38083001 38084000 "*" 3.12868788432397e-07 4.25120562657041e-07
-58.1395348837209
"chr2" 38108001 38109000 "*" 4.03597338882733e-08 6.19153923694837e-08
58.6206896551724
"chr2" 38157001 38158000 "*" 5.01025332333427e-10 1.01358031135889e-09 100
"chr2" 38333001 38334000 "*" 6.70841160399505e-12 1.75486545176836e-11 100
"chr2" 38605001 38606000 "*" 4.78841410966879e-11 1.11468187156588e-10 100
"chr2" 38883001 38884000 "*" 1.39680973898493e-07 1.98463037958589e-07
-62.0689655172414
"chr2" 38904001 38905000 "*" 8.01666433236647e-09 1.36392368970109e-08 -100
"chr2" 39352001 39353000 "*" 3.88022947106492e-13 1.1867462770515e-12 100
"chr2" 39628001 39629000 "*" 7.0006535457523e-08 1.03695832512054e-07 -100
"chr2" 39731001 39732000 "*" 3.52704532247117e-11 8.35581292290093e-11 100
"chr2" 39964001 39965000 "*" 1.5634673446896e-05 1.66336190218994e-05
-54.5454545454545
"chr2" 40044001 40045000 "*" 8.01666433236647e-09 1.36392368970109e-08 100
"chr2" 40058001 40059000 "*" 4.46609634552342e-06 5.1543008088351e-06
54.0540540540541
"chr2" 40498001 40499000 "*" 8.52140580320793e-12 2.19817259951489e-11 -100
"chr2" 41892001 41893000 "*" 0 0 -100
"chr2" 42012001 42013000 "*" 3.40125705378114e-10 7.03784073980162e-10 -100
"chr2" 42043001 42044000 "*" 1.08274803567454e-08 1.80773809016129e-08 93.75
"chr2" 42181001 42182000 "*" 6.92287338566189e-11 1.57605630124247e-10 100
"chr2" 42182001 42183000 "*" 4.32209823486573e-12 1.15935290479641e-11 -100
"chr2" 42194001 42195000 "*" 0.000126887439664602 0.000117029410540844
-56.5217391304348
"chr2" 42220001 42221000 "*" 1.73226843980245e-09 3.24496642599465e-09
-86.5248226950355

"chr2" 42274001 42275000 "*" 0 0 63.9109053115441
"chr2" 42275001 42276000 "*" 0 0 55.7896108317345
"chr2" 42280001 42281000 "*" 0.000135810753319365 0.000124644681570312 -57.1428571428571
"chr2" 42360001 42361000 "*" 1.01474384450739e-13 3.33229688064048e-13 -57.8651685393258
"chr2" 42370001 42371000 "*" 2.08995487582797e-10 4.46944006972663e-10 100
"chr2" 42391001 42392000 "*" 4.01313771103418e-09 7.11882087237587e-09 100
"chr2" 42540001 42541000 "*" 3.99680288865056e-15 1.5566998858822e-14 100
"chr2" 42795001 42796000 "*" 5.10702591327572e-15 1.96271840288234e-14 91.869918699187
"chr2" 43063001 43064000 "*" 3.6700841921089e-08 5.65530226072258e-08 -100
"chr2" 43083001 43084000 "*" 0 0 60.5263157894737
"chr2" 43148001 43149000 "*" 2.02327044007689e-12 5.65300384792621e-12 -100
"chr2" 43201001 43202000 "*" 3.6700841921089e-08 5.65530226072258e-08 -100
"chr2" 43313001 43314000 "*" 0 0 -59.4627594627595
"chr2" 43388001 43389000 "*" 7.50998221166199e-07 9.69923424620037e-07 -65.7894736842105
"chr2" 43389001 43390000 "*" 0 0 -100
"chr2" 43397001 43398000 "*" 6.98496116502412e-09 1.19804756703193e-08 -61.2903225806452
"chr2" 43405001 43406000 "*" 0.000190673084083492 0.000170833626656188 -54.0983606557377
"chr2" 43426001 43427000 "*" 0 0 100
"chr2" 43448001 43449000 "*" 2.31920554982779e-08 3.68285147128001e-08 -52.1978021978022
"chr2" 43459001 43460000 "*" 8.77964367873574e-13 2.57039492383246e-12 -52.3718093517055
"chr2" 43476001 43477000 "*" 3.01077274222905e-09 5.44733914264292e-09 -96
"chr2" 43495001 43496000 "*" 4.71134242729931e-12 1.257615036806e-11 100
"chr2" 43504001 43505000 "*" 3.95881444958945e-07 5.30253885378003e-07 -74.6031746031746
"chr2" 43806001 43807000 "*" 2.69007038866675e-13 8.38831022628993e-13 100
"chr2" 43863001 43864000 "*" 3.80806497446429e-14 1.31902680029991e-13 58.1395348837209
"chr2" 43864001 43865000 "*" 0 0 71.1363024556576
"chr2" 43875001 43876000 "*" 0.000241617096093272 0.000212792315955059 -51.1111111111111
"chr2" 44100001 44101000 "*" 6.66133814775094e-16 2.81595744474255e-15 -65.8536585365854
"chr2" 44110001 44111000 "*" 6.59550958292954e-09 1.13456194382773e-08 -100
"chr2" 44125001 44126000 "*" 2.02327044007689e-12 5.65300384792621e-12 100
"chr2" 44313001 44314000 "*" 1.11022302462516e-16 5.03662826618488e-16 58.8235294117647
"chr2" 44344001 44345000 "*" 8.58968451922237e-12 2.213554983903e-11 -100
"chr2" 45043001 45044000 "*" 0 0 100
"chr2" 45188001 45189000 "*" 3.95353005888666e-09 7.03928529283733e-09 100
"chr2" 45196001 45197000 "*" 0 0 100
"chr2" 45239001 45240000 "*" 0 0 100
"chr2" 45242001 45243000 "*" 8.25450818808804e-13 2.42473843346228e-12 -100
"chr2" 45344001 45345000 "*" 3.76365605347928e-14 1.3043487970679e-13 100
"chr2" 45380001 45381000 "*" 1.49954150541198e-07 2.12143450071834e-07

-59.0361445783133
"chr2" 45446001 45447000 "*" 0 0 100
"chr2" 45493001 45494000 "*" 2.198229649919e-05 2.28595866462216e-05
-57.8947368421053
"chr2" 45499001 45500000 "*" 9.22717446893984e-09 1.55374184879414e-08
-70.5882352941177
"chr2" 45529001 45530000 "*" 8.29371961108549e-08 1.21617933368927e-07
-78.7671232876712
"chr2" 45533001 45534000 "*" 0 0 100
"chr2" 45981001 45982000 "*" 2.1094237467878e-15 8.47434540879246e-15 -100
"chr2" 46002001 46003000 "*" 0 0 -99.0990990990991
"chr2" 46154001 46155000 "*" 3.40125705378114e-10 7.03784073980162e-10 -100
"chr2" 46260001 46261000 "*" 0.000455208012464681 0.000382689032955743
-51.219512195122
"chr2" 46300001 46301000 "*" 1.67299207820548e-08 2.71377429441055e-08 -100
"chr2" 46301001 46302000 "*" 4.44089209850063e-15 1.71914916534614e-14 -100
"chr2" 46399001 46400000 "*" 1.12567732912794e-10 2.49563754426169e-10
-72.093023255814
"chr2" 46499001 46500000 "*" 5.30231414330729e-12 1.40618384350073e-11
92.8571428571429
"chr2" 46525001 46526000 "*" 0 0 53.0507685142059
"chr2" 46734001 46735000 "*" 7.67056418382595e-11 1.7359448580318e-10 100
"chr2" 46883001 46884000 "*" 8.02691246803988e-14 2.67352274101148e-13 -100
"chr2" 46885001 46886000 "*" 1.12396517547575e-05 1.22201409227529e-05
-53.8461538461538
"chr2" 46895001 46896000 "*" 2.40287094814828e-05 2.48476321258601e-05
-55.5555555555556
"chr2" 46968001 46969000 "*" 3.7274627828765e-12 1.00985250136599e-11 100
"chr2" 47032001 47033000 "*" 5.04263297784746e-13 1.52078941532818e-12 100
"chr2" 47054001 47055000 "*" 8.8118947028093e-08 1.28770353209608e-07
75.5555555555556
"chr2" 47056001 47057000 "*" 0 0 100
"chr2" 47125001 47126000 "*" 4.31432667369336e-13 1.31038221181026e-12
67.6470588235294
"chr2" 47264001 47265000 "*" 1.96644922567657e-11 4.82930612417392e-11 100
"chr2" 47270001 47271000 "*" 2.88779000712225e-11 6.92935809466068e-11 -100
"chr2" 47271001 47272000 "*" 1.10280834797472e-05 1.20060810167597e-05
62.8571428571429
"chr2" 47298001 47299000 "*" 0 0 -100
"chr2" 47335001 47336000 "*" 1.96509475358653e-13 6.23868565133654e-13 -100
"chr2" 47529001 47530000 "*" 0 0 -100
"chr2" 47541001 47542000 "*" 0 0 -100
"chr2" 47550001 47551000 "*" 5.63084545279757e-06 6.4072097045087e-06
-65.1162790697674
"chr2" 47597001 47598000 "*" 0 0 -93.9637826961771
"chr2" 47605001 47606000 "*" 0 0 -100
"chr2" 47617001 47618000 "*" 4.65637306490407e-10 9.46720524046618e-10 100
"chr2" 47618001 47619000 "*" 6.59550958292954e-09 1.13456194382773e-08 100
"chr2" 47619001 47620000 "*" 9.04570973681018e-10 1.76414389822173e-09 -100
"chr2" 47722001 47723000 "*" 3.59919335446435e-08 5.57459557969866e-08
53.5714285714286
"chr2" 47763001 47764000 "*" 3.63445940010365e-11 8.58565209111204e-11 -100

"chr2" 47768001 47769000 "*" 3.7274627828765e-12 1.00985250136599e-11 -100
"chr2" 47796001 47797000 "*" 0 0 67.643385667899
"chr2" 47841001 47842000 "*" 2.08995487582797e-10 4.46944006972663e-10 100
"chr2" 47845001 47846000 "*" 1.17702637125738e-06 1.48016917543092e-06 -91.6666666666667
"chr2" 47883001 47884000 "*" 1.37828859436695e-10 3.01445544267661e-10 100
"chr2" 48271001 48272000 "*" 0 0 97.9591836734694
"chr2" 48333001 48334000 "*" 4.88498130835069e-15 1.8824139250584e-14 58.5585585585586
"chr2" 48648001 48649000 "*" 0 0 -70
"chr2" 48800001 48801000 "*" 1.12376774552558e-12 3.24761818527664e-12 -100
"chr2" 48824001 48825000 "*" 0 0 -100
"chr2" 49162001 49163000 "*" 1.13140401492018e-08 1.87801380787728e-08 100
"chr2" 49392001 49393000 "*" 2.43005615629954e-09 4.45014222280278e-09 100
"chr2" 49999001 50000000 "*" 5.63660229602192e-13 1.68905058935478e-12 -100
"chr2" 50429001 50430000 "*" 2.74846672088103e-08 4.3268595828283e-08 -77.3584905660377
"chr2" 50758001 50759000 "*" 0 0 -89.7196261682243
"chr2" 51545001 51546000 "*" 3.98855990479952e-06 4.63822245103344e-06 62.962962962963
"chr2" 52607001 52608000 "*" 1.13140401492018e-08 1.87801380787728e-08 100
"chr2" 52629001 52630000 "*" 3.34128893442198e-08 5.19772864618523e-08 100
"chr2" 52663001 52664000 "*" 2.08814465718632e-09 3.86232567960463e-09 -100
"chr2" 52800001 52801000 "*" 0 0 -93.8650306748466
"chr2" 54343001 54344000 "*" 0 0 -99.1525423728814
"chr2" 54540001 54541000 "*" 3.77475828372553e-15 1.47379557022071e-14 -51.5262515262515
"chr2" 54558001 54559000 "*" 0 0 -55.2
"chr2" 54658001 54659000 "*" 1.67299207820548e-08 2.71377429441055e-08 100
"chr2" 54685001 54686000 "*" 0 0 100
"chr2" 54785001 54786000 "*" 0 0 -86.8932038834951
"chr2" 54852001 54853000 "*" 5.52331320680111e-08 8.33164275044501e-08 -66.6666666666667
"chr2" 54951001 54952000 "*" 0 0 53.7058152793615
"chr2" 54952001 54953000 "*" 0 0 69.9220779220779
"chr2" 54963001 54964000 "*" 8.7349629751543e-09 1.47505871898511e-08 -100
"chr2" 55027001 55028000 "*" 3.95353005888666e-09 7.03928529283733e-09 -100
"chr2" 55074001 55075000 "*" 3.6700841921089e-08 5.65530226072258e-08 -100
"chr2" 55125001 55126000 "*" 5.51532957082657e-08 8.32110440175318e-08 76
"chr2" 55450001 55451000 "*" 0 0 -51.4765965711461
"chr2" 56051001 56052000 "*" 2.23832063994678e-12 6.23130721053538e-12 -61.0852713178295
"chr2" 56144001 56145000 "*" 2.91882857985648e-05 2.9787858580933e-05 61.7283950617284
"chr2" 56905001 56906000 "*" 1.99840144432528e-12 5.59476580335607e-12 100
"chr2" 57311001 57312000 "*" 2.12670872595799e-05 2.21666904114718e-05 -50.8771929824561
"chr2" 58264001 58265000 "*" 1.09615555864417e-05 1.193832842474e-05 81.8181818181818
"chr2" 58271001 58272000 "*" 2.00227675550835e-08 3.20459176617961e-08 -100
"chr2" 58274001 58275000 "*" 0 0 -76.8421052631579
"chr2" 58985001 58986000 "*" 1.9373391779709e-13 6.15971322898373e-13 100

"chr2" 59293001 59294000 "*" 9.45798994678171e-12 2.42828147239866e-11
50.6329113924051
"chr2" 59477001 59478000 "*" 1.53210777398272e-14 5.5628583449216e-14 100
"chr2" 59757001 59758000 "*" 7.03992419914812e-12 1.83313429961219e-11 100
"chr2" 60142001 60143000 "*" 1.90181204118289e-13 6.05298617539364e-13 -100
"chr2" 60455001 60456000 "*" 0 0 -100
"chr2" 60501001 60502000 "*" 2.33590924381133e-13 7.3418676838825e-13 -100
"chr2" 60525001 60526000 "*" 0 0 -75
"chr2" 60526001 60527000 "*" 6.88338275267597e-15 2.60518659957509e-14 100
"chr2" 60545001 60546000 "*" 1.25147570662065e-06 1.56816530203441e-06
53.3333333333333
"chr2" 60548001 60549000 "*" 6.59550958292954e-09 1.13456194382773e-08 -100
"chr2" 60672001 60673000 "*" 1.93720595120794e-11 4.76309924836636e-11 -100
"chr2" 60681001 60682000 "*" 1.67299207820548e-08 2.71377429441055e-08 -100
"chr2" 60741001 60742000 "*" 0 0 100
"chr2" 60759001 60760000 "*" 8.79185613200661e-13 2.57360896095839e-12
-79.6296296296296
"chr2" 60777001 60778000 "*" 0 0 68.169014084507
"chr2" 60783001 60784000 "*" 1.46549439250521e-14 5.33330076973935e-14 60.9375
"chr2" 60808001 60809000 "*" 0 0 -67.1052631578947
"chr2" 60906001 60907000 "*" 8.7349629751543e-09 1.47505871898511e-08 100
"chr2" 61696001 61697000 "*" 5.48638023900594e-10 1.10374357336421e-09 100
"chr2" 61718001 61719000 "*" 3.4609876564673e-07 4.67482227931052e-07 60
"chr2" 62082001 62083000 "*" 4.18887147191072e-13 1.2739946038722e-12 100
"chr2" 62515001 62516000 "*" 2.69007038866675e-13 8.38831022628993e-13 100
"chr2" 62545001 62546000 "*" 0 0 -100
"chr2" 62763001 62764000 "*" 1.48087875295744e-10 3.22075573380618e-10 -100
"chr2" 62797001 62798000 "*" 0 0 -87.0967741935484
"chr2" 62798001 62799000 "*" 0 0 51.4892070194978
"chr2" 63550001 63551000 "*" 3.33066907387547e-16 1.4495649018245e-15 100
"chr2" 64455001 64456000 "*" 0 0 93.0379746835443
"chr2" 64565001 64566000 "*" 3.4609877241909e-07 4.67482227931052e-07
69.6969696969697
"chr2" 64736001 64737000 "*" 6.59550958292954e-09 1.13456194382773e-08 100
"chr2" 64825001 64826000 "*" 2.12204920302383e-10 4.53435429623062e-10
-70.8333333333333
"chr2" 64870001 64871000 "*" 0 0 -90.2439024390244
"chr2" 65023001 65024000 "*" 9.65338919911574e-13 2.80678910656113e-12 100
"chr2" 65064001 65065000 "*" 1.11022302462516e-15 4.59817122935606e-15 -100
"chr2" 65086001 65087000 "*" 3.33066907387547e-16 1.4495649018245e-15
-68.1701030927835
"chr2" 65205001 65206000 "*" 1.20326970609597e-10 2.65801965649386e-10
-52.3809523809524
"chr2" 65215001 65216000 "*" 0 0 100
"chr2" 65394001 65395000 "*" 3.67355701413885e-10 7.56823317368456e-10
76.9230769230769
"chr2" 65694001 65695000 "*" 0.000896607310246877 0.000717057981883268
53.8461538461538
"chr2" 65841001 65842000 "*" 1.28907528396116e-06 1.61243929530756e-06 -65.625
"chr2" 65881001 65882000 "*" 3.7274627828765e-12 1.00985250136599e-11 -100
"chr2" 65926001 65927000 "*" 2.88710388929303e-10 6.03399264731139e-10 -100
"chr2" 66107001 66108000 "*" 8.80406858527749e-14 2.91456141063452e-13 100

"chr2" 66117001 66118000 "*" 3.33066907387547e-16 1.4495649018245e-15 -100
"chr2" 66196001 66197000 "*" 4.26951363152739e-10 8.72510873516957e-10 100
"chr2" 66781001 66782000 "*" 9.65729496371637e-10 1.87255912493232e-09 -100
"chr2" 66802001 66803000 "*" 0 0 63.3698968726653
"chr2" 66869001 66870000 "*" 2.68450373042128e-10 5.64465213127486e-10 -100
"chr2" 66888001 66889000 "*" 1.24629227171713e-06 1.56195179408462e-06
-69.1192865105909
"chr2" 67049001 67050000 "*" 1.53210777398272e-14 5.5628583449216e-14 100
"chr2" 68107001 68108000 "*" 3.41948691584548e-13 1.05531228559899e-12
-88.0952380952381
"chr2" 68119001 68120000 "*" 2.68450373042128e-10 5.64465213127486e-10 100
"chr2" 68132001 68133000 "*" 6.33271213246189e-12 1.66478362665335e-11
66.6666666666667
"chr2" 68143001 68144000 "*" 4.99900121297969e-12 1.32921056339624e-11 100
"chr2" 68350001 68351000 "*" 8.52140580320793e-12 2.19817259951489e-11 -100
"chr2" 68864001 68865000 "*" 1.67299207820548e-08 2.71377429441055e-08 -100
"chr2" 68965001 68966000 "*" 1.12458486967171e-09 2.15740815043925e-09 -100
"chr2" 69026001 69027000 "*" 3.34128893442198e-08 5.19772864618523e-08 -100
"chr2" 69160001 69161000 "*" 3.6700841921089e-08 5.65530226072258e-08 -100
"chr2" 69352001 69353000 "*" 5.794809077031e-12 1.52911192757929e-11 -100
"chr2" 69384001 69385000 "*" 1.277796757293e-10 2.80903421750896e-10
-77.2727272727273
"chr2" 69675001 69676000 "*" 4.57593962721603e-11 1.06844131750909e-10 -76
"chr2" 69679001 69680000 "*" 4.99020824662466e-11 1.15790792586157e-10 100
"chr2" 69746001 69747000 "*" 2.33028818463765e-10 4.93719283454501e-10 100
"chr2" 69892001 69893000 "*" 2.22044604925031e-16 9.81641919380259e-16 100
"chr2" 69959001 69960000 "*" 8.59312621059871e-14 2.85424634637979e-13
58.5365853658537
"chr2" 70055001 70056000 "*" 1.99190709415653e-06 2.42339922031098e-06
-57.1428571428571
"chr2" 70173001 70174000 "*" 6.60323198964363e-06 7.43143807773833e-06
61.5384615384615
"chr2" 70225001 70226000 "*" 1.54630752646767e-11 3.85079502648228e-11 -100
"chr2" 70239001 70240000 "*" 0 0 -97.7272727272727
"chr2" 70351001 70352000 "*" 0 0 -50.3205128205128
"chr2" 70558001 70559000 "*" 4.79616346638068e-14 1.64380445416596e-13 100
"chr2" 70757001 70758000 "*" 1.13140401492018e-08 1.87801380787728e-08 100
"chr2" 70844001 70845000 "*" 3.34128893442198e-08 5.19772864618523e-08 -100
"chr2" 70900001 70901000 "*" 3.88022947106492e-13 1.1867462770515e-12 100
"chr2" 70959001 70960000 "*" 1.52794177310511e-09 2.87642564232045e-09 -100
"chr2" 71016001 71017000 "*" 0 0 100
"chr2" 71057001 71058000 "*" 2.00227675550835e-08 3.20459176617961e-08 100
"chr2" 71114001 71115000 "*" 0 0 61.8979819239817
"chr2" 71125001 71126000 "*" 2.81168421878419e-11 6.77030902833177e-11
-54.2168674698795
"chr2" 71150001 71151000 "*" 4.82154201264517e-06 5.54097950067908e-06
52.9411764705882
"chr2" 71155001 71156000 "*" 8.80406858527749e-14 2.91456141063452e-13 100
"chr2" 71190001 71191000 "*" 2.62088622582368e-07 3.59779054681028e-07
76.9230769230769
"chr2" 71285001 71286000 "*" 6.93882802971224e-06 7.78842164960861e-06
-57.8947368421053

"chr2" 71294001 71295000 "*" 0 0 100
"chr2" 71317001 71318000 "*" 1.52794177310511e-09 2.87642564232045e-09 100
"chr2" 71694001 71695000 "*" 0 0 -84.7388386343741
"chr2" 71698001 71699000 "*" 3.34128893442198e-08 5.19772864618523e-08 100
"chr2" 71706001 71707000 "*" 4.36206768372749e-07 5.8099159078825e-07 67.3076923076923
"chr2" 71723001 71724000 "*" 3.91259913357089e-10 8.03807012274225e-10 -58.3870967741936
"chr2" 71738001 71739000 "*" 4.44089209850063e-16 1.91071758245033e-15 -97.6744186046512
"chr2" 71748001 71749000 "*" 0 0 100
"chr2" 71749001 71750000 "*" 1.37828859436695e-10 3.01445544267661e-10 -100
"chr2" 71754001 71755000 "*" 8.71525074330748e-14 2.88848349331391e-13 -100
"chr2" 71778001 71779000 "*" 8.65973959207622e-15 3.24130830964561e-14 -74.7368421052632
"chr2" 71781001 71782000 "*" 4.59370052219299e-06 5.2929254827923e-06 -54.1666666666667
"chr2" 71782001 71783000 "*" 1.44040327776374e-07 2.04203102566604e-07 62.962962962963
"chr2" 71800001 71801000 "*" 2.9422908554011e-10 6.14302077875174e-10 -79.5918367346939
"chr2" 71802001 71803000 "*" 5.28975930021991e-05 5.18114848606884e-05 60
"chr2" 71823001 71824000 "*" 1.64793040235978e-06 2.02978163244315e-06 73.134328358209
"chr2" 71827001 71828000 "*" 2.27906582495052e-12 6.34008266313177e-12 -63.5922330097087
"chr2" 71830001 71831000 "*" 0 0 69.6969696969697
"chr2" 71837001 71838000 "*" 2.96451752035409e-12 8.11696741222615e-12 69.8641765704584
"chr2" 71863001 71864000 "*" 4.05394384728197e-10 8.31268267251814e-10 61.7557583659279
"chr2" 71864001 71865000 "*" 2.86893383344422e-05 2.93084297927469e-05 -62.8571428571429
"chr2" 71872001 71873000 "*" 0 0 -100
"chr2" 71898001 71899000 "*" 2.22044604925031e-16 9.81641919380259e-16 -82.3529411764706
"chr2" 71900001 71901000 "*" 1.43019320708593e-05 1.52998435464261e-05 53.3333333333333
"chr2" 71908001 71909000 "*" 1.12458486967171e-09 2.15740815043925e-09 -100
"chr2" 71929001 71930000 "*" 0 0 100
"chr2" 71931001 71932000 "*" 6.92287338566189e-11 1.57605630124247e-10 100
"chr2" 71971001 71972000 "*" 0 0 100
"chr2" 71998001 71999000 "*" 3.6700841921089e-08 5.65530226072258e-08 100
"chr2" 72012001 72013000 "*" 0 0 -68.4210526315789
"chr2" 72017001 72018000 "*" 2.69007038866675e-13 8.38831022628993e-13 100
"chr2" 72043001 72044000 "*" 2.08814465718632e-09 3.86232567960463e-09 -100
"chr2" 72077001 72078000 "*" 0 0 100
"chr2" 72099001 72100000 "*" 1.4432899320127e-15 5.90750815956055e-15 100
"chr2" 72105001 72106000 "*" 1.92946192356658e-09 3.59193011611483e-09 100
"chr2" 72106001 72107000 "*" 1.17409967304027e-08 1.9453419231455e-08 87.2222222222222
"chr2" 72156001 72157000 "*" 1.66065738627097e-06 2.0442198524453e-06 68.75

"chr2" 72217001 72218000 "*" 3.95353005888666e-09 7.03928529283733e-09 100
"chr2" 72238001 72239000 "*" 2.10202097394863e-07 2.92124932133967e-07
-61.7647058823529
"chr2" 72254001 72255000 "*" 4.08209022140227e-11 9.57770690255255e-11 100
"chr2" 72255001 72256000 "*" 3.07568618351794e-07 4.18239045466418e-07
-57.7777777777778
"chr2" 72332001 72333000 "*" 8.99280649946377e-15 3.35888902220833e-14
51.6769230769231
"chr2" 72362001 72363000 "*" 6.8616123893861e-10 1.35884327209647e-09
54.0540540540541
"chr2" 72372001 72373000 "*" 0 0 100
"chr2" 72431001 72432000 "*" 1.25316423904565e-11 3.16165323392645e-11
66.6666666666667
"chr2" 73053001 73054000 "*" 5.39430722312773e-11 1.24737268974759e-10
-88.8157894736842
"chr2" 73090001 73091000 "*" 0 0 81.3559322033898
"chr2" 73100001 73101000 "*" 2.88779000712225e-11 6.92935809466068e-11 -100
"chr2" 73121001 73122000 "*" 0 0 -100
"chr2" 73122001 73123000 "*" 3.54404590119373e-08 5.49458858333929e-08
51.107544141252
"chr2" 73125001 73126000 "*" 0 0 -100
"chr2" 73141001 73142000 "*" 8.53998026739688e-08 1.25047521194141e-07
68.9655172413793
"chr2" 73143001 73144000 "*" 0 0 51.6362328162304
"chr2" 73162001 73163000 "*" 1.05326892207991e-07 1.52413224946622e-07
68.4210526315789
"chr2" 73250001 73251000 "*" 9.08108033215171e-11 2.03407753381119e-10 -100
"chr2" 73292001 73293000 "*" 9.08108033215171e-11 2.03407753381119e-10 -100
"chr2" 73310001 73311000 "*" 4.78284079008517e-13 1.44820017204545e-12
54.5454545454545
"chr2" 73319001 73320000 "*" 3.99680288865056e-14 1.37975970265307e-13 -100
"chr2" 73320001 73321000 "*" 0 0 -100
"chr2" 73376001 73377000 "*" 5.03441732746523e-12 1.3378171545226e-11 100
"chr2" 73503001 73504000 "*" 3.77475828372553e-15 1.47379557022071e-14 -100
"chr2" 73535001 73536000 "*" 5.01025332333427e-10 1.01358031135889e-09 100
"chr2" 73870001 73871000 "*" 4.79616346638068e-14 1.64380445416596e-13 -100
"chr2" 73871001 73872000 "*" 1.17671309529666e-06 1.47988678701818e-06
76.4705882352941
"chr2" 74006001 74007000 "*" 5.31904520428839e-11 1.23064908938601e-10
66.6666666666667
"chr2" 74148001 74149000 "*" 6.08317113348633e-08 9.12516119858089e-08
-55.5555555555556
"chr2" 74227001 74228000 "*" 0 0 100
"chr2" 74231001 74232000 "*" 1.51914614832105e-06 1.88121349342686e-06
-52.3809523809524
"chr2" 74433001 74434000 "*" 3.04324343503026e-11 7.26599935673121e-11 -100
"chr2" 74640001 74641000 "*" 0 0 100
"chr2" 74652001 74653000 "*" 2.22044604925031e-16 9.81641919380259e-16 -100
"chr2" 74668001 74669000 "*" 0 0 -97.6708074534161
"chr2" 74669001 74670000 "*" 0 0 -78.8405797101449
"chr2" 74684001 74685000 "*" 0 0 73.5543859649123
"chr2" 74709001 74710000 "*" 0 0 50.7042253521127

"chr2" 74710001 74711000 "*" 1.45445218091567e-08 2.38427403092739e-08
-57.7777777777778
"chr2" 74751001 74752000 "*" 0 0 89.4736842105263
"chr2" 74781001 74782000 "*" 5.55111512312578e-14 1.88312739567913e-13
-62.1794871794872
"chr2" 74782001 74783000 "*" 0 0 -60.0758910421466
"chr2" 74902001 74903000 "*" 0 0 -91.8032786885246
"chr2" 74997001 74998000 "*" 4.51551152025331e-09 7.9662089949093e-09
79.2452830188679
"chr2" 75060001 75061000 "*" 3.95353005888666e-09 7.03928529283733e-09 100
"chr2" 75095001 75096000 "*" 8.3882012447134e-11 1.88922989503423e-10 100
"chr2" 75146001 75147000 "*" 6.07037193844917e-08 9.10710817455973e-08 60
"chr2" 75186001 75187000 "*" 1.84042225903625e-09 3.43698916505445e-09
-50.6666666666667
"chr2" 75273001 75274000 "*" 0 0 100
"chr2" 75426001 75427000 "*" 0 0 72.6739258702999
"chr2" 75427001 75428000 "*" 0 0 64.6240129707146
"chr2" 75608001 75609000 "*" 0 0 76.271186440678
"chr2" 75787001 75788000 "*" 0 0 52.9104256265757
"chr2" 77231001 77232000 "*" 2.88779000712225e-11 6.92935809466068e-11 100
"chr2" 77638001 77639000 "*" 0 0 -100
"chr2" 78725001 78726000 "*" 1.32149270839488e-05 1.42155827319109e-05
54.5454545454545
"chr2" 79219001 79220000 "*" 7.63138641168837e-09 1.3031858669913e-08
63.302752293578
"chr2" 79740001 79741000 "*" 0 0 78.5244649039232
"chr2" 79824001 79825000 "*" 1.34559030584569e-13 4.35581312463895e-13 -100
"chr2" 80023001 80024000 "*" 1.04604103157158e-11 2.67011144039329e-11
-97.8723404255319
"chr2" 80384001 80385000 "*" 7.0006535457523e-08 1.03695832512054e-07 100
"chr2" 80428001 80429000 "*" 0 0 -100
"chr2" 80531001 80532000 "*" 0 0 62.2696817420435
"chr2" 80558001 80559000 "*" 5.03264097062583e-13 1.51942428521737e-12 100
"chr2" 81098001 81099000 "*" 0 0 100
"chr2" 81123001 81124000 "*" 0 0 -100
"chr2" 82137001 82138000 "*" 1.38590361409285e-10 3.02706178892116e-10 100
"chr2" 82509001 82510000 "*" 0 0 100
"chr2" 82561001 82562000 "*" 0.000499129428774947 0.000416865202314435
-52.3809523809524
"chr2" 82735001 82736000 "*" 3.29605178150949e-07 4.46465082305147e-07
54.8387096774194
"chr2" 83700001 83701000 "*" 1.97619698383278e-14 7.08978387753544e-14 79.6875
"chr2" 84102001 84103000 "*" 1.12458486967171e-09 2.15740815043925e-09 -100
"chr2" 84103001 84104000 "*" 0 0 -100
"chr2" 84743001 84744000 "*" 0 0 77.5510204081633
"chr2" 85049001 85050000 "*" 1.93720595120794e-11 4.76309924836636e-11 100
"chr2" 85061001 85062000 "*" 3.66181607480698e-07 4.92728439089831e-07 75
"chr2" 85104001 85105000 "*" 1.5277158427196e-09 2.87642564232045e-09 100
"chr2" 85153001 85154000 "*" 0 0 -86.1486486486486
"chr2" 85173001 85174000 "*" 1.5277158427196e-09 2.87642564232045e-09 -100
"chr2" 85321001 85322000 "*" 2.68450373042128e-10 5.64465213127486e-10 100
"chr2" 85360001 85361000 "*" 0 0 55.5742805289085

"chr2" 85408001 85409000 "*" 0 0 -100
"chr2" 85417001 85418000 "*" 2.91028312560115e-11 6.97590507139978e-11 -100
"chr2" 85441001 85442000 "*" 2.88710388929303e-10 6.03399264731139e-10 100
"chr2" 85501001 85502000 "*" 4.4144158688586e-05 4.37833412825727e-05 -60
"chr2" 85553001 85554000 "*" 0 0 100
"chr2" 85567001 85568000 "*" 0 0 80
"chr2" 85573001 85574000 "*" 0 0 -83.7837837837838
"chr2" 85655001 85656000 "*" 3.95353005888666e-09 7.03928529283733e-09 100
"chr2" 85657001 85658000 "*" 2.67283972732457e-11 6.45686178157698e-11 100
"chr2" 85668001 85669000 "*" 1.67299207820548e-08 2.71377429441055e-08 100
"chr2" 85747001 85748000 "*" 6.37490060739765e-13 1.89496793587877e-12 -100
"chr2" 85839001 85840000 "*" 0 0 59.7402597402597
"chr2" 85875001 85876000 "*" 8.12538925032413e-12 2.104021133343e-11
51.8518518518519
"chr2" 85941001 85942000 "*" 3.52704532247117e-11 8.35581292290093e-11 100
"chr2" 85946001 85947000 "*" 4.78841410966879e-11 1.11468187156588e-10 -100
"chr2" 85959001 85960000 "*" 5.9929838869266e-13 1.79153028100592e-12
-88.3720930232558
"chr2" 85968001 85969000 "*" 1.85331335833538e-08 2.98924036132437e-08
-59.6774193548387
"chr2" 85971001 85972000 "*" 3.6700841921089e-08 5.65530226072258e-08 100
"chr2" 85978001 85979000 "*" 1.11135878277935e-08 1.85265035113315e-08
83.6065573770492
"chr2" 86004001 86005000 "*" 6.66133814775094e-16 2.81595744474255e-15
-62.3376623376623
"chr2" 86023001 86024000 "*" 0 0 50.9817456665133
"chr2" 86047001 86048000 "*" 1.67299207820548e-08 2.71377429441055e-08 100
"chr2" 86054001 86055000 "*" 1.24865673356567e-11 3.15113900554592e-11
67.7966101694915
"chr2" 86055001 86056000 "*" 0 0 -71.0843373493976
"chr2" 86077001 86078000 "*" 1.88737914186277e-15 7.62011598380321e-15 100
"chr2" 86186001 86187000 "*" 0 0 100
"chr2" 86208001 86209000 "*" 2.02227329104687e-07 2.81656956190631e-07
66.6666666666667
"chr2" 86216001 86217000 "*" 8.01666433236647e-09 1.36392368970109e-08 100
"chr2" 86218001 86219000 "*" 1.39779633423487e-07 1.98463037958589e-07 100
"chr2" 86268001 86269000 "*" 5.12901088933759e-07 6.76904585220232e-07 -68.75
"chr2" 86349001 86350000 "*" 3.52704532247117e-11 8.35581292290093e-11 -100
"chr2" 86460001 86461000 "*" 2.69207922398707e-07 3.69017444205844e-07 -68.75
"chr2" 86463001 86464000 "*" 1.88014123825297e-07 2.62910515528934e-07 75
"chr2" 86527001 86528000 "*" 3.77475828372553e-15 1.47379557022071e-14 -100
"chr2" 86563001 86564000 "*" 0 0 -100
"chr2" 86564001 86565000 "*" 0 0 62.6825518831668
"chr2" 86851001 86852000 "*" 0 0 -73.5294117647059
"chr2" 87034001 87035000 "*" 0 0 -76.7857142857143
"chr2" 87510001 87511000 "*" 4.07890388132159e-11 9.57770690255255e-11
85.7142857142857
"chr2" 87581001 87582000 "*" 1.21988899315362e-07 1.74954588732735e-07
56.4102564102564
"chr2" 88303001 88304000 "*" 0 0 100
"chr2" 88464001 88465000 "*" 5.66647839761458e-11 1.30625016646338e-10
-64.9350649350649

"chr2" 88476001 88477000 "*" 7.0006535457523e-08 1.03695832512054e-07 -100
"chr2" 88486001 88487000 "*" 1.68128622135555e-10 3.63745992546112e-10 71.6981132075472
"chr2" 88582001 88583000 "*" 1.52466927971773e-12 4.33322475981408e-12 100
"chr2" 88650001 88651000 "*" 0 0 57.7632805219012
"chr2" 88806001 88807000 "*" 1.39779633423487e-07 1.98463037958589e-07 100
"chr2" 88966001 88967000 "*" 8.88178419700125e-16 3.70670032207938e-15 100
"chr2" 88991001 88992000 "*" 1.11022302462516e-16 5.03662826618488e-16 100
"chr2" 89127001 89128000 "*" 4.73234496034536e-09 8.30627300264826e-09 100
"chr2" 89132001 89133000 "*" 3.68371999570627e-13 1.13149976091305e-12 100
"chr2" 89160001 89161000 "*" 2.88710388929303e-10 6.03399264731139e-10 100
"chr2" 89331001 89332000 "*" 0 0 100
"chr2" 89835001 89836000 "*" 2.73625566649116e-12 7.52483363579137e-12 100
"chr2" 89845001 89846000 "*" 2.01570315994104e-10 4.32228745881377e-10 -51.1758118701008
"chr2" 89846001 89847000 "*" 2.22044604925031e-16 9.81641919380259e-16 58.3333333333333
"chr2" 89850001 89851000 "*" 4.21884749357559e-15 1.63874445239189e-14 100
"chr2" 89857001 89858000 "*" 5.84878689924295e-09 1.01615053708877e-08 -57.8947368421053
"chr2" 89858001 89859000 "*" 2.31469791711625e-06 2.79034791839104e-06 75
"chr2" 89889001 89890000 "*" 1.14124265593318e-11 2.89138062669327e-11 100
"chr2" 90449001 90450000 "*" 0 0 77.2114644844779
"chr2" 90450001 90451000 "*" 0 0 60.8967760492245
"chr2" 91670001 91671000 "*" 0 0 64.9576388602289
"chr2" 91671001 91672000 "*" 0 0 64.6949347064115
"chr2" 91693001 91694000 "*" 0 0 100
"chr2" 91769001 91770000 "*" 0 0 88.0952380952381
"chr2" 91779001 91780000 "*" 1.52794177310511e-09 2.87642564232045e-09 100
"chr2" 91787001 91788000 "*" 1.74041669964708e-10 3.76021879095037e-10 94.2307692307692
"chr2" 91793001 91794000 "*" 4.18887147191072e-13 1.2739946038722e-12 -100
"chr2" 91812001 91813000 "*" 8.36752109822925e-08 1.22659171705446e-07 -69.7368421052632
"chr2" 91846001 91847000 "*" 2.88710388929303e-10 6.03399264731139e-10 100
"chr2" 91872001 91873000 "*" 0.000133736476111479 0.000122859923752738 -65
"chr2" 91959001 91960000 "*" 3.61932706027801e-14 1.25777907157994e-13 100
"chr2" 92038001 92039000 "*" 2.88779000712225e-11 6.92935809466068e-11 100
"chr2" 92174001 92175000 "*" 4.93227775288929e-08 7.48653958597467e-08 80
"chr2" 92271001 92272000 "*" 1.88737914186277e-15 7.62011598380321e-15 66.6666666666667
"chr2" 92290001 92291000 "*" 7.06507540060297e-06 7.92127836627521e-06 -51.1627906976744
"chr2" 95524001 95525000 "*" 8.44359027141195e-11 1.90129411297129e-10 52.9411764705882
"chr2" 95612001 95613000 "*" 1.11022302462516e-16 5.03662826618488e-16 70.3296703296703
"chr2" 95671001 95672000 "*" 0 0 -100
"chr2" 95684001 95685000 "*" 2.5524971303259e-07 3.50962872069262e-07 60.377358490566
"chr2" 95686001 95687000 "*" 0.000120483966197349 0.000111549020346707 -52.1739130434783

"chr2" 95720001 95721000 "*" 2.75335310107039e-14 9.69727903855348e-14 100
"chr2" 95721001 95722000 "*" 1.59990865498116e-05 1.69994141681406e-05
52.6315789473684
"chr2" 95725001 95726000 "*" 1.02242997401802e-08 1.71241409823705e-08
51.6666666666667
"chr2" 95825001 95826000 "*" 8.852918398361e-13 2.59084695290603e-12
-55.5555555555556
"chr2" 95933001 95934000 "*" 9.35164168325286e-11 2.09181556710247e-10
61.1111111111111
"chr2" 95935001 95936000 "*" 0 0 -100
"chr2" 95943001 95944000 "*" 5.85087533977457e-14 1.9792613012899e-13
61.4466292134831
"chr2" 95948001 95949000 "*" 0 0 -100
"chr2" 95962001 95963000 "*" 0 0 90
"chr2" 95963001 95964000 "*" 0 0 55.7142857142857
"chr2" 95967001 95968000 "*" 4.85861351151584e-11 1.13042390550356e-10
78.2608695652174
"chr2" 95999001 96000000 "*" 7.7715611723761e-16 3.26213507634405e-15
67.2727272727273
"chr2" 96003001 96004000 "*" 2.88710388929303e-10 6.03399264731139e-10 100
"chr2" 96010001 96011000 "*" 1.89581683684992e-12 5.32311367606535e-12 100
"chr2" 96038001 96039000 "*" 3.76476627650391e-13 1.15494788269122e-12
94.8275862068966
"chr2" 96040001 96041000 "*" 0 0 100
"chr2" 96051001 96052000 "*" 2.88102874890228e-13 8.95163570703315e-13
-88.0952380952381
"chr2" 96056001 96057000 "*" 7.43849426498855e-15 2.80357739563059e-14
60.3448275862069
"chr2" 96058001 96059000 "*" 7.67056418382595e-11 1.7359448580318e-10 -100
"chr2" 96067001 96068000 "*" 0 0 59.8130841121495
"chr2" 96069001 96070000 "*" 8.06443922751754e-09 1.37154829365946e-08
77.7777777777778
"chr2" 96196001 96197000 "*" 0 0 -100
"chr2" 96374001 96375000 "*" 4.72585619581967e-05 4.66476159461294e-05
62.0689655172414
"chr2" 96675001 96676000 "*" 0 0 92.8571428571429
"chr2" 96734001 96735000 "*" 3.10862446895044e-15 1.22466437093774e-14 -100
"chr2" 96737001 96738000 "*" 2.1094237467878e-15 8.47434540879246e-15 -100
"chr2" 96766001 96767000 "*" 6.43929354282591e-15 2.44732760419317e-14
-53.3333333333333
"chr2" 96777001 96778000 "*" 3.6892711108294e-13 1.13266255090498e-12 100
"chr2" 96822001 96823000 "*" 1.41109876605761e-06 1.75583535616452e-06 -90
"chr2" 96861001 96862000 "*" 1.25632597777203e-05 1.35579387395931e-05
-53.5714285714286
"chr2" 96987001 96988000 "*" 0 0 82.0244328097731
"chr2" 96994001 96995000 "*" 4.83921813732024e-09 8.48582169090024e-09
-66.6666666666667
"chr2" 97037001 97038000 "*" 0 0 70.7317073170732
"chr2" 97060001 97061000 "*" 0 0 -100
"chr2" 97070001 97071000 "*" 7.0006535457523e-08 1.03695832512054e-07 100
"chr2" 97243001 97244000 "*" 1.42066919561756e-07 2.01552752488489e-07
-59.2592592592593

"chr2" 97455001 97456000 "*" 7.21644966006352e-15 2.72345776413425e-14 100
"chr2" 97483001 97484000 "*" 0 0 95.6521739130435
"chr2" 97548001 97549000 "*" 1.0073053502424e-12 2.92513817101396e-12 63.4146341463415
"chr2" 97563001 97564000 "*" 6.92287338566189e-11 1.57605630124247e-10 -100
"chr2" 97580001 97581000 "*" 2.60353883896336e-07 3.5753307375039e-07 52.0833333333333
"chr2" 97594001 97595000 "*" 2.47094567029649e-11 5.99483916328199e-11 -64.1025641025641
"chr2" 97616001 97617000 "*" 7.81205544697627e-10 1.53677438683776e-09 -77.8606965174129
"chr2" 97635001 97636000 "*" 2.05456412771809e-07 2.85920374137537e-07 62.5
"chr2" 97661001 97662000 "*" 4.01313771103418e-09 7.11882087237587e-09 -100
"chr2" 97667001 97668000 "*" 1.1669829780292e-07 1.6775923726183e-07 -66.6666666666667
"chr2" 98288001 98289000 "*" 1.89425152787859e-05 1.98874471894124e-05 -62.5
"chr2" 98317001 98318000 "*" 0 0 -90.9722222222222
"chr2" 98326001 98327000 "*" 0 0 -67.6923076923077
"chr2" 98356001 98357000 "*" 0 0 100
"chr2" 98423001 98424000 "*" 3.46146957430626e-07 4.67482227931052e-07 -79.5918367346939
"chr2" 98645001 98646000 "*" 3.70931586224366e-07 4.98613126477778e-07 68.8888888888889
"chr2" 98756001 98757000 "*" 0.00131430603037452 0.00102233782225828 53.8461538461538
"chr2" 98801001 98802000 "*" 3.11220239861409e-05 3.16170106772858e-05 63.8297872340426
"chr2" 98802001 98803000 "*" 2.8689339294119e-05 2.93084297927469e-05 -62.8571428571429
"chr2" 98914001 98915000 "*" 0 0 100
"chr2" 98983001 98984000 "*" 0 0 95.2380952380952
"chr2" 98991001 98992000 "*" 0.000923822137060548 0.000737225242626394 60
"chr2" 98999001 99000000 "*" 7.88258347483861e-15 2.96145301916012e-14 -100
"chr2" 99010001 99011000 "*" 6.19715390115516e-12 1.63056411736457e-11 93.75
"chr2" 99015001 99016000 "*" 9.0072393987839e-13 2.63157725646847e-12 -100
"chr2" 99097001 99098000 "*" 0 0 -91.4772727272727
"chr2" 99106001 99107000 "*" 7.05749408145273e-05 6.7761869294483e-05 51.5151515151515
"chr2" 99367001 99368000 "*" 1.70280504141473e-08 2.75934340951488e-08 -65.5172413793103
"chr2" 99515001 99516000 "*" 3.05311331771918e-12 8.34639368772922e-12 100
"chr2" 99537001 99538000 "*" 5.55111512312578e-16 2.36485094870365e-15 100
"chr2" 99630001 99631000 "*" 3.03979064142368e-13 9.42350883309283e-13 100
"chr2" 99643001 99644000 "*" 5.88418203051333e-15 2.245355428447e-14 100
"chr2" 100152001 100153000 "*" 1.17280144961152e-05 1.27141447388175e-05 51.5151515151515
"chr2" 100483001 100484000 "*" 3.6700841921089e-08 5.65530226072258e-08 -100
"chr2" 100632001 100633000 "*" 2.25437823964114e-09 4.15368864286732e-09 -66.6666666666667
"chr2" 100637001 100638000 "*" 7.49830527668749e-08 1.10574283479921e-07 58.0645161290323
"chr2" 100661001 100662000 "*" 0.000105690228288213 9.87313780634119e-05

-50.7936507936508
"chr2" 100814001 100815000 "*" 0 0 71.4285714285714
"chr2" 100816001 100817000 "*" 4.08209022140227e-11 9.57770690255255e-11 -100
"chr2" 100856001 100857000 "*" 0 0 100
"chr2" 100882001 100883000 "*" 4.02167188440217e-12 1.08266700497926e-11 100
"chr2" 100912001 100913000 "*" 8.0269858726556e-07 1.03260151830174e-06
65.3846153846154
"chr2" 100956001 100957000 "*" 3.6892711108294e-13 1.13266255090498e-12 100
"chr2" 101033001 101034000 "*" 0 0 52.2635989487777
"chr2" 101034001 101035000 "*" 0 0 50.3706063720452
"chr2" 101111001 101112000 "*" 0 0 100
"chr2" 101180001 101181000 "*" 0 0 100
"chr2" 101207001 101208000 "*" 1.41964683231244e-07 2.014179973408e-07
-79.1666666666667
"chr2" 101218001 101219000 "*" 3.33066907387547e-16 1.4495649018245e-15 100
"chr2" 101229001 101230000 "*" 1.07784403721167e-05 1.17508652306678e-05
58.8235294117647
"chr2" 101231001 101232000 "*" 0 0 60.6246385193754
"chr2" 101237001 101238000 "*" 1.98294727693504e-06 2.41330824932622e-06
-56.5217391304348
"chr2" 101240001 101241000 "*" 2.1094237467878e-15 8.47434540879246e-15 100
"chr2" 101378001 101379000 "*" 1.12458486967171e-09 2.15740815043925e-09 -100
"chr2" 101408001 101409000 "*" 1.32747716630188e-06 1.65737306735709e-06
65.1162790697674
"chr2" 101434001 101435000 "*" 8.06524846908019e-11 1.8220531017013e-10
-80.8333333333333
"chr2" 101477001 101478000 "*" 1.34088296022128e-11 3.36717371544155e-11
97.0588235294118
"chr2" 101486001 101487000 "*" 1.54630752646767e-11 3.85079502648228e-11 100
"chr2" 101582001 101583000 "*" 0 0 100
"chr2" 101601001 101602000 "*" 6.66133814775094e-16 2.81595744474255e-15 -100
"chr2" 101670001 101671000 "*" 0 0 64.6698016765488
"chr2" 101689001 101690000 "*" 0 0 -100
"chr2" 101835001 101836000 "*" 4.29323243622548e-13 1.30421278296101e-12
-96.0629921259843
"chr2" 101899001 101900000 "*" 3.04324343503026e-11 7.26599935673121e-11 100
"chr2" 101958001 101959000 "*" 9.64766710964682e-10 1.87255912493232e-09
-66.1538461538462
"chr2" 101985001 101986000 "*" 7.22586614987364e-08 1.06824950031765e-07
-85.7142857142857
"chr2" 102003001 102004000 "*" 0 0 50.3046127067015
"chr2" 102018001 102019000 "*" 4.75887536044439e-08 7.23914801846659e-08 75
"chr2" 102231001 102232000 "*" 8.7349629751543e-09 1.47505871898511e-08 -100
"chr2" 102241001 102242000 "*" 1.11022302462516e-16 5.03662826618488e-16
-91.6666666666667
"chr2" 102255001 102256000 "*" 1.74527059471075e-13 5.58544834994603e-13
54.8387096774194
"chr2" 102596001 102597000 "*" 4.53703741243316e-12 1.21404030535971e-11
-87.7777777777778
"chr2" 102679001 102680000 "*" 3.56775498033812e-10 7.35898629809829e-10 100
"chr2" 102719001 102720000 "*" 1.48087875295744e-10 3.22075573380618e-10 100
"chr2" 102730001 102731000 "*" 4.44089209850063e-16 1.91071758245033e-15

66.6666666666667
"chr2" 102847001 102848000 "*" 3.95861121660346e-12 1.0684496262032e-11 -100
"chr2" 102861001 102862000 "*" 1.12634901405784e-10 2.49563754426169e-10 -100
"chr2" 103063001 103064000 "*" 0 0 -100
"chr2" 103236001 103237000 "*" 0 0 65.7894736842105
"chr2" 104486001 104487000 "*" 0.000199851252180694 0.000178453492150885
-54.5454545454545
"chr2" 104591001 104592000 "*" 0 0 -96.6666666666667
"chr2" 104914001 104915000 "*" 0 0 -100
"chr2" 104985001 104986000 "*" 4.1153940362193e-06 4.77326970666537e-06
66.6666666666667
"chr2" 105022001 105023000 "*" 1.51094172169408e-05 1.61080928716407e-05
-64.2857142857143
"chr2" 105046001 105047000 "*" 0 0 -100
"chr2" 105067001 105068000 "*" 2.77555756156289e-15 1.10015415195978e-14 -100
"chr2" 105274001 105275000 "*" 2.96831905299832e-06 3.52052627123658e-06
-68.4210526315789
"chr2" 105319001 105320000 "*" 5.48638023900594e-10 1.10374357336421e-09 100
"chr2" 105361001 105362000 "*" 1.91224813761437e-12 5.36527594469447e-12 100
"chr2" 105362001 105363000 "*" 8.323711719882e-10 1.63257457037061e-09
54.1218637992832
"chr2" 105471001 105472000 "*" 0 0 61.055041282433
"chr2" 105495001 105496000 "*" 1.14124265593318e-11 2.89138062669327e-11 100
"chr2" 105525001 105526000 "*" 9.82012229755469e-07 1.24765566259992e-06
66.6666666666667
"chr2" 105565001 105566000 "*" 7.0006535457523e-08 1.03695832512054e-07 100
"chr2" 105720001 105721000 "*" 2.67283972732457e-11 6.45686178157698e-11 -100
"chr2" 105881001 105882000 "*" 2.69007038866675e-13 8.38831022628993e-13 -100
"chr2" 105883001 105884000 "*" 0 0 100
"chr2" 106129001 106130000 "*" 8.25450818808804e-13 2.42473843346228e-12 100
"chr2" 106134001 106135000 "*" 1.8678632915492e-05 1.96378419686094e-05
53.3333333333333
"chr2" 106163001 106164000 "*" 1.35447209004269e-14 4.94674708073053e-14 100
"chr2" 106179001 106180000 "*" 1.73411632831133e-08 2.80719158400032e-08
-67.1052631578947
"chr2" 106184001 106185000 "*" 0 0 -100
"chr2" 106236001 106237000 "*" 0 0 -81.1881188118812
"chr2" 106258001 106259000 "*" 0 0 65.1376146788991
"chr2" 106268001 106269000 "*" 7.82905962282143e-11 1.77042116309379e-10
51.2820512820513
"chr2" 106269001 106270000 "*" 0 0 100
"chr2" 106286001 106287000 "*" 5.6621374255883e-15 2.16468580166064e-14 -100
"chr2" 106339001 106340000 "*" 1.27594668164477e-09 2.43300659713168e-09
-57.1428571428571
"chr2" 106408001 106409000 "*" 8.3882012447134e-11 1.88922989503423e-10 100
"chr2" 106439001 106440000 "*" 4.08209022140227e-11 9.57770690255255e-11 100
"chr2" 106663001 106664000 "*" 4.86753970463383e-11 1.13145908161915e-10 100
"chr2" 106670001 106671000 "*" 2.67283972732457e-11 6.45686178157698e-11 100
"chr2" 106878001 106879000 "*" 0 0 100
"chr2" 106901001 106902000 "*" 9.65338919911574e-13 2.80678910656113e-12 100
"chr2" 106913001 106914000 "*" 0 0 -100
"chr2" 106975001 106976000 "*" 2.50163223469713e-11 6.06505441673257e-11

-60.6986899563319
"chr2" 107151001 107152000 "*" 3.68371999570627e-13 1.13149976091305e-12 100
"chr2" 107188001 107189000 "*" 8.7349629751543e-09 1.47505871898511e-08 -100
"chr2" 107230001 107231000 "*" 5.01025332333427e-10 1.01358031135889e-09 -100
"chr2" 107433001 107434000 "*" 6.59550958292954e-09 1.13456194382773e-08 100
"chr2" 107557001 107558000 "*" 4.77395900588817e-14 1.63848357364541e-13
75.7575757575758
"chr2" 107616001 107617000 "*" 0 0 100
"chr2" 107921001 107922000 "*" 6.76354444673066e-08 1.00821173534175e-07 68
"chr2" 107981001 107982000 "*" 6.37038500395093e-07 8.3108270131212e-07
71.0526315789474
"chr2" 108152001 108153000 "*" 0 0 51.2396694214876
"chr2" 108153001 108154000 "*" 2.4535928844216e-14 8.69144396197119e-14 100
"chr2" 108154001 108155000 "*" 2.56461518688411e-14 9.06700695401459e-14 -80
"chr2" 108311001 108312000 "*" 5.48638023900594e-10 1.10374357336421e-09 -100
"chr2" 109151001 109152000 "*" 0 0 77.7777777777778
"chr2" 109201001 109202000 "*" 6.50998721596352e-09 1.12407094700425e-08
53.4653465346535
"chr2" 109230001 109231000 "*" 4.44089209850063e-16 1.91071758245033e-15 100
"chr2" 109332001 109333000 "*" 1.98365768255826e-11 4.85612402473442e-11 -100
"chr2" 109534001 109535000 "*" 2.33028818463765e-10 4.93719283454501e-10 100
"chr2" 109554001 109555000 "*" 2.17316653561284e-09 4.01194447718549e-09
-58.0645161290323
"chr2" 109588001 109589000 "*" 0 0 -61.0294117647059
"chr2" 109637001 109638000 "*" 6.3575811282135e-12 1.67100531699095e-11
-93.3774834437086
"chr2" 109649001 109650000 "*" 3.63445940010365e-11 8.58565209111204e-11 -100
"chr2" 109670001 109671000 "*" 1.11022302462516e-16 5.03662826618488e-16 100
"chr2" 109684001 109685000 "*" 4.80306192940372e-06 5.52065480293394e-06 -62
"chr2" 109701001 109702000 "*" 0 0 68.75
"chr2" 109703001 109704000 "*" 1.34559030584569e-13 4.35581312463895e-13 100
"chr2" 109717001 109718000 "*" 9.63829016598083e-12 2.46993429847588e-11 100
"chr2" 109719001 109720000 "*" 0 0 100
"chr2" 109737001 109738000 "*" 0 0 100
"chr2" 109744001 109745000 "*" 0 0 67.9661016949153
"chr2" 109747001 109748000 "*" 8.77520278663724e-13 2.56927515694566e-12
65.3991200502828
"chr2" 109757001 109758000 "*" 2.41741355514469e-05 2.49875818383709e-05
-54.5454545454545
"chr2" 109787001 109788000 "*" 0 0 81.8181818181818
"chr2" 109841001 109842000 "*" 9.65338919911574e-13 2.80678910656113e-12 100
"chr2" 109872001 109873000 "*" 1.38620814826851e-09 2.63314184190467e-09
53.1055900621118
"chr2" 109874001 109875000 "*" 9.71063229826541e-11 2.16781365050398e-10 100
"chr2" 109878001 109879000 "*" 4.88498130835069e-15 1.8824139250584e-14 -100
"chr2" 109884001 109885000 "*" 1.60982338570648e-14 5.83411082284088e-14
79.1666666666667
"chr2" 109887001 109888000 "*" 2.18020225661864e-06 2.63912432489913e-06
-72.5806451612903
"chr2" 109898001 109899000 "*" 0 0 50.4950495049505
"chr2" 109914001 109915000 "*" 0 0 -100
"chr2" 109917001 109918000 "*" 2.22044604925031e-16 9.81641919380259e-16

90.7407407407407
"chr2" 109928001 109929000 "*" 1.4432899320127e-15 5.90750815956055e-15 -100
"chr2" 109953001 109954000 "*" 4.44089209850063e-16 1.91071758245033e-15
65.8536585365854
"chr2" 109958001 109959000 "*" 2.58886467818797e-10 5.46048705194878e-10
51.6666666666667
"chr2" 109963001 109964000 "*" 2.22044604925031e-16 9.81641919380259e-16 100
"chr2" 109994001 109995000 "*" 0 0 100
"chr2" 110053001 110054000 "*" 0 0 -69.8354978354978
"chr2" 110252001 110253000 "*" 2.22044604925031e-16 9.81641919380259e-16 -75
"chr2" 110308001 110309000 "*" 2.02327044007689e-12 5.65300384792621e-12 -100
"chr2" 110468001 110469000 "*" 9.65729496371637e-10 1.87255912493232e-09 -100
"chr2" 111490001 111491000 "*" 0 0 65.4835451742181
"chr2" 111528001 111529000 "*" 6.66133814775094e-16 2.81595744474255e-15 75
"chr2" 111584001 111585000 "*" 0 0 100
"chr2" 111624001 111625000 "*" 1.27870359323268e-06 1.60033571077451e-06
74.5762711864407
"chr2" 111633001 111634000 "*" 1.84067439068514e-09 3.43730608534096e-09
55.0662370211242
"chr2" 111715001 111716000 "*" 9.5812247025151e-14 3.15667542866807e-13 100
"chr2" 111716001 111717000 "*" 1.0769163338864e-14 3.98631098593833e-14 100
"chr2" 111812001 111813000 "*" 6.59550958292954e-09 1.13456194382773e-08 -100
"chr2" 111827001 111828000 "*" 1.17905685215192e-13 3.83653245040640e-13 -100
"chr2" 111875001 111876000 "*" 0 0 -89.6643765300482
"chr2" 111923001 111924000 "*" 1.67299207820548e-08 2.71377429441055e-08 100
"chr2" 111931001 111932000 "*" 5.794809077031e-12 1.52911192757929e-11 -100
"chr2" 111974001 111975000 "*" 2.77282466787376e-06 3.30388613532549e-06
64.2857142857143
"chr2" 111985001 111986000 "*" 1.49699759144362e-07 2.11797927814947e-07
81.8181818181818
"chr2" 112419001 112420000 "*" 1.11022302462516e-16 5.03662826618488e-16 -100
"chr2" 112460001 112461000 "*" 0 0 -79.4444444444444
"chr2" 112642001 112643000 "*" 0 0 100
"chr2" 112655001 112656000 "*" 0 0 61.2903225806452
"chr2" 112657001 112658000 "*" 4.34874358745674e-13 1.32045089065566e-12
52.1517671517672
"chr2" 112837001 112838000 "*" 2.64951482975562e-09 4.82186011375752e-09 -100
"chr2" 112895001 112896000 "*" 0 0 62.2222222222222
"chr2" 112905001 112906000 "*" 6.55384191361463e-10 1.30082815732091e-09
-91.6666666666667
"chr2" 112928001 112929000 "*" 8.88178419700125e-16 3.70670032207938e-15
58.3333333333333
"chr2" 113196001 113197000 "*" 9.04570973681018e-10 1.76414389822173e-09 -100
"chr2" 113238001 113239000 "*" 2.97063718068813e-09 5.37833954875687e-09
-63.8297872340426
"chr2" 113423001 113424000 "*" 6.50701714732804e-13 1.93300005442895e-12
88.8888888888889
"chr2" 113441001 113442000 "*" 2.00227675550835e-08 3.20459176617961e-08 -100
"chr2" 113471001 113472000 "*" 2.3990809339125e-12 6.65441738642456e-12 -100
"chr2" 113478001 113479000 "*" 1.56541446472147e-14 5.67785758911957e-14
54.5454545454545
"chr2" 113925001 113926000 "*" 3.40125705378114e-10 7.03784073980162e-10 100

"chr2" 113964001 113965000 "*" 1.25284005392245e-10 2.75649590272477e-10 100
"chr2" 113967001 113968000 "*" 1.27842547659185e-07 1.82874939148203e-07
55.1724137931034
"chr2" 113997001 113998000 "*" 2.97932325143968e-07 4.05982673792714e-07
-72.5806451612903
"chr2" 113998001 113999000 "*" 0.00012368331194601 0.000114289083653649
-54.3478260869565
"chr2" 114256001 114257000 "*" 0 0 62.9170829170829
"chr2" 114260001 114261000 "*" 0 0 86.512928022362
"chr2" 114454001 114455000 "*" 2.52323501881424e-06 3.02392506768979e-06
-64.5833333333333
"chr2" 114545001 114546000 "*" 0 0 -100
"chr2" 114830001 114831000 "*" 2.33574271035764e-11 5.67937521824243e-11
-71.7948717948718
"chr2" 114880001 114881000 "*" 1.35014666113875e-10 2.96074812132628e-10 90
"chr2" 115116001 115117000 "*" 2.28663760926118e-07 3.16284944821387e-07 -75
"chr2" 116280001 116281000 "*" 2.22044604925031e-16 9.81641919380259e-16 100
"chr2" 116891001 116892000 "*" 0 0 61.8935165306999
"chr2" 117142001 117143000 "*" 2.00227675550835e-08 3.20459176617961e-08 100
"chr2" 117195001 117196000 "*" 3.61932706027801e-14 1.25777907157994e-13 -100
"chr2" 117242001 117243000 "*" 3.6700841921089e-08 5.65530226072258e-08 -100
"chr2" 117512001 117513000 "*" 4.0504599674307e-11 9.52896065926421e-11
-92.5373134328358
"chr2" 117866001 117867000 "*" 4.44089209850063e-15 1.71914916534614e-14 100
"chr2" 118772001 118773000 "*" 3.51635214279611e-08 5.45428915898085e-08
50.5376344086022
"chr2" 119067001 119068000 "*" 0 0 92.9138321995465
"chr2" 119152001 119153000 "*" 0 0 98.3870967741936
"chr2" 119234001 119235000 "*" 4.08209022140227e-11 9.57770690255255e-11 -100
"chr2" 119241001 119242000 "*" 5.48638023900594e-10 1.10374357336421e-09 100
"chr2" 119319001 119320000 "*" 5.1461945815845e-11 1.1922448258169e-10
-58.4905660377358
"chr2" 119332001 119333000 "*" 0.000354677012256444 0.00030353099993751
51.7241379310345
"chr2" 119390001 119391000 "*" 1.09345976717634e-10 2.42792541973934e-10
-55.5555555555556
"chr2" 119527001 119528000 "*" 1.12458486967171e-09 2.15740815043925e-09 -100
"chr2" 119543001 119544000 "*" 1.67299207820548e-08 2.71377429441055e-08 100
"chr2" 119573001 119574000 "*" 1.90181204118289e-13 6.05298617539364e-13 100
"chr2" 119724001 119725000 "*" 1.80023693285847e-07 2.52295437591977e-07
-64.1025641025641
"chr2" 119751001 119752000 "*" 0.000137599100368035 0.000126143016114881
-57.6923076923077
"chr2" 119752001 119753000 "*" 0 0 -100
"chr2" 119769001 119770000 "*" 0 0 55.7366875548694
"chr2" 119817001 119818000 "*" 3.75143965891667e-08 5.7741567259096e-08 -62.5
"chr2" 119823001 119824000 "*" 2.68450373042128e-10 5.64465213127486e-10 -100
"chr2" 119887001 119888000 "*" 1.17683640610267e-14 4.33290554866129e-14
-54.2857142857143
"chr2" 119892001 119893000 "*" 9.9031893796564e-14 3.25554945402539e-13 -75
"chr2" 119893001 119894000 "*" 0 0 100
"chr2" 119932001 119933000 "*" 0 0 -91.2621359223301

"chr2" 119934001 119935000 "*" 4.85167461761193e-14 1.6617373494005e-13 100
"chr2" 120161001 120162000 "*" 1.67299207820548e-08 2.71377429441055e-08 -100
"chr2" 120189001 120190000 "*" 0 0 67.070868945869
"chr2" 120192001 120193000 "*" 9.04570973681018e-10 1.76414389822173e-09 -100
"chr2" 120193001 120194000 "*" 4.38219785847682e-06 5.06174739125268e-06 -64.1025641025641
"chr2" 120200001 120201000 "*" 0 0 -100
"chr2" 120202001 120203000 "*" 2.64951482975562e-09 4.82186011375752e-09 100
"chr2" 120248001 120249000 "*" 1.5277158427196e-09 2.87642564232045e-09 -100
"chr2" 120301001 120302000 "*" 0 0 62.2710622710623
"chr2" 120429001 120430000 "*" 4.07485156728171e-12 1.09641715304993e-11 58.3333333333333
"chr2" 120432001 120433000 "*" 8.01666433236647e-09 1.36392368970109e-08 -100
"chr2" 120435001 120436000 "*" 0 0 85.8090185676393
"chr2" 120450001 120451000 "*" 3.14570388848523e-07 4.27293435984224e-07 -69.7674418604651
"chr2" 120455001 120456000 "*" 2.33028818463765e-10 4.93719283454501e-10 100
"chr2" 120458001 120459000 "*" 1.29037891483108e-11 3.24582280286866e-11 -100
"chr2" 120468001 120469000 "*" 3.15780734894133e-11 7.52724745048982e-11 -73.0055658627087
"chr2" 120913001 120914000 "*" 2.15706130735072e-07 2.9931023448867e-07 71.4285714285714
"chr2" 120957001 120958000 "*" 1.22888268672128e-07 1.76183855749412e-07 59.4594594594595
"chr2" 120962001 120963000 "*" 0 0 -63.1578947368421
"chr2" 120974001 120975000 "*" 0 0 -50.204765450484
"chr2" 120991001 120992000 "*" 3.05311331771918e-12 8.34639368772922e-12 100
"chr2" 121025001 121026000 "*" 0 0 -100
"chr2" 121064001 121065000 "*" 2.34257058195908e-14 8.33889706074077e-14 100
"chr2" 121081001 121082000 "*" 7.88258347483861e-15 2.96145301916012e-14 50.7765314926661
"chr2" 121103001 121104000 "*" 0 0 62.8450366949241
"chr2" 121105001 121106000 "*" 0 0 100
"chr2" 121115001 121116000 "*" 0 0 100
"chr2" 121138001 121139000 "*" 5.07371922253697e-14 1.73287898516782e-13 100
"chr2" 121155001 121156000 "*" 2.42033137975994e-09 4.44218489843109e-09 -53.030303030303
"chr2" 121157001 121158000 "*" 4.9960036108132e-13 1.51000283831609e-12 -57.0246538331645
"chr2" 121161001 121162000 "*" 2.71893618730701e-13 8.46692454027087e-13 -100
"chr2" 121264001 121265000 "*" 6.66133814775094e-15 2.52791665956799e-14 -100
"chr2" 121268001 121269000 "*" 0 0 -95.8333333333333
"chr2" 121284001 121285000 "*" 2.47035725209344e-12 6.83870005605395e-12 62.4544033350703
"chr2" 121285001 121286000 "*" 0 0 68.7054026503568
"chr2" 121286001 121287000 "*" 1.11022302462516e-16 5.03662826618488e-16 100
"chr2" 121293001 121294000 "*" 2.79440914852103e-11 6.73046092948135e-11 -100
"chr2" 121300001 121301000 "*" 1.69482179817049e-05 1.79420595392413e-05 66.6666666666667
"chr2" 121353001 121354000 "*" 9.57597811490629e-07 1.21811245340392e-06 64.7058823529412
"chr2" 121368001 121369000 "*" 6.92287338566189e-11 1.57605630124247e-10 100

"chr2" 121373001 121374000 "*" 0 0 -100
"chr2" 121427001 121428000 "*" 0 0 -55.5985915492958
"chr2" 121437001 121438000 "*" 1.11022302462516e-16 5.03662826618488e-16
-73.0769230769231
"chr2" 121440001 121441000 "*" 0 0 100
"chr2" 121490001 121491000 "*" 1.11022302462516e-16 5.03662826618488e-16 100
"chr2" 121494001 121495000 "*" 0 0 61.5023474178404
"chr2" 121511001 121512000 "*" 4.46309655899313e-14 1.53545649559889e-13
75.2293577981651
"chr2" 121520001 121521000 "*" 2.43005615629954e-09 4.45014222280278e-09 100
"chr2" 121531001 121532000 "*" 2.32813768263895e-12 6.47055136541934e-12
-57.5757575757576
"chr2" 121559001 121560000 "*" 2.22385682313586e-07 3.08045655982239e-07
-79.3650793650794
"chr2" 121573001 121574000 "*" 2.62206345547078e-06 3.13437095470483e-06
59.2592592592593
"chr2" 121583001 121584000 "*" 0 0 100
"chr2" 121620001 121621000 "*" 0 0 -96.2473621433159
"chr2" 121662001 121663000 "*" 8.88178419700125e-16 3.70670032207938e-15 100
"chr2" 121672001 121673000 "*" 4.59603466396175e-11 1.07289303403951e-10
-52.4475524475524
"chr2" 121680001 121681000 "*" 4.73234496034536e-09 8.30627300264826e-09 -100
"chr2" 121699001 121700000 "*" 7.7715611723761e-16 3.26213507634405e-15 100
"chr2" 121749001 121750000 "*" 0 0 100
"chr2" 121750001 121751000 "*" 6.14967021750346e-09 1.065052727662e-08
-64.7058823529412
"chr2" 121761001 121762000 "*" 0 0 100
"chr2" 121773001 121774000 "*" 8.21130941019987e-11 1.85349390452663e-10 -75
"chr2" 121779001 121780000 "*" 9.34677604758072e-07 1.19141004630311e-06
58.0645161290323
"chr2" 121813001 121814000 "*" 6.24654217240561e-10 1.2462108191303e-09 100
"chr2" 121828001 121829000 "*" 1.12634901405784e-10 2.49563754426169e-10 100
"chr2" 121942001 121943000 "*" 0 0 100
"chr2" 121950001 121951000 "*" 5.00231689404274e-09 8.75507251377598e-09
-57.1428571428571
"chr2" 121975001 121976000 "*" 0 0 100
"chr2" 121981001 121982000 "*" 0.000320710195636176 0.000276619581787338
-52.3809523809524
"chr2" 122040001 122041000 "*" 2.77555756156289e-15 1.10015415195978e-14 -100
"chr2" 122109001 122110000 "*" 2.22044604925031e-16 9.81641919380259e-16
-82.4283559577677
"chr2" 122498001 122499000 "*" 1.46890570040803e-07 2.07982206673328e-07
68.2432432432432
"chr2" 122512001 122513000 "*" 1.23367982496347e-12 3.5502486884306e-12 92.5
"chr2" 122627001 122628000 "*" 0 0 100
"chr2" 122657001 122658000 "*" 1.20591092667155e-10 2.66075324129686e-10 100
"chr2" 122710001 122711000 "*" 0 0 -100
"chr2" 122835001 122836000 "*" 2.64193625865161e-08 4.16780459116382e-08
-73.9130434782609
"chr2" 122997001 122998000 "*" 4.08209022140227e-11 9.57770690255255e-11 -100
"chr2" 124195001 124196000 "*" 3.34128893442198e-08 5.19772864618523e-08 -100
"chr2" 124386001 124387000 "*" 3.01154941317883e-07 4.10059151293247e-07

71.0843373493976
"chr2" 124461001 124462000 "*" 8.24434965818188e-06 9.14596506410368e-06
75.4545454545455
"chr2" 125119001 125120000 "*" 0 0 83.3333333333333
"chr2" 125187001 125188000 "*" 3.04324343503026e-11 7.26599935673121e-11 100
"chr2" 126143001 126144000 "*" 1.01562092069685e-11 2.59715130444356e-11 97.5
"chr2" 126166001 126167000 "*" 0 0 100
"chr2" 126478001 126479000 "*" 0 0 -100
"chr2" 126722001 126723000 "*" 0 0 80.2696078431373
"chr2" 126932001 126933000 "*" 6.16805551856281e-07 8.06313781087357e-07
73.1707317073171
"chr2" 127130001 127131000 "*" 9.5812247025151e-14 3.15667542866807e-13 100
"chr2" 127235001 127236000 "*" 0 0 -100
"chr2" 127395001 127396000 "*" 6.80697527566387e-07 8.84457105404304e-07
74.2574257425743
"chr2" 127426001 127427000 "*" 6.04140587556756e-08 9.06560860575318e-08
-51.8518518518519
"chr2" 127478001 127479000 "*" 8.7349629751543e-09 1.47505871898511e-08 100
"chr2" 127506001 127507000 "*" 5.9313010614126e-08 8.91097063444377e-08 75
"chr2" 127534001 127535000 "*" 4.78696075001395e-08 7.27908295654722e-08
77.9661016949153
"chr2" 127535001 127536000 "*" 0 0 90.3225806451613
"chr2" 127592001 127593000 "*" 9.99200722162641e-15 3.71463121338097e-14
-82.4561403508772
"chr2" 127593001 127594000 "*" 0 0 -100
"chr2" 127596001 127597000 "*" 7.03992419914812e-12 1.83313429961219e-11 -100
"chr2" 127663001 127664000 "*" 7.0006535457523e-08 1.03695832512054e-07 100
"chr2" 127729001 127730000 "*" 0 0 -64.3795620437956
"chr2" 127730001 127731000 "*" 0 0 -72.3404255319149
"chr2" 127799001 127800000 "*" 9.80763359414993e-10 1.8980032424715e-09 100
"chr2" 127822001 127823000 "*" 0 0 -80.8612440191388
"chr2" 127831001 127832000 "*" 6.92287338566189e-11 1.57605630124247e-10 -100
"chr2" 127949001 127950000 "*" 2.75837855657768e-05 2.82530803747995e-05
-66.6666666666667
"chr2" 127977001 127978000 "*" 0 0 67.772529368274
"chr2" 127989001 127990000 "*" 1.83952852950142e-12 5.17499701329218e-12 100
"chr2" 128005001 128006000 "*" 9.06552610757672e-11 2.03407753381119e-10
60.8695652173913
"chr2" 128050001 128051000 "*" 2.77278200400133e-12 7.62000631602555e-12
-79.6610169491525
"chr2" 128070001 128071000 "*" 1.7159362819541e-10 3.7093460996491e-10
93.3333333333333
"chr2" 128159001 128160000 "*" 0 0 -97.5409836065574
"chr2" 128166001 128167000 "*" 1.73105973999554e-12 4.88600205806858e-12
-56.9620253164557
"chr2" 128174001 128175000 "*" 8.55981951985996e-14 2.84329661419124e-13
97.3684210526316
"chr2" 128318001 128319000 "*" 3.33066907387547e-16 1.4495649018245e-15 -100
"chr2" 128324001 128325000 "*" 0 0 -77.5423728813559
"chr2" 128327001 128328000 "*" 1.43636699576621e-05 1.5361751244027e-05
-64.367816091954
"chr2" 128340001 128341000 "*" 3.05311331771918e-12 8.34639368772922e-12 -100

"chr2" 128349001 128350000 "*" 2.22044604925031e-16 9.81641919380259e-16
-96.4912280701754
"chr2" 128377001 128378000 "*" 1.17461596005342e-13 3.8279067575877e-13 -100
"chr2" 128407001 128408000 "*" 0 0 -62.0675653727465
"chr2" 128433001 128434000 "*" 0 0 67.5675675675676
"chr2" 128569001 128570000 "*" 4.2601588923219e-11 9.97866148461769e-11
-53.0739045127534
"chr2" 128757001 128758000 "*" 6.59550958292954e-09 1.13456194382773e-08 100
"chr2" 128796001 128797000 "*" 9.18738640720562e-10 1.79018370564662e-09 56
"chr2" 128824001 128825000 "*" 1.52794177310511e-09 2.87642564232045e-09 -100
"chr2" 128847001 128848000 "*" 1.11022302462516e-16 5.03662826618488e-16 -100
"chr2" 128966001 128967000 "*" 2.77555756156289e-15 1.10015415195978e-14 -100
"chr2" 128993001 128994000 "*" 1.05181275533717e-06 1.33139864743141e-06
69.6969696969697
"chr2" 128996001 128997000 "*" 2.32377783682125e-10 4.93204456530415e-10
-60.6060606060606
"chr2" 129075001 129076000 "*" 0 0 54.1728114955568
"chr2" 129079001 129080000 "*" 0 0 54.18335645681
"chr2" 129124001 129125000 "*" 1.13328566130333e-05 1.23144129895198e-05
69.2307692307692
"chr2" 129132001 129133000 "*" 1.91489268885903e-10 4.11999548210973e-10
-76.7441860465116
"chr2" 129138001 129139000 "*" 0 0 -100
"chr2" 129142001 129143000 "*" 0.000358795706608572 0.000306860535716085
-50.8196721311475
"chr2" 129170001 129171000 "*" 0 0 100
"chr2" 129194001 129195000 "*" 3.86522494011388e-08 5.94227458478777e-08
-55.2991452991453
"chr2" 129199001 129200000 "*" 1.07365605295229e-09 2.068317352042e-09
50.6493506493507
"chr2" 129210001 129211000 "*" 5.08126873910442e-12 1.34992308582054e-11
85.7142857142857
"chr2" 129214001 129215000 "*" 1.13140401492018e-08 1.87801380787728e-08 100
"chr2" 129229001 129230000 "*" 4.56037858279634e-05 4.51247837423645e-05
69.2307692307692
"chr2" 129230001 129231000 "*" 0 0 -100
"chr2" 129278001 129279000 "*" 0 0 100
"chr2" 129279001 129280000 "*" 6.4399653571745e-05 6.22599140150143e-05
61.5384615384615
"chr2" 129309001 129310000 "*" 1.60123908825582e-05 1.7012032608364e-05
53.5714285714286
"chr2" 129363001 129364000 "*" 0 0 74.6212121212121
"chr2" 129371001 129372000 "*" 4.01313771103418e-09 7.11882087237587e-09 100
"chr2" 129377001 129378000 "*" 0.000271909868466036 0.000237433097161646
51.1363636363636
"chr2" 129389001 129390000 "*" 7.14213854724477e-09 1.2237982715147e-08
-61.3065326633166
"chr2" 129390001 129391000 "*" 0 0 100
"chr2" 129396001 129397000 "*" 1.19904086659517e-14 4.40901388691796e-14
-79.1666666666667
"chr2" 129401001 129402000 "*" 9.88421338687928e-06 1.0841293662501e-05
64.7058823529412

"chr2" 129403001 129404000 "*" 0 0 91.6666666666667
"chr2" 129416001 129417000 "*" 3.90831811358794e-12 1.05610567215048e-11 100
"chr2" 129424001 129425000 "*" 7.82712499494131e-05 7.46478152805681e-05
-56.5217391304348
"chr2" 129425001 129426000 "*" 6.66133814775094e-16 2.81595744474255e-15 75
"chr2" 129428001 129429000 "*" 5.55111512312578e-16 2.36485094870365e-15 100
"chr2" 129439001 129440000 "*" 6.21414031343193e-12 1.63459166469347e-11
61.1111111111111
"chr2" 129440001 129441000 "*" 3.574918139293e-14 1.24374547149444e-13
74.468085106383
"chr2" 129449001 129450000 "*" 6.63085901209115e-06 7.46031544242657e-06
67.4418604651163
"chr2" 129513001 129514000 "*" 2.0042856263558e-10 4.29868894112068e-10
-97.0588235294118
"chr2" 129527001 129528000 "*" 0 0 100
"chr2" 129528001 129529000 "*" 3.28626015289046e-14 1.14719512441843e-13
97.6377952755905
"chr2" 129531001 129532000 "*" 0 0 84.7222222222222
"chr2" 129539001 129540000 "*" 6.10963346581173e-09 1.0587123964306e-08
64.8648648648649
"chr2" 129541001 129542000 "*" 0 0 -70.1086956521739
"chr2" 129546001 129547000 "*" 8.25117751901416e-13 2.42473843346228e-12
-78.8461538461538
"chr2" 129548001 129549000 "*" 1.50657264441634e-13 4.85113349213551e-13
82.1428571428571
"chr2" 129552001 129553000 "*" 1.92946192356658e-09 3.59193011611483e-09 100
"chr2" 129569001 129570000 "*" 1.0547118733939e-14 3.91001935116974e-14
-77.5510204081633
"chr2" 129625001 129626000 "*" 7.16924741794855e-10 1.41628603646556e-09
-66.6666666666667
"chr2" 129706001 129707000 "*" 0.00020976332305167 0.000186660769941092
52.3809523809524
"chr2" 129712001 129713000 "*" 5.568333683037e-08 8.39545433133528e-08
79.1666666666667
"chr2" 129763001 129764000 "*" 1.34336985979644e-14 4.91028542731765e-14
70.5882352941177
"chr2" 129767001 129768000 "*" 6.6481443683486e-08 9.92215375307238e-08
-67.5675675675676
"chr2" 129774001 129775000 "*" 0 0 -100
"chr2" 129808001 129809000 "*" 8.7349629751543e-09 1.47505871898511e-08 100
"chr2" 129845001 129846000 "*" 2.64951482975562e-09 4.82186011375752e-09 100
"chr2" 129901001 129902000 "*" 0 0 73.6842105263158
"chr2" 129948001 129949000 "*" 3.34128893442198e-08 5.19772864618523e-08 100
"chr2" 129951001 129952000 "*" 6.70841160399505e-12 1.75486545176836e-11 100
"chr2" 130057001 130058000 "*" 2.09438022480413e-11 5.11561782012635e-11
-92.156862745098
"chr2" 130091001 130092000 "*" 3.99680288865056e-15 1.5566998858822e-14 -100
"chr2" 130188001 130189000 "*" 1.89581683684992e-12 5.32311367606535e-12 100
"chr2" 130215001 130216000 "*" 6.44564592999775e-06 7.26663863268796e-06
66.6666666666667
"chr2" 130240001 130241000 "*" 7.88258347483861e-15 2.96145301916012e-14
81.8181818181818

"chr2" 130248001 130249000 "*" 4.14335232790108e-13 1.26153063645578e-12 -100
"chr2" 130308001 130309000 "*" 2.69007038866675e-13 8.38831022628993e-13 100
"chr2" 130385001 130386000 "*" 1.16573417585641e-14 4.29335582900508e-14 100
"chr2" 130393001 130394000 "*" 3.04324343503026e-11 7.26599935673121e-11 100
"chr2" 130403001 130404000 "*" 1.78079773149875e-13 5.69259378953817e-13
-57.1428571428571
"chr2" 130465001 130466000 "*" 8.01666433236647e-09 1.36392368970109e-08 100
"chr2" 130478001 130479000 "*" 1.39779633423487e-07 1.98463037958589e-07 100
"chr2" 130489001 130490000 "*" 7.03992419914812e-12 1.83313429961219e-11 100
"chr2" 130492001 130493000 "*" 4.52467396883094e-10 9.22191990618632e-10
-50.9803921568627
"chr2" 130496001 130497000 "*" 4.59076887615595e-10 9.34931929815876e-10
55.5555555555556
"chr2" 130500001 130501000 "*" 6.88338275267597e-15 2.60518659957509e-14 -100
"chr2" 130519001 130520000 "*" 0 0 65.0793650793651
"chr2" 130553001 130554000 "*" 1.01258840057294e-06 1.28414601227441e-06 -56.25
"chr2" 130564001 130565000 "*" 1.66457907346818e-07 2.34196590452475e-07
68.4210526315789
"chr2" 130570001 130571000 "*" 3.56775498033812e-10 7.35898629809829e-10 100
"chr2" 130590001 130591000 "*" 2.01809627897731e-08 3.22829990076203e-08
82.7586206896552
"chr2" 130640001 130641000 "*" 4.01313771103418e-09 7.11882087237587e-09 100
"chr2" 130649001 130650000 "*" 5.65303359678637e-11 1.30329493286827e-10
86.3636363636364
"chr2" 130713001 130714000 "*" 1.11022302462516e-16 5.03662826618488e-16 -100
"chr2" 130810001 130811000 "*" 2.43005615629954e-09 4.45014222280278e-09 -100
"chr2" 130886001 130887000 "*" 2.1467494448757e-11 5.23862246311679e-11
52.7777777777778
"chr2" 130895001 130896000 "*" 0 0 -100
"chr2" 130913001 130914000 "*" 0 0 -100
"chr2" 131094001 131095000 "*" 9.41876798776775e-10 1.83329867152656e-09 -62.5
"chr2" 131114001 131115000 "*" 0 0 53.4841734122395
"chr2" 131117001 131118000 "*" 2.43005615629954e-09 4.45014222280278e-09 -100
"chr2" 131138001 131139000 "*" 2.77555756156289e-15 1.10015415195978e-14
66.6666666666667
"chr2" 131487001 131488000 "*" 1.00919272938427e-13 3.31458972974436e-13
-96.1904761904762
"chr2" 131513001 131514000 "*" 0 0 82.2067185287687
"chr2" 131518001 131519000 "*" 2.22044604925031e-16 9.81641919380259e-16 -100
"chr2" 131546001 131547000 "*" 6.70841160399505e-12 1.75486545176836e-11 100
"chr2" 131580001 131581000 "*" 5.07806923333298e-06 5.81678794270412e-06
50.197628458498
"chr2" 131733001 131734000 "*" 2.32565644520122e-09 4.2771758058095e-09
72.972972972973
"chr2" 131769001 131770000 "*" 1.78688110125114e-05 1.88505925150618e-05 65.625
"chr2" 131785001 131786000 "*" 1.25284005392245e-10 2.75649590272477e-10 100
"chr2" 131915001 131916000 "*" 0 0 100
"chr2" 131976001 131977000 "*" 1.55328039319613e-09 2.92201622641589e-09
-50.9834368530021
"chr2" 132146001 132147000 "*" 8.69011131499065e-07 1.11229659818976e-06
-57.6923076923077
"chr2" 132293001 132294000 "*" 6.66133814775094e-16 2.81595744474255e-15 100

"chr2" 133039001 133040000 "*" 1.82323758313174e-08 2.94312091461566e-08
53.6492374727669
"chr2" 133043001 133044000 "*" 1.11022302462516e-16 5.03662826618488e-16
64.7058823529412
"chr2" 133060001 133061000 "*" 2.22044604925031e-16 9.81641919380259e-16 100
"chr2" 133105001 133106000 "*" 5.794809077031e-12 1.52911192757929e-11 -100
"chr2" 133363001 133364000 "*" 4.73234496034536e-09 8.30627300264826e-09 -100
"chr2" 133427001 133428000 "*" 0 0 64.16794711171
"chr2" 133451001 133452000 "*" 1.14352971536391e-14 4.21921674335999e-14 -100
"chr2" 133694001 133695000 "*" 1.12458486967171e-09 2.15740815043925e-09 100
"chr2" 134024001 134025000 "*" 0 0 51.8334339228386
"chr2" 134347001 134348000 "*" 9.65729496371637e-10 1.87255912493232e-09 -100
"chr2" 134538001 134539000 "*" 0 0 100
"chr2" 134577001 134578000 "*" 0 0 100
"chr2" 134598001 134599000 "*" 0 0 100
"chr2" 134604001 134605000 "*" 3.76365605347928e-14 1.3043487970679e-13 -100
"chr2" 134834001 134835000 "*" 3.6700841921089e-08 5.65530226072258e-08 100
"chr2" 135204001 135205000 "*" 1.98638744342006e-08 3.19202499485717e-08
77.2727272727273
"chr2" 135219001 135220000 "*" 2.42028619368284e-14 8.59499916624584e-14
53.7037037037037
"chr2" 135356001 135357000 "*" 1.66533453693773e-11 4.13277177577036e-11
-76.1904761904762
"chr2" 135476001 135477000 "*" 0 0 52.1237814988391
"chr2" 135499001 135500000 "*" 1.70641278884887e-12 4.81986032810646e-12 100
"chr2" 135559001 135560000 "*" 2.05108707795887e-10 4.39590100856526e-10
-67.948717948718
"chr2" 135809001 135810000 "*" 6.4152538836737e-10 1.27477697728417e-09 100
"chr2" 136105001 136106000 "*" 1.32162481070175e-07 1.88682311059292e-07
83.3333333333333
"chr2" 136632001 136633000 "*" 0 0 -61.1827956989247
"chr2" 136634001 136635000 "*" 0 0 -78.6885245901639
"chr2" 136656001 136657000 "*" 1.4432899320127e-15 5.90750815956055e-15 -100
"chr2" 136722001 136723000 "*" 3.33066907387547e-16 1.4495649018245e-15 -100
"chr2" 136875001 136876000 "*" 0 0 73.9173228346457
"chr2" 136876001 136877000 "*" 2.06035888350442e-08 3.291739145856e-08
80.1385681293303
"chr2" 137695001 137696000 "*" 2.43005615629954e-09 4.45014222280278e-09 -100
"chr2" 137978001 137979000 "*" 3.56131733214582e-07 4.80158368901456e-07
-66.6666666666667
"chr2" 139447001 139448000 "*" 3.34128893442198e-08 5.19772864618523e-08 100
"chr2" 139658001 139659000 "*" 6.89260337694009e-09 1.18313113707883e-08
55.7142857142857
"chr2" 139659001 139660000 "*" 0 0 60.4938271604938
"chr2" 140749001 140750000 "*" 2.22044604925031e-16 9.81641919380259e-16 -100
"chr2" 142888001 142889000 "*" 0 0 60.5275255717734
"chr2" 142957001 142958000 "*" 1.98325800226939e-11 4.85612402473442e-11 100
"chr2" 143938001 143939000 "*" 1.49613654798486e-12 4.26244554429779e-12 -100
"chr2" 144035001 144036000 "*" 6.77335965093562e-12 1.76897011246462e-11 -100
"chr2" 144419001 144420000 "*" 5.96919281781183e-08 8.96418253658915e-08
-81.4814814814815
"chr2" 145281001 145282000 "*" 0 0 78.4054487179487

"chr2" 145333001 145334000 "*" 8.3882012447134e-11 1.88922989503423e-10 100
"chr2" 145348001 145349000 "*" 0 0 100
"chr2" 146560001 146561000 "*" 0 0 100
"chr2" 147907001 147908000 "*" 3.07314299563544e-08 4.81129071584046e-08 90.9090909090909
"chr2" 148079001 148080000 "*" 3.95353005888666e-09 7.03928529283733e-09 -100
"chr2" 148494001 148495000 "*" 8.09791567135676e-10 1.59023287870581e-09 62.0689655172414
"chr2" 148601001 148602000 "*" 0 0 52.6356034230306
"chr2" 149567001 149568000 "*" 3.85540880221136e-06 4.49215246381664e-06 -80
"chr2" 149666001 149667000 "*" 1.58458801635675e-11 3.94224099794118e-11 51.5463917525773
"chr2" 149854001 149855000 "*" 0 0 -67.530487804878
"chr2" 150023001 150024000 "*" 3.6700841921089e-08 5.65530226072258e-08 -100
"chr2" 151812001 151813000 "*" 1.11022302462516e-16 5.03662826618488e-16 68.2926829268293
"chr2" 152118001 152119000 "*" 0 0 -80.672268907563
"chr2" 152146001 152147000 "*" 0 0 -77.7777777777778
"chr2" 152167001 152168000 "*" 3.5527136788005e-15 1.39277426410519e-14 100
"chr2" 152171001 152172000 "*" 1.53210777398272e-14 5.5628583449216e-14 100
"chr2" 152199001 152200000 "*" 1.52794177310511e-09 2.87642564232045e-09 100
"chr2" 152955001 152956000 "*" 0 0 81.5673981191223
"chr2" 153648001 153649000 "*" 1.38590361409285e-10 3.02706178892116e-10 100
"chr2" 154526001 154527000 "*" 4.60954030279126e-07 6.12056624724009e-07 -76.0869565217391
"chr2" 154727001 154728000 "*" 3.48610029732299e-14 1.21446614245198e-13 51.9157088122605
"chr2" 155048001 155049000 "*" 0 0 -58.8904922238256
"chr2" 155730001 155731000 "*" 5.10702591327572e-15 1.96271840288234e-14 -100
"chr2" 156188001 156189000 "*" 2.15125472990962e-10 4.58126659943874e-10 -100
"chr2" 156704001 156705000 "*" 3.56775498033812e-10 7.35898629809829e-10 100
"chr2" 157188001 157189000 "*" 0 0 -100
"chr2" 157292001 157293000 "*" 7.90545406914589e-12 2.04967942061334e-11 -61.0244988864143
"chr2" 158607001 158608000 "*" 6.44564628715649e-06 7.26663863268796e-06 52.1739130434783
"chr2" 158954001 158955000 "*" 2.00227675550835e-08 3.20459176617961e-08 -100
"chr2" 159599001 159600000 "*" 5.55111512312578e-16 2.36485094870365e-15 100
"chr2" 159715001 159716000 "*" 6.95445921961024e-08 1.03496858721798e-07 -69.7860962566845
"chr2" 159750001 159751000 "*" 2.08995487582797e-10 4.46944006972663e-10 100
"chr2" 159814001 159815000 "*" 6.24654217240561e-10 1.2462108191303e-09 100
"chr2" 160041001 160042000 "*" 1.5277158427196e-09 2.87642564232045e-09 100
"chr2" 160355001 160356000 "*" 1.98365768255826e-11 4.85612402473442e-11 -100
"chr2" 161078001 161079000 "*" 8.01666433236647e-09 1.36392368970109e-08 100
"chr2" 161263001 161264000 "*" 0 0 59.4676549865229
"chr2" 161276001 161277000 "*" 5.03441732746523e-12 1.3378171545226e-11 100
"chr2" 161350001 161351000 "*" 0 0 60
"chr2" 161750001 161751000 "*" 4.77395900588817e-15 1.84201484337652e-14 100
"chr2" 162929001 162930000 "*" 1.35036426485158e-12 3.86629547363774e-12 -100
"chr2" 163064001 163065000 "*" 2.82773804372027e-13 8.79129664109438e-13 100
"chr2" 163772001 163773000 "*" 0 0 -100

"chr2" 166179001 166180000 "*" 0 0 -100
"chr2" 166650001 166651000 "*" 0 0 -62.4471774239266
"chr2" 166651001 166652000 "*" 6.05556308646005e-05 5.8787575452483e-05
-50.6172839506173
"chr2" 166828001 166829000 "*" 0 0 100
"chr2" 167232001 167233000 "*" 0 0 70.3012912482066
"chr2" 167599001 167600000 "*" 1.77079773067135e-09 3.31334585803845e-09
95.2380952380952
"chr2" 168149001 168150000 "*" 0 0 62.9705590278085
"chr2" 168819001 168820000 "*" 5.8225857468841e-09 1.01168272803324e-08 60
"chr2" 169102001 169103000 "*" 0 0 100
"chr2" 170219001 170220000 "*" 0 0 56.0951789618017
"chr2" 170221001 170222000 "*" 0 0 86.8932038834951
"chr2" 170264001 170265000 "*" 3.24935789386416e-08 5.07231761192387e-08
79.1666666666667
"chr2" 170945001 170946000 "*" 3.6700841921089e-08 5.65530226072258e-08 -100
"chr2" 170995001 170996000 "*" 1.21382983565566e-05 1.31297425905988e-05
-59.375
"chr2" 171188001 171189000 "*" 3.56775498033812e-10 7.35898629809829e-10 -100
"chr2" 171265001 171266000 "*" 0 0 100
"chr2" 171553001 171554000 "*" 8.43347613965761e-12 2.17859001990012e-11 100
"chr2" 171564001 171565000 "*" 6.4152538836737e-10 1.27477697728417e-09 -100
"chr2" 171571001 171572000 "*" 0 0 -83.7894736842105
"chr2" 171673001 171674000 "*" 0 0 61.4814814814815
"chr2" 171677001 171678000 "*" 8.88178419700125e-16 3.70670032207938e-15 -100
"chr2" 171779001 171780000 "*" 1.20591092667155e-10 2.66075324129686e-10 100
"chr2" 171785001 171786000 "*" 0 0 74.5562130177515
"chr2" 171832001 171833000 "*" 1.35036426485158e-12 3.86629547363774e-12 -100
"chr2" 171838001 171839000 "*" 0 0 100
"chr2" 172162001 172163000 "*" 6.93112234273485e-13 2.05218842007357e-12 100
"chr2" 172175001 172176000 "*" 5.794809077031e-12 1.52911192757929e-11 -100
"chr2" 172379001 172380000 "*" 0 0 64.9307479224377
"chr2" 172380001 172381000 "*" 0 0 67.4074074074074
"chr2" 172950001 172951000 "*" 0 0 82.3244552058111
"chr2" 172959001 172960000 "*" 0 0 -66.1033215232048
"chr2" 172963001 172964000 "*" 0 0 -97.7029096477795
"chr2" 173099001 173100000 "*" 0 0 50.5702408032003
"chr2" 173115001 173116000 "*" 4.61959809339163e-06 5.3205598540144e-06
52.3809523809524
"chr2" 173116001 173117000 "*" 8.3882012447134e-11 1.88922989503423e-10 -100
"chr2" 173402001 173403000 "*" 0 0 100
"chr2" 173520001 173521000 "*" 2.08995487582797e-10 4.46944006972663e-10 -100
"chr2" 173673001 173674000 "*" 4.65637306490407e-10 9.46720524046618e-10 100
"chr2" 174080001 174081000 "*" 4.25785910196375e-07 5.67982624562217e-07
-70.8333333333333
"chr2" 174344001 174345000 "*" 3.40125705378114e-10 7.03784073980162e-10 -100
"chr2" 174528001 174529000 "*" 1.52794177310511e-09 2.87642564232045e-09 -100
"chr2" 174695001 174696000 "*" 2.00227675550835e-08 3.20459176617961e-08 -100
"chr2" 175112001 175113000 "*" 4.44089209850063e-16 1.91071758245033e-15 -100
"chr2" 175199001 175200000 "*" 0 0 52.2836504876072
"chr2" 175201001 175202000 "*" 0 0 51.4090415349388
"chr2" 175613001 175614000 "*" 1.32095667737531e-10 2.89894765044537e-10

-62.2222222222222
"chr2" 175618001 175619000 "*" 0 0 -100
"chr2" 175637001 175638000 "*" 0.000209763322864043 0.000186660769941092
-56.5217391304348
"chr2" 175869001 175870000 "*" 0 0 83.6195508586526
"chr2" 175870001 175871000 "*" 0 0 91.4893617021277
"chr2" 176048001 176049000 "*" 4.44089209850063e-15 1.71914916534614e-14 100
"chr2" 176680001 176681000 "*" 4.86753970463383e-11 1.13145908161915e-10 -100
"chr2" 176943001 176944000 "*" 8.21565038222616e-15 3.08158744293689e-14 100
"chr2" 176949001 176950000 "*" 3.40172334745148e-13 1.05002442013229e-12
57.7281191806332
"chr2" 176983001 176984000 "*" 1.01155839704603e-07 1.46695662213855e-07
-81.6666666666667
"chr2" 177074001 177075000 "*" 2.00227675550835e-08 3.20459176617961e-08 100
"chr2" 177374001 177375000 "*" 9.65729496371637e-10 1.87255912493232e-09 -100
"chr2" 177418001 177419000 "*" 2.66453525910038e-15 1.05861327033776e-14 -100
"chr2" 177508001 177509000 "*" 7.105427357601e-15 2.68362283258525e-14 87.5
"chr2" 178483001 178484000 "*" 0 0 64.5669291338583
"chr2" 178700001 178701000 "*" 0 0 -70.7395569271465
"chr2" 179052001 179053000 "*" 5.01025332333427e-10 1.01358031135889e-09 -100
"chr2" 179058001 179059000 "*" 0 0 85.499462943072
"chr2" 179059001 179060000 "*" 0 0 77.2745868106693
"chr2" 179211001 179212000 "*" 5.09592368302947e-14 1.73761636562837e-13 100
"chr2" 180379001 180380000 "*" 6.24654217240561e-10 1.2462108191303e-09 100
"chr2" 180869001 180870000 "*" 5.08482145278322e-14 1.73432706170696e-13 -100
"chr2" 181947001 181948000 "*" 3.34128893442198e-08 5.19772864618523e-08 100
"chr2" 183472001 183473000 "*" 8.7349629751543e-09 1.47505871898511e-08 100
"chr2" 183679001 183680000 "*" 2.58681964737661e-14 9.14008479605639e-14 100
"chr2" 183689001 183690000 "*" 6.32031397662658e-07 8.24885904538156e-07
51.4285714285714
"chr2" 183937001 183938000 "*" 7.47069073270268e-13 2.20716573010896e-12
-62.1621621621622
"chr2" 184088001 184089000 "*" 0 0 -74.6268656716418
"chr2" 185546001 185547000 "*" 8.7349629751543e-09 1.47505871898511e-08 100
"chr2" 186337001 186338000 "*" 8.43347613965761e-12 2.17859001990012e-11 100
"chr2" 186432001 186433000 "*" 7.0006535457523e-08 1.03695832512054e-07 100
"chr2" 187599001 187600000 "*" 7.18203274630014e-11 1.63247175597028e-10
66.6666666666667
"chr2" 187986001 187987000 "*" 0 0 100
"chr2" 188404001 188405000 "*" 7.10435377193619e-10 1.40434703159151e-09
75.5395683453237
"chr2" 188690001 188691000 "*" 7.31858089020321e-07 9.46552240977687e-07 68.75
"chr2" 188732001 188733000 "*" 2.22044604925031e-15 8.90449334476539e-15
91.6666666666667
"chr2" 188905001 188906000 "*" 0 0 100
"chr2" 189157001 189158000 "*" 0 0 65.5080213903743
"chr2" 189470001 189471000 "*" 2.455369241261e-12 6.8003688121118e-12 100
"chr2" 190517001 190518000 "*" 6.66133814775094e-16 2.81595744474255e-15
-55.7692307692308
"chr2" 191044001 191045000 "*" 2.88710388929303e-10 6.03399264731139e-10 -100
"chr2" 191045001 191046000 "*" 0 0 -71.7729196941935
"chr2" 191086001 191087000 "*" 4.71134242729931e-12 1.257615036806e-11 100

"chr2" 191208001 191209000 "*" 0 0 -81.8513119533528
"chr2" 191399001 191400000 "*" 0 0 93.4523809523809
"chr2" 191512001 191513000 "*" 2.68141064907468e-12 7.40011637970864e-12
66.6666666666667
"chr2" 191916001 191917000 "*" 0.000525299954949099 0.000436849389802675
-54.5454545454545
"chr2" 192342001 192343000 "*" 1.65000946239502e-09 3.09685703416913e-09
-83.5443037974684
"chr2" 192417001 192418000 "*" 9.58591753854598e-05 9.01307748135006e-05
54.0540540540541
"chr2" 193222001 193223000 "*" 4.46630721295804e-09 7.88495447686300e-09
55.1724137931034
"chr2" 194064001 194065000 "*" 6.24654217240561e-10 1.2462108191303e-09 -100
"chr2" 194155001 194156000 "*" 1.11022302462516e-16 5.03662826618488e-16 -100
"chr2" 194885001 194886000 "*" 2.69018141096922e-12 7.41487347763769e-12 100
"chr2" 195040001 195041000 "*" 1.22124532708767e-15 5.03921984217778e-15
69.6969696969697
"chr2" 195707001 195708000 "*" 1.11022302462516e-16 5.03662826618488e-16 100
"chr2" 196989001 196990000 "*" 4.99020824662466e-11 1.15790792586157e-10 100
"chr2" 197339001 197340000 "*" 4.65637306490407e-10 9.46720524046618e-10 100
"chr2" 197663001 197664000 "*" 3.97459842815806e-14 1.37266254951776e-13
98.5507246376812
"chr2" 197664001 197665000 "*" 0 0 -98.3333333333333
"chr2" 198052001 198053000 "*" 2.02060590481778e-13 6.40127405465654e-13 100
"chr2" 198239001 198240000 "*" 5.01025332333427e-10 1.01358031135889e-09 100
"chr2" 198245001 198246000 "*" 4.08209022140227e-11 9.57770690255255e-11 100
"chr2" 198321001 198322000 "*" 2.43438049718492e-08 3.85481082003822e-08
-57.6923076923077
"chr2" 198505001 198506000 "*" 3.69056931970313e-06 4.31497859976197e-06
-54.8387096774194
"chr2" 198757001 198758000 "*" 0 0 100
"chr2" 198870001 198871000 "*" 1.33226762955019e-15 5.48133041560118e-15 100
"chr2" 198914001 198915000 "*" 1.77550829594253e-07 2.48984630600584e-07
71.1111111111111
"chr2" 199159001 199160000 "*" 7.48371140613102e-08 1.10376633100146e-07
80.4347826086957
"chr2" 199241001 199242000 "*" 8.25450818808804e-13 2.42473843346228e-12 100
"chr2" 199742001 199743000 "*" 2.74687161905263e-10 5.76953704674915e-10
66.6666666666667
"chr2" 200298001 200299000 "*" 0 0 -100
"chr2" 200332001 200333000 "*" 5.44009282066327e-15 2.08486137681812e-14
-79.4392523364486
"chr2" 200677001 200678000 "*" 1.38590361409285e-10 3.02706178892116e-10 -100
"chr2" 201336001 201337000 "*" 1.12843068222901e-12 3.259512533505e-12 100
"chr2" 201561001 201562000 "*" 7.7715611723761e-16 3.26213507634405e-15 100
"chr2" 201581001 201582000 "*" 1.98365768255826e-11 4.85612402473442e-11 100
"chr2" 201605001 201606000 "*" 1.89848137210902e-14 6.82212372863451e-14 100
"chr2" 201607001 201608000 "*" 1.93549143379101e-09 3.60259188423792e-09
57.1428571428571
"chr2" 201618001 201619000 "*" 6.4324989779152e-11 1.47230020825092e-10 100
"chr2" 201753001 201754000 "*" 7.7715611723761e-16 3.26213507634405e-15
78.5714285714286

"chr2" 201990001 201991000 "*" 2.22044604925031e-16 9.81641919380259e-16 100
"chr2" 202110001 202111000 "*" 1.46549439250521e-14 5.33330076973935e-14 100
"chr2" 202754001 202755000 "*" 9.65729496371637e-10 1.87255912493232e-09 -100
"chr2" 203002001 203003000 "*" 4.71103192345446e-07 6.24717132053794e-07
-71.7948717948718
"chr2" 203047001 203048000 "*" 3.44722487444216e-07 4.6586018916251e-07 -80
"chr2" 203825001 203826000 "*" 0 0 90.2777777777778
"chr2" 204063001 204064000 "*" 4.73234496034536e-09 8.30627300264826e-09 -100
"chr2" 204283001 204284000 "*" 2.79987144580218e-12 7.68634726811898e-12 100
"chr2" 204339001 204340000 "*" 4.98823204964083e-13 1.50781787389592e-12
-64.2857142857143
"chr2" 204823001 204824000 "*" 1.74853465040314e-11 4.32752710900609e-11 -100
"chr2" 204987001 204988000 "*" 1.66533453693773e-15 6.7629186866784e-15 -100
"chr2" 205319001 205320000 "*" 5.40375078574851e-05 5.28457203825496e-05
-58.6206896551724
"chr2" 206429001 206430000 "*" 0 0 98.1132075471698
"chr2" 206514001 206515000 "*" 6.59550958292954e-09 1.13456194382773e-08 -100
"chr2" 206546001 206547000 "*" 7.92483196843108e-08 1.16491268402433e-07
-60.655737704918
"chr2" 206605001 206606000 "*" 8.88178419700125e-16 3.70670032207938e-15
-64.5161290322581
"chr2" 206673001 206674000 "*" 1.12798659301916e-13 3.68863190206523e-13
-98.1132075471698
"chr2" 206692001 206693000 "*" 0 0 -100
"chr2" 206847001 206848000 "*" 5.50059198078934e-07 7.23608323517707e-07
64.2857142857143
"chr2" 206963001 206964000 "*" 3.6892711108294e-13 1.13266255090498e-12 -100
"chr2" 207095001 207096000 "*" 4.79888864202138e-08 7.29535840441379e-08
90.9090909090909
"chr2" 207114001 207115000 "*" 2.00227675550835e-08 3.20459176617961e-08 100
"chr2" 207307001 207308000 "*" 0 0 57.0397111913357
"chr2" 207308001 207309000 "*" 0 0 75.3307449812085
"chr2" 207373001 207374000 "*" 3.40125705378114e-10 7.03784073980162e-10 -100
"chr2" 207409001 207410000 "*" 1.96509475358653e-13 6.23868565133654e-13 -100
"chr2" 207593001 207594000 "*" 5.24021992465151e-09 9.15128659652885e-09
85.2173913043478
"chr2" 207723001 207724000 "*" 3.56775498033812e-10 7.35898629809829e-10 -100
"chr2" 207746001 207747000 "*" 0 0 100
"chr2" 207799001 207800000 "*" 3.23373829846929e-06 3.81325174359463e-06
-66.6666666666667
"chr2" 208030001 208031000 "*" 0 0 86.8055555555556
"chr2" 208092001 208093000 "*" 9.65729496371637e-10 1.87255912493232e-09 -100
"chr2" 208121001 208122000 "*" 9.99200722162641e-16 4.15382497462808e-15 -100
"chr2" 208126001 208127000 "*" 2.62900812231237e-13 8.21294938531841e-13 -100
"chr2" 208577001 208578000 "*" 0 0 64.1791044776119
"chr2" 208766001 208767000 "*" 8.87188211784462e-10 1.73474586092709e-09
66.6666666666667
"chr2" 208890001 208891000 "*" 0 0 -100
"chr2" 208942001 208943000 "*" 0 0 -100
"chr2" 210172001 210173000 "*" 7.35558233211364e-08 1.08588645363448e-07
64.5161290322581
"chr2" 210288001 210289000 "*" 0 0 79.0007593605795

"chr2" 210798001 210799000 "*" 2.17381668221606e-13 6.85151076607736e-13 -100
"chr2" 211036001 211037000 "*" 0 0 100
"chr2" 211397001 211398000 "*" 9.0072393987839e-13 2.63157725646847e-12 -100
"chr2" 211814001 211815000 "*" 2.64951482975562e-09 4.82186011375752e-09 100
"chr2" 212248001 212249000 "*" 0 0 -100
"chr2" 213421001 213422000 "*" 3.49194319015922e-08 5.41783859504158e-08
-71.4285714285714
"chr2" 214016001 214017000 "*" 0 0 98.159509202454
"chr2" 214473001 214474000 "*" 0 0 -93.0555555555556
"chr2" 216154001 216155000 "*" 0 0 -100
"chr2" 216292001 216293000 "*" 3.52704532247117e-11 8.35581292290093e-11 -100
"chr2" 217123001 217124000 "*" 0.000411208966935583 0.000348393043769852 60
"chr2" 217148001 217149000 "*" 0 0 82.6589595375723
"chr2" 217156001 217157000 "*" 7.0006535457523e-08 1.03695832512054e-07 100
"chr2" 217171001 217172000 "*" 5.99520433297585e-15 2.28531569014092e-14 100
"chr2" 217215001 217216000 "*" 4.72724753575182e-06 5.43823522669881e-06
60.9756097560976
"chr2" 217259001 217260000 "*" 0 0 100
"chr2" 217499001 217500000 "*" 1.67199587508549e-13 5.35958264711306e-13
76.5625
"chr2" 217541001 217542000 "*" 2.63705812741932e-09 4.81075612212998e-09
56.6265060240964
"chr2" 217543001 217544000 "*" 3.51196849379676e-12 9.54602288297317e-12
78.2312925170068
"chr2" 217555001 217556000 "*" 6.24654217240561e-10 1.2462108191303e-09 100
"chr2" 217556001 217557000 "*" 0 0 91.9540229885057
"chr2" 217558001 217559000 "*" 1.65254476769405e-11 4.10274120796921e-11
59.8516949152542
"chr2" 217688001 217689000 "*" 1.39779633423487e-07 1.98463037958589e-07 100
"chr2" 217724001 217725000 "*" 6.51415985325476e-05 6.29407268479517e-05
54.0540540540541
"chr2" 217780001 217781000 "*" 1.05349062806681e-12 3.05468319131578e-12
69.0265486725664
"chr2" 217839001 217840000 "*" 1.4432899320127e-15 5.90750815956055e-15 100
"chr2" 217859001 217860000 "*" 3.95353005888666e-09 7.03928529283733e-09 100
"chr2" 218201001 218202000 "*" 1.24344978758018e-14 4.56627092294518e-14
70.8333333333333
"chr2" 218473001 218474000 "*" 1.49383453384999e-08 2.444556877867e-08
66.6666666666667
"chr2" 218476001 218477000 "*" 1.11022302462516e-16 5.03662826618488e-16 100
"chr2" 218662001 218663000 "*" 6.42352513291478e-07 8.37465176000401e-07
-88.2352941176471
"chr2" 218665001 218666000 "*" 2.84687198259803e-08 4.47501946080966e-08
-55.2238805970149
"chr2" 218687001 218688000 "*" 0.000233299213712068 0.000206023994887949 55
"chr2" 218695001 218696000 "*" 2.00227675550835e-08 3.20459176617961e-08 100
"chr2" 218701001 218702000 "*" 1.50983850133457e-06 1.8707430185703e-06
59.5744680851064
"chr2" 218754001 218755000 "*" 1.64594005092056e-10 3.56357694289122e-10
-79.1666666666667
"chr2" 218780001 218781000 "*" 1.17461596005342e-13 3.8279067575877e-13 100
"chr2" 218790001 218791000 "*" 3.53400456509778e-06 4.14231950904509e-06

64.5161290322581
"chr2" 218797001 218798000 "*" 2.33146835171283e-15 9.32818831833246e-15
57.1428571428571
"chr2" 218810001 218811000 "*" 3.82360809680904e-13 1.17205079466646e-12
76.0869565217391
"chr2" 218845001 218846000 "*" 5.12525754059467e-07 6.76484167684476e-07
-70.6060606060606
"chr2" 218854001 218855000 "*" 1.11022302462516e-16 5.03662826618488e-16 -100
"chr2" 218866001 218867000 "*" 1.20591092667155e-10 2.66075324129686e-10 100
"chr2" 218869001 218870000 "*" 1.67299207820548e-08 2.71377429441055e-08 -100
"chr2" 218932001 218933000 "*" 4.01313771103418e-09 7.11882087237587e-09 -100
"chr2" 218935001 218936000 "*" 7.47435446868394e-12 1.94201181372257e-11 96.875
"chr2" 218942001 218943000 "*" 1.75485082021254e-09 3.284665679682e-09
-57.5757575757576
"chr2" 218944001 218945000 "*" 0 0 100
"chr2" 218990001 218991000 "*" 1.74382747852864e-05 1.84311384275609e-05
-70.3703703703704
"chr2" 219141001 219142000 "*" 1.6543322267637e-11 4.10681214226464e-11
51.5151515151515
"chr2" 219149001 219150000 "*" 3.33066907387547e-16 1.4495649018245e-15 -100
"chr2" 219156001 219157000 "*" 0 0 -100
"chr2" 219232001 219233000 "*" 0 0 -62.9251700680272
"chr2" 219238001 219239000 "*" 5.55111512312578e-16 2.36485094870365e-15
99.0291262135922
"chr2" 219258001 219259000 "*" 0 0 100
"chr2" 219266001 219267000 "*" 2.64951482975562e-09 4.82186011375752e-09 100
"chr2" 219283001 219284000 "*" 1.43636692098159e-05 1.5361751244027e-05
64.367816091954
"chr2" 219290001 219291000 "*" 0 0 -62.2159090909091
"chr2" 219522001 219523000 "*" 1.95399252334028e-14 7.01253661173697e-14 -100
"chr2" 219709001 219710000 "*" 0 0 64.4153225806452
"chr2" 219720001 219721000 "*" 6.4152538836737e-10 1.27477697728417e-09 -100
"chr2" 219771001 219772000 "*" 2.12648565423024e-10 4.54336840422348e-10
-96.1538461538462
"chr2" 219823001 219824000 "*" 8.3882012447134e-11 1.88922989503423e-10 100
"chr2" 219824001 219825000 "*" 0 0 73.1807750311455
"chr2" 219825001 219826000 "*" 0 0 66.3465996110266
"chr2" 219844001 219845000 "*" 0 0 100
"chr2" 219859001 219860000 "*" 6.4152538836737e-10 1.27477697728417e-09 100
"chr2" 219924001 219925000 "*" 0 0 71.3051823416507
"chr2" 219925001 219926000 "*" 0 0 58.7512794268168
"chr2" 220040001 220041000 "*" 9.65729496371637e-10 1.87255912493232e-09 -100
"chr2" 220117001 220118000 "*" 0 0 -85.8156028368794
"chr2" 220143001 220144000 "*" 5.04263297784746e-13 1.52078941532818e-12 100
"chr2" 220154001 220155000 "*" 0 0 -100
"chr2" 220168001 220169000 "*" 3.77475828372553e-15 1.47379557022071e-14 100
"chr2" 220298001 220299000 "*" 6.92287338566189e-11 1.57605630124247e-10 100
"chr2" 220306001 220307000 "*" 0 0 75.8126353897742
"chr2" 220341001 220342000 "*" 0 0 -64.1806839701459
"chr2" 220343001 220344000 "*" 5.99834626413553e-11 1.37943599816489e-10 -87.5
"chr2" 220346001 220347000 "*" 2.46804798820222e-11 5.98815739718167e-11
-51.0204081632653

"chr2" 220374001 220375000 "*" 0 0 90.2439024390244
"chr2" 220380001 220381000 "*" 0.000238053476519395 0.0002099134694894
51.5151515151515
"chr2" 220381001 220382000 "*" 8.00660004962594e-09 1.36392368970109e-08
-73.0769230769231
"chr2" 220392001 220393000 "*" 3.03090885722668e-14 1.06210046699299e-13 100
"chr2" 220406001 220407000 "*" 0 0 -67.504835589942
"chr2" 220408001 220409000 "*" 0 0 66.1354581673307
"chr2" 220411001 220412000 "*" 2.33711849872975e-09 4.29740308430524e-09
-81.5384615384615
"chr2" 220431001 220432000 "*" 0 0 -96.5277777777778
"chr2" 220492001 220493000 "*" 0 0 -100
"chr2" 220566001 220567000 "*" 2.43005615629954e-09 4.45014222280278e-09 100
"chr2" 220796001 220797000 "*" 3.56775498033812e-10 7.35898629809829e-10 -100
"chr2" 220946001 220947000 "*" 1.49106038972446e-05 1.59124089221917e-05
-56.6666666666667
"chr2" 221613001 221614000 "*" 0 0 98.1132075471698
"chr2" 222436001 222437000 "*" 0 0 65.8722353378971
"chr2" 222437001 222438000 "*" 0 0 88.255033557047
"chr2" 222681001 222682000 "*" 6.24654217240561e-10 1.2462108191303e-09 100
"chr2" 223156001 223157000 "*" 0 0 -97.3684210526316
"chr2" 223310001 223311000 "*" 0.00011705526393524 0.000108597608652661
-50.9090909090909
"chr2" 223397001 223398000 "*" 2.88710388929303e-10 6.03399264731139e-10 -100
"chr2" 223654001 223655000 "*" 2.43005615629954e-09 4.45014222280278e-09 100
"chr2" 223695001 223696000 "*" 1.39779633423487e-07 1.98463037958589e-07 100
"chr2" 223838001 223839000 "*" 1.15352895579957e-06 1.45239389013135e-06
77.7777777777778
"chr2" 224084001 224085000 "*" 2.73780997872564e-13 8.52506199209635e-13
72.2222222222222
"chr2" 224487001 224488000 "*" 1.37828859436695e-10 3.01445544267661e-10 -100
"chr2" 224808001 224809000 "*" 6.92287338566189e-11 1.57605630124247e-10 -100
"chr2" 224871001 224872000 "*" 0 0 -100
"chr2" 224940001 224941000 "*" 8.45376425440136e-06 9.36283854341238e-06
67.5675675675676
"chr2" 225249001 225250000 "*" 0 0 98
"chr2" 225261001 225262000 "*" 1.84705436501176e-08 2.97954860987945e-08
64.1025641025641
"chr2" 225397001 225398000 "*" 5.01025332333427e-10 1.01358031135889e-09 -100
"chr2" 225519001 225520000 "*" 2.89768209427166e-14 1.01828132245762e-13
57.7922077922078
"chr2" 225763001 225764000 "*" 7.02493618831568e-12 1.83060326284385e-11
-57.1428571428571
"chr2" 225859001 225860000 "*" 3.95353005888666e-09 7.03928529283733e-09 -100
"chr2" 225865001 225866000 "*" 4.79616346638068e-14 1.64380445416596e-13 100
"chr2" 226094001 226095000 "*" 0 0 55.6770431352371
"chr2" 227664001 227665000 "*" 0 0 -64.7058823529412
"chr2" 228028001 228029000 "*" 0 0 58.3629893238434
"chr2" 228029001 228030000 "*" 0 0 55.4960229783473
"chr2" 228324001 228325000 "*" 3.76365605347928e-14 1.3043487970679e-13 -100
"chr2" 228335001 228336000 "*" 1.25284005392245e-10 2.75649590272477e-10 -100
"chr2" 228612001 228613000 "*" 3.56131733214582e-07 4.80158368901456e-07

-66.6666666666667
"chr2" 229217001 229218000 "*" 0 0 92.0634920634921
"chr2" 229407001 229408000 "*" 8.01666433236647e-09 1.36392368970109e-08 100
"chr2" 229553001 229554000 "*" 1.08311198898647e-07 1.5646019706886e-07
-53.8461538461538
"chr2" 229890001 229891000 "*" 6.66133814775094e-16 2.81595744474255e-15
-93.2038834951456
"chr2" 229968001 229969000 "*" 3.34128893442198e-08 5.19772864618523e-08 -100
"chr2" 230088001 230089000 "*" 8.7349629751543e-09 1.47505871898511e-08 100
"chr2" 230354001 230355000 "*" 1.68753899743024e-14 6.09826805967439e-14 100
"chr2" 231279001 231280000 "*" 6.37156993832377e-13 1.89496793587877e-12
75.609756097561
"chr2" 231440001 231441000 "*" 6.63806998257854e-08 9.90835922040079e-08
79.1044776119403
"chr2" 231510001 231511000 "*" 3.90831811358794e-12 1.05610567215048e-11 -100
"chr2" 231516001 231517000 "*" 6.37490060739765e-13 1.89496793587877e-12 100
"chr2" 231576001 231577000 "*" 9.13071840358270e-11 2.04420777057026e-10
-54.1666666666667
"chr2" 231750001 231751000 "*" 1.13140401492018e-08 1.87801380787728e-08 100
"chr2" 231758001 231759000 "*" 7.66062739687712e-07 9.88262253282658e-07
-52.3076923076923
"chr2" 231806001 231807000 "*" 4.99020824662466e-11 1.15790792586157e-10 100
"chr2" 231808001 231809000 "*" 2.69084754478399e-12 7.41487347763769e-12 -100
"chr2" 231820001 231821000 "*" 1.52794177310511e-09 2.87642564232045e-09 -100
"chr2" 231837001 231838000 "*" 0 0 -100
"chr2" 231868001 231869000 "*" 3.6700841921089e-08 5.65530226072258e-08 100
"chr2" 231880001 231881000 "*" 2.16382467499443e-13 6.82775854166559e-13 100
"chr2" 231915001 231916000 "*" 9.99200722162641e-16 4.15382497462808e-15 100
"chr2" 232254001 232255000 "*" 1.77635683940025e-15 7.19870740878856e-15
73.015873015873
"chr2" 232270001 232271000 "*" 4.99900121297969e-12 1.32921056339624e-11 -100
"chr2" 232276001 232277000 "*" 0 0 -53.3950617283951
"chr2" 232277001 232278000 "*" 0 0 -88.9194373401535
"chr2" 232283001 232284000 "*" 0 0 66.2650602409639
"chr2" 232388001 232389000 "*" 0 0 -100
"chr2" 232398001 232399000 "*" 0 0 59.4594594594595
"chr2" 232413001 232414000 "*" 6.60582699651968e-14 2.22176301799e-13 -100
"chr2" 232435001 232436000 "*" 1.13140401492018e-08 1.87801380787728e-08 -100
"chr2" 232465001 232466000 "*" 7.7715611723761e-16 3.26213507634405e-15 -100
"chr2" 232478001 232479000 "*" 0 0 -77.0712909441233
"chr2" 232479001 232480000 "*" 0 0 -64.0425531914894
"chr2" 232505001 232506000 "*" 0 0 -100
"chr2" 232531001 232532000 "*" 3.75408798674037e-05 3.76260100201549e-05
-54.3478260869565
"chr2" 232551001 232552000 "*" 9.09704322982208e-09 1.53300548732219e-08 58
"chr2" 232570001 232571000 "*" 1.14908083048704e-13 3.75128751466461e-13
-66.6666666666667
"chr2" 232580001 232581000 "*" 2.10755481412361e-05 2.19780359607569e-05
52.1739130434783
"chr2" 232719001 232720000 "*" 7.88258347483861e-15 2.96145301916012e-14
-52.8089887640449
"chr2" 232749001 232750000 "*" 2.02060590481778e-13 6.40127405465654e-13 100

"chr2" 232768001 232769000 "*" 2.96028846991092e-08 4.64323581179945e-08
-56.5217391304348
"chr2" 232836001 232837000 "*" 0 0 -100
"chr2" 233122001 233123000 "*" 7.74977961803813e-08 1.1407490804743e-07
81.8181818181818
"chr2" 233180001 233181000 "*" 0 0 100
"chr2" 233234001 233235000 "*" 5.79344017204164e-10 1.16206967026747e-09
74.0740740740741
"chr2" 233280001 233281000 "*" 2.20192752919957e-11 5.36667613349327e-11 -60
"chr2" 233289001 233290000 "*" 3.92685883809918e-13 1.19951200966592e-12 -100
"chr2" 233311001 233312000 "*" 2.16589898405228e-06 2.62321901854342e-06
-58.3333333333333
"chr2" 233312001 233313000 "*" 3.92563759277209e-12 1.06051085542769e-11 -52
"chr2" 233313001 233314000 "*" 3.51607631898787e-13 1.08255764767511e-12 90
"chr2" 233357001 233358000 "*" 0 0 57.3565323565324
"chr2" 233359001 233360000 "*" 0 0 100
"chr2" 233361001 233362000 "*" 0 0 -59.7938144329897
"chr2" 233364001 233365000 "*" 0 0 78.6195286195286
"chr2" 233396001 233397000 "*" 1.17117426867708e-12 3.3785324022221e-12
-55.2631578947368
"chr2" 233406001 233407000 "*" 5.55111512312578e-16 2.36485094870365e-15
64.367816091954
"chr2" 233434001 233435000 "*" 3.55031964938135e-08 5.50389123170478e-08
61.1111111111111
"chr2" 233500001 233501000 "*" 1.89581683684992e-12 5.32311367606535e-12 -100
"chr2" 233512001 233513000 "*" 1.20925511013503e-05 1.30850105740421e-05
68.1818181818182
"chr2" 233561001 233562000 "*" 0 0 -100
"chr2" 233877001 233878000 "*" 0 0 100
"chr2" 233907001 233908000 "*" 1.38781086646e-05 1.48765977564982e-05
66.6666666666667
"chr2" 233918001 233919000 "*" 4.44089209850063e-16 1.91071758245033e-15 100
"chr2" 233945001 233946000 "*" 1.01030295240889e-14 3.75322807935163e-14
87.6543209876543
"chr2" 233969001 233970000 "*" 1.51914612356308e-06 1.88121349342686e-06
52.3809523809524
"chr2" 233982001 233983000 "*" 8.3969561479269e-05 7.9676887269758e-05
57.1428571428571
"chr2" 233986001 233987000 "*" 4.44089209850063e-16 1.91071758245033e-15 100
"chr2" 234059001 234060000 "*" 1.29308440732778e-07 1.84867219207644e-07
54.5454545454545
"chr2" 234061001 234062000 "*" 0 0 -95.4545454545455
"chr2" 234071001 234072000 "*" 0 0 100
"chr2" 234091001 234092000 "*" 1.15592780791651e-07 1.66296116792401e-07
-58.5365853658537
"chr2" 234098001 234099000 "*" 2.00227675550835e-08 3.20459176617961e-08 100
"chr2" 234119001 234120000 "*" 4.42090808405737e-13 1.34148377460197e-12
57.7299412915851
"chr2" 234186001 234187000 "*" 6.4152538836737e-10 1.27477697728417e-09 -100
"chr2" 234236001 234237000 "*" 2.67563748934663e-14 9.43871209921996e-14 56
"chr2" 234315001 234316000 "*" 1.19904086659517e-14 4.40901388691796e-14 -100
"chr2" 234330001 234331000 "*" 1.79595756044648e-08 2.90194928352674e-08

-72.5806451612903
"chr2" 234360001 234361000 "*" 1.11022302462516e-16 5.03662826618488e-16 100
"chr2" 234475001 234476000 "*" 0 0 -88.1188118811881
"chr2" 234621001 234622000 "*" 9.23500165228575e-11 2.06694462151983e-10
-58.3333333333333
"chr2" 234657001 234658000 "*" 3.07531777821168e-14 1.07626052665081e-13 100
"chr2" 234726001 234727000 "*" 6.43090025675974e-10 1.2776729800285e-09
-72.972972972973
"chr2" 234777001 234778000 "*" 0 0 95.3246753246753
"chr2" 234836001 234837000 "*" 1.14118493410587e-06 1.43776332576622e-06
74.6031746031746
"chr2" 234849001 234850000 "*" 6.4152538836737e-10 1.27477697728417e-09 100
"chr2" 234867001 234868000 "*" 2.43005615629954e-09 4.45014222280278e-09 -100
"chr2" 234902001 234903000 "*" 1.11022302462516e-16 5.03662826618488e-16 100
"chr2" 234918001 234919000 "*" 0 0 100
"chr2" 235111001 235112000 "*" 1.68753899743024e-14 6.09826805967439e-14 -100
"chr2" 235167001 235168000 "*" 8.67242865021378e-06 9.58909945706894e-06
72.2222222222222
"chr2" 235370001 235371000 "*" 0 0 -100
"chr2" 235377001 235378000 "*" 4.89213718164461e-05 4.81845684656791e-05
64.5161290322581
"chr2" 235393001 235394000 "*" 0 0 -100
"chr2" 235443001 235444000 "*" 6.59550958292954e-09 1.13456194382773e-08 100
"chr2" 235457001 235458000 "*" 3.04201108747293e-14 1.06563274901204e-13 100
"chr2" 235469001 235470000 "*" 3.62253696684078e-06 4.24060787189294e-06
54.2372881355932
"chr2" 235476001 235477000 "*" 0 0 100
"chr2" 235477001 235478000 "*" 0 0 -100
"chr2" 235515001 235516000 "*" 1.87405646556726e-13 5.97285292681429e-13 100
"chr2" 235530001 235531000 "*" 9.0072393987839e-13 2.63157725646847e-12 100
"chr2" 235694001 235695000 "*" 2.00950367457153e-14 7.20431616826861e-14 -81
"chr2" 235707001 235708000 "*" 1.39779633423487e-07 1.98463037958589e-07 100
"chr2" 235845001 235846000 "*" 3.95353005888666e-09 7.03928529283733e-09 -100
"chr2" 235909001 235910000 "*" 0 0 69.3519351935194
"chr2" 235926001 235927000 "*" 8.3882012447134e-11 1.88922989503423e-10 100
"chr2" 235997001 235998000 "*" 0 0 100
"chr2" 236025001 236026000 "*" 3.63445940010365e-11 8.58565209111204e-11 -100
"chr2" 236064001 236065000 "*" 1.90070181815827e-13 6.05298617539364e-13 81.25
"chr2" 236094001 236095000 "*" 9.2148511043888e-15 3.43705279328267e-14 100
"chr2" 236151001 236152000 "*" 3.9190872769268e-14 1.35416418933905e-13 100
"chr2" 236201001 236202000 "*" 3.76365605347928e-14 1.3043487970679e-13 100
"chr2" 236252001 236253000 "*" 0 0 -56.25
"chr2" 236272001 236273000 "*" 4.08209022140227e-11 9.57770690255255e-11 -100
"chr2" 236278001 236279000 "*" 2.1094237467878e-15 8.47434540879246e-15 100
"chr2" 236282001 236283000 "*" 4.91828799908944e-14 1.68192819018269e-13 100
"chr2" 236301001 236302000 "*" 4.73234496034536e-09 8.30627300264826e-09 100
"chr2" 236413001 236414000 "*" 9.08108033215171e-11 2.03407753381119e-10 100
"chr2" 236415001 236416000 "*" 1.51094692313336e-11 3.76892557121403e-11
-56.6371681415929
"chr2" 236464001 236465000 "*" 6.55053365550273e-07 8.53210276885233e-07
57.8947368421053
"chr2" 236508001 236509000 "*" 1.98638733239775e-08 3.19202499485717e-08

-77.2727272727273
"chr2" 236531001 236532000 "*" 1.11022302462516e-16 5.03662826618488e-16 100
"chr2" 236550001 236551000 "*" 1.47246328463524e-08 2.41180848031871e-08
72.7272727272727
"chr2" 236611001 236612000 "*" 2.84200885047881e-10 5.96020717471281e-10
-66.6666666666667
"chr2" 236617001 236618000 "*" 6.24005067617617e-08 9.34435715465956e-08
52.3809523809524
"chr2" 236680001 236681000 "*" 6.60744514657807e-09 1.1365222233045e-08
-72.7272727272727
"chr2" 236781001 236782000 "*" 1.17905685215192e-13 3.83653245040640e-13 100
"chr2" 237051001 237052000 "*" 2.88657986402541e-15 1.14155613124573e-14 100
"chr2" 237076001 237077000 "*" 0 0 54.4678079695374
"chr2" 237125001 237126000 "*" 5.12702102994922e-11 1.18809982571841e-10
-60.5263157894737
"chr2" 237147001 237148000 "*" 0 0 100
"chr2" 237166001 237167000 "*" 1.59301718808402e-05 1.69294262236803e-05
-66.6666666666667
"chr2" 237172001 237173000 "*" 1.3532508447156e-12 3.87402820252333e-12
-68.4210526315789
"chr2" 237199001 237200000 "*" 3.63445940010365e-11 8.58565209111204e-11 -100
"chr2" 237202001 237203000 "*" 1.07882591748876e-11 2.74553537455803e-11 100
"chr2" 237217001 237218000 "*" 1.11022302462516e-16 5.03662826618488e-16
81.1594202898551
"chr2" 237229001 237230000 "*" 5.74796876762207e-11 1.3240108304464e-10 67.5
"chr2" 237231001 237232000 "*" 2.15307771611606e-11 5.25298918072789e-11
62.8571428571429
"chr2" 237244001 237245000 "*" 0.000229234831768954 0.000202707360857361
54.1666666666667
"chr2" 237264001 237265000 "*" 6.70841160399505e-12 1.75486545176836e-11 -100
"chr2" 237274001 237275000 "*" 7.20645765284189e-13 2.13076041737731e-12
-66.6666666666667
"chr2" 237308001 237309000 "*" 3.40887318373007e-11 8.09678464968521e-11
68.3544303797468
"chr2" 237357001 237358000 "*" 7.88258347483861e-15 2.96145301916012e-14 -100
"chr2" 237407001 237408000 "*" 6.4152538836737e-10 1.27477697728417e-09 -100
"chr2" 237423001 237424000 "*" 3.96511236044894e-06 4.61243492558304e-06
-67.5675675675676
"chr2" 237453001 237454000 "*" 4.66293670342566e-15 1.80217883586487e-14
-51.5274034141959
"chr2" 237482001 237483000 "*" 0 0 100
"chr2" 237487001 237488000 "*" 0 0 -72.2222222222222
"chr2" 237521001 237522000 "*" 7.0006535457523e-08 1.03695832512054e-07 -100
"chr2" 237526001 237527000 "*" 7.00237092754463e-08 1.03702852845168e-07
82.7586206896552
"chr2" 237527001 237528000 "*" 0 0 -100
"chr2" 237529001 237530000 "*" 5.3296589364038e-11 1.23296793405689e-10
-61.9047619047619
"chr2" 237531001 237532000 "*" 3.58120389308825e-05 3.60126966183746e-05
-61.5384615384615
"chr2" 237533001 237534000 "*" 2.02327044007689e-12 5.65300384792621e-12 100
"chr2" 237537001 237538000 "*" 0 0 58.7155963302752

"chr2" 237540001 237541000 "*" 0 0 53.8461538461538
"chr2" 237545001 237546000 "*" 6.59550958292954e-09 1.13456194382773e-08 100
"chr2" 237549001 237550000 "*" 2.30138209977859e-06 2.77614150127454e-06
-51.2820512820513
"chr2" 237551001 237552000 "*" 9.7405096211034e-08 1.41531991781167e-07 72
"chr2" 237724001 237725000 "*" 1.11022302462516e-16 5.03662826618488e-16
-61.5384615384615
"chr2" 237725001 237726000 "*" 6.5154749595564e-08 9.73303157291081e-08
-66.1971830985916
"chr2" 237797001 237798000 "*" 0 0 100
"chr2" 237880001 237881000 "*" 1.85296222809939e-12 5.21103120049943e-12
-73.9487179487179
"chr2" 237918001 237919000 "*" 6.92287338566189e-11 1.57605630124247e-10 -100
"chr2" 238039001 238040000 "*" 3.95353005888666e-09 7.03928529283733e-09 100
"chr2" 238109001 238110000 "*" 0 0 65.5172413793103
"chr2" 238120001 238121000 "*" 0 0 -100
"chr2" 238246001 238247000 "*" 1.36604061395929e-11 3.42333952202875e-11 100
"chr2" 238252001 238253000 "*" 1.96509475358653e-13 6.23868565133654e-13 100
"chr2" 238255001 238256000 "*" 2.00227675550835e-08 3.20459176617961e-08 100
"chr2" 238259001 238260000 "*" 1.39779633423487e-07 1.98463037958589e-07 100
"chr2" 238320001 238321000 "*" 0 0 -94.5701357466063
"chr2" 238342001 238343000 "*" 0 0 86.8686868686869
"chr2" 238357001 238358000 "*" 0 0 -100
"chr2" 238365001 238366000 "*" 0 0 100
"chr2" 238417001 238418000 "*" 8.71525074330748e-14 2.88848349331391e-13 100
"chr2" 238456001 238457000 "*" 6.76862340087681e-06 7.60546331588873e-06
70.4545454545455
"chr2" 238473001 238474000 "*" 1.12843068222901e-12 3.259512533505e-12 -100
"chr2" 238480001 238481000 "*" 0 0 100
"chr2" 238499001 238500000 "*" 1.81267778565086e-10 3.90965632318115e-10
83.8235294117647
"chr2" 238547001 238548000 "*" 9.63829016598083e-12 2.46993429847588e-11 -100
"chr2" 238600001 238601000 "*" 0 0 -64.2298471092245
"chr2" 238789001 238790000 "*" 1.29037891483108e-11 3.24582280286866e-11 -100
"chr2" 238819001 238820000 "*" 0 0 62.9032258064516
"chr2" 238829001 238830000 "*" 6.94999613415348e-14 2.33262715411123e-13
61.5384615384615
"chr2" 238884001 238885000 "*" 2.68450373042128e-10 5.64465213127486e-10 100
"chr2" 238928001 238929000 "*" 1.80966353013901e-14 6.52206904171103e-14 100
"chr2" 238934001 238935000 "*" 1.11022302462516e-14 4.10577217300208e-14
59.2592592592593
"chr2" 239008001 239009000 "*" 1.22124532708767e-15 5.03921984217778e-15
-57.5621890547264
"chr2" 239052001 239053000 "*" 0 0 -82.6086956521739
"chr2" 239112001 239113000 "*" 0 0 -75
"chr2" 239129001 239130000 "*" 0 0 -59.8540145985401
"chr2" 239130001 239131000 "*" 9.65894031423886e-15 3.59545795222595e-14
62.6168224299065
"chr2" 239172001 239173000 "*" 4.86753970463383e-11 1.13145908161915e-10 -100
"chr2" 239327001 239328000 "*" 9.80763359414993e-10 1.8980032424715e-09 -100
"chr2" 239334001 239335000 "*" 0 0 -53.7763433741587
"chr2" 239366001 239367000 "*" 2.33028818463765e-10 4.93719283454501e-10 100

"chr2" 239406001 239407000 "*" 3.19744231092045e-14 1.1176868990786e-13
64.7058823529412
"chr2" 239431001 239432000 "*" 0 0 100
"chr2" 239484001 239485000 "*" 1.00604635733248e-10 2.24296854644337e-10
57.3770491803279
"chr2" 239496001 239497000 "*" 1.93720595120794e-11 4.76309924836636e-11 100
"chr2" 239544001 239545000 "*" 4.18614440889087e-08 6.41153587808138e-08 -80
"chr2" 239547001 239548000 "*" 0 0 53.4285714285714
"chr2" 239595001 239596000 "*" 1.50623957750895e-12 4.28756717183481e-12 100
"chr2" 239605001 239606000 "*" 4.03688232508692e-06 4.68957697124928e-06
52.3809523809524
"chr2" 239608001 239609000 "*" 1.43329792479108e-13 4.62322269077954e-13
63.8297872340426
"chr2" 239613001 239614000 "*" 0 0 100
"chr2" 239656001 239657000 "*" 2.15125472990962e-10 4.58126659943874e-10 -100
"chr2" 239663001 239664000 "*" 7.93809462606987e-14 2.64679416473686e-13 100
"chr2" 239673001 239674000 "*" 2.75891949430562e-06 3.28830469657595e-06
-51.6129032258064
"chr2" 239686001 239687000 "*" 0 0 100
"chr2" 239702001 239703000 "*" 2.00227675550835e-08 3.20459176617961e-08 100
"chr2" 239716001 239717000 "*" 9.61415627542817e-06 1.05670450583329e-05
-66.6666666666667
"chr2" 239744001 239745000 "*" 1.28907532781497e-06 1.61243929530756e-06 65.625
"chr2" 239773001 239774000 "*" 0 0 67.4157303370787
"chr2" 239781001 239782000 "*" 0 0 100
"chr2" 239795001 239796000 "*" 0 0 100
"chr2" 239799001 239800000 "*" 0 0 -82.9787234042553
"chr2" 239844001 239845000 "*" 0 0 100
"chr2" 239849001 239850000 "*" 3.36430883152161e-12 9.15899139449024e-12
58.695652173913
"chr2" 239864001 239865000 "*" 1.11022302462516e-16 5.03662826618488e-16 100
"chr2" 239865001 239866000 "*" 0 0 100
"chr2" 239876001 239877000 "*" 6.59550958292954e-09 1.13456194382773e-08 100
"chr2" 239891001 239892000 "*" 3.46500605985511e-13 1.06777978272295e-12 100
"chr2" 239918001 239919000 "*" 0 0 86.6071428571429
"chr2" 239919001 239920000 "*" 8.65973959207622e-14 2.87362558212404e-13 100
"chr2" 239951001 239952000 "*" 3.04324343503026e-11 7.26599935673121e-11 100
"chr2" 239955001 239956000 "*" 0 0 100
"chr2" 239960001 239961000 "*" 5.53260992575133e-10 1.11245364740133e-09
70.6806282722513
"chr2" 239961001 239962000 "*" 0 0 64.7058823529412
"chr2" 239963001 239964000 "*" 1.88088877806081e-10 4.0506821693965e-10
60.7142857142857
"chr2" 240120001 240121000 "*" 0 0 100
"chr2" 240134001 240135000 "*" 0 0 100
"chr2" 240138001 240139000 "*" 0 0 -100
"chr2" 240143001 240144000 "*" 0 0 100
"chr2" 240169001 240170000 "*" 0 0 -68.3333333333333
"chr2" 240241001 240242000 "*" 5.55111512312578e-16 2.36485094870365e-15
-66.5584415584416
"chr2" 240263001 240264000 "*" 8.3882012447134e-11 1.88922989503423e-10 100
"chr2" 240347001 240348000 "*" 4.26951363152739e-10 8.72510873516957e-10 100

"chr2" 240348001 240349000 "*" 3.99680288865056e-15 1.5566998858822e-14
70.8333333333333
"chr2" 240379001 240380000 "*" 3.03979064142368e-13 9.42350883309283e-13 100
"chr2" 240387001 240388000 "*" 0 0 -100
"chr2" 240392001 240393000 "*" 0 0 81.9359756097561
"chr2" 240397001 240398000 "*" 2.52449547821776e-07 3.47341761878397e-07
-66.6666666666667
"chr2" 240401001 240402000 "*" 1.99840144432528e-15 8.0570469535907e-15
-98.4615384615385
"chr2" 240415001 240416000 "*" 2.22044604925031e-16 9.81641919380259e-16 100
"chr2" 240434001 240435000 "*" 6.90747459231034e-12 1.80140148623876e-11 100
"chr2" 240444001 240445000 "*" 6.16511286466448e-11 1.41517273500636e-10
50.6183491515674
"chr2" 240458001 240459000 "*" 2.4113033791906e-10 5.10106489523846e-10 78
"chr2" 240459001 240460000 "*" 0 0 100
"chr2" 240479001 240480000 "*" 0 0 66.6666666666667
"chr2" 240494001 240495000 "*" 0 0 100
"chr2" 240496001 240497000 "*" 2.22044604925031e-16 9.81641919380259e-16 100
"chr2" 240497001 240498000 "*" 1.11022302462516e-16 5.03662826618488e-16 100
"chr2" 240543001 240544000 "*" 4.32209823486573e-12 1.15935290479641e-11 -100
"chr2" 240581001 240582000 "*" 0 0 61.0486891385768
"chr2" 240588001 240589000 "*" 4.63584504117875e-10 9.43535904233645e-10
77.7777777777778
"chr2" 240594001 240595000 "*" 0 0 100
"chr2" 240626001 240627000 "*" 7.00293936239937e-06 7.85615355394095e-06
57.6923076923077
"chr2" 240636001 240637000 "*" 0.000430302178842101 0.000363344149266076
-54.5454545454545
"chr2" 240641001 240642000 "*" 0 0 -91.1504424778761
"chr2" 240645001 240646000 "*" 0 0 78.8732394366197
"chr2" 240659001 240660000 "*" 0 0 -84.2105263157895
"chr2" 240667001 240668000 "*" 2.43103236541309e-06 2.92113003733492e-06
55.5072463768116
"chr2" 240669001 240670000 "*" 3.40972369228254e-08 5.29598726926497e-08
61.1111111111111
"chr2" 240690001 240691000 "*" 0 0 63.9344262295082
"chr2" 240698001 240699000 "*" 0 0 67.4074074074074
"chr2" 240702001 240703000 "*" 0 0 100
"chr2" 240712001 240713000 "*" 5.04402234979828e-07 6.66415809115096e-07
-51.8518518518519
"chr2" 240732001 240733000 "*" 1.98365768255826e-11 4.85612402473442e-11 -100
"chr2" 240739001 240740000 "*" 6.99440505513849e-15 2.64480529781883e-14 100
"chr2" 240741001 240742000 "*" 5.36518000042729e-05 5.25070408481107e-05
-56.4102564102564
"chr2" 240742001 240743000 "*" 1.49432208829126e-08 2.44525876862879e-08
-59.504132231405
"chr2" 240774001 240775000 "*" 6.13951112171662e-11 1.4096063499088e-10
58.695652173913
"chr2" 240778001 240779000 "*" 4.85167461761193e-14 1.6617373494005e-13 100
"chr2" 240810001 240811000 "*" 6.32209617990043e-10 1.26065087319343e-09
61.2244897959184
"chr2" 240811001 240812000 "*" 4.99900121297969e-12 1.32921056339624e-11 -100

"chr2" 240858001 240859000 "*" 1.94826821342531e-10 4.18791532986873e-10 61.1111111111111
"chr2" 240908001 240909000 "*" 1.35846889293134e-12 3.88829887027683e-12 71.4285714285714
"chr2" 240930001 240931000 "*" 0 0 100
"chr2" 240940001 240941000 "*" 1.37828859436695e-10 3.01445544267661e-10 100
"chr2" 240993001 240994000 "*" 0 0 55.2082293019075
"chr2" 241045001 241046000 "*" 1.11022302462516e-16 5.03662826618488e-16 66.2921348314607
"chr2" 241046001 241047000 "*" 0 0 87.8205128205128
"chr2" 241048001 241049000 "*" 4.05744147502496e-06 4.71181941756713e-06 51.8518518518519
"chr2" 241049001 241050000 "*" 3.48054918219987e-12 9.46339661057498e-12 -59.2592592592593
"chr2" 241109001 241110000 "*" 0.000181796931795097 0.000163413457407753 50.7246376811594
"chr2" 241128001 241129000 "*" 9.04570973681018e-10 1.76414389822173e-09 100
"chr2" 241134001 241135000 "*" 1.83952852950142e-12 5.17499701329218e-12 100
"chr2" 241138001 241139000 "*" 3.48466044908236e-09 6.25816899925074e-09 50.3538377789875
"chr2" 241147001 241148000 "*" 0 0 100
"chr2" 241148001 241149000 "*" 5.34423616471713e-11 1.23627165813682e-10 90.8045977011494
"chr2" 241151001 241152000 "*" 0 0 100
"chr2" 241167001 241168000 "*" 4.22739621086521e-12 1.13632574968e-11 91.1764705882353
"chr2" 241175001 241176000 "*" 1.94952498588918e-10 4.19050882945867e-10 -74.5614035087719
"chr2" 241176001 241177000 "*" 3.63445940010365e-11 8.58565209111204e-11 100
"chr2" 241178001 241179000 "*" 2.17381668221606e-13 6.85151076607736e-13 -100
"chr2" 241191001 241192000 "*" 4.75075534467351e-12 1.26781104210381e-11 59.0909090909091
"chr2" 241235001 241236000 "*" 1.12634901405784e-10 2.49563754426169e-10 100
"chr2" 241243001 241244000 "*" 0 0 61.8055555555556
"chr2" 241258001 241259000 "*" 0 0 100
"chr2" 241267001 241268000 "*" 0 0 64.985754985755
"chr2" 241277001 241278000 "*" 2.88710388929303e-10 6.03399264731139e-10 -100
"chr2" 241291001 241292000 "*" 0 0 100
"chr2" 241308001 241309000 "*" 9.43986026791244e-07 1.20230128224618e-06 -72.1311475409836
"chr2" 241318001 241319000 "*" 4.43395932303581e-07 5.90039475484287e-07 -67.8571428571429
"chr2" 241371001 241372000 "*" 0 0 100
"chr2" 241397001 241398000 "*" 9.42744771137427e-10 1.83477401762767e-09 54.2372881355932
"chr2" 241449001 241450000 "*" 9.27835586139736e-12 2.38458397452593e-11 55.5178268251273
"chr2" 241519001 241520000 "*" 3.46246967553032e-06 4.06366812326797e-06 -67.4311926605505
"chr2" 241523001 241524000 "*" 4.70405192576351e-09 8.28066210457007e-09 76.0869565217391
"chr2" 241577001 241578000 "*" 3.90765308999619e-10 8.02830445931594e-10

-67.3076923076923
"chr2" 241578001 241579000 "*" 7.07826042223303e-09 1.21322670999066e-08
62.3188405797101
"chr2" 241584001 241585000 "*" 1.16025189456082e-10 2.56707299819029e-10 75
"chr2" 241588001 241589000 "*" 0 0 -67.6923076923077
"chr2" 241633001 241634000 "*" 2.41169097183747e-05 2.49323451373781e-05 -60
"chr2" 241638001 241639000 "*" 6.7481105592293e-08 1.00609078436921e-07
-76.9230769230769
"chr2" 241699001 241700000 "*" 5.51780843238703e-14 1.87243792572872e-13 100
"chr2" 241701001 241702000 "*" 5.05765823011206e-06 5.79476070757596e-06
64.7058823529412
"chr2" 241732001 241733000 "*" 0 0 73.7089201877934
"chr2" 241739001 241740000 "*" 0 0 100
"chr2" 241763001 241764000 "*" 2.68909460143796e-07 3.68650739656412e-07
70.4761904761905
"chr2" 241796001 241797000 "*" 0 0 83.8383838383838
"chr2" 241837001 241838000 "*" 0 0 -52.6315789473684
"chr2" 241844001 241845000 "*" 0 0 97.2602739726027
"chr2" 241867001 241868000 "*" 1.92946192356658e-09 3.59193011611483e-09 100
"chr2" 241887001 241888000 "*" 1.04422847035934e-08 1.74688855906386e-08
64.9122807017544
"chr2" 241910001 241911000 "*" 2.2428975343658e-09 4.13381400626996e-09
73.1034482758621
"chr2" 241913001 241914000 "*" 5.51850422814137e-06 6.28718511088478e-06
69.4444444444444
"chr2" 241923001 241924000 "*" 0 0 67.8288076880098
"chr2" 241941001 241942000 "*" 1.33226762955019e-15 5.48133041560118e-15
-87.3417721518987
"chr2" 241959001 241960000 "*" 0 0 57.3285667858304
"chr2" 241978001 241979000 "*" 1.13421072533981e-09 2.1751239394754e-09
55.952380952381
"chr2" 241990001 241991000 "*" 5.55111512312578e-16 2.36485094870365e-15
63.2077550121172
"chr2" 241991001 241992000 "*" 0 0 64.1318104404619
"chr2" 242012001 242013000 "*" 0 0 62.796442687747
"chr2" 242047001 242048000 "*" 0 0 -100
"chr2" 242147001 242148000 "*" 2.22044604925031e-16 9.81641919380259e-16 100
"chr2" 242154001 242155000 "*" 0 0 70.4225352112676
"chr2" 242286001 242287000 "*" 6.4324989779152e-11 1.47230020825092e-10 100
"chr2" 242357001 242358000 "*" 9.08108033215171e-11 2.03407753381119e-10 -100
"chr2" 242471001 242472000 "*" 1.19232574924411e-08 1.97401088837468e-08 -80
"chr2" 242489001 242490000 "*" 0 0 -98.3193277310924
"chr2" 242510001 242511000 "*" 0 0 -64.8853625809463
"chr2" 242557001 242558000 "*" 9.2861067158001e-07 1.18407475076477e-06
-59.4594594594595
"chr2" 242711001 242712000 "*" 0.000257691951909389 0.000225933396425958 52
"chr2" 242776001 242777000 "*" 3.90465437760668e-13 1.19307893943277e-12 100
"chr2" 242786001 242787000 "*" 0 0 -50.9413401733474
"chr2" 242817001 242818000 "*" 0 0 95.7746478873239
"chr2" 242824001 242825000 "*" 5.99520433297585e-14 2.02586694155801e-13
-72.2007722007722
"chr2" 242830001 242831000 "*" 0 0 67.2068511198946

"chr2" 242852001 242853000 "*" 5.16253706450698e-14 1.75961089260605e-13
-60.4166666666667
"chr2" 242864001 242865000 "*" 7.23212134623452e-09 1.23842797479751e-08
-57.2043010752688
"chr2" 242971001 242972000 "*" 2.00227675550835e-08 3.20459176617961e-08 -100
"chr2" 242981001 242982000 "*" 3.68394204031119e-11 8.69761737460497e-11
60.7142857142857
"chr2" 242996001 242997000 "*" 1.61330021475337e-06 1.99027220773611e-06 75
"chr20" 277001 278000 "*" 0 0 60.8695652173913
"chr20" 343001 344000 "*" 2.00227675550835e-08 3.20459176617961e-08 -100
"chr20" 444001 445000 "*" 1.14124265593318e-11 2.89138062669327e-11 -100
"chr20" 507001 508000 "*" 9.65729496371637e-10 1.87255912493232e-09 -100
"chr20" 520001 521000 "*" 3.75774066613266e-05 3.76580940841167e-05 -60
"chr20" 526001 527000 "*" 1.29037891483108e-11 3.24582280286866e-11 -100
"chr20" 555001 556000 "*" 0 0 100
"chr20" 587001 588000 "*" 3.46500605985511e-13 1.06777978272295e-12 100
"chr20" 589001 590000 "*" 0 0 -67.4698795180723
"chr20" 636001 637000 "*" 8.88178419700125e-16 3.70670032207938e-15 100
"chr20" 653001 654000 "*" 0 0 100
"chr20" 664001 665000 "*" 3.6700841921089e-08 5.65530226072258e-08 100
"chr20" 688001 689000 "*" 5.93800322923332e-06 6.7326516966807e-06
-63.1578947368421
"chr20" 699001 700000 "*" 2.88431091788333e-07 3.93691762673522e-07
-56.6037735849057
"chr20" 700001 701000 "*" 1.55431223447522e-15 6.33705141101682e-15
56.8181818181818
"chr20" 702001 703000 "*" 7.21644966006352e-15 2.72345776413425e-14 100
"chr20" 707001 708000 "*" 1.93720595120794e-11 4.76309924836636e-11 100
"chr20" 713001 714000 "*" 1.25284005392245e-10 2.75649590272477e-10 100
"chr20" 832001 833000 "*" 4.49145921455063e-07 5.97290646423486e-07 70
"chr20" 860001 861000 "*" 0 0 100
"chr20" 914001 915000 "*" 6.46149800331841e-14 2.17664365839349e-13 100
"chr20" 920001 921000 "*" 1.0273815131967e-08 1.72018899950586e-08
-63.8888888888889
"chr20" 921001 922000 "*" 2.70339306496226e-13 8.42355472617862e-13 100
"chr20" 943001 944000 "*" 4.21884749357559e-15 1.63874445239189e-14 -100
"chr20" 946001 947000 "*" 8.00170272263756e-08 1.1755907199822e-07
-51.8518518518519
"chr20" 971001 972000 "*" 2.22044604925031e-15 8.90449334476539e-15 100
"chr20" 973001 974000 "*" 0 0 -99.2125984251968
"chr20" 974001 975000 "*" 4.08209022140227e-11 9.57770690255255e-11 100
"chr20" 993001 994000 "*" 8.88178419700125e-16 3.70670032207938e-15 100
"chr20" 1029001 1030000 "*" 8.7349629751543e-09 1.47505871898511e-08 100
"chr20" 1034001 1035000 "*" 2.29816166097407e-14 8.18850276730353e-14
82.258064516129
"chr20" 1075001 1076000 "*" 0 0 100
"chr20" 1086001 1087000 "*" 0 0 100
"chr20" 1100001 1101000 "*" 5.08482145278322e-14 1.73432706170696e-13 100
"chr20" 1192001 1193000 "*" 6.92287338566189e-11 1.57605630124247e-10 -100
"chr20" 1206001 1207000 "*" 0 0 96.6101694915254
"chr20" 1207001 1208000 "*" 0 0 83.15220451602
"chr20" 1210001 1211000 "*" 2.08995487582797e-10 4.46944006972663e-10 100

"chr20" 1221001 1222000 "*" 2.00227675550835e-08 3.20459176617961e-08 100
"chr20" 1302001 1303000 "*" 6.92588197903632e-09 1.18854993761495e-08 64.1891891891892
"chr20" 1343001 1344000 "*" 1.00808250635964e-13 3.31107370956809e-13 -100
"chr20" 1385001 1386000 "*" 1.5277158427196e-09 2.87642564232045e-09 -100
"chr20" 1454001 1455000 "*" 0.000139451556847803 0.000127724714298755 -52.3809523809524
"chr20" 1456001 1457000 "*" 2.08995487582797e-10 4.46944006972663e-10 100
"chr20" 1609001 1610000 "*" 0 0 100
"chr20" 1766001 1767000 "*" 5.04263297784746e-13 1.52078941532818e-12 -100
"chr20" 1791001 1792000 "*" 2.23872680060744e-07 3.09982983274799e-07 -62.8571428571429
"chr20" 1800001 1801000 "*" 5.55111512312578e-16 2.36485094870365e-15 -74.6835443037975
"chr20" 1847001 1848000 "*" 2.00227675550835e-08 3.20459176617961e-08 -100
"chr20" 1855001 1856000 "*" 0 0 86.8131868131868
"chr20" 1868001 1869000 "*" 8.56759108103233e-13 2.51149198099779e-12 69.5652173913043
"chr20" 1869001 1870000 "*" 9.04570973681018e-10 1.76414389822173e-09 -100
"chr20" 1879001 1880000 "*" 1.11022302462516e-16 5.03662826618488e-16 100
"chr20" 1880001 1881000 "*" 1.12458486967171e-09 2.15740815043925e-09 -100
"chr20" 1882001 1883000 "*" 0 0 -98.7775061124694
"chr20" 1888001 1889000 "*" 8.88178419700125e-16 3.70670032207938e-15 -100
"chr20" 1893001 1894000 "*" 9.23006016062544e-09 1.55419634659478e-08 -61.1111111111111
"chr20" 1896001 1897000 "*" 0 0 100
"chr20" 1900001 1901000 "*" 2.08995487582797e-10 4.46944006972663e-10 100
"chr20" 1907001 1908000 "*" 0.000126887439664713 0.000117029410540844 54.5454545454545
"chr20" 1923001 1924000 "*" 1.25284005392245e-10 2.75649590272477e-10 -100
"chr20" 2058001 2059000 "*" 1.96068431268515e-07 2.73554106454811e-07 51.1111111111111
"chr20" 2105001 2106000 "*" 9.64449142770718e-09 1.62018552751288e-08 68.5714285714286
"chr20" 2114001 2115000 "*" 5.44009282066327e-15 2.08486137681812e-14 100
"chr20" 2116001 2117000 "*" 8.57092175010621e-13 2.51149198099779e-12 -100
"chr20" 2148001 2149000 "*" 3.59900997892737e-12 9.77242196462433e-12 91.1764705882353
"chr20" 2158001 2159000 "*" 2.41241494891575e-07 3.32656142521228e-07 77.5510204081633
"chr20" 2174001 2175000 "*" 0 0 100
"chr20" 2228001 2229000 "*" 4.08209022140227e-11 9.57770690255255e-11 -100
"chr20" 2294001 2295000 "*" 0 0 -100
"chr20" 2315001 2316000 "*" 1.96509475358653e-13 6.23868565133654e-13 100
"chr20" 2355001 2356000 "*" 0.000792090690089564 0.000639295093468792 -52.3809523809524
"chr20" 2364001 2365000 "*" 0.000525299926963374 0.000436849389802675 54.5454545454545
"chr20" 2377001 2378000 "*" 0 0 83.9285714285714
"chr20" 2378001 2379000 "*" 8.2620091101937e-08 1.21184895912793e-07 -66.304347826087
"chr20" 2466001 2467000 "*" 1.12055920098442e-11 2.84500040664577e-11 100

"chr20" 2577001 2578000 "*" 4.28237822980293e-09 7.57162087834286e-09
69.7674418604651
"chr20" 2639001 2640000 "*" 0 0 100
"chr20" 2670001 2671000 "*" 2.38031816479634e-13 7.47299807843568e-13 100
"chr20" 2673001 2674000 "*" 0 0 50.0743140562115
"chr20" 2681001 2682000 "*" 2.32480701356508e-13 7.30972762493372e-13 68.75
"chr20" 2686001 2687000 "*" 1.16408228700848e-06 1.46479698533027e-06
-66.6666666666667
"chr20" 2687001 2688000 "*" 0 0 100
"chr20" 2690001 2691000 "*" 0 0 -95.8762886597938
"chr20" 2693001 2694000 "*" 3.6700841921089e-08 5.65530226072258e-08 100
"chr20" 2787001 2788000 "*" 4.77395900588817e-15 1.84201484337652e-14 100
"chr20" 3040001 3041000 "*" 3.56131752643485e-07 4.80158368901456e-07
-62.962962962963
"chr20" 3081001 3082000 "*" 5.27631605073964e-10 1.06513151973685e-09
53.4883720930233
"chr20" 3139001 3140000 "*" 2.22044604925031e-16 9.81641919380259e-16 100
"chr20" 3183001 3184000 "*" 0 0 -93.4065934065934
"chr20" 3230001 3231000 "*" 4.08209022140227e-11 9.57770690255255e-11 -100
"chr20" 3442001 3443000 "*" 1.29037891483108e-11 3.24582280286866e-11 -100
"chr20" 3451001 3452000 "*" 0 0 60.8239025534068
"chr20" 3692001 3693000 "*" 0 0 -100
"chr20" 3693001 3694000 "*" 1.93720595120794e-11 4.76309924836636e-11 100
"chr20" 3792001 3793000 "*" 1.19434717671396e-09 2.28461917938237e-09
-56.7567567567568
"chr20" 3797001 3798000 "*" 2.44756081845265e-05 2.52637134962309e-05
64.5161290322581
"chr20" 3800001 3801000 "*" 0 0 -86.6666666666667
"chr20" 3873001 3874000 "*" 7.88258347483861e-15 2.96145301916012e-14 -100
"chr20" 4023001 4024000 "*" 4.73234496034536e-09 8.30627300264826e-09 -100
"chr20" 4025001 4026000 "*" 1.14124265593318e-11 2.89138062669327e-11 100
"chr20" 4053001 4054000 "*" 5.63660229602192e-13 1.68905058935478e-12 100
"chr20" 4072001 4073000 "*" 0.000726374156166165 0.000590081342527627
52.1739130434783
"chr20" 4088001 4089000 "*" 1.11022302462516e-16 5.03662826618488e-16 -100
"chr20" 4127001 4128000 "*" 4.71134242729931e-12 1.257615036806e-11 100
"chr20" 4153001 4154000 "*" 4.44089209850063e-16 1.91071758245033e-15
-61.2648221343874
"chr20" 4176001 4177000 "*" 1.67711100562684e-09 3.14567022972737e-09
-60.6060606060606
"chr20" 4215001 4216000 "*" 1.33122005641084e-08 2.191997641159e-08
94.1176470588235
"chr20" 4230001 4231000 "*" 0 0 75.8969264007822
"chr20" 4371001 4372000 "*" 2.33912889058274e-12 6.50001598562471e-12
59.280303030303
"chr20" 4372001 4373000 "*" 2.59135654046005e-08 4.09033331891195e-08
-54.5454545454545
"chr20" 4411001 4412000 "*" 2.64918568193551e-08 4.17868767779178e-08
-82.2429906542056
"chr20" 4584001 4585000 "*" 2.68450373042128e-10 5.64465213127486e-10 100
"chr20" 4590001 4591000 "*" 5.48638023900594e-10 1.10374357336421e-09 100
"chr20" 4615001 4616000 "*" 5.28466159721575e-14 1.79969154943562e-13

52.1094201502601
"chr20" 4668001 4669000 "*" 1.16573417585641e-14 4.29335582900508e-14 -100
"chr20" 4701001 4702000 "*" 0 0 -84.4827586206897
"chr20" 4705001 4706000 "*" 0 0 56.5217391304348
"chr20" 4708001 4709000 "*" 3.6700841921089e-08 5.65530226072258e-08 100
"chr20" 4741001 4742000 "*" 0 0 67.1428571428571
"chr20" 4773001 4774000 "*" 0 0 73.4177215189873
"chr20" 4785001 4786000 "*" 3.5527136788005e-15 1.39277426410519e-14 -100
"chr20" 4791001 4792000 "*" 3.58894225660489e-06 4.20417278477504e-06
-63.6363636363636
"chr20" 4792001 4793000 "*" 1.13140401492018e-08 1.87801380787728e-08 100
"chr20" 4804001 4805000 "*" 0 0 88.1749105403814
"chr20" 4877001 4878000 "*" 0 0 78.5714285714286
"chr20" 4917001 4918000 "*" 2.71626054981766e-11 6.55700665738762e-11
67.3469387755102
"chr20" 5012001 5013000 "*" 7.76974961325116e-06 8.65538265223903e-06
54.4554455445545
"chr20" 5032001 5033000 "*" 1.67299207820548e-08 2.71377429441055e-08 100
"chr20" 5053001 5054000 "*" 2.33028818463765e-10 4.93719283454501e-10 100
"chr20" 5397001 5398000 "*" 1.12458486967171e-09 2.15740815043925e-09 -100
"chr20" 5592001 5593000 "*" 5.55111512312578e-16 2.36485094870365e-15 100
"chr20" 5862001 5863000 "*" 6.88338275267597e-15 2.60518659957509e-14 100
"chr20" 5892001 5893000 "*" 0 0 51.7145625404194
"chr20" 5908001 5909000 "*" 3.4325259023249e-05 3.46019675789391e-05
64.8648648648649
"chr20" 6104001 6105000 "*" 6.55031584528842e-15 2.48816226484197e-14
73.015873015873
"chr20" 6673001 6674000 "*" 4.43011545663285e-05 4.39265220551362e-05
-63.8888888888889
"chr20" 6675001 6676000 "*" 9.5812247025151e-14 3.15667542866807e-13 -100
"chr20" 6747001 6748000 "*" 4.14335232790108e-13 1.26153063645578e-12 100
"chr20" 6748001 6749000 "*" 0 0 62.5534188034188
"chr20" 6749001 6750000 "*" 0 0 62.6189328620647
"chr20" 6768001 6769000 "*" 2.00227675550835e-08 3.20459176617961e-08 100
"chr20" 6790001 6791000 "*" 8.38498487665262e-06 9.29270979340893e-06
-60.2905569007264
"chr20" 6867001 6868000 "*" 1.13140401492018e-08 1.87801380787728e-08 100
"chr20" 7039001 7040000 "*" 1.29037891483108e-11 3.24582280286866e-11 100
"chr20" 8806001 8807000 "*" 6.77335965093562e-12 1.76897011246462e-11 100
"chr20" 8942001 8943000 "*" 4.73234496034536e-09 8.30627300264826e-09 -100
"chr20" 9243001 9244000 "*" 5.48638023900594e-10 1.10374357336421e-09 100
"chr20" 9417001 9418000 "*" 7.0006535457523e-08 1.03695832512054e-07 -100
"chr20" 9497001 9498000 "*" 0 0 -52.1367521367521
"chr20" 9499001 9500000 "*" 8.7349629751543e-09 1.47505871898511e-08 -100
"chr20" 9554001 9555000 "*" 1.49880108324396e-13 4.82666827405525e-13
86.0759493670886
"chr20" 9953001 9954000 "*" 7.55818962971944e-10 1.48929330186584e-09 -75
"chr20" 10015001 10016000 "*" 0 0 97.6744186046512
"chr20" 10152001 10153000 "*" 5.22470955388599e-13 1.57246009980991e-12
-57.7956989247312
"chr20" 10252001 10253000 "*" 8.19565414096335e-05 7.7896169099937e-05
54.5454545454545

"chr20" 10321001 10322000 "*" 6.59550958292954e-09 1.13456194382773e-08 -100
"chr20" 10407001 10408000 "*" 3.5527136788005e-15 1.39277426410519e-14 -100
"chr20" 10413001 10414000 "*" 5.51447776331315e-13 1.65662160105256e-12
66.1016949152542
"chr20" 11663001 11664000 "*" 2.69873012825883e-12 7.43462950485254e-12
-68.1818181818182
"chr20" 12597001 12598000 "*" 3.95861121660346e-12 1.0684496262032e-11 -100
"chr20" 12677001 12678000 "*" 0 0 100
"chr20" 12812001 12813000 "*" 3.33066907387547e-16 1.4495649018245e-15 100
"chr20" 13052001 13053000 "*" 2.1094237467878e-15 8.47434540879246e-15 100
"chr20" 13201001 13202000 "*" 0 0 67.2550873077035
"chr20" 13202001 13203000 "*" 0 0 85.4970760233918
"chr20" 13254001 13255000 "*" 4.79616346638068e-14 1.64380445416596e-13 100
"chr20" 13806001 13807000 "*" 5.48948864776966e-05 5.36254382701292e-05 -75
"chr20" 13975001 13976000 "*" 0 0 -51.419528233673
"chr20" 14050001 14051000 "*" 7.85371767619836e-13 2.31548899886231e-12
-66.6666666666667
"chr20" 14214001 14215000 "*" 2.08814465718632e-09 3.86232567960463e-09 -100
"chr20" 14937001 14938000 "*" 7.0006535457523e-08 1.03695832512054e-07 100
"chr20" 15011001 15012000 "*" 5.6621374255883e-15 2.16468580166064e-14
-92.3566878980892
"chr20" 15201001 15202000 "*" 1.61204383175573e-13 5.17337152201509e-13 -100
"chr20" 15282001 15283000 "*" 2.35965390560366e-08 3.74302033844861e-08
-66.6666666666667
"chr20" 15387001 15388000 "*" 0 0 100
"chr20" 15565001 15566000 "*" 1.55431223447522e-15 6.33705141101682e-15 -100
"chr20" 15681001 15682000 "*" 0 0 -100
"chr20" 15831001 15832000 "*" 4.99020824662466e-11 1.15790792586157e-10 -100
"chr20" 16141001 16142000 "*" 5.55111512312578e-16 2.36485094870365e-15 -100
"chr20" 16217001 16218000 "*" 9.04570973681018e-10 1.76414389822173e-09 -100
"chr20" 16241001 16242000 "*" 5.35493871467452e-12 1.41932759497193e-11
-59.0277777777778
"chr20" 16546001 16547000 "*" 5.48638023900594e-10 1.10374357336421e-09 -100
"chr20" 16555001 16556000 "*" 0 0 -90
"chr20" 17387001 17388000 "*" 0 0 -100
"chr20" 17412001 17413000 "*" 1.38458133847053e-11 3.46813577125413e-11
-67.9245283018868
"chr20" 17418001 17419000 "*" 8.47311110163673e-12 2.18815101835634e-11
-60.1293103448276
"chr20" 17426001 17427000 "*" 1.40240555834836e-08 2.3025296966441e-08
-65.3846153846154
"chr20" 17797001 17798000 "*" 1.15345036417747e-05 1.25186825636811e-05
52.3809523809524
"chr20" 17891001 17892000 "*" 8.7349629751543e-09 1.47505871898511e-08 100
"chr20" 17917001 17918000 "*" 9.65729496371637e-10 1.87255912493232e-09 100
"chr20" 17989001 17990000 "*" 0.000183887360524659 0.000165164039707606
-58.3333333333333
"chr20" 18060001 18061000 "*" 2.1094237467878e-15 8.47434540879246e-15 100
"chr20" 18189001 18190000 "*" 0 0 -81.2252252252252
"chr20" 18193001 18194000 "*" 0 0 -54.7511312217195
"chr20" 18370001 18371000 "*" 1.14352971536391e-14 4.21921674335999e-14 100
"chr20" 18487001 18488000 "*" 2.79440914852103e-11 6.73046092948135e-11 100

"chr20" 18489001 18490000 "*" 0 0 -100
"chr20" 18608001 18609000 "*" 1.68753899743024e-14 6.09826805967439e-14 100
"chr20" 18797001 18798000 "*" 9.08108033215171e-11 2.03407753381119e-10 100
"chr20" 18901001 18902000 "*" 3.33066907387547e-16 1.4495649018245e-15 -100
"chr20" 18922001 18923000 "*" 3.05311331771918e-12 8.34639368772922e-12 100
"chr20" 18997001 18998000 "*" 1.81691733547318e-06 2.22403164521072e-06
-51.1627906976744
"chr20" 19005001 19006000 "*" 4.99900121297969e-12 1.32921056339624e-11 -100
"chr20" 19031001 19032000 "*" 5.27009225148589e-09 9.20114230711386e-09
62.0689655172414
"chr20" 19058001 19059000 "*" 6.24654217240561e-10 1.2462108191303e-09 -100
"chr20" 19192001 19193000 "*" 0 0 59.5091146127703
"chr20" 19193001 19194000 "*" 0 0 59.0573054871282
"chr20" 19194001 19195000 "*" 4.65637306490407e-10 9.46720524046618e-10 100
"chr20" 19213001 19214000 "*" 8.65973959207622e-15 3.24130830964561e-14 100
"chr20" 19225001 19226000 "*" 1.26565424807268e-14 4.63923203146481e-14 -100
"chr20" 19271001 19272000 "*" 7.0006535457523e-08 1.03695832512054e-07 -100
"chr20" 19309001 19310000 "*" 0 0 78.5714285714286
"chr20" 19411001 19412000 "*" 1.50138574905867e-08 2.45599831107531e-08
-76.1904761904762
"chr20" 19497001 19498000 "*" 3.34128893442198e-08 5.19772864618523e-08 100
"chr20" 19506001 19507000 "*" 1.04017927604616e-09 2.00739718149955e-09
-60.5263157894737
"chr20" 19633001 19634000 "*" 0 0 -100
"chr20" 19638001 19639000 "*" 0 0 -62.1621621621622
"chr20" 19677001 19678000 "*" 1.11022302462516e-16 5.03662826618488e-16 -100
"chr20" 19870001 19871000 "*" 0 0 78.2608695652174
"chr20" 19909001 19910000 "*" 5.86063637508794e-07 7.68390936108992e-07
-62.9032258064516
"chr20" 19912001 19913000 "*" 0 0 100
"chr20" 19945001 19946000 "*" 2.44756081845265e-05 2.52637134962309e-05
-64.5161290322581
"chr20" 19977001 19978000 "*" 0 0 -54.2168674698795
"chr20" 20212001 20213000 "*" 1.07882591748876e-11 2.74553537455803e-11 -100
"chr20" 20335001 20336000 "*" 3.58120389308825e-05 3.60126966183746e-05
61.5384615384615
"chr20" 20349001 20350000 "*" 0 0 53.785022371391
"chr20" 20353001 20354000 "*" 6.25046514546312e-09 1.08158837586659e-08
-90.3225806451613
"chr20" 20360001 20361000 "*" 0 0 100
"chr20" 20383001 20384000 "*" 0 0 -100
"chr20" 20528001 20529000 "*" 2.00227675550835e-08 3.20459176617961e-08 -100
"chr20" 20637001 20638000 "*" 3.95353005888666e-09 7.03928529283733e-09 100
"chr20" 20664001 20665000 "*" 1.11022302462516e-16 5.03662826618488e-16 -100
"chr20" 20773001 20774000 "*" 0 0 100
"chr20" 20785001 20786000 "*" 3.44294259768674e-09 6.18657759024717e-09
-54.5454545454545
"chr20" 20804001 20805000 "*" 3.17619319734064e-09 5.73171036802447e-09
66.6666666666667
"chr20" 20813001 20814000 "*" 6.24654217240561e-10 1.2462108191303e-09 -100
"chr20" 20871001 20872000 "*" 3.13902409559974e-05 3.18712470329053e-05
56.5217391304348

"chr20" 20875001 20876000 "*" 2.29457564060453e-10 4.87242101222392e-10
-90.9090909090909
"chr20" 21010001 21011000 "*" 1.61237689866311e-12 4.56615813671789e-12 -100
"chr20" 21056001 21057000 "*" 3.61932706027801e-14 1.25777907157994e-13 100
"chr20" 21081001 21082000 "*" 0 0 52.724556706631
"chr20" 21202001 21203000 "*" 5.01025332333427e-10 1.01358031135889e-09 -100
"chr20" 21247001 21248000 "*" 1.26565424807268e-14 4.63923203146481e-14 100
"chr20" 21487001 21488000 "*" 0 0 64.1071205069319
"chr20" 21495001 21496000 "*" 0 0 72.2757598220904
"chr20" 21561001 21562000 "*" 6.70463684571132e-13 1.98922584795814e-12 70
"chr20" 21650001 21651000 "*" 0 0 100
"chr20" 21651001 21652000 "*" 1.60982338570648e-14 5.83411082284088e-14 -75
"chr20" 21672001 21673000 "*" 2.0698628067084e-07 2.87958098902623e-07
-50.9803921568627
"chr20" 21697001 21698000 "*" 2.64951482975562e-09 4.82186011375752e-09 100
"chr20" 21812001 21813000 "*" 2.02327044007689e-12 5.65300384792621e-12 -100
"chr20" 21843001 21844000 "*" 0 0 -76.3800904977376
"chr20" 22141001 22142000 "*" 6.32827124036339e-15 2.40666772484995e-14 -100
"chr20" 22402001 22403000 "*" 1.67299207820548e-08 2.71377429441055e-08 -100
"chr20" 22439001 22440000 "*" 0 0 100
"chr20" 22483001 22484000 "*" 1.08278107591175e-08 1.80775706443205e-08
-52.0547945205479
"chr20" 22484001 22485000 "*" 0 0 -100
"chr20" 22491001 22492000 "*" 7.88258347483861e-15 2.96145301916012e-14 -100
"chr20" 22564001 22565000 "*" 4.44089209850063e-16 1.91071758245033e-15
69.754431851206
"chr20" 22565001 22566000 "*" 0 0 54.0007012622721
"chr20" 22601001 22602000 "*" 1.33226762955019e-15 5.48133041560118e-15 -100
"chr20" 22785001 22786000 "*" 0 0 100
"chr20" 22814001 22815000 "*" 1.39779633423487e-07 1.98463037958589e-07 100
"chr20" 22957001 22958000 "*" 5.55111512312578e-16 2.36485094870365e-15 -100
"chr20" 22990001 22991000 "*" 5.794809077031e-12 1.52911192757929e-11 -100
"chr20" 23020001 23021000 "*" 0 0 100
"chr20" 23075001 23076000 "*" 2.22044604925031e-16 9.81641919380259e-16
-72.2222222222222
"chr20" 23078001 23079000 "*" 0 0 100
"chr20" 23110001 23111000 "*" 2.33028818463765e-10 4.93719283454501e-10 100
"chr20" 23128001 23129000 "*" 1.30340183090993e-13 4.22662705118657e-13 -100
"chr20" 23137001 23138000 "*" 1.20591092667155e-10 2.66075324129686e-10 100
"chr20" 23144001 23145000 "*" 0 0 85.8267716535433
"chr20" 23181001 23182000 "*" 0 0 54.6683250414594
"chr20" 23182001 23183000 "*" 2.43438050828715e-08 3.85481082003822e-08
-52.1739130434783
"chr20" 23216001 23217000 "*" 0 0 -100
"chr20" 23237001 23238000 "*" 1.65900401183361e-06 2.04236557510993e-06 -56
"chr20" 23271001 23272000 "*" 6.67788927882773e-05 6.43705713226498e-05
-56.3636363636364
"chr20" 23272001 23273000 "*" 2.67283972732457e-11 6.45686178157698e-11 -100
"chr20" 23290001 23291000 "*" 6.66133814775094e-16 2.81595744474255e-15 100
"chr20" 23308001 23309000 "*" 5.794809077031e-12 1.52911192757929e-11 -100
"chr20" 23326001 23327000 "*" 9.04570973681018e-10 1.76414389822173e-09 -100
"chr20" 23371001 23372000 "*" 3.597027969926e-07 4.84612690151399e-07

90.9090909090909
"chr20" 23472001 23473000 "*" 2.00227675550835e-08 3.20459176617961e-08 -100
"chr20" 23515001 23516000 "*" 0 0 -66.6666666666667
"chr20" 23583001 23584000 "*" 2.43005615629954e-09 4.45014222280278e-09 -100
"chr20" 23586001 23587000 "*" 1.04448884208352e-07 1.51208292669393e-07
-66.1016949152542
"chr20" 23610001 23611000 "*" 1.42821350301858e-08 2.342872909134e-08
-51.8518518518519
"chr20" 23614001 23615000 "*" 5.44009282066327e-15 2.08486137681812e-14 -100
"chr20" 23617001 23618000 "*" 1.57556190316654e-11 3.92048694223802e-11
-66.6666666666667
"chr20" 23703001 23704000 "*" 1.11022302462516e-16 5.03662826618488e-16 100
"chr20" 23729001 23730000 "*" 0 0 -100
"chr20" 23733001 23734000 "*" 5.9260374385417e-12 1.56171407287545e-11
78.2608695652174
"chr20" 23754001 23755000 "*" 6.47903952710749e-12 1.70126696132164e-11
-81.3559322033898
"chr20" 23779001 23780000 "*" 6.52733422867868e-12 1.71146951718423e-11 100
"chr20" 23806001 23807000 "*" 1.11022302462516e-16 5.03662826618488e-16 100
"chr20" 23898001 23899000 "*" 6.92287338566189e-11 1.57605630124247e-10 100
"chr20" 23909001 23910000 "*" 2.88779000712225e-11 6.92935809466068e-11 -100
"chr20" 23961001 23962000 "*" 1.4432899320127e-15 5.90750815956055e-15
50.5747126436782
"chr20" 23974001 23975000 "*" 0 0 100
"chr20" 23997001 23998000 "*" 6.27479350343574e-05 6.07659167366224e-05
51.4563106796116
"chr20" 23999001 24000000 "*" 1.11022302462516e-16 5.03662826618488e-16 100
"chr20" 24069001 24070000 "*" 2.4535928844216e-14 8.69144396197119e-14 -100
"chr20" 24077001 24078000 "*" 2.13548900784843e-09 3.94614299128544e-09
-50.9027373325568
"chr20" 24093001 24094000 "*" 1.39779633423487e-07 1.98463037958589e-07 -100
"chr20" 24116001 24117000 "*" 2.79079925835646e-09 5.06660143341042e-09
-51.0144927536232
"chr20" 24215001 24216000 "*" 1.22124532708767e-15 5.03921984217778e-15 100
"chr20" 24222001 24223000 "*" 1.11022302462516e-16 5.03662826618488e-16
64.7058823529412
"chr20" 24305001 24306000 "*" 8.41097733683682e-08 1.23270195074072e-07
61.9047619047619
"chr20" 24403001 24404000 "*" 0 0 -100
"chr20" 24448001 24449000 "*" 1.25284005392245e-10 2.75649590272477e-10 100
"chr20" 24482001 24483000 "*" 1.12376774552558e-12 3.24761818527664e-12 -100
"chr20" 24487001 24488000 "*" 1.37828859436695e-10 3.01445544267661e-10 100
"chr20" 24562001 24563000 "*" 0 0 -75.8064516129032
"chr20" 24640001 24641000 "*" 1.65027954746044e-07 2.32243498393663e-07
80.7017543859649
"chr20" 24683001 24684000 "*" 0 0 100
"chr20" 24710001 24711000 "*" 3.23407967073308e-13 1.00020209365026e-12
-53.7199434229137
"chr20" 24715001 24716000 "*" 5.77315972805081e-13 1.72829442922152e-12
56.4516129032258
"chr20" 24751001 24752000 "*" 2.70339306496226e-13 8.42355472617862e-13 -100
"chr20" 24752001 24753000 "*" 0 0 100

"chr20" 24757001 24758000 "*" 0 0 100
"chr20" 24763001 24764000 "*" 1.23416250628061e-05 1.33334219460316e-05 61.2244897959184
"chr20" 24771001 24772000 "*" 0 0 -100
"chr20" 24777001 24778000 "*" 2.13922224379104e-07 2.96983147907201e-07 -56
"chr20" 24780001 24781000 "*" 0 0 100
"chr20" 24785001 24786000 "*" 5.39579492198072e-12 1.42979302662116e-11 -56.8965517241379
"chr20" 24803001 24804000 "*" 2.43005615629954e-09 4.45014222280278e-09 -100
"chr20" 24823001 24824000 "*" 6.70841160399505e-12 1.75486545176836e-11 -100
"chr20" 24840001 24841000 "*" 1.22604442154284e-08 2.0267374786427e-08 -75.8064516129032
"chr20" 24873001 24874000 "*" 0 0 53.5284841316775
"chr20" 24890001 24891000 "*" 2.35932440695308e-07 3.25820146356964e-07 60
"chr20" 24989001 24990000 "*" 5.18656239179016e-07 6.84131900980763e-07 -57.1428571428571
"chr20" 25020001 25021000 "*" 1.61870516990348e-13 5.19414948860765e-13 82.6086956521739
"chr20" 25052001 25053000 "*" 1.93720595120794e-11 4.76309924836636e-11 -100
"chr20" 25113001 25114000 "*" 0 0 97.2972972972973
"chr20" 25127001 25128000 "*" 0 0 -70.1923076923077
"chr20" 25236001 25237000 "*" 2.62873611767134e-10 5.53968123011938e-10 76.4705882352941
"chr20" 25250001 25251000 "*" 2.3990809339125e-12 6.65441738642456e-12 100
"chr20" 25290001 25291000 "*" 0 0 -66.4798206278027
"chr20" 25334001 25335000 "*" 1.51323398256409e-13 4.86995056237989e-13 100
"chr20" 25490001 25491000 "*" 1.14130926931466e-13 3.7276674571693e-13 100
"chr20" 25527001 25528000 "*" 6.93112234273485e-13 2.05218842007357e-12 100
"chr20" 25605001 25606000 "*" 0 0 -100
"chr20" 25844001 25845000 "*" 2.22044604925031e-16 9.81641919380259e-16 66.0596667958156
"chr20" 25889001 25890000 "*" 7.99844594256971e-06 8.89173797399106e-06 54.5454545454545
"chr20" 26256001 26257000 "*" 7.0006535457523e-08 1.03695832512054e-07 100
"chr20" 29517001 29518000 "*" 1.92813588657526e-05 2.02169795190732e-05 75
"chr20" 29534001 29535000 "*" 2.22044604925031e-16 9.81641919380259e-16 -51.3510726072607
"chr20" 29866001 29867000 "*" 1.92946192356658e-09 3.59193011611483e-09 -100
"chr20" 29874001 29875000 "*" 1.49281698114123e-11 3.72570777374921e-11 -65.9090909090909
"chr20" 29920001 29921000 "*" 2.6215245296779e-07 3.59842941619008e-07 56.5217391304348
"chr20" 29981001 29982000 "*" 1.30340183090993e-13 4.22662705118657e-13 -100
"chr20" 30034001 30035000 "*" 2.00443736941835e-07 2.79303416105452e-07 -69.0476190476191
"chr20" 30064001 30065000 "*" 0 0 -89.4230769230769
"chr20" 30091001 30092000 "*" 0 0 100
"chr20" 30143001 30144000 "*" 2.00227675550835e-08 3.20459176617961e-08 -100
"chr20" 30214001 30215000 "*" 9.71063229826541e-11 2.16781365050398e-10 -100
"chr20" 30250001 30251000 "*" 2.22044604925031e-16 9.81641919380259e-16 -94.4444444444444
"chr20" 30252001 30253000 "*" 0 0 100

"chr20" 30309001 30310000 "*" 0 0 -100
"chr20" 30411001 30412000 "*" 1.59955722778271e-07 2.25516334443401e-07
52.1428571428571
"chr20" 30412001 30413000 "*" 0 0 89.7959183673469
"chr20" 30420001 30421000 "*" 2.00227675550835e-08 3.20459176617961e-08 -100
"chr20" 30440001 30441000 "*" 2.70339306496226e-13 8.42355472617862e-13 -100
"chr20" 30449001 30450000 "*" 7.60460893851622e-05 7.26527774072396e-05
54.6116504854369
"chr20" 30457001 30458000 "*" 0 0 66.920281626164
"chr20" 30458001 30459000 "*" 0 0 77.9623123602683
"chr20" 30489001 30490000 "*" 8.25450818808804e-13 2.42473843346228e-12 100
"chr20" 30513001 30514000 "*" 1.40872646881007e-10 3.07521971832011e-10
87.9598662207358
"chr20" 30562001 30563000 "*" 2.18713935851156e-14 7.80893646430581e-14 100
"chr20" 30606001 30607000 "*" 0 0 59.8729096989967
"chr20" 30659001 30660000 "*" 5.45395950624084e-11 1.26067465997336e-10
57.6923076923077
"chr20" 30798001 30799000 "*" 1.13268638823882e-05 1.23088634379463e-05 -70
"chr20" 30980001 30981000 "*" 0 0 -73.75
"chr20" 31025001 31026000 "*" 7.7715611723761e-16 3.26213507634405e-15 -100
"chr20" 31105001 31106000 "*" 5.62421294025839e-06 6.40036130723781e-06
-56.8047337278106
"chr20" 31150001 31151000 "*" 0 0 87.8048780487805
"chr20" 31176001 31177000 "*" 9.65729496371637e-10 1.87255912493232e-09 100
"chr20" 31240001 31241000 "*" 2.33028818463765e-10 4.93719283454501e-10 100
"chr20" 31496001 31497000 "*" 0 0 61.3166026781534
"chr20" 31531001 31532000 "*" 0 0 100
"chr20" 31549001 31550000 "*" 0 0 64.8
"chr20" 31570001 31571000 "*" 2.26731966534999e-11 5.51863535220454e-11
-90.9090909090909
"chr20" 31576001 31577000 "*" 0 0 -75.609756097561
"chr20" 31597001 31598000 "*" 2.15125472990962e-10 4.58126659943874e-10 -100
"chr20" 31602001 31603000 "*" 1.07436282092976e-12 3.11260666494848e-12
-57.1428571428571
"chr20" 31620001 31621000 "*" 6.88338275267597e-15 2.60518659957509e-14 100
"chr20" 31652001 31653000 "*" 0 0 100
"chr20" 31656001 31657000 "*" 3.1863400806742e-14 1.11403950494973e-13
53.5714285714286
"chr20" 31657001 31658000 "*" 5.00377517198558e-13 1.51224212502994e-12
76.1904761904762
"chr20" 31693001 31694000 "*" 3.52704532247117e-11 8.35581292290093e-11 100
"chr20" 31699001 31700000 "*" 3.69720567845455e-05 3.7093341644624e-05 62.5
"chr20" 31720001 31721000 "*" 5.295763827462e-14 1.80214784640623e-13 100
"chr20" 31721001 31722000 "*" 2.34257058195908e-14 8.33889706074077e-14 -100
"chr20" 31765001 31766000 "*" 5.55111512312578e-16 2.36485094870365e-15 -100
"chr20" 31770001 31771000 "*" 1.17017506795491e-13 3.81716338420629e-13
-69.4444444444444
"chr20" 31781001 31782000 "*" 8.43347613965761e-12 2.17859001990012e-11 100
"chr20" 31802001 31803000 "*" 0 0 -100
"chr20" 31877001 31878000 "*" 1.07185123543463e-06 1.35516016195071e-06
58.7301587301587
"chr20" 31890001 31891000 "*" 5.33790495471642e-07 7.03071689168268e-07

-66.6666666666667
"chr20" 31915001 31916000 "*" 6.62958576924666e-12 1.73620572057889e-11 100
"chr20" 31921001 31922000 "*" 6.75630401625327e-08 1.00725834595596e-07
50.9358288770053
"chr20" 32011001 32012000 "*" 1.52466927971773e-12 4.33322475981408e-12 -100
"chr20" 32063001 32064000 "*" 5.72875080706581e-14 1.93967931988877e-13 100
"chr20" 32077001 32078000 "*" 0 0 75
"chr20" 32239001 32240000 "*" 2.22044604925031e-16 9.81641919380259e-16 100
"chr20" 32261001 32262000 "*" 4.47419878923938e-14 1.53914905710088e-13
69.2307692307692
"chr20" 32262001 32263000 "*" 0 0 -50.6846699399891
"chr20" 32307001 32308000 "*" 1.10800257857591e-13 3.62754915982774e-13
74.1935483870968
"chr20" 32308001 32309000 "*" 0 0 66.7753828701078
"chr20" 32336001 32337000 "*" 1.34559030584569e-13 4.35581312463895e-13 100
"chr20" 32398001 32399000 "*" 0 0 100
"chr20" 32414001 32415000 "*" 6.92287338566189e-11 1.57605630124247e-10 100
"chr20" 32448001 32449000 "*" 2.08995487582797e-10 4.46944006972663e-10 100
"chr20" 32476001 32477000 "*" 1.67299207820548e-08 2.71377429441055e-08 -100
"chr20" 32542001 32543000 "*" 2.69007038866675e-13 8.38831022628993e-13 100
"chr20" 32549001 32550000 "*" 1.65513770644665e-06 2.0381472699397e-06
64.7058823529412
"chr20" 32600001 32601000 "*" 2.55351295663786e-15 1.01678090381833e-14 100
"chr20" 32671001 32672000 "*" 0 0 -69.7674418604651
"chr20" 32701001 32702000 "*" 7.08893914920239e-05 6.80387209642958e-05
53.8461538461538
"chr20" 32804001 32805000 "*" 0 0 -63.3004926108374
"chr20" 32843001 32844000 "*" 9.93683158201364e-07 1.26156135328477e-06
-56.5217391304348
"chr20" 32893001 32894000 "*" 7.105427357601e-15 2.68362283258525e-14 -100
"chr20" 33103001 33104000 "*" 0 0 100
"chr20" 33105001 33106000 "*" 1.62203583897735e-13 5.20463679134111e-13 56
"chr20" 33126001 33127000 "*" 1.25093098791718e-05 1.35030371915773e-05
-65.7894736842105
"chr20" 33157001 33158000 "*" 1.1988837312904e-06 1.5057940841428e-06
-58.3333333333333
"chr20" 33224001 33225000 "*" 3.597027969926e-07 4.84612690151399e-07
90.9090909090909
"chr20" 33461001 33462000 "*" 3.1298308389438e-10 6.51565257264151e-10
80.1587301587302
"chr20" 33465001 33466000 "*" 1.67299207820548e-08 2.71377429441055e-08 100
"chr20" 33598001 33599000 "*" 1.09008912785313e-07 1.57413554003421e-07
52.1739130434783
"chr20" 33617001 33618000 "*" 0 0 100
"chr20" 33759001 33760000 "*" 0 0 -100
"chr20" 33814001 33815000 "*" 0 0 51.1175898931001
"chr20" 33815001 33816000 "*" 0 0 74.2116704805492
"chr20" 33828001 33829000 "*" 2.64951482975562e-09 4.82186011375752e-09 -100
"chr20" 33837001 33838000 "*" 4.13590561021415e-07 5.52580636132098e-07 -70
"chr20" 33865001 33866000 "*" 0 0 -79.7316384180791
"chr20" 33867001 33868000 "*" 4.44089209850063e-16 1.91071758245033e-15
96.551724137931

"chr20" 33873001 33874000 "*" 4.68434735778622e-07 6.21336632612129e-07
70.1298701298701
"chr20" 33891001 33892000 "*" 1.43773881688958e-13 4.63718846735935e-13
88.6363636363636
"chr20" 34026001 34027000 "*" 6.24654217240561e-10 1.2462108191303e-09 -100
"chr20" 34028001 34029000 "*" 6.70841160399505e-12 1.75486545176836e-11 -100
"chr20" 34082001 34083000 "*" 1.15502918340837e-09 2.21275873837915e-09 92
"chr20" 34184001 34185000 "*" 6.59550958292954e-09 1.13456194382773e-08 100
"chr20" 34201001 34202000 "*" 3.10862446895044e-15 1.22466437093774e-14 100
"chr20" 34205001 34206000 "*" 0 0 52.4122807017544
"chr20" 34207001 34208000 "*" 0 0 74.8031496062992
"chr20" 34311001 34312000 "*" 1.67299207820548e-08 2.71377429441055e-08 100
"chr20" 34340001 34341000 "*" 5.48638023900594e-10 1.10374357336421e-09 100
"chr20" 34359001 34360000 "*" 0 0 62.7760252365931
"chr20" 34490001 34491000 "*" 1.48487888651516e-11 3.70689457767456e-11 -100
"chr20" 34494001 34495000 "*" 0.00026590838562468 0.000232535857460809
-53.5714285714286
"chr20" 34548001 34549000 "*" 0.000591775622736268 0.000487833560499899
-51.7241379310345
"chr20" 34600001 34601000 "*" 1.38590361409285e-10 3.02706178892116e-10 100
"chr20" 34638001 34639000 "*" 0 0 -80
"chr20" 34679001 34680000 "*" 0 0 100
"chr20" 34742001 34743000 "*" 0 0 -53.7686930164128
"chr20" 34759001 34760000 "*" 1.12458486967171e-09 2.15740815043925e-09 100
"chr20" 34829001 34830000 "*" 3.23373829846929e-06 3.81325174359463e-06
-66.6666666666667
"chr20" 34863001 34864000 "*" 0 0 -92.7083333333333
"chr20" 34867001 34868000 "*" 6.93112234273485e-13 2.05218842007357e-12 100
"chr20" 34964001 34965000 "*" 1.52566070887872e-09 2.87642564232045e-09
62.9213483146067
"chr20" 34971001 34972000 "*" 8.58946247461745e-12 2.213554983903e-11
77.0833333333333
"chr20" 34981001 34982000 "*" 4.03652517699804e-06 4.6892927905593e-06
-54.5454545454545
"chr20" 34989001 34990000 "*" 5.24174958993484e-09 9.1537662620124e-09
-50.733137829912
"chr20" 34992001 34993000 "*" 6.66133814775094e-16 2.81595744474255e-15 -100
"chr20" 35003001 35004000 "*" 1.20966398009514e-08 2.00140491666149e-08 90
"chr20" 35020001 35021000 "*" 8.5757788870211e-08 1.25531089622924e-07
-86.4285714285714
"chr20" 35036001 35037000 "*" 0 0 71.551724137931
"chr20" 35126001 35127000 "*" 1.66992206362959e-06 2.05480552961511e-06
59.2592592592593
"chr20" 35147001 35148000 "*" 2.33028818463765e-10 4.93719283454501e-10 100
"chr20" 35174001 35175000 "*" 5.55111512312578e-16 2.36485094870365e-15 -100
"chr20" 35208001 35209000 "*" 2.455369241261e-12 6.8003688121118e-12 -100
"chr20" 35238001 35239000 "*" 5.72875080706581e-14 1.93967931988877e-13 -100
"chr20" 35241001 35242000 "*" 3.6892711108294e-13 1.13266255090498e-12 100
"chr20" 35249001 35250000 "*" 1.54630752646767e-11 3.85079502648228e-11 -100
"chr20" 35260001 35261000 "*" 1.54630752646767e-11 3.85079502648228e-11 100
"chr20" 35261001 35262000 "*" 4.05342426290645e-13 1.23654269958768e-12
66.6666666666667

"chr20" 35263001 35264000 "*" 1.79703474323389e-10 3.87732058944655e-10
-63.4037819799778
"chr20" 35278001 35279000 "*" 1.35890187991095e-11 3.40841971363279e-11 -100
"chr20" 35437001 35438000 "*" 0.000209763311656563 0.000186660769941092
-52.3809523809524
"chr20" 35508001 35509000 "*" 0.000126887439664713 0.000117029410540844
-54.5454545454545
"chr20" 35653001 35654000 "*" 4.08209022140227e-11 9.57770690255255e-11 100
"chr20" 35799001 35800000 "*" 1.12634901405784e-10 2.49563754426169e-10 100
"chr20" 35882001 35883000 "*" 0 0 100
"chr20" 35974001 35975000 "*" 0 0 -96.2059620596206
"chr20" 36050001 36051000 "*" 2.08550269498176e-07 2.89983958475591e-07
57.6923076923077
"chr20" 36072001 36073000 "*" 5.72126176556775e-05 5.57465732173144e-05
68.4210526315789
"chr20" 36093001 36094000 "*" 1.03886985030055e-05 1.13522277663458e-05
70.5882352941177
"chr20" 36100001 36101000 "*" 0 0 -100
"chr20" 36152001 36153000 "*" 1.12132525487141e-14 4.14352271980498e-14
59.4594594594595
"chr20" 36157001 36158000 "*" 1.43329792479108e-13 4.62322269077954e-13
72.5806451612903
"chr20" 36209001 36210000 "*" 0 0 -80.3030303030303
"chr20" 36219001 36220000 "*" 1.11022302462516e-15 4.59817122935606e-15
83.8709677419355
"chr20" 36232001 36233000 "*" 2.08995487582797e-10 4.46944006972663e-10 -100
"chr20" 36260001 36261000 "*" 1.765254609154e-14 6.36724611765475e-14
-77.0833333333333
"chr20" 36273001 36274000 "*" 2.65909381849116e-05 2.73008516066207e-05
-61.2903225806452
"chr20" 36281001 36282000 "*" 0 0 84.3137254901961
"chr20" 36480001 36481000 "*" 1.13140401492018e-08 1.87801380787728e-08 100
"chr20" 36512001 36513000 "*" 0.000174588517416696 0.000157342656028109
-58.6206896551724
"chr20" 36563001 36564000 "*" 6.16959703788611e-05 5.98208095078624e-05 -70
"chr20" 36569001 36570000 "*" 1.67299207820548e-08 2.71377429441055e-08 -100
"chr20" 36686001 36687000 "*" 6.37490060739765e-13 1.89496793587877e-12 -100
"chr20" 36748001 36749000 "*" 1.5477064074787e-11 3.85404852168246e-11
-72.7272727272727
"chr20" 36847001 36848000 "*" 1.55366941534396e-10 3.37159429902653e-10
83.0508474576271
"chr20" 36849001 36850000 "*" 0 0 50.3937007874016
"chr20" 36865001 36866000 "*" 0.00012822881503527 0.000118164639635152
-55.2631578947368
"chr20" 36874001 36875000 "*" 1.87978521637433e-11 4.63681633784719e-11
91.9540229885057
"chr20" 36884001 36885000 "*" 5.44009282066327e-15 2.08486137681812e-14 100
"chr20" 36911001 36912000 "*" 9.65729496371637e-10 1.87255912493232e-09 100
"chr20" 36932001 36933000 "*" 2.22044604925031e-15 8.90449334476539e-15
80.4270462633452
"chr20" 36957001 36958000 "*" 1.43708190680769e-07 2.03759937148811e-07
-55.0649350649351

"chr20" 36965001 36966000 "*" 5.33967933202817e-08 8.0673341162609e-08
-67.3469387755102
"chr20" 36966001 36967000 "*" 2.15125472990962e-10 4.58126659943874e-10 100
"chr20" 36995001 36996000 "*" 3.90335334943259e-07 5.23262271935061e-07 65.625
"chr20" 37050001 37051000 "*" 7.07564340451938e-10 1.39906965894392e-09
-54.7126436781609
"chr20" 37064001 37065000 "*" 0 0 -78.8461538461538
"chr20" 37075001 37076000 "*" 0 0 -100
"chr20" 37081001 37082000 "*" 0 0 -75.7575757575758
"chr20" 37262001 37263000 "*" 9.43684273835288e-07 1.20197202396502e-06
-59.1836734693878
"chr20" 37273001 37274000 "*" 8.04728939041155e-09 1.36871529290745e-08
65.3061224489796
"chr20" 37274001 37275000 "*" 0 0 64.7058823529412
"chr20" 37299001 37300000 "*" 2.43005615629954e-09 4.45014222280278e-09 -100
"chr20" 37304001 37305000 "*" 3.4715113006456e-09 6.23562839508134e-09
76.5957446808511
"chr20" 37305001 37306000 "*" 1.19904086659517e-14 4.40901388691796e-14 100
"chr20" 37344001 37345000 "*" 0 0 100
"chr20" 37433001 37434000 "*" 1.32444702649614e-07 1.89065803623889e-07
-50.5747126436782
"chr20" 37436001 37437000 "*" 4.55725856837175e-05 4.50963674763679e-05 -56
"chr20" 37458001 37459000 "*" 8.00994964211821e-06 8.90333967966777e-06
-72.7272727272727
"chr20" 37496001 37497000 "*" 2.4049820690486e-08 3.81064634011469e-08 -60
"chr20" 37507001 37508000 "*" 0 0 100
"chr20" 37518001 37519000 "*" 0 0 58.7155963302752
"chr20" 37537001 37538000 "*" 6.50295179486227e-05 6.28382569664639e-05
72.7272727272727
"chr20" 37561001 37562000 "*" 2.08785967781289e-06 2.53403795774669e-06
-72.7272727272727
"chr20" 37626001 37627000 "*" 5.33431854243815e-10 1.07644989448352e-09
-69.0476190476191
"chr20" 37678001 37679000 "*" 0 0 58.955223880597
"chr20" 37684001 37685000 "*" 6.30384633382164e-13 1.87665599796749e-12 -100
"chr20" 37688001 37689000 "*" 1.96509475358653e-13 6.23868565133654e-13 100
"chr20" 37700001 37701000 "*" 4.52958795027492e-07 6.02025166595169e-07
63.8888888888889
"chr20" 37738001 37739000 "*" 0 0 90
"chr20" 37748001 37749000 "*" 6.59550958292954e-09 1.13456194382773e-08 100
"chr20" 37779001 37780000 "*" 1.87263111511848e-05 1.96842454397447e-05
61.2903225806452
"chr20" 37839001 37840000 "*" 3.33066907387547e-16 1.4495649018245e-15 100
"chr20" 37926001 37927000 "*" 1.04966035863185e-10 2.33489999443519e-10
58.0586080586081
"chr20" 38024001 38025000 "*" 2.1094237467878e-15 8.47434540879246e-15 -100
"chr20" 38244001 38245000 "*" 9.65338919911574e-13 2.80678910656113e-12 100
"chr20" 38571001 38572000 "*" 9.88628795889346e-05 9.27525037076316e-05
-54.3859649122807
"chr20" 38880001 38881000 "*" 0 0 -100
"chr20" 38891001 38892000 "*" 0 0 100
"chr20" 39026001 39027000 "*" 6.59550958292954e-09 1.13456194382773e-08 100

"chr20" 39062001 39063000 "*" 0.000471727282945977 0.000395504194557835
-53.8461538461538
"chr20" 39273001 39274000 "*" 2.08814465718632e-09 3.86232567960463e-09 100
"chr20" 39274001 39275000 "*" 1.53210777398272e-14 5.5628583449216e-14 100
"chr20" 39276001 39277000 "*" 1.20281562487889e-12 3.46585342181251e-12 100
"chr20" 39296001 39297000 "*" 0 0 -60.0903614457831
"chr20" 39313001 39314000 "*" 1.11022302462516e-16 5.03662826618488e-16 -100
"chr20" 39318001 39319000 "*" 0 0 75.1500600240096
"chr20" 39341001 39342000 "*" 0 0 100
"chr20" 39391001 39392000 "*" 3.34128893442198e-08 5.19772864618523e-08 100
"chr20" 39431001 39432000 "*" 2.69207922398707e-07 3.69017444205844e-07 68.75
"chr20" 39444001 39445000 "*" 0 0 100
"chr20" 39566001 39567000 "*" 3.88022947106492e-13 1.1867462770515e-12 100
"chr20" 39590001 39591000 "*" 3.80553856649257e-07 5.10864147295928e-07
68.4931506849315
"chr20" 39637001 39638000 "*" 1.39680959354571e-07 1.98463037958589e-07
-66.6666666666667
"chr20" 39649001 39650000 "*" 1.93720595120794e-11 4.76309924836636e-11 -100
"chr20" 39978001 39979000 "*" 3.56775498033812e-10 7.35898629809829e-10 100
"chr20" 40002001 40003000 "*" 2.27105001471273e-11 5.52739236233349e-11
52.6315789473684
"chr20" 40005001 40006000 "*" 2.07072226032778e-09 3.84161227320661e-09
-61.1111111111111
"chr20" 40010001 40011000 "*" 0 0 62.0253164556962
"chr20" 40030001 40031000 "*" 9.08108033215171e-11 2.03407753381119e-10 -100
"chr20" 40155001 40156000 "*" 1.65267703956529e-05 1.75228091743656e-05
64.2857142857143
"chr20" 40744001 40745000 "*" 1.01037411770477e-09 1.95240745173124e-09
-85.4166666666667
"chr20" 40834001 40835000 "*" 8.8342340305303e-06 9.75817539917938e-06
-78.5714285714286
"chr20" 41103001 41104000 "*" 0.000139451556673498 0.000127724714298755
58.3333333333333
"chr20" 41438001 41439000 "*" 7.31599500203295e-07 9.46261816963704e-07
65.2173913043478
"chr20" 41534001 41535000 "*" 8.84503581488616e-12 2.27686703812906e-11
56.5217391304348
"chr20" 41580001 41581000 "*" 1.15972060166225e-06 1.45972698357591e-06
64.1025641025641
"chr20" 41640001 41641000 "*" 1.20591092667155e-10 2.66075324129686e-10 100
"chr20" 41722001 41723000 "*" 0 0 -100
"chr20" 41753001 41754000 "*" 2.43005615629954e-09 4.45014222280278e-09 -100
"chr20" 41813001 41814000 "*" 3.40848437252461e-08 5.29435815243835e-08
65.9090909090909
"chr20" 41846001 41847000 "*" 2.800398140268e-06 3.33469699199634e-06 -60
"chr20" 41955001 41956000 "*" 2.69084754478399e-12 7.41487347763769e-12 -100
"chr20" 41992001 41993000 "*" 5.75794967261345e-12 1.52188950945173e-11
-51.9083969465649
"chr20" 41994001 41995000 "*" 0 0 100
"chr20" 42052001 42053000 "*" 1.5277158427196e-09 2.87642564232045e-09 100
"chr20" 42056001 42057000 "*" 3.04162505615491e-06 3.60157948723361e-06
-59.1549295774648

"chr20" 42157001 42158000 "*" 1.13748691576987e-08 1.88752888120012e-08
-52.9411764705882
"chr20" 42167001 42168000 "*" 8.030686559235e-05 7.64432131329181e-05 -68.75
"chr20" 42194001 42195000 "*" 8.34186798015502e-06 9.24761438950058e-06
60.6060606060606
"chr20" 42305001 42306000 "*" 0 0 94.9640287769784
"chr20" 42311001 42312000 "*" 5.88811444046655e-10 1.18036899963715e-09
-73.2142857142857
"chr20" 42371001 42372000 "*" 5.22962956606143e-07 6.89561826220703e-07
-71.1864406779661
"chr20" 42428001 42429000 "*" 0 0 -100
"chr20" 42429001 42430000 "*" 7.0006535457523e-08 1.03695832512054e-07 -100
"chr20" 42523001 42524000 "*" 1.5277158427196e-09 2.87642564232045e-09 100
"chr20" 42567001 42568000 "*" 0.000120483972759877 0.000111549020346707
-52.1739130434783
"chr20" 42578001 42579000 "*" 1.82520154545784e-09 3.40978610311475e-09
86.3013698630137
"chr20" 42582001 42583000 "*" 1.11022302462516e-16 5.03662826618488e-16 100
"chr20" 42599001 42600000 "*" 2.57682764015499e-13 8.0653464369776e-13 62.5
"chr20" 42611001 42612000 "*" 1.61592517144982e-10 3.50041000363085e-10
62.962962962963
"chr20" 42628001 42629000 "*" 9.5812247025151e-14 3.15667542866807e-13 100
"chr20" 42647001 42648000 "*" 0 0 100
"chr20" 42657001 42658000 "*" 2.46125342329151e-12 6.81553393059181e-12
62.5764993880049
"chr20" 42658001 42659000 "*" 3.05311331771918e-12 8.34639368772922e-12 100
"chr20" 42659001 42660000 "*" 2.13194424336649e-08 3.40031048932509e-08
61.8181818181818
"chr20" 42661001 42662000 "*" 2.15833528827147e-09 3.98588754314856e-09
52.0833333333333
"chr20" 42668001 42669000 "*" 1.8922016766898e-05 1.98739312700751e-05
50.3649635036496
"chr20" 42683001 42684000 "*" 2.49341289126903e-05 2.57048700295864e-05
64.1509433962264
"chr20" 42726001 42727000 "*" 0 0 67.0588235294118
"chr20" 42738001 42739000 "*" 4.35140812271584e-12 1.16680214202521e-11
-60.6060606060606
"chr20" 42741001 42742000 "*" 3.6700841921089e-08 5.65530226072258e-08 100
"chr20" 42760001 42761000 "*" 4.99020824662466e-11 1.15790792586157e-10 -100
"chr20" 42780001 42781000 "*" 3.42066490859327e-05 3.44923029230399e-05
-59.6153846153846
"chr20" 42795001 42796000 "*" 6.04438721296674e-11 1.38849255306602e-10 -100
"chr20" 42843001 42844000 "*" 4.08209022140227e-11 9.57770690255255e-11 -100
"chr20" 42848001 42849000 "*" 0 0 -98.0392156862745
"chr20" 42869001 42870000 "*" 6.4152538836737e-10 1.27477697728417e-09 -100
"chr20" 42904001 42905000 "*" 3.6700841921089e-08 5.65530226072258e-08 100
"chr20" 42919001 42920000 "*" 7.03992419914812e-12 1.83313429961219e-11 -100
"chr20" 42938001 42939000 "*" 9.80763359414993e-10 1.8980032424715e-09 -100
"chr20" 42954001 42955000 "*" 2.69007038866675e-13 8.38831022628993e-13 100
"chr20" 42972001 42973000 "*" 0 0 -100
"chr20" 42985001 42986000 "*" 0 0 -100
"chr20" 43017001 43018000 "*" 0 0 -80.6868131868132

"chr20" 43031001 43032000 "*" 2.55351295663786e-15 1.01678090381833e-14 -97.1698113207547
"chr20" 43055001 43056000 "*" 3.52704532247117e-11 8.35581292290093e-11 -100
"chr20" 43071001 43072000 "*" 2.22044604925031e-16 9.81641919380259e-16 100
"chr20" 43080001 43081000 "*" 6.92287338566189e-11 1.57605630124247e-10 -100
"chr20" 43089001 43090000 "*" 2.00227675550835e-08 3.20459176617961e-08 -100
"chr20" 43095001 43096000 "*" 1.49613654798486e-12 4.26244554429779e-12 -100
"chr20" 43096001 43097000 "*" 0 0 -100
"chr20" 43239001 43240000 "*" 1.11022302462516e-16 5.03662826618488e-16 -100
"chr20" 43270001 43271000 "*" 2.00227675550835e-08 3.20459176617961e-08 100
"chr20" 43330001 43331000 "*" 7.0006535457523e-08 1.03695832512054e-07 100
"chr20" 43394001 43395000 "*" 2.00227675550835e-08 3.20459176617961e-08 100
"chr20" 43401001 43402000 "*" 1.66533453693773e-15 6.7629186866784e-15 -100
"chr20" 43402001 43403000 "*" 2.84200885047881e-10 5.96020717471281e-10 76.1904761904762
"chr20" 43417001 43418000 "*" 6.90747459231034e-12 1.80140148623876e-11 -100
"chr20" 43420001 43421000 "*" 3.40125705378114e-10 7.03784073980162e-10 -100
"chr20" 43441001 43442000 "*" 1.26529786648177e-10 2.78280435481264e-10 -64.5161290322581
"chr20" 43450001 43451000 "*" 1.48087875295744e-10 3.22075573380618e-10 -100
"chr20" 43465001 43466000 "*" 5.08482145278322e-14 1.73432706170696e-13 100
"chr20" 43584001 43585000 "*" 2.95423767674441e-05 3.0118633488738e-05 76.9230769230769
"chr20" 43613001 43614000 "*" 7.57017337704724e-10 1.49151366838456e-09 -86.6666666666667
"chr20" 43629001 43630000 "*" 0 0 -100
"chr20" 43922001 43923000 "*" 0 0 83.5553255118472
"chr20" 43934001 43935000 "*" 0 0 100
"chr20" 43937001 43938000 "*" 3.68394204031119e-12 9.99162931596777e-12 89.3203883495146
"chr20" 43965001 43966000 "*" 3.92810339033822e-07 5.26410901634535e-07 -76
"chr20" 43976001 43977000 "*" 0 0 -55.1724137931034
"chr20" 44002001 44003000 "*" 0 0 52.112676056338
"chr20" 44034001 44035000 "*" 2.63677968348475e-13 8.23661048571723e-13 53.8888888888889
"chr20" 44039001 44040000 "*" 3.50630706691391e-08 5.43921383052272e-08 81.25
"chr20" 44048001 44049000 "*" 0 0 -100
"chr20" 44076001 44077000 "*" 1.67299207820548e-08 2.71377429441055e-08 -100
"chr20" 44099001 44100000 "*" 0 0 56.8577558787696
"chr20" 44238001 44239000 "*" 6.59550958292954e-09 1.13456194382773e-08 -100
"chr20" 44483001 44484000 "*" 3.04324343503026e-11 7.26599935673121e-11 -100
"chr20" 44530001 44531000 "*" 1.32149270839488e-05 1.42155827319109e-05 -54.5454545454545
"chr20" 44551001 44552000 "*" 9.80763359414993e-10 1.8980032424715e-09 -100
"chr20" 44591001 44592000 "*" 0 0 62.1359223300971
"chr20" 44679001 44680000 "*" 1.01234828298047e-07 1.46802558595138e-07 78.9473684210526
"chr20" 44680001 44681000 "*" 7.04750038460489e-05 6.76721492292335e-05 59.6153846153846
"chr20" 44741001 44742000 "*" 0 0 100
"chr20" 44751001 44752000 "*" 0 0 92.6829268292683
"chr20" 44761001 44762000 "*" 1.25122134875255e-13 4.06357992793329e-13

-83.3333333333333
"chr20" 44763001 44764000 "*" 0 0 -100
"chr20" 44774001 44775000 "*" 3.95353005888666e-09 7.03928529283733e-09 -100
"chr20" 44792001 44793000 "*" 1.5277158427196e-09 2.87642564232045e-09 100
"chr20" 44797001 44798000 "*" 2.55300891538468e-10 5.38867519330175e-10
60.5263157894737
"chr20" 44817001 44818000 "*" 9.08108033215171e-11 2.03407753381119e-10 -100
"chr20" 44822001 44823000 "*" 9.88315162357978e-05 9.27293342863802e-05
-54.2857142857143
"chr20" 44835001 44836000 "*" 1.36604061395929e-11 3.42333952202875e-11 100
"chr20" 44887001 44888000 "*" 0 0 100
"chr20" 44892001 44893000 "*" 0 0 100
"chr20" 44912001 44913000 "*" 5.01025332333427e-10 1.01358031135889e-09 100
"chr20" 44932001 44933000 "*" 2.42028619368284e-14 8.59499916624584e-14
65.5172413793103
"chr20" 44946001 44947000 "*" 3.33144622999271e-12 9.0713029318117e-12
-59.108734402852
"chr20" 44955001 44956000 "*" 2.88779000712225e-11 6.92935809466068e-11 -100
"chr20" 44957001 44958000 "*" 2.94277935353193e-11 7.05136611827262e-11
67.6470588235294
"chr20" 44984001 44985000 "*" 2.27595720048157e-13 7.16207285187952e-13 -60
"chr20" 45191001 45192000 "*" 3.2700897545368e-10 6.78798757234889e-10
66.6666666666667
"chr20" 45211001 45212000 "*" 4.01313771103418e-09 7.11882087237587e-09 100
"chr20" 45227001 45228000 "*" 3.26993987442847e-11 7.78252567137542e-11
-65.2777777777778
"chr20" 45264001 45265000 "*" 1.5277158427196e-09 2.87642564232045e-09 -100
"chr20" 45269001 45270000 "*" 6.93112234273485e-13 2.05218842007357e-12 100
"chr20" 45338001 45339000 "*" 0 0 55.8243298538259
"chr20" 45343001 45344000 "*" 0 0 55.7324840764331
"chr20" 45384001 45385000 "*" 7.67056418382595e-11 1.7359448580318e-10 -100
"chr20" 45394001 45395000 "*" 0 0 -100
"chr20" 45412001 45413000 "*" 2.79987144580218e-12 7.68634726811898e-12 100
"chr20" 45413001 45414000 "*" 1.5277158427196e-09 2.87642564232045e-09 100
"chr20" 45421001 45422000 "*" 0.000447727107318552 0.000376920426399982 60
"chr20" 45444001 45445000 "*" 2.52467619143992e-07 3.4735516511414e-07
53.6585365853659
"chr20" 45523001 45524000 "*" 0 0 60.2388683840396
"chr20" 45528001 45529000 "*" 1.07090780687713e-10 2.37962479641495e-10
-51.6129032258064
"chr20" 45571001 45572000 "*" 3.39622219236446e-10 7.03784073980162e-10
62.8571428571429
"chr20" 45580001 45581000 "*" 9.06273663336954e-07 1.15739767942151e-06 75
"chr20" 45605001 45606000 "*" 1.9373391779709e-13 6.15971322898373e-13 100
"chr20" 45618001 45619000 "*" 1.13140401492018e-08 1.87801380787728e-08 -100
"chr20" 45628001 45629000 "*" 8.98708536797299e-05 8.48863151558674e-05
62.0689655172414
"chr20" 45700001 45701000 "*" 0 0 66.9924532970432
"chr20" 45762001 45763000 "*" 0.000343975177368727 0.000295290538521052 56
"chr20" 45814001 45815000 "*" 3.7274627828765e-12 1.00985250136599e-11 100
"chr20" 45834001 45835000 "*" 2.00227675550835e-08 3.20459176617961e-08 -100
"chr20" 45985001 45986000 "*" 0 0 100

"chr20" 46001001 46002000 "*" 1.01034994037796e-07 1.4653060412968e-07 77.7777777777778
"chr20" 46037001 46038000 "*" 6.37490060739765e-13 1.89496793587877e-12 100
"chr20" 46270001 46271000 "*" 9.34807786734382e-14 3.0890138653016e-13 -76.0869565217391
"chr20" 46330001 46331000 "*" 1.07341802113581e-10 2.38494848023441e-10 -50.4322268326418
"chr20" 46369001 46370000 "*" 0 0 -54.668349238899
"chr20" 46372001 46373000 "*" 7.23432425075998e-11 1.64368563112802e-10 -58.6206896551724
"chr20" 46413001 46414000 "*" 0 0 72.5998333796168
"chr20" 46415001 46416000 "*" 0 0 71.1827956989247
"chr20" 46424001 46425000 "*" 0 0 -79.6203796203796
"chr20" 46427001 46428000 "*" 0 0 -94.4444444444444
"chr20" 46454001 46455000 "*" 1.55431223447522e-15 6.33705141101682e-15 53.9627659574468
"chr20" 46468001 46469000 "*" 8.32667268468867e-14 2.76905102014264e-13 86.6666666666667
"chr20" 46471001 46472000 "*" 3.61143759329829e-05 3.62939716999617e-05 53.4188034188034
"chr20" 46494001 46495000 "*" 1.96644922567657e-11 4.82930612417392e-11 -100
"chr20" 46541001 46542000 "*" 2.99059645825217e-06 3.54501296729526e-06 -63.4615384615385
"chr20" 46570001 46571000 "*" 9.34330390833793e-12 2.40031437270538e-11 81.25
"chr20" 46573001 46574000 "*" 4.46405532761318e-05 4.42311628182131e-05 52.3809523809524
"chr20" 46584001 46585000 "*" 7.0006535457523e-08 1.03695832512054e-07 -100
"chr20" 46609001 46610000 "*" 1.11022302462516e-16 5.03662826618488e-16 60
"chr20" 46647001 46648000 "*" 1.73527858748912e-12 4.89724700978558e-12 -78.5714285714286
"chr20" 46655001 46656000 "*" 3.33066907387547e-15 1.30941888440006e-14 100
"chr20" 46662001 46663000 "*" 6.30384633382164e-13 1.87665599796749e-12 100
"chr20" 46688001 46689000 "*" 4.07970324189932e-11 9.57770690255255e-11 68.9655172413793
"chr20" 46705001 46706000 "*" 1.55431223447522e-15 6.33705141101682e-15 100
"chr20" 46725001 46726000 "*" 1.07882591748876e-11 2.74553537455803e-11 100
"chr20" 46726001 46727000 "*" 9.93161875307846e-05 9.31526365269394e-05 60.6060606060606
"chr20" 46753001 46754000 "*" 0 0 -72.0822929106503
"chr20" 46767001 46768000 "*" 4.44089209850063e-16 1.91071758245033e-15 -75
"chr20" 46784001 46785000 "*" 6.08402217494586e-14 2.05454787937634e-13 90.4761904761905
"chr20" 46816001 46817000 "*" 1.20591092667155e-10 2.66075324129686e-10 100
"chr20" 46818001 46819000 "*" 4.01313771103418e-09 7.11882087237587e-09 100
"chr20" 46823001 46824000 "*" 0 0 -69.5135917063468
"chr20" 46825001 46826000 "*" 1.11022302462516e-16 5.03662826618488e-16 -51.3274336283186
"chr20" 46830001 46831000 "*" 2.60361667936415e-06 3.11419458317291e-06 80
"chr20" 46831001 46832000 "*" 1.53210777398272e-14 5.5628583449216e-14 -100
"chr20" 46836001 46837000 "*" 8.7349629751543e-09 1.47505871898511e-08 100
"chr20" 46851001 46852000 "*" 0 0 100
"chr20" 46877001 46878000 "*" 1.07864652044709e-05 1.17574627643031e-05

63.8888888888889
"chr20" 46897001 46898000 "*" 6.41059415551481e-08 9.58562859580824e-08
-72.7272727272727
"chr20" 46917001 46918000 "*" 0 0 87.378640776699
"chr20" 46938001 46939000 "*" 2.43005615629954e-09 4.45014222280278e-09 -100
"chr20" 46954001 46955000 "*" 1.06581410364015e-14 3.94854473541761e-14
-55.4626532887402
"chr20" 47033001 47034000 "*" 7.54951656745106e-14 2.5237892556906e-13 -100
"chr20" 47048001 47049000 "*" 2.3990809339125e-12 6.65441738642456e-12 100
"chr20" 47080001 47081000 "*" 0 0 100
"chr20" 47084001 47085000 "*" 9.88098491916389e-15 3.67401261259622e-14
-70.5882352941177
"chr20" 47117001 47118000 "*" 0 0 -74.0716180371353
"chr20" 47118001 47119000 "*" 1.53210777398272e-14 5.5628583449216e-14 100
"chr20" 47131001 47132000 "*" 0 0 -61.3636363636364
"chr20" 47168001 47169000 "*" 8.58968451922237e-12 2.213554983903e-11 -100
"chr20" 47182001 47183000 "*" 1.57118762444952e-12 4.4540497263512e-12
81.4814814814815
"chr20" 47222001 47223000 "*" 3.85228382526748e-09 6.88444235776972e-09
-61.0169491525424
"chr20" 47245001 47246000 "*" 2.37587727269783e-14 8.44268514944447e-14
-71.0526315789474
"chr20" 47256001 47257000 "*" 2.08995487582797e-10 4.46944006972663e-10 100
"chr20" 47286001 47287000 "*" 1.14130926931466e-13 3.7276674571693e-13 80
"chr20" 47289001 47290000 "*" 5.52458079283724e-12 1.46257146490184e-11
-56.5217391304348
"chr20" 47299001 47300000 "*" 4.44089209850063e-16 1.91071758245033e-15 -100
"chr20" 47323001 47324000 "*" 3.6700841921089e-08 5.65530226072258e-08 -100
"chr20" 47327001 47328000 "*" 9.47242284610184e-13 2.76110257009846e-12
-52.7629233511586
"chr20" 47336001 47337000 "*" 1.93720595120794e-11 4.76309924836636e-11 -100
"chr20" 47374001 47375000 "*" 1.16632431490515e-08 1.9331887981357e-08
76.9230769230769
"chr20" 47418001 47419000 "*" 6.50524861756097e-08 9.7186255022972e-08
80.3921568627451
"chr20" 47435001 47436000 "*" 1.76336723001214e-12 4.97399316794182e-12
68.3035714285714
"chr20" 47436001 47437000 "*" 1.11022302462516e-16 5.03662826618488e-16
87.1794871794872
"chr20" 47440001 47441000 "*" 2.07546515529344e-08 3.31473076384131e-08
-76.5957446808511
"chr20" 47444001 47445000 "*" 0 0 69.2014965944831
"chr20" 47463001 47464000 "*" 9.08108033215171e-11 2.03407753381119e-10 100
"chr20" 47466001 47467000 "*" 9.41836343471003e-07 1.19980154694393e-06
69.811320754717
"chr20" 47474001 47475000 "*" 2.35410283488946e-08 3.73499633162348e-08 70
"chr20" 47477001 47478000 "*" 1.39779633423487e-07 1.98463037958589e-07 -100
"chr20" 47515001 47516000 "*" 2.08814465718632e-09 3.86232567960463e-09 -100
"chr20" 47549001 47550000 "*" 0 0 100
"chr20" 47624001 47625000 "*" 9.0072393987839e-13 2.63157725646847e-12 -100
"chr20" 47937001 47938000 "*" 3.63445940010365e-11 8.58565209111204e-11 100
"chr20" 47954001 47955000 "*" 1.11769089872205e-07 1.61092542901763e-07 -71.25

"chr20" 47968001 47969000 "*" 2.12496686913255e-13 6.7178544377836e-13
71.4285714285714
"chr20" 47993001 47994000 "*" 2.03553206532536e-05 2.12753843906822e-05
56.5217391304348
"chr20" 48056001 48057000 "*" 0 0 100
"chr20" 48069001 48070000 "*" 6.4152538836737e-10 1.27477697728417e-09 -100
"chr20" 48079001 48080000 "*" 0 0 -100
"chr20" 48104001 48105000 "*" 1.12634901405784e-10 2.49563754426169e-10 -100
"chr20" 48131001 48132000 "*" 1.12458486967171e-09 2.15740815043925e-09 100
"chr20" 48134001 48135000 "*" 2.82201584322905e-09 5.12048751598707e-09 81.25
"chr20" 48166001 48167000 "*" 1.48214773787458e-13 4.77488235285846e-13 -100
"chr20" 48199001 48200000 "*" 6.59550958292954e-09 1.13456194382773e-08 100
"chr20" 48215001 48216000 "*" 1.41109876605761e-06 1.75583535616452e-06 -90
"chr20" 48219001 48220000 "*" 5.70957392476146e-10 1.14621387656155e-09
-69.6969696969697
"chr20" 48225001 48226000 "*" 1.5277158427196e-09 2.87642564232045e-09 100
"chr20" 48232001 48233000 "*" 4.44089209850063e-15 1.71914916534614e-14
-51.7786124613345
"chr20" 48234001 48235000 "*" 1.05162970331918e-06 1.33118796422389e-06
61.5384615384615
"chr20" 48319001 48320000 "*" 0 0 -100
"chr20" 48359001 48360000 "*" 4.01313771103418e-09 7.11882087237587e-09 -100
"chr20" 48484001 48485000 "*" 3.07531777821168e-14 1.07626052665081e-13
62.8571428571429
"chr20" 48505001 48506000 "*" 0 0 100
"chr20" 48615001 48616000 "*" 7.43194394914326e-12 1.93147394970013e-11
-51.219512195122
"chr20" 48622001 48623000 "*" 9.4210245304982e-07 1.20008555109384e-06
72.4137931034483
"chr20" 48630001 48631000 "*" 1.00403096947588e-09 1.94055485697779e-09
54.5454545454545
"chr20" 48639001 48640000 "*" 4.27128901137053e-08 6.53414432861274e-08
72.9166666666667
"chr20" 48655001 48656000 "*" 1.7356014847536e-08 2.80948669481175e-08
60.9756097560976
"chr20" 48663001 48664000 "*" 0 0 -100
"chr20" 48667001 48668000 "*" 2.51889176183795e-10 5.31989487017935e-10
59.0361445783133
"chr20" 48688001 48689000 "*" 8.00647770304863e-10 1.57305430211923e-09
55.5555555555556
"chr20" 48789001 48790000 "*" 0 0 100
"chr20" 48795001 48796000 "*" 1.11022302462516e-16 5.03662826618488e-16 100
"chr20" 48804001 48805000 "*" 3.99791311167519e-13 1.22027792394232e-12 -100
"chr20" 48809001 48810000 "*" 5.60821500350528e-10 1.12700361439991e-09
-67.110125646711
"chr20" 48830001 48831000 "*" 0.000387465840556378 0.000329754187927705
-50.9433962264151
"chr20" 48850001 48851000 "*" 0 0 -60.6382978723404
"chr20" 48860001 48861000 "*" 5.53979750961275e-10 1.11379168305788e-09 -68.75
"chr20" 48865001 48866000 "*" 2.21226192920199e-09 4.08138922573277e-09
-74.7093023255814
"chr20" 48946001 48947000 "*" 0 0 100

"chr20" 48948001 48949000 "*" 3.65130148338721e-12 9.90602415539447e-12 -100
"chr20" 48969001 48970000 "*" 2.02393657389166e-13 6.410608062171e-13
-51.8518518518518
"chr20" 48970001 48971000 "*" 0 0 -100
"chr20" 48985001 48986000 "*" 3.7274627828765e-12 1.00985250136599e-11 100
"chr20" 49017001 49018000 "*" 1.35447209004269e-14 4.94674708073053e-14 -100
"chr20" 49031001 49032000 "*" 1.9373391779709e-13 6.15971322898373e-13 -100
"chr20" 49064001 49065000 "*" 4.78284079008517e-13 1.44820017204545e-12
-54.5454545454545
"chr20" 49109001 49110000 "*" 0 0 -100
"chr20" 49151001 49152000 "*" 3.46600526057728e-12 9.42600366624647e-12
-86.3636363636364
"chr20" 49239001 49240000 "*" 3.61932706027801e-14 1.25777907157994e-13 -100
"chr20" 49250001 49251000 "*" 1.11022302462516e-15 4.59817122935606e-15 -100
"chr20" 49252001 49253000 "*" 0 0 -99.4413407821229
"chr20" 49268001 49269000 "*" 5.63660229602192e-13 1.68905058935478e-12 100
"chr20" 49319001 49320000 "*" 7.0006535457523e-08 1.03695832512054e-07 -100
"chr20" 49324001 49325000 "*" 4.03051019459699e-06 4.68321829238973e-06
58.3333333333333
"chr20" 49346001 49347000 "*" 4.32209823486573e-12 1.15935290479641e-11 -100
"chr20" 49360001 49361000 "*" 1.41166905942569e-08 2.31705470059551e-08 -90
"chr20" 49364001 49365000 "*" 3.6700841921089e-08 5.65530226072258e-08 100
"chr20" 49379001 49380000 "*" 0 0 -56.8421052631579
"chr20" 49419001 49420000 "*" 3.88578058618805e-15 1.5152981210988e-14 -100
"chr20" 49548001 49549000 "*" 0 0 60.4054054054054
"chr20" 49594001 49595000 "*" 1.98365768255826e-11 4.85612402473442e-11 100
"chr20" 49600001 49601000 "*" 7.0006535457523e-08 1.03695832512054e-07 100
"chr20" 49624001 49625000 "*" 7.88258347483861e-15 2.96145301916012e-14 100
"chr20" 49625001 49626000 "*" 3.6700841921089e-08 5.65530226072258e-08 100
"chr20" 49638001 49639000 "*" 3.45022422520991e-07 4.66205089083695e-07
-77.2727272727273
"chr20" 49699001 49700000 "*" 7.85417227922025e-08 1.15517757200783e-07
-66.6666666666667
"chr20" 49794001 49795000 "*" 6.11886952806007e-06 6.92255677472338e-06
-57.5757575757576
"chr20" 49847001 49848000 "*" 2.90961819082725e-08 4.56745252179978e-08
-74.1379310344828
"chr20" 49874001 49875000 "*" 0 0 -96
"chr20" 49891001 49892000 "*" 0 0 100
"chr20" 49905001 49906000 "*" 2.70541700153615e-10 5.68690552277906e-10
54.5454545454545
"chr20" 49931001 49932000 "*" 2.04988070962031e-09 3.80458088311340e-09
-70.2702702702703
"chr20" 49942001 49943000 "*" 4.08209022140227e-11 9.57770690255255e-11 -100
"chr20" 49978001 49979000 "*" 1.98365768255826e-11 4.85612402473442e-11 100
"chr20" 49983001 49984000 "*" 0 0 95
"chr20" 49985001 49986000 "*" 0 0 -82.1428571428571
"chr20" 49986001 49987000 "*" 0 0 -55.1020408163265
"chr20" 49989001 49990000 "*" 1.92426406031565e-07 2.68738008279018e-07
-65.546218487395
"chr20" 49993001 49994000 "*" 3.66181607480698e-07 4.92728439089831e-07 -75
"chr20" 50009001 50010000 "*" 4.07260223278527e-06 4.72810858730247e-06

51.1111111111111
"chr20" 50041001 50042000 "*" 0 0 -100
"chr20" 50111001 50112000 "*" 1.77476355833761e-09 3.32009595176923e-09 -75
"chr20" 50148001 50149000 "*" 3.99546075805501e-05 3.98784360862687e-05
51.8518518518519
"chr20" 50159001 50160000 "*" 0 0 62.687091731
"chr20" 50187001 50188000 "*" 0 0 83.1168831168831
"chr20" 50245001 50246000 "*" 1.11022302462516e-16 5.03662826618488e-16 100
"chr20" 50259001 50260000 "*" 2.00227675550835e-08 3.20459176617961e-08 100
"chr20" 50359001 50360000 "*" 1.14124265593318e-11 2.89138062669327e-11 100
"chr20" 50380001 50381000 "*" 1.67299207820548e-08 2.71377429441055e-08 -100
"chr20" 50415001 50416000 "*" 1.13140401492018e-08 1.87801380787728e-08 100
"chr20" 50459001 50460000 "*" 0 0 100
"chr20" 50630001 50631000 "*" 4.01313771103418e-09 7.11882087237587e-09 -100
"chr20" 50717001 50718000 "*" 2.69084754478399e-12 7.41487347763769e-12 -100
"chr20" 50798001 50799000 "*" 4.9585469064084e-09 8.68302175679199e-09
88.4615384615385
"chr20" 50954001 50955000 "*" 3.84137166520304e-14 1.32929369817675e-13 -100
"chr20" 51300001 51301000 "*" 3.33644088579277e-07 4.51620565574885e-07
68.4210526315789
"chr20" 51383001 51384000 "*" 5.88957105307486e-09 1.02259718257691e-08
-92.3076923076923
"chr20" 51405001 51406000 "*" 3.33066907387547e-16 1.4495649018245e-15 76
"chr20" 51590001 51591000 "*" 0 0 86.046511627907
"chr20" 51633001 51634000 "*" 2.08814465718632e-09 3.86232567960463e-09 100
"chr20" 51669001 51670000 "*" 1.35036426485158e-12 3.86629547363774e-12 100
"chr20" 51759001 51760000 "*" 9.47020240005259e-14 3.12491189162604e-13 100
"chr20" 51774001 51775000 "*" 2.33146835171283e-15 9.32818831833246e-15 100
"chr20" 51899001 51900000 "*" 8.7349629751543e-09 1.47505871898511e-08 100
"chr20" 51900001 51901000 "*" 3.40125705378114e-10 7.03784073980162e-10 100
"chr20" 52112001 52113000 "*" 2.4535928844216e-14 8.69144396197119e-14 -100
"chr20" 52118001 52119000 "*" 2.68450373042128e-10 5.64465213127486e-10 -100
"chr20" 52228001 52229000 "*" 4.14335232790108e-13 1.26153063645578e-12 100
"chr20" 52244001 52245000 "*" 7.0006535457523e-08 1.03695832512054e-07 -100
"chr20" 52294001 52295000 "*" 5.68671679301858e-07 7.46915090250131e-07
-51.8072289156626
"chr20" 52353001 52354000 "*" 3.29235203322398e-07 4.46007461671159e-07 52
"chr20" 52360001 52361000 "*" 0.000234428549617305 0.00020696211908328
55.2631578947368
"chr20" 52375001 52376000 "*" 2.74464854510548e-07 3.75815727722768e-07
-52.3809523809524
"chr20" 52560001 52561000 "*" 7.63240809442678e-07 9.84819844409228e-07
53.8461538461538
"chr20" 52705001 52706000 "*" 1.17905685215192e-13 3.83653245040640e-13 -100
"chr20" 52721001 52722000 "*" 6.59550958292954e-09 1.13456194382773e-08 -100
"chr20" 52760001 52761000 "*" 2.87422404430249e-05 2.93567189445702e-05 -56
"chr20" 52764001 52765000 "*" 2.62927705163563e-07 3.60853669669655e-07
54.1666666666667
"chr20" 52768001 52769000 "*" 1.16573417585641e-14 4.29335582900508e-14 -100
"chr20" 52773001 52774000 "*" 0 0 100
"chr20" 53068001 53069000 "*" 1.11022302462516e-16 5.03662826618488e-16 -100
"chr20" 53173001 53174000 "*" 3.6700841921089e-08 5.65530226072258e-08 100

"chr20" 53313001 53314000 "*" 2.78418621491028e-10 5.84496868167592e-10 65.625
"chr20" 53411001 53412000 "*" 1.00307315265002e-06 1.27246592909776e-06 50.6572295247725
"chr20" 53730001 53731000 "*" 4.73234496034536e-09 8.30627300264826e-09 -100
"chr20" 54290001 54291000 "*" 2.00227675550835e-08 3.20459176617961e-08 100
"chr20" 54350001 54351000 "*" 2.88779000712225e-11 6.92935809466068e-11 -100
"chr20" 54522001 54523000 "*" 0 0 -95.8333333333333
"chr20" 54525001 54526000 "*" 0 0 100
"chr20" 54741001 54742000 "*" 2.15125472990962e-10 4.58126659943874e-10 -100
"chr20" 54742001 54743000 "*" 6.08662167959073e-05 5.90595322645893e-05 -64.2857142857143
"chr20" 54747001 54748000 "*" 0 0 100
"chr20" 54770001 54771000 "*" 1.67299207820548e-08 2.71377429441055e-08 -100
"chr20" 54893001 54894000 "*" 7.0006535457523e-08 1.03695832512054e-07 100
"chr20" 54992001 54993000 "*" 1.55405438517775e-08 2.53822178196812e-08 53.7037037037037
"chr20" 55019001 55020000 "*" 0 0 100
"chr20" 55021001 55022000 "*" 6.20694606823236e-11 1.42438331492694e-10 -65.9659090909091
"chr20" 55022001 55023000 "*" 1.2691762085737e-09 2.42059457962459e-09 -83.3333333333333
"chr20" 55115001 55116000 "*" 2.68450373042128e-10 5.64465213127486e-10 -100
"chr20" 55188001 55189000 "*" 1.5277158427196e-09 2.87642564232045e-09 100
"chr20" 55199001 55200000 "*" 0 0 -100
"chr20" 55203001 55204000 "*" 0 0 75.8664955070603
"chr20" 55204001 55205000 "*" 0 0 65.2249134948097
"chr20" 55218001 55219000 "*" 0.000496254900866688 0.000414667937798419 54.1666666666667
"chr20" 55221001 55222000 "*" 7.77546844432653e-06 8.66140627117292e-06 67.6470588235294
"chr20" 55232001 55233000 "*" 2.81561951531728e-09 5.10932615271847e-09 -71.6755319148936
"chr20" 55233001 55234000 "*" 1.74825329768424e-08 2.82886856362649e-08 64.8648648648649
"chr20" 55248001 55249000 "*" 1.17815757150197e-11 2.98053091283373e-11 -74.8275862068966
"chr20" 55264001 55265000 "*" 8.01462617383564e-08 1.17734428441707e-07 -71.4285714285714
"chr20" 55274001 55275000 "*" 2.07652925743318e-07 2.88856621379191e-07 73.4375
"chr20" 55281001 55282000 "*" 1.46184566574004e-06 1.81497227178446e-06 58.3333333333333
"chr20" 55342001 55343000 "*" 5.21804821573824e-15 2.00446252136729e-14 93.4782608695652
"chr20" 55353001 55354000 "*" 7.67056418382595e-11 1.7359448580318e-10 -100
"chr20" 55389001 55390000 "*" 3.56183971206292e-11 8.43393090910203e-11 56.5217391304348
"chr20" 55391001 55392000 "*" 1.11022302462516e-16 5.03662826618488e-16 100
"chr20" 55417001 55418000 "*" 2.62303026490329e-05 2.69534827773176e-05 51.1764705882353
"chr20" 55418001 55419000 "*" 2.04047712060174e-09 3.78895698465745e-09 -62.962962962963
"chr20" 55523001 55524000 "*" 0 0 100

"chr20" 55547001 55548000 "*" 1.11022302462516e-16 5.03662826618488e-16 100
"chr20" 55549001 55550000 "*" 1.37643063613524e-09 2.61498651228495e-09
-51.063829787234
"chr20" 55565001 55566000 "*" 6.60582699651968e-14 2.22176301799e-13 100
"chr20" 55570001 55571000 "*" 2.52467581285387e-07 3.4735516511414e-07
-55.8139534883721
"chr20" 55575001 55576000 "*" 6.59550958292954e-09 1.13456194382773e-08 -100
"chr20" 55588001 55589000 "*" 7.96029908656237e-14 2.6539855762804e-13 80
"chr20" 55589001 55590000 "*" 0 0 53.2312171656434
"chr20" 55597001 55598000 "*" 1.35007560686518e-11 3.38925638226179e-11
-60.2974828375286
"chr20" 55599001 55600000 "*" 5.6362137179633e-11 1.29963270300065e-10 100
"chr20" 55608001 55609000 "*" 3.94216216874632e-05 3.93770971245002e-05
71.4285714285714
"chr20" 55623001 55624000 "*" 3.33066907387547e-16 1.4495649018245e-15
-61.6908212560386
"chr20" 55641001 55642000 "*" 2.43005615629954e-09 4.45014222280278e-09 100
"chr20" 55644001 55645000 "*" 4.1153938973304e-06 4.77326970666537e-06
66.6666666666667
"chr20" 55651001 55652000 "*" 4.25215418431435e-13 1.29196918865689e-12
-65.3846153846154
"chr20" 55665001 55666000 "*" 0 0 -70.5913503971756
"chr20" 55691001 55692000 "*" 1.87994064759778e-12 5.28440715440909e-12 -68.75
"chr20" 55701001 55702000 "*" 1.32862584267812e-08 2.18824442693839e-08
53.5140562248996
"chr20" 55706001 55707000 "*" 0 0 -100
"chr20" 55723001 55724000 "*" 7.22671589237223e-10 1.42708041441925e-09
54.5454545454545
"chr20" 55730001 55731000 "*" 1.80989033669032e-05 1.90667975357288e-05 -68.75
"chr20" 55743001 55744000 "*" 0 0 -52.0390514769469
"chr20" 55792001 55793000 "*" 0.000471255033835716 0.000395163867608855
-51.2820512820513
"chr20" 55795001 55796000 "*" 1.75928160928152e-11 4.35257527084094e-11
-56.5217391304348
"chr20" 55805001 55806000 "*" 1.19199317083485e-10 2.63443558487241e-10
-58.4516565246788
"chr20" 55846001 55847000 "*" 6.4152538836737e-10 1.27477697728417e-09 -100
"chr20" 55850001 55851000 "*" 1.39779633423487e-07 1.98463037958589e-07 -100
"chr20" 55858001 55859000 "*" 1.84915416312492e-11 4.56517512311393e-11
-71.5909090909091
"chr20" 55878001 55879000 "*" 4.44089209850063e-16 1.91071758245033e-15 -100
"chr20" 55925001 55926000 "*" 0 0 85
"chr20" 55992001 55993000 "*" 8.57092175010621e-13 2.51149198099779e-12 -100
"chr20" 55999001 56000000 "*" 4.59859922252726e-07 6.10788604030894e-07
-63.6363636363636
"chr20" 56012001 56013000 "*" 2.41690761848545e-08 3.82830615477435e-08
-82.4561403508772
"chr20" 56046001 56047000 "*" 2.55545802962942e-07 3.51329444221192e-07
72.2222222222222
"chr20" 56074001 56075000 "*" 1.29037891483108e-11 3.24582280286866e-11 100
"chr20" 56137001 56138000 "*" 9.12825370846804e-13 2.66562577521868e-12
-57.8525641025641

"chr20" 56153001 56154000 "*" 0.00037987339733192 0.000323695610297176 52.6315789473684
"chr20" 56162001 56163000 "*" 0 0 -77.6315789473684
"chr20" 56188001 56189000 "*" 1.02144959157613e-11 2.61109568616629e-11 -55.4843304843305
"chr20" 56191001 56192000 "*" 3.08531722392758e-09 5.57543783255698e-09 -58.3333333333333
"chr20" 56195001 56196000 "*" 5.06261699229071e-14 1.7297955084095e-13 68
"chr20" 56222001 56223000 "*" 0 0 62.7906976744186
"chr20" 56302001 56303000 "*" 5.55111512312578e-16 2.36485094870365e-15 54.0895061728395
"chr20" 56304001 56305000 "*" 1.62291792327185e-06 2.00133797384099e-06 61.1111111111111
"chr20" 56310001 56311000 "*" 6.4152538836737e-10 1.27477697728417e-09 100
"chr20" 56323001 56324000 "*" 1.11022302462516e-16 5.03662826618488e-16 -72.2222222222222
"chr20" 56368001 56369000 "*" 0 0 73.0769230769231
"chr20" 56378001 56379000 "*" 1.23623824466179e-05 1.33534251888187e-05 -66.6666666666667
"chr20" 56412001 56413000 "*" 0 0 72.093023255814
"chr20" 56442001 56443000 "*" 0 0 100
"chr20" 56443001 56444000 "*" 0 0 -63.7037037037037
"chr20" 56446001 56447000 "*" 0 0 97.3684210526316
"chr20" 56450001 56451000 "*" 1.98365768255826e-11 4.85612402473442e-11 -100
"chr20" 56454001 56455000 "*" 7.47896278241456e-09 1.27859689378443e-08 59.1397849462366
"chr20" 56469001 56470000 "*" 0.000262931009909484 0.000230192831154051 -59.3301435406699
"chr20" 56487001 56488000 "*" 2.55520049563529e-11 6.18952954496944e-11 59.7315436241611
"chr20" 56496001 56497000 "*" 6.60582699651968e-14 2.22176301799e-13 100
"chr20" 56505001 56506000 "*" 7.34456939710526e-12 1.90959942220558e-11 -97.2222222222222
"chr20" 56535001 56536000 "*" 0 0 100
"chr20" 56544001 56545000 "*" 1.66415359270644e-08 2.70939697607352e-08 -66.6666666666667
"chr20" 56545001 56546000 "*" 2.00227675550835e-08 3.20459176617961e-08 100
"chr20" 56548001 56549000 "*" 4.64978269221206e-08 7.08450680950107e-08 -68.0851063829787
"chr20" 56558001 56559000 "*" 2.93098878501041e-14 1.02950853891608e-13 -90
"chr20" 56583001 56584000 "*" 1.70428209322893e-07 2.39503790264489e-07 57.0247933884297
"chr20" 56592001 56593000 "*" 0 0 -100
"chr20" 56600001 56601000 "*" 2.43005615629954e-09 4.45014222280278e-09 100
"chr20" 56628001 56629000 "*" 2.30658880884249e-09 4.24423320622348e-09 -50.7936507936508
"chr20" 56630001 56631000 "*" 0 0 100
"chr20" 56640001 56641000 "*" 4.01313771103418e-09 7.11882087237587e-09 -100
"chr20" 56644001 56645000 "*" 0 0 100
"chr20" 56675001 56676000 "*" 2.64325332233195e-06 3.15856923971574e-06 53.8461538461538
"chr20" 56676001 56677000 "*" 0 0 -85.7142857142857

"chr20" 56711001 56712000 "*" 0.00020976332305167 0.000186660769941092
-52.3809523809524
"chr20" 56724001 56725000 "*" 6.92897336951326e-07 8.99090158231749e-07
-77.5510204081633
"chr20" 56750001 56751000 "*" 3.23634452570332e-11 7.70784708086468e-11
65.1162790697674
"chr20" 56766001 56767000 "*" 9.82749104316838e-10 1.90158137084983e-09
57.4468085106383
"chr20" 56774001 56775000 "*" 0 0 -100
"chr20" 56775001 56776000 "*" 2.00227675550835e-08 3.20459176617961e-08 100
"chr20" 56804001 56805000 "*" 0 0 69.5535714285714
"chr20" 56822001 56823000 "*" 3.05311331771918e-12 8.34639368772922e-12 100
"chr20" 56847001 56848000 "*" 1.83952852950142e-12 5.17499701329218e-12 -100
"chr20" 56920001 56921000 "*" 0 0 72.7272727272727
"chr20" 56950001 56951000 "*" 1.22980784212912e-05 1.32891296008561e-05
-69.2307692307692
"chr20" 57044001 57045000 "*" 2.61113353161591e-12 7.21261075142292e-12
-90.983606557377
"chr20" 57068001 57069000 "*" 3.30332519458132e-05 3.34028011767552e-05
62.0751341681574
"chr20" 57074001 57075000 "*" 0 0 100
"chr20" 57100001 57101000 "*" 9.65729496371637e-10 1.87255912493232e-09 -100
"chr20" 57110001 57111000 "*" 1.86667281631969e-09 3.48394183905826e-09
56.5217391304348
"chr20" 57120001 57121000 "*" 1.10289555266263e-12 3.19139052726536e-12
76.9230769230769
"chr20" 57129001 57130000 "*" 0 0 -100
"chr20" 57146001 57147000 "*" 3.6700841921089e-08 5.65530226072258e-08 -100
"chr20" 57152001 57153000 "*" 1.4432899320127e-15 5.90750815956055e-15
-66.6666666666667
"chr20" 57169001 57170000 "*" 7.53263278910898e-05 7.19956482159304e-05
-61.7647058823529
"chr20" 57275001 57276000 "*" 0 0 -100
"chr20" 57284001 57285000 "*" 1.39779633423487e-07 1.98463037958589e-07 -100
"chr20" 57297001 57298000 "*" 0 0 100
"chr20" 57298001 57299000 "*" 2.08980171423345e-06 2.53621046689567e-06
57.0135746606335
"chr20" 57322001 57323000 "*" 3.88022947106492e-13 1.1867462770515e-12 100
"chr20" 57324001 57325000 "*" 0.000455208012568153 0.000382689032955743
-51.219512195122
"chr20" 57327001 57328000 "*" 4.49673631663927e-12 1.20371980644711e-11
68.2336182336182
"chr20" 57341001 57342000 "*" 3.530509218308e-14 1.22891039008692e-13 -100
"chr20" 57361001 57362000 "*" 0 0 100
"chr20" 57376001 57377000 "*" 3.55759866010885e-12 9.6653269851957e-12
58.9351851851852
"chr20" 57379001 57380000 "*" 1.55431223447522e-15 6.33705141101682e-15
-61.4864864864865
"chr20" 57382001 57383000 "*" 8.57092175010621e-13 2.51149198099779e-12 -100
"chr20" 57387001 57388000 "*" 4.67040218166126e-07 6.19565759085307e-07 -80
"chr20" 57391001 57392000 "*" 3.71866981652147e-11 8.77712337620108e-11
74.3589743589744

"chr20" 57418001 57419000 "*" 3.63445940010365e-11 8.58565209111204e-11 -100
"chr20" 57467001 57468000 "*" 0 0 50.9090909090909
"chr20" 57587001 57588000 "*" 7.68311810395161e-07 9.90964461140931e-07 -60
"chr20" 57616001 57617000 "*" 4.14335232790108e-13 1.26153063645578e-12 -100
"chr20" 57622001 57623000 "*" 6.92287338566189e-11 1.57605630124247e-10 -100
"chr20" 57699001 57700000 "*" 0 0 -89.247311827957
"chr20" 57701001 57702000 "*" 1.66351331376546e-07 2.34054541106703e-07
-54.5454545454545
"chr20" 57702001 57703000 "*" 0 0 -65.0537634408602
"chr20" 57733001 57734000 "*" 1.31610942394333e-05 1.4161144785177e-05
-66.6666666666667
"chr20" 57764001 57765000 "*" 5.55111512312578e-16 2.36485094870365e-15 -100
"chr20" 57820001 57821000 "*" 2.00227675550835e-08 3.20459176617961e-08 -100
"chr20" 57847001 57848000 "*" 0 0 -100
"chr20" 57888001 57889000 "*" 6.62958576924666e-12 1.73620572057889e-11 100
"chr20" 57906001 57907000 "*" 1.54630752646767e-11 3.85079502648228e-11 -100
"chr20" 57928001 57929000 "*" 0 0 61.9047619047619
"chr20" 57946001 57947000 "*" 3.02737834800837e-11 7.24512795818724e-11
-59.5238095238095
"chr20" 57963001 57964000 "*" 5.08482145278322e-14 1.73432706170696e-13 100
"chr20" 57965001 57966000 "*" 2.55997001374908e-10 5.40241101640176e-10
84.6153846153846
"chr20" 57981001 57982000 "*" 3.31690230837012e-12 9.03347040781321e-12 100
"chr20" 58000001 58001000 "*" 6.4152538836737e-10 1.27477697728417e-09 -100
"chr20" 58003001 58004000 "*" 8.11296663183469e-09 1.37928190725433e-08 56
"chr20" 58035001 58036000 "*" 0 0 100
"chr20" 58046001 58047000 "*" 1.11022302462516e-16 5.03662826618488e-16 100
"chr20" 58058001 58059000 "*" 1.00494357280212e-09 1.94222868368164e-09
56.283422459893
"chr20" 58069001 58070000 "*" 9.08108033215171e-11 2.03407753381119e-10 -100
"chr20" 58071001 58072000 "*" 2.56742405113641e-10 5.41773031605747e-10
-74.7474747474747
"chr20" 58138001 58139000 "*" 7.0006535457523e-08 1.03695832512054e-07 100
"chr20" 58144001 58145000 "*" 9.08108033215171e-11 2.03407753381119e-10 -100
"chr20" 58148001 58149000 "*" 0 0 100
"chr20" 58150001 58151000 "*" 5.48638023900594e-10 1.10374357336421e-09 -100
"chr20" 58160001 58161000 "*" 1.96002567509623e-07 2.73471365106896e-07 -78
"chr20" 58200001 58201000 "*" 1.60841673313428e-10 3.48541264741657e-10
51.4486638537271
"chr20" 58203001 58204000 "*" 2.43005615629954e-09 4.45014222280278e-09 100
"chr20" 58247001 58248000 "*" 2.67283972732457e-11 6.45686178157698e-11 -100
"chr20" 58248001 58249000 "*" 5.6621374255883e-15 2.16468580166064e-14
-54.6789860222696
"chr20" 58260001 58261000 "*" 1.77635683940025e-15 7.19870740878856e-15 -100
"chr20" 58268001 58269000 "*" 4.34645670899236e-07 5.79023271110259e-07
66.6666666666667
"chr20" 58301001 58302000 "*" 3.25678226653636e-06 3.838579860199e-06 -65
"chr20" 58329001 58330000 "*" 0 0 68.5714285714286
"chr20" 58334001 58335000 "*" 7.7715611723761e-16 3.26213507634405e-15 100
"chr20" 58345001 58346000 "*" 1.26565424807268e-14 4.63923203146481e-14 100
"chr20" 58352001 58353000 "*" 2.88710388929303e-10 6.03399264731139e-10 100
"chr20" 58360001 58361000 "*" 0 0 100

"chr20" 58361001 58362000 "*" 8.3882012447134e-11 1.88922989503423e-10 -100
"chr20" 58380001 58381000 "*" 2.02327044007689e-12 5.65300384792621e-12 100
"chr20" 58407001 58408000 "*" 3.73034936274053e-14 1.29420415488591e-13 -100
"chr20" 58409001 58410000 "*" 1.14124265593318e-11 2.89138062669327e-11 -100
"chr20" 58513001 58514000 "*" 1.67299207820548e-08 2.71377429441055e-08 -100
"chr20" 58570001 58571000 "*" 1.11022302462516e-16 5.03662826618488e-16 -100
"chr20" 58587001 58588000 "*" 6.04417627059206e-11 1.38849255306602e-10
67.741935483871
"chr20" 58606001 58607000 "*" 9.2148511043888e-15 3.43705279328267e-14 -100
"chr20" 58628001 58629000 "*" 1.70344255812083e-07 2.39410014315768e-07
-58.3333333333333
"chr20" 58639001 58640000 "*" 0 0 100
"chr20" 58656001 58657000 "*" 3.40125705378114e-10 7.03784073980162e-10 -100
"chr20" 58664001 58665000 "*" 1.50723877823111e-12 4.29011858312785e-12
70.2702702702703
"chr20" 58707001 58708000 "*" 2.16901478444775e-06 2.62630620055891e-06
-66.6666666666667
"chr20" 58711001 58712000 "*" 2.77655010094691e-10 5.82996509542432e-10 -90
"chr20" 58715001 58716000 "*" 3.03979064142368e-13 9.42350883309283e-13 -100
"chr20" 58814001 58815000 "*" 4.01313771103418e-09 7.11882087237587e-09 100
"chr20" 58815001 58816000 "*" 1.10655694607331e-08 1.84506058413625e-08
55.9523809523809
"chr20" 58934001 58935000 "*" 2.02327044007689e-12 5.65300384792621e-12 -100
"chr20" 58939001 58940000 "*" 3.95861121660346e-12 1.0684496262032e-11 -100
"chr20" 58972001 58973000 "*" 8.32556246166405e-13 2.44440509740029e-12
83.3333333333333
"chr20" 59052001 59053000 "*" 4.73234496034536e-09 8.30627300264826e-09 -100
"chr20" 59056001 59057000 "*" 0 0 -60.8333333333333
"chr20" 59060001 59061000 "*" 0 0 -93.0379746835443
"chr20" 59062001 59063000 "*" 1.97746120145226e-07 2.75751809844255e-07
-51.8518518518519
"chr20" 59226001 59227000 "*" 2.22044604925031e-16 9.81641919380259e-16 -100
"chr20" 59251001 59252000 "*" 6.44564592999775e-06 7.26663863268796e-06
66.6666666666667
"chr20" 59328001 59329000 "*" 7.29221839946836e-05 6.98571211774533e-05 60
"chr20" 59351001 59352000 "*" 1.05656372539897e-10 2.34919091152156e-10
-90.6666666666667
"chr20" 59359001 59360000 "*" 2.79987144580218e-12 7.68634726811898e-12 100
"chr20" 59382001 59383000 "*" 2.22044604925031e-16 9.81641919380259e-16 100
"chr20" 59387001 59388000 "*" 2.88779000712225e-11 6.92935809466068e-11 -100
"chr20" 59478001 59479000 "*" 4.44089209850063e-16 1.91071758245033e-15 -100
"chr20" 59550001 59551000 "*" 6.04438721296674e-11 1.38849255306602e-10 100
"chr20" 59564001 59565000 "*" 2.88710388929303e-10 6.03399264731139e-10 -100
"chr20" 59572001 59573000 "*" 8.37108160567368e-14 2.78337535503486e-13
-79.5918367346939
"chr20" 59619001 59620000 "*" 5.25833514877228e-05 5.15382873150132e-05
-54.7169811320755
"chr20" 59666001 59667000 "*" 5.04263297784746e-13 1.52078941532818e-12 100
"chr20" 59690001 59691000 "*" 0 0 -100
"chr20" 59692001 59693000 "*" 1.11022302462516e-16 5.03662826618488e-16 -100
"chr20" 59693001 59694000 "*" 2.4535928844216e-14 8.69144396197119e-14 -100
"chr20" 59699001 59700000 "*" 1.21380683282268e-12 3.49595385723632e-12

-79.1666666666667
"chr20" 59711001 59712000 "*" 4.63041827103439e-10 9.42500393091927e-10
65.8385093167702
"chr20" 59732001 59733000 "*" 3.76365605347928e-14 1.3043487970679e-13 -100
"chr20" 59735001 59736000 "*" 5.46136436074818e-09 9.51795202365188e-09
-66.6666666666667
"chr20" 59814001 59815000 "*" 5.01025332333427e-10 1.01358031135889e-09 -100
"chr20" 59843001 59844000 "*" 3.93129973019768e-13 1.20082455865539e-12
-59.8579423159707
"chr20" 59853001 59854000 "*" 3.10862446895044e-15 1.22466437093774e-14 100
"chr20" 59883001 59884000 "*" 0.000320710195636176 0.000276619581787338
52.3809523809524
"chr20" 59891001 59892000 "*" 6.08402217494586e-14 2.05454787937634e-13 100
"chr20" 59892001 59893000 "*" 1.56863411149288e-12 4.4469621057462e-12 -100
"chr20" 59897001 59898000 "*" 2.08814465718632e-09 3.86232567960463e-09 100
"chr20" 59932001 59933000 "*" 1.13140401492018e-08 1.87801380787728e-08 -100
"chr20" 59960001 59961000 "*" 2.75335310107039e-14 9.69727903855348e-14 -100
"chr20" 59964001 59965000 "*" 9.29589738518644e-13 2.71277623738875e-12
-82.2784810126582
"chr20" 59969001 59970000 "*" 6.66133814775094e-16 2.81595744474255e-15 -100
"chr20" 59974001 59975000 "*" 5.29376542601767e-12 1.40405064755592e-11
-75.8620689655172
"chr20" 59990001 59991000 "*" 0 0 -100
"chr20" 59991001 59992000 "*" 1.93720595120794e-11 4.76309924836636e-11 -100
"chr20" 59992001 59993000 "*" 1.13140401492018e-08 1.87801380787728e-08 -100
"chr20" 60015001 60016000 "*" 1.93806003467856e-08 3.1188723137734e-08
63.4146341463415
"chr20" 60052001 60053000 "*" 2.3743562671541e-11 5.77006811197935e-11
52.212389380531
"chr20" 60087001 60088000 "*" 3.19744231092045e-14 1.1176868990786e-13
50.9803921568627
"chr20" 60088001 60089000 "*" 1.45168321807887e-11 3.63021774960241e-11
-63.0952380952381
"chr20" 60094001 60095000 "*" 5.73208147613968e-13 1.71636660394621e-12
50.3937007874016
"chr20" 60095001 60096000 "*" 1.04239684661778e-08 1.74410431181914e-08
-54.3572984749455
"chr20" 60103001 60104000 "*" 0 0 -100
"chr20" 60107001 60108000 "*" 1.01718633516157e-12 2.95280366517669e-12
-61.978021978022
"chr20" 60129001 60130000 "*" 0 0 100
"chr20" 60146001 60147000 "*" 5.37090823704567e-07 7.0728461177587e-07
66.2921348314607
"chr20" 60160001 60161000 "*" 6.57719213537078e-05 6.34967922170184e-05
-55.1724137931034
"chr20" 60193001 60194000 "*" 1.32083807850014e-05 1.4209273834717e-05 60
"chr20" 60196001 60197000 "*" 1.23534515950041e-12 3.55491847700212e-12 60
"chr20" 60202001 60203000 "*" 5.55111512312578e-16 2.36485094870365e-15
-68.4615384615385
"chr20" 60203001 60204000 "*" 0 0 -59.7574958145198
"chr20" 60205001 60206000 "*" 5.44009282066327e-15 2.08486137681812e-14 -100
"chr20" 60212001 60213000 "*" 6.38756773585492e-07 8.33076784507971e-07

73.6842105263158
"chr20" 60216001 60217000 "*" 8.88178419700125e-16 3.70670032207938e-15 100
"chr20" 60223001 60224000 "*" 3.04978264864531e-13 9.45378176930334e-13
-77.2277227722772
"chr20" 60229001 60230000 "*" 9.31062556153162e-06 1.02542410936219e-05
53.6363636363636
"chr20" 60230001 60231000 "*" 1.11022302462516e-15 4.59817122935606e-15
-80.4878048780488
"chr20" 60233001 60234000 "*" 1.11022302462516e-16 5.03662826618488e-16 -100
"chr20" 60244001 60245000 "*" 4.40227827567252e-05 4.36712678996e-05
-62.7906976744186
"chr20" 60249001 60250000 "*" 0 0 -80.7017543859649
"chr20" 60254001 60255000 "*" 2.22044604925031e-16 9.81641919380259e-16
-66.6666666666667
"chr20" 60258001 60259000 "*" 0 0 58.8888888888889
"chr20" 60277001 60278000 "*" 1.1123213461417e-11 2.82726976479608e-11
-74.6268656716418
"chr20" 60304001 60305000 "*" 0 0 -74.5614035087719
"chr20" 60312001 60313000 "*" 1.77846626314704e-12 5.01454835807685e-12
80.9210526315789
"chr20" 60314001 60315000 "*" 1.11022302462516e-16 5.03662826618488e-16
-50.2702702702703
"chr20" 60354001 60355000 "*" 0 0 53.2544378698225
"chr20" 60366001 60367000 "*" 4.10007006124147e-09 7.26360432338776e-09
-50.997150997151
"chr20" 60377001 60378000 "*" 0 0 -54.2178910544728
"chr20" 60382001 60383000 "*" 1.12909681604378e-13 3.69211767905576e-13
58.849104859335
"chr20" 60387001 60388000 "*" 0 0 74.6666666666667
"chr20" 60397001 60398000 "*" 0 0 -100
"chr20" 60407001 60408000 "*" 2.00227675550835e-08 3.20459176617961e-08 100
"chr20" 60409001 60410000 "*" 0 0 -60.8326875053819
"chr20" 60424001 60425000 "*" 4.04946212464452e-05 4.0376350196346e-05
64.7058823529412
"chr20" 60433001 60434000 "*" 1.04637758679971e-07 1.51468575948217e-07
78.6885245901639
"chr20" 60435001 60436000 "*" 0 0 78.4810126582279
"chr20" 60466001 60467000 "*" 6.04227778921995e-12 1.59119096591558e-11
-91.304347826087
"chr20" 60472001 60473000 "*" 1.11022302462516e-16 5.03662826618488e-16 100
"chr20" 60487001 60488000 "*" 3.597027969926e-07 4.84612690151399e-07
92.8571428571429
"chr20" 60498001 60499000 "*" 0 0 73.4516103252617
"chr20" 60533001 60534000 "*" 9.20084652911157e-09 1.54977786734887e-08
-57.4468085106383
"chr20" 60667001 60668000 "*" 1.17905685215192e-13 3.83653245040640e-13 -100
"chr20" 60677001 60678000 "*" 2.60796939599572e-11 6.31093762662651e-11
-53.5555555555556
"chr20" 60691001 60692000 "*" 1.11022302462516e-16 5.03662826618488e-16
-93.8202247191011
"chr20" 60787001 60788000 "*" 0 0 78.448275862069
"chr20" 60792001 60793000 "*" 0 0 100

"chr20" 60807001 60808000 "*" 1.72850400659286e-10 3.73515841079823e-10 54.8107615139079
"chr20" 60811001 60812000 "*" 0 0 -64.5161290322581
"chr20" 60814001 60815000 "*" 0 0 -55.0128205128205
"chr20" 60945001 60946000 "*" 0 0 -71.6522318454364
"chr20" 60964001 60965000 "*" 0 0 -94.8717948717949
"chr20" 60985001 60986000 "*" 0 0 -74.2168674698795
"chr20" 61005001 61006000 "*" 4.08209022140227e-11 9.57770690255255e-11 100
"chr20" 61006001 61007000 "*" 1.54768420301821e-11 3.85404852168246e-11 -59.4377510040161
"chr20" 61018001 61019000 "*" 0 0 100
"chr20" 61157001 61158000 "*" 7.17275669748219e-06 8.03444987004551e-06 -75.2941176470588
"chr20" 61163001 61164000 "*" 0 0 -62.5833333333333
"chr20" 61165001 61166000 "*" 1.89581683684992e-12 5.32311367606535e-12 -100
"chr20" 61176001 61177000 "*" 1.82851681795881e-07 2.56099513054524e-07 66.6666666666667
"chr20" 61177001 61178000 "*" 8.88178419700125e-16 3.70670032207938e-15 60
"chr20" 61183001 61184000 "*" 3.9669761919825e-09 7.06247234072352e-09 -71.470207253886
"chr20" 61192001 61193000 "*" 0 0 100
"chr20" 61195001 61196000 "*" 4.22826752499716e-08 6.47129910406812e-08 -81.4814814814815
"chr20" 61268001 61269000 "*" 0 0 -91.8181818181818
"chr20" 61300001 61301000 "*" 0 0 -99.0131578947368
"chr20" 61309001 61310000 "*" 9.63829016598083e-12 2.46993429847588e-11 100
"chr20" 61313001 61314000 "*" 6.60582699651968e-14 2.22176301799e-13 -100
"chr20" 61317001 61318000 "*" 0 0 58.5106382978723
"chr20" 61323001 61324000 "*" 0 0 55.4767669779776
"chr20" 61325001 61326000 "*" 0 0 -76
"chr20" 61336001 61337000 "*" 0 0 63.768115942029
"chr20" 61342001 61343000 "*" 0 0 100
"chr20" 61355001 61356000 "*" 0 0 100
"chr20" 61360001 61361000 "*" 1.01030295240889e-14 3.75322807935163e-14 53.3333333333333
"chr20" 61404001 61405000 "*" 1.05997544075365e-10 2.35639968359142e-10 -60
"chr20" 61406001 61407000 "*" 8.58979554152484e-12 2.213554983903e-11 52.9411764705882
"chr20" 61423001 61424000 "*" 4.05744146803055e-06 4.71181941756713e-06 -62.8571428571429
"chr20" 61493001 61494000 "*" 0 0 -57.9391272460579
"chr20" 61502001 61503000 "*" 2.98663827569268e-06 3.54089377916502e-06 52.3344947735192
"chr20" 61531001 61532000 "*" 0 0 -81.8181818181818
"chr20" 61570001 61571000 "*" 0 0 -76.271186440678
"chr20" 61607001 61608000 "*" 2.91028312560115e-11 6.97590507139978e-11 100
"chr20" 61613001 61614000 "*" 1.27141630557048e-11 3.20605327517734e-11 -54.0229885057471
"chr20" 61624001 61625000 "*" 2.1852519793697e-12 6.0868191214295e-12 64.7058823529412
"chr20" 61650001 61651000 "*" 0 0 -89.4117647058823
"chr20" 61686001 61687000 "*" 5.50598079418485e-06 6.27403146214765e-06

-63.8888888888889
"chr20" 61701001 61702000 "*" 0 0 62.5046728971963
"chr20" 61702001 61703000 "*" 1.33337785257481e-13 4.3198011975565e-13
66.6666666666667
"chr20" 61711001 61712000 "*" 1.11066711383501e-11 2.82340927198126e-11
-70.4545454545455
"chr20" 61731001 61732000 "*" 1.33487071396488e-08 2.19761834835088e-08
-79.3103448275862
"chr20" 61733001 61734000 "*" 0 0 61.9880936168557
"chr20" 61738001 61739000 "*" 6.20625772995709e-12 1.63272418223453e-11
55.5555555555556
"chr20" 61752001 61753000 "*" 0 0 -73.684668989547
"chr20" 61774001 61775000 "*" 0 0 -100
"chr20" 61794001 61795000 "*" 3.10862446895044e-15 1.22466437093774e-14 -68.75
"chr20" 61800001 61801000 "*" 3.482998811144e-06 4.08626671722421e-06
54.2857142857143
"chr20" 61811001 61812000 "*" 6.92287338566189e-11 1.57605630124247e-10 -100
"chr20" 61840001 61841000 "*" 6.3086638842913e-06 7.12231755712437e-06
-58.0645161290323
"chr20" 61854001 61855000 "*" 1.99840144432528e-15 8.0570469535907e-15
76.9230769230769
"chr20" 61866001 61867000 "*" 1.13029585691038e-11 2.86884756834616e-11
-70.2702702702703
"chr20" 61873001 61874000 "*" 1.18504542845344e-08 1.96274997248588e-08
-85.3333333333333
"chr20" 61882001 61883000 "*" 3.05311331771918e-12 8.34639368772922e-12 -100
"chr20" 61899001 61900000 "*" 0 0 -100
"chr20" 61972001 61973000 "*" 2.64951482975562e-09 4.82186011375752e-09 -100
"chr20" 61984001 61985000 "*" 0 0 69.4915254237288
"chr20" 61998001 61999000 "*" 1.77635683940025e-15 7.19870740878856e-15
-54.8036758563074
"chr20" 62010001 62011000 "*" 4.44089209850063e-15 1.71914916534614e-14
67.8571428571429
"chr20" 62017001 62018000 "*" 2.25259931008193e-06 2.72138877153708e-06
-63.7931034482759
"chr20" 62073001 62074000 "*" 0 0 62.406015037594
"chr20" 62079001 62080000 "*" 0 0 51.0167464114833
"chr20" 62105001 62106000 "*" 0 0 87.9699248120301
"chr20" 62111001 62112000 "*" 1.32095667737531e-10 2.89894765044537e-10
-62.2222222222222
"chr20" 62116001 62117000 "*" 0 0 72.1311475409836
"chr20" 62133001 62134000 "*" 0 0 -57.9483583567068
"chr20" 62167001 62168000 "*" 0 0 -59.7956417474716
"chr20" 62168001 62169000 "*" 0 0 -85.2266666666667
"chr20" 62208001 62209000 "*" 5.50675727239991e-10 1.10770940054774e-09
-56.1226935058711
"chr20" 62274001 62275000 "*" 1.21707045973585e-05 1.31627471442909e-05
-60.3448275862069
"chr20" 62284001 62285000 "*" 0 0 75.0583119987679
"chr20" 62290001 62291000 "*" 0 0 -67.6493710691824
"chr20" 62300001 62301000 "*" 2.42722508758675e-11 5.89288146271158e-11
-92.5925925925926

"chr20" 62372001 62373000 "*" 5.90457316373971e-09 1.02505259500106e-08
-73.3333333333333
"chr20" 62409001 62410000 "*" 1.4432899320127e-15 5.90750815956055e-15
-98.0769230769231
"chr20" 62446001 62447000 "*" 0 0 87.9120879120879
"chr20" 62450001 62451000 "*" 0 0 100
"chr20" 62451001 62452000 "*" 2.28312193817359e-07 3.15824864044251e-07 -75
"chr20" 62460001 62461000 "*" 0 0 56.6276803118908
"chr20" 62478001 62479000 "*" 1.67299207820548e-08 2.71377429441055e-08 -100
"chr20" 62490001 62491000 "*" 0 0 -100
"chr20" 62495001 62496000 "*" 5.10702591327572e-15 1.96271840288234e-14 -100
"chr20" 62601001 62602000 "*" 0 0 58.2961777043273
"chr20" 62610001 62611000 "*" 0 0 -50.4668365342523
"chr20" 62634001 62635000 "*" 1.29037891483108e-11 3.24582280286866e-11 100
"chr20" 62691001 62692000 "*" 2.50655052269622e-11 6.07609535191461e-11
-66.6666666666667
"chr20" 62714001 62715000 "*" 0 0 55.9362301661049
"chr20" 62715001 62716000 "*" 0 0 50.6915402229436
"chr20" 62731001 62732000 "*" 1.61307704127367e-06 1.99008519856275e-06 -67.5
"chr20" 62732001 62733000 "*" 8.01666433236647e-09 1.36392368970109e-08 -100
"chr20" 62743001 62744000 "*" 0 0 50.8563899868248
"chr20" 62760001 62761000 "*" 1.32187696344488e-08 2.17786017778174e-08
81.0344827586207
"chr20" 62773001 62774000 "*" 0 0 56.0975609756098
"chr20" 62779001 62780000 "*" 3.95861121660346e-12 1.0684496262032e-11 -100
"chr20" 62787001 62788000 "*" 0 0 75.3424657534247
"chr20" 62796001 62797000 "*" 3.74444919515327e-12 1.01419093479931e-11
57.1428571428571
"chr20" 62802001 62803000 "*" 3.33066907387547e-16 1.4495649018245e-15
62.962962962963
"chr20" 62810001 62811000 "*" 6.59550958292954e-09 1.13456194382773e-08 100
"chr20" 62815001 62816000 "*" 0 0 100
"chr20" 62825001 62826000 "*" 4.71134242729931e-12 1.257615036806e-11 -100
"chr20" 62828001 62829000 "*" 2.79440914852103e-11 6.73046092948135e-11 -100
"chr20" 62830001 62831000 "*" 8.06610334080915e-12 2.08931809569875e-11
57.6923076923077
"chr20" 62859001 62860000 "*" 6.98872323123823e-06 7.84104914795963e-06
51.7241379310345
"chr20" 62861001 62862000 "*" 3.03979064142368e-13 9.42350883309283e-13 100
"chr20" 62877001 62878000 "*" 9.96869253810928e-13 2.89584084136165e-12
-68.1318681318681
"chr20" 62909001 62910000 "*" 0 0 -92.5373134328358
"chr21" 9912001 9913000 "*" 1.77224901420914e-12 4.99803208970683e-12
-74.5098039215686
"chr21" 10201001 10202000 "*" 1.67299207820548e-08 2.71377429441055e-08 -100
"chr21" 10613001 10614000 "*" 5.04148556235151e-09 8.82066396036712e-09
93.3333333333333
"chr21" 10730001 10731000 "*" 8.2022381598934e-06 9.10251728824986e-06
66.6666666666667
"chr21" 10776001 10777000 "*" 1.17461596005342e-13 3.8279067575877e-13 100
"chr21" 10781001 10782000 "*" 4.25703045037196e-08 6.51316810519424e-08 75
"chr21" 10785001 10786000 "*" 7.90290055618925e-12 2.04914476750626e-11

-73.1707317073171
"chr21" 10794001 10795000 "*" 7.0006535457523e-08 1.03695832512054e-07 100
"chr21" 10808001 10809000 "*" 4.28478405645194e-06 4.95677832652837e-06
71.1538461538462
"chr21" 10810001 10811000 "*" 7.69917077061422e-05 7.35006305210438e-05
-51.6129032258064
"chr21" 10812001 10813000 "*" 9.03721542044877e-14 2.98877811377079e-13
55.5555555555556
"chr21" 10830001 10831000 "*" 2.85782508768762e-12 7.83899372243486e-12
-59.6491228070175
"chr21" 10896001 10897000 "*" 0 0 -78.125
"chr21" 11085001 11086000 "*" 2.33603371065927e-05 2.4195479448311e-05
52.1212121212121
"chr21" 11144001 11145000 "*" 0.000342455907834882 0.000294074065554863
51.1494252873563
"chr21" 14732001 14733000 "*" 0 0 75
"chr21" 14889001 14890000 "*" 0 0 -100
"chr21" 15359001 15360000 "*" 4.04946233650838e-05 4.0376350196346e-05
-64.7058823529412
"chr21" 15387001 15388000 "*" 2.32899292074151e-08 3.69740801818502e-08
-61.0169491525424
"chr21" 15430001 15431000 "*" 2.69438782396492e-09 4.89935513071423e-09
-55.5102040816327
"chr21" 15465001 15466000 "*" 0 0 -71.1111111111111
"chr21" 15646001 15647000 "*" 0 0 54.9032767469404
"chr21" 16436001 16437000 "*" 0 0 89.7810218978102
"chr21" 16451001 16452000 "*" 8.90075568804605e-08 1.29980078783428e-07
-66.6666666666667
"chr21" 16662001 16663000 "*" 5.99210681073714e-11 1.37807711320413e-10
54.1488857278331
"chr21" 16704001 16705000 "*" 1.61237689866311e-12 4.56615813671789e-12 -100
"chr21" 16793001 16794000 "*" 3.33066907387547e-16 1.4495649018245e-15 -100
"chr21" 17340001 17341000 "*" 2.62900812231237e-13 8.21294938531841e-13 100
"chr21" 17769001 17770000 "*" 7.93359049566789e-08 1.16611793378838e-07
54.5454545454545
"chr21" 18878001 18879000 "*" 5.55111512312578e-16 2.36485094870365e-15 68.75
"chr21" 18885001 18886000 "*" 0 0 57.6219512195122
"chr21" 18906001 18907000 "*" 6.93112234273485e-13 2.05218842007357e-12 100
"chr21" 19275001 19276000 "*" 1.92946192356658e-09 3.59193011611483e-09 -100
"chr21" 19432001 19433000 "*" 2.4535928844216e-14 8.69144396197119e-14 -100
"chr21" 19616001 19617000 "*" 5.89336968115362e-09 1.02321412879568e-08
-52.3610427939006
"chr21" 19774001 19775000 "*" 0 0 100
"chr21" 19815001 19816000 "*" 1.93720595120794e-11 4.76309924836636e-11 100
"chr21" 20359001 20360000 "*" 7.9125284102588e-10 1.55554860384252e-09 -87.5
"chr21" 20360001 20361000 "*" 3.32729299667989e-09 5.99038474059895e-09
-69.4444444444444
"chr21" 20657001 20658000 "*" 2.33518887782225e-07 3.22641982615608e-07
-74.5098039215686
"chr21" 20933001 20934000 "*" 3.2700897545368e-10 6.78798757234889e-10
-66.6666666666667
"chr21" 21371001 21372000 "*" 0 0 -100

"chr21" 22369001 22370000 "*" 0 0 -53.3734194004775
"chr21" 23084001 23085000 "*" 7.0006535457523e-08 1.03695832512054e-07 -100
"chr21" 23100001 23101000 "*" 0 0 -100
"chr21" 23811001 23812000 "*" 1.37828859436695e-10 3.01445544267661e-10 -100
"chr21" 26931001 26932000 "*" 2.88710388929303e-10 6.03399264731139e-10 100
"chr21" 27184001 27185000 "*" 9.04570973681018e-10 1.76414389822173e-09 -100
"chr21" 27577001 27578000 "*" 4.44089209850063e-16 1.91071758245033e-15
-98.4732824427481
"chr21" 27998001 27999000 "*" 0 0 91.9354838709677
"chr21" 28336001 28337000 "*" 3.96347399345132e-11 9.32984740262792e-11
-66.6666666666667
"chr21" 28855001 28856000 "*" 3.11908276984241e-11 7.43983059948603e-11
82.3529411764706
"chr21" 29199001 29200000 "*" 4.85023132767992e-12 1.29319908309707e-11
74.6835443037975
"chr21" 29453001 29454000 "*" 7.0006535457523e-08 1.03695832512054e-07 100
"chr21" 29859001 29860000 "*" 2.43005615629954e-09 4.45014222280278e-09 100
"chr21" 29976001 29977000 "*" 8.3882012447134e-11 1.88922989503423e-10 -100
"chr21" 30375001 30376000 "*" 0 0 79.8165137614679
"chr21" 30396001 30397000 "*" 0 0 -87.9585326953748
"chr21" 30536001 30537000 "*" 2.37587727269783e-14 8.44268514944447e-14 100
"chr21" 30710001 30711000 "*" 1.83952852950142e-12 5.17499701329218e-12 -100
"chr21" 31470001 31471000 "*" 6.46149800331841e-14 2.17664365839349e-13 -100
"chr21" 31691001 31692000 "*" 0 0 -100
"chr21" 32397001 32398000 "*" 1.77980741256079e-10 3.84134306400813e-10
-66.6666666666667
"chr21" 32513001 32514000 "*" 0 0 -66.6666666666667
"chr21" 32718001 32719000 "*" 4.02167188440217e-12 1.08266700497926e-11 100
"chr21" 32843001 32844000 "*" 3.93892275870611e-05 3.93480384469493e-05
55.4838709677419
"chr21" 32868001 32869000 "*" 1.52794177310511e-09 2.87642564232045e-09 100
"chr21" 32943001 32944000 "*" 2.43005615629954e-09 4.45014222280278e-09 -100
"chr21" 32946001 32947000 "*" 8.77076189453874e-15 3.27889038038365e-14 100
"chr21" 33245001 33246000 "*" 0 0 69.8905109489051
"chr21" 33296001 33297000 "*" 2.05849160495042e-07 2.86428700589167e-07
-53.3333333333333
"chr21" 33314001 33315000 "*" 1.17461596005342e-13 3.8279067575877e-13 -100
"chr21" 33439001 33440000 "*" 3.74922315415915e-13 1.15034884906114e-12
95.6521739130435
"chr21" 33440001 33441000 "*" 0.00016961084973377 0.000153222593791434
-51.7241379310345
"chr21" 33446001 33447000 "*" 0 0 59.0909090909091
"chr21" 33732001 33733000 "*" 6.77335965093562e-12 1.76897011246462e-11 -100
"chr21" 33921001 33922000 "*" 8.7349629751543e-09 1.47505871898511e-08 -100
"chr21" 33953001 33954000 "*" 0 0 -65.8536585365854
"chr21" 34292001 34293000 "*" 0 0 100
"chr21" 34324001 34325000 "*" 3.34128893442198e-08 5.19772864618523e-08 100
"chr21" 34412001 34413000 "*" 9.08108033215171e-11 2.03407753381119e-10 -100
"chr21" 34448001 34449000 "*" 1.74583569823028e-10 3.77134050007725e-10 65.625
"chr21" 34482001 34483000 "*" 0 0 59.1530280487133
"chr21" 34556001 34557000 "*" 0 0 -72.8513650151668
"chr21" 34567001 34568000 "*" 7.0006535457523e-08 1.03695832512054e-07 100

"chr21" 34603001 34604000 "*" 0 0 84.6153846153846
"chr21" 34625001 34626000 "*" 3.95353005888666e-09 7.03928529283733e-09 100
"chr21" 34638001 34639000 "*" 0 0 92.3913043478261
"chr21" 34643001 34644000 "*" 4.43136049954607e-05 4.39299853239263e-05
-65.5172413793103
"chr21" 34660001 34661000 "*" 1.91224813761437e-12 5.36527594469447e-12 100
"chr21" 34734001 34735000 "*" 4.08209022140227e-11 9.57770690255255e-11 -100
"chr21" 34763001 34764000 "*" 2.08814465718632e-09 3.86232567960463e-09 -100
"chr21" 34993001 34994000 "*" 9.52204174803351e-05 8.95780133799817e-05
59.2592592592593
"chr21" 35401001 35402000 "*" 1.68753899743024e-14 6.09826805967439e-14 -100
"chr21" 35565001 35566000 "*" 2.38031816479634e-13 7.47299807843568e-13 100
"chr21" 35653001 35654000 "*" 2.61776622600962e-09 4.77711122078405e-09
61.5384615384615
"chr21" 35669001 35670000 "*" 2.08814465718632e-09 3.86232567960463e-09 100
"chr21" 35714001 35715000 "*" 0 0 -95.2380952380952
"chr21" 35779001 35780000 "*" 4.44089209850063e-15 1.71914916534614e-14 100
"chr21" 35889001 35890000 "*" 4.01313771103418e-09 7.11882087237587e-09 -100
"chr21" 35986001 35987000 "*" 0 0 66.6666666666667
"chr21" 36076001 36077000 "*" 7.0006535457523e-08 1.03695832512054e-07 -100
"chr21" 36079001 36080000 "*" 0 0 100
"chr21" 36227001 36228000 "*" 2.08814465718632e-09 3.86232567960463e-09 -100
"chr21" 36666001 36667000 "*" 1.37828859436695e-10 3.01445544267661e-10 -100
"chr21" 36709001 36710000 "*" 1.52466927971773e-12 4.33322475981408e-12 -100
"chr21" 36800001 36801000 "*" 3.03090885722668e-14 1.06210046699299e-13 100
"chr21" 37084001 37085000 "*" 1.56863411149288e-12 4.4469621057462e-12 100
"chr21" 37094001 37095000 "*" 2.00227675550835e-08 3.20459176617961e-08 100
"chr21" 37183001 37184000 "*" 4.28237856286984e-09 7.57162087834286e-09
60.6060606060606
"chr21" 37196001 37197000 "*" 0 0 100
"chr21" 37204001 37205000 "*" 0 0 61.0526315789474
"chr21" 37303001 37304000 "*" 0.000525299954949099 0.000436849389802675
-54.5454545454545
"chr21" 37355001 37356000 "*" 1.32162481070175e-07 1.88682311059292e-07
83.3333333333333
"chr21" 37366001 37367000 "*" 0 0 100
"chr21" 37393001 37394000 "*" 4.01313771103418e-09 7.11882087237587e-09 100
"chr21" 37442001 37443000 "*" 0 0 82.4480369515012
"chr21" 37528001 37529000 "*" 0 0 60.7142857142857
"chr21" 37582001 37583000 "*" 1.47400556205213e-08 2.41423981171165e-08
55.1724137931034
"chr21" 37639001 37640000 "*" 3.99680288865056e-14 1.37975970265307e-13 -100
"chr21" 37655001 37656000 "*" 3.6700841921089e-08 5.65530226072258e-08 -100
"chr21" 37827001 37828000 "*" 0 0 100
"chr21" 37881001 37882000 "*" 4.69801004654435e-07 6.23099316580956e-07
55.5555555555556
"chr21" 37898001 37899000 "*" 1.20591092667155e-10 2.66075324129686e-10 100
"chr21" 37914001 37915000 "*" 1.36604061395929e-11 3.42333952202875e-11 100
"chr21" 37961001 37962000 "*" 0 0 61.9047619047619
"chr21" 37979001 37980000 "*" 1.19647828710212e-05 1.29575155566641e-05
-60.8695652173913
"chr21" 38011001 38012000 "*" 2.00227675550835e-08 3.20459176617961e-08 -100

"chr21" 38026001 38027000 "*" 0 0 -60.6060606060606
"chr21" 38330001 38331000 "*" 0 0 57.6923076923077
"chr21" 38369001 38370000 "*" 9.80763359414993e-10 1.8980032424715e-09 100
"chr21" 38379001 38380000 "*" 0 0 56.7312686746244
"chr21" 38382001 38383000 "*" 0 0 100
"chr21" 38919001 38920000 "*" 0 0 -66.3461538461538
"chr21" 38924001 38925000 "*" 0 0 63.2734303912648
"chr21" 39065001 39066000 "*" 5.1281201507436e-13 1.54461625452838e-12 100
"chr21" 39112001 39113000 "*" 8.7349629751543e-09 1.47505871898511e-08 100
"chr21" 39597001 39598000 "*" 2.96524256027109e-06 3.51837835523079e-06
73.3333333333333
"chr21" 39614001 39615000 "*" 4.02167188440217e-12 1.08266700497926e-11 -100
"chr21" 39726001 39727000 "*" 3.21964677141295e-15 1.26720521345953e-14
66.6666666666667
"chr21" 39776001 39777000 "*" 1.25508492487825e-11 3.16621166034499e-11
59.2592592592593
"chr21" 39951001 39952000 "*" 7.93809462606987e-14 2.64679416473686e-13 100
"chr21" 39970001 39971000 "*" 1.52487595883599e-08 2.49295718350367e-08
66.6666666666667
"chr21" 40025001 40026000 "*" 0 0 -100
"chr21" 40033001 40034000 "*" 0 0 67.4376687212941
"chr21" 40094001 40095000 "*" 0 0 -100
"chr21" 40106001 40107000 "*" 0 0 80.8219178082192
"chr21" 40177001 40178000 "*" 0 0 -53.7190082644628
"chr21" 40189001 40190000 "*" 7.34908023325431e-09 1.25734591572739e-08 -95
"chr21" 40244001 40245000 "*" 0 0 100
"chr21" 40285001 40286000 "*" 1.0769163338864e-14 3.98631098593833e-14
-63.4502923976608
"chr21" 40307001 40308000 "*" 0 0 -100
"chr21" 40338001 40339000 "*" 5.52133250231179e-08 8.32925710218402e-08
76.984126984127
"chr21" 40342001 40343000 "*" 1.92946192356658e-09 3.59193011611483e-09 -100
"chr21" 40354001 40355000 "*" 0 0 -100
"chr21" 40362001 40363000 "*" 4.46118697539077e-11 1.04272771442106e-10
50.7042253521127
"chr21" 40384001 40385000 "*" 0 0 -58.8235294117647
"chr21" 40392001 40393000 "*" 3.6892711108294e-13 1.13266255090498e-12 -100
"chr21" 40407001 40408000 "*" 0.000257691937994853 0.000225933396425958 -52
"chr21" 40451001 40452000 "*" 0 0 -99.4117647058823
"chr21" 40454001 40455000 "*" 0 0 -78.7234042553192
"chr21" 40467001 40468000 "*" 1.19073048432394e-06 1.49613945244789e-06
54.1666666666667
"chr21" 40532001 40533000 "*" 1.86019089021272e-10 4.00881697003673e-10 -75
"chr21" 40686001 40687000 "*" 0 0 56.25
"chr21" 40758001 40759000 "*" 0 0 -64.2857142857143
"chr21" 40897001 40898000 "*" 2.3990809339125e-12 6.65441738642456e-12 -100
"chr21" 40901001 40902000 "*" 1.46658822863799e-08 2.40261015608166e-08 55
"chr21" 40981001 40982000 "*" 1.54700896537463e-09 2.91080938365739e-09
-72.7272727272727
"chr21" 40985001 40986000 "*" 0 0 78.5407725321888
"chr21" 40987001 40988000 "*" 4.73234496034536e-09 8.30627300264826e-09 -100
"chr21" 41022001 41023000 "*" 0 0 -62.0689655172414

"chr21" 41024001 41025000 "*" 2.88710388929303e-10 6.03399264731139e-10 100
"chr21" 41170001 41171000 "*" 9.67474404035551e-05 9.0904864753806e-05 58.0645161290323
"chr21" 41260001 41261000 "*" 0 0 88.2352941176471
"chr21" 41513001 41514000 "*" 5.14200543855203e-06 5.88639366700709e-06 70.5882352941177
"chr21" 41550001 41551000 "*" 5.87032228382967e-08 8.82523920088212e-08 -65.9090909090909
"chr21" 41663001 41664000 "*" 0 0 -86.9565217391304
"chr21" 41725001 41726000 "*" 1.38331568422245e-11 3.46517367072943e-11 54.1832669322709
"chr21" 41730001 41731000 "*" 2.15125472990962e-10 4.58126659943874e-10 100
"chr21" 41807001 41808000 "*" 1.2670753335442e-10 2.78634619866839e-10 -66.6666666666667
"chr21" 41836001 41837000 "*" 4.65637306490407e-10 9.46720524046618e-10 -100
"chr21" 41919001 41920000 "*" 2.33028818463765e-10 4.93719283454501e-10 100
"chr21" 41931001 41932000 "*" 3.47684745372057e-06 4.07927945666551e-06 -65.2542372881356
"chr21" 42100001 42101000 "*" 4.6209147619436e-11 1.07842934795068e-10 66.6666666666667
"chr21" 42163001 42164000 "*" 1.92946192356658e-09 3.59193011611483e-09 -100
"chr21" 42225001 42226000 "*" 6.70841160399505e-12 1.75486545176836e-11 -100
"chr21" 42254001 42255000 "*" 8.43347613965761e-12 2.17859001990012e-11 100
"chr21" 42382001 42383000 "*" 2.90305779415689e-10 6.06536007254861e-10 -80.3571428571429
"chr21" 42383001 42384000 "*" 0 0 -81.5126050420168
"chr21" 42384001 42385000 "*" 0 0 -56.4541738454782
"chr21" 42509001 42510000 "*" 1.54630752646767e-11 3.85079502648228e-11 -100
"chr21" 42512001 42513000 "*" 7.7876054271897e-08 1.14599333101476e-07 60
"chr21" 42698001 42699000 "*" 5.15576359383374e-10 1.04177898761168e-09 80.7228915662651
"chr21" 42736001 42737000 "*" 2.22044604925031e-16 9.81641919380259e-16 100
"chr21" 42750001 42751000 "*" 1.98325800226939e-11 4.85612402473442e-11 -100
"chr21" 42766001 42767000 "*" 8.05178146379149e-12 2.08586750924985e-11 52.9411764705882
"chr21" 42792001 42793000 "*" 3.70125700088764e-08 5.70049331569128e-08 -79.5275590551181
"chr21" 42849001 42850000 "*" 2.93489454961104e-10 6.12803872944259e-10 87.6923076923077
"chr21" 42850001 42851000 "*" 2.59385846135274e-11 6.27806615350756e-11 73.1707317073171
"chr21" 42852001 42853000 "*" 0 0 55.6451612903226
"chr21" 42879001 42880000 "*" 0 0 84.8661233993015
"chr21" 42905001 42906000 "*" 1.70898526441476e-08 2.76865893867779e-08 -78.021978021978
"chr21" 42921001 42922000 "*" 2.92057711348548e-10 6.100587743092e-10 -56.1920129084308
"chr21" 42952001 42953000 "*" 4.56972459872418e-10 9.30987166849462e-10 69.7368421052632
"chr21" 43023001 43024000 "*" 2.66453525910038e-14 9.40074020513994e-14 -100
"chr21" 43084001 43085000 "*" 6.50723919193297e-12 1.70813384097865e-11 -61.0169491525424

"chr21" 43104001 43105000 "*" 2.82773804372027e-13 8.79129664109438e-13 -100
"chr21" 43136001 43137000 "*" 0 0 54.2857142857143
"chr21" 43167001 43168000 "*" 1.89848137210902e-14 6.82212372863451e-14 100
"chr21" 43173001 43174000 "*" 0 0 -100
"chr21" 43237001 43238000 "*" 9.38138455808257e-14 3.09940584172412e-13
-54.4444444444444
"chr21" 43297001 43298000 "*" 3.04324343503026e-11 7.26599935673121e-11 -100
"chr21" 43306001 43307000 "*" 0 0 -100
"chr21" 43318001 43319000 "*" 8.62570015414121e-11 1.94046719905861e-10
-75.9259259259259
"chr21" 43353001 43354000 "*" 0 0 -76
"chr21" 43364001 43365000 "*" 0 0 -52.5252525252525
"chr21" 43369001 43370000 "*" 8.17846350642526e-08 1.20022814812237e-07
57.1428571428571
"chr21" 43384001 43385000 "*" 1.1801515320542e-10 2.60909395353556e-10
54.3308702791461
"chr21" 43388001 43389000 "*" 5.55111512312578e-16 2.36485094870365e-15 100
"chr21" 43469001 43470000 "*" 0 0 100
"chr21" 43487001 43488000 "*" 1.19571019752129e-13 3.88829351764533e-13
55.2941176470588
"chr21" 43491001 43492000 "*" 7.50402105020864e-06 8.37838983711095e-06
-66.6666666666667
"chr21" 43505001 43506000 "*" 3.33066907387547e-16 1.4495649018245e-15 100
"chr21" 43521001 43522000 "*" 2.55351295663786e-15 1.01678090381833e-14
73.7226277372263
"chr21" 43535001 43536000 "*" 7.99844594256971e-06 8.89173797399106e-06
-54.5454545454545
"chr21" 43539001 43540000 "*" 2.72842552995201e-08 4.2968501610316e-08
-51.2820512820513
"chr21" 43563001 43564000 "*" 1.11022302462516e-16 5.03662826618488e-16
66.6666666666667
"chr21" 43572001 43573000 "*" 2.88779000712225e-11 6.92935809466068e-11 100
"chr21" 43638001 43639000 "*" 1.27675647831893e-14 4.67848770441012e-14
56.1067153580848
"chr21" 43639001 43640000 "*" 0 0 56.2506214576912
"chr21" 43659001 43660000 "*" 2.19383400335005e-11 5.34788778871231e-11
-97.2972972972973
"chr21" 43660001 43661000 "*" 2.20525753213963e-09 4.06902688447164e-09
-52.6315789473684
"chr21" 43672001 43673000 "*" 4.22959001156187e-10 8.65582300346791e-10 -60
"chr21" 43684001 43685000 "*" 1.67299207820548e-08 2.71377429441055e-08 100
"chr21" 43736001 43737000 "*" 4.51551129820871e-09 7.9662089949093e-09 56
"chr21" 43741001 43742000 "*" 1.17442722213923e-11 2.97163462475643e-11
-53.8768751217612
"chr21" 43771001 43772000 "*" 0 0 100
"chr21" 43774001 43775000 "*" 3.52704532247117e-11 8.35581292290093e-11 100
"chr21" 43786001 43787000 "*" 5.42158273830751e-09 9.45316480711079e-09
67.3076923076923
"chr21" 43789001 43790000 "*" 3.59712259978551e-14 1.25126181142626e-13
62.2641509433962
"chr21" 43831001 43832000 "*" 3.33066907387547e-16 1.4495649018245e-15
-77.7777777777778

"chr21" 43865001 43866000 "*" 2.43005615629954e-09 4.45014222280278e-09 -100
"chr21" 43880001 43881000 "*" 9.65729496371637e-10 1.87255912493232e-09 100
"chr21" 43902001 43903000 "*" 7.82712499494131e-05 7.46478152805681e-05
56.5217391304348
"chr21" 43960001 43961000 "*" 1.35036426485158e-12 3.86629547363774e-12 -100
"chr21" 44014001 44015000 "*" 0 0 -91.8032786885246
"chr21" 44041001 44042000 "*" 5.48638023900594e-10 1.10374357336421e-09 100
"chr21" 44061001 44062000 "*" 1.1501433139216e-10 2.54545042978714e-10
-86.4864864864865
"chr21" 44071001 44072000 "*" 0 0 -88.562091503268
"chr21" 44074001 44075000 "*" 0 0 65.4008438818565
"chr21" 44092001 44093000 "*" 2.84381074244777e-10 5.96368614458273e-10
59.2592592592593
"chr21" 44103001 44104000 "*" 2.08748776042711e-07 2.90229945171996e-07
-59.2592592592593
"chr21" 44118001 44119000 "*" 1.16573417585641e-14 4.29335582900508e-14 100
"chr21" 44141001 44142000 "*" 1.67299207820548e-08 2.71377429441055e-08 -100
"chr21" 44201001 44202000 "*" 0 0 95.1219512195122
"chr21" 44203001 44204000 "*" 8.93018738534757e-06 9.85776757582955e-06
-61.0169491525424
"chr21" 44342001 44343000 "*" 0 0 100
"chr21" 44436001 44437000 "*" 2.52666776390242e-11 6.12308157839103e-11
-58.7301587301587
"chr21" 44440001 44441000 "*" 0 0 65.5172413793103
"chr21" 44575001 44576000 "*" 8.82599619300706e-06 9.74972217948123e-06
-59.2592592592593
"chr21" 44585001 44586000 "*" 5.10027686750902e-10 1.03096692961214e-09 72.5
"chr21" 44600001 44601000 "*" 1.12634901405784e-10 2.49563754426169e-10 -100
"chr21" 44616001 44617000 "*" 5.48638023900594e-10 1.10374357336421e-09 -100
"chr21" 44727001 44728000 "*" 6.93806123663876e-11 1.5792553022765e-10
-70.5882352941177
"chr21" 44728001 44729000 "*" 1.09455956720517e-06 1.38200609773459e-06
-54.6875
"chr21" 44745001 44746000 "*" 2.03166461432147e-09 3.7732651503826e-09
66.6666666666667
"chr21" 44761001 44762000 "*" 0 0 -82.1786893573787
"chr21" 44781001 44782000 "*" 0 0 -51.6814627550121
"chr21" 44785001 44786000 "*" 0 0 100
"chr21" 44884001 44885000 "*" 4.10782519111308e-15 1.59859775077462e-14
67.5675675675676
"chr21" 44887001 44888000 "*" 1.12489795256465e-10 2.49480848992626e-10 60
"chr21" 44992001 44993000 "*" 3.6427075800205e-09 6.5252880340617e-09
-66.6666666666667
"chr21" 45032001 45033000 "*" 1.11022302462516e-16 5.03662826618488e-16
-84.8484848484848
"chr21" 45068001 45069000 "*" 0 0 -100
"chr21" 45089001 45090000 "*" 1.11022302462516e-16 5.03662826618488e-16 100
"chr21" 45132001 45133000 "*" 4.1153940362193e-06 4.77326970666537e-06
66.6666666666667
"chr21" 45136001 45137000 "*" 4.32986979603811e-15 1.68006959513174e-14 100
"chr21" 45151001 45152000 "*" 2.00284233642378e-13 6.35465444640339e-13
66.6666666666667

"chr21" 45195001 45196000 "*" 0 0 55.5555555555556
"chr21" 45235001 45236000 "*" 0 0 -56.390977443609
"chr21" 45247001 45248000 "*" 0 0 63.855421686747
"chr21" 45272001 45273000 "*" 0 0 -100
"chr21" 45359001 45360000 "*" 0 0 -61.5756302521008
"chr21" 45407001 45408000 "*" 4.85167461761193e-14 1.6617373494005e-13 100
"chr21" 45624001 45625000 "*" 5.30686605770825e-14 1.8057048995867e-13
52.3809523809524
"chr21" 45631001 45632000 "*" 4.79616346638068e-14 1.64380445416596e-13 -100
"chr21" 45633001 45634000 "*" 6.92287338566189e-11 1.57605630124247e-10 100
"chr21" 45644001 45645000 "*" 1.72100800277519e-09 3.22495937542133e-09
53.0952380952381
"chr21" 45664001 45665000 "*" 7.7715611723761e-16 3.26213507634405e-15 100
"chr21" 45682001 45683000 "*" 5.05706587716759e-12 1.34366440083293e-11
-95.2380952380952
"chr21" 45706001 45707000 "*" 0 0 61.0810810810811
"chr21" 45710001 45711000 "*" 0 0 -63.3333333333333
"chr21" 45764001 45765000 "*" 3.67483821150927e-14 1.27627245811088e-13
-50.6329113924051
"chr21" 45789001 45790000 "*" 0 0 64.8125830474954
"chr21" 45819001 45820000 "*" 0 0 50.2878835811452
"chr21" 45845001 45846000 "*" 0 0 -56.8181818181818
"chr21" 45846001 45847000 "*" 0 0 -74.3119266055046
"chr21" 45945001 45946000 "*" 1.1330425486733e-10 2.5092017593116e-10
67.6923076923077
"chr21" 46000001 46001000 "*" 0 0 52.7465069860279
"chr21" 46046001 46047000 "*" 1.14124265593318e-11 2.89138062669327e-11 100
"chr21" 46056001 46057000 "*" 2.33982844211056e-09 4.30200663260379e-09
-73.9130434782609
"chr21" 46065001 46066000 "*" 8.3882012447134e-11 1.88922989503423e-10 100
"chr21" 46085001 46086000 "*" 0 0 83.3333333333333
"chr21" 46114001 46115000 "*" 2.38575468822777e-05 2.46816531377115e-05
66.6666666666667
"chr21" 46120001 46121000 "*" 3.49848927605478e-10 7.23006353364999e-10
-82.1917808219178
"chr21" 46121001 46122000 "*" 0 0 -81.8181818181818
"chr21" 46229001 46230000 "*" 2.88657986402541e-15 1.14155613124573e-14 100
"chr21" 46279001 46280000 "*" 0 0 -55.9322033898305
"chr21" 46303001 46304000 "*" 4.48663328711518e-12 1.20116956597601e-11
-54.6875
"chr21" 46352001 46353000 "*" 0 0 62.9315018977047
"chr21" 46419001 46420000 "*" 0 0 67.9245283018868
"chr21" 46455001 46456000 "*" 4.50885773162213e-10 9.19305634880493e-10
-57.1428571428571
"chr21" 46469001 46470000 "*" 2.64795850801747e-08 4.17690999234936e-08
76.7441860465116
"chr21" 46478001 46479000 "*" 2.15125472990962e-10 4.58126659943874e-10 -100
"chr21" 46484001 46485000 "*" 1.14651987903613e-09 2.19787056960479e-09
-79.3103448275862
"chr21" 46569001 46570000 "*" 2.00227675550835e-08 3.20459176617961e-08 -100
"chr21" 46574001 46575000 "*" 9.65729496371637e-10 1.87255912493232e-09 -100
"chr21" 46597001 46598000 "*" 1.38590361409285e-10 3.02706178892116e-10 100

"chr21" 46621001 46622000 "*" 0 0 100
"chr21" 46625001 46626000 "*" 1.32463262281224e-06 1.65411876342065e-06 -52.3809523809524
"chr21" 46651001 46652000 "*" 8.26947311860504e-06 9.17188458178241e-06 -67.1232876712329
"chr21" 46656001 46657000 "*" 2.69018141096922e-12 7.41487347763769e-12 100
"chr21" 46666001 46667000 "*" 4.89385216051463e-06 5.61726783723613e-06 -63.7931034482759
"chr21" 46673001 46674000 "*" 7.48072703782299e-08 1.10340422120243e-07 -64.3939393939394
"chr21" 46690001 46691000 "*" 0 0 -100
"chr21" 46754001 46755000 "*" 3.33066907387547e-16 1.4495649018245e-15 -77.4193548387097
"chr21" 46776001 46777000 "*" 3.02275793284679e-09 5.46819335770128e-09 73.3333333333333
"chr21" 46808001 46809000 "*" 8.43769498715119e-15 3.16245570171727e-14 -53.1339977851606
"chr21" 46811001 46812000 "*" 6.9747714270818e-09 1.19647208487175e-08 65.5172413793103
"chr21" 46823001 46824000 "*" 0 0 55.327868852459
"chr21" 46854001 46855000 "*" 4.4531045517715e-12 1.19280583021799e-11 62.2641509433962
"chr21" 46856001 46857000 "*" 4.47782921852991e-11 1.04638295807416e-10 -52.4364406779661
"chr21" 46992001 46993000 "*" 5.1281201507436e-13 1.54461625452838e-12 -100
"chr21" 47029001 47030000 "*" 3.90954095252916e-06 4.55140971080702e-06 -68.75
"chr21" 47030001 47031000 "*" 2.82678370677747e-06 3.36347040828362e-06 56.6037735849057
"chr21" 47037001 47038000 "*" 9.0072393987839e-13 2.63157725646847e-12 100
"chr21" 47050001 47051000 "*" 0 0 -53.6945812807882
"chr21" 47058001 47059000 "*" 3.95353005888666e-09 7.03928529283733e-09 -100
"chr21" 47063001 47064000 "*" 0 0 62.1676034747062
"chr21" 47064001 47065000 "*" 0 0 74.9478079331942
"chr21" 47124001 47125000 "*" 1.73138170467269e-11 4.28978464366132e-11 -65.3846153846154
"chr21" 47209001 47210000 "*" 0 0 -97.8798586572438
"chr21" 47217001 47218000 "*" 0 0 -100
"chr21" 47249001 47250000 "*" 1.39779633423487e-07 1.98463037958589e-07 -100
"chr21" 47313001 47314000 "*" 3.56131733214582e-07 4.80158368901456e-07 -66.6666666666667
"chr21" 47347001 47348000 "*" 3.2085445411667e-14 1.12142674960866e-13 -73.8461538461538
"chr21" 47399001 47400000 "*" 0 0 69.3154761904762
"chr21" 47451001 47452000 "*" 4.16633780092601e-05 4.14702975818333e-05 -51.219512195122
"chr21" 47454001 47455000 "*" 3.6700841921089e-08 5.65530226072258e-08 -100
"chr21" 47466001 47467000 "*" 1.34906900095544e-08 2.21932680341382e-08 85.1351351351351
"chr21" 47497001 47498000 "*" 9.7399638021578e-08 1.41531991781167e-07 -71.6666666666667
"chr21" 47511001 47512000 "*" 2.22044604925031e-15 8.90449334476539e-15 -71.4285714285714

"chr21" 47519001 47520000 "*" 0 0 66.3551401869159
"chr21" 47533001 47534000 "*" 4.88498130835069e-15 1.8824139250584e-14 100
"chr21" 47544001 47545000 "*" 0 0 61.2259755387303
"chr21" 47553001 47554000 "*" 0 0 100
"chr21" 47732001 47733000 "*" 8.01666433236647e-09 1.36392368970109e-08 -100
"chr21" 48088001 48089000 "*" 2.08832950931992e-13 6.60553481926902e-13
-90.5882352941177
"chr22" 16201001 16202000 "*" 0 0 -94.4444444444444
"chr22" 16339001 16340000 "*" 2.91028312560115e-11 6.97590507139978e-11 -100
"chr22" 16420001 16421000 "*" 0 0 100
"chr22" 16856001 16857000 "*" 0 0 -100
"chr22" 16869001 16870000 "*" 7.82712499494131e-05 7.46478152805681e-05
56.5217391304348
"chr22" 16878001 16879000 "*" 2.22044604925031e-16 9.81641919380259e-16 100
"chr22" 17039001 17040000 "*" 1.13140401492018e-08 1.87801380787728e-08 100
"chr22" 17041001 17042000 "*" 1.51088033917279e-06 1.87200608100818e-06
-84.6153846153846
"chr22" 17048001 17049000 "*" 3.38335419769464e-06 3.97764446268794e-06
68.5185185185185
"chr22" 17072001 17073000 "*" 0 0 -68.75
"chr22" 17239001 17240000 "*" 1.49383437841877e-08 2.444556877867e-08
66.6666666666667
"chr22" 17341001 17342000 "*" 3.6700841921089e-08 5.65530226072258e-08 100
"chr22" 17442001 17443000 "*" 1.16573417585641e-14 4.29335582900508e-14 100
"chr22" 17445001 17446000 "*" 0 0 100
"chr22" 17491001 17492000 "*" 1.67299207820548e-08 2.71377429441055e-08 100
"chr22" 17537001 17538000 "*" 0 0 -54.2857142857143
"chr22" 17542001 17543000 "*" 4.21884749357559e-15 1.63874445239189e-14 100
"chr22" 17565001 17566000 "*" 7.7188255787064e-12 2.00322203463915e-11
64.390243902439
"chr22" 17593001 17594000 "*" 0 0 -72.2222222222222
"chr22" 17595001 17596000 "*" 0 0 100
"chr22" 17601001 17602000 "*" 0 0 53.0201718527574
"chr22" 17602001 17603000 "*" 0 0 66.2072526512293
"chr22" 17675001 17676000 "*" 0.000403052046117347 0.000341881569533569
52.9411764705882
"chr22" 17679001 17680000 "*" 0 0 55
"chr22" 17711001 17712000 "*" 1.37828859436695e-10 3.01445544267661e-10 -100
"chr22" 17751001 17752000 "*" 2.83661982791727e-13 8.81792354118164e-13
-85.5263157894737
"chr22" 17758001 17759000 "*" 3.33066907387547e-16 1.4495649018245e-15 -100
"chr22" 17767001 17768000 "*" 3.18010950906e-10 6.61437851003952e-10 -75
"chr22" 17836001 17837000 "*" 2.02060590481778e-13 6.40127405465654e-13 100
"chr22" 17868001 17869000 "*" 0 0 100
"chr22" 17893001 17894000 "*" 0 0 100
"chr22" 17959001 17960000 "*" 0 0 100
"chr22" 18010001 18011000 "*" 6.39841080118941e-09 1.10576663486113e-08
84.4961240310078
"chr22" 18032001 18033000 "*" 4.86753970463383e-11 1.13145908161915e-10 -100
"chr22" 18044001 18045000 "*" 2.15125472990962e-10 4.58126659943874e-10 -100
"chr22" 18054001 18055000 "*" 0 0 95.8620689655172
"chr22" 18225001 18226000 "*" 2.88710388929303e-10 6.03399264731139e-10 100

"chr22" 18251001 18252000 "*" 1.07882591748876e-11 2.74553537455803e-11 100
"chr22" 18283001 18284000 "*" 0 0 58.9473684210526
"chr22" 18373001 18374000 "*" 0 0 100
"chr22" 18400001 18401000 "*" 0 0 57.9508803283953
"chr22" 18430001 18431000 "*" 0 0 -100
"chr22" 18438001 18439000 "*" 4.24031642021205e-08 6.48914354320773e-08 -62.5
"chr22" 18448001 18449000 "*" 1.25284005392245e-10 2.75649590272477e-10 -100
"chr22" 18469001 18470000 "*" 2.4535928844216e-14 8.69144396197119e-14 -100
"chr22" 18470001 18471000 "*" 3.07531777821168e-14 1.07626052665081e-13 -100
"chr22" 18506001 18507000 "*" 0 0 -53.3333333333333
"chr22" 18507001 18508000 "*" 0 0 -81.9742489270386
"chr22" 18548001 18549000 "*" 0 0 92.6829268292683
"chr22" 18612001 18613000 "*" 5.33790485035546e-07 7.03071689168268e-07
-66.6666666666667
"chr22" 18640001 18641000 "*" 6.01530692967422e-06 6.81427821060929e-06
-67.9012345679012
"chr22" 18837001 18838000 "*" 0.000170900372238547 0.00015425218250759
53.8461538461538
"chr22" 18901001 18902000 "*" 2.08748765606614e-07 2.90229945171996e-07
-59.2592592592593
"chr22" 18944001 18945000 "*" 0 0 -100
"chr22" 18952001 18953000 "*" 0 0 100
"chr22" 18957001 18958000 "*" 0 0 63.09963099631
"chr22" 18982001 18983000 "*" 1.68454139526375e-12 4.76324551413128e-12
60.4166666666667
"chr22" 19004001 19005000 "*" 7.43027861460632e-12 1.93110132482767e-11
59.5505617977528
"chr22" 19023001 19024000 "*" 9.67004254448511e-14 3.18178990782609e-13 -100
"chr22" 19055001 19056000 "*" 2.35367281220533e-14 8.37555800557029e-14
-57.1428571428571
"chr22" 19139001 19140000 "*" 7.0006535457523e-08 1.03695832512054e-07 -100
"chr22" 19165001 19166000 "*" 0 0 65.6453440362568
"chr22" 19167001 19168000 "*" 8.96851255483e-08 1.30893916746167e-07
81.5384615384615
"chr22" 19170001 19171000 "*" 0 0 70.4545454545455
"chr22" 19197001 19198000 "*" 2.23421281475567e-12 6.22007897750945e-12
53.4883720930233
"chr22" 19232001 19233000 "*" 1.07882591748876e-11 2.74553537455803e-11 -100
"chr22" 19370001 19371000 "*" 1.04366204567441e-09 2.01374565079736e-09
62.962962962963
"chr22" 19406001 19407000 "*" 1.50266585818848e-06 1.86246459132231e-06
53.6585365853659
"chr22" 19531001 19532000 "*" 5.48638023900594e-10 1.10374357336421e-09 100
"chr22" 19542001 19543000 "*" 0 0 -100
"chr22" 19583001 19584000 "*" 3.34128893442198e-08 5.19772864618523e-08 100
"chr22" 19586001 19587000 "*" 3.40125705378114e-10 7.03784073980162e-10 100
"chr22" 19587001 19588000 "*" 1.96933358509455e-10 4.2312335719586e-10
77.1929824561404
"chr22" 19592001 19593000 "*" 1.9610443070972e-05 2.05436946691909e-05
-66.6666666666667
"chr22" 19594001 19595000 "*" 8.77076189453874e-15 3.27889038038365e-14
-83.3333333333333

"chr22" 19598001 19599000 "*" 2.12001965871345e-07 2.94434925523572e-07
-55.5555555555556
"chr22" 19616001 19617000 "*" 1.72084568816899e-13 5.50960611810785e-13
96.969696969697
"chr22" 19628001 19629000 "*" 1.98294731001969e-06 2.41330824932622e-06
56.5217391304348
"chr22" 19674001 19675000 "*" 4.99900121297969e-12 1.32921056339624e-11 -100
"chr22" 19702001 19703000 "*" 0 0 81.4155148576188
"chr22" 19716001 19717000 "*" 8.12662159788147e-11 1.83492407415953e-10
88.0239520958084
"chr22" 19729001 19730000 "*" 1.21014309684142e-14 4.44905345865734e-14
63.6363636363636
"chr22" 19739001 19740000 "*" 0 0 86.2903225806452
"chr22" 19741001 19742000 "*" 3.90997234589463e-11 9.21066599197414e-11 80
"chr22" 19753001 19754000 "*" 0 0 50.2894252708789
"chr22" 19754001 19755000 "*" 0 0 52.2236655001475
"chr22" 19764001 19765000 "*" 2.08995487582797e-10 4.46944006972663e-10 100
"chr22" 19766001 19767000 "*" 1.13725884764992e-06 1.43296856092383e-06
-72.2222222222222
"chr22" 19859001 19860000 "*" 1.00641051048456e-10 2.24372044192682e-10
-56.7266775777414
"chr22" 19897001 19898000 "*" 0 0 -96.1648745519713
"chr22" 19929001 19930000 "*" 1.01767538840392e-09 1.96571242605259e-09
76.9230769230769
"chr22" 19941001 19942000 "*" 0 0 -100
"chr22" 19974001 19975000 "*" 0 0 -50.4539559014267
"chr22" 20000001 20001000 "*" 5.63660229602192e-13 1.68905058935478e-12 100
"chr22" 20001001 20002000 "*" 2.60353898218213e-07 3.5753307375039e-07
51.063829787234
"chr22" 20092001 20093000 "*" 8.57092175010621e-13 2.51149198099779e-12 -100
"chr22" 20160001 20161000 "*" 9.11671580805518e-06 1.0052892250396e-05 -60
"chr22" 20163001 20164000 "*" 1.55431223447522e-15 6.33705141101682e-15
56.701030927835
"chr22" 20174001 20175000 "*" 8.12683254025615e-13 2.39237407085304e-12
82.1428571428571
"chr22" 20204001 20205000 "*" 0 0 100
"chr22" 20209001 20210000 "*" 0 0 53.0769230769231
"chr22" 20210001 20211000 "*" 1.22124532708767e-15 5.03921984217778e-15 100
"chr22" 20251001 20252000 "*" 0 0 -80.8641975308642
"chr22" 20261001 20262000 "*" 0 0 -62.4329692154916
"chr22" 20266001 20267000 "*" 0.000174588508199403 0.000157342656028109
-58.6206896551724
"chr22" 20272001 20273000 "*" 8.75692571788989e-08 1.28029890642991e-07 -56.8
"chr22" 20274001 20275000 "*" 0 0 -75
"chr22" 20277001 20278000 "*" 0 0 -76.5957446808511
"chr22" 20284001 20285000 "*" 3.34128893442198e-08 5.19772864618523e-08 -100
"chr22" 20308001 20309000 "*" 6.55031584528842e-15 2.48816226484197e-14
-84.4155844155844
"chr22" 20781001 20782000 "*" 1.69376734859839e-11 4.20083199148365e-11
96.2962962962963
"chr22" 20861001 20862000 "*" 0 0 -100
"chr22" 20885001 20886000 "*" 0 0 -100

"chr22" 20918001 20919000 "*" 2.15125472990962e-10 4.58126659943874e-10 -100
"chr22" 20925001 20926000 "*" 3.63445940010365e-11 8.58565209111204e-11 100
"chr22" 20955001 20956000 "*" 1.91489268885903e-10 4.11999548210973e-10
76.7441860465116
"chr22" 20970001 20971000 "*" 5.05151476204446e-14 1.7263557281889e-13
60.6060606060606
"chr22" 21021001 21022000 "*" 7.37983563148248e-10 1.4555253161506e-09 70
"chr22" 21023001 21024000 "*" 3.96248602081428e-05 3.95701587394277e-05
-51.1111111111111
"chr22" 21122001 21123000 "*" 3.10862446895044e-15 1.22466437093774e-14 100
"chr22" 21141001 21142000 "*" 6.24654217240561e-10 1.2462108191303e-09 100
"chr22" 21316001 21317000 "*" 1.07882591748876e-11 2.74553537455803e-11 -100
"chr22" 21352001 21353000 "*" 5.87992754486066e-09 1.02105033599712e-08
62.962962962963
"chr22" 21368001 21369000 "*" 0 0 65.7400543612552
"chr22" 21369001 21370000 "*" 0 0 100
"chr22" 21375001 21376000 "*" 2.27948777742348e-07 3.15400606163355e-07
66.6666666666667
"chr22" 21379001 21380000 "*" 3.5527136788005e-15 1.39277426410519e-14
-77.2727272727273
"chr22" 21397001 21398000 "*" 0.000525299954949099 0.000436849389802675
54.5454545454545
"chr22" 21398001 21399000 "*" 3.25818815749557e-05 3.29735679006858e-05
66.6666666666667
"chr22" 21400001 21401000 "*" 0 0 64.1791044776119
"chr22" 21401001 21402000 "*" 0 0 60.5964052287582
"chr22" 21403001 21404000 "*" 1.27220456391797e-12 3.65644590663683e-12
-86.4197530864197
"chr22" 21406001 21407000 "*" 0 0 -100
"chr22" 21409001 21410000 "*" 0 0 93.6170212765958
"chr22" 21431001 21432000 "*" 0 0 -52.9258883248731
"chr22" 21434001 21435000 "*" 9.7687799984425e-05 9.1741893612339e-05
52.1739130434783
"chr22" 21451001 21452000 "*" 1.33122005641084e-08 2.191997641159e-08
92.3076923076923
"chr22" 21457001 21458000 "*" 1.58285373885736e-06 1.95525661000438e-06
58.8235294117647
"chr22" 21811001 21812000 "*" 1.99110505860745e-10 4.27184805977799e-10
-63.3333333333333
"chr22" 21824001 21825000 "*" 0 0 67.8125
"chr22" 21921001 21922000 "*" 0 0 100
"chr22" 21990001 21991000 "*" 0 0 -71.969696969697
"chr22" 21994001 21995000 "*" 2.00227675550835e-08 3.20459176617961e-08 -100
"chr22" 22042001 22043000 "*" 1.65423230669148e-14 5.98698695225911e-14 -100
"chr22" 22051001 22052000 "*" 4.91745150155154e-08 7.46593756906867e-08
-67.741935483871
"chr22" 22106001 22107000 "*" 0 0 -100
"chr22" 22292001 22293000 "*" 0 0 -84.1543388744853
"chr22" 22319001 22320000 "*" 2.23138518773425e-09 4.11432348480929e-09
53.8461538461538
"chr22" 22384001 22385000 "*" 2.73014943985572e-11 6.58595916221891e-11 100
"chr22" 22465001 22466000 "*" 1.92946192356658e-09 3.59193011611483e-09 100

"chr22" 22568001 22569000 "*" 2.17381668221606e-13 6.85151076607736e-13 100
"chr22" 22601001 22602000 "*" 1.56863411149288e-12 4.4469621057462e-12 -100
"chr22" 22703001 22704000 "*" 6.92287338566189e-11 1.57605630124247e-10 100
"chr22" 22914001 22915000 "*" 1.67299207820548e-08 2.71377429441055e-08 100
"chr22" 22958001 22959000 "*" 1.5277158427196e-09 2.87642564232045e-09 100
"chr22" 22961001 22962000 "*" 0 0 76
"chr22" 23191001 23192000 "*" 5.48638023900594e-10 1.10374357336421e-09 -100
"chr22" 23230001 23231000 "*" 3.31147540832966e-07 4.48459595110263e-07
65.5172413793103
"chr22" 23231001 23232000 "*" 2.1094237467878e-15 8.47434540879246e-15 100
"chr22" 23236001 23237000 "*" 8.88178419700125e-16 3.70670032207938e-15 100
"chr22" 23259001 23260000 "*" 0 0 -100
"chr22" 23267001 23268000 "*" 0.000174588517416696 0.000157342656028109
58.6206896551724
"chr22" 23272001 23273000 "*" 4.71077067927261e-05 4.65201892615276e-05
62.2222222222222
"chr22" 23275001 23276000 "*" 6.14967032852576e-09 1.065052727662e-08
67.5675675675676
"chr22" 23380001 23381000 "*" 8.01666433236647e-09 1.36392368970109e-08 -100
"chr22" 23389001 23390000 "*" 7.93809462606987e-14 2.64679416473686e-13 100
"chr22" 23409001 23410000 "*" 0.000321268697584531 0.000277075512580754 60
"chr22" 23410001 23411000 "*" 2.71893618730701e-13 8.46692454027087e-13 -100
"chr22" 23417001 23418000 "*" 6.59550958292954e-09 1.13456194382773e-08 100
"chr22" 23451001 23452000 "*" 1.12458486967171e-09 2.15740815043925e-09 100
"chr22" 23466001 23467000 "*" 3.26800283612094e-07 4.42859928232537e-07
-65.1685393258427
"chr22" 23482001 23483000 "*" 6.67013488931945e-05 6.43065783242906e-05
-52.3809523809524
"chr22" 23487001 23488000 "*" 0 0 -88.5310290652003
"chr22" 23505001 23506000 "*" 2.64795847471078e-08 4.17690999234936e-08
58.3333333333333
"chr22" 23515001 23516000 "*" 0 0 -100
"chr22" 23519001 23520000 "*" 3.51385587293862e-11 8.33569499258023e-11
52.6403700372607
"chr22" 23580001 23581000 "*" 5.0548121244276e-11 1.17211570113753e-10
55.8823529411765
"chr22" 23583001 23584000 "*" 4.10749212420569e-12 1.10502147013417e-11
62.2222222222222
"chr22" 23612001 23613000 "*" 0 0 100
"chr22" 23727001 23728000 "*" 3.35015414298923e-08 5.21025604149515e-08
-68.1159420289855
"chr22" 23745001 23746000 "*" 0 0 -91.4302623791675
"chr22" 23760001 23761000 "*" 0 0 -81.592039800995
"chr22" 23808001 23809000 "*" 1.35447209004269e-14 4.94674708073053e-14 -100
"chr22" 23853001 23854000 "*" 5.07371922253697e-14 1.73287898516782e-13 -100
"chr22" 23897001 23898000 "*" 1.87627691161651e-14 6.74814792086625e-14
-70.5882352941177
"chr22" 23905001 23906000 "*" 2.08995487582797e-10 4.46944006972663e-10 100
"chr22" 23915001 23916000 "*" 1.47430528896209e-08 2.41468330640695e-08
-51.7241379310345
"chr22" 23949001 23950000 "*" 0 0 -66.6666666666667
"chr22" 24061001 24062000 "*" 2.82075643731616e-07 3.85584889618316e-07 -80

"chr22" 24076001 24077000 "*" 4.99900121297969e-12 1.32921056339624e-11 -100
"chr22" 24083001 24084000 "*" 3.6700841921089e-08 5.65530226072258e-08 100
"chr22" 24111001 24112000 "*" 6.92287338566189e-11 1.57605630124247e-10 -100
"chr22" 24140001 24141000 "*" 2.55351295663786e-15 1.01678090381833e-14 100
"chr22" 24147001 24148000 "*" 1.97184979455756e-08 3.17049637913348e-08
-58.6206896551724
"chr22" 24158001 24159000 "*" 2.62900812231237e-13 8.21294938531841e-13 100
"chr22" 24164001 24165000 "*" 2.3990809339125e-12 6.65441738642456e-12 100
"chr22" 24171001 24172000 "*" 1.11022302462516e-16 5.03662826618488e-16 -100
"chr22" 24238001 24239000 "*" 3.81419340556022e-11 8.9931694108601e-11
-52.1739130434783
"chr22" 24297001 24298000 "*" 2.47563636257553e-10 5.23132152838563e-10
-57.9545454545455
"chr22" 24374001 24375000 "*" 0 0 -81.5789473684211
"chr22" 24513001 24514000 "*" 1.39779633423487e-07 1.98463037958589e-07 -100
"chr22" 24551001 24552000 "*" 0 0 -59.4948954746252
"chr22" 24552001 24553000 "*" 0 0 -52.6957200937293
"chr22" 24565001 24566000 "*" 1.34141907581764e-08 2.20765850734954e-08
54.1666666666667
"chr22" 24666001 24667000 "*" 0 0 -100
"chr22" 24797001 24798000 "*" 1.0551626239419e-10 2.34638843495876e-10
-62.8571428571429
"chr22" 24831001 24832000 "*" 1.90181204118289e-13 6.05298617539364e-13 100
"chr22" 24906001 24907000 "*" 3.63445940010365e-11 8.58565209111204e-11 100
"chr22" 24907001 24908000 "*" 5.63660229602192e-13 1.68905058935478e-12 -100
"chr22" 24919001 24920000 "*" 3.10329539843224e-12 8.47829787178896e-12
-67.741935483871
"chr22" 25093001 25094000 "*" 4.65294469620403e-13 1.40984134650271e-12
-55.9139784946237
"chr22" 25134001 25135000 "*" 1.20591092667155e-10 2.66075324129686e-10 100
"chr22" 25203001 25204000 "*" 3.15626080826803e-10 6.56821496688106e-10 96
"chr22" 25210001 25211000 "*" 5.48638023900594e-10 1.10374357336421e-09 100
"chr22" 25220001 25221000 "*" 1.96644922567657e-11 4.82930612417392e-11 100
"chr22" 25223001 25224000 "*" 6.75905029834389e-07 8.78517448080164e-07
52.1780303030303
"chr22" 25263001 25264000 "*" 2.68450373042128e-10 5.64465213127486e-10 -100
"chr22" 25279001 25280000 "*" 3.47937334499449e-09 6.24921205966029e-09
54.2372881355932
"chr22" 25493001 25494000 "*" 8.01666433236647e-09 1.36392368970109e-08 -100
"chr22" 25519001 25520000 "*" 1.67299207820548e-08 2.71377429441055e-08 -100
"chr22" 25536001 25537000 "*" 6.00350913426695e-09 1.0412745056916e-08
51.6129032258064
"chr22" 25538001 25539000 "*" 2.02884612776622e-07 2.82539375333911e-07
81.304347826087
"chr22" 25573001 25574000 "*" 1.33226762955019e-14 4.8714125500746e-14
88.3720930232558
"chr22" 25598001 25599000 "*" 1.15907283770866e-13 3.78316697336986e-13
75.609756097561
"chr22" 25604001 25605000 "*" 1.45513899046934e-05 1.55529419444988e-05
-61.2903225806452
"chr22" 25714001 25715000 "*" 0.000110843658785664 0.000103206787806409
58.974358974359

"chr22" 25723001 25724000 "*" 0 0 100
"chr22" 25730001 25731000 "*" 3.88022947106492e-13 1.1867462770515e-12 100
"chr22" 25785001 25786000 "*" 8.67652622971171e-07 1.1107452797228e-06
54.1666666666667
"chr22" 25791001 25792000 "*" 7.21644966006352e-15 2.72345776413425e-14 -100
"chr22" 25796001 25797000 "*" 0 0 -58.0645161290323
"chr22" 25797001 25798000 "*" 0 0 -100
"chr22" 25811001 25812000 "*" 1.19731371169829e-05 1.29653851905981e-05
-65.1162790697674
"chr22" 25856001 25857000 "*" 8.7349629751543e-09 1.47505871898511e-08 -100
"chr22" 25960001 25961000 "*" 0 0 56.1959654178674
"chr22" 26157001 26158000 "*" 2.09519928384694e-06 2.54242805999191e-06 52.875
"chr22" 26162001 26163000 "*" 3.6700841921089e-08 5.65530226072258e-08 100
"chr22" 26180001 26181000 "*" 1.30744385318238e-08 2.15506005383921e-08
76.5432098765432
"chr22" 26231001 26232000 "*" 5.63420986532392e-08 8.488944423918e-08
65.3061224489796
"chr22" 26236001 26237000 "*" 1.55326570827619e-07 2.19312390210205e-07
78.5714285714286
"chr22" 26566001 26567000 "*" 0 0 100
"chr22" 26797001 26798000 "*" 1.39779633423487e-07 1.98463037958589e-07 100
"chr22" 26838001 26839000 "*" 2.16382467499443e-13 6.82775854166559e-13 100
"chr22" 26844001 26845000 "*" 2.88710388929303e-10 6.03399264731139e-10 100
"chr22" 26866001 26867000 "*" 1.35036426485158e-12 3.86629547363774e-12 100
"chr22" 26988001 26989000 "*" 1.25284005392245e-10 2.75649590272477e-10 100
"chr22" 27006001 27007000 "*" 2.56321526226344e-07 3.52320422170438e-07
-54.9295774647887
"chr22" 27008001 27009000 "*" 2.03970174084134e-12 5.69795835861569e-12
-66.6666666666667
"chr22" 27015001 27016000 "*" 8.88178419700125e-16 3.70670032207938e-15 100
"chr22" 27041001 27042000 "*" 0 0 -62.9396122834105
"chr22" 27053001 27054000 "*" 0 0 58.6392670157068
"chr22" 27065001 27066000 "*" 5.04263297784746e-13 1.52078941532818e-12 -100
"chr22" 27152001 27153000 "*" 0 0 51.4245967359729
"chr22" 27154001 27155000 "*" 0 0 100
"chr22" 27162001 27163000 "*" 2.88710388929303e-10 6.03399264731139e-10 100
"chr22" 27225001 27226000 "*" 8.01666433236647e-09 1.36392368970109e-08 -100
"chr22" 27281001 27282000 "*" 9.08108033215171e-11 2.03407753381119e-10 100
"chr22" 27288001 27289000 "*" 9.67004254448511e-14 3.18178990782609e-13 -100
"chr22" 27496001 27497000 "*" 0 0 -81.1111111111111
"chr22" 27505001 27506000 "*" 0 0 -100
"chr22" 27530001 27531000 "*" 4.78841410966879e-11 1.11468187156588e-10 -100
"chr22" 27555001 27556000 "*" 0 0 -100
"chr22" 27579001 27580000 "*" 8.88178419700125e-16 3.70670032207938e-15 96
"chr22" 27601001 27602000 "*" 1.98124849859482e-10 4.25157611354621e-10 -100
"chr22" 27633001 27634000 "*" 2.6542434916621e-11 6.41751484698500e-11
70.5882352941177
"chr22" 27636001 27637000 "*" 5.03441732746523e-12 1.3378171545226e-11 -100
"chr22" 27684001 27685000 "*" 0 0 62.2984135308771
"chr22" 27723001 27724000 "*" 3.52704532247117e-11 8.35581292290093e-11 100
"chr22" 27742001 27743000 "*" 0 0 -94.137022397892
"chr22" 27802001 27803000 "*" 2.33028818463765e-10 4.93719283454501e-10 -100

"chr22" 27805001 27806000 "*" 0 0 70.5680256527714
"chr22" 27884001 27885000 "*" 2.4535928844216e-14 8.69144396197119e-14 -100
"chr22" 27890001 27891000 "*" 0 0 100
"chr22" 27891001 27892000 "*" 0.000241621869001674 0.000212792315955059
57.6923076923077
"chr22" 27897001 27898000 "*" 1.12634901405784e-10 2.49563754426169e-10 100
"chr22" 27900001 27901000 "*" 2.51910625692631e-08 3.98224378422946e-08
60.3921568627451
"chr22" 27947001 27948000 "*" 3.6700841921089e-08 5.65530226072258e-08 100
"chr22" 27956001 27957000 "*" 2.84732681876676e-10 5.97064881384397e-10
-57.8947368421053
"chr22" 27976001 27977000 "*" 9.63829016598083e-12 2.46993429847588e-11 -100
"chr22" 27978001 27979000 "*" 2.02327044007689e-12 5.65300384792621e-12 -100
"chr22" 27987001 27988000 "*" 0 0 -80.4934210526316
"chr22" 28009001 28010000 "*" 2.88710388929303e-10 6.03399264731139e-10 -100
"chr22" 28010001 28011000 "*" 0 0 51.3971160174714
"chr22" 28037001 28038000 "*" 9.5812247025151e-14 3.15667542866807e-13 -100
"chr22" 28081001 28082000 "*" 4.13089673578781e-10 8.46277737348438e-10
76.7441860465116
"chr22" 28101001 28102000 "*" 8.75060357508062e-10 1.7117141314083e-09
88.135593220339
"chr22" 28103001 28104000 "*" 3.81968487053186e-05 3.82379755710845e-05
-54.5454545454545
"chr22" 28106001 28107000 "*" 7.44848627221018e-13 2.20091743656881e-12
80.0505050505051
"chr22" 28158001 28159000 "*" 4.44089209850063e-16 1.91071758245033e-15
-88.4297520661157
"chr22" 28162001 28163000 "*" 3.88022947106492e-13 1.1867462770515e-12 100
"chr22" 28179001 28180000 "*" 9.08108033215171e-11 2.03407753381119e-10 -100
"chr22" 28197001 28198000 "*" 0 0 76.3715372080391
"chr22" 28203001 28204000 "*" 6.59550958292954e-09 1.13456194382773e-08 100
"chr22" 28402001 28403000 "*" 1.40193623376916e-09 2.66180528624095e-09
-57.1428571428571
"chr22" 28414001 28415000 "*" 6.70841160399505e-12 1.75486545176836e-11 -100
"chr22" 28427001 28428000 "*" 1.99281884993852e-07 2.77767997023852e-07 90
"chr22" 28493001 28494000 "*" 7.17417665108133e-06 8.03571539886257e-06
74.0740740740741
"chr22" 29075001 29076000 "*" 0 0 76.1047989993935
"chr22" 29076001 29077000 "*" 0 0 57.0397111913357
"chr22" 29149001 29150000 "*" 0 0 100
"chr22" 29281001 29282000 "*" 6.4152538836737e-10 1.27477697728417e-09 -100
"chr22" 29341001 29342000 "*" 6.99440505513849e-15 2.64480529781883e-14 -100
"chr22" 29400001 29401000 "*" 3.68371999570627e-13 1.13149976091305e-12 100
"chr22" 29427001 29428000 "*" 1.98124849859482e-10 4.25157611354621e-10 -100
"chr22" 29468001 29469000 "*" 5.295763827462e-14 1.80214784640623e-13 -100
"chr22" 29474001 29475000 "*" 6.60582699651968e-14 2.22176301799e-13 100
"chr22" 29586001 29587000 "*" 0 0 -55.0873655913978
"chr22" 29597001 29598000 "*" 3.71924713249427e-14 1.29110427909498e-13
-94.1176470588235
"chr22" 29603001 29604000 "*" 1.13140401492018e-08 1.87801380787728e-08 -100
"chr22" 29787001 29788000 "*" 5.81824915185658e-05 5.66333692500392e-05
-53.8461538461538

"chr22" 29808001 29809000 "*" 2.68450373042128e-10 5.64465213127486e-10 -100
"chr22" 29860001 29861000 "*" 6.70841160399505e-12 1.75486545176836e-11 -100
"chr22" 29963001 29964000 "*" 1.83952852950142e-12 5.17499701329218e-12 -100
"chr22" 29977001 29978000 "*" 0 0 100
"chr22" 30025001 30026000 "*" 0 0 100
"chr22" 30102001 30103000 "*" 1.20591092667155e-10 2.66075324129686e-10 100
"chr22" 30107001 30108000 "*" 0 0 -92.9411764705882
"chr22" 30112001 30113000 "*" 5.295763827462e-14 1.80214784640623e-13 100
"chr22" 30113001 30114000 "*" 7.44226902327227e-12 1.93403677928798e-11
83.2526230831316
"chr22" 30115001 30116000 "*" 0 0 59.1232671412514
"chr22" 30121001 30122000 "*" 0 0 52.7027027027027
"chr22" 30123001 30124000 "*" 1.35016442470715e-11 3.38925638226179e-11
78.4313725490196
"chr22" 30131001 30132000 "*" 6.15840711759574e-13 1.83676803572802e-12 100
"chr22" 30144001 30145000 "*" 1.60982338570648e-14 5.83411082284088e-14 54
"chr22" 30148001 30149000 "*" 3.84137166520304e-14 1.32929369817675e-13 -100
"chr22" 30149001 30150000 "*" 2.16382467499443e-13 6.82775854166559e-13 -100
"chr22" 30162001 30163000 "*" 3.24995585998522e-12 8.86330021397331e-12
-51.9480519480519
"chr22" 30182001 30183000 "*" 1.12687636999453e-13 3.68543487142567e-13 -100
"chr22" 30183001 30184000 "*" 3.63445940010365e-11 8.58565209111204e-11 -100
"chr22" 30601001 30602000 "*" 0 0 -100
"chr22" 30603001 30604000 "*" 0.000224398531401881 0.000198755270160845
53.3333333333333
"chr22" 30641001 30642000 "*" 1.37923006349183e-12 3.94487974816619e-12
-92.8571428571429
"chr22" 30644001 30645000 "*" 7.66732788370916e-10 1.50965716509256e-09
-81.7204301075269
"chr22" 30647001 30648000 "*" 0 0 -74.2424242424242
"chr22" 30659001 30660000 "*" 0 0 -82.4732824427481
"chr22" 30673001 30674000 "*" 0 0 100
"chr22" 30686001 30687000 "*" 4.57183624291702e-10 9.31326369875184e-10
-90.9090909090909
"chr22" 30761001 30762000 "*" 1.40781186708239e-09 2.67223111923349e-09
-54.5454545454545
"chr22" 30782001 30783000 "*" 1.93978166862507e-11 4.76901049825701e-11
-55.5555555555556
"chr22" 30790001 30791000 "*" 1.07066806753764e-07 1.54793993440068e-07
-81.8181818181818
"chr22" 30807001 30808000 "*" 7.0006535457523e-08 1.03695832512054e-07 100
"chr22" 30841001 30842000 "*" 2.37587727269783e-14 8.44268514944447e-14 100
"chr22" 30846001 30847000 "*" 0.000451115314036077 0.000379477851858741
-52.1739130434783
"chr22" 30881001 30882000 "*" 0.000120483966197127 0.000111549020346707 56
"chr22" 30884001 30885000 "*" 2.00227675550835e-08 3.20459176617961e-08 -100
"chr22" 30891001 30892000 "*" 2.0820028701074e-08 3.32485381445412e-08
79.1044776119403
"chr22" 30902001 30903000 "*" 0 0 96.7213114754098
"chr22" 30921001 30922000 "*" 1.75082170983387e-13 5.6014942024636e-13 100
"chr22" 30924001 30925000 "*" 0 0 100
"chr22" 30939001 30940000 "*" 3.43058914609173e-14 1.19587680585466e-13

59.4594594594595
"chr22" 30947001 30948000 "*" 4.32209823486573e-12 1.15935290479641e-11 -100
"chr22" 30956001 30957000 "*" 4.11987440829265e-07 5.50491688288806e-07
-67.741935483871
"chr22" 30970001 30971000 "*" 0 0 74.0040628483084
"chr22" 31049001 31050000 "*" 2.04117044155794e-07 2.84127576499834e-07
52.9411764705882
"chr22" 31088001 31089000 "*" 1.12634901405784e-10 2.49563754426169e-10 -100
"chr22" 31158001 31159000 "*" 3.88022947106492e-13 1.1867462770515e-12 100
"chr22" 31183001 31184000 "*" 0 0 100
"chr22" 31218001 31219000 "*" 0 0 63.9973891507634
"chr22" 31220001 31221000 "*" 6.72456246242348e-09 1.15564227525732e-08 56.25
"chr22" 31224001 31225000 "*" 1.92625803974167e-07 2.68993984113541e-07 52
"chr22" 31317001 31318000 "*" 4.13947411570526e-07 5.53030830644023e-07
-75.4716981132076
"chr22" 31327001 31328000 "*" 0 0 -100
"chr22" 31342001 31343000 "*" 2.22660986411993e-05 2.3134811016358e-05
-53.3333333333333
"chr22" 31451001 31452000 "*" 1.28345509275984e-06 1.6057763296708e-06
59.4594594594595
"chr22" 31603001 31604000 "*" 4.01313771103418e-09 7.11882087237587e-09 100
"chr22" 31662001 31663000 "*" 0 0 -98.9795918367347
"chr22" 31669001 31670000 "*" 1.99840144432528e-15 8.0570469535907e-15 68.75
"chr22" 31687001 31688000 "*" 0 0 63.7462235649547
"chr22" 32026001 32027000 "*" 0 0 -92.0382165605096
"chr22" 32262001 32263000 "*" 1.93720595120794e-11 4.76309924836636e-11 -100
"chr22" 32520001 32521000 "*" 0 0 63.265306122449
"chr22" 32605001 32606000 "*" 3.34128893442198e-08 5.19772864618523e-08 100
"chr22" 32608001 32609000 "*" 3.66318381517239e-06 4.2844613387728e-06
-72.7272727272727
"chr22" 32737001 32738000 "*" 2.46357648865381e-05 2.54173878212536e-05 52.5
"chr22" 32810001 32811000 "*" 9.5812247025151e-14 3.15667542866807e-13 -100
"chr22" 32924001 32925000 "*" 6.40800805495889e-06 7.22831013269588e-06
54.1666666666667
"chr22" 33012001 33013000 "*" 2.14273043752655e-14 7.65660719399733e-14
-62.1394230769231
"chr22" 33020001 33021000 "*" 1.97383664968243e-10 4.24014404203188e-10
61.5384615384615
"chr22" 33022001 33023000 "*" 0.00020930071782832 0.000186304773393882
-58.3333333333333
"chr22" 33150001 33151000 "*" 4.01313771103418e-09 7.11882087237587e-09 100
"chr22" 33197001 33198000 "*" 0 0 50.120089491347
"chr22" 33214001 33215000 "*" 2.00227675550835e-08 3.20459176617961e-08 -100
"chr22" 33276001 33277000 "*" 6.67186759512051e-05 6.43207036784863e-05
54.6511627906977
"chr22" 33291001 33292000 "*" 1.00808250635964e-13 3.31107370956809e-13 100
"chr22" 33347001 33348000 "*" 7.08254540604525e-06 7.93982511214607e-06
63.855421686747
"chr22" 33437001 33438000 "*" 6.81146250514075e-11 1.55544800479628e-10
-56.4102564102564
"chr22" 33562001 33563000 "*" 2.88710388929303e-10 6.03399264731139e-10 100
"chr22" 33670001 33671000 "*" 0 0 100

"chr22" 33713001 33714000 "*" 4.98067487253451e-09 8.71957375821299e-09
54.5454545454545
"chr22" 33802001 33803000 "*" 4.44089209850063e-15 1.71914916534614e-14 -100
"chr22" 33811001 33812000 "*" 1.98124849859482e-10 4.25157611354621e-10 100
"chr22" 34083001 34084000 "*" 0 0 88.5714285714286
"chr22" 34133001 34134000 "*" 5.44009282066327e-15 2.08486137681812e-14 100
"chr22" 34315001 34316000 "*" 3.89607252793134e-07 5.22319826834853e-07
50.8503401360544
"chr22" 34730001 34731000 "*" 3.84336960645992e-06 4.47937651296003e-06
-76.4705882352941
"chr22" 35454001 35455000 "*" 4.90407947348892e-05 4.82947780802418e-05
-53.2818532818533
"chr22" 35540001 35541000 "*" 2.64951482975562e-09 4.82186011375752e-09 100
"chr22" 35588001 35589000 "*" 8.00762828668811e-05 7.62332949242677e-05
52.1739130434783
"chr22" 35625001 35626000 "*" 4.01313771103418e-09 7.11882087237587e-09 100
"chr22" 35733001 35734000 "*" 1.92946192356658e-09 3.59193011611483e-09 -100
"chr22" 35747001 35748000 "*" 0 0 -55.0880108785979
"chr22" 35777001 35778000 "*" 0 0 -62.9310344827586
"chr22" 35786001 35787000 "*" 0 0 100
"chr22" 35858001 35859000 "*" 9.07473543665382e-06 1.00087182976611e-05
-66.6666666666667
"chr22" 35876001 35877000 "*" 0 0 -68.75
"chr22" 35919001 35920000 "*" 8.39094571603027e-10 1.64494937720704e-09
52.1739130434783
"chr22" 35921001 35922000 "*" 3.88578058618805e-15 1.5152981210988e-14 100
"chr22" 35931001 35932000 "*" 8.06688049692639e-13 2.37556391305009e-12 100
"chr22" 35958001 35959000 "*" 1.15463194561016e-14 4.25829614430727e-14
-85.8490566037736
"chr22" 35975001 35976000 "*" 0 0 100
"chr22" 35979001 35980000 "*" 7.93809462606987e-14 2.64679416473686e-13 100
"chr22" 35996001 35997000 "*" 1.66533453693773e-15 6.7629186866784e-15
92.8571428571429
"chr22" 36013001 36014000 "*" 5.23711640720848e-09 9.14701594673664e-09
-56.9230769230769
"chr22" 36016001 36017000 "*" 8.79143562393381e-08 1.28496145949125e-07
67.6056338028169
"chr22" 36020001 36021000 "*" 1.37601430878487e-05 1.47579219146799e-05
67.3913043478261
"chr22" 36037001 36038000 "*" 1.11022302462516e-16 5.03662826618488e-16 100
"chr22" 36044001 36045000 "*" 1.08311192792421e-07 1.5646019706886e-07
53.8461538461538
"chr22" 36109001 36110000 "*" 1.83304482703761e-11 4.52714667747443e-11
96.969696969697
"chr22" 36519001 36520000 "*" 1.99840144432528e-15 8.0570469535907e-15 100
"chr22" 36544001 36545000 "*" 0.000165437398924162 0.000149718354277254
-54.4117647058824
"chr22" 36552001 36553000 "*" 2.1094237467878e-15 8.47434540879246e-15 100
"chr22" 36563001 36564000 "*" 6.93112234273485e-13 2.05218842007357e-12 -100
"chr22" 36576001 36577000 "*" 0 0 76.9230769230769
"chr22" 36747001 36748000 "*" 4.73234496034536e-09 8.30627300264826e-09 -100
"chr22" 36754001 36755000 "*" 1.77959869063216e-11 4.40062179040773e-11

58.3333333333333
"chr22" 36764001 36765000 "*" 4.71134242729931e-12 1.257615036806e-11 -100
"chr22" 36767001 36768000 "*" 1.6053158802265e-11 3.99118984252235e-11
-81.6326530612245
"chr22" 36822001 36823000 "*" 8.5131901528257e-13 2.4986134296918e-12
76.1194029850746
"chr22" 36831001 36832000 "*" 0 0 68.0851063829787
"chr22" 36848001 36849000 "*" 7.21999127151207e-11 1.64065256432683e-10
-64.6017699115044
"chr22" 36869001 36870000 "*" 1.15972064318459e-06 1.45972698357591e-06
51.7241379310345
"chr22" 36945001 36946000 "*" 1.98124849859482e-10 4.25157611354621e-10 100
"chr22" 36962001 36963000 "*" 6.21724893790088e-15 2.36606385879553e-14
-76.9230769230769
"chr22" 36966001 36967000 "*" 1.88349336127658e-12 5.2940367709714e-12 60
"chr22" 36989001 36990000 "*" 0 0 62.1111677715451
"chr22" 37005001 37006000 "*" 0 0 -100
"chr22" 37045001 37046000 "*" 1.5785740136165e-06 1.9503745122263e-06
66.6666666666667
"chr22" 37082001 37083000 "*" 3.10862446895044e-15 1.22466437093774e-14 -100
"chr22" 37139001 37140000 "*" 1.55431223447522e-15 6.33705141101682e-15 100
"chr22" 37150001 37151000 "*" 2.68673971959288e-14 9.47266815431045e-14
-82.7586206896552
"chr22" 37213001 37214000 "*" 0 0 52.2205967105555
"chr22" 37242001 37243000 "*" 2.4535928844216e-14 8.69144396197119e-14 100
"chr22" 37247001 37248000 "*" 5.6362137179633e-11 1.29963270300065e-10 100
"chr22" 37261001 37262000 "*" 0 0 -74.8363338788871
"chr22" 37262001 37263000 "*" 6.59550958292954e-09 1.13456194382773e-08 -100
"chr22" 37273001 37274000 "*" 8.32544299760318e-05 7.90441752423741e-05
-58.0645161290323
"chr22" 37310001 37311000 "*" 1.73743464060294e-09 3.25384007288034e-09
66.6666666666667
"chr22" 37421001 37422000 "*" 2.02983543307056e-08 3.24558454470611e-08
51.0204081632653
"chr22" 37422001 37423000 "*" 2.50910403565285e-14 8.87903196465237e-14 -100
"chr22" 37464001 37465000 "*" 6.88338275267597e-15 2.60518659957509e-14 100
"chr22" 37470001 37471000 "*" 0 0 53.8311688311688
"chr22" 37475001 37476000 "*" 3.16784820597604e-09 5.71726966031043e-09
63.4146341463415
"chr22" 37480001 37481000 "*" 1.35550015656349e-10 2.9721752497615e-10
-85.7142857142857
"chr22" 37488001 37489000 "*" 0 0 86.4864864864865
"chr22" 37496001 37497000 "*" 1.11022302462516e-16 5.03662826618488e-16
87.8453038674033
"chr22" 37500001 37501000 "*" 2.88779000712225e-11 6.92935809466068e-11 -100
"chr22" 37504001 37505000 "*" 7.7715611723761e-16 3.26213507634405e-15 100
"chr22" 37509001 37510000 "*" 2.1094237467878e-15 8.47434540879246e-15 100
"chr22" 37511001 37512000 "*" 8.96610711231816e-08 1.30861118541309e-07 52
"chr22" 37523001 37524000 "*" 0 0 92.1348314606742
"chr22" 37524001 37525000 "*" 3.01124347679149e-10 6.28100752075489e-10
-67.0886075949367
"chr22" 37531001 37532000 "*" 4.27807678171632e-10 8.74110895401584e-10

60.663082437276
"chr22" 37571001 37572000 "*" 1.02716278815862e-08 1.71992636592045e-08
84.5360824742268
"chr22" 37577001 37578000 "*" 2.61235477694299e-13 8.17255745261122e-13
-55.1724137931034
"chr22" 37581001 37582000 "*" 8.88178419700125e-16 3.70670032207938e-15
-51.1217948717949
"chr22" 37584001 37585000 "*" 0 0 -90
"chr22" 37638001 37639000 "*" 4.44089209850063e-16 1.91071758245033e-15 -100
"chr22" 37639001 37640000 "*" 4.29291602266346e-10 8.77013982761394e-10
95.4545454545455
"chr22" 37654001 37655000 "*" 3.34128893442198e-08 5.19772864618523e-08 100
"chr22" 37695001 37696000 "*" 1.32517415929456e-07 1.89159889248804e-07
-64.2857142857143
"chr22" 37709001 37710000 "*" 4.55959257342764e-05 4.51180768024439e-05
51.4374514374514
"chr22" 37710001 37711000 "*" 0 0 98.4455958549223
"chr22" 37772001 37773000 "*" 0 0 100
"chr22" 37791001 37792000 "*" 9.67004254448511e-14 3.18178990782609e-13 100
"chr22" 37793001 37794000 "*" 0 0 -51.0380479735318
"chr22" 37809001 37810000 "*" 1.26565424807268e-14 4.63923203146481e-14 100
"chr22" 37827001 37828000 "*" 0 0 97.8260869565217
"chr22" 37858001 37859000 "*" 4.99020824662466e-11 1.15790792586157e-10 -100
"chr22" 37892001 37893000 "*" 0 0 -71.2581991651759
"chr22" 37894001 37895000 "*" 8.76154804263507e-09 1.4792785255972e-08
85.2272727272727
"chr22" 37905001 37906000 "*" 3.61290507178147e-07 4.86547393947888e-07
-52.3809523809524
"chr22" 37908001 37909000 "*" 1.70228731111166e-09 3.19109795885331e-09
69.6969696969697
"chr22" 37915001 37916000 "*" 0 0 -58.4158415841584
"chr22" 37934001 37935000 "*" 2.91028312560115e-11 6.97590507139978e-11 100
"chr22" 37945001 37946000 "*" 3.33066907387547e-16 1.4495649018245e-15 -100
"chr22" 37963001 37964000 "*" 0 0 -100
"chr22" 37972001 37973000 "*" 2.27526627649688e-07 3.14837392572814e-07
-54.8387096774194
"chr22" 38006001 38007000 "*" 5.59736062055727e-07 7.35711789432905e-07
-66.6666666666667
"chr22" 38019001 38020000 "*" 7.26694260322347e-11 1.65091683132473e-10
54.1666666666667
"chr22" 38062001 38063000 "*" 9.04570973681018e-10 1.76414389822173e-09 100
"chr22" 38071001 38072000 "*" 0 0 -70.9302325581395
"chr22" 38076001 38077000 "*" 8.7349629751543e-09 1.47505871898511e-08 100
"chr22" 38146001 38147000 "*" 5.47240031067986e-12 1.44944740856163e-11
-50.5175734232066
"chr22" 38177001 38178000 "*" 2.12790891473968e-06 2.57994252743677e-06
-71.1111111111111
"chr22" 38213001 38214000 "*" 9.41103766758378e-05 8.86297231089954e-05 -60
"chr22" 38218001 38219000 "*" 3.88578058618805e-15 1.5152981210988e-14 100
"chr22" 38273001 38274000 "*" 0.000231703492257207 0.000204716698390421
-52.9411764705882
"chr22" 38301001 38302000 "*" 0 0 -84.2105263157895

"chr22" 38303001 38304000 "*" 5.63901248784582e-06 6.41584484544392e-06
-53.4722222222222
"chr22" 38339001 38340000 "*" 0 0 51.3513513513514
"chr22" 38371001 38372000 "*" 2.21042339987321e-09 4.07819777337298e-09 87.5
"chr22" 38391001 38392000 "*" 2.02060590481778e-13 6.40127405465654e-13 100
"chr22" 38396001 38397000 "*" 4.64135434419255e-07 6.15986676197051e-07
-59.2592592592593
"chr22" 38432001 38433000 "*" 0 0 -100
"chr22" 38452001 38453000 "*" 3.33066907387547e-16 1.4495649018245e-15
62.0689655172414
"chr22" 38535001 38536000 "*" 8.7349629751543e-09 1.47505871898511e-08 -100
"chr22" 38614001 38615000 "*" 0 0 -72.7272727272727
"chr22" 38693001 38694000 "*" 2.55608867405499e-11 6.19150117430613e-11
51.8518518518519
"chr22" 38741001 38742000 "*" 6.39165064653113e-07 8.33531059852563e-07
-92.3076923076923
"chr22" 38799001 38800000 "*" 0 0 -85.1485148514851
"chr22" 38832001 38833000 "*" 7.3229193842006e-05 7.01270617172158e-05
-59.2592592592593
"chr22" 38842001 38843000 "*" 0 0 100
"chr22" 38850001 38851000 "*" 0 0 81.8747056580924
"chr22" 38851001 38852000 "*" 0 0 80.6222707423581
"chr22" 38861001 38862000 "*" 0 0 68.8888888888889
"chr22" 38966001 38967000 "*" 0 0 64.2384105960265
"chr22" 39178001 39179000 "*" 0 0 100
"chr22" 39257001 39258000 "*" 2.90130868931815e-05 2.96184839910609e-05
53.4883720930233
"chr22" 39264001 39265000 "*" 0 0 -100
"chr22" 39269001 39270000 "*" 0 0 81.16930171278
"chr22" 39278001 39279000 "*" 0.000320710195636176 0.000276619581787338
52.3809523809524
"chr22" 39330001 39331000 "*" 9.00168828366077e-13 2.63157725646847e-12 52.5
"chr22" 39331001 39332000 "*" 1.15990550497713e-11 2.937207455111e-11
64.5161290322581
"chr22" 39345001 39346000 "*" 7.67056418382595e-11 1.7359448580318e-10 100
"chr22" 39360001 39361000 "*" 3.05599878736018e-10 6.36989975018463e-10
76.9230769230769
"chr22" 39383001 39384000 "*" 6.59550958292954e-09 1.13456194382773e-08 100
"chr22" 39395001 39396000 "*" 2.09832151654155e-12 5.85504623594219e-12
60.0831600831601
"chr22" 39409001 39410000 "*" 1.36604061395929e-11 3.42333952202875e-11 100
"chr22" 39410001 39411000 "*" 9.38682975792915e-09 1.5788727503198e-08
56.4102564102564
"chr22" 39488001 39489000 "*" 1.56863411149288e-12 4.4469621057462e-12 100
"chr22" 39493001 39494000 "*" 3.53310225520431e-09 6.34187411210968e-09
56.5217391304348
"chr22" 39584001 39585000 "*" 8.69011083648452e-07 1.11229659818976e-06
66.6666666666667
"chr22" 39585001 39586000 "*" 3.45657893365825e-05 3.48312803960454e-05
-51.2820512820513
"chr22" 39605001 39606000 "*" 0 0 100
"chr22" 39632001 39633000 "*" 4.08209022140227e-11 9.57770690255255e-11 100

"chr22" 39639001 39640000 "*" 0 0 71.3461538461538
"chr22" 39657001 39658000 "*" 4.44089209850063e-16 1.91071758245033e-15 100
"chr22" 39675001 39676000 "*" 0 0 100
"chr22" 39706001 39707000 "*" 7.2616323637531e-06 8.12679745947123e-06 76.4705882352941
"chr22" 39707001 39708000 "*" 0 0 -63.3333333333333
"chr22" 39732001 39733000 "*" 4.01313771103418e-09 7.11882087237587e-09 100
"chr22" 39745001 39746000 "*" 0 0 83.0525896414343
"chr22" 39746001 39747000 "*" 0 0 78.1369103503053
"chr22" 39748001 39749000 "*" 4.44089209850063e-16 1.91071758245033e-15 -62.8571428571429
"chr22" 39791001 39792000 "*" 6.61459775841422e-12 1.73315131393635e-11 86.5384615384615
"chr22" 39867001 39868000 "*" 1.41914275886279e-08 2.3288633896473e-08 52.3809523809524
"chr22" 39872001 39873000 "*" 1.11022302462516e-15 4.59817122935606e-15 66.7777777777778
"chr22" 39873001 39874000 "*" 3.12345482811338e-10 6.50254136486909e-10 -85.9550561797753
"chr22" 39920001 39921000 "*" 6.96331881044898e-12 1.81539744299358e-11 -78.2608695652174
"chr22" 39931001 39932000 "*" 0 0 -62.2769979839477
"chr22" 39936001 39937000 "*" 5.55111512312578e-16 2.36485094870365e-15 100
"chr22" 39952001 39953000 "*" 9.08108033215171e-11 2.03407753381119e-10 100
"chr22" 39967001 39968000 "*" 3.24185123190546e-14 1.13268811149135e-13 51.2820512820513
"chr22" 39976001 39977000 "*" 7.0006535457523e-08 1.03695832512054e-07 100
"chr22" 40012001 40013000 "*" 2.04988059859801e-09 3.80458088311340e-09 -66.6666666666667
"chr22" 40013001 40014000 "*" 1.11022302462516e-16 5.03662826618488e-16 -69.1275167785235
"chr22" 40014001 40015000 "*" 7.78377362564697e-13 2.29551678017433e-12 -100
"chr22" 40020001 40021000 "*" 0 0 90.2985074626866
"chr22" 40023001 40024000 "*" 0 0 60.2564102564103
"chr22" 40038001 40039000 "*" 8.7349629751543e-09 1.47505871898511e-08 100
"chr22" 40043001 40044000 "*" 6.15488325740321e-05 5.96864706387621e-05 55.7142857142857
"chr22" 40049001 40050000 "*" 8.7349629751543e-09 1.47505871898511e-08 -100
"chr22" 40059001 40060000 "*" 1.67150737695465e-11 4.14735013917257e-11 58.8785046728972
"chr22" 40062001 40063000 "*" 0 0 81.0344827586207
"chr22" 40070001 40071000 "*" 5.68123437183488e-10 1.14082514207793e-09 84.375
"chr22" 40073001 40074000 "*" 0 0 68.7931034482759
"chr22" 40091001 40092000 "*" 2.23421314782257e-09 4.11871828003941e-09 64.8648648648649
"chr22" 40096001 40097000 "*" 1.25717547483362e-10 2.76566987983258e-10 52.3809523809524
"chr22" 40110001 40111000 "*" 6.98872284599084e-06 7.84104914795963e-06 -58.8235294117647
"chr22" 40355001 40356000 "*" 2.88710388929303e-10 6.03399264731139e-10 -100
"chr22" 40384001 40385000 "*" 8.52184989241778e-12 2.19821916521953e-11 -76.1904761904762

"chr22" 40429001 40430000 "*" 2.79987144580218e-12 7.68634726811898e-12 100
"chr22" 41051001 41052000 "*" 1.54529942730797e-06 1.9118118543793e-06
75.5555555555556
"chr22" 41346001 41347000 "*" 0 0 55.6937799043062
"chr22" 41405001 41406000 "*" 8.7349629751543e-09 1.47505871898511e-08 100
"chr22" 41444001 41445000 "*" 0 0 100
"chr22" 41464001 41465000 "*" 5.95279381343516e-12 1.56856703724177e-11
-66.6666666666667
"chr22" 41495001 41496000 "*" 1.11022302462516e-16 5.03662826618488e-16 -100
"chr22" 41612001 41613000 "*" 6.9792616130826e-11 1.58832996661374e-10
56.5217391304348
"chr22" 41643001 41644000 "*" 1.43218770176645e-14 5.21983361092297e-14 100
"chr22" 41809001 41810000 "*" 1.11022302462516e-16 5.03662826618488e-16
-52.0408163265306
"chr22" 41840001 41841000 "*" 0 0 -76.0174719496543
"chr22" 41952001 41953000 "*" 0 0 -100
"chr22" 41973001 41974000 "*" 0 0 -81.3008130081301
"chr22" 42245001 42246000 "*" 4.04946220224911e-05 4.0376350196346e-05
64.7058823529412
"chr22" 42314001 42315000 "*" 0 0 -66.6666666666667
"chr22" 42329001 42330000 "*" 0 0 -100
"chr22" 42371001 42372000 "*" 1.56863411149288e-12 4.4469621057462e-12 -100
"chr22" 42430001 42431000 "*" 7.0006535457523e-08 1.03695832512054e-07 -100
"chr22" 42557001 42558000 "*" 1.28256072429167e-10 2.81876405375473e-10
-70.3703703703704
"chr22" 42607001 42608000 "*" 5.99520433297585e-15 2.28531569014092e-14 100
"chr22" 42679001 42680000 "*" 0 0 59.7691562634524
"chr22" 42680001 42681000 "*" 3.37740502232009e-09 6.07535874407838e-09
51.4285714285714
"chr22" 42699001 42700000 "*" 2.34923192010683e-13 7.38235039488955e-13 93.75
"chr22" 42715001 42716000 "*" 0 0 -100
"chr22" 42750001 42751000 "*" 1.45441247934031e-09 2.75535548368821e-09
-63.6363636363636
"chr22" 42751001 42752000 "*" 2.01809635669292e-08 3.22829990076203e-08
-52.3809523809524
"chr22" 42753001 42754000 "*" 3.3873015503616e-11 8.04875666391327e-11
-81.8181818181818
"chr22" 42768001 42769000 "*" 7.0006535457523e-08 1.03695832512054e-07 -100
"chr22" 42769001 42770000 "*" 1.67299207820548e-08 2.71377429441055e-08 100
"chr22" 42775001 42776000 "*" 9.65729496371637e-10 1.87255912493232e-09 100
"chr22" 42776001 42777000 "*" 3.73034936274053e-14 1.29420415488591e-13 100
"chr22" 42779001 42780000 "*" 5.1281201507436e-13 1.54461625452838e-12 100
"chr22" 42804001 42805000 "*" 1.48087875295744e-10 3.22075573380618e-10 -100
"chr22" 42821001 42822000 "*" 1.93720595120794e-11 4.76309924836636e-11 100
"chr22" 42916001 42917000 "*" 0 0 -80.3571428571429
"chr22" 42956001 42957000 "*" 0.000299166016444086 0.000259267996470385
54.1666666666667
"chr22" 42978001 42979000 "*" 0 0 -100
"chr22" 43017001 43018000 "*" 0 0 -100
"chr22" 43049001 43050000 "*" 5.08482145278322e-14 1.73432706170696e-13 100
"chr22" 43063001 43064000 "*" 9.2148511043888e-15 3.43705279328267e-14 -100
"chr22" 43067001 43068000 "*" 1.83186799063151e-14 6.59696304569048e-14 -100

"chr22" 43094001 43095000 "*" 2.82773804372027e-13 8.79129664109438e-13 -100
"chr22" 43097001 43098000 "*" 7.44056039003738e-10 1.46691232861555e-09
-69.2307692307692
"chr22" 43116001 43117000 "*" 0 0 71.3413570556428
"chr22" 43119001 43120000 "*" 3.90465437760668e-13 1.19307893943277e-12 100
"chr22" 43278001 43279000 "*" 1.46549439250521e-14 5.33330076973935e-14 -100
"chr22" 43306001 43307000 "*" 7.7715611723761e-16 3.26213507634405e-15
-72.5490196078431
"chr22" 43343001 43344000 "*" 0 0 -53.5313647567785
"chr22" 43368001 43369000 "*" 1.37001521238744e-13 4.42851918810123e-13 -100
"chr22" 43391001 43392000 "*" 0.000265000805704796 0.000231819965735711
66.0606060606061
"chr22" 43424001 43425000 "*" 2.57276422388486e-11 6.22881820165752e-11 87.5
"chr22" 43468001 43469000 "*" 0 0 100
"chr22" 43479001 43480000 "*" 0 0 66.6666666666667
"chr22" 43500001 43501000 "*" 0 0 100
"chr22" 43509001 43510000 "*" 8.82625959883754e-05 8.34561020110797e-05
61.1111111111111
"chr22" 43543001 43544000 "*" 2.64951482975562e-09 4.82186011375752e-09 100
"chr22" 43544001 43545000 "*" 1.9373391779709e-13 6.15971322898373e-13 100
"chr22" 43548001 43549000 "*" 1.9373391779709e-13 6.15971322898373e-13
-70.0854700854701
"chr22" 43603001 43604000 "*" 1.59872115546023e-13 5.13278895274981e-13
57.70887166236
"chr22" 43604001 43605000 "*" 2.22044604925031e-16 9.81641919380259e-16
51.4056224899598
"chr22" 43609001 43610000 "*" 1.22124532708767e-15 5.03921984217778e-15 100
"chr22" 43633001 43634000 "*" 4.02167188440217e-12 1.08266700497926e-11 100
"chr22" 43638001 43639000 "*" 1.15314159332414e-08 1.91244059393851e-08
-57.8881206788183
"chr22" 43639001 43640000 "*" 0 0 51.5151515151515
"chr22" 43658001 43659000 "*" 4.76831052420579e-09 8.36693501129188e-09
69.6969696969697
"chr22" 43666001 43667000 "*" 0 0 100
"chr22" 43669001 43670000 "*" 6.59550958292954e-09 1.13456194382773e-08 100
"chr22" 43677001 43678000 "*" 5.55111512312578e-16 2.36485094870365e-15 89.0625
"chr22" 43678001 43679000 "*" 5.55111512312578e-16 2.36485094870365e-15
51.7241379310345
"chr22" 43680001 43681000 "*" 7.54951656745106e-15 2.84375115626978e-14
58.6111111111111
"chr22" 43688001 43689000 "*" 0 0 100
"chr22" 43702001 43703000 "*" 2.97410207572568e-10 6.20710657827741e-10
-64.4736842105263
"chr22" 43705001 43706000 "*" 3.03090885722668e-14 1.06210046699299e-13 -100
"chr22" 43725001 43726000 "*" 1.08204889492924e-10 2.40335646734638e-10
67.6470588235294
"chr22" 43753001 43754000 "*" 1.93720595120794e-11 4.76309924836636e-11 100
"chr22" 43783001 43784000 "*" 8.56967979746548e-05 8.11800488313117e-05
57.1428571428571
"chr22" 43784001 43785000 "*" 6.15195683284497e-09 1.06542659963611e-08
-82.6530612244898
"chr22" 43788001 43789000 "*" 2.46023981997912e-06 2.95350280548749e-06

57.1428571428571
"chr22" 43797001 43798000 "*" 5.77315972805081e-15 2.205310974382e-14
65.1162790697674
"chr22" 43801001 43802000 "*" 2.06711314731933e-11 5.05167981954628e-11
-83.3333333333333
"chr22" 43802001 43803000 "*" 4.88498130835069e-15 1.8824139250584e-14
-76.4705882352941
"chr22" 43814001 43815000 "*" 0 0 98.7654320987654
"chr22" 43818001 43819000 "*" 1.11022302462516e-16 5.03662826618488e-16
78.1512605042017
"chr22" 43824001 43825000 "*" 3.6700841921089e-08 5.65530226072258e-08 -100
"chr22" 43830001 43831000 "*" 0 0 79.0186125211506
"chr22" 43847001 43848000 "*" 0 0 83.0508474576271
"chr22" 43883001 43884000 "*" 0 0 75.9259259259259
"chr22" 43884001 43885000 "*" 4.7483429336137e-05 4.68579300606429e-05
59.5238095238095
"chr22" 43898001 43899000 "*" 0 0 87.5
"chr22" 43900001 43901000 "*" 3.65130148338721e-12 9.90602415539447e-12 100
"chr22" 43906001 43907000 "*" 1.87405646556726e-13 5.97285292681429e-13 100
"chr22" 43923001 43924000 "*" 0 0 67.4083438685209
"chr22" 43959001 43960000 "*" 5.63660229602192e-13 1.68905058935478e-12 -100
"chr22" 43964001 43965000 "*" 0 0 -92.8571428571429
"chr22" 44013001 44014000 "*" 0 0 100
"chr22" 44054001 44055000 "*" 5.07371922253697e-14 1.73287898516782e-13 100
"chr22" 44080001 44081000 "*" 2.64951482975562e-09 4.82186011375752e-09 100
"chr22" 44086001 44087000 "*" 4.97894849793568e-08 7.55366770785768e-08
-64.2857142857143
"chr22" 44193001 44194000 "*" 5.794809077031e-12 1.52911192757929e-11 100
"chr22" 44224001 44225000 "*" 0 0 58.4415584415584
"chr22" 44227001 44228000 "*" 0 0 69.0384615384615
"chr22" 44232001 44233000 "*" 5.1052191873957e-07 6.73988631871006e-07
70.6666666666667
"chr22" 44275001 44276000 "*" 1.21307297540341e-10 2.67570631889524e-10
82.4742268041237
"chr22" 44285001 44286000 "*" 4.91828799908944e-14 1.68192819018269e-13 100
"chr22" 44289001 44290000 "*" 0 0 60.1990049751244
"chr22" 44291001 44292000 "*" 1.88224480446308e-09 3.51131877692111e-09
66.6666666666667
"chr22" 44297001 44298000 "*" 1.0551626239419e-10 2.34638843495876e-10
73.469387755102
"chr22" 44303001 44304000 "*" 0 0 100
"chr22" 44323001 44324000 "*" 2.88912860302304e-09 5.23531426675085e-09 -85
"chr22" 44325001 44326000 "*" 3.6700841921089e-08 5.65530226072258e-08 100
"chr22" 44340001 44341000 "*" 0 0 100
"chr22" 44348001 44349000 "*" 1.51056944730499e-12 4.29915863794587e-12
85.7142857142857
"chr22" 44391001 44392000 "*" 1.39610545346613e-12 3.99150416037401e-12
-58.8946459412781
"chr22" 44397001 44398000 "*" 2.44805414828519e-08 3.87506495191586e-08
-66.6666666666667
"chr22" 44419001 44420000 "*" 0 0 89.7683397683398
"chr22" 44420001 44421000 "*" 0 0 68.9565780946209

"chr22" 44421001 44422000 "*" 3.32279759263088e-11 7.9017888031321e-11 51.6935483870968
"chr22" 44427001 44428000 "*" 0 0 -100
"chr22" 44438001 44439000 "*" 1.09804983994266e-08 1.83170906849249e-08 -83.3333333333333
"chr22" 44481001 44482000 "*" 9.31108323776186e-09 1.56720652184097e-08 -63.3333333333333
"chr22" 44527001 44528000 "*" 0 0 -96.9230769230769
"chr22" 44532001 44533000 "*" 3.34128893442198e-08 5.19772864618523e-08 -100
"chr22" 44553001 44554000 "*" 2.09067207990188e-11 5.1073083948126e-11 -66.6666666666667
"chr22" 44568001 44569000 "*" 0 0 77.027027027027
"chr22" 44581001 44582000 "*" 5.6362137179633e-11 1.29963270300065e-10 100
"chr22" 44584001 44585000 "*" 2.39687045766601e-08 3.79822661439144e-08 52.1739130434783
"chr22" 44587001 44588000 "*" 1.98549366947631e-08 3.19077318271278e-08 76.9230769230769
"chr22" 44592001 44593000 "*" 3.5487279781421e-10 7.32971065982104e-10 75.8620689655172
"chr22" 44600001 44601000 "*" 0 0 -100
"chr22" 44601001 44602000 "*" 0 0 79.0476190476191
"chr22" 44615001 44616000 "*" 2.97883939737176e-12 8.15511035653874e-12 86.71875
"chr22" 44616001 44617000 "*" 4.03125379533353e-06 4.68388654489485e-06 57.1428571428571
"chr22" 44660001 44661000 "*" 1.90178608416858e-08 3.06345311263988e-08 67.1232876712329
"chr22" 44676001 44677000 "*" 1.19415964894287e-08 1.97700784133167e-08 -77.4647887323944
"chr22" 44678001 44679000 "*" 1.14130926931466e-13 3.7276674571693e-13 100
"chr22" 44679001 44680000 "*" 1.4363131384254e-07 2.03671717589617e-07 -79.7297297297297
"chr22" 44688001 44689000 "*" 1.11022302462516e-16 5.03662826618488e-16 -78.3132530120482
"chr22" 44708001 44709000 "*" 1.16573417585641e-14 4.29335582900508e-14 -70.3703703703704
"chr22" 44712001 44713000 "*" 3.33066907387547e-16 1.4495649018245e-15 92.2330097087379
"chr22" 44715001 44716000 "*" 0 0 100
"chr22" 44723001 44724000 "*" 2.40287107785564e-05 2.48476321258601e-05 -55.5555555555556
"chr22" 44736001 44737000 "*" 3.80662125865072e-06 4.43958699582065e-06 -66.6666666666667
"chr22" 44748001 44749000 "*" 2.88710388929303e-10 6.03399264731139e-10 100
"chr22" 44755001 44756000 "*" 0 0 -100
"chr22" 44787001 44788000 "*" 6.51502929382985e-09 1.12484832601884e-08 64.3888888888889
"chr22" 44788001 44789000 "*" 8.52140580320793e-12 2.19817259951489e-11 -100
"chr22" 44793001 44794000 "*" 1.17905685215192e-13 3.83653245040640e-13 100
"chr22" 44797001 44798000 "*" 0 0 100
"chr22" 44802001 44803000 "*" 0 0 100
"chr22" 44806001 44807000 "*" 5.57708323967177e-11 1.28799221678243e-10

-71.6981132075472
"chr22" 44824001 44825000 "*" 0 0 59.2061214729794
"chr22" 44837001 44838000 "*" 1.31682611037576e-07 1.8803584459342e-07
-74.2857142857143
"chr22" 44847001 44848000 "*" 1.37828859436695e-10 3.01445544267661e-10 100
"chr22" 44884001 44885000 "*" 3.10336973692316e-05 3.15338093902317e-05
-51.8518518518519
"chr22" 44922001 44923000 "*" 2.87647388130807e-08 4.51908085966587e-08 62.5
"chr22" 44941001 44942000 "*" 2.75002243199651e-13 8.56213041814349e-13
86.3636363636364
"chr22" 44968001 44969000 "*" 0 0 73.0259671436142
"chr22" 44972001 44973000 "*" 3.31491121774441e-07 4.48888447535355e-07
-55.5555555555556
"chr22" 44995001 44996000 "*" 0 0 -100
"chr22" 45017001 45018000 "*" 2.41584530158434e-13 7.58025515135821e-13
59.3011811023622
"chr22" 45019001 45020000 "*" 0 0 -50.3597122302158
"chr22" 45024001 45025000 "*" 1.17905685215192e-13 3.83653245040640e-13 -100
"chr22" 45060001 45061000 "*" 0 0 -100
"chr22" 45064001 45065000 "*" 0 0 72.7406007914104
"chr22" 45087001 45088000 "*" 0 0 74.5283018867925
"chr22" 45090001 45091000 "*" 1.91224813761437e-12 5.36527594469447e-12 -100
"chr22" 45152001 45153000 "*" 3.6700841921089e-08 5.65530226072258e-08 -100
"chr22" 45154001 45155000 "*" 1.11022302462516e-16 5.03662826618488e-16
76.4705882352941
"chr22" 45175001 45176000 "*" 1.68996264759969e-08 2.7398105072003e-08
-52.3809523809524
"chr22" 45178001 45179000 "*" 0 0 -100
"chr22" 45241001 45242000 "*" 1.28230759344206e-13 4.161621411106e-13 -53.125
"chr22" 45314001 45315000 "*" 4.57732074465866e-10 9.323525225507e-10
-77.0833333333333
"chr22" 45336001 45337000 "*" 1.89848137210902e-14 6.82212372863451e-14 100
"chr22" 45363001 45364000 "*" 1.18127729820117e-13 3.84315779147484e-13
64.7540983606557
"chr22" 45367001 45368000 "*" 9.96182603074658e-09 1.67100409101998e-08
53.2327586206897
"chr22" 45374001 45375000 "*" 1.88245228693873e-06 2.29966065245188e-06
-52.1739130434783
"chr22" 45379001 45380000 "*" 2.88779000712225e-11 6.92935809466068e-11 100
"chr22" 45383001 45384000 "*" 2.07546496655553e-08 3.31473076384131e-08 56
"chr22" 45385001 45386000 "*" 2.86992651865603e-12 7.87063470151354e-12
56.2208309618668
"chr22" 45386001 45387000 "*" 6.24654217240561e-10 1.2462108191303e-09 100
"chr22" 45390001 45391000 "*" 0 0 -74.4444444444444
"chr22" 45391001 45392000 "*" 3.46500605985511e-13 1.06777978272295e-12 100
"chr22" 45402001 45403000 "*" 0 0 93.8775510204082
"chr22" 45436001 45437000 "*" 0 0 100
"chr22" 45445001 45446000 "*" 1.11022302462516e-16 5.03662826618488e-16
-56.6686080372743
"chr22" 45456001 45457000 "*" 0 0 100
"chr22" 45466001 45467000 "*" 1.97471896823398e-06 2.40413608358628e-06
-72.2222222222222

"chr22" 45487001 45488000 "*" 0 0 55.3411020796172
"chr22" 45492001 45493000 "*" 1.67299207820548e-08 2.71377429441055e-08 -100
"chr22" 45628001 45629000 "*" 5.63660229602192e-13 1.68905058935478e-12 -100
"chr22" 45668001 45669000 "*" 0 0 -85.2941176470588
"chr22" 45706001 45707000 "*" 0 0 77.8513565642279
"chr22" 45761001 45762000 "*" 0 0 100
"chr22" 45898001 45899000 "*" 0 0 65.8129118643178
"chr22" 45936001 45937000 "*" 1.11022302462516e-15 4.59817122935606e-15 -100
"chr22" 45944001 45945000 "*" 4.36317648677687e-14 1.50213399380385e-13
-95.8801498127341
"chr22" 45973001 45974000 "*" 2.06501482580279e-14 7.3890370570066e-14 -53.125
"chr22" 45974001 45975000 "*" 7.21644966006352e-15 2.72345776413425e-14 -100
"chr22" 45977001 45978000 "*" 0 0 70
"chr22" 45990001 45991000 "*" 2.88710388929303e-10 6.03399264731139e-10 -100
"chr22" 46159001 46160000 "*" 1.12634901405784e-10 2.49563754426169e-10 100
"chr22" 46260001 46261000 "*" 6.92287338566189e-11 1.57605630124247e-10 100
"chr22" 46293001 46294000 "*" 7.0006535457523e-08 1.03695832512054e-07 100
"chr22" 46298001 46299000 "*" 0 0 100
"chr22" 46331001 46332000 "*" 6.01700157343288e-05 5.84315834391449e-05
58.1395348837209
"chr22" 46390001 46391000 "*" 1.10280834797472e-05 1.20060810167597e-05
62.8571428571429
"chr22" 46397001 46398000 "*" 0 0 51.575456053068
"chr22" 46403001 46404000 "*" 0 0 -51.4448191247535
"chr22" 46426001 46427000 "*" 1.17461596005342e-13 3.8279067575877e-13 100
"chr22" 46433001 46434000 "*" 0 0 -81.7610062893082
"chr22" 46434001 46435000 "*" 4.01313771103418e-09 7.11882087237587e-09 100
"chr22" 46450001 46451000 "*" 0 0 -86
"chr22" 46465001 46466000 "*" 0 0 -88.0952380952381
"chr22" 46470001 46471000 "*" 6.97407565031227e-09 1.19637733705378e-08
-51.8518518518519
"chr22" 46474001 46475000 "*" 0 0 -83.248730964467
"chr22" 46475001 46476000 "*" 0 0 -62.1719007237598
"chr22" 46477001 46478000 "*" 0 0 -95.8904109589041
"chr22" 46479001 46480000 "*" 2.56461518688411e-13 8.02862858170504e-13
-61.3333333333333
"chr22" 46489001 46490000 "*" 4.38871161634324e-13 1.33210178550071e-12 -87.5
"chr22" 46490001 46491000 "*" 2.79776202205539e-14 9.84620232065018e-14
-79.3650793650794
"chr22" 46502001 46503000 "*" 2.62012633811537e-14 9.25110548865337e-14
51.5360321561872
"chr22" 46547001 46548000 "*" 0 0 73.5328490718321
"chr22" 46558001 46559000 "*" 0 0 69.7478991596639
"chr22" 46622001 46623000 "*" 1.6899627253153e-08 2.7398105072003e-08
52.3809523809524
"chr22" 46726001 46727000 "*" 0.000147580450223783 0.000134596509548505
-51.8518518518519
"chr22" 46730001 46731000 "*" 1.11022302462516e-16 5.03662826618488e-16 -100
"chr22" 46779001 46780000 "*" 2.64951482975562e-09 4.82186011375752e-09 100
"chr22" 46783001 46784000 "*" 0 0 100
"chr22" 46809001 46810000 "*" 2.00227675550835e-08 3.20459176617961e-08 100
"chr22" 46813001 46814000 "*" 2.0209418805095e-08 3.23241780046683e-08

-81.3333333333333
"chr22" 46852001 46853000 "*" 2.71065789814173e-08 4.27015900327592e-08
57.1182548794489
"chr22" 46857001 46858000 "*" 0 0 82.6666666666667
"chr22" 46863001 46864000 "*" 0.00026590838562468 0.000232535857460809
-53.5714285714286
"chr22" 46867001 46868000 "*" 0 0 -100
"chr22" 46947001 46948000 "*" 0 0 100
"chr22" 46959001 46960000 "*" 3.88578058618805e-15 1.5152981210988e-14
-72.9166666666667
"chr22" 46993001 46994000 "*" 4.21884749357559e-15 1.63874445239189e-14
79.1666666666667
"chr22" 47019001 47020000 "*" 5.63660229602192e-13 1.68905058935478e-12 -100
"chr22" 47029001 47030000 "*" 4.66071625737641e-13 1.41214482395985e-12
55.3191489361702
"chr22" 47081001 47082000 "*" 0 0 -59.4594594594595
"chr22" 47117001 47118000 "*" 2.66453525910038e-15 1.05861327033776e-14 100
"chr22" 47133001 47134000 "*" 0 0 86.7803837953092
"chr22" 47134001 47135000 "*" 0 0 69.726368159204
"chr22" 47136001 47137000 "*" 0 0 -63.1578947368421
"chr22" 47146001 47147000 "*" 5.55950319114373e-09 9.68069235315351e-09
-52.027027027027
"chr22" 47194001 47195000 "*" 0 0 67.1428571428571
"chr22" 47195001 47196000 "*" 0 0 100
"chr22" 47226001 47227000 "*" 0 0 -100
"chr22" 47525001 47526000 "*" 3.33066907387547e-16 1.4495649018245e-15
-96.7741935483871
"chr22" 47526001 47527000 "*" 0 0 75.8620689655172
"chr22" 47586001 47587000 "*" 0 0 61.4583333333333
"chr22" 47604001 47605000 "*" 0 0 96.4601769911504
"chr22" 47621001 47622000 "*" 9.67004254448511e-14 3.18178990782609e-13 100
"chr22" 47672001 47673000 "*" 3.06218383983037e-10 6.38199431469613e-10
81.6666666666667
"chr22" 47675001 47676000 "*" 6.93112234273485e-13 2.05218842007357e-12 -100
"chr22" 47681001 47682000 "*" 1.34559030584569e-13 4.35581312463895e-13 100
"chr22" 47682001 47683000 "*" 8.68346339277792e-09 1.47166612287853e-08
-53.0054644808743
"chr22" 47686001 47687000 "*" 4.79616346638068e-14 1.64380445416596e-13 100
"chr22" 47689001 47690000 "*" 1.4432899320127e-15 5.90750815956055e-15 100
"chr22" 47692001 47693000 "*" 0 0 74.2068965517241
"chr22" 47728001 47729000 "*" 9.75745070297407e-08 1.41765802248358e-07
59.2592592592593
"chr22" 47738001 47739000 "*" 3.33066907387547e-16 1.4495649018245e-15 100
"chr22" 47739001 47740000 "*" 2.22044604925031e-16 9.81641919380259e-16
85.7142857142857
"chr22" 47766001 47767000 "*" 0 0 75
"chr22" 47767001 47768000 "*" 1.12458486967171e-09 2.15740815043925e-09 -100
"chr22" 47770001 47771000 "*" 0 0 58.6021505376344
"chr22" 47793001 47794000 "*" 1.14124265593318e-11 2.89138062669327e-11 100
"chr22" 47803001 47804000 "*" 1.14124265593318e-11 2.89138062669327e-11 100
"chr22" 47816001 47817000 "*" 6.4324989779152e-11 1.47230020825092e-10 100
"chr22" 47840001 47841000 "*" 1.22124532708767e-15 5.03921984217778e-15

78.7037037037037
"chr22" 47842001 47843000 "*" 0 0 100
"chr22" 47945001 47946000 "*" 7.88830466502688e-08 1.15989087971183e-07
-72.7272727272727
"chr22" 47949001 47950000 "*" 0.000161489563003547 0.000146410937143468
-53.6585365853659
"chr22" 47965001 47966000 "*" 1.10578213252666e-13 3.62070592239648e-13
73.6111111111111
"chr22" 47966001 47967000 "*" 4.08209022140227e-11 9.57770690255255e-11 -100
"chr22" 47971001 47972000 "*" 8.99893899175552e-06 9.92945404379429e-06 60
"chr22" 47988001 47989000 "*" 3.33066907387547e-16 1.4495649018245e-15 -100
"chr22" 47992001 47993000 "*" 2.68450373042128e-10 5.64465213127486e-10 100
"chr22" 47997001 47998000 "*" 0 0 75.3623188405797
"chr22" 48001001 48002000 "*" 6.12430883784043e-10 1.22528336979697e-09
68.1818181818182
"chr22" 48009001 48010000 "*" 3.32900373933853e-12 9.0649481544848e-12
-58.3333333333333
"chr22" 48022001 48023000 "*" 9.29399944338272e-07 1.18499888988659e-06
58.6206896551724
"chr22" 48179001 48180000 "*" 0.000470021944885546 0.00039420914918048
55.3465346534653
"chr22" 48198001 48199000 "*" 2.04794026181787e-09 3.80188414441656e-09
79.7872340425532
"chr22" 48202001 48203000 "*" 3.75377506856012e-12 1.01651879013373e-11
-69.7674418604651
"chr22" 48211001 48212000 "*" 2.15125472990962e-10 4.58126659943874e-10 100
"chr22" 48214001 48215000 "*" 9.65729496371637e-10 1.87255912493232e-09 -100
"chr22" 48228001 48229000 "*" 8.73203944706802e-07 1.11735455122568e-06
-71.9483568075117
"chr22" 48239001 48240000 "*" 2.25552909682847e-12 6.27795683857045e-12
50.8500303582271
"chr22" 48284001 48285000 "*" 1.11022302462516e-16 5.03662826618488e-16 100
"chr22" 48286001 48287000 "*" 5.30115611407922e-08 8.01261580085136e-08
-71.2121212121212
"chr22" 48310001 48311000 "*" 2.1094237467878e-14 7.54243990081593e-14 -90
"chr22" 48314001 48315000 "*" 1.33761890452888e-11 3.35917943142279e-11
68.2291666666667
"chr22" 48390001 48391000 "*" 0 0 -100
"chr22" 48463001 48464000 "*" 8.97002472299846e-11 2.01499262998218e-10
-66.6666666666667
"chr22" 48464001 48465000 "*" 6.60582699651968e-14 2.22176301799e-13 -100
"chr22" 48475001 48476000 "*" 9.67004254448511e-14 3.18178990782609e-13 100
"chr22" 48486001 48487000 "*" 1.16573417585641e-14 4.29335582900508e-14
71.7171717171717
"chr22" 48492001 48493000 "*" 9.67004254448511e-14 3.18178990782609e-13 -100
"chr22" 48500001 48501000 "*" 6.18061157808825e-12 1.62638768634927e-11
-67.3076923076923
"chr22" 48502001 48503000 "*" 6.43996536897617e-05 6.22599140150143e-05
-52.3809523809524
"chr22" 48510001 48511000 "*" 1.78688104160996e-05 1.88505925150618e-05 -65.625
"chr22" 48542001 48543000 "*" 1.11022302462516e-16 5.03662826618488e-16 -100
"chr22" 48569001 48570000 "*" 1.11022302462516e-16 5.03662826618488e-16 100

"chr22" 48573001 48574000 "*" 1.10102207906415e-07 1.58898767538761e-07 67.3469387755102
"chr22" 48579001 48580000 "*" 1.14124265593318e-11 2.89138062669327e-11 -100
"chr22" 48622001 48623000 "*" 5.6576965334898e-13 1.69506711979052e-12 -75.8620689655172
"chr22" 48649001 48650000 "*" 1.62092561595273e-14 5.87307003677525e-14 81.8181818181818
"chr22" 48663001 48664000 "*" 2.02386996051018e-11 4.95035238532956e-11 -76.9230769230769
"chr22" 48704001 48705000 "*" 8.25450818808804e-13 2.42473843346228e-12 -100
"chr22" 48705001 48706000 "*" 1.24822073788167e-08 2.0617618535879e-08 58.7155963302752
"chr22" 48721001 48722000 "*" 5.71156365758441e-07 7.50013209833307e-07 -68.2926829268293
"chr22" 48727001 48728000 "*" 1.04360964314765e-14 3.87023752069057e-14 -60.7843137254902
"chr22" 48736001 48737000 "*" 5.82568437934583e-11 1.34128240272449e-10 -78.7610619469027
"chr22" 48748001 48749000 "*" 1.65027954746044e-07 2.32243498393663e-07 80.7017543859649
"chr22" 48759001 48760000 "*" 1.39779633423487e-07 1.98463037958589e-07 100
"chr22" 48785001 48786000 "*" 1.78972144881939e-08 2.89243321796634e-08 -79.1666666666667
"chr22" 48787001 48788000 "*" 1.11022302462516e-16 5.03662826618488e-16 99.3548387096774
"chr22" 48798001 48799000 "*" 8.43542171335976e-07 1.08157311768213e-06 -58.0645161290323
"chr22" 48830001 48831000 "*" 1.90817864564874e-06 2.32887155826907e-06 -75
"chr22" 48848001 48849000 "*" 1.31881827680758e-09 2.5107942443474e-09 83.974358974359
"chr22" 48856001 48857000 "*" 4.59370052219299e-06 5.2929254827923e-06 -65.625
"chr22" 48895001 48896000 "*" 0 0 -94.7867298578199
"chr22" 48899001 48900000 "*" 1.26565424807268e-14 4.63923203146481e-14 -95.8333333333333
"chr22" 48914001 48915000 "*" 8.1028184162335e-11 1.83006752981946e-10 56.4102564102564
"chr22" 48924001 48925000 "*" 0 0 90.7142857142857
"chr22" 48933001 48934000 "*" 0 0 -73.2450704225352
"chr22" 48935001 48936000 "*" 6.27164986610751e-13 1.86907261026719e-12 56.25
"chr22" 48936001 48937000 "*" 9.56235091109647e-13 2.78624438617287e-12 -65.2173913043478
"chr22" 48940001 48941000 "*" 1.07864652044709e-05 1.17574627643031e-05 63.8888888888889
"chr22" 48963001 48964000 "*" 2.13768114321056e-10 4.56658633853484e-10 61.2612612612613
"chr22" 48982001 48983000 "*" 0 0 100
"chr22" 49002001 49003000 "*" 0 0 70.5882352941177
"chr22" 49007001 49008000 "*" 0 0 73.8738738738739
"chr22" 49024001 49025000 "*" 0 0 54.3587662337662
"chr22" 49031001 49032000 "*" 3.84137166520304e-14 1.32929369817675e-13 100
"chr22" 49038001 49039000 "*" 0 0 55.1112579863406
"chr22" 49048001 49049000 "*" 2.88710388929303e-10 6.03399264731139e-10 100

"chr22" 49050001 49051000 "*" 4.48248224387271e-05 4.43987771277389e-05
-52.112676056338
"chr22" 49057001 49058000 "*" 2.27950991416037e-12 6.34110655382771e-12
-54.7224224945926
"chr22" 49066001 49067000 "*" 2.00227675550835e-08 3.20459176617961e-08 -100
"chr22" 49080001 49081000 "*" 6.80491588855858e-07 8.84203296553687e-07
-63.6363636363636
"chr22" 49090001 49091000 "*" 0 0 100
"chr22" 49097001 49098000 "*" 0 0 -53.8336703867376
"chr22" 49101001 49102000 "*" 1.12458486967171e-09 2.15740815043925e-09 -100
"chr22" 49104001 49105000 "*" 0 0 -100
"chr22" 49108001 49109000 "*" 2.54111226405396e-06 3.04366700991447e-06
-77.2727272727273
"chr22" 49115001 49116000 "*" 0 0 -100
"chr22" 49151001 49152000 "*" 3.68560737484813e-12 9.99551976518564e-12
-53.0427850119586
"chr22" 49154001 49155000 "*" 1.1953414396304e-05 1.29468849991476e-05 -56
"chr22" 49179001 49180000 "*" 8.52140580320793e-12 2.19817259951489e-11 100
"chr22" 49181001 49182000 "*" 4.8193382617967e-09 8.45256077486903e-09
-53.5714285714286
"chr22" 49196001 49197000 "*" 2.77832201689421e-11 6.69790410797025e-11
58.3892617449664
"chr22" 49199001 49200000 "*" 3.32867844399232e-10 6.90341926237044e-10
-66.1971830985916
"chr22" 49202001 49203000 "*" 3.00837132982679e-12 8.23244629232561e-12
-65.5172413793103
"chr22" 49231001 49232000 "*" 0 0 -56.9107981220657
"chr22" 49254001 49255000 "*" 8.43347613965761e-12 2.17859001990012e-11 -100
"chr22" 49260001 49261000 "*" 0 0 -100
"chr22" 49266001 49267000 "*" 7.55107087968554e-12 1.96127242053365e-11
-57.6923076923077
"chr22" 49267001 49268000 "*" 6.92897336951326e-07 8.99090158231749e-07
-77.5510204081633
"chr22" 49291001 49292000 "*" 0 0 -76.0869565217391
"chr22" 49292001 49293000 "*" 0 0 -76.219512195122
"chr22" 49293001 49294000 "*" 0 0 -84.1772151898734
"chr22" 49337001 49338000 "*" 1.1330425486733e-10 2.5092017593116e-10
67.6923076923077
"chr22" 49342001 49343000 "*" 2.22044604925031e-16 9.81641919380259e-16
-66.2337662337662
"chr22" 49352001 49353000 "*" 0 0 -100
"chr22" 49353001 49354000 "*" 0 0 -59.8634095087996
"chr22" 49400001 49401000 "*" 0 0 -53.6723163841808
"chr22" 49410001 49411000 "*" 1.15093024000146e-09 2.20571681096608e-09
-63.1336405529954
"chr22" 49411001 49412000 "*" 7.061018436616e-14 2.36703186996926e-13
-50.8196721311475
"chr22" 49413001 49414000 "*" 2.22044604925031e-16 9.81641919380259e-16
73.5849056603774
"chr22" 49430001 49431000 "*" 2.54481447026222e-09 4.65029142152584e-09
-50.5376344086022
"chr22" 49442001 49443000 "*" 0 0 65.3846153846154

"chr22" 49471001 49472000 "*" 6.93112234273485e-13 2.05218842007357e-12 100
"chr22" 49472001 49473000 "*" 1.05708464204213e-09 2.03804142360432e-09 55.3191489361702
"chr22" 49476001 49477000 "*" 1.00725389140521e-08 1.68835269251214e-08 76
"chr22" 49481001 49482000 "*" 3.00774120298719e-05 3.06285363410996e-05 -53.6585365853659
"chr22" 49489001 49490000 "*" 2.23896678974711e-10 4.75967044139767e-10 -87.4015748031496
"chr22" 49492001 49493000 "*" 0 0 100
"chr22" 49495001 49496000 "*" 2.02327044007689e-12 5.65300384792621e-12 -100
"chr22" 49515001 49516000 "*" 0 0 -56.25
"chr22" 49547001 49548000 "*" 0 0 -52.547065337763
"chr22" 49550001 49551000 "*" 1.33226762955019e-15 5.48133041560118e-15 -62.3931623931624
"chr22" 49555001 49556000 "*" 1.89581683684992e-12 5.32311367606535e-12 -100
"chr22" 49562001 49563000 "*" 1.88737914186277e-15 7.62011598380321e-15 -56.6473988439306
"chr22" 49575001 49576000 "*" 0 0 61.9047619047619
"chr22" 49584001 49585000 "*" 1.38590361409285e-10 3.02706178892116e-10 100
"chr22" 49587001 49588000 "*" 7.62453534464136e-06 8.5034977509117e-06 -66.6666666666667
"chr22" 49591001 49592000 "*" 2.97681182126475e-06 3.52994626402082e-06 -64.4444444444444
"chr22" 49604001 49605000 "*" 2.22158402785055e-10 4.72404212792847e-10 65.7142857142857
"chr22" 49610001 49611000 "*" 1.11022302462516e-16 5.03662826618488e-16 -75
"chr22" 49632001 49633000 "*" 0 0 -100
"chr22" 49634001 49635000 "*" 4.22564653057567e-06 4.8931850353025e-06 -52.3809523809524
"chr22" 49635001 49636000 "*" 5.97396778712067e-08 8.97070718617899e-08 -77.3584905660377
"chr22" 49658001 49659000 "*" 2.22044604925031e-16 9.81641919380259e-16 81.9444444444444
"chr22" 49671001 49672000 "*" 4.30186268829402e-08 6.57958786098507e-08 52
"chr22" 49673001 49674000 "*" 4.73446715165693e-09 8.30964813814393e-09 -51.1111111111111
"chr22" 49680001 49681000 "*" 1.35003119794419e-13 4.3679847476192e-13 -100
"chr22" 49687001 49688000 "*" 1.04694031222152e-13 3.43437506784719e-13 -61.641852770885
"chr22" 49704001 49705000 "*" 0 0 60.6060606060606
"chr22" 49706001 49707000 "*" 1.37828859436695e-10 3.01445544267661e-10 -100
"chr22" 49720001 49721000 "*" 9.94681337296299e-10 1.92368789521523e-09 -56.7555147058823
"chr22" 49727001 49728000 "*" 0 0 -100
"chr22" 49751001 49752000 "*" 9.10382880192628e-15 3.39898678115074e-14 -93.3333333333333
"chr22" 49777001 49778000 "*" 2.44249065417534e-15 9.75180348030994e-15 -80.7692307692308
"chr22" 49794001 49795000 "*" 1.13879055207988e-06 1.43481170115068e-06 64.5833333333333
"chr22" 49795001 49796000 "*" 6.66133814775094e-16 2.81595744474255e-15 73.5364875701684

"chr22" 49821001 49822000 "*" 2.64951482975562e-09 4.82186011375752e-09 100
"chr22" 49829001 49830000 "*" 0 0 60.6432748538012
"chr22" 49859001 49860000 "*" 2.11275441586167e-13 6.68101883706181e-13
93.3333333333333
"chr22" 49907001 49908000 "*" 0 0 100
"chr22" 49916001 49917000 "*" 4.02167188440217e-12 1.08266700497926e-11 -100
"chr22" 49925001 49926000 "*" 1.11022302462516e-15 4.59817122935606e-15
-51.7241379310345
"chr22" 49942001 49943000 "*" 4.16249257284562e-10 8.5239468094019e-10
83.3333333333333
"chr22" 49946001 49947000 "*" 2.87370127693976e-12 7.88046852923037e-12
-95.2380952380952
"chr22" 49967001 49968000 "*" 2.22044604925031e-15 8.90449334476539e-15
-67.515923566879
"chr22" 49978001 49979000 "*" 0 0 -92.5
"chr22" 49982001 49983000 "*" 6.21724893790088e-15 2.36606385879553e-14
88.8235294117647
"chr22" 50007001 50008000 "*" 4.14335232790108e-13 1.26153063645578e-12 -100
"chr22" 50011001 50012000 "*" 0 0 -100
"chr22" 50013001 50014000 "*" 0 0 -58.2118758434548
"chr22" 50020001 50021000 "*" 0 0 -100
"chr22" 50021001 50022000 "*" 0 0 -54.8204527712724
"chr22" 50032001 50033000 "*" 4.88498130835069e-15 1.8824139250584e-14
-54.2512077294686
"chr22" 50044001 50045000 "*" 3.11972669919669e-13 9.65803196243413e-13
55.4347826086957
"chr22" 50066001 50067000 "*" 0 0 100
"chr22" 50071001 50072000 "*" 7.10838164136307e-05 6.82135523203633e-05
55.2910052910053
"chr22" 50083001 50084000 "*" 0 0 70.6422018348624
"chr22" 50099001 50100000 "*" 0 0 -75.2112676056338
"chr22" 50107001 50108000 "*" 5.43095219185386e-06 6.19387126034938e-06
57.8947368421053
"chr22" 50121001 50122000 "*" 4.60568376881021e-07 6.1163705722328e-07
77.4193548387097
"chr22" 50129001 50130000 "*" 2.79674420313736e-05 2.86207364427666e-05
56.0283687943262
"chr22" 50132001 50133000 "*" 1.88737914186277e-15 7.62011598380321e-15 -100
"chr22" 50147001 50148000 "*" 0 0 -100
"chr22" 50154001 50155000 "*" 2.03994621195136e-08 3.26068834654036e-08
60.3009259259259
"chr22" 50246001 50247000 "*" 0 0 -100
"chr22" 50257001 50258000 "*" 0 0 98.8888888888889
"chr22" 50312001 50313000 "*" 0 0 63.4710539336962
"chr22" 50359001 50360000 "*" 4.03584943242663e-10 8.27659721474221e-10
-70.4545454545455
"chr22" 50428001 50429000 "*" 0 0 85.9375
"chr22" 50431001 50432000 "*" 3.34876072205503e-07 4.53221975892337e-07
-69.7247706422018
"chr22" 50498001 50499000 "*" 0 0 -100
"chr22" 50530001 50531000 "*" 0 0 100
"chr22" 50592001 50593000 "*" 0 0 100

"chr22" 50601001 50602000 "*" 3.41264554393206e-08 5.30042678128133e-08
51.3085818624467
"chr22" 50603001 50604000 "*" 0 0 100
"chr22" 50638001 50639000 "*" 0 0 -58.2089552238806
"chr22" 50640001 50641000 "*" 0 0 66.5226781857451
"chr22" 50700001 50701000 "*" 0 0 -69.4444444444444
"chr22" 50709001 50710000 "*" 0 0 73.2637069294264
"chr22" 50712001 50713000 "*" 2.00227675550835e-08 3.20459176617961e-08 100
"chr22" 50741001 50742000 "*" 5.794809077031e-12 1.52911192757929e-11 100
"chr22" 50742001 50743000 "*" 2.22337681599072e-09 4.10073588712469e-09
83.6734693877551
"chr22" 50743001 50744000 "*" 0 0 -91.7695473251029
"chr22" 50805001 50806000 "*" 3.50992568343145e-11 8.32708221852483e-11
-53.8461538461538
"chr22" 50852001 50853000 "*" 1.39779633423487e-07 1.98463037958589e-07 -100
"chr22" 51015001 51016000 "*" 0 0 64.7058823529412
"chr22" 51020001 51021000 "*" 0 0 -61.9043943603993
"chr22" 51040001 51041000 "*" 2.4535928844216e-14 8.69144396197119e-14 -100
"chr22" 51058001 51059000 "*" 4.44089209850063e-15 1.71914916534614e-14 100
"chr22" 51104001 51105000 "*" 0.000299166016444086 0.000259267996470385
-54.1666666666667
"chr22" 51162001 51163000 "*" 1.46624214547586e-07 2.07629778995107e-07
53.5714285714286
"chr22" 51173001 51174000 "*" 0 0 73.3333333333333
"chr22" 51178001 51179000 "*" 0 0 70.6701722980533
"chr3" 253001 254000 "*" 7.03992419914812e-12 1.83313429961219e-11 -100
"chr3" 578001 579000 "*" 1.11022302462516e-16 5.03662826618488e-16 97.5
"chr3" 661001 662000 "*" 9.41469124882133e-14 3.10868561210786e-13 100
"chr3" 834001 835000 "*" 0 0 100
"chr3" 1429001 1430000 "*" 1.73292091261157e-05 1.83235260735554e-05
57.1428571428571
"chr3" 1529001 1530000 "*" 3.33066907387547e-16 1.4495649018245e-15 100
"chr3" 1642001 1643000 "*" 2.22044604925031e-16 9.81641919380259e-16
-71.1111111111111
"chr3" 2393001 2394000 "*" 7.70959959206508e-08 1.13505504048778e-07
-55.8558558558559
"chr3" 3106001 3107000 "*" 3.72545775839672e-06 4.3519872100983e-06
73.6842105263158
"chr3" 3689001 3690000 "*" 0 0 -100
"chr3" 3829001 3830000 "*" 2.00227675550835e-08 3.20459176617961e-08 -100
"chr3" 3840001 3841000 "*" 0 0 67.7497581776815
"chr3" 4114001 4115000 "*" 1.52794177310511e-09 2.87642564232045e-09 -100
"chr3" 4521001 4522000 "*" 1.35036426485158e-12 3.86629547363774e-12 -100
"chr3" 4669001 4670000 "*" 1.26565424807268e-14 4.63923203146481e-14 100
"chr3" 4735001 4736000 "*" 1.11022302462516e-16 5.03662826618488e-16
51.2763596004439
"chr3" 4910001 4911000 "*" 0 0 52.4590163934426
"chr3" 5022001 5023000 "*" 1.29037891483108e-11 3.24582280286866e-11 -100
"chr3" 5146001 5147000 "*" 0 0 75
"chr3" 5150001 5151000 "*" 1.11022302462516e-16 5.03662826618488e-16 100
"chr3" 6683001 6684000 "*" 5.51780843238703e-14 1.87243792572872e-13 100
"chr3" 7012001 7013000 "*" 3.2700897545368e-10 6.78798757234889e-10

66.6666666666667
"chr3" 8090001 8091000 "*" 4.01313771103418e-09 7.11882087237587e-09 -100
"chr3" 8180001 8181000 "*" 7.0006535457523e-08 1.03695832512054e-07 100
"chr3" 8454001 8455000 "*" 5.62883073484954e-13 1.68905058935478e-12 100
"chr3" 8557001 8558000 "*" 6.66133814775094e-16 2.81595744474255e-15 -100
"chr3" 8649001 8650000 "*" 0 0 100
"chr3" 8666001 8667000 "*" 0.000315653397011784 0.000272553948292613
-57.1428571428571
"chr3" 8667001 8668000 "*" 1.89451579669653e-05 1.98897202749988e-05
-60.8695652173913
"chr3" 8678001 8679000 "*" 0.000115274736804838 0.000107060060050355
-54.1666666666667
"chr3" 8683001 8684000 "*" 0.000296795293734387 0.000257472949048318
-53.8461538461538
"chr3" 8691001 8692000 "*" 1.85147340836522e-06 2.26387187248592e-06
-62.7450980392157
"chr3" 8757001 8758000 "*" 4.35448918034709e-08 6.65605059543182e-08
76.4705882352941
"chr3" 8775001 8776000 "*" 2.1094237467878e-15 8.47434540879246e-15
63.3333333333333
"chr3" 8906001 8907000 "*" 1.02301417337358e-08 1.71328932446907e-08 90
"chr3" 9029001 9030000 "*" 9.0072393987839e-13 2.63157725646847e-12 100
"chr3" 9035001 9036000 "*" 6.59550958292954e-09 1.13456194382773e-08 -100
"chr3" 9089001 9090000 "*" 2.98469027271153e-11 7.14706850563657e-11
92.4528301886792
"chr3" 9100001 9101000 "*" 6.60582699651968e-14 2.22176301799e-13 100
"chr3" 9166001 9167000 "*" 0 0 -100
"chr3" 9209001 9210000 "*" 1.35890187991095e-11 3.40841971363279e-11 -100
"chr3" 9235001 9236000 "*" 2.99103253276201e-09 5.4130326770981e-09
-82.6666666666667
"chr3" 9346001 9347000 "*" 2.33028818463765e-10 4.93719283454501e-10 100
"chr3" 9536001 9537000 "*" 0 0 -61.7647058823529
"chr3" 9543001 9544000 "*" 0 0 100
"chr3" 9547001 9548000 "*" 0 0 82.3943661971831
"chr3" 9629001 9630000 "*" 2.15125472990962e-10 4.58126659943874e-10 -100
"chr3" 9642001 9643000 "*" 0 0 54.7410151426529
"chr3" 9745001 9746000 "*" 0 0 62.849208982289
"chr3" 9754001 9755000 "*" 1.98294731001969e-06 2.41330824932622e-06
56.5217391304348
"chr3" 9792001 9793000 "*" 0 0 62.5
"chr3" 9809001 9810000 "*" 5.97521117271516e-05 5.80588790261978e-05
65.6521739130435
"chr3" 9810001 9811000 "*" 3.95353005888666e-09 7.03928529283733e-09 -100
"chr3" 9823001 9824000 "*" 7.88258347483861e-15 2.96145301916012e-14 100
"chr3" 9851001 9852000 "*" 0 0 -82.9276781363837
"chr3" 9944001 9945000 "*" 0 0 56.1904761904762
"chr3" 10054001 10055000 "*" 2.07935805240922e-08 3.32075734485725e-08
78.0487804878049
"chr3" 10057001 10058000 "*" 5.01025332333427e-10 1.01358031135889e-09 -100
"chr3" 10061001 10062000 "*" 1.96509475358653e-13 6.23868565133654e-13 -100
"chr3" 10157001 10158000 "*" 0 0 -100
"chr3" 10206001 10207000 "*" 0 0 -92.5826149425287

"chr3" 10207001 10208000 "*" 0 0 -56.7567567567568
"chr3" 10240001 10241000 "*" 5.72875080706581e-14 1.93967931988877e-13 100
"chr3" 10289001 10290000 "*" 0 0 78.0821917808219
"chr3" 10385001 10386000 "*" 5.45774536675481e-12 1.44570356509603e-11
-76.3636363636364
"chr3" 10396001 10397000 "*" 2.08995487582797e-10 4.46944006972663e-10 -100
"chr3" 10449001 10450000 "*" 5.76576553168451e-09 1.00214420049533e-08
66.6666666666667
"chr3" 10452001 10453000 "*" 2.39995221849032e-05 2.48205262414204e-05
57.6388888888889
"chr3" 10468001 10469000 "*" 9.02738661601177e-10 1.76353962238896e-09 -60
"chr3" 10509001 10510000 "*" 2.68450373042128e-10 5.64465213127486e-10 100
"chr3" 10525001 10526000 "*" 5.03264097062583e-13 1.51942428521737e-12 100
"chr3" 10542001 10543000 "*" 9.41010101354056e-05 8.86297231089954e-05
-59.0909090909091
"chr3" 10546001 10547000 "*" 1.67643676718399e-14 6.06392829981219e-14
51.9047619047619
"chr3" 10548001 10549000 "*" 0 0 68.1029185867896
"chr3" 10550001 10551000 "*" 1.39779633423487e-07 1.98463037958589e-07 100
"chr3" 10554001 10555000 "*" 0 0 100
"chr3" 10558001 10559000 "*" 1.29037891483108e-11 3.24582280286866e-11 100
"chr3" 10575001 10576000 "*" 8.80406858527749e-14 2.91456141063452e-13 100
"chr3" 10638001 10639000 "*" 1.33226762955019e-15 5.48133041560118e-15 100
"chr3" 10641001 10642000 "*" 0 0 100
"chr3" 10667001 10668000 "*" 7.0006535457523e-08 1.03695832512054e-07 -100
"chr3" 10726001 10727000 "*" 1.39779633423487e-07 1.98463037958589e-07 100
"chr3" 10766001 10767000 "*" 0 0 100
"chr3" 10881001 10882000 "*" 2.08995487582797e-10 4.46944006972663e-10 100
"chr3" 10886001 10887000 "*" 9.08108033215171e-11 2.03407753381119e-10 100
"chr3" 10967001 10968000 "*" 2.22044604925031e-16 9.81641919380259e-16 100
"chr3" 10972001 10973000 "*" 3.99665338590705e-06 4.64666236277004e-06
58.6206896551724
"chr3" 10979001 10980000 "*" 8.00992605576312e-12 2.07541138227407e-11
73.2394366197183
"chr3" 10987001 10988000 "*" 6.76132659700457e-05 6.51054864379892e-05 -60
"chr3" 10991001 10992000 "*" 0 0 100
"chr3" 11015001 11016000 "*" 0.00010347159895574 9.67886604809062e-05 60
"chr3" 11019001 11020000 "*" 1.49880108324396e-14 5.44951848293151e-14 100
"chr3" 11026001 11027000 "*" 0 0 100
"chr3" 11046001 11047000 "*" 1.12055920098442e-11 2.84500040664577e-11 100
"chr3" 11094001 11095000 "*" 6.4152538836737e-10 1.27477697728417e-09 100
"chr3" 11096001 11097000 "*" 1.90181204118289e-13 6.05298617539364e-13 100
"chr3" 11118001 11119000 "*" 1.71604619403354e-10 3.70942260395654e-10
66.6666666666667
"chr3" 11174001 11175000 "*" 5.93988880304153e-09 1.03092599025277e-08
52.7272727272727
"chr3" 11179001 11180000 "*" 0 0 -85.8974358974359
"chr3" 11195001 11196000 "*" 0 0 -66.6666666666667
"chr3" 11227001 11228000 "*" 3.25951488022724e-11 7.75904249326549e-11
80.8823529411765
"chr3" 11376001 11377000 "*" 3.05311331771918e-12 8.34639368772922e-12 -100
"chr3" 11554001 11555000 "*" 0.000245538078540553 0.000215951647556943

54.5454545454545
"chr3" 11775001 11776000 "*" 0 0 100
"chr3" 11793001 11794000 "*" 1.02301417337358e-08 1.71328932446907e-08 -90
"chr3" 11941001 11942000 "*" 3.18702467250187e-06 3.76252872071621e-06
-65.3543307086614
"chr3" 11943001 11944000 "*" 4.03010957938932e-14 1.39096982198858e-13
66.6666666666667
"chr3" 11960001 11961000 "*" 4.44089209850063e-16 1.91071758245033e-15
77.2727272727273
"chr3" 11971001 11972000 "*" 0 0 100
"chr3" 12036001 12037000 "*" 0.000187786450556171 0.00016839982858272
51.5151515151515
"chr3" 12281001 12282000 "*" 1.67299207820548e-08 2.71377429441055e-08 -100
"chr3" 12510001 12511000 "*" 3.95353005888666e-09 7.03928529283733e-09 -100
"chr3" 12621001 12622000 "*" 0.000171830972439557 0.000155038437122457
59.2592592592593
"chr3" 12622001 12623000 "*" 1.43218770176645e-14 5.21983361092297e-14
58.3333333333333
"chr3" 12702001 12703000 "*" 0 0 -100
"chr3" 12867001 12868000 "*" 5.58502324388144e-08 8.41879077739806e-08 -60
"chr3" 12885001 12886000 "*" 3.33066907387547e-16 1.4495649018245e-15 100
"chr3" 12892001 12893000 "*" 2.22044604925031e-15 8.90449334476539e-15
-80.7017543859649
"chr3" 12906001 12907000 "*" 9.69457847332933e-12 2.48374835346384e-11
70.2702702702703
"chr3" 12937001 12938000 "*" 5.87943027596793e-11 1.35305900331253e-10
83.8709677419355
"chr3" 12970001 12971000 "*" 0.000153084554265992 0.00013925242430994
53.1914893617021
"chr3" 12982001 12983000 "*" 6.92287338566189e-11 1.57605630124247e-10 -100
"chr3" 13008001 13009000 "*" 0 0 70.4545454545455
"chr3" 13066001 13067000 "*" 1.89424675944849e-09 3.53307614828567e-09
-58.8235294117647
"chr3" 13078001 13079000 "*" 2.43005615629954e-09 4.45014222280278e-09 -100
"chr3" 13082001 13083000 "*" 0 0 82.3529411764706
"chr3" 13091001 13092000 "*" 0 0 57.5342465753425
"chr3" 13102001 13103000 "*" 2.04549566173995e-08 3.26899482308671e-08
65.5913978494624
"chr3" 13125001 13126000 "*" 1.53210777398272e-14 5.5628583449216e-14 -100
"chr3" 13131001 13132000 "*" 0 0 100
"chr3" 13136001 13137000 "*" 0 0 100
"chr3" 13157001 13158000 "*" 3.530509218308e-13 1.08684087355982e-12
63.1076783280327
"chr3" 13160001 13161000 "*" 6.59550958292954e-09 1.13456194382773e-08 -100
"chr3" 13184001 13185000 "*" 1.17905685215192e-13 3.83653245040640e-13 100
"chr3" 13207001 13208000 "*" 1.11022302462516e-15 4.59817122935606e-15 100
"chr3" 13226001 13227000 "*" 2.64951482975562e-09 4.82186011375752e-09 100
"chr3" 13238001 13239000 "*" 0 0 100
"chr3" 13240001 13241000 "*" 3.44169137633799e-14 1.19969681326249e-13
98.3050847457627
"chr3" 13251001 13252000 "*" 4.89213692542734e-05 4.81845684656791e-05
64.5161290322581

"chr3" 13256001 13257000 "*" 2.57127652503186e-13 8.04827375828401e-13
57.5757575757576
"chr3" 13268001 13269000 "*" 1.96644922567657e-11 4.82930612417392e-11 100
"chr3" 13291001 13292000 "*" 3.49194319015922e-08 5.41783859504158e-08
-71.4285714285714
"chr3" 13307001 13308000 "*" 3.26405569239796e-14 1.13973003849234e-13 100
"chr3" 13308001 13309000 "*" 0 0 -100
"chr3" 13326001 13327000 "*" 1.23905852245088e-08 2.04731737213761e-08
51.2820512820513
"chr3" 13341001 13342000 "*" 3.31956684362922e-14 1.15814343608523e-13 -75
"chr3" 13343001 13344000 "*" 1.11022302462516e-16 5.03662826618488e-16 100
"chr3" 13397001 13398000 "*" 1.39779633423487e-07 1.98463037958589e-07 -100
"chr3" 13501001 13502000 "*" 3.04324343503026e-11 7.26599935673121e-11 100
"chr3" 13521001 13522000 "*" 0 0 -70.5056179775281
"chr3" 13561001 13562000 "*" 6.04438721296674e-11 1.38849255306602e-10 100
"chr3" 13573001 13574000 "*" 3.57602836231763e-13 1.10032561446484e-12
59.4594594594595
"chr3" 13592001 13593000 "*" 0 0 -75.5555555555556
"chr3" 13606001 13607000 "*" 1.51656465163796e-13 4.87803420672651e-13 -100
"chr3" 13622001 13623000 "*" 4.32209823486573e-12 1.15935290479641e-11 100
"chr3" 13626001 13627000 "*" 3.04324343503026e-11 7.26599935673121e-11 100
"chr3" 13666001 13667000 "*" 1.92273974519708e-11 4.73730678997854e-11
97.7272727272727
"chr3" 13683001 13684000 "*" 1.22457599616155e-12 3.52441478267078e-12 -100
"chr3" 13709001 13710000 "*" 3.33066907387547e-16 1.4495649018245e-15 -100
"chr3" 13724001 13725000 "*" 1.12843068222901e-12 3.259512533505e-12 100
"chr3" 13739001 13740000 "*" 0 0 100
"chr3" 13810001 13811000 "*" 1.37425119284273e-07 1.95826814576532e-07
-57.1428571428571
"chr3" 13830001 13831000 "*" 8.3882012447134e-11 1.88922989503423e-10 100
"chr3" 13833001 13834000 "*" 3.33066907387547e-15 1.30941888440006e-14
55.1020408163265
"chr3" 13837001 13838000 "*" 0 0 55.984555984556
"chr3" 13871001 13872000 "*" 0 0 84.5070422535211
"chr3" 13874001 13875000 "*" 1.11022302462516e-16 5.03662826618488e-16
95.4545454545455
"chr3" 13902001 13903000 "*" 3.61932706027801e-14 1.25777907157994e-13 100
"chr3" 13907001 13908000 "*" 1.30828681221828e-12 3.75562014164691e-12
84.8101265822785
"chr3" 13920001 13921000 "*" 0 0 65.3390160529618
"chr3" 13921001 13922000 "*" 0 0 77.9727799082638
"chr3" 13977001 13978000 "*" 0 0 -100
"chr3" 14105001 14106000 "*" 3.33066907387547e-16 1.4495649018245e-15 100
"chr3" 14328001 14329000 "*" 1.39779633423487e-07 1.98463037958589e-07 -100
"chr3" 14348001 14349000 "*" 1.90520128851013e-06 2.32564618288801e-06
-67.5675675675676
"chr3" 14353001 14354000 "*" 0.000115428328377853 0.000107189271846237
54.5454545454545
"chr3" 14371001 14372000 "*" 1.4432899320127e-15 5.90750815956055e-15
-58.0645161290323
"chr3" 14387001 14388000 "*" 9.20794250323098e-05 8.68455135226072e-05
-64.2857142857143

"chr3" 14402001 14403000 "*" 3.84485043294358e-05 3.84728134870345e-05 -59.375
"chr3" 14433001 14434000 "*" 1.53210777398272e-14 5.5628583449216e-14 100
"chr3" 14443001 14444000 "*" 0 0 66.6666666666667
"chr3" 14452001 14453000 "*" 2.22044604925031e-16 9.81641919380259e-16 -100
"chr3" 14462001 14463000 "*" 1.07882591748876e-11 2.74553537455803e-11 100
"chr3" 14471001 14472000 "*" 3.6774594813771e-08 5.66494642520582e-08
55.1724137931034
"chr3" 14473001 14474000 "*" 1.35447209004269e-14 4.94674708073053e-14 -100
"chr3" 14477001 14478000 "*" 0 0 -66.6666666666667
"chr3" 14509001 14510000 "*" 4.75366453437376e-05 4.6905450034535e-05
-54.1666666666667
"chr3" 14538001 14539000 "*" 6.60282939435319e-12 1.73039395883942e-11
-74.468085106383
"chr3" 14581001 14582000 "*" 0 0 -82.5
"chr3" 14587001 14588000 "*" 1.16713305686744e-11 2.95455097228367e-11
-85.2459016393443
"chr3" 14607001 14608000 "*" 0.000201738106613503 0.000179962977547952
56.8181818181818
"chr3" 14617001 14618000 "*" 1.11022302462516e-16 5.03662826618488e-16 -100
"chr3" 14634001 14635000 "*" 1.11022302462516e-16 5.03662826618488e-16
-68.9956331877729
"chr3" 14643001 14644000 "*" 0 0 52.8085769652035
"chr3" 14644001 14645000 "*" 0 0 61.4192156501598
"chr3" 14660001 14661000 "*" 1.67606599155157e-07 2.35749100857771e-07
-56.6037735849057
"chr3" 14724001 14725000 "*" 0 0 -100
"chr3" 14803001 14804000 "*" 1.62191426955616e-08 2.64300079144354e-08
58.8235294117647
"chr3" 14833001 14834000 "*" 0.000214547426948486 0.00019064542382489
-50.9433962264151
"chr3" 14849001 14850000 "*" 1.98325800226939e-11 4.85612402473442e-11 100
"chr3" 14859001 14860000 "*" 6.77335965093562e-12 1.76897011246462e-11 -100
"chr3" 14861001 14862000 "*" 2.55351295663786e-15 1.01678090381833e-14
-58.3981693363844
"chr3" 14878001 14879000 "*" 1.20591092667155e-10 2.66075324129686e-10 -100
"chr3" 14920001 14921000 "*" 7.7715611723761e-16 3.26213507634405e-15
-89.5833333333333
"chr3" 14926001 14927000 "*" 2.38575468822777e-05 2.46816531377115e-05
-66.6666666666667
"chr3" 14941001 14942000 "*" 8.52140580320793e-12 2.19817259951489e-11 100
"chr3" 14943001 14944000 "*" 2.73014943985572e-11 6.58595916221891e-11 100
"chr3" 14948001 14949000 "*" 6.19782003496994e-12 1.63065872932661e-11
-76.5957446808511
"chr3" 14955001 14956000 "*" 9.5881362849326e-09 1.6110105266243e-08 60
"chr3" 14956001 14957000 "*" 1.55431223447522e-15 6.33705141101682e-15 -100
"chr3" 15105001 15106000 "*" 0 0 -100
"chr3" 15119001 15120000 "*" 4.01313771103418e-09 7.11882087237587e-09 -100
"chr3" 15160001 15161000 "*" 0 0 -52.8901543984288
"chr3" 15311001 15312000 "*" 0 0 -86.7158671586716
"chr3" 15347001 15348000 "*" 8.86513085163187e-13 2.59432994755486e-12
-98.0392156862745
"chr3" 15494001 15495000 "*" 6.59550958292954e-09 1.13456194382773e-08 100

"chr3" 15540001 15541000 "*" 8.25450818808804e-13 2.42473843346228e-12 100
"chr3" 16011001 16012000 "*" 3.6700841921089e-08 5.65530226072258e-08 100
"chr3" 16289001 16290000 "*" 0 0 -91.304347826087
"chr3" 16321001 16322000 "*" 1.67299207820548e-08 2.71377429441055e-08 -100
"chr3" 16407001 16408000 "*" 0 0 100
"chr3" 16509001 16510000 "*" 8.1956545862738e-05 7.7896169099937e-05
-54.5454545454545
"chr3" 16555001 16556000 "*" 0 0 65.8735482546991
"chr3" 16816001 16817000 "*" 6.81451797213128e-08 1.01537446338105e-07
51.0869565217391
"chr3" 17783001 17784000 "*" 4.03010957938932e-14 1.39096982198858e-13
57.1428571428571
"chr3" 17965001 17966000 "*" 8.3882012447134e-11 1.88922989503423e-10 -100
"chr3" 17992001 17993000 "*" 5.6362137179633e-11 1.29963270300065e-10 -100
"chr3" 19144001 19145000 "*" 0 0 -89.4366197183099
"chr3" 19531001 19532000 "*" 7.67056418382595e-11 1.7359448580318e-10 -100
"chr3" 19610001 19611000 "*" 3.78560138791784e-08 5.82437312896488e-08 75
"chr3" 19868001 19869000 "*" 6.4324989779152e-11 1.47230020825092e-10 -100
"chr3" 20966001 20967000 "*" 4.02167188440217e-12 1.08266700497926e-11 -100
"chr3" 21429001 21430000 "*" 4.1564529408733e-05 4.13773391809997e-05
-56.5217391304348
"chr3" 22358001 22359000 "*" 2.64951482975562e-09 4.82186011375752e-09 -100
"chr3" 23230001 23231000 "*" 1.80180457876045e-05 1.89967331199196e-05 60
"chr3" 23244001 23245000 "*" 0 0 85.6007380597544
"chr3" 23245001 23246000 "*" 0 0 85.8695652173913
"chr3" 23316001 23317000 "*" 1.99362748531939e-12 5.58533782705436e-12 -75
"chr3" 23672001 23673000 "*" 5.10702591327572e-15 1.96271840288234e-14 -100
"chr3" 23803001 23804000 "*" 1.11022302462516e-16 5.03662826618488e-16 100
"chr3" 23986001 23987000 "*" 0 0 100
"chr3" 24126001 24127000 "*" 7.7715611723761e-16 3.26213507634405e-15 -100
"chr3" 24320001 24321000 "*" 2.62900812231237e-13 8.21294938531841e-13 -100
"chr3" 24536001 24537000 "*" 0 0 75.4282339043119
"chr3" 25427001 25428000 "*" 1.96644922567657e-11 4.82930612417392e-11 100
"chr3" 25497001 25498000 "*" 1.55431223447522e-14 5.6388152448458e-14 -100
"chr3" 25619001 25620000 "*" 4.32209823486573e-12 1.15935290479641e-11 100
"chr3" 26095001 26096000 "*" 1.20266063774466e-09 2.29962481156257e-09 75
"chr3" 26200001 26201000 "*" 1.56863411149288e-12 4.4469621057462e-12 100
"chr3" 27656001 27657000 "*" 1.28785870856518e-14 4.71440608755782e-14 100
"chr3" 27674001 27675000 "*" 0 0 78.6729857819905
"chr3" 27759001 27760000 "*" 6.10622663543836e-15 2.32593540648772e-14
62.2222222222222
"chr3" 27763001 27764000 "*" 0 0 53.3724516662905
"chr3" 32025001 32026000 "*" 0 0 -100
"chr3" 32127001 32128000 "*" 6.77335965093562e-12 1.76897011246462e-11 100
"chr3" 32145001 32146000 "*" 8.3882012447134e-11 1.88922989503423e-10 -100
"chr3" 32381001 32382000 "*" 2.455369241261e-12 6.8003688121118e-12 100
"chr3" 32434001 32435000 "*" 7.7715611723761e-16 3.26213507634405e-15
-75.9615384615385
"chr3" 32542001 32543000 "*" 1.345879296899e-09 2.56015124985088e-09 -58
"chr3" 33195001 33196000 "*" 8.52847792387479e-11 1.91942413833238e-10
62.7906976744186
"chr3" 33215001 33216000 "*" 4.04946212464452e-05 4.0376350196346e-05

-64.7058823529412
"chr3" 33318001 33319000 "*" 0 0 99.4047619047619
"chr3" 33927001 33928000 "*" 1.11731823793093e-09 2.14826105494601e-09
88.3720930232558
"chr3" 34005001 34006000 "*" 6.24654217240561e-10 1.2462108191303e-09 -100
"chr3" 34246001 34247000 "*" 1.89581683684992e-12 5.32311367606535e-12 -100
"chr3" 35587001 35588000 "*" 3.6700841921089e-08 5.65530226072258e-08 100
"chr3" 35750001 35751000 "*" 4.99020824662466e-11 1.15790792586157e-10 -100
"chr3" 36857001 36858000 "*" 2.88710388929303e-10 6.03399264731139e-10 -100
"chr3" 36872001 36873000 "*" 1.24050436589584e-10 2.73360976324848e-10 68.75
"chr3" 36955001 36956000 "*" 5.01025332333427e-10 1.01358031135889e-09 -100
"chr3" 37493001 37494000 "*" 2.60838562882171e-06 3.11967503311925e-06
-67.0886075949367
"chr3" 37494001 37495000 "*" 0 0 51.7611976143778
"chr3" 37521001 37522000 "*" 7.27481882512038e-08 1.07497206386442e-07
51.7241379310345
"chr3" 37584001 37585000 "*" 4.99020824662466e-11 1.15790792586157e-10 -100
"chr3" 37646001 37647000 "*" 2.04695393968279e-08 3.27119999299478e-08
86.6666666666667
"chr3" 37744001 37745000 "*" 6.37490060739765e-13 1.89496793587877e-12 -100
"chr3" 37807001 37808000 "*" 6.88338275267597e-15 2.60518659957509e-14 100
"chr3" 37896001 37897000 "*" 3.6700841921089e-08 5.65530226072258e-08 -100
"chr3" 37901001 37902000 "*" 0 0 -100
"chr3" 38012001 38013000 "*" 2.73625566649116e-12 7.52483363579137e-12 100
"chr3" 38028001 38029000 "*" 6.26498852795976e-12 1.64749932126438e-11
83.0508474576271
"chr3" 38038001 38039000 "*" 5.41788646406527e-05 5.29743727453169e-05
-51.7241379310345
"chr3" 38040001 38041000 "*" 0 0 -55.7308479199818
"chr3" 38046001 38047000 "*" 0 0 -73.6271602413335
"chr3" 38057001 38058000 "*" 1.11022302462516e-16 5.03662826618488e-16 100
"chr3" 38179001 38180000 "*" 0 0 -80
"chr3" 38180001 38181000 "*" 0 0 -76.5690376569038
"chr3" 38323001 38324000 "*" 0 0 100
"chr3" 38324001 38325000 "*" 6.93112234273485e-13 2.05218842007357e-12 -100
"chr3" 38348001 38349000 "*" 1.01316732781243e-10 2.25805994726174e-10 85
"chr3" 38376001 38377000 "*" 0 0 82.2033898305085
"chr3" 38585001 38586000 "*" 0 0 100
"chr3" 38618001 38619000 "*" 4.71103192345446e-07 6.24717132053794e-07
-71.7948717948718
"chr3" 38640001 38641000 "*" 1.11022302462516e-16 5.03662826618488e-16
-72.1212121212121
"chr3" 38665001 38666000 "*" 0 0 63.2630410654828
"chr3" 38687001 38688000 "*" 1.39091355655374e-06 1.73242473179782e-06
70.9677419354839
"chr3" 38690001 38691000 "*" 0 0 57.8881766381766
"chr3" 38695001 38696000 "*" 6.59550958292954e-09 1.13456194382773e-08 100
"chr3" 38871001 38872000 "*" 3.95353005888666e-09 7.03928529283733e-09 100
"chr3" 39028001 39029000 "*" 6.24654217240561e-10 1.2462108191303e-09 100
"chr3" 39193001 39194000 "*" 1.67299207820548e-08 2.71377429441055e-08 -100
"chr3" 39309001 39310000 "*" 5.27142773876221e-11 1.22010586806028e-10
-73.394495412844

"chr3" 39395001 39396000 "*" 5.794809077031e-12 1.52911192757929e-11 -100
"chr3" 39471001 39472000 "*" 7.53774820339004e-12 1.95787303791712e-11
57.5746268656716
"chr3" 39523001 39524000 "*" 2.73625566649116e-12 7.52483363579137e-12 100
"chr3" 41289001 41290000 "*" 2.88779000712225e-11 6.92935809466068e-11 -100
"chr3" 41306001 41307000 "*" 0 0 -100
"chr3" 41398001 41399000 "*" 0 0 100
"chr3" 42054001 42055000 "*" 3.33683969344634e-10 6.91982883132938e-10
54.9180327868852
"chr3" 42131001 42132000 "*" 3.40340680804463e-06 3.99960927822812e-06
-66.6666666666667
"chr3" 42203001 42204000 "*" 2.64951482975562e-09 4.82186011375752e-09 100
"chr3" 42271001 42272000 "*" 5.01025332333427e-10 1.01358031135889e-09 100
"chr3" 42392001 42393000 "*" 1.29037891483108e-11 3.24582280286866e-11 100
"chr3" 42542001 42543000 "*" 0 0 52.4271844660194
"chr3" 42546001 42547000 "*" 1.11022302462516e-16 5.03662826618488e-16 100
"chr3" 42549001 42550000 "*" 2.15125472990962e-10 4.58126659943874e-10 100
"chr3" 42558001 42559000 "*" 2.88779000712225e-11 6.92935809466068e-11 100
"chr3" 42694001 42695000 "*" 1.54630752646767e-11 3.85079502648228e-11 -100
"chr3" 42696001 42697000 "*" 0 0 -95.0530035335689
"chr3" 42739001 42740000 "*" 3.88169574261354e-09 6.93447853396753e-09
-66.6666666666667
"chr3" 42750001 42751000 "*" 1.3417011834882e-08 2.20794819956426e-08
54.5454545454545
"chr3" 42774001 42775000 "*" 0.000281157030617152 0.00024485915731249
53.8461538461538
"chr3" 42846001 42847000 "*" 0 0 75.6878093703235
"chr3" 42853001 42854000 "*" 6.52733422867868e-12 1.71146951718423e-11 -100
"chr3" 43020001 43021000 "*" 0 0 85.8190709046455
"chr3" 43021001 43022000 "*" 0 0 72.7619693136935
"chr3" 43043001 43044000 "*" 1.52794177310511e-09 2.87642564232045e-09 100
"chr3" 43057001 43058000 "*" 3.03090885722668e-14 1.06210046699299e-13 -100
"chr3" 43074001 43075000 "*" 3.31767707417718e-07 4.49226630918757e-07
52.5773195876289
"chr3" 43327001 43328000 "*" 0 0 -91.5492957746479
"chr3" 43496001 43497000 "*" 2.08995487582797e-10 4.46944006972663e-10 -100
"chr3" 43812001 43813000 "*" 3.07963307077941e-08 4.82018501272284e-08
-66.6666666666667
"chr3" 43828001 43829000 "*" 4.20365394160349e-07 5.61132687506387e-07
79.1666666666667
"chr3" 43935001 43936000 "*" 0 0 62.9366737288922
"chr3" 44036001 44037000 "*" 0 0 51.5296747161001
"chr3" 44040001 44041000 "*" 0 0 53.2106307560088
"chr3" 44055001 44056000 "*" 1.11022302462516e-16 5.03662826618488e-16
97.4358974358974
"chr3" 44056001 44057000 "*" 7.0006535457523e-08 1.03695832512054e-07 -100
"chr3" 44109001 44110000 "*" 0 0 100
"chr3" 44147001 44148000 "*" 2.1094237467878e-15 8.47434540879246e-15 100
"chr3" 44244001 44245000 "*" 9.0072393987839e-13 2.63157725646847e-12 -100
"chr3" 44453001 44454000 "*" 2.69018141096922e-12 7.41487347763769e-12 100
"chr3" 44462001 44463000 "*" 0 0 100
"chr3" 44466001 44467000 "*" 0 0 -100

"chr3" 44487001 44488000 "*" 1.98325800226939e-11 4.85612402473442e-11 100
"chr3" 44610001 44611000 "*" 3.34128893442198e-08 5.19772864618523e-08 100
"chr3" 44619001 44620000 "*" 0 0 52.6315789473684
"chr3" 44712001 44713000 "*" 7.0006535457523e-08 1.03695832512054e-07 100
"chr3" 44770001 44771000 "*" 0 0 68.1818181818182
"chr3" 44771001 44772000 "*" 0 0 73.8589211618257
"chr3" 44969001 44970000 "*" 9.65338919911574e-13 2.80678910656113e-12 100
"chr3" 45072001 45073000 "*" 1.98365768255826e-11 4.85612402473442e-11 -100
"chr3" 45121001 45122000 "*" 3.07531777821168e-14 1.07626052665081e-13 100
"chr3" 45302001 45303000 "*" 5.6362137179633e-11 1.29963270300065e-10 100
"chr3" 45362001 45363000 "*" 4.02167188440217e-12 1.08266700497926e-11 100
"chr3" 45363001 45364000 "*" 4.02167188440217e-12 1.08266700497926e-11 100
"chr3" 45371001 45372000 "*" 2.00227675550835e-08 3.20459176617961e-08 100
"chr3" 45533001 45534000 "*" 1.5277158427196e-09 2.87642564232045e-09 100
"chr3" 45618001 45619000 "*" 9.99200722162641e-16 4.15382497462808e-15 100
"chr3" 45637001 45638000 "*" 0 0 -100
"chr3" 45666001 45667000 "*" 0.000172581294497709 0.000155676695599483
54.2857142857143
"chr3" 45702001 45703000 "*" 3.15469872447238e-12 8.61309039677907e-12
82.5757575757576
"chr3" 45706001 45707000 "*" 5.7065463465733e-14 1.93318145576218e-13 -100
"chr3" 45799001 45800000 "*" 4.02167188440217e-12 1.08266700497926e-11 100
"chr3" 45822001 45823000 "*" 6.9613448339112e-10 1.37757981016631e-09
57.3770491803279
"chr3" 45838001 45839000 "*" 0 0 78.0936454849498
"chr3" 45939001 45940000 "*" 6.66133814775094e-15 2.52791665956799e-14 -100
"chr3" 45949001 45950000 "*" 9.47020240005259e-14 3.12491189162604e-13 -100
"chr3" 46195001 46196000 "*" 6.88338275267597e-15 2.60518659957509e-14 100
"chr3" 46399001 46400000 "*" 9.63829016598083e-12 2.46993429847588e-11 -100
"chr3" 46496001 46497000 "*" 6.70841160399505e-12 1.75486545176836e-11 100
"chr3" 46703001 46704000 "*" 1.03283833808865e-05 1.12938649360329e-05
-58.0152671755725
"chr3" 46734001 46735000 "*" 0 0 -100
"chr3" 46736001 46737000 "*" 0 0 100
"chr3" 46742001 46743000 "*" 0 0 61.8835720816824
"chr3" 46743001 46744000 "*" 2.22044604925031e-16 9.81641919380259e-16
87.9069767441861
"chr3" 46890001 46891000 "*" 2.7739215369138e-09 5.03716664165354e-09
-94.1176470588235
"chr3" 46894001 46895000 "*" 4.75952610656805e-13 1.44150704337162e-12
84.6153846153846
"chr3" 46899001 46900000 "*" 3.73034936274053e-14 1.29420415488591e-13
73.3333333333333
"chr3" 46907001 46908000 "*" 1.87510396266743e-05 1.97080033495427e-05
-66.6666666666667
"chr3" 46933001 46934000 "*" 0 0 57.1948998178506
"chr3" 46936001 46937000 "*" 2.16382467499443e-13 6.82775854166559e-13 -100
"chr3" 46963001 46964000 "*" 5.43117762319412e-06 6.19387126034938e-06
84.6153846153846
"chr3" 46990001 46991000 "*" 6.35280716920761e-11 1.45637313256185e-10
64.2857142857143
"chr3" 47034001 47035000 "*" 5.10501769013239e-06 5.84637189700889e-06

-58.3333333333333
"chr3" 47423001 47424000 "*" 0 0 -100
"chr3" 47560001 47561000 "*" 1.56863411149288e-12 4.4469621057462e-12 100
"chr3" 47608001 47609000 "*" 0 0 72.5
"chr3" 48053001 48054000 "*" 0 0 -100
"chr3" 48189001 48190000 "*" 1.92946192356658e-09 3.59193011611483e-09 100
"chr3" 48259001 48260000 "*" 7.0006535457523e-08 1.03695832512054e-07 100
"chr3" 48380001 48381000 "*" 6.91004031772025e-09 1.18592913246344e-08
-56.5217391304348
"chr3" 48440001 48441000 "*" 8.7349629751543e-09 1.47505871898511e-08 100
"chr3" 48475001 48476000 "*" 4.5729012104756e-05 4.52411800862675e-05
-57.8947368421053
"chr3" 48590001 48591000 "*" 2.58681964737661e-14 9.14008479605639e-14 -100
"chr3" 48593001 48594000 "*" 2.12747486294518e-10 4.54524902925783e-10
53.3333333333333
"chr3" 48620001 48621000 "*" 1.16917537873462e-08 1.93756788670422e-08
-70.4545454545455
"chr3" 48633001 48634000 "*" 7.91722243320692e-12 2.05253922596697e-11
68.4210526315789
"chr3" 48673001 48674000 "*" 0 0 65.8163265306122
"chr3" 48700001 48701000 "*" 0 0 85.3968253968254
"chr3" 48721001 48722000 "*" 7.0006535457523e-08 1.03695832512054e-07 -100
"chr3" 48722001 48723000 "*" 1.29037891483108e-11 3.24582280286866e-11 100
"chr3" 48772001 48773000 "*" 7.67056418382595e-11 1.7359448580318e-10 100
"chr3" 48948001 48949000 "*" 1.03576166310404e-07 1.50002166837079e-07 -60
"chr3" 49054001 49055000 "*" 2.08995487582797e-10 4.46944006972663e-10 -100
"chr3" 49259001 49260000 "*" 6.46149800331841e-14 2.17664365839349e-13 -100
"chr3" 49364001 49365000 "*" 7.0006535457523e-08 1.03695832512054e-07 100
"chr3" 49395001 49396000 "*" 0 0 -85.1851851851852
"chr3" 49403001 49404000 "*" 2.17381668221606e-13 6.85151076607736e-13 100
"chr3" 49578001 49579000 "*" 1.33226762955019e-15 5.48133041560118e-15 100
"chr3" 49580001 49581000 "*" 0 0 -100
"chr3" 49592001 49593000 "*" 1.11022302462516e-16 5.03662826618488e-16
51.9436997319035
"chr3" 49665001 49666000 "*" 2.08814465718632e-09 3.86232567960463e-09 100
"chr3" 49712001 49713000 "*" 0.000131232126691527 0.000120712161400679 52
"chr3" 49725001 49726000 "*" 0 0 100
"chr3" 49760001 49761000 "*" 0 0 -79.5992714025501
"chr3" 49835001 49836000 "*" 6.70841160399505e-12 1.75486545176836e-11 -100
"chr3" 49910001 49911000 "*" 1.0131347689013e-05 1.10909163221897e-05 62.5
"chr3" 49913001 49914000 "*" 4.73234496034536e-09 8.30627300264826e-09 100
"chr3" 49921001 49922000 "*" 2.02327044007689e-12 5.65300384792621e-12 -100
"chr3" 49941001 49942000 "*" 0 0 -90.3485254691689
"chr3" 49975001 49976000 "*" 8.88178419700125e-16 3.70670032207938e-15 100
"chr3" 50191001 50192000 "*" 0 0 84.2424242424242
"chr3" 50201001 50202000 "*" 1.49880108324396e-14 5.44951848293151e-14 -100
"chr3" 50257001 50258000 "*" 2.43005615629954e-09 4.45014222280278e-09 -100
"chr3" 50262001 50263000 "*" 5.55111512312578e-16 2.36485094870365e-15 -100
"chr3" 50264001 50265000 "*" 0 0 65.4320987654321
"chr3" 50265001 50266000 "*" 2.43005615629954e-09 4.45014222280278e-09 -100
"chr3" 50304001 50305000 "*" 1.92625793205004e-07 2.68993984113541e-07
-73.3333333333333

"chr3" 50308001 50309000 "*" 1.5277158427196e-09 2.87642564232045e-09 -100
"chr3" 50333001 50334000 "*" 9.14157638476354e-13 2.66886212024714e-12 -90.9090909090909
"chr3" 50375001 50376000 "*" 7.43849426498855e-15 2.80357739563059e-14 52.4050632911392
"chr3" 50397001 50398000 "*" 1.66500147003035e-12 4.71006997680282e-12 64.5833333333333
"chr3" 50403001 50404000 "*" 9.93161875307846e-05 9.31526365269394e-05 -60.6060606060606
"chr3" 50404001 50405000 "*" 4.50215680303234e-06 5.19390556817063e-06 57.6923076923077
"chr3" 50412001 50413000 "*" 0 0 53.5528666558389
"chr3" 50423001 50424000 "*" 2.82773804372027e-13 8.79129664109438e-13 100
"chr3" 50425001 50426000 "*" 0 0 -67.7886497064579
"chr3" 50428001 50429000 "*" 9.45333145097038e-10 1.83955395995353e-09 62.5
"chr3" 50434001 50435000 "*" 1.18738976429e-08 1.96630728742272e-08 -65.3846153846154
"chr3" 50446001 50447000 "*" 0 0 73.972602739726
"chr3" 50450001 50451000 "*" 2.08995487582797e-10 4.46944006972663e-10 100
"chr3" 50459001 50460000 "*" 3.56775498033812e-10 7.35898629809829e-10 100
"chr3" 50462001 50463000 "*" 1.11061780883048e-08 1.85148915254297e-08 52.3809523809524
"chr3" 50469001 50470000 "*" 1.89425159169421e-05 1.98874471894124e-05 62.5
"chr3" 50474001 50475000 "*" 4.89292739658254e-09 8.5744149247165e-09 52
"chr3" 50476001 50477000 "*" 0.00112127033826392 0.000882170541990551 53.3333333333333
"chr3" 50482001 50483000 "*" 1.5277158427196e-09 2.87642564232045e-09 -100
"chr3" 50483001 50484000 "*" 7.9562239657327e-05 7.57887419325014e-05 -61.1111111111111
"chr3" 50536001 50537000 "*" 0 0 96.875
"chr3" 50539001 50540000 "*" 1.51434420558871e-13 4.8733354760812e-13 -57.0247933884297
"chr3" 50540001 50541000 "*" 0 0 52.1052631578947
"chr3" 50567001 50568000 "*" 0 0 74.8904939422181
"chr3" 50568001 50569000 "*" 3.31932736852281e-09 5.97720427518928e-09 55.9139784946237
"chr3" 50569001 50570000 "*" 1.13140401492018e-08 1.87801380787728e-08 100
"chr3" 50570001 50571000 "*" 0 0 59.41647597254
"chr3" 50573001 50574000 "*" 1.35036426485158e-12 3.86629547363774e-12 100
"chr3" 50584001 50585000 "*" 3.32033289751621e-11 7.8963779356202e-11 82.6086956521739
"chr3" 50594001 50595000 "*" 5.07371922253697e-14 1.73287898516782e-13 100
"chr3" 50616001 50617000 "*" 3.887996002927e-08 5.97507926181626e-08 -52.9411764705882
"chr3" 50684001 50685000 "*" 8.7349629751543e-09 1.47505871898511e-08 -100
"chr3" 50712001 50713000 "*" 0 0 79.5151802938598
"chr3" 51615001 51616000 "*" 0 0 100
"chr3" 51744001 51745000 "*" 9.97457187179585e-07 1.26568823004915e-06 68.75
"chr3" 51745001 51746000 "*" 2.1524589532973e-07 2.9870143746761e-07 77.3584905660377
"chr3" 51753001 51754000 "*" 0 0 -100
"chr3" 51879001 51880000 "*" 6.60582699651968e-14 2.22176301799e-13 -100

"chr3" 51907001 51908000 "*" 3.55504514715221e-12 9.65870420664016e-12 80
"chr3" 51988001 51989000 "*" 6.24654217240561e-10 1.2462108191303e-09 100
"chr3" 51996001 51997000 "*" 1.98365768255826e-11 4.85612402473442e-11 100
"chr3" 52008001 52009000 "*" 1.11022302462516e-16 5.03662826618488e-16
-50.1195516811955
"chr3" 52016001 52017000 "*" 1.91274773797545e-11 4.71394150510811e-11
-56.7567567567568
"chr3" 52049001 52050000 "*" 1.13140401492018e-08 1.87801380787728e-08 100
"chr3" 52057001 52058000 "*" 1.95399252334028e-14 7.01253661173697e-14 -100
"chr3" 52077001 52078000 "*" 1.13140401492018e-08 1.87801380787728e-08 100
"chr3" 52114001 52115000 "*" 3.04201108747293e-14 1.06563274901204e-13 100
"chr3" 52215001 52216000 "*" 3.6700841921089e-08 5.65530226072258e-08 100
"chr3" 52252001 52253000 "*" 1.12458486967171e-09 2.15740815043925e-09 100
"chr3" 52281001 52282000 "*" 0.000285593505977677 0.000248445226337853
52.1739130434783
"chr3" 52283001 52284000 "*" 2.25158780509105e-11 5.48210405304675e-11
55.1724137931034
"chr3" 52334001 52335000 "*" 0 0 -100
"chr3" 52405001 52406000 "*" 6.0675591218029e-09 1.05177116301873e-08
-82.2222222222222
"chr3" 52424001 52425000 "*" 3.29762048112414e-07 4.4664129931678e-07
57.1428571428571
"chr3" 52425001 52426000 "*" 2.31220553814637e-09 4.25337583083948e-09
59.8290598290598
"chr3" 52460001 52461000 "*" 2.15125472990962e-10 4.58126659943874e-10 -100
"chr3" 52486001 52487000 "*" 3.25996230010617e-10 6.7693239698185e-10
83.0769230769231
"chr3" 52488001 52489000 "*" 2.02579219621413e-07 2.82132925455933e-07 -56.25
"chr3" 52490001 52491000 "*" 1.21839982547556e-10 2.68710055617777e-10
72.4489795918367
"chr3" 52533001 52534000 "*" 4.73234496034536e-09 8.30627300264826e-09 100
"chr3" 52543001 52544000 "*" 5.63660229602192e-13 1.68905058935478e-12 -100
"chr3" 52547001 52548000 "*" 1.72488727745446e-08 2.793390184933e-08
-58.5365853658537
"chr3" 52566001 52567000 "*" 4.65637306490407e-10 9.46720524046618e-10 -100
"chr3" 52805001 52806000 "*" 0 0 -75.3333333333333
"chr3" 52815001 52816000 "*" 7.01059210683752e-11 1.59506838684118e-10
51.3513513513514
"chr3" 52816001 52817000 "*" 7.16315895488151e-13 2.11848386291434e-12
-61.1111111111111
"chr3" 52820001 52821000 "*" 6.88338275267597e-15 2.60518659957509e-14 -100
"chr3" 52844001 52845000 "*" 8.52111484483942e-07 1.09183873929303e-06
-72.972972972973
"chr3" 52856001 52857000 "*" 3.90831811358794e-12 1.05610567215048e-11 100
"chr3" 52876001 52877000 "*" 0 0 -53.5714285714286
"chr3" 52930001 52931000 "*" 2.22012963568829e-10 4.72106983629482e-10 64
"chr3" 53081001 53082000 "*" 0 0 -100
"chr3" 53233001 53234000 "*" 1.5785740136165e-06 1.9503745122263e-06
66.6666666666667
"chr3" 53276001 53277000 "*" 0 0 68
"chr3" 53637001 53638000 "*" 3.6700841921089e-08 5.65530226072258e-08 100
"chr3" 53675001 53676000 "*" 2.15125472990962e-10 4.58126659943874e-10 -100

"chr3" 53759001 53760000 "*" 6.24654217240561e-10 1.2462108191303e-09 100
"chr3" 53829001 53830000 "*" 1.48087875295744e-10 3.22075573380618e-10 -100
"chr3" 54082001 54083000 "*" 1.39779633423487e-07 1.98463037958589e-07 100
"chr3" 54154001 54155000 "*" 0 0 67.2131147540984
"chr3" 54156001 54157000 "*" 0 0 62.4755603430967
"chr3" 54237001 54238000 "*" 8.11898925867638e-09 1.3801503462213e-08
-84.5454545454545
"chr3" 54251001 54252000 "*" 3.07531777821168e-14 1.07626052665081e-13 -100
"chr3" 54306001 54307000 "*" 2.64951482975562e-09 4.82186011375752e-09 -100
"chr3" 54345001 54346000 "*" 9.02763398480388e-08 1.31712990276921e-07
58.1538461538462
"chr3" 54376001 54377000 "*" 1.11022302462516e-16 5.03662826618488e-16 -100
"chr3" 54522001 54523000 "*" 4.81119522000029e-08 7.3130006853651e-08 -81.25
"chr3" 54571001 54572000 "*" 0 0 98.8235294117647
"chr3" 54633001 54634000 "*" 1.11023489957063e-07 1.60098147216736e-07
66.6666666666667
"chr3" 54709001 54710000 "*" 2.88710388929303e-10 6.03399264731139e-10 -100
"chr3" 54930001 54931000 "*" 3.40125705378114e-10 7.03784073980162e-10 -100
"chr3" 55139001 55140000 "*" 4.79226938610022e-06 5.50916092610823e-06
-58.6206896551724
"chr3" 55190001 55191000 "*" 9.63829016598083e-12 2.46993429847588e-11 -100
"chr3" 55439001 55440000 "*" 1.28785870856518e-14 4.71440608755782e-14 -100
"chr3" 55521001 55522000 "*" 0 0 83.0811939731313
"chr3" 55547001 55548000 "*" 2.35932444136999e-07 3.25820146356964e-07
-70.5882352941177
"chr3" 55882001 55883000 "*" 5.8616765863384e-07 7.68442756313584e-07
91.6666666666667
"chr3" 56501001 56502000 "*" 2.44249065417534e-15 9.75180348030994e-15
84.3971631205674
"chr3" 56502001 56503000 "*" 0 0 90.0949796472185
"chr3" 56835001 56836000 "*" 4.78841410966879e-11 1.11468187156588e-10 -100
"chr3" 56859001 56860000 "*" 4.01313771103418e-09 7.11882087237587e-09 -100
"chr3" 56942001 56943000 "*" 1.29037891483108e-11 3.24582280286866e-11 100
"chr3" 57161001 57162000 "*" 3.77475828372553e-15 1.47379557022071e-14 100
"chr3" 58090001 58091000 "*" 3.33066907387547e-16 1.4495649018245e-15 -100
"chr3" 58097001 58098000 "*" 8.88178419700125e-16 3.70670032207938e-15 100
"chr3" 58282001 58283000 "*" 1.67299207820548e-08 2.71377429441055e-08 -100
"chr3" 58291001 58292000 "*" 0 0 57.1428571428571
"chr3" 58398001 58399000 "*" 2.52627301788877e-06 3.02719727625952e-06
-60.7142857142857
"chr3" 58472001 58473000 "*" 7.48416884022163e-11 1.6982312542875e-10 -65
"chr3" 58539001 58540000 "*" 1.11022302462516e-16 5.03662826618488e-16 -100
"chr3" 58551001 58552000 "*" 1.09366072531536e-07 1.57901959213512e-07
-52.0833333333333
"chr3" 58617001 58618000 "*" 2.88657986402541e-15 1.14155613124573e-14 100
"chr3" 59627001 59628000 "*" 9.04570973681018e-10 1.76414389822173e-09 100
"chr3" 60087001 60088000 "*" 3.7274627828765e-12 1.00985250136599e-11 100
"chr3" 60261001 60262000 "*" 3.10862446895044e-15 1.22466437093774e-14 -100
"chr3" 60371001 60372000 "*" 2.64951482975562e-09 4.82186011375752e-09 -100
"chr3" 61416001 61417000 "*" 1.78512936888087e-06 2.1878826958132e-06 -60
"chr3" 61490001 61491000 "*" 1.13140401492018e-08 1.87801380787728e-08 -100
"chr3" 61513001 61514000 "*" 0 0 100

"chr3" 61786001 61787000 "*" 6.4152538836737e-10 1.27477697728417e-09 -100
"chr3" 61815001 61816000 "*" 1.39779633423487e-07 1.98463037958589e-07 100
"chr3" 61852001 61853000 "*" 3.65130148338721e-12 9.90602415539447e-12 -100
"chr3" 62365001 62366000 "*" 0 0 100
"chr3" 62709001 62710000 "*" 5.03264097062583e-13 1.51942428521737e-12 100
"chr3" 62860001 62861000 "*" 0 0 54.6817405497725
"chr3" 63603001 63604000 "*" 2.33028818463765e-10 4.93719283454501e-10 100
"chr3" 63719001 63720000 "*" 4.99900121297969e-12 1.32921056339624e-11 100
"chr3" 64175001 64176000 "*" 2.33028818463765e-10 4.93719283454501e-10 -100
"chr3" 64279001 64280000 "*" 3.7274627828765e-12 1.00985250136599e-11 100
"chr3" 64312001 64313000 "*" 0 0 -100
"chr3" 64430001 64431000 "*" 0 0 67.0606412613202
"chr3" 64532001 64533000 "*" 1.5277158427196e-09 2.87642564232045e-09 -100
"chr3" 64673001 64674000 "*" 0 0 75.9074284785436
"chr3" 64761001 64762000 "*" 1.49613654798486e-12 4.26244554429779e-12 -100
"chr3" 66025001 66026000 "*" 0 0 -53.030303030303
"chr3" 66169001 66170000 "*" 3.63445940010365e-11 8.58565209111204e-11 100
"chr3" 66487001 66488000 "*" 4.99277286181155e-11 1.15839212736154e-10
-68.5185185185185
"chr3" 66522001 66523000 "*" 7.99844592513921e-06 8.89173797399106e-06
-66.6666666666667
"chr3" 66550001 66551000 "*" 0 0 81.0729100458301
"chr3" 66687001 66688000 "*" 0 0 -100
"chr3" 66827001 66828000 "*" 1.5277158427196e-09 2.87642564232045e-09 -100
"chr3" 67022001 67023000 "*" 0 0 -97.1428571428571
"chr3" 67069001 67070000 "*" 0.000319504735349585 0.000275693893819955
53.4632034632035
"chr3" 67697001 67698000 "*" 1.00808250635964e-13 3.31107370956809e-13 -100
"chr3" 67698001 67699000 "*" 2.08814465718632e-09 3.86232567960463e-09 -100
"chr3" 67705001 67706000 "*" 4.34645670899236e-07 5.79023271110259e-07
66.6666666666667
"chr3" 68980001 68981000 "*" 0 0 52.6074097135741
"chr3" 69250001 69251000 "*" 3.40125705378114e-10 7.03784073980162e-10 100
"chr3" 69256001 69257000 "*" 6.90747459231034e-12 1.80140148623876e-11 -100
"chr3" 69789001 69790000 "*" 0 0 -95.6349206349206
"chr3" 69970001 69971000 "*" 3.6700841921089e-08 5.65530226072258e-08 100
"chr3" 70114001 70115000 "*" 4.73234496034536e-09 8.30627300264826e-09 100
"chr3" 70246001 70247000 "*" 2.31469791711625e-06 2.79034791839104e-06 -75
"chr3" 70526001 70527000 "*" 2.33028818463765e-10 4.93719283454501e-10 -100
"chr3" 70753001 70754000 "*" 0 0 -100
"chr3" 71114001 71115000 "*" 0 0 66.8632590650939
"chr3" 71296001 71297000 "*" 2.28177730898782e-06 2.75380678531544e-06
-51.2820512820513
"chr3" 71310001 71311000 "*" 3.84137166520304e-14 1.32929369817675e-13 -100
"chr3" 71555001 71556000 "*" 2.4535928844216e-14 8.69144396197119e-14 100
"chr3" 71630001 71631000 "*" 0 0 100
"chr3" 71652001 71653000 "*" 0 0 -100
"chr3" 71669001 71670000 "*" 2.22044604925031e-16 9.81641919380259e-16
-55.5555555555556
"chr3" 71801001 71802000 "*" 3.6700841921089e-08 5.65530226072258e-08 -100
"chr3" 71858001 71859000 "*" 2.15125472990962e-10 4.58126659943874e-10 -100
"chr3" 71861001 71862000 "*" 0.000923822137060548 0.000737225242626394 -60

"chr3" 72022001 72023000 "*" 1.52992305169075e-06 1.89382781181468e-06
62.962962962963
"chr3" 72096001 72097000 "*" 2.79440914852103e-11 6.73046092948135e-11 100
"chr3" 72373001 72374000 "*" 6.59550958292954e-09 1.13456194382773e-08 -100
"chr3" 72401001 72402000 "*" 5.01025332333427e-10 1.01358031135889e-09 -100
"chr3" 72402001 72403000 "*" 1.14124265593318e-11 2.89138062669327e-11 -100
"chr3" 72427001 72428000 "*" 0 0 100
"chr3" 72447001 72448000 "*" 2.33028818463765e-10 4.93719283454501e-10 100
"chr3" 72627001 72628000 "*" 0 0 100
"chr3" 72640001 72641000 "*" 1.65279911978899e-09 3.10181364854418e-09
78.9473684210526
"chr3" 72651001 72652000 "*" 4.14335232790108e-13 1.26153063645578e-12 100
"chr3" 72701001 72702000 "*" 6.52733422867868e-12 1.71146951718423e-11 100
"chr3" 72990001 72991000 "*" 3.76365605347928e-14 1.3043487970679e-13 -100
"chr3" 73054001 73055000 "*" 2.62317811916546e-09 4.78656814015791e-09
70.3703703703704
"chr3" 73149001 73150000 "*" 5.80264323257751e-08 8.73025977059619e-08
-63.9344262295082
"chr3" 73483001 73484000 "*" 1.88737914186277e-15 7.62011598380321e-15 -100
"chr3" 73529001 73530000 "*" 7.0006535457523e-08 1.03695832512054e-07 100
"chr3" 73581001 73582000 "*" 1.17350573702879e-13 3.82772874833348e-13
-58.974358974359
"chr3" 73610001 73611000 "*" 1.99840144432528e-12 5.59476580335607e-12 -100
"chr3" 73674001 73675000 "*" 0 0 96.0591133004926
"chr3" 75034001 75035000 "*" 0 0 -63.2144227080936
"chr3" 75429001 75430000 "*" 1.9720003407997e-11 4.84222590452449e-11
-65.0153846153846
"chr3" 75494001 75495000 "*" 1.42212164266908e-09 2.69804222416496e-09
-58.3333333333333
"chr3" 75540001 75541000 "*" 0 0 -100
"chr3" 75820001 75821000 "*" 0 0 -100
"chr3" 75834001 75835000 "*" 0 0 94.6153846153846
"chr3" 76058001 76059000 "*" 1.66613648022729e-06 2.05048022219327e-06
-57.1428571428571
"chr3" 76063001 76064000 "*" 7.89449616789284e-11 1.78478309196764e-10
-88.6075949367089
"chr3" 76659001 76660000 "*" 0 0 -80
"chr3" 77088001 77089000 "*" 0 0 68.4659996176152
"chr3" 77147001 77148000 "*" 1.11022302462516e-16 5.03662826618488e-16
-56.9950738916256
"chr3" 77161001 77162000 "*" 0 0 -89.6103896103896
"chr3" 77265001 77266000 "*" 2.02188485953769e-08 3.23386403954282e-08
-62.7906976744186
"chr3" 77366001 77367000 "*" 1.11022302462516e-15 4.59817122935606e-15 -100
"chr3" 78491001 78492000 "*" 0 0 -100
"chr3" 78669001 78670000 "*" 1.20591092667155e-10 2.66075324129686e-10 -100
"chr3" 79068001 79069000 "*" 0 0 78.3687943262411
"chr3" 79549001 79550000 "*" 1.22402199487226e-10 2.69878621790792e-10
-70.2702702702703
"chr3" 82045001 82046000 "*" 1.14797060746241e-13 3.74810322436155e-13
70.5882352941177
"chr3" 82988001 82989000 "*" 1.85444070810448e-06 2.26726742717718e-06 60

"chr3" 84241001 84242000 "*" 8.88178419700125e-16 3.70670032207938e-15 100
"chr3" 85174001 85175000 "*" 0 0 100
"chr3" 85413001 85414000 "*" 0 0 -93.3333333333333
"chr3" 85734001 85735000 "*" 0 0 -100
"chr3" 87019001 87020000 "*" 9.0072393987839e-13 2.63157725646847e-12 -100
"chr3" 87039001 87040000 "*" 0 0 100
"chr3" 87437001 87438000 "*" 2.88779000712225e-11 6.92935809466068e-11 100
"chr3" 88018001 88019000 "*" 3.61932706027801e-14 1.25777907157994e-13 100
"chr3" 88108001 88109000 "*" 0 0 -78.2608695652174
"chr3" 89164001 89165000 "*" 2.1094237467878e-14 7.54243990081593e-14
-53.3980582524272
"chr3" 89619001 89620000 "*" 2.66891642597411e-06 3.18759115320439e-06
61.2903225806452
"chr3" 90227001 90228000 "*" 8.77756756167969e-11 1.97330112766911e-10
-57.6923076923077
"chr3" 94136001 94137000 "*" 4.65637306490407e-10 9.46720524046618e-10 -100
"chr3" 94970001 94971000 "*" 1.36604061395929e-11 3.42333952202875e-11 -100
"chr3" 95149001 95150000 "*" 8.23452417364479e-13 2.42236624914744e-12
-85.7142857142857
"chr3" 96386001 96387000 "*" 4.79616346638068e-14 1.64380445416596e-13 -100
"chr3" 96418001 96419000 "*" 3.39622219236446e-10 7.03784073980162e-10
-71.7391304347826
"chr3" 97359001 97360000 "*" 3.9190872769268e-14 1.35416418933905e-13 100
"chr3" 97965001 97966000 "*" 5.07358994716878e-06 5.81213600720194e-06
-70.6349206349206
"chr3" 98191001 98192000 "*" 1.92946192356658e-09 3.59193011611483e-09 100
"chr3" 98451001 98452000 "*" 0 0 56.4556823168056
"chr3" 98647001 98648000 "*" 2.16493489801906e-14 7.73462471679269e-14
97.7777777777778
"chr3" 98753001 98754000 "*" 7.68807417728112e-09 1.31238255467993e-08
54.1176470588235
"chr3" 98787001 98788000 "*" 3.63445940010365e-11 8.58565209111204e-11 100
"chr3" 99979001 99980000 "*" 0 0 100
"chr3" 100053001 100054000 "*" 1.11022302462516e-16 5.03662826618488e-16 100
"chr3" 100118001 100119000 "*" 2.70290456683142e-11 6.52665464305038e-11
85.0746268656716
"chr3" 100219001 100220000 "*" 1.87405646556726e-13 5.97285292681429e-13 100
"chr3" 100402001 100403000 "*" 1.68996273641753e-08 2.7398105072003e-08
83.0508474576271
"chr3" 101759001 101760000 "*" 0 0 100
"chr3" 102107001 102108000 "*" 2.68450373042128e-10 5.64465213127486e-10 100
"chr3" 102459001 102460000 "*" 6.44621932877065e-06 7.26708819733716e-06 -60
"chr3" 103017001 103018000 "*" 1.92946192356658e-09 3.59193011611483e-09 -100
"chr3" 103795001 103796000 "*" 0 0 -100
"chr3" 105573001 105574000 "*" 4.73234496034536e-09 8.30627300264826e-09 100
"chr3" 105751001 105752000 "*" 7.0006535457523e-08 1.03695832512054e-07 100
"chr3" 106152001 106153000 "*" 0 0 100
"chr3" 106363001 106364000 "*" 1.20281562487889e-12 3.46585342181251e-12 100
"chr3" 106606001 106607000 "*" 1.12055920098442e-11 2.84500040664577e-11 -100
"chr3" 106849001 106850000 "*" 1.12458486967171e-09 2.15740815043925e-09 -100
"chr3" 106851001 106852000 "*" 1.69864122767649e-14 6.13572966736024e-14
-62.0689655172414

"chr3" 107482001 107483000 "*" 1.5277158427196e-09 2.87642564232045e-09 100
"chr3" 108656001 108657000 "*" 4.86832796298131e-13 1.47290845675726e-12 -100
"chr3" 108950001 108951000 "*" 2.54365212812857e-05 2.61900915216325e-05
51.4285714285714
"chr3" 108953001 108954000 "*" 1.52794177310511e-09 2.87642564232045e-09 100
"chr3" 109035001 109036000 "*" 0 0 -81.4102564102564
"chr3" 109078001 109079000 "*" 2.88779000712225e-11 6.92935809466068e-11 -100
"chr3" 109119001 109120000 "*" 4.18887147191072e-13 1.2739946038722e-12 -100
"chr3" 109977001 109978000 "*" 1.11022302462516e-16 5.03662826618488e-16 -100
"chr3" 111578001 111579000 "*" 0 0 72.8695652173913
"chr3" 111717001 111718000 "*" 4.27540447489605e-10 8.73629089877964e-10
-52.7777777777778
"chr3" 112675001 112676000 "*" 5.48638023900594e-10 1.10374357336421e-09 -100
"chr3" 112925001 112926000 "*" 4.01313771103418e-09 7.11882087237587e-09 100
"chr3" 112930001 112931000 "*" 0 0 70.0121094587832
"chr3" 112942001 112943000 "*" 0 0 92.6101694915254
"chr3" 112970001 112971000 "*" 4.86753970463383e-11 1.13145908161915e-10 100
"chr3" 113036001 113037000 "*" 1.16408235084631e-06 1.46479698533027e-06 60
"chr3" 113251001 113252000 "*" 0 0 73.0909090909091
"chr3" 113950001 113951000 "*" 1.67299207820548e-08 2.71377429441055e-08 100
"chr3" 114062001 114063000 "*" 7.22200077518664e-13 2.13505334148473e-12
-60.7142857142857
"chr3" 114131001 114132000 "*" 7.0006535457523e-08 1.03695832512054e-07 100
"chr3" 114532001 114533000 "*" 0.000129328979378784 0.000119106074697934
53.8461538461538
"chr3" 114973001 114974000 "*" 0 0 100
"chr3" 114974001 114975000 "*" 8.01666433236647e-09 1.36392368970109e-08 100
"chr3" 115106001 115107000 "*" 2.16901478444775e-06 2.62630620055891e-06
-66.6666666666667
"chr3" 115394001 115395000 "*" 5.55111512312578e-16 2.36485094870365e-15
-79.3650793650794
"chr3" 115646001 115647000 "*" 4.44089209850063e-16 1.91071758245033e-15 100
"chr3" 115827001 115828000 "*" 0 0 -100
"chr3" 116036001 116037000 "*" 2.75837855657768e-05 2.82530803747995e-05
-66.6666666666667
"chr3" 116164001 116165000 "*" 5.08482145278322e-14 1.73432706170696e-13 -100
"chr3" 116583001 116584000 "*" 1.24984033345044e-07 1.79000832707579e-07
66.6666666666667
"chr3" 116785001 116786000 "*" 0 0 100
"chr3" 118898001 118899000 "*" 1.32452160350738e-10 2.90646525334638e-10
93.5483870967742
"chr3" 118917001 118918000 "*" 2.15125472990962e-10 4.58126659943874e-10 100
"chr3" 119013001 119014000 "*" 2.22044604925031e-16 9.81641919380259e-16
-64.1744548286604
"chr3" 119014001 119015000 "*" 0 0 -98.5294117647059
"chr3" 119041001 119042000 "*" 1.88493887165464e-10 4.05868937704982e-10
54.296875
"chr3" 119094001 119095000 "*" 0.000792090690089564 0.000639295093468792
52.3809523809524
"chr3" 119271001 119272000 "*" 0 0 100
"chr3" 119331001 119332000 "*" 6.92287338566189e-11 1.57605630124247e-10 -100
"chr3" 119364001 119365000 "*" 0 0 100

"chr3" 119392001 119393000 "*" 4.16020390836547e-06 4.82249302255461e-06
-68.8888888888889
"chr3" 120003001 120004000 "*" 0 0 65.4943022295624
"chr3" 120138001 120139000 "*" 7.62926906106021e-06 8.50700498478588e-06
62.8571428571429
"chr3" 120626001 120627000 "*" 6.89448498292222e-14 2.31502074572357e-13
60.5769230769231
"chr3" 120627001 120628000 "*" 0 0 81.7431324092977
"chr3" 120815001 120816000 "*" 2.68450373042128e-10 5.64465213127486e-10 -100
"chr3" 121345001 121346000 "*" 0 0 -100
"chr3" 121354001 121355000 "*" 1.08761799566537e-09 2.09366644836183e-09
-78.4313725490196
"chr3" 121450001 121451000 "*" 1.20591092667155e-10 2.66075324129686e-10 -100
"chr3" 121740001 121741000 "*" 0 0 80.3921568627451
"chr3" 121766001 121767000 "*" 1.55431223447522e-15 6.33705141101682e-15
80.327868852459
"chr3" 121795001 121796000 "*" 3.40125705378114e-10 7.03784073980162e-10 100
"chr3" 122283001 122284000 "*" 0 0 -65.9387860082305
"chr3" 122284001 122285000 "*" 1.50656154218609e-11 3.75866112196181e-11
74.5454545454545
"chr3" 122399001 122400000 "*" 0 0 -64.7887323943662
"chr3" 122630001 122631000 "*" 0 0 68.2491289198606
"chr3" 122634001 122635000 "*" 3.56775498033812e-10 7.35898629809829e-10 -100
"chr3" 122693001 122694000 "*" 0 0 100
"chr3" 122705001 122706000 "*" 1.52794177310511e-09 2.87642564232045e-09 -100
"chr3" 122732001 122733000 "*" 7.88814680241501e-10 1.55090157976543e-09
-79.1666666666667
"chr3" 122746001 122747000 "*" 0 0 87.2525732383215
"chr3" 122773001 122774000 "*" 0 0 100
"chr3" 122811001 122812000 "*" 2.64951482975562e-09 4.82186011375752e-09 100
"chr3" 122824001 122825000 "*" 9.65729496371637e-10 1.87255912493232e-09 -100
"chr3" 122921001 122922000 "*" 0 0 -100
"chr3" 123002001 123003000 "*" 8.55836845836677e-10 1.67587963267183e-09
57.1428571428571
"chr3" 123068001 123069000 "*" 0 0 -59.4897959183673
"chr3" 123069001 123070000 "*" 8.99330498960182e-09 1.5165043858425e-08
95.4545454545455
"chr3" 123070001 123071000 "*" 2.55351295663786e-15 1.01678090381833e-14 100
"chr3" 123108001 123109000 "*" 1.39779633423487e-07 1.98463037958589e-07 100
"chr3" 123121001 123122000 "*" 5.03264097062583e-13 1.51942428521737e-12 100
"chr3" 123132001 123133000 "*" 8.52140580320793e-12 2.19817259951489e-11 -100
"chr3" 123139001 123140000 "*" 4.18887147191072e-13 1.2739946038722e-12 -100
"chr3" 123162001 123163000 "*" 1.06670036137402e-08 1.78212246787701e-08
-59.0909090909091
"chr3" 123168001 123169000 "*" 0 0 62.6528268910295
"chr3" 123196001 123197000 "*" 1.07746053223945e-06 1.36177735814262e-06
-53.125
"chr3" 123351001 123352000 "*" 4.65637306490407e-10 9.46720524046618e-10 -100
"chr3" 123355001 123356000 "*" 1.34559030584569e-13 4.35581312463895e-13 -100
"chr3" 123411001 123412000 "*" 0 0 100
"chr3" 123445001 123446000 "*" 3.52704532247117e-11 8.35581292290093e-11 -100
"chr3" 123497001 123498000 "*" 4.01313771103418e-09 7.11882087237587e-09 -100

"chr3" 123506001 123507000 "*" 5.03883436420161e-07 6.65772560462085e-07
-64.3825301204819
"chr3" 123521001 123522000 "*" 8.02691246803988e-14 2.67352274101148e-13 100
"chr3" 123603001 123604000 "*" 0 0 81.183800623053
"chr3" 123699001 123700000 "*" 1.98365768255826e-11 4.85612402473442e-11 100
"chr3" 123705001 123706000 "*" 1.92946192356658e-09 3.59193011611483e-09 -100
"chr3" 124409001 124410000 "*" 7.0006535457523e-08 1.03695832512054e-07 -100
"chr3" 124480001 124481000 "*" 0 0 -100
"chr3" 124523001 124524000 "*" 8.53858317384493e-09 1.44808302221037e-08
52.3809523809524
"chr3" 124561001 124562000 "*" 2.4572282120161e-07 3.38544529513571e-07
78.5714285714286
"chr3" 124640001 124641000 "*" 1.11022302462516e-16 5.03662826618488e-16 -100
"chr3" 124717001 124718000 "*" 1.67299207820548e-08 2.71377429441055e-08 100
"chr3" 124786001 124787000 "*" 0 0 100
"chr3" 124883001 124884000 "*" 0.000201461795166558 0.000179722257706492 56
"chr3" 124905001 124906000 "*" 6.04438721296674e-11 1.38849255306602e-10 100
"chr3" 124931001 124932000 "*" 0 0 -71.1286953727506
"chr3" 125126001 125127000 "*" 3.76365605347928e-14 1.3043487970679e-13 100
"chr3" 125460001 125461000 "*" 2.3806321358677e-09 4.37326826783753e-09 -90
"chr3" 125648001 125649000 "*" 0 0 -86.0479797979798
"chr3" 125691001 125692000 "*" 0 0 100
"chr3" 125709001 125710000 "*" 0 0 55.4701426024955
"chr3" 125782001 125783000 "*" 1.49613654798486e-12 4.26244554429779e-12 100
"chr3" 125783001 125784000 "*" 5.6362137179633e-11 1.29963270300065e-10 100
"chr3" 125822001 125823000 "*" 4.59076887615595e-10 9.34931929815876e-10
80.6451612903226
"chr3" 125826001 125827000 "*" 0 0 51.0152284263959
"chr3" 125853001 125854000 "*" 0 0 58.0311161706511
"chr3" 125869001 125870000 "*" 1.50473010274155e-07 2.12852218185397e-07
74.4186046511628
"chr3" 125905001 125906000 "*" 0 0 100
"chr3" 125986001 125987000 "*" 0 0 53.7195842550226
"chr3" 126010001 126011000 "*" 1.15490073060442e-09 2.21266513204891e-09
83.5164835164835
"chr3" 126011001 126012000 "*" 1.88737914186277e-15 7.62011598380321e-15 100
"chr3" 126041001 126042000 "*" 3.17249519987683e-08 4.957901152449e-08 -80
"chr3" 126057001 126058000 "*" 5.33577904326954e-05 5.22413734060765e-05
-55.8139534883721
"chr3" 126061001 126062000 "*" 1.11022302462516e-16 5.03662826618488e-16
-59.5744680851064
"chr3" 126075001 126076000 "*" 0 0 -62.1479828740276
"chr3" 126091001 126092000 "*" 0 0 50.0380734818199
"chr3" 126093001 126094000 "*" 3.26817186646622e-08 5.10025469630698e-08
63.5653650254669
"chr3" 126176001 126177000 "*" 3.99680288865056e-14 1.37975970265307e-13 100
"chr3" 126199001 126200000 "*" 4.08209022140227e-11 9.57770690255255e-11 100
"chr3" 126206001 126207000 "*" 0 0 -54.9019607843137
"chr3" 126211001 126212000 "*" 0 0 72.7272727272727
"chr3" 126212001 126213000 "*" 1.11022302462516e-16 5.03662826618488e-16
85.4545454545455
"chr3" 126220001 126221000 "*" 1.35036426485158e-12 3.86629547363774e-12 -100

"chr3" 126224001 126225000 "*" 0 0 -100
"chr3" 126240001 126241000 "*" 2.95319324550292e-13 9.16834332047142e-13 -62.5
"chr3" 126245001 126246000 "*" 9.93649607039515e-14 3.26598367505166e-13
-60.6060606060606
"chr3" 126263001 126264000 "*" 6.88338275267597e-15 2.60518659957509e-14 100
"chr3" 126265001 126266000 "*" 3.34128893442198e-08 5.19772864618523e-08 100
"chr3" 126271001 126272000 "*" 1.2951651792048e-05 1.39512662107282e-05
62.7777777777778
"chr3" 126288001 126289000 "*" 0 0 -100
"chr3" 126296001 126297000 "*" 1.90520136289507e-06 2.32564618288801e-06 -52
"chr3" 126382001 126383000 "*" 4.97966215928791e-07 6.58402762862229e-07
-62.2641509433962
"chr3" 126396001 126397000 "*" 3.40125705378114e-10 7.03784073980162e-10 100
"chr3" 126465001 126466000 "*" 3.77475828372553e-15 1.47379557022071e-14 100
"chr3" 126590001 126591000 "*" 1.58067969713294e-07 2.22994335091428e-07
-55.8823529411765
"chr3" 126630001 126631000 "*" 0 0 -100
"chr3" 126642001 126643000 "*" 3.49848927605478e-10 7.23006353364999e-10
-51.8518518518519
"chr3" 126650001 126651000 "*" 1.13140401492018e-08 1.87801380787728e-08 100
"chr3" 126703001 126704000 "*" 0 0 -59.7723704866562
"chr3" 126706001 126707000 "*" 3.10529379987656e-13 9.61584922605664e-13
52.7386541471049
"chr3" 126756001 126757000 "*" 0 0 69.6629213483146
"chr3" 126761001 126762000 "*" 1.15387344123974e-09 2.21084932030456e-09
-55.8823529411765
"chr3" 126783001 126784000 "*" 5.9204381397393e-09 1.02769984435798e-08
71.3250517598344
"chr3" 126787001 126788000 "*" 0 0 62.1615573953905
"chr3" 126789001 126790000 "*" 0 0 100
"chr3" 126796001 126797000 "*" 0 0 100
"chr3" 126797001 126798000 "*" 1.64242106581725e-08 2.67500657345423e-08
79.6875
"chr3" 126800001 126801000 "*" 1.66533453693773e-15 6.7629186866784e-15 100
"chr3" 126804001 126805000 "*" 5.38204482847249e-06 6.14355131621693e-06
51.6483516483516
"chr3" 126811001 126812000 "*" 1.48487888651516e-11 3.70689457767456e-11 100
"chr3" 126825001 126826000 "*" 3.6700841921089e-08 5.65530226072258e-08 -100
"chr3" 126826001 126827000 "*" 2.64951482975562e-09 4.82186011375752e-09 100
"chr3" 126840001 126841000 "*" 1.13725894979044e-06 1.43296856092383e-06
72.2222222222222
"chr3" 126873001 126874000 "*" 3.6700841921089e-08 5.65530226072258e-08 100
"chr3" 126916001 126917000 "*" 1.33226762955019e-15 5.48133041560118e-15
-82.1428571428571
"chr3" 126978001 126979000 "*" 0 0 84.5070422535211
"chr3" 127003001 127004000 "*" 0 0 80.8080808080808
"chr3" 127058001 127059000 "*" 2.15125472990962e-10 4.58126659943874e-10 100
"chr3" 127107001 127108000 "*" 6.4152538836737e-10 1.27477697728417e-09 100
"chr3" 127126001 127127000 "*" 6.66133814775094e-16 2.81595744474255e-15 100
"chr3" 127141001 127142000 "*" 0 0 61.5794143744454
"chr3" 127142001 127143000 "*" 4.52706545917003e-07 6.01757031895544e-07
68.0851063829787

"chr3" 127162001 127163000 "*" 1.98124849859482e-10 4.25157611354621e-10 100
"chr3" 127165001 127166000 "*" 3.61932706027801e-14 1.25777907157994e-13 100
"chr3" 127180001 127181000 "*" 0 0 77.4085637823372
"chr3" 127185001 127186000 "*" 4.29589697148458e-12 1.1540320863616e-11
63.3333333333333
"chr3" 127190001 127191000 "*" 0 0 100
"chr3" 127199001 127200000 "*" 2.69007038866675e-13 8.38831022628993e-13 -100
"chr3" 127206001 127207000 "*" 1.55431223447522e-15 6.33705141101682e-15 100
"chr3" 127209001 127210000 "*" 9.04570973681018e-10 1.76414389822173e-09 -100
"chr3" 127242001 127243000 "*" 3.032263773739e-06 3.5910046598153e-06
68.3333333333333
"chr3" 127253001 127254000 "*" 3.3939517862791e-13 1.04766431437273e-12
76.3157894736842
"chr3" 127258001 127259000 "*" 0 0 -74.6165644171779
"chr3" 127261001 127262000 "*" 0 0 -100
"chr3" 127263001 127264000 "*" 0 0 -100
"chr3" 127291001 127292000 "*" 5.55111512312578e-16 2.36485094870365e-15 -100
"chr3" 127300001 127301000 "*" 0 0 -88.3408071748879
"chr3" 127407001 127408000 "*" 2.01805239186115e-12 5.64750607350457e-12
-52.1080139372822
"chr3" 127441001 127442000 "*" 0 0 -100
"chr3" 127458001 127459000 "*" 8.7349629751543e-09 1.47505871898511e-08 -100
"chr3" 127478001 127479000 "*" 0 0 100
"chr3" 127590001 127591000 "*" 1.74571238575894e-08 2.82503113648306e-08
-72.7272727272727
"chr3" 127708001 127709000 "*" 8.02698571500393e-07 1.03260151830174e-06
-65.3846153846154
"chr3" 127741001 127742000 "*" 1.91224813761437e-12 5.36527594469447e-12 100
"chr3" 127743001 127744000 "*" 9.04570973681018e-10 1.76414389822173e-09 100
"chr3" 127754001 127755000 "*" 7.03992419914812e-12 1.83313429961219e-11 100
"chr3" 127790001 127791000 "*" 2.91516766948163e-07 3.97688746901603e-07
-74.2857142857143
"chr3" 127896001 127897000 "*" 1.39779633423487e-07 1.98463037958589e-07 -100
"chr3" 127945001 127946000 "*" 2.74687161905263e-10 5.76953704674915e-10
68.2926829268293
"chr3" 128078001 128079000 "*" 1.29037891483108e-11 3.24582280286866e-11 -100
"chr3" 128136001 128137000 "*" 4.05897537802957e-13 1.23819083946783e-12
-59.825327510917
"chr3" 128158001 128159000 "*" 3.75178930145381e-08 5.77458831847156e-08
-61.5384615384615
"chr3" 128159001 128160000 "*" 7.43849426498855e-15 2.80357739563059e-14 -100
"chr3" 128187001 128188000 "*" 1.36535227568402e-12 3.90692827150239e-12
51.840354767184
"chr3" 128207001 128208000 "*" 0 0 91.7647058823529
"chr3" 128277001 128278000 "*" 4.24210222504939e-10 8.6801511858932e-10 53.125
"chr3" 128296001 128297000 "*" 5.24502663523663e-12 1.39147780323618e-11
54.5454545454545
"chr3" 128321001 128322000 "*" 5.0875319446142e-07 6.71813087869187e-07
77.7777777777778
"chr3" 128332001 128333000 "*" 1.29308440954823e-07 1.84867219207644e-07
-54.5454545454545
"chr3" 128566001 128567000 "*" 6.88338275267597e-15 2.60518659957509e-14

-57.5757575757576
"chr3" 128580001 128581000 "*" 8.24230983198504e-06 9.14418873206944e-06
68.5185185185185
"chr3" 128713001 128714000 "*" 0 0 100
"chr3" 128728001 128729000 "*" 3.02532772167297e-08 4.74034684418066e-08 81.25
"chr3" 128729001 128730000 "*" 8.7349629751543e-09 1.47505871898511e-08 100
"chr3" 128758001 128759000 "*" 1.11022302462516e-16 5.03662826618488e-16
86.6666666666667
"chr3" 128838001 128839000 "*" 0 0 -100
"chr3" 129060001 129061000 "*" 5.7065463465733e-14 1.93318145576218e-13 -100
"chr3" 129081001 129082000 "*" 0 0 100
"chr3" 129144001 129145000 "*" 3.04324343503026e-11 7.26599935673121e-11 100
"chr3" 129236001 129237000 "*" 2.93692590636052e-06 3.48641648244637e-06
65.7894736842105
"chr3" 129241001 129242000 "*" 3.5527136788005e-15 1.39277426410519e-14 100
"chr3" 129253001 129254000 "*" 3.88022947106492e-13 1.1867462770515e-12 -100
"chr3" 129331001 129332000 "*" 4.95803398337102e-12 1.32088640732338e-11
54.5454545454545
"chr3" 129345001 129346000 "*" 0 0 -85.7142857142857
"chr3" 129634001 129635000 "*" 2.59712167327564e-08 4.09881271012004e-08
66.6666666666667
"chr3" 129639001 129640000 "*" 3.33066907387547e-16 1.4495649018245e-15
57.4074074074074
"chr3" 129659001 129660000 "*" 1.54630752646767e-11 3.85079502648228e-11 100
"chr3" 129661001 129662000 "*" 3.81746082744883e-07 5.12357408535159e-07
-53.3333333333333
"chr3" 129692001 129693000 "*" 1.28299625146155e-07 1.8349100845649e-07
51.3533834586466
"chr3" 129712001 129713000 "*" 1.12376774552558e-12 3.24761818527664e-12 100
"chr3" 129821001 129822000 "*" 2.42228059832428e-09 4.44537111137461e-09
-72.7272727272727
"chr3" 129842001 129843000 "*" 1.67299207820548e-08 2.71377429441055e-08 100
"chr3" 129902001 129903000 "*" 0 0 -100
"chr3" 130170001 130171000 "*" 2.64951482975562e-09 4.82186011375752e-09 -100
"chr3" 130266001 130267000 "*" 0 0 68.0824639841033
"chr3" 130380001 130381000 "*" 2.48184182993771e-06 2.97686444206053e-06 90
"chr3" 130534001 130535000 "*" 1.11022302462516e-16 5.03662826618488e-16
-63.8095238095238
"chr3" 132120001 132121000 "*" 4.60438853711054e-09 8.11429966472216e-09
-86.1702127659574
"chr3" 132162001 132163000 "*" 0 0 100
"chr3" 133104001 133105000 "*" 3.29514193708746e-13 1.01810519085382e-12
64.2857142857143
"chr3" 133472001 133473000 "*" 0.000184006388664448 0.000165249884973406
54.1666666666667
"chr3" 133476001 133477000 "*" 0.000340738326746304 0.000292680508109892
57.5757575757576
"chr3" 133478001 133479000 "*" 1.17794662912729e-13 3.83653245040640e-13 -100
"chr3" 133546001 133547000 "*" 1.5277158427196e-09 2.87642564232045e-09 -100
"chr3" 133558001 133559000 "*" 1.22124532708767e-15 5.03921984217778e-15 100
"chr3" 133560001 133561000 "*" 0 0 -68.6928104575163
"chr3" 133577001 133578000 "*" 4.0365252444996e-06 4.6892927905593e-06

54.5454545454545
"chr3" 133587001 133588000 "*" 7.0006535457523e-08 1.03695832512054e-07 -100
"chr3" 133606001 133607000 "*" 1.55431223447522e-14 5.6388152448458e-14 100
"chr3" 133615001 133616000 "*" 0 0 79.00399543379
"chr3" 133645001 133646000 "*" 8.96470120359538e-10 1.7517861492618e-09
50.7692307692308
"chr3" 133654001 133655000 "*" 4.44089209850063e-16 1.91071758245033e-15
54.5454545454545
"chr3" 133672001 133673000 "*" 1.16573417585641e-14 4.29335582900508e-14 100
"chr3" 133689001 133690000 "*" 6.11406060668784e-07 7.99681626961299e-07
68.5185185185185
"chr3" 133718001 133719000 "*" 3.12976220495642e-08 4.8939620585343e-08
-78.6885245901639
"chr3" 133788001 133789000 "*" 8.29336599394992e-14 2.75830476998008e-13
-95.6521739130435
"chr3" 133790001 133791000 "*" 2.44756068935592e-05 2.52637134962309e-05
-64.5161290322581
"chr3" 133920001 133921000 "*" 4.73234496034536e-09 8.30627300264826e-09 -100
"chr3" 134042001 134043000 "*" 3.05311331771918e-12 8.34639368772922e-12 100
"chr3" 134073001 134074000 "*" 7.76974595773083e-10 1.52892015230691e-09 60
"chr3" 134094001 134095000 "*" 1.19043664925655e-08 1.97111834928772e-08
-54.5454545454545
"chr3" 134362001 134363000 "*" 2.15125472990962e-10 4.58126659943874e-10 -100
"chr3" 134363001 134364000 "*" 3.04324343503026e-11 7.26599935673121e-11 100
"chr3" 134378001 134379000 "*" 0 0 100
"chr3" 134418001 134419000 "*" 5.1281201507436e-13 1.54461625452838e-12 100
"chr3" 134830001 134831000 "*" 2.73625566649116e-12 7.52483363579137e-12 -100
"chr3" 134968001 134969000 "*" 8.10964029263062e-09 1.37881408088721e-08
-77.2727272727273
"chr3" 135460001 135461000 "*" 1.39779633423487e-07 1.98463037958589e-07 100
"chr3" 135532001 135533000 "*" 5.6362137179633e-11 1.29963270300065e-10 -100
"chr3" 135870001 135871000 "*" 2.02060590481778e-13 6.40127405465654e-13 100
"chr3" 135872001 135873000 "*" 3.6700841921089e-08 5.65530226072258e-08 100
"chr3" 136537001 136538000 "*" 0 0 56.9899497487437
"chr3" 136539001 136540000 "*" 0 0 80.3571428571429
"chr3" 137681001 137682000 "*" 1.98365768255826e-11 4.85612402473442e-11 -100
"chr3" 137690001 137691000 "*" 1.35036426485158e-12 3.86629547363774e-12 -100
"chr3" 137770001 137771000 "*" 7.7715611723761e-16 3.26213507634405e-15
-95.4545454545455
"chr3" 137851001 137852000 "*" 2.00227675550835e-08 3.20459176617961e-08 100
"chr3" 137944001 137945000 "*" 2.15125472990962e-10 4.58126659943874e-10 -100
"chr3" 138059001 138060000 "*" 1.46549439250521e-14 5.33330076973935e-14 -100
"chr3" 138068001 138069000 "*" 1.48487888651516e-11 3.70689457767456e-11 -100
"chr3" 138069001 138070000 "*" 7.68311851473413e-07 9.90964461140931e-07
-67.741935483871
"chr3" 138080001 138081000 "*" 1.75796345258661e-08 2.84391855516502e-08
-54.3071161048689
"chr3" 138108001 138109000 "*" 1.33231203847117e-11 3.34696082983262e-11 75
"chr3" 138129001 138130000 "*" 7.1291469727619e-07 9.23469497775327e-07
-56.5217391304348
"chr3" 138153001 138154000 "*" 0 0 53.8012804533085
"chr3" 138327001 138328000 "*" 0 0 -80.0433839479393

"chr3" 138601001 138602000 "*" 4.27954982562539e-09 7.56742156628225e-09 -90.9090909090909
"chr3" 138665001 138666000 "*" 0 0 65.2989058196782
"chr3" 138666001 138667000 "*" 0 0 56.7379353600945
"chr3" 138682001 138683000 "*" 1.35890187991095e-11 3.40841971363279e-11 100
"chr3" 138708001 138709000 "*" 3.05391568145019e-07 4.15441232105128e-07 66.6666666666667
"chr3" 138741001 138742000 "*" 1.04471986617227e-13 3.42749557361457e-13 52.1739130434783
"chr3" 138842001 138843000 "*" 4.26951363152739e-10 8.72510873516957e-10 100
"chr3" 139021001 139022000 "*" 3.63445940010365e-11 8.58565209111204e-11 -100
"chr3" 139477001 139478000 "*" 9.04570973681018e-10 1.76414389822173e-09 100
"chr3" 139481001 139482000 "*" 0 0 -100
"chr3" 139498001 139499000 "*" 1.20591092667155e-10 2.66075324129686e-10 -100
"chr3" 139654001 139655000 "*" 0 0 90.2501687876009
"chr3" 139655001 139656000 "*" 0 0 75
"chr3" 139683001 139684000 "*" 9.65729496371637e-10 1.87255912493232e-09 -100
"chr3" 139801001 139802000 "*" 9.61897228535236e-13 2.801372325071e-12 -100
"chr3" 140166001 140167000 "*" 2.04271792814126e-08 3.26480585104971e-08 -80.7692307692308
"chr3" 140474001 140475000 "*" 3.95353005888666e-09 7.03928529283733e-09 100
"chr3" 140652001 140653000 "*" 2.22044604925031e-16 9.81641919380259e-16 -100
"chr3" 140809001 140810000 "*" 0 0 -73.6842105263158
"chr3" 140998001 140999000 "*" 9.31334537819239e-05 8.77800738206373e-05 -51.7241379310345
"chr3" 141020001 141021000 "*" 1.29380277602564e-08 2.13388283390091e-08 53.8461538461538
"chr3" 141027001 141028000 "*" 1.05051199961181e-08 1.75687166260914e-08 53.3333333333333
"chr3" 141378001 141379000 "*" 4.02885502737149e-10 8.26306603562486e-10 57.0815450643777
"chr3" 141379001 141380000 "*" 0 0 57.2491441131589
"chr3" 141515001 141516000 "*" 1.84048428297778e-05 1.93665920541667e-05 59.2592592592593
"chr3" 141546001 141547000 "*" 0.000233814694374557 0.000206450780504946 -51.063829787234
"chr3" 141868001 141869000 "*" 0 0 57.7481767223193
"chr3" 142169001 142170000 "*" 1.11022302462516e-16 5.03662826618488e-16 100
"chr3" 142419001 142420000 "*" 1.15766174424436e-10 2.56161408095129e-10 -88.2352941176471
"chr3" 142443001 142444000 "*" 0 0 82.3404255319149
"chr3" 142530001 142531000 "*" 3.95949939502316e-12 1.06865476585232e-11 84.3243243243243
"chr3" 142848001 142849000 "*" 1.15339349182619e-09 2.21004254568532e-09 -95
"chr3" 142896001 142897000 "*" 4.14335232790108e-13 1.26153063645578e-12 100
"chr3" 142905001 142906000 "*" 3.63445940010365e-11 8.58565209111204e-11 -100
"chr3" 143153001 143154000 "*" 0 0 72.2222222222222
"chr3" 143303001 143304000 "*" 3.77475828372553e-15 1.47379557022071e-14 -100
"chr3" 145145001 145146000 "*" 0 0 70.9415584415584
"chr3" 145597001 145598000 "*" 2.64951482975562e-09 4.82186011375752e-09 100
"chr3" 145878001 145879000 "*" 0 0 62.2338493819522
"chr3" 145879001 145880000 "*" 1.58617563528196e-12 4.49485758210279e-12

56.3492063492063
"chr3" 145969001 145970000 "*" 4.89603562592311e-06 5.61954204465069e-06
73.5294117647059
"chr3" 146127001 146128000 "*" 0 0 -98.1481481481482
"chr3" 146688001 146689000 "*" 1.00050873408364e-06 1.26940594284362e-06
-61.5384615384615
"chr3" 147079001 147080000 "*" 5.55111512312578e-16 2.36485094870365e-15
-81.8181818181818
"chr3" 147145001 147146000 "*" 7.0006535457523e-08 1.03695832512054e-07 -100
"chr3" 148685001 148686000 "*" 1.39779633423487e-07 1.98463037958589e-07 -100
"chr3" 148803001 148804000 "*" 0 0 75.2293577981651
"chr3" 149139001 149140000 "*" 4.44089209850063e-15 1.71914916534614e-14 -100
"chr3" 149688001 149689000 "*" 0 0 55.026455026455
"chr3" 149785001 149786000 "*" 2.37587727269783e-14 8.44268514944447e-14 100
"chr3" 149884001 149885000 "*" 4.44089209850063e-16 1.91071758245033e-15 -100
"chr3" 149973001 149974000 "*" 1.56863411149288e-12 4.4469621057462e-12 100
"chr3" 150358001 150359000 "*" 0 0 100
"chr3" 150572001 150573000 "*" 1.37828859436695e-10 3.01445544267661e-10 100
"chr3" 150636001 150637000 "*" 5.08482145278322e-14 1.73432706170696e-13 100
"chr3" 150952001 150953000 "*" 4.14335232790108e-13 1.26153063645578e-12 -100
"chr3" 151178001 151179000 "*" 0 0 -73.2683982683983
"chr3" 151346001 151347000 "*" 1.90181204118289e-13 6.05298617539364e-13 100
"chr3" 151439001 151440000 "*" 0 0 -100
"chr3" 151632001 151633000 "*" 3.74406028402774e-09 6.69964892688968e-09
81.8713450292398
"chr3" 151718001 151719000 "*" 0 0 -100
"chr3" 151836001 151837000 "*" 3.6700841921089e-08 5.65530226072258e-08 100
"chr3" 152017001 152018000 "*" 2.73625566649116e-12 7.52483363579137e-12 100
"chr3" 152382001 152383000 "*" 1.12458486967171e-09 2.15740815043925e-09 -100
"chr3" 152545001 152546000 "*" 2.16382467499443e-13 6.82775854166559e-13 100
"chr3" 154437001 154438000 "*" 9.61415627542817e-06 1.05670450583329e-05
-66.6666666666667
"chr3" 154657001 154658000 "*" 5.51780843238703e-14 1.87243792572872e-13 100
"chr3" 154739001 154740000 "*" 9.43689570931383e-15 3.51625644188498e-14 -100
"chr3" 154850001 154851000 "*" 2.14353390592947e-09 3.95978075470562e-09
96.1538461538462
"chr3" 155693001 155694000 "*" 0.000412523424090483 0.000349386048469569 -52
"chr3" 156007001 156008000 "*" 9.62896429257398e-13 2.80291205576417e-12 -100
"chr3" 156125001 156126000 "*" 0.000726374117344109 0.000590081342527627
-52.1739130434783
"chr3" 156177001 156178000 "*" 5.62883073484954e-13 1.68905058935478e-12 -100
"chr3" 156323001 156324000 "*" 0.000135810753319365 0.000124644681570312
57.1428571428571
"chr3" 156544001 156545000 "*" 0 0 -78.3969289210021
"chr3" 156801001 156802000 "*" 8.7349629751543e-09 1.47505871898511e-08 100
"chr3" 156817001 156818000 "*" 2.80422884912923e-09 5.08976366659213e-09
60.7142857142857
"chr3" 157217001 157218000 "*" 0 0 -57.2512271307452
"chr3" 157331001 157332000 "*" 2.73014943985572e-11 6.58595916221891e-11 100
"chr3" 157388001 157389000 "*" 6.59550958292954e-09 1.13456194382773e-08 100
"chr3" 157426001 157427000 "*" 3.6700841921089e-08 5.65530226072258e-08 -100
"chr3" 157501001 157502000 "*" 0 0 -61.9047619047619

"chr3" 157811001 157812000 "*" 1.9373391779709e-13 6.15971322898373e-13 100
"chr3" 157815001 157816000 "*" 0 0 79.11227154047
"chr3" 157816001 157817000 "*" 0 0 90.625
"chr3" 157823001 157824000 "*" 0 0 53.7103986300067
"chr3" 158441001 158442000 "*" 2.22044604925031e-16 9.81641919380259e-16 100
"chr3" 159414001 159415000 "*" 2.64951482975562e-09 4.82186011375752e-09 100
"chr3" 159450001 159451000 "*" 3.6700841921089e-08 5.65530226072258e-08 100
"chr3" 159706001 159707000 "*" 0 0 56.9593147751606
"chr3" 159707001 159708000 "*" 0 0 100
"chr3" 159789001 159790000 "*" 0 0 100
"chr3" 159943001 159944000 "*" 0 0 51.9336763663027
"chr3" 160733001 160734000 "*" 3.95353005888666e-09 7.03928529283733e-09 -100
"chr3" 160818001 160819000 "*" 0 0 -100
"chr3" 161250001 161251000 "*" 1.35447209004269e-14 4.94674708073053e-14 100
"chr3" 161418001 161419000 "*" 1.12458486967171e-09 2.15740815043925e-09 100
"chr3" 163671001 163672000 "*" 5.31008570447966e-12 1.40811054886149e-11 53.125
"chr3" 163721001 163722000 "*" 0 0 -62.0859213250518
"chr3" 163742001 163743000 "*" 0 0 75.077519379845
"chr3" 164645001 164646000 "*" 2.73014943985572e-11 6.58595916221891e-11 100
"chr3" 165430001 165431000 "*" 1.26078925077877e-10 2.77325409449444e-10
82.8125
"chr3" 166142001 166143000 "*" 1.53911390610162e-06 1.9046291594191e-06
-72.7272727272727
"chr3" 167141001 167142000 "*" 1.5277158427196e-09 2.87642564232045e-09 100
"chr3" 167582001 167583000 "*" 1.67299207820548e-08 2.71377429441055e-08 -100
"chr3" 167967001 167968000 "*" 0 0 71.7148552665193
"chr3" 167968001 167969000 "*" 0 0 93.6507936507936
"chr3" 167974001 167975000 "*" 1.39779633423487e-07 1.98463037958589e-07 -100
"chr3" 168838001 168839000 "*" 5.26393684785464e-06 6.01894736582001e-06
-51.0489510489511
"chr3" 168867001 168868000 "*" 2.65276689503935e-12 7.32421650426789e-12 -60
"chr3" 169156001 169157000 "*" 2.00227675550835e-08 3.20459176617961e-08 -100
"chr3" 169418001 169419000 "*" 3.31491103455761e-07 4.48888447535355e-07
-68.4210526315789
"chr3" 169487001 169488000 "*" 0 0 -68.6154345006485
"chr3" 169558001 169559000 "*" 1.67299207820548e-08 2.71377429441055e-08 -100
"chr3" 169756001 169757000 "*" 6.63136212608606e-11 1.51556566692433e-10
73.469387755102
"chr3" 170136001 170137000 "*" 0 0 51.1194199245669
"chr3" 170143001 170144000 "*" 2.68450373042128e-10 5.64465213127486e-10 100
"chr3" 170261001 170262000 "*" 0.000126887446533108 0.000117029410540844
54.5454545454545
"chr3" 170488001 170489000 "*" 6.24654217240561e-10 1.2462108191303e-09 -100
"chr3" 170538001 170539000 "*" 5.10480546722647e-13 1.53881640171109e-12 -80
"chr3" 170664001 170665000 "*" 2.06926697998711e-11 5.05664708451183e-11 80
"chr3" 171281001 171282000 "*" 2.33028818463765e-10 4.93719283454501e-10 100
"chr3" 171394001 171395000 "*" 1.10442103402608e-05 1.20217549185931e-05
-59.2592592592593
"chr3" 171558001 171559000 "*" 2.16382467499443e-13 6.82775854166559e-13 -100
"chr3" 172049001 172050000 "*" 8.3882012447134e-11 1.88922989503423e-10 100
"chr3" 172074001 172075000 "*" 4.01313771103418e-09 7.11882087237587e-09 100
"chr3" 172279001 172280000 "*" 6.38756773585492e-07 8.33076784507971e-07

-54.5454545454545
"chr3" 172332001 172333000 "*" 3.95861121660346e-12 1.0684496262032e-11 -100
"chr3" 172452001 172453000 "*" 0 0 100
"chr3" 172997001 172998000 "*" 4.73234496034536e-09 8.30627300264826e-09 100
"chr3" 173023001 173024000 "*" 0 0 -100
"chr3" 173109001 173110000 "*" 8.12127827920506e-06 9.01867152379192e-06
66.6666666666667
"chr3" 173113001 173114000 "*" 0 0 86.8372803666921
"chr3" 173237001 173238000 "*" 7.0006535457523e-08 1.03695832512054e-07 -100
"chr3" 173930001 173931000 "*" 1.55431223447522e-15 6.33705141101682e-15
-75.2551020408163
"chr3" 174453001 174454000 "*" 2.00227675550835e-08 3.20459176617961e-08 100
"chr3" 174587001 174588000 "*" 0 0 -66.6666666666667
"chr3" 174906001 174907000 "*" 5.48638023900594e-10 1.10374357336421e-09 100
"chr3" 175548001 175549000 "*" 6.59550958292954e-09 1.13456194382773e-08 100
"chr3" 175786001 175787000 "*" 2.4535928844216e-14 8.69144396197119e-14 -100
"chr3" 175987001 175988000 "*" 1.32615242542933e-06 1.65582211629877e-06
57.1428571428571
"chr3" 177032001 177033000 "*" 1.33226762955019e-15 5.48133041560118e-15
66.6666666666667
"chr3" 177046001 177047000 "*" 2.08814465718632e-09 3.86232567960463e-09 100
"chr3" 179041001 179042000 "*" 1.77635683940025e-15 7.19870740878856e-15
68.0555555555556
"chr3" 179212001 179213000 "*" 2.20793159977362e-07 3.0595150827012e-07
57.8787878787879
"chr3" 179921001 179922000 "*" 1.77635683940025e-15 7.19870740878856e-15 100
"chr3" 179960001 179961000 "*" 1.9023893571557e-11 4.6891062269334e-11
85.7142857142857
"chr3" 180320001 180321000 "*" 0 0 75.9008835958509
"chr3" 180586001 180587000 "*" 1.11022302462516e-16 5.03662826618488e-16 100
"chr3" 181194001 181195000 "*" 4.08209022140227e-11 9.57770690255255e-11 100
"chr3" 181419001 181420000 "*" 3.26405569239796e-14 1.13973003849234e-13 100
"chr3" 181420001 181421000 "*" 1.52100554373646e-13 4.89194101432238e-13
95.4545454545455
"chr3" 181429001 181430000 "*" 1.26369971154006e-07 1.80846008275585e-07
-60.5263157894737
"chr3" 181508001 181509000 "*" 0 0 100
"chr3" 181596001 181597000 "*" 3.63445940010365e-11 8.58565209111204e-11 100
"chr3" 182091001 182092000 "*" 5.99520433297585e-15 2.28531569014092e-14
98.5507246376812
"chr3" 182835001 182836000 "*" 2.79987144580218e-12 7.68634726811898e-12 100
"chr3" 182879001 182880000 "*" 6.67140531707133e-08 9.95366380813771e-08
-70.5882352941177
"chr3" 182977001 182978000 "*" 2.33028818463765e-10 4.93719283454501e-10 -100
"chr3" 182983001 182984000 "*" 6.44706510399828e-13 1.91553161055386e-12
81.4814814814815
"chr3" 183020001 183021000 "*" 0 0 -71.4285714285714
"chr3" 183146001 183147000 "*" 0 0 53.9775543439779
"chr3" 183258001 183259000 "*" 2.77275786553233e-07 3.79440378232185e-07
-52.0127118644068
"chr3" 183496001 183497000 "*" 6.22911122682979e-09 1.07818408147802e-08
84.9557522123894

"chr3" 183546001 183547000 "*" 4.44089209850063e-16 1.91071758245033e-15 -100
"chr3" 183624001 183625000 "*" 1.67299207820548e-08 2.71377429441055e-08 100
"chr3" 183626001 183627000 "*" 9.04570973681018e-10 1.76414389822173e-09 -100
"chr3" 183628001 183629000 "*" 5.55111512312578e-16 2.36485094870365e-15 100
"chr3" 183635001 183636000 "*" 0.000126887439664602 0.000117029410540844
-56.5217391304348
"chr3" 183736001 183737000 "*" 2.82773804372027e-13 8.79129664109438e-13 -100
"chr3" 183755001 183756000 "*" 5.7835307716303e-06 6.56902676498958e-06
-68.2926829268293
"chr3" 183775001 183776000 "*" 3.42845474810005e-06 4.02641014303533e-06
-68.5714285714286
"chr3" 183777001 183778000 "*" 1.83952852950142e-12 5.17499701329218e-12 -100
"chr3" 183804001 183805000 "*" 2.38871094593307e-06 2.87375451142406e-06 -62
"chr3" 183872001 183873000 "*" 0 0 100
"chr3" 183946001 183947000 "*" 4.01313771103418e-09 7.11882087237587e-09 -100
"chr3" 183978001 183979000 "*" 0 0 58.3481956985105
"chr3" 183999001 184000000 "*" 3.6700841921089e-08 5.65530226072258e-08 -100
"chr3" 184004001 184005000 "*" 1.44609524355133e-09 2.74090612237498e-09
73.3333333333333
"chr3" 184059001 184060000 "*" 1.12458486967171e-09 2.15740815043925e-09 -100
"chr3" 184103001 184104000 "*" 1.0769163338864e-14 3.98631098593833e-14
64.9122807017544
"chr3" 184105001 184106000 "*" 2.37587727269783e-14 8.44268514944447e-14
82.8947368421053
"chr3" 184127001 184128000 "*" 6.55420939743578e-10 1.30087013762846e-09
52.6315789473684
"chr3" 184183001 184184000 "*" 4.25122695935087e-08 6.50500488737231e-08
58.3333333333333
"chr3" 184244001 184245000 "*" 1.38590361409285e-10 3.02706178892116e-10 -100
"chr3" 184278001 184279000 "*" 0 0 -100
"chr3" 184282001 184283000 "*" 8.69011083648452e-07 1.11229659818976e-06
66.6666666666667
"chr3" 184283001 184284000 "*" 0 0 -85.8695652173913
"chr3" 184285001 184286000 "*" 0.000131232110224921 0.000120712161400679 -52
"chr3" 184286001 184287000 "*" 0 0 -50.9481915933529
"chr3" 184313001 184314000 "*" 1.13140401492018e-08 1.87801380787728e-08 -100
"chr3" 184324001 184325000 "*" 8.44866073321793e-05 8.01219179790309e-05
62.962962962963
"chr3" 184328001 184329000 "*" 0 0 -100
"chr3" 184336001 184337000 "*" 2.15125472990962e-10 4.58126659943874e-10 100
"chr3" 184337001 184338000 "*" 6.59550958292954e-09 1.13456194382773e-08 -100
"chr3" 184364001 184365000 "*" 1.3172503088299e-09 2.50792361237215e-09 -75
"chr3" 184374001 184375000 "*" 1.04604103157158e-11 2.67011144039329e-11
97.8723404255319
"chr3" 184397001 184398000 "*" 5.7654991891809e-12 1.52378834692581e-11
-53.8736263736264
"chr3" 184401001 184402000 "*" 9.65419955090141e-09 1.62165308096456e-08 -72
"chr3" 184414001 184415000 "*" 2.69007038866675e-13 8.38831022628993e-13 -100
"chr3" 184456001 184457000 "*" 2.44249065417534e-15 9.75180348030994e-15 100
"chr3" 184488001 184489000 "*" 0 0 -67.2238007688451
"chr3" 184583001 184584000 "*" 5.48638023900594e-10 1.10374357336421e-09 100
"chr3" 184870001 184871000 "*" 0 0 66.0956495528986

"chr3" 185069001 185070000 "*" 4.78841410966879e-11 1.11468187156588e-10 100
"chr3" 185232001 185233000 "*" 7.54951656745106e-14 2.5237892556906e-13 -100
"chr3" 185245001 185246000 "*" 5.1281201507436e-13 1.54461625452838e-12 100
"chr3" 185271001 185272000 "*" 0 0 -60.9022556390977
"chr3" 185555001 185556000 "*" 9.32032229172819e-13 2.7181920310254e-12 -100
"chr3" 185597001 185598000 "*" 1.91224813761437e-12 5.36527594469447e-12 100
"chr3" 185784001 185785000 "*" 1.12458486967171e-09 2.15740815043925e-09 100
"chr3" 185828001 185829000 "*" 5.35349542474250e-12 1.41908034722565e-11
-71.8562874251497
"chr3" 185869001 185870000 "*" 7.0006535457523e-08 1.03695832512054e-07 100
"chr3" 185880001 185881000 "*" 9.80763359414993e-10 1.8980032424715e-09 100
"chr3" 185906001 185907000 "*" 1.12055920098442e-11 2.84500040664577e-11 100
"chr3" 185926001 185927000 "*" 1.02897014242487e-08 1.72267598842224e-08
54.320987654321
"chr3" 185941001 185942000 "*" 9.65729496371637e-10 1.87255912493232e-09 100
"chr3" 185966001 185967000 "*" 3.1696723024055e-10 6.59431448778094e-10
-72.2222222222222
"chr3" 185969001 185970000 "*" 1.93720595120794e-11 4.76309924836636e-11 100
"chr3" 185978001 185979000 "*" 1.13140401492018e-08 1.87801380787728e-08 100
"chr3" 186101001 186102000 "*" 6.05182570723173e-13 1.80827773689544e-12
-51.1111111111111
"chr3" 186122001 186123000 "*" 2.15125472990962e-10 4.58126659943874e-10 100
"chr3" 186127001 186128000 "*" 4.95927881046798e-05 4.88061303458408e-05
-66.6666666666667
"chr3" 186131001 186132000 "*" 9.63829016598083e-12 2.46993429847588e-11 100
"chr3" 186133001 186134000 "*" 0 0 100
"chr3" 186157001 186158000 "*" 2.08814465718632e-09 3.86232567960463e-09 100
"chr3" 186170001 186171000 "*" 1.18738976429e-08 1.96630728742272e-08
-65.3846153846154
"chr3" 186335001 186336000 "*" 0 0 -66.5487308182828
"chr3" 186451001 186452000 "*" 3.6700841921089e-08 5.65530226072258e-08 -100
"chr3" 186648001 186649000 "*" 0 0 85.1224105461394
"chr3" 186656001 186657000 "*" 4.22135256750167e-08 6.46213810841317e-08
-92.3076923076923
"chr3" 186915001 186916000 "*" 2.33982833108826e-09 4.30200663260379e-09 60
"chr3" 186941001 186942000 "*" 3.52704532247117e-11 8.35581292290093e-11 100
"chr3" 186944001 186945000 "*" 0 0 75
"chr3" 186965001 186966000 "*" 3.68371999570627e-13 1.13149976091305e-12 100
"chr3" 186966001 186967000 "*" 0 0 100
"chr3" 186993001 186994000 "*" 2.7739215369138e-09 5.03716664165354e-09
-93.3333333333333
"chr3" 187043001 187044000 "*" 3.95353005888666e-09 7.03928529283733e-09 -100
"chr3" 187398001 187399000 "*" 3.95353005888666e-09 7.03928529283733e-09 -100
"chr3" 187457001 187458000 "*" 1.74131487007401e-10 3.76196440285642e-10
-87.5335120643432
"chr3" 187513001 187514000 "*" 1.55431223447522e-14 5.6388152448458e-14 100
"chr3" 187526001 187527000 "*" 1.90181204118289e-13 6.05298617539364e-13 100
"chr3" 188299001 188300000 "*" 5.88418203051333e-15 2.245355428447e-14 100
"chr3" 188640001 188641000 "*" 5.6362137179633e-11 1.29963270300065e-10 100
"chr3" 189272001 189273000 "*" 1.38590361409285e-10 3.02706178892116e-10 -100
"chr3" 189732001 189733000 "*" 9.08108033215171e-11 2.03407753381119e-10 100
"chr3" 189985001 189986000 "*" 9.0072393987839e-13 2.63157725646847e-12 -100

"chr3" 190132001 190133000 "*" 1.98365768255826e-11 4.85612402473442e-11 100
"chr3" 190342001 190343000 "*" 0 0 62.6876276756373
"chr3" 190471001 190472000 "*" 0 0 100
"chr3" 190755001 190756000 "*" 0 0 100
"chr3" 190854001 190855000 "*" 1.57004726518695e-05 1.66995748290365e-05 56.0509554140127
"chr3" 190959001 190960000 "*" 4.56820693495175e-08 6.96765988070186e-08 78.494623655914
"chr3" 191141001 191142000 "*" 2.18158824338843e-13 6.87314823207509e-13 100
"chr3" 191350001 191351000 "*" 4.77395900588817e-15 1.84201484337652e-14 100
"chr3" 191426001 191427000 "*" 3.5527136788005e-15 1.39277426410519e-14 -100
"chr3" 192183001 192184000 "*" 0 0 100
"chr3" 192289001 192290000 "*" 0 0 82.7309236947791
"chr3" 192444001 192445000 "*" 2.02718650754719e-08 3.24159766118952e-08 -93.3333333333333
"chr3" 192445001 192446000 "*" 0 0 52.62492474413
"chr3" 192463001 192464000 "*" 2.62230709136002e-06 3.1345273044775e-06 70.8333333333333
"chr3" 192552001 192553000 "*" 1.11022302462516e-16 5.03662826618488e-16 100
"chr3" 192712001 192713000 "*" 0 0 100
"chr3" 192745001 192746000 "*" 2.22044604925031e-16 9.81641919380259e-16 100
"chr3" 193064001 193065000 "*" 0.000285593505977677 0.000248445226337853 -52.1739130434783
"chr3" 193514001 193515000 "*" 3.42522732399431e-09 6.15633712685039e-09 -58.1818181818182
"chr3" 193601001 193602000 "*" 0 0 -100
"chr3" 193720001 193721000 "*" 3.00177499523713e-08 4.7051219130391e-08 -51.7857142857143
"chr3" 193721001 193722000 "*" 0 0 76.6304347826087
"chr3" 193736001 193737000 "*" 2.64951482975562e-09 4.82186011375752e-09 100
"chr3" 193789001 193790000 "*" 0.000295324442221245 0.000256300942920687 -51.4705882352941
"chr3" 193807001 193808000 "*" 0 0 -100
"chr3" 193854001 193855000 "*" 0 0 -50.5154639175258
"chr3" 193912001 193913000 "*" 2.88779000712225e-11 6.92935809466068e-11 100
"chr3" 193949001 193950000 "*" 5.03264097062583e-13 1.51942428521737e-12 100
"chr3" 193969001 193970000 "*" 0 0 -60.8108108108108
"chr3" 194031001 194032000 "*" 0 0 100
"chr3" 194048001 194049000 "*" 0 0 -84.0236686390533
"chr3" 194060001 194061000 "*" 1.25284005392245e-10 2.75649590272477e-10 -100
"chr3" 194077001 194078000 "*" 2.22044604925031e-16 9.81641919380259e-16 -58.2938388625592
"chr3" 194078001 194079000 "*" 4.18887147191072e-13 1.2739946038722e-12 -100
"chr3" 194082001 194083000 "*" 6.38756785908967e-07 8.33076784507971e-07 -73.6842105263158
"chr3" 194097001 194098000 "*" 0 0 -100
"chr3" 194102001 194103000 "*" 0 0 -75.1444074916856
"chr3" 194108001 194109000 "*" 4.01313771103418e-09 7.11882087237587e-09 100
"chr3" 194130001 194131000 "*" 1.12458486967171e-09 2.15740815043925e-09 100
"chr3" 194231001 194232000 "*" 6.24654217240561e-10 1.2462108191303e-09 -100
"chr3" 194291001 194292000 "*" 0 0 -100
"chr3" 194396001 194397000 "*" 1.11022302462516e-16 5.03662826618488e-16

-71.1111111111111
"chr3" 194456001 194457000 "*" 2.7630262211531e-07 3.78189313644252e-07
73.6842105263158
"chr3" 194459001 194460000 "*" 2.54484454444981e-05 2.62004281959145e-05
-62.0689655172414
"chr3" 194461001 194462000 "*" 8.33285843659937e-08 1.22170379073957e-07
-61.038961038961
"chr3" 194519001 194520000 "*" 7.15371095694195e-11 1.62643316993898e-10
-83.9080459770115
"chr3" 194539001 194540000 "*" 1.1186240822525e-10 2.48175218416007e-10
-86.3247863247863
"chr3" 194592001 194593000 "*" 8.58968451922237e-12 2.213554983903e-11 -100
"chr3" 194621001 194622000 "*" 7.03992419914812e-12 1.83313429961219e-11 -100
"chr3" 194660001 194661000 "*" 0 0 -61.1342680308197
"chr3" 194677001 194678000 "*" 0 0 98.8505747126437
"chr3" 194729001 194730000 "*" 1.48087875295744e-10 3.22075573380618e-10 100
"chr3" 194760001 194761000 "*" 6.4152538836737e-10 1.27477697728417e-09 -100
"chr3" 194800001 194801000 "*" 3.04324343503026e-11 7.26599935673121e-11 -100
"chr3" 194819001 194820000 "*" 0 0 -95.3917050691244
"chr3" 194865001 194866000 "*" 5.64878919528944e-07 7.42155605113794e-07
-64.8648648648649
"chr3" 194981001 194982000 "*" 0 0 -88.8501742160279
"chr3" 195110001 195111000 "*" 0 0 -100
"chr3" 195164001 195165000 "*" 0 0 -66.6666666666667
"chr3" 195296001 195297000 "*" 1.89848137210902e-14 6.82212372863451e-14 100
"chr3" 195309001 195310000 "*" 2.69018141096922e-12 7.41487347763769e-12 -100
"chr3" 195310001 195311000 "*" 2.4944490917278e-12 6.90310225986415e-12
-62.2950819672131
"chr3" 195352001 195353000 "*" 0 0 -80.3418803418803
"chr3" 195420001 195421000 "*" 5.01025332333427e-10 1.01358031135889e-09 -100
"chr3" 195431001 195432000 "*" 5.1309423376722e-10 1.03693943751378e-09
-91.8032786885246
"chr3" 195499001 195500000 "*" 6.4152538836737e-10 1.27477697728417e-09 100
"chr3" 195546001 195547000 "*" 4.08209022140227e-11 9.57770690255255e-11 -100
"chr3" 195556001 195557000 "*" 0 0 -99.4117647058823
"chr3" 195634001 195635000 "*" 3.99680288865056e-15 1.5566998858822e-14
-53.6104769914694
"chr3" 195734001 195735000 "*" 0 0 100
"chr3" 195809001 195810000 "*" 0 0 52.4752475247525
"chr3" 195819001 195820000 "*" 1.07835962381841e-12 3.12375201314424e-12
92.8571428571429
"chr3" 195828001 195829000 "*" 8.54871728961371e-14 2.83983502011742e-13
-96.6666666666667
"chr3" 195869001 195870000 "*" 0 0 -53.6324786324786
"chr3" 195915001 195916000 "*" 0 0 -94.2307692307692
"chr3" 195934001 195935000 "*" 0 0 52.1245711835334
"chr3" 195985001 195986000 "*" 7.14371894972032e-11 1.62429427307063e-10
95.4545454545455
"chr3" 196045001 196046000 "*" 0 0 -55.2123552123552
"chr3" 196065001 196066000 "*" 0 0 -76.3005780346821
"chr3" 196128001 196129000 "*" 2.66453525910038e-15 1.05861327033776e-14 100
"chr3" 196162001 196163000 "*" 2.15125472990962e-10 4.58126659943874e-10 -100

"chr3" 196248001 196249000 "*" 3.33066907387547e-16 1.4495649018245e-15 100
"chr3" 196388001 196389000 "*" 0 0 -76.177336276674
"chr3" 196399001 196400000 "*" 3.61932706027801e-14 1.25777907157994e-13 100
"chr3" 196516001 196517000 "*" 5.295763827462e-14 1.80214784640623e-13 -100
"chr3" 196716001 196717000 "*" 0 0 -90.5829596412556
"chr3" 196731001 196732000 "*" 6.76853332270966e-08 1.00888316368488e-07
-55.9322033898305
"chr3" 197066001 197067000 "*" 1.14130926931466e-13 3.7276674571693e-13 -100
"chr3" 197108001 197109000 "*" 0 0 -98.4375
"chr3" 197126001 197127000 "*" 1.39779633423487e-07 1.98463037958589e-07 100
"chr3" 197183001 197184000 "*" 0 0 -91.1949685534591
"chr3" 197184001 197185000 "*" 0 0 -100
"chr3" 197243001 197244000 "*" 5.85556236831763e-09 1.01720905235371e-08
-68.0851063829787
"chr3" 197283001 197284000 "*" 1.33126620838198e-10 2.92034323972915e-10
81.5789473684211
"chr3" 197300001 197301000 "*" 2.85974146935208e-05 2.92231123809482e-05
64.4444444444444
"chr3" 197314001 197315000 "*" 2.06501482580279e-14 7.3890370570066e-14 -100
"chr3" 197368001 197369000 "*" 1.01311657951797e-08 1.6976337869355e-08
53.5714285714286
"chr3" 197413001 197414000 "*" 2.4080754057465e-09 4.42115016058741e-09
51.6483516483516
"chr3" 197462001 197463000 "*" 3.07531777821168e-14 1.07626052665081e-13 -100
"chr4" 67001 68000 "*" 5.25379739713117e-12 1.39362731674666e-11
66.6666666666667
"chr4" 165001 166000 "*" 1.36074485013182e-11 3.41283700414759e-11
95.2380952380952
"chr4" 263001 264000 "*" 0 0 100
"chr4" 549001 550000 "*" 0.000379410315624273 0.000323350549361915
-50.9090909090909
"chr4" 593001 594000 "*" 9.89926678407471e-08 1.4370356981146e-07
-80.4347826086957
"chr4" 614001 615000 "*" 0 0 52.2522522522523
"chr4" 620001 621000 "*" 0 0 61.4217443249701
"chr4" 626001 627000 "*" 1.74747993852975e-11 4.32752710900609e-11
-60.7142857142857
"chr4" 627001 628000 "*" 1.13140401492018e-08 1.87801380787728e-08 100
"chr4" 641001 642000 "*" 1.0325074129014e-14 3.83008663711937e-14 -100
"chr4" 696001 697000 "*" 3.77475828372553e-15 1.47379557022071e-14 100
"chr4" 938001 939000 "*" 1.07056101761316e-08 1.78842911260166e-08
65.2173913043478
"chr4" 1003001 1004000 "*" 3.26960680752109e-12 8.91485179037936e-12
57.7464788732394
"chr4" 1072001 1073000 "*" 1.11022302462516e-16 5.03662826618488e-16
-65.7620416966211
"chr4" 1074001 1075000 "*" 1.73192314933956e-08 2.80418555140501e-08
52.4612736660929
"chr4" 1079001 1080000 "*" 4.40915653987517e-08 6.73677252860833e-08
-59.0909090909091
"chr4" 1092001 1093000 "*" 0 0 66.6666666666667
"chr4" 1145001 1146000 "*" 2.96999919102703e-08 4.65750338512475e-08

-81.6666666666667
"chr4" 1169001 1170000 "*" 2.2832757906599e-10 4.84953758410551e-10
-50.2717391304348
"chr4" 1241001 1242000 "*" 6.21724893790088e-15 2.36606385879553e-14
58.4905660377358
"chr4" 1271001 1272000 "*" 0 0 100
"chr4" 1282001 1283000 "*" 0 0 52.8894269572236
"chr4" 1393001 1394000 "*" 0 0 62.7906976744186
"chr4" 1394001 1395000 "*" 7.48512363202281e-13 2.21127316713914e-12
70.4545454545455
"chr4" 1412001 1413000 "*" 8.88178419700125e-15 3.31875892636354e-14
66.6666666666667
"chr4" 1413001 1414000 "*" 0 0 72.3878592532904
"chr4" 1422001 1423000 "*" 9.76746115290972e-05 9.17305410688939e-05
57.1428571428571
"chr4" 1478001 1479000 "*" 1.39779633423487e-07 1.98463037958589e-07 -100
"chr4" 1503001 1504000 "*" 0 0 54.0453442523506
"chr4" 1516001 1517000 "*" 0.00020976332305167 0.000186660769941092
52.3809523809524
"chr4" 1519001 1520000 "*" 0 0 -63.2606199770379
"chr4" 1547001 1548000 "*" 4.73234496034536e-09 8.30627300264826e-09 -100
"chr4" 1568001 1569000 "*" 1.33226762955019e-15 5.48133041560118e-15
-55.0847457627119
"chr4" 1596001 1597000 "*" 1.81041160396411e-07 2.53674483917665e-07
60.4166666666667
"chr4" 1597001 1598000 "*" 0 0 71.6417910447761
"chr4" 1611001 1612000 "*" 1.03654906880024e-07 1.50110982429641e-07
-60.5263157894737
"chr4" 1634001 1635000 "*" 0 0 58.1818181818182
"chr4" 1635001 1636000 "*" 1.09995346164737e-11 2.7971973818643e-11 76.5625
"chr4" 1685001 1686000 "*" 0 0 72.4674639001082
"chr4" 1712001 1713000 "*" 0.000252175885068984 0.000221423024653662
61.4285714285714
"chr4" 1724001 1725000 "*" 1.15225606833746e-11 2.91854584951278e-11
-57.1666666666667
"chr4" 1725001 1726000 "*" 1.83956171240229e-06 2.25009868786108e-06
-75.609756097561
"chr4" 1728001 1729000 "*" 9.65110769080013e-09 1.621199033097e-08 72.5
"chr4" 1779001 1780000 "*" 1.18514509317436e-09 2.267744844238e-09
-55.7251908396947
"chr4" 1797001 1798000 "*" 1.25055919508732e-07 1.79091485605621e-07
-62.7450980392157
"chr4" 1925001 1926000 "*" 1.20966398009514e-08 2.00140491666149e-08
-86.6666666666667
"chr4" 1930001 1931000 "*" 0 0 -100
"chr4" 2043001 2044000 "*" 0 0 60.4772057093018
"chr4" 2044001 2045000 "*" 0 0 85.6749311294766
"chr4" 2066001 2067000 "*" 0 0 -72.6757664391723
"chr4" 2080001 2081000 "*" 0 0 -65.3846153846154
"chr4" 2117001 2118000 "*" 8.7349629751543e-09 1.47505871898511e-08 100
"chr4" 2244001 2245000 "*" 5.55111512312578e-16 2.36485094870365e-15
86.2595419847328

"chr4" 2308001 2309000 "*" 4.25421852618957e-05 4.2288507820221e-05
-53.8461538461538
"chr4" 2347001 2348000 "*" 0 0 -100
"chr4" 2390001 2391000 "*" 8.90625305727255e-08 1.30046695793726e-07
-58.3333333333333
"chr4" 2415001 2416000 "*" 2.22044604925031e-16 9.81641919380259e-16
-78.2608695652174
"chr4" 2421001 2422000 "*" 0 0 81.6993464052288
"chr4" 2424001 2425000 "*" 1.32162483623688e-07 1.88682311059292e-07
83.3333333333333
"chr4" 2440001 2441000 "*" 2.1516286374812e-07 2.98606087005393e-07
76.1904761904762
"chr4" 2447001 2448000 "*" 0 0 -56.5821256038647
"chr4" 2589001 2590000 "*" 1.34456009104333e-07 1.91808919676809e-07
62.1621621621622
"chr4" 2810001 2811000 "*" 1.67299207820548e-08 2.71377429441055e-08 100
"chr4" 2938001 2939000 "*" 0 0 -75
"chr4" 2939001 2940000 "*" 0 0 -94.7368421052632
"chr4" 2983001 2984000 "*" 1.00821901938275e-08 1.68993646953445e-08
59.0909090909091
"chr4" 3256001 3257000 "*" 4.91828799908944e-14 1.68192819018269e-13 -100
"chr4" 3267001 3268000 "*" 0 0 73.1990924560408
"chr4" 3313001 3314000 "*" 1.06121187160024e-06 1.34248198346621e-06
63.0434782608696
"chr4" 3477001 3478000 "*" 0 0 -59.002624671916
"chr4" 3486001 3487000 "*" 0 0 -57.7757260549376
"chr4" 3546001 3547000 "*" 1.99840144432528e-12 5.59476580335607e-12 100
"chr4" 3557001 3558000 "*" 1.80966353013901e-14 6.52206904171103e-14
98.9130434782609
"chr4" 3568001 3569000 "*" 9.8862168868119e-05 9.27525037076316e-05
54.7169811320755
"chr4" 3583001 3584000 "*" 4.36509717260947e-11 1.02127008313766e-10
-63.2183908045977
"chr4" 3587001 3588000 "*" 1.14352971536391e-14 4.21921674335999e-14 100
"chr4" 3602001 3603000 "*" 1.98538780671331e-06 2.41610225827831e-06
-72.972972972973
"chr4" 3612001 3613000 "*" 2.55351295663786e-15 1.01678090381833e-14 100
"chr4" 3628001 3629000 "*" 0 0 52.8970718722272
"chr4" 3632001 3633000 "*" 0 0 62.2252747252747
"chr4" 3641001 3642000 "*" 0 0 65.6792287467134
"chr4" 3708001 3709000 "*" 0 0 -61.7704517704518
"chr4" 3709001 3710000 "*" 0 0 54.4199736120978
"chr4" 3710001 3711000 "*" 9.52854019020677e-08 1.38616238089484e-07
80.8219178082192
"chr4" 3721001 3722000 "*" 0 0 -67.0940170940171
"chr4" 3741001 3742000 "*" 0 0 90.8496732026144
"chr4" 3755001 3756000 "*" 0 0 100
"chr4" 3756001 3757000 "*" 0 0 92.6108374384236
"chr4" 3760001 3761000 "*" 1.82076576038526e-14 6.55981396322478e-14
80.1724137931034
"chr4" 3782001 3783000 "*" 2.29036789534121e-11 5.57278288031257e-11
55.1456310679612

"chr4" 3784001 3785000 "*" 0 0 81.7708333333333
"chr4" 3785001 3786000 "*" 3.3973690527489e-10 7.03784073980162e-10
62.7906976744186
"chr4" 3788001 3789000 "*" 1.07708972429954e-06 1.36134988793805e-06
59.2592592592593
"chr4" 3791001 3792000 "*" 3.28292587472578e-05 3.32114246615133e-05
58.3333333333333
"chr4" 3792001 3793000 "*" 1.67299207820548e-08 2.71377429441055e-08 -100
"chr4" 3803001 3804000 "*" 0 0 100
"chr4" 3808001 3809000 "*" 9.04570973681018e-10 1.76414389822173e-09 100
"chr4" 3863001 3864000 "*" 0 0 56.7479850705901
"chr4" 3870001 3871000 "*" 2.22044604925031e-15 8.90449334476539e-15
61.5384615384615
"chr4" 3896001 3897000 "*" 0 0 100
"chr4" 3912001 3913000 "*" 0 0 100
"chr4" 3914001 3915000 "*" 6.57204968224079e-09 1.13412966131796e-08
81.4285714285714
"chr4" 4038001 4039000 "*" 6.99440505513849e-15 2.64480529781883e-14
-97.6608187134503
"chr4" 4177001 4178000 "*" 4.83571523790527e-06 5.55604261294267e-06
66.6666666666667
"chr4" 4193001 4194000 "*" 3.94905369072163e-07 5.29022972265959e-07
78.8461538461538
"chr4" 4199001 4200000 "*" 7.95707545298008e-07 1.02409783586519e-06
72.5806451612903
"chr4" 4321001 4322000 "*" 3.17554406104037e-08 4.96229433769447e-08
61.3636363636364
"chr4" 4359001 4360000 "*" 9.32032229172819e-13 2.7181920310254e-12 100
"chr4" 4384001 4385000 "*" 0 0 100
"chr4" 4388001 4389000 "*" 0 0 64.636922632331
"chr4" 4393001 4394000 "*" 0 0 75
"chr4" 4397001 4398000 "*" 0 0 100
"chr4" 4398001 4399000 "*" 0 0 93.5064935064935
"chr4" 4409001 4410000 "*" 0 0 53.3231474407945
"chr4" 4414001 4415000 "*" 0 0 71.7948717948718
"chr4" 4730001 4731000 "*" 4.99900121297969e-12 1.32921056339624e-11 -100
"chr4" 4842001 4843000 "*" 1.67299207820548e-08 2.71377429441055e-08 100
"chr4" 4945001 4946000 "*" 6.15840711759574e-13 1.83676803572802e-12 100
"chr4" 4986001 4987000 "*" 8.3882012447134e-11 1.88922989503423e-10 -100
"chr4" 4989001 4990000 "*" 0.000138951373611085 0.000127295950042963 -70
"chr4" 4996001 4997000 "*" 0 0 55.1401869158879
"chr4" 4997001 4998000 "*" 0 0 -100
"chr4" 5103001 5104000 "*" 5.48638023900594e-10 1.10374357336421e-09 -100
"chr4" 5208001 5209000 "*" 1.96644922567657e-11 4.82930612417392e-11 -100
"chr4" 5400001 5401000 "*" 1.38590361409285e-10 3.02706178892116e-10 -100
"chr4" 5450001 5451000 "*" 1.35003119794419e-13 4.3679847476192e-13 -100
"chr4" 5463001 5464000 "*" 1.83700960554312e-07 2.57211250637662e-07
52.7829313543599
"chr4" 5503001 5504000 "*" 3.73981179357941e-10 7.69883519166722e-10
71.2121212121212
"chr4" 5616001 5617000 "*" 8.57092175010621e-13 2.51149198099779e-12 -100
"chr4" 5658001 5659000 "*" 1.56863411149288e-12 4.4469621057462e-12 100

"chr4" 5689001 5690000 "*" 3.6700841921089e-08 5.65530226072258e-08 100
"chr4" 5725001 5726000 "*" 9.04570973681018e-10 1.76414389822173e-09 -100
"chr4" 5758001 5759000 "*" 0 0 100
"chr4" 5769001 5770000 "*" 1.67299207820548e-08 2.71377429441055e-08 100
"chr4" 5795001 5796000 "*" 0 0 56.25
"chr4" 5799001 5800000 "*" 2.43005615629954e-09 4.45014222280278e-09 100
"chr4" 5809001 5810000 "*" 8.43347613965761e-12 2.17859001990012e-11 100
"chr4" 5829001 5830000 "*" 9.80763359414993e-10 1.8980032424715e-09 100
"chr4" 5834001 5835000 "*" 7.0006535457523e-08 1.03695832512054e-07 -100
"chr4" 5838001 5839000 "*" 2.37587727269783e-14 8.44268514944447e-14 100
"chr4" 5840001 5841000 "*" 3.04201108747293e-14 1.06563274901204e-13
50.990990990991
"chr4" 5913001 5914000 "*" 3.52704532247117e-11 8.35581292290093e-11 -100
"chr4" 5926001 5927000 "*" 7.28924698378819e-11 1.6558486649022e-10
58.7104406980183
"chr4" 5933001 5934000 "*" 2.77282466987216e-06 3.30388613532549e-06 60
"chr4" 5935001 5936000 "*" 5.38290356844051e-06 6.14394292224725e-06
-52.9411764705882
"chr4" 5947001 5948000 "*" 9.86133327263161e-06 1.08220339572054e-05
-67.9245283018868
"chr4" 5957001 5958000 "*" 0 0 76.7441860465116
"chr4" 5959001 5960000 "*" 1.87405646556726e-13 5.97285292681429e-13 100
"chr4" 5960001 5961000 "*" 5.43117762319412e-06 6.19387126034938e-06
83.3333333333333
"chr4" 6006001 6007000 "*" 4.79616346638068e-14 1.64380445416596e-13 100
"chr4" 6011001 6012000 "*" 1.86465842766381e-10 4.01792565502082e-10
64.2857142857143
"chr4" 6020001 6021000 "*" 4.13329959148001e-09 7.31905277636459e-09
-89.6551724137931
"chr4" 6048001 6049000 "*" 0 0 78.4090909090909
"chr4" 6051001 6052000 "*" 0 0 100
"chr4" 6082001 6083000 "*" 0 0 100
"chr4" 6086001 6087000 "*" 2.05355636474502e-05 2.14532877489624e-05 55
"chr4" 6096001 6097000 "*" 5.794809077031e-12 1.52911192757929e-11 100
"chr4" 6097001 6098000 "*" 0 0 99.0196078431373
"chr4" 6112001 6113000 "*" 1.07787112568758e-11 2.74553537455803e-11
75.5555555555556
"chr4" 6115001 6116000 "*" 0 0 60
"chr4" 6127001 6128000 "*" 1.29220323330514e-09 2.4626536786286e-09
65.3061224489796
"chr4" 6129001 6130000 "*" 5.55111512312578e-16 2.36485094870365e-15
71.0526315789474
"chr4" 6137001 6138000 "*" 7.95908609829699e-06 8.85270264440226e-06
-71.4285714285714
"chr4" 6142001 6143000 "*" 2.88657986402541e-15 1.14155613124573e-14 100
"chr4" 6144001 6145000 "*" 1.90181204118289e-13 6.05298617539364e-13 100
"chr4" 6267001 6268000 "*" 1.67299207820548e-08 2.71377429441055e-08 -100
"chr4" 6275001 6276000 "*" 4.23558679241154e-08 6.48226241972226e-08
-72.7272727272727
"chr4" 6332001 6333000 "*" 0 0 -100
"chr4" 6333001 6334000 "*" 5.03264097062583e-13 1.51942428521737e-12 -100
"chr4" 6355001 6356000 "*" 3.33066907387547e-16 1.4495649018245e-15 100

"chr4" 6357001 6358000 "*" 3.88022947106492e-13 1.1867462770515e-12 -100
"chr4" 6377001 6378000 "*" 0 0 100
"chr4" 6383001 6384000 "*" 6.38468389269065e-10 1.27237057725591e-09
-76.9230769230769
"chr4" 6387001 6388000 "*" 0 0 -100
"chr4" 6394001 6395000 "*" 2.43005615629954e-09 4.45014222280278e-09 100
"chr4" 6395001 6396000 "*" 3.21304205463946e-10 6.67824922105025e-10 -80
"chr4" 6405001 6406000 "*" 1.22124532708767e-15 5.03921984217778e-15 100
"chr4" 6421001 6422000 "*" 4.73234496034536e-09 8.30627300264826e-09 100
"chr4" 6443001 6444000 "*" 0 0 75.6410256410256
"chr4" 6448001 6449000 "*" 1.69827999441097e-08 2.75249203590438e-08
-55.8139534883721
"chr4" 6452001 6453000 "*" 0 0 80.6451612903226
"chr4" 6455001 6456000 "*" 1.29171007223761e-10 2.83767570085854e-10
-74.2268041237113
"chr4" 6472001 6473000 "*" 0 0 57.2743207712533
"chr4" 6511001 6512000 "*" 1.23271976959494e-07 1.76691451470357e-07
75.9259259259259
"chr4" 6521001 6522000 "*" 2.64951482975562e-09 4.82186011375752e-09 -100
"chr4" 6530001 6531000 "*" 0 0 99.0196078431373
"chr4" 6534001 6535000 "*" 6.66133814775094e-16 2.81595744474255e-15
84.8837209302326
"chr4" 6544001 6545000 "*" 0 0 60.9034267912773
"chr4" 6564001 6565000 "*" 0 0 50.5125702227281
"chr4" 6565001 6566000 "*" 0 0 56.3303316044741
"chr4" 6577001 6578000 "*" 9.76996261670138e-15 3.63402770315216e-14
-64.9350649350649
"chr4" 6676001 6677000 "*" 0 0 82.0598006644518
"chr4" 6690001 6691000 "*" 3.32538344860467e-06 3.91451251683331e-06 -65
"chr4" 6783001 6784000 "*" 2.1094237467878e-15 8.47434540879246e-15 -100
"chr4" 6918001 6919000 "*" 4.77395900588817e-15 1.84201484337652e-14 -100
"chr4" 6927001 6928000 "*" 0 0 -71.9298245614035
"chr4" 6947001 6948000 "*" 1.12055920098442e-11 2.84500040664577e-11 -100
"chr4" 6949001 6950000 "*" 1.29037891483108e-11 3.24582280286866e-11 100
"chr4" 7072001 7073000 "*" 1.76718384370389e-08 2.85794772616816e-08
62.406015037594
"chr4" 7106001 7107000 "*" 1.09690034832965e-13 3.59303458454092e-13
73.0769230769231
"chr4" 7107001 7108000 "*" 5.55111512312578e-16 2.36485094870365e-15
68.5714285714286
"chr4" 7109001 7110000 "*" 0 0 100
"chr4" 7113001 7114000 "*" 0 0 100
"chr4" 7133001 7134000 "*" 0 0 73.9009816474605
"chr4" 7159001 7160000 "*" 6.4152538836737e-10 1.27477697728417e-09 100
"chr4" 7188001 7189000 "*" 0 0 100
"chr4" 7194001 7195000 "*" 0 0 51.4943511521511
"chr4" 7210001 7211000 "*" 7.14168231219503e-08 1.05649694445114e-07
-53.3333333333333
"chr4" 7221001 7222000 "*" 2.60353922976186e-07 3.5753307375039e-07
52.0833333333333
"chr4" 7255001 7256000 "*" 1.30021882149833e-10 2.85539026058085e-10
77.2727272727273

"chr4" 7259001 7260000 "*" 0.000334851753864718 0.000287983064674082
-55.5555555555556
"chr4" 7262001 7263000 "*" 1.98086799474417e-06 2.41125089823315e-06
-53.1914893617021
"chr4" 7277001 7278000 "*" 1.7080541536707e-08 2.76725799133848e-08
68.9393939393939
"chr4" 7278001 7279000 "*" 4.99900121297969e-12 1.32921056339624e-11 100
"chr4" 7285001 7286000 "*" 4.44089209850063e-16 1.91071758245033e-15
-57.1428571428571
"chr4" 7307001 7308000 "*" 3.60213525674169e-10 7.42696145837762e-10
-70.1298701298701
"chr4" 7308001 7309000 "*" 0 0 -71.6049382716049
"chr4" 7309001 7310000 "*" 3.11193985025326e-08 4.86773608235298e-08
94.1176470588235
"chr4" 7324001 7325000 "*" 0 0 73.9583333333333
"chr4" 7326001 7327000 "*" 0 0 84.375
"chr4" 7330001 7331000 "*" 0 0 -85.4226566992524
"chr4" 7333001 7334000 "*" 1.55431223447522e-15 6.33705141101682e-15
-54.7619047619048
"chr4" 7337001 7338000 "*" 0 0 63.7907137907138
"chr4" 7340001 7341000 "*" 1.48087875295744e-10 3.22075573380618e-10 -100
"chr4" 7362001 7363000 "*" 0 0 -100
"chr4" 7433001 7434000 "*" 0 0 63.7254901960784
"chr4" 7445001 7446000 "*" 1.16573417585641e-14 4.29335582900508e-14 100
"chr4" 7453001 7454000 "*" 7.93809462606987e-14 2.64679416473686e-13 100
"chr4" 7472001 7473000 "*" 0 0 64.9253731343284
"chr4" 7475001 7476000 "*" 2.99760216648792e-15 1.18450729613838e-14
-54.0540540540541
"chr4" 7476001 7477000 "*" 4.01313771103418e-09 7.11882087237587e-09 -100
"chr4" 7479001 7480000 "*" 0.000285593505977677 0.000248445226337853
-52.1739130434783
"chr4" 7492001 7493000 "*" 0 0 -96.969696969697
"chr4" 7499001 7500000 "*" 1.11022302462516e-15 4.59817122935606e-15 65.625
"chr4" 7515001 7516000 "*" 6.44405218075406e-09 1.1132160470276e-08
-51.8181818181818
"chr4" 7516001 7517000 "*" 1.20484025778467e-06 1.51291091626352e-06
-52.7777777777778
"chr4" 7525001 7526000 "*" 8.92319551581977e-12 2.29613626613262e-11
-52.1967213114754
"chr4" 7540001 7541000 "*" 1.67299207820548e-08 2.71377429441055e-08 100
"chr4" 7544001 7545000 "*" 4.0635857900817e-08 6.23149132293182e-08
-66.6666666666667
"chr4" 7575001 7576000 "*" 8.29336599394992e-14 2.75830476998008e-13
-60.3448275862069
"chr4" 7586001 7587000 "*" 1.67299207820548e-08 2.71377429441055e-08 100
"chr4" 7589001 7590000 "*" 0 0 -73.8095238095238
"chr4" 7597001 7598000 "*" 8.77756756167969e-11 1.97330112766911e-10
-62.0689655172414
"chr4" 7608001 7609000 "*" 1.54765008828495e-05 1.64771109106961e-05
-66.6666666666667
"chr4" 7624001 7625000 "*" 2.00227675550835e-08 3.20459176617961e-08 -100
"chr4" 7633001 7634000 "*" 1.13140401492018e-08 1.87801380787728e-08 100

"chr4" 7638001 7639000 "*" 0 0 54.5454545454545
"chr4" 7642001 7643000 "*" 2.22044604925031e-16 9.81641919380259e-16 -100
"chr4" 7646001 7647000 "*" 3.40125705378114e-10 7.03784073980162e-10 100
"chr4" 7665001 7666000 "*" 3.44169137633799e-15 1.3517917469209e-14
64.1456582633053
"chr4" 7717001 7718000 "*" 6.70990774054303e-09 1.15329015552141e-08
-69.6428571428571
"chr4" 7897001 7898000 "*" 0 0 100
"chr4" 7902001 7903000 "*" 6.93882802971224e-06 7.78842164960861e-06
-57.8947368421053
"chr4" 7930001 7931000 "*" 8.80406858527749e-14 2.91456141063452e-13 -100
"chr4" 7967001 7968000 "*" 0 0 -52.1008403361345
"chr4" 7974001 7975000 "*" 7.35548151276078e-08 1.08588645363448e-07
-59.0909090909091
"chr4" 7981001 7982000 "*" 6.62720955890705e-09 1.1398042897325e-08
-85.4166666666667
"chr4" 7993001 7994000 "*" 3.18559262413309e-07 4.32247043218458e-07
-75.4098360655738
"chr4" 8014001 8015000 "*" 0 0 100
"chr4" 8043001 8044000 "*" 0.000129328979378784 0.000119106074697934
-53.8461538461538
"chr4" 8054001 8055000 "*" 2.14666248776751e-06 2.60139414196789e-06
-72.8813559322034
"chr4" 8055001 8056000 "*" 0 0 100
"chr4" 8068001 8069000 "*" 2.69018141096922e-12 7.41487347763769e-12 -100
"chr4" 8082001 8083000 "*" 6.38322295021254e-09 1.10339339031813e-08
-63.1578947368421
"chr4" 8097001 8098000 "*" 1.10494946525819e-11 2.80930267106138e-11
60.8250526949714
"chr4" 8119001 8120000 "*" 2.17081908004957e-12 6.04883980726504e-12 62.5
"chr4" 8123001 8124000 "*" 2.00839345154691e-13 6.37153994630405e-13 -76.875
"chr4" 8137001 8138000 "*" 0 0 100
"chr4" 8138001 8139000 "*" 0 0 73.1584525702173
"chr4" 8165001 8166000 "*" 0 0 90.4761904761905
"chr4" 8175001 8176000 "*" 2.67086353034074e-12 7.37296041114003e-12
78.4313725490196
"chr4" 8180001 8181000 "*" 8.79770700734639e-11 1.97761548204376e-10
80.2631578947368
"chr4" 8227001 8228000 "*" 1.34559030584569e-13 4.35581312463895e-13 100
"chr4" 8252001 8253000 "*" 7.3276508194553e-09 1.25380818491884e-08
79.2452830188679
"chr4" 8285001 8286000 "*" 0 0 78.4466019417476
"chr4" 8287001 8288000 "*" 0 0 100
"chr4" 8291001 8292000 "*" 3.33066907387547e-16 1.4495649018245e-15
88.4393063583815
"chr4" 8311001 8312000 "*" 0 0 72.1739130434783
"chr4" 8326001 8327000 "*" 1.51323398256409e-13 4.86995056237989e-13 100
"chr4" 8332001 8333000 "*" 0 0 52.7354997943233
"chr4" 8340001 8341000 "*" 5.01025332333427e-10 1.01358031135889e-09 100
"chr4" 8346001 8347000 "*" 0 0 68.1571815718157
"chr4" 8381001 8382000 "*" 0 0 -100
"chr4" 8414001 8415000 "*" 1.11022302462516e-16 5.03662826618488e-16 -100

"chr4" 8429001 8430000 "*" 0 0 52.3364485981308
"chr4" 8470001 8471000 "*" 0 0 -100
"chr4" 8484001 8485000 "*" 5.55111512312578e-16 2.36485094870365e-15 100
"chr4" 8508001 8509000 "*" 3.93644894458589e-10 8.0840803123314e-10
-68.8888888888889
"chr4" 8509001 8510000 "*" 4.44089209850063e-16 1.91071758245033e-15 100
"chr4" 8510001 8511000 "*" 2.72196980709571e-05 2.79065470622823e-05
53.6231884057971
"chr4" 8519001 8520000 "*" 7.06899855540399e-07 9.16019649164048e-07
50.4504504504505
"chr4" 8545001 8546000 "*" 0 0 -78.5714285714286
"chr4" 8549001 8550000 "*" 3.88578058618805e-15 1.5152981210988e-14 100
"chr4" 8555001 8556000 "*" 3.01980662698043e-14 1.0593227230447e-13 100
"chr4" 8556001 8557000 "*" 0 0 -72.7272727272727
"chr4" 8584001 8585000 "*" 0 0 62.1848739495798
"chr4" 8591001 8592000 "*" 5.07239805713766e-11 1.175997368229e-10
66.6666666666667
"chr4" 8595001 8596000 "*" 7.15882908508547e-12 1.86345610231918e-11
53.4343434343434
"chr4" 8598001 8599000 "*" 1.17017506795491e-13 3.81716338420629e-13
69.4444444444444
"chr4" 8604001 8605000 "*" 0 0 75
"chr4" 8613001 8614000 "*" 0 0 100
"chr4" 8622001 8623000 "*" 9.65729496371637e-10 1.87255912493232e-09 100
"chr4" 8652001 8653000 "*" 5.48638023900594e-10 1.10374357336421e-09 100
"chr4" 8658001 8659000 "*" 5.88418203051333e-15 2.245355428447e-14
54.5880067120021
"chr4" 8659001 8660000 "*" 3.7607964253894e-07 5.05231943813116e-07
72.972972972973
"chr4" 8678001 8679000 "*" 0 0 100
"chr4" 8684001 8685000 "*" 3.95353005888666e-09 7.03928529283733e-09 100
"chr4" 8686001 8687000 "*" 0 0 100
"chr4" 8691001 8692000 "*" 0 0 90.5660377358491
"chr4" 8704001 8705000 "*" 2.75570111174517e-09 5.00626020213722e-09 75
"chr4" 8708001 8709000 "*" 3.62590069080682e-10 7.47392908618654e-10
53.5947712418301
"chr4" 8709001 8710000 "*" 0 0 100
"chr4" 8711001 8712000 "*" 1.39163902623807e-10 3.03927057501008e-10 76.8
"chr4" 8729001 8730000 "*" 1.39578348878899e-11 3.49525043638484e-11
85.3333333333333
"chr4" 8732001 8733000 "*" 0 0 100
"chr4" 8733001 8734000 "*" 2.00200533894135e-05 2.09454363748417e-05
61.7647058823529
"chr4" 8737001 8738000 "*" 5.53306662154363e-07 7.27639394821453e-07
76.1904761904762
"chr4" 8740001 8741000 "*" 1.80120700576936e-08 2.90998042864353e-08
82.5396825396825
"chr4" 8761001 8762000 "*" 2.22044604925031e-16 9.81641919380259e-16
58.1818181818182
"chr4" 8764001 8765000 "*" 0 0 -81.5217391304348
"chr4" 8765001 8766000 "*" 0 0 -51.4184397163121
"chr4" 8770001 8771000 "*" 0 0 50.5494505494505

"chr4" 8783001 8784000 "*" 1.22124532708767e-15 5.03921984217778e-15
-56.1991869918699
"chr4" 8788001 8789000 "*" 0 0 70.5357142857143
"chr4" 8795001 8796000 "*" 3.40117045638522e-10 7.03784073980162e-10
-66.6666666666667
"chr4" 8837001 8838000 "*" 1.49310831476512e-08 2.44375206748162e-08 75
"chr4" 8879001 8880000 "*" 2.07598464985193e-06 2.52050554207248e-06
68.3544303797468
"chr4" 8881001 8882000 "*" 3.14048786975718e-12 8.57597580834377e-12
-57.1428571428571
"chr4" 8882001 8883000 "*" 2.64202910396083e-05 2.71356701416907e-05
60.9756097560976
"chr4" 8903001 8904000 "*" 6.43996536897617e-05 6.22599140150143e-05
52.3809523809524
"chr4" 8917001 8918000 "*" 0 0 70.3680292175868
"chr4" 8922001 8923000 "*" 9.99200722162641e-16 4.15382497462808e-15
50.9611215493568
"chr4" 8940001 8941000 "*" 3.86668249641176e-08 5.94418666890193e-08
75.7575757575758
"chr4" 8942001 8943000 "*" 1.29751764887942e-12 3.72637314900227e-12
68.8473520249221
"chr4" 8945001 8946000 "*" 0 0 -100
"chr4" 8978001 8979000 "*" 0 0 100
"chr4" 8995001 8996000 "*" 0 0 100
"chr4" 9382001 9383000 "*" 1.4432899320127e-15 5.90750815956055e-15
55.5927866455465
"chr4" 9495001 9496000 "*" 3.68371999570627e-13 1.13149976091305e-12
63.3333333333333
"chr4" 9499001 9500000 "*" 0 0 50.5255507100526
"chr4" 9517001 9518000 "*" 1.3988810110277e-14 5.10290212559087e-14
66.6666666666667
"chr4" 9670001 9671000 "*" 0 0 100
"chr4" 9836001 9837000 "*" 1.12055920098442e-11 2.84500040664577e-11 -100
"chr4" 10019001 10020000 "*" 1.11022302462516e-16 5.03662826618488e-16
-82.7586206896552
"chr4" 10077001 10078000 "*" 2.6013813325676e-09 4.74908099597444e-09 93.75
"chr4" 10117001 10118000 "*" 0 0 -60
"chr4" 10132001 10133000 "*" 6.91004031772025e-09 1.18592913246344e-08
80.7692307692308
"chr4" 10147001 10148000 "*" 3.04324343503026e-11 7.26599935673121e-11 100
"chr4" 10156001 10157000 "*" 1.98124849859482e-10 4.25157611354621e-10 -100
"chr4" 10173001 10174000 "*" 5.14200543855203e-06 5.88639366700709e-06
-70.5882352941177
"chr4" 10589001 10590000 "*" 8.3882012447134e-11 1.88922989503423e-10 100
"chr4" 10596001 10597000 "*" 7.52721026020531e-05 7.19471184388947e-05
57.1428571428571
"chr4" 10600001 10601000 "*" 2.69007038866675e-13 8.38831022628993e-13 100
"chr4" 10966001 10967000 "*" 3.33066907387547e-16 1.4495649018245e-15 -100
"chr4" 11023001 11024000 "*" 7.0006535457523e-08 1.03695832512054e-07 -100
"chr4" 11072001 11073000 "*" 2.00227675550835e-08 3.20459176617961e-08 -100
"chr4" 11531001 11532000 "*" 2.15125472990962e-10 4.58126659943874e-10 -100
"chr4" 12005001 12006000 "*" 0 0 -100

"chr4" 12239001 12240000 "*" 4.17443857259059e-14 1.43911659396181e-13
-93.3333333333333
"chr4" 12296001 12297000 "*" 4.01313771103418e-09 7.11882087237587e-09 -100
"chr4" 12606001 12607000 "*" 3.95861121660346e-12 1.0684496262032e-11 100
"chr4" 13239001 13240000 "*" 1.11022302462516e-16 5.03662826618488e-16 100
"chr4" 13532001 13533000 "*" 0 0 62.8708901363272
"chr4" 13546001 13547000 "*" 0 0 70.4792985711013
"chr4" 13781001 13782000 "*" 7.0006535457523e-08 1.03695832512054e-07 -100
"chr4" 14591001 14592000 "*" 5.27355936696949e-14 1.79605736163153e-13
68.3568075117371
"chr4" 14904001 14905000 "*" 3.95353005888666e-09 7.03928529283733e-09 100
"chr4" 15907001 15908000 "*" 0 0 -73.2456140350877
"chr4" 15965001 15966000 "*" 2.33028818463765e-10 4.93719283454501e-10 100
"chr4" 15992001 15993000 "*" 1.55431223447522e-15 6.33705141101682e-15 -100
"chr4" 16015001 16016000 "*" 3.95353005888666e-09 7.03928529283733e-09 100
"chr4" 16057001 16058000 "*" 1.12634901405784e-10 2.49563754426169e-10 -100
"chr4" 16063001 16064000 "*" 8.88178419700125e-16 3.70670032207938e-15
68.3333333333333
"chr4" 16117001 16118000 "*" 3.26405569239796e-14 1.13973003849234e-13 100
"chr4" 16160001 16161000 "*" 4.02167188440217e-12 1.08266700497926e-11 100
"chr4" 16363001 16364000 "*" 4.99900121297969e-12 1.32921056339624e-11 100
"chr4" 16395001 16396000 "*" 1.13407062629634e-08 1.88212211206977e-08
76.9230769230769
"chr4" 17031001 17032000 "*" 7.0006535457523e-08 1.03695832512054e-07 -100
"chr4" 17092001 17093000 "*" 1.36604061395929e-11 3.42333952202875e-11 -100
"chr4" 17301001 17302000 "*" 8.71525074330748e-14 2.88848349331391e-13 100
"chr4" 17490001 17491000 "*" 4.03597383291654e-08 6.19153923694837e-08
58.6206896551724
"chr4" 17574001 17575000 "*" 2.4535928844216e-14 8.69144396197119e-14 100
"chr4" 17649001 17650000 "*" 2.22044604925031e-16 9.81641919380259e-16 100
"chr4" 17980001 17981000 "*" 8.01666433236647e-09 1.36392368970109e-08 -100
"chr4" 18247001 18248000 "*" 0 0 -100
"chr4" 18419001 18420000 "*" 0 0 100
"chr4" 19007001 19008000 "*" 9.65729496371637e-10 1.87255912493232e-09 -100
"chr4" 19457001 19458000 "*" 2.64996014021079e-09 4.82256530146407e-09
-73.2283464566929
"chr4" 20181001 20182000 "*" 7.78332953643712e-12 2.01920837403161e-11
-54.9881491057962
"chr4" 20636001 20637000 "*" 1.22124532708767e-15 5.03921984217778e-15 -100
"chr4" 21256001 21257000 "*" 1.12458486967171e-09 2.15740815043925e-09 -100
"chr4" 22226001 22227000 "*" 1.12458486967171e-09 2.15740815043925e-09 100
"chr4" 22270001 22271000 "*" 4.07050825823596e-07 5.44261714716217e-07
-68.5714285714286
"chr4" 23284001 23285000 "*" 9.08108033215171e-11 2.03407753381119e-10 100
"chr4" 23765001 23766000 "*" 2.68450373042128e-10 5.64465213127486e-10 -100
"chr4" 24472001 24473000 "*" 0 0 55.659276546091
"chr4" 24473001 24474000 "*" 0 0 52.5668897460711
"chr4" 24586001 24587000 "*" 0 0 95.1515151515152
"chr4" 24732001 24733000 "*" 3.9190872769268e-14 1.35416418933905e-13 100
"chr4" 24734001 24735000 "*" 1.42307277073428e-11 3.56166194273734e-11
71.0526315789474
"chr4" 24800001 24801000 "*" 4.14335232790108e-13 1.26153063645578e-12 100

"chr4" 24858001 24859000 "*" 2.08995487582797e-10 4.46944006972663e-10 -100
"chr4" 24886001 24887000 "*" 2.68450373042128e-10 5.64465213127486e-10 100
"chr4" 24960001 24961000 "*" 5.55111512312578e-16 2.36485094870365e-15
-57.7858147478401
"chr4" 25077001 25078000 "*" 1.71606062693286e-10 3.70942260395654e-10
58.8709677419355
"chr4" 25161001 25162000 "*" 0 0 64.2857142857143
"chr4" 25306001 25307000 "*" 4.46405531835392e-05 4.42311628182131e-05
62.962962962963
"chr4" 25587001 25588000 "*" 1.07075632804765e-08 1.78871956021807e-08 66
"chr4" 25634001 25635000 "*" 3.63445940010365e-11 8.58565209111204e-11 100
"chr4" 25655001 25656000 "*" 0 0 100
"chr4" 25656001 25657000 "*" 0 0 100
"chr4" 25664001 25665000 "*" 0 0 89.4009216589862
"chr4" 25667001 25668000 "*" 0 0 -100
"chr4" 25766001 25767000 "*" 0 0 100
"chr4" 25797001 25798000 "*" 3.95353005888666e-09 7.03928529283733e-09 -100
"chr4" 25811001 25812000 "*" 3.46500605985511e-13 1.06777978272295e-12 100
"chr4" 25854001 25855000 "*" 1.52794177310511e-09 2.87642564232045e-09 100
"chr4" 25856001 25857000 "*" 2.88710388929303e-10 6.03399264731139e-10 100
"chr4" 25888001 25889000 "*" 1.1953414396304e-05 1.29468849991476e-05 -56
"chr4" 25892001 25893000 "*" 9.20147040339714e-06 1.0141522991798e-05
-53.1914893617021
"chr4" 25915001 25916000 "*" 1.46327394645596e-12 4.17581067551841e-12
61.4035087719298
"chr4" 25936001 25937000 "*" 8.00994948813027e-06 8.90333967966777e-06
64.7058823529412
"chr4" 25972001 25973000 "*" 3.46146926677449e-07 4.67482227931052e-07
79.5918367346939
"chr4" 26175001 26176000 "*" 1.52794177310511e-09 2.87642564232045e-09 -100
"chr4" 26477001 26478000 "*" 8.3882012447134e-11 1.88922989503423e-10 100
"chr4" 26478001 26479000 "*" 1.11022302462516e-16 5.03662826618488e-16 100
"chr4" 26492001 26493000 "*" 8.26183566005056e-12 2.13782647853716e-11
94.8717948717949
"chr4" 27980001 27981000 "*" 0 0 100
"chr4" 28748001 28749000 "*" 0.000323564814758259 0.000278902921428228
56.8181818181818
"chr4" 29510001 29511000 "*" 0 0 100
"chr4" 29543001 29544000 "*" 4.73234496034536e-09 8.30627300264826e-09 100
"chr4" 30095001 30096000 "*" 9.2148511043888e-15 3.43705279328267e-14 100
"chr4" 30541001 30542000 "*" 2.83871526285395e-09 5.14843604263424e-09
70.5882352941177
"chr4" 30721001 30722000 "*" 0 0 68.1845931845932
"chr4" 31837001 31838000 "*" 8.77076189453874e-15 3.27889038038365e-14 -100
"chr4" 32217001 32218000 "*" 2.00227675550835e-08 3.20459176617961e-08 100
"chr4" 32653001 32654000 "*" 0 0 100
"chr4" 32750001 32751000 "*" 0 0 100
"chr4" 33091001 33092000 "*" 1.26565424807268e-14 4.63923203146481e-14 -100
"chr4" 34658001 34659000 "*" 1.39779633423487e-07 1.98463037958589e-07 100
"chr4" 35496001 35497000 "*" 4.48262880037298e-07 5.96211412499071e-07
76.7123287671233
"chr4" 35580001 35581000 "*" 1.11022302462516e-16 5.03662826618488e-16 100

"chr4" 36867001 36868000 "*" 9.04570973681018e-10 1.76414389822173e-09 100
"chr4" 37455001 37456000 "*" 0 0 64.6551724137931
"chr4" 37457001 37458000 "*" 0 0 -100
"chr4" 37543001 37544000 "*" 1.7145251884898e-10 3.70658395178989e-10
-94.4444444444444
"chr4" 37645001 37646000 "*" 3.40125705378114e-10 7.03784073980162e-10 100
"chr4" 37719001 37720000 "*" 2.22044604925031e-16 9.81641919380259e-16 -100
"chr4" 37926001 37927000 "*" 2.1997412891217e-08 3.50174565604339e-08 -80
"chr4" 38081001 38082000 "*" 2.52449542825772e-07 3.47341761878397e-07
66.6666666666667
"chr4" 38259001 38260000 "*" 1.4432899320127e-15 5.90750815956055e-15 -100
"chr4" 38345001 38346000 "*" 3.25124442309699e-05 3.2934466660002e-05
61.5384615384615
"chr4" 38414001 38415000 "*" 8.77076189453874e-15 3.27889038038365e-14 -100
"chr4" 38496001 38497000 "*" 8.7349629751543e-09 1.47505871898511e-08 -100
"chr4" 38730001 38731000 "*" 2.43005615629954e-09 4.45014222280278e-09 100
"chr4" 38869001 38870000 "*" 0 0 -75.1245506716277
"chr4" 38951001 38952000 "*" 2.00227675550835e-08 3.20459176617961e-08 -100
"chr4" 39044001 39045000 "*" 2.88710388929303e-10 6.03399264731139e-10 100
"chr4" 39064001 39065000 "*" 9.14157638476354e-13 2.66886212024714e-12
90.9090909090909
"chr4" 39810001 39811000 "*" 3.33066907387547e-16 1.4495649018245e-15 100
"chr4" 40052001 40053000 "*" 3.56775498033812e-10 7.35898629809829e-10 -100
"chr4" 40266001 40267000 "*" 1.17905685215192e-13 3.83653245040640e-13 100
"chr4" 40306001 40307000 "*" 3.56775498033812e-10 7.35898629809829e-10 100
"chr4" 40329001 40330000 "*" 6.93311655863837e-08 1.03197650079882e-07 78
"chr4" 40339001 40340000 "*" 3.38408881147556e-05 3.41565268180454e-05
63.3333333333333
"chr4" 40341001 40342000 "*" 6.04072347698548e-12 1.59088211501136e-11
62.0689655172414
"chr4" 40406001 40407000 "*" 1.0293074289347e-05 1.12574691787191e-05
51.7241379310345
"chr4" 41752001 41753000 "*" 1.06133435373579e-10 2.35910622903034e-10
86.7924528301887
"chr4" 41790001 41791000 "*" 0 0 100
"chr4" 41852001 41853000 "*" 4.48820347553891e-09 7.9215985863704e-09
86.6666666666667
"chr4" 42267001 42268000 "*" 3.97459842815806e-14 1.37266254951776e-13
54.5454545454545
"chr4" 42308001 42309000 "*" 1.67299207820548e-08 2.71377429441055e-08 100
"chr4" 42323001 42324000 "*" 2.64951482975562e-09 4.82186011375752e-09 -100
"chr4" 42349001 42350000 "*" 7.78377362564697e-13 2.29551678017433e-12 -100
"chr4" 42398001 42399000 "*" 0 0 -100
"chr4" 42658001 42659000 "*" 0 0 97.2034956304619
"chr4" 44302001 44303000 "*" 1.35036426485158e-12 3.86629547363774e-12 -100
"chr4" 45570001 45571000 "*" 0 0 97.7272727272727
"chr4" 45798001 45799000 "*" 3.47506345921289e-09 6.2417400235238e-09
-81.3559322033898
"chr4" 45942001 45943000 "*" 3.40117045638522e-10 7.03784073980162e-10
63.4146341463415
"chr4" 46392001 46393000 "*" 0 0 58.5947210645609
"chr4" 47033001 47034000 "*" 1.50237045026813e-10 3.26460338863082e-10

94.7368421052632
"chr4" 47620001 47621000 "*" 4.99900121297969e-12 1.32921056339624e-11 100
"chr4" 47770001 47771000 "*" 1.35036426485158e-12 3.86629547363774e-12 100
"chr4" 47822001 47823000 "*" 2.64951482975562e-09 4.82186011375752e-09 -100
"chr4" 47915001 47916000 "*" 2.4535928844216e-14 8.69144396197119e-14 -100
"chr4" 48343001 48344000 "*" 0 0 60
"chr4" 48463001 48464000 "*" 1.39779633423487e-07 1.98463037958589e-07 -100
"chr4" 48731001 48732000 "*" 2.08814465718632e-09 3.86232567960463e-09 -100
"chr4" 48751001 48752000 "*" 2.88779000712225e-11 6.92935809466068e-11 -100
"chr4" 48898001 48899000 "*" 2.71893618730701e-13 8.46692454027087e-13 100
"chr4" 48908001 48909000 "*" 0 0 -54.8496961944356
"chr4" 48946001 48947000 "*" 6.77335965093562e-12 1.76897011246462e-11 -100
"chr4" 49098001 49099000 "*" 5.78292191910634e-10 1.16015519718754e-09
58.5365853658537
"chr4" 49101001 49102000 "*" 1.40076839016956e-12 4.0041493019352e-12
-77.0390070921986
"chr4" 49123001 49124000 "*" 2.04058991926104e-13 6.46212870199377e-13
63.510101010101
"chr4" 49154001 49155000 "*" 1.99281884993852e-07 2.77767997023852e-07 90
"chr4" 49155001 49156000 "*" 6.93034146070914e-07 8.99225754397336e-07
-72.463768115942
"chr4" 49215001 49216000 "*" 1.07882591748876e-11 2.74553537455803e-11 100
"chr4" 49308001 49309000 "*" 0 0 66.4772727272727
"chr4" 49324001 49325000 "*" 1.33540956070988e-11 3.3540350570138e-11
68.9655172413793
"chr4" 49514001 49515000 "*" 0 0 -68.5259928963513
"chr4" 49526001 49527000 "*" 0 0 -62.2289972899729
"chr4" 49620001 49621000 "*" 0 0 100
"chr4" 49633001 49634000 "*" 1.43679113950856e-06 1.78554011167915e-06 81.25
"chr4" 49649001 49650000 "*" 4.32209823486573e-12 1.15935290479641e-11 -100
"chr4" 52733001 52734000 "*" 1.20281562487889e-12 3.46585342181251e-12 100
"chr4" 52862001 52863000 "*" 0.000229234810879997 0.000202707360857361
54.1666666666667
"chr4" 52917001 52918000 "*" 0 0 75.5089487678082
"chr4" 53617001 53618000 "*" 0 0 63.5538550518308
"chr4" 53737001 53738000 "*" 2.92612156727046e-10 6.11125079829759e-10
55.1724137931034
"chr4" 53767001 53768000 "*" 0 0 100
"chr4" 53860001 53861000 "*" 3.76365605347928e-14 1.3043487970679e-13 100
"chr4" 53919001 53920000 "*" 7.76467778962342e-12 2.01455759651491e-11
-94.4444444444444
"chr4" 54374001 54375000 "*" 0 0 66.6666666666667
"chr4" 54560001 54561000 "*" 2.08814465718632e-09 3.86232567960463e-09 -100
"chr4" 54568001 54569000 "*" 0 0 -100
"chr4" 54962001 54963000 "*" 1.19160237233018e-11 3.01317289318662e-11
-66.6666666666667
"chr4" 54965001 54966000 "*" 0 0 58.3333333333333
"chr4" 55096001 55097000 "*" 0 0 64.9619549616637
"chr4" 55100001 55101000 "*" 0 0 67.7069732445172
"chr4" 55143001 55144000 "*" 3.6700841921089e-08 5.65530226072258e-08 -100
"chr4" 55184001 55185000 "*" 3.9190872769268e-14 1.35416418933905e-13 100
"chr4" 55420001 55421000 "*" 1.65090882311425e-05 1.75053966080746e-05

-58.5365853658537
"chr4" 55524001 55525000 "*" 0 0 82.4
"chr4" 55575001 55576000 "*" 6.42819131257966e-14 2.16647391790064e-13
-93.5064935064935
"chr4" 55604001 55605000 "*" 0 0 100
"chr4" 55731001 55732000 "*" 1.12458486967171e-09 2.15740815043925e-09 -100
"chr4" 56276001 56277000 "*" 7.67056418382595e-11 1.7359448580318e-10 -100
"chr4" 57218001 57219000 "*" 6.18660678242122e-11 1.41987208654994e-10
-66.6666666666667
"chr4" 57372001 57373000 "*" 0 0 70.2127659574468
"chr4" 57396001 57397000 "*" 0 0 90.1574803149606
"chr4" 57501001 57502000 "*" 2.03463942582971e-07 2.83279970699238e-07 -62.5
"chr4" 57593001 57594000 "*" 1.5277158427196e-09 2.87642564232045e-09 -100
"chr4" 57687001 57688000 "*" 0 0 59.514348785872
"chr4" 57709001 57710000 "*" 0 0 -100
"chr4" 57918001 57919000 "*" 1.17939143751133e-06 1.48285273216832e-06
66.0377358490566
"chr4" 57953001 57954000 "*" 3.6700841921089e-08 5.65530226072258e-08 100
"chr4" 58596001 58597000 "*" 0 0 -50.2325884705678
"chr4" 58970001 58971000 "*" 3.95353005888666e-09 7.03928529283733e-09 -100
"chr4" 60907001 60908000 "*" 0 0 -70.7317073170732
"chr4" 61978001 61979000 "*" 3.56775498033812e-10 7.35898629809829e-10 100
"chr4" 62529001 62530000 "*" 8.7349629751543e-09 1.47505871898511e-08 100
"chr4" 63413001 63414000 "*" 1.11022302462516e-16 5.03662826618488e-16 100
"chr4" 63438001 63439000 "*" 3.63445940010365e-11 8.58565209111204e-11 100
"chr4" 63598001 63599000 "*" 0 0 -75.1304347826087
"chr4" 64787001 64788000 "*" 1.4432899320127e-15 5.90750815956055e-15 -100
"chr4" 65323001 65324000 "*" 3.88022947106492e-13 1.1867462770515e-12 -100
"chr4" 65349001 65350000 "*" 1.89581683684992e-12 5.32311367606535e-12 -100
"chr4" 65508001 65509000 "*" 0 0 100
"chr4" 65614001 65615000 "*" 4.63584393095573e-10 9.43535904233645e-10
77.7777777777778
"chr4" 65803001 65804000 "*" 1.22839738381231e-10 2.70807533413599e-10
91.6666666666667
"chr4" 66533001 66534000 "*" 1.4432899320127e-15 5.90750815956055e-15 -100
"chr4" 66959001 66960000 "*" 4.73234496034536e-09 8.30627300264826e-09 100
"chr4" 67019001 67020000 "*" 6.65646821215171e-05 6.41959941692905e-05
-53.8461538461538
"chr4" 67237001 67238000 "*" 3.04324343503026e-11 7.26599935673121e-11 100
"chr4" 68141001 68142000 "*" 2.23308815183731e-06 2.69938122569763e-06
64.1025641025641
"chr4" 68148001 68149000 "*" 2.31469803679829e-06 2.79034791839104e-06 -75
"chr4" 68278001 68279000 "*" 4.21884749357559e-15 1.63874445239189e-14 100
"chr4" 68792001 68793000 "*" 2.88779000712225e-11 6.92935809466068e-11 -100
"chr4" 68830001 68831000 "*" 1.35003119794419e-13 4.3679847476192e-13 100
"chr4" 70058001 70059000 "*" 2.43005615629954e-09 4.45014222280278e-09 100
"chr4" 70133001 70134000 "*" 2.66279684968396e-08 4.19936300492313e-08
57.1428571428571
"chr4" 70181001 70182000 "*" 1.35036426485158e-12 3.86629547363774e-12 100
"chr4" 70524001 70525000 "*" 7.78879582052916e-07 1.00381845714364e-06
56.7567567567568
"chr4" 70790001 70791000 "*" 1.18948405458674e-07 1.70767216681657e-07

65.5555555555556
"chr4" 71165001 71166000 "*" 6.77335965093562e-12 1.76897011246462e-11 -100
"chr4" 71897001 71898000 "*" 2.88710388929303e-10 6.03399264731139e-10 -100
"chr4" 72052001 72053000 "*" 0 0 52.8824833702882
"chr4" 72597001 72598000 "*" 0 0 -100
"chr4" 72679001 72680000 "*" 1.12458486967171e-09 2.15740815043925e-09 100
"chr4" 73008001 73009000 "*" 4.79616346638068e-14 1.64380445416596e-13 100
"chr4" 73596001 73597000 "*" 2.88710388929303e-10 6.03399264731139e-10 100
"chr4" 73935001 73936000 "*" 0 0 58.4795321637427
"chr4" 74342001 74343000 "*" 2.44637754498456e-10 5.17159059877136e-10 53
"chr4" 74486001 74487000 "*" 2.8332891588434e-13 8.80789811257736e-13
-52.3809523809524
"chr4" 74735001 74736000 "*" 4.56276173510517e-08 6.95986372109215e-08
-78.1906300484653
"chr4" 74964001 74965000 "*" 0 0 -57.7685070122493
"chr4" 75719001 75720000 "*" 0 0 -84.1320553780618
"chr4" 75938001 75939000 "*" 0 0 -89.1891891891892
"chr4" 76326001 76327000 "*" 0 0 -85.981308411215
"chr4" 76792001 76793000 "*" 1.46549439250521e-14 5.33330076973935e-14 100
"chr4" 76794001 76795000 "*" 8.01666433236647e-09 1.36392368970109e-08 -100
"chr4" 76812001 76813000 "*" 2.37587727269783e-14 8.44268514944447e-14 100
"chr4" 76861001 76862000 "*" 0 0 -91.4335664335664
"chr4" 76862001 76863000 "*" 0 0 -65.6040268456376
"chr4" 76987001 76988000 "*" 0.00020976332305167 0.000186660769941092
-52.3809523809524
"chr4" 77228001 77229000 "*" 7.105427357601e-15 2.68362283258525e-14 -100
"chr4" 78300001 78301000 "*" 1.92946192356658e-09 3.59193011611483e-09 100
"chr4" 78474001 78475000 "*" 1.14124265593318e-11 2.89138062669327e-11 100
"chr4" 80555001 80556000 "*" 2.00227675550835e-08 3.20459176617961e-08 100
"chr4" 80644001 80645000 "*" 5.55111512312578e-15 2.12516350804551e-14 -100
"chr4" 81000001 81001000 "*" 2.58681964737661e-14 9.14008479605639e-14 100
"chr4" 81123001 81124000 "*" 0 0 85.6386109476162
"chr4" 81257001 81258000 "*" 0 0 90.2214022140221
"chr4" 82310001 82311000 "*" 1.49613654798486e-12 4.26244554429779e-12 -100
"chr4" 83482001 83483000 "*" 0 0 51.6771216321471
"chr4" 83660001 83661000 "*" 2.69018141096922e-12 7.41487347763769e-12 100
"chr4" 84430001 84431000 "*" 3.33066907387547e-16 1.4495649018245e-15 -100
"chr4" 84469001 84470000 "*" 9.80763359414993e-10 1.8980032424715e-09 -100
"chr4" 84486001 84487000 "*" 4.96332553372447e-05 4.88430452861491e-05
61.2903225806452
"chr4" 84953001 84954000 "*" 1.12458486967171e-09 2.15740815043925e-09 100
"chr4" 85420001 85421000 "*" 0 0 79.1228070175439
"chr4" 85464001 85465000 "*" 0 0 -58.5106382978723
"chr4" 85955001 85956000 "*" 2.00227675550835e-08 3.20459176617961e-08 100
"chr4" 87154001 87155000 "*" 3.34128893442198e-08 5.19772864618523e-08 -100
"chr4" 87280001 87281000 "*" 2.68648345902456e-07 3.68335157375939e-07
75.8064516129032
"chr4" 87515001 87516000 "*" 0 0 54.4378698224852
"chr4" 87567001 87568000 "*" 2.17381668221606e-13 6.85151076607736e-13 -100
"chr4" 87620001 87621000 "*" 8.7349629751543e-09 1.47505871898511e-08 100
"chr4" 88420001 88421000 "*" 1.39779633423487e-07 1.98463037958589e-07 100
"chr4" 88667001 88668000 "*" 3.33066907387547e-16 1.4495649018245e-15 -100

"chr4" 88928001 88929000 "*" 0 0 76.7441860465116
"chr4" 89079001 89080000 "*" 6.30384633382164e-13 1.87665599796749e-12 -100
"chr4" 89445001 89446000 "*" 2.64951482975562e-09 4.82186011375752e-09 100
"chr4" 89534001 89535000 "*" 6.59550958292954e-09 1.13456194382773e-08 100
"chr4" 89579001 89580000 "*" 1.07882591748876e-11 2.74553537455803e-11 -100
"chr4" 89847001 89848000 "*" 2.00227675550835e-08 3.20459176617961e-08 100
"chr4" 90059001 90060000 "*" 7.2106471171729e-07 9.33678515696183e-07
-50.9433962264151
"chr4" 90410001 90411000 "*" 5.45632428128329e-11 1.26115136754623e-10
64.4444444444444
"chr4" 90550001 90551000 "*" 3.56775498033812e-10 7.35898629809829e-10 100
"chr4" 91048001 91049000 "*" 0 0 -84.8966613672496
"chr4" 91491001 91492000 "*" 4.73234496034536e-09 8.30627300264826e-09 100
"chr4" 92326001 92327000 "*" 3.40125705378114e-10 7.03784073980162e-10 100
"chr4" 92520001 92521000 "*" 0 0 -100
"chr4" 95087001 95088000 "*" 2.1658988651474e-06 2.62321901854342e-06
66.6666666666667
"chr4" 95461001 95462000 "*" 0.000170900372238547 0.00015425218250759
53.8461538461538
"chr4" 95539001 95540000 "*" 8.47441006257554e-11 1.90771892038926e-10 -53.125
"chr4" 95678001 95679000 "*" 0 0 78.4431137724551
"chr4" 95679001 95680000 "*" 0 0 79.2649453982325
"chr4" 95868001 95869000 "*" 2.32788939891204e-06 2.80535789292305e-06
-51.5151515151515
"chr4" 96146001 96147000 "*" 1.13156151115845e-11 2.87188517375303e-11
-86.9047619047619
"chr4" 96171001 96172000 "*" 9.27052878907375e-11 2.07433950121104e-10
-68.2926829268293
"chr4" 96219001 96220000 "*" 3.7274627828765e-12 1.00985250136599e-11 -100
"chr4" 96468001 96469000 "*" 0 0 79.7297297297297
"chr4" 98184001 98185000 "*" 5.01025332333427e-10 1.01358031135889e-09 100
"chr4" 99064001 99065000 "*" 4.44089209850063e-16 1.91071758245033e-15 75
"chr4" 99161001 99162000 "*" 8.25604029586202e-11 1.86283449634714e-10 80
"chr4" 99722001 99723000 "*" 0 0 100
"chr4" 99858001 99859000 "*" 1.74853465040314e-11 4.32752710900609e-11 -100
"chr4" 100008001 100009000 "*" 5.55111512312578e-15 2.12516350804551e-14
88.2352941176471
"chr4" 100575001 100576000 "*" 2.03914842789032e-08 3.25953809507838e-08
-68.3453237410072
"chr4" 101165001 101166000 "*" 6.70841160399505e-12 1.75486545176836e-11 -100
"chr4" 101719001 101720000 "*" 6.4152538836737e-10 1.27477697728417e-09 -100
"chr4" 102267001 102268000 "*" 1.11022302462516e-16 5.03662826618488e-16
-62.1359223300971
"chr4" 103158001 103159000 "*" 4.73234496034536e-09 8.30627300264826e-09 100
"chr4" 103940001 103941000 "*" 0 0 -71.3513513513514
"chr4" 104228001 104229000 "*" 1.35893490389449e-05 1.45920069681021e-05
50.8064516129032
"chr4" 104467001 104468000 "*" 7.0006535457523e-08 1.03695832512054e-07 -100
"chr4" 106372001 106373000 "*" 2.79987144580218e-12 7.68634726811898e-12 100
"chr4" 106816001 106817000 "*" 0 0 59.0145824127265
"chr4" 107678001 107679000 "*" 4.59062171609403e-08 6.99952857751411e-08
-63.6363636363636

"chr4" 108852001 108853000 "*" 0 0 -52.8010862186015
"chr4" 108853001 108854000 "*" 0 0 71.5670359052712
"chr4" 108980001 108981000 "*" 1.90181204118289e-13 6.05298617539364e-13 100
"chr4" 109087001 109088000 "*" 0 0 82.8571428571429
"chr4" 109088001 109089000 "*" 0 0 70.6666666666667
"chr4" 109089001 109090000 "*" 0 0 88.8482632541133
"chr4" 109093001 109094000 "*" 0 0 75.278293135436
"chr4" 109271001 109272000 "*" 0 0 -91.796875
"chr4" 109293001 109294000 "*" 2.00227675550835e-08 3.20459176617961e-08 -100
"chr4" 109661001 109662000 "*" 5.794809077031e-12 1.52911192757929e-11 -100
"chr4" 109710001 109711000 "*" 4.69933963664904e-06 5.40777096921492e-06
67.4608150470219
"chr4" 110222001 110223000 "*" 1.11022302462516e-15 4.59817122935606e-15 -100
"chr4" 110480001 110481000 "*" 4.44089209850063e-15 1.71914916534614e-14
-76.984126984127
"chr4" 110599001 110600000 "*" 5.07371922253697e-14 1.73287898516782e-13 100
"chr4" 111531001 111532000 "*" 9.50883816130954e-12 2.44096072524629e-11
51.1904761904762
"chr4" 111745001 111746000 "*" 1.49880108324396e-14 5.44951848293151e-14 -100
"chr4" 112865001 112866000 "*" 0 0 100
"chr4" 112951001 112952000 "*" 2.08995487582797e-10 4.46944006972663e-10 100
"chr4" 113308001 113309000 "*" 3.40125705378114e-10 7.03784073980162e-10 100
"chr4" 113437001 113438000 "*" 0 0 91.726618705036
"chr4" 113867001 113868000 "*" 1.26876102179985e-05 1.36836355238707e-05
-59.0909090909091
"chr4" 115068001 115069000 "*" 5.1281201507436e-13 1.54461625452838e-12 100
"chr4" 115650001 115651000 "*" 6.35203854082445e-06 7.168860341053e-06 -70
"chr4" 116297001 116298000 "*" 7.9984455014781e-06 8.89173797399106e-06
66.6666666666667
"chr4" 117282001 117283000 "*" 1.28022768979275e-05 1.37992738751043e-05
54.7619047619048
"chr4" 117519001 117520000 "*" 0 0 78.5185185185185
"chr4" 118006001 118007000 "*" 0 0 94.6826758147513
"chr4" 118075001 118076000 "*" 6.70841160399505e-12 1.75486545176836e-11 100
"chr4" 118083001 118084000 "*" 0 0 99.3103448275862
"chr4" 118770001 118771000 "*" 9.0072393987839e-13 2.63157725646847e-12 -100
"chr4" 118954001 118955000 "*" 9.64974877781799e-10 1.87255912493232e-09
50.6140350877193
"chr4" 119512001 119513000 "*" 6.0507154842071e-14 2.04421084237304e-13
58.5714285714286
"chr4" 119554001 119555000 "*" 0 0 58.4615384615385
"chr4" 120087001 120088000 "*" 4.73234496034536e-09 8.30627300264826e-09 -100
"chr4" 120222001 120223000 "*" 0 0 -100
"chr4" 120548001 120549000 "*" 0 0 -76.9230769230769
"chr4" 120549001 120550000 "*" 0 0 -96.7741935483871
"chr4" 121317001 121318000 "*" 7.03992419914812e-12 1.83313429961219e-11 -100
"chr4" 121577001 121578000 "*" 7.0006535457523e-08 1.03695832512054e-07 -100
"chr4" 122193001 122194000 "*" 2.88431048933724e-07 3.93691762673522e-07
-56.6037735849057
"chr4" 122343001 122344000 "*" 9.13713549266504e-14 3.02110477479709e-13
50.3597122302158
"chr4" 122471001 122472000 "*" 0 0 100

"chr4" 122622001 122623000 "*" 3.6700841921089e-08 5.65530226072258e-08 -100
"chr4" 122872001 122873000 "*" 0 0 66.8735119047619
"chr4" 122873001 122874000 "*" 0 0 75.2808988764045
"chr4" 122969001 122970000 "*" 1.19904086659517e-14 4.40901388691796e-14 100
"chr4" 123727001 123728000 "*" 3.6700841921089e-08 5.65530226072258e-08 -100
"chr4" 124320001 124321000 "*" 0 0 -100
"chr4" 125798001 125799000 "*" 5.10702591327572e-15 1.96271840288234e-14 -100
"chr4" 128501001 128502000 "*" 1.11022302462516e-15 4.59817122935606e-15
99.1869918699187
"chr4" 128702001 128703000 "*" 0 0 -87.1794871794872
"chr4" 128765001 128766000 "*" 4.14275458382463e-08 6.34857724979108e-08
57.1428571428571
"chr4" 129581001 129582000 "*" 9.0072393987839e-13 2.63157725646847e-12 -100
"chr4" 131271001 131272000 "*" 2.06501482580279e-14 7.3890370570066e-14 100
"chr4" 131773001 131774000 "*" 3.12379488263126e-05 3.1728595486819e-05
-51.6129032258064
"chr4" 132536001 132537000 "*" 0 0 100
"chr4" 134454001 134455000 "*" 1.13140401492018e-08 1.87801380787728e-08 -100
"chr4" 134681001 134682000 "*" 2.66453525910038e-15 1.05861327033776e-14 100
"chr4" 135887001 135888000 "*" 3.52704532247117e-11 8.35581292290093e-11 100
"chr4" 136329001 136330000 "*" 3.10862446895044e-15 1.22466437093774e-14 100
"chr4" 137744001 137745000 "*" 0 0 82.8571428571429
"chr4" 137867001 137868000 "*" 1.96751503978021e-11 4.83178132611025e-11
61.7647058823529
"chr4" 138620001 138621000 "*" 1.29037891483108e-11 3.24582280286866e-11 100
"chr4" 139688001 139689000 "*" 5.794809077031e-12 1.52911192757929e-11 100
"chr4" 139692001 139693000 "*" 8.7349629751543e-09 1.47505871898511e-08 -100
"chr4" 139850001 139851000 "*" 3.6828107230491e-10 7.5865479699098e-10
-80.9523809523809
"chr4" 140414001 140415000 "*" 1.67299207820548e-08 2.71377429441055e-08 100
"chr4" 140554001 140555000 "*" 9.65729496371637e-10 1.87255912493232e-09 100
"chr4" 140649001 140650000 "*" 4.44089209850063e-16 1.91071758245033e-15 100
"chr4" 140655001 140656000 "*" 2.15716333684668e-13 6.81679773304372e-13
64.1509433962264
"chr4" 141067001 141068000 "*" 5.99964522507435e-13 1.79332876977318e-12 97.5
"chr4" 141192001 141193000 "*" 0 0 100
"chr4" 141208001 141209000 "*" 2.08814465718632e-09 3.86232567960463e-09 -100
"chr4" 141274001 141275000 "*" 3.63445940010365e-11 8.58565209111204e-11 -100
"chr4" 141295001 141296000 "*" 0 0 96.3917525773196
"chr4" 141348001 141349000 "*" 0 0 53.49143709049
"chr4" 141488001 141489000 "*" 2.85418764178758e-08 4.48575831312927e-08 -60
"chr4" 141489001 141490000 "*" 0 0 64.5299145299145
"chr4" 141511001 141512000 "*" 2.41784284078506e-05 2.49875818383709e-05
-67.741935483871
"chr4" 141529001 141530000 "*" 6.24654217240561e-10 1.2462108191303e-09 100
"chr4" 141851001 141852000 "*" 0 0 100
"chr4" 141951001 141952000 "*" 1.12132525487141e-14 4.14352271980498e-14 100
"chr4" 142536001 142537000 "*" 1.46549439250521e-14 5.33330076973935e-14 100
"chr4" 142558001 142559000 "*" 0 0 -100
"chr4" 143569001 143570000 "*" 1.13140401492018e-08 1.87801380787728e-08 100
"chr4" 143617001 143618000 "*" 6.59550958292954e-09 1.13456194382773e-08 100
"chr4" 143768001 143769000 "*" 0 0 65.5594405594406

"chr4" 144220001 144221000 "*" 2.00227675550835e-08 3.20459176617961e-08 -100
"chr4" 145243001 145244000 "*" 1.98365768255826e-11 4.85612402473442e-11 100
"chr4" 145567001 145568000 "*" 0 0 61.8615874443423
"chr4" 145631001 145632000 "*" 1.92946192356658e-09 3.59193011611483e-09 100
"chr4" 145801001 145802000 "*" 1.29867794296246e-09 2.47408848239392e-09 70
"chr4" 146191001 146192000 "*" 2.4535928844216e-14 8.69144396197119e-14 -100
"chr4" 146300001 146301000 "*" 7.31636085049558e-10 1.44375732115544e-09 -75
"chr4" 146803001 146804000 "*" 4.08209022140227e-11 9.57770690255255e-11 100
"chr4" 147133001 147134000 "*" 2.64951482975562e-09 4.82186011375752e-09 100
"chr4" 147468001 147469000 "*" 2.88779000712225e-11 6.92935809466068e-11 100
"chr4" 148385001 148386000 "*" 2.43005615629954e-09 4.45014222280278e-09 100
"chr4" 148991001 148992000 "*" 2.08814465718632e-09 3.86232567960463e-09 100
"chr4" 149606001 149607000 "*" 7.7715611723761e-16 3.26213507634405e-15 -100
"chr4" 150066001 150067000 "*" 1.39779633423487e-07 1.98463037958589e-07 100
"chr4" 151000001 151001000 "*" 0 0 65.0485436893204
"chr4" 151242001 151243000 "*" 6.59550958292954e-09 1.13456194382773e-08 -100
"chr4" 151713001 151714000 "*" 3.88022947106492e-13 1.1867462770515e-12 -100
"chr4" 152123001 152124000 "*" 1.67377583903949e-07 2.35442859751953e-07
83.3333333333333
"chr4" 152762001 152763000 "*" 0 0 -76.7441860465116
"chr4" 152781001 152782000 "*" 2.31469803679829e-06 2.79034791839104e-06 75
"chr4" 152857001 152858000 "*" 1.55431223447522e-14 5.6388152448458e-14 100
"chr4" 152945001 152946000 "*" 1.33366451215977e-09 2.5381897183578e-09
-71.0526315789474
"chr4" 153111001 153112000 "*" 7.0006535457523e-08 1.03695832512054e-07 -100
"chr4" 153165001 153166000 "*" 0 0 -100
"chr4" 153630001 153631000 "*" 2.00227675550835e-08 3.20459176617961e-08 100
"chr4" 153856001 153857000 "*" 0 0 83.402489626556
"chr4" 153948001 153949000 "*" 3.46525030892053e-11 8.22600663018898e-11
52.7777777777778
"chr4" 154435001 154436000 "*" 3.46500605985511e-13 1.06777978272295e-12 -100
"chr4" 154604001 154605000 "*" 5.85495285587712e-09 1.01713703737891e-08 -87.5
"chr4" 154926001 154927000 "*" 1.25284005392245e-10 2.75649590272477e-10 -100
"chr4" 155338001 155339000 "*" 9.99519578215313e-10 1.93237278235505e-09
73.134328358209
"chr4" 155923001 155924000 "*" 5.95647872136951e-08 8.94637798673435e-08
-51.7241379310345
"chr4" 156417001 156418000 "*" 1.23966884502114e-05 1.33880538279549e-05
73.3333333333333
"chr4" 156557001 156558000 "*" 1.4432899320127e-15 5.90750815956055e-15 -100
"chr4" 156871001 156872000 "*" 4.01313771103418e-09 7.11882087237587e-09 100
"chr4" 157892001 157893000 "*" 0 0 96.8901846452867
"chr4" 157893001 157894000 "*" 0 0 94.8863636363636
"chr4" 158125001 158126000 "*" 2.22044604925031e-16 9.81641919380259e-16
50.6387921022067
"chr4" 158126001 158127000 "*" 1.20262658387382e-07 1.72594592069524e-07
66.6666666666667
"chr4" 158152001 158153000 "*" 2.61657427258477e-06 3.12843692934624e-06 52
"chr4" 158549001 158550000 "*" 3.63445940010365e-11 8.58565209111204e-11 100
"chr4" 159309001 159310000 "*" 2.88779000712225e-11 6.92935809466068e-11 100
"chr4" 159829001 159830000 "*" 8.7349629751543e-09 1.47505871898511e-08 -100
"chr4" 160326001 160327000 "*" 2.18158824338843e-13 6.87314823207509e-13 -100

"chr4" 161327001 161328000 "*" 4.86832796298131e-13 1.47290845675726e-12 100
"chr4" 161975001 161976000 "*" 0 0 100
"chr4" 162070001 162071000 "*" 2.08995487582797e-10 4.46944006972663e-10 100
"chr4" 162687001 162688000 "*" 6.35875241350448e-10 1.26753599068815e-09 -82.2222222222222
"chr4" 163085001 163086000 "*" 0 0 100
"chr4" 163661001 163662000 "*" 0 0 -57.6190476190476
"chr4" 164253001 164254000 "*" 0 0 66.3475177304965
"chr4" 164415001 164416000 "*" 0 0 100
"chr4" 165932001 165933000 "*" 2.48682086256835e-06 2.98236493157237e-06 -60
"chr4" 166300001 166301000 "*" 0 0 53.0069651192857
"chr4" 166433001 166434000 "*" 1.25284005392245e-10 2.75649590272477e-10 100
"chr4" 166437001 166438000 "*" 0 0 100
"chr4" 166793001 166794000 "*" 1.0325074129014e-14 3.83008663711937e-14 -100
"chr4" 168273001 168274000 "*" 1.11022302462516e-16 5.03662826618488e-16 -100
"chr4" 169558001 169559000 "*" 3.95353005888666e-09 7.03928529283733e-09 100
"chr4" 169640001 169641000 "*" 0 0 100
"chr4" 169900001 169901000 "*" 9.62896429257398e-13 2.80291205576417e-12 -100
"chr4" 170079001 170080000 "*" 5.6362137179633e-11 1.29963270300065e-10 -100
"chr4" 171123001 171124000 "*" 2.88779000712225e-11 6.92935809466068e-11 100
"chr4" 172640001 172641000 "*" 6.59550958292954e-09 1.13456194382773e-08 -100
"chr4" 173409001 173410000 "*" 6.24654217240561e-10 1.2462108191303e-09 100
"chr4" 174601001 174602000 "*" 8.99589425173986e-09 1.51691033251272e-08 72.972972972973
"chr4" 174803001 174804000 "*" 1.25284005392245e-10 2.75649590272477e-10 -100
"chr4" 175473001 175474000 "*" 9.08108033215171e-11 2.03407753381119e-10 -100
"chr4" 176987001 176988000 "*" 0 0 72.7461447212337
"chr4" 177116001 177117000 "*" 0 0 82.6086956521739
"chr4" 178054001 178055000 "*" 8.25450818808804e-13 2.42473843346228e-12 100
"chr4" 179132001 179133000 "*" 9.08108033215171e-11 2.03407753381119e-10 100
"chr4" 179162001 179163000 "*" 3.33066907387547e-16 1.4495649018245e-15 100
"chr4" 179336001 179337000 "*" 5.63660229602192e-13 1.68905058935478e-12 100
"chr4" 179734001 179735000 "*" 1.82244885849059e-10 3.92971469542746e-10 58.4615384615385
"chr4" 180276001 180277000 "*" 2.88710388929303e-10 6.03399264731139e-10 -100
"chr4" 180530001 180531000 "*" 1.52794177310511e-09 2.87642564232045e-09 -100
"chr4" 180978001 180979000 "*" 8.3882012447134e-11 1.88922989503423e-10 100
"chr4" 180979001 180980000 "*" 0 0 93.8693913816082
"chr4" 180980001 180981000 "*" 0 0 88.9176928062217
"chr4" 181055001 181056000 "*" 1.51914609258785e-06 1.88121349342686e-06 73.6842105263158
"chr4" 181064001 181065000 "*" 3.88169574261354e-09 6.93447853396753e-09 66.6666666666667
"chr4" 181487001 181488000 "*" 7.0006535457523e-08 1.03695832512054e-07 100
"chr4" 181488001 181489000 "*" 1.11022302462516e-16 5.03662826618488e-16 -100
"chr4" 181489001 181490000 "*" 4.1943348794149e-10 8.58599117047547e-10 61.1344537815126
"chr4" 181564001 181565000 "*" 4.02167188440217e-12 1.08266700497926e-11 -100
"chr4" 181735001 181736000 "*" 2.22044604925031e-16 9.81641919380259e-16 100
"chr4" 181889001 181890000 "*" 3.88022947106492e-13 1.1867462770515e-12 100
"chr4" 182139001 182140000 "*" 6.77335965093562e-12 1.76897011246462e-11 -100
"chr4" 182492001 182493000 "*" 2.15125472990962e-10 4.58126659943874e-10 -100

"chr4" 182765001 182766000 "*" 0 0 -100
"chr4" 182795001 182796000 "*" 0 0 -100
"chr4" 183115001 183116000 "*" 0 0 100
"chr4" 183131001 183132000 "*" 8.88178419700125e-16 3.70670032207938e-15
-63.3333333333333
"chr4" 183196001 183197000 "*" 9.08108033215171e-11 2.03407753381119e-10 100
"chr4" 183212001 183213000 "*" 2.89801516117905e-12 7.94270405097209e-12
-66.6666666666667
"chr4" 183642001 183643000 "*" 0 0 100
"chr4" 183688001 183689000 "*" 3.33066907387547e-16 1.4495649018245e-15 -100
"chr4" 183726001 183727000 "*" 6.77335965093562e-12 1.76897011246462e-11 -100
"chr4" 183757001 183758000 "*" 2.28067464913551e-09 4.19951877674167e-09
50.1041666666667
"chr4" 183762001 183763000 "*" 9.08108033215171e-11 2.03407753381119e-10 -100
"chr4" 183766001 183767000 "*" 3.33066907387547e-16 1.4495649018245e-15
71.8181818181818
"chr4" 183777001 183778000 "*" 4.08209022140227e-11 9.57770690255255e-11 100
"chr4" 183817001 183818000 "*" 1.52331027680752e-07 2.15297901653729e-07
56.5217391304348
"chr4" 183834001 183835000 "*" 5.17893470552977e-07 6.83168992790292e-07
66.6666666666667
"chr4" 183981001 183982000 "*" 3.76365605347928e-14 1.3043487970679e-13 -100
"chr4" 184115001 184116000 "*" 8.01666433236647e-09 1.36392368970109e-08 100
"chr4" 184245001 184246000 "*" 9.61897228535236e-13 2.801372325071e-12 100
"chr4" 184311001 184312000 "*" 1.5634673446896e-05 1.66336190218994e-05
54.5454545454545
"chr4" 184403001 184404000 "*" 0 0 -65.9574468085106
"chr4" 184435001 184436000 "*" 4.03835798135077e-09 7.16066482954804e-09
-81.8181818181818
"chr4" 184642001 184643000 "*" 0 0 100
"chr4" 184718001 184719000 "*" 0 0 66.7077031415538
"chr4" 184719001 184720000 "*" 0 0 65.3723459309203
"chr4" 184739001 184740000 "*" 0 0 73.9130434782609
"chr4" 184749001 184750000 "*" 1.75082170983387e-13 5.6014942024636e-13 100
"chr4" 184800001 184801000 "*" 1.62092561595273e-14 5.87307003677525e-14
64.2857142857143
"chr4" 184886001 184887000 "*" 3.77475828372553e-15 1.47379557022071e-14 100
"chr4" 184922001 184923000 "*" 2.66453525910038e-15 1.05861327033776e-14
60.3174603174603
"chr4" 184938001 184939000 "*" 4.52464326521351e-05 4.47913781470911e-05
51.063829787234
"chr4" 184949001 184950000 "*" 6.99440505513849e-14 2.34592719571672e-13
67.7272727272727
"chr4" 184955001 184956000 "*" 8.7349629751543e-09 1.47505871898511e-08 -100
"chr4" 185019001 185020000 "*" 0 0 -83.75
"chr4" 185073001 185074000 "*" 4.22135256750167e-08 6.46213810841317e-08
-92.3076923076923
"chr4" 185090001 185091000 "*" 9.31699162265431e-13 2.7181920310254e-12
-53.2019704433498
"chr4" 185239001 185240000 "*" 3.6700841921089e-08 5.65530226072258e-08 100
"chr4" 185269001 185270000 "*" 0 0 100
"chr4" 185410001 185411000 "*" 1.13140401492018e-08 1.87801380787728e-08 -100

"chr4" 185434001 185435000 "*" 2.455369241261e-12 6.8003688121118e-12 -100
"chr4" 185470001 185471000 "*" 3.86299240373589e-08 5.93895182974888e-08
79.3893129770992
"chr4" 185501001 185502000 "*" 2.88779000712225e-11 6.92935809466068e-11 100
"chr4" 185654001 185655000 "*" 0 0 -100
"chr4" 185669001 185670000 "*" 3.30369065437708e-12 9.00336905360004e-12 80
"chr4" 185684001 185685000 "*" 6.11364625591193e-10 1.22332619666323e-09
96.6666666666667
"chr4" 185779001 185780000 "*" 6.66133814775094e-15 2.52791665956799e-14 -100
"chr4" 185790001 185791000 "*" 1.0325074129014e-14 3.83008663711937e-14 -100
"chr4" 185802001 185803000 "*" 5.295763827462e-14 1.80214784640623e-13 100
"chr4" 185803001 185804000 "*" 3.05637626318855e-10 6.37020893543828e-10
-66.6666666666667
"chr4" 185811001 185812000 "*" 2.79440914852103e-11 6.73046092948135e-11 100
"chr4" 185819001 185820000 "*" 5.10702591327572e-14 1.74125964028248e-13
-56.8181818181818
"chr4" 185880001 185881000 "*" 1.12055920098442e-11 2.84500040664577e-11 -100
"chr4" 185890001 185891000 "*" 5.63660229602192e-13 1.68905058935478e-12 100
"chr4" 185906001 185907000 "*" 0 0 100
"chr4" 185914001 185915000 "*" 4.63838322639631e-07 6.15651140119366e-07
73.3333333333333
"chr4" 185954001 185955000 "*" 2.93384128102758e-09 5.3134507630004e-09
74.2424242424242
"chr4" 185984001 185985000 "*" 1.65900401183361e-06 2.04236557510993e-06
66.6666666666667
"chr4" 186318001 186319000 "*" 6.70463462526527e-10 1.32889205920977e-09 52
"chr4" 186391001 186392000 "*" 2.82773804372027e-13 8.79129664109438e-13 100
"chr4" 186456001 186457000 "*" 0 0 62.7012637115915
"chr4" 186631001 186632000 "*" 2.88657986402541e-15 1.14155613124573e-14 -100
"chr4" 186877001 186878000 "*" 0 0 100
"chr4" 187085001 187086000 "*" 1.11022302462516e-16 5.03662826618488e-16 100
"chr4" 187126001 187127000 "*" 0 0 100
"chr4" 187186001 187187000 "*" 0 0 100
"chr4" 187194001 187195000 "*" 6.27196450553313e-07 8.18806291294633e-07
78.2608695652174
"chr4" 187214001 187215000 "*" 0 0 80.5895344886171
"chr4" 187292001 187293000 "*" 0 0 100
"chr4" 187421001 187422000 "*" 0 0 100
"chr4" 187459001 187460000 "*" 4.44089209850063e-16 1.91071758245033e-15 77
"chr4" 187488001 187489000 "*" 8.1415238173399e-07 1.0463674424606e-06
82.3529411764706
"chr4" 187507001 187508000 "*" 2.04117044155794e-07 2.84127576499834e-07
52.9411764705882
"chr4" 187553001 187554000 "*" 9.1506337806968e-05 8.63421053552552e-05 70
"chr4" 187742001 187743000 "*" 9.22717424689523e-09 1.55374184879414e-08
-70.5882352941177
"chr4" 188012001 188013000 "*" 0.000106439195992625 9.93653667048631e-05
55.5555555555556
"chr4" 188109001 188110000 "*" 1.51238141588017e-06 1.87358759645321e-06
54.6666666666667
"chr4" 188268001 188269000 "*" 0 0 -69.4736842105263
"chr4" 188667001 188668000 "*" 2.84063537536738e-06 3.37908416856722e-06

67.5675675675676
"chr4" 188710001 188711000 "*" 6.40654973516064e-10 1.27477697728417e-09
61.7977528089888
"chr4" 188891001 188892000 "*" 1.13140401492018e-08 1.87801380787728e-08 -100
"chr4" 188917001 188918000 "*" 6.7390537594747e-14 2.26447131529591e-13 -100
"chr4" 188954001 188955000 "*" 3.95353005888666e-09 7.03928529283733e-09 100
"chr4" 188977001 188978000 "*" 3.40125705378114e-10 7.03784073980162e-10 -100
"chr4" 189087001 189088000 "*" 9.08108033215171e-11 2.03407753381119e-10 100
"chr4" 189099001 189100000 "*" 0 0 100
"chr4" 189265001 189266000 "*" 0 0 100
"chr4" 189379001 189380000 "*" 1.92650087800228e-05 2.02005979454001e-05
-53.5714285714286
"chr4" 189745001 189746000 "*" 2.22044604925031e-16 9.81641919380259e-16 -60
"chr4" 189794001 189795000 "*" 4.13447054370408e-13 1.26039083611961e-12
54.3695014662757
"chr4" 189804001 189805000 "*" 5.42243361323358e-10 1.0930673207541e-09
80.7142857142857
"chr4" 189869001 189870000 "*" 4.44089209850063e-16 1.91071758245033e-15 100
"chr4" 190158001 190159000 "*" 2.03613792493229e-11 4.97992146820484e-11
-56.5217391304348
"chr4" 190412001 190413000 "*" 0.000349595182828621 0.000299726368053657
58.3333333333333
"chr4" 190462001 190463000 "*" 9.24239252028114e-05 8.71605750827097e-05
50.6493506493507
"chr4" 190580001 190581000 "*" 4.83921813732024e-09 8.48582169090024e-09
66.6666666666667
"chr4" 190583001 190584000 "*" 3.67272878776248e-12 9.96318438329627e-12
58.1395348837209
"chr4" 190590001 190591000 "*" 8.35053628467364e-08 1.22420950660501e-07
-65.8536585365854
"chr4" 190599001 190600000 "*" 6.52733422867868e-12 1.71146951718423e-11 100
"chr4" 190637001 190638000 "*" 3.6700841921089e-08 5.65530226072258e-08 100
"chr4" 190653001 190654000 "*" 0 0 100
"chr4" 190741001 190742000 "*" 2.73625566649116e-12 7.52483363579137e-12 -100
"chr4" 190749001 190750000 "*" 5.7065463465733e-14 1.93318145576218e-13 100
"chr4" 190760001 190761000 "*" 0 0 100
"chr4" 190769001 190770000 "*" 9.32032229172819e-13 2.7181920310254e-12 -100
"chr4" 190838001 190839000 "*" 7.64943663966733e-13 2.25797532092373e-12
94.6524064171123
"chr4" 190862001 190863000 "*" 6.15057782482609e-10 1.23030286697572e-09
-61.1111111111111
"chr4" 190868001 190869000 "*" 1.39779633423487e-07 1.98463037958589e-07 100
"chr4" 190939001 190940000 "*" 1.33226762955019e-15 5.48133041560118e-15 100
"chr4_gl000193_random" 3001 4000 "*" 0 0 -82.8125
"chr4_gl000194_random" 67001 68000 "*" 6.07678540820444e-09
1.05326102161257e-08 -63.2352941176471
"chr5" 54001 55000 "*" 0 0 66.116806461634
"chr5" 79001 80000 "*" 4.13002965160558e-14 1.42427783430954e-13 -100
"chr5" 89001 90000 "*" 1.70830016799073e-12 4.82486452788367e-12
-53.0612244897959
"chr5" 107001 108000 "*" 9.04570973681018e-10 1.76414389822173e-09 -100
"chr5" 225001 226000 "*" 0 0 -100

"chr5" 244001 245000 "*" 3.56775498033812e-10 7.35898629809829e-10 100
"chr5" 302001 303000 "*" 2.62234678416462e-13 8.20227833391735e-13
84.1269841269841
"chr5" 348001 349000 "*" 1.81829222789531e-08 2.93596591298279e-08
61.5384615384615
"chr5" 431001 432000 "*" 0 0 78.2783588093323
"chr5" 442001 443000 "*" 3.23963078585621e-11 7.7152331657594e-11
-52.7985074626866
"chr5" 473001 474000 "*" 0 0 74.1776315789474
"chr5" 505001 506000 "*" 4.77450709079896e-07 6.3276214692419e-07
-50.8771929824561
"chr5" 506001 507000 "*" 0 0 82.4399260628466
"chr5" 519001 520000 "*" 4.89608353859694e-14 1.67632817250644e-13
-60.1779755283649
"chr5" 566001 567000 "*" 2.49134046725885e-13 7.80773828942576e-13
-64.968364968365
"chr5" 581001 582000 "*" 1.35036426485158e-12 3.86629547363774e-12 100
"chr5" 587001 588000 "*" 1.70641278884887e-12 4.81986032810646e-12 -100
"chr5" 611001 612000 "*" 3.45191221606811e-07 4.66402949645677e-07
53.7037037037037
"chr5" 763001 764000 "*" 6.24654217240561e-10 1.2462108191303e-09 100
"chr5" 809001 810000 "*" 0 0 100
"chr5" 954001 955000 "*" 9.65729496371637e-10 1.87255912493232e-09 -100
"chr5" 991001 992000 "*" 1.95288230031565e-13 6.20723903144594e-13
-63.1578947368421
"chr5" 993001 994000 "*" 6.38526231888648e-10 1.27245544366795e-09
-88.695652173913
"chr5" 995001 996000 "*" 8.19565414095225e-05 7.7896169099937e-05
58.3333333333333
"chr5" 998001 999000 "*" 0 0 65.8119658119658
"chr5" 1006001 1007000 "*" 0.000726374117344109 0.000590081342527627
52.1739130434783
"chr5" 1010001 1011000 "*" 3.33066907387547e-16 1.4495649018245e-15
67.8571428571429
"chr5" 1098001 1099000 "*" 1.22457599616155e-13 3.97936846180533e-13
53.1914893617021
"chr5" 1138001 1139000 "*" 0 0 95.5555555555556
"chr5" 1152001 1153000 "*" 3.07753822426093e-13 9.53485764160054e-13
69.5652173913043
"chr5" 1158001 1159000 "*" 4.68383125253879e-05 4.62705854911143e-05
59.3406593406593
"chr5" 1168001 1169000 "*" 0 0 -75.2631578947368
"chr5" 1209001 1210000 "*" 0 0 -90.6384615384615
"chr5" 1237001 1238000 "*" 7.78522815392257e-06 8.67135145310036e-06 -55
"chr5" 1262001 1263000 "*" 1.37828859436695e-10 3.01445544267661e-10 -100
"chr5" 1315001 1316000 "*" 0 0 -51.5789473684211
"chr5" 1328001 1329000 "*" 7.0006535457523e-08 1.03695832512054e-07 100
"chr5" 1349001 1350000 "*" 4.06627287397043e-10 8.33714363788151e-10
-68.2539682539683
"chr5" 1392001 1393000 "*" 0 0 68.0851063829787
"chr5" 1408001 1409000 "*" 7.506772981003e-12 1.95000965118339e-11
-80.7692307692308

"chr5" 1411001 1412000 "*" 0 0 71.6014773283496
"chr5" 1420001 1421000 "*" 1.37889699658444e-13 4.45602024816198e-13 70.5882352941177
"chr5" 1516001 1517000 "*" 1.10442097427388e-05 1.20217549185931e-05 -57.6923076923077
"chr5" 1545001 1546000 "*" 5.57331958361829e-14 1.89035234335933e-13 65.5172413793103
"chr5" 1550001 1551000 "*" 1.11022302462516e-16 5.03662826618488e-16 51.6666666666667
"chr5" 1555001 1556000 "*" 0 0 -54.7368421052632
"chr5" 1560001 1561000 "*" 2.4535928844216e-14 8.69144396197119e-14 100
"chr5" 1654001 1655000 "*" 1.01141317543352e-13 3.32149014630216e-13 95.1388888888889
"chr5" 1659001 1660000 "*" 2.32862640281439e-08 3.69689652174363e-08 -56.5217391304348
"chr5" 1671001 1672000 "*" 1.14130926931466e-13 3.7276674571693e-13 -80
"chr5" 1704001 1705000 "*" 0 0 78.2312925170068
"chr5" 1763001 1764000 "*" 0 0 100
"chr5" 1778001 1779000 "*" 2.07633910065397e-12 5.796422699246e-12 79.3814432989691
"chr5" 1832001 1833000 "*" 0 0 50.1694915254237
"chr5" 1862001 1863000 "*" 2.1094237467878e-15 8.47434540879246e-15 50.9090909090909
"chr5" 1863001 1864000 "*" 1.13140401492018e-08 1.87801380787728e-08 100
"chr5" 1864001 1865000 "*" 0 0 -61.8983957219251
"chr5" 1890001 1891000 "*" 3.08737257981306e-10 6.4307948292093e-10 -50.8064516129032
"chr5" 1892001 1893000 "*" 0 0 100
"chr5" 1915001 1916000 "*" 0 0 100
"chr5" 1938001 1939000 "*" 0 0 100
"chr5" 1956001 1957000 "*" 0 0 -100
"chr5" 1966001 1967000 "*" 5.21591658753096e-11 1.20765963947665e-10 -66.8918918918919
"chr5" 2001001 2002000 "*" 0 0 -52.4984147114775
"chr5" 2016001 2017000 "*" 2.4789188368679e-08 3.92161381524865e-08 -66.6666666666667
"chr5" 2025001 2026000 "*" 0 0 100
"chr5" 2037001 2038000 "*" 2.00227675550835e-08 3.20459176617961e-08 -100
"chr5" 2042001 2043000 "*" 0 0 97.6744186046512
"chr5" 2097001 2098000 "*" 0 0 70.2877884366088
"chr5" 2135001 2136000 "*" 1.16573417585641e-14 4.29335582900508e-14 100
"chr5" 2145001 2146000 "*" 1.27376832943504e-05 1.373302218998e-05 -53.6842105263158
"chr5" 2154001 2155000 "*" 0 0 100
"chr5" 2163001 2164000 "*" 1.11022302462516e-16 5.03662826618488e-16 75.1479289940828
"chr5" 2166001 2167000 "*" 5.55111512312578e-16 2.36485094870365e-15 100
"chr5" 2168001 2169000 "*" 1.11022302462516e-16 5.03662826618488e-16 -100
"chr5" 2169001 2170000 "*" 0 0 57.1428571428571
"chr5" 2172001 2173000 "*" 2.84902157421385e-09 5.16589235957774e-09 -75
"chr5" 2178001 2179000 "*" 0 0 85.7782754759238
"chr5" 2179001 2180000 "*" 2.22044604925031e-16 9.81641919380259e-16 -100

"chr5" 2185001 2186000 "*" 7.01670117786435e-07 9.09779967759556e-07
56.0975609756098
"chr5" 2187001 2188000 "*" 0 0 -100
"chr5" 2188001 2189000 "*" 2.05613304160579e-13 6.50887909862391e-13
61.6071428571429
"chr5" 2200001 2201000 "*" 4.7482617837602e-10 9.64533069050774e-10
92.3076923076923
"chr5" 2219001 2220000 "*" 1.52466927971773e-12 4.33322475981408e-12 100
"chr5" 2225001 2226000 "*" 3.52467621755892e-09 6.32782751489234e-09
78.8888888888889
"chr5" 2240001 2241000 "*" 2.17381668221606e-13 6.85151076607736e-13 100
"chr5" 2242001 2243000 "*" 2.59792187762287e-14 9.17736859954129e-14
-68.1159420289855
"chr5" 2249001 2250000 "*" 1.54781894301514e-07 2.18595147882214e-07
55.6962025316456
"chr5" 2256001 2257000 "*" 0.000110002183517421 0.000102477059743551
54.421768707483
"chr5" 2270001 2271000 "*" 1.95924387824675e-11 4.81558130907082e-11
66.6666666666667
"chr5" 2286001 2287000 "*" 8.65479909961664e-11 1.94664583883693e-10
88.135593220339
"chr5" 2325001 2326000 "*" 0 0 100
"chr5" 2384001 2385000 "*" 9.25481913327530e-11 2.07110219502964e-10
-67.8571428571429
"chr5" 2391001 2392000 "*" 2.7630383406807e-06 3.29290106239042e-06
-68.1818181818182
"chr5" 2447001 2448000 "*" 3.88022947106492e-13 1.1867462770515e-12 -100
"chr5" 2516001 2517000 "*" 1.309944922534e-07 1.87123731726588e-07
60.6060606060606
"chr5" 2533001 2534000 "*" 1.90551403345118e-06 2.32599377195959e-06
-60.5263157894737
"chr5" 2536001 2537000 "*" 8.67285784778016e-06 9.5894468867938e-06 -66.875
"chr5" 2537001 2538000 "*" 0 0 -87.8378378378378
"chr5" 2549001 2550000 "*" 2.80937484387067e-09 5.09865996492029e-09
-91.304347826087
"chr5" 2565001 2566000 "*" 3.95353005888666e-09 7.03928529283733e-09 100
"chr5" 2580001 2581000 "*" 2.43005615629954e-09 4.45014222280278e-09 -100
"chr5" 2587001 2588000 "*" 9.65338919911574e-13 2.80678910656113e-12 -100
"chr5" 2590001 2591000 "*" 3.32982534878568e-08 5.19190403402735e-08
-52.6315789473684
"chr5" 2592001 2593000 "*" 0 0 -84.0425531914894
"chr5" 2618001 2619000 "*" 3.65208016528396e-06 4.27237321401963e-06 -80
"chr5" 2641001 2642000 "*" 5.72875080706581e-14 1.93967931988877e-13
70.8333333333333
"chr5" 2669001 2670000 "*" 6.47802710991296e-05 6.26115434315376e-05
53.5714285714286
"chr5" 2697001 2698000 "*" 0 0 71.1538461538462
"chr5" 2698001 2699000 "*" 1.98729921407903e-14 7.12685498544555e-14
98.2300884955752
"chr5" 2706001 2707000 "*" 6.4152538836737e-10 1.27477697728417e-09 100
"chr5" 2709001 2710000 "*" 4.73234496034536e-09 8.30627300264826e-09 -100
"chr5" 2715001 2716000 "*" 5.08482145278322e-14 1.73432706170696e-13 100

"chr5" 2716001 2717000 "*" 0 0 100
"chr5" 2737001 2738000 "*" 1.43218770176645e-14 5.21983361092297e-14 -100
"chr5" 2814001 2815000 "*" 2.22044604925031e-16 9.81641919380259e-16
-73.2142857142857
"chr5" 2823001 2824000 "*" 0 0 -78.8990825688073
"chr5" 2841001 2842000 "*" 8.3882012447134e-11 1.88922989503423e-10 100
"chr5" 2864001 2865000 "*" 1.89425150440847e-05 1.98874471894124e-05 -62.5
"chr5" 2887001 2888000 "*" 1.88737914186277e-15 7.62011598380321e-15 -100
"chr5" 2895001 2896000 "*" 3.18078896555107e-13 9.84341434658554e-13
65.359477124183
"chr5" 2898001 2899000 "*" 3.88578058618805e-15 1.5152981210988e-14
-50.6172839506173
"chr5" 2914001 2915000 "*" 3.26405569239796e-14 1.13973003849234e-13 100
"chr5" 2917001 2918000 "*" 5.8646884903979e-08 8.81724636506667e-08
75.7731958762887
"chr5" 2997001 2998000 "*" 4.02167188440217e-12 1.08266700497926e-11 100
"chr5" 3000001 3001000 "*" 5.6362137179633e-11 1.29963270300065e-10 -100
"chr5" 3002001 3003000 "*" 0 0 100
"chr5" 3027001 3028000 "*" 2.31105012904465e-09 4.2514379630808e-09
-78.7610619469027
"chr5" 3029001 3030000 "*" 5.77315972805081e-13 1.72829442922152e-12
56.4516129032258
"chr5" 3031001 3032000 "*" 0 0 -51.8801410105758
"chr5" 3067001 3068000 "*" 0 0 100
"chr5" 3099001 3100000 "*" 1.15173001635771e-06 1.45028219004578e-06
72.9166666666667
"chr5" 3128001 3129000 "*" 1.24344978758018e-14 4.56627092294518e-14
74.0740740740741
"chr5" 3153001 3154000 "*" 3.21964677141295e-15 1.26720521345953e-14
-60.8695652173913
"chr5" 3163001 3164000 "*" 2.62900812231237e-13 8.21294938531841e-13
66.6666666666667
"chr5" 3180001 3181000 "*" 0 0 -98.7012987012987
"chr5" 3206001 3207000 "*" 2.22044604925031e-16 9.81641919380259e-16 -100
"chr5" 3231001 3232000 "*" 5.1281201507436e-13 1.54461625452838e-12 100
"chr5" 3235001 3236000 "*" 0 0 100
"chr5" 3284001 3285000 "*" 3.6700841921089e-08 5.65530226072258e-08 100
"chr5" 3287001 3288000 "*" 7.0006535457523e-08 1.03695832512054e-07 -100
"chr5" 3289001 3290000 "*" 2.62230704028976e-06 3.1345273044775e-06
-70.8333333333333
"chr5" 3306001 3307000 "*" 0 0 -55.7109557109557
"chr5" 3311001 3312000 "*" 0 0 -88.7573964497041
"chr5" 3335001 3336000 "*" 1.35890187991095e-11 3.40841971363279e-11 100
"chr5" 3342001 3343000 "*" 6.74782572440691e-05 6.50041430488692e-05
58.5714285714286
"chr5" 3382001 3383000 "*" 9.82436354490801e-13 2.8547096611003e-12
-60.9237536656892
"chr5" 3392001 3393000 "*" 9.86378351419681e-06 1.08245798552885e-05
-51.6483516483516
"chr5" 3489001 3490000 "*" 0 0 73.5632183908046
"chr5" 3728001 3729000 "*" 3.63596930341714e-11 8.58897564338675e-11
56.9444444444444

"chr5" 3768001 3769000 "*" 0 0 -66.6666666666667
"chr5" 3789001 3790000 "*" 2.11273923023114e-07 2.9348734792333e-07
58.8235294117647
"chr5" 3858001 3859000 "*" 2.43005615629954e-09 4.45014222280278e-09 -100
"chr5" 4002001 4003000 "*" 6.55653309422632e-12 1.7186391858546e-11
66.6666666666667
"chr5" 4013001 4014000 "*" 2.22044604925031e-16 9.81641919380259e-16
66.6666666666667
"chr5" 4172001 4173000 "*" 1.76033188026281e-10 3.80157191309706e-10
52.1929824561403
"chr5" 4191001 4192000 "*" 1.82123929270972e-08 2.94017975572653e-08
-53.2258064516129
"chr5" 4195001 4196000 "*" 1.19904086659517e-14 4.40901388691796e-14 -100
"chr5" 4351001 4352000 "*" 2.37587727269783e-14 8.44268514944447e-14 -100
"chr5" 4375001 4376000 "*" 0 0 100
"chr5" 4399001 4400000 "*" 2.73625566649116e-12 7.52483363579137e-12 -100
"chr5" 4463001 4464000 "*" 1.13140401492018e-08 1.87801380787728e-08 100
"chr5" 4556001 4557000 "*" 3.41362518918586e-07 4.61573800213058e-07 73
"chr5" 4577001 4578000 "*" 5.08482145278322e-14 1.73432706170696e-13 100
"chr5" 4692001 4693000 "*" 7.73809894616395e-06 8.62231249105587e-06
58.0357142857143
"chr5" 4855001 4856000 "*" 2.31469791711625e-06 2.79034791839104e-06 75
"chr5" 4880001 4881000 "*" 0 0 -51.6325059223401
"chr5" 4943001 4944000 "*" 1.31178778173258e-07 1.87370935061106e-07
-53.2147742818057
"chr5" 5041001 5042000 "*" 0.0007801470789216 0.000630674635502966
-54.9407114624506
"chr5" 5156001 5157000 "*" 1.11022302462516e-16 5.03662826618488e-16 100
"chr5" 5265001 5266000 "*" 1.86039445903008e-05 1.95647430201968e-05
56.9444444444444
"chr5" 5308001 5309000 "*" 7.90629228752948e-10 1.55439590586289e-09
54.2857142857143
"chr5" 5324001 5325000 "*" 0 0 -100
"chr5" 5334001 5335000 "*" 4.79616346638068e-14 1.64380445416596e-13 -100
"chr5" 5512001 5513000 "*" 0 0 -67.7777777777778
"chr5" 5518001 5519000 "*" 5.04263297784746e-13 1.52078941532818e-12 100
"chr5" 5571001 5572000 "*" 3.33066907387547e-16 1.4495649018245e-15
91.2698412698413
"chr5" 5572001 5573000 "*" 3.22375459660407e-12 8.79500753078322e-12 75
"chr5" 5665001 5666000 "*" 0 0 -100
"chr5" 5757001 5758000 "*" 7.67056418382595e-11 1.7359448580318e-10 -100
"chr5" 5829001 5830000 "*" 2.69018141096922e-12 7.41487347763769e-12 -100
"chr5" 6044001 6045000 "*" 2.58183519008526e-09 4.71546194625964e-09
81.8181818181818
"chr5" 6046001 6047000 "*" 0 0 65.8823529411765
"chr5" 6087001 6088000 "*" 2.22044604925031e-16 9.81641919380259e-16 100
"chr5" 6091001 6092000 "*" 1.52794177310511e-09 2.87642564232045e-09 -100
"chr5" 6107001 6108000 "*" 4.44200176641374e-09 7.84403761500069e-09
-86.7469879518072
"chr5" 6197001 6198000 "*" 1.49613654798486e-12 4.26244554429779e-12 100
"chr5" 6232001 6233000 "*" 6.92287338566189e-11 1.57605630124247e-10 -100
"chr5" 6349001 6350000 "*" 0 0 76.6042780748663

"chr5" 6354001 6355000 "*" 1.07882591748876e-11 2.74553537455803e-11 100
"chr5" 6369001 6370000 "*" 6.4324989779152e-11 1.47230020825092e-10 100
"chr5" 6418001 6419000 "*" 1.17461596005342e-13 3.8279067575877e-13 100
"chr5" 6435001 6436000 "*" 4.11539381706127e-06 4.77326970666537e-06
-66.6666666666667
"chr5" 6478001 6479000 "*" 0.000299166032473375 0.000259267996470385
-54.1666666666667
"chr5" 6543001 6544000 "*" 3.69434035925043e-05 3.70668239602475e-05
-61.5384615384615
"chr5" 6547001 6548000 "*" 8.7349629751543e-09 1.47505871898511e-08 -100
"chr5" 6554001 6555000 "*" 0 0 64.7619047619048
"chr5" 6582001 6583000 "*" 0 0 53.2374100719424
"chr5" 6593001 6594000 "*" 4.51551152025331e-09 7.9662089949093e-09 56
"chr5" 6597001 6598000 "*" 1.19495335848541e-08 1.9782040720134e-08
-52.3809523809524
"chr5" 6613001 6614000 "*" 2.08814465718632e-09 3.86232567960463e-09 100
"chr5" 6631001 6632000 "*" 4.01313771103418e-09 7.11882087237587e-09 100
"chr5" 6660001 6661000 "*" 3.6700841921089e-08 5.65530226072258e-08 100
"chr5" 6702001 6703000 "*" 0 0 52.7950310559006
"chr5" 6790001 6791000 "*" 2.44805401505843e-08 3.87506495191586e-08 68.75
"chr5" 6811001 6812000 "*" 1.48087875295744e-10 3.22075573380618e-10 100
"chr5" 6891001 6892000 "*" 4.39648317751562e-14 1.5131009830411e-13
-63.8297872340426
"chr5" 7081001 7082000 "*" 1.99962268965237e-11 4.8926146525693e-11
93.3333333333333
"chr5" 7306001 7307000 "*" 6.58243449525031e-07 8.57004003654891e-07
81.8181818181818
"chr5" 7372001 7373000 "*" 9.21464274328265e-07 1.17559142617104e-06
-81.4814814814815
"chr5" 7474001 7475000 "*" 6.4152538836737e-10 1.27477697728417e-09 100
"chr5" 7598001 7599000 "*" 0 0 100
"chr5" 7706001 7707000 "*" 8.25450818808804e-13 2.42473843346228e-12 100
"chr5" 7826001 7827000 "*" 0 0 73.9130434782609
"chr5" 7827001 7828000 "*" 4.19161879261498e-06 4.85560209921896e-06
-66.6666666666667
"chr5" 7848001 7849000 "*" 1.67299207820548e-08 2.71377429441055e-08 100
"chr5" 7850001 7851000 "*" 0 0 73.4251491530848
"chr5" 7929001 7930000 "*" 5.794809077031e-12 1.52911192757929e-11 -100
"chr5" 8058001 8059000 "*" 1.11022302462516e-16 5.03662826618488e-16 -100
"chr5" 8181001 8182000 "*" 9.08108033215171e-11 2.03407753381119e-10 100
"chr5" 8374001 8375000 "*" 6.24654217240561e-10 1.2462108191303e-09 100
"chr5" 8430001 8431000 "*" 2.50411913427229e-11 6.07090727285435e-11
-59.0909090909091
"chr5" 8615001 8616000 "*" 3.34128893442198e-08 5.19772864618523e-08 -100
"chr5" 8871001 8872000 "*" 0 0 100
"chr5" 9090001 9091000 "*" 2.88779000712225e-11 6.92935809466068e-11 -100
"chr5" 9118001 9119000 "*" 1.12086118164711e-10 2.48625239383088e-10
-63.4660421545667
"chr5" 9516001 9517000 "*" 0 0 -100
"chr5" 9522001 9523000 "*" 8.65973959207622e-14 2.87362558212404e-13 -100
"chr5" 9530001 9531000 "*" 9.08108033215171e-11 2.03407753381119e-10 -100
"chr5" 9547001 9548000 "*" 1.17128529097954e-13 3.82063554513641e-13

82.8571428571429
"chr5" 9562001 9563000 "*" 1.07882591748876e-11 2.74553537455803e-11 -100
"chr5" 10073001 10074000 "*" 0 0 -100
"chr5" 10074001 10075000 "*" 1.55275792224074e-12 4.40959864274368e-12 -80
"chr5" 10076001 10077000 "*" 0 0 100
"chr5" 10127001 10128000 "*" 1.84741111297626e-13 5.89739624939843e-13
-65.8878504672897
"chr5" 10307001 10308000 "*" 0 0 -68.8654353562005
"chr5" 10410001 10411000 "*" 1.11022302462516e-16 5.03662826618488e-16 100
"chr5" 10442001 10443000 "*" 0 0 71.0527692563792
"chr5" 10460001 10461000 "*" 0 0 -83.8383838383838
"chr5" 10471001 10472000 "*" 6.92287338566189e-11 1.57605630124247e-10 100
"chr5" 10503001 10504000 "*" 2.69706510458434e-08 4.25043195508366e-08
-62.962962962963
"chr5" 10511001 10512000 "*" 1.25551145254699e-05 1.35497076198712e-05
64.5161290322581
"chr5" 10530001 10531000 "*" 8.88178419700125e-16 3.70670032207938e-15 -100
"chr5" 10536001 10537000 "*" 3.03979064142368e-13 9.42350883309283e-13 100
"chr5" 10601001 10602000 "*" 1.00598051000489e-08 1.68638772431386e-08
-79.7814207650273
"chr5" 10625001 10626000 "*" 3.6427076910428e-09 6.5252880340617e-09
66.6666666666667
"chr5" 10662001 10663000 "*" 3.46192373656962e-08 5.37286189448515e-08
-79.9086757990868
"chr5" 10715001 10716000 "*" 2.15125472990962e-10 4.58126659943874e-10 100
"chr5" 10776001 10777000 "*" 7.0006535457523e-08 1.03695832512054e-07 100
"chr5" 10868001 10869000 "*" 1.38777878078145e-13 4.48315906406355e-13
-76.9230769230769
"chr5" 10875001 10876000 "*" 4.86753970463383e-11 1.13145908161915e-10 100
"chr5" 10882001 10883000 "*" 0 0 100
"chr5" 10994001 10995000 "*" 0 0 100
"chr5" 11151001 11152000 "*" 4.74104333569869e-09 8.32066493888611e-09
-71.4285714285714
"chr5" 11246001 11247000 "*" 1.39779633423487e-07 1.98463037958589e-07 -100
"chr5" 11311001 11312000 "*" 9.65729496371637e-10 1.87255912493232e-09 -100
"chr5" 11313001 11314000 "*" 0 0 100
"chr5" 11812001 11813000 "*" 3.6700841921089e-08 5.65530226072258e-08 -100
"chr5" 12126001 12127000 "*" 3.95353005888666e-09 7.03928529283733e-09 -100
"chr5" 12166001 12167000 "*" 2.08814465718632e-09 3.86232567960463e-09 100
"chr5" 12266001 12267000 "*" 1.13140401492018e-08 1.87801380787728e-08 100
"chr5" 12303001 12304000 "*" 3.52704532247117e-11 8.35581292290093e-11 -100
"chr5" 12806001 12807000 "*" 3.88022947106492e-13 1.1867462770515e-12 100
"chr5" 13707001 13708000 "*" 7.105427357601e-15 2.68362283258525e-14 -100
"chr5" 14122001 14123000 "*" 7.99844592513921e-06 8.89173797399106e-06
-66.6666666666667
"chr5" 14162001 14163000 "*" 2.15125472990962e-10 4.58126659943874e-10 -100
"chr5" 14262001 14263000 "*" 5.43594952717719e-06 6.19887830900258e-06 60
"chr5" 14360001 14361000 "*" 2.73625566649116e-12 7.52483363579137e-12 100
"chr5" 14446001 14447000 "*" 0 0 -58.8235294117647
"chr5" 14481001 14482000 "*" 5.10702591327572e-15 1.96271840288234e-14 -100
"chr5" 14557001 14558000 "*" 4.08537648155516e-11 9.58460845439556e-11
-93.3333333333333

"chr5" 14850001 14851000 "*" 4.08209022140227e-11 9.57770690255255e-11 -100
"chr5" 14895001 14896000 "*" 1.13140401492018e-08 1.87801380787728e-08 100
"chr5" 15077001 15078000 "*" 9.04570973681018e-10 1.76414389822173e-09 100
"chr5" 15096001 15097000 "*" 0 0 -100
"chr5" 15292001 15293000 "*" 0 0 91.3907284768212
"chr5" 15734001 15735000 "*" 2.33146835171283e-15 9.32818831833246e-15
-80.6451612903226
"chr5" 15869001 15870000 "*" 7.0006535457523e-08 1.03695832512054e-07 100
"chr5" 16206001 16207000 "*" 8.25450818808804e-13 2.42473843346228e-12 100
"chr5" 16466001 16467000 "*" 0 0 83.0508474576271
"chr5" 16554001 16555000 "*" 2.90915069811604e-11 6.97590507139978e-11
-92.2077922077922
"chr5" 16591001 16592000 "*" 2.55351295663786e-15 1.01678090381833e-14 -100
"chr5" 16593001 16594000 "*" 0 0 100
"chr5" 16603001 16604000 "*" 1.93720595120794e-11 4.76309924836636e-11 -100
"chr5" 16617001 16618000 "*" 0 0 56.7460317460317
"chr5" 16750001 16751000 "*" 4.08209022140227e-11 9.57770690255255e-11 100
"chr5" 16770001 16771000 "*" 0 0 80.7228915662651
"chr5" 16879001 16880000 "*" 7.0006535457523e-08 1.03695832512054e-07 100
"chr5" 16896001 16897000 "*" 0 0 100
"chr5" 16935001 16936000 "*" 0 0 -55.0724637681159
"chr5" 16973001 16974000 "*" 2.19383400335005e-11 5.34788778871231e-11
97.2972972972973
"chr5" 17126001 17127000 "*" 9.08108033215171e-11 2.03407753381119e-10 -100
"chr5" 17143001 17144000 "*" 1.17905685215192e-13 3.83653245040640e-13 100
"chr5" 17218001 17219000 "*" 0 0 54.1563050731755
"chr5" 17511001 17512000 "*" 4.18887147191072e-13 1.2739946038722e-12 -100
"chr5" 17625001 17626000 "*" 0.000144400838577918 0.000131924792739962
-53.8461538461538
"chr5" 18733001 18734000 "*" 0 0 100
"chr5" 18793001 18794000 "*" 4.08209022140227e-11 9.57770690255255e-11 100
"chr5" 19094001 19095000 "*" 3.6700841921089e-08 5.65530226072258e-08 100
"chr5" 19142001 19143000 "*" 0 0 100
"chr5" 19143001 19144000 "*" 0 0 -75.9290780141844
"chr5" 19654001 19655000 "*" 2.15125472990962e-10 4.58126659943874e-10 100
"chr5" 20418001 20419000 "*" 0 0 -100
"chr5" 20552001 20553000 "*" 1.5277158427196e-09 2.87642564232045e-09 100
"chr5" 20553001 20554000 "*" 2.69018141096922e-12 7.41487347763769e-12 100
"chr5" 20969001 20970000 "*" 2.75837855657768e-05 2.82530803747995e-05
-66.6666666666667
"chr5" 21189001 21190000 "*" 0.000896607310246877 0.000717057981883268
53.8461538461538
"chr5" 21530001 21531000 "*" 2.69007038866675e-13 8.38831022628993e-13 100
"chr5" 21624001 21625000 "*" 1.86517468137026e-14 6.70995420767425e-14 100
"chr5" 22326001 22327000 "*" 1.14109801607754e-08 1.89325740250273e-08 -75
"chr5" 22753001 22754000 "*" 4.32209823486573e-12 1.15935290479641e-11 -100
"chr5" 23234001 23235000 "*" 0 0 100
"chr5" 23305001 23306000 "*" 0 0 63.2478632478632
"chr5" 23324001 23325000 "*" 2.22044604925031e-16 9.81641919380259e-16 -100
"chr5" 23527001 23528000 "*" 5.08482145278322e-14 1.73432706170696e-13 -100
"chr5" 23737001 23738000 "*" 3.04324343503026e-11 7.26599935673121e-11 -100
"chr5" 24868001 24869000 "*" 4.73234496034536e-09 8.30627300264826e-09 -100

"chr5" 26226001 26227000 "*" 2.22044604925031e-16 9.81641919380259e-16 100
"chr5" 26232001 26233000 "*" 8.65973959207622e-14 2.87362558212404e-13 100
"chr5" 26416001 26417000 "*" 9.26392664801767e-08 1.34981038124536e-07
-81.1594202898551
"chr5" 26982001 26983000 "*" 0 0 100
"chr5" 27171001 27172000 "*" 4.43235559366428e-10 9.04370375508963e-10
-57.5757575757576
"chr5" 27493001 27494000 "*" 2.78987943858056e-12 7.66471949267203e-12
60.9756097560976
"chr5" 27783001 27784000 "*" 9.71063229826541e-11 2.16781365050398e-10 100
"chr5" 28082001 28083000 "*" 3.58894225660489e-06 4.20417278477504e-06
-63.6363636363636
"chr5" 28084001 28085000 "*" 0 0 98.1818181818182
"chr5" 28188001 28189000 "*" 0 0 100
"chr5" 28862001 28863000 "*" 1.98325800226939e-11 4.85612402473442e-11 100
"chr5" 29396001 29397000 "*" 0 0 -100
"chr5" 30070001 30071000 "*" 0 0 -100
"chr5" 30698001 30699000 "*" 7.8614662526455e-06 8.75240972186174e-06
66.6666666666667
"chr5" 30763001 30764000 "*" 1.17461596005342e-13 3.8279067575877e-13 100
"chr5" 31379001 31380000 "*" 2.67283972732457e-11 6.45686178157698e-11 100
"chr5" 31643001 31644000 "*" 7.0006535457523e-08 1.03695832512054e-07 100
"chr5" 31663001 31664000 "*" 1.16727250853987e-06 1.46847890858587e-06
76.6666666666667
"chr5" 31693001 31694000 "*" 1.11022302462516e-16 5.03662826618488e-16
-71.3356973995272
"chr5" 31775001 31776000 "*" 1.37828859436695e-10 3.01445544267661e-10 -100
"chr5" 31785001 31786000 "*" 2.88710388929303e-10 6.03399264731139e-10 -100
"chr5" 31799001 31800000 "*" 6.59550958292954e-09 1.13456194382773e-08 100
"chr5" 31802001 31803000 "*" 0 0 98.9010989010989
"chr5" 31910001 31911000 "*" 3.33066907387547e-16 1.4495649018245e-15 100
"chr5" 31932001 31933000 "*" 1.13140401492018e-08 1.87801380787728e-08 100
"chr5" 31944001 31945000 "*" 1.46469895667689e-05 1.56483031663302e-05
57.7777777777778
"chr5" 31963001 31964000 "*" 6.57252030578093e-14 2.21243451061355e-13
68.1818181818182
"chr5" 31978001 31979000 "*" 2.16382467499443e-13 6.82775854166559e-13 -100
"chr5" 32320001 32321000 "*" 2.37587727269783e-14 8.44268514944447e-14
50.5154639175258
"chr5" 32329001 32330000 "*" 1.11022302462516e-16 5.03662826618488e-16
-78.7234042553192
"chr5" 32377001 32378000 "*" 1.11022302462516e-16 5.03662826618488e-16 -100
"chr5" 32452001 32453000 "*" 2.00227675550835e-08 3.20459176617961e-08 100
"chr5" 32668001 32669000 "*" 1.11022302462516e-16 5.03662826618488e-16 100
"chr5" 32710001 32711000 "*" 0 0 85.9038142620232
"chr5" 32782001 32783000 "*" 0 0 71.6417910447761
"chr5" 33297001 33298000 "*" 3.49804499810702e-08 5.42669997904552e-08
52.3809523809524
"chr5" 33549001 33550000 "*" 5.14033260401447e-13 1.54807107150133e-12
65.6716417910448
"chr5" 33727001 33728000 "*" 6.6532020337462e-08 9.92934679052014e-08
57.5757575757576

"chr5" 34657001 34658000 "*" 8.11321898552819e-09 1.37928190725433e-08 -68.75
"chr5" 34839001 34840000 "*" 0 0 67.8125
"chr5" 35229001 35230000 "*" 0 0 -100
"chr5" 35348001 35349000 "*" 4.01313771103418e-09 7.11882087237587e-09 100
"chr5" 35532001 35533000 "*" 3.33066907387547e-16 1.4495649018245e-15 100
"chr5" 35823001 35824000 "*" 8.7349629751543e-09 1.47505871898511e-08 100
"chr5" 35926001 35927000 "*" 1.43403343636495e-05 1.53387611027915e-05
-63.8297872340426
"chr5" 35946001 35947000 "*" 1.25093098791718e-05 1.35030371915773e-05
65.7894736842105
"chr5" 35991001 35992000 "*" 0 0 54.5454545454545
"chr5" 36078001 36079000 "*" 1.21844645484259e-10 2.68713233609563e-10
-68.6274509803922
"chr5" 36333001 36334000 "*" 0 0 -100
"chr5" 36432001 36433000 "*" 6.32827124036339e-15 2.40666772484995e-14 100
"chr5" 37086001 37087000 "*" 0.000236804549143432 0.000208871802823329
55.8823529411765
"chr5" 37225001 37226000 "*" 0 0 -100
"chr5" 37248001 37249000 "*" 0 0 90.9090909090909
"chr5" 37249001 37250000 "*" 0 0 60.3631558630519
"chr5" 37770001 37771000 "*" 1.39779633423487e-07 1.98463037958589e-07 -100
"chr5" 37842001 37843000 "*" 2.69018141096922e-12 7.41487347763769e-12 100
"chr5" 38195001 38196000 "*" 8.7349629751543e-09 1.47505871898511e-08 -100
"chr5" 38455001 38456000 "*" 1.48214773787458e-13 4.77488235285846e-13 100
"chr5" 38473001 38474000 "*" 3.9190872769268e-14 1.35416418933905e-13 100
"chr5" 38770001 38771000 "*" 0 0 -100
"chr5" 40680001 40681000 "*" 0 0 50.4018773009583
"chr5" 40800001 40801000 "*" 9.0072393987839e-13 2.63157725646847e-12 100
"chr5" 41206001 41207000 "*" 0 0 96.7741935483871
"chr5" 41494001 41495000 "*" 6.17195672347748e-09 1.06866836714935e-08 80
"chr5" 41587001 41588000 "*" 7.89349696717068e-10 1.55191689908115e-09
60.7142857142857
"chr5" 41925001 41926000 "*" 0 0 -56.398188621763
"chr5" 42423001 42424000 "*" 0 0 65.925908129925
"chr5" 42507001 42508000 "*" 1.5277158427196e-09 2.87642564232045e-09 -100
"chr5" 42904001 42905000 "*" 3.20512737694845e-06 3.78213360996072e-06
71.0526315789474
"chr5" 42992001 42993000 "*" 0 0 50.7364547839624
"chr5" 43043001 43044000 "*" 0 0 -54.1666666666667
"chr5" 43120001 43121000 "*" 1.80373504909959e-06 2.20912770564462e-06
-58.3333333333333
"chr5" 43187001 43188000 "*" 2.02327044007689e-12 5.65300384792621e-12 100
"chr5" 43193001 43194000 "*" 0 0 89.0322580645161
"chr5" 43206001 43207000 "*" 0 0 100
"chr5" 43280001 43281000 "*" 6.15577588902738e-11 1.41306836819921e-10
69.4610778443114
"chr5" 43557001 43558000 "*" 1.04041445680991e-06 1.31753670081634e-06
69.4444444444444
"chr5" 43603001 43604000 "*" 0 0 61.5988829186884
"chr5" 45891001 45892000 "*" 0 0 100
"chr5" 49501001 49502000 "*" 3.7952974096811e-12 1.02736270423463e-11
80.03663003663

"chr5" 49522001 49523000 "*" 0.000282447773526706 0.000245898526777105 73.3333333333333
"chr5" 49963001 49964000 "*" 0 0 53.2163742690058
"chr5" 50260001 50261000 "*" 0 0 58.3826429980276
"chr5" 50678001 50679000 "*" 0 0 92.258064516129
"chr5" 50679001 50680000 "*" 0 0 58.9605734767025
"chr5" 51759001 51760000 "*" 3.40736328041658e-11 8.09342881111001e-11 -81.8181818181818
"chr5" 51760001 51761000 "*" 3.26405569239796e-14 1.13973003849234e-13 -100
"chr5" 52776001 52777000 "*" 0 0 61.4329383146587
"chr5" 53041001 53042000 "*" 3.19300141882195e-13 9.87864167952596e-13 66.6666666666667
"chr5" 53119001 53120000 "*" 1.16408228700848e-06 1.46479698533027e-06 66.6666666666667
"chr5" 53122001 53123000 "*" 6.4152538836737e-10 1.27477697728417e-09 -100
"chr5" 53133001 53134000 "*" 2.16937579011756e-13 6.84372103056288e-13 -80
"chr5" 53155001 53156000 "*" 2.15125472990962e-10 4.58126659943874e-10 100
"chr5" 53519001 53520000 "*" 6.66133814775094e-16 2.81595744474255e-15 51.063829787234
"chr5" 53528001 53529000 "*" 5.48638023900594e-10 1.10374357336421e-09 -100
"chr5" 53607001 53608000 "*" 4.76196859722222e-11 1.10985775753449e-10 62.5
"chr5" 53777001 53778000 "*" 2.08995487582797e-10 4.46944006972663e-10 -100
"chr5" 53811001 53812000 "*" 0.000471727282945866 0.000395504194557835 53.8461538461538
"chr5" 54281001 54282000 "*" 0 0 53.6608388738406
"chr5" 54333001 54334000 "*" 3.90831811358794e-12 1.05610567215048e-11 100
"chr5" 54472001 54473000 "*" 0 0 -92.7480916030534
"chr5" 54528001 54529000 "*" 0 0 -75.2577319587629
"chr5" 55219001 55220000 "*" 1.42169386485591e-07 2.01687677516026e-07 -60.4651162790698
"chr5" 55382001 55383000 "*" 3.84894338623099e-11 9.07305146999912e-11 -84.375
"chr5" 55409001 55410000 "*" 8.52140580320793e-12 2.19817259951489e-11 100
"chr5" 55455001 55456000 "*" 4.01313771103418e-09 7.11882087237587e-09 -100
"chr5" 55566001 55567000 "*" 0 0 -100
"chr5" 55594001 55595000 "*" 2.38322361845178e-10 5.04370967350592e-10 87.5
"chr5" 55665001 55666000 "*" 1.67299207820548e-08 2.71377429441055e-08 -100
"chr5" 55752001 55753000 "*" 1.39779633423487e-07 1.98463037958589e-07 100
"chr5" 55777001 55778000 "*" 2.11130257721237e-09 3.90352493855835e-09 52.824302134647
"chr5" 55818001 55819000 "*" 7.03992419914812e-12 1.83313429961219e-11 100
"chr5" 55884001 55885000 "*" 6.4152538836737e-10 1.27477697728417e-09 100
"chr5" 56125001 56126000 "*" 0 0 -100
"chr5" 56431001 56432000 "*" 3.88022947106492e-13 1.1867462770515e-12 -100
"chr5" 56442001 56443000 "*" 9.41469124882133e-14 3.10868561210786e-13 -100
"chr5" 56692001 56693000 "*" 5.10702591327572e-15 1.96271840288234e-14 -100
"chr5" 57584001 57585000 "*" 7.0938042107116e-09 1.21584096863937e-08 53.8461538461538
"chr5" 57878001 57879000 "*" 0 0 69.0697674418605
"chr5" 58106001 58107000 "*" 2.15125472990962e-10 4.58126659943874e-10 -100
"chr5" 59064001 59065000 "*" 0 0 100
"chr5" 60556001 60557000 "*" 0 0 -100
"chr5" 60768001 60769000 "*" 7.7715611723761e-16 3.26213507634405e-15 100

"chr5" 60880001 60881000 "*" 1.10111923912193e-07 1.58907290044317e-07
-78.9473684210526
"chr5" 60993001 60994000 "*" 1.39779633423487e-07 1.98463037958589e-07 100
"chr5" 61548001 61549000 "*" 0 0 -100
"chr5" 61549001 61550000 "*" 8.71525074330748e-14 2.88848349331391e-13 100
"chr5" 63461001 63462000 "*" 0 0 61.5894974590627
"chr5" 63971001 63972000 "*" 0 0 -88.1818181818182
"chr5" 63978001 63979000 "*" 1.88737914186277e-15 7.62011598380321e-15 -100
"chr5" 65221001 65222000 "*" 0 0 77.7222322124318
"chr5" 65552001 65553000 "*" 3.6700841921089e-08 5.65530226072258e-08 -100
"chr5" 65892001 65893000 "*" 0 0 63.8368741574078
"chr5" 66046001 66047000 "*" 1.87318113131774e-07 2.61985564391649e-07
-79.1666666666667
"chr5" 66488001 66489000 "*" 1.12458486967171e-09 2.15740815043925e-09 -100
"chr5" 66639001 66640000 "*" 2.22044604925031e-16 9.81641919380259e-16 -100
"chr5" 67076001 67077000 "*" 6.88338275267597e-15 2.60518659957509e-14 -100
"chr5" 68198001 68199000 "*" 1.01918473660589e-13 3.34635318978813e-13
98.4126984126984
"chr5" 68401001 68402000 "*" 1.11022302462516e-16 5.03662826618488e-16 -100
"chr5" 68711001 68712000 "*" 0 0 -67.866344605475
"chr5" 70681001 70682000 "*" 0 0 100
"chr5" 70747001 70748000 "*" 3.6700841921089e-08 5.65530226072258e-08 100
"chr5" 70748001 70749000 "*" 0 0 -75.5102040816327
"chr5" 70761001 70762000 "*" 6.59550958292954e-09 1.13456194382773e-08 -100
"chr5" 71041001 71042000 "*" 8.25450818808804e-13 2.42473843346228e-12 -100
"chr5" 71403001 71404000 "*" 0 0 66.1473306141442
"chr5" 71404001 71405000 "*" 0 0 53.8163777420595
"chr5" 71473001 71474000 "*" 1.17905685215192e-13 3.83653245040640e-13 100
"chr5" 71475001 71476000 "*" 1.11022302462516e-16 5.03662826618488e-16
-90.1408450704225
"chr5" 71852001 71853000 "*" 0 0 -60.7594936708861
"chr5" 72318001 72319000 "*" 5.794809077031e-12 1.52911192757929e-11 -100
"chr5" 72431001 72432000 "*" 1.88737914186277e-15 7.62011598380321e-15 -100
"chr5" 72470001 72471000 "*" 1.41964690558716e-07 2.014179973408e-07
79.1666666666667
"chr5" 72618001 72619000 "*" 2.68450373042128e-10 5.64465213127486e-10 -100
"chr5" 72742001 72743000 "*" 0 0 83.3333333333333
"chr5" 72840001 72841000 "*" 4.71134242729931e-12 1.257615036806e-11 100
"chr5" 72921001 72922000 "*" 0 0 -64.0449438202247
"chr5" 73651001 73652000 "*" 2.08814465718632e-09 3.86232567960463e-09 -100
"chr5" 73658001 73659000 "*" 4.08209022140227e-11 9.57770690255255e-11 100
"chr5" 74630001 74631000 "*" 5.77315972805081e-15 2.205310974382e-14
-87.1794871794872
"chr5" 75127001 75128000 "*" 8.10462807976364e-15 3.04213344674586e-14
50.4672897196262
"chr5" 75216001 75217000 "*" 1.38590361409285e-10 3.02706178892116e-10 100
"chr5" 75290001 75291000 "*" 3.95353005888666e-09 7.03928529283733e-09 100
"chr5" 75464001 75465000 "*" 0 0 -100
"chr5" 75553001 75554000 "*" 7.30226989986704e-12 1.89925278834201e-11
-92.4528301886792
"chr5" 75599001 75600000 "*" 1.35036426485158e-12 3.86629547363774e-12 -100
"chr5" 75600001 75601000 "*" 3.6700841921089e-08 5.65530226072258e-08 -100

"chr5" 75788001 75789000 "*" 5.55111512312578e-16 2.36485094870365e-15 -100
"chr5" 76097001 76098000 "*" 2.88779000712225e-11 6.92935809466068e-11 -100
"chr5" 76384001 76385000 "*" 4.80049333617671e-12 1.28063362452587e-11
63.3333333333333
"chr5" 76701001 76702000 "*" 3.77475828372553e-15 1.47379557022071e-14 100
"chr5" 76940001 76941000 "*" 0 0 50.2293221393035
"chr5" 77180001 77181000 "*" 4.73234496034536e-09 8.30627300264826e-09 100
"chr5" 77816001 77817000 "*" 3.83190479347206e-10 7.87927836531292e-10 62.5
"chr5" 78088001 78089000 "*" 2.88710388929303e-10 6.03399264731139e-10 100
"chr5" 78460001 78461000 "*" 6.98330282489223e-14 2.34267456154807e-13 100
"chr5" 78807001 78808000 "*" 6.59550958292954e-09 1.13456194382773e-08 100
"chr5" 78890001 78891000 "*" 4.48406133335411e-08 6.84482503893299e-08
-66.6666666666667
"chr5" 79144001 79145000 "*" 0 0 100
"chr5" 79286001 79287000 "*" 0 0 -83.7037037037037
"chr5" 79398001 79399000 "*" 0 0 -67.2413793103448
"chr5" 79549001 79550000 "*" 7.0006535457523e-08 1.03695832512054e-07 -100
"chr5" 79552001 79553000 "*" 0 0 -100
"chr5" 79638001 79639000 "*" 4.44089209850063e-16 1.91071758245033e-15 -100
"chr5" 79683001 79684000 "*" 3.95353005888666e-09 7.03928529283733e-09 -100
"chr5" 79866001 79867000 "*" 0 0 61.9558625336927
"chr5" 79892001 79893000 "*" 8.3882012447134e-11 1.88922989503423e-10 -100
"chr5" 80547001 80548000 "*" 0 0 -100
"chr5" 81426001 81427000 "*" 9.14157638476354e-13 2.66886212024714e-12
98.3606557377049
"chr5" 82842001 82843000 "*" 4.91828799908944e-14 1.68192819018269e-13 100
"chr5" 83009001 83010000 "*" 1.83952852950142e-12 5.17499701329218e-12 -100
"chr5" 84229001 84230000 "*" 0 0 83.5443037974684
"chr5" 84943001 84944000 "*" 1.11022302462516e-16 5.03662826618488e-16 -59.375
"chr5" 85400001 85401000 "*" 6.24654217240561e-10 1.2462108191303e-09 100
"chr5" 87311001 87312000 "*" 1.51656465163796e-13 4.87803420672651e-13 -100
"chr5" 87348001 87349000 "*" 1.00577998818352e-07 1.45905892474209e-07
66.6666666666667
"chr5" 87386001 87387000 "*" 2.11985984321927e-11 5.17557928578922e-11
61.7021276595745
"chr5" 87438001 87439000 "*" 0 0 -71.7948717948718
"chr5" 87898001 87899000 "*" 0 0 95.7507082152975
"chr5" 87956001 87957000 "*" 0 0 88.2967373596113
"chr5" 87957001 87958000 "*" 0 0 100
"chr5" 87963001 87964000 "*" 4.45257164471968e-11 1.04083070373096e-10
-90.9090909090909
"chr5" 87969001 87970000 "*" 1.11022302462516e-16 5.03662826618488e-16
57.7817213842059
"chr5" 87970001 87971000 "*" 0 0 52.9874718003321
"chr5" 87971001 87972000 "*" 0 0 84.1757118373974
"chr5" 87976001 87977000 "*" 0 0 -64.6860347522295
"chr5" 87987001 87988000 "*" 0 0 88.7323943661972
"chr5" 88917001 88918000 "*" 9.65729496371637e-10 1.87255912493232e-09 100
"chr5" 89964001 89965000 "*" 9.44796552104776e-09 1.58870702326918e-08
59.0909090909091
"chr5" 91215001 91216000 "*" 1.19904086659517e-14 4.40901388691796e-14 100
"chr5" 91333001 91334000 "*" 2.02327044007689e-12 5.65300384792621e-12 -100

"chr5" 91912001 91913000 "*" 8.02691246803988e-14 2.67352274101148e-13 100
"chr5" 92397001 92398000 "*" 6.24654217240561e-10 1.2462108191303e-09 100
"chr5" 92947001 92948000 "*" 1.4432899320127e-15 5.90750815956055e-15
71.9394329896907
"chr5" 93701001 93702000 "*" 2.06120231993623e-10 4.41723951173619e-10
-51.8518518518519
"chr5" 93808001 93809000 "*" 1.23623818034657e-05 1.33534251888187e-05
-66.6666666666667
"chr5" 93903001 93904000 "*" 2.88710388929303e-10 6.03399264731139e-10 100
"chr5" 94619001 94620000 "*" 0 0 54.0914258519948
"chr5" 94621001 94622000 "*" 0 0 87.1287128712871
"chr5" 94889001 94890000 "*" 4.38571401417676e-12 1.17569864514837e-11
84.6153846153846
"chr5" 95179001 95180000 "*" 1.70641278884887e-12 4.81986032810646e-12 100
"chr5" 95398001 95399000 "*" 9.71063229826541e-11 2.16781365050398e-10 -100
"chr5" 95562001 95563000 "*" 3.6700841921089e-08 5.65530226072258e-08 100
"chr5" 95767001 95768000 "*" 1.66163844109724e-05 1.76120026560188e-05
60.2150537634409
"chr5" 95788001 95789000 "*" 3.34128893442198e-08 5.19772864618523e-08 -100
"chr5" 96139001 96140000 "*" 3.43503003819023e-13 1.05967782244095e-12 -100
"chr5" 96726001 96727000 "*" 3.53781981488055e-09 6.34912718011365e-09
66.6666666666667
"chr5" 97309001 97310000 "*" 0 0 67.4418604651163
"chr5" 97641001 97642000 "*" 2.6569935140941e-09 4.83462661545569e-09
66.6666666666667
"chr5" 98181001 98182000 "*" 3.55123767059595e-08 5.50471549649875e-08
-58.3333333333333
"chr5" 98282001 98283000 "*" 0 0 -100
"chr5" 98296001 98297000 "*" 3.63445940010365e-11 8.58565209111204e-11 -100
"chr5" 98351001 98352000 "*" 5.55111512312578e-16 2.36485094870365e-15 -100
"chr5" 98699001 98700000 "*" 8.7349629751543e-09 1.47505871898511e-08 100
"chr5" 99383001 99384000 "*" 2.64951482975562e-09 4.82186011375752e-09 100
"chr5" 99385001 99386000 "*" 1.37828859436695e-10 3.01445544267661e-10 100
"chr5" 99738001 99739000 "*" 0 0 100
"chr5" 100218001 100219000 "*" 6.4324989779152e-11 1.47230020825092e-10 -100
"chr5" 100240001 100241000 "*" 3.53781981488055e-09 6.34912718011365e-09
59.4594594594595
"chr5" 101435001 101436000 "*" 4.01313771103418e-09 7.11882087237587e-09 -100
"chr5" 102000001 102001000 "*" 6.88338275267597e-15 2.60518659957509e-14 100
"chr5" 102201001 102202000 "*" 0 0 76.2962962962963
"chr5" 106081001 106082000 "*" 6.59550958292954e-09 1.13456194382773e-08 100
"chr5" 107144001 107145000 "*" 2.08507209609188e-07 2.89943420778527e-07
-63.6363636363636
"chr5" 107934001 107935000 "*" 4.26951363152739e-10 8.72510873516957e-10 -100
"chr5" 108627001 108628000 "*" 5.10702591327572e-15 1.96271840288234e-14 -100
"chr5" 109258001 109259000 "*" 1.98124849859482e-10 4.25157611354621e-10 -100
"chr5" 109603001 109604000 "*" 3.95353005888666e-09 7.03928529283733e-09 100
"chr5" 109700001 109701000 "*" 0 0 82.1428571428571
"chr5" 110230001 110231000 "*" 0 0 -62.4906785980611
"chr5" 110560001 110561000 "*" 0 0 89.3009985734665
"chr5" 110947001 110948000 "*" 1.94554372612288e-11 4.782471785654e-11
-53.6082474226804

"chr5" 111065001 111066000 "*" 1.30340183090993e-13 4.22662705118657e-13 -100
"chr5" 111689001 111690000 "*" 8.1956545862738e-05 7.7896169099937e-05
-54.5454545454545
"chr5" 111704001 111705000 "*" 7.68311851473413e-07 9.90964461140931e-07
67.741935483871
"chr5" 111756001 111757000 "*" 0 0 71.7460317460317
"chr5" 111806001 111807000 "*" 1.67299207820548e-08 2.71377429441055e-08 -100
"chr5" 112073001 112074000 "*" 0 0 -85.6492027334852
"chr5" 112197001 112198000 "*" 5.03264097062583e-13 1.51942428521737e-12 100
"chr5" 112630001 112631000 "*" 0 0 92.8416485900217
"chr5" 112823001 112824000 "*" 0 0 68.7723210067837
"chr5" 112824001 112825000 "*" 0 0 87.2
"chr5" 113997001 113998000 "*" 3.26405569239796e-14 1.13973003849234e-13 100
"chr5" 114264001 114265000 "*" 1.36824995777829e-10 2.99894878209423e-10
-86.0869565217391
"chr5" 115907001 115908000 "*" 0 0 100
"chr5" 115909001 115910000 "*" 0 0 83.8689820192814
"chr5" 116032001 116033000 "*" 4.73234496034536e-09 8.30627300264826e-09 100
"chr5" 116430001 116431000 "*" 3.30043248286671e-09 5.94497676880652e-09
-84.8101265822785
"chr5" 117564001 117565000 "*" 4.65637306490407e-10 9.46720524046618e-10 100
"chr5" 117570001 117571000 "*" 7.92025334206414e-11 1.7900239794719e-10
85.4166666666667
"chr5" 119083001 119084000 "*" 2.88779000712225e-11 6.92935809466068e-11 100
"chr5" 119150001 119151000 "*" 1.52794177310511e-09 2.87642564232045e-09 100
"chr5" 120559001 120560000 "*" 8.52140580320793e-12 2.19817259951489e-11 100
"chr5" 120599001 120600000 "*" 3.92685883809918e-13 1.19951200966592e-12 100
"chr5" 120952001 120953000 "*" 4.44320025216882e-10 9.06450423929973e-10
-58.9041095890411
"chr5" 121412001 121413000 "*" 0 0 86.4453665283541
"chr5" 121413001 121414000 "*" 0 0 50.3152993870816
"chr5" 121725001 121726000 "*" 5.794809077031e-12 1.52911192757929e-11 100
"chr5" 121899001 121900000 "*" 2.62900812231237e-13 8.21294938531841e-13 100
"chr5" 121920001 121921000 "*" 1.55431223447522e-14 5.6388152448458e-14 100
"chr5" 122181001 122182000 "*" 0 0 -100
"chr5" 122356001 122357000 "*" 3.40125705378114e-10 7.03784073980162e-10 -100
"chr5" 122648001 122649000 "*" 1.12376774552558e-12 3.24761818527664e-12 100
"chr5" 123624001 123625000 "*" 2.15125472990962e-10 4.58126659943874e-10 -100
"chr5" 123694001 123695000 "*" 6.92287338566189e-11 1.57605630124247e-10 100
"chr5" 124135001 124136000 "*" 1.50623957750895e-12 4.28756717183481e-12 -100
"chr5" 124820001 124821000 "*" 4.73234496034536e-09 8.30627300264826e-09 -100
"chr5" 125126001 125127000 "*" 1.14124265593318e-11 2.89138062669327e-11 -100
"chr5" 125665001 125666000 "*" 2.43005615629954e-09 4.45014222280278e-09 -100
"chr5" 126016001 126017000 "*" 3.95353005888666e-09 7.03928529283733e-09 -100
"chr5" 126187001 126188000 "*" 0 0 -100
"chr5" 126366001 126367000 "*" 0 0 57.9240037071362
"chr5" 126433001 126434000 "*" 1.35036426485158e-12 3.86629547363774e-12 100
"chr5" 126904001 126905000 "*" 8.01666433236647e-09 1.36392368970109e-08 -100
"chr5" 126989001 126990000 "*" 4.73234496034536e-09 8.30627300264826e-09 -100
"chr5" 127599001 127600000 "*" 0 0 -66.6666666666667
"chr5" 128796001 128797000 "*" 0 0 96.2857142857143
"chr5" 128797001 128798000 "*" 0 0 55.9877175025589

"chr5" 129240001 129241000 "*" 0 0 79.2431417054749
"chr5" 129614001 129615000 "*" 3.04628685166008e-07 4.14477782922613e-07
-92.3076923076923
"chr5" 130524001 130525000 "*" 6.66133814775094e-16 2.81595744474255e-15 -100
"chr5" 130755001 130756000 "*" 1.41954322647742e-05 1.51939032939975e-05
-71.4285714285714
"chr5" 130961001 130962000 "*" 4.78841410966879e-11 1.11468187156588e-10 -100
"chr5" 131236001 131237000 "*" 3.07531777821168e-14 1.07626052665081e-13 -100
"chr5" 131301001 131302000 "*" 3.73034936274053e-14 1.29420415488591e-13 -100
"chr5" 131392001 131393000 "*" 5.03393993156465e-11 1.16750305560645e-10 56
"chr5" 131415001 131416000 "*" 1.13140401492018e-08 1.87801380787728e-08 -100
"chr5" 131625001 131626000 "*" 3.05311331771918e-12 8.34639368772922e-12 -100
"chr5" 131648001 131649000 "*" 1.12458486967171e-09 2.15740815043925e-09 100
"chr5" 131787001 131788000 "*" 3.95353005888666e-09 7.03928529283733e-09 100
"chr5" 131821001 131822000 "*" 1.17092345819358e-08 1.940250185437e-08
-61.1111111111111
"chr5" 131831001 131832000 "*" 0 0 -77.5862068965517
"chr5" 131864001 131865000 "*" 2.15125472990962e-10 4.58126659943874e-10 100
"chr5" 132002001 132003000 "*" 4.26810808917821e-11 9.99587738000797e-11 52.5
"chr5" 132082001 132083000 "*" 0 0 -79.773156899811
"chr5" 132086001 132087000 "*" 3.18355668937897e-07 4.31991940783722e-07
-54.0540540540541
"chr5" 132165001 132166000 "*" 0 0 -67.1360337448049
"chr5" 132166001 132167000 "*" 0 0 -59.105180533752
"chr5" 132290001 132291000 "*" 0 0 100
"chr5" 132406001 132407000 "*" 9.08108033215171e-11 2.03407753381119e-10 100
"chr5" 132447001 132448000 "*" 1.11022302462516e-16 5.03662826618488e-16 100
"chr5" 132457001 132458000 "*" 0 0 -100
"chr5" 132537001 132538000 "*" 5.55111512312578e-16 2.36485094870365e-15
82.4561403508772
"chr5" 132625001 132626000 "*" 0 0 100
"chr5" 132666001 132667000 "*" 3.6700841921089e-08 5.65530226072258e-08 100
"chr5" 132670001 132671000 "*" 9.99200722162641e-15 3.71463121338097e-14
72.2222222222222
"chr5" 132693001 132694000 "*" 1.65423230669148e-14 5.98698695225911e-14 -100
"chr5" 132775001 132776000 "*" 4.28237856286984e-09 7.57162087834286e-09
-69.7674418604651
"chr5" 132786001 132787000 "*" 2.79987144580218e-12 7.68634726811898e-12 -100
"chr5" 132804001 132805000 "*" 0 0 -100
"chr5" 132916001 132917000 "*" 2.75335310107039e-14 9.69727903855348e-14 100
"chr5" 132946001 132947000 "*" 0 0 92.4272818455366
"chr5" 132948001 132949000 "*" 0 0 93.3974358974359
"chr5" 133005001 133006000 "*" 0.000118603937450446 0.000109926776654688
61.5384615384615
"chr5" 133022001 133023000 "*" 3.6700841921089e-08 5.65530226072258e-08 100
"chr5" 133024001 133025000 "*" 1.20994325669699e-11 3.05658541534845e-11 60
"chr5" 133135001 133136000 "*" 0 0 100
"chr5" 133184001 133185000 "*" 5.27009202944129e-09 9.20114230711386e-09
-62.0689655172414
"chr5" 133245001 133246000 "*" 5.69716129827924e-09 9.90984279067739e-09
71.7948717948718
"chr5" 133260001 133261000 "*" 2.88710388929303e-10 6.03399264731139e-10 -100

"chr5" 133304001 133305000 "*" 1.11022302462516e-16 5.03662826618488e-16 -100
"chr5" 133391001 133392000 "*" 2.1316282072803e-14 7.61791482056144e-14
-90.8045977011494
"chr5" 133547001 133548000 "*" 9.04570973681018e-10 1.76414389822173e-09 -100
"chr5" 133585001 133586000 "*" 1.59650012099277e-08 2.60326554446991e-08
-61.4942528735632
"chr5" 133747001 133748000 "*" 0 0 -75
"chr5" 133754001 133755000 "*" 3.31161027566917e-06 3.8994000350537e-06
66.6666666666667
"chr5" 133769001 133770000 "*" 2.65180414293908e-08 4.18265968961815e-08
77.7777777777778
"chr5" 133802001 133803000 "*" 5.66652058608952e-10 1.13800752810875e-09
-51.6129032258064
"chr5" 133908001 133909000 "*" 6.12207373684726e-09 1.06048652687696e-08 -60
"chr5" 133916001 133917000 "*" 0 0 -100
"chr5" 134034001 134035000 "*" 0 0 -100
"chr5" 134261001 134262000 "*" 1.67299207820548e-08 2.71377429441055e-08 -100
"chr5" 134283001 134284000 "*" 0 0 -78.6885245901639
"chr5" 134339001 134340000 "*" 3.9190872769268e-14 1.35416418933905e-13 -100
"chr5" 134361001 134362000 "*" 1.98124849859482e-10 4.25157611354621e-10 -100
"chr5" 134370001 134371000 "*" 1.98528951056431e-08 3.19051889717718e-08
-55.5555555555556
"chr5" 134470001 134471000 "*" 1.11022302462516e-16 5.03662826618488e-16
-70.5882352941177
"chr5" 134471001 134472000 "*" 0 0 -58.4397944199706
"chr5" 134489001 134490000 "*" 2.88779000712225e-11 6.92935809466068e-11 -100
"chr5" 134581001 134582000 "*" 3.7274627828765e-12 1.00985250136599e-11 -100
"chr5" 134582001 134583000 "*" 0 0 -92.5824175824176
"chr5" 134661001 134662000 "*" 2.75837855657768e-05 2.82530803747995e-05
66.6666666666667
"chr5" 134735001 134736000 "*" 0 0 73.8261987199664
"chr5" 134736001 134737000 "*" 2.68450373042128e-10 5.64465213127486e-10 100
"chr5" 134771001 134772000 "*" 6.71435517740626e-07 8.73129962575465e-07
66.6666666666667
"chr5" 134813001 134814000 "*" 7.63309104812038e-09 1.30345027323176e-08
-51.3350020859408
"chr5" 134872001 134873000 "*" 0 0 76.8674075988628
"chr5" 134902001 134903000 "*" 7.14983627858601e-14 2.3959391226828e-13
53.2710280373832
"chr5" 134903001 134904000 "*" 9.04570973681018e-10 1.76414389822173e-09 100
"chr5" 134907001 134908000 "*" 1.4391484892684e-08 2.36039305895519e-08
73.015873015873
"chr5" 135049001 135050000 "*" 4.85273081718418e-08 7.37333916927297e-08
74.7747747747748
"chr5" 135163001 135164000 "*" 2.455369241261e-12 6.8003688121118e-12 100
"chr5" 135165001 135166000 "*" 2.08814465718632e-09 3.86232567960463e-09 -100
"chr5" 135207001 135208000 "*" 1.39680959354571e-07 1.98463037958589e-07
-66.6666666666667
"chr5" 135350001 135351000 "*" 4.78841410966879e-11 1.11468187156588e-10 100
"chr5" 135391001 135392000 "*" 0 0 100
"chr5" 135423001 135424000 "*" 8.80406858527749e-14 2.91456141063452e-13 -100
"chr5" 135452001 135453000 "*" 1.39779633423487e-07 1.98463037958589e-07 100

"chr5" 135548001 135549000 "*" 0 0 -100
"chr5" 135552001 135553000 "*" 5.10702591327572e-15 1.96271840288234e-14 100
"chr5" 135651001 135652000 "*" 6.26246119994622e-06 7.07245363946971e-06 -66.0714285714286
"chr5" 135780001 135781000 "*" 1.35498279263402e-11 3.40114697187815e-11 59.6638655462185
"chr5" 136423001 136424000 "*" 6.4152538836737e-10 1.27477697728417e-09 100
"chr5" 136479001 136480000 "*" 4.26951363152739e-10 8.72510873516957e-10 100
"chr5" 136835001 136836000 "*" 0 0 53.0233134920635
"chr5" 136950001 136951000 "*" 1.29037891483108e-11 3.24582280286866e-11 -100
"chr5" 136975001 136976000 "*" 0 0 100
"chr5" 137480001 137481000 "*" 4.44089209850063e-16 1.91071758245033e-15 100
"chr5" 137547001 137548000 "*" 3.6700841921089e-08 5.65530226072258e-08 -100
"chr5" 137549001 137550000 "*" 5.32907051820075e-15 2.04626240984625e-14 -81.5126050420168
"chr5" 137783001 137784000 "*" 2.08180650607126e-07 2.89508626629748e-07 53.3333333333333
"chr5" 137787001 137788000 "*" 5.01025332333427e-10 1.01358031135889e-09 100
"chr5" 137790001 137791000 "*" 0 0 -100
"chr5" 137812001 137813000 "*" 2.6316739898391e-09 4.80154546935319e-09 50.9803921568627
"chr5" 137911001 137912000 "*" 0 0 -58.0139767518865
"chr5" 137938001 137939000 "*" 3.46500605985511e-13 1.06777978272295e-12 -100
"chr5" 137981001 137982000 "*" 6.08662167959073e-05 5.90595322645893e-05 64.2857142857143
"chr5" 138032001 138033000 "*" 2.69018141096922e-12 7.41487347763769e-12 100
"chr5" 138672001 138673000 "*" 0 0 -100
"chr5" 138680001 138681000 "*" 1.67299207820548e-08 2.71377429441055e-08 -100
"chr5" 138722001 138723000 "*" 2.88710388929303e-10 6.03399264731139e-10 100
"chr5" 138731001 138732000 "*" 6.11832806640678e-12 1.61045549953474e-11 -59.0909090909091
"chr5" 138861001 138862000 "*" 1.67556168939598e-09 3.14290562290774e-09 -87.7551020408163
"chr5" 139029001 139030000 "*" 1.11022302462516e-16 5.03662826618488e-16 90.0621118012422
"chr5" 139040001 139041000 "*" 0 0 -76.880709651794
"chr5" 139047001 139048000 "*" 0 0 -64.640946853306
"chr5" 139064001 139065000 "*" 0 0 -100
"chr5" 139071001 139072000 "*" 4.08209022140227e-11 9.57770690255255e-11 100
"chr5" 139125001 139126000 "*" 3.24754667602178e-11 7.73276012738648e-11 -55.1724137931034
"chr5" 139134001 139135000 "*" 6.92287338566189e-11 1.57605630124247e-10 -100
"chr5" 139142001 139143000 "*" 3.33066907387547e-16 1.4495649018245e-15 -100
"chr5" 139192001 139193000 "*" 2.8387625583548e-11 6.83215813633077e-11 65.3846153846154
"chr5" 139202001 139203000 "*" 4.56634730028327e-13 1.38435677008113e-12 -74.0740740740741
"chr5" 139204001 139205000 "*" 4.19675405538555e-12 1.1282709471651e-11 68.1306306306306
"chr5" 139226001 139227000 "*" 4.65637306490407e-10 9.46720524046618e-10 100
"chr5" 139227001 139228000 "*" 0 0 55.6569370753013
"chr5" 139236001 139237000 "*" 2.30604424444891e-12 6.41235278998423e-12 58.75

"chr5" 139244001 139245000 "*" 6.4152538836737e-10 1.27477697728417e-09 100
"chr5" 139256001 139257000 "*" 2.66891642597411e-06 3.18759115320439e-06
61.2903225806452
"chr5" 139270001 139271000 "*" 4.73234496034536e-09 8.30627300264826e-09 100
"chr5" 139283001 139284000 "*" 0 0 56.4141493930024
"chr5" 139285001 139286000 "*" 0 0 100
"chr5" 139304001 139305000 "*" 3.24846816113222e-11 7.7345126390972e-11
-61.9718309859155
"chr5" 139362001 139363000 "*" 2.88710388929303e-10 6.03399264731139e-10 -100
"chr5" 139422001 139423000 "*" 0 0 88.4440775897648
"chr5" 139423001 139424000 "*" 0 0 88.5714285714286
"chr5" 139488001 139489000 "*" 7.96572725558775e-08 1.17059398619726e-07
78.4883720930233
"chr5" 139492001 139493000 "*" 1.33226762955019e-15 5.48133041560118e-15
56.3380281690141
"chr5" 139618001 139619000 "*" 1.25284005392245e-10 2.75649590272477e-10 -100
"chr5" 139693001 139694000 "*" 9.99200722162641e-16 4.15382497462808e-15
-97.2222222222222
"chr5" 139782001 139783000 "*" 3.6892711108294e-13 1.13266255090498e-12 -100
"chr5" 139943001 139944000 "*" 1.61237689866311e-12 4.56615813671789e-12 100
"chr5" 140012001 140013000 "*" 0 0 85.7371794871795
"chr5" 140261001 140262000 "*" 0 0 50.4761904761905
"chr5" 140354001 140355000 "*" 3.95353005888666e-09 7.03928529283733e-09 100
"chr5" 140532001 140533000 "*" 2.29243291016701e-11 5.57748191208025e-11
66.6666666666667
"chr5" 140614001 140615000 "*" 6.4956040546349e-11 1.48608631534465e-10
-60.8695652173913
"chr5" 140622001 140623000 "*" 9.2148511043888e-15 3.43705279328267e-14 100
"chr5" 140803001 140804000 "*" 4.17776924166446e-13 1.27154538549514e-12
-58.974358974359
"chr5" 140819001 140820000 "*" 2.36632935468606e-12 6.57165986616064e-12
-76.7441860465116
"chr5" 141137001 141138000 "*" 1.68305258618773e-10 3.6409036579438e-10
-54.9295774647887
"chr5" 141154001 141155000 "*" 2.02327044007689e-12 5.65300384792621e-12 -100
"chr5" 141175001 141176000 "*" 1.11022302462516e-16 5.03662826618488e-16
74.468085106383
"chr5" 141190001 141191000 "*" 5.04263297784746e-13 1.52078941532818e-12 100
"chr5" 141293001 141294000 "*" 0 0 54.4379180943058
"chr5" 141313001 141314000 "*" 3.95353005888666e-09 7.03928529283733e-09 100
"chr5" 141690001 141691000 "*" 1.40433358830627e-07 1.99353883410833e-07
-77.7049180327869
"chr5" 141699001 141700000 "*" 1.20591092667155e-10 2.66075324129686e-10 -100
"chr5" 141703001 141704000 "*" 3.33066907387547e-16 1.4495649018245e-15
-91.5254237288136
"chr5" 141705001 141706000 "*" 0 0 -94.300518134715
"chr5" 141722001 141723000 "*" 8.80406858527749e-14 2.91456141063452e-13 -100
"chr5" 141732001 141733000 "*" 2.33146835171283e-15 9.32818831833246e-15 100
"chr5" 142007001 142008000 "*" 5.01025332333427e-10 1.01358031135889e-09 100
"chr5" 142032001 142033000 "*" 2.10408912515447e-10 4.49851315143281e-10
71.4285714285714
"chr5" 142444001 142445000 "*" 9.65729496371637e-10 1.87255912493232e-09 -100

"chr5" 142491001 142492000 "*" 1.91224813761437e-12 5.36527594469447e-12 -100
"chr5" 142587001 142588000 "*" 7.2471517675865e-10 1.43077717181584e-09
-95.6521739130435
"chr5" 142617001 142618000 "*" 5.6621374255883e-15 2.16468580166064e-14 100
"chr5" 142783001 142784000 "*" 0 0 64.4327503284984
"chr5" 142907001 142908000 "*" 4.01313771103418e-09 7.11882087237587e-09 -100
"chr5" 143015001 143016000 "*" 1.56863411149288e-12 4.4469621057462e-12 -100
"chr5" 143192001 143193000 "*" 0 0 100
"chr5" 143353001 143354000 "*" 4.42779146681005e-12 1.18636840803301e-11 62.5
"chr5" 143638001 143639000 "*" 0 0 -100
"chr5" 143801001 143802000 "*" 4.01313771103418e-09 7.11882087237587e-09 -100
"chr5" 143995001 143996000 "*" 4.78841410966879e-11 1.11468187156588e-10 -100
"chr5" 144036001 144037000 "*" 1.39779633423487e-07 1.98463037958589e-07 -100
"chr5" 145438001 145439000 "*" 6.37490060739765e-13 1.89496793587877e-12 -100
"chr5" 145581001 145582000 "*" 4.18423790060629e-08 6.40896891088311e-08 60
"chr5" 145723001 145724000 "*" 0.000139451556673498 0.000127724714298755
58.3333333333333
"chr5" 145998001 145999000 "*" 2.455369241261e-12 6.8003688121118e-12 -100
"chr5" 146188001 146189000 "*" 4.99020824662466e-11 1.15790792586157e-10 -100
"chr5" 146534001 146535000 "*" 0 0 100
"chr5" 146832001 146833000 "*" 0 0 86.9198312236287
"chr5" 146833001 146834000 "*" 0 0 96.4125560538117
"chr5" 146850001 146851000 "*" 6.59550958292954e-09 1.13456194382773e-08 -100
"chr5" 146892001 146893000 "*" 2.70339306496226e-13 8.42355472617862e-13 100
"chr5" 147197001 147198000 "*" 6.59550958292954e-09 1.13456194382773e-08 100
"chr5" 147341001 147342000 "*" 1.11022302462516e-16 5.03662826618488e-16 -100
"chr5" 147835001 147836000 "*" 1.72610481463664e-10 3.73016724751756e-10
-66.6666666666667
"chr5" 147852001 147853000 "*" 1.96509475358653e-13 6.23868565133654e-13 100
"chr5" 148172001 148173000 "*" 2.79987144580218e-12 7.68634726811898e-12 100
"chr5" 148205001 148206000 "*" 0 0 94.5121951219512
"chr5" 148216001 148217000 "*" 5.46561531589163e-08 8.24908389065331e-08
78.9473684210526
"chr5" 148223001 148224000 "*" 1.39779633423487e-07 1.98463037958589e-07 -100
"chr5" 148454001 148455000 "*" 2.43005615629954e-09 4.45014222280278e-09 100
"chr5" 148586001 148587000 "*" 3.33066907387547e-16 1.4495649018245e-15
-81.4519345831671
"chr5" 148651001 148652000 "*" 0 0 64.0106275121699
"chr5" 148662001 148663000 "*" 4.70422271470206e-07 6.23903462099788e-07
-64.2857142857143
"chr5" 148684001 148685000 "*" 3.23130888713052e-09 5.82587689288471e-09
80.9523809523809
"chr5" 148685001 148686000 "*" 1.53760537635605e-09 2.89396461573497e-09
-78.2608695652174
"chr5" 148712001 148713000 "*" 7.0006535457523e-08 1.03695832512054e-07 -100
"chr5" 148756001 148757000 "*" 0 0 100
"chr5" 148772001 148773000 "*" 0 0 63.3451075168867
"chr5" 148785001 148786000 "*" 0 0 -100
"chr5" 148851001 148852000 "*" 0 0 -59.0163934426229
"chr5" 148852001 148853000 "*" 4.44089209850063e-16 1.91071758245033e-15 -100
"chr5" 148960001 148961000 "*" 2.90581101536347e-06 3.45237921768075e-06
-68.4848484848485

"chr5" 148961001 148962000 "*" 0 0 -64.5083932853717
"chr5" 149025001 149026000 "*" 0 0 76.9230769230769
"chr5" 149154001 149155000 "*" 2.4743410653727e-08 3.91466886830741e-08
-85.4545454545455
"chr5" 149160001 149161000 "*" 1.46149758961656e-12 4.17102684849443e-12
-95.7746478873239
"chr5" 149232001 149233000 "*" 8.57092175010621e-13 2.51149198099779e-12 -100
"chr5" 149447001 149448000 "*" 9.65729496371637e-10 1.87255912493232e-09 -100
"chr5" 149461001 149462000 "*" 4.06771755168123e-08 6.23725393697639e-08
-65.2777777777778
"chr5" 149504001 149505000 "*" 8.56120729864074e-10 1.67639616737578e-09
-63.2804232804233
"chr5" 149506001 149507000 "*" 5.48638023900594e-10 1.10374357336421e-09 -100
"chr5" 149532001 149533000 "*" 7.07661398369197e-08 1.04737273899807e-07
-65.5172413793103
"chr5" 149590001 149591000 "*" 1.17905685215192e-13 3.83653245040640e-13 100
"chr5" 149598001 149599000 "*" 6.62958576924666e-12 1.73620572057889e-11 -100
"chr5" 149618001 149619000 "*" 7.94902956313681e-06 8.84364017398143e-06
63.6363636363636
"chr5" 149622001 149623000 "*" 8.25538540860649e-08 1.2109413635802e-07
60.6060606060606
"chr5" 149650001 149651000 "*" 1.26565424807268e-14 4.63923203146481e-14 -100
"chr5" 149660001 149661000 "*" 3.15283238405684e-08 4.92791128504977e-08
55.2173913043478
"chr5" 149667001 149668000 "*" 2.84266206793671e-05 2.90599754501846e-05
-51.6666666666667
"chr5" 149668001 149669000 "*" 0 0 100
"chr5" 149671001 149672000 "*" 7.66053886991358e-15 2.88426834415713e-14
54.1666666666667
"chr5" 149710001 149711000 "*" 3.03979064142368e-13 9.42350883309283e-13 -100
"chr5" 149720001 149721000 "*" 9.80763359414993e-10 1.8980032424715e-09 100
"chr5" 149877001 149878000 "*" 0 0 -99.5169082125604
"chr5" 149889001 149890000 "*" 0 0 100
"chr5" 149964001 149965000 "*" 3.57747192967217e-05 3.59800533827634e-05
-53.8461538461538
"chr5" 149978001 149979000 "*" 2.1316282072803e-12 5.94400609072327e-12 62.5
"chr5" 150402001 150403000 "*" 3.04324343503026e-11 7.26599935673121e-11 100
"chr5" 150427001 150428000 "*" 7.7715611723761e-16 3.26213507634405e-15 100
"chr5" 150438001 150439000 "*" 9.77801195567451e-08 1.42032362421848e-07
82.1428571428571
"chr5" 150442001 150443000 "*" 0.000808777120367532 0.000651834190530565
-58.3333333333333
"chr5" 150471001 150472000 "*" 1.7739599909028e-06 2.17512049368634e-06
66.6666666666667
"chr5" 150496001 150497000 "*" 2.22044604925031e-16 9.81641919380259e-16 100
"chr5" 150546001 150547000 "*" 1.66533453693773e-15 6.7629186866784e-15 100
"chr5" 150629001 150630000 "*" 2.31469791711625e-06 2.79034791839104e-06 -75
"chr5" 150738001 150739000 "*" 0 0 67.3166666666667
"chr5" 150801001 150802000 "*" 3.60556792551314e-05 3.6239786682982e-05
60.7142857142857
"chr5" 150838001 150839000 "*" 3.04201108747293e-14 1.06563274901204e-13
-61.9920891765552

"chr5" 150880001 150881000 "*" 3.03090885722668e-14 1.06210046699299e-13 100
"chr5" 150889001 150890000 "*" 4.73234496034536e-09 8.30627300264826e-09 -100
"chr5" 150920001 150921000 "*" 4.88498130835069e-15 1.8824139250584e-14 -100
"chr5" 150974001 150975000 "*" 8.24113111086433e-10 1.61717625106406e-09
-78.8461538461538
"chr5" 151228001 151229000 "*" 2.18713935851156e-14 7.80893646430581e-14 -100
"chr5" 151504001 151505000 "*" 1.17905685215192e-13 3.83653245040640e-13 -100
"chr5" 152456001 152457000 "*" 8.36159640416057e-08 1.22576630789268e-07
51.219512195122
"chr5" 152475001 152476000 "*" 1.74853465040314e-11 4.32752710900609e-11 100
"chr5" 152870001 152871000 "*" 5.07848207931261e-11 1.17730972026569e-10
-51.5151515151515
"chr5" 153311001 153312000 "*" 3.45022373671178e-07 4.66205089083695e-07
-52.3809523809524
"chr5" 153749001 153750000 "*" 1.12458486967171e-09 2.15740815043925e-09 -100
"chr5" 153765001 153766000 "*" 0 0 -100
"chr5" 153863001 153864000 "*" 5.69100322422855e-13 1.70455606161772e-12
56.2091503267974
"chr5" 153864001 153865000 "*" 4.02167188440217e-12 1.08266700497926e-11 -100
"chr5" 153879001 153880000 "*" 1.12376774552558e-12 3.24761818527664e-12 100
"chr5" 153885001 153886000 "*" 3.33066907387547e-16 1.4495649018245e-15
-52.0833333333333
"chr5" 153888001 153889000 "*" 1.70641278884887e-12 4.81986032810646e-12 100
"chr5" 153941001 153942000 "*" 0 0 100
"chr5" 153945001 153946000 "*" 0 0 100
"chr5" 153988001 153989000 "*" 1.29037891483108e-11 3.24582280286866e-11 -100
"chr5" 154027001 154028000 "*" 0 0 82.89387487717
"chr5" 154062001 154063000 "*" 0 0 -91.6666666666667
"chr5" 154161001 154162000 "*" 8.58968451922237e-12 2.213554983903e-11 100
"chr5" 154939001 154940000 "*" 8.51539899693332e-06 9.42634437184662e-06 71.875
"chr5" 154965001 154966000 "*" 0 0 -100
"chr5" 155620001 155621000 "*" 1.14124265593318e-11 2.89138062669327e-11 -100
"chr5" 155801001 155802000 "*" 0 0 -100
"chr5" 156378001 156379000 "*" 6.55064225085766e-10 1.30028591327274e-09
-86.9565217391304
"chr5" 156418001 156419000 "*" 8.01666433236647e-09 1.36392368970109e-08 100
"chr5" 156644001 156645000 "*" 4.99900121297969e-12 1.32921056339624e-11 100
"chr5" 156779001 156780000 "*" 1.10442097427388e-05 1.20217549185931e-05
59.2592592592593
"chr5" 156887001 156888000 "*" 0 0 57.576863594185
"chr5" 156916001 156917000 "*" 6.4152538836737e-10 1.27477697728417e-09 100
"chr5" 157002001 157003000 "*" 0 0 67.9121995655525
"chr5" 157046001 157047000 "*" 1.46911291798446e-07 2.0800406109474e-07
73.4741784037559
"chr5" 157115001 157116000 "*" 0 0 100
"chr5" 157193001 157194000 "*" 5.01025332333427e-10 1.01358031135889e-09 100
"chr5" 157365001 157366000 "*" 7.0006535457523e-08 1.03695832512054e-07 -100
"chr5" 157373001 157374000 "*" 0 0 100
"chr5" 158004001 158005000 "*" 2.69007038866675e-13 8.38831022628993e-13 100
"chr5" 158106001 158107000 "*" 2.8421709430404e-14 9.99616402633955e-14 100
"chr5" 158362001 158363000 "*" 1.35447209004269e-14 4.94674708073053e-14 100
"chr5" 158488001 158489000 "*" 3.68776608039578e-06 4.31211440036956e-06

-62.0689655172414
"chr5" 158526001 158527000 "*" 0 0 74.2453436095055
"chr5" 158737001 158738000 "*" 0 0 100
"chr5" 158743001 158744000 "*" 0 0 -100
"chr5" 158758001 158759000 "*" 1.98365768255826e-11 4.85612402473442e-11 100
"chr5" 158860001 158861000 "*" 0 0 67.7966101694915
"chr5" 159047001 159048000 "*" 2.64951482975562e-09 4.82186011375752e-09 100
"chr5" 159309001 159310000 "*" 4.01313771103418e-09 7.11882087237587e-09 100
"chr5" 159378001 159379000 "*" 3.63445940010365e-11 8.58565209111204e-11 100
"chr5" 159436001 159437000 "*" 0 0 -100
"chr5" 159615001 159616000 "*" 0 0 69.0909090909091
"chr5" 159625001 159626000 "*" 0 0 -100
"chr5" 159626001 159627000 "*" 0 0 82.258064516129
"chr5" 159716001 159717000 "*" 3.34128893442198e-08 5.19772864618523e-08 -100
"chr5" 159894001 159895000 "*" 0 0 100
"chr5" 160360001 160361000 "*" 2.22044604925031e-16 9.81641919380259e-16 100
"chr5" 160361001 160362000 "*" 2.06501482580279e-14 7.3890370570066e-14 75
"chr5" 160974001 160975000 "*" 0 0 83.015873015873
"chr5" 161495001 161496000 "*" 2.06501482580279e-14 7.3890370570066e-14 -95
"chr5" 162177001 162178000 "*" 5.55111512312578e-16 2.36485094870365e-15 100
"chr5" 162309001 162310000 "*" 7.35548152386301e-08 1.08588645363448e-07 55
"chr5" 162592001 162593000 "*" 0 0 100
"chr5" 164173001 164174000 "*" 3.90465437760668e-13 1.19307893943277e-12 -100
"chr5" 164653001 164654000 "*" 7.91660904608804e-08 1.16374497590614e-07
62.1951219512195
"chr5" 164891001 164892000 "*" 1.67299207820548e-08 2.71377429441055e-08 -100
"chr5" 165828001 165829000 "*" 1.73560145144691e-08 2.80948669481175e-08
-60.9756097560976
"chr5" 166236001 166237000 "*" 0 0 -62.2641509433962
"chr5" 166889001 166890000 "*" 2.15125472990962e-10 4.58126659943874e-10 -100
"chr5" 167288001 167289000 "*" 1.65143565489245e-10 3.57501128117734e-10
84.3243243243243
"chr5" 167333001 167334000 "*" 5.51780843238703e-14 1.87243792572872e-13 -100
"chr5" 167442001 167443000 "*" 4.2142935618994e-07 5.62423023854377e-07
-71.2121212121212
"chr5" 167466001 167467000 "*" 5.08482145278322e-14 1.73432706170696e-13 -100
"chr5" 167555001 167556000 "*" 5.794809077031e-12 1.52911192757929e-11 -100
"chr5" 167581001 167582000 "*" 0 0 -100
"chr5" 167582001 167583000 "*" 0 0 -59.887839433294
"chr5" 167589001 167590000 "*" 1.11022302462516e-16 5.03662826618488e-16 -100
"chr5" 167605001 167606000 "*" 7.00550728538474e-14 2.34936744632826e-13 52
"chr5" 167626001 167627000 "*" 2.60902410786912e-14 9.21424641147685e-14
-81.8181818181818
"chr5" 167683001 167684000 "*" 7.82707232360735e-14 2.61332834389318e-13
66.6666666666667
"chr5" 167695001 167696000 "*" 6.49580389477933e-12 1.70534680245236e-11
-76.8115942028985
"chr5" 167719001 167720000 "*" 0 0 -100
"chr5" 167741001 167742000 "*" 1.13140401492018e-08 1.87801380787728e-08 -100
"chr5" 167778001 167779000 "*" 1.15345030045066e-05 1.25186825636811e-05
52.3809523809524
"chr5" 168145001 168146000 "*" 2.43005615629954e-09 4.45014222280278e-09 100

"chr5" 168148001 168149000 "*" 2.69018141096922e-12 7.41487347763769e-12 100
"chr5" 168251001 168252000 "*" 9.79912069397626e-05 9.1998862536197e-05
-56.7567567567568
"chr5" 168271001 168272000 "*" 6.34375885155691e-11 1.45441875258413e-10
-66.6666666666667
"chr5" 168336001 168337000 "*" 1.45430656406376e-09 2.75521741516792e-09
-78.9473684210526
"chr5" 168375001 168376000 "*" 7.0006535457523e-08 1.03695832512054e-07 -100
"chr5" 168530001 168531000 "*" 4.04946233650838e-05 4.0376350196346e-05
-64.7058823529412
"chr5" 168631001 168632000 "*" 2.08995487582797e-10 4.46944006972663e-10 -100
"chr5" 168652001 168653000 "*" 1.22457599616155e-12 3.52441478267078e-12 -100
"chr5" 168713001 168714000 "*" 2.77555756156289e-15 1.10015415195978e-14 -100
"chr5" 168948001 168949000 "*" 0 0 100
"chr5" 169063001 169064000 "*" 5.17798914856371e-10 1.04609249525638e-09
-53.3333333333333
"chr5" 169071001 169072000 "*" 8.7349629751543e-09 1.47505871898511e-08 100
"chr5" 169075001 169076000 "*" 2.1094237467878e-15 8.47434540879246e-15 100
"chr5" 169390001 169391000 "*" 7.08114522834924e-05 6.79693915501506e-05 -62.5
"chr5" 169417001 169418000 "*" 7.54691820148423e-09 1.28968575931701e-08
74.2424242424242
"chr5" 169446001 169447000 "*" 1.054283589208e-06 1.33429012030175e-06
51.1111111111111
"chr5" 169447001 169448000 "*" 4.86753970463383e-11 1.13145908161915e-10 100
"chr5" 169535001 169536000 "*" 1.17905685215192e-13 3.83653245040640e-13 100
"chr5" 169575001 169576000 "*" 1.98124849859482e-10 4.25157611354621e-10 100
"chr5" 169689001 169690000 "*" 2.43005615629954e-09 4.45014222280278e-09 -100
"chr5" 169704001 169705000 "*" 2.16382467499443e-13 6.82775854166559e-13 100
"chr5" 169724001 169725000 "*" 1.12458486967171e-09 2.15740815043925e-09 100
"chr5" 169746001 169747000 "*" 6.48495701582874e-11 1.48385397387759e-10
95.8333333333333
"chr5" 169783001 169784000 "*" 1.11022302462516e-16 5.03662826618488e-16 100
"chr5" 169789001 169790000 "*" 0 0 98.3739837398374
"chr5" 169805001 169806000 "*" 0 0 50.7853403141361
"chr5" 169809001 169810000 "*" 1.93720595120794e-11 4.76309924836636e-11 -100
"chr5" 169816001 169817000 "*" 2.67660076822551e-05 2.74676737224733e-05
-65.7894736842105
"chr5" 169821001 169822000 "*" 0 0 100
"chr5" 169837001 169838000 "*" 1.78512946491516e-06 2.1878826958132e-06
-65.5172413793103
"chr5" 169840001 169841000 "*" 7.56453604285889e-05 7.22898040868576e-05
58.2089552238806
"chr5" 169842001 169843000 "*" 2.02327044007689e-12 5.65300384792621e-12 100
"chr5" 169863001 169864000 "*" 3.33066907387547e-16 1.4495649018245e-15
51.5269374819937
"chr5" 169867001 169868000 "*" 6.65646857338498e-05 6.41959941692905e-05
53.8461538461538
"chr5" 169911001 169912000 "*" 1.2404663962684e-09 2.36887234713147e-09
83.3333333333333
"chr5" 169917001 169918000 "*" 3.95353005888666e-09 7.03928529283733e-09 100
"chr5" 169980001 169981000 "*" 0 0 100
"chr5" 170032001 170033000 "*" 5.01025332333427e-10 1.01358031135889e-09 100

"chr5" 170053001 170054000 "*" 6.66133814775094e-16 2.81595744474255e-15 71.4285714285714
"chr5" 170056001 170057000 "*" 1.36471023370888e-09 2.59372372847633e-09 71.7391304347826
"chr5" 170068001 170069000 "*" 8.24873502835999e-11 1.86128689830788e-10 -59.2592592592593
"chr5" 170079001 170080000 "*" 3.90465437760668e-13 1.19307893943277e-12 100
"chr5" 170168001 170169000 "*" 1.56863411149288e-12 4.4469621057462e-12 100
"chr5" 170169001 170170000 "*" 0 0 100
"chr5" 170290001 170291000 "*" 2.33028818463765e-10 4.93719283454501e-10 100
"chr5" 170749001 170750000 "*" 4.73234496034536e-09 8.30627300264826e-09 -100
"chr5" 170756001 170757000 "*" 1.37828859436695e-10 3.01445544267661e-10 100
"chr5" 170764001 170765000 "*" 1.29268928894533e-10 2.83960248803703e-10 -69.4444444444444
"chr5" 170775001 170776000 "*" 8.28241718409117e-06 9.18550771594789e-06 57.5
"chr5" 170862001 170863000 "*" 4.44089209850063e-16 1.91071758245033e-15 -58.3333333333333
"chr5" 170867001 170868000 "*" 5.32907051820075e-13 1.60271247130866e-12 85.7142857142857
"chr5" 170869001 170870000 "*" 1.89692705987454e-12 5.32605167298056e-12 56.25
"chr5" 170874001 170875000 "*" 6.92287338566189e-11 1.57605630124247e-10 100
"chr5" 170875001 170876000 "*" 3.95353005888666e-09 7.03928529283733e-09 100
"chr5" 170881001 170882000 "*" 2.67283972732457e-11 6.45686178157698e-11 100
"chr5" 170894001 170895000 "*" 0.000354266918853763 0.000303242292809723 56.5217391304348
"chr5" 170955001 170956000 "*" 0 0 52.8301886792453
"chr5" 171048001 171049000 "*" 2.73014943985572e-11 6.58595916221891e-11 100
"chr5" 171073001 171074000 "*" 8.27655866064703e-09 1.40544602518333e-08 57.9710144927536
"chr5" 171075001 171076000 "*" 1.46549439250521e-14 5.33330076973935e-14 100
"chr5" 171120001 171121000 "*" 2.00227675550835e-08 3.20459176617961e-08 -100
"chr5" 171131001 171132000 "*" 2.08814465718632e-09 3.86232567960463e-09 -100
"chr5" 171151001 171152000 "*" 6.59550958292954e-09 1.13456194382773e-08 -100
"chr5" 171176001 171177000 "*" 6.84867742828388e-06 7.69126994020664e-06 61.3333333333333
"chr5" 171206001 171207000 "*" 2.4535928844216e-14 8.69144396197119e-14 100
"chr5" 171486001 171487000 "*" 9.13602526964041e-13 2.66761500900036e-12 85.7142857142857
"chr5" 171530001 171531000 "*" 0 0 -100
"chr5" 171578001 171579000 "*" 8.03801469828613e-14 2.67679295675503e-13 -94.4723618090452
"chr5" 171654001 171655000 "*" 4.73234496034536e-09 8.30627300264826e-09 100
"chr5" 171839001 171840000 "*" 1.39779633423487e-07 1.98463037958589e-07 100
"chr5" 171840001 171841000 "*" 1.42829192917304e-10 3.11605478626202e-10 81.8181818181818
"chr5" 171847001 171848000 "*" 0 0 100
"chr5" 171886001 171887000 "*" 7.84651036493944e-05 7.48061766364768e-05 -56.5217391304348
"chr5" 171893001 171894000 "*" 0 0 -92.7143778207608
"chr5" 171911001 171912000 "*" 5.00566255112744e-12 1.33081206745024e-11 -67.743119266055
"chr5" 171919001 171920000 "*" 1.50623957750895e-12 4.28756717183481e-12 -100

"chr5" 171924001 171925000 "*" 1.22124532708767e-15 5.03921984217778e-15 -100
"chr5" 171925001 171926000 "*" 3.95353005888666e-09 7.03928529283733e-09 -100
"chr5" 171949001 171950000 "*" 2.04292838201781e-09 3.79300264241924e-09
66.6666666666667
"chr5" 171954001 171955000 "*" 1.35890187991095e-11 3.40841971363279e-11 -100
"chr5" 171991001 171992000 "*" 2.88779000712225e-11 6.92935809466068e-11 100
"chr5" 172011001 172012000 "*" 1.11022302462516e-16 5.03662826618488e-16 -100
"chr5" 172014001 172015000 "*" 6.21414031343193e-12 1.63459166469347e-11
-76.6666666666667
"chr5" 172039001 172040000 "*" 1.17461596005342e-13 3.8279067575877e-13 100
"chr5" 172084001 172085000 "*" 1.70641278884887e-12 4.81986032810646e-12 100
"chr5" 172086001 172087000 "*" 7.27481818119102e-08 1.07497206386442e-07
70.8333333333333
"chr5" 172124001 172125000 "*" 1.11022302462516e-16 5.03662826618488e-16 -100
"chr5" 172128001 172129000 "*" 8.65973959207622e-14 2.87362558212404e-13 -100
"chr5" 172140001 172141000 "*" 0 0 100
"chr5" 172153001 172154000 "*" 0.000792090732462114 0.000639295093468792
52.3809523809524
"chr5" 172199001 172200000 "*" 1.08091313677505e-12 3.13082270510317e-12
76.551724137931
"chr5" 172247001 172248000 "*" 0 0 -67.741935483871
"chr5" 172277001 172278000 "*" 0 0 100
"chr5" 172294001 172295000 "*" 1.35447209004269e-14 4.94674708073053e-14 -100
"chr5" 172335001 172336000 "*" 4.0635857900817e-08 6.23149132293182e-08
66.6666666666667
"chr5" 172347001 172348000 "*" 5.10444134667054e-05 5.01471223720755e-05
-63.8235294117647
"chr5" 172359001 172360000 "*" 0 0 -71.8367346938775
"chr5" 172360001 172361000 "*" 2.87422404341431e-05 2.93567189445702e-05
57.6923076923077
"chr5" 172387001 172388000 "*" 0 0 -97.6744186046512
"chr5" 172409001 172410000 "*" 6.4324989779152e-11 1.47230020825092e-10 100
"chr5" 172638001 172639000 "*" 1.38107564939816e-07 1.96735395029093e-07
81.4814814814815
"chr5" 172665001 172666000 "*" 0 0 59.7051597051597
"chr5" 172666001 172667000 "*" 1.24344978758018e-14 4.56627092294518e-14 100
"chr5" 172710001 172711000 "*" 0 0 91.6083916083916
"chr5" 172711001 172712000 "*" 3.26516591542259e-13 1.00940437789288e-12
63.6363636363636
"chr5" 172714001 172715000 "*" 1.99110040377537e-05 2.08378814937083e-05
-55.3956834532374
"chr5" 172731001 172732000 "*" 2.23658616183009e-07 3.09711172095053e-07
72.1518987341772
"chr5" 172738001 172739000 "*" 4.08209022140227e-11 9.57770690255255e-11 -100
"chr5" 172753001 172754000 "*" 1.52794177310511e-09 2.87642564232045e-09 100
"chr5" 172754001 172755000 "*" 0 0 90.3809523809524
"chr5" 172755001 172756000 "*" 0 0 93.3575505967826
"chr5" 172783001 172784000 "*" 9.08108033215171e-11 2.03407753381119e-10 100
"chr5" 172835001 172836000 "*" 1.94820970467191e-09 3.62505125190229e-09
92.8571428571429
"chr5" 172851001 172852000 "*" 2.37283860671234e-07 3.27577954042927e-07
64.7058823529412

"chr5" 172876001 172877000 "*" 1.11535071055657e-05 1.21313840509748e-05 -64.1509433962264
"chr5" 172897001 172898000 "*" 9.38683120121908e-09 1.5788727503198e-08 56.4102564102564
"chr5" 172902001 172903000 "*" 0 0 100
"chr5" 172905001 172906000 "*" 2.33028818463765e-10 4.93719283454501e-10 100
"chr5" 172925001 172926000 "*" 1.26565424807268e-14 4.63923203146481e-14 -100
"chr5" 172926001 172927000 "*" 2.64325346821526e-06 3.15856923971574e-06 -53.8461538461538
"chr5" 173000001 173001000 "*" 0 0 100
"chr5" 173004001 173005000 "*" 3.29028693180078e-09 5.92785412906053e-09 88.8888888888889
"chr5" 173011001 173012000 "*" 1.37828859436695e-10 3.01445544267661e-10 -100
"chr5" 173024001 173025000 "*" 7.67056418382595e-11 1.7359448580318e-10 -100
"chr5" 173094001 173095000 "*" 7.99360577730113e-14 2.66349298093451e-13 -88.3116883116883
"chr5" 173129001 173130000 "*" 2.15125472990962e-10 4.58126659943874e-10 -100
"chr5" 173171001 173172000 "*" 0 0 100
"chr5" 173180001 173181000 "*" 4.91828799908944e-14 1.68192819018269e-13 -100
"chr5" 173278001 173279000 "*" 0 0 100
"chr5" 173282001 173283000 "*" 4.24070778493046e-11 9.93449579809611e-11 -65.2173913043478
"chr5" 173283001 173284000 "*" 3.66181607480698e-07 4.92728439089831e-07 -75
"chr5" 173327001 173328000 "*" 2.08814465718632e-09 3.86232567960463e-09 -100
"chr5" 173478001 173479000 "*" 2.02060590481778e-13 6.40127405465654e-13 100
"chr5" 173526001 173527000 "*" 5.23756826442678e-06 5.99068636099677e-06 55.3846153846154
"chr5" 173536001 173537000 "*" 8.01666433236647e-09 1.36392368970109e-08 100
"chr5" 173538001 173539000 "*" 1.68625191587779e-09 3.16206767921358e-09 -86.8217054263566
"chr5" 173563001 173564000 "*" 3.6700841921089e-08 5.65530226072258e-08 100
"chr5" 173573001 173574000 "*" 5.6362137179633e-11 1.29963270300065e-10 -100
"chr5" 173730001 173731000 "*" 1.49613654798486e-12 4.26244554429779e-12 -100
"chr5" 173732001 173733000 "*" 2.66891642775047e-06 3.18759115320439e-06 57.1428571428571
"chr5" 173734001 173735000 "*" 1.34559030584569e-13 4.35581312463895e-13 100
"chr5" 173740001 173741000 "*" 2.88779000712225e-11 6.92935809466068e-11 100
"chr5" 173809001 173810000 "*" 0 0 -100
"chr5" 173811001 173812000 "*" 0 0 -94.3262411347518
"chr5" 173859001 173860000 "*" 8.45376425440136e-06 9.36283854341238e-06 67.5675675675676
"chr5" 173959001 173960000 "*" 3.34128893442198e-08 5.19772864618523e-08 100
"chr5" 174027001 174028000 "*" 6.92287338566189e-11 1.57605630124247e-10 100
"chr5" 174035001 174036000 "*" 1.10911280160053e-13 3.63089891158335e-13 -60
"chr5" 174137001 174138000 "*" 3.13097324733391e-08 4.89567196034953e-08 75
"chr5" 174140001 174141000 "*" 6.45251750253095e-07 8.41021546439509e-07 67.7966101694915
"chr5" 174147001 174148000 "*" 0 0 53.6552290938977
"chr5" 174249001 174250000 "*" 1.37828859436695e-10 3.01445544267661e-10 -100
"chr5" 174344001 174345000 "*" 9.2148511043888e-15 3.43705279328267e-14 66.6666666666667
"chr5" 174608001 174609000 "*" 0 0 100

"chr5" 174658001 174659000 "*" 1.35447209004269e-14 4.94674708073053e-14 100
"chr5" 174717001 174718000 "*" 3.04324343503026e-11 7.26599935673121e-11 -100
"chr5" 174778001 174779000 "*" 4.19331236400922e-13 1.27525223807619e-12 -96.25
"chr5" 174999001 175000000 "*" 1.37828859436695e-10 3.01445544267661e-10 100
"chr5" 175014001 175015000 "*" 0 0 -100
"chr5" 175024001 175025000 "*" 1.25284005392245e-10 2.75649590272477e-10 100
"chr5" 175086001 175087000 "*" 3.33066907387547e-15 1.30941888440006e-14 75
"chr5" 175091001 175092000 "*" 0 0 100
"chr5" 175103001 175104000 "*" 0 0 80.7692307692308
"chr5" 175106001 175107000 "*" 2.79987144580218e-12 7.68634726811898e-12 100
"chr5" 175111001 175112000 "*" 1.88245228693873e-06 2.29966065245188e-06
52.1739130434783
"chr5" 175113001 175114000 "*" 4.50885884184515e-10 9.19305634880493e-10
57.1428571428571
"chr5" 175121001 175122000 "*" 0 0 100
"chr5" 175137001 175138000 "*" 2.10755461717005e-05 2.19780359607569e-05
-52.1739130434783
"chr5" 175160001 175161000 "*" 1.01696429055664e-13 3.33932558863409e-13
61.8556701030928
"chr5" 175182001 175183000 "*" 4.73234496034536e-09 8.30627300264826e-09 100
"chr5" 175224001 175225000 "*" 0 0 58.6501343426837
"chr5" 175247001 175248000 "*" 0 0 77.2727272727273
"chr5" 175283001 175284000 "*" 5.05876518452908e-09 8.84866908967622e-09
-78.5714285714286
"chr5" 175294001 175295000 "*" 8.54554205176328e-11 1.92300513491153e-10
83.3333333333333
"chr5" 175320001 175321000 "*" 1.72586500646332e-10 3.72974565751066e-10
61.5384615384615
"chr5" 175321001 175322000 "*" 1.58761892521397e-14 5.75689238630984e-14
-60.4014598540146
"chr5" 175329001 175330000 "*" 1.39779633423487e-07 1.98463037958589e-07 100
"chr5" 175433001 175434000 "*" 0 0 -100
"chr5" 175467001 175468000 "*" 1.83186799063151e-14 6.59696304569048e-14 100
"chr5" 175473001 175474000 "*" 0 0 -100
"chr5" 175610001 175611000 "*" 4.25535940928867e-08 6.51108922874553e-08
-58.3333333333333
"chr5" 175624001 175625000 "*" 1.89425159169421e-05 1.98874471894124e-05 62.5
"chr5" 175850001 175851000 "*" 4.44089209850063e-15 1.71914916534614e-14 -100
"chr5" 175868001 175869000 "*" 2.67283972732457e-11 6.45686178157698e-11 100
"chr5" 175873001 175874000 "*" 1.11022302462516e-16 5.03662826618488e-16 100
"chr5" 175874001 175875000 "*" 0 0 88.8059701492537
"chr5" 175980001 175981000 "*" 0 0 -72.6520438683948
"chr5" 176000001 176001000 "*" 2.00910399428267e-11 4.91538834837561e-11
-72.7272727272727
"chr5" 176001001 176002000 "*" 3.84614562420893e-12 1.04062035914347e-11
66.6666666666667
"chr5" 176006001 176007000 "*" 0 0 100
"chr5" 176022001 176023000 "*" 3.80584452841504e-13 1.16686304767341e-12
59.1836734693878
"chr5" 176048001 176049000 "*" 5.87992721179376e-09 1.02105033599712e-08
62.962962962963
"chr5" 176049001 176050000 "*" 3.22190249247845e-05 3.26590558043081e-05

-54.5454545454545
"chr5" 176050001 176051000 "*" 0 0 100
"chr5" 176052001 176053000 "*" 3.7514393036453e-08 5.7741567259096e-08 62.5
"chr5" 176073001 176074000 "*" 1.46448074703764e-05 1.56461723225653e-05 64.8648648648649
"chr5" 176075001 176076000 "*" 1.3988810110277e-14 5.10290212559087e-14 -51.063829787234
"chr5" 176103001 176104000 "*" 1.0769163338864e-14 3.98631098593833e-14 -100
"chr5" 176109001 176110000 "*" 1.01587627199251e-11 2.59772463955703e-11 88.8888888888889
"chr5" 176124001 176125000 "*" 1.86517468137026e-14 6.70995420767425e-14 100
"chr5" 176125001 176126000 "*" 6.87620183015270e-10 1.36147387384037e-09 -84.1269841269841
"chr5" 176162001 176163000 "*" 8.7349629751543e-09 1.47505871898511e-08 100
"chr5" 176165001 176166000 "*" 1.49613654798486e-12 4.26244554429779e-12 100
"chr5" 176176001 176177000 "*" 1.80488957113312e-12 5.08664373959514e-12 -65.625
"chr5" 176202001 176203000 "*" 7.80486786311485e-14 2.60664512069585e-13 -58.8235294117647
"chr5" 176218001 176219000 "*" 4.73234496034536e-09 8.30627300264826e-09 100
"chr5" 176224001 176225000 "*" 0 0 69.2771084337349
"chr5" 176240001 176241000 "*" 4.01567668006919e-12 1.08266700497926e-11 61.6666666666667
"chr5" 176243001 176244000 "*" 1.44236241883533e-06 1.79195636844577e-06 -56.25
"chr5" 176245001 176246000 "*" 0 0 -52.7095516569201
"chr5" 176259001 176260000 "*" 8.67084182232247e-14 2.87708076903e-13 -98.4375
"chr5" 176276001 176277000 "*" 1.8189115658096e-08 2.93670613897249e-08 -62.962962962963
"chr5" 176284001 176285000 "*" 3.45168338355961e-13 1.06461834285352e-12 -63.6363636363636
"chr5" 176299001 176300000 "*" 7.0006535457523e-08 1.03695832512054e-07 100
"chr5" 176318001 176319000 "*" 6.42486923885244e-07 8.37601132445655e-07 66.6666666666667
"chr5" 176321001 176322000 "*" 7.0006535457523e-08 1.03695832512054e-07 100
"chr5" 176324001 176325000 "*" 1.13140401492018e-08 1.87801380787728e-08 -100
"chr5" 176428001 176429000 "*" 3.03090885722668e-14 1.06210046699299e-13 -100
"chr5" 176530001 176531000 "*" 1.67299207820548e-08 2.71377429441055e-08 -100
"chr5" 176531001 176532000 "*" 0 0 -74.3119266055046
"chr5" 176779001 176780000 "*" 2.91620934332881e-05 2.97633133955608e-05 -61.5384615384615
"chr5" 176790001 176791000 "*" 1.89737114908439e-13 6.04461882592279e-13 -90.9677419354839
"chr5" 176797001 176798000 "*" 2.00227675550835e-08 3.20459176617961e-08 -100
"chr5" 176805001 176806000 "*" 3.88022947106492e-13 1.1867462770515e-12 100
"chr5" 176815001 176816000 "*" 4.12718748066254e-11 9.67725582422511e-11 -53.8461538461538
"chr5" 176900001 176901000 "*" 0 0 67.3704414587332
"chr5" 176942001 176943000 "*" 1.9373391779709e-13 6.15971322898373e-13 -100
"chr5" 176996001 176997000 "*" 3.46500605985511e-13 1.06777978272295e-12 -100
"chr5" 177371001 177372000 "*" 0 0 -99.5098039215686
"chr5" 177387001 177388000 "*" 3.96387478396321e-10 8.13699713702712e-10 50.7142857142857

"chr5" 177396001 177397000 "*" 7.03992419914812e-12 1.83313429961219e-11 100
"chr5" 177409001 177410000 "*" 0 0 56.4516129032258
"chr5" 177423001 177424000 "*" 6.77335965093562e-12 1.76897011246462e-11 100
"chr5" 177521001 177522000 "*" 2.22044604925031e-16 9.81641919380259e-16 -85.1851851851852
"chr5" 177578001 177579000 "*" 9.47129314965434e-06 1.04214210309839e-05 63.0050505050505
"chr5" 177628001 177629000 "*" 4.78841410966879e-11 1.11468187156588e-10 -100
"chr5" 177629001 177630000 "*" 6.92287338566189e-11 1.57605630124247e-10 -100
"chr5" 177654001 177655000 "*" 1.98365768255826e-11 4.85612402473442e-11 100
"chr5" 177680001 177681000 "*" 3.68971520003925e-12 1.00056842563147e-11 -56.25
"chr5" 177681001 177682000 "*" 1.56157224606712e-06 1.93054397956841e-06 78.125
"chr5" 177691001 177692000 "*" 3.7274627828765e-12 1.00985250136599e-11 100
"chr5" 177695001 177696000 "*" 1.6219141030227e-08 2.64300079144354e-08 66.6666666666667
"chr5" 177715001 177716000 "*" 0 0 76.9047619047619
"chr5" 177716001 177717000 "*" 0 0 65.5172413793103
"chr5" 177750001 177751000 "*" 1.67299207820548e-08 2.71377429441055e-08 100
"chr5" 177758001 177759000 "*" 2.68648345902456e-07 3.68335157375939e-07 -75.8064516129032
"chr5" 177777001 177778000 "*" 0 0 52.9588525919917
"chr5" 177808001 177809000 "*" 0 0 100
"chr5" 177816001 177817000 "*" 0 0 100
"chr5" 177821001 177822000 "*" 8.53124237920611e-11 1.91999449631174e-10 58.8235294117647
"chr5" 177826001 177827000 "*" 2.50979936833318e-09 4.58992668566555e-09 83.0985915492958
"chr5" 177846001 177847000 "*" 6.98726632109015e-10 1.38244699331437e-09 68.4210526315789
"chr5" 177850001 177851000 "*" 2.18713935851156e-14 7.80893646430581e-14 -72.2222222222222
"chr5" 177857001 177858000 "*" 1.5373219007242e-05 1.63757232722891e-05 57.4468085106383
"chr5" 177858001 177859000 "*" 0 0 100
"chr5" 177866001 177867000 "*" 5.63660229602192e-13 1.68905058935478e-12 100
"chr5" 177951001 177952000 "*" 0 0 66.6666666666667
"chr5" 178009001 178010000 "*" 0.000390793259243072 0.000332358767661237 52.7777777777778
"chr5" 178069001 178070000 "*" 8.71525074330748e-14 2.88848349331391e-13 -100
"chr5" 178077001 178078000 "*" 8.7349629751543e-09 1.47505871898511e-08 -100
"chr5" 178094001 178095000 "*" 0 0 100
"chr5" 178096001 178097000 "*" 9.65729496371637e-10 1.87255912493232e-09 100
"chr5" 178127001 178128000 "*" 0 0 70.2702702702703
"chr5" 178128001 178129000 "*" 1.11022302462516e-16 5.03662826618488e-16 -100
"chr5" 178197001 178198000 "*" 0 0 -61.3108339447298
"chr5" 178205001 178206000 "*" 0 0 -100
"chr5" 178245001 178246000 "*" 3.45545814184334e-12 9.39854604504933e-12 65.625
"chr5" 178256001 178257000 "*" 1.11022302462516e-16 5.03662826618488e-16 100
"chr5" 178260001 178261000 "*" 0 0 100
"chr5" 178323001 178324000 "*" 0 0 -53.8485528942116
"chr5" 178351001 178352000 "*" 6.4152538836737e-10 1.27477697728417e-09 -100
"chr5" 178386001 178387000 "*" 9.52327106062967e-12 2.44451523156523e-11

-85.0267379679144
"chr5" 178397001 178398000 "*" 6.15840711759574e-13 1.83676803572802e-12 100
"chr5" 178412001 178413000 "*" 2.12496686913255e-13 6.7178544377836e-13 75
"chr5" 178423001 178424000 "*" 6.66133814775094e-16 2.81595744474255e-15 -100
"chr5" 178424001 178425000 "*" 0 0 58.0786026200873
"chr5" 178482001 178483000 "*" 3.40125705378114e-10 7.03784073980162e-10 -100
"chr5" 178558001 178559000 "*" 4.89762674860117e-11 1.13829418217247e-10
55.8823529411765
"chr5" 178592001 178593000 "*" 1.11022302462516e-14 4.10577217300208e-14
83.1325301204819
"chr5" 178625001 178626000 "*" 9.99960114711484e-11 2.23017436846733e-10 81.25
"chr5" 178639001 178640000 "*" 0 0 66.6043882341669
"chr5" 178657001 178658000 "*" 0.000361614415223421 0.000309112746898742
51.7241379310345
"chr5" 178688001 178689000 "*" 0 0 100
"chr5" 178695001 178696000 "*" 0 0 93.9393939393939
"chr5" 178698001 178699000 "*" 5.36237720893951e-14 1.82370018611088e-13
58.5714285714286
"chr5" 178713001 178714000 "*" 5.96531749463836e-06 6.76187393577205e-06
63.1578947368421
"chr5" 178714001 178715000 "*" 2.81186185446813e-12 7.71773969739836e-12
51.3513513513514
"chr5" 178716001 178717000 "*" 4.44089209850063e-16 1.91071758245033e-15
71.3794926004228
"chr5" 178722001 178723000 "*" 4.37928145968769e-08 6.69259754758625e-08
58.5365853658537
"chr5" 178724001 178725000 "*" 1.4432899320127e-15 5.90750815956055e-15
-67.5324675324675
"chr5" 178739001 178740000 "*" 1.39566486312415e-06 1.73784950985638e-06
-71.8181818181818
"chr5" 178742001 178743000 "*" 6.5377903091246e-10 1.29788928714556e-09
52.1739130434783
"chr5" 178745001 178746000 "*" 9.36259381134619e-09 1.57533610932766e-08
57.6271186440678
"chr5" 178746001 178747000 "*" 1.43218770176645e-14 5.21983361092297e-14 100
"chr5" 178783001 178784000 "*" 6.12430772761741e-10 1.22528336979697e-09
-63.1578947368421
"chr5" 178788001 178789000 "*" 0 0 100
"chr5" 178795001 178796000 "*" 5.35982369598287e-12 1.42057721384202e-11
-54.8554336989033
"chr5" 178832001 178833000 "*" 3.43058914609173e-14 1.19587680585466e-13
98.5714285714286
"chr5" 178863001 178864000 "*" 1.58777990755254e-10 3.44221343707728e-10
78.5714285714286
"chr5" 178865001 178866000 "*" 1.23355115011492e-09 2.35616886119023e-09
50.8613445378151
"chr5" 178866001 178867000 "*" 1.89140223483264e-07 2.64383247523815e-07
51.778329197684
"chr5" 178868001 178869000 "*" 3.41105608795411e-05 3.44037314231588e-05
-51.6129032258064
"chr5" 178878001 178879000 "*" 2.33146835171283e-15 9.32818831833246e-15
89.051094890511

"chr5" 178890001 178891000 "*" 2.58132164199232e-07 3.54639731715552e-07 66.6666666666667
"chr5" 178921001 178922000 "*" 1.43218770176645e-14 5.21983361092297e-14 96.2962962962963
"chr5" 178923001 178924000 "*" 2.71303979282322e-10 5.70235407328575e-10 -72.7272727272727
"chr5" 178926001 178927000 "*" 0 0 -78.3333333333333
"chr5" 179075001 179076000 "*" 0 0 100
"chr5" 179105001 179106000 "*" 0 0 51.7435487151163
"chr5" 179246001 179247000 "*" 0 0 -100
"chr5" 179286001 179287000 "*" 0 0 -63.9344262295082
"chr5" 179314001 179315000 "*" 0 0 100
"chr5" 179328001 179329000 "*" 3.5527136788005e-15 1.39277426410519e-14 -100
"chr5" 179392001 179393000 "*" 1.11022302462516e-16 5.03662826618488e-16 -100
"chr5" 179393001 179394000 "*" 2.46227482847416e-10 5.20427372274224e-10 -51.3513513513514
"chr5" 179505001 179506000 "*" 0 0 70
"chr5" 179546001 179547000 "*" 0 0 57.6082897684839
"chr5" 179549001 179550000 "*" 4.02167188440217e-12 1.08266700497926e-11 100
"chr5" 179560001 179561000 "*" 3.47943895917524e-13 1.07191058753964e-12 83.8709677419355
"chr5" 179566001 179567000 "*" 2.22044604925031e-16 9.81641919380259e-16 67.1641791044776
"chr5" 179567001 179568000 "*" 6.45987877290199e-06 7.28081397894206e-06 -68.4210526315789
"chr5" 179571001 179572000 "*" 8.3882012447134e-11 1.88922989503423e-10 100
"chr5" 179583001 179584000 "*" 2.67753064497889e-07 3.67210293236612e-07 56.25
"chr5" 179602001 179603000 "*" 1.11022302462516e-16 5.03662826618488e-16 60.0340136054422
"chr5" 179603001 179604000 "*" 3.03090885722668e-14 1.06210046699299e-13 -100
"chr5" 179606001 179607000 "*" 0 0 57.1078431372549
"chr5" 179608001 179609000 "*" 6.36834972644884e-07 8.30830174625137e-07 61.5384615384615
"chr5" 179619001 179620000 "*" 3.40125705378114e-10 7.03784073980162e-10 -100
"chr5" 179625001 179626000 "*" 3.56775498033812e-10 7.35898629809829e-10 -100
"chr5" 179626001 179627000 "*" 0 0 100
"chr5" 179638001 179639000 "*" 0 0 84.375
"chr5" 179640001 179641000 "*" 5.295763827462e-14 1.80214784640623e-13 100
"chr5" 179706001 179707000 "*" 7.7715611723761e-16 3.26213507634405e-15 100
"chr5" 179718001 179719000 "*" 0 0 100
"chr5" 179731001 179732000 "*" 6.10963346581173e-09 1.0587123964306e-08 64.8648648648649
"chr5" 179777001 179778000 "*" 0 0 78.5714285714286
"chr5" 179786001 179787000 "*" 0.000285593505977677 0.000248445226337853 -52.1739130434783
"chr5" 179801001 179802000 "*" 1.48087875295744e-10 3.22075573380618e-10 100
"chr5" 179803001 179804000 "*" 2.49111842265393e-12 6.89480014214944e-12 65.625
"chr5" 179810001 179811000 "*" 3.90831811358794e-12 1.05610567215048e-11 100
"chr5" 179863001 179864000 "*" 0 0 -70.2272727272727
"chr5" 180060001 180061000 "*" 4.9737991503207e-14 1.70042351188371e-13 81.4814814814815
"chr5" 180064001 180065000 "*" 6.28097573951436e-12 1.65149517426544e-11 96

"chr5" 180096001 180097000 "*" 1.52845815415414e-06 1.89221106972853e-06
71.5596330275229
"chr5" 180109001 180110000 "*" 2.22044604925031e-16 9.81641919380259e-16
85.2173913043478
"chr5" 180166001 180167000 "*" 1.1540792765885e-08 1.91395764532531e-08
-57.8947368421053
"chr5" 180171001 180172000 "*" 5.98831817466561e-09 1.03874786659479e-08
85.5670103092783
"chr5" 180211001 180212000 "*" 1.12622295678211e-06 1.41959920389863e-06 -68.75
"chr5" 180238001 180239000 "*" 5.98645105867046e-05 5.81619942065767e-05
-50.8714596949891
"chr5" 180294001 180295000 "*" 4.0258796296655e-11 9.47220264141414e-11 60
"chr5" 180466001 180467000 "*" 4.02167188440217e-12 1.08266700497926e-11 100
"chr5" 180472001 180473000 "*" 3.6913450492837e-05 3.70385531425908e-05
58.974358974359
"chr5" 180478001 180479000 "*" 3.01536573488193e-12 8.25050372060282e-12
51.1627906976744
"chr5" 180499001 180500000 "*" 1.67299207820548e-08 2.71377429441055e-08 100
"chr5" 180535001 180536000 "*" 6.15840711759574e-13 1.83676803572802e-12 100
"chr5" 180557001 180558000 "*" 5.01025332333427e-10 1.01358031135889e-09 100
"chr5" 180558001 180559000 "*" 2.40341176960257e-08 3.80823058212267e-08
-61.9047619047619
"chr5" 180618001 180619000 "*" 0 0 82.2208270166251
"chr5" 180648001 180649000 "*" 0 0 75.8241758241758
"chr5" 180651001 180652000 "*" 0 0 -52.5251648733079
"chr5" 180672001 180673000 "*" 3.79682288986771e-06 4.42902645302486e-06
53.6764705882353
"chr5" 180707001 180708000 "*" 6.92287338566189e-11 1.57605630124247e-10 100
"chr6" 225001 226000 "*" 0 0 65.3778080326753
"chr6" 281001 282000 "*" 4.84749018703212e-10 9.83683447656256e-10
-71.8023255813954
"chr6" 299001 300000 "*" 1.71659664260915e-09 3.21705443372083e-09
54.9689440993789
"chr6" 347001 348000 "*" 1.72972747236599e-13 5.53740506536757e-13
-90.3703703703704
"chr6" 354001 355000 "*" 0 0 -55.3634158581465
"chr6" 371001 372000 "*" 3.42259554031443e-12 9.31219957288356e-12
-92.8571428571429
"chr6" 381001 382000 "*" 0 0 -51.3062409288824
"chr6" 383001 384000 "*" 1.2623235789988e-13 4.09851934012467e-13
74.7368421052632
"chr6" 398001 399000 "*" 7.0006535457523e-08 1.03695832512054e-07 -100
"chr6" 404001 405000 "*" 5.12901088933759e-07 6.76904585220232e-07 -68.75
"chr6" 436001 437000 "*" 1.01933322893544e-08 1.70753613870261e-08
59.1715976331361
"chr6" 442001 443000 "*" 1.67299207820548e-08 2.71377429441055e-08 100
"chr6" 446001 447000 "*" 0 0 57.2916666666667
"chr6" 452001 453000 "*" 4.03084694711175e-06 4.68347907838358e-06 70.3125
"chr6" 558001 559000 "*" 0 0 100
"chr6" 783001 784000 "*" 0 0 55.8618177402708
"chr6" 805001 806000 "*" 8.69533023362123e-09 1.47358747051732e-08
-71.6417910447761

"chr6" 846001 847000 "*" 1.08860161829316e-06 1.37510812430546e-06 66.6666666666667
"chr6" 874001 875000 "*" 2.71893618730701e-13 8.46692454027087e-13 100
"chr6" 882001 883000 "*" 4.36314785523528e-05 4.33072980789599e-05 57.2222222222222
"chr6" 909001 910000 "*" 4.96800378613216e-11 1.15403999195475e-10 86.2068965517241
"chr6" 935001 936000 "*" 6.52733422867868e-12 1.71146951718423e-11 -100
"chr6" 962001 963000 "*" 1.0131347689013e-05 1.10909163221897e-05 62.5
"chr6" 995001 996000 "*" 0 0 -100
"chr6" 1028001 1029000 "*" 1.98365768255826e-11 4.85612402473442e-11 -100
"chr6" 1078001 1079000 "*" 1.61792801378624e-12 4.5807897612306e-12 -60
"chr6" 1205001 1206000 "*" 6.37490060739765e-13 1.89496793587877e-12 100
"chr6" 1263001 1264000 "*" 5.39619190149665e-05 5.27870047241069e-05 59.2592592592593
"chr6" 1282001 1283000 "*" 2.4535928844216e-14 8.69144396197119e-14 -100
"chr6" 1308001 1309000 "*" 1.39779633423487e-07 1.98463037958589e-07 100
"chr6" 1311001 1312000 "*" 1.45252976313515e-09 2.75203876995615e-09 77.0440251572327
"chr6" 1312001 1313000 "*" 0 0 62.3152709359606
"chr6" 1381001 1382000 "*" 0 0 72.8967591045773
"chr6" 1406001 1407000 "*" 4.99900121297969e-12 1.32921056339624e-11 100
"chr6" 1445001 1446000 "*" 8.7349629751543e-09 1.47505871898511e-08 100
"chr6" 1473001 1474000 "*" 2.02327044007689e-12 5.65300384792621e-12 -100
"chr6" 1507001 1508000 "*" 2.71893618730701e-13 8.46692454027087e-13 -100
"chr6" 1524001 1525000 "*" 0 0 54.0850625964672
"chr6" 1577001 1578000 "*" 0 0 96.969696969697
"chr6" 1611001 1612000 "*" 0 0 50.6134969325153
"chr6" 1641001 1642000 "*" 0.000120483972759877 0.000111549020346707 -52.1739130434783
"chr6" 1737001 1738000 "*" 6.59550958292954e-09 1.13456194382773e-08 -100
"chr6" 1941001 1942000 "*" 2.60361667936415e-06 3.11419458317291e-06 80
"chr6" 2123001 2124000 "*" 9.99200722162641e-16 4.15382497462808e-15 100
"chr6" 2444001 2445000 "*" 4.78841410966879e-11 1.11468187156588e-10 100
"chr6" 2523001 2524000 "*" 7.7715611723761e-16 3.26213507634405e-15 100
"chr6" 2546001 2547000 "*" 4.48406157760317e-08 6.84482503893299e-08 66.6666666666667
"chr6" 2634001 2635000 "*" 0 0 64.8326154562538
"chr6" 2786001 2787000 "*" 5.40022471184898e-11 1.24863461258992e-10 -83.108935128519
"chr6" 2810001 2811000 "*" 9.04570973681018e-10 1.76414389822173e-09 -100
"chr6" 2841001 2842000 "*" 0 0 -95.9016393442623
"chr6" 2962001 2963000 "*" 0.000334851753864718 0.000287983064674082 55.5555555555556
"chr6" 3015001 3016000 "*" 3.26184634857896e-11 7.76392759152788e-11 68.2926829268293
"chr6" 3157001 3158000 "*" 4.22879287143019e-10 8.65440402477824e-10 -53.4883720930233
"chr6" 3218001 3219000 "*" 8.65973959207622e-14 2.87362558212404e-13 -100
"chr6" 3227001 3228000 "*" 0 0 79.0144962192739
"chr6" 3228001 3229000 "*" 0 0 78.9898605720608
"chr6" 3231001 3232000 "*" 0 0 69.683257918552

"chr6" 3232001 3233000 "*" 0 0 100
"chr6" 3247001 3248000 "*" 2.41022546365599e-09 4.42480537812895e-09
58.3333333333333
"chr6" 3248001 3249000 "*" 0 0 84
"chr6" 3258001 3259000 "*" 6.9792616130826e-11 1.58832996661374e-10
86.8421052631579
"chr6" 3336001 3337000 "*" 5.794809077031e-12 1.52911192757929e-11 -100
"chr6" 3349001 3350000 "*" 1.77806292118721e-05 1.8763499200702e-05
-52.1739130434783
"chr6" 3396001 3397000 "*" 2.33028818463765e-10 4.93719283454501e-10 100
"chr6" 3493001 3494000 "*" 4.73234496034536e-09 8.30627300264826e-09 -100
"chr6" 3517001 3518000 "*" 2.83798429201454e-09 5.14749425698542e-09 -93.75
"chr6" 3518001 3519000 "*" 0 0 -100
"chr6" 3548001 3549000 "*" 7.44124761808962e-10 1.467013137464e-09
-73.3333333333333
"chr6" 3575001 3576000 "*" 8.01666433236647e-09 1.36392368970109e-08 100
"chr6" 3603001 3604000 "*" 1.12458486967171e-09 2.15740815043925e-09 -100
"chr6" 3607001 3608000 "*" 2.22044604925031e-16 9.81641919380259e-16 -100
"chr6" 3749001 3750000 "*" 0 0 -74.025974025974
"chr6" 3751001 3752000 "*" 0 0 77.1143214975604
"chr6" 3752001 3753000 "*" 0 0 57.25208210076
"chr6" 3757001 3758000 "*" 1.54543045027822e-13 4.96800711807723e-13
51.219512195122
"chr6" 3774001 3775000 "*" 2.33028818463765e-10 4.93719283454501e-10 100
"chr6" 3777001 3778000 "*" 8.01666433236647e-09 1.36392368970109e-08 -100
"chr6" 3840001 3841000 "*" 8.65973959207622e-15 3.24130830964561e-14
-91.9254658385093
"chr6" 3870001 3871000 "*" 0 0 -100
"chr6" 3872001 3873000 "*" 0 0 72.7272727272727
"chr6" 3888001 3889000 "*" 2.64951482975562e-09 4.82186011375752e-09 -100
"chr6" 3933001 3934000 "*" 2.33844237211844e-05 2.42186234361163e-05
-63.1578947368421
"chr6" 4088001 4089000 "*" 0 0 94.3396226415094
"chr6" 4135001 4136000 "*" 5.58071477918531e-10 1.12161263408564e-09 -80
"chr6" 4165001 4166000 "*" 3.68371999570627e-13 1.13149976091305e-12 -100
"chr6" 4254001 4255000 "*" 3.95353005888666e-09 7.03928529283733e-09 100
"chr6" 4281001 4282000 "*" 1.11022302462516e-16 5.03662826618488e-16
-60.9351432880845
"chr6" 4341001 4342000 "*" 2.17381668221606e-13 6.85151076607736e-13 -100
"chr6" 4376001 4377000 "*" 1.5277158427196e-09 2.87642564232045e-09 100
"chr6" 4471001 4472000 "*" 1.29052324382428e-12 3.70730704018115e-12
-77.5510204081633
"chr6" 4481001 4482000 "*" 3.08051012253285e-05 3.13190817292576e-05
-61.9718309859155
"chr6" 4511001 4512000 "*" 1.38280720207717e-10 3.02378302118387e-10
-64.8648648648649
"chr6" 4521001 4522000 "*" 9.71063229826541e-11 2.16781365050398e-10 100
"chr6" 4613001 4614000 "*" 2.08995487582797e-10 4.46944006972663e-10 100
"chr6" 4615001 4616000 "*" 1.36604061395929e-11 3.42333952202875e-11 100
"chr6" 4713001 4714000 "*" 9.41163845049608e-05 8.8633379222919e-05
51.8518518518519
"chr6" 4716001 4717000 "*" 9.38521149684846e-09 1.57866400973037e-08

69.8412698412698
"chr6" 4775001 4776000 "*" 0 0 52.5453624505602
"chr6" 4776001 4777000 "*" 0 0 74.6606334841629
"chr6" 4958001 4959000 "*" 5.24469356832924e-13 1.57813291117778e-12
-82.6086956521739
"chr6" 4964001 4965000 "*" 1.11022302462516e-16 5.03662826618488e-16 100
"chr6" 4983001 4984000 "*" 5.12901088933759e-07 6.76904585220232e-07 68.75
"chr6" 5058001 5059000 "*" 0 0 -100
"chr6" 5068001 5069000 "*" 4.98801967507845e-08 7.56660392877468e-08
-51.7188693659282
"chr6" 5125001 5126000 "*" 0 0 -100
"chr6" 5129001 5130000 "*" 0 0 -60.5263157894737
"chr6" 5144001 5145000 "*" 1.88292021974235e-07 2.63281458379181e-07
54.1666666666667
"chr6" 5146001 5147000 "*" 3.46090843228453e-06 4.06200733549184e-06
52.3809523809524
"chr6" 5203001 5204000 "*" 2.22044604925031e-16 9.81641919380259e-16 100
"chr6" 5303001 5304000 "*" 2.22044604925031e-16 9.81641919380259e-16 100
"chr6" 5660001 5661000 "*" 1.98325800226939e-11 4.85612402473442e-11 -100
"chr6" 5729001 5730000 "*" 0 0 -75.609756097561
"chr6" 5735001 5736000 "*" 1.99840144432528e-12 5.59476580335607e-12 100
"chr6" 5764001 5765000 "*" 1.4255263636187e-13 4.59957800504786e-13
-64.7058823529412
"chr6" 5776001 5777000 "*" 0.000216683727490286 0.000192426836197816 -70
"chr6" 6007001 6008000 "*" 0 0 63.95369795526
"chr6" 6008001 6009000 "*" 0 0 100
"chr6" 6009001 6010000 "*" 0 0 66.6666666666667
"chr6" 6158001 6159000 "*" 6.30384633382164e-13 1.87665599796749e-12 100
"chr6" 6271001 6272000 "*" 2.62900812231237e-13 8.21294938531841e-13
-66.6666666666667
"chr6" 6517001 6518000 "*" 0 0 100
"chr6" 6655001 6656000 "*" 1.86675099822509e-08 3.01004068443148e-08
-54.8780487804878
"chr6" 6664001 6665000 "*" 5.51780843238703e-14 1.87243792572872e-13 100
"chr6" 6679001 6680000 "*" 0 0 -71.2328767123288
"chr6" 6791001 6792000 "*" 6.59550958292954e-09 1.13456194382773e-08 -100
"chr6" 6802001 6803000 "*" 0 0 96.875
"chr6" 6825001 6826000 "*" 7.25646932875357e-07 9.39130690477135e-07 -75
"chr6" 6909001 6910000 "*" 9.47020240005259e-14 3.12491189162604e-13
68.8311688311688
"chr6" 7042001 7043000 "*" 3.88022947106492e-13 1.1867462770515e-12 -100
"chr6" 7044001 7045000 "*" 0 0 -90.6542056074766
"chr6" 7107001 7108000 "*" 0 0 52.6108543057696
"chr6" 7232001 7233000 "*" 6.13937179827495e-06 6.94260980394374e-06
-66.6666666666667
"chr6" 7428001 7429000 "*" 9.80763359414993e-10 1.8980032424715e-09 -100
"chr6" 7433001 7434000 "*" 0 0 -58.7622903412377
"chr6" 7446001 7447000 "*" 4.9960036108132e-15 1.9234186548721e-14
-60.6060606060606
"chr6" 7500001 7501000 "*" 0 0 -56.0975609756098
"chr6" 7617001 7618000 "*" 4.99900121297969e-12 1.32921056339624e-11 100
"chr6" 7698001 7699000 "*" 3.6700841921089e-08 5.65530226072258e-08 -100

"chr6" 7704001 7705000 "*" 4.60260718426753e-11 1.07436717245276e-10
79.6116504854369
"chr6" 7726001 7727000 "*" 0 0 90.5903398926655
"chr6" 7727001 7728000 "*" 0 0 60.3399493466996
"chr6" 7728001 7729000 "*" 0 0 80.5793316891532
"chr6" 7735001 7736000 "*" 0 0 -90.6976744186046
"chr6" 7759001 7760000 "*" 5.8616765863384e-07 7.68442756313584e-07
91.6666666666667
"chr6" 7798001 7799000 "*" 3.6700841921089e-08 5.65530226072258e-08 -100
"chr6" 7829001 7830000 "*" 1.59227548923724e-08 2.59658003984163e-08
61.5384615384615
"chr6" 7917001 7918000 "*" 5.33495114529714e-06 6.09237962218305e-06
-66.6666666666667
"chr6" 7972001 7973000 "*" 4.86753970463383e-11 1.13145908161915e-10 100
"chr6" 8017001 8018000 "*" 6.04438721296674e-11 1.38849255306602e-10 100
"chr6" 8122001 8123000 "*" 0 0 -93.3333333333333
"chr6" 8244001 8245000 "*" 1.13140401492018e-08 1.87801380787728e-08 -100
"chr6" 9042001 9043000 "*" 1.11022302462516e-16 5.03662826618488e-16 100
"chr6" 9297001 9298000 "*" 4.73234496034536e-09 8.30627300264826e-09 100
"chr6" 9335001 9336000 "*" 0 0 -100
"chr6" 9882001 9883000 "*" 0 0 100
"chr6" 9898001 9899000 "*" 1.66943792123675e-10 3.61266959467532e-10
-69.0476190476191
"chr6" 9997001 9998000 "*" 1.16573417585641e-14 4.29335582900508e-14 100
"chr6" 10238001 10239000 "*" 9.63829016598083e-12 2.46993429847588e-11 -100
"chr6" 10311001 10312000 "*" 1.26565424807268e-14 4.63923203146481e-14 100
"chr6" 10316001 10317000 "*" 1.11022302462516e-16 5.03662826618488e-16
-66.6666666666667
"chr6" 10322001 10323000 "*" 1.66533453693773e-15 6.7629186866784e-15 -100
"chr6" 10337001 10338000 "*" 0 0 57.0512820512821
"chr6" 10405001 10406000 "*" 1.04916075827077e-13 3.44152363709097e-13
-73.1707317073171
"chr6" 10414001 10415000 "*" 0 0 97.0588235294118
"chr6" 10615001 10616000 "*" 4.71134242729931e-12 1.257615036806e-11 100
"chr6" 10661001 10662000 "*" 6.06224518463483e-05 5.88490203691753e-05
54.8387096774194
"chr6" 10862001 10863000 "*" 0 0 -100
"chr6" 11097001 11098000 "*" 3.69896122176527e-07 4.97384915956605e-07
-76.5957446808511
"chr6" 11216001 11217000 "*" 6.59550958292954e-09 1.13456194382773e-08 -100
"chr6" 11280001 11281000 "*" 7.7715611723761e-16 3.26213507634405e-15 100
"chr6" 11419001 11420000 "*" 8.65973959207622e-15 3.24130830964561e-14 -100
"chr6" 11483001 11484000 "*" 1.1099341179488e-07 1.6006307649158e-07
-90.4761904761905
"chr6" 11537001 11538000 "*" 0 0 64.8648648648649
"chr6" 11538001 11539000 "*" 0 0 80
"chr6" 11647001 11648000 "*" 0 0 100
"chr6" 11776001 11777000 "*" 1.98365768255826e-11 4.85612402473442e-11 100
"chr6" 11869001 11870000 "*" 0 0 100
"chr6" 11959001 11960000 "*" 1.37001521238744e-13 4.42851918810123e-13 100
"chr6" 12003001 12004000 "*" 9.1572733840195e-09 1.54278121036964e-08
-60.2822580645161

"chr6" 12256001 12257000 "*" 1.67299207820548e-08 2.71377429441055e-08 100
"chr6" 12569001 12570000 "*" 7.0006535457523e-08 1.03695832512054e-07 100
"chr6" 13013001 13014000 "*" 0 0 73.0402645399728
"chr6" 13015001 13016000 "*" 0 0 68.75
"chr6" 13134001 13135000 "*" 8.77076189453874e-15 3.27889038038365e-14 100
"chr6" 13251001 13252000 "*" 4.72528449702025e-09 8.30627300264826e-09
91.6666666666667
"chr6" 13300001 13301000 "*" 1.89581683684992e-12 5.32311367606535e-12 100
"chr6" 13614001 13615000 "*" 1.4432899320127e-15 5.90750815956055e-15 -100
"chr6" 13925001 13926000 "*" 0 0 70.6748105084995
"chr6" 13977001 13978000 "*" 1.52794177310511e-09 2.87642564232045e-09 -100
"chr6" 14003001 14004000 "*" 3.02987580799163e-05 3.08411348302445e-05
-57.1428571428571
"chr6" 14032001 14033000 "*" 3.40125705378114e-10 7.03784073980162e-10 100
"chr6" 14140001 14141000 "*" 0 0 58.3333333333333
"chr6" 14174001 14175000 "*" 4.14335232790108e-13 1.26153063645578e-12 -100
"chr6" 14210001 14211000 "*" 6.49201060687332e-08 9.69989088568228e-08
77.7777777777778
"chr6" 14247001 14248000 "*" 8.7349629751543e-09 1.47505871898511e-08 100
"chr6" 14252001 14253000 "*" 3.6700841921089e-08 5.65530226072258e-08 100
"chr6" 14275001 14276000 "*" 4.77395900588817e-15 1.84201484337652e-14
-72.2222222222222
"chr6" 14300001 14301000 "*" 1.4432899320127e-15 5.90750815956055e-15 -100
"chr6" 14317001 14318000 "*" 1.52794177310511e-09 2.87642564232045e-09 100
"chr6" 14473001 14474000 "*" 3.07473755245535e-06 3.63861669378059e-06 -62.5
"chr6" 14487001 14488000 "*" 1.51914614832105e-06 1.88121349342686e-06
-52.3809523809524
"chr6" 14506001 14507000 "*" 3.39440209273789e-09 6.10422192745839e-09
61.5384615384615
"chr6" 14755001 14756000 "*" 1.11022302462516e-15 4.59817122935606e-15
62.4458874458875
"chr6" 14801001 14802000 "*" 2.69018141096922e-12 7.41487347763769e-12 -100
"chr6" 14810001 14811000 "*" 1.20591092667155e-10 2.66075324129686e-10 100
"chr6" 14836001 14837000 "*" 6.24654217240561e-10 1.2462108191303e-09 100
"chr6" 14891001 14892000 "*" 5.295763827462e-14 1.80214784640623e-13 100
"chr6" 14904001 14905000 "*" 9.63829016598083e-12 2.46993429847588e-11 -100
"chr6" 14963001 14964000 "*" 5.6362137179633e-11 1.29963270300065e-10 100
"chr6" 15021001 15022000 "*" 2.73014943985572e-11 6.58595916221891e-11 100
"chr6" 15192001 15193000 "*" 2.22044604925031e-16 9.81641919380259e-16 100
"chr6" 15215001 15216000 "*" 2.79440914852103e-11 6.73046092948135e-11 -100
"chr6" 15383001 15384000 "*" 5.82080730282541e-08 8.75553486242363e-08 -90
"chr6" 15700001 15701000 "*" 1.7880684276772e-05 1.88607324291789e-05
64.2857142857143
"chr6" 15875001 15876000 "*" 4.71134242729931e-12 1.257615036806e-11 -100
"chr6" 16239001 16240000 "*" 2.01561515589255e-07 2.80767138483327e-07
-56.0606060606061
"chr6" 16346001 16347000 "*" 2.02060590481778e-13 6.40127405465654e-13 -100
"chr6" 16356001 16357000 "*" 1.13363522569188e-07 1.63277727194719e-07
-81.5789473684211
"chr6" 16391001 16392000 "*" 4.99020824662466e-11 1.15790792586157e-10 100
"chr6" 16733001 16734000 "*" 1.24846286975178e-07 1.78815836572989e-07 -93.75
"chr6" 16762001 16763000 "*" 0 0 -61.5384615384615

"chr6" 16850001 16851000 "*" 9.04570973681018e-10 1.76414389822173e-09 100
"chr6" 16961001 16962000 "*" 2.15125472990962e-10 4.58126659943874e-10 100
"chr6" 17211001 17212000 "*" 2.11424519702685e-06 2.56432546771576e-06
54.5454545454545
"chr6" 17322001 17323000 "*" 3.6700841921089e-08 5.65530226072258e-08 100
"chr6" 17384001 17385000 "*" 0 0 -100
"chr6" 17409001 17410000 "*" 0.000672965876117981 0.000549698110587813 52
"chr6" 17454001 17455000 "*" 0 0 100
"chr6" 17974001 17975000 "*" 6.24654217240561e-10 1.2462108191303e-09 -100
"chr6" 17986001 17987000 "*" 6.4152538836737e-10 1.27477697728417e-09
66.6666666666667
"chr6" 17987001 17988000 "*" 0 0 -79.4117647058823
"chr6" 18065001 18066000 "*" 1.98325800226939e-11 4.85612402473442e-11 -100
"chr6" 18277001 18278000 "*" 0 0 87.0828230251072
"chr6" 19805001 19806000 "*" 0 0 65.5172413793103
"chr6" 19839001 19840000 "*" 0 0 74.8005571736102
"chr6" 19845001 19846000 "*" 1.29780288227188e-06 1.62254962560876e-06
58.6206896551724
"chr6" 20245001 20246000 "*" 9.97938786898578e-05 9.35733209966104e-05
54.5977011494253
"chr6" 20340001 20341000 "*" 1.59301727618022e-05 1.69294262236803e-05
52.3809523809524
"chr6" 20568001 20569000 "*" 1.83952852950142e-12 5.17499701329218e-12
-66.6666666666667
"chr6" 21309001 21310000 "*" 2.80791846737349e-05 2.87238108173818e-05
-53.1468531468531
"chr6" 21377001 21378000 "*" 9.72102042732237e-05 9.13221607712885e-05
58.3333333333333
"chr6" 21458001 21459000 "*" 1.02437835991509e-10 2.28170470255037e-10
-66.6666666666667
"chr6" 21587001 21588000 "*" 0 0 -85.7142857142857
"chr6" 21588001 21589000 "*" 0 0 -100
"chr6" 21596001 21597000 "*" 0 0 -50.4252928903868
"chr6" 21696001 21697000 "*" 1.87405646556726e-13 5.97285292681429e-13 100
"chr6" 22286001 22287000 "*" 2.23653187492179e-06 2.70311306704149e-06
-66.6666666666667
"chr6" 22357001 22358000 "*" 0 0 -56.6037735849057
"chr6" 24126001 24127000 "*" 0 0 56.8669582789758
"chr6" 24221001 24222000 "*" 1.91145432815176e-10 4.11344660543415e-10 -60
"chr6" 24271001 24272000 "*" 2.08814465718632e-09 3.86232567960463e-09 -100
"chr6" 24358001 24359000 "*" 0 0 95.1492537313433
"chr6" 24374001 24375000 "*" 1.1604364773099e-05 1.25899461299632e-05
-60.8695652173913
"chr6" 24494001 24495000 "*" 3.23013726877264e-10 6.71106819599e-10
-50.1214329083182
"chr6" 24564001 24565000 "*" 0 0 -66.6666666666667
"chr6" 24911001 24912000 "*" 0 0 95.2025586353945
"chr6" 25029001 25030000 "*" 1.96005695991541e-05 2.0534467543854e-05 60
"chr6" 25344001 25345000 "*" 6.04438721296674e-11 1.38849255306602e-10 -100
"chr6" 25607001 25608000 "*" 2.88779000712225e-11 6.92935809466068e-11 -100
"chr6" 25685001 25686000 "*" 4.90718576884319e-14 1.6794399166207e-13 100
"chr6" 26002001 26003000 "*" 3.84614562420893e-12 1.04062035914347e-11

66.6666666666667
"chr6" 26023001 26024000 "*" 5.08482145278322e-14 1.73432706170696e-13 -100
"chr6" 26053001 26054000 "*" 0 0 -100
"chr6" 26189001 26190000 "*" 0 0 -75
"chr6" 26234001 26235000 "*" 0 0 87.292817679558
"chr6" 26250001 26251000 "*" 0 0 -52.5821596244131
"chr6" 26502001 26503000 "*" 0 0 90.5172413793103
"chr6" 26556001 26557000 "*" 1.16980699083058e-06 1.47157860501514e-06
-52.3809523809524
"chr6" 26571001 26572000 "*" 1.32893696047631e-13 4.30574836768249e-13
63.6363636363636
"chr6" 27014001 27015000 "*" 0 0 90.4761904761905
"chr6" 27168001 27169000 "*" 6.37490060739765e-13 1.89496793587877e-12 -100
"chr6" 27229001 27230000 "*" 0 0 100
"chr6" 27259001 27260000 "*" 0 0 96.3414634146341
"chr6" 27378001 27379000 "*" 1.52794177310511e-09 2.87642564232045e-09 -100
"chr6" 27473001 27474000 "*" 7.22364786875929e-08 1.06794048697674e-07 80
"chr6" 27486001 27487000 "*" 0 0 100
"chr6" 27583001 27584000 "*" 3.95353005888666e-09 7.03928529283733e-09 100
"chr6" 27598001 27599000 "*" 0 0 77.3462783171521
"chr6" 27615001 27616000 "*" 0 0 100
"chr6" 27646001 27647000 "*" 1.67299207820548e-08 2.71377429441055e-08 -100
"chr6" 27662001 27663000 "*" 2.18713935851156e-14 7.80893646430581e-14 100
"chr6" 27707001 27708000 "*" 1.25093098791718e-05 1.35030371915773e-05
65.7894736842105
"chr6" 27725001 27726000 "*" 0 0 -53.4437946718648
"chr6" 28175001 28176000 "*" 0 0 87.2443181818182
"chr6" 28304001 28305000 "*" 4.02167188440217e-12 1.08266700497926e-11 -100
"chr6" 28398001 28399000 "*" 5.56368978665844e-08 8.38936188628635e-08
-78.4313725490196
"chr6" 28436001 28437000 "*" 3.82249787378441e-13 1.17179661412484e-12
66.6666666666667
"chr6" 28446001 28447000 "*" 3.47499806707674e-14 1.2108513088053e-13
-76.9230769230769
"chr6" 33462001 33463000 "*" 1.11022302462516e-16 5.03662826618488e-16 100
"chr6" 33526001 33527000 "*" 3.33066907387547e-16 1.4495649018245e-15 -100
"chr6" 33600001 33601000 "*" 0 0 -78.5977859778598
"chr6" 33649001 33650000 "*" 5.794809077031e-12 1.52911192757929e-11 100
"chr6" 33699001 33700000 "*" 6.4152538836737e-10 1.27477697728417e-09 -100
"chr6" 33712001 33713000 "*" 1.37828859436695e-10 3.01445544267661e-10 -100
"chr6" 33716001 33717000 "*" 2.22044604925031e-16 9.81641919380259e-16 -100
"chr6" 33749001 33750000 "*" 1.40908092058911e-05 1.50885051053891e-05
61.3636363636364
"chr6" 33768001 33769000 "*" 1.35003119794419e-13 4.3679847476192e-13 100
"chr6" 33769001 33770000 "*" 3.33066907387547e-16 1.4495649018245e-15 100
"chr6" 33770001 33771000 "*" 5.51780843238703e-14 1.87243792572872e-13 100
"chr6" 33773001 33774000 "*" 6.4152538836737e-10 1.27477697728417e-09 -100
"chr6" 33827001 33828000 "*" 0 0 -60
"chr6" 33858001 33859000 "*" 4.02167188440217e-12 1.08266700497926e-11 -100
"chr6" 33887001 33888000 "*" 4.90718576884319e-14 1.6794399166207e-13 -100
"chr6" 33927001 33928000 "*" 2.59839720762045e-06 3.10853141312577e-06
-71.4285714285714

"chr6" 33949001 33950000 "*" 0 0 -100
"chr6" 33955001 33956000 "*" 4.22520890053235e-07 5.63816587435143e-07 51.8518518518519
"chr6" 33975001 33976000 "*" 5.15174656934292e-07 6.79754547675047e-07 57.1428571428571
"chr6" 34026001 34027000 "*" 7.99360577730113e-15 3.00221693743992e-14 -63.5714285714286
"chr6" 34036001 34037000 "*" 2.40882539492837e-06 2.89682508998185e-06 65.625
"chr6" 34042001 34043000 "*" 0 0 100
"chr6" 34043001 34044000 "*" 1.13140401492018e-08 1.87801380787728e-08 -100
"chr6" 34046001 34047000 "*" 0 0 77.7777777777778
"chr6" 34047001 34048000 "*" 2.4974244894338e-11 6.05590925882328e-11 -61.4678899082569
"chr6" 34050001 34051000 "*" 3.59781426872985e-09 6.45124306801663e-09 53.6474164133739
"chr6" 34051001 34052000 "*" 0 0 70.7432432432432
"chr6" 34055001 34056000 "*" 0 0 72.6154868727152
"chr6" 34065001 34066000 "*" 4.00586621385202e-06 4.65588073919783e-06 -64.8648648648649
"chr6" 34083001 34084000 "*" 0 0 -76.1904761904762
"chr6" 34091001 34092000 "*" 1.5634673446896e-05 1.66336190218994e-05 54.5454545454545
"chr6" 34092001 34093000 "*" 0 0 65.3348554033486
"chr6" 34119001 34120000 "*" 0 0 73.6842105263158
"chr6" 34120001 34121000 "*" 1.28785870856518e-14 4.71440608755782e-14 72.6495726495726
"chr6" 34165001 34166000 "*" 9.65729496371637e-10 1.87255912493232e-09 -100
"chr6" 34175001 34176000 "*" 1.30340183090993e-13 4.22662705118657e-13 -100
"chr6" 34191001 34192000 "*" 4.44089209850063e-15 1.71914916534614e-14 50.2256608639587
"chr6" 34203001 34204000 "*" 0 0 -72.0117296918768
"chr6" 34212001 34213000 "*" 0 0 -87.5675675675676
"chr6" 34218001 34219000 "*" 4.01313771103418e-09 7.11882087237587e-09 -100
"chr6" 34244001 34245000 "*" 5.48450174164827e-14 1.86325824996938e-13 68.4210526315789
"chr6" 34409001 34410000 "*" 8.7349629751543e-09 1.47505871898511e-08 100
"chr6" 34465001 34466000 "*" 0 0 66.6666666666667
"chr6" 34483001 34484000 "*" 0.000313805247935783 0.000271112523987329 60
"chr6" 34947001 34948000 "*" 4.65637306490407e-10 9.46720524046618e-10 -100
"chr6" 35149001 35150000 "*" 0 0 -92.6966292134831
"chr6" 35183001 35184000 "*" 5.04263297784746e-13 1.52078941532818e-12 -100
"chr6" 35286001 35287000 "*" 0 0 61.4340969236849
"chr6" 35311001 35312000 "*" 2.61211192886801e-06 3.12359423291548e-06 72.2222222222222
"chr6" 35394001 35395000 "*" 1.05849192744145e-08 1.76915297872532e-08 -72.7272727272727
"chr6" 35453001 35454000 "*" 0 0 -62.2641509433962
"chr6" 35468001 35469000 "*" 6.66133814775094e-16 2.81595744474255e-15 -100
"chr6" 35530001 35531000 "*" 1.12055920098442e-11 2.84500040664577e-11 100
"chr6" 35544001 35545000 "*" 9.65729496371637e-10 1.87255912493232e-09 100
"chr6" 35657001 35658000 "*" 0 0 -72.8602150537635
"chr6" 35658001 35659000 "*" 3.34128893442198e-08 5.19772864618523e-08 100

"chr6" 35693001 35694000 "*" 1.49613654798486e-12 4.26244554429779e-12 100
"chr6" 35703001 35704000 "*" 4.17234969685154e-07 5.57208763360643e-07
64.7058823529412
"chr6" 35705001 35706000 "*" 0 0 -69.4444444444444
"chr6" 35744001 35745000 "*" 0 0 73.3118971061093
"chr6" 35925001 35926000 "*" 0 0 -100
"chr6" 35987001 35988000 "*" 4.44089209850063e-16 1.91071758245033e-15 -100
"chr6" 36098001 36099000 "*" 0 0 -78.3166904422254
"chr6" 36099001 36100000 "*" 0 0 -100
"chr6" 36110001 36111000 "*" 1.01034983823745e-07 1.4653060412968e-07
54.5454545454545
"chr6" 36215001 36216000 "*" 7.14512775001896e-06 8.00640387871802e-06
63.6363636363636
"chr6" 36247001 36248000 "*" 2.82773804372027e-13 8.79129664109438e-13 -100
"chr6" 36276001 36277000 "*" 6.11364625591193e-10 1.22332619666323e-09
96.6666666666667
"chr6" 36288001 36289000 "*" 4.34379199276691e-11 1.01642803346655e-10
-55.9523809523809
"chr6" 36308001 36309000 "*" 1.33221246645387e-06 1.66281219733866e-06
67.6923076923077
"chr6" 36336001 36337000 "*" 2.22044604925031e-16 9.81641919380259e-16 62.5
"chr6" 36340001 36341000 "*" 2.43005615629954e-09 4.45014222280278e-09 100
"chr6" 36358001 36359000 "*" 6.66133814775094e-16 2.81595744474255e-15 100
"chr6" 36567001 36568000 "*" 0 0 -71.8675179569034
"chr6" 36595001 36596000 "*" 4.44089209850063e-16 1.91071758245033e-15
-93.3333333333333
"chr6" 36641001 36642000 "*" 0 0 -100
"chr6" 36699001 36700000 "*" 3.42205142844776e-06 4.01962584432396e-06
-60.5263157894737
"chr6" 36702001 36703000 "*" 1.65223390524716e-11 4.1020915472308e-11
72.1705426356589
"chr6" 36707001 36708000 "*" 1.80770820534804e-09 3.37846860013928e-09 -52
"chr6" 36729001 36730000 "*" 0 0 -100
"chr6" 36765001 36766000 "*" 7.3554808244225e-08 1.08588645363448e-07 -55
"chr6" 36805001 36806000 "*" 1.93720595120794e-11 4.76309924836636e-11 -100
"chr6" 36813001 36814000 "*" 3.24632510040335e-06 3.82701178814152e-06
65.5172413793103
"chr6" 36875001 36876000 "*" 1.13140401492018e-08 1.87801380787728e-08 -100
"chr6" 36917001 36918000 "*" 6.4152538836737e-10 1.27477697728417e-09 -100
"chr6" 36931001 36932000 "*" 1.29037891483108e-11 3.24582280286866e-11 75
"chr6" 36952001 36953000 "*" 0 0 -100
"chr6" 36992001 36993000 "*" 0 0 -100
"chr6" 37105001 37106000 "*" 4.25205759491121e-10 8.69924118657886e-10
66.6666666666667
"chr6" 37106001 37107000 "*" 4.78841410966879e-11 1.11468187156588e-10 100
"chr6" 37190001 37191000 "*" 1.91069382537989e-13 6.08032649314634e-13 81
"chr6" 37453001 37454000 "*" 5.28156407497704e-11 1.22228238232754e-10
71.4285714285714
"chr6" 37483001 37484000 "*" 9.08108033215171e-11 2.03407753381119e-10 100
"chr6" 37501001 37502000 "*" 6.66133814775094e-16 2.81595744474255e-15 100
"chr6" 37512001 37513000 "*" 1.40665257220007e-13 4.54219470790239e-13
-77.7777777777778

"chr6" 37515001 37516000 "*" 5.98093130577126e-09 1.03755270702472e-08 75
"chr6" 37541001 37542000 "*" 4.48406133335411e-08 6.84482503893299e-08
66.6666666666667
"chr6" 37544001 37545000 "*" 2.15705897588236e-10 4.59280628014627e-10 -52
"chr6" 37549001 37550000 "*" 3.04324343503026e-11 7.26599935673121e-11 100
"chr6" 37556001 37557000 "*" 3.52704532247117e-11 8.35581292290093e-11 100
"chr6" 37557001 37558000 "*" 0 0 100
"chr6" 37604001 37605000 "*" 0 0 100
"chr6" 37664001 37665000 "*" 0 0 80.0310692308836
"chr6" 37667001 37668000 "*" 0 0 90.5325443786982
"chr6" 37670001 37671000 "*" 0 0 100
"chr6" 37750001 37751000 "*" 5.51780843238703e-14 1.87243792572872e-13 100
"chr6" 37791001 37792000 "*" 0 0 100
"chr6" 38020001 38021000 "*" 1.88092874608969e-10 4.0506821693965e-10
-80.7692307692308
"chr6" 38084001 38085000 "*" 2.30926389122033e-14 8.22700632271376e-14 88.8
"chr6" 38133001 38134000 "*" 0 0 -78.6407766990291
"chr6" 38180001 38181000 "*" 8.25450818808804e-13 2.42473843346228e-12 -100
"chr6" 38559001 38560000 "*" 3.90831811358794e-12 1.05610567215048e-11 -100
"chr6" 38585001 38586000 "*" 7.23027242033325e-06 8.09409072666536e-06
-60.8695652173913
"chr6" 38607001 38608000 "*" 0 0 60.1359003397508
"chr6" 39055001 39056000 "*" 4.10928544795075e-05 4.09327063440139e-05
-66.6666666666667
"chr6" 39056001 39057000 "*" 5.79099879161049e-09 1.00638314552934e-08
55.8823529411765
"chr6" 39132001 39133000 "*" 3.6700841921089e-08 5.65530226072258e-08 100
"chr6" 39134001 39135000 "*" 7.97140131680862e-14 2.65641277678906e-13 100
"chr6" 39249001 39250000 "*" 1.29282817784571e-09 2.46378838503747e-09 -80
"chr6" 39261001 39262000 "*" 1.60518918485009e-05 1.70507414869409e-05
-55.3191489361702
"chr6" 39266001 39267000 "*" 5.48638023900594e-10 1.10374357336421e-09 -100
"chr6" 39288001 39289000 "*" 0 0 -100
"chr6" 39307001 39308000 "*" 3.68371999570627e-13 1.13149976091305e-12 100
"chr6" 39409001 39410000 "*" 9.04570973681018e-10 1.76414389822173e-09 100
"chr6" 39760001 39761000 "*" 0 0 50.4315380888471
"chr6" 39782001 39783000 "*" 3.73034936274053e-14 1.29420415488591e-13 -100
"chr6" 39816001 39817000 "*" 2.08814465718632e-09 3.86232567960463e-09 -100
"chr6" 39828001 39829000 "*" 3.04201108747293e-14 1.06563274901204e-13 -100
"chr6" 39841001 39842000 "*" 4.86753970463383e-11 1.13145908161915e-10 -100
"chr6" 39853001 39854000 "*" 1.29037891483108e-11 3.24582280286866e-11
-66.6666666666667
"chr6" 39864001 39865000 "*" 0 0 -52.8089887640449
"chr6" 39879001 39880000 "*" 1.74853465040314e-11 4.32752710900609e-11 -100
"chr6" 40008001 40009000 "*" 4.53591360638494e-06 5.23031683672762e-06 90
"chr6" 40130001 40131000 "*" 7.0006535457523e-08 1.03695832512054e-07 100
"chr6" 40366001 40367000 "*" 3.95353005888666e-09 7.03928529283733e-09 100
"chr6" 40387001 40388000 "*" 2.73625566649116e-12 7.52483363579137e-12 -100
"chr6" 40498001 40499000 "*" 4.86806139137208e-05 4.79644298527637e-05
66.6666666666667
"chr6" 40507001 40508000 "*" 4.44089209850063e-16 1.91071758245033e-15 -100
"chr6" 40574001 40575000 "*" 6.92287338566189e-11 1.57605630124247e-10 100

"chr6" 40607001 40608000 "*" 7.67056418382595e-11 1.7359448580318e-10 -100
"chr6" 40617001 40618000 "*" 6.24654217240561e-10 1.2462108191303e-09 -100
"chr6" 40782001 40783000 "*" 5.01025332333427e-10 1.01358031135889e-09 100
"chr6" 40783001 40784000 "*" 1.73037564055178e-07 2.43019221408804e-07
-58.974358974359
"chr6" 40837001 40838000 "*" 9.40446676089834e-09 1.58174336517085e-08
56.5217391304348
"chr6" 40865001 40866000 "*" 7.0006535457523e-08 1.03695832512054e-07 100
"chr6" 40904001 40905000 "*" 4.44089209850063e-15 1.71914916534614e-14 100
"chr6" 41057001 41058000 "*" 2.18752359892882e-07 3.03284837335955e-07
-62.0689655172414
"chr6" 41118001 41119000 "*" 0 0 -100
"chr6" 41131001 41132000 "*" 4.08209022140227e-11 9.57770690255255e-11 100
"chr6" 41191001 41192000 "*" 3.71480624039577e-12 1.00717606565582e-11 52
"chr6" 41207001 41208000 "*" 0 0 89.2561983471074
"chr6" 41243001 41244000 "*" 3.31690230837012e-12 9.03347040781321e-12 -100
"chr6" 41254001 41255000 "*" 4.32209823486573e-12 1.15935290479641e-11 100
"chr6" 41388001 41389000 "*" 0.000145914216309273 0.000133213803069232
64.7058823529412
"chr6" 41395001 41396000 "*" 0 0 -63.1578947368421
"chr6" 41431001 41432000 "*" 8.35519651687378e-07 1.07184706335147e-06
60.2857142857143
"chr6" 41604001 41605000 "*" 0 0 -84.5369906551742
"chr6" 41631001 41632000 "*" 4.01313771103418e-09 7.11882087237587e-09 -100
"chr6" 41645001 41646000 "*" 0 0 63.7592137592138
"chr6" 41646001 41647000 "*" 3.51407791754355e-11 8.33574753346092e-11
-96.7741935483871
"chr6" 41655001 41656000 "*" 1.60436434548661e-06 1.97992193970072e-06 73.4375
"chr6" 41661001 41662000 "*" 2.37587727269783e-14 8.44268514944447e-14 -100
"chr6" 41680001 41681000 "*" 1.20122249176458e-05 1.30050098695175e-05
-59.7014925373134
"chr6" 41756001 41757000 "*" 0 0 69.3877551020408
"chr6" 41997001 41998000 "*" 7.67056418382595e-11 1.7359448580318e-10 100
"chr6" 42000001 42001000 "*" 0 0 100
"chr6" 42061001 42062000 "*" 3.53495112070945e-07 4.76842459153734e-07
-63.4146341463415
"chr6" 42067001 42068000 "*" 6.34990274681613e-05 6.14454813633287e-05
53.448275862069
"chr6" 42086001 42087000 "*" 2.64951482975562e-09 4.82186011375752e-09 -100
"chr6" 42103001 42104000 "*" 6.67017106771306e-05 6.43065783242906e-05
-60.7142857142857
"chr6" 42110001 42111000 "*" 0 0 -66.3934426229508
"chr6" 42129001 42130000 "*" 6.9127142343639e-08 1.02919682435385e-07
50.6132756132756
"chr6" 42162001 42163000 "*" 5.25299859166495e-09 9.17245030097968e-09
52.3809523809524
"chr6" 42255001 42256000 "*" 0.000107302650472541 0.000100116528873172
-59.4594594594595
"chr6" 42262001 42263000 "*" 3.34128893442198e-08 5.19772864618523e-08 -100
"chr6" 42371001 42372000 "*" 8.01666433236647e-09 1.36392368970109e-08 100
"chr6" 42413001 42414000 "*" 0 0 100
"chr6" 42421001 42422000 "*" 2.83145663582118e-08 4.451459157891e-08

50.5813953488372
"chr6" 42642001 42643000 "*" 1.39779633423487e-07 1.98463037958589e-07 100
"chr6" 42834001 42835000 "*" 3.34128893442198e-08 5.19772864618523e-08 100
"chr6" 42848001 42849000 "*" 0 0 -100
"chr6" 42858001 42859000 "*" 7.67452768002386e-11 1.73679471800727e-10
-50.8312020460358
"chr6" 42948001 42949000 "*" 1.89848137210902e-14 6.82212372863451e-14 -100
"chr6" 43009001 43010000 "*" 1.25284005392245e-10 2.75649590272477e-10 100
"chr6" 43064001 43065000 "*" 1.88370181564146e-07 2.63373029465817e-07
-76.9230769230769
"chr6" 43081001 43082000 "*" 2.22044604925031e-16 9.81641919380259e-16
-58.8235294117647
"chr6" 43101001 43102000 "*" 2.64951482975562e-09 4.82186011375752e-09 100
"chr6" 43110001 43111000 "*" 7.00963011079914e-07 9.08933792624803e-07
73.3333333333333
"chr6" 43111001 43112000 "*" 0 0 -72.9032258064516
"chr6" 43183001 43184000 "*" 6.92287338566189e-11 1.57605630124247e-10 -100
"chr6" 43210001 43211000 "*" 0 0 90.1960784313726
"chr6" 43211001 43212000 "*" 0 0 58.8603474771621
"chr6" 43219001 43220000 "*" 0 0 75
"chr6" 43238001 43239000 "*" 4.79616346638068e-14 1.64380445416596e-13 -100
"chr6" 43271001 43272000 "*" 1.34174751309502e-08 2.20798085573453e-08
-86.3636363636364
"chr6" 43422001 43423000 "*" 0 0 63.8721301705439
"chr6" 43423001 43424000 "*" 0 0 80.1657244600613
"chr6" 43468001 43469000 "*" 9.04570973681018e-10 1.76414389822173e-09 -100
"chr6" 43603001 43604000 "*" 0 0 90.5660377358491
"chr6" 43623001 43624000 "*" 2.16715534406831e-13 6.83749209869485e-13 68.75
"chr6" 43685001 43686000 "*" 0 0 -61.3636363636364
"chr6" 43692001 43693000 "*" 0 0 -84.1004184100418
"chr6" 43693001 43694000 "*" 0 0 -100
"chr6" 43737001 43738000 "*" 5.10027686750902e-10 1.03096692961214e-09
66.6666666666667
"chr6" 43750001 43751000 "*" 0 0 -100
"chr6" 43911001 43912000 "*" 8.01666433236647e-09 1.36392368970109e-08 100
"chr6" 43968001 43969000 "*" 4.13002965160558e-14 1.42427783430954e-13 -100
"chr6" 43991001 43992000 "*" 9.32032229172819e-13 2.7181920310254e-12 100
"chr6" 44012001 44013000 "*" 1.6587858476802e-06 2.04236557510993e-06
63.6363636363636
"chr6" 44022001 44023000 "*" 3.46500605985511e-13 1.06777978272295e-12 -100
"chr6" 44032001 44033000 "*" 1.05434549979577e-11 2.69023892583463e-11
70.5882352941177
"chr6" 44096001 44097000 "*" 1.89703808217700e-12 5.32618408455207e-12
-66.6666666666667
"chr6" 44119001 44120000 "*" 0 0 -68.9911265432099
"chr6" 44129001 44130000 "*" 0 0 -100
"chr6" 44236001 44237000 "*" 3.33066907387547e-16 1.4495649018245e-15 -100
"chr6" 44237001 44238000 "*" 1.65762831239036e-08 2.69908935123112e-08
78.9473684210526
"chr6" 44259001 44260000 "*" 5.2003549414481e-06 5.95024319668787e-06
66.6666666666667
"chr6" 44264001 44265000 "*" 8.11041367398957e-09 1.3788893142866e-08

85.8974358974359
"chr6" 44265001 44266000 "*" 0 0 67.7605991891706
"chr6" 44329001 44330000 "*" 8.25450818808804e-13 2.42473843346228e-12 100
"chr6" 44429001 44430000 "*" 3.40125705378114e-10 7.03784073980162e-10 -100
"chr6" 44463001 44464000 "*" 6.24654217240561e-10 1.2462108191303e-09 100
"chr6" 44566001 44567000 "*" 1.21712351308645e-09 2.32599961709438e-09
81.6326530612245
"chr6" 44609001 44610000 "*" 0 0 100
"chr6" 44632001 44633000 "*" 0 0 -85.0746268656716
"chr6" 44651001 44652000 "*" 1.67299207820548e-08 2.71377429441055e-08 100
"chr6" 44747001 44748000 "*" 2.77555756156289e-15 1.10015415195978e-14 100
"chr6" 44756001 44757000 "*" 2.06501482580279e-14 7.3890370570066e-14 100
"chr6" 45390001 45391000 "*" 0 0 64.3153526970954
"chr6" 45895001 45896000 "*" 4.08209022140227e-11 9.57770690255255e-11 -100
"chr6" 45897001 45898000 "*" 4.02167188440217e-12 1.08266700497926e-11 -100
"chr6" 46173001 46174000 "*" 2.38031816479634e-13 7.47299807843568e-13 100
"chr6" 46703001 46704000 "*" 0 0 50.0041211621677
"chr6" 46863001 46864000 "*" 6.30384633382164e-13 1.87665599796749e-12 -100
"chr6" 46894001 46895000 "*" 1.29037891483108e-11 3.24582280286866e-11 -100
"chr6" 47210001 47211000 "*" 0 0 -100
"chr6" 47462001 47463000 "*" 7.0006535457523e-08 1.03695832512054e-07 100
"chr6" 47502001 47503000 "*" 2.33028818463765e-10 4.93719283454501e-10 -100
"chr6" 48951001 48952000 "*" 0 0 56.0343481654957
"chr6" 49518001 49519000 "*" 2.22044604925031e-15 8.90449334476539e-15
-55.8823529411765
"chr6" 49525001 49526000 "*" 4.01313771103418e-09 7.11882087237587e-09 100
"chr6" 50873001 50874000 "*" 0 0 -100
"chr6" 51088001 51089000 "*" 1.39230795903611e-06 1.73395458284971e-06 -75
"chr6" 51409001 51410000 "*" 1.39779633423487e-07 1.98463037958589e-07 100
"chr6" 51719001 51720000 "*" 0 0 -100
"chr6" 51760001 51761000 "*" 1.93720595120794e-11 4.76309924836636e-11 -100
"chr6" 51920001 51921000 "*" 1.22124532708767e-15 5.03921984217778e-15 -100
"chr6" 51950001 51951000 "*" 6.99440505513849e-14 2.34592719571672e-13
-52.5423728813559
"chr6" 51983001 51984000 "*" 5.39619190880192e-05 5.27870047241069e-05
52.1739130434783
"chr6" 52000001 52001000 "*" 2.77655010094691e-10 5.82996509542432e-10 -90
"chr6" 52227001 52228000 "*" 8.72992785838633e-08 1.27655311279844e-07
-73.2558139534884
"chr6" 52285001 52286000 "*" 0 0 91.5789473684211
"chr6" 52368001 52369000 "*" 1.11022302462516e-16 5.03662826618488e-16 -100
"chr6" 52369001 52370000 "*" 1.66533453693773e-15 6.7629186866784e-15
65.8227848101266
"chr6" 52371001 52372000 "*" 1.98325800226939e-11 4.85612402473442e-11 100
"chr6" 52535001 52536000 "*" 0 0 -68.75
"chr6" 52660001 52661000 "*" 2.91028312560115e-11 6.97590507139978e-11 100
"chr6" 52734001 52735000 "*" 1.36604061395929e-11 3.42333952202875e-11 100
"chr6" 52746001 52747000 "*" 1.52100632089258e-09 2.8746597910115e-09
-66.6666666666667
"chr6" 52899001 52900000 "*" 8.29789400635939e-07 1.06506939918901e-06 90
"chr6" 53069001 53070000 "*" 1.47803887560194e-05 1.5780310930685e-05
65.7142857142857

"chr6" 53071001 53072000 "*" 3.26405569239796e-14 1.13973003849234e-13 -100
"chr6" 53223001 53224000 "*" 2.15344186926814e-10 4.58569010036229e-10 95
"chr6" 53257001 53258000 "*" 2.33028818463765e-10 4.93719283454501e-10 -100
"chr6" 53517001 53518000 "*" 0 0 71.6049382716049
"chr6" 53609001 53610000 "*" 1.74853465040314e-11 4.32752710900609e-11 100
"chr6" 54160001 54161000 "*" 0 0 -100
"chr6" 55398001 55399000 "*" 1.7755082201254e-05 1.87393864811155e-05
64.6153846153846
"chr6" 56293001 56294000 "*" 4.65637306490407e-10 9.46720524046618e-10 100
"chr6" 56650001 56651000 "*" 6.77335965093562e-12 1.76897011246462e-11 100
"chr6" 56716001 56717000 "*" 4.13002965160558e-14 1.42427783430954e-13 -100
"chr6" 58142001 58143000 "*" 4.08209022140227e-11 9.57770690255255e-11 100
"chr6" 63305001 63306000 "*" 1.12458486967171e-09 2.15740815043925e-09 100
"chr6" 63553001 63554000 "*" 0 0 100
"chr6" 63939001 63940000 "*" 4.65637306490407e-10 9.46720524046618e-10 -100
"chr6" 64186001 64187000 "*" 2.33028818463765e-10 4.93719283454501e-10 100
"chr6" 64415001 64416000 "*" 6.70841160399505e-12 1.75486545176836e-11 100
"chr6" 64588001 64589000 "*" 7.99844565668728e-06 8.89173797399106e-06
66.6666666666667
"chr6" 64908001 64909000 "*" 0 0 -100
"chr6" 65279001 65280000 "*" 2.61627457331937e-06 3.12839253998582e-06
-63.6363636363636
"chr6" 65391001 65392000 "*" 5.59924264731393e-08 8.43900425199566e-08
77.0491803278689
"chr6" 67740001 67741000 "*" 0 0 87.0967741935484
"chr6" 67833001 67834000 "*" 1.39779633423487e-07 1.98463037958589e-07 100
"chr6" 68372001 68373000 "*" 1.89581683684992e-12 5.32311367606535e-12 100
"chr6" 68876001 68877000 "*" 0 0 100
"chr6" 69243001 69244000 "*" 8.9207485842735e-10 1.74356506625072e-09
71.3114754098361
"chr6" 69534001 69535000 "*" 4.73234496034536e-09 8.30627300264826e-09 100
"chr6" 69920001 69921000 "*" 2.22044604925031e-15 8.90449334476539e-15 -100
"chr6" 69942001 69943000 "*" 0 0 100
"chr6" 70311001 70312000 "*" 0 0 100
"chr6" 70577001 70578000 "*" 0 0 92.5531914893617
"chr6" 71123001 71124000 "*" 0 0 75.1724137931034
"chr6" 71183001 71184000 "*" 2.18158824338843e-13 6.87314823207509e-13 -100
"chr6" 71665001 71666000 "*" 0 0 61.241866910953
"chr6" 71999001 72000000 "*" 2.88657986402541e-14 1.01472190734842e-13
51.7678200037814
"chr6" 72400001 72401000 "*" 3.04324343503026e-11 7.26599935673121e-11 -100
"chr6" 72595001 72596000 "*" 6.73765848707708e-05 6.4912196768517e-05 65
"chr6" 73122001 73123000 "*" 1.16573417585641e-14 4.29335582900508e-14 100
"chr6" 73124001 73125000 "*" 4.73234496034536e-09 8.30627300264826e-09 100
"chr6" 73196001 73197000 "*" 8.3882012447134e-11 1.88922989503423e-10 100
"chr6" 73209001 73210000 "*" 0 0 -100
"chr6" 73796001 73797000 "*" 8.7349629751543e-09 1.47505871898511e-08 100
"chr6" 73859001 73860000 "*" 0 0 100
"chr6" 73904001 73905000 "*" 1.67299207820548e-08 2.71377429441055e-08 100
"chr6" 74025001 74026000 "*" 3.81189302345319e-10 7.83967584656543e-10 83.75
"chr6" 74082001 74083000 "*" 7.7715611723761e-16 3.26213507634405e-15 100
"chr6" 74224001 74225000 "*" 6.77335965093562e-12 1.76897011246462e-11 -100

"chr6" 74405001 74406000 "*" 0 0 57.5788102094671
"chr6" 74406001 74407000 "*" 0 0 61.9399747312212
"chr6" 74612001 74613000 "*" 5.76264480800504e-07 7.56363654553562e-07
50.2487562189055
"chr6" 74620001 74621000 "*" 4.70376358387981e-05 4.64559371515489e-05 60
"chr6" 74731001 74732000 "*" 4.73234496034536e-09 8.30627300264826e-09 100
"chr6" 75741001 75742000 "*" 6.15840711759574e-13 1.83676803572802e-12 100
"chr6" 76207001 76208000 "*" 2.08814465718632e-09 3.86232567960463e-09 100
"chr6" 76211001 76212000 "*" 6.4152538836737e-10 1.27477697728417e-09 100
"chr6" 76458001 76459000 "*" 6.88159569328661e-08 1.02471009495575e-07
54.5454545454545
"chr6" 76589001 76590000 "*" 3.6700841921089e-08 5.65530226072258e-08 100
"chr6" 76614001 76615000 "*" 5.01025332333427e-10 1.01358031135889e-09 100
"chr6" 76644001 76645000 "*" 4.02167188440217e-12 1.08266700497926e-11 100
"chr6" 76951001 76952000 "*" 6.04438721296674e-11 1.38849255306602e-10 -100
"chr6" 77585001 77586000 "*" 9.67004254448511e-14 3.18178990782609e-13 -100
"chr6" 77720001 77721000 "*" 4.44089209850063e-16 1.91071758245033e-15 -100
"chr6" 79172001 79173000 "*" 9.29915022762628e-09 1.56529277294275e-08
-50.7246376811594
"chr6" 79449001 79450000 "*" 5.01025332333427e-10 1.01358031135889e-09 100
"chr6" 80447001 80448000 "*" 1.92946192356658e-09 3.59193011611483e-09 -100
"chr6" 80487001 80488000 "*" 0 0 -87.037037037037
"chr6" 80777001 80778000 "*" 0 0 66.6666666666667
"chr6" 81156001 81157000 "*" 1.07882591748876e-11 2.74553537455803e-11 100
"chr6" 81557001 81558000 "*" 0 0 58.2089552238806
"chr6" 81768001 81769000 "*" 1.50623957750895e-12 4.28756717183481e-12 100
"chr6" 81984001 81985000 "*" 9.04570973681018e-10 1.76414389822173e-09 100
"chr6" 82047001 82048000 "*" 2.54058807325208e-09 4.64317868304603e-09 -60
"chr6" 82715001 82716000 "*" 1.12055920098442e-11 2.84500040664577e-11 -100
"chr6" 84234001 84235000 "*" 0 0 -100
"chr6" 84743001 84744000 "*" 0 0 54.2763157894737
"chr6" 85132001 85133000 "*" 6.69020394639119e-13 1.98522618657383e-12
-76.6467065868264
"chr6" 85297001 85298000 "*" 0 0 -94.6372239747634
"chr6" 85477001 85478000 "*" 0 0 79.3814432989691
"chr6" 86024001 86025000 "*" 0 0 -100
"chr6" 86162001 86163000 "*" 4.73234496034536e-09 8.30627300264826e-09 -100
"chr6" 86620001 86621000 "*" 2.16382467499443e-13 6.82775854166559e-13 100
"chr6" 87185001 87186000 "*" 9.08108033215171e-11 2.03407753381119e-10 75
"chr6" 87646001 87647000 "*" 0 0 80.9954751131222
"chr6" 88411001 88412000 "*" 0 0 -100
"chr6" 88786001 88787000 "*" 3.530509218308e-14 1.22891039008692e-13 -100
"chr6" 88875001 88876000 "*" 0 0 93.5546875
"chr6" 89021001 89022000 "*" 5.63660229602192e-13 1.68905058935478e-12 -100
"chr6" 89188001 89189000 "*" 6.4324989779152e-11 1.47230020825092e-10 100
"chr6" 89275001 89276000 "*" 1.34456009104333e-07 1.91808919676809e-07
-62.1621621621622
"chr6" 89304001 89305000 "*" 0 0 -83.3333333333333
"chr6" 89913001 89914000 "*" 2.02327044007689e-12 5.65300384792621e-12 100
"chr6" 89996001 89997000 "*" 1.5277158427196e-09 2.87642564232045e-09 100
"chr6" 90116001 90117000 "*" 4.91828799908944e-14 1.68192819018269e-13 -100
"chr6" 90121001 90122000 "*" 0 0 89.1352549889135

"chr6" 90143001 90144000 "*" 0 0 53.3601692411869
"chr6" 90205001 90206000 "*" 2.64951482975562e-09 4.82186011375752e-09 -100
"chr6" 90240001 90241000 "*" 2.33590924381133e-13 7.3418676838825e-13 -100
"chr6" 90253001 90254000 "*" 6.59550958292954e-09 1.13456194382773e-08 100
"chr6" 90254001 90255000 "*" 0 0 100
"chr6" 90272001 90273000 "*" 9.65729496371637e-10 1.87255912493232e-09 -100
"chr6" 90286001 90287000 "*" 6.77335965093562e-12 1.76897011246462e-11 -100
"chr6" 90562001 90563000 "*" 9.65729496371637e-10 1.87255912493232e-09 -100
"chr6" 90640001 90641000 "*" 4.84057238736568e-14 1.65847962714061e-13
-69.7674418604651
"chr6" 90922001 90923000 "*" 5.48638023900594e-10 1.10374357336421e-09 100
"chr6" 91005001 91006000 "*" 0 0 72.65662078785
"chr6" 91448001 91449000 "*" 2.88779000712225e-11 6.92935809466068e-11 -100
"chr6" 91658001 91659000 "*" 0 0 -100
"chr6" 93798001 93799000 "*" 1.11022302462516e-16 5.03662826618488e-16 100
"chr6" 94127001 94128000 "*" 0 0 56.0533707865169
"chr6" 95054001 95055000 "*" 2.15125472990962e-10 4.58126659943874e-10 100
"chr6" 95221001 95222000 "*" 0 0 99.3333333333333
"chr6" 95946001 95947000 "*" 0 0 100
"chr6" 96824001 96825000 "*" 8.77076189453874e-15 3.27889038038365e-14 100
"chr6" 97285001 97286000 "*" 2.38697950294409e-14 8.4806915820873e-14
-50.0315671895488
"chr6" 97291001 97292000 "*" 0 0 100
"chr6" 99293001 99294000 "*" 0 0 97.986577181208
"chr6" 99762001 99763000 "*" 2.68450373042128e-10 5.64465213127486e-10 100
"chr6" 100039001 100040000 "*" 0 0 82.3219814241486
"chr6" 100909001 100910000 "*" 0 0 82.8125
"chr6" 102281001 102282000 "*" 1.14124265593318e-11 2.89138062669327e-11 100
"chr6" 102409001 102410000 "*" 1.98365768255826e-11 4.85612402473442e-11 100
"chr6" 102837001 102838000 "*" 5.79564123137644e-07 7.60299674531037e-07
79.3103448275862
"chr6" 103952001 103953000 "*" 2.08814465718632e-09 3.86232567960463e-09 100
"chr6" 104213001 104214000 "*" 3.04324343503026e-11 7.26599935673121e-11 100
"chr6" 104822001 104823000 "*" 1.26098514297013e-07 1.8047301459019e-07
76.5432098765432
"chr6" 104872001 104873000 "*" 1.5277158427196e-09 2.87642564232045e-09 100
"chr6" 104921001 104922000 "*" 0 0 -100
"chr6" 105539001 105540000 "*" 4.44089209850063e-15 1.71914916534614e-14 -100
"chr6" 105542001 105543000 "*" 1.22519986378578e-08 2.02546180433529e-08 56
"chr6" 105553001 105554000 "*" 9.71063229826541e-11 2.16781365050398e-10 100
"chr6" 105627001 105628000 "*" 0 0 73.2083673989679
"chr6" 105760001 105761000 "*" 1.4432899320127e-15 5.90750815956055e-15 -100
"chr6" 106328001 106329000 "*" 1.01104680183539e-11 2.58593007673131e-11
-76.6666666666667
"chr6" 106439001 106440000 "*" 6.4152538836737e-10 1.27477697728417e-09 100
"chr6" 106440001 106441000 "*" 1.42219569454483e-13 4.58954156292913e-13 -60
"chr6" 107073001 107074000 "*" 5.45877356672442e-06 6.22347026184263e-06
62.2222222222222
"chr6" 107781001 107782000 "*" 0 0 82.0900900900901
"chr6" 107899001 107900000 "*" 3.40125705378114e-10 7.03784073980162e-10 -100
"chr6" 107957001 107958000 "*" 0 0 100
"chr6" 108010001 108011000 "*" 6.6672514031918e-07 8.67356243114672e-07

-68.9655172413793
"chr6" 108034001 108035000 "*" 6.13999610421523e-06 6.94260980394374e-06
-72.2222222222222
"chr6" 108552001 108553000 "*" 1.78764125724484e-09 3.34246220795886e-09
86.6666666666667
"chr6" 108994001 108995000 "*" 8.55441273373003e-11 1.92484549887909e-10
78.2051282051282
"chr6" 109245001 109246000 "*" 0 0 -51.8518518518519
"chr6" 109276001 109277000 "*" 5.63660229602192e-13 1.68905058935478e-12 -100
"chr6" 109716001 109717000 "*" 1.004450997244e-05 1.1004376066266e-05
-83.3333333333333
"chr6" 109776001 109777000 "*" 0 0 -93.1623931623932
"chr6" 109787001 109788000 "*" 1.97840457016873e-07 2.75860297995596e-07
57.6923076923077
"chr6" 110677001 110678000 "*" 6.6743402238334e-07 8.68224270398495e-07
-52.0125786163522
"chr6" 110864001 110865000 "*" 6.89052814806246e-10 1.36414852716244e-09
-53.125
"chr6" 111150001 111151000 "*" 8.25450818808804e-13 2.42473843346228e-12 100
"chr6" 111207001 111208000 "*" 3.15857384111107e-05 3.20545210911178e-05
-58.3333333333333
"chr6" 111279001 111280000 "*" 0 0 80.9523809523809
"chr6" 111872001 111873000 "*" 0 0 100
"chr6" 111873001 111874000 "*" 1.74190771632388e-05 1.84120144479049e-05
72.7272727272727
"chr6" 112230001 112231000 "*" 9.80763359414993e-10 1.8980032424715e-09 -100
"chr6" 112346001 112347000 "*" 1.12458486967171e-09 2.15740815043925e-09 -100
"chr6" 112582001 112583000 "*" 4.73234496034536e-09 8.30627300264826e-09 -100
"chr6" 112805001 112806000 "*" 0 0 -100
"chr6" 113738001 113739000 "*" 2.08995487582797e-10 4.46944006972663e-10 -100
"chr6" 113804001 113805000 "*" 2.68450373042128e-10 5.64465213127486e-10 -100
"chr6" 114630001 114631000 "*" 2.08814465718632e-09 3.86232567960463e-09 100
"chr6" 114663001 114664000 "*" 0 0 67.8685607691204
"chr6" 114664001 114665000 "*" 0 0 75.1109559666883
"chr6" 114746001 114747000 "*" 4.21884749357559e-15 1.63874445239189e-14
-76.1904761904762
"chr6" 114765001 114766000 "*" 3.63840069184107e-11 8.59447570041556e-11
-61.2745098039216
"chr6" 115423001 115424000 "*" 6.92287338566189e-11 1.57605630124247e-10 -100
"chr6" 116170001 116171000 "*" 8.3882012447134e-11 1.88922989503423e-10 -100
"chr6" 116574001 116575000 "*" 1.48421164247736e-10 3.22758357929575e-10
62.1621621621622
"chr6" 116580001 116581000 "*" 6.66133814775094e-15 2.52791665956799e-14
96.2962962962963
"chr6" 116600001 116601000 "*" 5.55111512312578e-16 2.36485094870365e-15 -100
"chr6" 116691001 116692000 "*" 5.6532556413913e-13 1.69385831018468e-12
-60.5882352941176
"chr6" 117260001 117261000 "*" 3.37878894640653e-06 3.97266932237199e-06 -66
"chr6" 117528001 117529000 "*" 2.64951482975562e-09 4.82186011375752e-09 100
"chr6" 117587001 117588000 "*" 0 0 88.0132850241546
"chr6" 117802001 117803000 "*" 0 0 67.5324675324675
"chr6" 118946001 118947000 "*" 0 0 100

"chr6" 118972001 118973000 "*" 0 0 88.0952380952381
"chr6" 119009001 119010000 "*" 1.11022302462516e-16 5.03662826618488e-16 80
"chr6" 119090001 119091000 "*" 2.16589898094366e-06 2.62321901854342e-06
66.6666666666667
"chr6" 120076001 120077000 "*" 3.6700841921089e-08 5.65530226072258e-08 100
"chr6" 121107001 121108000 "*" 9.81786704157273e-08 1.4257900259851e-07
-63.6363636363636
"chr6" 121662001 121663000 "*" 3.6700841921089e-08 5.65530226072258e-08 100
"chr6" 121758001 121759000 "*" 0 0 96.2962962962963
"chr6" 121801001 121802000 "*" 1.78705816811231e-09 3.34167163843762e-09
-66.6666666666667
"chr6" 122932001 122933000 "*" 0 0 -78.6096256684492
"chr6" 123066001 123067000 "*" 0 0 -100
"chr6" 123110001 123111000 "*" 6.19948536950687e-13 1.84855702778088e-12
-66.6666666666667
"chr6" 123793001 123794000 "*" 5.10702591327572e-15 1.96271840288234e-14 -100
"chr6" 123907001 123908000 "*" 1.41778362938716e-07 2.01170748993017e-07
-66.6666666666667
"chr6" 124850001 124851000 "*" 8.43347613965761e-12 2.17859001990012e-11 -100
"chr6" 125475001 125476000 "*" 9.31892474298479e-09 1.56836811335503e-08
-53.3333333333333
"chr6" 125684001 125685000 "*" 0 0 61.1904761904762
"chr6" 126070001 126071000 "*" 0 0 65.3076923076923
"chr6" 126071001 126072000 "*" 0 0 93.4322033898305
"chr6" 126306001 126307000 "*" 3.52704532247117e-11 8.35581292290093e-11 -100
"chr6" 128388001 128389000 "*" 7.52398143788469e-13 2.2223590306936e-12 80
"chr6" 128523001 128524000 "*" 1.99840144432528e-12 5.59476580335607e-12 100
"chr6" 129115001 129116000 "*" 3.95353005888666e-09 7.03928529283733e-09 100
"chr6" 129478001 129479000 "*" 3.9190872769268e-14 1.35416418933905e-13 100
"chr6" 129574001 129575000 "*" 9.65729496371637e-10 1.87255912493232e-09 100
"chr6" 129980001 129981000 "*" 1.67299207820548e-08 2.71377429441055e-08 -100
"chr6" 129981001 129982000 "*" 6.99249547153613e-11 1.59121144852268e-10
92.3076923076923
"chr6" 130122001 130123000 "*" 3.10862446895044e-15 1.22466437093774e-14 -100
"chr6" 130686001 130687000 "*" 0 0 54.6734955185659
"chr6" 130993001 130994000 "*" 8.71525074330748e-14 2.88848349331391e-13 100
"chr6" 131191001 131192000 "*" 3.42392780794398e-13 1.05652643760713e-12
-94.1176470588235
"chr6" 131699001 131700000 "*" 6.24654217240561e-10 1.2462108191303e-09 -100
"chr6" 131755001 131756000 "*" 1.13140401492018e-08 1.87801380787728e-08 100
"chr6" 131880001 131881000 "*" 6.67924974218082e-08 9.96483280978404e-08
60.8695652173913
"chr6" 133038001 133039000 "*" 2.22044604925031e-15 8.90449334476539e-15
-60.3661327231121
"chr6" 133098001 133099000 "*" 3.95353005888666e-09 7.03928529283733e-09 -100
"chr6" 133561001 133562000 "*" 8.44985278414256e-08 1.23807313906787e-07
50.7631430186546
"chr6" 133585001 133586000 "*" 8.52140580320793e-12 2.19817259951489e-11 -100
"chr6" 134159001 134160000 "*" 0 0 -75
"chr6" 134175001 134176000 "*" 1.71473946153355e-12 4.84255946814781e-12
55.9475806451613
"chr6" 134216001 134217000 "*" 1.11577413974828e-13 3.65127289161778e-13 -71

"chr6" 134345001 134346000 "*" 8.88178419700125e-16 3.70670032207938e-15 -75
"chr6" 134363001 134364000 "*" 8.01666433236647e-09 1.36392368970109e-08 100
"chr6" 134629001 134630000 "*" 4.1582953203001e-07 5.55429664022256e-07
58.8235294117647
"chr6" 135168001 135169000 "*" 3.34128893442198e-08 5.19772864618523e-08 100
"chr6" 135577001 135578000 "*" 8.12683254025615e-13 2.39237407085304e-12
51.063829787234
"chr6" 136929001 136930000 "*" 8.97060203897126e-14 2.96780698277594e-13
-69.5652173913043
"chr6" 136978001 136979000 "*" 0 0 -100
"chr6" 137020001 137021000 "*" 4.18887147191072e-13 1.2739946038722e-12 -100
"chr6" 137365001 137366000 "*" 0 0 -62.6619282356987
"chr6" 137416001 137417000 "*" 2.15125472990962e-10 4.58126659943874e-10 -100
"chr6" 137663001 137664000 "*" 0 0 -100
"chr6" 138382001 138383000 "*" 8.71525074330748e-14 2.88848349331391e-13 -100
"chr6" 138429001 138430000 "*" 3.77475828372553e-15 1.47379557022071e-14 -100
"chr6" 138482001 138483000 "*" 1.88689429503341e-06 2.30468204378799e-06
-64.3260409315455
"chr6" 139018001 139019000 "*" 2.98824465383429e-06 3.54254647244229e-06
66.6666666666667
"chr6" 139310001 139311000 "*" 1.98365768255826e-11 4.85612402473442e-11 100
"chr6" 139450001 139451000 "*" 3.6700841921089e-08 5.65530226072258e-08 -100
"chr6" 139463001 139464000 "*" 6.70841160399505e-12 1.75486545176836e-11 -100
"chr6" 139942001 139943000 "*" 9.43689570931383e-15 3.51625644188498e-14 -100
"chr6" 140096001 140097000 "*" 2.73014943985572e-11 6.58595916221891e-11 100
"chr6" 141363001 141364000 "*" 1.29037891483108e-11 3.24582280286866e-11 -100
"chr6" 141395001 141396000 "*" 3.95353005888666e-09 7.03928529283733e-09 -100
"chr6" 143247001 143248000 "*" 0 0 -63.6377063102315
"chr6" 143268001 143269000 "*" 0 0 -67.6674364896074
"chr6" 143382001 143383000 "*" 0 0 -100
"chr6" 143740001 143741000 "*" 4.78841410966879e-11 1.11468187156588e-10 -100
"chr6" 143744001 143745000 "*" 3.61213425836837e-09 6.47511047170427e-09
-53.3333333333333
"chr6" 143832001 143833000 "*" 0 0 -84.8314606741573
"chr6" 144193001 144194000 "*" 1.20591092667155e-10 2.66075324129686e-10 -100
"chr6" 144284001 144285000 "*" 2.35592441111265e-08 3.73767311333139e-08
-63.6363636363636
"chr6" 144806001 144807000 "*" 3.33066907387547e-16 1.4495649018245e-15 100
"chr6" 145904001 145905000 "*" 1.98325800226939e-11 4.85612402473442e-11 -100
"chr6" 146588001 146589000 "*" 1.71500642316413e-05 1.8142382703915e-05
67.8571428571429
"chr6" 146687001 146688000 "*" 2.02327044007689e-12 5.65300384792621e-12 100
"chr6" 146864001 146865000 "*" 0 0 59.8329010671365
"chr6" 146865001 146866000 "*" 0 0 50.7159507159507
"chr6" 147178001 147179000 "*" 1.49875638566499e-08 2.45208190427346e-08
77.7777777777778
"chr6" 148014001 148015000 "*" 0 0 -100
"chr6" 148114001 148115000 "*" 3.6700841921089e-08 5.65530226072258e-08 -100
"chr6" 148500001 148501000 "*" 0 0 100
"chr6" 148646001 148647000 "*" 1.39779633423487e-07 1.98463037958589e-07 100
"chr6" 148703001 148704000 "*" 8.7349629751543e-09 1.47505871898511e-08 100
"chr6" 148765001 148766000 "*" 2.30404601619938e-07 3.18550210701194e-07 87.5

"chr6" 149041001 149042000 "*" 1.38590361409285e-10 3.02706178892116e-10 100
"chr6" 149794001 149795000 "*" 5.6621374255883e-15 2.16468580166064e-14 100
"chr6" 150167001 150168000 "*" 6.59976938922613e-05 6.36911591975017e-05
-63.3333333333333
"chr6" 150221001 150222000 "*" 4.73234496034536e-09 8.30627300264826e-09 -100
"chr6" 150244001 150245000 "*" 6.31460439493026e-11 1.44821231300727e-10
54.2222222222222
"chr6" 150260001 150261000 "*" 0 0 50.9433962264151
"chr6" 150262001 150263000 "*" 0 0 56.4834437086093
"chr6" 150311001 150312000 "*" 0 0 59.3763724198507
"chr6" 150312001 150313000 "*" 0 0 86.3636363636364
"chr6" 150326001 150327000 "*" 0 0 -71.4285714285714
"chr6" 150347001 150348000 "*" 0 0 -100
"chr6" 150373001 150374000 "*" 6.60582699651968e-14 2.22176301799e-13 100
"chr6" 150393001 150394000 "*" 9.12492303939416e-13 2.66483976917262e-12
-72.4137931034483
"chr6" 150420001 150421000 "*" 1.55431223447522e-15 6.33705141101682e-15 -100
"chr6" 150448001 150449000 "*" 2.11273934569434e-07 2.9348734792333e-07
-61.1111111111111
"chr6" 150472001 150473000 "*" 8.7349629751543e-09 1.47505871898511e-08 -100
"chr6" 150489001 150490000 "*" 9.62323387643238e-09 1.61671214954144e-08
-81.5533980582524
"chr6" 150522001 150523000 "*" 4.08188371991969e-10 8.36832789731227e-10
62.2434017595308
"chr6" 150532001 150533000 "*" 1.20591092667155e-10 2.66075324129686e-10 100
"chr6" 150552001 150553000 "*" 1.10170558231815e-08 1.83740321715113e-08
-69.2307692307692
"chr6" 150599001 150600000 "*" 1.5226708782734e-12 4.33226938571494e-12
-51.219512195122
"chr6" 150639001 150640000 "*" 1.45439216225896e-13 4.68981286729281e-13
-61.6666666666667
"chr6" 150665001 150666000 "*" 1.39779633423487e-07 1.98463037958589e-07 -100
"chr6" 150690001 150691000 "*" 1.83952852950142e-12 5.17499701329218e-12 100
"chr6" 150895001 150896000 "*" 4.71134242729931e-12 1.257615036806e-11 100
"chr6" 151029001 151030000 "*" 8.7349629751543e-09 1.47505871898511e-08 100
"chr6" 151042001 151043000 "*" 6.59550958292954e-09 1.13456194382773e-08 -100
"chr6" 151131001 151132000 "*" 0.000370088534552782 0.000315890305211296 -52.5
"chr6" 151134001 151135000 "*" 0 0 -100
"chr6" 151262001 151263000 "*" 7.86685565423717e-06 8.75794273207872e-06
-62.0689655172414
"chr6" 151271001 151272000 "*" 0.000241617096093272 0.000212792315955059
-51.1111111111111
"chr6" 151501001 151502000 "*" 3.88022947106492e-13 1.1867462770515e-12 -100
"chr6" 151641001 151642000 "*" 0 0 65.625
"chr6" 151646001 151647000 "*" 2.82079914981637e-11 6.79088459735627e-11
72.7272727272727
"chr6" 151997001 151998000 "*" 3.01170199890066e-12 8.2410199079151e-12
-53.3333333333333
"chr6" 152084001 152085000 "*" 7.0006535457523e-08 1.03695832512054e-07 100
"chr6" 152125001 152126000 "*" 0 0 98.989898989899
"chr6" 152265001 152266000 "*" 5.295763827462e-14 1.80214784640623e-13 100
"chr6" 152584001 152585000 "*" 4.10611433743213e-10 8.41448713890374e-10

51.3513513513514
"chr6" 152623001 152624000 "*" 0 0 100
"chr6" 152690001 152691000 "*" 6.09053945120897e-05 5.90954842827366e-05
-51.8518518518519
"chr6" 152699001 152700000 "*" 4.73234496034536e-09 8.30627300264826e-09 100
"chr6" 152702001 152703000 "*" 2.08814465718632e-09 3.86232567960463e-09 100
"chr6" 153305001 153306000 "*" 0 0 -100
"chr6" 153450001 153451000 "*" 0 0 79.6610169491525
"chr6" 153451001 153452000 "*" 0 0 71.3150538449232
"chr6" 153454001 153455000 "*" 7.41628980449605e-14 2.48193787819199e-13 -90
"chr6" 154178001 154179000 "*" 0 0 -50.8063492063492
"chr6" 154691001 154692000 "*" 5.23068752311673e-07 6.89690420948954e-07 75
"chr6" 154725001 154726000 "*" 0 0 -100
"chr6" 154764001 154765000 "*" 1.10822617749307e-09 2.13166437424721e-09
-51.0204081632653
"chr6" 154863001 154864000 "*" 9.46679348934332e-06 1.0416882274051e-05
66.6666666666667
"chr6" 155045001 155046000 "*" 4.48393544516534e-11 1.04769763310752e-10
-57.7777777777778
"chr6" 155314001 155315000 "*" 1.28785870856518e-14 4.71440608755782e-14 -100
"chr6" 155317001 155318000 "*" 0 0 90.4320987654321
"chr6" 155728001 155729000 "*" 0.000257691937994853 0.000225933396425958 52
"chr6" 155730001 155731000 "*" 0 0 -100
"chr6" 155743001 155744000 "*" 3.84137166520304e-14 1.32929369817675e-13 -100
"chr6" 155948001 155949000 "*" 2.69018141096922e-12 7.41487347763769e-12 100
"chr6" 156712001 156713000 "*" 2.22044604925031e-16 9.81641919380259e-16 100
"chr6" 156759001 156760000 "*" 9.61897228535236e-13 2.801372325071e-12 100
"chr6" 156830001 156831000 "*" 0 0 100
"chr6" 156965001 156966000 "*" 1.79306902658993e-10 3.86916446396834e-10 75
"chr6" 156987001 156988000 "*" 3.77475828372553e-15 1.47379557022071e-14 -100
"chr6" 157028001 157029000 "*" 1.38590361409285e-10 3.02706178892116e-10 100
"chr6" 157040001 157041000 "*" 0 0 -68.2926829268293
"chr6" 157091001 157092000 "*" 0 0 -100
"chr6" 157469001 157470000 "*" 0 0 -58.1081081081081
"chr6" 157704001 157705000 "*" 1.66528321210446e-06 2.04949060799586e-06
-61.1111111111111
"chr6" 157710001 157711000 "*" 1.309944922534e-07 1.87123731726588e-07
60.6060606060606
"chr6" 157881001 157882000 "*" 1.07792778293314e-05 1.17513174008031e-05
55.5555555555556
"chr6" 157912001 157913000 "*" 0 0 -100
"chr6" 157940001 157941000 "*" 2.38575481272818e-05 2.46816531377115e-05
-66.6666666666667
"chr6" 157980001 157981000 "*" 4.35292410995158e-05 4.32104485605312e-05 -56.25
"chr6" 157994001 157995000 "*" 1.24344978758018e-14 4.56627092294518e-14 -100
"chr6" 158024001 158025000 "*" 7.32747196252603e-15 2.76435639707144e-14
-99.009900990099
"chr6" 158060001 158061000 "*" 3.02845526434226e-11 7.24708136656982e-11 68
"chr6" 158063001 158064000 "*" 1.92946192356658e-09 3.59193011611483e-09 100
"chr6" 158110001 158111000 "*" 2.88769008705003e-13 8.97133044859218e-13
-71.4285714285714
"chr6" 158147001 158148000 "*" 6.59550958292954e-09 1.13456194382773e-08 -100

"chr6" 158195001 158196000 "*" 1.0769163338864e-14 3.98631098593833e-14 100
"chr6" 158395001 158396000 "*" 0 0 -100
"chr6" 158404001 158405000 "*" 0 0 -100
"chr6" 158407001 158408000 "*" 0.000370785441302535 0.000316420385049094
58.3333333333333
"chr6" 158411001 158412000 "*" 0 0 -56.4411919831224
"chr6" 158417001 158418000 "*" 2.18713935851156e-14 7.80893646430581e-14 100
"chr6" 158461001 158462000 "*" 3.0364599723498e-13 9.41948588486367e-13
92.7272727272727
"chr6" 158465001 158466000 "*" 6.98330282489223e-14 2.34267456154807e-13 100
"chr6" 158468001 158469000 "*" 0 0 -100
"chr6" 158630001 158631000 "*" 4.43201031430362e-13 1.34475479022741e-12
-93.7106918238994
"chr6" 158652001 158653000 "*" 0 0 100
"chr6" 158787001 158788000 "*" 5.10702591327572e-15 1.96271840288234e-14 100
"chr6" 158843001 158844000 "*" 3.26787774618253e-09 5.88938958107451e-09
-53.5714285714286
"chr6" 158957001 158958000 "*" 0 0 51.9398907103825
"chr6" 158970001 158971000 "*" 0 0 -100
"chr6" 158990001 158991000 "*" 0 0 100
"chr6" 159058001 159059000 "*" 6.59550958292954e-09 1.13456194382773e-08 100
"chr6" 159217001 159218000 "*" 3.6700841921089e-08 5.65530226072258e-08 -100
"chr6" 159259001 159260000 "*" 7.97140131680862e-14 2.65641277678906e-13 100
"chr6" 159290001 159291000 "*" 0 0 -55.5555555555556
"chr6" 159311001 159312000 "*" 7.54951656745106e-14 2.5237892556906e-13 -100
"chr6" 159331001 159332000 "*" 2.37587727269783e-14 8.44268514944447e-14
-98.3606557377049
"chr6" 159423001 159424000 "*" 0 0 -100
"chr6" 159488001 159489000 "*" 1.1534503225108e-05 1.25186825636811e-05
-67.741935483871
"chr6" 159489001 159490000 "*" 2.88779000712225e-11 6.92935809466068e-11 100
"chr6" 159521001 159522000 "*" 4.86753970463383e-11 1.13145908161915e-10 -100
"chr6" 159523001 159524000 "*" 1.39779633423487e-07 1.98463037958589e-07 -100
"chr6" 159554001 159555000 "*" 1.7531087692646e-11 4.33871904864699e-11
64.0350877192982
"chr6" 159583001 159584000 "*" 5.7863184284912e-05 5.63390103085987e-05 52
"chr6" 159588001 159589000 "*" 0.0003207101954813 0.000276619581787338
54.5454545454545
"chr6" 159636001 159637000 "*" 1.8223488384983e-08 2.94185709797487e-08
-51.5151515151515
"chr6" 159735001 159736000 "*" 1.08764344308732e-07 1.57082136228727e-07
63.8297872340426
"chr6" 159913001 159914000 "*" 1.12132525487141e-14 4.14352271980498e-14 100
"chr6" 159993001 159994000 "*" 1.17461596005342e-13 3.8279067575877e-13 -100
"chr6" 160018001 160019000 "*" 4.32209823486573e-12 1.15935290479641e-11 100
"chr6" 160266001 160267000 "*" 1.0325074129014e-14 3.83008663711937e-14 -100
"chr6" 160267001 160268000 "*" 0 0 100
"chr6" 160493001 160494000 "*" 2.18742150148721e-06 2.64736366894304e-06 60
"chr6" 160517001 160518000 "*" 0 0 -76.9230769230769
"chr6" 160544001 160545000 "*" 1.4432899320127e-15 5.90750815956055e-15 100
"chr6" 160545001 160546000 "*" 0 0 100
"chr6" 160548001 160549000 "*" 9.39988931469848e-07 1.19766766011864e-06

54.5454545454545
"chr6" 160558001 160559000 "*" 9.08108033215171e-11 2.03407753381119e-10 100
"chr6" 160683001 160684000 "*" 1.52794177310511e-09 2.87642564232045e-09 100
"chr6" 160721001 160722000 "*" 1.4122036873232e-13 4.55906060950416e-13
71.3675213675214
"chr6" 160767001 160768000 "*" 2.74151812362788e-11 6.61185397373184e-11
-85.7142857142857
"chr6" 160842001 160843000 "*" 8.3882012447134e-11 1.88922989503423e-10 100
"chr6" 160904001 160905000 "*" 4.93878095642053e-07 6.53262965576014e-07
67.3469387755102
"chr6" 160941001 160942000 "*" 0 0 81.8295739348371
"chr6" 161111001 161112000 "*" 1.67305924669847e-09 3.13863524858287e-09
-84.6153846153846
"chr6" 161161001 161162000 "*" 0 0 80.6451612903226
"chr6" 161178001 161179000 "*" 1.42219569454483e-13 4.58954156292913e-13
94.4444444444444
"chr6" 161186001 161187000 "*" 1.13140401492018e-08 1.87801380787728e-08 100
"chr6" 161260001 161261000 "*" 2.63843280556841e-10 5.55941322584367e-10
-58.5185185185185
"chr6" 161274001 161275000 "*" 1.98365768255826e-11 4.85612402473442e-11 100
"chr6" 161642001 161643000 "*" 3.72337605192286e-10 7.66708206534601e-10 -75
"chr6" 161788001 161789000 "*" 0 0 66.7992926613616
"chr6" 161797001 161798000 "*" 3.13178038940931e-05 3.18058313017091e-05
52.3809523809524
"chr6" 161897001 161898000 "*" 0 0 100
"chr6" 161920001 161921000 "*" 3.34128893442198e-08 5.19772864618523e-08 100
"chr6" 162103001 162104000 "*" 2.08224789632894e-08 3.32518143869947e-08
71.4285714285714
"chr6" 162223001 162224000 "*" 0 0 -91.6666666666667
"chr6" 162259001 162260000 "*" 0 0 100
"chr6" 162449001 162450000 "*" 0 0 97.0588235294118
"chr6" 162678001 162679000 "*" 9.71063229826541e-11 2.16781365050398e-10 100
"chr6" 162693001 162694000 "*" 6.92287338566189e-11 1.57605630124247e-10 -100
"chr6" 163269001 163270000 "*" 1.20281562487889e-12 3.46585342181251e-12 -100
"chr6" 163320001 163321000 "*" 2.88710388929303e-10 6.03399264731139e-10 100
"chr6" 163431001 163432000 "*" 1.49972523288966e-09 2.83643172292987e-09
-66.6666666666667
"chr6" 163442001 163443000 "*" 9.95295942662899e-07 1.26313797719273e-06
71.4285714285714
"chr6" 163560001 163561000 "*" 0 0 100
"chr6" 163571001 163572000 "*" 1.78767853690198e-05 1.88575738253536e-05
-57.1825764596849
"chr6" 163677001 163678000 "*" 1.97471879237465e-06 2.40413608358628e-06
-72.2222222222222
"chr6" 163680001 163681000 "*" 3.95353005888666e-09 7.03928529283733e-09 100
"chr6" 163720001 163721000 "*" 0 0 100
"chr6" 163753001 163754000 "*" 0 0 100
"chr6" 163757001 163758000 "*" 1.08811648136253e-07 1.57142290787618e-07
-77.3148148148148
"chr6" 163765001 163766000 "*" 1.44508516264352e-10 3.15079631238497e-10
-56.8783068783069
"chr6" 163794001 163795000 "*" 2.25375273998907e-14 8.03542019974573e-14

-68.1818181818182
"chr6" 163800001 163801000 "*" 9.41103768226093e-05 8.86297231089954e-05
-52.3809523809524
"chr6" 164041001 164042000 "*" 0 0 100
"chr6" 164063001 164064000 "*" 1.27098331859088e-12 3.65331361734749e-12
61.7647058823529
"chr6" 164088001 164089000 "*" 6.52733422867868e-12 1.71146951718423e-11 100
"chr6" 164127001 164128000 "*" 1.00808250635964e-13 3.31107370956809e-13 100
"chr6" 164143001 164144000 "*" 2.74225087082414e-14 9.66348222810204e-14
63.0252100840336
"chr6" 164151001 164152000 "*" 1.66339164664464e-11 4.128073042425e-11
73.015873015873
"chr6" 164169001 164170000 "*" 0 0 -89.0909090909091
"chr6" 164228001 164229000 "*" 0 0 -86.3636363636364
"chr6" 164288001 164289000 "*" 0 0 75
"chr6" 164380001 164381000 "*" 4.33618629891441e-09 7.66350836024395e-09
58.974358974359
"chr6" 164442001 164443000 "*" 6.59550958292954e-09 1.13456194382773e-08 -100
"chr6" 164512001 164513000 "*" 4.02167188440217e-12 1.08266700497926e-11 -100
"chr6" 165339001 165340000 "*" 3.33066907387547e-16 1.4495649018245e-15 -100
"chr6" 165711001 165712000 "*" 0 0 100
"chr6" 166035001 166036000 "*" 3.88022947106492e-13 1.1867462770515e-12 100
"chr6" 166037001 166038000 "*" 4.99020824662466e-11 1.15790792586157e-10 100
"chr6" 166047001 166048000 "*" 0 0 100
"chr6" 166090001 166091000 "*" 1.67299207820548e-08 2.71377429441055e-08 -100
"chr6" 166206001 166207000 "*" 6.4152538836737e-10 1.27477697728417e-09 -100
"chr6" 166219001 166220000 "*" 0 0 73.9130434782609
"chr6" 166220001 166221000 "*" 5.25841424847684e-08 7.95060575196433e-08
60.6060606060606
"chr6" 166228001 166229000 "*" 0 0 -99.3464052287582
"chr6" 166239001 166240000 "*" 3.73034936274053e-14 1.29420415488591e-13 100
"chr6" 166251001 166252000 "*" 5.55111512312578e-16 2.36485094870365e-15 100
"chr6" 166259001 166260000 "*" 1.60460096768622e-05 1.70453625649539e-05
-68.5714285714286
"chr6" 166263001 166264000 "*" 2.77555756156289e-15 1.10015415195978e-14
87.7551020408163
"chr6" 166321001 166322000 "*" 8.7349629751543e-09 1.47505871898511e-08 100
"chr6" 166390001 166391000 "*" 5.74788266005655e-06 6.53227345745443e-06
58.4905660377358
"chr6" 166400001 166401000 "*" 0 0 60
"chr6" 166405001 166406000 "*" 7.68773620318797e-07 9.91437385905438e-07 52
"chr6" 166487001 166488000 "*" 3.33408433084159e-08 5.19772864618523e-08 60
"chr6" 166511001 166512000 "*" 1.11250002854391e-06 1.40327708766571e-06 -52
"chr6" 166512001 166513000 "*" 1.18793863634892e-14 4.37320283507971e-14
94.4444444444444
"chr6" 166575001 166576000 "*" 5.08482145278322e-14 1.73432706170696e-13 -100
"chr6" 166582001 166583000 "*" 0 0 54.6736396133986
"chr6" 166703001 166704000 "*" 1.07882591748876e-11 2.74553537455803e-11 -100
"chr6" 166751001 166752000 "*" 1.93720595120794e-11 4.76309924836636e-11 100
"chr6" 166820001 166821000 "*" 3.04778453030785e-07 4.14668048576323e-07
64.8648648648649
"chr6" 166845001 166846000 "*" 0 0 -100

"chr6" 166882001 166883000 "*" 5.81945766020553e-08 8.75397840749566e-08
77.7777777777778
"chr6" 166887001 166888000 "*" 1.13140401492018e-08 1.87801380787728e-08 100
"chr6" 166965001 166966000 "*" 6.88338275267597e-15 2.60518659957509e-14 100
"chr6" 166974001 166975000 "*" 4.14335232790108e-13 1.26153063645578e-12 100
"chr6" 167026001 167027000 "*" 1.12843068222901e-12 3.259512533505e-12 100
"chr6" 167034001 167035000 "*" 2.82773804372027e-13 8.79129664109438e-13 -100
"chr6" 167039001 167040000 "*" 5.55111512312578e-16 2.36485094870365e-15
61.2903225806452
"chr6" 167042001 167043000 "*" 0 0 100
"chr6" 167050001 167051000 "*" 2.68837461181448e-08 4.23761701646127e-08
79.0697674418605
"chr6" 167077001 167078000 "*" 1.14130926931466e-13 3.7276674571693e-13 100
"chr6" 167099001 167100000 "*" 4.91828799908944e-14 1.68192819018269e-13 100
"chr6" 167101001 167102000 "*" 0 0 62.0029455081001
"chr6" 167103001 167104000 "*" 9.08108033215171e-11 2.03407753381119e-10 100
"chr6" 167109001 167110000 "*" 1.11022302462516e-16 5.03662826618488e-16 100
"chr6" 167126001 167127000 "*" 3.72875064158507e-11 8.79967182610069e-11
-69.8412698412698
"chr6" 167169001 167170000 "*" 0 0 100
"chr6" 167189001 167190000 "*" 0 0 -53.5211267605634
"chr6" 167234001 167235000 "*" 3.90831811358794e-12 1.05610567215048e-11 100
"chr6" 167237001 167238000 "*" 3.05311331771918e-12 8.34639368772922e-12 100
"chr6" 167276001 167277000 "*" 0 0 88.8308977035491
"chr6" 167321001 167322000 "*" 8.46418490851875e-10 1.65844924386433e-09
56.6666666666667
"chr6" 167364001 167365000 "*" 0 0 -50.7936507936508
"chr6" 167436001 167437000 "*" 1.1191541582356e-08 1.86478806092309e-08
54.8387096774194
"chr6" 167492001 167493000 "*" 8.7349629751543e-09 1.47505871898511e-08 100
"chr6" 167535001 167536000 "*" 3.6892711108294e-13 1.13266255090498e-12 -100
"chr6" 167547001 167548000 "*" 2.43005615629954e-09 4.45014222280278e-09 -100
"chr6" 167558001 167559000 "*" 6.84288148367074e-09 1.17505576721128e-08 -75
"chr6" 167652001 167653000 "*" 1.5277158427196e-09 2.87642564232045e-09 100
"chr6" 167670001 167671000 "*" 1.67299207820548e-08 2.71377429441055e-08 -100
"chr6" 167701001 167702000 "*" 2.22044604925031e-16 9.81641919380259e-16 -100
"chr6" 167739001 167740000 "*" 6.60833471228806e-05 6.37657026117485e-05
57.1428571428571
"chr6" 167744001 167745000 "*" 0 0 -100
"chr6" 167745001 167746000 "*" 2.455369241261e-12 6.8003688121118e-12 100
"chr6" 167780001 167781000 "*" 2.00227675550835e-08 3.20459176617961e-08 -100
"chr6" 167791001 167792000 "*" 1.52351253634997e-06 1.88642448146651e-06
53.5714285714286
"chr6" 167809001 167810000 "*" 9.34219368531330e-12 2.40010308530028e-11
52.49343832021
"chr6" 167814001 167815000 "*" 6.53492593372107e-10 1.29735152943986e-09
-63.3802816901408
"chr6" 167818001 167819000 "*" 3.33066907387547e-14 1.16191961407382e-13
65.7894736842105
"chr6" 167823001 167824000 "*" 8.00762828668811e-05 7.62332949242677e-05
52.1739130434783
"chr6" 167826001 167827000 "*" 0 0 67.7083333333333

"chr6" 167905001 167906000 "*" 1.90115722165096e-07 2.65666667785906e-07
-57.8947368421053
"chr6" 167922001 167923000 "*" 2.69007038866675e-13 8.38831022628993e-13 100
"chr6" 168049001 168050000 "*" 0 0 100
"chr6" 168062001 168063000 "*" 0 0 -66.0955236667381
"chr6" 168085001 168086000 "*" 1.70641278884887e-12 4.81986032810646e-12 100
"chr6" 168152001 168153000 "*" 3.11972669919669e-14 1.09125269919044e-13
63.4146341463415
"chr6" 168157001 168158000 "*" 2.51385057215003e-09 4.59673075630309e-09
-53.125
"chr6" 168205001 168206000 "*" 0 0 89.5833333333333
"chr6" 168380001 168381000 "*" 1.68753899743024e-14 6.09826805967439e-14 100
"chr6" 168400001 168401000 "*" 3.14848147553448e-12 8.59667865299575e-12
-75.8620689655172
"chr6" 168403001 168404000 "*" 3.3744118610457e-12 9.1855963533528e-12
-66.6666666666667
"chr6" 168462001 168463000 "*" 0 0 61.7769472521872
"chr6" 168470001 168471000 "*" 0 0 -100
"chr6" 168471001 168472000 "*" 2.70339306496226e-13 8.42355472617862e-13 -100
"chr6" 168472001 168473000 "*" 0 0 -58.3977819932876
"chr6" 168497001 168498000 "*" 0 0 58.8929588929589
"chr6" 168504001 168505000 "*" 1.66533453693773e-15 6.7629186866784e-15 -100
"chr6" 168514001 168515000 "*" 3.88022947106492e-13 1.1867462770515e-12 100
"chr6" 168528001 168529000 "*" 0 0 -100
"chr6" 168551001 168552000 "*" 0 0 74.5454545454545
"chr6" 168559001 168560000 "*" 0 0 -62.8522022174753
"chr6" 168601001 168602000 "*" 5.08482145278322e-14 1.73432706170696e-13 -100
"chr6" 168607001 168608000 "*" 1.17461596005342e-13 3.8279067575877e-13 -100
"chr6" 168645001 168646000 "*" 0 0 -57.8947368421053
"chr6" 168656001 168657000 "*" 1.13328566130333e-05 1.23144129895198e-05
69.2307692307692
"chr6" 168666001 168667000 "*" 0 0 94.2857142857143
"chr6" 168716001 168717000 "*" 1.12055920098442e-11 2.84500040664577e-11 100
"chr6" 168720001 168721000 "*" 0 0 53.5101696988967
"chr6" 168740001 168741000 "*" 0 0 100
"chr6" 168748001 168749000 "*" 0 0 54.6099290780142
"chr6" 168756001 168757000 "*" 0 0 84.6153846153846
"chr6" 168791001 168792000 "*" 2.08814465718632e-09 3.86232567960463e-09 -100
"chr6" 168817001 168818000 "*" 2.89560758280905e-05 2.95664357493375e-05
50.817341862118
"chr6" 168828001 168829000 "*" 1.74853465040314e-11 4.32752710900609e-11 100
"chr6" 168829001 168830000 "*" 6.52733422867868e-12 1.71146951718423e-11 -100
"chr6" 168842001 168843000 "*" 0 0 54.6833976833977
"chr6" 168849001 168850000 "*" 9.51239198521137e-10 1.85039912631335e-09
-82.6923076923077
"chr6" 168863001 168864000 "*" 1.48087875295744e-10 3.22075573380618e-10 100
"chr6" 168865001 168866000 "*" 0 0 -100
"chr6" 168874001 168875000 "*" 0.000168529599185452 0.000152316702506419 60
"chr6" 168902001 168903000 "*" 4.2708164887939e-06 4.94144268742661e-06
54.5454545454545
"chr6" 168916001 168917000 "*" 1.08473570126222e-08 1.81076666826488e-08
55.1020408163265

"chr6" 168927001 168928000 "*" 1.11022302462516e-16 5.03662826618488e-16 -100
"chr6" 168996001 168997000 "*" 4.69269068048561e-12 1.254241999153e-11 56.3025210084034
"chr6" 168997001 168998000 "*" 0 0 -100
"chr6" 169035001 169036000 "*" 3.40125705378114e-10 7.03784073980162e-10 -100
"chr6" 169039001 169040000 "*" 4.30888397962015e-07 5.74421647770785e-07 -53.3333333333333
"chr6" 169100001 169101000 "*" 6.66133814775094e-16 2.81595744474255e-15 -56.5217391304348
"chr6" 169116001 169117000 "*" 4.44089209850063e-16 1.91071758245033e-15 100
"chr6" 169119001 169120000 "*" 6.24145735095283e-09 1.0801637484149e-08 59.375
"chr6" 169190001 169191000 "*" 6.46149800331841e-14 2.17664365839349e-13 100
"chr6" 169249001 169250000 "*" 3.43701215632652e-06 4.03606219665671e-06 56.7567567567568
"chr6" 169272001 169273000 "*" 1.07309516828025e-09 2.06752338922509e-09 69.4444444444444
"chr6" 169283001 169284000 "*" 0 0 -100
"chr6" 169295001 169296000 "*" 0 0 63.3802816901408
"chr6" 169315001 169316000 "*" 8.80406858527749e-14 2.91456141063452e-13 100
"chr6" 169364001 169365000 "*" 3.47943895917524e-13 1.07191058753964e-12 -66.6666666666667
"chr6" 169392001 169393000 "*" 0 0 80
"chr6" 169440001 169441000 "*" 1.04296238312429e-10 2.32074356782748e-10 95.6521739130435
"chr6" 169458001 169459000 "*" 1.11022302462516e-16 5.03662826618488e-16 52.1739130434783
"chr6" 169484001 169485000 "*" 0 0 100
"chr6" 169525001 169526000 "*" 3.5527136788005e-15 1.39277426410519e-14 100
"chr6" 169540001 169541000 "*" 7.51509965368768e-13 2.21981423380301e-12 56.9047619047619
"chr6" 169563001 169564000 "*" 4.73234496034536e-09 8.30627300264826e-09 100
"chr6" 169565001 169566000 "*" 6.05956069050784e-05 5.88243301190207e-05 66
"chr6" 169572001 169573000 "*" 1.11022302462516e-16 5.03662826618488e-16 69.2307692307692
"chr6" 169623001 169624000 "*" 9.23017218212863e-12 2.37256608870072e-11 -52.3809523809524
"chr6" 169628001 169629000 "*" 0 0 100
"chr6" 169629001 169630000 "*" 3.33066907387547e-16 1.4495649018245e-15 -100
"chr6" 169645001 169646000 "*" 2.22044604925031e-16 9.81641919380259e-16 -100
"chr6" 169647001 169648000 "*" 8.67084182232247e-14 2.87708076903e-13 -69.0909090909091
"chr6" 169683001 169684000 "*" 4.44089209850063e-15 1.71914916534614e-14 100
"chr6" 169689001 169690000 "*" 4.55191440096314e-15 1.76090186737198e-14 -60.2739726027397
"chr6" 169732001 169733000 "*" 2.68450373042128e-10 5.64465213127486e-10 100
"chr6" 169789001 169790000 "*" 7.86685607689908e-06 8.75794273207872e-06 62.0689655172414
"chr6" 169798001 169799000 "*" 1.66533453693773e-15 6.7629186866784e-15 77.9411764705882
"chr6" 169823001 169824000 "*" 2.59296883964311e-09 4.73475956664494e-09 -84.6153846153846
"chr6" 169828001 169829000 "*" 1.19249055074988e-12 3.43835931071107e-12

75.3086419753086
"chr6" 169845001 169846000 "*" 0 0 69.4444444444444
"chr6" 169981001 169982000 "*" 2.56461518688411e-14 9.06700695401459e-14 90
"chr6" 170199001 170200000 "*" 9.51329330867168e-08 1.38406505437483e-07
72.2222222222222
"chr6" 170205001 170206000 "*" 2.1094237467878e-15 8.47434540879246e-15 100
"chr6" 170226001 170227000 "*" 6.43596287375203e-13 1.91250548472177e-12 75
"chr6" 170227001 170228000 "*" 1.29037891483108e-11 3.24582280286866e-11 -100
"chr6" 170228001 170229000 "*" 5.48137975087126e-05 5.35512424681957e-05
50.7246376811594
"chr6" 170229001 170230000 "*" 1.03143049656751e-11 2.63507496674929e-11
-81.8181818181818
"chr6" 170260001 170261000 "*" 2.62012633811537e-14 9.25110548865337e-14
78.5714285714286
"chr6" 170348001 170349000 "*" 1.11022302462516e-16 5.03662826618488e-16
-64.2857142857143
"chr6" 170369001 170370000 "*" 0 0 100
"chr6" 170458001 170459000 "*" 7.97140131680862e-14 2.65641277678906e-13 100
"chr6" 170494001 170495000 "*" 0 0 50.4817683733233
"chr6" 170495001 170496000 "*" 4.21884749357559e-15 1.63874445239189e-14 -100
"chr6" 170496001 170497000 "*" 0 0 100
"chr6" 170499001 170500000 "*" 3.01743863906356e-06 3.57481852520308e-06
66.6666666666667
"chr6" 170508001 170509000 "*" 0 0 58.3333333333333
"chr6" 170562001 170563000 "*" 0 0 59.8260033042642
"chr6" 170574001 170575000 "*" 0 0 51.4851485148515
"chr6" 170578001 170579000 "*" 1.55764290354909e-13 5.00572224822352e-13
59.9629040805511
"chr6" 170589001 170590000 "*" 0 0 100
"chr6" 170598001 170599000 "*" 0 0 64.7473508457634
"chr6" 170606001 170607000 "*" 0 0 58.840140738559
"chr6" 170753001 170754000 "*" 1.15739173800478e-09 2.21697927461109e-09
-78.5714285714286
"chr6" 170800001 170801000 "*" 0 0 100
"chr6" 170805001 170806000 "*" 1.11022302462516e-16 5.03662826618488e-16 100
"chr6_ssto_hap7" 3901001 3902000 "*" 5.6362137179633e-11 1.29963270300065e-10
-100
"chr7" 41001 42000 "*" 1.67334371914407e-08 2.71418641479927e-08
76.4705882352941
"chr7" 44001 45000 "*" 0 0 100
"chr7" 61001 62000 "*" 2.68673971959288e-14 9.47266815431045e-14 -100
"chr7" 66001 67000 "*" 0 0 -92.3076923076923
"chr7" 87001 88000 "*" 2.70572986238449e-09 4.91922822432777e-09
77.2247360482655
"chr7" 123001 124000 "*" 1.37923006349183e-12 3.94487974816619e-12
57.5757575757576
"chr7" 124001 125000 "*" 0 0 -100
"chr7" 138001 139000 "*" 2.88710388929303e-10 6.03399264731139e-10 -100
"chr7" 166001 167000 "*" 0 0 -96.551724137931
"chr7" 167001 168000 "*" 0 0 -58.974358974359
"chr7" 190001 191000 "*" 2.048838876334e-11 5.00907543355513e-11
-52.3628435249975

"chr7" 289001 290000 "*" 3.70481423317415e-13 1.13726706610467e-12
66.6666666666667
"chr7" 310001 311000 "*" 9.23995291390867e-09 1.55576789814298e-08
-51.6129032258064
"chr7" 356001 357000 "*" 2.52364795727544e-12 6.97973797190925e-12
-65.7894736842105
"chr7" 362001 363000 "*" 0 0 -61.6504854368932
"chr7" 369001 370000 "*" 0 0 -100
"chr7" 370001 371000 "*" 2.65132586527717e-05 2.72257904722968e-05
53.5211267605634
"chr7" 394001 395000 "*" 0 0 -53.903743315508
"chr7" 407001 408000 "*" 1.5277158427196e-09 2.87642564232045e-09 100
"chr7" 413001 414000 "*" 0 0 73.6486486486486
"chr7" 422001 423000 "*" 1.37347001039068e-06 1.71182312352142e-06
-76.9230769230769
"chr7" 445001 446000 "*" 1.18504552837351e-08 1.96274997248588e-08
85.3333333333333
"chr7" 448001 449000 "*" 3.81840004060052e-10 7.8526711223357e-10
-90.5882352941177
"chr7" 454001 455000 "*" 1.03834741915776e-08 1.73750322935822e-08
-72.6190476190476
"chr7" 484001 485000 "*" 1.88737914186277e-15 7.62011598380321e-15 -100
"chr7" 496001 497000 "*" 2.88657986402541e-15 1.14155613124573e-14
-74.3801652892562
"chr7" 508001 509000 "*" 2.72777440635252e-08 4.29598698288959e-08
64.7058823529412
"chr7" 513001 514000 "*" 3.88578058618805e-15 1.5152981210988e-14 -65
"chr7" 514001 515000 "*" 0 0 -100
"chr7" 517001 518000 "*" 0 0 -88.4146341463415
"chr7" 554001 555000 "*" 0 0 -65.5105973025048
"chr7" 575001 576000 "*" 5.44086738047955e-05 5.31828617901015e-05
-58.1818181818182
"chr7" 578001 579000 "*" 9.35164168325286e-11 2.09181556710247e-10
73.0769230769231
"chr7" 583001 584000 "*" 0 0 -61.1111111111111
"chr7" 767001 768000 "*" 4.52848869514355e-12 1.21190833459878e-11
-77.0491803278689
"chr7" 771001 772000 "*" 0 0 -53.5826032540676
"chr7" 772001 773000 "*" 1.11022302462516e-15 4.59817122935606e-15
-68.6746987951807
"chr7" 845001 846000 "*" 5.30653299080086e-12 1.40721319531155e-11
64.5161290322581
"chr7" 871001 872000 "*" 1.21930932719394e-05 1.31814908822931e-05
-55.5555555555556
"chr7" 941001 942000 "*" 3.96349619791181e-14 1.36905506457895e-13
-54.9114331723027
"chr7" 1015001 1016000 "*" 6.01507510467059e-10 1.20455604498427e-09
-50.6830601092896
"chr7" 1126001 1127000 "*" 0 0 -57.5136612021858
"chr7" 1178001 1179000 "*" 1.08922989516813e-07 1.57297638899522e-07
74.6478873239437
"chr7" 1224001 1225000 "*" 4.88154529021401e-08 7.41479737543463e-08

77.7777777777778
"chr7" 1257001 1258000 "*" 8.80406858527749e-14 2.91456141063452e-13 100
"chr7" 1335001 1336000 "*" 9.11646313994652e-11 2.04134505657007e-10
-54.4891640866873
"chr7" 1342001 1343000 "*" 4.04835942546811e-10 8.30143581992234e-10
52.6315789473684
"chr7" 1352001 1353000 "*" 2.39311903627026e-11 5.81295458151708e-11
-87.8048780487805
"chr7" 1356001 1357000 "*" 2.37587727269783e-14 8.44268514944447e-14 -100
"chr7" 1372001 1373000 "*" 0 0 50.2613042490343
"chr7" 1381001 1382000 "*" 0 0 65.7142857142857
"chr7" 1382001 1383000 "*" 0 0 81.8181818181818
"chr7" 1402001 1403000 "*" 0.000194421313536641 0.000173919609733995
-57.4279379157428
"chr7" 1406001 1407000 "*" 1.53486647203049e-06 1.89963692046958e-06
-52.6315789473684
"chr7" 1410001 1411000 "*" 2.52225684782559e-10 5.32659726135026e-10
74.3589743589744
"chr7" 1411001 1412000 "*" 1.34481314972845e-12 3.85582900100956e-12
-61.7486338797814
"chr7" 1472001 1473000 "*" 0 0 66.839378238342
"chr7" 1608001 1609000 "*" 3.33035407029669e-08 5.19263134452441e-08
-80.327868852459
"chr7" 1628001 1629000 "*" 0 0 -100
"chr7" 1630001 1631000 "*" 2.64951482975562e-09 4.82186011375752e-09 100
"chr7" 1635001 1636000 "*" 0 0 70.5882352941177
"chr7" 1646001 1647000 "*" 3.6892711108294e-13 1.13266255090498e-12 -100
"chr7" 1652001 1653000 "*" 4.32209823486573e-12 1.15935290479641e-11 -100
"chr7" 1661001 1662000 "*" 2.79869036612368e-07 3.82788144935124e-07
-53.8461538461538
"chr7" 1704001 1705000 "*" 0 0 55.2076154542129
"chr7" 1715001 1716000 "*" 0 0 52.2463385670933
"chr7" 1743001 1744000 "*" 0 0 92.5
"chr7" 1750001 1751000 "*" 6.30384633382164e-13 1.87665599796749e-12 -100
"chr7" 1761001 1762000 "*" 1.30329413927655e-10 2.86184248893354e-10 60
"chr7" 1774001 1775000 "*" 0 0 100
"chr7" 1781001 1782000 "*" 3.21964677141295e-15 1.26720521345953e-14
-72.2222222222222
"chr7" 2282001 2283000 "*" 1.55553347980231e-12 4.41702959978244e-12
-56.6666666666667
"chr7" 2292001 2293000 "*" 1.60349511446611e-12 4.54254789857261e-12
-62.962962962963
"chr7" 2356001 2357000 "*" 1.67299207820548e-08 2.71377429441055e-08 -100
"chr7" 2432001 2433000 "*" 2.64951482975562e-09 4.82186011375752e-09 -100
"chr7" 2469001 2470000 "*" 7.67056418382595e-11 1.7359448580318e-10 -100
"chr7" 2497001 2498000 "*" 0 0 78.4
"chr7" 2528001 2529000 "*" 9.4014351859073e-10 1.83013852209252e-09
52.0408163265306
"chr7" 2595001 2596000 "*" 0 0 -68.9655172413793
"chr7" 2652001 2653000 "*" 0 0 -86.2068965517241
"chr7" 2672001 2673000 "*" 0 0 -62.9247311827957
"chr7" 2673001 2674000 "*" 0 0 -99.21875

"chr7" 2714001 2715000 "*" 0 0 -100
"chr7" 2720001 2721000 "*" 0 0 53.9855072463768
"chr7" 2726001 2727000 "*" 1.22457599616155e-12 3.52441478267078e-12 -100
"chr7" 2729001 2730000 "*" 0 0 62.6666666666667
"chr7" 2730001 2731000 "*" 9.65110769080013e-09 1.621199033097e-08 -72.5
"chr7" 2746001 2747000 "*" 5.22804022295986e-13 1.57329219304489e-12 -75
"chr7" 2750001 2751000 "*" 7.65537615521339e-07 9.87630651953536e-07 -71.4285714285714
"chr7" 2803001 2804000 "*" 8.18289880299972e-12 2.11812372254089e-11 52.5675675675676
"chr7" 2844001 2845000 "*" 6.24654217240561e-10 1.2462108191303e-09 -100
"chr7" 2889001 2890000 "*" 1.74853465040314e-11 4.32752710900609e-11 100
"chr7" 2903001 2904000 "*" 0 0 -100
"chr7" 2926001 2927000 "*" 3.6700841921089e-08 5.65530226072258e-08 100
"chr7" 2934001 2935000 "*" 0 0 55.5452003727866
"chr7" 2954001 2955000 "*" 0 0 -58.8390911920324
"chr7" 2961001 2962000 "*" 1.22124532708767e-15 5.03921984217778e-15 -62.5874125874126
"chr7" 2965001 2966000 "*" 5.47961676033992e-12 1.45126661460521e-11 -65.9574468085106
"chr7" 2969001 2970000 "*" 4.74065231514942e-14 1.62745413342582e-13 -88
"chr7" 3015001 3016000 "*" 0 0 -100
"chr7" 3035001 3036000 "*" 1.52794177310511e-09 2.87642564232045e-09 100
"chr7" 3038001 3039000 "*" 3.44722487444216e-07 4.6586018916251e-07 -80
"chr7" 3054001 3055000 "*" 0 0 -100
"chr7" 3058001 3059000 "*" 1.15463194561016e-14 4.25829614430727e-14 100
"chr7" 3118001 3119000 "*" 9.56739454327504e-09 1.60781690395183e-08 -66.6666666666667
"chr7" 3141001 3142000 "*" 0 0 -93.9393939393939
"chr7" 3157001 3158000 "*" 4.73234496034536e-09 8.30627300264826e-09 -100
"chr7" 3169001 3170000 "*" 5.8035102279419e-09 1.00847337822923e-08 65.7142857142857
"chr7" 3185001 3186000 "*" 1.48087875295744e-10 3.22075573380618e-10 100
"chr7" 3189001 3190000 "*" 1.54529956475358e-06 1.9118118543793e-06 -75.5555555555556
"chr7" 3215001 3216000 "*" 7.86622250670277e-08 1.15684789204325e-07 -92.3076923076923
"chr7" 3237001 3238000 "*" 2.88710388929303e-10 6.03399264731139e-10 100
"chr7" 3255001 3256000 "*" 6.59550958292954e-09 1.13456194382773e-08 -100
"chr7" 3319001 3320000 "*" 0 0 -98.4375
"chr7" 3331001 3332000 "*" 0 0 -100
"chr7" 3339001 3340000 "*" 2.22044604925031e-16 9.81641919380259e-16 -52.3809523809524
"chr7" 3418001 3419000 "*" 4.77395900588817e-15 1.84201484337652e-14 -70.5035971223022
"chr7" 3420001 3421000 "*" 5.52733414593831e-11 1.27671509722674e-10 -85
"chr7" 3496001 3497000 "*" 7.93809462606987e-14 2.64679416473686e-13 -100
"chr7" 3501001 3502000 "*" 0 0 -100
"chr7" 3584001 3585000 "*" 0 0 -100
"chr7" 3591001 3592000 "*" 4.08209022140227e-11 9.57770690255255e-11 -100
"chr7" 3655001 3656000 "*" 4.99900121297969e-12 1.32921056339624e-11 -100
"chr7" 3666001 3667000 "*" 0 0 -100

"chr7" 3681001 3682000 "*" 3.92027021955954e-08 6.02223699362347e-08 82.5
"chr7" 3697001 3698000 "*" 1.98365768255826e-11 4.85612402473442e-11 -100
"chr7" 3699001 3700000 "*" 1.38444811170757e-13 4.47326582344615e-13
-94.3502824858757
"chr7" 3776001 3777000 "*" 2.73850603860204e-07 3.75017753563321e-07
-57.1428571428571
"chr7" 3840001 3841000 "*" 2.08814465718632e-09 3.86232567960463e-09 -100
"chr7" 3920001 3921000 "*" 5.58442181386454e-14 1.89404095749931e-13
-69.2307692307692
"chr7" 3922001 3923000 "*" 1.00808250635964e-13 3.31107370956809e-13 -100
"chr7" 3940001 3941000 "*" 1.66533453693773e-15 6.7629186866784e-15 -100
"chr7" 3976001 3977000 "*" 2.98943036991517e-09 5.41025055645908e-09
-85.1851851851852
"chr7" 3980001 3981000 "*" 0 0 -93.0232558139535
"chr7" 3996001 3997000 "*" 0 0 -52.212389380531
"chr7" 4000001 4001000 "*" 2.90172342076733e-06 3.44781746527743e-06 -60
"chr7" 4003001 4004000 "*" 0 0 -100
"chr7" 4005001 4006000 "*" 0 0 -82.1727019498607
"chr7" 4029001 4030000 "*" 6.35269614690515e-13 1.89059122997644e-12
-90.4761904761905
"chr7" 4031001 4032000 "*" 6.92287338566189e-11 1.57605630124247e-10 -100
"chr7" 4033001 4034000 "*" 1.92946192356658e-09 3.59193011611483e-09 -100
"chr7" 4034001 4035000 "*" 4.44089209850063e-15 1.71914916534614e-14 -100
"chr7" 4062001 4063000 "*" 1.20159437955181e-12 3.46389024677651e-12
61.8556701030928
"chr7" 4091001 4092000 "*" 0 0 -67.1232876712329
"chr7" 4093001 4094000 "*" 3.13297928711265e-08 4.89862497444354e-08
-81.8181818181818
"chr7" 4107001 4108000 "*" 2.00227675550835e-08 3.20459176617961e-08 -100
"chr7" 4116001 4117000 "*" 0 0 -100
"chr7" 4149001 4150000 "*" 0 0 -100
"chr7" 4182001 4183000 "*" 5.65727885426526e-07 7.43212462033977e-07
-61.2903225806452
"chr7" 4190001 4191000 "*" 1.78535778716515e-07 2.50302722885035e-07 -65
"chr7" 4202001 4203000 "*" 2.62012633811537e-14 9.25110548865337e-14
-84.7058823529412
"chr7" 4207001 4208000 "*" 0 0 81.9277108433735
"chr7" 4209001 4210000 "*" 9.88098491916389e-15 3.67401261259622e-14
-65.3968253968254
"chr7" 4216001 4217000 "*" 0.000159640753977897 0.000144832370404278
-55.5555555555556
"chr7" 4217001 4218000 "*" 0 0 92.2077922077922
"chr7" 4222001 4223000 "*" 1.37828859436695e-10 3.01445544267661e-10 -100
"chr7" 4265001 4266000 "*" 0 0 -72.5098039215686
"chr7" 4266001 4267000 "*" 0 0 -60
"chr7" 4301001 4302000 "*" 0 0 -100
"chr7" 4309001 4310000 "*" 0.00016961084973377 0.000153222593791434
-51.7241379310345
"chr7" 4318001 4319000 "*" 1.39779633423487e-07 1.98463037958589e-07 -100
"chr7" 4354001 4355000 "*" 6.59550958292954e-09 1.13456194382773e-08 100
"chr7" 4376001 4377000 "*" 6.93112234273485e-13 2.05218842007357e-12 -100
"chr7" 4408001 4409000 "*" 3.5613175242144e-07 4.80158368901456e-07

66.6666666666667
"chr7" 4489001 4490000 "*" 1.29037891483108e-11 3.24582280286866e-11 -100
"chr7" 4493001 4494000 "*" 0 0 80
"chr7" 4512001 4513000 "*" 8.65635896296624e-09 1.46719174639529e-08
55.6701030927835
"chr7" 4562001 4563000 "*" 1.96859348933209e-07 2.74616246379259e-07 60
"chr7" 4671001 4672000 "*" 0 0 -94.1860465116279
"chr7" 4788001 4789000 "*" 2.56593635228342e-11 6.2140914153063e-11
64.7058823529412
"chr7" 4820001 4821000 "*" 0 0 50.2222222222222
"chr7" 4847001 4848000 "*" 1.90936544353093e-09 3.56039906900536e-09
83.3333333333333
"chr7" 4862001 4863000 "*" 0 0 54.6218487394958
"chr7" 4890001 4891000 "*" 0 0 -100
"chr7" 4906001 4907000 "*" 3.65831983661913e-05 3.6733698352541e-05 65.625
"chr7" 4942001 4943000 "*" 1.37001521238744e-13 4.42851918810123e-13 100
"chr7" 4950001 4951000 "*" 0 0 -100
"chr7" 4960001 4961000 "*" 6.92287338566189e-11 1.57605630124247e-10 100
"chr7" 4987001 4988000 "*" 1.07528297377257e-09 2.07126012084627e-09
-54.1666666666667
"chr7" 5001001 5002000 "*" 3.71042809810262e-08 5.7138795641956e-08
55.5555555555556
"chr7" 5067001 5068000 "*" 9.44810896186254e-12 2.42581926689337e-11
53.2808398950131
"chr7" 5072001 5073000 "*" 0 0 -93.75
"chr7" 5114001 5115000 "*" 6.92287338566189e-11 1.57605630124247e-10 -100
"chr7" 5117001 5118000 "*" 3.41356554134364e-09 6.13669911406708e-09
66.6666666666667
"chr7" 5169001 5170000 "*" 2.71005440311001e-12 7.46311461268516e-12
68.5714285714286
"chr7" 5203001 5204000 "*" 7.6127992798547e-13 2.24763794190841e-12
91.9540229885057
"chr7" 5222001 5223000 "*" 1.49972523288966e-09 2.83643172292987e-09
66.6666666666667
"chr7" 5281001 5282000 "*" 0 0 -100
"chr7" 5312001 5313000 "*" 0 0 91.6666666666667
"chr7" 5314001 5315000 "*" 4.11539381761639e-06 4.77326970666537e-06
56.5217391304348
"chr7" 5341001 5342000 "*" 5.08344895622237e-07 6.71305812617882e-07 53.125
"chr7" 5436001 5437000 "*" 9.01168435429867e-08 1.31496388134128e-07
-73.4432234432234
"chr7" 5441001 5442000 "*" 7.7715611723761e-16 3.26213507634405e-15 -100
"chr7" 5466001 5467000 "*" 0 0 74.025974025974
"chr7" 5468001 5469000 "*" 0 0 88.7323943661972
"chr7" 5526001 5527000 "*" 0 0 -66.6250990478417
"chr7" 5552001 5553000 "*" 2.66453525910038e-15 1.05861327033776e-14 -100
"chr7" 5603001 5604000 "*" 3.65754238007554e-09 6.5511582950949e-09
-57.1428571428571
"chr7" 5618001 5619000 "*" 5.48638023900594e-10 1.10374357336421e-09 100
"chr7" 5623001 5624000 "*" 1.76194282680564e-05 1.86072784086141e-05 65
"chr7" 5629001 5630000 "*" 0 0 60.8522479266696
"chr7" 5805001 5806000 "*" 0 0 -100

"chr7" 6136001 6137000 "*" 1.39779633423487e-07 1.98463037958589e-07 100
"chr7" 6253001 6254000 "*" 8.01666433236647e-09 1.36392368970109e-08 100
"chr7" 6272001 6273000 "*" 1.11022302462516e-16 5.03662826618488e-16
50.4854368932039
"chr7" 6316001 6317000 "*" 8.7349629751543e-09 1.47505871898511e-08 -100
"chr7" 6322001 6323000 "*" 1.41274921662271e-05 1.51258433455559e-05
53.3333333333333
"chr7" 6413001 6414000 "*" 0 0 100
"chr7" 6568001 6569000 "*" 6.59550958292954e-09 1.13456194382773e-08 100
"chr7" 6572001 6573000 "*" 1.75746084352113e-11 4.3487155984754e-11
64.5454545454545
"chr7" 6585001 6586000 "*" 4.03360817794463e-05 4.02351074444744e-05
-58.0645161290323
"chr7" 6586001 6587000 "*" 6.66133814775094e-15 2.52791665956799e-14 100
"chr7" 6642001 6643000 "*" 0.000285593490543912 0.000248445226337853
52.1739130434783
"chr7" 6652001 6653000 "*" 1.51656465163796e-13 4.87803420672651e-13 100
"chr7" 6671001 6672000 "*" 2.75335310107039e-14 9.69727903855348e-14 -100
"chr7" 6759001 6760000 "*" 2.41147102286732e-11 5.85650866396907e-11 -85
"chr7" 6776001 6777000 "*" 6.59550958292954e-09 1.13456194382773e-08 -100
"chr7" 6911001 6912000 "*" 1.37584737158747e-07 1.96040848961799e-07
52.1739130434783
"chr7" 7097001 7098000 "*" 3.00631018856912e-07 4.09425962268758e-07
66.6666666666667
"chr7" 7683001 7684000 "*" 1.26078925077877e-10 2.77325409449444e-10 82.8125
"chr7" 7915001 7916000 "*" 7.9590863601986e-06 8.85270264440226e-06
-71.4285714285714
"chr7" 8472001 8473000 "*" 8.81478174685757e-06 9.73784941153298e-06
66.2790697674419
"chr7" 8473001 8474000 "*" 0 0 57.984223553633
"chr7" 9229001 9230000 "*" 2.00227675550835e-08 3.20459176617961e-08 -100
"chr7" 9766001 9767000 "*" 1.39110836183676e-07 1.98113805774322e-07
72.9372937293729
"chr7" 10125001 10126000 "*" 1.37828859436695e-10 3.01445544267661e-10 -100
"chr7" 10609001 10610000 "*" 2.33028818463765e-10 4.93719283454501e-10 -100
"chr7" 10693001 10694000 "*" 1.93210128673904e-07 2.69746728165517e-07 -80
"chr7" 10712001 10713000 "*" 3.6700841921089e-08 5.65530226072258e-08 -100
"chr7" 11641001 11642000 "*" 6.4152538836737e-10 1.27477697728417e-09 100
"chr7" 12308001 12309000 "*" 5.21499377015289e-09 9.11009415448405e-09 93.75
"chr7" 12631001 12632000 "*" 2.02327044007689e-12 5.65300384792621e-12 100
"chr7" 12632001 12633000 "*" 0 0 100
"chr7" 12726001 12727000 "*" 0 0 -55
"chr7" 12798001 12799000 "*" 0.000353293070441074 0.000302601276615348
52.2727272727273
"chr7" 13081001 13082000 "*" 0 0 -100
"chr7" 14107001 14108000 "*" 4.38609149000513e-11 1.02592295052525e-10
64.5161290322581
"chr7" 14611001 14612000 "*" 1.35447209004269e-14 4.94674708073053e-14 -100
"chr7" 14942001 14943000 "*" 1.09579243456892e-08 1.82823979609085e-08
76.7857142857143
"chr7" 15912001 15913000 "*" 5.63660229602192e-13 1.68905058935478e-12 100
"chr7" 16586001 16587000 "*" 5.52118017971281e-10 1.11028928479571e-09

-55.5555555555556
"chr7" 16752001 16753000 "*" 4.88993821656791e-06 5.61308433440487e-06
-51.219512195122
"chr7" 16794001 16795000 "*" 5.54128078440463e-05 5.41036098133341e-05
-58.5106382978723
"chr7" 17073001 17074000 "*" 3.91446555448871e-05 3.91201221655578e-05 -80
"chr7" 17453001 17454000 "*" 1.88737914186277e-15 7.62011598380321e-15 100
"chr7" 17781001 17782000 "*" 9.65729496371637e-10 1.87255912493232e-09 100
"chr7" 18415001 18416000 "*" 8.58968451922237e-12 2.213554983903e-11 100
"chr7" 19148001 19149000 "*" 5.08482145278322e-14 1.73432706170696e-13 100
"chr7" 19813001 19814000 "*" 0 0 53.1990586174813
"chr7" 20546001 20547000 "*" 0 0 -100
"chr7" 20688001 20689000 "*" 2.88779000712225e-11 6.92935809466068e-11 -100
"chr7" 20825001 20826000 "*" 0 0 91.981845688351
"chr7" 20826001 20827000 "*" 0 0 94.7368421052632
"chr7" 20830001 20831000 "*" 2.06501482580279e-14 7.3890370570066e-14
61.1675126903553
"chr7" 20912001 20913000 "*" 1.52466927971773e-12 4.33322475981408e-12 100
"chr7" 20984001 20985000 "*" 8.52140580320793e-12 2.19817259951489e-11 -100
"chr7" 21260001 21261000 "*" 6.51108822413704e-09 1.12423770428598e-08
-68.5393258426966
"chr7" 22528001 22529000 "*" 6.04438721296674e-11 1.38849255306602e-10 -100
"chr7" 22602001 22603000 "*" 4.36618494992214e-06 5.04443830498714e-06 -60
"chr7" 22603001 22604000 "*" 1.12458486967171e-09 2.15740815043925e-09 100
"chr7" 22766001 22767000 "*" 2.33146835171283e-15 9.32818831833246e-15 100
"chr7" 22767001 22768000 "*" 7.66053886991358e-15 2.88426834415713e-14
57.4468085106383
"chr7" 22894001 22895000 "*" 0 0 -72.4922037422037
"chr7" 22912001 22913000 "*" 2.00227675550835e-08 3.20459176617961e-08 100
"chr7" 23053001 23054000 "*" 0 0 87.9183673469388
"chr7" 23336001 23337000 "*" 1.39779633423487e-07 1.98463037958589e-07 100
"chr7" 23375001 23376000 "*" 2.73625566649116e-12 7.52483363579137e-12 100
"chr7" 23513001 23514000 "*" 0 0 58.5446186869674
"chr7" 23514001 23515000 "*" 0 0 97.2144846796657
"chr7" 23581001 23582000 "*" 1.2404662852461e-09 2.36887234713147e-09
-56.5217391304348
"chr7" 23868001 23869000 "*" 3.34128893442198e-08 5.19772864618523e-08 100
"chr7" 24212001 24213000 "*" 3.33066907387547e-16 1.4495649018245e-15 -100
"chr7" 24765001 24766000 "*" 1.35621640029449e-07 1.933989792704e-07
-68.4210526315789
"chr7" 25020001 25021000 "*" 3.76942921320733e-12 1.02059222659945e-11
-60.9958506224066
"chr7" 25067001 25068000 "*" 2.69007038866675e-13 8.38831022628993e-13 100
"chr7" 25073001 25074000 "*" 7.97140131680862e-14 2.65641277678906e-13 -100
"chr7" 25219001 25220000 "*" 0 0 100
"chr7" 25288001 25289000 "*" 2.33028818463765e-10 4.93719283454501e-10 100
"chr7" 25290001 25291000 "*" 4.73234496034536e-09 8.30627300264826e-09 100
"chr7" 25296001 25297000 "*" 2.88710388929303e-10 6.03399264731139e-10 100
"chr7" 25439001 25440000 "*" 4.01313771103418e-09 7.11882087237587e-09 -100
"chr7" 25513001 25514000 "*" 1.98124849859482e-10 4.25157611354621e-10 -100
"chr7" 25702001 25703000 "*" 1.39779633423487e-07 1.98463037958589e-07 -100
"chr7" 25727001 25728000 "*" 3.33066907387547e-16 1.4495649018245e-15 100

"chr7" 25991001 25992000 "*" 8.7349629751543e-09 1.47505871898511e-08 100
"chr7" 26125001 26126000 "*" 1.52794177310511e-09 2.87642564232045e-09 100
"chr7" 26141001 26142000 "*" 2.6165741273676e-06 3.12843692934624e-06
66.6666666666667
"chr7" 26385001 26386000 "*" 0 0 -100
"chr7" 26555001 26556000 "*" 5.28078811012911e-07 6.9591133871736e-07
-80.7692307692308
"chr7" 26653001 26654000 "*" 0 0 -60.8695652173913
"chr7" 27131001 27132000 "*" 4.24031642021205e-08 6.48914354320773e-08 75
"chr7" 27135001 27136000 "*" 0 0 -70.5882352941177
"chr7" 27152001 27153000 "*" 0 0 -100
"chr7" 27155001 27156000 "*" 0 0 -72.3118279569892
"chr7" 27162001 27163000 "*" 0 0 56.8658088235294
"chr7" 27199001 27200000 "*" 2.88657986402541e-15 1.14155613124573e-14
76.271186440678
"chr7" 27209001 27210000 "*" 0 0 -56.8375260071874
"chr7" 27213001 27214000 "*" 0 0 -74.7760039896225
"chr7" 27214001 27215000 "*" 0 0 54.7059374414684
"chr7" 27233001 27234000 "*" 0 0 -82.1917808219178
"chr7" 27264001 27265000 "*" 0 0 62.6596980255517
"chr7" 27275001 27276000 "*" 0 0 -56.578253517029
"chr7" 27380001 27381000 "*" 3.31690230837012e-12 9.03347040781321e-12 100
"chr7" 27735001 27736000 "*" 2.33028818463765e-10 4.93719283454501e-10 -100
"chr7" 27982001 27983000 "*" 1.67299207820548e-08 2.71377429441055e-08 100
"chr7" 28143001 28144000 "*" 4.8105963657008e-13 1.45623421922739e-12
87.8504672897196
"chr7" 28177001 28178000 "*" 3.7274627828765e-12 1.00985250136599e-11 100
"chr7" 28539001 28540000 "*" 4.65637306490407e-10 9.46720524046618e-10 -100
"chr7" 28550001 28551000 "*" 6.51443988530787e-06 7.33754914986653e-06
57.1428571428571
"chr7" 28551001 28552000 "*" 1.5277158427196e-09 2.87642564232045e-09 100
"chr7" 28893001 28894000 "*" 0 0 56.3061353573688
"chr7" 28894001 28895000 "*" 0 0 88.6792452830189
"chr7" 28948001 28949000 "*" 5.295763827462e-14 1.80214784640623e-13 100
"chr7" 28961001 28962000 "*" 3.31690230837012e-12 9.03347040781321e-12 100
"chr7" 29025001 29026000 "*" 9.0072393987839e-13 2.63157725646847e-12 100
"chr7" 29185001 29186000 "*" 8.89428530825853e-11 1.99846326244974e-10
66.6666666666667
"chr7" 29186001 29187000 "*" 3.66373598126302e-15 1.43441054525534e-14
89.2307692307692
"chr7" 29233001 29234000 "*" 0 0 55.2096705177578
"chr7" 29526001 29527000 "*" 2.88710388929303e-10 6.03399264731139e-10 -100
"chr7" 29846001 29847000 "*" 0 0 64.0702595419789
"chr7" 29869001 29870000 "*" 7.0006535457523e-08 1.03695832512054e-07 100
"chr7" 29968001 29969000 "*" 0 0 -61.9047619047619
"chr7" 30188001 30189000 "*" 5.58502354364165e-08 8.41879077739806e-08 -60
"chr7" 30514001 30515000 "*" 1.14124265593318e-11 2.89138062669327e-11 100
"chr7" 30612001 30613000 "*" 1.40068135581206e-05 1.50039561086948e-05
62.0689655172414
"chr7" 30689001 30690000 "*" 1.51864076869401e-11 3.78766406159505e-11 52.5
"chr7" 30690001 30691000 "*" 1.49613654798486e-12 4.26244554429779e-12 100
"chr7" 30692001 30693000 "*" 6.76862340087681e-06 7.60546331588873e-06

-70.4545454545455
"chr7" 30694001 30695000 "*" 0 0 100
"chr7" 30730001 30731000 "*" 4.01313771103418e-09 7.11882087237587e-09 100
"chr7" 30744001 30745000 "*" 8.01462659572039e-08 1.17734428441707e-07
71.4285714285714
"chr7" 30776001 30777000 "*" 4.57411886145564e-14 1.57248434075695e-13
72.3404255319149
"chr7" 30886001 30887000 "*" 0 0 100
"chr7" 30929001 30930000 "*" 1.98365768255826e-11 4.85612402473442e-11 100
"chr7" 30932001 30933000 "*" 0 0 100
"chr7" 30953001 30954000 "*" 1.35890187991095e-11 3.40841971363279e-11 -100
"chr7" 30959001 30960000 "*" 8.7349629751543e-09 1.47505871898511e-08 -100
"chr7" 30964001 30965000 "*" 3.34128893442198e-08 5.19772864618523e-08 -100
"chr7" 30966001 30967000 "*" 4.02167188440217e-12 1.08266700497926e-11 -100
"chr7" 30968001 30969000 "*" 2.33028818463765e-10 4.93719283454501e-10 100
"chr7" 30978001 30979000 "*" 0 0 100
"chr7" 31011001 31012000 "*" 3.52704532247117e-11 8.35581292290093e-11 -100
"chr7" 31018001 31019000 "*" 3.05247971343903e-09 5.51860864899255e-09
80.1470588235294
"chr7" 31031001 31032000 "*" 0.000499129401911658 0.000416865202314435
-52.3809523809524
"chr7" 31036001 31037000 "*" 0 0 66.6666666666667
"chr7" 31043001 31044000 "*" 2.73014943985572e-11 6.58595916221891e-11 100
"chr7" 31068001 31069000 "*" 4.85722573273506e-13 1.47002943516802e-12
-55.8620689655172
"chr7" 31096001 31097000 "*" 3.65831971539388e-05 3.6733698352541e-05 -65.625
"chr7" 31146001 31147000 "*" 0 0 61.7283950617284
"chr7" 31147001 31148000 "*" 4.73234496034536e-09 8.30627300264826e-09 -100
"chr7" 31455001 31456000 "*" 0 0 100
"chr7" 31519001 31520000 "*" 3.1276351653009e-07 4.2499136916602e-07 -90
"chr7" 32109001 32110000 "*" 7.34777936695075e-05 7.03396943214155e-05
57.8947368421053
"chr7" 32198001 32199000 "*" 7.0006535457523e-08 1.03695832512054e-07 -100
"chr7" 32245001 32246000 "*" 5.51780843238703e-14 1.87243792572872e-13 100
"chr7" 32247001 32248000 "*" 1.12458486967171e-09 2.15740815043925e-09 -100
"chr7" 32252001 32253000 "*" 7.34462490825649e-11 1.66806501914271e-10
63.3333333333333
"chr7" 32291001 32292000 "*" 0 0 78.8450543167524
"chr7" 32306001 32307000 "*" 0 0 92.9577464788732
"chr7" 32339001 32340000 "*" 3.23373847044284e-06 3.81325174359463e-06
-66.6666666666667
"chr7" 32513001 32514000 "*" 3.25818826539814e-05 3.29735679006858e-05
66.6666666666667
"chr7" 32945001 32946000 "*" 2.53960166229916e-08 4.01228567809867e-08
59.0163934426229
"chr7" 33567001 33568000 "*" 4.02167188440217e-12 1.08266700497926e-11 100
"chr7" 33744001 33745000 "*" 8.58497828382099e-10 1.68085351630486e-09
51.1627906976744
"chr7" 33769001 33770000 "*" 6.7390537594747e-14 2.26447131529591e-13 -100
"chr7" 33943001 33944000 "*" 0 0 89.7887323943662
"chr7" 33944001 33945000 "*" 0 0 51.7212038440309
"chr7" 33945001 33946000 "*" 0 0 66.7858467079824

"chr7" 34277001 34278000 "*" 4.76460759735176e-10 9.67735605684694e-10 -85.7142857142857
"chr7" 34279001 34280000 "*" 2.88710388929303e-10 6.03399264731139e-10 -100
"chr7" 34938001 34939000 "*" 1.39779633423487e-07 1.98463037958589e-07 -100
"chr7" 35299001 35300000 "*" 0 0 -100
"chr7" 35355001 35356000 "*" 0 0 100
"chr7" 35517001 35518000 "*" 3.55791675021866e-06 4.16929278397655e-06 57.1428571428571
"chr7" 35532001 35533000 "*" 4.00709354586581e-10 8.22085984817432e-10 73.6842105263158
"chr7" 35570001 35571000 "*" 0 0 -69.8412698412698
"chr7" 35571001 35572000 "*" 0 0 66.6666666666667
"chr7" 36026001 36027000 "*" 5.95319260554561e-09 1.03311085509556e-08 -86.6666666666667
"chr7" 36074001 36075000 "*" 2.91211499359179e-13 9.04418038733369e-13 80.6306306306306
"chr7" 36193001 36194000 "*" 0 0 -66.0508083140878
"chr7" 36255001 36256000 "*" 1.96644922567657e-11 4.82930612417392e-11 100
"chr7" 36274001 36275000 "*" 8.65973959207622e-14 2.87362558212404e-13 100
"chr7" 36340001 36341000 "*" 1.19904086659517e-14 4.40901388691796e-14 -100
"chr7" 36347001 36348000 "*" 0 0 -100
"chr7" 36584001 36585000 "*" 4.46405531835392e-05 4.42311628182131e-05 62.962962962963
"chr7" 36689001 36690000 "*" 8.28038748679205e-11 1.86807532887257e-10 -70
"chr7" 36696001 36697000 "*" 8.25450818808804e-13 2.42473843346228e-12 100
"chr7" 36723001 36724000 "*" 6.37490060739765e-13 1.89496793587877e-12 100
"chr7" 36763001 36764000 "*" 3.36462461225651e-08 5.23100510222184e-08 88.4615384615385
"chr7" 36776001 36777000 "*" 3.49096196394783e-10 7.21575902470817e-10 69.6428571428571
"chr7" 36792001 36793000 "*" 1.16408228700848e-06 1.46479698533027e-06 66.6666666666667
"chr7" 36795001 36796000 "*" 4.14335232790108e-13 1.26153063645578e-12 100
"chr7" 36973001 36974000 "*" 0.000199851262950967 0.000178453492150885 -54.5454545454545
"chr7" 37236001 37237000 "*" 6.77335965093562e-12 1.76897011246462e-11 -100
"chr7" 37363001 37364000 "*" 2.43809560015462e-05 2.51806608296571e-05 59.0909090909091
"chr7" 37759001 37760000 "*" 1.52794177310511e-09 2.87642564232045e-09 -100
"chr7" 37886001 37887000 "*" 0 0 58.780487804878
"chr7" 37956001 37957000 "*" 0 0 53.1493897323285
"chr7" 37982001 37983000 "*" 1.98043541566051e-08 3.18307369376677e-08 55.4054054054054
"chr7" 38105001 38106000 "*" 0 0 100
"chr7" 38281001 38282000 "*" 4.48406133335411e-08 6.84482503893299e-08 66.6666666666667
"chr7" 38449001 38450000 "*" 6.24654217240561e-10 1.2462108191303e-09 -100
"chr7" 38732001 38733000 "*" 6.87628272766361e-07 8.92892153029571e-07 -58.3333333333333
"chr7" 38742001 38743000 "*" 0 0 100
"chr7" 38749001 38750000 "*" 0 0 100
"chr7" 39317001 39318000 "*" 4.57183624291702e-10 9.31326369875184e-10

-90.9090909090909
"chr7" 39332001 39333000 "*" 9.25072787483217e-07 1.17990633180095e-06
52.7777777777778
"chr7" 39379001 39380000 "*" 0 0 68.1111111111111
"chr7" 39446001 39447000 "*" 2.77555756156289e-15 1.10015415195978e-14
78.0645161290323
"chr7" 39453001 39454000 "*" 0 0 76.378896882494
"chr7" 39459001 39460000 "*" 1.11022302462516e-16 5.03662826618488e-16 100
"chr7" 39559001 39560000 "*" 6.92287338566189e-11 1.57605630124247e-10 -100
"chr7" 39561001 39562000 "*" 8.7349629751543e-09 1.47505871898511e-08 100
"chr7" 39772001 39773000 "*" 2.77555756156289e-14 9.77094403418417e-14
59.5238095238095
"chr7" 39940001 39941000 "*" 6.24654217240561e-10 1.2462108191303e-09 100
"chr7" 39950001 39951000 "*" 1.39779633423487e-07 1.98463037958589e-07 100
"chr7" 40190001 40191000 "*" 2.22044604925031e-16 9.81641919380259e-16 100
"chr7" 40510001 40511000 "*" 2.64951482975562e-09 4.82186011375752e-09 100
"chr7" 41044001 41045000 "*" 2.91028312560115e-11 6.97590507139978e-11 100
"chr7" 41048001 41049000 "*" 3.33066907387547e-16 1.4495649018245e-15
-71.0526315789474
"chr7" 41246001 41247000 "*" 0 0 94.7976878612717
"chr7" 41739001 41740000 "*" 1.15145414314455e-08 1.90979393180535e-08
58.3333333333333
"chr7" 41754001 41755000 "*" 1.92946192356658e-09 3.59193011611483e-09 -100
"chr7" 41993001 41994000 "*" 1.36604061395929e-11 3.42333952202875e-11 100
"chr7" 42018001 42019000 "*" 1.78688100713753e-05 1.88505925150618e-05 -65.625
"chr7" 42064001 42065000 "*" 1.42297285066206e-11 3.56162562690713e-11
-63.9344262295082
"chr7" 42087001 42088000 "*" 4.01313771103418e-09 7.11882087237587e-09 -100
"chr7" 42264001 42265000 "*" 4.14335232790108e-13 1.26153063645578e-12 100
"chr7" 42276001 42277000 "*" 0 0 68.6665764844747
"chr7" 42277001 42278000 "*" 0 0 50.1689182607069
"chr7" 42376001 42377000 "*" 2.64951482975562e-09 4.82186011375752e-09 100
"chr7" 43151001 43152000 "*" 2.15125472990962e-10 4.58126659943874e-10 -100
"chr7" 43248001 43249000 "*" 0 0 100
"chr7" 43256001 43257000 "*" 2.68450373042128e-10 5.64465213127486e-10 100
"chr7" 43359001 43360000 "*" 8.88178419700125e-16 3.70670032207938e-15 100
"chr7" 43369001 43370000 "*" 4.01313771103418e-09 7.11882087237587e-09 -100
"chr7" 43818001 43819000 "*" 0 0 -100
"chr7" 44024001 44025000 "*" 3.33066907387547e-16 1.4495649018245e-15 100
"chr7" 44102001 44103000 "*" 1.56863411149288e-12 4.4469621057462e-12 -100
"chr7" 44127001 44128000 "*" 1.14130926931466e-13 3.7276674571693e-13
-73.0769230769231
"chr7" 44142001 44143000 "*" 3.83499898504169e-09 6.85560912741795e-09
-50.7246376811594
"chr7" 44146001 44147000 "*" 5.33495142740481e-06 6.09237962218305e-06
66.6666666666667
"chr7" 44168001 44169000 "*" 2.455369241261e-12 6.8003688121118e-12 100
"chr7" 44180001 44181000 "*" 2.75335310107039e-14 9.69727903855348e-14 100
"chr7" 44197001 44198000 "*" 0 0 -53.7877061013601
"chr7" 44228001 44229000 "*" 3.6700841921089e-08 5.65530226072258e-08 -100
"chr7" 44240001 44241000 "*" 0 0 66.6666666666667
"chr7" 44308001 44309000 "*" 2.82425030362354e-07 3.86043503628749e-07

-57.1428571428571
"chr7" 44317001 44318000 "*" 3.89677179413184e-12 1.05401013025378e-11
-53.1645569620253
"chr7" 44333001 44334000 "*" 1.25917409943277e-07 1.80241654364416e-07
62.6865671641791
"chr7" 44360001 44361000 "*" 0 0 61.7021276595745
"chr7" 44365001 44366000 "*" 0 0 61.0835921680687
"chr7" 44369001 44370000 "*" 0 0 100
"chr7" 44570001 44571000 "*" 4.44089209850063e-16 1.91071758245033e-15 -56.25
"chr7" 44576001 44577000 "*" 1.0325074129014e-14 3.83008663711937e-14 -100
"chr7" 44605001 44606000 "*" 1.73542880066435e-09 3.25044851591565e-09
64.367816091954
"chr7" 44612001 44613000 "*" 2.02060590481778e-13 6.40127405465654e-13 -100
"chr7" 44647001 44648000 "*" 1.37828859436695e-10 3.01445544267661e-10 100
"chr7" 44739001 44740000 "*" 4.63796966942986e-07 6.15615843513385e-07 55
"chr7" 44749001 44750000 "*" 1.06326059068351e-12 3.08194051101437e-12
-91.304347826087
"chr7" 44808001 44809000 "*" 0 0 100
"chr7" 44822001 44823000 "*" 5.794809077031e-12 1.52911192757929e-11 -100
"chr7" 44886001 44887000 "*" 2.95924516002799e-08 4.64194833077295e-08
-63.3333333333333
"chr7" 44910001 44911000 "*" 2.1582735598713e-13 6.81978931365217e-13
-70.2589489718203
"chr7" 44923001 44924000 "*" 6.92287338566189e-11 1.57605630124247e-10 -100
"chr7" 44951001 44952000 "*" 1.11022302462516e-16 5.03662826618488e-16
60.7142857142857
"chr7" 44999001 45000000 "*" 2.00227675550835e-08 3.20459176617961e-08 100
"chr7" 45091001 45092000 "*" 2.67283972732457e-11 6.45686178157698e-11 -100
"chr7" 45126001 45127000 "*" 4.46831460720887e-12 1.19653412271985e-11
54.394880481837
"chr7" 45200001 45201000 "*" 1.67299207820548e-08 2.71377429441055e-08 100
"chr7" 45216001 45217000 "*" 2.15070528053474e-09 3.9725885757664e-09
78.8461538461538
"chr7" 45217001 45218000 "*" 3.33066907387547e-16 1.4495649018245e-15
65.7142857142857
"chr7" 45219001 45220000 "*" 2.37169239802881e-09 4.3576136542416e-09
60.4166666666667
"chr7" 45229001 45230000 "*" 0 0 55.5555555555556
"chr7" 45230001 45231000 "*" 0 0 100
"chr7" 45256001 45257000 "*" 0 0 -77.0833333333333
"chr7" 45258001 45259000 "*" 0 0 100
"chr7" 45264001 45265000 "*" 1.14130926931466e-13 3.7276674571693e-13 -100
"chr7" 45277001 45278000 "*" 1.33135739033285e-06 1.66191903055614e-06
-51.8518518518519
"chr7" 45388001 45389000 "*" 5.35815304347764e-08 8.0945115347028e-08
60.1215521271622
"chr7" 45608001 45609000 "*" 3.29490690287315e-09 5.93592111612489e-09
-73.3333333333333
"chr7" 45616001 45617000 "*" 9.08108033215171e-11 2.03407753381119e-10 -100
"chr7" 45626001 45627000 "*" 1.11022302462516e-16 5.03662826618488e-16 100
"chr7" 45629001 45630000 "*" 3.30926196112635e-06 3.89685498077294e-06
-61.038961038961

"chr7" 45631001 45632000 "*" 1.89848137210902e-14 6.82212372863451e-14 -100
"chr7" 45649001 45650000 "*" 2.30549256352575e-07 3.18718504627128e-07
-52.0574162679426
"chr7" 45662001 45663000 "*" 1.04186075766677e-07 1.50843540960324e-07
-52.3809523809524
"chr7" 45687001 45688000 "*" 6.19032932323016e-05 6.00078781925201e-05
-58.8089330024814
"chr7" 45700001 45701000 "*" 1.36125488658934e-09 2.58768733150129e-09
53.8461538461538
"chr7" 45702001 45703000 "*" 0 0 -78.0487804878049
"chr7" 45754001 45755000 "*" 2.19352143782103e-07 3.04065846808353e-07
-52.3809523809524
"chr7" 45854001 45855000 "*" 2.99258027014471e-05 3.04808480823419e-05
-57.6329331046312
"chr7" 45905001 45906000 "*" 8.39328606616618e-14 2.79009090750649e-13
-87.2340425531915
"chr7" 45912001 45913000 "*" 1.00446317929936e-10 2.23961847972101e-10
67.2897196261682
"chr7" 45928001 45929000 "*" 0 0 81.2013223219782
"chr7" 45956001 45957000 "*" 8.51605785889831e-10 1.66802527211457e-09
64.8648648648649
"chr7" 46223001 46224000 "*" 3.49387185849537e-13 1.07603895170356e-12
-73.98753894081
"chr7" 46256001 46257000 "*" 1.4432899320127e-15 5.90750815956055e-15 100
"chr7" 46430001 46431000 "*" 0 0 100
"chr7" 46495001 46496000 "*" 2.33028818463765e-10 4.93719283454501e-10 100
"chr7" 46881001 46882000 "*" 0 0 100
"chr7" 46899001 46900000 "*" 6.25987782720827e-08 9.37253242615875e-08
62.7906976744186
"chr7" 47076001 47077000 "*" 9.63829016598083e-12 2.46993429847588e-11 100
"chr7" 47193001 47194000 "*" 1.50623957750895e-12 4.28756717183481e-12 100
"chr7" 47231001 47232000 "*" 5.794809077031e-12 1.52911192757929e-11 100
"chr7" 47286001 47287000 "*" 1.13140401492018e-08 1.87801380787728e-08 100
"chr7" 47338001 47339000 "*" 8.21864699740438e-06 9.11963501990967e-06
-59.233926128591
"chr7" 47394001 47395000 "*" 2.88657986402541e-15 1.14155613124573e-14
-56.8932038834951
"chr7" 47404001 47405000 "*" 7.5726662701614e-06 8.44903872709532e-06
-58.3333333333333
"chr7" 47434001 47435000 "*" 3.5527136788005e-15 1.39277426410519e-14
-92.8104575163399
"chr7" 47449001 47450000 "*" 2.85005352651524e-12 7.81870499366183e-12
-57.6923076923077
"chr7" 47496001 47497000 "*" 8.2022381598934e-06 9.10251728824986e-06
-66.6666666666667
"chr7" 47544001 47545000 "*" 0 0 100
"chr7" 47607001 47608000 "*" 1.01030295240889e-14 3.75322807935163e-14
-65.5172413793103
"chr7" 47632001 47633000 "*" 6.56739884696833e-10 1.30336388995608e-09
-89.1089108910891
"chr7" 47636001 47637000 "*" 0 0 100
"chr7" 47658001 47659000 "*" 2.08814465718632e-09 3.86232567960463e-09 -100

"chr7" 47672001 47673000 "*" 9.65729496371637e-10 1.87255912493232e-09 -100
"chr7" 47674001 47675000 "*" 3.34725824058246e-07 4.53033333120427e-07
70.3703703703704
"chr7" 47697001 47698000 "*" 2.61596887682902e-05 2.68855595976014e-05
52.6315789473684
"chr7" 47719001 47720000 "*" 3.99680288865056e-15 1.5566998858822e-14 -100
"chr7" 47797001 47798000 "*" 1.63786761930851e-11 4.0679978854173e-11
64.0909090909091
"chr7" 47817001 47818000 "*" 1.28785870856518e-14 4.71440608755782e-14 100
"chr7" 47845001 47846000 "*" 1.98325800226939e-11 4.85612402473442e-11 -100
"chr7" 47892001 47893000 "*" 1.49883800426576e-06 1.85791347166305e-06
65.3846153846154
"chr7" 47902001 47903000 "*" 6.19534423762502e-11 1.4218382701847e-10
56.9036697247706
"chr7" 47984001 47985000 "*" 6.59550958292954e-09 1.13456194382773e-08 -100
"chr7" 48049001 48050000 "*" 7.4951922419686e-06 8.36942869052848e-06
71.0526315789474
"chr7" 48128001 48129000 "*" 0 0 -56.7415730337079
"chr7" 48129001 48130000 "*" 0 0 -69.3313953488372
"chr7" 48258001 48259000 "*" 1.74853465040314e-11 4.32752710900609e-11 -100
"chr7" 48326001 48327000 "*" 4.93500784792822e-07 6.52819474468471e-07
66.6666666666667
"chr7" 48483001 48484000 "*" 6.92287338566189e-11 1.57605630124247e-10 100
"chr7" 48495001 48496000 "*" 0 0 90.4761904761905
"chr7" 49445001 49446000 "*" 2.17381668221606e-13 6.85151076607736e-13 -60
"chr7" 49448001 49449000 "*" 7.0006535457523e-08 1.03695832512054e-07 -100
"chr7" 49831001 49832000 "*" 2.65468190095985e-05 2.72579034500328e-05
51.8518518518519
"chr7" 49832001 49833000 "*" 2.88779000712225e-11 6.92935809466068e-11 -100
"chr7" 49925001 49926000 "*" 2.93692584918404e-06 3.48641648244637e-06
-65.7894736842105
"chr7" 50109001 50110000 "*" 6.92287338566189e-11 1.57605630124247e-10 -100
"chr7" 50235001 50236000 "*" 0 0 100
"chr7" 50244001 50245000 "*" 1.11022302462516e-16 5.03662826618488e-16
99.009900990099
"chr7" 50321001 50322000 "*" 3.90831811358794e-12 1.05610567215048e-11 -100
"chr7" 50322001 50323000 "*" 6.4152538836737e-10 1.27477697728417e-09 -100
"chr7" 50354001 50355000 "*" 2.73014943985572e-11 6.58595916221891e-11 100
"chr7" 50418001 50419000 "*" 2.00227675550835e-08 3.20459176617961e-08 100
"chr7" 50425001 50426000 "*" 9.65729496371637e-10 1.87255912493232e-09 100
"chr7" 50440001 50441000 "*" 1.11022302462516e-16 5.03662826618488e-16
-82.6923076923077
"chr7" 50535001 50536000 "*" 0 0 -93.1818181818182
"chr7" 50541001 50542000 "*" 1.11022302462516e-15 4.59817122935606e-15
-78.2608695652174
"chr7" 50570001 50571000 "*" 6.23834317536875e-13 1.85961172622026e-12
-70.2702702702703
"chr7" 50734001 50735000 "*" 0 0 94.4444444444444
"chr7" 50760001 50761000 "*" 1.35036426485158e-12 3.86629547363774e-12 100
"chr7" 50821001 50822000 "*" 6.9333427887841e-13 2.05277298063267e-12
-87.2727272727273
"chr7" 50855001 50856000 "*" 8.65973959207622e-14 2.87362558212404e-13 100

"chr7" 51029001 51030000 "*" 0 0 -93.2885906040269
"chr7" 51041001 51042000 "*" 9.63829016598083e-12 2.46993429847588e-11 -100
"chr7" 51079001 51080000 "*" 4.73234496034536e-09 8.30627300264826e-09 100
"chr7" 51080001 51081000 "*" 3.40125705378114e-10 7.03784073980162e-10 100
"chr7" 51096001 51097000 "*" 0 0 60
"chr7" 51107001 51108000 "*" 2.69118061169138e-12 7.41554626115558e-12 -54
"chr7" 51176001 51177000 "*" 1.35036426485158e-12 3.86629547363774e-12 100
"chr7" 51297001 51298000 "*" 0 0 100
"chr7" 51333001 51334000 "*" 1.11022302462516e-16 5.03662826618488e-16 -100
"chr7" 51344001 51345000 "*" 9.65338919911574e-13 2.80678910656113e-12 100
"chr7" 51436001 51437000 "*" 1.74853465040314e-11 4.32752710900609e-11 -100
"chr7" 51437001 51438000 "*" 3.34128893442198e-08 5.19772864618523e-08 -100
"chr7" 51546001 51547000 "*" 2.08814465718632e-09 3.86232567960463e-09 100
"chr7" 51941001 51942000 "*" 0 0 100
"chr7" 51967001 51968000 "*" 2.15125472990962e-10 4.58126659943874e-10 -100
"chr7" 51969001 51970000 "*" 0 0 -89.3203883495146
"chr7" 52174001 52175000 "*" 4.73234496034536e-09 8.30627300264826e-09 -100
"chr7" 53188001 53189000 "*" 1.11022302462516e-15 4.59817122935606e-15 100
"chr7" 53861001 53862000 "*" 1.4432899320127e-15 5.90750815956055e-15 100
"chr7" 53921001 53922000 "*" 4.02167188440217e-12 1.08266700497926e-11 100
"chr7" 53967001 53968000 "*" 9.67004254448511e-14 3.18178990782609e-13 100
"chr7" 54146001 54147000 "*" 1.90181204118289e-13 6.05298617539364e-13 100
"chr7" 54310001 54311000 "*" 1.51656465163796e-13 4.87803420672651e-13 100
"chr7" 54326001 54327000 "*" 1.50623957750895e-12 4.28756717183481e-12 100
"chr7" 54515001 54516000 "*" 1.12458486967171e-09 2.15740815043925e-09 100
"chr7" 54614001 54615000 "*" 0 0 59.1823839915582
"chr7" 54662001 54663000 "*" 1.5277158427196e-09 2.87642564232045e-09 -100
"chr7" 54732001 54733000 "*" 0 0 67.9849012775842
"chr7" 54852001 54853000 "*" 1.25284005392245e-10 2.75649590272477e-10 100
"chr7" 55037001 55038000 "*" 0 0 100
"chr7" 55049001 55050000 "*" 2.70915808564531e-07 3.71205024353761e-07
-61.8181818181818
"chr7" 55088001 55089000 "*" 0 0 -100
"chr7" 55136001 55137000 "*" 4.01313771103418e-09 7.11882087237587e-09 100
"chr7" 55211001 55212000 "*" 0 0 100
"chr7" 55317001 55318000 "*" 2.1094237467878e-15 8.47434540879246e-15 -100
"chr7" 55346001 55347000 "*" 0 0 -83.1168831168831
"chr7" 55380001 55381000 "*" 5.75373082511987e-12 1.52087081957095e-11
60.6060606060606
"chr7" 55483001 55484000 "*" 3.77475828372553e-15 1.47379557022071e-14 -100
"chr7" 55484001 55485000 "*" 3.04324343503026e-11 7.26599935673121e-11 100
"chr7" 55942001 55943000 "*" 2.79987144580218e-12 7.68634726811898e-12 100
"chr7" 56020001 56021000 "*" 1.01085806392121e-11 2.5855266374848e-11
-59.6899224806202
"chr7" 56131001 56132000 "*" 0 0 -100
"chr7" 56344001 56345000 "*" 2.00227675550835e-08 3.20459176617961e-08 100
"chr7" 56672001 56673000 "*" 2.64325332233195e-06 3.15856923971574e-06
53.8461538461538
"chr7" 57063001 57064000 "*" 9.54223234927998e-06 1.04936619493498e-05
67.3417721518987
"chr7" 57245001 57246000 "*" 9.65338919911574e-13 2.80678910656113e-12 100
"chr7" 57268001 57269000 "*" 1.36604061395929e-11 3.42333952202875e-11 -100

"chr7" 57269001 57270000 "*" 3.15717379906211e-07 4.28711514914722e-07 53.9473684210526
"chr7" 57270001 57271000 "*" 1.33235292798517e-08 2.1936464870907e-08 90.9090909090909
"chr7" 57294001 57295000 "*" 1.90817864564874e-06 2.32887155826907e-06 75
"chr7" 57302001 57303000 "*" 3.01647595790655e-13 9.35923352982664e-13 -63.4615384615385
"chr7" 57376001 57377000 "*" 0 0 100
"chr7" 57395001 57396000 "*" 3.84137166520304e-14 1.32929369817675e-13 -100
"chr7" 57420001 57421000 "*" 0 0 83.8235294117647
"chr7" 57426001 57427000 "*" 2.00227675550835e-08 3.20459176617961e-08 -100
"chr7" 57453001 57454000 "*" 1.3131162823754e-10 2.88242388672858e-10 59.7402597402597
"chr7" 57551001 57552000 "*" 2.74978409153803e-07 3.76481834934674e-07 72.2222222222222
"chr7" 57707001 57708000 "*" 3.02546876440601e-12 8.27706096806656e-12 -67.6470588235294
"chr7" 57708001 57709000 "*" 0 0 83.3333333333333
"chr7" 57709001 57710000 "*" 9.59876232942136e-08 1.39596398821248e-07 -71.2643678160919
"chr7" 57715001 57716000 "*" 0 0 70.4131516331387
"chr7" 61055001 61056000 "*" 0 0 76.4227642276423
"chr7" 61746001 61747000 "*" 2.96843660763102e-11 7.11039353170169e-11 -79.1666666666667
"chr7" 61753001 61754000 "*" 2.70847788641504e-11 6.53935510679517e-11 -66.6666666666667
"chr7" 61780001 61781000 "*" 0 0 100
"chr7" 61782001 61783000 "*" 0 0 -100
"chr7" 61795001 61796000 "*" 4.67825534977706e-08 7.12495675341246e-08 -65.993265993266
"chr7" 62476001 62477000 "*" 2.62900812231237e-13 8.21294938531841e-13 -100
"chr7" 62507001 62508000 "*" 4.73234496034536e-09 8.30627300264826e-09 100
"chr7" 62694001 62695000 "*" 5.75760550347582e-10 1.15532640056407e-09 61.6736990154712
"chr7" 63016001 63017000 "*" 8.56161925799626e-08 1.25345822649662e-07 -56.6666666666667
"chr7" 63029001 63030000 "*" 0 0 -100
"chr7" 63035001 63036000 "*" 8.10462807976364e-15 3.04213344674586e-14 -66.6666666666667
"chr7" 63213001 63214000 "*" 7.58451423443773e-07 9.78988397580507e-07 68.2352941176471
"chr7" 63231001 63232000 "*" 0 0 78.9473684210526
"chr7" 63232001 63233000 "*" 5.00279030424267e-08 7.5876302199909e-08 -50.609756097561
"chr7" 63350001 63351000 "*" 2.88710388929303e-10 6.03399264731139e-10 100
"chr7" 63459001 63460000 "*" 0 0 -85.8490566037736
"chr7" 63551001 63552000 "*" 6.4152538836737e-10 1.27477697728417e-09 -100
"chr7" 63581001 63582000 "*" 0 0 -100
"chr7" 63641001 63642000 "*" 6.69197808278454e-10 1.32657262837556e-09 -76.271186440678
"chr7" 63961001 63962000 "*" 0 0 100
"chr7" 64083001 64084000 "*" 3.04324343503026e-11 7.26599935673121e-11 100

"chr7" 64348001 64349000 "*" 1.97419818270816e-08 3.17390516605308e-08 71.4285714285714
"chr7" 64363001 64364000 "*" 0 0 -100
"chr7" 64541001 64542000 "*" 0 0 79.2746113989637
"chr7" 64601001 64602000 "*" 1.11022302462516e-15 4.59817122935606e-15 51.8590998043053
"chr7" 64756001 64757000 "*" 2.79440914852103e-11 6.73046092948135e-11 -100
"chr7" 64779001 64780000 "*" 2.00227675550835e-08 3.20459176617961e-08 100
"chr7" 64887001 64888000 "*" 3.40125705378114e-10 7.03784073980162e-10 100
"chr7" 65112001 65113000 "*" 2.8199664825479e-14 9.92183471463377e-14 -60.9164420485175
"chr7" 65433001 65434000 "*" 3.33066907387547e-16 1.4495649018245e-15 51.4285714285714
"chr7" 65575001 65576000 "*" 2.69490696425123e-08 4.24751240833492e-08 -80.8510638297872
"chr7" 65654001 65655000 "*" 1.48087875295744e-10 3.22075573380618e-10 100
"chr7" 65743001 65744000 "*" 0 0 100
"chr7" 65986001 65987000 "*" 2.08814465718632e-09 3.86232567960463e-09 -100
"chr7" 65989001 65990000 "*" 3.95353005888666e-09 7.03928529283733e-09 100
"chr7" 66007001 66008000 "*" 1.13882125951648e-10 2.521329619527e-10 -73.6842105263158
"chr7" 66256001 66257000 "*" 2.1094237467878e-15 8.47434540879246e-15 -100
"chr7" 66310001 66311000 "*" 0 0 -79.2452830188679
"chr7" 66888001 66889000 "*" 1.4432899320127e-15 5.90750815956055e-15 -100
"chr7" 67090001 67091000 "*" 0 0 100
"chr7" 67125001 67126000 "*" 5.01025332333427e-10 1.01358031135889e-09 -100
"chr7" 67162001 67163000 "*" 6.59550958292954e-09 1.13456194382773e-08 -100
"chr7" 67253001 67254000 "*" 3.66181608701943e-07 4.92728439089831e-07 -54.5454545454545
"chr7" 67451001 67452000 "*" 6.55384191361463e-10 1.30082815732091e-09 96.1538461538462
"chr7" 67487001 67488000 "*" 0 0 100
"chr7" 67509001 67510000 "*" 1.82576453955363e-09 3.41068507338628e-09 53.2258064516129
"chr7" 67642001 67643000 "*" 1.12059396206732e-08 1.86692603788351e-08 -60
"chr7" 67671001 67672000 "*" 2.16382467499443e-13 6.82775854166559e-13 100
"chr7" 68410001 68411000 "*" 2.02327044007689e-12 5.65300384792621e-12 -100
"chr7" 68803001 68804000 "*" 2.25100731721106e-07 3.11620182818972e-07 62.962962962963
"chr7" 68892001 68893000 "*" 4.26951363152739e-10 8.72510873516957e-10 100
"chr7" 68913001 68914000 "*" 1.21231457095305e-08 2.00547207561099e-08 56.472049689441
"chr7" 69063001 69064000 "*" 0 0 80.4649300125863
"chr7" 69122001 69123000 "*" 2.15125472990962e-10 4.58126659943874e-10 100
"chr7" 69575001 69576000 "*" 9.65338919911574e-13 2.80678910656113e-12 -100
"chr7" 69722001 69723000 "*" 2.26428459693118e-05 2.35011539701264e-05 -68.1818181818182
"chr7" 69970001 69971000 "*" 2.4535928844216e-14 8.69144396197119e-14 -100
"chr7" 70149001 70150000 "*" 3.26386408011636e-07 4.42320630305102e-07 -64.8832684824903
"chr7" 70153001 70154000 "*" 1.13140401492018e-08 1.87801380787728e-08 -100
"chr7" 70160001 70161000 "*" 0 0 72.9357798165138

"chr7" 70212001 70213000 "*" 1.44362299892009e-12 4.12255313375858e-12
-66.6666666666667
"chr7" 70358001 70359000 "*" 4.21277148177079e-06 4.87902124303931e-06
-70.9090909090909
"chr7" 70620001 70621000 "*" 0 0 100
"chr7" 70639001 70640000 "*" 3.33066907387547e-16 1.4495649018245e-15 100
"chr7" 70664001 70665000 "*" 7.0006535457523e-08 1.03695832512054e-07 100
"chr7" 70882001 70883000 "*" 2.00227675550835e-08 3.20459176617961e-08 100
"chr7" 71097001 71098000 "*" 1.11022302462516e-16 5.03662826618488e-16 -100
"chr7" 71175001 71176000 "*" 7.67056418382595e-11 1.7359448580318e-10 100
"chr7" 71288001 71289000 "*" 0 0 100
"chr7" 71297001 71298000 "*" 0 0 100
"chr7" 71339001 71340000 "*" 4.85167461761193e-14 1.6617373494005e-13 100
"chr7" 71439001 71440000 "*" 9.65729496371637e-10 1.87255912493232e-09 100
"chr7" 71454001 71455000 "*" 5.7065463465733e-14 1.93318145576218e-13 100
"chr7" 71574001 71575000 "*" 2.6002897091093e-07 3.5712799351642e-07
82.1428571428571
"chr7" 71672001 71673000 "*" 2.16382467499443e-13 6.82775854166559e-13 -100
"chr7" 71774001 71775000 "*" 0 0 80
"chr7" 71822001 71823000 "*" 9.5812247025151e-14 3.15667542866807e-13 100
"chr7" 71871001 71872000 "*" 0 0 100
"chr7" 71898001 71899000 "*" 2.68673971959288e-14 9.47266815431045e-14 100
"chr7" 72003001 72004000 "*" 0 0 -100
"chr7" 72012001 72013000 "*" 3.12868741914052e-07 4.25120562657041e-07
-55.5555555555556
"chr7" 72019001 72020000 "*" 0.000726374117344109 0.000590081342527627
52.1739130434783
"chr7" 72275001 72276000 "*" 0 0 78.9473684210526
"chr7" 72314001 72315000 "*" 4.26951363152739e-10 8.72510873516957e-10 100
"chr7" 72733001 72734000 "*" 1.67494906833099e-11 4.15539517766454e-11
55.1020408163265
"chr7" 72735001 72736000 "*" 8.01666433236647e-09 1.36392368970109e-08 100
"chr7" 72754001 72755000 "*" 2.4634847495264e-08 3.89838047190206e-08
73.0769230769231
"chr7" 72757001 72758000 "*" 9.67004254448511e-14 3.18178990782609e-13 100
"chr7" 72772001 72773000 "*" 6.28520124834608e-09 1.08743118306163e-08
-80.6282722513089
"chr7" 72780001 72781000 "*" 0 0 81.5197428833793
"chr7" 72787001 72788000 "*" 0 0 63.1578947368421
"chr7" 72792001 72793000 "*" 0 0 58.5592401126382
"chr7" 72815001 72816000 "*" 1.98365768255826e-11 4.85612402473442e-11 -100
"chr7" 72837001 72838000 "*" 0 0 -100
"chr7" 72845001 72846000 "*" 1.98538791740255e-06 2.41610225827831e-06
-52.3809523809524
"chr7" 72846001 72847000 "*" 2.88657986402541e-15 1.14155613124573e-14
66.6666666666667
"chr7" 72847001 72848000 "*" 0 0 77.9527559055118
"chr7" 72937001 72938000 "*" 0 0 -100
"chr7" 72947001 72948000 "*" 1.88414144886018e-06 2.30162304314199e-06 60
"chr7" 72975001 72976000 "*" 3.5032281042735e-07 4.72754385845506e-07
57.1428571428571
"chr7" 72982001 72983000 "*" 3.13178038940931e-05 3.18058313017091e-05

52.3809523809524
"chr7" 72993001 72994000 "*" 0 0 -100
"chr7" 73015001 73016000 "*" 4.24327240011735e-13 1.28950544965043e-12
-66.7073170731707
"chr7" 73073001 73074000 "*" 0 0 -66.6666666666667
"chr7" 73084001 73085000 "*" 0 0 -73.3699633699634
"chr7" 73162001 73163000 "*" 3.34128893442198e-08 5.19772864618523e-08 -100
"chr7" 73181001 73182000 "*" 0 0 93.6416184971098
"chr7" 73224001 73225000 "*" 1.67299207820548e-08 2.71377429441055e-08 -100
"chr7" 73246001 73247000 "*" 2.22044604925031e-16 9.81641919380259e-16
-52.4018379281537
"chr7" 73308001 73309000 "*" 2.89768209427166e-14 1.01828132245762e-13
70.9677419354839
"chr7" 73318001 73319000 "*" 1.11022302462516e-16 5.03662826618488e-16
78.2608695652174
"chr7" 73417001 73418000 "*" 0 0 -55.191637630662
"chr7" 73450001 73451000 "*" 1.00808250635964e-13 3.31107370956809e-13 100
"chr7" 73553001 73554000 "*" 0 0 100
"chr7" 73580001 73581000 "*" 9.32920407592519e-12 2.39698743716789e-11
-90.9090909090909
"chr7" 73614001 73615000 "*" 0 0 -100
"chr7" 73627001 73628000 "*" 0 0 53.1645569620253
"chr7" 73669001 73670000 "*" 0 0 -100
"chr7" 73684001 73685000 "*" 2.18713935851156e-14 7.80893646430581e-14 -100
"chr7" 73691001 73692000 "*" 7.03881397612349e-14 2.35996780481906e-13
-91.8918918918919
"chr7" 73705001 73706000 "*" 4.86753970463383e-11 1.13145908161915e-10 100
"chr7" 73708001 73709000 "*" 7.7715611723761e-16 3.26213507634405e-15 100
"chr7" 73711001 73712000 "*" 1.88245228693873e-06 2.29966065245188e-06
52.1739130434783
"chr7" 73725001 73726000 "*" 1.22124532708767e-15 5.03921984217778e-15 100
"chr7" 73727001 73728000 "*" 0 0 -57.6106194690265
"chr7" 73895001 73896000 "*" 0 0 -58.8235294117647
"chr7" 73996001 73997000 "*" 0.000140863899290244 0.000128912110636932
-55.03300330033
"chr7" 74068001 74069000 "*" 8.57092175010621e-13 2.51149198099779e-12 100
"chr7" 74073001 74074000 "*" 0 0 -100
"chr7" 74098001 74099000 "*" 5.55111512312578e-16 2.36485094870365e-15 100
"chr7" 74479001 74480000 "*" 1.67299207820548e-08 2.71377429441055e-08 100
"chr7" 75162001 75163000 "*" 2.43005615629954e-09 4.45014222280278e-09 100
"chr7" 75226001 75227000 "*" 1.83605353143435e-11 4.53390608738371e-11
-66.6666666666667
"chr7" 75245001 75246000 "*" 2.88710388929303e-10 6.03399264731139e-10 100
"chr7" 75247001 75248000 "*" 2.78284795207639e-08 4.37734975842904e-08
53.8461538461538
"chr7" 75248001 75249000 "*" 9.04570973681018e-10 1.76414389822173e-09 -100
"chr7" 75290001 75291000 "*" 0 0 84.9315068493151
"chr7" 75296001 75297000 "*" 9.58990664656767e-10 1.86473828588299e-09
74.0740740740741
"chr7" 75330001 75331000 "*" 0.000449815378086083 0.000378571446274996
63.6363636363636
"chr7" 75353001 75354000 "*" 1.17794662912729e-13 3.83653245040640e-13 100

"chr7" 75441001 75442000 "*" 2.69007038866675e-13 8.38831022628993e-13 -100
"chr7" 75457001 75458000 "*" 6.30384633382164e-13 1.87665599796749e-12 -100
"chr7" 75574001 75575000 "*" 1.52794177310511e-09 2.87642564232045e-09 100
"chr7" 75582001 75583000 "*" 5.03264097062583e-13 1.51942428521737e-12 -100
"chr7" 75600001 75601000 "*" 1.02362562870439e-13 3.36027280445527e-13
52.3809523809524
"chr7" 75620001 75621000 "*" 3.69082542306387e-12 1.00083695108923e-11
52.8256880733945
"chr7" 75659001 75660000 "*" 2.17381668221606e-13 6.85151076607736e-13 -100
"chr7" 75678001 75679000 "*" 2.08814465718632e-09 3.86232567960463e-09 -100
"chr7" 75798001 75799000 "*" 1.49613654798486e-12 4.26244554429779e-12 -100
"chr7" 75836001 75837000 "*" 8.10462807976364e-15 3.04213344674586e-14
66.6666666666667
"chr7" 75846001 75847000 "*" 1.96005695991541e-05 2.0534467543854e-05 -60
"chr7" 75866001 75867000 "*" 1.04935969358344e-06 1.32845565076529e-06
-53.968253968254
"chr7" 75890001 75891000 "*" 0 0 60.7843137254902
"chr7" 75905001 75906000 "*" 0 0 100
"chr7" 75922001 75923000 "*" 7.54931107238566e-06 8.42480033451236e-06
-69.3877551020408
"chr7" 75926001 75927000 "*" 2.62840407905252e-06 3.14168014490826e-06
-60.4651162790698
"chr7" 75935001 75936000 "*" 0.000118603931205552 0.000109926776654688
61.5384615384615
"chr7" 75976001 75977000 "*" 0 0 -100
"chr7" 75998001 75999000 "*" 1.66549307678565e-09 3.12479266179538e-09
68.9189189189189
"chr7" 76009001 76010000 "*" 0 0 -100
"chr7" 76023001 76024000 "*" 1.73876912867854e-10 3.75704847922646e-10
53.9960561419789
"chr7" 76026001 76027000 "*" 1.31923309609761e-07 1.88369873957834e-07
67.2862453531599
"chr7" 76027001 76028000 "*" 0 0 86.864406779661
"chr7" 76040001 76041000 "*" 0 0 -69.6969696969697
"chr7" 76130001 76131000 "*" 0 0 -88.1578947368421
"chr7" 76624001 76625000 "*" 0 0 -54.0540540540541
"chr7" 77510001 77511000 "*" 1.49383441172546e-08 2.444556877867e-08
-66.6666666666667
"chr7" 77711001 77712000 "*" 2.88710388929303e-10 6.03399264731139e-10 100
"chr7" 78410001 78411000 "*" 7.99236967718997e-08 1.17432296521813e-07
77.1739130434783
"chr7" 78452001 78453000 "*" 2.66453525910038e-14 9.40074020513994e-14 100
"chr7" 78797001 78798000 "*" 1.13140401492018e-08 1.87801380787728e-08 100
"chr7" 79335001 79336000 "*" 3.27522453602569e-11 7.79443598335838e-11 -84
"chr7" 79764001 79765000 "*" 0 0 92.6470588235294
"chr7" 80650001 80651000 "*" 9.99200722162641e-16 4.15382497462808e-15 100
"chr7" 80699001 80700000 "*" 0 0 100
"chr7" 81067001 81068000 "*" 3.4216607325277e-10 7.0790060003635e-10
63.6363636363636
"chr7" 81809001 81810000 "*" 3.6700841921089e-08 5.65530226072258e-08 100
"chr7" 82176001 82177000 "*" 8.7349629751543e-09 1.47505871898511e-08 100
"chr7" 82545001 82546000 "*" 0 0 100

"chr7" 83278001 83279000 "*" 6.3061889044036e-10 1.25756910202723e-09 57.3979591836735
"chr7" 83292001 83293000 "*" 2.2573304221396e-06 2.7265120493757e-06 90.9090909090909
"chr7" 85244001 85245000 "*" 3.6700841921089e-08 5.65530226072258e-08 100
"chr7" 85838001 85839000 "*" 0.000108097719693223 0.000100808725869662 -52.1739130434783
"chr7" 86974001 86975000 "*" 0 0 100
"chr7" 87168001 87169000 "*" 3.90831811358794e-12 1.05610567215048e-11 -100
"chr7" 87229001 87230000 "*" 0 0 83.943661971831
"chr7" 87256001 87257000 "*" 0 0 64.0718562874251
"chr7" 87257001 87258000 "*" 0 0 68.2927472064094
"chr7" 87505001 87506000 "*" 0 0 -54.8148148148148
"chr7" 87974001 87975000 "*" 0 0 100
"chr7" 89840001 89841000 "*" 1.19237952844742e-13 3.8777650116948e-13 -62.1917808219178
"chr7" 89841001 89842000 "*" 0 0 -60.8695652173913
"chr7" 90062001 90063000 "*" 1.58287862728201e-06 1.95525839023886e-06 70.8955223880597
"chr7" 90225001 90226000 "*" 0 0 81.2937986811571
"chr7" 90226001 90227000 "*" 8.27427015792637e-12 2.14091117204921e-11 70.9480122324159
"chr7" 90388001 90389000 "*" 1.61922278313353e-05 1.71897390133207e-05 -51.4285714285714
"chr7" 90396001 90397000 "*" 4.915795842797e-05 4.83987297040465e-05 -58.3333333333333
"chr7" 91763001 91764000 "*" 0 0 73.4028683181226
"chr7" 91764001 91765000 "*" 0 0 83.0188679245283
"chr7" 92462001 92463000 "*" 1.63424829224823e-13 5.24281442455049e-13 -68.3982683982684
"chr7" 92621001 92622000 "*" 3.63445940010365e-11 8.58565209111204e-11 -100
"chr7" 92768001 92769000 "*" 3.95353005888666e-09 7.03928529283733e-09 100
"chr7" 94038001 94039000 "*" 0 0 100
"chr7" 94218001 94219000 "*" 3.07531777821168e-14 1.07626052665081e-13 100
"chr7" 94663001 94664000 "*" 0 0 73.5294117647059
"chr7" 94711001 94712000 "*" 7.00293936239937e-06 7.85615355394095e-06 57.6923076923077
"chr7" 95201001 95202000 "*" 2.02227318224502e-07 2.81656956190631e-07 -66.6666666666667
"chr7" 95401001 95402000 "*" 0 0 82.7977315689981
"chr7" 96030001 96031000 "*" 4.26951363152739e-10 8.72510873516957e-10 -100
"chr7" 96361001 96362000 "*" 2.89337077219454e-06 3.43828475546463e-06 63.6363636363636
"chr7" 96422001 96423000 "*" 1.09686691580624e-05 1.19451401973408e-05 -66.6666666666667
"chr7" 96518001 96519000 "*" 1.37828859436695e-10 3.01445544267661e-10 100
"chr7" 96635001 96636000 "*" 0 0 64.3510702733353
"chr7" 96746001 96747000 "*" 1.28639805263564e-06 1.60933764599525e-06 67.1052631578947
"chr7" 96862001 96863000 "*" 1.20591092667155e-10 2.66075324129686e-10 -100
"chr7" 96893001 96894000 "*" 1.5277158427196e-09 2.87642564232045e-09 100
"chr7" 96976001 96977000 "*" 2.77788437808013e-08 4.36995426069457e-08 90

"chr7" 97178001 97179000 "*" 1.86517468137026e-14 6.70995420767425e-14 -100
"chr7" 97185001 97186000 "*" 1.49880108324396e-14 5.44951848293151e-14 100
"chr7" 97363001 97364000 "*" 1.78426615704552e-08 2.88451106005232e-08 80
"chr7" 97558001 97559000 "*" 5.44009282066327e-15 2.08486137681812e-14 100
"chr7" 97639001 97640000 "*" 7.88830466502688e-08 1.15989087971183e-07
-72.7272727272727
"chr7" 97668001 97669000 "*" 0 0 -73.0769230769231
"chr7" 97714001 97715000 "*" 4.08209022140227e-11 9.57770690255255e-11 -100
"chr7" 97763001 97764000 "*" 0.000183887360524659 0.000165164039707606
58.3333333333333
"chr7" 97887001 97888000 "*" 6.93268023987947e-05 6.66409676902322e-05 -52
"chr7" 97889001 97890000 "*" 0 0 100
"chr7" 97910001 97911000 "*" 0 0 -70.2755905511811
"chr7" 97917001 97918000 "*" 1.08311198898647e-07 1.5646019706886e-07
53.8461538461538
"chr7" 98030001 98031000 "*" 0 0 -100
"chr7" 98062001 98063000 "*" 1.07882591748876e-11 2.74553537455803e-11 -100
"chr7" 98086001 98087000 "*" 6.4152538836737e-10 1.27477697728417e-09 100
"chr7" 98087001 98088000 "*" 0.000231175672664174 0.000204278487663226
54.5454545454545
"chr7" 98127001 98128000 "*" 6.4152538836737e-10 1.27477697728417e-09 100
"chr7" 98154001 98155000 "*" 3.6700841921089e-08 5.65530226072258e-08 -100
"chr7" 98224001 98225000 "*" 4.01313771103418e-09 7.11882087237587e-09 100
"chr7" 98273001 98274000 "*" 2.66753123167041e-05 2.73797260365203e-05
55.9523809523809
"chr7" 98285001 98286000 "*" 5.6156190808565e-12 1.48577674405101e-11
-55.9322033898305
"chr7" 98304001 98305000 "*" 6.66133814775094e-16 2.81595744474255e-15 -100
"chr7" 98315001 98316000 "*" 2.79163895522405e-06 3.32473631338096e-06
58.5365853658537
"chr7" 98341001 98342000 "*" 0 0 100
"chr7" 98383001 98384000 "*" 9.65338919911574e-13 2.80678910656113e-12 100
"chr7" 98426001 98427000 "*" 4.92582776201456e-06 5.65100933800874e-06
-64.2857142857143
"chr7" 98469001 98470000 "*" 0.00137258543519492 0.00106445405043295
53.0434782608696
"chr7" 98473001 98474000 "*" 3.10862446895044e-15 1.22466437093774e-14 100
"chr7" 98580001 98581000 "*" 7.777778421314e-12 2.01783102999799e-11
-57.3333333333333
"chr7" 98638001 98639000 "*" 7.37188088351104e-14 2.4676700929657e-13
94.8717948717949
"chr7" 98639001 98640000 "*" 8.80406858527749e-14 2.91456141063452e-13 100
"chr7" 98744001 98745000 "*" 2.455369241261e-12 6.8003688121118e-12 100
"chr7" 98762001 98763000 "*" 3.63986951690265e-10 7.50142469577035e-10
-69.4444444444444
"chr7" 98766001 98767000 "*" 2.37587727269783e-14 8.44268514944447e-14 -100
"chr7" 98830001 98831000 "*" 5.96443145983727e-08 8.95735478766908e-08
54.5454545454545
"chr7" 98836001 98837000 "*" 0 0 -60.8695652173913
"chr7" 98847001 98848000 "*" 6.98330282489223e-14 2.34267456154807e-13 -100
"chr7" 98850001 98851000 "*" 1.77791781297287e-09 3.3257729002305e-09
-80.2197802197802

"chr7" 98971001 98972000 "*" 0 0 60
"chr7" 98982001 98983000 "*" 8.90316709245553e-10 1.74033267882731e-09
62.7906976744186
"chr7" 98989001 98990000 "*" 6.59550958292954e-09 1.13456194382773e-08 -100
"chr7" 99037001 99038000 "*" 0 0 83.1168831168831
"chr7" 99065001 99066000 "*" 0 0 -100
"chr7" 99069001 99070000 "*" 1.11022302462516e-16 5.03662826618488e-16
-84.7222222222222
"chr7" 99145001 99146000 "*" 8.88178419700125e-16 3.70670032207938e-15 -100
"chr7" 99175001 99176000 "*" 1.13140401492018e-08 1.87801380787728e-08 -100
"chr7" 99485001 99486000 "*" 1.95066851560455e-10 4.192858786069e-10
54.2857142857143
"chr7" 99541001 99542000 "*" 3.58120370212989e-05 3.60126966183746e-05
-61.5384615384615
"chr7" 99574001 99575000 "*" 3.72545756543996e-06 4.3519872100983e-06
-73.6842105263158
"chr7" 99585001 99586000 "*" 2.75837870061801e-05 2.82530803747995e-05
66.6666666666667
"chr7" 99589001 99590000 "*" 7.0006535457523e-08 1.03695832512054e-07 -100
"chr7" 99657001 99658000 "*" 0 0 100
"chr7" 99699001 99700000 "*" 0 0 -100
"chr7" 99700001 99701000 "*" 0 0 100
"chr7" 99728001 99729000 "*" 0 0 -100
"chr7" 99746001 99747000 "*" 0 0 100
"chr7" 99755001 99756000 "*" 4.44089209850063e-16 1.91071758245033e-15
70.1754385964912
"chr7" 99765001 99766000 "*" 2.10123767274695e-08 3.3539652900685e-08
-66.5861513687601
"chr7" 99770001 99771000 "*" 2.15125472990962e-10 4.58126659943874e-10 -100
"chr7" 99867001 99868000 "*" 8.01666433236647e-09 1.36392368970109e-08 100
"chr7" 99972001 99973000 "*" 9.09275987837077e-11 2.03642018108493e-10
53.6842105263158
"chr7" 99996001 99997000 "*" 7.0006535457523e-08 1.03695832512054e-07 100
"chr7" 100043001 100044000 "*" 2.00227675550835e-08 3.20459176617961e-08 -100
"chr7" 100048001 100049000 "*" 0 0 -100
"chr7" 100077001 100078000 "*" 9.67793313266796e-08 1.40685931288877e-07
53.3898305084746
"chr7" 100161001 100162000 "*" 0 0 100
"chr7" 100164001 100165000 "*" 7.18592296777842e-09 1.23102247942501e-08
51.9230769230769
"chr7" 100185001 100186000 "*" 6.77335965093562e-12 1.76897011246462e-11 -100
"chr7" 100192001 100193000 "*" 1.98528951056431e-08 3.19051889717718e-08
55.5555555555556
"chr7" 100226001 100227000 "*" 2.27526614993145e-07 3.14837392572814e-07 -65
"chr7" 100228001 100229000 "*" 4.88929452480136e-10 9.9168406590276e-10
-82.5396825396825
"chr7" 100230001 100231000 "*" 0 0 61.9911012235818
"chr7" 100239001 100240000 "*" 1.98365768255826e-11 4.85612402473442e-11 -100
"chr7" 100289001 100290000 "*" 7.80486786311485e-13 2.30149354573478e-12
-84.1328413284133
"chr7" 100292001 100293000 "*" 0 0 -88.8888888888889
"chr7" 100312001 100313000 "*" 1.4873571501095e-06 1.84447733364305e-06

73.1707317073171
"chr7" 100346001 100347000 "*" 4.44089209850063e-16 1.91071758245033e-15 100
"chr7" 100366001 100367000 "*" 4.8664294816092e-11 1.13145908161915e-10
59.1549295774648
"chr7" 100370001 100371000 "*" 9.04570973681018e-10 1.76414389822173e-09 100
"chr7" 100373001 100374000 "*" 2.39687045766601e-08 3.79822661439144e-08
52.1739130434783
"chr7" 100387001 100388000 "*" 1.17702637125738e-06 1.48016917543092e-06
-91.6666666666667
"chr7" 100392001 100393000 "*" 1.52688972576698e-12 4.33938755962772e-12
-61.9047619047619
"chr7" 100427001 100428000 "*" 2.15125472990962e-10 4.58126659943874e-10 -100
"chr7" 100435001 100436000 "*" 0 0 -79.5876288659794
"chr7" 100486001 100487000 "*" 0 0 87.6712328767123
"chr7" 100493001 100494000 "*" 0 0 55.3372626064178
"chr7" 100494001 100495000 "*" 0 0 62.5450798557445
"chr7" 100500001 100501000 "*" 0 0 100
"chr7" 100518001 100519000 "*" 6.37490060739765e-13 1.89496793587877e-12 100
"chr7" 100539001 100540000 "*" 2.64951482975562e-09 4.82186011375752e-09 100
"chr7" 100553001 100554000 "*" 1.07882591748876e-11 2.74553537455803e-11 -100
"chr7" 100631001 100632000 "*" 1.37828859436695e-10 3.01445544267661e-10 -100
"chr7" 100646001 100647000 "*" 2.33146835171283e-15 9.32818831833246e-15
56.9587337478802
"chr7" 100648001 100649000 "*" 2.91028312560115e-11 6.97590507139978e-11 -100
"chr7" 100685001 100686000 "*" 4.78841410966879e-11 1.11468187156588e-10 -100
"chr7" 100694001 100695000 "*" 1.99684713209081e-12 5.59417005570418e-12
-52.9411764705882
"chr7" 100738001 100739000 "*" 0 0 57.6309067688378
"chr7" 100757001 100758000 "*" 2.33590924381133e-13 7.3418676838825e-13 -100
"chr7" 100764001 100765000 "*" 0 0 81.5789473684211
"chr7" 100809001 100810000 "*" 0 0 81.2777284826974
"chr7" 100825001 100826000 "*" 9.88312023331162e-08 1.43474171574152e-07
60.8108108108108
"chr7" 100833001 100834000 "*" 0 0 100
"chr7" 100838001 100839000 "*" 3.40125705378114e-10 7.03784073980162e-10 -100
"chr7" 100991001 100992000 "*" 1.39779633423487e-07 1.98463037958589e-07 100
"chr7" 101050001 101051000 "*" 9.71063229826541e-11 2.16781365050398e-10 -100
"chr7" 101061001 101062000 "*" 3.99791311167519e-13 1.22027792394232e-12 -100
"chr7" 101076001 101077000 "*" 0 0 -92.3076923076923
"chr7" 101078001 101079000 "*" 1.32558408694194e-11 3.33056084662582e-11 56
"chr7" 101096001 101097000 "*" 2.8988135904906e-05 2.95955482172808e-05
-56.6666666666667
"chr7" 101104001 101105000 "*" 1.11022302462516e-16 5.03662826618488e-16 100
"chr7" 101131001 101132000 "*" 0.000105894805935924 9.88981208988924e-05
-54.8387096774194
"chr7" 101141001 101142000 "*" 6.33715302456039e-13 1.88610013222453e-12
68.1818181818182
"chr7" 101148001 101149000 "*" 1.14092513214814e-08 1.89300825733629e-08
-82.0512820512821
"chr7" 101176001 101177000 "*" 0 0 -100
"chr7" 101182001 101183000 "*" 2.43005615629954e-09 4.45014222280278e-09 -100
"chr7" 101193001 101194000 "*" 6.28386231937839e-14 2.12005864521753e-13 -60

"chr7" 101200001 101201000 "*" 5.818110966338e-07 7.63127325060352e-07 64.3835616438356
"chr7" 101257001 101258000 "*" 7.0006535457523e-08 1.03695832512054e-07 -100
"chr7" 101269001 101270000 "*" 7.88258347483861e-15 2.96145301916012e-14 -53.4883720930233
"chr7" 101363001 101364000 "*" 1.46913487153455e-07 2.0800406109474e-07 81.8181818181818
"chr7" 101399001 101400000 "*" 7.0006535457523e-08 1.03695832512054e-07 100
"chr7" 101511001 101512000 "*" 4.53110349241115e-09 7.99173517127516e-09 -78.2608695652174
"chr7" 101586001 101587000 "*" 0 0 -100
"chr7" 101627001 101628000 "*" 0 0 100
"chr7" 101706001 101707000 "*" 4.86832796298131e-13 1.47290845675726e-12 -100
"chr7" 101740001 101741000 "*" 0 0 -88.8888888888889
"chr7" 101862001 101863000 "*" 0 0 -100
"chr7" 101920001 101921000 "*" 0 0 100
"chr7" 101924001 101925000 "*" 3.68934893746342e-09 6.60472390900594e-09 72.289156626506
"chr7" 101934001 101935000 "*" 1.4521717162097e-13 4.6831960578311e-13 -53.9325842696629
"chr7" 101945001 101946000 "*" 1.20281562487889e-12 3.46585342181251e-12 100
"chr7" 101955001 101956000 "*" 9.06273616041453e-07 1.15739767942151e-06 52.3809523809524
"chr7" 102036001 102037000 "*" 6.52733422867868e-12 1.71146951718423e-11 -100
"chr7" 102073001 102074000 "*" 0 0 -53.1583548868894
"chr7" 102074001 102075000 "*" 5.46018452673991e-10 1.10035808895315e-09 66.6666666666667
"chr7" 102137001 102138000 "*" 4.35971455825523e-07 5.8071523924332e-07 -57.1428571428571
"chr7" 102233001 102234000 "*" 2.02784055125527e-06 2.46523636443825e-06 -50.5411255411255
"chr7" 102236001 102237000 "*" 8.65973959207622e-15 3.24130830964561e-14 52.4752475247525
"chr7" 102385001 102386000 "*" 1.34353794756237e-09 2.55598898770358e-09 -80
"chr7" 102615001 102616000 "*" 0 0 100
"chr7" 102742001 102743000 "*" 0 0 100
"chr7" 102920001 102921000 "*" 0 0 51.469620819409
"chr7" 103016001 103017000 "*" 0 0 -100
"chr7" 103102001 103103000 "*" 3.04324343503026e-11 7.26599935673121e-11 100
"chr7" 103244001 103245000 "*" 5.48638023900594e-10 1.10374357336421e-09 -100
"chr7" 103631001 103632000 "*" 1.51205371412555e-06 1.87332076018969e-06 67.7966101694915
"chr7" 103871001 103872000 "*" 7.26436569564726e-07 9.40094283678952e-07 -55.9139784946237
"chr7" 104005001 104006000 "*" 6.1780917180787e-05 5.98962110119704e-05 65.7407407407407
"chr7" 104455001 104456000 "*" 8.90644368256588e-08 1.30047202396328e-07 -79.6116504854369
"chr7" 104531001 104532000 "*" 2.52725618210548e-11 6.12432958532577e-11 -83.8709677419355
"chr7" 104624001 104625000 "*" 0 0 -60.9177215189873
"chr7" 105048001 105049000 "*" 1.97615809753859e-05 2.06905910202848e-05

64.7058823529412
"chr7" 105134001 105135000 "*" 6.4152538836737e-10 1.27477697728417e-09 100
"chr7" 105222001 105223000 "*" 0.000188643712552339 0.000169112219382544
56.4197530864197
"chr7" 105242001 105243000 "*" 1.12687636999453e-13 3.68543487142567e-13 100
"chr7" 105244001 105245000 "*" 6.19726492345762e-12 1.63056411736457e-11
74.3589743589744
"chr7" 105317001 105318000 "*" 4.02167188440217e-12 1.08266700497926e-11 100
"chr7" 105331001 105332000 "*" 2.38575481272818e-05 2.46816531377115e-05
-66.6666666666667
"chr7" 105374001 105375000 "*" 1.13140401492018e-08 1.87801380787728e-08 100
"chr7" 105418001 105419000 "*" 0 0 -100
"chr7" 105618001 105619000 "*" 8.43347613965761e-12 2.17859001990012e-11 100
"chr7" 105635001 105636000 "*" 3.91549989540163e-05 3.91281205357464e-05
-52.3809523809524
"chr7" 105669001 105670000 "*" 0 0 100
"chr7" 105753001 105754000 "*" 1.67299207820548e-08 2.71377429441055e-08 -100
"chr7" 105841001 105842000 "*" 8.7349629751543e-09 1.47505871898511e-08 100
"chr7" 106027001 106028000 "*" 0 0 100
"chr7" 106349001 106350000 "*" 4.35473335169689e-09 7.69481973450083e-09
88.8888888888889
"chr7" 106478001 106479000 "*" 4.01313771103418e-09 7.11882087237587e-09 100
"chr7" 106613001 106614000 "*" 0 0 53.5714285714286
"chr7" 106684001 106685000 "*" 0 0 85.7618253734759
"chr7" 106723001 106724000 "*" 2.43005615629954e-09 4.45014222280278e-09 100
"chr7" 107302001 107303000 "*" 0 0 65.7739871451255
"chr7" 107470001 107471000 "*" 2.88657986402541e-15 1.14155613124573e-14
51.1111111111111
"chr7" 107726001 107727000 "*" 9.41469124882133e-14 3.10868561210786e-13 -100
"chr7" 107756001 107757000 "*" 5.33495114529714e-06 6.09237962218305e-06
66.6666666666667
"chr7" 108096001 108097000 "*" 0 0 57.6344391380541
"chr7" 108634001 108635000 "*" 5.648789203061e-07 7.42155605113794e-07
-55.1724137931034
"chr7" 108950001 108951000 "*" 4.90718576884319e-14 1.6794399166207e-13 100
"chr7" 110715001 110716000 "*" 9.65729496371637e-10 1.87255912493232e-09 100
"chr7" 112255001 112256000 "*" 5.02714998518172e-07 6.64281343138596e-07 -90
"chr7" 112580001 112581000 "*" 3.33066907387547e-16 1.4495649018245e-15 100
"chr7" 112625001 112626000 "*" 4.01313771103418e-09 7.11882087237587e-09 100
"chr7" 112790001 112791000 "*" 7.34312610717325e-12 1.90934321207989e-11 -90
"chr7" 113258001 113259000 "*" 4.44089209850063e-16 1.91071758245033e-15 -100
"chr7" 113723001 113724000 "*" 0.000567718558392993 0.000469453324997592
50.7142857142857
"chr7" 114025001 114026000 "*" 4.99900121297969e-12 1.32921056339624e-11 100
"chr7" 115928001 115929000 "*" 6.24654217240561e-10 1.2462108191303e-09 100
"chr7" 116356001 116357000 "*" 1.11022302462516e-16 5.03662826618488e-16
-82.1052631578947
"chr7" 116445001 116446000 "*" 1.11022302462516e-15 4.59817122935606e-15 100
"chr7" 116572001 116573000 "*" 6.60582699651968e-14 2.22176301799e-13 -100
"chr7" 116889001 116890000 "*" 1.77635683940025e-15 7.19870740878856e-15 -100
"chr7" 117365001 117366000 "*" 4.73234496034536e-09 8.30627300264826e-09 100
"chr7" 117432001 117433000 "*" 5.5330668036202e-07 7.27639394821453e-07

76.1904761904762
"chr7" 117513001 117514000 "*" 0 0 58.6497890295359
"chr7" 117699001 117700000 "*" 2.88710388929303e-10 6.03399264731139e-10 100
"chr7" 118249001 118250000 "*" 3.10071932249079e-05 3.15124728670996e-05
58.6206896551724
"chr7" 118828001 118829000 "*" 9.61415627542817e-06 1.05670450583329e-05
66.6666666666667
"chr7" 118829001 118830000 "*" 1.10111923912193e-07 1.58907290044317e-07 60
"chr7" 119364001 119365000 "*" 6.37482022725067e-09 1.10212365258961e-08
78.1818181818182
"chr7" 119649001 119650000 "*" 0.000333336288153019 0.000286777321730898 52
"chr7" 119687001 119688000 "*" 0 0 100
"chr7" 119913001 119914000 "*" 0 0 53.6358137374567
"chr7" 121504001 121505000 "*" 6.92287338566189e-11 1.57605630124247e-10 -100
"chr7" 121817001 121818000 "*" 1.66533453693773e-14 6.02507639546739e-14
-66.6666666666667
"chr7" 121945001 121946000 "*" 0 0 69.9139865370232
"chr7" 122127001 122128000 "*" 0.00139193432787144 0.00107848655779008
-52.2144522144522
"chr7" 122569001 122570000 "*" 3.56292773062705e-11 8.43626766564336e-11 -56
"chr7" 122676001 122677000 "*" 1.15339349182619e-09 2.21004254568532e-09 95
"chr7" 123635001 123636000 "*" 3.40125705378114e-10 7.03784073980162e-10 100
"chr7" 123636001 123637000 "*" 2.88779000712225e-11 6.92935809466068e-11 100
"chr7" 123672001 123673000 "*" 0 0 51.0155736935923
"chr7" 124406001 124407000 "*" 1.31659621094293e-07 1.88012207853257e-07
54.0983606557377
"chr7" 124674001 124675000 "*" 2.02327044007689e-12 5.65300384792621e-12 -100
"chr7" 124721001 124722000 "*" 2.02327044007689e-12 5.65300384792621e-12 100
"chr7" 124939001 124940000 "*" 6.30384633382164e-13 1.87665599796749e-12 100
"chr7" 124955001 124956000 "*" 7.0006535457523e-08 1.03695832512054e-07 100
"chr7" 126330001 126331000 "*" 7.71940883315114e-06 8.6029809164006e-06
58.8071348940914
"chr7" 126597001 126598000 "*" 0 0 -72.972972972973
"chr7" 126895001 126896000 "*" 0 0 -100
"chr7" 126935001 126936000 "*" 3.3744118610457e-12 9.1855963533528e-12
66.6666666666667
"chr7" 127111001 127112000 "*" 2.50910403565285e-14 8.87903196465237e-14 100
"chr7" 127291001 127292000 "*" 3.15194537137131e-11 7.51370378959519e-11
-54.8872180451128
"chr7" 127702001 127703000 "*" 6.24654217240561e-10 1.2462108191303e-09 100
"chr7" 127761001 127762000 "*" 4.10307059439674e-08 6.28938130251532e-08
57.6923076923077
"chr7" 127788001 127789000 "*" 1.38590361409285e-10 3.02706178892116e-10 -100
"chr7" 127807001 127808000 "*" 0 0 59.1547266139657
"chr7" 127843001 127844000 "*" 0 0 100
"chr7" 127872001 127873000 "*" 9.08108033215171e-11 2.03407753381119e-10 100
"chr7" 127897001 127898000 "*" 9.04570973681018e-10 1.76414389822173e-09 100
"chr7" 127969001 127970000 "*" 0 0 100
"chr7" 128044001 128045000 "*" 5.285394344412e-11 1.22310092143078e-10
-88.6363636363636
"chr7" 128096001 128097000 "*" 4.44089209850063e-16 1.91071758245033e-15 -100
"chr7" 128114001 128115000 "*" 1.56863411149288e-12 4.4469621057462e-12 -100

"chr7" 128441001 128442000 "*" 0 0 -100
"chr7" 128502001 128503000 "*" 0 0 -100
"chr7" 128514001 128515000 "*" 0 0 63.3333333333333
"chr7" 128518001 128519000 "*" 3.54449802841827e-12 9.63130374931744e-12
-55.8139534883721
"chr7" 128530001 128531000 "*" 0 0 -53.2497781721384
"chr7" 128558001 128559000 "*" 1.96644922567657e-11 4.82930612417392e-11 -100
"chr7" 128785001 128786000 "*" 0 0 63.2911392405063
"chr7" 128814001 128815000 "*" 2.08995487582797e-10 4.46944006972663e-10 -100
"chr7" 128828001 128829000 "*" 0 0 50.0649823248077
"chr7" 129150001 129151000 "*" 0 0 100
"chr7" 129156001 129157000 "*" 3.55024902823908e-05 3.57199333322631e-05
55.7377049180328
"chr7" 129161001 129162000 "*" 6.59550958292954e-09 1.13456194382773e-08 100
"chr7" 129168001 129169000 "*" 2.00227675550835e-08 3.20459176617961e-08 100
"chr7" 129173001 129174000 "*" 8.34608049338215e-10 1.63669244542139e-09
-56.5217391304348
"chr7" 129300001 129301000 "*" 2.43005615629954e-09 4.45014222280278e-09 -100
"chr7" 129303001 129304000 "*" 4.25381951885129e-12 1.14320727179292e-11
52.1739130434783
"chr7" 129367001 129368000 "*" 1.19904086659517e-14 4.40901388691796e-14 100
"chr7" 129375001 129376000 "*" 0 0 -75
"chr7" 129413001 129414000 "*" 1.13140401492018e-08 1.87801380787728e-08 100
"chr7" 129436001 129437000 "*" 2.62088654667814e-07 3.59779054681028e-07
76.9230769230769
"chr7" 129593001 129594000 "*" 1.35447209004269e-14 4.94674708073053e-14 -100
"chr7" 129608001 129609000 "*" 2.15070516951243e-09 3.9725885757664e-09
-57.6923076923077
"chr7" 129625001 129626000 "*" 4.18887147191072e-13 1.2739946038722e-12 100
"chr7" 129691001 129692000 "*" 8.57092175010621e-13 2.51149198099779e-12 100
"chr7" 129845001 129846000 "*" 0 0 87.9120879120879
"chr7" 129950001 129951000 "*" 6.37490060739765e-13 1.89496793587877e-12 -100
"chr7" 129985001 129986000 "*" 0.000451115314036077 0.000379477851858741
-52.1739130434783
"chr7" 130025001 130026000 "*" 1.31211004283927e-08 2.16253763756563e-08
-70.2702702702703
"chr7" 130324001 130325000 "*" 0 0 92.3076923076923
"chr7" 130352001 130353000 "*" 4.10560474506383e-13 1.25200298811809e-12
95.8333333333333
"chr7" 130472001 130473000 "*" 4.71134242729931e-12 1.257615036806e-11 100
"chr7" 130487001 130488000 "*" 1.96644922567657e-11 4.82930612417392e-11 100
"chr7" 130523001 130524000 "*" 1.07882591748876e-11 2.74553537455803e-11 -100
"chr7" 130527001 130528000 "*" 0 0 -100
"chr7" 130581001 130582000 "*" 6.88338275267597e-15 2.60518659957509e-14 -100
"chr7" 130582001 130583000 "*" 1.6501991884077e-08 2.68735989791713e-08
-61.1111111111111
"chr7" 130583001 130584000 "*" 6.92946419336149e-05 6.66169634886293e-05
-50.3649635036496
"chr7" 130586001 130587000 "*" 0 0 -100
"chr7" 130626001 130627000 "*" 8.80406858527749e-14 2.91456141063452e-13 100
"chr7" 130746001 130747000 "*" 1.5277158427196e-09 2.87642564232045e-09 -100
"chr7" 130792001 130793000 "*" 0 0 88.780487804878

"chr7" 130851001 130852000 "*" 2.455369241261e-12 6.8003688121118e-12 100
"chr7" 131211001 131212000 "*" 5.48638023900594e-10 1.10374357336421e-09 100
"chr7" 131242001 131243000 "*" 0 0 71.8037214885954
"chr7" 131269001 131270000 "*" 2.08614236996141e-11 5.09698923781446e-11
66.6666666666667
"chr7" 131297001 131298000 "*" 3.36302361403362e-07 4.55012026436444e-07
-58.3333333333333
"chr7" 131308001 131309000 "*" 1.37744875505774e-08 2.26351518554286e-08
67.2131147540984
"chr7" 131309001 131310000 "*" 1.26453902904444e-09 2.41208143008691e-09
72.5490196078431
"chr7" 131329001 131330000 "*" 6.20614670765463e-14 2.09451667576313e-13
-88.034188034188
"chr7" 131338001 131339000 "*" 1.07360369303588e-05 1.17076970499658e-05
-58.3333333333333
"chr7" 131374001 131375000 "*" 9.02562580229471e-10 1.76323694103923e-09
-56.7796610169492
"chr7" 131627001 131628000 "*" 7.55798055762646e-06 8.43366888574497e-06
68.9393939393939
"chr7" 131761001 131762000 "*" 3.61932706027801e-14 1.25777907157994e-13 -100
"chr7" 131791001 131792000 "*" 2.43005615629954e-09 4.45014222280278e-09 -100
"chr7" 131837001 131838000 "*" 8.01666433236647e-09 1.36392368970109e-08 100
"chr7" 131846001 131847000 "*" 0 0 -92.9032258064516
"chr7" 131886001 131887000 "*" 0 0 -100
"chr7" 131977001 131978000 "*" 6.60582699651968e-14 2.22176301799e-13 100
"chr7" 132072001 132073000 "*" 9.30270600640881e-06 1.02464660737553e-05
-57.4468085106383
"chr7" 132083001 132084000 "*" 4.2854608750531e-14 1.47617059233354e-13
61.7732558139535
"chr7" 132245001 132246000 "*" 1.11022302462516e-16 5.03662826618488e-16
59.9707602339181
"chr7" 132275001 132276000 "*" 1.25284005392245e-10 2.75649590272477e-10 100
"chr7" 132340001 132341000 "*" 2.97917526871361e-05 3.0356175174783e-05 61
"chr7" 132435001 132436000 "*" 0 0 100
"chr7" 132774001 132775000 "*" 2.08814465718632e-09 3.86232567960463e-09 100
"chr7" 132807001 132808000 "*" 1.21597316471433e-06 1.5259025508738e-06
-70.5882352941177
"chr7" 132917001 132918000 "*" 3.6700841921089e-08 5.65530226072258e-08 100
"chr7" 133491001 133492000 "*" 2.50910403565285e-14 8.87903196465237e-14 100
"chr7" 133580001 133581000 "*" 4.01313771103418e-09 7.11882087237587e-09 100
"chr7" 133630001 133631000 "*" 0 0 -66.6666666666667
"chr7" 133819001 133820000 "*" 3.40125705378114e-10 7.03784073980162e-10 100
"chr7" 133895001 133896000 "*" 1.0900891889154e-07 1.57413554003421e-07
-52.1739130434783
"chr7" 134032001 134033000 "*" 4.01313771103418e-09 7.11882087237587e-09 100
"chr7" 134880001 134881000 "*" 3.42845474810005e-06 4.02641014303533e-06
68.5714285714286
"chr7" 134945001 134946000 "*" 0 0 100
"chr7" 135004001 135005000 "*" 5.51780843238703e-14 1.87243792572872e-13 100
"chr7" 135020001 135021000 "*" 0.000201461795166558 0.000179722257706492 56
"chr7" 135251001 135252000 "*" 5.7065463465733e-14 1.93318145576218e-13 100
"chr7" 135376001 135377000 "*" 4.26951363152739e-10 8.72510873516957e-10 100

"chr7" 135433001 135434000 "*" 0 0 100
"chr7" 135477001 135478000 "*" 6.4152538836737e-10 1.27477697728417e-09 -100
"chr7" 135514001 135515000 "*" 1.52466927971773e-12 4.33322475981408e-12 100
"chr7" 135690001 135691000 "*" 4.90718576884319e-14 1.6794399166207e-13 -100
"chr7" 136515001 136516000 "*" 8.3882012447134e-11 1.88922989503423e-10 100
"chr7" 136550001 136551000 "*" 0 0 -100
"chr7" 136644001 136645000 "*" 9.80763359414993e-10 1.8980032424715e-09 -100
"chr7" 136778001 136779000 "*" 2.39657070688626e-06 2.8828775643494e-06
67.3469387755102
"chr7" 137150001 137151000 "*" 9.52204174803351e-05 8.95780133799817e-05
59.2592592592593
"chr7" 137177001 137178000 "*" 3.95353005888666e-09 7.03928529283733e-09 -100
"chr7" 137194001 137195000 "*" 2.1094237467878e-15 8.47434540879246e-15 100
"chr7" 137249001 137250000 "*" 2.30567689407657e-06 2.78112115402733e-06
58.7628865979381
"chr7" 137687001 137688000 "*" 0 0 -100
"chr7" 137690001 137691000 "*" 2.41741368378623e-05 2.49875818383709e-05
-62.962962962963
"chr7" 137852001 137853000 "*" 1.13140401492018e-08 1.87801380787728e-08 -100
"chr7" 137873001 137874000 "*" 0 0 100
"chr7" 138305001 138306000 "*" 1.4772116863071e-10 3.21689080917524e-10
-57.5757575757576
"chr7" 138350001 138351000 "*" 3.6700841921089e-08 5.65530226072258e-08 -100
"chr7" 138427001 138428000 "*" 1.37828859436695e-10 3.01445544267661e-10 100
"chr7" 138432001 138433000 "*" 1.65487579195656e-09 3.10536155804828e-09
-86.2745098039216
"chr7" 138438001 138439000 "*" 0 0 -80.4347826086957
"chr7" 138470001 138471000 "*" 2.77555756156289e-15 1.10015415195978e-14 100
"chr7" 138481001 138482000 "*" 1.54630752646767e-11 3.85079502648228e-11 -100
"chr7" 138486001 138487000 "*" 0 0 -100
"chr7" 138520001 138521000 "*" 3.90831811358794e-12 1.05610567215048e-11 -100
"chr7" 138646001 138647000 "*" 2.00227675550835e-08 3.20459176617961e-08 -100
"chr7" 138650001 138651000 "*" 1.11022302462516e-16 5.03662826618488e-16
55.5555555555556
"chr7" 138719001 138720000 "*" 5.7065463465733e-14 1.93318145576218e-13 -100
"chr7" 139024001 139025000 "*" 0 0 100
"chr7" 139142001 139143000 "*" 1.98124849859482e-10 4.25157611354621e-10 100
"chr7" 139167001 139168000 "*" 0 0 50.7120276983291
"chr7" 139168001 139169000 "*" 0 0 53.2132311502346
"chr7" 139335001 139336000 "*" 1.39779633423487e-07 1.98463037958589e-07 100
"chr7" 139517001 139518000 "*" 0 0 59.4076655052265
"chr7" 139545001 139546000 "*" 4.73234496034536e-09 8.30627300264826e-09 100
"chr7" 139629001 139630000 "*" 2.17316653561284e-09 4.01194447718549e-09
-58.0645161290323
"chr7" 139657001 139658000 "*" 0 0 100
"chr7" 139707001 139708000 "*" 3.34128893442198e-08 5.19772864618523e-08 -100
"chr7" 139713001 139714000 "*" 2.83590713134885e-08 4.45820386216971e-08
52.2727272727273
"chr7" 139922001 139923000 "*" 0 0 100
"chr7" 139928001 139929000 "*" 1.61604063464438e-12 4.5759121090749e-12
-95.6521739130435
"chr7" 139932001 139933000 "*" 0 0 -62.7269426289034

"chr7" 139956001 139957000 "*" 6.59550958292954e-09 1.13456194382773e-08 -100
"chr7" 139981001 139982000 "*" 1.66992206351857e-06 2.05480552961511e-06
-63.3333333333333
"chr7" 140209001 140210000 "*" 4.32209823486573e-12 1.15935290479641e-11 100
"chr7" 140229001 140230000 "*" 5.6362137179633e-11 1.29963270300065e-10 100
"chr7" 140233001 140234000 "*" 0 0 -68.1528662420382
"chr7" 140234001 140235000 "*" 1.49613654798486e-12 4.26244554429779e-12 -100
"chr7" 140260001 140261000 "*" 8.58968451922237e-12 2.213554983903e-11 -100
"chr7" 140270001 140271000 "*" 5.1281201507436e-13 1.54461625452838e-12 100
"chr7" 140297001 140298000 "*" 1.37828859436695e-10 3.01445544267661e-10 100
"chr7" 140310001 140311000 "*" 9.80763359414993e-10 1.8980032424715e-09 -100
"chr7" 140342001 140343000 "*" 0.000672965912057566 0.000549698110587813 -52
"chr7" 140352001 140353000 "*" 2.77555756156289e-15 1.10015415195978e-14
-52.3809523809524
"chr7" 140361001 140362000 "*" 4.28180813027979e-09 7.57125434329492e-09
80.8510638297872
"chr7" 140387001 140388000 "*" 2.98184810176849e-11 7.14067275232606e-11 -76
"chr7" 140531001 140532000 "*" 4.08209022140227e-11 9.57770690255255e-11 -100
"chr7" 140714001 140715000 "*" 1.10144116050037e-10 2.44506149738735e-10
55.2123552123552
"chr7" 140771001 140772000 "*" 0 0 53.8956887102667
"chr7" 140861001 140862000 "*" 0 0 75.7281553398058
"chr7" 140880001 140881000 "*" 3.33066907387547e-16 1.4495649018245e-15 100
"chr7" 140901001 140902000 "*" 4.61959783659704e-06 5.3205598540144e-06
52.3809523809524
"chr7" 141103001 141104000 "*" 1.25469645784193e-09 2.39423784078678e-09
88.2352941176471
"chr7" 141147001 141148000 "*" 9.48424341062903e-07 1.20745624368669e-06
-53.6585365853659
"chr7" 141400001 141401000 "*" 0 0 90.5172413793103
"chr7" 141485001 141486000 "*" 7.7715611723761e-16 3.26213507634405e-15
66.6666666666667
"chr7" 141576001 141577000 "*" 2.08995487582797e-10 4.46944006972663e-10 100
"chr7" 141946001 141947000 "*" 2.02327044007689e-12 5.65300384792621e-12 -100
"chr7" 142143001 142144000 "*" 8.35800011422805e-08 1.22526064720153e-07 -75
"chr7" 142240001 142241000 "*" 1.13140401492018e-08 1.87801380787728e-08 -100
"chr7" 142334001 142335000 "*" 0 0 95.6331877729258
"chr7" 142462001 142463000 "*" 3.6700841921089e-08 5.65530226072258e-08 100
"chr7" 142487001 142488000 "*" 4.01313771103418e-09 7.11882087237587e-09 100
"chr7" 142547001 142548000 "*" 0 0 54.469305794607
"chr7" 142560001 142561000 "*" 2.88710388929303e-10 6.03399264731139e-10 100
"chr7" 142586001 142587000 "*" 6.97020330342468e-10 1.37926735835552e-09
-92.8571428571429
"chr7" 142589001 142590000 "*" 3.9190872769268e-14 1.35416418933905e-13 100
"chr7" 142748001 142749000 "*" 7.0006535457523e-08 1.03695832512054e-07 100
"chr7" 142768001 142769000 "*" 1.48087875295744e-10 3.22075573380618e-10 100
"chr7" 143017001 143018000 "*" 7.12547354453363e-10 1.40825483981431e-09 59.375
"chr7" 143175001 143176000 "*" 1.12458486967171e-09 2.15740815043925e-09 100
"chr7" 143826001 143827000 "*" 2.22044604925031e-16 9.81641919380259e-16 100
"chr7" 143892001 143893000 "*" 0 0 -58.6102719033233
"chr7" 144077001 144078000 "*" 5.295763827462e-14 1.80214784640623e-13 -100
"chr7" 144129001 144130000 "*" 1.11022302462516e-15 4.59817122935606e-15 -100

"chr7" 144163001 144164000 "*" 1.99840144432528e-12 5.59476580335607e-12 100
"chr7" 144782001 144783000 "*" 1.67299207820548e-08 2.71377429441055e-08 100
"chr7" 145663001 145664000 "*" 5.7065463465733e-14 1.93318145576218e-13 100
"chr7" 146203001 146204000 "*" 1.92946192356658e-09 3.59193011611483e-09 -100
"chr7" 147220001 147221000 "*" 0 0 100
"chr7" 147368001 147369000 "*" 5.58502353253942e-08 8.41879077739806e-08
-73.6842105263158
"chr7" 147498001 147499000 "*" 2.35367281220533e-14 8.37555800557029e-14
54.6511627906977
"chr7" 147718001 147719000 "*" 5.7969137756686e-07 7.60454651384167e-07
-62.3188405797101
"chr7" 147804001 147805000 "*" 3.56775498033812e-10 7.35898629809829e-10 -100
"chr7" 147850001 147851000 "*" 8.7349629751543e-09 1.47505871898511e-08 -100
"chr7" 147906001 147907000 "*" 3.06584300058255e-07 4.16988931731194e-07
-57.8947368421053
"chr7" 148067001 148068000 "*" 9.34051801460001e-08 1.36032848893872e-07
-52.3809523809524
"chr7" 148162001 148163000 "*" 5.1223403296774e-10 1.03532645132705e-09
-73.9130434782609
"chr7" 148273001 148274000 "*" 1.98325800226939e-11 4.85612402473442e-11 100
"chr7" 148381001 148382000 "*" 0 0 100
"chr7" 148384001 148385000 "*" 5.6362137179633e-11 1.29963270300065e-10 -100
"chr7" 148436001 148437000 "*" 1.93720595120794e-11 4.76309924836636e-11 100
"chr7" 148611001 148612000 "*" 3.17809112360123e-11 7.57451532425199e-11
73.3333333333333
"chr7" 148655001 148656000 "*" 1.11022302462516e-16 5.03662826618488e-16
-84.6153846153846
"chr7" 148661001 148662000 "*" 7.07358329687935e-08 1.04697992268079e-07
81.8181818181818
"chr7" 148663001 148664000 "*" 1.73352405763438e-08 2.80628728469971e-08
-87.2340425531915
"chr7" 148692001 148693000 "*" 5.88795574296697e-07 7.71742279883976e-07 -75
"chr7" 148700001 148701000 "*" 1.60982338570648e-13 5.16743870241079e-13
53.921568627451
"chr7" 148821001 148822000 "*" 0 0 -100
"chr7" 148883001 148884000 "*" 1.17905685215192e-13 3.83653245040640e-13 -100
"chr7" 148935001 148936000 "*" 2.84630707891864e-09 5.16130702871299e-09
56.5217391304348
"chr7" 148936001 148937000 "*" 0 0 -100
"chr7" 149100001 149101000 "*" 7.52445745058061e-05 7.19216306068777e-05 -56.25
"chr7" 149158001 149159000 "*" 0 0 -80.4347826086957
"chr7" 149166001 149167000 "*" 2.1094237467878e-15 8.47434540879246e-15 -100
"chr7" 149322001 149323000 "*" 0 0 -100
"chr7" 149388001 149389000 "*" 0 0 57.3770491803279
"chr7" 149425001 149426000 "*" 1.42505651723468e-09 2.70336426982488e-09
-54.5454545454545
"chr7" 149426001 149427000 "*" 0 0 57.1428571428571
"chr7" 149431001 149432000 "*" 0 0 50.6025077348966
"chr7" 149433001 149434000 "*" 3.6700841921089e-08 5.65530226072258e-08 100
"chr7" 149466001 149467000 "*" 7.79358799718466e-11 1.7626865650437e-10
-53.4883720930233
"chr7" 149471001 149472000 "*" 1.20091159239166e-07 1.72351431381562e-07

70.6959706959707
"chr7" 149472001 149473000 "*" 9.28146448586631e-14 3.0676096022063e-13
-52.1739130434783
"chr7" 149476001 149477000 "*" 1.84705462036305e-08 2.97954860987945e-08
-64.1025641025641
"chr7" 149495001 149496000 "*" 1.07580611086178e-13 3.52629222803534e-13
94.1176470588235
"chr7" 149520001 149521000 "*" 1.47348799828251e-12 4.20352064420197e-12
67.1232876712329
"chr7" 149550001 149551000 "*" 3.99680288865056e-15 1.5566998858822e-14 100
"chr7" 149561001 149562000 "*" 1.99840144432528e-12 5.59476580335607e-12 100
"chr7" 149571001 149572000 "*" 2.72664113509791e-11 6.58035103459143e-11
53.742802303263
"chr7" 149634001 149635000 "*" 4.10782519111308e-15 1.59859775077462e-14
-54.1666666666667
"chr7" 149642001 149643000 "*" 2.05509798067993e-09 3.81389861881893e-09
55.7692307692308
"chr7" 149644001 149645000 "*" 2.75891949397256e-06 3.28830469657595e-06
-59.4594594594595
"chr7" 149720001 149721000 "*" 5.18803490645592e-06 5.93671675289902e-06 75
"chr7" 149735001 149736000 "*" 2.88779000712225e-11 6.92935809466068e-11 100
"chr7" 149744001 149745000 "*" 0 0 58.1648151069248
"chr7" 149977001 149978000 "*" 1.96509475358653e-13 6.23868565133654e-13 100
"chr7" 150439001 150440000 "*" 2.70548178482599e-06 3.22867336080627e-06
-66.6666666666667
"chr7" 150549001 150550000 "*" 2.68951676152263e-07 3.68696493018621e-07
55.5555555555556
"chr7" 150559001 150560000 "*" 0 0 95.4545454545455
"chr7" 150593001 150594000 "*" 7.93809462606987e-14 2.64679416473686e-13 100
"chr7" 150599001 150600000 "*" 0 0 100
"chr7" 150612001 150613000 "*" 1.12634901405784e-10 2.49563754426169e-10 100
"chr7" 150659001 150660000 "*" 3.95353005888666e-09 7.03928529283733e-09 100
"chr7" 150671001 150672000 "*" 0 0 92.1652058703628
"chr7" 150676001 150677000 "*" 0 0 82.7338129496403
"chr7" 150688001 150689000 "*" 1.62177271612052e-10 3.5126448030081e-10
72.4137931034483
"chr7" 150718001 150719000 "*" 3.62601393355533e-10 7.47397778855089e-10 66.875
"chr7" 150744001 150745000 "*" 0 0 100
"chr7" 150748001 150749000 "*" 0 0 52.8256353756018
"chr7" 150863001 150864000 "*" 1.3172503088299e-09 2.50792361237215e-09 -75
"chr7" 150864001 150865000 "*" 0 0 65.3731726948248
"chr7" 150866001 150867000 "*" 2.22044604925031e-16 9.81641919380259e-16
52.6315789473684
"chr7" 150898001 150899000 "*" 2.84573700715107e-07 3.8881484784083e-07 75
"chr7" 150939001 150940000 "*" 4.11539404343575e-06 4.77326970666537e-06
56.5217391304348
"chr7" 150984001 150985000 "*" 3.00861638513616e-06 3.5651261369125e-06
56.4102564102564
"chr7" 151001001 151002000 "*" 0 0 78.4313725490196
"chr7" 151078001 151079000 "*" 0 0 56.3928579908351
"chr7" 151127001 151128000 "*" 6.85192762995834e-05 6.59156283435548e-05
-50.9090909090909

"chr7" 151130001 151131000 "*" 0 0 -60.2941176470588
"chr7" 151148001 151149000 "*" 1.07792778236693e-05 1.17513174008031e-05
58.6206896551724
"chr7" 151282001 151283000 "*" 0 0 -72.0749043193002
"chr7" 151376001 151377000 "*" 6.52733422867868e-12 1.71146951718423e-11 100
"chr7" 151400001 151401000 "*" 1.1988837312904e-06 1.5057940841428e-06
-58.3333333333333
"chr7" 151428001 151429000 "*" 2.68453836005378e-06 3.20505570814476e-06
69.9029126213592
"chr7" 151442001 151443000 "*" 7.99844594256971e-06 8.89173797399106e-06
54.5454545454545
"chr7" 151494001 151495000 "*" 5.97385209077927e-08 8.97069497046889e-08
52.755905511811
"chr7" 151516001 151517000 "*" 4.08537648155516e-11 9.58460845439556e-11
93.3333333333333
"chr7" 151518001 151519000 "*" 9.53836173378519e-08 1.38751147105513e-07 72
"chr7" 151548001 151549000 "*" 1.49383446723661e-08 2.444556877867e-08
-69.6969696969697
"chr7" 151557001 151558000 "*" 0 0 -100
"chr7" 151569001 151570000 "*" 5.12854203549296e-11 1.18835323590281e-10
69.2307692307692
"chr7" 151583001 151584000 "*" 6.51910969651226e-10 1.29430402625998e-09
-66.6666666666667
"chr7" 151584001 151585000 "*" 2.22044604925031e-16 9.81641919380259e-16 -100
"chr7" 151593001 151594000 "*" 1.81518016173499e-08 2.93136260967905e-08
75.4385964912281
"chr7" 151723001 151724000 "*" 3.88022947106492e-13 1.1867462770515e-12 100
"chr7" 151964001 151965000 "*" 9.65729496371637e-10 1.87255912493232e-09 100
"chr7" 152098001 152099000 "*" 0 0 100
"chr7" 152105001 152106000 "*" 8.64488327589896e-08 1.26480485233459e-07
58.8235294117647
"chr7" 152154001 152155000 "*" 1.98729921407903e-12 5.56816970844325e-12
66.6666666666667
"chr7" 152294001 152295000 "*" 6.16805551856281e-07 8.06313781087357e-07
73.1707317073171
"chr7" 152322001 152323000 "*" 3.19744231092045e-14 1.1176868990786e-13
76.6666666666667
"chr7" 152380001 152381000 "*" 5.01025332333427e-10 1.01358031135889e-09 -100
"chr7" 152435001 152436000 "*" 1.67299207820548e-08 2.71377429441055e-08 100
"chr7" 152436001 152437000 "*" 0 0 100
"chr7" 152596001 152597000 "*" 2.88779000712225e-11 6.92935809466068e-11 100
"chr7" 152605001 152606000 "*" 6.44564592999775e-06 7.26663863268796e-06
-66.6666666666667
"chr7" 152616001 152617000 "*" 1.36604061395929e-11 3.42333952202875e-11 -100
"chr7" 152619001 152620000 "*" 0 0 -59.1823899371069
"chr7" 152631001 152632000 "*" 0 0 70.5882352941177
"chr7" 152654001 152655000 "*" 1.27542421068938e-12 3.66506798413395e-12
-60.2409638554217
"chr7" 152704001 152705000 "*" 1.75928160928152e-11 4.35257527084094e-11
-56.5217391304348
"chr7" 152707001 152708000 "*" 8.44866073321793e-05 8.01219179790309e-05
62.962962962963

"chr7" 152716001 152717000 "*" 1.16018306073329e-13 3.78664250293869e-13
-50.3228523301516
"chr7" 152720001 152721000 "*" 4.71174774152594e-06 5.42137638083795e-06
-53.5714285714286
"chr7" 152724001 152725000 "*" 5.10702591327572e-15 1.96271840288234e-14
-66.6666666666667
"chr7" 152780001 152781000 "*" 0 0 62.614412136536
"chr7" 152868001 152869000 "*" 3.7274627828765e-12 1.00985250136599e-11 -100
"chr7" 152982001 152983000 "*" 9.80763359414993e-10 1.8980032424715e-09 100
"chr7" 153057001 153058000 "*" 5.27631494051661e-10 1.06513151973685e-09
-66.6666666666667
"chr7" 153098001 153099000 "*" 0 0 -100
"chr7" 153108001 153109000 "*" 5.03264097062583e-13 1.51942428521737e-12 -100
"chr7" 153150001 153151000 "*" 7.34908023325431e-09 1.25734591572739e-08 95
"chr7" 153168001 153169000 "*" 4.39648317751562e-14 1.5131009830411e-13
-85.1351351351351
"chr7" 153172001 153173000 "*" 4.32209823486573e-12 1.15935290479641e-11 -100
"chr7" 153228001 153229000 "*" 0 0 -73.0769230769231
"chr7" 153254001 153255000 "*" 1.25284005392245e-10 2.75649590272477e-10 -100
"chr7" 153540001 153541000 "*" 0 0 54.7368421052632
"chr7" 153632001 153633000 "*" 0 0 55.9405940594059
"chr7" 153647001 153648000 "*" 0 0 -100
"chr7" 153848001 153849000 "*" 1.52794177310511e-09 2.87642564232045e-09 100
"chr7" 153896001 153897000 "*" 2.70339306496226e-13 8.42355472617862e-13 100
"chr7" 154028001 154029000 "*" 1.20591092667155e-10 2.66075324129686e-10 100
"chr7" 154072001 154073000 "*" 1.22862224838372e-07 1.76152573195251e-07
-54.1666666666667
"chr7" 154100001 154101000 "*" 2.55351295663786e-15 1.01678090381833e-14
86.1111111111111
"chr7" 154372001 154373000 "*" 1.11022302462516e-16 5.03662826618488e-16
-82.2916666666667
"chr7" 154498001 154499000 "*" 7.03992419914812e-12 1.83313429961219e-11 100
"chr7" 154541001 154542000 "*" 1.11022302462516e-16 5.03662826618488e-16 -93.75
"chr7" 154543001 154544000 "*" 1.33434374660624e-11 3.35156000674969e-11 71.875
"chr7" 154551001 154552000 "*" 9.62896429257398e-13 2.80291205576417e-12 100
"chr7" 154576001 154577000 "*" 9.08108033215171e-11 2.03407753381119e-10 -100
"chr7" 154587001 154588000 "*" 2.67085242811049e-11 6.45561379532306e-11
63.8297872340426
"chr7" 154589001 154590000 "*" 2.74687161905263e-10 5.76953704674915e-10
66.6666666666667
"chr7" 154673001 154674000 "*" 1.49106017250933e-05 1.59124089221917e-05
-56.6666666666667
"chr7" 154791001 154792000 "*" 0 0 100
"chr7" 154799001 154800000 "*" 1.83941750719896e-12 5.17499701329218e-12
-62.2222222222222
"chr7" 154803001 154804000 "*" 1.98365768255826e-11 4.85612402473442e-11 100
"chr7" 154913001 154914000 "*" 3.52704532247117e-11 8.35581292290093e-11 100
"chr7" 154916001 154917000 "*" 5.55757757350062e-05 5.42480095378277e-05
-58.252427184466
"chr7" 154946001 154947000 "*" 6.15840711759574e-13 1.83676803572802e-12 100
"chr7" 154955001 154956000 "*" 4.15429479705409e-08 6.36532640392233e-08
-83.3333333333333

"chr7" 154963001 154964000 "*" 2.15125472990962e-10 4.58126659943874e-10 100
"chr7" 154997001 154998000 "*" 0 0 -68.9569536423841
"chr7" 155005001 155006000 "*" 1.13686816183289e-07 1.63723575527561e-07
-51.8518518518519
"chr7" 155054001 155055000 "*" 1.71196390397199e-13 5.4828532997284e-13
-97.2222222222222
"chr7" 155058001 155059000 "*" 1.25243371229544e-10 2.75649590272477e-10
-65.625
"chr7" 155067001 155068000 "*" 5.01337082958742e-10 1.0141620111296e-09
-66.6666666666667
"chr7" 155109001 155110000 "*" 3.02357555993504e-05 3.07815127852254e-05
-72.2222222222222
"chr7" 155135001 155136000 "*" 1.39779633423487e-07 1.98463037958589e-07 100
"chr7" 155152001 155153000 "*" 2.02060590481778e-13 6.40127405465654e-13
66.6666666666667
"chr7" 155153001 155154000 "*" 1.16527842930481e-09 2.2312662587881e-09
-57.7922077922078
"chr7" 155155001 155156000 "*" 5.794809077031e-12 1.52911192757929e-11 100
"chr7" 155156001 155157000 "*" 0 0 -69.2307692307692
"chr7" 155168001 155169000 "*" 7.82695993573057e-08 1.15142528036045e-07
64.7058823529412
"chr7" 155177001 155178000 "*" 4.44089209850063e-15 1.71914916534614e-14
-51.8812230152436
"chr7" 155182001 155183000 "*" 2.66279220895171e-11 6.43687794881457e-11
63.4920634920635
"chr7" 155205001 155206000 "*" 0 0 53.4466964387954
"chr7" 155214001 155215000 "*" 4.02167188440217e-12 1.08266700497926e-11 -100
"chr7" 155229001 155230000 "*" 3.12164139724125e-05 3.17090411597004e-05
54.1666666666667
"chr7" 155265001 155266000 "*" 3.84137166520304e-14 1.32929369817675e-13 100
"chr7" 155267001 155268000 "*" 0 0 100
"chr7" 155337001 155338000 "*" 9.65729496371637e-10 1.87255912493232e-09 100
"chr7" 155340001 155341000 "*" 1.20105703160789e-10 2.6536238618325e-10
64.7058823529412
"chr7" 155349001 155350000 "*" 0.000216683727490286 0.000192426836197816 70
"chr7" 155361001 155362000 "*" 0.000496254900866688 0.000414667937798419
54.1666666666667
"chr7" 155364001 155365000 "*" 1.41109876605761e-06 1.75583535616452e-06 90
"chr7" 155371001 155372000 "*" 3.0477846868493e-07 4.14668048576323e-07
56.6666666666667
"chr7" 155378001 155379000 "*" 3.34128893442198e-08 5.19772864618523e-08 -100
"chr7" 155386001 155387000 "*" 1.10692880417318e-08 1.84563442150892e-08
52.6315789473684
"chr7" 155393001 155394000 "*" 9.0072393987839e-13 2.63157725646847e-12 100
"chr7" 155602001 155603000 "*" 0 0 68.1776699230498
"chr7" 155670001 155671000 "*" 3.6700841921089e-08 5.65530226072258e-08 100
"chr7" 155704001 155705000 "*" 8.69750937937397e-11 1.95588304295377e-10
-73.3333333333333
"chr7" 155719001 155720000 "*" 0 0 100
"chr7" 155721001 155722000 "*" 1.56874513379535e-13 5.03965323085983e-13
-51.8796992481203
"chr7" 155749001 155750000 "*" 5.39431832535797e-11 1.24737268974759e-10

-82.3443223443223
"chr7" 155753001 155754000 "*" 4.16020331528433e-06 4.82249302255461e-06
-68.8888888888889
"chr7" 155754001 155755000 "*" 2.23154827949656e-14 7.95931582053377e-14
-80.2816901408451
"chr7" 155756001 155757000 "*" 0 0 53.4405634339012
"chr7" 155770001 155771000 "*" 3.04324343503026e-11 7.26599935673121e-11 -100
"chr7" 155776001 155777000 "*" 2.4535928844216e-14 8.69144396197119e-14 -100
"chr7" 155799001 155800000 "*" 1.63270530428861e-09 3.06548312300294e-09
88.0434782608696
"chr7" 155823001 155824000 "*" 0 0 -100
"chr7" 155884001 155885000 "*" 1.59172675040509e-11 3.95858417680319e-11 84.375
"chr7" 155891001 155892000 "*" 2.15125472990962e-10 4.58126659943874e-10 -100
"chr7" 155893001 155894000 "*" 5.51780843238703e-14 1.87243792572872e-13 100
"chr7" 155937001 155938000 "*" 3.54448328678814e-05 3.56662254642776e-05 -60
"chr7" 155940001 155941000 "*" 1.20472640086078e-07 1.72881072111193e-07
-50.9433962264151
"chr7" 155951001 155952000 "*" 0 0 100
"chr7" 155955001 155956000 "*" 3.65263375101677e-14 1.26882502648938e-13
69.2307692307692
"chr7" 155973001 155974000 "*" 4.34121823711386e-07 5.78408567117475e-07
71.4285714285714
"chr7" 155997001 155998000 "*" 3.6700841921089e-08 5.65530226072258e-08 100
"chr7" 156042001 156043000 "*" 4.73234496034536e-09 8.30627300264826e-09 -100
"chr7" 156057001 156058000 "*" 2.81173456739836e-08 4.42070321258553e-08
-65.7142857142857
"chr7" 156137001 156138000 "*" 0 0 -100
"chr7" 156173001 156174000 "*" 1.93720595120794e-11 4.76309924836636e-11 100
"chr7" 156207001 156208000 "*" 7.04457048250617e-10 1.39315702524552e-09
-67.5675675675676
"chr7" 156236001 156237000 "*" 9.61897228535236e-13 2.801372325071e-12 -100
"chr7" 156251001 156252000 "*" 1.12798481666232e-08 1.87801380787728e-08
52.1739130434783
"chr7" 156342001 156343000 "*" 5.45713698452133e-07 7.18197642404551e-07
54.8387096774194
"chr7" 156410001 156411000 "*" 0 0 100
"chr7" 156758001 156759000 "*" 4.44089209850063e-16 1.91071758245033e-15 100
"chr7" 156809001 156810000 "*" 6.30103080823119e-10 1.25663062491693e-09
54.9462365591398
"chr7" 156814001 156815000 "*" 0 0 61.8077924154634
"chr7" 156836001 156837000 "*" 0 0 -100
"chr7" 156839001 156840000 "*" 2.68141064907468e-12 7.40011637970864e-12
67.2268907563025
"chr7" 156843001 156844000 "*" 0 0 100
"chr7" 156857001 156858000 "*" 2.22044604925031e-16 9.81641919380259e-16
-69.7674418604651
"chr7" 156868001 156869000 "*" 6.59550958292954e-09 1.13456194382773e-08 100
"chr7" 156879001 156880000 "*" 8.04911692853238e-14 2.68027613922457e-13
-82.8125
"chr7" 156905001 156906000 "*" 2.34402411924961e-08 3.72020866732378e-08
-66.6666666666667
"chr7" 156911001 156912000 "*" 7.0006535457523e-08 1.03695832512054e-07 -100

"chr7" 156976001 156977000 "*" 6.59550958292954e-09 1.13456194382773e-08 -100
"chr7" 157101001 157102000 "*" 9.35229671483739e-12 2.40247663489449e-11 -80
"chr7" 157179001 157180000 "*" 0 0 -100
"chr7" 157231001 157232000 "*" 3.65130148338721e-12 9.90602415539447e-12 100
"chr7" 157232001 157233000 "*" 3.70415934591861e-07 4.97992215904616e-07 60
"chr7" 157238001 157239000 "*" 2.22044604925031e-16 9.81641919380259e-16 100
"chr7" 157251001 157252000 "*" 1.09008917226205e-07 1.57413554003421e-07
52.1739130434783
"chr7" 157268001 157269000 "*" 0 0 100
"chr7" 157365001 157366000 "*" 0 0 53.7313432835821
"chr7" 157366001 157367000 "*" 1.06576415803694e-07 1.5411973295234e-07
59.0909090909091
"chr7" 157368001 157369000 "*" 0 0 100
"chr7" 157372001 157373000 "*" 0 0 94.1176470588235
"chr7" 157390001 157391000 "*" 8.25996878681678e-05 7.84689966164452e-05 55
"chr7" 157401001 157402000 "*" 0 0 93.1972789115646
"chr7" 157421001 157422000 "*" 0 0 52.6315789473684
"chr7" 157422001 157423000 "*" 3.63445940010365e-11 8.58565209111204e-11 100
"chr7" 157423001 157424000 "*" 0 0 -52.5552486187845
"chr7" 157497001 157498000 "*" 1.11022302462516e-16 5.03662826618488e-16 -100
"chr7" 157499001 157500000 "*" 2.32558734318822e-05 2.40932654202135e-05 80
"chr7" 157547001 157548000 "*" 3.33066907387547e-16 1.4495649018245e-15 100
"chr7" 157556001 157557000 "*" 3.26405569239796e-14 1.13973003849234e-13 100
"chr7" 157560001 157561000 "*" 4.96120877713224e-08 7.52826079518588e-08
79.1666666666667
"chr7" 157564001 157565000 "*" 3.77475828372553e-15 1.47379557022071e-14 -100
"chr7" 157572001 157573000 "*" 2.56593635228342e-11 6.2140914153063e-11 -76
"chr7" 157639001 157640000 "*" 3.47522011168167e-12 9.45013953170389e-12 -84
"chr7" 157640001 157641000 "*" 0 0 100
"chr7" 157668001 157669000 "*" 1.11022302462516e-16 5.03662826618488e-16
60.3729603729604
"chr7" 157681001 157682000 "*" 1.48054134507802e-07 2.09572769517404e-07
-50.381679389313
"chr7" 157692001 157693000 "*" 1.79990597537483e-07 2.52257533148791e-07
62.2754491017964
"chr7" 157717001 157718000 "*" 1.33235292798517e-08 2.1936464870907e-08
90.9090909090909
"chr7" 157746001 157747000 "*" 3.79779790455892e-05 3.80316501795406e-05
-59.5833333333333
"chr7" 157755001 157756000 "*" 3.06878988887149e-09 5.54641273345846e-09
58.6206896551724
"chr7" 157780001 157781000 "*" 1.90958360235527e-13 6.07725724484217e-13
58.3391853932584
"chr7" 157801001 157802000 "*" 1.34336985979644e-14 4.91028542731765e-14
-70.5882352941177
"chr7" 157822001 157823000 "*" 1.40068128089421e-05 1.50039561086948e-05
54.1666666666667
"chr7" 157839001 157840000 "*" 8.77304878819096e-05 8.29840275600617e-05
57.5757575757576
"chr7" 157841001 157842000 "*" 1.31883393095222e-12 3.78420384740592e-12 -75
"chr7" 157845001 157846000 "*" 0 0 -100
"chr7" 157852001 157853000 "*" 4.32209823486573e-12 1.15935290479641e-11 100

"chr7" 157869001 157870000 "*" 1.11022302462516e-16 5.03662826618488e-16 -100
"chr7" 157902001 157903000 "*" 3.95752999871046e-06 4.60432112953806e-06
61.6666666666667
"chr7" 157927001 157928000 "*" 1.38590361409285e-10 3.02706178892116e-10 100
"chr7" 157930001 157931000 "*" 3.96248652272391e-05 3.95701587394277e-05
51.1111111111111
"chr7" 157979001 157980000 "*" 0 0 -75
"chr7" 157989001 157990000 "*" 1.32764432780874e-09 2.5273090081967e-09
-51.5151515151515
"chr7" 158043001 158044000 "*" 4.91828799908944e-14 1.68192819018269e-13 -100
"chr7" 158048001 158049000 "*" 3.33066907387547e-16 1.4495649018245e-15 -100
"chr7" 158056001 158057000 "*" 4.62915594745539e-10 9.4228944742555e-10
-52.5737131434283
"chr7" 158074001 158075000 "*" 6.4152538836737e-10 1.27477697728417e-09 100
"chr7" 158077001 158078000 "*" 1.26518795440234e-11 3.19102298953095e-11
87.1794871794872
"chr7" 158078001 158079000 "*" 0 0 75
"chr7" 158106001 158107000 "*" 1.21922773882455e-05 1.31811221609643e-05
-58.8235294117647
"chr7" 158126001 158127000 "*" 9.23483511883205e-13 2.69542831003999e-12
-86.8852459016394
"chr7" 158135001 158136000 "*" 9.09525621484164e-09 1.53277270380992e-08
-75.609756097561
"chr7" 158143001 158144000 "*" 9.20005425175674e-08 1.34097229374078e-07
54.5454545454545
"chr7" 158148001 158149000 "*" 0 0 -100
"chr7" 158206001 158207000 "*" 9.46317535444052e-09 1.59107213149503e-08
60.2307225258045
"chr7" 158232001 158233000 "*" 1.98931358053045e-08 3.19617290079798e-08 87.5
"chr7" 158309001 158310000 "*" 7.00130639019747e-08 1.03702852845168e-07
-72.7272727272727
"chr7" 158323001 158324000 "*" 1.50990331349021e-14 5.48844964705101e-14
63.1578947368421
"chr7" 158338001 158339000 "*" 0 0 62.6373626373626
"chr7" 158354001 158355000 "*" 7.21644966006352e-15 2.72345776413425e-14
87.012987012987
"chr7" 158360001 158361000 "*" 1.65900401183361e-06 2.04236557510993e-06
-66.6666666666667
"chr7" 158380001 158381000 "*" 0 0 80.3061224489796
"chr7" 158576001 158577000 "*" 0 0 100
"chr7" 158617001 158618000 "*" 3.34128893442198e-08 5.19772864618523e-08 100
"chr7" 158629001 158630000 "*" 7.15982828580763e-13 2.11757391829393e-12
-66.6666666666667
"chr7" 158647001 158648000 "*" 3.90465437760668e-13 1.19307893943277e-12 100
"chr7" 158806001 158807000 "*" 1.11022302462516e-16 5.03662826618488e-16 68.75
"chr7" 158909001 158910000 "*" 4.91828799908944e-14 1.68192819018269e-13 -100
"chr7" 158911001 158912000 "*" 0 0 85.9872611464968
"chr7" 158921001 158922000 "*" 9.08108033215171e-11 2.03407753381119e-10 100
"chr7" 158941001 158942000 "*" 9.26392694777789e-08 1.34981038124536e-07
81.1594202898551
"chr7" 158943001 158944000 "*" 1.95399252334028e-14 7.01253661173697e-14 100
"chr7" 158950001 158951000 "*" 2.74580358450294e-12 7.54984612585509e-12

52.9166666666667
"chr7" 158958001 158959000 "*" 1.16059570322014e-05 1.25913170315835e-05
-60.7142857142857
"chr7" 158997001 158998000 "*" 0 0 100
"chr7" 159019001 159020000 "*" 0 0 100
"chr7_gl000195_random" 22001 23000 "*" 4.32209823486573e-12
1.15935290479641e-11 100
"chr7_gl000195_random" 24001 25000 "*" 2.81154646697424e-06
3.34643884318492e-06 55
"chr7_gl000195_random" 33001 34000 "*" 0 0 69.9000114929319
"chr7_gl000195_random" 86001 87000 "*" 2.28705943072782e-14
8.14964112513644e-14 86.9565217391304
"chr8" 224001 225000 "*" 1.11022302462516e-16 5.03662826618488e-16 100
"chr8" 236001 237000 "*" 1.39779633423487e-07 1.98463037958589e-07 -100
"chr8" 285001 286000 "*" 1.03834827450688e-05 1.13502457227881e-05
-57.1428571428571
"chr8" 294001 295000 "*" 1.34097659421073e-07 1.9132716764845e-07
-65.5172413793103
"chr8" 299001 300000 "*" 0 0 -69.1729323308271
"chr8" 331001 332000 "*" 0 0 88.8888888888889
"chr8" 350001 351000 "*" 1.5277158427196e-09 2.87642564232045e-09 -100
"chr8" 357001 358000 "*" 0 0 -80
"chr8" 439001 440000 "*" 0 0 100
"chr8" 451001 452000 "*" 4.0705084769499e-07 5.44261714716217e-07
-68.5714285714286
"chr8" 512001 513000 "*" 4.44089209850063e-16 1.91071758245033e-15 100
"chr8" 522001 523000 "*" 7.65054686269195e-13 2.25814319276556e-12 90
"chr8" 579001 580000 "*" 8.37752089921651e-11 1.88922989503423e-10
-50.8771929824561
"chr8" 622001 623000 "*" 1.36893940627658e-10 3.00038107307763e-10
58.3333333333333
"chr8" 690001 691000 "*" 2.23097096352376e-11 5.43476262638853e-11
95.6521739130435
"chr8" 696001 697000 "*" 0 0 71.57874617737
"chr8" 709001 710000 "*" 1.5413226250871e-12 4.37876421934895e-12
84.8837209302326
"chr8" 751001 752000 "*" 2.99693603267315e-12 8.20249914342802e-12 80
"chr8" 785001 786000 "*" 1.35036426485158e-12 3.86629547363774e-12 100
"chr8" 810001 811000 "*" 2.16382467499443e-13 6.82775854166559e-13 -100
"chr8" 820001 821000 "*" 9.62430062312336e-08 1.39943403562665e-07
-61.9047619047619
"chr8" 840001 841000 "*" 0 0 100
"chr8" 844001 845000 "*" 1.06807895861039e-11 2.72378086982553e-11
56.3636363636364
"chr8" 848001 849000 "*" 0 0 -89.0243902439024
"chr8" 849001 850000 "*" 2.00994776378138e-12 5.62539160280717e-12
85.2941176470588
"chr8" 860001 861000 "*" 0 0 100
"chr8" 862001 863000 "*" 0 0 53.2994923857868
"chr8" 874001 875000 "*" 2.04550820726013e-11 5.00166559583469e-11
-52.3888520238885
"chr8" 916001 917000 "*" 9.82476163424728e-07 1.24808378702455e-06

-55.5555555555556
"chr8" 928001 929000 "*" 0 0 -62.042482727117
"chr8" 939001 940000 "*" 0 0 83.3333333333333
"chr8" 978001 979000 "*" 8.74150577973021e-05 8.27062898721633e-05 -53.125
"chr8" 995001 996000 "*" 5.794809077031e-12 1.52911192757929e-11 100
"chr8" 1003001 1004000 "*" 1.79523063081888e-13 5.7367518231213e-13 70
"chr8" 1010001 1011000 "*" 0 0 -95.5555555555556
"chr8" 1011001 1012000 "*" 0 0 100
"chr8" 1027001 1028000 "*" 4.85167461761193e-14 1.6617373494005e-13 80
"chr8" 1038001 1039000 "*" 1.17810923272454e-06 1.48139691706456e-06
-57.4074074074074
"chr8" 1052001 1053000 "*" 1.13055163786235e-07 1.62858910042063e-07
-71.830985915493
"chr8" 1060001 1061000 "*" 4.43011600304022e-05 4.39265220551362e-05
63.8888888888889
"chr8" 1067001 1068000 "*" 0 0 -97.2222222222222
"chr8" 1110001 1111000 "*" 2.79868308528108e-07 3.82788144935124e-07
75.609756097561
"chr8" 1111001 1112000 "*" 5.44009282066327e-15 2.08486137681812e-14 -100
"chr8" 1133001 1134000 "*" 1.13242748511766e-14 4.1830649980713e-14
64.1666666666667
"chr8" 1135001 1136000 "*" 0 0 -60.6382978723404
"chr8" 1155001 1156000 "*" 0.000195183235292329 0.000174565611738003
51.937984496124
"chr8" 1156001 1157000 "*" 0 0 -62.0481927710843
"chr8" 1183001 1184000 "*" 0 0 -100
"chr8" 1190001 1191000 "*" 0.00011461664363277 0.000106520035249614
55.6962025316456
"chr8" 1197001 1198000 "*" 0 0 -52.7904695526472
"chr8" 1204001 1205000 "*" 2.44249065417534e-15 9.75180348030994e-15
-62.2950819672131
"chr8" 1250001 1251000 "*" 0 0 100
"chr8" 1252001 1253000 "*" 0 0 -100
"chr8" 1267001 1268000 "*" 0 0 66.6666666666667
"chr8" 1313001 1314000 "*" 8.61712767807887e-09 1.46086889771042e-08 76
"chr8" 1333001 1334000 "*" 0 0 -88.7840670859539
"chr8" 1369001 1370000 "*" 6.4152538836737e-10 1.27477697728417e-09 100
"chr8" 1379001 1380000 "*" 0 0 68.5714285714286
"chr8" 1408001 1409000 "*" 0 0 100
"chr8" 1414001 1415000 "*" 0 0 100
"chr8" 1416001 1417000 "*" 0 0 84.375
"chr8" 1494001 1495000 "*" 3.64797081431334e-12 9.90149732934698e-12
-55.9473684210526
"chr8" 1543001 1544000 "*" 9.56709081567642e-06 1.05191958287164e-05
54.5454545454545
"chr8" 1604001 1605000 "*" 1.55431223447522e-15 6.33705141101682e-15 -100
"chr8" 1614001 1615000 "*" 0 0 100
"chr8" 1615001 1616000 "*" 0 0 71.4285714285714
"chr8" 1642001 1643000 "*" 0 0 98.0861244019139
"chr8" 1678001 1679000 "*" 0 0 100
"chr8" 1690001 1691000 "*" 5.93492033296172e-09 1.03010653728046e-08
58.8235294117647

"chr8" 1697001 1698000 "*" 3.30335758746969e-12 9.00334395866367e-12
58.0542264752791
"chr8" 1702001 1703000 "*" 1.55196222539900e-09 2.91960235645947e-09
61.2903225806452
"chr8" 1749001 1750000 "*" 0 0 -100
"chr8" 1754001 1755000 "*" 2.55351295663786e-15 1.01678090381833e-14 100
"chr8" 1761001 1762000 "*" 4.19435610332641e-06 4.85857056843185e-06
-61.5384615384615
"chr8" 1771001 1772000 "*" 0 0 95.3789279112754
"chr8" 1776001 1777000 "*" 0 0 -100
"chr8" 1784001 1785000 "*" 0 0 -100
"chr8" 1849001 1850000 "*" 0 0 75
"chr8" 1855001 1856000 "*" 1.12843068222901e-12 3.259512533505e-12 -100
"chr8" 1922001 1923000 "*" 0 0 57.7531391072175
"chr8" 1927001 1928000 "*" 2.24442686658222e-12 6.2478893514843e-12
-55.7377049180328
"chr8" 1969001 1970000 "*" 4.08373335147871e-12 1.09877151902352e-11
81.8181818181818
"chr8" 1995001 1996000 "*" 2.67283972732457e-11 6.45686178157698e-11 -100
"chr8" 1999001 2000000 "*" 2.48931644186712e-08 3.93716649086578e-08
-66.6666666666667
"chr8" 2027001 2028000 "*" 2.00227675550835e-08 3.20459176617961e-08 100
"chr8" 2044001 2045000 "*" 0 0 52.7777777777778
"chr8" 2061001 2062000 "*" 1.35003119794419e-13 4.3679847476192e-13 100
"chr8" 2083001 2084000 "*" 3.63445940010365e-11 8.58565209111204e-11 100
"chr8" 2085001 2086000 "*" 0 0 -80.4347826086957
"chr8" 2158001 2159000 "*" 9.07951158701792e-09 1.53048405218937e-08
53.2608695652174
"chr8" 2161001 2162000 "*" 0 0 100
"chr8" 2174001 2175000 "*" 2.8689339294119e-05 2.93084297927469e-05
62.8571428571429
"chr8" 2183001 2184000 "*" 0 0 100
"chr8" 2185001 2186000 "*" 2.4535928844216e-14 8.69144396197119e-14 100
"chr8" 2196001 2197000 "*" 0 0 56.103552532124
"chr8" 2201001 2202000 "*" 0 0 85.593220338983
"chr8" 2206001 2207000 "*" 2.71318634226247e-09 4.93192458650319e-09
57.089552238806
"chr8" 2216001 2217000 "*" 1.38590361409285e-10 3.02706178892116e-10 100
"chr8" 2253001 2254000 "*" 2.64951482975562e-09 4.82186011375752e-09 100
"chr8" 2271001 2272000 "*" 0 0 100
"chr8" 2364001 2365000 "*" 0 0 -100
"chr8" 2480001 2481000 "*" 0 0 68.4953610326745
"chr8" 2481001 2482000 "*" 4.57376300611045e-07 6.07664036836632e-07
51.9230769230769
"chr8" 2505001 2506000 "*" 2.00227675550835e-08 3.20459176617961e-08 100
"chr8" 2668001 2669000 "*" 8.3882012447134e-11 1.88922989503423e-10 -100
"chr8" 2996001 2997000 "*" 2.22044604925031e-16 9.81641919380259e-16 100
"chr8" 3231001 3232000 "*" 0 0 -100
"chr8" 3279001 3280000 "*" 1.92946192356658e-09 3.59193011611483e-09 100
"chr8" 3316001 3317000 "*" 5.53435075545394e-12 1.46506494296025e-11
-59.4134897360704
"chr8" 3325001 3326000 "*" 5.48638023900594e-10 1.10374357336421e-09 100

"chr8" 3342001 3343000 "*" 2.4535928844216e-14 8.69144396197119e-14 -100
"chr8" 3452001 3453000 "*" 6.30384633382164e-13 1.87665599796749e-12 100
"chr8" 3545001 3546000 "*" 7.0006535457523e-08 1.03695832512054e-07 -100
"chr8" 3569001 3570000 "*" 0 0 -100
"chr8" 3720001 3721000 "*" 0 0 75
"chr8" 3732001 3733000 "*" 6.81146250514075e-11 1.55544800479628e-10
-72.1311475409836
"chr8" 4044001 4045000 "*" 1.32736761893071e-06 1.65728591505476e-06
51.6129032258064
"chr8" 4045001 4046000 "*" 3.00652418849801e-07 4.0944173474794e-07 75
"chr8" 4077001 4078000 "*" 4.40758540776187e-14 1.51685935033756e-13 -75
"chr8" 4411001 4412000 "*" 0 0 -71.7647058823529
"chr8" 4656001 4657000 "*" 5.40826383677029e-10 1.09031635616681e-09 75
"chr8" 4848001 4849000 "*" 1.13131726209303e-13 3.69879839085845e-13
69.3009118541033
"chr8" 5127001 5128000 "*" 2.00227675550835e-08 3.20459176617961e-08 -100
"chr8" 5426001 5427000 "*" 9.63829016598083e-12 2.46993429847588e-11 100
"chr8" 5830001 5831000 "*" 1.98638744342006e-08 3.19202499485717e-08
77.2727272727273
"chr8" 5925001 5926000 "*" 2.00227675550835e-08 3.20459176617961e-08 -100
"chr8" 5933001 5934000 "*" 1.41778362938716e-07 2.01170748993017e-07
66.6666666666667
"chr8" 5990001 5991000 "*" 0 0 75
"chr8" 6033001 6034000 "*" 0 0 100
"chr8" 6167001 6168000 "*" 1.13140401492018e-08 1.87801380787728e-08 100
"chr8" 6227001 6228000 "*" 3.3004321497998e-09 5.94497676880652e-09
-84.8101265822785
"chr8" 6425001 6426000 "*" 4.44089209850063e-15 1.71914916534614e-14 -100
"chr8" 6442001 6443000 "*" 0 0 -100
"chr8" 6513001 6514000 "*" 3.04324343503026e-11 7.26599935673121e-11 100
"chr8" 6671001 6672000 "*" 0 0 100
"chr8" 6684001 6685000 "*" 0 0 100
"chr8" 6720001 6721000 "*" 1.24197541140347e-10 2.73656217617171e-10
84.2857142857143
"chr8" 6741001 6742000 "*" 6.15840711759574e-13 1.83676803572802e-12 100
"chr8" 6778001 6779000 "*" 4.08209022140227e-11 9.57770690255255e-11 -100
"chr8" 6783001 6784000 "*" 9.04570973681018e-10 1.76414389822173e-09 -100
"chr8" 6900001 6901000 "*" 0 0 100
"chr8" 6933001 6934000 "*" 0 0 100
"chr8" 7004001 7005000 "*" 2.03601926429542e-07 2.83453119363615e-07
56.4516129032258
"chr8" 7212001 7213000 "*" 0 0 87.1794871794872
"chr8" 7537001 7538000 "*" 0 0 83.4839073969509
"chr8" 8076001 8077000 "*" 0 0 80
"chr8" 8104001 8105000 "*" 3.48387985127374e-13 1.07312013500854e-12
-65.1162790697674
"chr8" 8232001 8233000 "*" 7.93809462606987e-14 2.64679416473686e-13 100
"chr8" 8256001 8257000 "*" 7.105427357601e-15 2.68362283258525e-14 100
"chr8" 8378001 8379000 "*" 3.6700841921089e-08 5.65530226072258e-08 100
"chr8" 8508001 8509000 "*" 7.93809462606987e-14 2.64679416473686e-13 100
"chr8" 8634001 8635000 "*" 0 0 -100
"chr8" 8820001 8821000 "*" 0 0 66.3809523809524

"chr8" 9136001 9137000 "*" 0 0 100
"chr8" 9182001 9183000 "*" 1.2862481679754e-07 1.83930856926331e-07 -61.2244897959184
"chr8" 9194001 9195000 "*" 3.37344255862959e-08 5.2442256253974e-08 63.4146341463415
"chr8" 9226001 9227000 "*" 1.19232574924411e-08 1.97401088837468e-08 -80
"chr8" 9246001 9247000 "*" 1.70641278884887e-12 4.81986032810646e-12 100
"chr8" 9664001 9665000 "*" 3.6700841921089e-08 5.65530226072258e-08 -100
"chr8" 9714001 9715000 "*" 5.88032135206973e-08 8.83915617299364e-08 78.1609195402299
"chr8" 9721001 9722000 "*" 5.295763827462e-14 1.80214784640623e-13 100
"chr8" 10062001 10063000 "*" 6.24654217240561e-10 1.2462108191303e-09 100
"chr8" 10192001 10193000 "*" 0 0 54.304437269247
"chr8" 10235001 10236000 "*" 1.2825885142842e-07 1.83438986189409e-07 67.1875
"chr8" 10236001 10237000 "*" 0 0 70
"chr8" 10247001 10248000 "*" 2.68073363951515e-07 3.67619339864965e-07 -50.7462686567164
"chr8" 10270001 10271000 "*" 0 0 100
"chr8" 10356001 10357000 "*" 6.88338275267597e-15 2.60518659957509e-14 -100
"chr8" 10387001 10388000 "*" 1.12687636999453e-13 3.68543487142567e-13 100
"chr8" 10390001 10391000 "*" 1.89865900779296e-11 4.68032644334601e-11 70.3703703703704
"chr8" 10447001 10448000 "*" 7.00550728538474e-14 2.34936744632826e-13 51.0655642249913
"chr8" 10467001 10468000 "*" 0 0 55.4181929181929
"chr8" 10489001 10490000 "*" 1.28195547621779e-07 1.83361025228838e-07 -55
"chr8" 10520001 10521000 "*" 0 0 100
"chr8" 10528001 10529000 "*" 7.99844565668728e-06 8.89173797399106e-06 -66.6666666666667
"chr8" 10551001 10552000 "*" 8.61712656785585e-09 1.46086889771042e-08 76
"chr8" 10571001 10572000 "*" 0 0 92.5742574257426
"chr8" 10781001 10782000 "*" 8.9538466579997e-06 9.88218350385102e-06 -65.625
"chr8" 10784001 10785000 "*" 2.30149233004795e-13 7.24024232946363e-13 80.8080808080808
"chr8" 10802001 10803000 "*" 6.46149800331841e-14 2.17664365839349e-13 100
"chr8" 10804001 10805000 "*" 0.000105773223154859 9.87995681627625e-05 -53.8461538461538
"chr8" 10890001 10891000 "*" 1.47442391629227e-10 3.21123918856096e-10 86.6666666666667
"chr8" 10906001 10907000 "*" 0 0 100
"chr8" 10909001 10910000 "*" 3.11195513802431e-13 9.63433042984731e-13 95.6521739130435
"chr8" 10917001 10918000 "*" 0 0 82
"chr8" 10943001 10944000 "*" 1.2447216257705e-08 2.05634869406559e-08 -63.9344262295082
"chr8" 10974001 10975000 "*" 2.88779000712225e-11 6.92935809466068e-11 100
"chr8" 11107001 11108000 "*" 8.7349629751543e-09 1.47505871898511e-08 100
"chr8" 11203001 11204000 "*" 1.20281562487889e-12 3.46585342181251e-12 100
"chr8" 11275001 11276000 "*" 8.02931499066517e-10 1.57731825794538e-09 71.7948717948718
"chr8" 11276001 11277000 "*" 1.90181204118289e-13 6.05298617539364e-13 100
"chr8" 11287001 11288000 "*" 5.01025332333427e-10 1.01358031135889e-09 -100

"chr8" 11297001 11298000 "*" 0 0 65.7142857142857
"chr8" 11314001 11315000 "*" 3.6700841921089e-08 5.65530226072258e-08 100
"chr8" 11337001 11338000 "*" 5.08482145278322e-14 1.73432706170696e-13 -100
"chr8" 11372001 11373000 "*" 3.46098784187454e-07 4.67482227931052e-07
-69.6969696969697
"chr8" 11400001 11401000 "*" 5.55590351503099e-10 1.11681423342968e-09 82
"chr8" 11413001 11414000 "*" 1.06581410364015e-14 3.94854473541761e-14
-64.4444444444444
"chr8" 11429001 11430000 "*" 0 0 100
"chr8" 11436001 11437000 "*" 1.27950700035129e-08 2.11118141112855e-08
68.141592920354
"chr8" 11455001 11456000 "*" 0 0 100
"chr8" 11473001 11474000 "*" 6.30384633382164e-13 1.87665599796749e-12 -100
"chr8" 11483001 11484000 "*" 8.12683254025615e-13 2.39237407085304e-12
67.741935483871
"chr8" 11498001 11499000 "*" 0 0 69.2307692307692
"chr8" 11550001 11551000 "*" 0 0 75.5996422473372
"chr8" 11582001 11583000 "*" 2.22044604925031e-16 9.81641919380259e-16
70.5882352941177
"chr8" 11604001 11605000 "*" 9.51506096136256e-10 1.85087513909456e-09
88.034188034188
"chr8" 11619001 11620000 "*" 1.74202673397517e-08 2.81935902938494e-08
-66.6666666666667
"chr8" 11654001 11655000 "*" 2.88710388929303e-10 6.03399264731139e-10 100
"chr8" 11726001 11727000 "*" 9.28209731299035e-11 2.07681659354512e-10
-53.2760032760033
"chr8" 11769001 11770000 "*" 0 0 -95.6521739130435
"chr8" 11772001 11773000 "*" 6.99440505513849e-15 2.64480529781883e-14
-95.2380952380952
"chr8" 11811001 11812000 "*" 6.17816609493893e-06 6.98330769549985e-06
62.6666666666667
"chr8" 11831001 11832000 "*" 2.22044604925031e-16 9.81641919380259e-16 100
"chr8" 12237001 12238000 "*" 1.23906440663291e-11 3.12816210393607e-11
-68.8888888888889
"chr8" 12432001 12433000 "*" 6.90747459231034e-12 1.80140148623876e-11 100
"chr8" 12443001 12444000 "*" 0 0 100
"chr8" 12506001 12507000 "*" 1.52794177310511e-09 2.87642564232045e-09 100
"chr8" 12571001 12572000 "*" 0 0 100
"chr8" 12809001 12810000 "*" 1.99840144432528e-15 8.0570469535907e-15
62.3574144486692
"chr8" 12862001 12863000 "*" 0 0 -100
"chr8" 12980001 12981000 "*" 8.01666433236647e-09 1.36392368970109e-08 100
"chr8" 12989001 12990000 "*" 0 0 61.9117853631128
"chr8" 12990001 12991000 "*" 0 0 57.0176440824587
"chr8" 13204001 13205000 "*" 4.26951363152739e-10 8.72510873516957e-10 100
"chr8" 13207001 13208000 "*" 4.73234496034536e-09 8.30627300264826e-09 -100
"chr8" 13379001 13380000 "*" 7.03970086335381e-07 9.12522359725664e-07
58.3333333333333
"chr8" 13656001 13657000 "*" 5.48948864776966e-05 5.36254382701292e-05 75
"chr8" 14755001 14756000 "*" 9.65729496371637e-10 1.87255912493232e-09 100
"chr8" 15857001 15858000 "*" 7.61612994892857e-14 2.54524180939423e-13
-75.4716981132076

"chr8" 17192001 17193000 "*" 3.33066907387547e-16 1.4495649018245e-15 -100
"chr8" 17289001 17290000 "*" 7.78377362564697e-13 2.29551678017433e-12 -100
"chr8" 17486001 17487000 "*" 0 0 62.3853211009174
"chr8" 18343001 18344000 "*" 2.37587727269783e-14 8.44268514944447e-14 100
"chr8" 18358001 18359000 "*" 6.4152538836737e-10 1.27477697728417e-09 -100
"chr8" 18628001 18629000 "*" 0 0 100
"chr8" 18859001 18860000 "*" 0 0 100
"chr8" 18910001 18911000 "*" 1.58067972821918e-07 2.22994335091428e-07 -62.5
"chr8" 18929001 18930000 "*" 0 0 -100
"chr8" 19071001 19072000 "*" 1.4432899320127e-15 5.90750815956055e-15 100
"chr8" 19171001 19172000 "*" 0 0 -61.965811965812
"chr8" 19172001 19173000 "*" 1.11188835916209e-12 3.21640817545736e-12
-74.6666666666667
"chr8" 19268001 19269000 "*" 0 0 100
"chr8" 19385001 19386000 "*" 0 0 100
"chr8" 19493001 19494000 "*" 1.28256072429167e-10 2.81876405375473e-10
-70.3703703703704
"chr8" 19588001 19589000 "*" 0 0 -100
"chr8" 19615001 19616000 "*" 0 0 55.1181102362205
"chr8" 19626001 19627000 "*" 7.0006535457523e-08 1.03695832512054e-07 -100
"chr8" 19796001 19797000 "*" 4.10682599039092e-12 1.10487790300602e-11
89.1891891891892
"chr8" 20036001 20037000 "*" 1.90032345415148e-09 3.54409312695040e-09
-51.5151515151515
"chr8" 20054001 20055000 "*" 0 0 -92.2705314009662
"chr8" 20102001 20103000 "*" 7.37376937287593e-10 1.45439765630417e-09
-53.8461538461538
"chr8" 20119001 20120000 "*" 1.12622297820941e-06 1.41959920389863e-06
79.1666666666667
"chr8" 20129001 20130000 "*" 9.30776011820456e-10 1.81270708267562e-09
-53.0434782608696
"chr8" 20145001 20146000 "*" 1.13140401492018e-08 1.87801380787728e-08 100
"chr8" 20161001 20162000 "*" 0 0 51.5831390143005
"chr8" 20188001 20189000 "*" 9.88564806680969e-08 1.43508370397219e-07
-60.8695652173913
"chr8" 20207001 20208000 "*" 0 0 -50.5799755799756
"chr8" 20482001 20483000 "*" 1.82686751662997e-05 1.92373864166158e-05
53.8461538461538
"chr8" 20676001 20677000 "*" 8.51605785889831e-10 1.66802527211457e-09
64.8648648648649
"chr8" 20853001 20854000 "*" 1.11022302462516e-16 5.03662826618488e-16 -100
"chr8" 20893001 20894000 "*" 0 0 -100
"chr8" 20908001 20909000 "*" 3.56775498033812e-10 7.35898629809829e-10 100
"chr8" 21022001 21023000 "*" 2.15125472990962e-10 4.58126659943874e-10 -100
"chr8" 21119001 21120000 "*" 1.11022302462516e-16 5.03662826618488e-16 100
"chr8" 21376001 21377000 "*" 1.87405646556726e-13 5.97285292681429e-13 100
"chr8" 21438001 21439000 "*" 2.74687161905263e-10 5.76953704674915e-10
-68.2926829268293
"chr8" 21472001 21473000 "*" 7.0006535457523e-08 1.03695832512054e-07 100
"chr8" 21503001 21504000 "*" 2.68450373042128e-10 5.64465213127486e-10 -100
"chr8" 21552001 21553000 "*" 4.44089209850063e-16 1.91071758245033e-15
75.4098360655738

"chr8" 21580001 21581000 "*" 3.22375459660407e-12 8.79500753078322e-12 -75
"chr8" 21588001 21589000 "*" 1.73605574360636e-12 4.89927448973084e-12
90.9090909090909
"chr8" 21603001 21604000 "*" 4.79616346638068e-14 1.64380445416596e-13 100
"chr8" 21613001 21614000 "*" 1.4413575866179e-07 2.04331436328624e-07
-79.4871794871795
"chr8" 21696001 21697000 "*" 0 0 -73.469387755102
"chr8" 21700001 21701000 "*" 2.08814465718632e-09 3.86232567960463e-09 100
"chr8" 21868001 21869000 "*" 0 0 60.4903786468032
"chr8" 21912001 21913000 "*" 8.5086138135182e-10 1.66684124279099e-09
-54.5454545454545
"chr8" 21939001 21940000 "*" 1.79511534192933e-07 2.51609216903976e-07
-52.8411850412822
"chr8" 21988001 21989000 "*" 0 0 60.9086946352251
"chr8" 21995001 21996000 "*" 6.4152538836737e-10 1.27477697728417e-09 100
"chr8" 22004001 22005000 "*" 1.87405646556726e-13 5.97285292681429e-13 -100
"chr8" 22010001 22011000 "*" 2.32087844369566e-05 2.40487702824021e-05
53.3333333333333
"chr8" 22014001 22015000 "*" 0 0 70.0178206059006
"chr8" 22021001 22022000 "*" 0 0 62.3241951772211
"chr8" 22223001 22224000 "*" 5.05617354867871e-05 4.96986880174839e-05
-62.9545454545455
"chr8" 22241001 22242000 "*" 1.32693855903199e-12 3.80654217518773e-12
-55.444587628866
"chr8" 22298001 22299000 "*" 0 0 62.640306122449
"chr8" 22299001 22300000 "*" 0 0 97.2477064220184
"chr8" 22325001 22326000 "*" 3.06532577099006e-13 9.49949114669084e-13
-66.6666666666667
"chr8" 22437001 22438000 "*" 0 0 -63.7101785492357
"chr8" 22454001 22455000 "*" 0 0 100
"chr8" 22457001 22458000 "*" 0 0 -71.4285714285714
"chr8" 22480001 22481000 "*" 0 0 -96.6666666666667
"chr8" 22536001 22537000 "*" 3.04324343503026e-11 7.26599935673121e-11 -100
"chr8" 22549001 22550000 "*" 0 0 51.0511475175786
"chr8" 22551001 22552000 "*" 0 0 -73.6842105263158
"chr8" 22558001 22559000 "*" 5.6362137179633e-11 1.29963270300065e-10 -100
"chr8" 22571001 22572000 "*" 7.66053886991358e-15 2.88426834415713e-14 60
"chr8" 22573001 22574000 "*" 0 0 86.231884057971
"chr8" 22576001 22577000 "*" 2.52417531321214e-10 5.33037880173179e-10
58.3333333333333
"chr8" 22602001 22603000 "*" 0 0 100
"chr8" 22611001 22612000 "*" 6.4152538836737e-10 1.27477697728417e-09 100
"chr8" 22632001 22633000 "*" 2.22044604925031e-16 9.81641919380259e-16
-75.8064516129032
"chr8" 22639001 22640000 "*" 1.11022302462516e-16 5.03662826618488e-16 -100
"chr8" 22657001 22658000 "*" 6.04438721296674e-11 1.38849255306602e-10 100
"chr8" 22661001 22662000 "*" 0 0 -100
"chr8" 22672001 22673000 "*" 1.16573417585641e-14 4.29335582900508e-14 -100
"chr8" 22710001 22711000 "*" 2.08814465718632e-09 3.86232567960463e-09 -100
"chr8" 22749001 22750000 "*" 1.20735643704961e-11 3.05060495458033e-11
51.8072289156626
"chr8" 22844001 22845000 "*" 4.19435610332641e-06 4.85857056843185e-06

-61.5384615384615
"chr8" 23019001 23020000 "*" 2.33028818463765e-10 4.93719283454501e-10 -100
"chr8" 23161001 23162000 "*" 2.14717132962505e-13 6.78727961658884e-13
-54.9107142857143
"chr8" 23172001 23173000 "*" 2.3990809339125e-12 6.65441738642456e-12 100
"chr8" 23193001 23194000 "*" 2.77555756156289e-15 1.10015415195978e-14
52.8571428571429
"chr8" 23399001 23400000 "*" 0 0 -100
"chr8" 23621001 23622000 "*" 7.03992419914812e-12 1.83313429961219e-11 100
"chr8" 24529001 24530000 "*" 1.37828859436695e-10 3.01445544267661e-10 100
"chr8" 24679001 24680000 "*" 8.39749638414933e-06 9.30620475488742e-06
65.9574468085106
"chr8" 24773001 24774000 "*" 1.86517468137026e-14 6.70995420767425e-14 100
"chr8" 25055001 25056000 "*" 3.6700841921089e-08 5.65530226072258e-08 100
"chr8" 25179001 25180000 "*" 6.38945786279876e-08 9.55539447381879e-08
57.4468085106383
"chr8" 25400001 25401000 "*" 4.26699914291007e-08 6.52806072729571e-08 -80
"chr8" 25479001 25480000 "*" 1.2890753294803e-06 1.61243929530756e-06
57.6923076923077
"chr8" 25543001 25544000 "*" 5.10702591327572e-15 1.96271840288234e-14 100
"chr8" 25765001 25766000 "*" 4.51370262688489e-06 5.20628946429878e-06
66.6666666666667
"chr8" 25792001 25793000 "*" 0 0 100
"chr8" 25860001 25861000 "*" 1.29867794296246e-09 2.47408848239392e-09 70
"chr8" 25868001 25869000 "*" 0 0 52.3076923076923
"chr8" 26115001 26116000 "*" 4.73234496034536e-09 8.30627300264826e-09 100
"chr8" 26332001 26333000 "*" 1.14566689468631e-10 2.53567797653911e-10
70.9677419354839
"chr8" 26339001 26340000 "*" 2.08995487582797e-10 4.46944006972663e-10 100
"chr8" 26421001 26422000 "*" 4.65637306490407e-10 9.46720524046618e-10 100
"chr8" 26435001 26436000 "*" 0 0 100
"chr8" 26441001 26442000 "*" 8.65973959207622e-15 3.24130830964561e-14 100
"chr8" 26456001 26457000 "*" 1.0325074129014e-14 3.83008663711937e-14 -100
"chr8" 26486001 26487000 "*" 0 0 -80
"chr8" 26499001 26500000 "*" 8.88178419700125e-16 3.70670032207938e-15
-68.2926829268293
"chr8" 26506001 26507000 "*" 6.99440505513849e-15 2.64480529781883e-14 100
"chr8" 26541001 26542000 "*" 2.66453525910038e-14 9.40074020513994e-14 100
"chr8" 26629001 26630000 "*" 1.54443136057836e-08 2.52344353683277e-08
84.6153846153846
"chr8" 26773001 26774000 "*" 3.6700841921089e-08 5.65530226072258e-08 -100
"chr8" 26870001 26871000 "*" 5.88418203051333e-15 2.245355428447e-14 -100
"chr8" 26980001 26981000 "*" 3.54213828098748e-05 3.5645209334001e-05
61.4285714285714
"chr8" 27102001 27103000 "*" 9.02722341322715e-13 2.63704632001509e-12
70.3389830508475
"chr8" 27178001 27179000 "*" 6.4324989779152e-11 1.47230020825092e-10 100
"chr8" 27221001 27222000 "*" 1.98365768255826e-11 4.85612402473442e-11 -100
"chr8" 27233001 27234000 "*" 1.67334377465522e-08 2.71418641479927e-08
-76.4705882352941
"chr8" 27317001 27318000 "*" 1.22457599616155e-12 3.52441478267078e-12 -100
"chr8" 27452001 27453000 "*" 1.67299207820548e-08 2.71377429441055e-08 100

"chr8" 27471001 27472000 "*" 0 0 -100
"chr8" 27529001 27530000 "*" 3.56775498033812e-10 7.35898629809829e-10 100
"chr8" 27695001 27696000 "*" 7.54951656745106e-15 2.84375115626978e-14
-92.6470588235294
"chr8" 27754001 27755000 "*" 0 0 100
"chr8" 27758001 27759000 "*" 9.44608870012686e-08 1.37464732372737e-07
55.0847457627119
"chr8" 27759001 27760000 "*" 1.91224813761437e-12 5.36527594469447e-12 -100
"chr8" 27765001 27766000 "*" 6.92287338566189e-11 1.57605630124247e-10 -100
"chr8" 27816001 27817000 "*" 1.58671964456403e-11 3.94719096379376e-11
66.6666666666667
"chr8" 27839001 27840000 "*" 1.53773566102799e-08 2.51299576527202e-08
-61.0007639419404
"chr8" 27918001 27919000 "*" 1.0500116598422e-07 1.51960339170451e-07
-62.962962962963
"chr8" 27923001 27924000 "*" 4.01313771103418e-09 7.11882087237587e-09 100
"chr8" 28176001 28177000 "*" 1.61285762523278e-10 3.49420989251166e-10
64.2857142857143
"chr8" 28209001 28210000 "*" 1.14130926931466e-13 3.7276674571693e-13 -100
"chr8" 28269001 28270000 "*" 0 0 -72.3926380368098
"chr8" 28284001 28285000 "*" 3.63445940010365e-11 8.58565209111204e-11 -100
"chr8" 28454001 28455000 "*" 5.91870164878827e-07 7.75564979754954e-07
57.6923076923077
"chr8" 28924001 28925000 "*" 1.22124532708767e-15 5.03921984217778e-15 -100
"chr8" 29121001 29122000 "*" 1.16068954447712e-09 2.22304092260494e-09
68.4210526315789
"chr8" 29323001 29324000 "*" 7.55853750034063e-07 9.75771469042378e-07
76.9230769230769
"chr8" 29452001 29453000 "*" 4.73234496034536e-09 8.30627300264826e-09 -100
"chr8" 29678001 29679000 "*" 3.7464975566337e-10 7.71164685864348e-10
73.6842105263158
"chr8" 29710001 29711000 "*" 6.76684073219747e-08 1.00866692882737e-07
-64.1509433962264
"chr8" 29732001 29733000 "*" 3.62488892768908e-06 4.24279146420347e-06
67.8571428571429
"chr8" 30835001 30836000 "*" 0 0 -100
"chr8" 31259001 31260000 "*" 0 0 100
"chr8" 31883001 31884000 "*" 0 0 -93.3333333333333
"chr8" 32156001 32157000 "*" 0 0 100
"chr8" 32170001 32171000 "*" 0 0 100
"chr8" 32504001 32505000 "*" 1.70303771085401e-11 4.2226939009068e-11
66.0194174757282
"chr8" 32614001 32615000 "*" 7.0006535457523e-08 1.03695832512054e-07 100
"chr8" 32621001 32622000 "*" 0 0 -91.2087912087912
"chr8" 33189001 33190000 "*" 1.57966116082209e-05 1.67960480619754e-05
61.9047619047619
"chr8" 33423001 33424000 "*" 0 0 -58.8541666666667
"chr8" 34497001 34498000 "*" 1.11022302462516e-16 5.03662826618488e-16 -100
"chr8" 35579001 35580000 "*" 4.15225354100102e-09 7.35199115373493e-09
86.3157894736842
"chr8" 36314001 36315000 "*" 2.19853864003028e-08 3.50003158916803e-08
-66.6666666666667

"chr8" 36485001 36486000 "*" 4.34330383459747e-05 4.31208808738151e-05
-59.5744680851064
"chr8" 36939001 36940000 "*" 6.4152538836737e-10 1.27477697728417e-09 -100
"chr8" 37038001 37039000 "*" 9.81047576509297e-10 1.89842111513741e-09
-76.890756302521
"chr8" 37075001 37076000 "*" 1.02560743453495e-07 1.48614175511706e-07
-66.6666666666667
"chr8" 37155001 37156000 "*" 1.89581683684992e-12 5.32311367606535e-12 100
"chr8" 37189001 37190000 "*" 1.11022302462516e-16 5.03662826618488e-16 100
"chr8" 37222001 37223000 "*" 0 0 100
"chr8" 37309001 37310000 "*" 0 0 100
"chr8" 37361001 37362000 "*" 2.37587727269783e-14 8.44268514944447e-14 -100
"chr8" 37372001 37373000 "*" 0 0 -100
"chr8" 37385001 37386000 "*" 0 0 -68.5714285714286
"chr8" 37451001 37452000 "*" 0 0 100
"chr8" 37457001 37458000 "*" 0 0 100
"chr8" 37556001 37557000 "*" 2.33146835171283e-14 8.30185660898472e-14
52.5585525585526
"chr8" 37641001 37642000 "*" 4.09672296086683e-14 1.41337614887091e-13
-52.0089285714286
"chr8" 37836001 37837000 "*" 9.32032229172819e-13 2.7181920310254e-12 100
"chr8" 37892001 37893000 "*" 4.01313771103418e-09 7.11882087237587e-09 100
"chr8" 38009001 38010000 "*" 0 0 -100
"chr8" 38126001 38127000 "*" 0 0 -60.2693602693603
"chr8" 38243001 38244000 "*" 0 0 100
"chr8" 38244001 38245000 "*" 0 0 99.7630331753554
"chr8" 38299001 38300000 "*" 6.4324989779152e-11 1.47230020825092e-10 100
"chr8" 38325001 38326000 "*" 0 0 75.9745770615336
"chr8" 38326001 38327000 "*" 0 0 70.8370199714492
"chr8" 38385001 38386000 "*" 0 0 -60.1110537190083
"chr8" 38486001 38487000 "*" 1.25284005392245e-10 2.75649590272477e-10 -100
"chr8" 38507001 38508000 "*" 1.522571191237e-05 1.62273010025363e-05
-50.1424501424501
"chr8" 38568001 38569000 "*" 3.76365605347928e-14 1.3043487970679e-13 -100
"chr8" 38577001 38578000 "*" 1.0715239806558e-10 2.38086710553983e-10
-79.6992481203008
"chr8" 38614001 38615000 "*" 2.70450328798688e-12 7.44930345040928e-12
-71.6857610474632
"chr8" 38646001 38647000 "*" 1.52794177310511e-09 2.87642564232045e-09 -100
"chr8" 38729001 38730000 "*" 0 0 -100
"chr8" 38847001 38848000 "*" 1.67299207820548e-08 2.71377429441055e-08 -100
"chr8" 39302001 39303000 "*" 3.6700841921089e-08 5.65530226072258e-08 100
"chr8" 39810001 39811000 "*" 2.00227675550835e-08 3.20459176617961e-08 100
"chr8" 39853001 39854000 "*" 1.77396024536591e-06 2.17512049368634e-06
66.6666666666667
"chr8" 39912001 39913000 "*" 1.07882591748876e-11 2.74553537455803e-11 -100
"chr8" 40264001 40265000 "*" 2.8966828935495e-12 7.94035734501975e-12
-83.3333333333333
"chr8" 40388001 40389000 "*" 4.01313771103418e-09 7.11882087237587e-09 100
"chr8" 40457001 40458000 "*" 5.51780843238703e-14 1.87243792572872e-13 -100
"chr8" 40654001 40655000 "*" 1.39779633423487e-07 1.98463037958589e-07 -100
"chr8" 40707001 40708000 "*" 8.7349629751543e-09 1.47505871898511e-08 -100

"chr8" 40744001 40745000 "*" 8.01666433236647e-09 1.36392368970109e-08 100
"chr8" 40960001 40961000 "*" 0 0 100
"chr8" 40976001 40977000 "*" 0.000171951107246215 0.000155141795915547
53.4883720930233
"chr8" 40982001 40983000 "*" 1.56863411149288e-12 4.4469621057462e-12 100
"chr8" 40986001 40987000 "*" 3.55123767059595e-08 5.50471549649875e-08
58.3333333333333
"chr8" 40999001 41000000 "*" 1.49613654798486e-12 4.26244554429779e-12 -100
"chr8" 41136001 41137000 "*" 4.02167188440217e-12 1.08266700497926e-11 -100
"chr8" 41142001 41143000 "*" 0 0 83.0769230769231
"chr8" 41158001 41159000 "*" 4.45065095888708e-11 1.04044004916479e-10
-58.8235294117647
"chr8" 41162001 41163000 "*" 1.56863411149288e-12 4.4469621057462e-12 100
"chr8" 41173001 41174000 "*" 0 0 100
"chr8" 41188001 41189000 "*" 6.24005077609624e-08 9.34435715465956e-08
52.3809523809524
"chr8" 41192001 41193000 "*" 1.87405646556726e-13 5.97285292681429e-13 100
"chr8" 41310001 41311000 "*" 3.90831811358794e-12 1.05610567215048e-11 100
"chr8" 41347001 41348000 "*" 6.66133814775094e-16 2.81595744474255e-15
-77.5862068965517
"chr8" 41467001 41468000 "*" 6.4152538836737e-10 1.27477697728417e-09 100
"chr8" 41521001 41522000 "*" 2.22044604925031e-16 9.81641919380259e-16 100
"chr8" 41525001 41526000 "*" 0 0 -93.1506849315068
"chr8" 41557001 41558000 "*" 2.88779000712225e-11 6.92935809466068e-11 -100
"chr8" 41607001 41608000 "*" 8.80406858527749e-14 2.91456141063452e-13 -100
"chr8" 41646001 41647000 "*" 9.0072393987839e-13 2.63157725646847e-12 100
"chr8" 41689001 41690000 "*" 1.07865402965146e-06 1.36322374054195e-06
72.8155339805825
"chr8" 41702001 41703000 "*" 4.72297404041644e-06 5.43376869874213e-06
67.1328671328671
"chr8" 41709001 41710000 "*" 0 0 69.7674418604651
"chr8" 41722001 41723000 "*" 3.33066907387547e-16 1.4495649018245e-15 100
"chr8" 41739001 41740000 "*" 1.20925511013503e-05 1.30850105740421e-05
68.1818181818182
"chr8" 41765001 41766000 "*" 4.32986979603811e-15 1.68006959513174e-14 100
"chr8" 41773001 41774000 "*" 0 0 100
"chr8" 42011001 42012000 "*" 0 0 74.7663551401869
"chr8" 42051001 42052000 "*" 1.20610797268439e-05 1.30541743073331e-05
-53.8461538461538
"chr8" 42056001 42057000 "*" 9.63829016598083e-12 2.46993429847588e-11 100
"chr8" 42060001 42061000 "*" 0 0 63.2394366197183
"chr8" 42241001 42242000 "*" 1.41778362938716e-07 2.01170748993017e-07
66.6666666666667
"chr8" 42248001 42249000 "*" 0 0 100
"chr8" 42270001 42271000 "*" 0 0 -62.1951219512195
"chr8" 42449001 42450000 "*" 5.10702591327572e-15 1.96271840288234e-14 100
"chr8" 42476001 42477000 "*" 3.31690230837012e-12 9.03347040781321e-12 100
"chr8" 42485001 42486000 "*" 1.50175871738156e-10 3.26344416764221e-10
84.3137254901961
"chr8" 42904001 42905000 "*" 1.61237689866311e-12 4.56615813671789e-12 100
"chr8" 42958001 42959000 "*" 8.06610334080915e-12 2.08931809569875e-11
57.6923076923077

"chr8" 43144001 43145000 "*" 9.99200722162641e-16 4.15382497462808e-15
90.8496732026144
"chr8" 46927001 46928000 "*" 4.16718881623979e-10 8.53230677040051e-10
70.4225352112676
"chr8" 46934001 46935000 "*" 4.27069934971769e-09 7.55257312576703e-09
81.0344827586207
"chr8" 46939001 46940000 "*" 1.34276423313651e-09 2.55469187219557e-09
54.0780141843972
"chr8" 46941001 46942000 "*" 1.45439216225896e-14 5.29728884617264e-14
75.5725190839695
"chr8" 47002001 47003000 "*" 0.000281157030617152 0.00024485915731249
53.8461538461538
"chr8" 47006001 47007000 "*" 3.83429898498377e-07 5.14451749900013e-07 85
"chr8" 47012001 47013000 "*" 0 0 100
"chr8" 47019001 47020000 "*" 3.12379471197888e-05 3.1728595486819e-05
-51.6129032258064
"chr8" 47022001 47023000 "*" 0 0 94.4444444444444
"chr8" 47023001 47024000 "*" 3.10862446895044e-15 1.22466437093774e-14 100
"chr8" 47029001 47030000 "*" 0 0 60
"chr8" 47030001 47031000 "*" 1.29037891483108e-11 3.24582280286866e-11 100
"chr8" 47056001 47057000 "*" 2.04977146367469e-11 5.01106166492934e-11
82.1138211382114
"chr8" 47094001 47095000 "*" 4.22763535290471e-09 7.47926975872859e-09
79.3103448275862
"chr8" 47095001 47096000 "*" 1.62978062701047e-06 2.00867221623465e-06
-52.6315789473684
"chr8" 47101001 47102000 "*" 0 0 89.2307692307692
"chr8" 47107001 47108000 "*" 0 0 -100
"chr8" 47114001 47115000 "*" 8.42683375434916e-06 9.3361115911752e-06
-55.1282051282051
"chr8" 47121001 47122000 "*" 4.7867645402988e-09 8.3982560352312e-09
-86.3636363636364
"chr8" 47125001 47126000 "*" 0 0 100
"chr8" 47143001 47144000 "*" 8.90065798841988e-13 2.60417849402953e-12
71.4285714285714
"chr8" 47145001 47146000 "*" 0 0 58.8550159517901
"chr8" 47152001 47153000 "*" 6.91668944341473e-14 2.32228953423403e-13
57.2727272727273
"chr8" 47160001 47161000 "*" 3.18967074974807e-13 9.86980164111026e-13
60.3462355054712
"chr8" 47162001 47163000 "*" 2.07785471293409e-05 2.16902849064988e-05
-60.7142857142857
"chr8" 47164001 47165000 "*" 2.1094237467878e-15 8.47434540879246e-15
57.9545454545455
"chr8" 47184001 47185000 "*" 6.4152538836737e-10 1.27477697728417e-09 100
"chr8" 47185001 47186000 "*" 1.38590361409285e-10 3.02706178892116e-10 100
"chr8" 47187001 47188000 "*" 1.756372824957e-13 5.61860756166042e-13
63.8888888888889
"chr8" 47252001 47253000 "*" 6.13999610421523e-06 6.94260980394374e-06
-72.2222222222222
"chr8" 47286001 47287000 "*" 0 0 -82.0652173913043
"chr8" 47292001 47293000 "*" 1.85877979674842e-10 4.00587949461252e-10

61.5384615384615
"chr8" 47295001 47296000 "*" 0 0 71.2765957446808
"chr8" 47298001 47299000 "*" 2.08995487582797e-10 4.46944006972663e-10 100
"chr8" 47299001 47300000 "*" 0 0 67.1794871794872
"chr8" 47300001 47301000 "*" 5.17794531418314e-06 5.92565750755961e-06
-50.8333333333333
"chr8" 47321001 47322000 "*" 0 0 100
"chr8" 47327001 47328000 "*" 1.83952852950142e-12 5.17499701329218e-12 -100
"chr8" 47334001 47335000 "*" 1.14508291737536e-10 2.53458726073407e-10
56.6037735849057
"chr8" 47343001 47344000 "*" 0 0 100
"chr8" 47344001 47345000 "*" 0 0 95.5696202531646
"chr8" 47349001 47350000 "*" 0 0 82.7272727272727
"chr8" 47351001 47352000 "*" 4.08209022140227e-11 9.57770690255255e-11 100
"chr8" 47352001 47353000 "*" 1.39779633423487e-07 1.98463037958589e-07 -100
"chr8" 47356001 47357000 "*" 1.86517468137026e-14 6.70995420767425e-14 100
"chr8" 47566001 47567000 "*" 1.39779633423487e-07 1.98463037958589e-07 100
"chr8" 47813001 47814000 "*" 3.34128893442198e-08 5.19772864618523e-08 100
"chr8" 47826001 47827000 "*" 9.80763359414993e-10 1.8980032424715e-09 100
"chr8" 47926001 47927000 "*" 0 0 100
"chr8" 48045001 48046000 "*" 1.38195899168636e-10 3.02200747503033e-10
85.6209150326797
"chr8" 48068001 48069000 "*" 1.14908083048704e-13 3.75128751466461e-13
66.6666666666667
"chr8" 48275001 48276000 "*" 1.93720595120794e-11 4.76309924836636e-11 -100
"chr8" 48647001 48648000 "*" 1.61726187997147e-12 4.57905920600701e-12
-56.7154255319149
"chr8" 48649001 48650000 "*" 2.1094237467878e-14 7.54243990081593e-14
-83.1932773109244
"chr8" 48993001 48994000 "*" 3.62598839842576e-13 1.11545114393694e-12
80.3921568627451
"chr8" 49029001 49030000 "*" 0.000301667553284757 0.000261300142255719
54.0540540540541
"chr8" 49062001 49063000 "*" 3.68371999570627e-13 1.13149976091305e-12 -100
"chr8" 49086001 49087000 "*" 3.6700841921089e-08 5.65530226072258e-08 100
"chr8" 49089001 49090000 "*" 1.88681847923533e-10 4.06252681614896e-10
67.1232876712329
"chr8" 49091001 49092000 "*" 2.17381668221606e-13 6.85151076607736e-13 -100
"chr8" 49178001 49179000 "*" 2.73896461067125e-11 6.60588656753372e-11
59.4155844155844
"chr8" 49183001 49184000 "*" 0 0 -97.8723404255319
"chr8" 49187001 49188000 "*" 0 0 100
"chr8" 49189001 49190000 "*" 1.98388849792508e-09 3.68880553433621e-09 75
"chr8" 49195001 49196000 "*" 4.44089209850063e-16 1.91071758245033e-15 60
"chr8" 49196001 49197000 "*" 0 0 100
"chr8" 49230001 49231000 "*" 0 0 76.4705882352941
"chr8" 49252001 49253000 "*" 1.16573417585641e-14 4.29335582900508e-14 100
"chr8" 49362001 49363000 "*" 1.89581683684992e-12 5.32311367606535e-12 100
"chr8" 49363001 49364000 "*" 1.5277158427196e-09 2.87642564232045e-09 100
"chr8" 49417001 49418000 "*" 1.29037891483108e-11 3.24582280286866e-11 100
"chr8" 49418001 49419000 "*" 3.67118602184746e-09 6.57390172683815e-09
74.7849462365591

"chr8" 49419001 49420000 "*" 1.48972777447298e-08 2.43864989177518e-08 79.7101449275362
"chr8" 49436001 49437000 "*" 0 0 100
"chr8" 49438001 49439000 "*" 3.00492018601162e-07 4.09250025069288e-07 71.6216216216216
"chr8" 49469001 49470000 "*" 0 0 77.7777777777778
"chr8" 49508001 49509000 "*" 9.63829016598083e-12 2.46993429847588e-11 100
"chr8" 49510001 49511000 "*" 2.22044604925031e-16 9.81641919380259e-16 100
"chr8" 49512001 49513000 "*" 6.4152538836737e-10 1.27477697728417e-09 100
"chr8" 49536001 49537000 "*" 1.34559030584569e-13 4.35581312463895e-13 100
"chr8" 49546001 49547000 "*" 0 0 80
"chr8" 49583001 49584000 "*" 1.88737914186277e-15 7.62011598380321e-15 100
"chr8" 49730001 49731000 "*" 0.000112925579479284 0.000105045585222338 -62.5
"chr8" 49736001 49737000 "*" 6.97602285604226e-06 7.82816889037512e-06 60
"chr8" 49781001 49782000 "*" 0 0 100
"chr8" 49826001 49827000 "*" 0 0 100
"chr8" 49885001 49886000 "*" 0.000114948885499011 0.000106778832916254 55.5555555555556
"chr8" 49932001 49933000 "*" 6.59550958292954e-09 1.13456194382773e-08 -100
"chr8" 49960001 49961000 "*" 4.66293670342566e-15 1.80217883586487e-14 62.4113475177305
"chr8" 50173001 50174000 "*" 2.43005615629954e-09 4.45014222280278e-09 -100
"chr8" 50377001 50378000 "*" 3.38229444452054e-12 9.20645280775935e-12 -74.5454545454545
"chr8" 50823001 50824000 "*" 0 0 93.2038834951456
"chr8" 51267001 51268000 "*" 3.04324343503026e-11 7.26599935673121e-11 100
"chr8" 51369001 51370000 "*" 2.08814465718632e-09 3.86232567960463e-09 100
"chr8" 52367001 52368000 "*" 7.32955945936808e-08 1.08233217915758e-07 80
"chr8" 52433001 52434000 "*" 9.41399987630653e-07 1.19926399797973e-06 68.6567164179104
"chr8" 52593001 52594000 "*" 2.75837870061801e-05 2.82530803747995e-05 -66.6666666666667
"chr8" 52950001 52951000 "*" 2.93066615419946e-08 4.59876196816e-08 -76.1904761904762
"chr8" 53013001 53014000 "*" 3.27325439331005e-06 3.85674099375811e-06 -63.5170603674541
"chr8" 53167001 53168000 "*" 6.88338275267597e-15 2.60518659957509e-14 100
"chr8" 53252001 53253000 "*" 1.60460099830617e-05 1.70453625649539e-05 68.5714285714286
"chr8" 53299001 53300000 "*" 1.12458486967171e-09 2.15740815043925e-09 -100
"chr8" 53302001 53303000 "*" 1.3164671464061e-08 2.16920433760696e-08 -51.1904761904762
"chr8" 53321001 53322000 "*" 3.22552687803679e-06 3.80464443926623e-06 -56.0975609756098
"chr8" 53322001 53323000 "*" 0 0 -57.6878612716763
"chr8" 53397001 53398000 "*" 2.64951482975562e-09 4.82186011375752e-09 100
"chr8" 53454001 53455000 "*" 7.7715611723761e-16 3.26213507634405e-15 -100
"chr8" 53757001 53758000 "*" 1.58346346035287e-08 2.58271480686597e-08 78.8461538461538
"chr8" 54416001 54417000 "*" 3.6374154733787e-06 4.25659102342033e-06 55.1724137931034
"chr8" 54579001 54580000 "*" 6.44564628715649e-06 7.26663863268796e-06

52.1739130434783
"chr8" 54605001 54606000 "*" 0 0 69.2349137931034
"chr8" 54643001 54644000 "*" 7.88258347483861e-15 2.96145301916012e-14 100
"chr8" 54795001 54796000 "*" 0 0 61.9635040645785
"chr8" 54928001 54929000 "*" 7.0006535457523e-08 1.03695832512054e-07 100
"chr8" 55035001 55036000 "*" 3.33066907387547e-16 1.4495649018245e-15 -100
"chr8" 55070001 55071000 "*" 9.89881233892653e-06 1.08554502667777e-05
-68.4210526315789
"chr8" 55158001 55159000 "*" 4.32209823486573e-12 1.15935290479641e-11 100
"chr8" 55162001 55163000 "*" 0 0 67.6700201207243
"chr8" 55209001 55210000 "*" 1.12055920098442e-11 2.84500040664577e-11 -100
"chr8" 55264001 55265000 "*" 1.45784415650496e-08 2.38945874392173e-08
57.6923076923077
"chr8" 55267001 55268000 "*" 1.26565424807268e-14 4.63923203146481e-14 -100
"chr8" 55340001 55341000 "*" 3.40125705378114e-10 7.03784073980162e-10 -100
"chr8" 55377001 55378000 "*" 1.57087676200263e-11 3.90941205551801e-11 93.75
"chr8" 55771001 55772000 "*" 5.48638023900594e-10 1.10374357336421e-09 100
"chr8" 55779001 55780000 "*" 4.78841410966879e-11 1.11468187156588e-10 100
"chr8" 55783001 55784000 "*" 0 0 100
"chr8" 56013001 56014000 "*" 0 0 70.1019940636268
"chr8" 56014001 56015000 "*" 0 0 56.0987101148653
"chr8" 56112001 56113000 "*" 0 0 100
"chr8" 56171001 56172000 "*" 2.40868092141744e-05 2.49033877499598e-05
63.4920634920635
"chr8" 56212001 56213000 "*" 8.01666433236647e-09 1.36392368970109e-08 100
"chr8" 56255001 56256000 "*" 3.95353005888666e-09 7.03928529283733e-09 100
"chr8" 56277001 56278000 "*" 4.88099984383705e-05 4.80839580539796e-05
52.9411764705882
"chr8" 56321001 56322000 "*" 8.57092175010621e-13 2.51149198099779e-12 100
"chr8" 56324001 56325000 "*" 3.07317337100432e-06 3.63687035006489e-06 60
"chr8" 56410001 56411000 "*" 0 0 98.8235294117647
"chr8" 56436001 56437000 "*" 0 0 75.9259259259259
"chr8" 56498001 56499000 "*" 1.13140401492018e-08 1.87801380787728e-08 100
"chr8" 56569001 56570000 "*" 1.54630752646767e-11 3.85079502648228e-11 -100
"chr8" 56642001 56643000 "*" 5.794809077031e-12 1.52911192757929e-11 100
"chr8" 56749001 56750000 "*" 5.6362137179633e-11 1.29963270300065e-10 100
"chr8" 56789001 56790000 "*" 1.0999424593372e-08 1.83470343859006e-08 60
"chr8" 56793001 56794000 "*" 0 0 -100
"chr8" 56930001 56931000 "*" 1.67299207820548e-08 2.71377429441055e-08 -100
"chr8" 56973001 56974000 "*" 6.87628248341454e-07 8.92892153029571e-07
-69.6969696969697
"chr8" 56975001 56976000 "*" 2.00227675550835e-08 3.20459176617961e-08 -100
"chr8" 56987001 56988000 "*" 1.80770864943725e-09 3.37846860013928e-09
-81.4516129032258
"chr8" 57054001 57055000 "*" 0 0 86.8421052631579
"chr8" 57124001 57125000 "*" 4.85138040851041e-10 9.84377070615041e-10 60
"chr8" 57214001 57215000 "*" 1.52466927971773e-12 4.33322475981408e-12 100
"chr8" 57232001 57233000 "*" 0 0 -58.9761248852158
"chr8" 57926001 57927000 "*" 1.15339349182619e-09 2.21004254568532e-09
-92.8571428571429
"chr8" 58115001 58116000 "*" 1.29037891483108e-11 3.24582280286866e-11 -100
"chr8" 58133001 58134000 "*" 3.66151553521377e-13 1.12588189950301e-12 75

"chr8" 58262001 58263000 "*" 0 0 -100
"chr8" 58332001 58333000 "*" 4.06193938840005e-06 4.71666114329722e-06 67.2131147540984
"chr8" 58650001 58651000 "*" 3.6700841921089e-08 5.65530226072258e-08 -100
"chr8" 58788001 58789000 "*" 2.43005615629954e-09 4.45014222280278e-09 -100
"chr8" 59323001 59324000 "*" 0 0 -64.5083932853717
"chr8" 59507001 59508000 "*" 5.04263297784746e-13 1.52078941532818e-12 -100
"chr8" 59689001 59690000 "*" 8.54871728961371e-15 3.20277157428094e-14 -63.953488372093
"chr8" 59697001 59698000 "*" 2.4535928844216e-14 8.69144396197119e-14 -100
"chr8" 60005001 60006000 "*" 1.17461596005342e-13 3.8279067575877e-13 100
"chr8" 60017001 60018000 "*" 4.71134242729931e-12 1.257615036806e-11 -100
"chr8" 60139001 60140000 "*" 4.86753970463383e-11 1.13145908161915e-10 100
"chr8" 60436001 60437000 "*" 1.39779633423487e-07 1.98463037958589e-07 -100
"chr8" 60514001 60515000 "*" 2.88710388929303e-10 6.03399264731139e-10 100
"chr8" 60578001 60579000 "*" 1.93755012034558e-11 4.76380503082282e-11 -76.0869565217391
"chr8" 60821001 60822000 "*" 0 0 100
"chr8" 61193001 61194000 "*" 0 0 59.9720853858785
"chr8" 61205001 61206000 "*" 9.80763359414993e-10 1.8980032424715e-09 -100
"chr8" 61311001 61312000 "*" 1.80989024236577e-05 1.90667975357288e-05 68.75
"chr8" 61823001 61824000 "*" 8.06021915877864e-14 2.68365162509737e-13 -57.6923076923077
"chr8" 61926001 61927000 "*" 1.51323398256409e-13 4.86995056237989e-13 100
"chr8" 62672001 62673000 "*" 1.11022302462516e-16 5.03662826618488e-16 -100
"chr8" 63055001 63056000 "*" 0 0 54.4624223526234
"chr8" 63160001 63161000 "*" 0 0 91.2596401028278
"chr8" 63161001 63162000 "*" 0 0 78.2806172182449
"chr8" 63162001 63163000 "*" 0 0 96.875
"chr8" 63522001 63523000 "*" 2.44249065417534e-15 9.75180348030994e-15 -100
"chr8" 63603001 63604000 "*" 1.8893420161703e-10 4.06754022981325e-10 72.972972972973
"chr8" 63662001 63663000 "*" 1.11022302462516e-16 5.03662826618488e-16 -100
"chr8" 63843001 63844000 "*" 1.87405646556726e-13 5.97285292681429e-13 -100
"chr8" 64674001 64675000 "*" 2.08814465718632e-09 3.86232567960463e-09 100
"chr8" 65255001 65256000 "*" 0 0 100
"chr8" 65285001 65286000 "*" 2.57009968862576e-11 6.22308980701977e-11 -87.5
"chr8" 65362001 65363000 "*" 2.69018141096922e-12 7.41487347763769e-12 100
"chr8" 65492001 65493000 "*" 0 0 100
"chr8" 65734001 65735000 "*" 1.13140401492018e-08 1.87801380787728e-08 -100
"chr8" 65766001 65767000 "*" 5.51780843238703e-14 1.87243792572872e-13 100
"chr8" 65927001 65928000 "*" 0 0 -100
"chr8" 66057001 66058000 "*" 2.00683913931243e-12 5.61744530708922e-12 -75
"chr8" 66157001 66158000 "*" 0 0 100
"chr8" 66367001 66368000 "*" 5.51780843238703e-14 1.87243792572872e-13 -100
"chr8" 67066001 67067000 "*" 5.10702591327572e-15 1.96271840288234e-14 -100
"chr8" 67149001 67150000 "*" 4.6296300126869e-14 1.59104322469884e-13 -51.7241379310345
"chr8" 67188001 67189000 "*" 3.88424259423203e-09 6.93843388289179e-09 -60
"chr8" 67191001 67192000 "*" 4.32087698953865e-12 1.15935290479641e-11 65.1162790697674
"chr8" 67241001 67242000 "*" 9.5812247025151e-14 3.15667542866807e-13 -100

"chr8" 67372001 67373000 "*" 2.12052597703405e-13 6.70432330365014e-13
58.3333333333333
"chr8" 67424001 67425000 "*" 6.66133814775094e-16 2.81595744474255e-15
69.2307692307692
"chr8" 67580001 67581000 "*" 0 0 -100
"chr8" 67592001 67593000 "*" 2.1094237467878e-15 8.47434540879246e-15 -100
"chr8" 67931001 67932000 "*" 0 0 92.7710843373494
"chr8" 67940001 67941000 "*" 0 0 91.2353923205342
"chr8" 67941001 67942000 "*" 6.75805410077501e-07 8.7840165536282e-07
66.2337662337662
"chr8" 68661001 68662000 "*" 9.08108033215171e-11 2.03407753381119e-10 100
"chr8" 68871001 68872000 "*" 3.6700841921089e-08 5.65530226072258e-08 -100
"chr8" 69104001 69105000 "*" 0 0 -59.4936708860759
"chr8" 69225001 69226000 "*" 0 0 100
"chr8" 69562001 69563000 "*" 9.65729496371637e-10 1.87255912493232e-09 -100
"chr8" 69668001 69669000 "*" 3.68371999570627e-13 1.13149976091305e-12 -100
"chr8" 69672001 69673000 "*" 9.55452211592878e-06 1.05063458854353e-05
-54.1666666666667
"chr8" 69722001 69723000 "*" 0 0 -100
"chr8" 69723001 69724000 "*" 2.06569428229386e-10 4.42595614308608e-10
79.7385620915033
"chr8" 70166001 70167000 "*" 1.06998077065157e-10 2.37773350307407e-10
-88.9908256880734
"chr8" 70187001 70188000 "*" 2.70541700153615e-10 5.68690552277906e-10
66.6666666666667
"chr8" 70281001 70282000 "*" 1.50768286744096e-13 4.85452096185033e-13
-66.6666666666667
"chr8" 70404001 70405000 "*" 5.55111512312578e-15 2.12516350804551e-14
53.2003904615814
"chr8" 70485001 70486000 "*" 0 0 -100
"chr8" 70488001 70489000 "*" 5.01025332333427e-10 1.01358031135889e-09 100
"chr8" 70632001 70633000 "*" 0 0 100
"chr8" 70690001 70691000 "*" 0 0 -100
"chr8" 70714001 70715000 "*" 1.83952852950142e-12 5.17499701329218e-12 100
"chr8" 70800001 70801000 "*" 9.65729496371637e-10 1.87255912493232e-09 100
"chr8" 70855001 70856000 "*" 0 0 72.6581158779301
"chr8" 70970001 70971000 "*" 0 0 100
"chr8" 71119001 71120000 "*" 1.6767565095277e-06 2.0626065508796e-06
-58.3333333333333
"chr8" 71490001 71491000 "*" 1.65900410042941e-06 2.04236557510993e-06
-66.6666666666667
"chr8" 71660001 71661000 "*" 2.15125472990962e-10 4.58126659943874e-10 100
"chr8" 71869001 71870000 "*" 1.29037891483108e-11 3.24582280286866e-11 100
"chr8" 71957001 71958000 "*" 5.55111512312578e-16 2.36485094870365e-15 -100
"chr8" 72024001 72025000 "*" 0 0 100
"chr8" 72179001 72180000 "*" 8.7349629751543e-09 1.47505871898511e-08 -100
"chr8" 72265001 72266000 "*" 8.63147219054561e-06 9.54685158036787e-06
-62.0689655172414
"chr8" 72273001 72274000 "*" 0 0 84.5238095238095
"chr8" 72274001 72275000 "*" 0 0 75.2895752895753
"chr8" 72739001 72740000 "*" 2.02060590481778e-13 6.40127405465654e-13 -100
"chr8" 72758001 72759000 "*" 2.22044604925031e-16 9.81641919380259e-16 100

"chr8" 73067001 73068000 "*" 1.34700062215387e-08 2.2161880946568e-08 65.3225806451613
"chr8" 73226001 73227000 "*" 1.13140401492018e-08 1.87801380787728e-08 -100
"chr8" 73448001 73449000 "*" 0 0 63.2923109708182
"chr8" 73597001 73598000 "*" 1.05639579316419e-05 1.15310512944319e-05 -53.448275862069
"chr8" 73841001 73842000 "*" 7.09271530396904e-11 1.61313716869008e-10 82.8571428571429
"chr8" 73849001 73850000 "*" 2.528045117689e-08 3.99493576342161e-08 55.3571428571429
"chr8" 73890001 73891000 "*" 4.67097782674841e-09 8.22452405271328e-09 72.7272727272727
"chr8" 74268001 74269000 "*" 0 0 -78.2608695652174
"chr8" 74352001 74353000 "*" 1.4432899320127e-15 5.90750815956055e-15 100
"chr8" 74364001 74365000 "*" 6.59550958292954e-09 1.13456194382773e-08 100
"chr8" 75002001 75003000 "*" 8.43347613965761e-12 2.17859001990012e-11 100
"chr8" 75224001 75225000 "*" 0 0 100
"chr8" 75390001 75391000 "*" 8.16636758216305e-11 1.84379855454435e-10 64.7058823529412
"chr8" 76090001 76091000 "*" 1.11022302462516e-16 5.03662826618488e-16 73.3333333333333
"chr8" 76273001 76274000 "*" 3.88022947106492e-13 1.1867462770515e-12 100
"chr8" 76320001 76321000 "*" 0 0 -53.8461538461538
"chr8" 76632001 76633000 "*" 8.56891819900341e-06 9.48156170078414e-06 52.3809523809524
"chr8" 76761001 76762000 "*" 0 0 83.3333333333333
"chr8" 77082001 77083000 "*" 0 0 -50.6493506493507
"chr8" 77130001 77131000 "*" 3.94129173741931e-14 1.36161086061753e-13 66.6666666666667
"chr8" 77560001 77561000 "*" 4.14335232790108e-13 1.26153063645578e-12 100
"chr8" 79428001 79429000 "*" 0 0 50.9767709296563
"chr8" 80105001 80106000 "*" 8.80406858527749e-14 2.91456141063452e-13 -100
"chr8" 80303001 80304000 "*" 3.33066907387547e-16 1.4495649018245e-15 100
"chr8" 80525001 80526000 "*" 0 0 88.5283893395133
"chr8" 80695001 80696000 "*" 0 0 -100
"chr8" 80946001 80947000 "*" 3.04324343503026e-11 7.26599935673121e-11 -100
"chr8" 81053001 81054000 "*" 4.99900121297969e-12 1.32921056339624e-11 -100
"chr8" 81109001 81110000 "*" 9.32032229172819e-13 2.7181920310254e-12 100
"chr8" 81113001 81114000 "*" 1.02868835671899e-09 1.98614052030224e-09 71.2341197822142
"chr8" 81114001 81115000 "*" 2.2360348705952e-07 3.09639973195055e-07 -59.375
"chr8" 81122001 81123000 "*" 2.68450373042128e-10 5.64465213127486e-10 100
"chr8" 81260001 81261000 "*" 5.62883073484954e-13 1.68905058935478e-12 100
"chr8" 81263001 81264000 "*" 1.5277158427196e-09 2.87642564232045e-09 100
"chr8" 81279001 81280000 "*" 0 0 -100
"chr8" 81318001 81319000 "*" 1.20281562487889e-12 3.46585342181251e-12 100
"chr8" 81397001 81398000 "*" 0 0 82.2916666666667
"chr8" 81398001 81399000 "*" 0 0 93.7349397590361
"chr8" 81399001 81400000 "*" 0 0 54.873399715505
"chr8" 81483001 81484000 "*" 6.92287338566189e-11 1.57605630124247e-10 -100
"chr8" 81527001 81528000 "*" 0 0 -100
"chr8" 81840001 81841000 "*" 8.01666433236647e-09 1.36392368970109e-08 100

"chr8" 81870001 81871000 "*" 8.01666433236647e-09 1.36392368970109e-08 100
"chr8" 82214001 82215000 "*" 2.88779000712225e-11 6.92935809466068e-11 100
"chr8" 82470001 82471000 "*" 0 0 56.043956043956
"chr8" 83855001 83856000 "*" 5.20861132002892e-12 1.38212476721179e-11
57.2261072261072
"chr8" 84643001 84644000 "*" 0 0 72.8473482556967
"chr8" 84686001 84687000 "*" 6.37490060739765e-13 1.89496793587877e-12 100
"chr8" 84838001 84839000 "*" 9.08108033215171e-11 2.03407753381119e-10 -100
"chr8" 85533001 85534000 "*" 6.27276008913213e-14 2.11648419515057e-13
-54.0909090909091
"chr8" 85819001 85820000 "*" 1.39779633423487e-07 1.98463037958589e-07 100
"chr8" 85855001 85856000 "*" 4.99900121297969e-12 1.32921056339624e-11 100
"chr8" 85895001 85896000 "*" 7.0006535457523e-08 1.03695832512054e-07 -100
"chr8" 86157001 86158000 "*" 0 0 -70.3703703703704
"chr8" 86375001 86376000 "*" 2.22044604925031e-16 9.81641919380259e-16 -60
"chr8" 86436001 86437000 "*" 3.21026538685487e-11 7.64813810727539e-11
-51.6891891891892
"chr8" 87005001 87006000 "*" 2.05391259555654e-14 7.35435952190685e-14 100
"chr8" 87510001 87511000 "*" 2.73625566649116e-12 7.52483363579137e-12 -100
"chr8" 87517001 87518000 "*" 4.02167188440217e-12 1.08266700497926e-11 100
"chr8" 87670001 87671000 "*" 0 0 84.0579710144928
"chr8" 87671001 87672000 "*" 1.15614329221359e-09 2.21475983570656e-09
-74.5098039215686
"chr8" 88188001 88189000 "*" 6.77335965093562e-12 1.76897011246462e-11 100
"chr8" 88196001 88197000 "*" 2.61522359323862e-10 5.51301646507333e-10
58.6206896551724
"chr8" 88984001 88985000 "*" 0 0 100
"chr8" 90062001 90063000 "*" 3.31690230837012e-12 9.03347040781321e-12 100
"chr8" 90071001 90072000 "*" 4.88498130835069e-15 1.8824139250584e-14 -100
"chr8" 91095001 91096000 "*" 0 0 100
"chr8" 91739001 91740000 "*" 1.52466927971773e-12 4.33322475981408e-12 100
"chr8" 92540001 92541000 "*" 1.25875536893805e-06 1.5765764865108e-06 60
"chr8" 93157001 93158000 "*" 1.66533453693773e-15 6.7629186866784e-15 -100
"chr8" 93243001 93244000 "*" 0 0 -79.7297297297297
"chr8" 93293001 93294000 "*" 2.68450373042128e-10 5.64465213127486e-10 100
"chr8" 93848001 93849000 "*" 1.22124532708767e-15 5.03921984217778e-15 100
"chr8" 94238001 94239000 "*" 0 0 100
"chr8" 94273001 94274000 "*" 8.7349629751543e-09 1.47505871898511e-08 100
"chr8" 94401001 94402000 "*" 1.37425132162861e-07 1.95826814576532e-07
61.5384615384615
"chr8" 94781001 94782000 "*" 1.58636145775048e-06 1.95929423010922e-06
57.7777777777778
"chr8" 94967001 94968000 "*" 1.67299207820548e-08 2.71377429441055e-08 -100
"chr8" 95045001 95046000 "*" 1.13140401492018e-08 1.87801380787728e-08 -100
"chr8" 95563001 95564000 "*" 1.16213363043904e-07 1.6712548668921e-07
-57.1428571428571
"chr8" 95653001 95654000 "*" 0 0 57.2809261004173
"chr8" 95680001 95681000 "*" 3.40125705378114e-10 7.03784073980162e-10 100
"chr8" 95906001 95907000 "*" 8.01666433236647e-09 1.36392368970109e-08 -100
"chr8" 95918001 95919000 "*" 1.83952852950142e-12 5.17499701329218e-12 -100
"chr8" 96232001 96233000 "*" 3.97459842815806e-13 1.21369463636147e-12
-58.8943623426382

"chr8" 96423001 96424000 "*" 1.17794662912729e-13 3.83653245040640e-13 100
"chr8" 96690001 96691000 "*" 1.22124532708767e-15 5.03921984217778e-15 -100
"chr8" 96764001 96765000 "*" 3.56775498033812e-10 7.35898629809829e-10 100
"chr8" 97128001 97129000 "*" 6.83719301175501e-08 1.01840744031014e-07
-66.6666666666667
"chr8" 97129001 97130000 "*" 0 0 -100
"chr8" 97193001 97194000 "*" 4.01313771103418e-09 7.11882087237587e-09 100
"chr8" 97275001 97276000 "*" 1.11022302462516e-16 5.03662826618488e-16 100
"chr8" 97329001 97330000 "*" 1.29037891483108e-11 3.24582280286866e-11 -100
"chr8" 97345001 97346000 "*" 0 0 100
"chr8" 97362001 97363000 "*" 4.86753970463383e-11 1.13145908161915e-10 -100
"chr8" 97480001 97481000 "*" 1.38590361409285e-10 3.02706178892116e-10 100
"chr8" 97483001 97484000 "*" 7.67056418382595e-11 1.7359448580318e-10 100
"chr8" 97490001 97491000 "*" 5.72265790788062e-05 5.57582226055108e-05
56.6666666666667
"chr8" 98342001 98343000 "*" 7.67056418382595e-11 1.7359448580318e-10 -100
"chr8" 98459001 98460000 "*" 3.63445940010365e-11 8.58565209111204e-11 -100
"chr8" 98599001 98600000 "*" 5.6362137179633e-11 1.29963270300065e-10 100
"chr8" 98610001 98611000 "*" 0 0 -73.0769230769231
"chr8" 98728001 98729000 "*" 0 0 -100
"chr8" 98772001 98773000 "*" 3.55833300347275e-06 4.16960480709109e-06
-69.7674418604651
"chr8" 98787001 98788000 "*" 2.79732037844482e-06 3.33126482101058e-06
63.8709677419355
"chr8" 98788001 98789000 "*" 0 0 56.1518609231293
"chr8" 98833001 98834000 "*" 1.92223400530267e-07 2.68476974496499e-07
-52.5423728813559
"chr8" 98880001 98881000 "*" 2.68756060073372e-07 3.68470725862618e-07
57.2916666666667
"chr8" 98933001 98934000 "*" 9.65338919911574e-13 2.80678910656113e-12 100
"chr8" 98963001 98964000 "*" 7.01621548637732e-08 1.03896816756352e-07
75.5102040816327
"chr8" 99014001 99015000 "*" 1.11022302462516e-16 5.03662826618488e-16 100
"chr8" 99017001 99018000 "*" 0 0 100
"chr8" 99077001 99078000 "*" 0 0 74.1575757575758
"chr8" 99188001 99189000 "*" 2.60612787996717e-05 2.67906916777296e-05
65.1162790697674
"chr8" 99234001 99235000 "*" 3.95353005888666e-09 7.03928529283733e-09 100
"chr8" 99306001 99307000 "*" 0 0 -73.5371179039301
"chr8" 99318001 99319000 "*" 3.744609147871e-06 4.3730118294436e-06 65.625
"chr8" 99950001 99951000 "*" 1.26565424807268e-14 4.63923203146481e-14 100
"chr8" 100881001 100882000 "*" 0 0 92.5
"chr8" 101170001 101171000 "*" 0 0 -88.2758620689655
"chr8" 101243001 101244000 "*" 9.06201294559317e-07 1.15739767942151e-06
-60.377358490566
"chr8" 101615001 101616000 "*" 4.01313771103418e-09 7.11882087237587e-09 100
"chr8" 101817001 101818000 "*" 1.75082170983387e-13 5.6014942024636e-13 100
"chr8" 101859001 101860000 "*" 0 0 80.9917355371901
"chr8" 101860001 101861000 "*" 8.40916225541832e-12 2.17432953970905e-11
90.9677419354839
"chr8" 101919001 101920000 "*" 1.24251666966391e-06 1.557547589828e-06
-57.1428571428571

"chr8" 102062001 102063000 "*" 3.76365605347928e-14 1.3043487970679e-13 100
"chr8" 102076001 102077000 "*" 6.77335965093562e-12 1.76897011246462e-11 -100
"chr8" 102093001 102094000 "*" 0 0 -69.5286195286195
"chr8" 102138001 102139000 "*" 0 0 -61.6352201257862
"chr8" 102139001 102140000 "*" 5.55111512312578e-16 2.36485094870365e-15 100
"chr8" 102145001 102146000 "*" 1.07292930096037e-09 2.06729933000587e-09 -68.75
"chr8" 102340001 102341000 "*" 5.794809077031e-12 1.52911192757929e-11 -100
"chr8" 102373001 102374000 "*" 5.25833514877228e-05 5.15382873150132e-05
54.7169811320755
"chr8" 102526001 102527000 "*" 3.85511955691697e-10 7.92486401489696e-10
81.3559322033898
"chr8" 102586001 102587000 "*" 1.07882591748876e-11 2.74553537455803e-11 -100
"chr8" 102768001 102769000 "*" 3.95861121660346e-12 1.0684496262032e-11 -100
"chr8" 103019001 103020000 "*" 9.65729496371637e-10 1.87255912493232e-09 -100
"chr8" 103115001 103116000 "*" 2.91028312560115e-11 6.97590507139978e-11 100
"chr8" 103136001 103137000 "*" 0 0 57.5784487862641
"chr8" 103177001 103178000 "*" 7.0006535457523e-08 1.03695832512054e-07 100
"chr8" 103209001 103210000 "*" 7.67056418382595e-11 1.7359448580318e-10 100
"chr8" 103377001 103378000 "*" 0 0 100
"chr8" 103475001 103476000 "*" 7.21644966006352e-15 2.72345776413425e-14 100
"chr8" 103572001 103573000 "*" 1.98365768255826e-11 4.85612402473442e-11 -100
"chr8" 103603001 103604000 "*" 0.000241617109017045 0.000212792315955059
-51.1111111111111
"chr8" 103669001 103670000 "*" 8.43036751518866e-12 2.17859001990012e-11 -80
"chr8" 103740001 103741000 "*" 5.09592368302947e-14 1.73761636562837e-13 100
"chr8" 103750001 103751000 "*" 0 0 -53.9378780828292
"chr8" 103759001 103760000 "*" 3.33066907387547e-16 1.4495649018245e-15 100
"chr8" 103763001 103764000 "*" 3.92115250658076e-06 4.56378095612863e-06
69.2307692307692
"chr8" 103766001 103767000 "*" 1.28656442055508e-07 1.83972926017948e-07
-50.8196721311475
"chr8" 103767001 103768000 "*" 5.43117762319412e-06 6.19387126034938e-06
84.6153846153846
"chr8" 103794001 103795000 "*" 2.33028818463765e-10 4.93719283454501e-10 100
"chr8" 103807001 103808000 "*" 1.77580172788794e-11 4.39201393155143e-11 92
"chr8" 103942001 103943000 "*" 2.15125472990962e-10 4.58126659943874e-10 100
"chr8" 104026001 104027000 "*" 9.5812247025151e-14 3.15667542866807e-13 100
"chr8" 104134001 104135000 "*" 7.54951656745106e-15 2.84375115626978e-14
60.6060606060606
"chr8" 104164001 104165000 "*" 1.4432899320127e-15 5.90750815956055e-15 100
"chr8" 104310001 104311000 "*" 2.98793212394344e-11 7.15442053443855e-11
65.6287425149701
"chr8" 105225001 105226000 "*" 8.01666433236647e-09 1.36392368970109e-08 -100
"chr8" 105388001 105389000 "*" 1.4432899320127e-15 5.90750815956055e-15
56.9444444444444
"chr8" 105503001 105504000 "*" 3.73034936274053e-14 1.29420415488591e-13 100
"chr8" 105600001 105601000 "*" 0 0 100
"chr8" 105713001 105714000 "*" 5.295763827462e-14 1.80214784640623e-13 -100
"chr8" 105758001 105759000 "*" 1.33946298497278e-09 2.54875990216383e-09 -93.75
"chr8" 106620001 106621000 "*" 6.59550958292954e-09 1.13456194382773e-08 -100
"chr8" 107306001 107307000 "*" 1.13361542375401e-11 2.87683533461181e-11
54.8387096774194

"chr8" 107379001 107380000 "*" 5.6621374255883e-15 2.16468580166064e-14 -100
"chr8" 107783001 107784000 "*" 4.36241330459275e-06 5.0406393836561e-06
-73.3333333333333
"chr8" 108499001 108500000 "*" 5.48638023900594e-10 1.10374357336421e-09 -100
"chr8" 108913001 108914000 "*" 2.88779000712225e-11 6.92935809466068e-11 -100
"chr8" 109067001 109068000 "*" 3.40125705378114e-10 7.03784073980162e-10 100
"chr8" 109152001 109153000 "*" 2.89801516117905e-12 7.94270405097209e-12
66.6666666666667
"chr8" 109273001 109274000 "*" 4.78841410966879e-11 1.11468187156588e-10 -100
"chr8" 109781001 109782000 "*" 6.4152538836737e-10 1.27477697728417e-09 -100
"chr8" 110086001 110087000 "*" 3.7274627828765e-12 1.00985250136599e-11 100
"chr8" 110704001 110705000 "*" 0 0 92.3076923076923
"chr8" 111088001 111089000 "*" 1.93720595120794e-11 4.76309924836636e-11 100
"chr8" 111842001 111843000 "*" 0 0 100
"chr8" 112297001 112298000 "*" 8.01666433236647e-09 1.36392368970109e-08 100
"chr8" 112770001 112771000 "*" 5.01728540380775e-07 6.6304977462997e-07
70.7692307692308
"chr8" 113202001 113203000 "*" 6.66133814775094e-16 2.81595744474255e-15 100
"chr8" 114446001 114447000 "*" 1.30340183090993e-13 4.22662705118657e-13 -100
"chr8" 114447001 114448000 "*" 1.11022302462516e-16 5.03662826618488e-16
90.1601830663616
"chr8" 114449001 114450000 "*" 0 0 93.5897435897436
"chr8" 114663001 114664000 "*" 1.83186799063151e-14 6.59696304569048e-14 100
"chr8" 114802001 114803000 "*" 1.11022302462516e-16 5.03662826618488e-16
66.6666666666667
"chr8" 115598001 115599000 "*" 0 0 73.8741875580316
"chr8" 115687001 115688000 "*" 1.48087875295744e-10 3.22075573380618e-10 100
"chr8" 115838001 115839000 "*" 6.4152538836737e-10 1.27477697728417e-09 100
"chr8" 116560001 116561000 "*" 2.35041208718201e-09 4.32060846896582e-09 60
"chr8" 116720001 116721000 "*" 1.36604061395929e-11 3.42333952202875e-11 100
"chr8" 116773001 116774000 "*" 2.15125472990962e-10 4.58126659943874e-10 -100
"chr8" 117128001 117129000 "*" 1.61237689866311e-12 4.56615813671789e-12 100
"chr8" 117483001 117484000 "*" 6.59550958292954e-09 1.13456194382773e-08 100
"chr8" 117589001 117590000 "*" 3.28514992986584e-13 1.01516837897203e-12
55.8823529411765
"chr8" 117631001 117632000 "*" 1.65095790604086e-05 1.75056944925865e-05
61.5384615384615
"chr8" 117643001 117644000 "*" 5.6621374255883e-15 2.16468580166064e-14 100
"chr8" 118203001 118204000 "*" 4.69074556974647e-09 8.25845934437494e-09
79.7101449275362
"chr8" 118295001 118296000 "*" 2.88710388929303e-10 6.03399264731139e-10 -100
"chr8" 118991001 118992000 "*" 3.6700841921089e-08 5.65530226072258e-08 -100
"chr8" 119123001 119124000 "*" 2.29816166097407e-13 7.23030986737613e-13
85.8333333333333
"chr8" 119175001 119176000 "*" 1.40665257220007e-13 4.54219470790239e-13
74.3589743589744
"chr8" 119383001 119384000 "*" 6.92287338566189e-11 1.57605630124247e-10 100
"chr8" 119649001 119650000 "*" 3.99791311167519e-13 1.22027792394232e-12 -100
"chr8" 119849001 119850000 "*" 3.13902409350142e-05 3.18712470329053e-05 -60
"chr8" 120188001 120189000 "*" 7.0006535457523e-08 1.03695832512054e-07 -100
"chr8" 120202001 120203000 "*" 6.77335965093562e-12 1.76897011246462e-11 100
"chr8" 120623001 120624000 "*" 1.24161251305877e-05 1.3408176739013e-05

-60.6837606837607
"chr8" 120875001 120876000 "*" 0.000214547458163739 0.00019064542382489
50.9433962264151
"chr8" 120885001 120886000 "*" 0 0 -100
"chr8" 120956001 120957000 "*" 1.96509475358653e-13 6.23868565133654e-13 100
"chr8" 120983001 120984000 "*" 1.11022302462516e-16 5.03662826618488e-16
60.2150537634409
"chr8" 121136001 121137000 "*" 7.18900861063076e-09 1.23150049017738e-08
-50.8474576271186
"chr8" 121926001 121927000 "*" 1.76327532319664e-06 2.16297328838588e-06
-83.3333333333333
"chr8" 121982001 121983000 "*" 2.93914359418279e-05 2.99757412716435e-05
59.4594594594595
"chr8" 122652001 122653000 "*" 0 0 88.8888888888889
"chr8" 122655001 122656000 "*" 8.64062160710333e-07 1.10645457436487e-06
72.7272727272727
"chr8" 123001001 123002000 "*" 0 0 100
"chr8" 123570001 123571000 "*" 0 0 100
"chr8" 123667001 123668000 "*" 5.48638023900594e-10 1.10374357336421e-09 100
"chr8" 123799001 123800000 "*" 4.32209823486573e-12 1.15935290479641e-11 -100
"chr8" 123809001 123810000 "*" 2.22044604925031e-16 9.81641919380259e-16
-80.5970149253731
"chr8" 123814001 123815000 "*" 0 0 65.5172413793103
"chr8" 123821001 123822000 "*" 8.01666433236647e-09 1.36392368970109e-08 -100
"chr8" 123950001 123951000 "*" 7.34485790570005e-05 7.03153538894558e-05
-54.1666666666667
"chr8" 124055001 124056000 "*" 2.62900812231237e-13 8.21294938531841e-13 100
"chr8" 124171001 124172000 "*" 0 0 83.3333333333333
"chr8" 124172001 124173000 "*" 9.67004254448511e-13 2.81143515281558e-12
-59.3582887700535
"chr8" 124372001 124373000 "*" 4.73234496034536e-09 8.30627300264826e-09 100
"chr8" 124405001 124406000 "*" 0 0 100
"chr8" 124525001 124526000 "*" 6.35821299288519e-06 7.17514911154058e-06
-66.6666666666667
"chr8" 124537001 124538000 "*" 2.39204430818596e-07 3.30087300183833e-07
55.0505050505051
"chr8" 124589001 124590000 "*" 4.29989377437323e-13 1.30609366528895e-12 -62.5
"chr8" 124601001 124602000 "*" 5.80137120120838e-06 6.5882135290028e-06 -70
"chr8" 124630001 124631000 "*" 1.56157221653519e-06 1.93054397956841e-06
-78.125
"chr8" 124652001 124653000 "*" 2.79987144580218e-12 7.68634726811898e-12 -100
"chr8" 124743001 124744000 "*" 2.22044604925031e-16 9.81641919380259e-16 -100
"chr8" 124845001 124846000 "*" 0.000525299954949099 0.000436849389802675
54.5454545454545
"chr8" 124870001 124871000 "*" 3.88486243174668e-10 7.98305411814578e-10
88.8888888888889
"chr8" 125011001 125012000 "*" 3.9190872769268e-14 1.35416418933905e-13 -100
"chr8" 125191001 125192000 "*" 2.08814465718632e-09 3.86232567960463e-09 -100
"chr8" 125214001 125215000 "*" 2.19853864003028e-08 3.50003158916803e-08
66.6666666666667
"chr8" 125266001 125267000 "*" 3.21305981820785e-10 6.67824922105025e-10
-56.9230769230769

"chr8" 125438001 125439000 "*" 1.01918473660589e-13 3.34635318978813e-13
74.5454545454545
"chr8" 125627001 125628000 "*" 3.46500605985511e-13 1.06777978272295e-12 100
"chr8" 125639001 125640000 "*" 3.34913319299801e-08 5.20876536205837e-08
-83.3333333333333
"chr8" 125640001 125641000 "*" 1.50623957750895e-12 4.28756717183481e-12 -100
"chr8" 125644001 125645000 "*" 1.89581683684992e-12 5.32311367606535e-12 100
"chr8" 125657001 125658000 "*" 1.41343951820527e-06 1.75856430329587e-06
-57.5757575757576
"chr8" 125677001 125678000 "*" 1.35447209004269e-14 4.94674708073053e-14 100
"chr8" 125839001 125840000 "*" 1.07882591748876e-11 2.74553537455803e-11 100
"chr8" 125918001 125919000 "*" 4.70028149734958e-08 7.15458292114991e-08
83.0769230769231
"chr8" 125922001 125923000 "*" 0 0 -100
"chr8" 126031001 126032000 "*" 6.28437806238225e-08 9.40583799191989e-08
88.2352941176471
"chr8" 126388001 126389000 "*" 4.87387907810444e-14 1.6691369115096e-13 74
"chr8" 126401001 126402000 "*" 6.83719338923083e-08 1.01840744031014e-07
-66.6666666666667
"chr8" 126525001 126526000 "*" 1.87405646556726e-13 5.97285292681429e-13 -100
"chr8" 126540001 126541000 "*" 1.39779633423487e-07 1.98463037958589e-07 100
"chr8" 127115001 127116000 "*" 9.80763359414993e-10 1.8980032424715e-09 100
"chr8" 127446001 127447000 "*" 3.04324343503026e-11 7.26599935673121e-11 -100
"chr8" 127534001 127535000 "*" 1.28826294076845e-09 2.45536878148726e-09
-84.2105263157895
"chr8" 127570001 127571000 "*" 0 0 52.3327626656521
"chr8" 127576001 127577000 "*" 7.67415337733501e-05 7.32777372339046e-05
-57.1428571428571
"chr8" 127598001 127599000 "*" 0 0 -89.662027833002
"chr8" 127625001 127626000 "*" 9.63829016598083e-12 2.46993429847588e-11 100
"chr8" 127630001 127631000 "*" 6.39165064653113e-07 8.33531059852563e-07
90.9090909090909
"chr8" 127631001 127632000 "*" 3.88428432307464e-07 5.20823182914617e-07
-80.4878048780488
"chr8" 127642001 127643000 "*" 5.24543741775574e-11 1.21429243775989e-10
-87.4172185430464
"chr8" 127652001 127653000 "*" 2.74320330673206e-07 3.75630170113502e-07
69.5652173913043
"chr8" 127666001 127667000 "*" 1.08850117808146e-09 2.09531821318215e-09
70.1754385964912
"chr8" 127703001 127704000 "*" 4.01313771103418e-09 7.11882087237587e-09 -100
"chr8" 127755001 127756000 "*" 1.29037891483108e-11 3.24582280286866e-11 100
"chr8" 127756001 127757000 "*" 1.13018483460792e-11 2.86865308025083e-11
58.2857142857143
"chr8" 127837001 127838000 "*" 1.27867878507715e-08 2.10989834353829e-08
-74.7870528109029
"chr8" 127871001 127872000 "*" 2.33735711896443e-08 3.70990984468011e-08 -93.75
"chr8" 127885001 127886000 "*" 5.05416592422314e-05 4.96812965620486e-05
52.8735632183908
"chr8" 127889001 127890000 "*" 0 0 -68.1784507107469
"chr8" 127915001 127916000 "*" 0.000726374117344109 0.000590081342527627
-52.1739130434783

"chr8" 127960001 127961000 "*" 3.59356988610671e-12 9.7595569087438e-12 -75.2577319587629
"chr8" 128029001 128030000 "*" 4.90718576884319e-14 1.6794399166207e-13 100
"chr8" 128066001 128067000 "*" 1.53838335403833e-08 2.51385720313054e-08 -84.9315068493151
"chr8" 128172001 128173000 "*" 6.66133814775094e-15 2.52791665956799e-14 -100
"chr8" 128193001 128194000 "*" 1.37828859436695e-10 3.01445544267661e-10 100
"chr8" 128196001 128197000 "*" 2.703514776492e-07 3.70469285641791e-07 61.0169491525424
"chr8" 128231001 128232000 "*" 1.32782673745169e-13 4.30248554172537e-13 -90.8382066276803
"chr8" 128278001 128279000 "*" 0 0 -77.2585669781931
"chr8" 128305001 128306000 "*" 5.39738487868036e-05 5.27974373872329e-05 51.5625
"chr8" 128307001 128308000 "*" 1.29010135907492e-11 3.24582280286866e-11 -90.6040268456376
"chr8" 128344001 128345000 "*" 4.55918891528739e-09 8.03855086785938e-09 -70.2928870292887
"chr8" 128368001 128369000 "*" 3.19089232192482e-05 3.2363861688848e-05 57.6086956521739
"chr8" 128405001 128406000 "*" 1.32762167925904e-07 1.8948979748136e-07 67.816091954023
"chr8" 128587001 128588000 "*" 0 0 -100
"chr8" 128691001 128692000 "*" 2.95708500408498e-05 3.01454511421846e-05 -50.2127659574468
"chr8" 128750001 128751000 "*" 0 0 -82.1268417680974
"chr8" 128777001 128778000 "*" 3.90798504668055e-14 1.3513357794283e-13 -91.6256157635468
"chr8" 128843001 128844000 "*" 0 0 -100
"chr8" 128864001 128865000 "*" 1.52700074806944e-12 4.33955520708214e-12 -93.2258064516129
"chr8" 128879001 128880000 "*" 1.0550682549848e-10 2.34636626890124e-10 -87.9189399844115
"chr8" 128955001 128956000 "*" 1.16116116721798e-10 2.56888032893327e-10 -79.0697674418605
"chr8" 129053001 129054000 "*" 3.17569101571102e-07 4.31029739565322e-07 -52.9878371232152
"chr8" 129085001 129086000 "*" 1.83952852950142e-12 5.17499701329218e-12 100
"chr8" 129103001 129104000 "*" 1.01951780351328e-12 2.95926270789761e-12 -62.1025308241402
"chr8" 129114001 129115000 "*" 4.11539404343575e-06 4.77326970666537e-06 -56.5217391304348
"chr8" 129464001 129465000 "*" 1.68988143922633e-07 2.37604170590785e-07 70
"chr8" 129710001 129711000 "*" 2.43005615629954e-09 4.45014222280278e-09 100
"chr8" 129772001 129773000 "*" 0 0 100
"chr8" 130240001 130241000 "*" 0.000134368085498937 0.000123398369467587 51.7241379310345
"chr8" 130695001 130696000 "*" 1.9373391779709e-13 6.15971322898373e-13 100
"chr8" 130741001 130742000 "*" 0.000114124648469827 0.00010609116835605 58.5365853658537
"chr8" 130947001 130948000 "*" 2.20304996467746e-10 4.68606448808096e-10 59.2592592592593

"chr8" 131029001 131030000 "*" 1.01691988163566e-11 2.60007440113907e-11
57.8125
"chr8" 131092001 131093000 "*" 6.4152538836737e-10 1.27477697728417e-09 -100
"chr8" 131388001 131389000 "*" 2.88710388929303e-10 6.03399264731139e-10 100
"chr8" 131391001 131392000 "*" 1.47572004616237e-08 2.41681061133224e-08
52.4717514124294
"chr8" 131399001 131400000 "*" 0 0 -100
"chr8" 131419001 131420000 "*" 1.70641278884887e-12 4.81986032810646e-12 -100
"chr8" 131460001 131461000 "*" 5.55111512312578e-16 2.36485094870365e-15 100
"chr8" 131651001 131652000 "*" 6.42233020664573e-06 7.24338804013403e-06
81.8181818181818
"chr8" 131762001 131763000 "*" 1.37828859436695e-10 3.01445544267661e-10 100
"chr8" 131777001 131778000 "*" 1.27802879390515e-10 2.80947025907458e-10
83.3333333333333
"chr8" 131805001 131806000 "*" 1.36604061395929e-11 3.42333952202875e-11 100
"chr8" 132574001 132575000 "*" 8.72635297355373e-14 2.89204816212186e-13
97.6190476190476
"chr8" 132897001 132898000 "*" 2.88779000712225e-11 6.92935809466068e-11 -100
"chr8" 133066001 133067000 "*" 1.52794177310511e-09 2.87642564232045e-09 100
"chr8" 133141001 133142000 "*" 0 0 52.9527559055118
"chr8" 133152001 133153000 "*" 0 0 84.3373493975904
"chr8" 133239001 133240000 "*" 1.12132525487141e-14 4.14352271980498e-14 100
"chr8" 133461001 133462000 "*" 3.22375459660407e-12 8.79500753078322e-12 75
"chr8" 133578001 133579000 "*" 5.78631927711237e-05 5.63390103085987e-05 -52
"chr8" 133935001 133936000 "*" 1.07882591748876e-11 2.74553537455803e-11 -100
"chr8" 133949001 133950000 "*" 1.20281562487889e-12 3.46585342181251e-12 100
"chr8" 134012001 134013000 "*" 0.000183887360524659 0.000165164039707606
-58.3333333333333
"chr8" 134023001 134024000 "*" 2.66110801422359e-05 2.73198497110791e-05 68.75
"chr8" 134068001 134069000 "*" 8.7349629751543e-09 1.47505871898511e-08 100
"chr8" 134081001 134082000 "*" 2.43005615629954e-09 4.45014222280278e-09 -100
"chr8" 134091001 134092000 "*" 1.00808250635964e-13 3.31107370956809e-13 100
"chr8" 134106001 134107000 "*" 3.04324343503026e-11 7.26599935673121e-11 -100
"chr8" 134143001 134144000 "*" 4.99900121297969e-12 1.32921056339624e-11 -100
"chr8" 134205001 134206000 "*" 0 0 100
"chr8" 134220001 134221000 "*" 4.79616346638068e-14 1.64380445416596e-13 100
"chr8" 134261001 134262000 "*" 0 0 80.4878048780488
"chr8" 134414001 134415000 "*" 6.8110184159309e-11 1.55543177152814e-10
-80.7692307692308
"chr8" 134486001 134487000 "*" 6.59550958292954e-09 1.13456194382773e-08 100
"chr8" 134538001 134539000 "*" 6.73352729130272e-09 1.15706370487096e-08 -65
"chr8" 134539001 134540000 "*" 5.01025332333427e-10 1.01358031135889e-09 100
"chr8" 134540001 134541000 "*" 1.52466927971773e-12 4.33322475981408e-12 -100
"chr8" 134548001 134549000 "*" 0 0 -100
"chr8" 134552001 134553000 "*" 8.18345391451203e-13 2.40802229859118e-12
80.3921568627451
"chr8" 134564001 134565000 "*" 1.11022302462516e-16 5.03662826618488e-16 100
"chr8" 134573001 134574000 "*" 9.80763359414993e-10 1.8980032424715e-09 100
"chr8" 134635001 134636000 "*" 1.77734582385014e-07 2.49233931005097e-07
66.6666666666667
"chr8" 134641001 134642000 "*" 0 0 100
"chr8" 134677001 134678000 "*" 0 0 99.0909090909091

"chr8" 134765001 134766000 "*" 1.13140401492018e-08 1.87801380787728e-08 100
"chr8" 134776001 134777000 "*" 1.30868649250715e-11 3.29018676604239e-11
-64.1791044776119
"chr8" 134805001 134806000 "*" 9.04570973681018e-10 1.76414389822173e-09 -100
"chr8" 134872001 134873000 "*" 1.17905685215192e-13 3.83653245040640e-13 100
"chr8" 134882001 134883000 "*" 1.18940972515524e-07 1.70759485059015e-07
77.6119402985075
"chr8" 134927001 134928000 "*" 1.15463194561016e-13 3.76896708443697e-13
-65.7142857142857
"chr8" 135023001 135024000 "*" 1.40892907385393e-06 1.75339719977747e-06
64.7058823529412
"chr8" 135335001 135336000 "*" 1.56863411149288e-12 4.4469621057462e-12 100
"chr8" 135470001 135471000 "*" 0 0 100
"chr8" 135484001 135485000 "*" 4.13002965160558e-14 1.42427783430954e-13 100
"chr8" 135509001 135510000 "*" 2.22044604925031e-16 9.81641919380259e-16
64.8648648648649
"chr8" 135595001 135596000 "*" 4.9258274978925e-06 5.65100933800874e-06
-58.3333333333333
"chr8" 135632001 135633000 "*" 2.51135822915138e-07 3.45648271894177e-07 52
"chr8" 135671001 135672000 "*" 5.794809077031e-12 1.52911192757929e-11 -100
"chr8" 135697001 135698000 "*" 4.36241330459275e-06 5.0406393836561e-06
77.7777777777778
"chr8" 135738001 135739000 "*" 1.37329037031009e-10 3.00960095715877e-10
64.1025641025641
"chr8" 135757001 135758000 "*" 2.34643193763873e-10 4.96975541537004e-10
-85.8585858585859
"chr8" 135954001 135955000 "*" 3.40125705378114e-10 7.03784073980162e-10 100
"chr8" 136038001 136039000 "*" 8.01666433236647e-09 1.36392368970109e-08 -100
"chr8" 136143001 136144000 "*" 0 0 -100
"chr8" 136144001 136145000 "*" 3.07963323731286e-08 4.82018501272284e-08
-66.6666666666667
"chr8" 136364001 136365000 "*" 1.30340183090993e-13 4.22662705118657e-13 -100
"chr8" 136416001 136417000 "*" 1.0325074129014e-14 3.83008663711937e-14 100
"chr8" 136455001 136456000 "*" 3.09419156963031e-13 9.58360506053136e-13 74
"chr8" 136469001 136470000 "*" 0 0 70.717653221582
"chr8" 136612001 136613000 "*" 7.86441590294729e-07 1.0129538811221e-06
-50.9803921568627
"chr8" 136679001 136680000 "*" 0 0 100
"chr8" 136696001 136697000 "*" 5.55111512312578e-16 2.36485094870365e-15
-66.5307971014493
"chr8" 136718001 136719000 "*" 2.69084754478399e-12 7.41487347763769e-12 -100
"chr8" 136722001 136723000 "*" 1.49310823704951e-08 2.44375206748162e-08 75
"chr8" 136734001 136735000 "*" 2.22044604925031e-16 9.81641919380259e-16 -100
"chr8" 136803001 136804000 "*" 2.09983736660924e-05 2.19044051649657e-05
-66.6666666666667
"chr8" 137543001 137544000 "*" 0 0 60.5128205128205
"chr8" 137805001 137806000 "*" 5.00207610887315e-08 7.5866849817322e-08
-79.0909090909091
"chr8" 138001001 138002000 "*" 0.000489333567287087 0.000409352035812982
51.3513513513514
"chr8" 138154001 138155000 "*" 4.73234496034536e-09 8.30627300264826e-09 -100
"chr8" 138272001 138273000 "*" 1.13140401492018e-08 1.87801380787728e-08 -100

"chr8" 138347001 138348000 "*" 0 0 100
"chr8" 138576001 138577000 "*" 0 0 -95.7142857142857
"chr8" 138869001 138870000 "*" 5.48638023900594e-10 1.10374357336421e-09 100
"chr8" 138991001 138992000 "*" 0 0 -83.7606837606838
"chr8" 139153001 139154000 "*" 6.65423272039334e-12 1.74238688480539e-11
91.6666666666667
"chr8" 139198001 139199000 "*" 0 0 100
"chr8" 139218001 139219000 "*" 8.41958391895048e-10 1.65021424853196e-09
-68.0851063829787
"chr8" 139275001 139276000 "*" 1.07882591748876e-11 2.74553537455803e-11 -100
"chr8" 139356001 139357000 "*" 5.91870164878827e-07 7.75564979754954e-07
-57.6923076923077
"chr8" 139621001 139622000 "*" 5.25841424847684e-08 7.95060575196433e-08
60.6060606060606
"chr8" 139744001 139745000 "*" 2.02327044007689e-12 5.65300384792621e-12 -100
"chr8" 139760001 139761000 "*" 0 0 100
"chr8" 139775001 139776000 "*" 4.71134242729931e-12 1.257615036806e-11 100
"chr8" 139776001 139777000 "*" 1.01294167498267e-08 1.69737481029529e-08
-59.2592592592593
"chr8" 139779001 139780000 "*" 2.67705857481815e-11 6.4668660671553e-11
67.1428571428571
"chr8" 139805001 139806000 "*" 2.69018141096922e-12 7.41487347763769e-12 100
"chr8" 139823001 139824000 "*" 1.98325800226939e-11 4.85612402473442e-11 -100
"chr8" 139869001 139870000 "*" 9.35527815004722e-06 1.03009706123167e-05
51.3513513513514
"chr8" 139883001 139884000 "*" 9.08108033215171e-11 2.03407753381119e-10 -100
"chr8" 139895001 139896000 "*" 0 0 -62.3853211009174
"chr8" 139925001 139926000 "*" 3.10862446895044e-15 1.22466437093774e-14
80.8219178082192
"chr8" 139947001 139948000 "*" 9.0072393987839e-13 2.63157725646847e-12 -100
"chr8" 140034001 140035000 "*" 1.7608881719422e-07 2.470340978413e-07
-52.3809523809524
"chr8" 140177001 140178000 "*" 1.52794177310511e-09 2.87642564232045e-09 -100
"chr8" 140254001 140255000 "*" 1.12458486967171e-09 2.15740815043925e-09 -100
"chr8" 140385001 140386000 "*" 0 0 100
"chr8" 140408001 140409000 "*" 0 0 100
"chr8" 140619001 140620000 "*" 3.63445940010365e-11 8.58565209111204e-11 100
"chr8" 140637001 140638000 "*" 9.45735739610321e-07 1.20440104189486e-06
70.4545454545455
"chr8" 140641001 140642000 "*" 1.05760955548817e-11 2.69807212925256e-11
-65.7142857142857
"chr8" 140644001 140645000 "*" 0 0 100
"chr8" 140651001 140652000 "*" 1.41431311106999e-12 4.04148218997523e-12
83.6820083682008
"chr8" 140661001 140662000 "*" 0 0 71.4285714285714
"chr8" 140664001 140665000 "*" 1.54765008828495e-05 1.64771109106961e-05
-66.6666666666667
"chr8" 140685001 140686000 "*" 0 0 -100
"chr8" 140691001 140692000 "*" 0 0 87.6288659793814
"chr8" 140693001 140694000 "*" 1.18003384841359e-11 2.98509646654514e-11
84.7750865051903
"chr8" 140716001 140717000 "*" 0 0 50.4399681490971

"chr8" 140726001 140727000 "*" 1.51914614288096e-06 1.88121349342686e-06 73.6842105263158
"chr8" 140745001 140746000 "*" 4.21884749357559e-15 1.63874445239189e-14 76.1904761904762
"chr8" 140780001 140781000 "*" 4.73234496034536e-09 8.30627300264826e-09 100
"chr8" 140789001 140790000 "*" 6.4152538836737e-10 1.27477697728417e-09 -100
"chr8" 140814001 140815000 "*" 0 0 -78.125
"chr8" 140840001 140841000 "*" 1.4508594325946e-10 3.16264212972944e-10 -92.3076923076923
"chr8" 140855001 140856000 "*" 7.06479319489972e-12 1.83955253325447e-11 -88.4615384615385
"chr8" 140884001 140885000 "*" 1.48900891616677e-11 3.71653742151198e-11 -59.375
"chr8" 140895001 140896000 "*" 1.77635683940025e-15 7.19870740878856e-15 100
"chr8" 140900001 140901000 "*" 0 0 100
"chr8" 140915001 140916000 "*" 0 0 63.953488372093
"chr8" 140962001 140963000 "*" 0 0 100
"chr8" 141224001 141225000 "*" 4.96802599059265e-12 1.32342158610897e-11 51.9230769230769
"chr8" 141275001 141276000 "*" 5.16020559615527e-12 1.37010837995059e-11 -51.5151515151515
"chr8" 141314001 141315000 "*" 1.68753899743024e-14 6.09826805967439e-14 -100
"chr8" 141409001 141410000 "*" 6.59550958292954e-09 1.13456194382773e-08 -100
"chr8" 141467001 141468000 "*" 0 0 -58.3333333333333
"chr8" 141487001 141488000 "*" 4.18887147191072e-13 1.2739946038722e-12 100
"chr8" 141522001 141523000 "*" 0 0 -100
"chr8" 141599001 141600000 "*" 0 0 -89.4194756554307
"chr8" 141603001 141604000 "*" 2.55351295663786e-15 1.01678090381833e-14 100
"chr8" 141704001 141705000 "*" 2.02060590481778e-13 6.40127405465654e-13 100
"chr8" 141804001 141805000 "*" 3.6700841921089e-08 5.65530226072258e-08 -100
"chr8" 142040001 142041000 "*" 0.000144402658994736 0.000131924792739962 -66.6666666666667
"chr8" 142052001 142053000 "*" 2.79440914852103e-11 6.73046092948135e-11 -100
"chr8" 142090001 142091000 "*" 0 0 100
"chr8" 142109001 142110000 "*" 0 0 -85
"chr8" 142185001 142186000 "*" 2.46469511466785e-14 8.7281767390389e-14 53.6585365853659
"chr8" 142244001 142245000 "*" 1.00594293006573e-07 1.4592190746725e-07 56.3218390804598
"chr8" 142270001 142271000 "*" 3.33066907387547e-16 1.4495649018245e-15 64.8648648648649
"chr8" 142275001 142276000 "*" 0 0 100
"chr8" 142279001 142280000 "*" 0 0 75.0425135638513
"chr8" 142305001 142306000 "*" 5.03393993156465e-11 1.16750305560645e-10 -56
"chr8" 142314001 142315000 "*" 2.08814465718632e-09 3.86232567960463e-09 100
"chr8" 142369001 142370000 "*" 1.99889903396144e-06 2.43162199863531e-06 -53.0864197530864
"chr8" 142375001 142376000 "*" 0 0 -65.8670143415906
"chr8" 142394001 142395000 "*" 0 0 83.7349397590361
"chr8" 142398001 142399000 "*" 0 0 82.089552238806
"chr8" 142444001 142445000 "*" 7.16093850883226e-14 2.39946670387204e-13 67.4603174603175

"chr8" 142469001 142470000 "*" 1.39571996626842e-07 1.98463037958589e-07
61.5217391304348
"chr8" 142479001 142480000 "*" 8.65973959207622e-14 2.87362558212404e-13 100
"chr8" 142489001 142490000 "*" 3.98903132747819e-13 1.21783436908132e-12
-57.8947368421053
"chr8" 142506001 142507000 "*" 0 0 89.2156862745098
"chr8" 142516001 142517000 "*" 0 0 91.3333333333333
"chr8" 142525001 142526000 "*" 0.000120656929643692 0.000111697991192225
56.0606060606061
"chr8" 142531001 142532000 "*" 2.02060590481778e-13 6.40127405465654e-13 -100
"chr8" 142532001 142533000 "*" 1.86821217281752e-05 1.96397758022194e-05 60
"chr8" 142538001 142539000 "*" 8.81497896898509e-09 1.48790809509528e-08
60.2739726027397
"chr8" 142541001 142542000 "*" 0 0 71.9541910331384
"chr8" 142549001 142550000 "*" 0.000572262462534168 0.000473009070524024
51.8939393939394
"chr8" 142563001 142564000 "*" 2.22044604925031e-16 9.81641919380259e-16 100
"chr8" 142567001 142568000 "*" 2.03213446070549e-10 4.3565131316088e-10
69.0909090909091
"chr8" 142571001 142572000 "*" 0 0 78.5714285714286
"chr8" 142576001 142577000 "*" 6.70463684571132e-13 1.98922584795814e-12
-83.0188679245283
"chr8" 142582001 142583000 "*" 1.93720595120794e-11 4.76309924836636e-11 100
"chr8" 142589001 142590000 "*" 4.31663183086961e-07 5.75408533798075e-07
-53.8461538461538
"chr8" 142598001 142599000 "*" 1.08110537369033e-05 1.17825703914403e-05
-64.8148148148148
"chr8" 142602001 142603000 "*" 0.000322941373535057 0.000278417329624087
-54.5454545454545
"chr8" 142611001 142612000 "*" 1.15384740040358e-06 1.45275097583569e-06 -56
"chr8" 142618001 142619000 "*" 0 0 91.8604651162791
"chr8" 142619001 142620000 "*" 0 0 76.5625
"chr8" 142620001 142621000 "*" 1.22852037431898e-08 2.03070965136608e-08
76.1904761904762
"chr8" 142626001 142627000 "*" 1.57549938650803e-08 2.57093167367977e-08
63.8888888888889
"chr8" 142629001 142630000 "*" 3.98542288060355e-09 7.09364760785609e-09
-64.4067796610169
"chr8" 142633001 142634000 "*" 0 0 57.6332134684537
"chr8" 142636001 142637000 "*" 1.80989024236577e-05 1.90667975357288e-05 68.75
"chr8" 142639001 142640000 "*" 0 0 65.748031496063
"chr8" 142650001 142651000 "*" 6.53921361504217e-14 2.20157828648574e-13
97.6744186046512
"chr8" 142653001 142654000 "*" 1.87620141645084e-10 4.04133644431758e-10
53.6585365853659
"chr8" 142657001 142658000 "*" 0 0 -100
"chr8" 142659001 142660000 "*" 7.80919773291089e-12 2.02579326189407e-11
57.2720125786163
"chr8" 142675001 142676000 "*" 1.11022302462516e-16 5.03662826618488e-16
75.5395683453237
"chr8" 142681001 142682000 "*" 0 0 72.1311475409836
"chr8" 142682001 142683000 "*" 0 0 100

"chr8" 142683001 142684000 "*" 0 0 81.8181818181818
"chr8" 142703001 142704000 "*" 0.000114260121221954 0.000106207633919052
55.1020408163265
"chr8" 142708001 142709000 "*" 0 0 -95.0819672131148
"chr8" 142716001 142717000 "*" 3.33066907387547e-16 1.4495649018245e-15
-76.5957446808511
"chr8" 142717001 142718000 "*" 2.8978121360046e-05 2.95864115614715e-05
-63.1578947368421
"chr8" 142734001 142735000 "*" 0 0 69.4199860237596
"chr8" 142739001 142740000 "*" 1.85215696113517e-08 2.98749045480115e-08
91.304347826087
"chr8" 142740001 142741000 "*" 5.02009545044757e-11 1.16451884243799e-10
97.0588235294118
"chr8" 142744001 142745000 "*" 2.4535928844216e-14 8.69144396197119e-14 100
"chr8" 142833001 142834000 "*" 0 0 100
"chr8" 142835001 142836000 "*" 2.02327044007689e-12 5.65300384792621e-12 -100
"chr8" 142841001 142842000 "*" 0 0 50.4310954063604
"chr8" 142845001 142846000 "*" 1.68753899743024e-14 6.09826805967439e-14 100
"chr8" 142856001 142857000 "*" 3.19523780767383e-08 4.99222667935413e-08
79.7619047619048
"chr8" 142867001 142868000 "*" 0 0 82.6568265682657
"chr8" 142873001 142874000 "*" 1.37828859436695e-10 3.01445544267661e-10 -100
"chr8" 142874001 142875000 "*" 6.92287338566189e-11 1.57605630124247e-10 100
"chr8" 142880001 142881000 "*" 0 0 65.8291457286432
"chr8" 142881001 142882000 "*" 0 0 57.9089827890254
"chr8" 142895001 142896000 "*" 4.31987778881648e-13 1.31202046034671e-12
71.9512195121951
"chr8" 142934001 142935000 "*" 3.64270735797589e-09 6.5252880340617e-09
66.6666666666667
"chr8" 142942001 142943000 "*" 0 0 53.9563862928349
"chr8" 142954001 142955000 "*" 7.67494956477321e-11 1.73684306427893e-10
-61.9281045751634
"chr8" 142987001 142988000 "*" 0 0 58.0148729601322
"chr8" 142990001 142991000 "*" 0 0 98.2817869415808
"chr8" 143005001 143006000 "*" 2.52509124720746e-12 6.98303501323638e-12
68.8888888888889
"chr8" 143006001 143007000 "*" 0 0 87.012987012987
"chr8" 143012001 143013000 "*" 5.7065463465733e-14 1.93318145576218e-13 100
"chr8" 143014001 143015000 "*" 5.94482056870405e-06 6.73964764208124e-06
64.1304347826087
"chr8" 143016001 143017000 "*" 8.54294412988565e-12 2.20330884185928e-11
-68.9320388349515
"chr8" 143017001 143018000 "*" 0 0 55.8513109857285
"chr8" 143029001 143030000 "*" 0 0 100
"chr8" 143048001 143049000 "*" 2.75335310107039e-14 9.69727903855348e-14 100
"chr8" 143060001 143061000 "*" 4.44089209850063e-16 1.91071758245033e-15 100
"chr8" 143068001 143069000 "*" 7.9590863601986e-06 8.85270264440226e-06
71.4285714285714
"chr8" 143078001 143079000 "*" 0 0 -61.038961038961
"chr8" 143093001 143094000 "*" 3.51352280603123e-12 9.54993647640235e-12
-67.3076923076923
"chr8" 143096001 143097000 "*" 0 0 83.433734939759

"chr8" 143099001 143100000 "*" 9.55706624949926e-11 2.13610437421291e-10
-52.7777777777778
"chr8" 143100001 143101000 "*" 5.27068548805687e-08 7.96829356899468e-08
76.5957446808511
"chr8" 143101001 143102000 "*" 0 0 -55.8139534883721
"chr8" 143104001 143105000 "*" 4.34297042772869e-12 1.16457707090012e-11
51.2631578947368
"chr8" 143106001 143107000 "*" 0 0 100
"chr8" 143110001 143111000 "*" 8.57092175010621e-13 2.51149198099779e-12 -100
"chr8" 143111001 143112000 "*" 3.56292773062705e-12 9.67917446211584e-12
87.9194630872483
"chr8" 143114001 143115000 "*" 2.98718576297041e-06 3.54149252433917e-06
-70.8333333333333
"chr8" 143119001 143120000 "*" 4.87999789969829e-05 4.80752233746951e-05
54.8872180451128
"chr8" 143124001 143125000 "*" 0 0 -73.1182795698925
"chr8" 143125001 143126000 "*" 0 0 77.1300448430493
"chr8" 143126001 143127000 "*" 2.30429864855886e-07 3.18579857629035e-07
72.6190476190476
"chr8" 143137001 143138000 "*" 8.01666433236647e-09 1.36392368970109e-08 100
"chr8" 143138001 143139000 "*" 3.29650389763181e-07 4.46504567878999e-07 68.75
"chr8" 143142001 143143000 "*" 3.64375345451862e-08 5.63984146563096e-08
55.9523809523809
"chr8" 143144001 143145000 "*" 1.11022302462516e-16 5.03662826618488e-16 100
"chr8" 143146001 143147000 "*" 3.58125009896071e-05 3.60127270429505e-05
55.5555555555556
"chr8" 143149001 143150000 "*" 1.67299207820548e-08 2.71377429441055e-08 -100
"chr8" 143150001 143151000 "*" 4.03502955802493e-05 4.02473601828171e-05
59.6153846153846
"chr8" 143151001 143152000 "*" 1.87510396266743e-05 1.97080033495427e-05
66.6666666666667
"chr8" 143158001 143159000 "*" 4.95492535890207e-13 1.49818449177123e-12 -90
"chr8" 143163001 143164000 "*" 1.88737914186277e-15 7.62011598380321e-15 100
"chr8" 143181001 143182000 "*" 0 0 -100
"chr8" 143195001 143196000 "*" 2.14690712430077e-07 2.98005376632534e-07
51.6339869281046
"chr8" 143213001 143214000 "*" 0 0 100
"chr8" 143223001 143224000 "*" 7.56166920079515e-07 9.76130266999869e-07 -74
"chr8" 143225001 143226000 "*" 0 0 69.8580093741384
"chr8" 143241001 143242000 "*" 0 0 84.0579710144928
"chr8" 143252001 143253000 "*" 0 0 50.6939823177108
"chr8" 143287001 143288000 "*" 0 0 73.2142857142857
"chr8" 143295001 143296000 "*" 7.32747196252603e-14 2.45319846132465e-13
89.344262295082
"chr8" 143300001 143301000 "*" 1.12132525487141e-14 4.14352271980498e-14 100
"chr8" 143309001 143310000 "*" 2.22044604925031e-16 9.81641919380259e-16 100
"chr8" 143315001 143316000 "*" 0 0 100
"chr8" 143328001 143329000 "*" 3.15091588376504e-08 4.92500807374512e-08 -51.25
"chr8" 143338001 143339000 "*" 9.65338919911574e-13 2.80678910656113e-12 -100
"chr8" 143342001 143343000 "*" 8.7349629751543e-09 1.47505871898511e-08 100
"chr8" 143349001 143350000 "*" 1.06581410364015e-14 3.94854473541761e-14
88.785046728972

"chr8" 143350001 143351000 "*" 2.00227675550835e-08 3.20459176617961e-08 100
"chr8" 143462001 143463000 "*" 3.10862446895044e-15 1.22466437093774e-14 56.8181818181818
"chr8" 143483001 143484000 "*" 0 0 -51.2181195279787
"chr8" 143488001 143489000 "*" 3.29235220752899e-07 4.46007461671159e-07 72.0930232558139
"chr8" 143496001 143497000 "*" 8.22082840690186e-08 1.20623296763529e-07 -54.5384615384615
"chr8" 143500001 143501000 "*" 6.86864152058542e-05 6.6065747506236e-05 -50.9433962264151
"chr8" 143506001 143507000 "*" 3.65263375101677e-14 1.26882502648938e-13 54.3010752688172
"chr8" 143515001 143516000 "*" 2.1888046930485e-12 6.09569696232637e-12 -58.2089552238806
"chr8" 143518001 143519000 "*" 4.27790025625541e-11 1.00162791392068e-10 -66.6666666666667
"chr8" 143527001 143528000 "*" 1.64201985342061e-13 5.26754366541111e-13 59.0740740740741
"chr8" 143540001 143541000 "*" 0 0 69.3877551020408
"chr8" 143560001 143561000 "*" 0 0 52.8668703067328
"chr8" 143567001 143568000 "*" 0 0 100
"chr8" 143577001 143578000 "*" 5.72126176558996e-05 5.57465732173144e-05 62.5
"chr8" 143578001 143579000 "*" 1.48370205010906e-12 4.23150104553102e-12 72
"chr8" 143593001 143594000 "*" 2.08814465718632e-09 3.86232567960463e-09 100
"chr8" 143605001 143606000 "*" 0 0 60.7442348008386
"chr8" 143627001 143628000 "*" 0 0 69.5488721804511
"chr8" 143628001 143629000 "*" 0 0 57.5342465753425
"chr8" 143633001 143634000 "*" 3.07963307077941e-08 4.82018501272284e-08 66.6666666666667
"chr8" 143651001 143652000 "*" 1.87849735766576e-13 5.98632031198879e-13 77.7777777777778
"chr8" 143673001 143674000 "*" 1.29037891483108e-11 3.24582280286866e-11 100
"chr8" 143686001 143687000 "*" 2.11424530849325e-06 2.56432546771576e-06 -70.5882352941177
"chr8" 143687001 143688000 "*" 0 0 87.8787878787879
"chr8" 143696001 143697000 "*" 1.11022302462516e-16 5.03662826618488e-16 63.3165829145729
"chr8" 143703001 143704000 "*" 1.52215262616551e-09 2.87642564232045e-09 53.3596837944664
"chr8" 143711001 143712000 "*" 0 0 100
"chr8" 143754001 143755000 "*" 1.50623957750895e-12 4.28756717183481e-12 -100
"chr8" 143756001 143757000 "*" 2.05391259555654e-14 7.35435952190685e-14 100
"chr8" 143766001 143767000 "*" 7.0006535457523e-08 1.03695832512054e-07 100
"chr8" 143844001 143845000 "*" 7.8042161621994e-10 1.53537711650689e-09 54.0942302847065
"chr8" 143853001 143854000 "*" 3.33066907387547e-16 1.4495649018245e-15 100
"chr8" 143855001 143856000 "*" 0 0 59.7806935332709
"chr8" 143863001 143864000 "*" 1.52466927971773e-12 4.33322475981408e-12 -100
"chr8" 143877001 143878000 "*" 3.53795803764712e-09 6.34923861574054e-09 50.8270676691729
"chr8" 143892001 143893000 "*" 0 0 63.6203522504892
"chr8" 143902001 143903000 "*" 6.93055056344427e-07 8.99238849022958e-07

69.6969696969697
"chr8" 143922001 143923000 "*" 4.85167461761193e-14 1.6617373494005e-13 80
"chr8" 143955001 143956000 "*" 1.16213356604611e-07 1.6712548668921e-07
69.2307692307692
"chr8" 143959001 143960000 "*" 0 0 -88.3116883116883
"chr8" 144000001 144001000 "*" 2.22044604925031e-16 9.81641919380259e-16 -100
"chr8" 144002001 144003000 "*" 2.76259015663527e-11 6.66074830172604e-11
67.741935483871
"chr8" 144005001 144006000 "*" 4.33619806727847e-12 1.16291060843037e-11
-70.5882352941177
"chr8" 144064001 144065000 "*" 2.22044604925031e-16 9.81641919380259e-16 -100
"chr8" 144107001 144108000 "*" 5.2939874706226e-12 1.40406488837616e-11
58.2608695652174
"chr8" 144108001 144109000 "*" 7.93809462606987e-14 2.64679416473686e-13
91.9191919191919
"chr8" 144117001 144118000 "*" 1.94225380223401e-09 3.61429159708956e-09
64.6153846153846
"chr8" 144151001 144152000 "*" 0 0 52.4590163934426
"chr8" 144165001 144166000 "*" 0 0 92.5170068027211
"chr8" 144176001 144177000 "*" 0 0 84.7826086956522
"chr8" 144190001 144191000 "*" 0 0 66.9368456602499
"chr8" 144193001 144194000 "*" 1.88737914186277e-15 7.62011598380321e-15 100
"chr8" 144212001 144213000 "*" 0 0 83.1632653061225
"chr8" 144275001 144276000 "*" 0 0 70.8771293947082
"chr8" 144301001 144302000 "*" 0 0 -57.3787061994609
"chr8" 144321001 144322000 "*" 3.30900862266503e-11 7.8705688286847e-11
54.1176470588235
"chr8" 144323001 144324000 "*" 9.65729496371637e-10 1.87255912493232e-09 100
"chr8" 144347001 144348000 "*" 3.6957848053909e-05 3.70799784206646e-05 -53.125
"chr8" 144397001 144398000 "*" 8.1690210151919e-13 2.4038601588706e-12
56.0248447204969
"chr8" 144438001 144439000 "*" 1.42815324899459e-07 2.02562841355905e-07
58.1313131313131
"chr8" 144440001 144441000 "*" 4.52782256132878e-12 1.21176894833597e-11
54.3478260869565
"chr8" 144465001 144466000 "*" 8.73323413586036e-10 1.70851683922568e-09
58.3333333333333
"chr8" 144473001 144474000 "*" 0 0 55.5555555555556
"chr8" 144474001 144475000 "*" 1.65423230669148e-14 5.98698695225911e-14
63.0434782608696
"chr8" 144477001 144478000 "*" 1.55431223447522e-15 6.33705141101682e-15 -100
"chr8" 144511001 144512000 "*" 0 0 53.0804121154136
"chr8" 144512001 144513000 "*" 0 0 59.175827739404
"chr8" 144514001 144515000 "*" 1.11577413974828e-13 3.65127289161778e-13
62.8552971576227
"chr8" 144515001 144516000 "*" 0 0 99.2307692307692
"chr8" 144531001 144532000 "*" 2.64951482975562e-09 4.82186011375752e-09 100
"chr8" 144635001 144636000 "*" 0 0 -63.8321080798555
"chr8" 144640001 144641000 "*" 0 0 -66.0112359550562
"chr8" 144660001 144661000 "*" 0 0 -85.3426713356678
"chr8" 144717001 144718000 "*" 0 0 -100
"chr8" 144762001 144763000 "*" 5.71864777754172e-12 1.51207662916488e-11

66.6666666666667
"chr8" 144763001 144764000 "*" 2.08785967781289e-06 2.53403795774669e-06
-72.7272727272727
"chr8" 144843001 144844000 "*" 0 0 53.6386349322795
"chr8" 144868001 144869000 "*" 5.41033884360331e-12 1.43346473854355e-11
82.1428571428571
"chr8" 144909001 144910000 "*" 1.58346329381942e-08 2.58271480686597e-08
-54.1666666666667
"chr8" 144910001 144911000 "*" 0 0 100
"chr8" 144976001 144977000 "*" 6.66133814775094e-16 2.81595744474255e-15
56.3380281690141
"chr8" 145023001 145024000 "*" 0 0 -80.2941176470588
"chr8" 145024001 145025000 "*" 0 0 -66.7863997215128
"chr8" 145027001 145028000 "*" 0 0 57.58407960199
"chr8" 145047001 145048000 "*" 0 0 54.4152115317005
"chr8" 145049001 145050000 "*" 0 0 -54.281153802303
"chr8" 145081001 145082000 "*" 1.35890187991095e-11 3.40841971363279e-11 -100
"chr8" 145091001 145092000 "*" 7.0006535457523e-08 1.03695832512054e-07 100
"chr8" 145122001 145123000 "*" 8.57092175010621e-13 2.51149198099779e-12 100
"chr8" 145277001 145278000 "*" 3.90972831887382e-09 6.98201504766899e-09
60.7142857142857
"chr8" 145547001 145548000 "*" 1.14352971536391e-14 4.21921674335999e-14 -56
"chr8" 145556001 145557000 "*" 0 0 50.578631452581
"chr8" 145593001 145594000 "*" 3.62598839842576e-13 1.11545114393694e-12
-80.3921568627451
"chr8" 145690001 145691000 "*" 0 0 70.1298701298701
"chr8" 145733001 145734000 "*" 3.88578058618805e-15 1.5152981210988e-14 -100
"chr8" 145744001 145745000 "*" 0.000240479789304304 0.000211878237500588
-57.1428571428571
"chr8" 145751001 145752000 "*" 0 0 -50.7492507492507
"chr8" 145840001 145841000 "*" 1.17794662912729e-13 3.83653245040640e-13 -100
"chr8" 146009001 146010000 "*" 8.28461780728595e-05 7.86861290576483e-05
61.5384615384615
"chr8" 146016001 146017000 "*" 5.01207103598134e-08 7.60032396338976e-08 60
"chr8" 146128001 146129000 "*" 1.14352971536391e-14 4.21921674335999e-14
78.5046728971963
"chr8" 146277001 146278000 "*" 0 0 78.1725888324873
"chr8" 146278001 146279000 "*" 4.32986979603811e-15 1.68006959513174e-14
-51.7530864197531
"chr9" 41001 42000 "*" 4.46588244162882e-09 7.88437146944486e-09
52.112676056338
"chr9" 73001 74000 "*" 0 0 69.8188976036559
"chr9" 215001 216000 "*" 0 0 88.7423043095866
"chr9" 377001 378000 "*" 0 0 -100
"chr9" 469001 470000 "*" 0 0 100
"chr9" 720001 721000 "*" 1.56863411149288e-12 4.4469621057462e-12 -100
"chr9" 885001 886000 "*" 0 0 -100
"chr9" 1171001 1172000 "*" 2.75827648309468e-05 2.82530803747995e-05
-53.8461538461538
"chr9" 2621001 2622000 "*" 0 0 88.8745148771022
"chr9" 2622001 2623000 "*" 0 0 83.8390092879257
"chr9" 2854001 2855000 "*" 8.06688049692639e-13 2.37556391305009e-12 100

"chr9" 3110001 3111000 "*" 1.97906624421762e-09 3.68024937271622e-09 75
"chr9" 3756001 3757000 "*" 1.13140401492018e-08 1.87801380787728e-08 -100
"chr9" 3829001 3830000 "*" 1.93720595120794e-11 4.76309924836636e-11 -100
"chr9" 3898001 3899000 "*" 1.04037820496394e-05 1.1367667257137e-05
66.6666666666667
"chr9" 4009001 4010000 "*" 0 0 100
"chr9" 4297001 4298000 "*" 0 0 -100
"chr9" 4771001 4772000 "*" 3.88333809553387e-12 1.0505087836071e-11
-71.4285714285714
"chr9" 5450001 5451000 "*" 0 0 -54.1666666666667
"chr9" 5517001 5518000 "*" 6.46149800331841e-14 2.17664365839349e-13 100
"chr9" 6069001 6070000 "*" 1.11022302462516e-16 5.03662826618488e-16 100
"chr9" 6528001 6529000 "*" 8.72524275052911e-12 2.24707054911043e-11
-59.6153846153846
"chr9" 6697001 6698000 "*" 0 0 100
"chr9" 6790001 6791000 "*" 6.78816465543575e-05 6.53391574231243e-05
63.6363636363636
"chr9" 6928001 6929000 "*" 2.08814465718632e-09 3.86232567960463e-09 100
"chr9" 7365001 7366000 "*" 1.4432899320127e-15 5.90750815956055e-15 -100
"chr9" 7375001 7376000 "*" 2.00227675550835e-08 3.20459176617961e-08 -100
"chr9" 8486001 8487000 "*" 0 0 -100
"chr9" 8567001 8568000 "*" 1.29738864318441e-07 1.85457145691328e-07 -90
"chr9" 8643001 8644000 "*" 0 0 -100
"chr9" 8723001 8724000 "*" 5.57115382049389e-07 7.32474873970709e-07
-75.2808988764045
"chr9" 8810001 8811000 "*" 1.87405646556726e-13 5.97285292681429e-13 -100
"chr9" 8857001 8858000 "*" 0 0 87.29792147806
"chr9" 9442001 9443000 "*" 3.08197911635943e-13 9.54755242368424e-13
91.7098445595855
"chr9" 10612001 10613000 "*" 0 0 61.766730025562
"chr9" 10880001 10881000 "*" 4.9960036108132e-15 1.9234186548721e-14
94.1176470588235
"chr9" 10967001 10968000 "*" 2.88710388929303e-10 6.03399264731139e-10 100
"chr9" 11471001 11472000 "*" 4.26951363152739e-10 8.72510873516957e-10 -100
"chr9" 12274001 12275000 "*" 2.15125472990962e-10 4.58126659943874e-10 -100
"chr9" 12432001 12433000 "*" 2.99760216648792e-15 1.18450729613838e-14 75
"chr9" 13034001 13035000 "*" 0 0 100
"chr9" 13569001 13570000 "*" 3.56775498033812e-10 7.35898629809829e-10 100
"chr9" 14313001 14314000 "*" 0 0 75.1500711677703
"chr9" 14419001 14420000 "*" 0 0 100
"chr9" 14438001 14439000 "*" 2.69018141096922e-12 7.41487347763769e-12 100
"chr9" 15414001 15415000 "*" 1.98365768255826e-11 4.85612402473442e-11 100
"chr9" 15524001 15525000 "*" 1.26565424807268e-14 4.63923203146481e-14 100
"chr9" 16052001 16053000 "*" 0 0 -84.2105263157895
"chr9" 16058001 16059000 "*" 6.4324989779152e-11 1.47230020825092e-10 100
"chr9" 16129001 16130000 "*" 1.07882591748876e-11 2.74553537455803e-11 -100
"chr9" 16192001 16193000 "*" 0 0 100
"chr9" 16207001 16208000 "*" 0 0 -100
"chr9" 16233001 16234000 "*" 3.95353005888666e-09 7.03928529283733e-09 100
"chr9" 16259001 16260000 "*" 2.88710388929303e-10 6.03399264731139e-10 -100
"chr9" 16276001 16277000 "*" 1.11022302462516e-16 5.03662826618488e-16 -100
"chr9" 16369001 16370000 "*" 7.88258347483861e-15 2.96145301916012e-14 100

"chr9" 16494001 16495000 "*" 1.22124532708767e-15 5.03921984217778e-15 100
"chr9" 16877001 16878000 "*" 0 0 -61.8831168831169
"chr9" 17203001 17204000 "*" 1.67299207820548e-08 2.71377429441055e-08 100
"chr9" 17579001 17580000 "*" 0 0 79.5109385756131
"chr9" 18504001 18505000 "*" 8.52140580320793e-12 2.19817259951489e-11 -100
"chr9" 18564001 18565000 "*" 1.51914614832105e-06 1.88121349342686e-06
52.3809523809524
"chr9" 18706001 18707000 "*" 0 0 -100
"chr9" 18809001 18810000 "*" 4.83921813732024e-09 8.48582169090024e-09
-66.6666666666667
"chr9" 19103001 19104000 "*" 0 0 98.3739837398374
"chr9" 19250001 19251000 "*" 2.33442109968118e-05 2.41802782933016e-05 -75
"chr9" 19550001 19551000 "*" 3.95353005888666e-09 7.03928529283733e-09 100
"chr9" 20087001 20088000 "*" 1.68753899743024e-14 6.09826805967439e-14 100
"chr9" 20186001 20187000 "*" 1.39779633423487e-07 1.98463037958589e-07 -100
"chr9" 20506001 20507000 "*" 2.73625566649116e-12 7.52483363579137e-12 -100
"chr9" 21449001 21450000 "*" 5.63660229602192e-13 1.68905058935478e-12 -100
"chr9" 21589001 21590000 "*" 2.68450373042128e-10 5.64465213127486e-10 -100
"chr9" 21960001 21961000 "*" 3.04324343503026e-11 7.26599935673121e-11 100
"chr9" 22508001 22509000 "*" 4.0956127378422e-13 1.24913862444325e-12
58.972073677956
"chr9" 23219001 23220000 "*" 0 0 -100
"chr9" 23531001 23532000 "*" 3.6700841921089e-08 5.65530226072258e-08 -100
"chr9" 23821001 23822000 "*" 0 0 50.3095668038825
"chr9" 23850001 23851000 "*" 0 0 56.4407059211611
"chr9" 24134001 24135000 "*" 6.90747459231034e-12 1.80140148623876e-11 -100
"chr9" 24223001 24224000 "*" 6.52733422867868e-12 1.71146951718423e-11 100
"chr9" 24362001 24363000 "*" 2.1094237467878e-15 8.47434540879246e-15 -100
"chr9" 24545001 24546000 "*" 1.83952852950142e-12 5.17499701329218e-12 -100
"chr9" 24765001 24766000 "*" 3.03090885722668e-14 1.06210046699299e-13 100
"chr9" 25798001 25799000 "*" 1.13140401492018e-08 1.87801380787728e-08 -100
"chr9" 26082001 26083000 "*" 0 0 100
"chr9" 27340001 27341000 "*" 1.87405646556726e-13 5.97285292681429e-13 -100
"chr9" 28388001 28389000 "*" 2.43005615629954e-09 4.45014222280278e-09 -100
"chr9" 28865001 28866000 "*" 2.58681964737661e-14 9.14008479605639e-14 100
"chr9" 29212001 29213000 "*" 0 0 55.905832370979
"chr9" 29450001 29451000 "*" 4.99900121297969e-12 1.32921056339624e-11 -100
"chr9" 30411001 30412000 "*" 6.92287338566189e-11 1.57605630124247e-10 100
"chr9" 30759001 30760000 "*" 0 0 -100
"chr9" 32010001 32011000 "*" 0 0 100
"chr9" 32520001 32521000 "*" 1.15345036093562e-05 1.25186825636811e-05
-67.741935483871
"chr9" 32707001 32708000 "*" 6.52733422867868e-12 1.71146951718423e-11 100
"chr9" 33000001 33001000 "*" 4.21884749357559e-15 1.63874445239189e-14 100
"chr9" 33044001 33045000 "*" 0 0 -68.2465618860511
"chr9" 33167001 33168000 "*" 0 0 -89.413988657845
"chr9" 33404001 33405000 "*" 5.17363929475323e-14 1.76317882907907e-13
-55.2631578947368
"chr9" 33523001 33524000 "*" 0 0 63.8134592680047
"chr9" 33625001 33626000 "*" 1.12860387702085e-10 2.50016866623037e-10
88.0281690140845
"chr9" 33676001 33677000 "*" 0 0 87.1463831403076

"chr9" 33734001 33735000 "*" 8.7349629751543e-09 1.47505871898511e-08 -100
"chr9" 33750001 33751000 "*" 0 0 75.7085020242915
"chr9" 34091001 34092000 "*" 3.52704532247117e-11 8.35581292290093e-11 -100
"chr9" 34379001 34380000 "*" 0 0 100
"chr9" 34420001 34421000 "*" 1.13140401492018e-08 1.87801380787728e-08 -100
"chr9" 34459001 34460000 "*" 1.07882591748876e-11 2.74553537455803e-11 100
"chr9" 34514001 34515000 "*" 4.26951363152739e-10 8.72510873516957e-10 100
"chr9" 34589001 34590000 "*" 0 0 50.9192742034249
"chr9" 34611001 34612000 "*" 4.04565270173407e-13 1.23430734204696e-12
54.5454545454545
"chr9" 34629001 34630000 "*" 1.18582498265241e-07 1.70291738475944e-07
-73.2142857142857
"chr9" 34636001 34637000 "*" 2.53130849614536e-14 8.95494773377891e-14 70
"chr9" 34653001 34654000 "*" 0 0 61.4035087719298
"chr9" 34654001 34655000 "*" 4.82225370745937e-12 1.2862329543521e-11
53.6585365853659
"chr9" 34958001 34959000 "*" 0 0 63.3146623647278
"chr9" 34981001 34982000 "*" 4.44089209850063e-15 1.71914916534614e-14 -100
"chr9" 34997001 34998000 "*" 2.52020626589911e-13 7.89493892812164e-13
66.6666666666667
"chr9" 35102001 35103000 "*" 0 0 -100
"chr9" 35610001 35611000 "*" 6.66133814775094e-15 2.52791665956799e-14 -85
"chr9" 35660001 35661000 "*" 5.51532945980426e-08 8.32110440175318e-08 -52
"chr9" 35673001 35674000 "*" 5.76205749780456e-13 1.72515655979454e-12
61.7283950617284
"chr9" 35748001 35749000 "*" 0 0 -100
"chr9" 35848001 35849000 "*" 1.12055920098442e-11 2.84500040664577e-11 -100
"chr9" 35859001 35860000 "*" 6.09707967607065e-05 5.91541255978299e-05 59.375
"chr9" 35861001 35862000 "*" 0 0 -100
"chr9" 35864001 35865000 "*" 1.25551145528924e-05 1.35497076198712e-05
-52.1739130434783
"chr9" 35914001 35915000 "*" 5.87852363453933e-08 8.83677237745202e-08 -75
"chr9" 35961001 35962000 "*" 3.99665316841435e-06 4.64666236277004e-06
58.6206896551724
"chr9" 36012001 36013000 "*" 1.29142809535132e-06 1.61507322514618e-06
63.855421686747
"chr9" 36037001 36038000 "*" 0 0 90.625
"chr9" 36135001 36136000 "*" 9.10727781167076e-06 1.00428848699875e-05
62.7906976744186
"chr9" 36136001 36137000 "*" 0 0 56.7276422764228
"chr9" 36137001 36138000 "*" 4.594378211209e-10 9.3557560542343e-10
51.4173998044966
"chr9" 36147001 36148000 "*" 1.11022302462516e-16 5.03662826618488e-16 100
"chr9" 36164001 36165000 "*" 5.08482145278322e-14 1.73432706170696e-13 -100
"chr9" 36165001 36166000 "*" 8.10795874883752e-13 2.38732360337017e-12
-89.5833333333333
"chr9" 36194001 36195000 "*" 2.91028312560115e-11 6.97590507139978e-11 100
"chr9" 36210001 36211000 "*" 9.99200722162641e-16 4.15382497462808e-15 -100
"chr9" 36258001 36259000 "*" 0 0 -53.3513291239985
"chr9" 36486001 36487000 "*" 0 0 67.7368927368927
"chr9" 36567001 36568000 "*" 5.81211284256256e-06 6.59969314258037e-06
69.6428571428571

"chr9" 36744001 36745000 "*" 0.000176914317124854 0.000159330245150163
-58.0645161290323
"chr9" 36746001 36747000 "*" 1.22507998810972e-05 1.32404427866807e-05
-57.8947368421053
"chr9" 36748001 36749000 "*" 1.74853465040314e-11 4.32752710900609e-11 -100
"chr9" 36761001 36762000 "*" 0 0 67.2727272727273
"chr9" 36765001 36766000 "*" 0 0 65.9574468085106
"chr9" 36771001 36772000 "*" 1.22457599616155e-12 3.52441478267078e-12 -100
"chr9" 36791001 36792000 "*" 0.000118603937450446 0.000109926776654688
61.5384615384615
"chr9" 36797001 36798000 "*" 0 0 76.5625
"chr9" 36803001 36804000 "*" 2.93914359418279e-05 2.99757412716435e-05
-59.4594594594595
"chr9" 36840001 36841000 "*" 3.84532217179157e-09 6.87273748366896e-09
-82.0512820512821
"chr9" 36845001 36846000 "*" 1.12458486967171e-09 2.15740815043925e-09 -100
"chr9" 36957001 36958000 "*" 1.59227548923724e-08 2.59658003984163e-08
-52.3809523809524
"chr9" 36970001 36971000 "*" 1.96644922567657e-11 4.82930612417392e-11 100
"chr9" 36985001 36986000 "*" 1.03229647052672e-11 2.63704499881421e-11
77.1739130434783
"chr9" 36995001 36996000 "*" 4.20620119401249e-07 5.61396948445937e-07 -55
"chr9" 37034001 37035000 "*" 0 0 72.1917198397388
"chr9" 37036001 37037000 "*" 0 0 58.8993178209158
"chr9" 37173001 37174000 "*" 4.44089209850063e-16 1.91071758245033e-15 85
"chr9" 37371001 37372000 "*" 7.82707232360735e-14 2.61332834389318e-13 57.5
"chr9" 37388001 37389000 "*" 4.26951363152739e-10 8.72510873516957e-10 100
"chr9" 37391001 37392000 "*" 4.27032853522746e-11 9.99995440481693e-11 -75
"chr9" 37392001 37393000 "*" 9.04570973681018e-10 1.76414389822173e-09 100
"chr9" 37411001 37412000 "*" 0 0 -100
"chr9" 37485001 37486000 "*" 0 0 86.8170426065163
"chr9" 37497001 37498000 "*" 2.07846326460981e-05 2.16960943332502e-05
52.6315789473684
"chr9" 37528001 37529000 "*" 1.34559030584569e-13 4.35581312463895e-13 -100
"chr9" 37625001 37626000 "*" 3.6700841921089e-08 5.65530226072258e-08 -100
"chr9" 37652001 37653000 "*" 4.14335232790108e-13 1.26153063645578e-12 -100
"chr9" 37728001 37729000 "*" 4.65637306490407e-10 9.46720524046618e-10 100
"chr9" 37943001 37944000 "*" 1.38590361409285e-10 3.02706178892116e-10 -100
"chr9" 37959001 37960000 "*" 4.99020824662466e-11 1.15790792586157e-10 100
"chr9" 37965001 37966000 "*" 2.69084754478399e-12 7.41487347763769e-12 -100
"chr9" 37969001 37970000 "*" 1.12458486967171e-09 2.15740815043925e-09 100
"chr9" 37988001 37989000 "*" 4.02167188440217e-12 1.08266700497926e-11 100
"chr9" 38058001 38059000 "*" 1.0769163338864e-14 3.98631098593833e-14
-71.7391304347826
"chr9" 38081001 38082000 "*" 5.87285109432401e-10 1.17746015305031e-09
-61.1111111111111
"chr9" 38091001 38092000 "*" 1.18804934778893e-07 1.70575925561201e-07
-61.5384615384615
"chr9" 38157001 38158000 "*" 7.51824682239111e-05 7.18663852855192e-05
53.8461538461538
"chr9" 38304001 38305000 "*" 0 0 -100
"chr9" 38336001 38337000 "*" 2.88710388929303e-10 6.03399264731139e-10 -100

"chr9" 38343001 38344000 "*" 5.84981729834233e-07 7.67032732489292e-07 62.5
"chr9" 38362001 38363000 "*" 0 0 100
"chr9" 38382001 38383000 "*" 2.79440914852103e-11 6.73046092948135e-11 100
"chr9" 38445001 38446000 "*" 2.17381668221606e-13 6.85151076607736e-13 100
"chr9" 38569001 38570000 "*" 4.99900121297969e-12 1.32921056339624e-11 100
"chr9" 38621001 38622000 "*" 1.85561011001312e-09 3.46411139292915e-09
58.5883681535855
"chr9" 38650001 38651000 "*" 1.16588069077883e-05 1.26437197277731e-05
63.1578947368421
"chr9" 38687001 38688000 "*" 1.11022302462516e-16 5.03662826618488e-16
-56.8840579710145
"chr9" 38693001 38694000 "*" 0 0 100
"chr9" 38696001 38697000 "*" 3.33066907387547e-16 1.4495649018245e-15 100
"chr9" 39132001 39133000 "*" 0 0 56.4293632220717
"chr9" 40633001 40634000 "*" 0 0 95.3551912568306
"chr9" 43863001 43864000 "*" 6.98330282489223e-14 2.34267456154807e-13 100
"chr9" 44227001 44228000 "*" 0 0 64.6188340807175
"chr9" 44245001 44246000 "*" 0 0 100
"chr9" 46755001 46756000 "*" 0 0 -100
"chr9" 46756001 46757000 "*" 1.75842710093965e-05 1.8573207420057e-05
57.5757575757576
"chr9" 66563001 66564000 "*" 1.99917860044252e-12 5.5967536720422e-12
71.4285714285714
"chr9" 66836001 66837000 "*" 0 0 -100
"chr9" 67339001 67340000 "*" 0 0 53.0769230769231
"chr9" 69065001 69066000 "*" 0 0 -51.9340863376141
"chr9" 69198001 69199000 "*" 1.24885501939787e-09 2.38390924977424e-09
54.2452830188679
"chr9" 69785001 69786000 "*" 0 0 60
"chr9" 70651001 70652000 "*" 0 0 61.9631901840491
"chr9" 71096001 71097000 "*" 1.39779633423487e-07 1.98463037958589e-07 -100
"chr9" 71320001 71321000 "*" 0 0 51.6863406408094
"chr9" 71420001 71421000 "*" 1.70641278884887e-12 4.81986032810646e-12 100
"chr9" 71426001 71427000 "*" 2.08939601620273e-07 2.90467202288664e-07
-90.9090909090909
"chr9" 71443001 71444000 "*" 5.412610470934e-09 9.4389019488003e-09
-82.089552238806
"chr9" 71594001 71595000 "*" 1.67299207820548e-08 2.71377429441055e-08 -100
"chr9" 71689001 71690000 "*" 9.63829016598083e-12 2.46993429847588e-11 -100
"chr9" 71736001 71737000 "*" 0 0 -83.3756345177665
"chr9" 71780001 71781000 "*" 1.07882591748876e-11 2.74553537455803e-11 100
"chr9" 71881001 71882000 "*" 5.51780843238703e-14 1.87243792572872e-13 -100
"chr9" 71950001 71951000 "*" 1.12055920098442e-11 2.84500040664577e-11 -100
"chr9" 71977001 71978000 "*" 0 0 100
"chr9" 72024001 72025000 "*" 2.88710388929303e-10 6.03399264731139e-10 100
"chr9" 72042001 72043000 "*" 6.3890279733414e-09 1.10435105893381e-08
81.4814814814815
"chr9" 72115001 72116000 "*" 4.03721420738634e-08 6.1932142423158e-08
62.962962962963
"chr9" 72207001 72208000 "*" 2.88779000712225e-11 6.92935809466068e-11 100
"chr9" 72221001 72222000 "*" 1.60310431596145e-10 3.47435209361035e-10 75
"chr9" 72715001 72716000 "*" 2.33028818463765e-10 4.93719283454501e-10 100

"chr9" 73033001 73034000 "*" 2.82773804372027e-13 8.79129664109438e-13 -100
"chr9" 73038001 73039000 "*" 3.6700841921089e-08 5.65530226072258e-08 -100
"chr9" 73081001 73082000 "*" 2.22044604925031e-16 9.81641919380259e-16 100
"chr9" 73219001 73220000 "*" 2.33028818463765e-10 4.93719283454501e-10 -100
"chr9" 73468001 73469000 "*" 4.79394346442064e-09 8.4097903736489e-09
57.6923076923077
"chr9" 73641001 73642000 "*" 3.25341350576602e-05 3.2954838151491e-05
60.8695652173913
"chr9" 74061001 74062000 "*" 0 0 58.8820592066981
"chr9" 74062001 74063000 "*" 5.84277071169481e-12 1.54074414136491e-11
86.0082304526749
"chr9" 74652001 74653000 "*" 2.08814465718632e-09 3.86232567960463e-09 -100
"chr9" 75060001 75061000 "*" 8.7349629751543e-09 1.47505871898511e-08 100
"chr9" 75075001 75076000 "*" 6.15840711759574e-13 1.83676803572802e-12 -100
"chr9" 75087001 75088000 "*" 4.78841410966879e-11 1.11468187156588e-10 100
"chr9" 75200001 75201000 "*" 1.11022302462516e-16 5.03662826618488e-16 100
"chr9" 77566001 77567000 "*" 1.11022302462516e-16 5.03662826618488e-16
62.8930817610063
"chr9" 77711001 77712000 "*" 2.00227675550835e-08 3.20459176617961e-08 100
"chr9" 78035001 78036000 "*" 5.55111512312578e-16 2.36485094870365e-15 100
"chr9" 78159001 78160000 "*" 9.08108033215171e-11 2.03407753381119e-10 -100
"chr9" 78195001 78196000 "*" 8.7349629751543e-09 1.47505871898511e-08 -100
"chr9" 78506001 78507000 "*" 0 0 65.9685863874346
"chr9" 78577001 78578000 "*" 4.01313771103418e-09 7.11882087237587e-09 -100
"chr9" 78775001 78776000 "*" 6.60582699651968e-14 2.22176301799e-13 -100
"chr9" 78911001 78912000 "*" 1.14124265593318e-11 2.89138062669327e-11 -100
"chr9" 78936001 78937000 "*" 4.48406133335411e-08 6.84482503893299e-08
66.6666666666667
"chr9" 79069001 79070000 "*" 9.0072393987839e-13 2.63157725646847e-12 -100
"chr9" 79197001 79198000 "*" 6.312023126398e-09 1.09176475181558e-08 -75
"chr9" 79323001 79324000 "*" 0 0 -95.9183673469388
"chr9" 79504001 79505000 "*" 1.9373391779709e-13 6.15971322898373e-13 -100
"chr9" 79521001 79522000 "*" 1.68428537783427e-09 3.15848752995564e-09
62.0833333333333
"chr9" 79629001 79630000 "*" 0 0 51.6184405857281
"chr9" 79636001 79637000 "*" 5.55111512312578e-15 2.12516350804551e-14 100
"chr9" 79655001 79656000 "*" 3.92685883809918e-13 1.19951200966592e-12 100
"chr9" 79662001 79663000 "*" 2.33028818463765e-10 4.93719283454501e-10 100
"chr9" 80263001 80264000 "*" 0 0 51.2068965517241
"chr9" 80272001 80273000 "*" 4.09955402957962e-11 9.61516531206922e-11 82.8125
"chr9" 81116001 81117000 "*" 4.42079706175491e-12 1.18476078442142e-11
53.4883720930233
"chr9" 81232001 81233000 "*" 1.5277158427196e-09 2.87642564232045e-09 100
"chr9" 81310001 81311000 "*" 1.88737914186277e-15 7.62011598380321e-15 100
"chr9" 81349001 81350000 "*" 0 0 100
"chr9" 81651001 81652000 "*" 4.77395900588817e-15 1.84201484337652e-14 -100
"chr9" 81972001 81973000 "*" 4.73234496034536e-09 8.30627300264826e-09 100
"chr9" 82006001 82007000 "*" 5.3179682879545e-13 1.5994888444522e-12
72.972972972973
"chr9" 82185001 82186000 "*" 0 0 60.1626794258373
"chr9" 82859001 82860000 "*" 0 0 100
"chr9" 84179001 84180000 "*" 4.01313771103418e-09 7.11882087237587e-09 100

"chr9" 84746001 84747000 "*" 3.95353005888666e-09 7.03928529283733e-09 100
"chr9" 84796001 84797000 "*" 2.69084754478399e-12 7.41487347763769e-12 -100
"chr9" 85072001 85073000 "*" 8.7349629751543e-09 1.47505871898511e-08 100
"chr9" 85539001 85540000 "*" 4.08209022140227e-11 9.57770690255255e-11 100
"chr9" 86153001 86154000 "*" 0 0 60.7583950049703
"chr9" 86237001 86238000 "*" 0 0 -81.8230377225021
"chr9" 86238001 86239000 "*" 0 0 -62.32741617357
"chr9" 86343001 86344000 "*" 6.71551103614831e-08 1.00155272781685e-07 75
"chr9" 86785001 86786000 "*" 5.1281201507436e-13 1.54461625452838e-12 -100
"chr9" 86868001 86869000 "*" 2.00227675550835e-08 3.20459176617961e-08 100
"chr9" 87024001 87025000 "*" 9.13491504661579e-13 2.66747761509305e-12 67.5
"chr9" 87283001 87284000 "*" 0 0 73.1075697211155
"chr9" 87339001 87340000 "*" 5.63660229602192e-13 1.68905058935478e-12 100
"chr9" 87913001 87914000 "*" 4.32209823486573e-12 1.15935290479641e-11 -100
"chr9" 87999001 88000000 "*" 3.40340631888036e-06 3.99960927822812e-06
66.6666666666667
"chr9" 88055001 88056000 "*" 0.000244506761165386 0.000215083158896703
53.030303030303
"chr9" 88120001 88121000 "*" 1.89848137210902e-14 6.82212372863451e-14
63.265306122449
"chr9" 88125001 88126000 "*" 6.56767396023383e-09 1.1334582616219e-08
-58.9285714285714
"chr9" 88543001 88544000 "*" 0 0 100
"chr9" 88644001 88645000 "*" 1.25284005392245e-10 2.75649590272477e-10 100
"chr9" 89279001 89280000 "*" 8.58968451922237e-12 2.213554983903e-11 100
"chr9" 89403001 89404000 "*" 0 0 -90.625
"chr9" 89424001 89425000 "*" 2.79617118348341e-09 5.07591201160347e-09
57.6923076923077
"chr9" 89433001 89434000 "*" 1.98325800226939e-11 4.85612402473442e-11 -100
"chr9" 89457001 89458000 "*" 1.37828859436695e-10 3.01445544267661e-10 100
"chr9" 89626001 89627000 "*" 1.64424029946986e-12 4.65275971097102e-12
56.3106796116505
"chr9" 89656001 89657000 "*" 1.11022302462516e-16 5.03662826618488e-16
73.1707317073171
"chr9" 89763001 89764000 "*" 0 0 88.0318868123746
"chr9" 89866001 89867000 "*" 8.29875134278346e-07 1.06514665656035e-06
54.0983606557377
"chr9" 90110001 90111000 "*" 3.31690230837012e-12 9.03347040781321e-12 100
"chr9" 90207001 90208000 "*" 0 0 100
"chr9" 90278001 90279000 "*" 0 0 93.75
"chr9" 90285001 90286000 "*" 4.32098801184111e-13 1.31226207772651e-12
54.3859649122807
"chr9" 90340001 90341000 "*" 0 0 56.1710760568045
"chr9" 90414001 90415000 "*" 1.07882591748876e-11 2.74553537455803e-11 -100
"chr9" 90443001 90444000 "*" 0 0 88.1188118811881
"chr9" 90449001 90450000 "*" 5.11120712420876e-09 8.9353393949628e-09
86.0215053763441
"chr9" 90483001 90484000 "*" 1.59857761472537e-07 2.25389652468643e-07
-78.4313725490196
"chr9" 90501001 90502000 "*" 7.20645765284189e-13 2.13076041737731e-12
-66.6666666666667
"chr9" 90629001 90630000 "*" 9.80679292184039e-06 1.0765012631704e-05

-55.1724137931034
"chr9" 90631001 90632000 "*" 0 0 -100
"chr9" 90936001 90937000 "*" 5.10702591327572e-15 1.96271840288234e-14 100
"chr9" 91138001 91139000 "*" 1.48487888651516e-11 3.70689457767456e-11 100
"chr9" 91149001 91150000 "*" 0 0 90.512174643157
"chr9" 91151001 91152000 "*" 1.11022302462516e-16 5.03662826618488e-16
66.6666666666667
"chr9" 91155001 91156000 "*" 1.67299207820548e-08 2.71377429441055e-08 -100
"chr9" 91193001 91194000 "*" 0 0 72.6256983240224
"chr9" 91287001 91288000 "*" 6.04438721296674e-11 1.38849255306602e-10 100
"chr9" 91319001 91320000 "*" 1.39779633423487e-07 1.98463037958589e-07 100
"chr9" 91366001 91367000 "*" 1.11022302462516e-16 5.03662826618488e-16 100
"chr9" 91369001 91370000 "*" 1.77302617032637e-13 5.66948923973361e-13 -60
"chr9" 91393001 91394000 "*" 7.62047056568349e-08 1.12250756179915e-07
52.3809523809524
"chr9" 91442001 91443000 "*" 4.32209823486573e-12 1.15935290479641e-11 100
"chr9" 91586001 91587000 "*" 2.57577514872764e-10 5.43480260685379e-10
86.8055555555556
"chr9" 91592001 91593000 "*" 7.03992419914812e-12 1.83313429961219e-11 100
"chr9" 91606001 91607000 "*" 0 0 50.6897689830751
"chr9" 91612001 91613000 "*" 1.07864652044709e-05 1.17574627643031e-05
-63.8888888888889
"chr9" 91684001 91685000 "*" 1.07882591748876e-11 2.74553537455803e-11 100
"chr9" 91698001 91699000 "*" 0 0 100
"chr9" 91759001 91760000 "*" 4.26951363152739e-10 8.72510873516957e-10 -100
"chr9" 91793001 91794000 "*" 0 0 50.193386947092
"chr9" 91794001 91795000 "*" 8.7349629751543e-09 1.47505871898511e-08 -100
"chr9" 92050001 92051000 "*" 3.99264177275427e-10 8.19342798154651e-10
86.046511627907
"chr9" 92137001 92138000 "*" 4.65637306490407e-10 9.46720524046618e-10 100
"chr9" 92153001 92154000 "*" 0 0 -56.5811965811966
"chr9" 92157001 92158000 "*" 1.84337545228175e-10 3.97370822448571e-10
82.7160493827161
"chr9" 92174001 92175000 "*" 2.51931808747941e-12 6.96891822169638e-12
-54.0540540540541
"chr9" 92210001 92211000 "*" 2.02046190989069e-06 2.45659870375847e-06
60.8695652173913
"chr9" 92219001 92220000 "*" 6.9798838930879e-09 1.19729983844322e-08
59.5505617977528
"chr9" 92242001 92243000 "*" 3.29002870835815e-07 4.4572162136317e-07
-50.8196721311475
"chr9" 92255001 92256000 "*" 4.14335232790108e-13 1.26153063645578e-12 -100
"chr9" 92262001 92263000 "*" 6.34673771315875e-08 9.49423067802588e-08
-74.4186046511628
"chr9" 92271001 92272000 "*" 0 0 100
"chr9" 92302001 92303000 "*" 1.12634901405784e-10 2.49563754426169e-10 100
"chr9" 92310001 92311000 "*" 0 0 -100
"chr9" 92333001 92334000 "*" 1.39779633423487e-07 1.98463037958589e-07 -100
"chr9" 92490001 92491000 "*" 4.99900121297969e-12 1.32921056339624e-11 100
"chr9" 92519001 92520000 "*" 2.15125472990962e-10 4.58126659943874e-10 100
"chr9" 92689001 92690000 "*" 3.95353005888666e-09 7.03928529283733e-09 100
"chr9" 92694001 92695000 "*" 1.83468527426012e-06 2.24475948937626e-06

66.6666666666667
"chr9" 92780001 92781000 "*" 5.8877959673298e-06 6.67999302531187e-06
71.7948717948718
"chr9" 92974001 92975000 "*" 6.4152538836737e-10 1.27477697728417e-09 100
"chr9" 92982001 92983000 "*" 1.9373391779709e-13 6.15971322898373e-13 -100
"chr9" 93145001 93146000 "*" 2.49750220504552e-11 6.05592163349763e-11
66.6666666666667
"chr9" 93229001 93230000 "*" 1.29037891483108e-11 3.24582280286866e-11 75
"chr9" 93398001 93399000 "*" 1.13140401492018e-08 1.87801380787728e-08 100
"chr9" 93404001 93405000 "*" 1.6495804722183e-11 4.09586951354932e-11
81.1320754716981
"chr9" 93456001 93457000 "*" 3.6700841921089e-08 5.65530226072258e-08 -100
"chr9" 93490001 93491000 "*" 2.22044604925031e-16 9.81641919380259e-16 100
"chr9" 93623001 93624000 "*" 2.7739215369138e-09 5.03716664165354e-09
-93.3333333333333
"chr9" 93628001 93629000 "*" 0.0003207101954813 0.000276619581787338
54.5454545454545
"chr9" 93683001 93684000 "*" 6.32381944215554e-05 6.12087074932446e-05
57.8947368421053
"chr9" 93703001 93704000 "*" 1.48087875295744e-10 3.22075573380618e-10 -100
"chr9" 93801001 93802000 "*" 2.00227675550835e-08 3.20459176617961e-08 100
"chr9" 93927001 93928000 "*" 2.02060590481778e-13 6.40127405465654e-13 -100
"chr9" 93939001 93940000 "*" 1.00430321503531e-07 1.457043649779e-07
52.9411764705882
"chr9" 93953001 93954000 "*" 6.59550958292954e-09 1.13456194382773e-08 100
"chr9" 93955001 93956000 "*" 0 0 -75
"chr9" 94005001 94006000 "*" 4.13329948045771e-09 7.31905277636459e-09 -80
"chr9" 94162001 94163000 "*" 2.02060590481778e-13 6.40127405465654e-13 100
"chr9" 94341001 94342000 "*" 1.39779633423487e-07 1.98463037958589e-07 100
"chr9" 94496001 94497000 "*" 6.00539917794407e-08 9.0164445268462e-08
51.1111111111111
"chr9" 94498001 94499000 "*" 7.0006535457523e-08 1.03695832512054e-07 100
"chr9" 94517001 94518000 "*" 1.55431223447522e-15 6.33705141101682e-15
96.1538461538462
"chr9" 94518001 94519000 "*" 0 0 -100
"chr9" 94564001 94565000 "*" 1.83186799063151e-14 6.59696304569048e-14 100
"chr9" 94581001 94582000 "*" 0 0 71.7171717171717
"chr9" 94600001 94601000 "*" 0 0 -100
"chr9" 94609001 94610000 "*" 2.88657986402541e-15 1.14155613124573e-14 100
"chr9" 94653001 94654000 "*" 6.1899748649008e-07 8.09014320124164e-07
79.1304347826087
"chr9" 94665001 94666000 "*" 3.6700841921089e-08 5.65530226072258e-08 100
"chr9" 94667001 94668000 "*" 1.4432899320127e-15 5.90750815956055e-15 100
"chr9" 94677001 94678000 "*" 3.31690230837012e-12 9.03347040781321e-12 -100
"chr9" 94698001 94699000 "*" 2.80906020666549e-09 5.09831082576912e-09
78.0821917808219
"chr9" 94703001 94704000 "*" 7.04690853067236e-05 6.76680270782408e-05 -59.375
"chr9" 94712001 94713000 "*" 0 0 55.7354642363547
"chr9" 94728001 94729000 "*" 2.1094237467878e-15 8.47434540879246e-15 100
"chr9" 94829001 94830000 "*" 6.24654217240561e-10 1.2462108191303e-09 100
"chr9" 94880001 94881000 "*" 7.21644966006352e-15 2.72345776413425e-14 100
"chr9" 94899001 94900000 "*" 3.95353005888666e-09 7.03928529283733e-09 -100

"chr9" 94901001 94902000 "*" 0 0 100
"chr9" 94923001 94924000 "*" 1.35003119794419e-13 4.3679847476192e-13 100
"chr9" 94933001 94934000 "*" 2.06899765957669e-05 2.16032359107815e-05 60.6060606060606
"chr9" 94935001 94936000 "*" 6.17297194471789e-08 9.25038473746934e-08 -58.6206896551724
"chr9" 95007001 95008000 "*" 0 0 100
"chr9" 95055001 95056000 "*" 0 0 -100
"chr9" 95277001 95278000 "*" 8.45523651094027e-11 1.90381376365023e-10 -68.8524590163934
"chr9" 95415001 95416000 "*" 2.76320482939241e-07 3.78207553610291e-07 76.1904761904762
"chr9" 95499001 95500000 "*" 0 0 -100
"chr9" 95526001 95527000 "*" 0 0 -100
"chr9" 95571001 95572000 "*" 0 0 59.0163049346723
"chr9" 95721001 95722000 "*" 0 0 -100
"chr9" 95722001 95723000 "*" 9.80260317362536e-12 2.51042171834956e-11 89.051094890511
"chr9" 95778001 95779000 "*" 9.61897228535236e-13 2.801372325071e-12 -100
"chr9" 95801001 95802000 "*" 1.40781175606008e-09 2.67223111923349e-09 -84.8484848484848
"chr9" 95817001 95818000 "*" 3.80408857991732e-06 4.43681931698391e-06 -53.8461538461538
"chr9" 95836001 95837000 "*" 8.01666433236647e-09 1.36392368970109e-08 100
"chr9" 95856001 95857000 "*" 0 0 100
"chr9" 95933001 95934000 "*" 1.56863411149288e-12 4.4469621057462e-12 100
"chr9" 95970001 95971000 "*" 1.33226762955019e-15 5.48133041560118e-15 52.6338514680484
"chr9" 95974001 95975000 "*" 2.17381668221606e-13 6.85151076607736e-13 100
"chr9" 96000001 96001000 "*" 5.10702591327572e-15 1.96271840288234e-14 -100
"chr9" 96104001 96105000 "*" 1.48534020527613e-07 2.10202108083997e-07 73.9130434782609
"chr9" 96179001 96180000 "*" 2.08814465718632e-09 3.86232567960463e-09 -100
"chr9" 96212001 96213000 "*" 0 0 -100
"chr9" 96329001 96330000 "*" 0 0 62.9429892141757
"chr9" 96354001 96355000 "*" 3.85705279803972e-06 4.49400519147012e-06 -68.75
"chr9" 96489001 96490000 "*" 1.11022302462516e-16 5.03662826618488e-16 100
"chr9" 96506001 96507000 "*" 6.59550958292954e-09 1.13456194382773e-08 -100
"chr9" 96592001 96593000 "*" 0 0 100
"chr9" 96626001 96627000 "*" 3.03090885722668e-14 1.06210046699299e-13 -67.741935483871
"chr9" 96679001 96680000 "*" 0 0 -63.6363636363636
"chr9" 96686001 96687000 "*" 0.000230459847340714 0.000203712858281793 66.6666666666667
"chr9" 96695001 96696000 "*" 0 0 53.9043381535039
"chr9" 96707001 96708000 "*" 0 0 -79.5918367346939
"chr9" 96708001 96709000 "*" 0 0 66.1213179471606
"chr9" 96730001 96731000 "*" 3.44613226843649e-13 1.06294549738291e-12 -58.3333333333333
"chr9" 97356001 97357000 "*" 9.04570973681018e-10 1.76414389822173e-09 100
"chr9" 97403001 97404000 "*" 4.10773637327111e-11 9.63381447368142e-11 60
"chr9" 97581001 97582000 "*" 3.77475828372553e-13 1.15780027124727e-12

-78.8461538461538
"chr9" 97588001 97589000 "*" 1.67299207820548e-08 2.71377429441055e-08 100
"chr9" 97611001 97612000 "*" 3.90465437760668e-13 1.19307893943277e-12 -100
"chr9" 97679001 97680000 "*" 0 0 -58.4905660377358
"chr9" 97810001 97811000 "*" 0 0 -93.4707903780069
"chr9" 97842001 97843000 "*" 1.08824060873758e-11 2.7685932902032e-11
74.2857142857143
"chr9" 97889001 97890000 "*" 0 0 77.5
"chr9" 97907001 97908000 "*" 1.57263091438153e-10 3.41078988754126e-10
-95.4545454545455
"chr9" 97961001 97962000 "*" 0 0 100
"chr9" 98145001 98146000 "*" 1.02875828966731e-08 1.72239045994312e-08 -80
"chr9" 98197001 98198000 "*" 3.4909608537248e-10 7.21575902470817e-10
-56.4102564102564
"chr9" 98210001 98211000 "*" 0 0 -100
"chr9" 98267001 98268000 "*" 1.81841097735003e-10 3.92141342732429e-10
58.5365853658537
"chr9" 98323001 98324000 "*" 3.89590918858929e-07 5.22306324903137e-07 -71.875
"chr9" 98357001 98358000 "*" 4.44089209850063e-15 1.71914916534614e-14 -100
"chr9" 98362001 98363000 "*" 1.20591092667155e-10 2.66075324129686e-10 100
"chr9" 98377001 98378000 "*" 2.16648032846933e-09 4.00030927571467e-09
-88.7323943661972
"chr9" 98847001 98848000 "*" 1.11022302462516e-16 5.03662826618488e-16 -60
"chr9" 98860001 98861000 "*" 0 0 -100
"chr9" 99010001 99011000 "*" 2.38229547200319e-10 5.04200128794648e-10
66.6666666666667
"chr9" 99023001 99024000 "*" 2.51935901984801e-06 3.01947631270519e-06
52.6315789473684
"chr9" 99051001 99052000 "*" 7.60806473376618e-09 1.29952260304067e-08 -53.125
"chr9" 99139001 99140000 "*" 0 0 100
"chr9" 99271001 99272000 "*" 1.29037891483108e-11 3.24582280286866e-11 -100
"chr9" 99390001 99391000 "*" 6.4324989779152e-11 1.47230020825092e-10 100
"chr9" 99416001 99417000 "*" 2.15125472990962e-10 4.58126659943874e-10 100
"chr9" 99428001 99429000 "*" 8.7349629751543e-09 1.47505871898511e-08 -100
"chr9" 99435001 99436000 "*" 1.37828859436695e-10 3.01445544267661e-10 100
"chr9" 99487001 99488000 "*" 3.6700841921089e-08 5.65530226072258e-08 100
"chr9" 99540001 99541000 "*" 0 0 -57.5350219329277
"chr9" 99800001 99801000 "*" 0 0 53.4335261455713
"chr9" 99884001 99885000 "*" 3.17763334312104e-07 4.31279330063049e-07
-51.7241379310345
"chr9" 99999001 100000000 "*" 1.20543575121701e-11 3.04593655104189e-11 75
"chr9" 100086001 100087000 "*" 3.05311331771918e-12 8.34639368772922e-12 100
"chr9" 100117001 100118000 "*" 6.4152538836737e-10 1.27477697728417e-09 -100
"chr9" 100264001 100265000 "*" 0 0 56.0056523787094
"chr9" 100268001 100269000 "*" 7.0006535457523e-08 1.03695832512054e-07 100
"chr9" 100284001 100285000 "*" 0 0 66.6666666666667
"chr9" 100294001 100295000 "*" 5.80312464748545e-11 1.33619900113832e-10 65.625
"chr9" 100317001 100318000 "*" 2.08814465718632e-09 3.86232567960463e-09 100
"chr9" 100330001 100331000 "*" 3.6700841921089e-08 5.65530226072258e-08 100
"chr9" 100426001 100427000 "*" 8.7349629751543e-09 1.47505871898511e-08 -100
"chr9" 100481001 100482000 "*" 7.105427357601e-15 2.68362283258525e-14
97.8260869565217

"chr9" 100496001 100497000 "*" 7.0006535457523e-08 1.03695832512054e-07 100
"chr9" 100506001 100507000 "*" 2.73625566649116e-12 7.52483363579137e-12 -100
"chr9" 100524001 100525000 "*" 7.12463501140181e-06 7.98483559457126e-06 -52
"chr9" 100796001 100797000 "*" 0 0 78.4313725490196
"chr9" 100797001 100798000 "*" 1.5673525105564e-06 1.93725608775872e-06 70.4918032786885
"chr9" 100818001 100819000 "*" 0 0 -90
"chr9" 100891001 100892000 "*" 2.88779000712225e-11 6.92935809466068e-11 100
"chr9" 100925001 100926000 "*" 2.88710388929303e-10 6.03399264731139e-10 -100
"chr9" 100972001 100973000 "*" 0 0 -100
"chr9" 100977001 100978000 "*" 6.11886953216789e-06 6.92255677472338e-06 53.3333333333333
"chr9" 101051001 101052000 "*" 6.09507309701218e-06 6.8968637727707e-06 57.1428571428571
"chr9" 101063001 101064000 "*" 6.37490060739765e-13 1.89496793587877e-12 100
"chr9" 101080001 101081000 "*" 0 0 100
"chr9" 101137001 101138000 "*" 2.30129248990352e-11 5.5982206740486e-11 -66.6666666666667
"chr9" 101218001 101219000 "*" 2.25100731721106e-07 3.11620182818972e-07 62.962962962963
"chr9" 101246001 101247000 "*" 9.08108033215171e-11 2.03407753381119e-10 100
"chr9" 101268001 101269000 "*" 4.99900121297969e-12 1.32921056339624e-11 100
"chr9" 101299001 101300000 "*" 0 0 100
"chr9" 101304001 101305000 "*" 0 0 100
"chr9" 101314001 101315000 "*" 8.3882012447134e-11 1.88922989503423e-10 100
"chr9" 101366001 101367000 "*" 8.97060203897126e-14 2.96780698277594e-13 -60
"chr9" 101437001 101438000 "*" 5.6362137179633e-11 1.29963270300065e-10 100
"chr9" 101459001 101460000 "*" 1.22707844552039e-07 1.75940305659382e-07 -73.6842105263158
"chr9" 101510001 101511000 "*" 6.2400506872784e-08 9.34435715465956e-08 52.3809523809524
"chr9" 101569001 101570000 "*" 0 0 100
"chr9" 101665001 101666000 "*" 1.11022302462516e-16 5.03662826618488e-16 -100
"chr9" 101704001 101705000 "*" 2.1094237467878e-15 8.47434540879246e-15 100
"chr9" 101720001 101721000 "*" 3.27008975453368e-10 6.78798757234889e-10 66.6666666666667
"chr9" 101750001 101751000 "*" 1.11022302462516e-16 5.03662826618488e-16 100
"chr9" 101785001 101786000 "*" 0 0 99.0740740740741
"chr9" 101868001 101869000 "*" 0 0 100
"chr9" 101973001 101974000 "*" 0 0 95
"chr9" 102131001 102132000 "*" 0 0 94.4444444444444
"chr9" 102412001 102413000 "*" 1.11022302462516e-16 5.03662826618488e-16 100
"chr9" 102901001 102902000 "*" 0 0 100
"chr9" 103235001 103236000 "*" 0 0 52.8492840763964
"chr9" 103430001 103431000 "*" 7.0006535457523e-08 1.03695832512054e-07 -100
"chr9" 103493001 103494000 "*" 2.66453525910038e-15 1.05861327033776e-14 65
"chr9" 103494001 103495000 "*" 3.20085624672117e-10 6.65473184120888e-10 57.8341013824885
"chr9" 103673001 103674000 "*" 1.39779633423487e-07 1.98463037958589e-07 -100
"chr9" 104067001 104068000 "*" 1.18055857498689e-05 1.27950784223023e-05 -61.5384615384615
"chr9" 105659001 105660000 "*" 8.25450818808804e-13 2.42473843346228e-12 -100

"chr9" 106000001 106001000 "*" 3.21964677141295e-15 1.26720521345953e-14 -95
"chr9" 106867001 106868000 "*" 8.7349629751543e-09 1.47505871898511e-08 100
"chr9" 107564001 107565000 "*" 6.4152538836737e-10 1.27477697728417e-09 100
"chr9" 107582001 107583000 "*" 0 0 100
"chr9" 107754001 107755000 "*" 0 0 -100
"chr9" 107952001 107953000 "*" 3.38200641936126e-08 5.25577529078797e-08
-69.7674418604651
"chr9" 108564001 108565000 "*" 3.03979064142368e-13 9.42350883309283e-13 -100
"chr9" 108580001 108581000 "*" 2.00227675550835e-08 3.20459176617961e-08 100
"chr9" 108610001 108611000 "*" 1.60123900065923e-05 1.7012032608364e-05
56.6666666666667
"chr9" 108667001 108668000 "*" 4.01313771103418e-09 7.11882087237587e-09 -100
"chr9" 108820001 108821000 "*" 6.59550958292954e-09 1.13456194382773e-08 -100
"chr9" 108882001 108883000 "*" 0 0 -100
"chr9" 109263001 109264000 "*" 0 0 -100
"chr9" 109458001 109459000 "*" 1.4777862439308e-06 1.83350792496369e-06 -59.375
"chr9" 109459001 109460000 "*" 3.46280781826636e-10 7.16022674457106e-10
59.4594594594595
"chr9" 109622001 109623000 "*" 0 0 100
"chr9" 109626001 109627000 "*" 1.0902390101819e-13 3.57149508278892e-13
69.2307692307692
"chr9" 109687001 109688000 "*" 1.39680959354571e-07 1.98463037958589e-07
-66.6666666666667
"chr9" 109694001 109695000 "*" 0 0 100
"chr9" 109722001 109723000 "*" 8.25450818808804e-13 2.42473843346228e-12 100
"chr9" 109736001 109737000 "*" 4.07152078294448e-05 4.0578340745223e-05
-60.5263157894737
"chr9" 110120001 110121000 "*" 4.90718576884319e-14 1.6794399166207e-13 100
"chr9" 110134001 110135000 "*" 1.25284005392245e-10 2.75649590272477e-10 -100
"chr9" 110152001 110153000 "*" 2.22044604925031e-15 8.90449334476539e-15
91.6666666666667
"chr9" 110305001 110306000 "*" 8.7349629751543e-09 1.47505871898511e-08 100
"chr9" 110391001 110392000 "*" 7.88258347483861e-15 2.96145301916012e-14 100
"chr9" 110523001 110524000 "*" 1.37001521238744e-13 4.42851918810123e-13 100
"chr9" 110582001 110583000 "*" 9.83657599817889e-14 3.23479868318879e-13
68.4210526315789
"chr9" 110661001 110662000 "*" 7.03970176041402e-07 9.12522359725664e-07
58.3333333333333
"chr9" 110711001 110712000 "*" 2.22044604925031e-16 9.81641919380259e-16 100
"chr9" 110732001 110733000 "*" 4.01313771103418e-09 7.11882087237587e-09 100
"chr9" 111010001 111011000 "*" 0 0 -100
"chr9" 111153001 111154000 "*" 2.00227675550835e-08 3.20459176617961e-08 -100
"chr9" 111273001 111274000 "*" 8.06688049692639e-13 2.37556391305009e-12 100
"chr9" 111543001 111544000 "*" 0 0 100
"chr9" 111624001 111625000 "*" 7.58679341572588e-10 1.4946469944646e-09
-51.7241379310345
"chr9" 111919001 111920000 "*" 2.02327044007689e-12 5.65300384792621e-12 100
"chr9" 112076001 112077000 "*" 0 0 100
"chr9" 112085001 112086000 "*" 1.52794177310511e-09 2.87642564232045e-09 100
"chr9" 112238001 112239000 "*" 2.00227675550835e-08 3.20459176617961e-08 100
"chr9" 112246001 112247000 "*" 0 0 -56.5217391304348
"chr9" 112259001 112260000 "*" 0 0 71.4285714285714

"chr9" 112445001 112446000 "*" 0.0003207101954813 0.000276619581787338
-54.5454545454545
"chr9" 112551001 112552000 "*" 2.6165741273676e-06 3.12843692934624e-06
-66.6666666666667
"chr9" 112597001 112598000 "*" 2.1094237467878e-15 8.47434540879246e-15 100
"chr9" 112629001 112630000 "*" 1.74853465040314e-11 4.32752710900609e-11 100
"chr9" 112665001 112666000 "*" 0 0 100
"chr9" 112694001 112695000 "*" 1.51323398256409e-13 4.86995056237989e-13 -100
"chr9" 112811001 112812000 "*" 0 0 75.1243781094527
"chr9" 112812001 112813000 "*" 9.63829016598083e-12 2.46993429847588e-11 100
"chr9" 113553001 113554000 "*" 1.37828859436695e-10 3.01445544267661e-10 100
"chr9" 114285001 114286000 "*" 1.87294624254264e-13 5.97285292681429e-13
95.6521739130435
"chr9" 114299001 114300000 "*" 9.80763359414993e-10 1.8980032424715e-09 100
"chr9" 114550001 114551000 "*" 2.82773804372027e-13 8.79129664109438e-13 -100
"chr9" 114645001 114646000 "*" 5.55111512312578e-16 2.36485094870365e-15 100
"chr9" 114958001 114959000 "*" 5.01025332333427e-10 1.01358031135889e-09 -100
"chr9" 114962001 114963000 "*" 2.02060590481778e-13 6.40127405465654e-13 -100
"chr9" 115110001 115111000 "*" 2.81293877080202e-11 6.77313433663339e-11
89.8305084745763
"chr9" 115519001 115520000 "*" 2.22044604925031e-16 9.81641919380259e-16
66.6666666666667
"chr9" 115851001 115852000 "*" 0 0 50.3176930596285
"chr9" 115998001 115999000 "*" 7.0006535457523e-08 1.03695832512054e-07 -100
"chr9" 116061001 116062000 "*" 1.96453964207421e-12 5.50754564434395e-12
65.0793650793651
"chr9" 116118001 116119000 "*" 6.89059920233603e-12 1.79801256496359e-11
-78.3783783783784
"chr9" 116150001 116151000 "*" 4.26951363152739e-10 8.72510873516957e-10 100
"chr9" 116290001 116291000 "*" 2.76928719153968e-05 2.83568021171378e-05
-51.6129032258064
"chr9" 116302001 116303000 "*" 7.93809462606987e-14 2.64679416473686e-13 -100
"chr9" 116309001 116310000 "*" 1.39779633423487e-07 1.98463037958589e-07 -100
"chr9" 116311001 116312000 "*" 0 0 -100
"chr9" 116343001 116344000 "*" 1.31452626561668e-11 3.30427097674444e-11
-54.1666666666667
"chr9" 116350001 116351000 "*" 4.34545732730385e-11 1.01678918651409e-10 -68.75
"chr9" 116382001 116383000 "*" 0 0 -66.6666666666667
"chr9" 116432001 116433000 "*" 0 0 100
"chr9" 116480001 116481000 "*" 1.3647835084285e-09 2.59380388341009e-09
63.8095238095238
"chr9" 116634001 116635000 "*" 1.52425849719862e-11 3.80099330328441e-11
74.2857142857143
"chr9" 116713001 116714000 "*" 0 0 99.1869918699187
"chr9" 116755001 116756000 "*" 0 0 84.0579710144928
"chr9" 116756001 116757000 "*" 1.53210777398272e-14 5.5628583449216e-14 100
"chr9" 116814001 116815000 "*" 1.98365768255826e-11 4.85612402473442e-11 100
"chr9" 116820001 116821000 "*" 0 0 -100
"chr9" 116835001 116836000 "*" 7.78377362564697e-13 2.29551678017433e-12 -100
"chr9" 116860001 116861000 "*" 0 0 68.010752688172
"chr9" 116897001 116898000 "*" 0 0 100
"chr9" 116916001 116917000 "*" 0 0 68.2398381056734

"chr9" 116917001 116918000 "*" 1.33164368421035e-10 2.92109446024297e-10 71.2643678160919
"chr9" 116984001 116985000 "*" 4.21884749357559e-15 1.63874445239189e-14 100
"chr9" 117020001 117021000 "*" 0.000319456317450229 0.000275654967013148 -54.8387096774194
"chr9" 117025001 117026000 "*" 7.7715611723761e-16 3.26213507634405e-15 -100
"chr9" 117053001 117054000 "*" 0.000380936249181008 0.000324518373061759 -51.8518518518519
"chr9" 117054001 117055000 "*" 3.10862446895044e-15 1.22466437093774e-14 -100
"chr9" 117065001 117066000 "*" 0 0 74
"chr9" 117070001 117071000 "*" 3.46038175891294e-09 6.21590494032964e-09 56.7567567567568
"chr9" 117118001 117119000 "*" 1.56157221675723e-06 1.93054397956841e-06 58.8235294117647
"chr9" 117124001 117125000 "*" 4.44089209850063e-16 1.91071758245033e-15 100
"chr9" 117133001 117134000 "*" 9.62896429257398e-13 2.80291205576417e-12 -100
"chr9" 117161001 117162000 "*" 2.33844237211844e-05 2.42186234361163e-05 -63.1578947368421
"chr9" 117171001 117172000 "*" 1.99840144432528e-12 5.59476580335607e-12 100
"chr9" 117232001 117233000 "*" 0 0 90.1408450704225
"chr9" 117235001 117236000 "*" 6.04438721296674e-11 1.38849255306602e-10 -100
"chr9" 117349001 117350000 "*" 0 0 84.4143067950249
"chr9" 117373001 117374000 "*" 0 0 74.6268656716418
"chr9" 117374001 117375000 "*" 4.75817232281628e-08 7.23847450932635e-08 79.2207792207792
"chr9" 117661001 117662000 "*" 1.5277158427196e-09 2.87642564232045e-09 -100
"chr9" 117840001 117841000 "*" 1.58474691258625e-07 2.23552987094937e-07 56.5217391304348
"chr9" 117880001 117881000 "*" 0 0 -61.9388046897341
"chr9" 118148001 118149000 "*" 1.35890187991095e-11 3.40841971363279e-11 100
"chr9" 118916001 118917000 "*" 0 0 57.5581761997856
"chr9" 119277001 119278000 "*" 4.78841410966879e-11 1.11468187156588e-10 100
"chr9" 119501001 119502000 "*" 2.88779000712225e-11 6.92935809466068e-11 -100
"chr9" 119694001 119695000 "*" 8.43347613965761e-12 2.17859001990012e-11 100
"chr9" 119730001 119731000 "*" 0 0 -100
"chr9" 119913001 119914000 "*" 6.4324989779152e-11 1.47230020825092e-10 100
"chr9" 120176001 120177000 "*" 0 0 67.9223448800914
"chr9" 120177001 120178000 "*" 0 0 62.8306425402912
"chr9" 120303001 120304000 "*" 0.000432672693284286 0.000365157266029702 -53.3333333333333
"chr9" 120786001 120787000 "*" 9.03037655763939e-09 1.52254012615925e-08 -81.6666666666667
"chr9" 121697001 121698000 "*" 0 0 -100
"chr9" 122289001 122290000 "*" 4.87567607398987e-08 7.40655736401423e-08 73.6842105263158
"chr9" 122616001 122617000 "*" 1.77806291762339e-05 1.8763499200702e-05 63.3333333333333
"chr9" 123630001 123631000 "*" 0 0 -73.1203007518797
"chr9" 123647001 123648000 "*" 1.92068583260152e-14 6.89775382589628e-14 100
"chr9" 123744001 123745000 "*" 0 0 100
"chr9" 124034001 124035000 "*" 7.04690890447335e-05 6.76680270782408e-05 -59.375

"chr9" 124043001 124044000 "*" 3.28055360654389e-11 7.80644993244159e-11 -52.5
"chr9" 124055001 124056000 "*" 6.71435517740626e-07 8.73129962575465e-07
66.6666666666667
"chr9" 124338001 124339000 "*" 4.44089209850063e-15 1.71914916534614e-14 100
"chr9" 124343001 124344000 "*" 0 0 100
"chr9" 124370001 124371000 "*" 0 0 88.6363636363636
"chr9" 124389001 124390000 "*" 2.6748776105201e-07 3.66870571349187e-07
-63.6363636363636
"chr9" 124399001 124400000 "*" 6.54896137319838e-11 1.4976773230754e-10 -58
"chr9" 124423001 124424000 "*" 5.84876591602779e-12 1.54217879800608e-11
-76.4705882352941
"chr9" 124437001 124438000 "*" 0.000120483972667174 0.000111549020346707 -56
"chr9" 124520001 124521000 "*" 9.56235091109647e-13 2.78624438617287e-12
-70.9090909090909
"chr9" 124603001 124604000 "*" 0 0 -58.6206896551724
"chr9" 124639001 124640000 "*" 3.25560134406544e-10 6.76094176171662e-10
86.3945578231292
"chr9" 124725001 124726000 "*" 2.69084754478399e-12 7.41487347763769e-12 -100
"chr9" 124745001 124746000 "*" 0 0 100
"chr9" 124749001 124750000 "*" 0 0 -100
"chr9" 124870001 124871000 "*" 8.01666433236647e-09 1.36392368970109e-08 100
"chr9" 124877001 124878000 "*" 0 0 83.8709677419355
"chr9" 124963001 124964000 "*" 1.67299207820548e-08 2.71377429441055e-08 -100
"chr9" 124972001 124973000 "*" 1.18085074163332e-07 1.69629981861935e-07
-52.5423728813559
"chr9" 124995001 124996000 "*" 0 0 -60.6060606060606
"chr9" 125136001 125137000 "*" 3.65130148338721e-12 9.90602415539447e-12 100
"chr9" 125152001 125153000 "*" 2.1094237467878e-15 8.47434540879246e-15 -100
"chr9" 125157001 125158000 "*" 3.6700841921089e-08 5.65530226072258e-08 100
"chr9" 125169001 125170000 "*" 0.000430302178842101 0.000363344149266076
54.5454545454545
"chr9" 125504001 125505000 "*" 1.1181056081e-12 3.23315952434874e-12
61.9718309859155
"chr9" 125520001 125521000 "*" 1.52794177310511e-09 2.87642564232045e-09 100
"chr9" 125601001 125602000 "*" 3.04324343503026e-11 7.26599935673121e-11 -100
"chr9" 126096001 126097000 "*" 1.12376774552558e-12 3.24761818527664e-12 -100
"chr9" 126142001 126143000 "*" 2.82307510701685e-12 7.74698708617421e-12
-64.2857142857143
"chr9" 126305001 126306000 "*" 1.99840144432528e-12 5.59476580335607e-12 100
"chr9" 126360001 126361000 "*" 4.08209022140227e-11 9.57770690255255e-11 100
"chr9" 126692001 126693000 "*" 0 0 51.3235294117647
"chr9" 126762001 126763000 "*" 0 0 75.9776536312849
"chr9" 126791001 126792000 "*" 4.03010957938932e-14 1.39096982198858e-13
-92.9166666666667
"chr9" 126860001 126861000 "*" 0 0 100
"chr9" 126862001 126863000 "*" 3.82454604652693e-08 5.88223003491028e-08
-52.9411764705882
"chr9" 126871001 126872000 "*" 9.65338919911574e-13 2.80678910656113e-12 -100
"chr9" 126881001 126882000 "*" 2.17684182051148e-05 2.26546862940728e-05
-59.1397849462366
"chr9" 126893001 126894000 "*" 1.8817586378006e-09 3.51056888315216e-09
84.8484848484848

"chr9" 126894001 126895000 "*" 0 0 -64.0243902439024
"chr9" 126902001 126903000 "*" 3.73256980878978e-13 1.1454077693028e-12 58.6206896551724
"chr9" 126971001 126972000 "*" 9.65729496371637e-10 1.87255912493232e-09 100
"chr9" 127045001 127046000 "*" 0 0 50.8064516129032
"chr9" 127125001 127126000 "*" 0 0 100
"chr9" 127183001 127184000 "*" 2.80999001844862e-10 5.89661649598696e-10 76.5957446808511
"chr9" 127198001 127199000 "*" 1.78532651551322e-07 2.50302546291261e-07 -52.7777777777778
"chr9" 127242001 127243000 "*" 1.35890187991095e-11 3.40841971363279e-11 100
"chr9" 127244001 127245000 "*" 0 0 100
"chr9" 127298001 127299000 "*" 3.300530959649e-10 6.84793846318851e-10 -51.6129032258064
"chr9" 127421001 127422000 "*" 7.59847740283703e-12 1.97309393892864e-11 80.6451612903226
"chr9" 127538001 127539000 "*" 3.67513797172592e-11 8.67756832290752e-11 -97.5609756097561
"chr9" 127557001 127558000 "*" 0 0 -66.6666666666667
"chr9" 127597001 127598000 "*" 1.34906912307997e-08 2.21932680341382e-08 -85.1351351351351
"chr9" 127610001 127611000 "*" 1.13140401492018e-08 1.87801380787728e-08 -100
"chr9" 127625001 127626000 "*" 2.64951482975562e-09 4.82186011375752e-09 100
"chr9" 127629001 127630000 "*" 3.41038863815868e-10 7.05621050782627e-10 -80
"chr9" 127636001 127637000 "*" 0 0 -100
"chr9" 128143001 128144000 "*" 4.19658641170884e-10 8.5901784711878e-10 85.2459016393443
"chr9" 128144001 128145000 "*" 1.67299207820548e-08 2.71377429441055e-08 -100
"chr9" 128164001 128165000 "*" 4.44089209850063e-16 1.91071758245033e-15 62.0193068523501
"chr9" 128186001 128187000 "*" 0 0 -57.3529411764706
"chr9" 128352001 128353000 "*" 1.14124265593318e-11 2.89138062669327e-11 -100
"chr9" 128734001 128735000 "*" 0 0 97.8723404255319
"chr9" 128755001 128756000 "*" 4.64830918200931e-08 7.08245292051479e-08 -54.5454545454545
"chr9" 128788001 128789000 "*" 3.07568572610606e-07 4.18239045466418e-07 -57.7777777777778
"chr9" 128791001 128792000 "*" 0.000182145353712748 0.000163691361803338 -54.8387096774194
"chr9" 128795001 128796000 "*" 0 0 100
"chr9" 128799001 128800000 "*" 0 0 63.9004149377593
"chr9" 128801001 128802000 "*" 5.56241275262437e-09 9.68555651506361e-09 56.0975609756098
"chr9" 128817001 128818000 "*" 0 0 -67.0234909307166
"chr9" 128935001 128936000 "*" 1.80409465144749e-10 3.89154643415121e-10 -81.0267857142857
"chr9" 128967001 128968000 "*" 2.10183995305036e-05 2.19239227829249e-05 62.7906976744186
"chr9" 128987001 128988000 "*" 0 0 98.5074626865672
"chr9" 128988001 128989000 "*" 1.3532508447156e-12 3.87402820252333e-12 76
"chr9" 129007001 129008000 "*" 7.7715611723761e-16 3.26213507634405e-15 100
"chr9" 129057001 129058000 "*" 2.00227675550835e-08 3.20459176617961e-08 100

"chr9" 129060001 129061000 "*" 0 0 73.7234042553192
"chr9" 129216001 129217000 "*" 2.82773804372027e-13 8.79129664109438e-13 100
"chr9" 129259001 129260000 "*" 2.04136707537828e-12 5.70241950889402e-12
-58.4057971014493
"chr9" 129285001 129286000 "*" 3.99114519211707e-10 8.19075987917563e-10
76.0869565217391
"chr9" 129314001 129315000 "*" 0 0 -100
"chr9" 129341001 129342000 "*" 5.58502354364165e-08 8.41879077739806e-08 -60
"chr9" 129348001 129349000 "*" 2.58179918336543e-05 2.65543423599634e-05
58.0952380952381
"chr9" 129366001 129367000 "*" 0 0 100
"chr9" 129392001 129393000 "*" 0 0 -100
"chr9" 129405001 129406000 "*" 5.01025332333427e-10 1.01358031135889e-09 -100
"chr9" 129411001 129412000 "*" 3.34128893442198e-08 5.19772864618523e-08 -100
"chr9" 129419001 129420000 "*" 1.37828859436695e-10 3.01445544267661e-10 -100
"chr9" 129446001 129447000 "*" 4.01313771103418e-09 7.11882087237587e-09 100
"chr9" 129467001 129468000 "*" 0 0 60.5125100887813
"chr9" 129542001 129543000 "*" 4.01313771103418e-09 7.11882087237587e-09 100
"chr9" 129543001 129544000 "*" 0.000216316337407707 0.000192133310270739
-52.1739130434783
"chr9" 129621001 129622000 "*" 1.59301718808402e-05 1.69294262236803e-05
-66.6666666666667
"chr9" 129636001 129637000 "*" 1.14124265593318e-11 2.89138062669327e-11 -100
"chr9" 129675001 129676000 "*" 2.82773804372027e-13 8.79129664109438e-13 100
"chr9" 129816001 129817000 "*" 7.88258347483861e-15 2.96145301916012e-14 100
"chr9" 129886001 129887000 "*" 5.04820683033813e-08 7.65275498213945e-08
-55.1724137931034
"chr9" 129941001 129942000 "*" 9.62896429257398e-13 2.80291205576417e-12 100
"chr9" 129948001 129949000 "*" 4.4905441476395e-08 6.85396785780768e-08
63.6363636363636
"chr9" 130007001 130008000 "*" 0 0 61.495874945723
"chr9" 130281001 130282000 "*" 1.11022302462516e-16 5.03662826618488e-16
-64.5161290322581
"chr9" 130300001 130301000 "*" 0 0 100
"chr9" 130324001 130325000 "*" 1.16149007278743e-06 1.46182177276213e-06
-75.4716981132076
"chr9" 130329001 130330000 "*" 0 0 100
"chr9" 130338001 130339000 "*" 1.15092973151931e-07 1.6561816717757e-07
73.5849056603774
"chr9" 130341001 130342000 "*" 0 0 -100
"chr9" 130391001 130392000 "*" 0 0 100
"chr9" 130430001 130431000 "*" 3.67483821150927e-14 1.27627245811088e-13
54.054834054834
"chr9" 130515001 130516000 "*" 2.71735967061204e-11 6.55947005633545e-11
78.6666666666667
"chr9" 130525001 130526000 "*" 1.10168968092683e-06 1.39050345400316e-06
54.2754275427543
"chr9" 130540001 130541000 "*" 6.12675200573065e-08 9.1864046786118e-08
51.6666666666667
"chr9" 130546001 130547000 "*" 0 0 100
"chr9" 130640001 130641000 "*" 0 0 -77.7777777777778
"chr9" 130645001 130646000 "*" 3.6700841921089e-08 5.65530226072258e-08 -100

"chr9" 130692001 130693000 "*" 0 0 58.7549039851332
"chr9" 130694001 130695000 "*" 1.11022302462516e-16 5.03662826618488e-16 100
"chr9" 130729001 130730000 "*" 1.40477241139525e-06 1.74851127817574e-06 72
"chr9" 130861001 130862000 "*" 0.000411208966935583 0.000348393043769852 -60
"chr9" 130874001 130875000 "*" 1.09579012530503e-12 3.1721506484884e-12 -57.5
"chr9" 130955001 130956000 "*" 0 0 -75.4648176682075
"chr9" 130966001 130967000 "*" 0 0 78.5414920368818
"chr9" 130987001 130988000 "*" 6.09713612931273e-10 1.22037391452386e-09
-52.3809523809524
"chr9" 131017001 131018000 "*" 5.51780843238703e-14 1.87243792572872e-13 100
"chr9" 131058001 131059000 "*" 1.19451015745398e-09 2.28482591782588e-09
-52.3809523809524
"chr9" 131068001 131069000 "*" 3.34494487663761e-09 6.02060458470289e-09
-66.6666666666667
"chr9" 131119001 131120000 "*" 8.25450818808804e-13 2.42473843346228e-12 100
"chr9" 131213001 131214000 "*" 6.52733422867868e-12 1.71146951718423e-11 -100
"chr9" 131217001 131218000 "*" 0 0 -55.2510683760684
"chr9" 131336001 131337000 "*" 3.49194319015922e-08 5.41783859504158e-08
-71.4285714285714
"chr9" 131419001 131420000 "*" 0 0 100
"chr9" 131447001 131448000 "*" 0.000896607310246877 0.000717057981883268
-53.8461538461538
"chr9" 131460001 131461000 "*" 0 0 -88.8888888888889
"chr9" 131638001 131639000 "*" 4.48752146553488e-13 1.36105323256995e-12
-72.9166666666667
"chr9" 131755001 131756000 "*" 3.33066907387547e-16 1.4495649018245e-15 -100
"chr9" 131894001 131895000 "*" 7.93809462606987e-14 2.64679416473686e-13 100
"chr9" 131895001 131896000 "*" 8.21565038222616e-15 3.08158744293689e-14 100
"chr9" 131910001 131911000 "*" 6.93267986140444e-05 6.66409676902322e-05 -52
"chr9" 131937001 131938000 "*" 0 0 -79.6747967479675
"chr9" 131946001 131947000 "*" 2.11221961493591e-05 2.20233781915554e-05 -52
"chr9" 132017001 132018000 "*" 1.87713000698864e-09 3.50224717311881e-09
-67.6470588235294
"chr9" 132034001 132035000 "*" 3.31690230837012e-12 9.03347040781321e-12 100
"chr9" 132035001 132036000 "*" 7.05324687544362e-13 2.08731000394342e-12
63.3997785160576
"chr9" 132036001 132037000 "*" 1.7534784735318e-10 3.78726179142052e-10
56.140350877193
"chr9" 132040001 132041000 "*" 2.30168661907726e-10 4.88677430469091e-10
-86.6666666666667
"chr9" 132042001 132043000 "*" 4.71077126048547e-05 4.65201892615276e-05
62.2222222222222
"chr9" 132051001 132052000 "*" 2.18935980456081e-13 6.89554885263635e-13
-60.9375
"chr9" 132075001 132076000 "*" 3.99906009418416e-08 6.13728100763028e-08 56.25
"chr9" 132084001 132085000 "*" 4.77223246475589e-08 7.25827507362187e-08
57.4212893553223
"chr9" 132107001 132108000 "*" 2.08995487582797e-10 4.46944006972663e-10 100
"chr9" 132149001 132150000 "*" 8.37826763522287e-09 1.42187770420251e-08
69.6969696969697
"chr9" 132173001 132174000 "*" 5.12929115925598e-06 5.87279945144906e-06
-50.2283105022831

"chr9" 132175001 132176000 "*" 0 0 100
"chr9" 132192001 132193000 "*" 3.33066907387547e-16 1.4495649018245e-15 -88.2352941176471
"chr9" 132227001 132228000 "*" 0 0 -100
"chr9" 132245001 132246000 "*" 0 0 -70.8955223880597
"chr9" 132246001 132247000 "*" 8.21565038222616e-15 3.08158744293689e-14 -62.7555555555556
"chr9" 132251001 132252000 "*" 0 0 -76.9230769230769
"chr9" 132259001 132260000 "*" 0 0 57.4410774410774
"chr9" 132276001 132277000 "*" 3.96762936054618e-05 3.96172468107732e-05 -71.304347826087
"chr9" 132299001 132300000 "*" 1.89581683684992e-12 5.32311367606535e-12 -100
"chr9" 132330001 132331000 "*" 0 0 -75.7731958762887
"chr9" 132331001 132332000 "*" 0 0 88.8888888888889
"chr9" 132342001 132343000 "*" 7.7715611723761e-16 3.26213507634405e-15 100
"chr9" 132359001 132360000 "*" 0 0 -67.4157303370787
"chr9" 132372001 132373000 "*" 0 0 -62.5560836501901
"chr9" 132374001 132375000 "*" 5.03441732746523e-12 1.3378171545226e-11 -100
"chr9" 132405001 132406000 "*" 0.000430041727701558 0.000363138932591808 61.5384615384615
"chr9" 132445001 132446000 "*" 5.68123437183488e-10 1.14082514207793e-09 84.375
"chr9" 132473001 132474000 "*" 3.63445940010365e-11 8.58565209111204e-11 100
"chr9" 132474001 132475000 "*" 0 0 100
"chr9" 132497001 132498000 "*" 6.43374242770278e-13 1.91198190776055e-12 73.9130434782609
"chr9" 132503001 132504000 "*" 1.10467190950203e-13 3.61721268846938e-13 58.8235294117647
"chr9" 132507001 132508000 "*" 5.55111512312578e-16 2.36485094870365e-15 100
"chr9" 132537001 132538000 "*" 2.95474755773739e-12 8.09234223318034e-12 -52.3809523809524
"chr9" 132539001 132540000 "*" 3.96205976915809e-08 6.08351982221736e-08 -68.075117370892
"chr9" 132546001 132547000 "*" 1.98365768255826e-11 4.85612402473442e-11 -100
"chr9" 132550001 132551000 "*" 1.53176493378204e-07 2.16415827665871e-07 -61.6666666666667
"chr9" 132635001 132636000 "*" 1.10089715121831e-12 3.18605002788397e-12 68.6274509803922
"chr9" 132705001 132706000 "*" 7.24119299411186e-07 9.37400919830833e-07 -60.7142857142857
"chr9" 132804001 132805000 "*" 0 0 86.0472482308952
"chr9" 132805001 132806000 "*" 0 0 87.6509795382323
"chr9" 132878001 132879000 "*" 1.92766098638941e-08 3.10267619627471e-08 51.5350877192982
"chr9" 132906001 132907000 "*" 0 0 -55.8144329896907
"chr9" 132914001 132915000 "*" 6.59039323247512e-06 7.41758959369498e-06 -54.1666666666667
"chr9" 132937001 132938000 "*" 7.97140131680862e-14 2.65641277678906e-13 100
"chr9" 133026001 133027000 "*" 3.07753822426093e-13 9.53485764160054e-13 69.5652173913043
"chr9" 133039001 133040000 "*" 0 0 -92.5925925925926
"chr9" 133047001 133048000 "*" 7.43365276667518e-07 9.60526488142265e-07 64.5161290322581

"chr9" 133057001 133058000 "*" 4.14335232790108e-13 1.26153063645578e-12 100
"chr9" 133068001 133069000 "*" 2.11062056720834e-10 4.51166768361064e-10
52.7777777777778
"chr9" 133240001 133241000 "*" 9.47973857505779e-05 8.92323920772727e-05
61.2903225806452
"chr9" 133246001 133247000 "*" 7.10505432266473e-10 1.4044522243975e-09
-65.4545454545455
"chr9" 133256001 133257000 "*" 4.68577992540631e-06 5.39298564287784e-06
-54.8701298701299
"chr9" 133282001 133283000 "*" 0 0 -100
"chr9" 133308001 133309000 "*" 0 0 59.1201337970706
"chr9" 133331001 133332000 "*" 1.17461596005342e-13 3.8279067575877e-13 100
"chr9" 133365001 133366000 "*" 6.25142326349248e-07 8.16495274023725e-07
-75.3086419753086
"chr9" 133369001 133370000 "*" 1.25284005392245e-10 2.75649590272477e-10 -100
"chr9" 133405001 133406000 "*" 2.46431841599559e-09 4.51021250080738e-09
-62.2222222222222
"chr9" 133408001 133409000 "*" 5.73982972262854e-10 1.15203667449908e-09 55
"chr9" 133418001 133419000 "*" 6.66133814775094e-16 2.81595744474255e-15
68.2352941176471
"chr9" 133419001 133420000 "*" 3.63015936200028e-08 5.62046932076955e-08
-51.8518518518519
"chr9" 133439001 133440000 "*" 1.39779633423487e-07 1.98463037958589e-07 100
"chr9" 133492001 133493000 "*" 3.07531777821168e-14 1.07626052665081e-13 100
"chr9" 133516001 133517000 "*" 2.64951482975562e-09 4.82186011375752e-09 -100
"chr9" 133522001 133523000 "*" 1.14124265593318e-11 2.89138062669327e-11 100
"chr9" 133526001 133527000 "*" 7.97151233911109e-12 2.06584328191039e-11 -52
"chr9" 133531001 133532000 "*" 0 0 100
"chr9" 133544001 133545000 "*" 2.08458805772693e-10 4.46464320373107e-10
88.0597014925373
"chr9" 133545001 133546000 "*" 2.60248147854192e-06 3.11310464797009e-06
-73.9130434782609
"chr9" 133571001 133572000 "*" 9.08108033215171e-11 2.03407753381119e-10 100
"chr9" 133710001 133711000 "*" 9.99200722162641e-16 4.15382497462808e-15
-65.1098901098901
"chr9" 133711001 133712000 "*" 0 0 -83.5227272727273
"chr9" 133808001 133809000 "*" 7.63722418639645e-12 1.98278489621246e-11
-54.8809523809524
"chr9" 133809001 133810000 "*" 0 0 75.1547987616099
"chr9" 133816001 133817000 "*" 2.02097823231995e-06 2.45715483608475e-06 -74
"chr9" 133821001 133822000 "*" 1.48214773787458e-13 4.77488235285846e-13 -100
"chr9" 133831001 133832000 "*" 5.3401949529075e-10 1.07750515562622e-09
54.8387096774194
"chr9" 133862001 133863000 "*" 0 0 74.2574257425743
"chr9" 133869001 133870000 "*" 2.22044604925031e-16 9.81641919380259e-16 100
"chr9" 133899001 133900000 "*" 1.39779633423487e-07 1.98463037958589e-07 100
"chr9" 133971001 133972000 "*" 0 0 59.4562647754137
"chr9" 133974001 133975000 "*" 2.6165741273676e-06 3.12843692934624e-06
-66.6666666666667
"chr9" 133990001 133991000 "*" 4.44089209850063e-16 1.91071758245033e-15
98.6301369863014
"chr9" 133996001 133997000 "*" 4.79616346638068e-14 1.64380445416596e-13 -100

"chr9" 134054001 134055000 "*" 2.67283972732457e-11 6.45686178157698e-11 100
"chr9" 134154001 134155000 "*" 4.50769179283217e-06 5.20007520812749e-06 64.2857142857143
"chr9" 134190001 134191000 "*" 7.50607564992123e-09 1.2830216853392e-08 -65
"chr9" 134209001 134210000 "*" 4.28237856286984e-09 7.57162087834286e-09 -60.6060606060606
"chr9" 134224001 134225000 "*" 2.79987144580218e-12 7.68634726811898e-12 -100
"chr9" 134372001 134373000 "*" 2.02864001153102e-08 3.24385970938323e-08 -60
"chr9" 134378001 134379000 "*" 0 0 69.9138858988159
"chr9" 134386001 134387000 "*" 6.67017106771306e-05 6.43065783242906e-05 -60.7142857142857
"chr9" 134421001 134422000 "*" 0 0 63.8665156472858
"chr9" 134451001 134452000 "*" 1.82839299256443e-11 4.51632655829828e-11 85.6375838926175
"chr9" 134523001 134524000 "*" 2.6569935140941e-09 4.83462661545569e-09 -66.6666666666667
"chr9" 134609001 134610000 "*" 3.99930422112504e-10 8.20609062234117e-10 -91.4285714285714
"chr9" 134611001 134612000 "*" 6.39864106144472e-09 1.1057835146776e-08 -60.4651162790698
"chr9" 134663001 134664000 "*" 1.11022302462516e-16 5.03662826618488e-16 56.7655317655318
"chr9" 134668001 134669000 "*" 3.51940698806175e-14 1.22560856705654e-13 -71.2643678160919
"chr9" 134689001 134690000 "*" 0 0 100
"chr9" 134706001 134707000 "*" 1.4071850989561e-05 1.50695589335136e-05 50.1411631846414
"chr9" 134989001 134990000 "*" 8.30925877233213e-05 7.89057879117877e-05 53.0612244897959
"chr9" 135002001 135003000 "*" 1.11022302462516e-16 5.03662826618488e-16 100
"chr9" 135007001 135008000 "*" 9.04570973681018e-10 1.76414389822173e-09 -100
"chr9" 135012001 135013000 "*" 2.27498986982155e-07 3.14825262153612e-07 73.6842105263158
"chr9" 135016001 135017000 "*" 2.43138842392909e-14 8.63295539017152e-14 66.6666666666667
"chr9" 135018001 135019000 "*" 6.27111362838662e-10 1.2508137164194e-09 54.9152542372881
"chr9" 135028001 135029000 "*" 6.15840711759574e-13 1.83676803572802e-12 -100
"chr9" 135030001 135031000 "*" 0 0 100
"chr9" 135037001 135038000 "*" 0 0 52.0253605986378
"chr9" 135038001 135039000 "*" 0 0 55.2645047097
"chr9" 135044001 135045000 "*" 0 0 100
"chr9" 135047001 135048000 "*" 3.34128893442198e-08 5.19772864618523e-08 100
"chr9" 135050001 135051000 "*" 0 0 88.8111888111888
"chr9" 135060001 135061000 "*" 1.11022302462516e-16 5.03662826618488e-16 100
"chr9" 135064001 135065000 "*" 5.99520433297585e-15 2.28531569014092e-14 89.041095890411
"chr9" 135089001 135090000 "*" 7.34485790570005e-05 7.03153538894558e-05 54.1666666666667
"chr9" 135097001 135098000 "*" 1.48245636766298e-07 2.09826037421521e-07 65
"chr9" 135103001 135104000 "*" 0 0 81.651376146789
"chr9" 135104001 135105000 "*" 0 0 100

"chr9" 135107001 135108000 "*" 2.40416220598227e-07 3.31688150083746e-07
78.3783783783784
"chr9" 135112001 135113000 "*" 3.82471831983366e-13 1.17234802303244e-12
-57.6923076923077
"chr9" 135117001 135118000 "*" 0 0 59.2603844454264
"chr9" 135126001 135127000 "*" 5.62883073484954e-13 1.68905058935478e-12 -100
"chr9" 135134001 135135000 "*" 1.77380332644361e-12 5.00207721766911e-12
66.6666666666667
"chr9" 135243001 135244000 "*" 0 0 -100
"chr9" 135248001 135249000 "*" 5.88418203051333e-15 2.245355428447e-14 100
"chr9" 135277001 135278000 "*" 5.30608890159101e-12 1.40714017162982e-11
-89.4736842105263
"chr9" 135298001 135299000 "*" 5.5973614321303e-07 7.35711789432905e-07
66.6666666666667
"chr9" 135305001 135306000 "*" 1.31610942394333e-05 1.4161144785177e-05
66.6666666666667
"chr9" 135338001 135339000 "*" 3.25080124097266e-06 3.83190944846261e-06
56.7441860465116
"chr9" 135365001 135366000 "*" 1.83952852950142e-12 5.17499701329218e-12 -100
"chr9" 135398001 135399000 "*" 1.17905685215192e-13 3.83653245040640e-13 100
"chr9" 135412001 135413000 "*" 4.69374403788692e-08 7.1452839075152e-08
-54.9295774647887
"chr9" 135415001 135416000 "*" 6.66133814775094e-16 2.81595744474255e-15
91.6666666666667
"chr9" 135442001 135443000 "*" 6.59550958292954e-09 1.13456194382773e-08 100
"chr9" 135447001 135448000 "*" 0 0 -63.4146341463415
"chr9" 135461001 135462000 "*" 0 0 59.4055944055944
"chr9" 135606001 135607000 "*" 1.92068583260152e-14 6.89775382589628e-14 100
"chr9" 135616001 135617000 "*" 2.44249065417534e-15 9.75180348030994e-15
-60.0035536602701
"chr9" 135709001 135710000 "*" 0 0 51.0551703346397
"chr9" 135727001 135728000 "*" 1.11022302462516e-16 5.03662826618488e-16 100
"chr9" 135737001 135738000 "*" 3.34128893442198e-08 5.19772864618523e-08 100
"chr9" 135751001 135752000 "*" 0 0 100
"chr9" 135755001 135756000 "*" 1.56439660115026e-08 2.55431203133619e-08
80.327868852459
"chr9" 135761001 135762000 "*" 1.67299207820548e-08 2.71377429441055e-08 100
"chr9" 135762001 135763000 "*" 1.88737914186277e-15 7.62011598380321e-15 -100
[truncated: 59,375 more chars]
