# Supplementary material for: Distinct epigenetic signatures elucidate enhancer-gene relationships that delineate CIMP and non-CIMP colorectal cancers
Source: Oncotarget. 2016 Mar 30;7(19):28027–39. doi: 10.18632/oncotarget.8473 (PMC5053707; doi:10.18632/oncotarget.8473)
Supplement: Supplementary file 4 [file oncotarget-07-28027-s004.pdf]

Chromosome	Enhancer	Caco-2	Colo320	LIM1215	SW403	SW480	Colo205	LIM2405	RKO	DLD1	HCT116	HT29	Avg. methylation level (non-CIMP)	Avg. methylation level (CIMP)	p-Value
Chr1	element1702	0.854118	0.593878	0	0.666667	0.5	1	1	1	1	0.918079	0.867043	0.522932345	0.964187018	0.008534
Chr1	element327	0	0	0	0.289828	0.857143	0	0.939827	0.888889	0.713462	0	0.865294	0.229394246	0.567911883	0.211097
Chr1	element2357	1	0	0.5	1	0	0	0	0	0	1	0	0.5	0.166666667	0.253586
Chr1	element1766	0.857143	0.384615	1	0	0	0	0	1	1	0	0	0.448351647	0.333333333	0.710436
Chr1	element2139	0.923077	1	0	1	0	1	0	1	0	0	0	0.584615385	0.333333333	0.449236
Chr2	element1479	0.847619	0.424242	0	1	0.875598	1	0	0.620968	0.720539	1	0	0.629491912	0.556917864	0.790603
Chr3	element1889	1	0.738095	0	0	0	0	1	0.5	0.5	1	1	0.347619048	0.666666667	0.265692
Chr5	element262	0	0	0	0.615385	0.6	0	0	1	0.600863	0	0	0.243076923	0.26681057	0.922384
Chr5	element1473	0	0.75	0.954545	0.333333	0	0	0	1	0	1	1	0.407575758	0.5	0.767313
Chr6	element2234	0	0.5	0	0.181818	0	0	1	1	0	1	0	0.136363636	0.5	0.199182
Chr6	element1690	0.818182	1	0	0	0	0	1	1	0	1	0	0.363636364	0.5	0.679709
Chr7	element1880	1	0	0	0	0.846154	0	1	1	0.909091	1	0.6	0.369230769	0.751515152	0.195246
Chr7	element2198	0.993007	0	0.846154	0.333333	1	0	1	0.976307	1	0	0.9	0.634498834	0.646051198	0.969055
Chr10	element1699	0.538626	0	0.634438	1	0	0.6	0.875	0.953095	0.842593	1	0.166667	0.434612796	0.739559084	0.207621
Chr10	element1437	1	0	0	0	0	0	1	1	0	0.666667	0	0.2	0.444444445	0.42057
Chr10	element285	0	0	0	0.801653	0	0	1	0.881142	1	1	0.439648	0.160330579	0.720131568	0.042078
Chr11	element2073	0.846032	0	1	0.953917	1	1	1	0.936344	1	1	1	0.759989661	0.989390622	0.219377
Chr12	element1973	1	1	0.5	0.845238	0	1	0.666667	1	1	1	1	0.669047619	0.94444445	0.165889
Chr14	element1931	0.911905	0.928571	1	0.285714	1	0	0.875	1	0.8	1	0	0.825238095	0.6125	0.415534
Chr15	element1944	1	1	0	0	0	1	1	1	1	1	1	0.4	1	0.023856
Chr16	element1613	0.885036	0	0.222129	0.5	1	0	0	1	1	0	0	0.521432994	0.333333333	0.532027
Chr17	element1917	0.808642	0.984797	0.888889	0.333333	0	0	0.8	0.9625	0.929825	0.909091	1	0.603132299	0.766902577	0.515192
Chr17	element2241	1	0.5	0.333333	0	1	0.5	0.636364	0	1	1	0	0.566666667	0.522727273	0.873678
Chr21	element1516	0	0	0	0	1	0	1	0	0.25	1	1	0.2	0.541666667	0.273001

Supplementary Table 1:

Beta-values for methylation for each enhancer within each cell type is given above. These 24 enhancers have already been identified by methylKit to contain a region (1000bp) that is differentially methylated between the CIMP and non-CIMP groups. Thus, the average methylation beta-value for each enhancer in the CIMP and non-CIMP groups was calculated and a T-test was also performed to identify the best candidates for further investigation. The p-values of the T-tests are given in the last column.
