# Supplementary material for: Distinct epigenetic signatures elucidate enhancer-gene relationships that delineate CIMP and non-CIMP colorectal cancers
Source: Oncotarget. 2016 Mar 30;7(19):28027–39. doi: 10.18632/oncotarget.8473 (PMC5053707; doi:10.18632/oncotarget.8473)
Supplement: Supplementary file 5 [file oncotarget-07-28027-s005.pdf]

Methylation levels (average β-value of bin) of enhancer elements 1702 and genomic region DC1A in 11 CRC cell lines

name	chr	start	stop	Caco-2	Colo320	LIM1215	SW403	SW480	Colo205	LIM2405	RKO	DLD1	HCT116	HT29
Element 1702	chr1	18958671	18960284	0.854118	0.593878	0	0.666667	0.5	1	1	1	1	0.918079	0.867043
DC1A	chr1	228194001	228195001	0.375921	0.042553	0	0.407953	0	0.904762	0.970149	0.776371	0.923077	0.722222	0.856201

Pearson, *r*	0.85340387
FDR adjusted p-value	0.02492156

Methylation levels (average β-value of bin) of enhancer elements 1702 and genomic region DC1B in 11 CRC cell lines

name	chr	start	stop	Caco-2	Colo320	LIM1215	SW403	SW480	Colo205	LIM2405	RKO	DLD1	HCT116	HT29
Element 1702	chr1	18958671	18960284	0.854118	0.593878	0	0.666667	0.5	1	1	1	1	0.918079	0.867043
DC1B	chr1	240118001	240119001	0	0.333333	0.758621	0	0	0	0	0	0	0	0

Pearson, *r*	-0.85477881
FDR adjusted p-value	0.02492156

Methylation levels (average β-value of bin) of enhancer elements 1944 and genomic region DC15A in 11 CRC cell lines

name	chr	start	stop	Caco-2	Colo320	LIM1215	SW403	SW480	Colo205	LIM2405	RKO	DLD1	HCT116	HT29
Element 1944	chr15	65377669	65381418	1	1	0	0	0	1	1	1	1	1	1
DC15A	chr15	65628001	65629000	0	0	1	0.5	1	0	0	0	0	0	0

Pearson, *r*	-0.949158
FDR adjusted p-value	0.001922

x
