# Supplementary material for: Distinct epigenetic signatures elucidate enhancer-gene relationships that delineate CIMP and non-CIMP colorectal cancers
Source: Oncotarget. 2016 Mar 30;7(19):28027–39. doi: 10.18632/oncotarget.8473 (PMC5053707; doi:10.18632/oncotarget.8473)
Supplement: Supplementary file 6 [file oncotarget-07-28027-s006.pdf]

Methylation levels (rpkm) of enhancer elements 1702 and genomic region DC1A in 24 colorectal cancers from the GEO Series: GSE39068

name	chr	start	stop	2T	7T	18T	23T	24T	31T	33T	34T	38T	40T	1T	3T	8T	14T	15T	16T	19T	21T	32T	35T	36T	37T	39T	41T
Element 17	chr1	18958671	18960284	2.29389	2.561133	1.135448	2.139331	2.099055	1.563733	0.395608	2.563352	1.789741	0.999998	1.137626	0.8591	2.865422	1.009093	0.695084	0.753357	1.520106	0.66767	0.76106	0.486104	1.591248	0.513887	2.164565	0.646655
DC1A	chr1	2.28E+08	2.28E+08	2.512816	0.809713	1.073218	2.284688	4.324626	5.042689	1.626576	10.4476	1.789169	2.527521	0.987697	0.874865	4.584903	0.739568	0.453067	1.068939	1.423162	0.236314	0.350607	0.166258	0.733059	0.527281	2.931699	0.537127

Pearson, r	0.634055
p-value	0.000879

Methylation levels (rpkm) of enhancer elements 1702 and genomic region DC1B in 24 colorectal cancers from the GEO Series: GSE39068

name	chr	start	stop	2T	7T	18T	23T	24T	31T	33T	34T	38T	40T	1T	3T	8T	14T	15T	16T	19T	21T	32T	35T	36T	37T	39T	41T
Element 17	chr1	18958671	18960284	2.29389	2.561133	1.135448	2.139331	2.099055	1.563733	0.395608	2.563352	1.789741	0.999998	1.137626	0.8591	2.865422	1.009093	0.695084	0.753357	1.520106	0.66767	0.76106	0.486104	1.591248	0.513887	2.164565	0.646655
DC1B	chr1	2.4E+08	2.4E+08	1.383461	0.998647	0.726	0.940754	0.689433	1.116595	0.924916	1.071549	0.634866	0.610091	1.128796	0.53464	0.564296	0.739568	1.049209	0.825998	0.553452	0.577657	0.993385	1.235059	0.994866	0.853693	0.802727	0.600318

Pearson, r	0.088944
p-value	0.6792

Methylation levels (rpkm) of enhancer elements 1944 and genomic region DC15A in 24 colorectal cancers from the GEO Series: GSE39068

name	chr	start	stop	2T	7T	18T	23T	24T	31T	33T	34T	38T	40T	1T	3T	8T	14T	15T	16T	19T	21T	32T	35T	36T	37T	39T	41T
Element 19	chr15	65377669	65381418	0.48234	0.331414	0.320181	0.17937	0.259319	0.269212	0.144729	0.051077	0.315825	0.104692	0.470802	0.246505	0.160044	0.227028	0.362816	0.285335	0.221602	0.413525	0.194976	0.240919	0.300506	0.268093	0.121112	0.143377
DC15A	chr15	65628001	65629000	0.452194	0.594384	0.473952	0.403583	0.501907	0.324497	0.415032	0.229847	0.462183	0.130865	0.317791	0.486522	0.28243	0.185077	0.548998	0.705232	0.514433	0.735935	0.672668	0.237749	0.41931	0.527809	0.209616	0.284646

Pearson, r	0.493031
p-value	0.014374

x
